# Harmonizing Labeling and Analytical Strategies to Obtain Protein Turnover Rates in Intact Adult Animals

**DOI:** 10.1016/j.mcpro.2022.100252

**Published:** 2022-05-28

**Authors:** Dean E. Hammond, Deborah M. Simpson, Catarina Franco, Marina Wright Muelas, John Waters, R.W. Ludwig, Mark C. Prescott, Jane L. Hurst, Robert J. Beynon, Edward Lau

**Affiliations:** 1 Department of Biochemistry and System Biology, Institute of Systems and Integrative Biology, University of Liverpool, Liverpool, United Kingdom; 2 Centre for Metabolomics Research, Institute of Systems and Integrative Biology, University of Liverpool, Liverpool, United Kingdom; 3 Mammalian Behaviour & Evolution Group, Institute of Infection, Veterinary and Ecological Sciences, University of Liverpool, Leahurst Campus, Neston, United Kingdom; 4 Department of Medicine/Cardiology, Consortium for Fibrosis Research and Translation, University of Colorado School of Medicine, Aurora, Colorado, USA

**Keywords:** protein turnover, proteostasis, heavy water, protein degradation, stable isotope labeling by amino acids, AA, amino acid, HW, heavy water, MS, mass spectrometry, MUP, major urinary protein, RIA, relative isotope abundance, RIA_p_, precursor RIA plateau

## Abstract

Changes in the abundance of individual proteins in the proteome can be elicited by modulation of protein synthesis (the rate of input of newly synthesized proteins into the protein pool) or degradation (the rate of removal of protein molecules from the pool). A full understanding of proteome changes therefore requires a definition of the roles of these two processes in proteostasis, collectively known as protein turnover. Because protein turnover occurs even in the absence of overt changes in pool abundance, turnover measurements necessitate monitoring the flux of stable isotope–labeled precursors through the protein pool such as labeled amino acids or metabolic precursors such as ammonium chloride or heavy water. In cells in culture, the ability to manipulate precursor pools by rapid medium changes is simple, but for more complex systems such as intact animals, the approach becomes more convoluted. Individual methods bring specific complications, and the suitability of different methods has not been comprehensively explored. In this study, we compare the turnover rates of proteins across four mouse tissues, obtained from the same inbred mouse strain maintained under identical husbandry conditions, measured using either [^13^C_6_]lysine or [^2^H_2_]O as the labeling precursor. We show that for long-lived proteins, the two approaches yield essentially identical measures of the first-order rate constant for degradation. For short-lived proteins, there is a need to compensate for the slower equilibration of lysine through the precursor pools. We evaluate different approaches to provide that compensation. We conclude that both labels are suitable, but careful determination of precursor enrichment kinetics in amino acid labeling is critical and has a considerable influence on the numerical values of the derived protein turnover rates.

## Highlights

• Controlled comparison of heavy water or amino acid labeling for protein turnover.
• Delays in amino acid precursor labeling mostly affect high turnover proteins
• Both methods produced similar turnover rates after adjustment of precursor kinetics.
• Recommendations for analytical workflows for protein turnover studies in animals.

## Graphical Abstract

**Figure:**
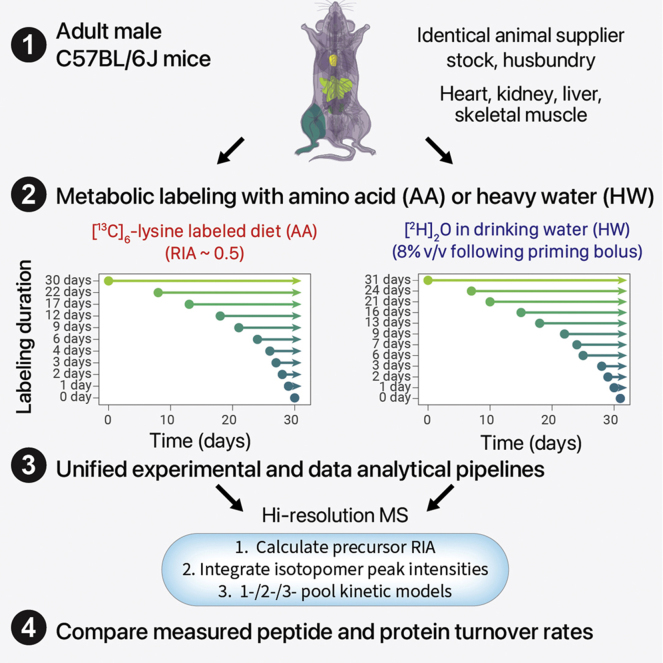

## In Brief

Protein turnover rates in adult animals differ greatly from cell culture and require careful considerations of precursor equilibration and availability. This study by Hammond *et al*. compared two approaches to measurement of protein turnover rates in the mouse, across four mouse tissues. Labeling with stable isotope–labeled amino acids or by heavy water had different analytical complications but yielded comparable turnover rates after optimization. Analytical and data filtering recommendations are provided for protein turnover studies in animals.

Changes in the proteome can be achieved by adjustment of the input into a protein pool (synthesis) or removal of a protein from the pool (degradation), the two processes constituting protein turnover. The simplest model of proteostasis, which is undoubtedly an oversimplification, has three parameters (pool size, synthesis, and degradation), linked by zero-order synthesis (the rate of synthesis is insensitive to the pool size) and first-order degradation (a proportion of the protein pool is degraded per unit time). At steady state, the unchanging pool size is given by the balance between the opposing fluxes of synthesis (molecules/time) and removal (protein pool multiplied by the fractional rate of degradation; thus, also with the dimensions of molecules/time). An adequate description of proteostasis requires that we can measure at least two of these parameters, from which the third can be calculated. Because protein turnover can occur in the absence of any change in pool size, it is evident that turnover parameters must be measured by the flux of a tracer through the protein pool. Most commonly, this is achieved in cells in culture with radiolabeled (*e.g.*, [^35^S]methionine) or stable isotope–labeled (*e.g.*, [^13^C_6_]lysine) protein precursors (“dynamic stable isotope labeling by amino acids [AAs]” (1, 2)). The ability to exchange culture media quickly *in vitro* means that precursor pools can be rapidly manipulated and thus, a transition from labeled to unlabeled media, or vice versa, can be made very rapid, relative to protein turnover rates, which minimizes the effects of precursor pool equilibration (3).

It is now clear that when compared with cells in culture, protein turnover in animal tissues occurs in completely different temporal regimes, with turnover rates spanning orders of magnitude. Moreover, different tissues have distinct average turnover rates (*e.g.*, liver has a higher turnover rate than skeletal muscle (4, 5, 6)), and larger animals have much lower average rates of protein turnover (6). This is in part because of the different energetics constraints between free-living animals and cultured cells. The latter grow exponentially in excess nutrients and through cell division can also remove “old” proteins *via* passive dilution, reducing the need for energetically costly proteostatic degradation. This casts doubt on the applicability of cell culture study to understanding turnover in organismal physiology, growth, and aging, and strongly calls for direct measurements of turnover in animal systems.

**Fig. 1:**
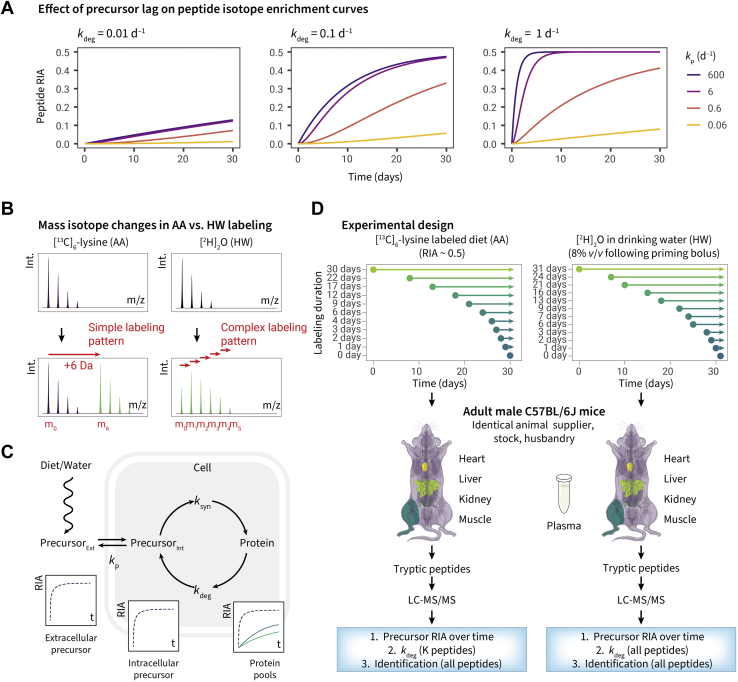
**Comparison of labeling strategies for turnover studies in intact adult animals**. *A*, the effect on protein labeling of a delay in precursor equilibration. The curves model the effect of a delay in precursor equilibration on labeling of protein pools, for three proteins with degradation rate constants of 0.01 d^–1^, 0.1 d^–1^, and 1 d^–1^ (half lives of 69, 6.9, and 0.69, respectively). Four precursor equilibration rates are modeled, with the *blue* and *magenta lines* representing such a high rate (600 d^–1^ and 6 d^–1^) as to be equivalent to near-instantaneous equilibration through the body, giving no perceptible delay. In amino acid (AA) labeling experiments, a *k*_p_ of 0.1 to 0.5 may represent a realistic range dependent on labeling routes and tissues examined (see the main text). As the delay becomes more prolonged (*orange* and *yellow lines*), the protein labeling becomes commensurately slower, leading to an underestimate of the true degradation rate constant across proteins with different *k*_deg_ values (*k*_deg_ of 0.01, 0.1, and 1 d^−1^ shown). *B*, mass spectrum features in AA labeling (*left*), which creates new peptide isotope clusters and elemental heavy water (HW) labeling (*right*), which shifts the endogenous isotopomer rightward in the mass spectrum. *C*, schematic of precursor introduction and pool enrichment showing the availability of intracellular precursors for protein synthesis. *D*, experimental design. Groups of mice, identical in strain (C57BL/6J), age, sex (male), supplier, and husbandry were each labeled with either [^13^C_6_]lysine or [^2^H_2_]O for up to 31 days, sampling tissues throughout the labeling period. Subsequently, tissues were recovered, and tryptic digests were prepared from tissue homogenates to gain protein identity and to assess the degree of isotopic incorporation into proteins.

Unlike cells in culture, in animal systems, the rapid exchange of precursor pools is not always feasible or practical. Isotopically labeled precursors can be administered enterally or parenterally, but in both circumstances, there is a delay in equilibration of the labeled precursor with the tissue pools, such that in the early phases of labeling, high turnover proteins are sampling a precursor pool that has yet to reach equilibrium. Early studies used radiolabeled AA precursors, and although scintillation counting permitted the measurement of very low levels of radiolabel incorporation, this approach was only suitable for total protein pools or measuring purified proteins (7, 8). The need to understand proteostasis on a proteome-wide scale has increased the need to measure protein turnover for multiple proteins in the same system and requires the deployment of stable isotopes. Stable isotope labeling, in combination with proteomics, can yield turnover rates for individual members of the proteome. An additional complication in animal tissues is that turnover rates can be low, and it is difficult to measure very low levels of stable isotope in proteomics-focused mass spectrometry (MS). Thus, labeling duration must be sufficient to lead to discernible incorporation of the label. Stable isotope administration is largely oral, through diet or drinking water and inevitably, this route of administration introduces a delay in equilibration of the precursor with whole-body metabolic pools. This delay can introduce systematic underestimates of rates of turnover, simply illustrated (Fig. 1*C*) by modeling of a two-compartment model (9).

For animal studies, two approaches are most used, both based on exposure of subjects to stable isotope precursors followed by measurement of the rate of isotope incorporation into individual proteins. First, a labeled essential AA can be provided in the diet, either with a relative isotope abundance (RIA) of 1, which requires a fully synthetic diet (10, 11, 12), or at a lower RIA by supplementation of a standard laboratory diet with pure labeled AA (4, 5, 13, 14). Typically, the labeled AA incorporates multiple heavy atom centers, such that labeled peptides yield *m/z* values that are well resolved from the natural isotope distribution of the unlabeled AA (Fig. 1*B*, *left*). Alternatively, animals can be provided with metabolically simple precursors, such as [^2^H]_2_O or [^15^N]H_4_Cl, that deliver a single labeled atom center to some or all AAs (15, 16, 17, 18). In this instance, the labeling trajectory leads to a gradual shift of the isotopic profile with considerable overlap between the unlabeled isotopomer profile and the labeled profile (Fig. 1*B*, *right*).

Thus, the incorporation of labels into proteins (and therefore, into peptides derived from those proteins; an essential element in the proteomics workflow) is very different with the two labeling protocols. For AA labeling (“AA”) strategies, the incorporation of one or more instances of the labeled AA creates “heavy” peptides that are offset by the number of heavy atom centers in the AA (such as [^13^C_6_]lysine or [^2^H_7_]valine (5)). By contrast, for example, the deuterium atoms in heavy water (“HW”) labeling strategies are incorporated stably into specific AAs, leading to complex labeling patterns wherein labeled peptides have mass shifts from 1 to many daltons higher (17, 18, 19, 20, 21, 22) (Fig. 1*B*).

A second difference between the AA and HW strategies pertains to the equilibration of the label with the AA pool that is the immediate precursor of protein synthesis. Dietary AAs need to cross the intestinal mucosal barrier, pass through the hepatic system, and are eventually transported to peripheral tissues through the blood (Fig. 1*C*). By contrast, water crosses all membranes and is rapidly equilibrated across all tissues (18). If equilibration of the label with the precursor is considerably faster than the rate of turnover of the protein pool, then it can be assumed that the precursor enrichment is constant over the labeling period. Under this circumstance, a simple monoexponential function will define the transition from unlabeled to labeled protein. In this regard, HW labeling should equilibrate rapidly, which can be aided by an initial bolus injection of pure [^2^H]_2_O. However, if the precursor pool equilibrates at rates similar to the fastest turnover proteins, then a more complex model is appropriate (5). It follows that the AA strategy could be compromised by a delay in pool equilibration, and this would be particularly evident in proteins that were substantially synthesized during the equilibration phase, specifically, high turnover proteins. Because of this unavoidable lag, there have been a number of different solutions to address slow precursor equilibration with AAs (9, 11, 12, 23, 24).

To explore the differences between the AA and HW strategies and to attempt to harmonize the two approaches, we compared the turnover profiles of multiple proteins, derived from four tissues, in mice that were otherwise identical in genotype, source, age, sex, and husbandry (Fig. 1*D*). This study allowed us to compare the two labeling approaches with a precision not previously realized. Here, we present the outcomes of these experiments and show that whilst each approach yields quantitatively comparable results for slow turnover proteins, they are increasingly discrepant for high turnover proteins in a simple exponential kinetics model. In particular, a HW methodology seems to consistently yield turnover rate constants that are higher than those obtained by an AA strategy. When two-compartment models are used to correct for the delay in equilibration of the labeled precursor(s), the rate constants converge more closely.

## Experimental Procedures

### Experimental Design and Statistical Rationale

We performed a controlled laboratory study comparing the analytical workflows of HW and AA labeling strategies. Both labeling studies were conducted with laboratory mice derived from the same supplier and maintained under identical conditions. The experiments are designed to enable comparison of sampling depth, data variance, and statistics as described in the Results section.

### Animal Husbandry

Fully grown adult male C57BL/6JOlaHsd mice (obtained from Harlan UK Ltd, at 6–13 weeks of age) were previously group housed and used in noninvasive behavioral studies. At the start of this experiment, males aged 15 to 16 months old were housed individually in 48 × 15 × 15 cm polypropylene cages (NKP Cages Ltd). Each cage contained substrate (Corn Cob Absorb 10/14; IPS Ltd), paper wool nest material, and environmental enrichment (hanging baskets and plastic tubes). Food (LabDiet 5002 Certified Rodent Diet; Purina Mills) and water were provided ad libitum. The mice were maintained on a reversed photoperiod (light 12 h; dark 12 h; lights on at 20:00 h) and at 19 to 21 °C. Animal use and care was in accordance with European Union directive 2010/63/European Union and UK Home Office code of practice for the housing and care of animals bred, supplied, and used for scientific purposes. HW labeling was carried out under UK Home Office license under the Animals in Scientific Procedures Act 1986 (PPL 40/3492). The University of Liverpool Animal Welfare Committee approved the work.

### Labeling With [^13^C_6_]lysine

This study used 11 mice. Standard laboratory diet (LabDiet 5002) was supplemented with pure crystalline [^13^C_6_]lysine (Cambridge Isotope Laboratories) to bring the RIA to 0.5. The dietary pellets were dissociated with water containing the dissolved [^13^C_6_]lysine to form a thick paste and mixed extensively. Once homogeneous, the paste was then extruded into strips 1 cm across and dried in a commercial foodstuff drying oven at 40 °C. The mice had access to the labeled diet for varying amounts of time with randomized assignment: 0, 1, 2, 3, 4, 6, 9, 12, 17, 22, or 30 days. The day that the animals were introduced to the labeled diet was staggered for all endpoints so that dissections took place on the same day. All mice were humanely killed on day 30, and the animals were dissected to recover liver, kidney, heart, and pooled hindlimb skeletal muscle from each animal. All tissues, and the carcasses, were frozen at –80 °C prior to analysis.

### Labeling With [^2^H_2_]O

For the HW labeling protocol, all animals (13) were provided free access to LabDiet 5002. At the start of the experiment, mice were injected with two successive 0.5 ml injections, 4 h apart, of 0.15 M sodium chloride dissolved in deuterated water. Thereafter, mice were given free access to 8% (v/v) [^2^H_2_]O for the duration of the experiment. After 0, 1, 2, 3, 6, 7, 9, 13, 16, 21, 24, and 31 days, mice were killed and dissected exactly as described for the [^13^C_6_]lysine labeling experiment, and tissues were stored at –80 °C prior to analysis. In addition, plasma samples were obtained by postmortem cardiac puncture.

### Preparation of Samples for Proteomics

Small portions (typically 50–100 mg wet weight) from the frozen organs from both studies were further cut into small pieces to facilitate homogenization in 1 ml of lysis buffer (7 M urea, 2 M thiourea, 2% [w/v] CHAPS, and 5 mM DTT) using a Precellys lysis kit (Stretton Scientific Ltd). Total protein extracted was quantified using a Bradford assay. Protein (200 μg, AA; 100 μg, HW) was reduced, alkylated, and digested with trypsin using a modified version of the filter-aided sample preparation approach (25). The labeling protocols were designed so that all labeling time points for a single tissue (11 samples, AA; 12 samples, HW) were prepared and analyzed concurrently.

Nontargeted MS1–data-dependent acquisition analyses were conducted on a Q-Exactive HF quadrupole-Orbitrap mass spectrometer coupled to a Dionex Ultimate 3000 RSLC nano-liquid chromatograph (Hemel Hempstead). One microgram of peptides from each time point were loaded onto a trapping column (Acclaim PepMap 100 C18, 75 μm × 2 cm, 3 μm packing material, 100 Å) using a loading buffer of 0.1% (v/v) TFA, 2% (v/v) acetonitrile in water for 7 min at a flow rate of 12 μl min^−1^. The trapping column was in-line to an analytical column (EASY-Spray PepMap RSLC C18, 75 μm × 50 cm, 2 μm packing material, 100 Å) and peptides eluted using a linear gradient of 96.2% A (0.1% [v/v] formic acid): 3.8% B (0.1% [v/v] formic acid in water:acetonitrile [80:20] [v/v]) to 50% A:50% B over 90 min at a flow rate of 300 nl min^–1^, followed by washing at 1% A:99% B for 8 min and then re-equilibration of the column to starting conditions. The column was maintained at 40 °C, and the eluent was introduced directly into the integrated nano-electrospray ionization source operating in positive ion mode. The mass spectrometer was operated in data-dependent acquisition mode with survey scans between *m/z* 350 and 2000 acquired at a mass resolution of 60,000 (full width at half maximum) at *m/z* 200. The maximum injection time was 100 ms, and the automatic gain control was set to 3e6. The 16 most intense precursor ions with charge states of 2+ to 5+ were selected for MS/MS with an isolation window of 1.2 *m/z* units. The maximum injection time was 45 ms, and the automatic gain control was set to 1e5. Fragmentation of the peptides was by higher-energy collisional dissociation using a stepped normalized collision energy of 28 to 30%. Dynamic exclusion of *m/z* values to prevent repeated fragmentation of the same peptide was used with an exclusion time of 20 s.

### Experimental Measurement of Precursor Enrichment

Plasma samples (50 μl) were treated with 2 μl of 10 M sodium hydroxide (BDH) and 1 μl of acetone (Fisher). After mixing, the samples were left overnight at 20 ^o^C to allow the exchange of deuterium from water to acetone to occur. To produce a calibration curve, 50 μl mixtures of between 0 and 10% (*v*/*v*) deuterium oxide (Cambridge Isotope Laboratories) in HPLC grade water (VWR International) were also treated and extracted. The acetone was then extracted from the samples using 200 μl of chloroform (VWR) for 15 s. Aliquots of the extracts were then analyzed by GC–MS on a Waters GCT Premier gas chromatograph-mass spectrometer (Waters). The chromatography column employed was a 30 m long, 0.25 mm internal diameter, 0.25 μm film thickness DB-17MS (Agilent J&W). The carrier gas was helium (BOC) at 1 ml min^−1^. The injector was operated in the splitless mode at 220 ^o^C, and the injection volume was 1 μl. The oven temperature program was 60 ^o^C to 100 ^o^C at 20 ^o^C min^−1^ with a 1 min hold, then from 100 ^o^C to 220 ^o^C at 50 ^o^C min^−1^. The mass spectrometer was operated in the positive ion electron ionization mode with source temperature 200 ^o^C, electron energy 70 eV, and trap current 200 μA. Mass spectra were recorded in low sensitivity mode between 40 and 100 *m/z* with a scan time of 0.1 s. The spectral intensities of ions at *m/z* 58 and *m/z* 59 were measured using the MassLynx software (Waters Corporation) supplied with the instrument. Comparison of the ratio of *m/z* 59 to *m/z* 58 for the biological samples against the curve generated from the calibration samples allowed the enrichment of deuterium oxide (HW) to be measured.

### Direct Analysis of Tissue Lysine Pools

Tissue homogenates (100 μl) were added to 350 μl methanol (LC–MS grade), cooled to –80 °C, and maintained on dry ice during the addition. The mixture was vortexed vigorously and centrifuged at 13,300 rpm for 15 min at 4 °C to sediment proteins. Aliquots were subsequently dried in a vacuum centrifuge and stored at –80 °C until LC–MS/MS analysis. Prior to analysis, samples were resuspended in 52 μl water (LC–MS grade), centrifuged at 13,300 rpm for 15 min at 4 °C to remove any particulates, and transferred to glass sample vials. Untargeted HPLC–MS/MS data acquisition was performed as published (26, 27, 28). Full-scan MS and data-dependent MS/MS data were acquired using a Thermo Fisher Scientific Vanquish HPLC system coupled to a Thermo Fisher Scientific Q-Exactive mass spectrometer operating in positive ionization mode as described (29). Raw instrument data (.raw files) were exported to Compound Discoverer 3.1 (Thermo Fisher Scientific) for deconvolution, alignment, and annotation, as described (29). The peak areas of [^12^C_6_]lysine and [^13^C_6_]lysine were retrieved and used to calculate the RIA.

### Postprocessing of Protein Labeling Data

**Fig. 2:**
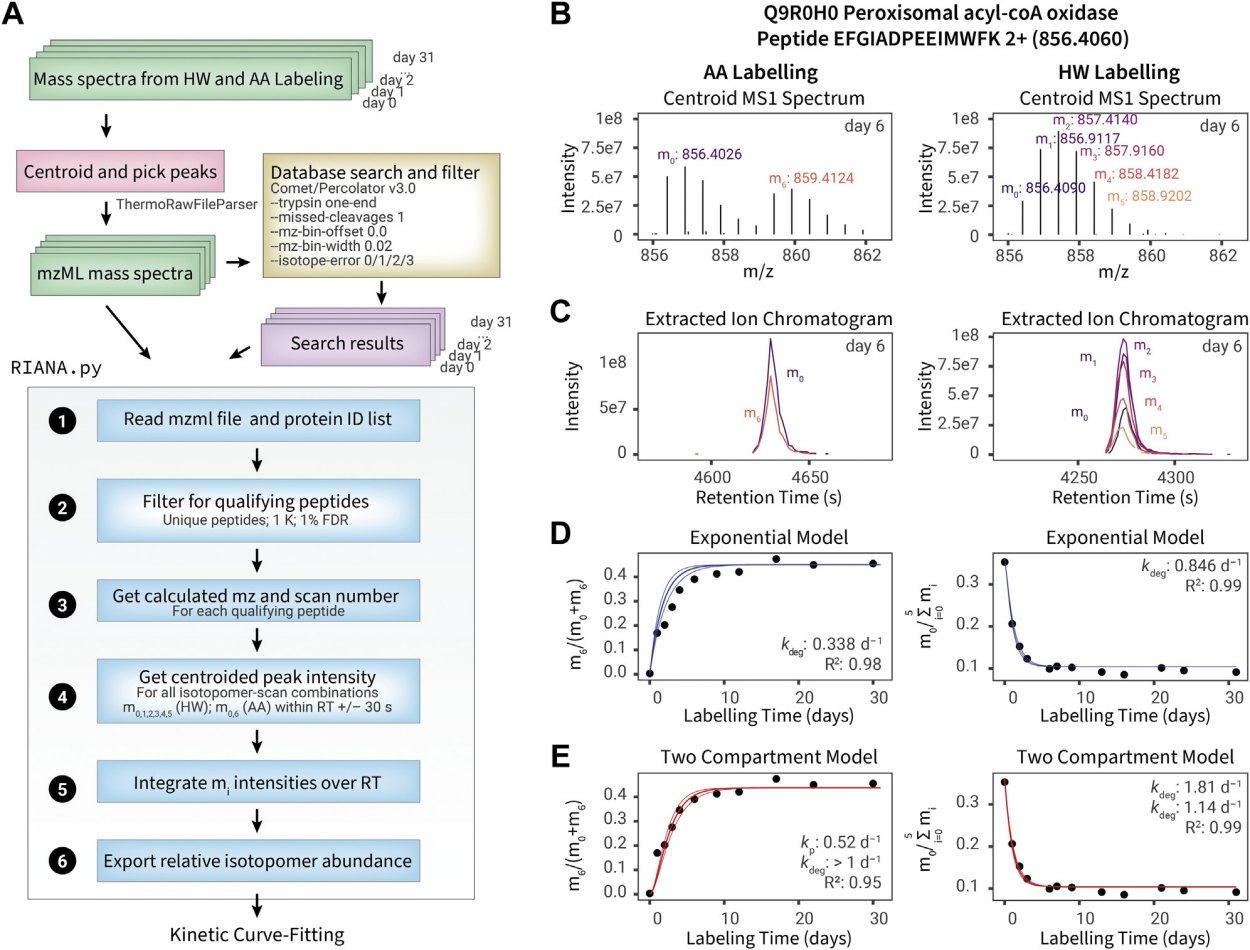
**Data analysis workflows**. *A*, schematic for the data analysis workflow for HW and AA labeling data. *B*, the MS1 spectrum for peptide EFGIADPEEIMWFK 2+ of peroxisomal acyl-CoA oxidase (Q9R0H0) for AA labeling (*left*) and HW labeling (*right*) data. For AA labeling, the intensity values for the major isotope of light (m_0_) and heavy (m_6_) versions of a peptide are measured for each MS1 scan within a specified retention time window (±30 s); for HW labeling, the intensities for each successive isotopomer within the isotopomer envelope (m_0_ to m_5_) are measured. *C*, the intensity over time values within the retention time windows are then integrated as shown in the extracted ion chromatograms for AA and HW labeling here. *D*, the data from each labeling time point are processed in the manner described previously, resulting in a peptide relative isotope abundance value for each peptide at each time point, which for AA labeling is defined as m_6_/(m_0_ + m_6_) and for HW labeling defined as m_0_/(m_0_ + m_1_ + m_2_ + m_3_ + m_4_ + m_5_). The data time series is then fitted to a simple exponential kinetics model using a quasi-Newton method to optimize for the protein turnover rate constant *k* that results in the least square error value. *E*, same as *D*, but the time-series data fitted to a two-compartment model to adjust for slow label enrichment in the animal body. The two-compartment model fits the AA data better than the exponential model and leads to a higher estimated *k*_deg_ but has a less pronounced effect on HW labeling because of fast label equilibration. The asymptote of m_0_/Σm_i_ in the HW experiment is a function of the number of accessible labeling sites on the peptide sequence and the precursor RIA and is greater than 0 because some hydrogen atoms cannot be labeled and precursor RIA is less than 1. AA, amino acid; HW, heavy water; MS, mass spectrometry; RIA, relative isotope abundance.

A summary of the workflow for data processing is given in Figure 2*A*. Thermo.raw mass spectrum files were converted to the mzML format using ThermoRawFileParser, version 1.2.0 (30). The centroid MS2 spectra were searched against the UniProt (31) *Mus musculus* reviewed database (retrieved April 27, 2021) using Comet, v.2020_01rev3 (32) with contaminant proteins and decoys appended using Philosopher, v.3.4.13 (33). Search settings include 20 ppm peptide mass tolerance, 0.02 fragment bin tolerance, 0/1/2/3 isotope error, trypsin specificity with 1 enzyme terminus (semitryptic) and two allowed missed cleavages, and +15.9949 methionine variable modifications. In addition, AA labeling experiments allowed +6.0201 lysine variable modifications. The Comet search results were postprocessed and filtered using Percolator, v.3.0.5 (34) standalone distribution with the -Y, -i 20, and -P DECOY_ arguments. Peptides identified at the 1% false discovery rate (Percolator *q* value) threshold were used for downstream analysis.

### Peak Integration

To integrate the HW and AA labeling data, we wrote an in-house Python program, Riana, v.0.6.4. Riana was written in Python (version 3.6 and greater) and accepts as input the path to the tab-delimited files generated from Percolator for each organ, labeling method, and experimental time point; and the path to the corresponding directory containing the mzML files to be integrated (Fig. 2*A*). For each qualifying peptide spectrum match, Riana reads the scan number, identified sequence, and charge from the database search result. It then strips any heavy modifications to calculate the theoretical *m/z* of the unlabeled peptide (m_0_ peak) and also calculates the theoretical *m/z* of subsequent labeled m_i_ peaks regardless of whether the heavy labeled peptide was explicitly selected for fragmentation and confidently identified. Riana uses the pymzml package, version 2.0.0 (35) to open mzML files and gathers the intensity values of the centroided peaks of all MS1 spectra for each isotopomer for each qualifying peptide within a retention time range and 25 ppm mass precision (Fig. 2*B*). It then integrates and returns the areas under curve of the isotopomer chromatograms using the trapezoid method in SciPy, version 1.6.3 (36). Additional arguments in Riana specify the *n*th isotopomer to be integrated (--iso), which was set to 0, 1, 2, 3, 4, 5, 6, 12 for both HW and AA data; the retention time window to integrate across (--rt), which was set to 0.5 min (Fig. 2*C*).

### Kinetic Models

The collated (RIA, t) series for each peptide charge combination for each fraction in each time point in each organ in each labeling method are then used for kinetic curve fitting with either a simple exponential (Fig. 2*D*) or a two-compartment (Fig. 2*E*) model. Kinetic curve fitting was performed using a custom R script written in R (version 4.1.1) running on platform x86_64-apple-darwin17.0 (64 bit). Optimization for the protein turnover rate constant *k*_deg_ was performed using the optim() function in the stats package of base R using the Broyden–Fletcher–Goldfarb–Shanno quasi-Newton method and a starting value of *k*_deg_ = 0.29. Fitting for the rate constant for precursors to reach plateau (*k*_p_) using the single exponential or Fornasiero double exponential model was optionally also performed using the nls() function with the default Gauss–Newton algorithm to retrieve the log likelihood and calculate the Akaike Information Criterion (prediction error, thus allowing model selection). Time series (*t*, *A*_*t*_) data were fitted to two models (one-compartment simple exponential *versus* two-compartment) to find the best estimate of protein turnover rate (*k*_deg_) that minimizes sums of squares of error. The one-compartment exponential model used is given by:

$$A_{t}=A_{t=0}+\left(A_{t\to \infty }-A_{t=0}\right)*\left(1-{\text{e}}^{-kt}\right)$$

Where *A*_*t*_ is the estimated time-dependent relative isotopomer abundance (RIA) of interest for a peptide under a label enrichment level of *p*, at a measured time point *t*, which for HW labeling data is defined as:

$$A_{t}=m_{i=0}/\overset{5}{\underset{i=0}{\sum }}m_{i}$$

where *m*_*i*_ is the chromatographic area under curve of the *i*^th^ isotopomer of the peptide integrated by Riana. *A*_0_ is the initial prelabeling RIA, which for HW is the isotope abundance based on natural isotope distribution calculated from Berglund and Wieser (37) and $A_{t\to \infty }$ is the asymptotic relative abundance, which for HW was defined by the number of accessible labeling sites at each AA based on tritiated water data in the study by Commerford *et al.* (38), the sequence of the peptide, and the deuterium enrichment level as previously described (15, 17). For AA labeling, *A*_*t*_ is defined as $m_{6}/\left(m_{0}+m_{6}\right)$ and *A*_0_ is 0% before enrichment, which $A_{t\to \infty }$ depends on the RIA of heavy AA in the feed using direct LC–MS measurement or by mass isotopomer distribution analysis as previously described (5). The fitting error of the one-compartment exponential model is given by:

$$dk=\frac{k\;*\;\text{e}}{\left(A_{t\to \infty }-\;A_{t=0}\right)*\sigma A}$$

Where σA is the residual error of RIA after fitting.

The two-compartment model used is given by:

$$A_{t}=\;A_{t=0}+\left(A_{t\to \infty }-A_{t=0}\right)*\;\frac{1-\left({\text{e}}^{-kt}k_{p}-\;{\text{e}}^{-k_{p}t}k\right)}{k_{p}-k}$$

Where *k*_p_ is the first-order label accumulation rate constant. The fitting error of the two-compartment model is given by:

$$dk=\;\frac{\sigma A*\;{\left(k_{p}-k\right)}^{2}}{\left(A_{t\to \infty }-A_{t=0}\right)*\left(t*\left(k-k_{p}\right)-1\right)*{\text{e}}^{\left(-t*\;k\right)}+{\text{e}}^{\left(-t*\;k_{p}\right)}*k_{p}}$$

The two-compartment three-exponent model for peptides and precursor kinetics is as described in the study by Fornasiero *et al.* (11). For precursor fitting, the model was scaled to 50% heavy lysine.

### Additional Data Analysis

Data analysis and visualizations were performed in R (version 4.1.1) unless otherwise specified. Robust correlation is performed using biweight midcorrelation implemented in the WGCNA (39) package (version 1.70-3). Data visualizations were generated with the aid of the ggplot2 (40), gganatogram (41), ggpubr (42), and plotly (43) packages in R. Kernel density estimations were performed using gaussian_kde in SciPy (version 1.6.3) (36) in Python 3.8. Peptide isotopomer integration output and R code for kinetic curve fitting have been uploaded to a runnable container at CodeOcean (https://codeocean.com/capsule/3856272/tree/v1).

## Results

For both labeling protocols (AA *versus* HW), peptides followed the expected trajectory of gradual incorporation of labels into peptides. For the AA protocol, the expected increase in intensity of [^13^C_6_]lysine terminated peptides led to clear separation between the unlabeled and labeled components of the peptide pool for all discernible isotopomers (m_0_, m_0_ + 6; m_1_, m_1_ + 6, etc). There was no evidence for partial loss of single labeled atom centers from the AA; the isotopomer profiles for labeled or unlabeled peptides are identical. For the HW strategy, the mass shift for the peptide was more subtle, evidenced as a gradual shift from the monoisotopic m_0_ pool and increased intensity of the m_1_, m_2_… m_n_ isotopomer intensities, reflecting gradual incorporation of deuterium into the peptides (Fig. 1*A*).

**Fig. 3:**
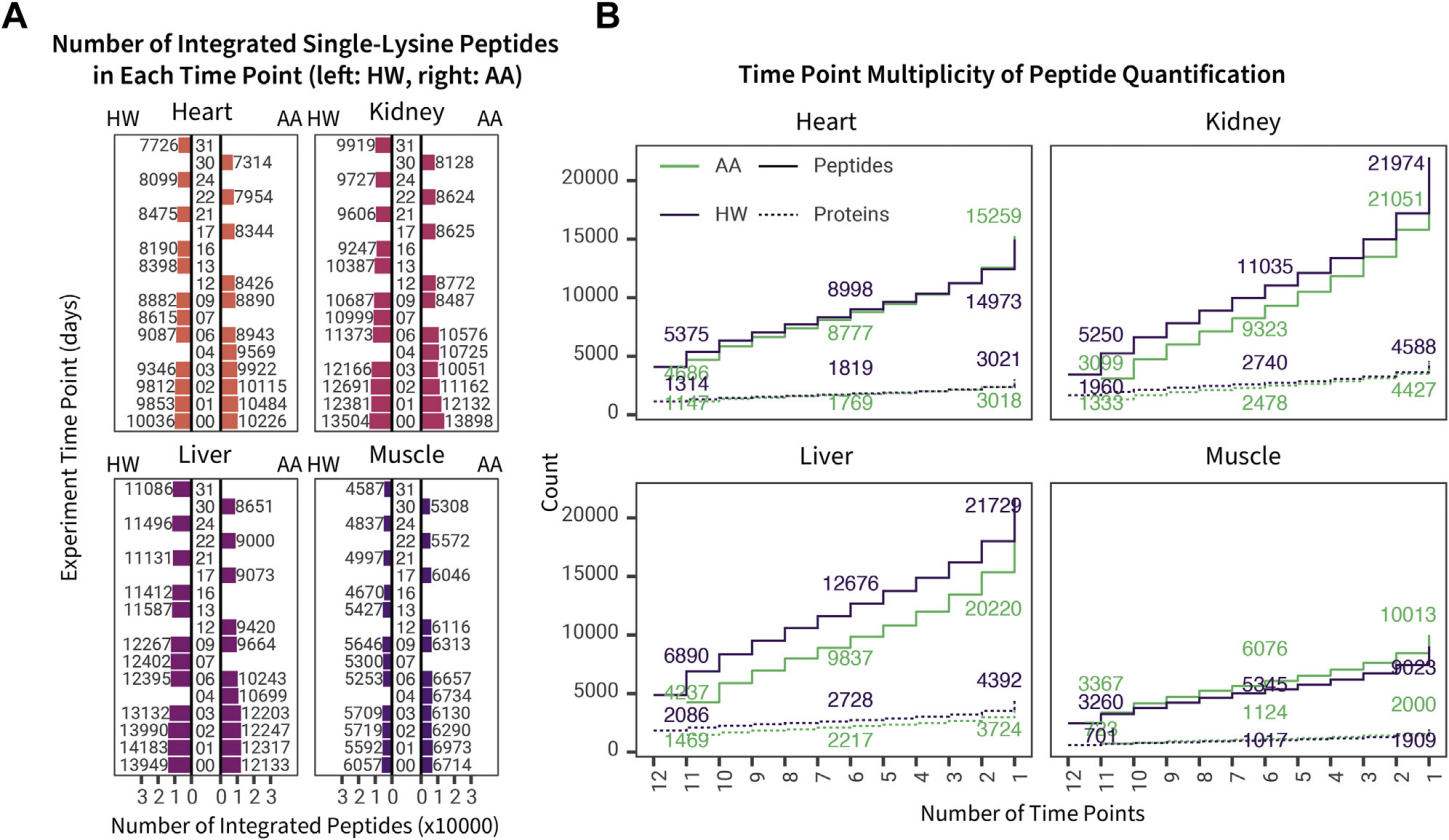
**Overall depths of the comparative analysis**. *A*, for each tissue, each bar defines the number of unique peptides that were integrated with Riana with quantified isotope ratios at each experimental time point in the labeling period. Because amino acid (AA) labeling requires peptides lysine for turnover calculation, only peptide sequences with a single lysine residue are included here for fair comparison. *B*, for each tissue, the cumulative number of peptides (*solid line*) and proteins (*dashed lines*) quantified at increasing numbers of minimal time points quantified in the heavy water (HW) labeling (*green*) and AA labeling (*blue*) datasets. For instance, at *x* = 6, the *y*-axis numbers denote the number of peptides or proteins quantified in at least six time points in a labeling method and tissue.

AA-labeled peptides conform to the kinetic model only when the peptide contains one lysine; for fair comparison, we initially filtered peptides in the HW experiment identically. With this filter, the HW and AA labeling experiments yielded similar numbers of quantifiable peptides over the entire labeling curve (Fig. 3, *A* and *B*). As we have observed previously (4), liver and kidney give the highest number of peptides, with heart intermediate and the lowest, skeletal muscle, providing about half as many peptides. We attribute this to the more pronounced dynamic range in protein expression in the two muscle tissues, such that the column loading and analysis are dominated by those proteins that are strongly expressed. There is a modest decline in the number of quantifiable peptides over the labeling trajectory, and this decline is slightly more pronounced with HW than AA labeling. Nevertheless, the results suggest that the analytical approach was able to capture isotopically labeled peptides to comparable depths in each method, regardless of the extent of labeling within the limits here. We note that HW labeling is compatible in theory with any peptide sequence, whereas AA labeling is restricted to those peptides containing that residue. When all peptides, including miscleaved peptides are admitted, regardless of the number of lysine residues in the sequence, HW labeling quantifies approximately twice the number of peptides (supplemental Fig. S1).

Both labeling protocols were extended over 30 (AA) or 31 d (HW), with the first practical sampling point being at 1 d. This labeling window imposes limits on the range of degradation rate constants (*k*_deg_) that can be recovered, further confounded by the differences in rates of precursor equilibration (*k*_p_). A protein that is extensively labeled (>80%) at 1 d would have a *k*_deg_ of at least 2 d^–1^ (half life less than 8 h). At this rate of labeling, there is no opportunity for multiple time points to define the labeling curve, and the errors in *k*_deg_ determination would be high. At the other extreme, a protein that was no more than 10% labeled at 30 d would have a *k*_deg_ of 0.003 d^–1^ or less (a half life of over 200 days), and once again, all the time points would have high errors, because of the low degree of incorporation. This is an inevitable consequence of stable isotope analysis by proteomic-compatible MS and imposes analytical restrictions on the range of rate constants that can be determined.

### Analysis of Raw Isotopomer Intensity Data by Nonlinear Curve Fitting

To compare HW and AA labeling, we first used a conventional one-compartment simple exponential model that is widely used in cell culture experiments *in vitro*, excluding any slow rise in labeling kinetics. The asymptotic values of the labeled RIA for each method are reflected in the labeling plateau of peptides, which is set to 0.45 for AA labels and 0.046 for HW labels for this analysis (see later). To minimize uncertainty of isotopomer quantification, we used a conservative filter to admit only peptides quantified at nine time points and/or greater and that fitted to the one-compartment model with *R*^2^ of ≥0.9 unless specified. For the one-compartment model, the best-fit peptide *k*_deg_ values from HW and AA labeling are concordant (biweight midcorrelation ≥∼0.75). However, AA labeling generally reported lower peptide turnover rates compared with HW, especially apparent for peptides from proteins with relatively high turnover within a tissue (supplemental Fig. S2).

As stated earlier, a major difference between labeling with water and a free AA in the diet is the rate at which the precursor pool equilibrates. The AA data within a tissue, particularly for relatively high turnover peptides, when compared with HW labeling, suggest that AA data require a kinetic model that acknowledges this delay in equilibration in preference to a simple one-compartment model that assumes near instantaneous equilibration of label precursor pool. We therefore investigated the application of a two-compartment model to fit the HW and AA data. In the two-compartment model described by Guan *et al.* (23), peptide isotope enrichment is described using two rate constants: the protein turnover rate *k*_deg_ and a composite rate constant that encompasses precursor availability kinetics; *k*_p_. This model therefore requires knowledge of the value of *k*_p_ in each tissue.

**Fig. 4:**
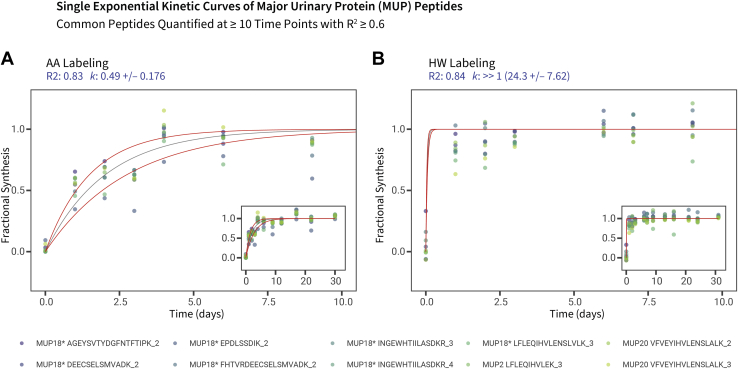
**Isotopic labeling of major urinary proteins (MUPs) in the liver**. Single exponential kinetic curves for MUP peptides commonly quantified at ≥10 time points in both the (*A*) AA and (*B*) HW datasets. To compare HW and AA data in the same scale and because each peptide in HW labeling has different initial and plateau m_0_/m_i_ values, the peptide isotope relative abundance data are normalized to 0 (before labeling) and 1 (plateau) as fractional synthesis. Peptides fitted at *R*^2^ ≥ 0.6 at the peptide level were combined in the fractional synthesis space then fitted to a single kinetic curve to estimate the overall MUP *k*_syn_. Because MUPs are secreted from the liver as soon as they are synthesized, the quantified label trajectory is assumed to be limited only by precursor availability. As expected, in AA labeling, the MUPs reflect delayed precursor kinetics with *k*_p_ of ∼0.49; whereas precursor kinetics is rapid in HW labeling with *k*_p_ >> 1. *x*-axis: time (days); *y*-axis: fractional synthesis. Main panels show expanded views from day 1 to day 10 of labeling, *insets* show the full data range. *Asterisks* after protein names denote peptide sequences present in multiple MUPs. *Red lines* denote models of best-fit first-order rate constant ± fitting error. The curve plotted for HW labeling cannot be construed as an accurate fit but reflects the rapidity of HW incorporation. AA, amino acid; HW, heavy water.

A clear indication of the behaviors of the HW and AA precursors can be gleaned from the labeling trajectories of the major urinary proteins (MUPs). MUPs are synthesized in large quantities in the liver and are immediately secreted and exported into the circulation, efficiently filtered by the glomerulus and excreted into urine, where they play multiple semiochemical roles (44, 45, 46). Because of the speed of this secretion and the lack of any intermediate protein pool (MUPs are very difficult to detect in plasma), the isotopomer signatures of MUPs in the liver at any time point should reflect new synthesis and rapid secretion and thus act as efficient and high-speed sensors of the precursor enrichment (47). We fitted the MUP peptide data to an exponential model with isotope relative abundance of each peptide represented in the same scale as fractional synthesis (*i.e.*, total fraction of protein pool with the isotope tracer signature). For HW-labeled MUPs, the proteins acquire label extremely rapidly, with an average rate constant (*k*_p_) of at least 2 d^–1^—the rapidity of labeling precludes accurate measurement of the true rate constant, but in the context of this system, it can be considered to be near instantaneous. By contrast, for AA-labeled MUPs, the rise to plateau was notably slower, yielding a *k*_p_ of approximately 0.5 d^–1^ (half time of about 1.4 d). If we take the AA-derived value to most closely reflect precursor behavior (because of the lack of an intracellular pool), then we can compare the AA *k*_p_ rate constant with those obtained by the other approaches. Unfortunately, in the absence of true secreted proteins from other tissues that do not mix into a pre-existing pool, this insight is restricted to the liver (Fig. 4).

**Fig. 5:**
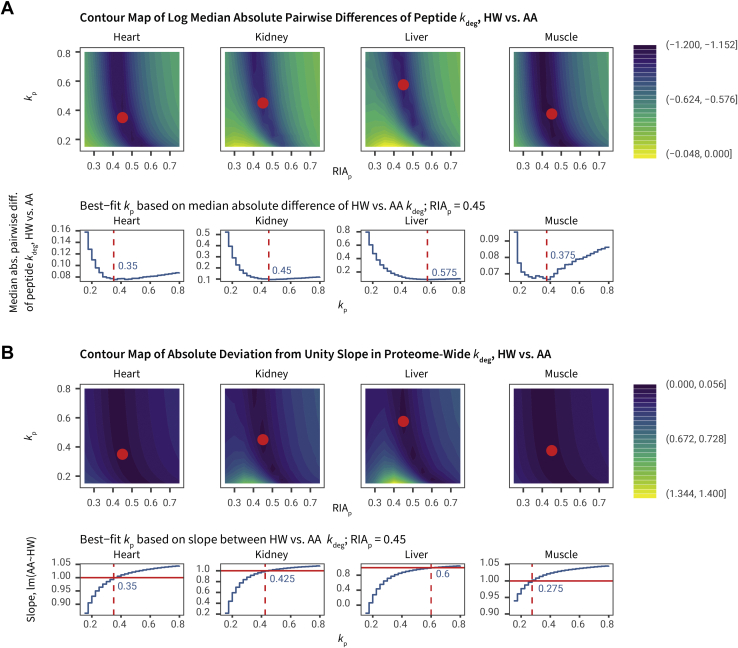
**Calibrating AA labeling precursor kinetics using HW-derived rate constants.***A*, 2D density plot of the log median absolute pairwise differences in peptide *k*_deg_ from HW *versus* AA labeling data, against different values of plateau RIA_p_ (*x*-axis) and *k*_p_ (*y*-axis). *Red dots* show the *k*_p_ values with minimal HW–AA differences at RIA_p_ = 0.45, also shown in the *blue numbers* in the line plots below. Common peptides in HW and AA labeling with one lysine are analyzed. *B*, 2D density plot of the absolute deviation from unity slope in proteome-wide *k*_deg_ from HW *versus* AA labeling data, against different values of plateau RIA_p_ (*x*-axis) and *k*_p_ (*y*-axis). *Red dots* show that in the values with minimal HW–AA differences (as in *A*), the slope of proteome-wide log HW *versus* AA *k*_deg_ values approach 1. *Blue numbers* in the line plots below show the *k*_p_ values where the slope between HW and AA *k*_deg_ is nearest to 1. AA, amino acid; HW, heavy water; RIA_p_, precursor RIA.

At the same time, because we collected both HW and AA labeling data, our experimental design allowed us to search for suitable *k*_p_ values that would bring the AA labeling data into concordance with the HW labeling data (Fig. 5*A*). If we assume the HW labeling data to be more accurate for our purpose, because of the rapid precursor equilibration of water, this would suggest that a “corrected” *k*_p_ for AA labeling is 0.28 to 0.38 d^–1^ for heart and skeletal muscle and 0.43 to 0.60 d^–1^ for liver and the kidney, values substantially higher than those acquired experimentally using LC–MS measurements of free lysine residues in this study (see the later paragraph). With these values of *k*_p_, the proteome-wide slope of log *k*_deg_ across shared peptides in HW and AA labeling approaches unity (Fig. 5*B*). Therefore, although a two-compartment model is sufficient to describe the behavior of AA labeling data with precursor delay, it is not clear *a priori* how to produce the requisite *k*_p_ values to accurately describe tissue-specific precursor kinetics. Because the HW labeling–derived *k*_p_ values require the external reference of HW data, they are unsuitable for experiments where only AA labeling is performed. We therefore explored additional approaches that could provide a self-sufficient estimate of the precursor kinetics parameters for two-compartment modeling of AA labeling. There are at least three methodological approaches by which this may be achieved, including (1) direct empirical measurements of label in the subject; (2) a data-driven iterative approach to find the *k*_p_ that best explains all peptide data in a two-compartment model fitting; and (3) calculation of RIA using mass isotopomer distribution analysis from peptides containing two labeling sites (*i.e.*, dilysine peptides).

We first experimentally measured the precursor enrichment using GC–MS and LC–MS method. For HW labeling, we measured whole-body water RIA enrichment using GC–MS by sampling plasma water (supplemental Fig. S3*A*). As water equilibrates rapidly across body compartments (18), it is assumed that the precursor kinetics for HW would be similar across tissues. The same assumption, however, cannot be made for AA labeling. We therefore measured label enrichment of free lysine from each tissue using LC–MS to assess AA labeling precursor enrichment (supplemental Fig. S3*B*). The GC–MS and LC–MS data allowed estimation of the plateau precursor RIA (RIA_p_) values of 0.45 for AA labels and 0.046 for HW labels (supplemental Fig. S3, *A* and *B*). The LC–MS data determined that lysine RIA enrichment curves in the four tissues vary between tissues, and we find slower precursor kinetics in the heart and the skeletal muscle, tissues that have lower metabolic turnover. The precursor RIA did not reach a true plateau until after 30 days. Remarkably, this is incompatible with the peptide RIA data, as the precursor enrichment cannot be slower than the peptide turnover curve. This discrepancy may be due to lysine metabolism complications or the inability to access the true protein synthesis precursor pool of lysine within the tissues. In fact, the LC-derived AA *k*_p_ values limit the rise of peptide RIA in the two-pool model with the consequence that the model cannot converge and does not explain the observed peptide RIA time series (supplemental Fig. S3*C*). We conclude that the LC-measured AA RIA as implemented underestimates true precursor enrichment rates and is unsuitable for explaining the peptide RIA curves and correcting for precursor delay.

We next assessed whether *k*_p_ could be gained directly from the peptide RIA data using a proteome-wide optimization approach, that is, locate a *k*_p_ value that gives the lowest sums of squares (fitting error) in all peptides from kinetic curve fitting to the two-compartment model. At very high *k*_p_, the two-compartment model approaches the one-compartment model, whereas an underestimated *k*_p_ will prevent the theoretically allowable peptide (t, RIA) values from ever reaching their actual experimental measurements within the experimental time frame. Hence, the goal is to find the lowest *k*_p_ that explains the data points better in a two-compartment model compared with the one-compartment model, using the median fitting sums of squares of all qualifying peptides as the target. We had limited success with this nested optimization approach, and the results suggested that the strategy for finding best-fit precursor kinetics parameters will be tissue specific. In the two low-turnover/slow-equilibration tissues (heart and skeletal muscle), the two-compartment model outperformed the one-compartment model at a *k*_p_ of ∼0.25 to 0.35 d^–1^, thus this model can be used to find precursor *k*_p_ in slow-equilibrium tissues. On the other hand, the two-compartment model never fitted the data better than the one-compartment model in the liver or the kidney (supplemental Fig. S4, *A* and *B*). Conversely, proteome-wide optimization over the fast-equilibration tissues allowed the effective plateau RIA_p_ in these tissues to be estimated directly from the data, which was not possible in the slow-equilibration tissues (heart and skeletal muscle). We interpret the results to suggest that although a two-compartment model is necessary to correct for labeling delay, the precise values of *k*_p_ cannot be easily found in fast equilibration tissues as different combinations of *k*_p_ and *k*_deg_ yield identical kinetic curves. Conversely, in some tissues, the best-fit *k*_p_ learned from the data will not necessarily give accurate absolute values of peptide *k*_deg_.

Finally, we estimated precursor RIA over time using mass isotopomer analysis with dilysine peptides. This is a commonly used method in dynamic stable isotope labeling by AA studies in animals, where the heavy–heavy and heavy–light peaks of a peptide containing two labeled AAs is used to reveal the true precursor RIA during the time when the peptides were made (5). However, there is no commonly accepted standard for selection of the dilysine peptide(s) for this calculation. We therefore calculated the precursor RIA, restricted to all dilysine peptides quantified at a minimum of nine time points in each tissue. Whilst there is a noticeable increase in estimated precursor RIA as labeling proceeds (supplemental Fig. S5*A*), the calculated precursor RIA values from each peptide have high variance, especially at earlier time points. Using a Gaussian kernel density estimate, we estimated the mode RIA at each time point as the representative tissue precursor RIA (supplemental Fig. S5*A*). The resulting tissue RIA estimates fitted well to single exponential curves. However, the derived *k*_p_ values remain lower than required to explain the peptide curves in AA labeling or the MUP-derived prediction, and the curve fitting is unable to converge to a satisfactory solution for a number of peptides.

As an alternative to kernel density estimates, we used all qualifying tissue-wide RIA_p_ values (dilysine peptides identified at ≥9 time points, 0 ≤ RIA ≤ 0.6) to define a single exponential kinetics model using weighted nonlinear least square fitting across each tissue to estimate the precursor rate constant and plateau (supplemental Fig. S6). The contribution of each RIA data point to the fitted curve is weighted by the square of the peptide isotopomer normalized intensity. This approach produces *k*_p_ values that align well with those derived from HW adjustment. For the liver, the MUP-derived *k*_p_ was essentially the same as the dilysine recovered parameter (0.49, *cf.* 0.52 d^–1^).

Because the RIA curve in the weighted fitting exhibited biphasic behavior, we also fitted the tissue RIA curves to the two-exponent kinetic model described by Fornasiero *et al.* (11), which accounts for label dilution from global protein degradation, using three parameters, the soluble precursor enrichment/breakdown rate constant, *b*, the global protein degradation rate constant, *a*, and the ratio of lysine pool in soluble *versus* protein-bound pools in a tissue, *r* (supplemental Fig. S6). Reutilization, represented by *a*, contributes to the slow phase precursor rise following the initial plateau as the reutilized lysine residues originating from protein degradation products slowly become labeled. The two-exponent kinetic model fitted to dilysine peptides derived RIA values significantly better than the single-exponent model, accounting for the extra number of parameters (Akaike weights 1 to 9e–44 in the heart; 1 to 1e–27 in the kidney; 1 to 1e–27 in the liver; and 1 to 9e–18 in the muscle). When incorporated into a two-compartment three-exponent model (*a*, *b*, and *k*_deg_) for individual protein *k*_deg_ values, the method by Fornasiero *et al.* (11) yielded results comparable to the two-compartment two-exponent model in the study by Guan *et al*. (23) for slow-turnover peptides within a tissue but was able to correct for fast-turnover proteins within a tissue in the AA labeling experiments (supplemental Fig. 7, *A* and *B*). Both two-compartment models led to higher intraprotein variance (supplemental Fig. 7*C*) than the single exponential model (see the later paragraphs) across various *R*^2^ cutoffs. The different methodologies and resultant values for various precursor kinetics parameters are summarized in Table 1.

**Table 1:** Methods for determination of AA labeling precursor kinetics

Method	Details/description	Heart	Kidney	Liver	Muscle
MUP secretion	Estimation of label delay in rapidly secreted proteins (MUPs) from the liver with no pre-existing pools (using RIA_p_ set to 0.45)	*—*	*—*	*k*_p_: 0.49 ± 0.18	*—*
Experimental	Measurement of free tissue lysine pool using LC–MS (using RIA_p_ set to 0.45)	*k*_p_: 0.10 ± 0.04	*k*_p_: 0.12 ± 0.02	*k*_p_: 0.20 ± 0.04	*k*_p_: 0.06 ± 0.02
Global optimization to two-compartment model	Using nested optimization, learn from the data the *k*_p_ values that minimize global fitting errors in the two-compartment model described in the study by Guan *et al.* (23)	*k*_p_: ∼0.35	*—*	*—*	*k*_p_: ∼0.25
KK peptides—one-pass fitting	Calculate RIA from m6 and m12 peaks of dilysine peptides at each time point, then fit all qualifying data points to a single exponential rise curve	*k*_p_: 0.37	*k*_p_: 0.58	*k*_p_: 0.52	*k*_p_: 0.31
		RIA_p_: 0.37	RIA_p_: 0.38	RIA_p_: 0.42	RIA_p_: 0.35
KK peptides—one-pass fitting to two-exponent precursor model	Calculate RIA from m6 and m12 peaks of dilysine peptides at each time point, then fit all qualifying data points to the double exponent model described in the study by Fornasiero *et al.* (11) that accounts for label dilution from proteome-wide degradation	*a*: 0.12	*a*: 0.11	*a*: 0.15	*a*: 0.08
		*b*: 0.84	*b*: 1.37	*b*: 1.05	*b*: 0.42
		*r*: 9.08	*r*: 11.2	*r*: 5.52	*r*: 6.04
KK peptides—best estimate RIAs	Calculate RIA from m6 and m12 peaks of dilysine peptides, find the estimated tissue RIA at each time point using kernel density estimation, and then fit to a simple exponential rise curve	*k*_p_: 0.19	*k*_p_: 0.37	*k*_p_: 0.30	*k*_p_: 0.16
		RIA_p_: 0.39	RIA_p_: 0.43	RIA_p_: 0.42	RIA_p_: 0.36
KK peptides—median *k*_p_	Calculate RIA from m6 and m12 peaks of dilysine peptides, fit each peptide time series to an exponential rise curve to derive, and then take the median of all best-fit *k*_p_ values	*k*_p_: 0.17	*k*_p_: 0.30	*k*_p_: 0.33	*k*_p_: 0.12
HW reference	Using nested optimization to the two-compartment model described in the study by Guan *et al.* (23) for AA, find the *k*_p_ that minimizes total error between HW and AA labeling results	*k*_p_: ∼0.35	*k*_p_: 0.44–0.45	*k*_p_: 0.58–0.60	*k*_p_: 0.28–0.38

*k*_p_, precursor RIA kinetics rate constant; RIA_p_, asymptotic precursor RIA.

The parameters *a*, *b*, *r* in the three exponent Fornasiero model are independent parameters used to calculate peptide isotope incorporation rate constants and relate to global protein degradation, soluble precursor pool exchange, and the ratio of protein bound and soluble precursors as given in the study by Fornasiero *et al.* (11).

From these analyses, and using the MUP-derived parameters as ground truth, we conclude that correction for slow equilibration (whether caused by slow uptake or reutilization) is feasible, and that analysis of dilysine peptides, two-pool modeling, or reutilization correction give the best estimates.

### Comparison of Peptide Turnover Rates Between HW and AA Labeling

**Fig. 6:**
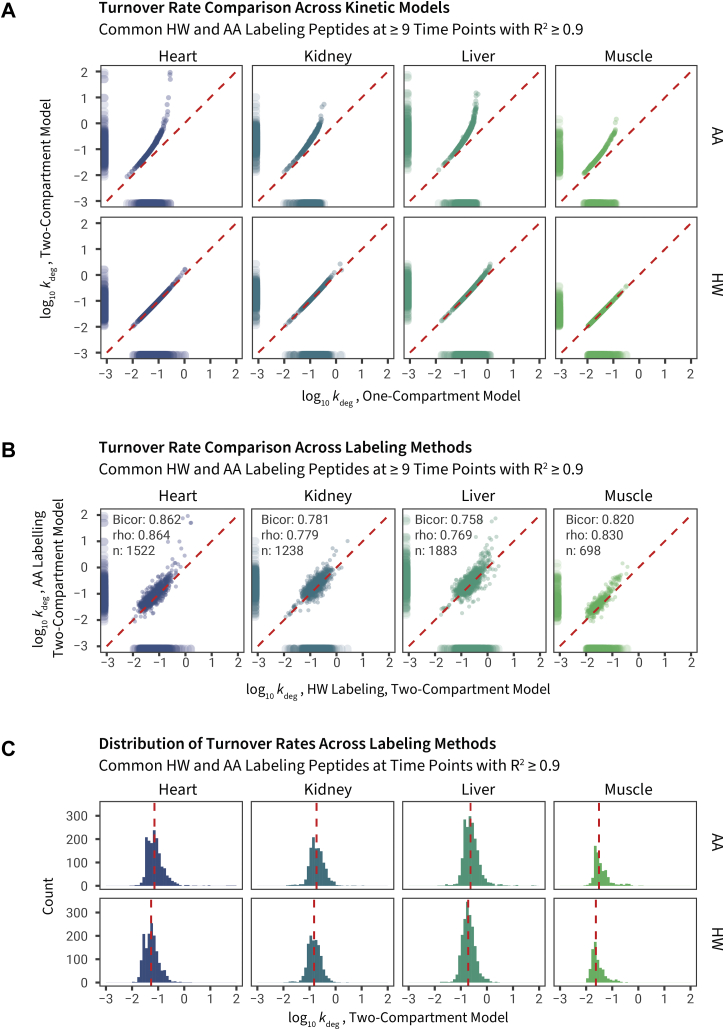
**Comparisons of turnover rate constants across labels and kinetic models**. *A*, degradation rate constants (*k*_deg_) were obtained for peptides from four tissues using amino acid (AA) or heavy water (HW) labeling and were fitted using a one-compartment (*x*-axis) or two-compartment (*y*-axis) kinetic model to derive the first order *k*_deg_ (plotted on a log base 10 scale). Data points are peptide time series commonly quantified in HW and AA experiments containing 1 lysine and with ≥9 time points and fitted with *R*^2^ ≥ 0.9. *Red dashed line*: unity. Marginal distribution shows data density. *B*, scatterplots of shared peptides quantified by HW and AA in each tissue using the two-compartment model (quantified time points ≥9, *R*^2^ ≥ 0.9, 1 lysine). *Numbers* denote robust correlation (biweight midcorrelation; bicor) coefficients, Spearman correlation coefficient (rho), and number of comparisons (n). Each data point is one peptide-charge time series. *C*, histogram showing distribution of *k*_deg_ across tissues and labels. *Red dashed lines* denote medians.

We next used the weighted dilysine peptide *k*_p_ and asymptotic RIA_p_ values in a two-compartment two-exponent model to derive peptide *k*_deg_ for all qualifying single lysine peptides in each tissue. We were thus able to fit the AA (slow equilibration) and HW data (rapid equilibration) using either model for comparison. We compared the best-fit peptide *k*_deg_ from the one-compartment model to a two-compartment model that accounts for the delay in precursor equilibration. As anticipated, the two-compartment model influenced the kinetics of the AA labeling experiment more than HW labeling, particularly for higher turnover proteins within a tissue. This is evident from the off-diagonal distribution between one-compartment and two-compartment models in AA labeling as well as the absolute differences in high-turnover peptides (Fig. 6*A*).

Fitting to the two-compartment model brought the median turnover rates of peptides quantified from the two methods into closer correspondence (Fig. 6*B*) and corrected the discrepancy between HW and AA labeling in relatively high turnover proteins within a tissue (Fig. 6*C*). For peptides integrated over at least nine time points and fitted with an *R*^2^ of ≥0.9, the agreement between the methods is good (robust correlation bicor: 0.758–0.862 in four tissues) when the two-compartment model is used to fit the (peptide RIA, t) data. Both AA and HW labeling data showed excellent agreement with the turnover rates derived in a previous study of HW labeling of heart proteins in an independent cohort of C57BL/6J mice (15) (biweight midcorrelation: 0.85–0.93; supplemental Fig. S8*A*). In addition, we also observed good agreement with a more recent study of AA labeling of liver and skeletal muscle proteins in NSBGW mice by Rolfs *et al.* (24) (biweight midcorrelation: 0.73–0.83; supplemental Fig. S8, *B* and *C*), however, although the rank of peptide *k*_deg_ values appear highly similar, a lower overall range of values was reported in the study by Rolfs *et al*., which we hypothesize may be due to differences in fitting strategies and RIA plateau rather than intrinsic differences in the data. Given there are several approaches to data modeling and they do not fully agree, this may represent a fruitful subject for a future community-wide comparative study. The *k*_deg_ values of peptides in HW and AA labeling are tabulated in supplemental Data S1. All fitted peptide curves using the aforementioned two-compartment model in AA and HW labeling are provided in supplemental Data S2–S5.

A few peptides showed unexpectedly high turnover rates in AA labeling, possibly because the precursor enrichment rate is underestimated, leading to overcorrection. For these peptides, it is likely that *k*_deg_ could not be determined with high accuracy as the nonlinear model would fail to converge when it is limited by *k*_p_, and these peptides may be more accurately categorized simply as having high turnover. Nevertheless, we conclude that the two-compartment model performed well in correcting the underestimation of *k*_deg_ for relatively high-turnover proteins (*k*_deg_ ≥1 d^−1^); proteins for which label enrichment is retarded measurably by the lag in precursor enrichment (∼1 d^−1^). This is more pronounced in tissues where the overall protein turnover rates are high, such as the kidney and the liver.

### Evaluation of Data Quality Filters

Using the one- and two-compartment model results, we examined the effect of various data quality filters on the depth and reliability of protein turnover measurements. We varied the filtering criteria by two metrics: first, the number of time points in which the degree of label incorporation into protein was quantifiable; and second, the coefficient of determination (*R*^2^) of the one-compartment and two-compartment kinetic models. We then considered their effects on interaction with the number of quantifiable peptides and the precision of turnover rates. To estimate precision, we considered the median values of the geometric CV in turnover rates among peptides uniquely mapping to the cognate protein, because peptides derived from the same protein should be synthesized and degraded together *in vivo.* Barring undocumented proteoforms or post-translational processing, peptides from the same protein should yield identical turnover rates, if the measurement is precise.

**Fig. 7:**
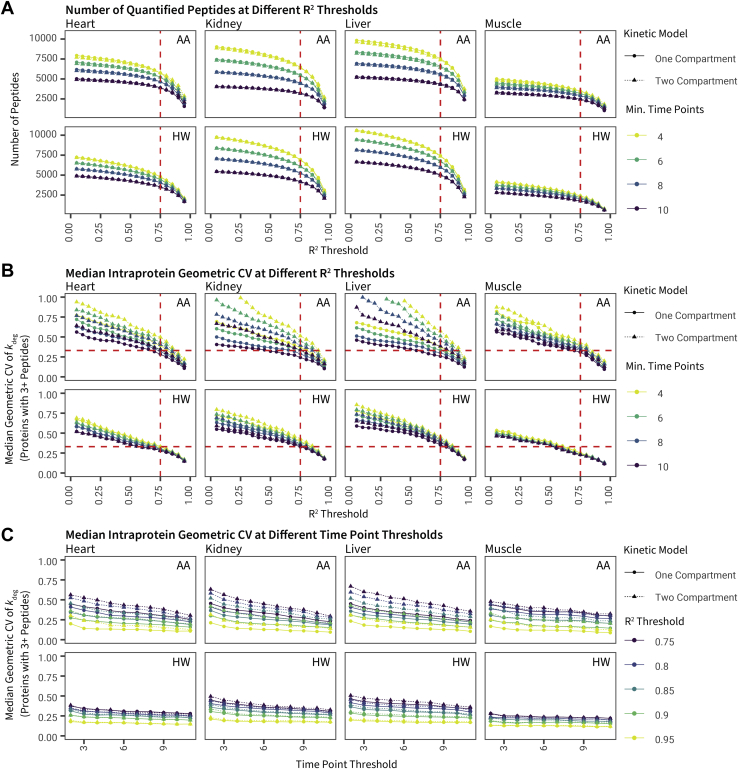
**Relationship between *R***^**2**^**and time point filters on peptide count and variance.***A*, the number of quantified peptides (*y*-axis) *versus R*^2^ coefficient-of-determination thresholds in kinetic curve fitting (*x*-axis) with various time point filters (*colors*). Both *R*^2^ and minimal time points have a large effect on the total number of fitted peptides. In all panels, only peptides with one lysine are included for both HW and AA. *Red dashed lines*: *R*^2^ = 0.75. *B*, intraprotein variance, measured as the geometric coefficient of variation (CV) of best-fit *k*_deg_ among peptides uniquely mapped to the same proteins (*y*-axis) *versus R*^2^ thresholds (*x*-axis) and time point thresholds (*color*). Only peptides belonging to proteins with three or more quantified peptides were used for the analysis. Two-compartment models led to higher intraprotein variance. *Horizontal red dashed lines*: geometric CV = 0.33; *vertical red dashed lines*: *R*^2^ = 0.75. *C*, intraprotein variance (*y*-axis) as in *B*, against the minimal number of required time points, at different *R*^2^ thresholds. It can be seen that *R*^2^ has a more pronounced effect on *k*_deg_ precision than minimal time point thresholds. AA, amino acid; HW, heavy water.

First, we observed an expected decrease in the number of available peptides as the thresholds for required time points and *R*^2^ were raised. There was a sharp decrease in quantifiable peptides at all time point cutoffs when the *R*^2^ threshold increased beyond ∼0.8, suggesting a drastic decrease in profiling depth if too stringent a threshold is used (Fig. 7*A*). Although the number of quantifiable peptides between the one-compartment and two-compartment models at each *R*^2^ threshold is similar, there is a noticeable difference in intraprotein geometric CV. We found that the two-compartment model led to increased variance at all *R*^2^ cutoffs. This increase was especially noticeable in fast equilibration tissues (liver and kidney) and is especially severe in AA labeling (Fig. 7*B*). This is probably attributable to the two-compartment model having more parameters that can vary. For example, true *k*_p_ may differ across cell types within a tissue, demanding more stringent time-point and *R*^2^ thresholds. In our experience, data quality and the ability to make inference about changes in turnover declined when the median geometric CV increased beyond ∼0.33.

When an *R*^2^ threshold of 0.8 to 0.95 is imposed, the inclusion of peptides quantified at fewer time points had a modest impact on intraprotein *k*_deg_ variance (Fig. 7*C*) while allowing more quantifiable peptides (Fig. 7*A*). Overall, we surmise that with a two-compartment model, a conservative *R*^2^ threshold is needed to minimize intraprotein variance, whereas the number of quantifiable data points has a lesser impact. Hence, we selected an *R*^2^ threshold of 0.9 and a time point threshold of 6 to investigate proteome-level features of protein turnover in the four tissues.

A relevant question is what factors contribute to quantitative errors at the peptide and protein levels across the two labeling methods. Some indication of the extent of quantitative variance can be gleaned from peptides that differentially ionize into multiple charged variants, such as [M + 2H]^2+^ or [M + 3H]^3+^. These should be labeled identically, but because they occupy different segments of the *m/z* dimension, they would be differently affected by background/other ions. As an alternative approach, we note here that the quantitative errors of HW labeling at the individual data point level may also be compared with AA labeling by considering the empirically derived fractional synthesis at day 0. Fractional synthesis is derived using the linear changes in peptide isotopomer profile from day 0 (prior to labeling, fractional synthesis of 0) to the plateau value when only labeled peptides exist (fractional synthesis of 1). For day 0 samples prior to label commencement, true fractional synthesis is expected to be 0, and deviation from 0 in the empirically derived values should then reflect quantitative errors. Among peptides integrated at ≥6 time points and whose entire peptide time series (all time points) are fitted to the two-compartment model with *R*^2^ of at least ≥0.5, it can be seen that HW labeling is associated with a wider distribution of derived day 0 fractional synthesis values than AA labeling (supplemental Fig. S9*A*). This distribution becomes narrower as only peptides passing more stringent *R*^2^ thresholds (≥0.9 or 0.95) are included such that the interquartile range of derived fractional synthesis is within 5% from 0, compared with almost 10% at *R*^2^ ≥0.5, hence it appears that quantitative errors at the individual isotopomer level contributes at least partially to model fitting variance in HW labeling. As may be expected, the precision and accuracy of isotopomer quantitation increases for peptides with higher intensity (supplemental Fig. S9*B*) for both HW and AA labeling. There appears to be a bias toward a slight negative fractional synthesis value in low-abundance peptides in HW labeling, which would be indicative of an under-reporting of m1 to m5 peaks that arise from naturally occurring isotopes for low-abundance peptides in the MS experiments. Nevertheless, the quantitative precision at the isotopomer level is compensated by kinetic modeling such that both methods had comparable precision at the peptide (Fig. 7) and protein levels (see later). This analysis also excludes a source of error in AA labeling, where some peptides have high day 0 m6 intensities because of interfering isobaric ions (data not shown). These factors should be further examined in future work to improve data quality and completeness.

### Protein-Level Data Summary and Quantitative Precision

Finally, we aggregated peptide-level turnover rates to the protein level to compare rate constants from different labeling or aggregation methods. In the aforementioned analyses, to avoid complications from different constituencies of peptides for the two labeling methods, the peptide-level comparisons are, where appropriate, based on the same peptide, labeled by either the HW or the AA protocol. This is perhaps the most stringent comparison of the two labeling methods, which is a primary purpose of this article. The nature of the proteomics workflow means that we obtain a peptide level measure of incorporation, rather than a direct protein-centric result. This raises the issue of how we aggregate the peptide-level measurements into a protein parameter. There are two common classes of approaches—the first is based on curve fitting to the (time, RIA) data for each peptide and then aggregating these peptide-sourced *k*_deg_ values into a protein *k*_deg_ value. Alternatively, all (time, RIA) data, from all peptides, could be combined into a single curve-fitting optimization. In the first approach, it is essential that the time course is represented by time points across the labeling period; otherwise, the fitting can be very poor and lead to spurious outliers. In the second approach, we can aggregate all time points, irrespective of the completeness of the labeling curve for any one peptide.

We therefore examined four methods where peptide turnover rates may be reduced to a single protein turnover rate. First (peptide median), the protein *k*_deg_ is taken as the median of the peptide *k*_deg_ of all qualifying constituent peptides that map uniquely to the protein in the protein sequence database, and the median absolute deviation is used as a measure of variance. Second (peptide harmonic mean), the harmonic mean and harmonic mean standard deviation of the *k*_deg_ of qualifying unique constituent peptides are used to calculate the protein *k*_deg_. Third (refitting), the isotope enrichment over time data of all qualifying unique constituent peptides are combined in fractional synthesis space, and optimization to a single kinetic curve is performed using all data points. This yields a protein level *k*_deg_ that best fits the model to the data, and the fitting error is reported. Fourth (weighted refitting), as aforementioned, but the optimization in addition accounts for normalized log peptide intensity for weighted least square calculations.

In our observation, protein-level refitting (methods 3 and 4) is more prone to error propagation from the peptide level such that if a peptide that fits poorly to the kinetic model is admitted to the protein level, the quality of curve fitting at the protein level decreases dramatically as measured in *R*^2^ and fitting error. We therefore selected only well-fitted peptides (*R*^2^ ≥ 0.9, six time points) based on the aforementioned analyses for comparisons. The *k*_deg_ values, error of best-fit values (*dk*_deg_), and *R*^2^ of proteins from the refitting methods, as well as the *k*_deg_ and variance of the median and harmonic mean methods for each protein are tabulated in supplemental Data S6. With the peptide-level quality filter in place and comparing proteins with at least two constituent peptides, there was excellent rank similarity between the four methods (bicor 0.93–0.99; Spearman's rho: 0.95–1.00) (supplemental Fig. S10*A*). There is a slight bias toward higher turnover rates in protein-level refitting when compared with peptide median summary (supplemental Fig. S10*B*). Weighted and nonweighted fitting produced virtually identical rate constants, when applied to the aforementioned two-compartment model in AA and HW labeling, which can be seen both from summary protein *k*_deg_ data (supplemental Fig. S11*A*) as well as in individual protein-level kinetics curves (supplemental Data S7–S10). The harmonic mean summary appears to lead to lower variance than the median, and both lead to lower apparent protein-level variance than refitting (supplemental Fig. S11*B*). This may be due to a combination of both the loss of individual peptide-level variances in the summary calculations as well as the nonlinear relationship between *k*_deg_ and isotopomer enrichment in kinetic curve fitting, where relatively small changes in curve position can lead to dramatic differences in *k*_deg_ when the kinetic curve rises sharply.

**Fig. 8:**
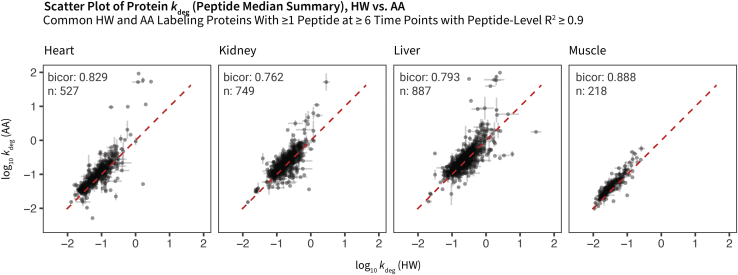
**Protein level data summary.** Scatterplot showing data distribution of log_10_ protein turnover rates in HW (*x*-axis) and AA (*y*-axis) labeling, represented as the median of best-fit *k*_deg_ values from qualifying peptides. Proteins with 1 or more common peptides with one lysine in HW and AA labeling integrated at ≥6 time points with peptide-level *R*^2^ ≥ 0.9 are compared. *Red dashed lines*: unity. Error bars represent median absolute deviation where a protein has two or more peptides. Peptide-level fitting error for proteins with only one constituent peptide is not shown. Bicor, biweight midcorrelation coefficient; n, number of proteins compared. AA, amino acid; HW, heavy water.

Taking the median peptide *k*_deg_ value as the aggregation method, we further compared the turnover rates of HW and AA labeling at the protein level using the two-compartment model. The overall agreement between HW and AA labeling at the protein level is comparable to the peptide level (Fig. 8). Moreover, at both the peptide and protein levels, HW and AA labeling achieved overall comparable quantitative precision as determined from *dk*_deg_ over *k*_deg_ at the peptide fitting level, or median absolute deviation over median of *k*_deg_ at the protein level, for proteins with more than one constituent peptides (supplemental Fig. S12). Quantitative precision is lower in AA labeling for fast-turnover peptides, but this difference is attenuated at the protein level.

## Discussion

There is a need for rigorous assessment of the strategies used to measure protein turnover rates in intact animals. Irrespective of the labeling precursor, there is potential for a delay in the equilibration of the precursor pool. Although this can be ameliorated by the use of HW as a precursor label, knowing the precursor enrichment kinetic rate constant (*k*_p_) in animal studies is recognized as a challenge. The primary focus of our study is the comparison of two labeling methods (HW *versus* AA) with different precursor equilibration rates across high- and low-turnover tissues. We also explore different approaches to derive the precursor RIA kinetics parameters in AA labeling, but none of the three approaches taken is entirely satisfactory for all tissues. If we assume the HW-fitted data have higher reliability because of the minor impact of complications from precursor delay, then tissue-specific values of the precursor enrichment rate constants allow the AA data to align closely with the HW data. These values were ∼0.28 to 0.38 d^–1^ for the adult mouse heart and muscle and ∼0.43 to 0.60 d^–1^ for the liver and kidney, but it was not entirely clear how they might be reliably derived without the external reference of HW data.

Surprisingly, direct LC–MS measurement of intracellular lysine pools gave *k*_p_ values that were considerably lower than required to be compatible with label enrichment of peptides. There may be preferential label reutilization of AAs released by degradation for synthesis *de novo*, such that the measured free lysine is decoupled from the true precursor of protein synthesis; the aminoacyl t-RNA pool. Other complications may also cause the precursor enrichment rate to deviate from exponential rise kinetics, for instance, unlabeled lysine is dietary and delayed by digestion prior to transport, whereas labeled lysine is a free AA that is available to membrane transporters immediately. Analytical approaches might require complex measurements of precursor RIA in multiple “compartments”: plasma, interstitial fluid, intracellular fluid, as well as in the aminoacyl tRNA pool.

We therefore explored two alternatives to estimate *k*_p_, but neither approach was entirely satisfactory. We previously advocated an approach based on mass isotopomer analysis of peptides containing two instances of the AA (5). This approach has the potential to reveal the true precursor isotopic enrichment based on the immediate protein precursor pool (*e.g.*, labeled aminoacyl t-RNA), but there is a technical challenge associated with determination of precursor pool behavior. Because all subsequent protein turnover measurements use these kinetics that define precursor behavior, it is important to analyze doubly labeled peptides carefully, either by manually extracting clean and readily isolatable extracted ion MS1 chromatograms, or by automating analysis of all peptides and modeling the best estimates for each time point. Unexpectedly, there is a wide distribution of calculated RIA values from the heavy–heavy (*i.e.*, m12) peaks that have identified two heavy lysines and the heavy–light (*i.e.*, m6) peaks that have incorporated one lysine. A best-fit exponential model of RIA over time has large residuals but yields values of *k*_p_ consistent with expectation. The RIA fitting also yields different best-fit RIA_p_ plateau values in each tissue, which is counterintuitive given that one might presume all tissues would equilibrate with the same dietary RIA. Over the 30 d labeling window used here, the precursor pool would be diluted by unlabeled lysine derived from the pre-existing protein pool (the subjects are adult, nongrowing). Furthermore, this approach will suffer from coupling to the rate of turnover of the protein when *k*_p_ is not constant or the protein has not completely turned over. A very high turnover protein (one that might not be measurable with any accuracy, as tissues cannot be sampled rapidly enough) would be replaced quickly, and thus become a high-frequency sensor of the precursor pool. By contrast, a protein with a lower rate of replacement would retain a significant proportion of the protein pool that was synthesized in the early stages of precursor pool equilibration, thus giving a dampened measure of the rate of precursor rise to plateau, although the plateau value for RIA should be the same in either instance. However, empirically, the *k*_p_ values calculated from dilysine peptides are not correlated with the *k*_deg_ of the peptides.

In parallel, we examined a two-compartment nested optimization method to gain the best fit *k*_p_ that explains the labeling curves directly from the peptide data. This method may compensate for the time integral in the dilysine peptide analysis and indeed appears to perform well for slow-equilibration tissues such as the heart and the skeletal muscle. However, when *k*_deg_ << *k*_p_ for most peptides in a fast-equilibration tissue such as the liver, the two-compartment model never outperformed the one-compartment model in minimizing fitting errors, likely because the initial sigmoidal “bend” in the kinetic curve of the two-compartment model is not apparent, and thus, different combinatorial values of *k*_p_ and *k*_deg_ can explain the peptide RIA data equally well. Optimization of complete datasets to derive precursor behavior may also introduce uncertainty, and the error gradients are shallow for many combinations of *k*_p_ and *k*_deg_.

Taken together, these complications serve to highlight the difficulties of using labeled AAs in intact animal systems. All the solutions that have been explored here are complicated and require additional analyses or complex modeling. We therefore proceeded with a two-compartment model with *k*_p_ values from automated dilysine analysis and weighted least square optimization of all data points, as the optimizations led to *k*_deg_ that agreed well with HW data. Although we place greater reliability on HW-derived values, it should be acknowledged that there can be no “ground truth” for turnover measurements. Whilst premixed synthetic isotope analogs with known isotope ratios may be used as a standard for the accuracy of MS quantification of isotopomer intensities, they cannot serve as a calibration target of turnover rates *in vivo* or precursor enrichment. In the steady state in particular, the true rate of replacement of a protein cannot be known without bias, whether measured by tracer experiments or by fluorophore tagging, the perturbations introduced and the analytical uncertainty in the measurements both introduce the potential for deviation from the true degradation rate. However, we reason that the HW method gives the closest approximation. This is based on the relative immunity from precursor pool kinetics due to of the fast equilibration of water, and is further supported by the labeling trajectory of the MUPs.

A more complex three-exponent two-compartment model (11) that accounts for label dilution from global protein degradation further improved data fit and concordance with HW results incrementally. However, one is left with the same problem of having to learn the model parameters from the data without guarantee that such values can be found. Moreover, even if a complex model fits the data more closely, it cannot be guaranteed that the resulting optimized *k*_deg_ are in fact accurate values as they are inside the cell. The lack of an acceptable gold standard of turnover rates of proteins *in vivo* complicates model comparisons. Literature values of turnover rates *in vivo* remain few and vary greatly by species, tissues, age, and physiological states, making direct comparisons difficult. Prior studies that compared fitting models largely used only two criteria to compare different models—(i) measures of model fit such as residual sums of square, coefficients of determination, or information criteria; (ii) the ability of the model to avoid unreasonable kinetic rate constants in the fitted results, for example, negative values, or values that are out of the allowable numerical ranges given the sampling time points. Generally speaking, however, the introduction of additional parameters in a complex model will allow the kinetics function to move more freely to the data points, which can lead to trade-offs between variance and bias and the possibility of overfitting.

In the absence of *k*_deg_ standards, we propose that intraprotein variance should be examined as one of multiple criteria by which analytical methods to derive turnover rates are evaluated. This is based on the simple assumption that a protein is created and destroyed in its entirety (*i.e.*, a protein is never partially excised from one terminus then repaired with replacement AAs). Hence, different peptides from the same protein should share similar turnover rates, if the modeling results are reliable, and by extension, this gives an estimate of whether the model will return identical *k*_deg_ for two polypeptide chains with equal true turnover rates. We found that the two-compartment model generally increases intraprotein variance at equal *R*^2^ cutoffs compared with the simple exponential model. However, because of the clear inadequacy of this exponential model in accounting for precursor delay in AA labeling, we conclude that a two-compartment model should be preferred, but at the same time, stringent data filtering strategies are needed for model fitting in order to maximize the number of quantified peptides while minimizing intraprotein variance.

In summary, this study is a highly controlled comparison of HW and AA labeling strategies for measurement of peptide turnover rates in four tissues in intact animals. In this article, we have evaluated strategies for high-quality measurements of turnover rates—discussion of the biological significance of these results will be addressed in a separate publication. It is possible to bring the two datasets into close agreement when the AA precursor behavior is addressed to provide a suitable *k*_p_ correction. For robust measurement of turnover rates in intact animals, we would recommend: (a) labeling with [^2^H_2_]O, (b) determination of labeling profiles in a peptide-specific analytical workflow to compensate for the specificity of HW labeling, (c) aggregation of data from multiple peptides to increase confidence in the extraction of the MS1 isotopomer profiles, (d) stringent quality filters at the peptide level in the analysis methods to minimize intraprotein variance, and (e) distribution of replicates in the time domain, to define as broad a range of turnover rates as possible. In reporting turnover rates, care should be given to the limitations imposed by the sampling time points—these intervals set limits on the range of turnover rates that are accessible. This is eased by a temporally expanding sampling window.

A theoretical analysis of kinetic curves can help set boundary conditions, which will be similar to those illustrated in Figure 1. A more sophisticated estimate will require additional information and for us to define additional criteria, for example, minimum of x informative time points (let us say, between 0.05 and 0.9 incorporation), compared with the time point–data quality relationship (Fig. 7). This then defines expectations on turnover rate and thus sets the range. For example, sampling of 1, 2, 3, 4, 8, 10, 13, 16, 20, 32 d, and working on the assumption of a measurable abundance for labeled or unlabeled peptide of 5% of the total, over three data points, should yield measurable variation in the RIA for proteins with *k*_deg_ values between 0.002 d^−1^ and 1 d^−1^. Beyond these limits, it is only possible to say “less than 0.002 d^−1^” and “greater than 1 d^−1^.” Ideally, sampling intervals less than 1 day would allow access to higher turnover proteins, arguably only feasible with HW strategies. At the other extreme, very low turnover proteins could require labeling periods of many months, with less frequent sampling.

AA labeling is analytically simple but metabolically complex, with tissue-dependent variation in behavior. Given the challenge of determination of *k*_p_ values for a two-compartment model in AA labeling, dilysine peptides and iterative two-compartment model fitting performed better than direct measurement of free lysine in tissues. These approaches provided *k*_p_ values that are consistent with the observed peptide data. Improved methods to model the RIA values of double-labeled peptides, such as a time integral of protein pool replacement, could improve measurements using AA labeling approaches. By contrast, HW equilibrates quickly and nonenzymatically, and hence, *k*_p_ is systems wide across tissues and requires minimal correction in precursor pool equilibration. HW labeling is also cheaper and enables the quantification of a substantially greater number of peptides. On the other hand, isolation and analysis of the subtly shifting isotopomer profile in HW labeling is more challenging. Because turnover measurements track the tracer through the protein/peptide pool, it is usual to acquire these data at the MS1 level. In a complex proteome analysis, it is inevitable that the isotopomer peaks will contain noise and potentially, contributions from other peptides. Whilst for AA labeling, this is true of the unlabeled (m_0_) and the labeled (m_6_) monoisotopic peaks, with HW protocols, there are more opportunities for interference in the intensity of the six isotopomer peaks (m_0_, m_1_, m_2_, m_3_, m_4_, and m_5_). For peptide ions that are high mass, have many potential label centers, or have high enrichment because of fast turnover, the m0 peak will be a minor ion and could further increase error through division of the small numerator by the sum of m_i_. On the other hand, shifts in isotopomer profiles may be subtle if precursor RIA is low or for select peptides with fewer labelable protons. HW labeling therefore places higher demand for accurate relative isotopomer abundance quantitation. To improve the isolation of the true peptides, high-resolution MS1 spectra are preferred, to allow the extraction of the area under the chromatogram for that ion.

Future studies of whole animal protein turnover will require even greater attention to a range of prerequisites, including choice of label—at present, the strongest case can be made for the use of HW. Furthermore, the duration of the labeling experiment (to cover the broadest range of turnover rates that are required) is critical, although it will always be challenging to recover accurate rate constants for particularly high and low turnover proteins. There is also an argument for an increased number of time points to scribe the labeling curve—we would argue that points distributed across the labeling curve are preferable to replicate samples with fewer time points. When only one parameter is to be optimized in a kinetic model (*e.g.*, in a monoexponential model or a two-compartment model after *k*_p_ and RIA_p_ are found), it would be perfectly possible to calculate an accurate turnover rate from a single time point, in an error-free world. However, as is evidenced from this study, there is considerable variance in such experiments, and multiple replicates are required. Furthermore, if all samples were taken at a single time point, then the range of measurable turnover rate constants is restricted, according to the argument in Figure 1. Distribution of sampling intervals across an extended period, with a sampling protocol that has some properties of a geometric expansion, allows multiple data points to be collected for either high turnover or low turnover proteins, and moreover allow one to fully define the shape of the curve of the two-compartment models to extract both rate constants. Given that these studies are conducted on animals, bringing the pressure of reduction of usage, we would argue that sampling should be distributed in time, rather than by taking multiple replicates at fewer time points, to obtain the broadest possible informative range of *k*_deg_ values.

Finally, there is a need for a well-conducted labeling study to be analyzed by many of the analytical packages for turnover determination—a detailed comparison of the resultant outputs would be especially informative. This would lead us to a more open democratization of the analytical workflow and a clearer understanding of where there are variances in the final outputs, and why. To this end, all raw data from this study are available (ProteomeXchange PXD029639). An animal that has been labeled in such studies is often the source of just one or a few tissues. The “3R” principles underpinning animal research (Replacement, Reduction, and Refinement), specifically, reduction, would be well served if, in future, labeling and turnover studies were enhanced by a willingness to share unused tissues, to allow others to replicate studies, improve data analysis, and extend understanding. Indeed, in a recent review, a future imperative was exactly this; “Data and tissue-sharing offer opportunities for more efficient use of information collected from animals and may avoid unnecessary repetition” (48).

## Data and Code Availability

Raw MS data have been deposited to ProteomeXchange (49) at accession PXD029639. The source code and instructions for Riana, v.0.6.4, can be accessed at http://github.com/ed-lau/riana.

## Supplemental data

This article contains supplemental data.

## Conflict of interest

The authors declare no competing interests.
