# Supplementary material for: Harmonizing Labeling and Analytical Strategies to Obtain Protein Turnover Rates in Intact Adult Animals
Source: Mol Cell Proteomics. 2022 May 28;21(7):100252. doi: 10.1016/j.mcpro.2022.100252 (PMC9249856; doi:10.1016/j.mcpro.2022.100252)

1433E − AAFDDAIAELDTLSEESYK_2

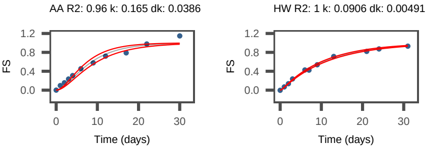

1433G − TAFDDAIAELDTLNEDSYK_2

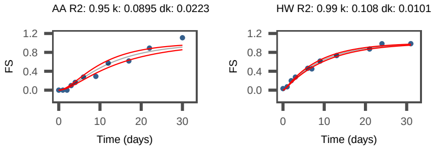

2AAA − DNTIEHLLPLFLAQLK_3

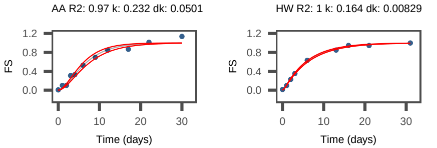

1433E − EAAENSLVAYK_2

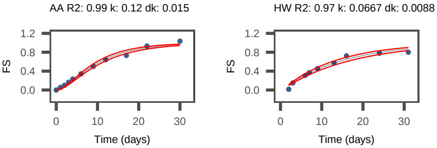

1433G − TAFDDAIAELDTLNEDSYK_3

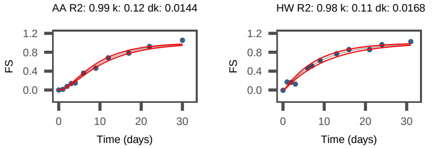

2AAA(Non−Unique) − QLSQSLLPAIVELAEDAK_3

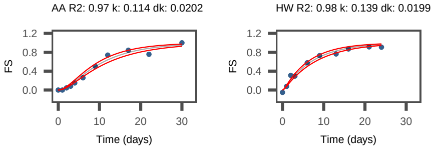

1433E − YDEMVESMK_2

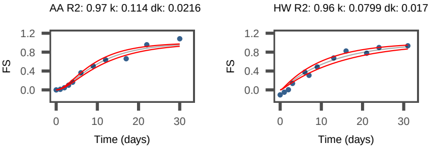

1433G(Non−Unique) − YDDMAAAMK_2

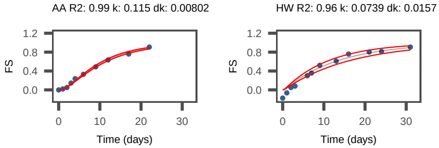

2AAA − SALASVIMGLSPILGK_2

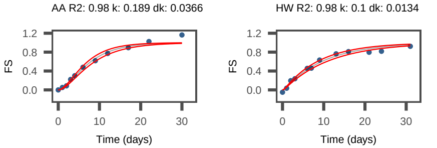

1433G − AYSEAHEISK_2

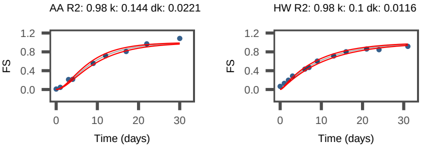

1433Z − DICNDVLSLLEK_2

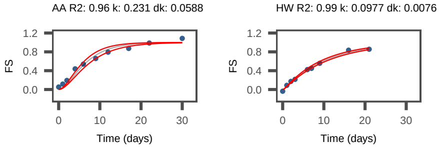

6PGL − IVAPISDSPKPPPQR_3

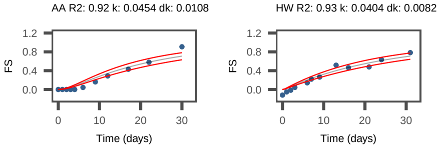

1433G − AYSEAHEISK_3

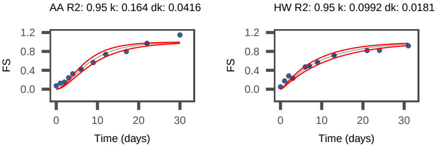

1433Z − TAFDEAIAELDTLSEESYK_3

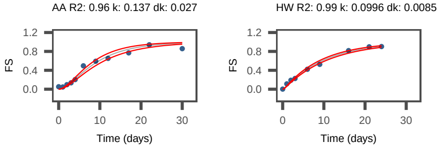

A1AT2 − ELDQDTVFALANYILFK_3

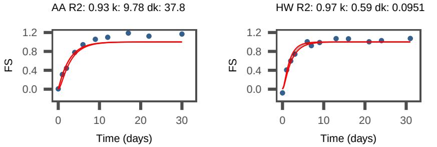

1433G − ELEAVCQDVLSLLDNYLIK_3

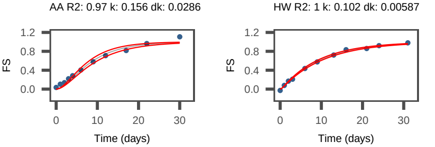

1433Z − YDDMAACMK_2

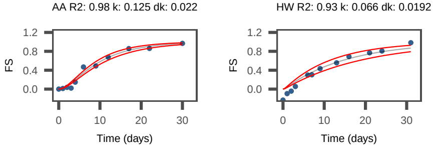

A1AT2(Non−Unique) − MQHLEQTLNK_2

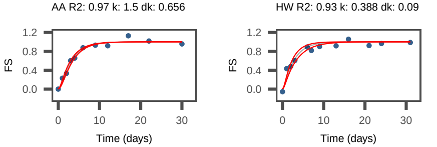

AATC – APPSVFAQVPQAPPVLVFK_2

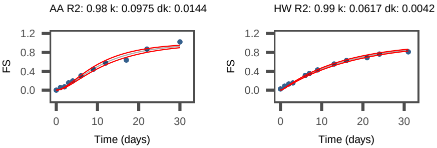

AATC – ITEQIGMFSFTGLNPK_2

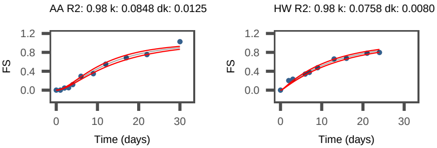

AATM – NLFAFFDMAYQGFASGDGDK_3

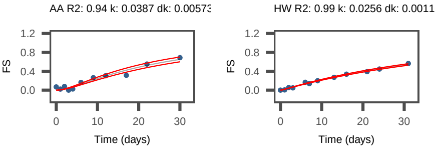

AATC – APPSVFAQVPQAPPVLVFK_3

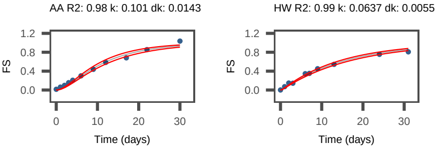

AATC – NLDYVATSIHEAVTK_2

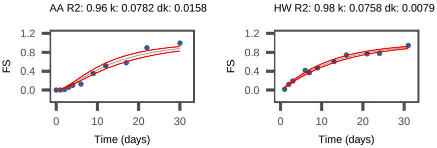

AATM – QWLQEVK_2

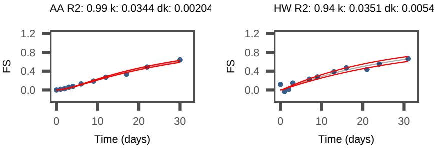

AATC – AQVPQAPPVLVFK_2

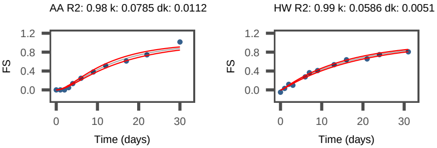

AATC – NLDYVATSIHEAVTK_3

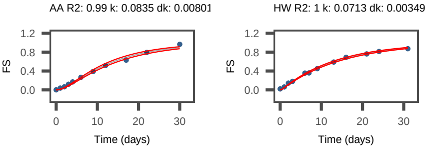

AATM – SSWWTHVEMGPPDPILGVTEAFK_2

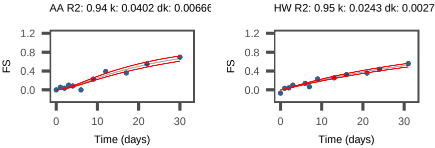

AATC – FLFPFFDSAYQGFASGDLEK_2

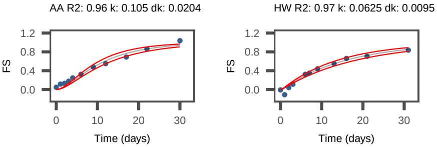

AATC – VGNLTVVGK_2

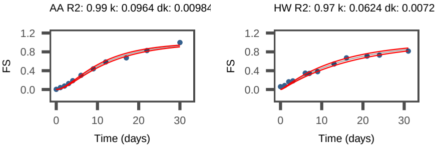

AATM – TCGFDFSGALEDISK_2

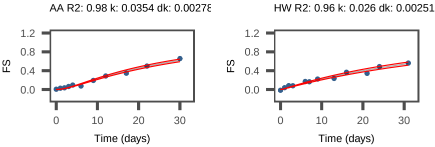

AATC – FLFPFFDSAYQGFASGDLEK_3

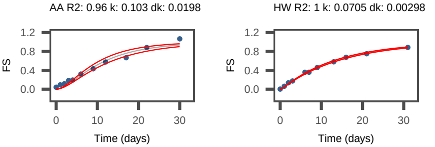

AATC – YFVSEGFELFCAQSFSK_2

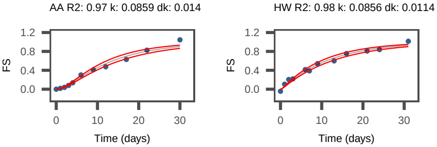

AATM – VGAFTVVCK_2

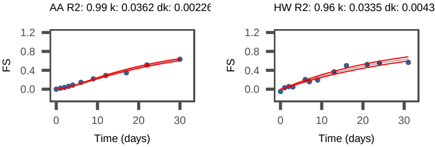

AATC – INMCGLTTK_2

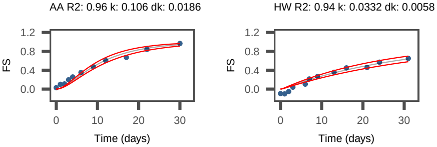

AATM – EIASVVK_2

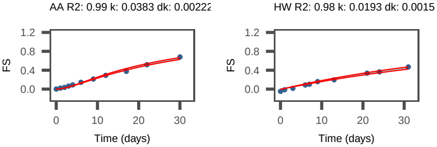

ABCB7 – QALIDMNTLFTLLK_2

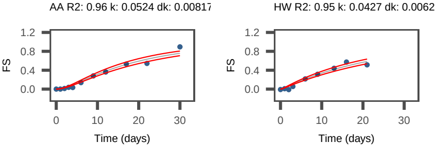

ABLM1 – CEACHQFITGK_3

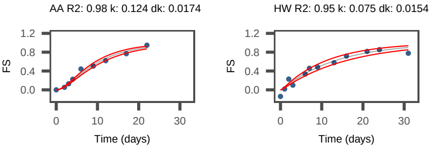

ACADM – CVTEPSAGSDVAAIK_2

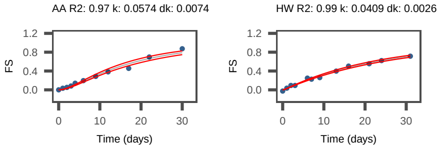

ACADV – ASNTSEVYFDGVK_2

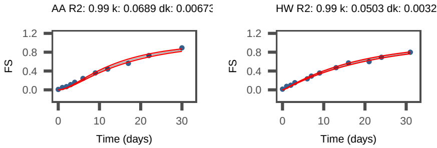

ACADL – FIPQMTAGK_2

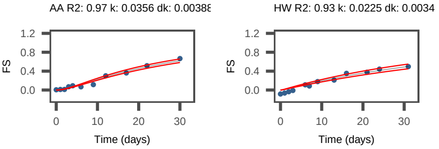

ACADM – ENVLIGEGAGFK_2

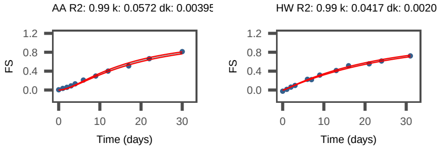

ACADV – EATQAVLDKPETLSSDASTR_3

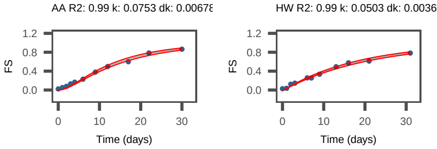

ACADL – RLDSGSASMAK_2

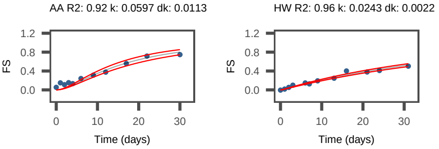

ACADS – EEGDSWVLNGTK_2

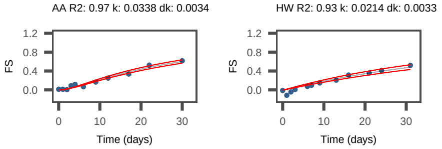

ACADV – FFEEVNDPAK_2

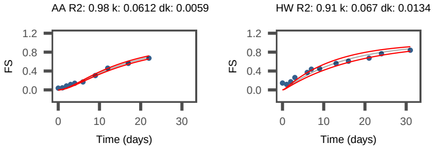

ACADL – TVAHIQTVQHK_2

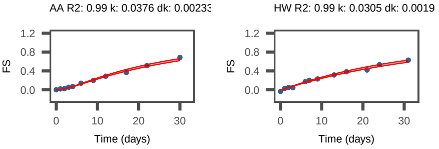

ACADS – EHLFPTAQVK_3

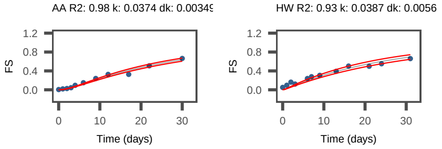

ACADV – GQLTIDQVFPYPSVLSEEQAQFLK_2

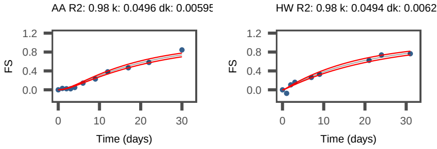

ACADL – TVAHIQTVQHK_3

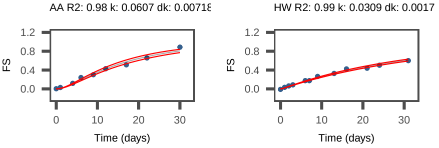

ACADS – ENLLGEPGMGFK_2

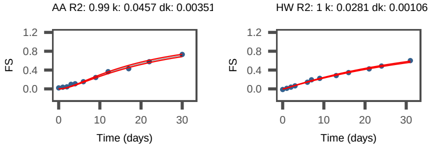

ACADV – GQLTIDQVFPYPSVLSEEQAQFLK_3

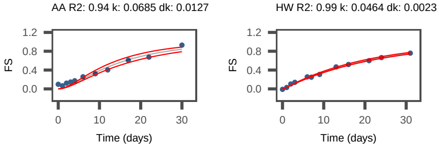

ACADM – AFTGFIVEADTPGIHIGK_3

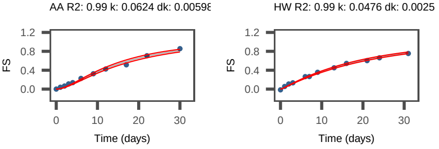

ACADS – NAFGAPLTK_2

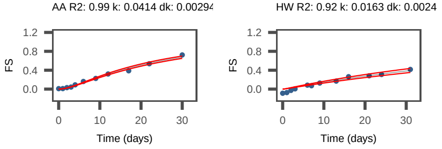

ACADV – IFCSEAAWK_2

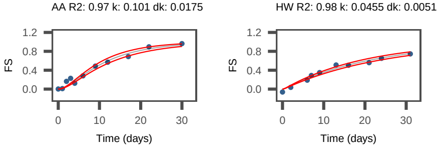

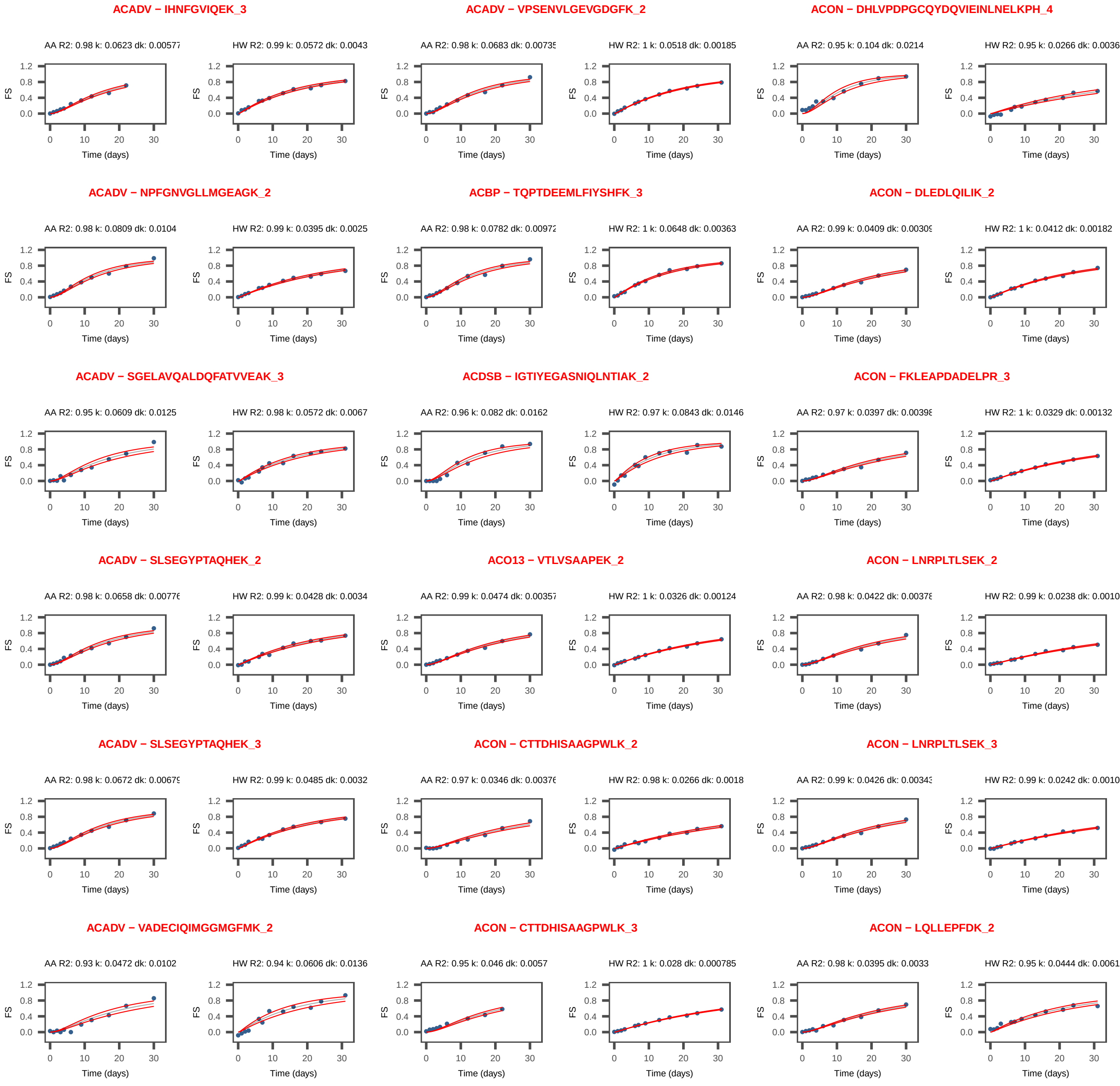
ACADV − IHNFGVIQEK_3
AA R2: 0.98 k: 0.0623 dk: 0.00577
HW R2: 0.99 k: 0.0572 dk: 0.0043
ACADV − VPSENVLGEVGDGFK_2
AA R2: 0.98 k: 0.0683 dk: 0.00735
HW R2: 1 k: 0.0518 dk: 0.00185
ACON − DHLVPDPGCQYDQVIEINLNELKPH_4
AA R2: 0.95 k: 0.104 dk: 0.0214
HW R2: 0.95 k: 0.0266 dk: 0.0036
ACADV − NPFGNVGLLMGEAGK_2
AA R2: 0.98 k: 0.0809 dk: 0.0104
HW R2: 0.99 k: 0.0395 dk: 0.0025
ACBP − TQPTDEEMLFIYSHFK_3
AA R2: 0.98 k: 0.0782 dk: 0.00972
HW R2: 1 k: 0.0648 dk: 0.00363
ACON − DLEDLQILIK_2
AA R2: 0.99 k: 0.0409 dk: 0.00309
HW R2: 1 k: 0.0412 dk: 0.00182
ACADV − SGELAVQALDQFATVVEAK_3
AA R2: 0.95 k: 0.0609 dk: 0.0125
HW R2: 0.98 k: 0.0572 dk: 0.0067
ACDSB − IGTIYEGASNIQLNTIAK_2
AA R2: 0.96 k: 0.082 dk: 0.0162
HW R2: 0.97 k: 0.0843 dk: 0.0146
ACON − FKLEAPDADELPR_3
AA R2: 0.97 k: 0.0397 dk: 0.00398
HW R2: 1 k: 0.0329 dk: 0.00132
ACADV − SLSEGYPTAQHEK_2
AA R2: 0.98 k: 0.0658 dk: 0.00776
HW R2: 0.99 k: 0.0428 dk: 0.0034
ACO13 − VTLVSAAPEK_2
AA R2: 0.99 k: 0.0474 dk: 0.00357
HW R2: 1 k: 0.0326 dk: 0.00124
ACON − LNRPLTLSEK_2
AA R2: 0.98 k: 0.0422 dk: 0.00378
HW R2: 0.99 k: 0.0238 dk: 0.0010
ACADV − SLSEGYPTAQHEK_3
AA R2: 0.98 k: 0.0672 dk: 0.00679
HW R2: 0.99 k: 0.0485 dk: 0.0032
ACON − CTTDHISAAGPWLK_2
AA R2: 0.97 k: 0.0346 dk: 0.00376
HW R2: 0.98 k: 0.0266 dk: 0.0018
ACON − LNRPLTLSEK_3
AA R2: 0.99 k: 0.0426 dk: 0.00343
HW R2: 0.99 k: 0.0242 dk: 0.0010
ACADV − VADECIQIMGGMGFMK_2
AA R2: 0.93 k: 0.0472 dk: 0.0102
HW R2: 0.94 k: 0.0606 dk: 0.0136
ACON − CTTDHISAAGPWLK_3
AA R2: 0.95 k: 0.046 dk: 0.0057
HW R2: 1 k: 0.028 dk: 0.000785
ACON − LQLLEPFDK_2
AA R2: 0.98 k: 0.0395 dk: 0.0033
HW R2: 0.95 k: 0.0444 dk: 0.0061
FS
Time (days)

ACON − LTGSLSGWTSPK_2

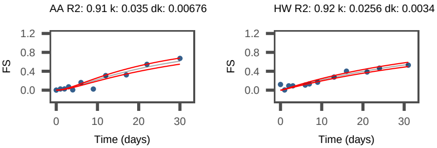

ACON − VAVPSTIHCDHLIEAQVGGEK_4

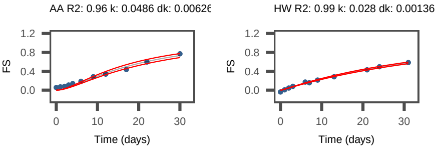

ACPM − SDAPPLTLDGIKDR_3

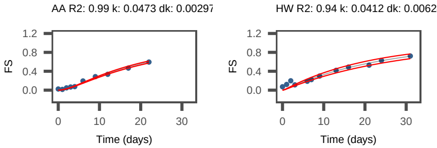

ACON − QALAHGLK_2

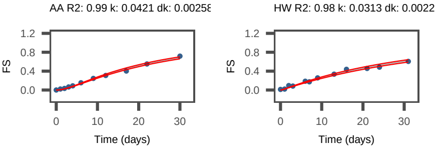

ACOT1(Non−Unique) − GPGIGLLGISK_2

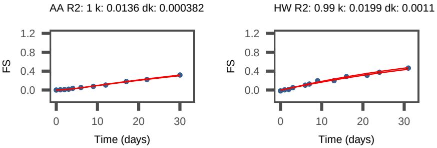

ACS2L − GEAAFAFIVLK_2

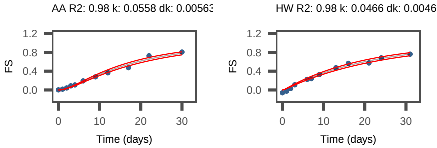

ACON − SDFDPGQDTYQHPPK_2

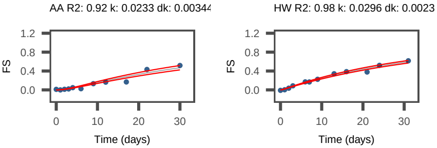

ACOT1(Non−Unique) − SDTTFLFLVGQDDHNWK_3

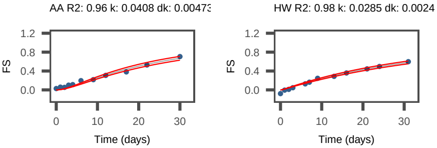

ACS2L − PFFGIVPVLMDEK_2

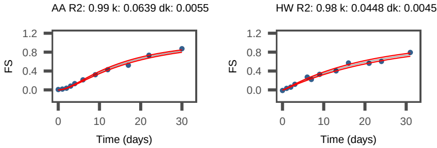

ACON − SDFDPGQDTYQHPPK_3

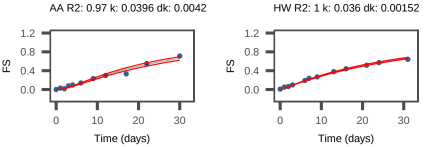

ACOT2 − DGLLDVVEALQSPLVDK_2

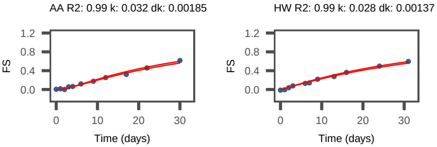

ACS2L − TLGSVGEPINHEAWEWLHK_4

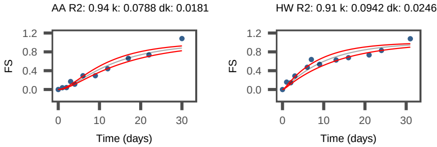

ACON − VAGILTVK_2

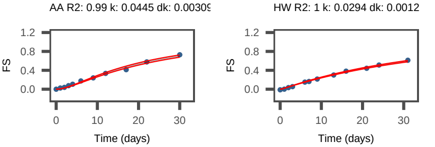

ACOX1 − YDGNVYENLFEWAK_2

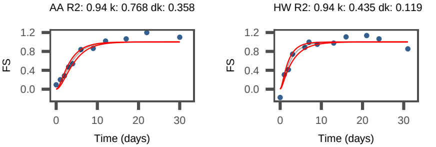

ACSF2 − YIVFVEGYPLTISGK_2

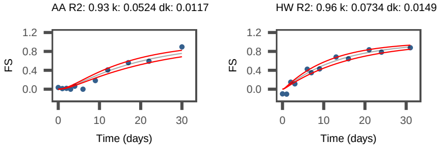

ACON − VAVPSTIHCDHLIEAQVGGEK_3

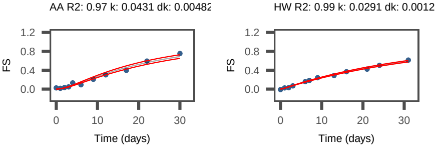

ACPM − SDAPPLTLDGIK_2

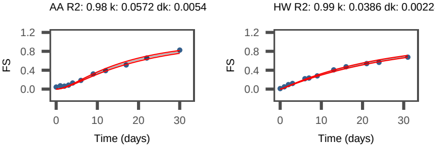

ACSL1 − EVAELAECIGSGLIQK_2

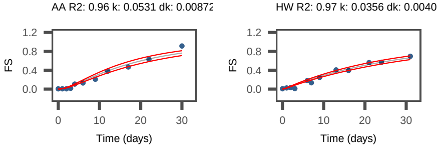

ACSL1 − EVAELAECIGSGLIQK_3

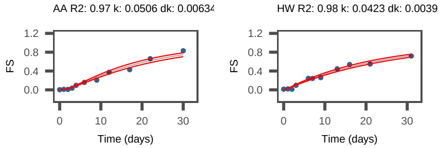

ACSL1 − SAVLEDDKLLVYYYDDVR_3

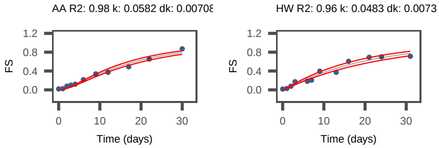

ACTN2 − ASTHETWAYGK_3

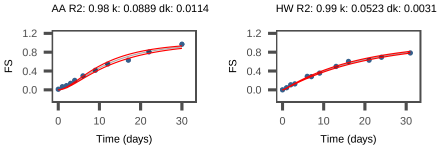

ACSL1 − GAMITHQNIINDCSGFIK_3

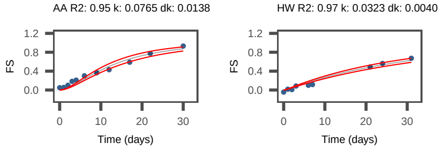

ACTA(Non−Unique) − ESAGIHETTYNSIMK_3

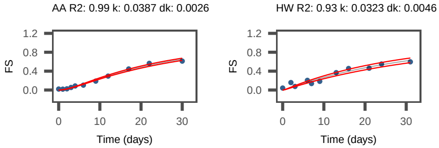

ACTN2 − HRPDLIDYSK_2

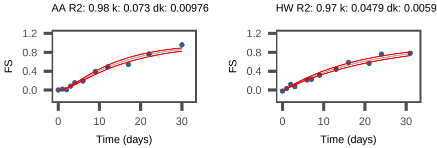

ACSL1 − LIAVVVPDVESLPSWAQK_2

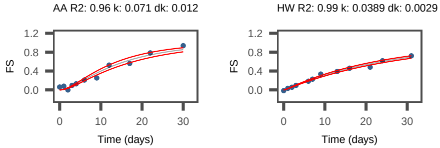

ACTA(Non−Unique) − VAPEEHPTLLTEAPLNPK_2

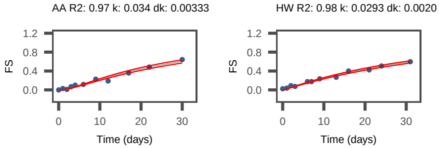

ACTN2 − HRPDLIDYSK_3

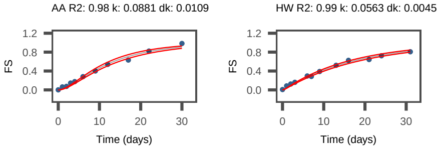

ACSL1 − LIAVVVPDVESLPSWAQK_3

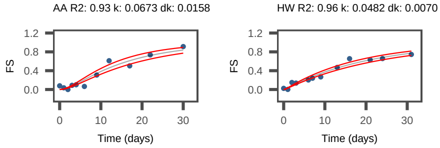

ACTA(Non−Unique) − YPIEHGIITNWDDMEK_2

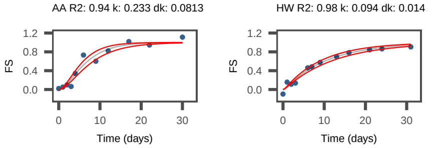

ACTN2 − ILASDKPYILAEELR_2

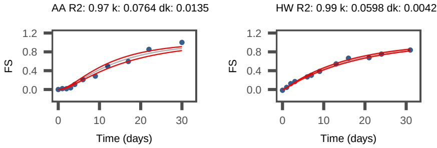

ACSL1 − LLLEGVENK_2

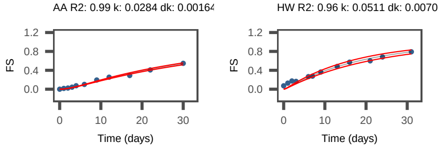

ACTN2 − AIGPWIQNK_2

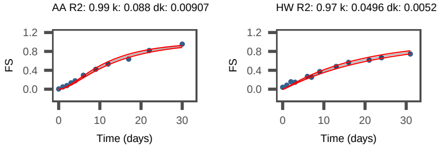

ACTN2 − ILASDKPYILAEELR_3

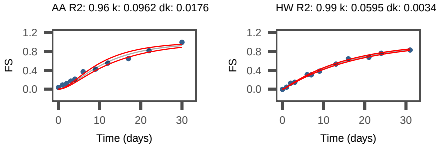

ACSL1 − LVDVEEMNYLASK_2

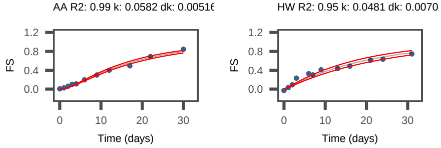

ACTN2 − ALDYIASK_2

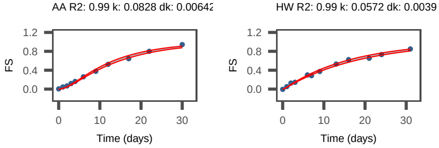

ACTN2 − ISNRPAFMPSEGK_3

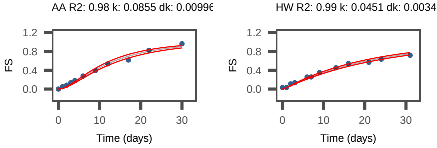

ACTN2 − LLETIDQLHLEFAK_2

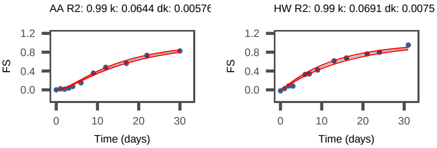

ACTN2 − LLETIDQLHLEFAK_3

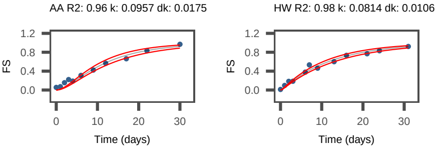

ACTN2 − MLDAEDIVNTPKPDER_3

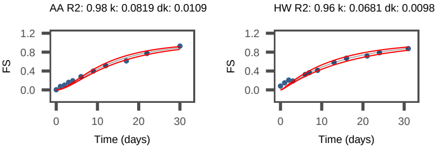

ACTN2 − QSILAIQNEVEK_2

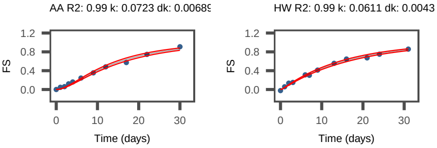

ACTN2 − QYEHNIINYK_2

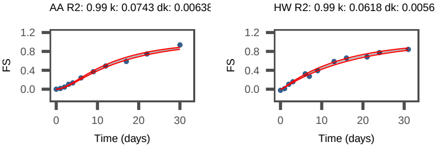

ACTN2 − SSIQITGALEDQMNQLK_3

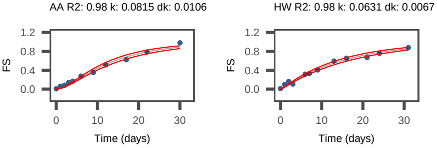

ACTN3(Non−Unique) − DLLLDPAWEK_2

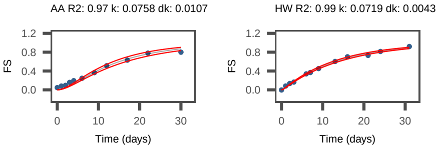

ACTN4(Non−Unique) − KHEAFESDLAAHQDR_2

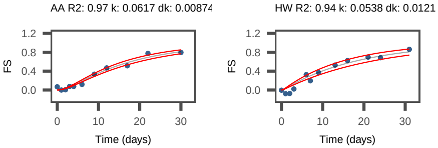

ACTN4(Non−Unique) − KHEAFESDLAAHQDR_4

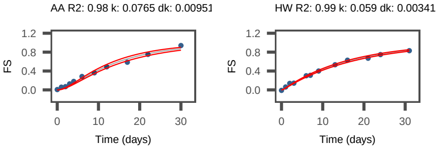

ACTZ(Non−Unique) − TLFSNIVLSGGSTLFK_3

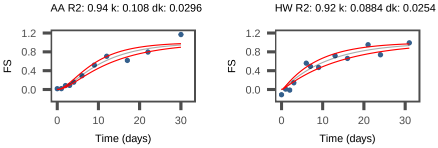

ACYP1 − GTVQGQLQGPVSK_2

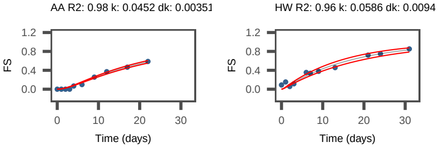

ADK − VEYHAGGSTQNSMK_3

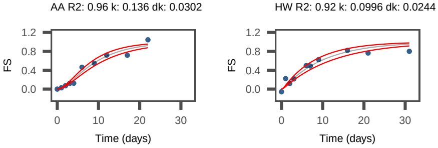

ADT1 − DEGANAFFK_2

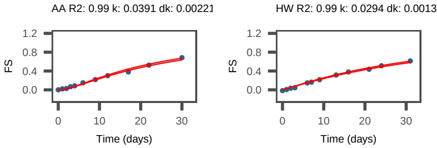

ADT1 − DFLAGGIAAAVSK_3

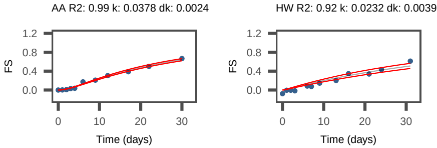

ADT1(Non−Unique) − LLLQVQHASK_3

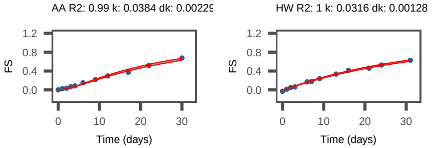

ADT1(Non−Unique) − VLVLYDEIK_2

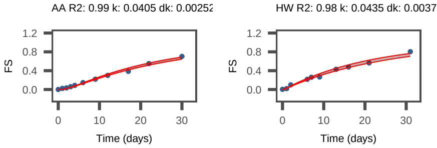

ADT2 − DFLAGGVAAAISK_2

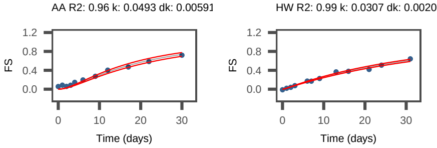

AIFM1 − ELWFSDDPNVTK_2

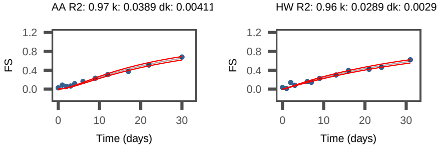

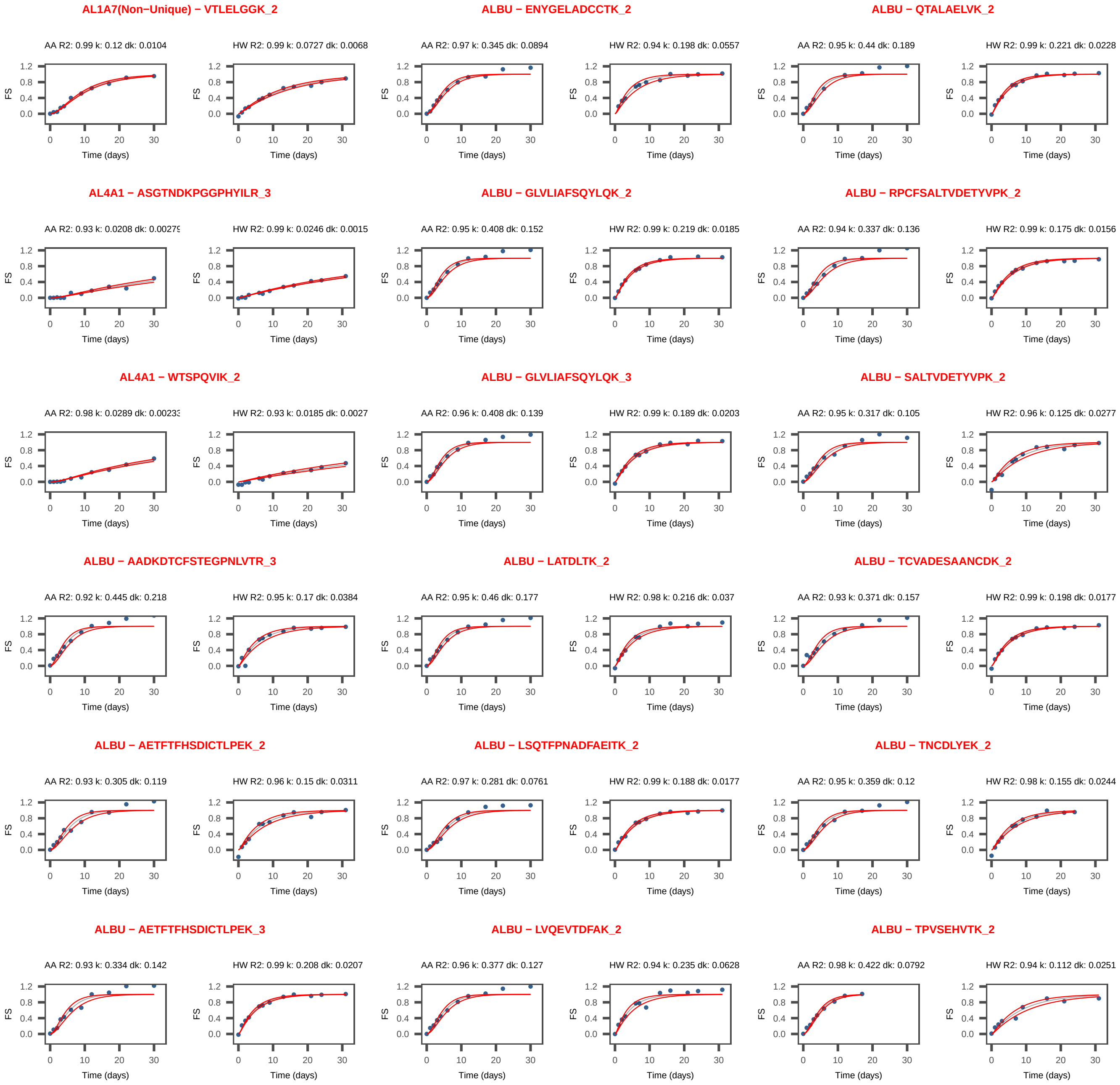
AL1A7(Non−Unique) − VTLELGGK_2
AA R2: 0.99 k: 0.12 dk: 0.0104
HW R2: 0.99 k: 0.0727 dk: 0.0068
ALBU − ENYGELADCCTK_2
AA R2: 0.97 k: 0.345 dk: 0.0894
HW R2: 0.94 k: 0.198 dk: 0.0557
ALBU − QTALAELVK_2
AA R2: 0.95 k: 0.44 dk: 0.189
HW R2: 0.99 k: 0.221 dk: 0.0228
AL4A1 − ASGTNDKPGGPHYILR_3
AA R2: 0.93 k: 0.0208 dk: 0.0027
HW R2: 0.99 k: 0.0246 dk: 0.0015
ALBU − GLVLIAFSQYLQK_2
AA R2: 0.95 k: 0.408 dk: 0.152
HW R2: 0.99 k: 0.219 dk: 0.0185
ALBU − RPCFSALTVDETYVPK_2
AA R2: 0.94 k: 0.337 dk: 0.136
HW R2: 0.99 k: 0.175 dk: 0.0156
AL4A1 − WTSPQVIK_2
AA R2: 0.98 k: 0.0289 dk: 0.0023
HW R2: 0.93 k: 0.0185 dk: 0.0027
ALBU − GLVLIAFSQYLQK_3
AA R2: 0.96 k: 0.408 dk: 0.139
HW R2: 0.99 k: 0.189 dk: 0.0203
ALBU − SALTVDETYVPK_2
AA R2: 0.95 k: 0.317 dk: 0.105
HW R2: 0.96 k: 0.125 dk: 0.0277
ALBU − AADKDTCFSTEGPNLVTR_3
AA R2: 0.92 k: 0.445 dk: 0.218
HW R2: 0.95 k: 0.17 dk: 0.0384
ALBU − LATDLTK_2
AA R2: 0.95 k: 0.46 dk: 0.177
HW R2: 0.98 k: 0.216 dk: 0.037
ALBU − TCVADESAANCDK_2
AA R2: 0.93 k: 0.371 dk: 0.157
HW R2: 0.99 k: 0.198 dk: 0.0177
ALBU − AETFTFHSDICTLPEK_2
AA R2: 0.93 k: 0.305 dk: 0.119
HW R2: 0.96 k: 0.15 dk: 0.0311
ALBU − LSQTFPNADFAEITK_2
AA R2: 0.97 k: 0.281 dk: 0.0761
HW R2: 0.99 k: 0.188 dk: 0.0177
ALBU − TNCDLYEK_2
AA R2: 0.95 k: 0.359 dk: 0.12
HW R2: 0.98 k: 0.155 dk: 0.0244
ALBU − AETFTFHSDICTLPEK_3
AA R2: 0.93 k: 0.334 dk: 0.142
HW R2: 0.99 k: 0.208 dk: 0.0207
ALBU − LVQEVTDFAK_2
AA R2: 0.96 k: 0.377 dk: 0.127
HW R2: 0.94 k: 0.235 dk: 0.0628
ALBU − TPVSEHVTK_2
AA R2: 0.98 k: 0.422 dk: 0.0792
HW R2: 0.94 k: 0.112 dk: 0.0251
FS
Time (days)

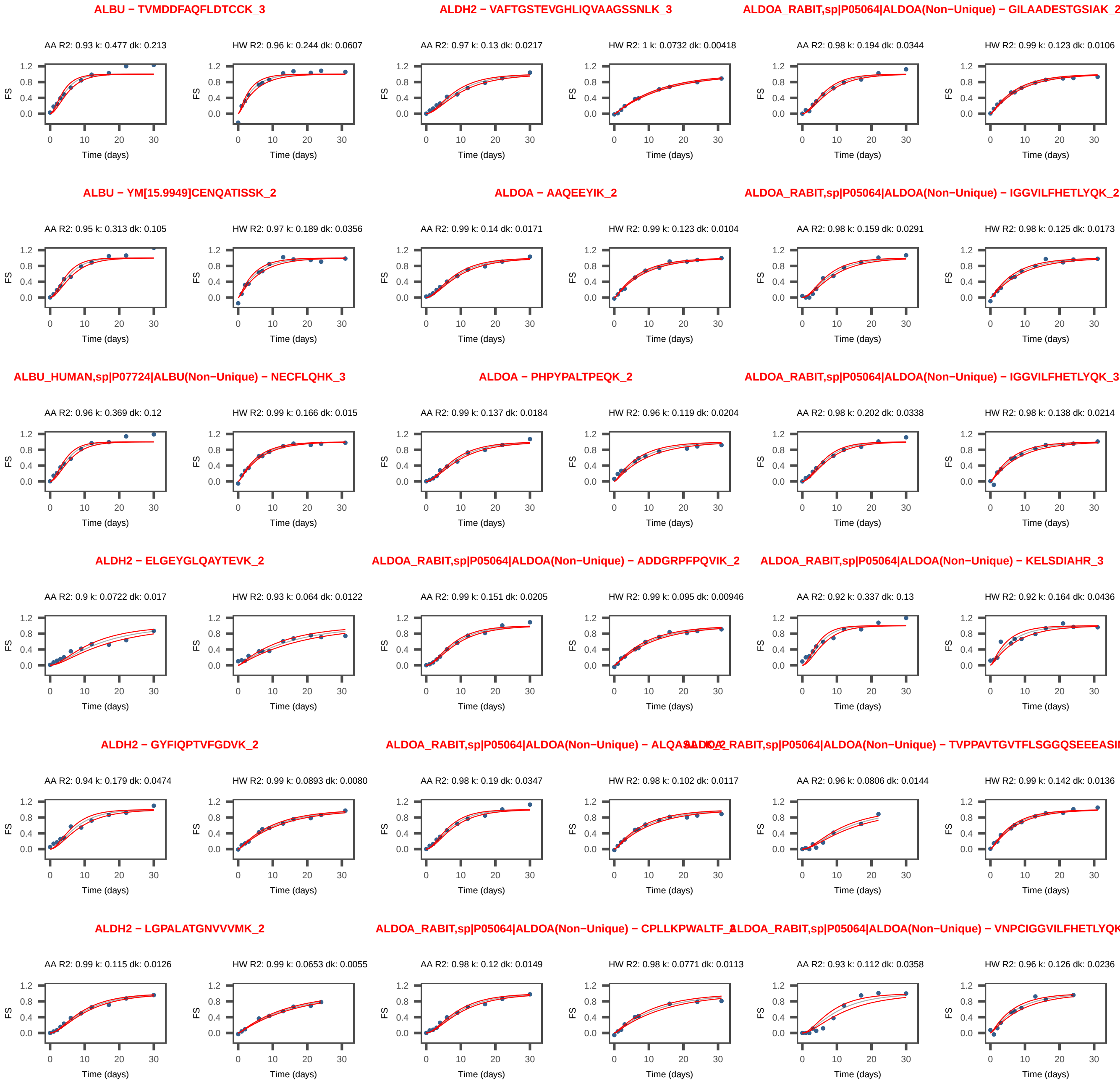
ALBU − TVMDDFAQFLDTCCK_3
AA R2: 0.93 k: 0.477 dk: 0.213
HW R2: 0.96 k: 0.244 dk: 0.0607
ALDH2 − VAFTGSTEVGHLIQVAAGSSNLK_3
AA R2: 0.97 k: 0.13 dk: 0.0217
HW R2: 1 k: 0.0732 dk: 0.00418
ALDOA_RABIT,sp|P05064|ALDOA(Non−Unique) − GILAADESTGSIAK_2
AA R2: 0.98 k: 0.194 dk: 0.0344
HW R2: 0.99 k: 0.123 dk: 0.0106
ALBU − YM[15.9949]CENQATISSK_2
AA R2: 0.95 k: 0.313 dk: 0.105
HW R2: 0.97 k: 0.189 dk: 0.0356
ALDOA − AAQEEYIK_2
AA R2: 0.99 k: 0.14 dk: 0.0171
HW R2: 0.99 k: 0.123 dk: 0.0104
ALDOA_RABIT,sp|P05064|ALDOA(Non−Unique) − IGGVILFHETLYQK_2
AA R2: 0.98 k: 0.159 dk: 0.0291
HW R2: 0.98 k: 0.125 dk: 0.0173
ALBU_HUMAN,sp|P07724|ALBU(Non−Unique) − NECFLQHK_3
AA R2: 0.96 k: 0.369 dk: 0.12
HW R2: 0.99 k: 0.166 dk: 0.015
ALDOA − PHPYPALTPEQK_2
AA R2: 0.99 k: 0.137 dk: 0.0184
HW R2: 0.96 k: 0.119 dk: 0.0204
ALDOA_RABIT,sp|P05064|ALDOA(Non−Unique) − IGGVILFHETLYQK_3
AA R2: 0.98 k: 0.202 dk: 0.0338
HW R2: 0.98 k: 0.138 dk: 0.0214
ALDH2 − ELGEYGLQAYTEVK_2
AA R2: 0.9 k: 0.0722 dk: 0.017
HW R2: 0.93 k: 0.064 dk: 0.0122
ALDOA_RABIT,sp|P05064|ALDOA(Non−Unique) − ADDGRPFPQVIK_2
AA R2: 0.99 k: 0.151 dk: 0.0205
HW R2: 0.99 k: 0.095 dk: 0.00946
ALDOA_RABIT,sp|P05064|ALDOA(Non−Unique) − KELSDIAHR_3
AA R2: 0.92 k: 0.337 dk: 0.13
HW R2: 0.92 k: 0.164 dk: 0.0436
ALDH2 − GYFIQPTVFGDVK_2
AA R2: 0.94 k: 0.179 dk: 0.0474
HW R2: 0.99 k: 0.0893 dk: 0.0080
ALDOA_RABIT,sp|P05064|ALDOA(Non−Unique) − ALQA
RABIT,sp|P05064|ALDOA(Non−Unique) − TVPPAVTGVTFLSGGQSEEEASI
AA R2: 0.98 k: 0.19 dk: 0.0347
HW R2: 0.98 k: 0.102 dk: 0.0117
AA R2: 0.96 k: 0.0806 dk: 0.0144
HW R2: 0.99 k: 0.142 dk: 0.0136
ALDH2 − LGPALATGNVVVMK_2
AA R2: 0.99 k: 0.115 dk: 0.0126
HW R2: 0.99 k: 0.0653 dk: 0.0055
ALDOA_RABIT,sp|P05064|ALDOA(Non−Unique) − CPLLKPWALTF_
ALDOA_RABIT,sp|P05064|ALDOA(Non−Unique) − VNPCIGGVILFHETLYQK
AA R2: 0.98 k: 0.12 dk: 0.0149
HW R2: 0.98 k: 0.0771 dk: 0.0113
AA R2: 0.93 k: 0.112 dk: 0.0358
HW R2: 0.96 k: 0.126 dk: 0.0236
FS
Time (days)

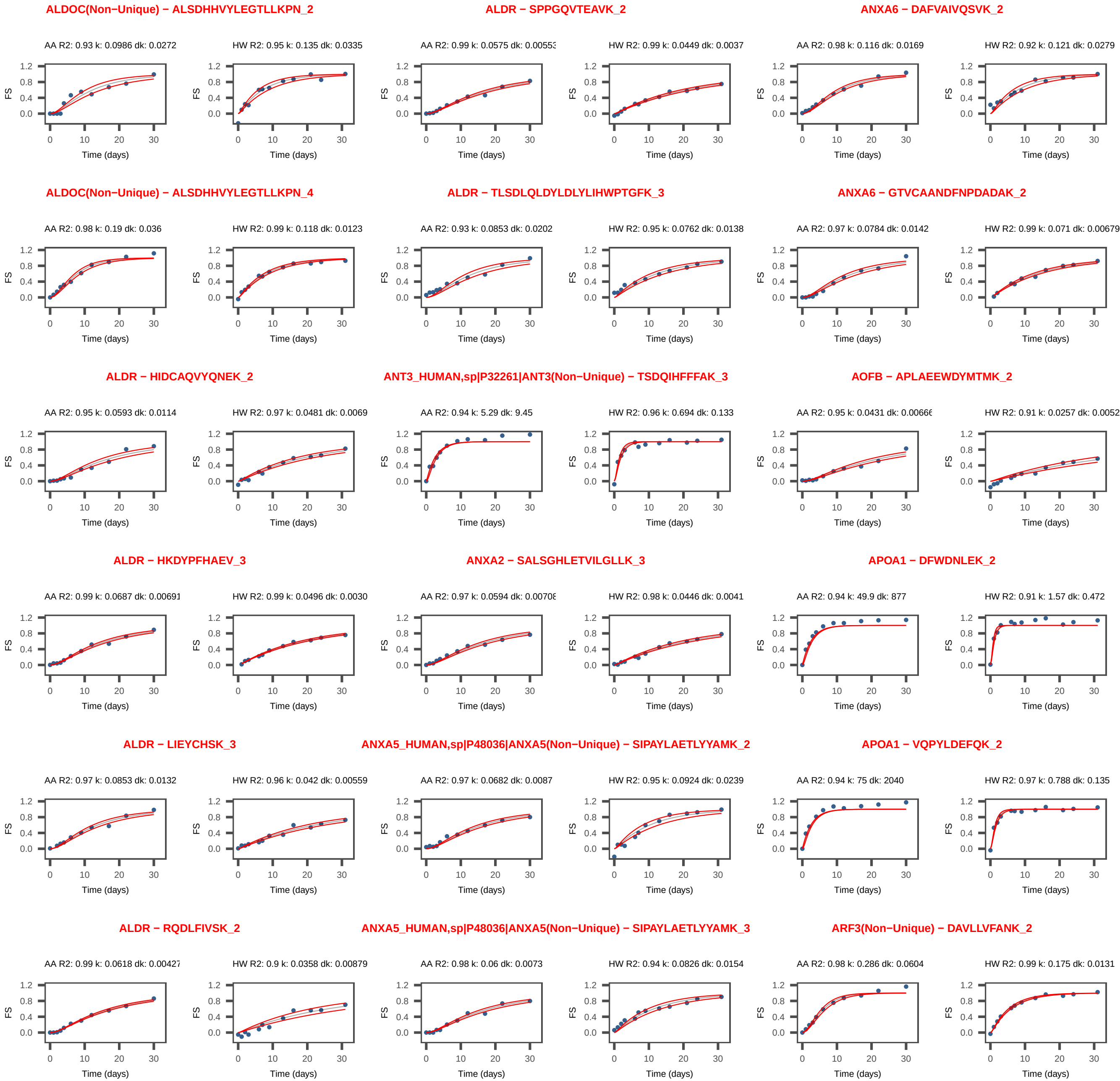
ALDOC(Non−Unique) − ALSDHHVYLEGTLLKPN_2
AA R2: 0.93 k: 0.0986 dk: 0.0272
HW R2: 0.95 k: 0.135 dk: 0.0335
ALDR − SPPGQVTEAVK_2
AA R2: 0.99 k: 0.0575 dk: 0.00553
HW R2: 0.99 k: 0.0449 dk: 0.0037
ANXA6 − DAFVAIVQSVK_2
AA R2: 0.98 k: 0.116 dk: 0.0169
HW R2: 0.92 k: 0.121 dk: 0.0279
ALDOC(Non−Unique) − ALSDHHVYLEGTLLKPN_4
AA R2: 0.98 k: 0.19 dk: 0.036
HW R2: 0.99 k: 0.118 dk: 0.0123
ALDR − TLSDLQLDYLDLYLIHWPTGFK_3
AA R2: 0.93 k: 0.0853 dk: 0.0202
HW R2: 0.95 k: 0.0762 dk: 0.0138
ANXA6 − GTVCAANDFNPDADAK_2
AA R2: 0.97 k: 0.0784 dk: 0.0142
HW R2: 0.99 k: 0.071 dk: 0.00679
ALDR − HIDCAQVYQNEK_2
AA R2: 0.95 k: 0.0593 dk: 0.0114
HW R2: 0.97 k: 0.0481 dk: 0.0069
ANT3_HUMAN,sp|P32261|ANT3(Non−Unique) − TSDQIHFFFAK_3
AA R2: 0.94 k: 5.29 dk: 9.45
HW R2: 0.96 k: 0.694 dk: 0.133
AOFB − APLAEEWDYMTMK_2
AA R2: 0.95 k: 0.0431 dk: 0.0066
HW R2: 0.91 k: 0.0257 dk: 0.0052
ALDR − HKDYPFHAEV_3
AA R2: 0.99 k: 0.0687 dk: 0.00691
HW R2: 0.99 k: 0.0496 dk: 0.0030
ANXA2 − SALSGHLETVILGLLK_3
AA R2: 0.97 k: 0.0594 dk: 0.00708
HW R2: 0.98 k: 0.0446 dk: 0.0041
APOA1 − DFWDNLEK_2
AA R2: 0.94 k: 49.9 dk: 877
HW R2: 0.91 k: 1.57 dk: 0.472
ALDR − LIEYCHSK_3
AA R2: 0.97 k: 0.0853 dk: 0.0132
HW R2: 0.96 k: 0.042 dk: 0.00559
ANXA5_HUMAN,sp|P48036|ANXA5(Non−Unique) − SIPAYLAETLYYAMK_2
AA R2: 0.97 k: 0.0682 dk: 0.0087
HW R2: 0.95 k: 0.0924 dk: 0.0239
APOA1 − VQPYLDEFQK_2
AA R2: 0.94 k: 75 dk: 2040
HW R2: 0.97 k: 0.788 dk: 0.135
ALDR − RQDLFIVSK_2
AA R2: 0.99 k: 0.0618 dk: 0.00427
HW R2: 0.9 k: 0.0358 dk: 0.00879
ANXA5_HUMAN,sp|P48036|ANXA5(Non−Unique) − SIPAYLAETLYYAMK_3
AA R2: 0.98 k: 0.06 dk: 0.0073
HW R2: 0.94 k: 0.0826 dk: 0.0154
ARF3(Non−Unique) − DAVLLVFANK_2
AA R2: 0.98 k: 0.286 dk: 0.0604
HW R2: 0.99 k: 0.175 dk: 0.0131
FS
Time (days)

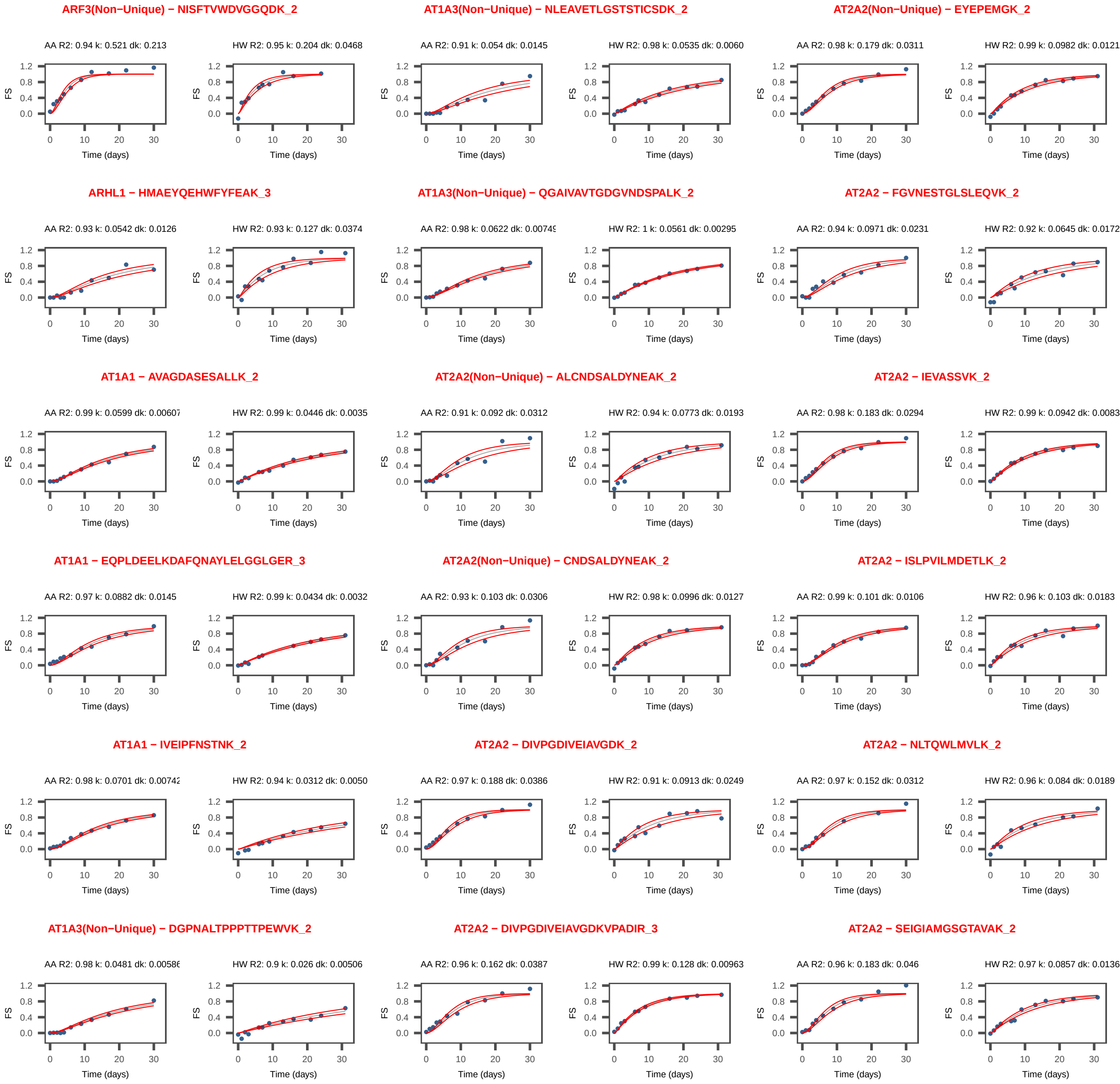
ARF3(Non−Unique) − NISFTVWDVGGQDK_2
AA R2: 0.94 k: 0.521 dk: 0.213
HW R2: 0.95 k: 0.204 dk: 0.0468
AT1A3(Non−Unique) − NLEAVETLGSTSTICSDK_2
AA R2: 0.91 k: 0.054 dk: 0.0145
HW R2: 0.98 k: 0.0535 dk: 0.0060
AT2A2(Non−Unique) − EYEPEMGK_2
AA R2: 0.98 k: 0.179 dk: 0.0311
HW R2: 0.99 k: 0.0982 dk: 0.0121
ARHL1 − HMAEYQEHWFYFEAK_3
AA R2: 0.93 k: 0.0542 dk: 0.0126
HW R2: 0.93 k: 0.127 dk: 0.0374
AT1A3(Non−Unique) − QGAIVAVTGDGVNDSPALK_2
AA R2: 0.98 k: 0.0622 dk: 0.0074
HW R2: 1 k: 0.0561 dk: 0.00295
AT2A2 − FGVNESTGLSLEQVK_2
AA R2: 0.94 k: 0.0971 dk: 0.0231
HW R2: 0.92 k: 0.0645 dk: 0.0172
AT1A1 − AVAGDASESALLK_2
AA R2: 0.99 k: 0.0599 dk: 0.00607
HW R2: 0.99 k: 0.0446 dk: 0.0035
AT2A2(Non−Unique) − ALCNDSALDYNEAK_2
AA R2: 0.91 k: 0.092 dk: 0.0312
HW R2: 0.94 k: 0.0773 dk: 0.0193
AT2A2 − IEVASSVK_2
AA R2: 0.98 k: 0.183 dk: 0.0294
HW R2: 0.99 k: 0.0942 dk: 0.0083
AT1A1 − EQPLDEELKDAFQNAYLELGGLGER_3
AA R2: 0.97 k: 0.0882 dk: 0.0145
HW R2: 0.99 k: 0.0434 dk: 0.0032
AT2A2(Non−Unique) − CNDSALDYNEAK_2
AA R2: 0.93 k: 0.103 dk: 0.0306
HW R2: 0.98 k: 0.0996 dk: 0.0127
AT2A2 − ISLPVILMDETLK_2
AA R2: 0.99 k: 0.101 dk: 0.0106
HW R2: 0.96 k: 0.103 dk: 0.0183
AT1A1 − IVEIPFNSTNK_2
AA R2: 0.98 k: 0.0701 dk: 0.00742
HW R2: 0.94 k: 0.0312 dk: 0.0050
AT2A2 − DIVPGDIVEIAVGDK_2
AA R2: 0.97 k: 0.188 dk: 0.0386
HW R2: 0.91 k: 0.0913 dk: 0.0249
AT2A2 − NLTQWLMVLK_2
AA R2: 0.97 k: 0.152 dk: 0.0312
HW R2: 0.96 k: 0.084 dk: 0.0189
AT1A3(Non−Unique) − DGPNALTPPPTTPEWVK_2
AA R2: 0.98 k: 0.0481 dk: 0.0058
HW R2: 0.9 k: 0.026 dk: 0.00506
AT2A2 − DIVPGDIVEIAVGDKVPADIR_3
AA R2: 0.96 k: 0.162 dk: 0.0387
HW R2: 0.99 k: 0.128 dk: 0.00963
AT2A2 − SEIGIAMGSGTAVAK_2
AA R2: 0.96 k: 0.183 dk: 0.046
HW R2: 0.97 k: 0.0857 dk: 0.0136
FS
Time (days)

AT2A2 − SEIGIAMGSGTAVAK_3

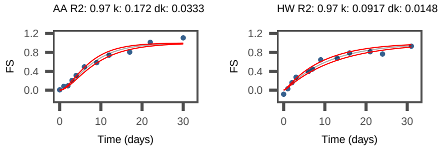

AT2A2(Non−Unique) − VIMITGDNK_2

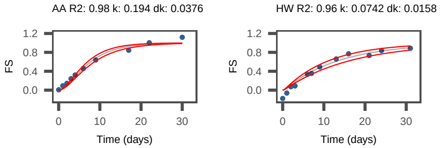

AT5F1 − PLPPLPEYGGK_2

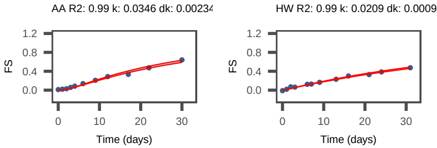

AT2A2 − SITGSTYAPIGEVQK_2

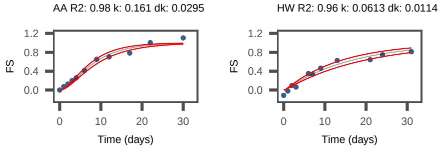

AT2A2 − VPMTPGVK_2

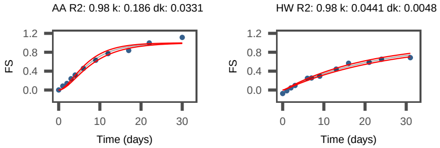

AT5F1 − QIQDAIDMEK_2

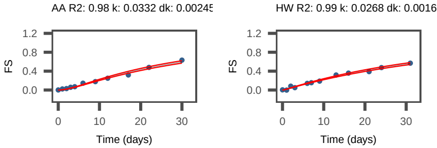

AT2A2(Non−Unique) − SLPSVETLGCTSVICSDK_2

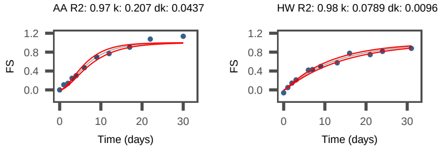

AT2A2 − VSFYQLSHFLQCK_3

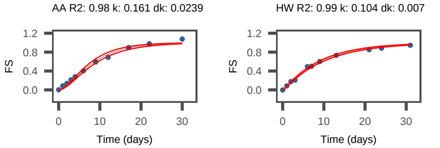

AT5F1 − TGVTGPYVLGTGLSLYFLSK_2

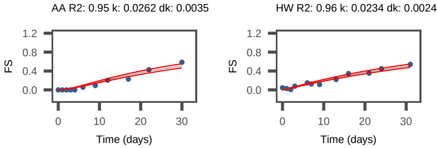

AT2A2 − TVEEVLGHFGVNESTGLSLEQVK_2

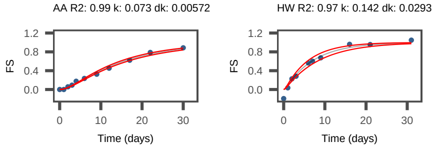

AT2A2 − WGSNELPAEEGK_2

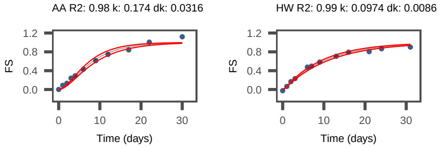

ATP5H − IPVPEDK_2

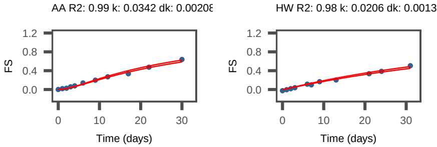

AT2A2(Non−Unique) − VDQSILTGESVSVIK_2

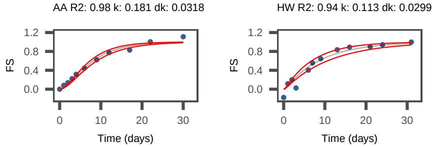

AT5F1 − IAQLEEVK_2

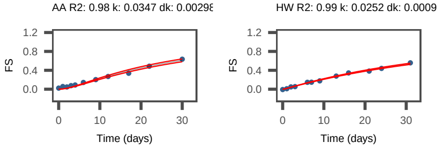

ATP5H − KYPYWPHQPIENL_2

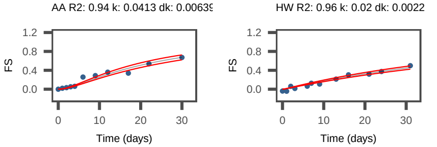

AT2A2 − VEGDTCSLNEFSITGSTYAPIGEVQK_2

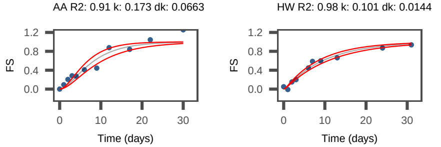

AT5F1 − LGLIPEEFFQFLYPK_3

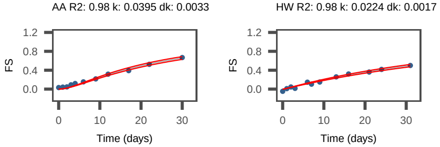

ATP5H − LASLSEKPPAIDWAY_2

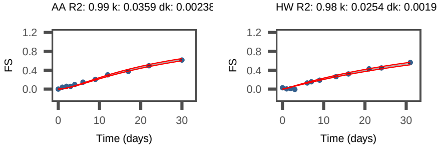

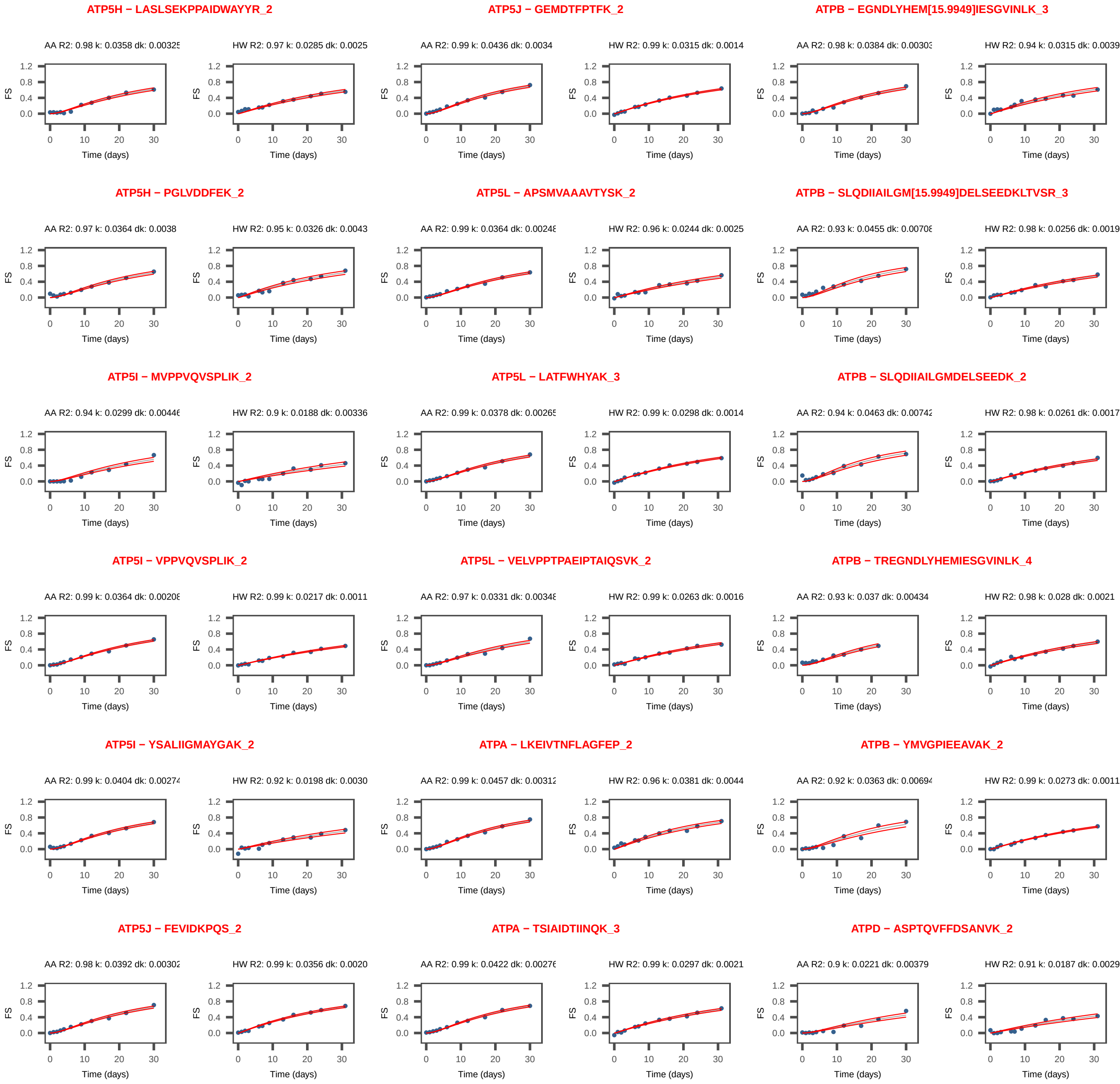
ATP5H – LASLSEKPPAIDWAYYR_2
AA R2: 0.98 k: 0.0358 dk: 0.00325
HW R2: 0.97 k: 0.0285 dk: 0.0025
ATP5J – GEMDTFPTFK_2
AA R2: 0.99 k: 0.0436 dk: 0.0034
HW R2: 0.99 k: 0.0315 dk: 0.0014
ATPB – EGNDLYHEM[15.9949]IESGVINLK_3
AA R2: 0.98 k: 0.0384 dk: 0.00303
HW R2: 0.94 k: 0.0315 dk: 0.0039
ATP5H – PGLVDDFEK_2
AA R2: 0.97 k: 0.0364 dk: 0.0038
HW R2: 0.95 k: 0.0326 dk: 0.0043
ATP5L – APSMVAAAVTYSK_2
AA R2: 0.99 k: 0.0364 dk: 0.00248
HW R2: 0.96 k: 0.0244 dk: 0.0025
ATPB – SLQDIIAILGM[15.9949]DELSEEDKLTVSR_3
AA R2: 0.93 k: 0.0455 dk: 0.00708
HW R2: 0.98 k: 0.0256 dk: 0.0019
ATP5I – MVPPVQVSPLIK_2
AA R2: 0.94 k: 0.0299 dk: 0.00446
HW R2: 0.9 k: 0.0188 dk: 0.00336
ATP5L – LATFWHYAK_3
AA R2: 0.99 k: 0.0378 dk: 0.00265
HW R2: 0.99 k: 0.0298 dk: 0.0014
ATPB – SLQDIIAILGMDELSEEDK_2
AA R2: 0.94 k: 0.0463 dk: 0.00742
HW R2: 0.98 k: 0.0261 dk: 0.0017
ATP5I – VPPVQVSPLIK_2
AA R2: 0.99 k: 0.0364 dk: 0.00208
HW R2: 0.99 k: 0.0217 dk: 0.0011
ATP5L – VELVPPTPAEIPTAIQSVK_2
AA R2: 0.97 k: 0.0331 dk: 0.00348
HW R2: 0.99 k: 0.0263 dk: 0.0016
ATPB – TREGNDLYHEMIESGVINLK_4
AA R2: 0.93 k: 0.037 dk: 0.00434
HW R2: 0.98 k: 0.028 dk: 0.0021
ATP5I – YSALIIGMAYGAK_2
AA R2: 0.99 k: 0.0404 dk: 0.00274
HW R2: 0.92 k: 0.0198 dk: 0.0030
ATPA – LKEIVTNFLAGFEP_2
AA R2: 0.99 k: 0.0457 dk: 0.00312
HW R2: 0.96 k: 0.0381 dk: 0.0044
ATPB – YMVGPIEEAVAK_2
AA R2: 0.92 k: 0.0363 dk: 0.00694
HW R2: 0.99 k: 0.0273 dk: 0.0011
ATP5J – FEVIDKPQS_2
AA R2: 0.98 k: 0.0392 dk: 0.00302
HW R2: 0.99 k: 0.0356 dk: 0.0020
ATPA – TSIAIDTIINQK_3
AA R2: 0.99 k: 0.0422 dk: 0.00276
HW R2: 0.99 k: 0.0297 dk: 0.0021
ATPD – ASPTQVFFDSANVK_2
AA R2: 0.9 k: 0.0221 dk: 0.00379
HW R2: 0.91 k: 0.0187 dk: 0.0029
FS
Time (days)

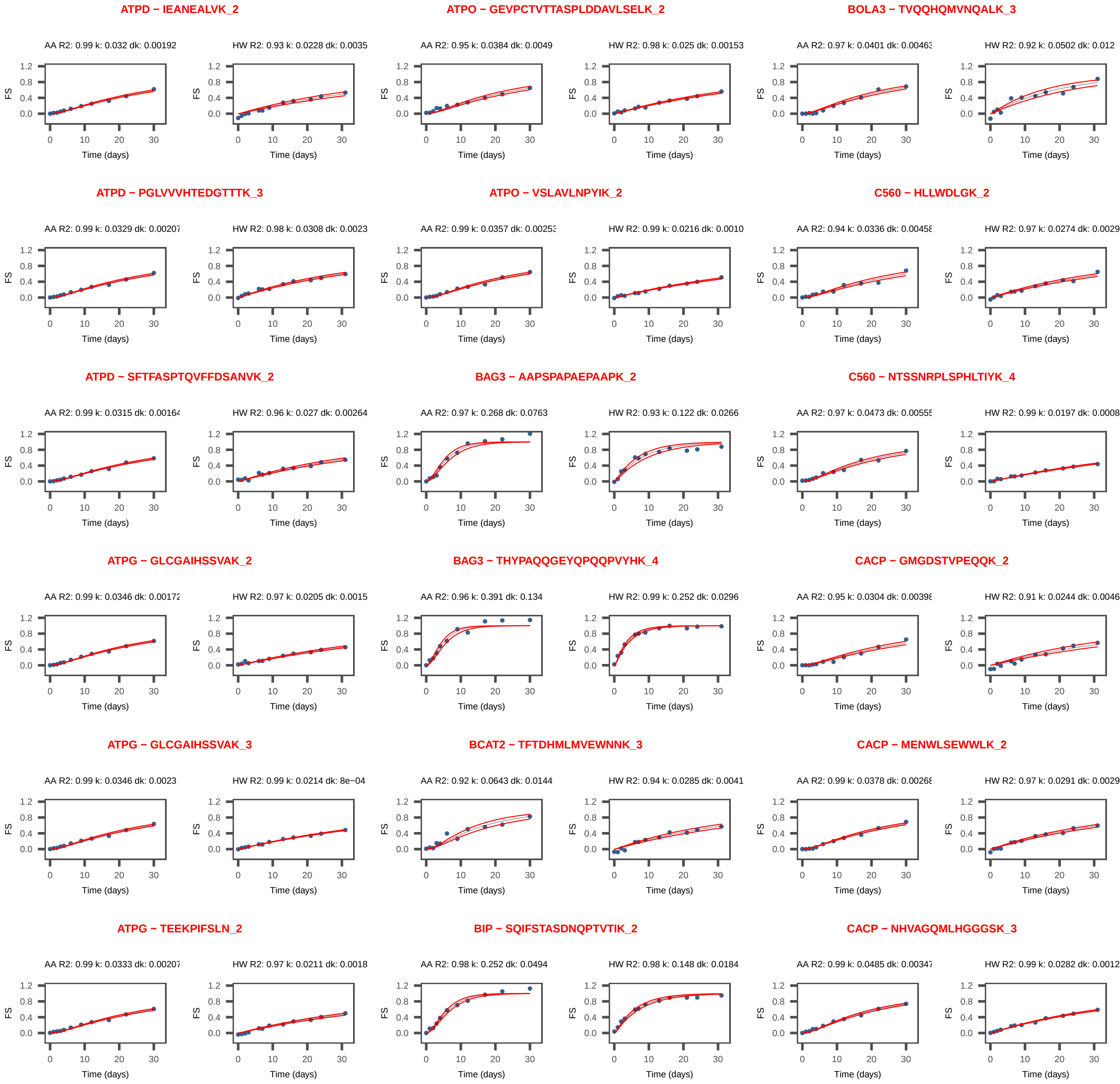
ATPD – IEANEALVK_2
AA R2: 0.99 k: 0.032 dk: 0.00192
HW R2: 0.93 k: 0.0228 dk: 0.0035
ATPO – GEVPCTVTTASPLDDAVLSELK_2
AA R2: 0.95 k: 0.0384 dk: 0.0049
HW R2: 0.98 k: 0.025 dk: 0.00153
BOLA3 – TVQQHQMVNQALK_3
AA R2: 0.97 k: 0.0401 dk: 0.00463
HW R2: 0.92 k: 0.0502 dk: 0.012
ATPD – PGLVVVHTEDGTTTK_3
AA R2: 0.99 k: 0.0329 dk: 0.00207
HW R2: 0.98 k: 0.0308 dk: 0.0023
ATPO – VSLAVLNPYIK_2
AA R2: 0.99 k: 0.0357 dk: 0.00253
HW R2: 0.99 k: 0.0216 dk: 0.0010
C560 – HLLWDLGK_2
AA R2: 0.94 k: 0.0336 dk: 0.00458
HW R2: 0.97 k: 0.0274 dk: 0.0029
ATPD – SFTFASPTQVFFDSANVK_2
AA R2: 0.99 k: 0.0315 dk: 0.00164
HW R2: 0.96 k: 0.027 dk: 0.00264
BAG3 – AAPSPAPAEPAAPK_2
AA R2: 0.97 k: 0.268 dk: 0.0763
HW R2: 0.93 k: 0.122 dk: 0.0266
C560 – NTSSNRPLSPHLTIYK_4
AA R2: 0.97 k: 0.0473 dk: 0.00555
HW R2: 0.99 k: 0.0197 dk: 0.0008
ATPG – GLCGAIHSSVAK_2
AA R2: 0.99 k: 0.0346 dk: 0.00172
HW R2: 0.97 k: 0.0205 dk: 0.0015
BAG3 – THYPAQQGEYQPQQPVYHK_4
AA R2: 0.96 k: 0.391 dk: 0.134
HW R2: 0.99 k: 0.252 dk: 0.0296
CACP – GMGDSTVPEQQK_2
AA R2: 0.95 k: 0.0304 dk: 0.00398
HW R2: 0.91 k: 0.0244 dk: 0.0046
ATPG – GLCGAIHSSVAK_3
AA R2: 0.99 k: 0.0346 dk: 0.0023
HW R2: 0.99 k: 0.0214 dk: 8e−04
BCAT2 – TFTDHMLMVEWNNK_3
AA R2: 0.92 k: 0.0643 dk: 0.0144
HW R2: 0.94 k: 0.0285 dk: 0.0041
CACP – MENWLSEWWLK_2
AA R2: 0.99 k: 0.0378 dk: 0.00268
HW R2: 0.97 k: 0.0291 dk: 0.0029
ATPG – TEEKPIFSLN_2
AA R2: 0.99 k: 0.0333 dk: 0.00207
HW R2: 0.97 k: 0.0211 dk: 0.0018
BIP – SQIFSTASDNQPTVTIK_2
AA R2: 0.98 k: 0.252 dk: 0.0494
HW R2: 0.98 k: 0.148 dk: 0.0184
CACP – NHVAGQMLHGGGSK_3
AA R2: 0.99 k: 0.0485 dk: 0.00347
HW R2: 0.99 k: 0.0282 dk: 0.0012
FS
Time (days)

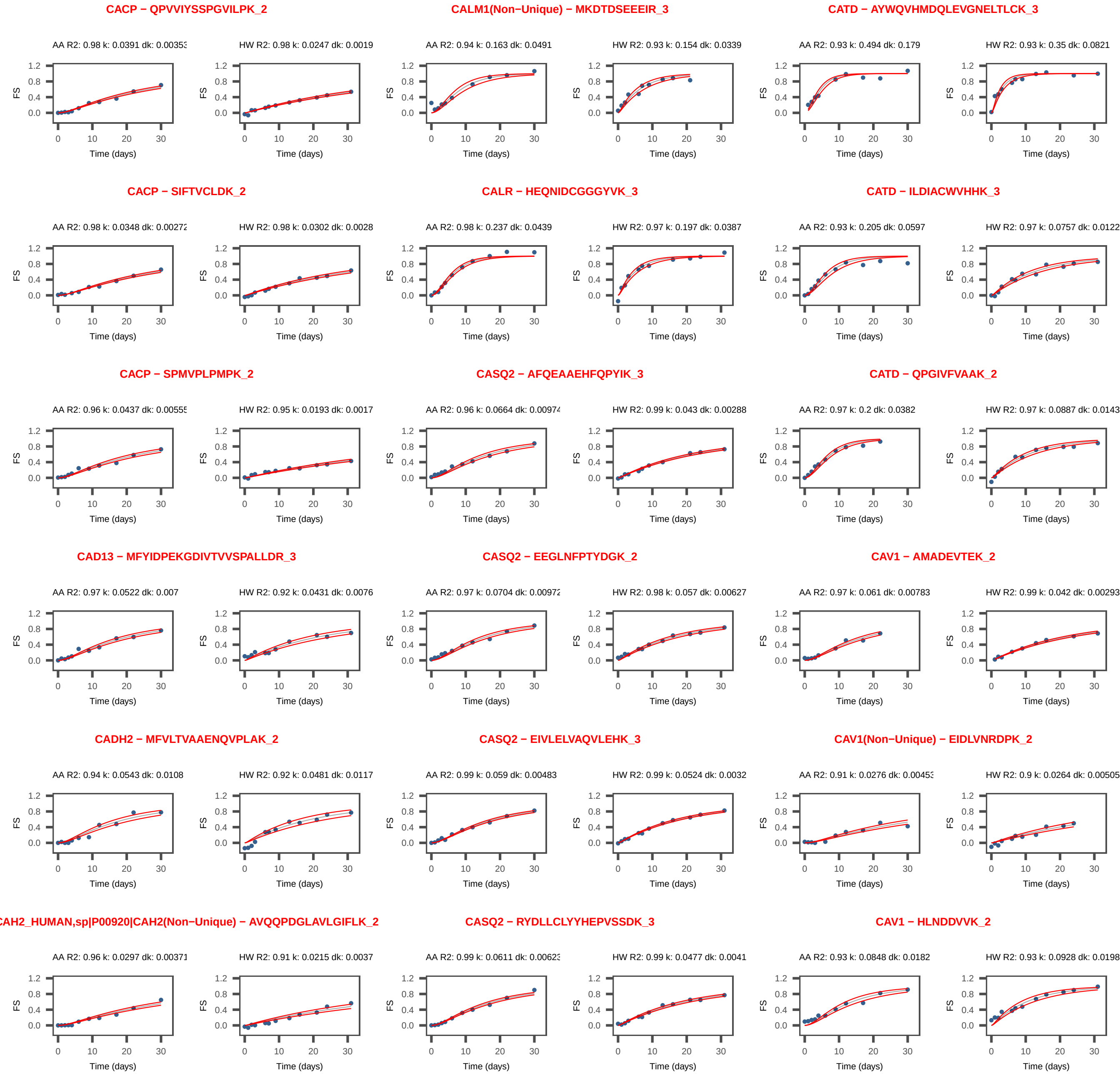
CACP – QPVVIYSSPGVILPK_2
AA R2: 0.98 k: 0.0391 dk: 0.00353
HW R2: 0.98 k: 0.0247 dk: 0.0019
CALM1(Non–Unique) – MKDTDSEEEIR_3
AA R2: 0.94 k: 0.163 dk: 0.0491
HW R2: 0.93 k: 0.154 dk: 0.0339
CATD – AYWQVHMDQLEVGNELTLCK_3
AA R2: 0.93 k: 0.494 dk: 0.179
HW R2: 0.93 k: 0.35 dk: 0.0821
CACP – SIFTVCLDK_2
AA R2: 0.98 k: 0.0348 dk: 0.00272
HW R2: 0.98 k: 0.0302 dk: 0.0028
CALR – HEQNIDCGGGYVK_3
AA R2: 0.98 k: 0.237 dk: 0.0439
HW R2: 0.97 k: 0.197 dk: 0.0387
CATD – ILDIACWVHHK_3
AA R2: 0.93 k: 0.205 dk: 0.0597
HW R2: 0.97 k: 0.0757 dk: 0.0122
CACP – SPMVPLPMPK_2
AA R2: 0.96 k: 0.0437 dk: 0.00555
HW R2: 0.95 k: 0.0193 dk: 0.0017
CASQ2 – AFQEAAEHFQPYIK_3
AA R2: 0.96 k: 0.0664 dk: 0.00974
HW R2: 0.99 k: 0.043 dk: 0.00288
CATD – QPGIVFVAAK_2
AA R2: 0.97 k: 0.2 dk: 0.0382
HW R2: 0.97 k: 0.0887 dk: 0.0143
CAD13 – MFYIDPEKGDIVTVVSPALLDR_3
AA R2: 0.97 k: 0.0522 dk: 0.007
HW R2: 0.92 k: 0.0431 dk: 0.0076
CASQ2 – EEGLNFPTYDGK_2
AA R2: 0.97 k: 0.0704 dk: 0.00972
HW R2: 0.98 k: 0.057 dk: 0.00627
CAV1 – AMADEVTEK_2
AA R2: 0.97 k: 0.061 dk: 0.00783
HW R2: 0.99 k: 0.042 dk: 0.00293
CADH2 – MFVLTVAAENQVPLAK_2
AA R2: 0.94 k: 0.0543 dk: 0.0108
HW R2: 0.92 k: 0.0481 dk: 0.0117
CASQ2 – EIVLELVAQVLEHK_3
AA R2: 0.99 k: 0.059 dk: 0.00483
HW R2: 0.99 k: 0.0524 dk: 0.0032
CAV1(Non–Unique) – EIDLVNRDPK_2
AA R2: 0.91 k: 0.0276 dk: 0.00453
HW R2: 0.9 k: 0.0264 dk: 0.00505
CAH2_HUMAN,sp|P00920|CAH2(Non–Unique) – AVQQPDGLAVLGIFLK_2
AA R2: 0.96 k: 0.0297 dk: 0.00371
HW R2: 0.91 k: 0.0215 dk: 0.0037
CASQ2 – RYDLLCLYYHEPVSSDK_3
AA R2: 0.99 k: 0.0611 dk: 0.00623
HW R2: 0.99 k: 0.0477 dk: 0.0041
CAV1 – HLNDDVVK_2
AA R2: 0.93 k: 0.0848 dk: 0.0182
HW R2: 0.93 k: 0.0928 dk: 0.0198
FS
Time (days)

CAVN1 – ATEEPSGTGSDELIK_2

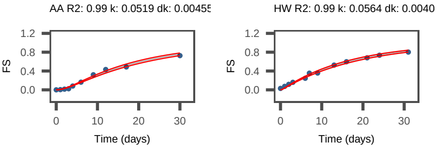

CAVN4 – VVIFQEDIPCPASLSVVK_2

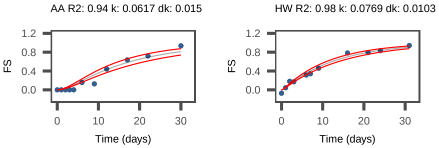

CH10 – VLQATVVAVGSGGK_2

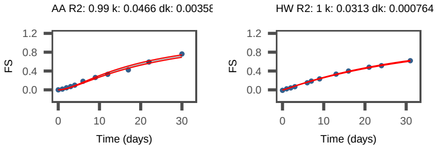

CAVN1 – KLEVNEAELLR_2

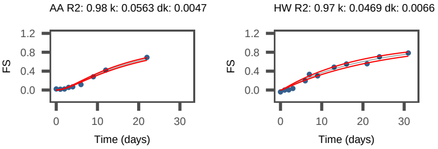

CAZA2 – FTVTPSTTQVVGILK_2

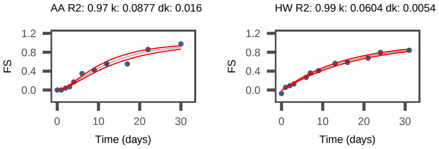

CH10 – VVLDDKDYFLFR_3

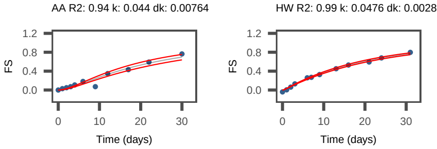

CAVN1 – QAEMEGAVQSIQGELSK_2

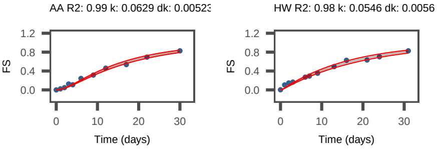

CD36 – EGKPVYISLPH_3

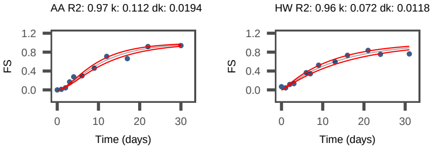

CH60 – ALMLQGVDLLADAVAVTMGPK_2

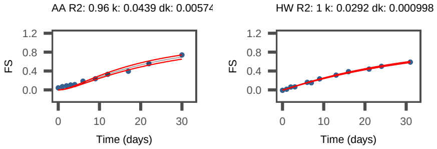

CAVN1 – VMIYQDEVK_2

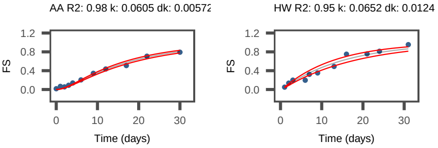

CD36 – LQVNILVK_2

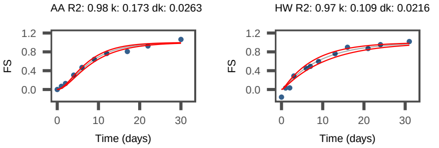

CH60 – CEFQDAYVLLSEK_3

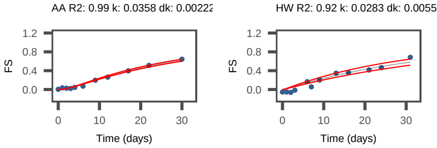

CAVN1 – VPPFTFHVK_3

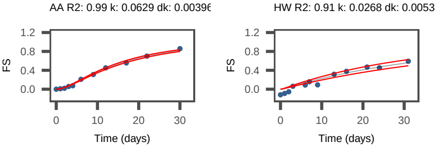

CD36 – SFVQVVLNSLIK_2

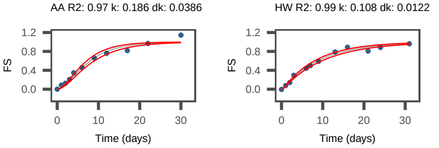

CH60 – DIGNIISDAMK_2

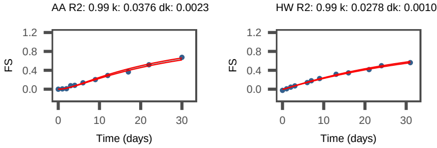

CAVN2 – VLIFQEESEIPASVFVK_2

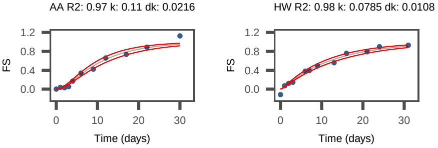

CH10 – VLLPEYGGTK_2

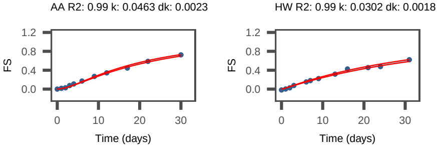

CH60 – GVMLAVDAVIAELK_2

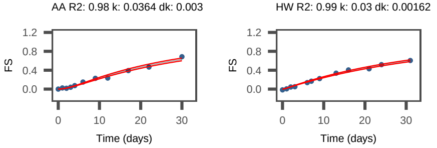

CH60 − GVMLAVDAVIAELK_3

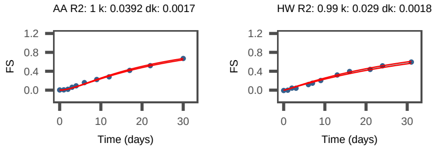

CH60 − VGGTSDVEVNEK_2

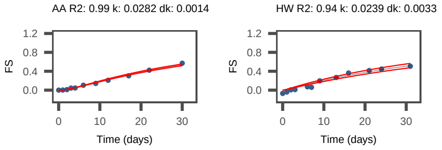

CLH1 − NLQNLLILTAIK_2

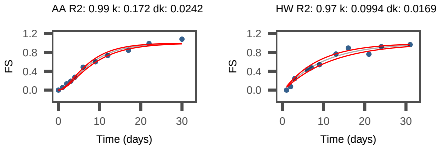

CH60 − IGIEIIK_2

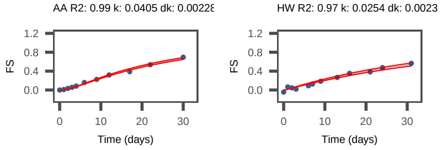

CISD1 − HNEETGDNVGPLIIK_3

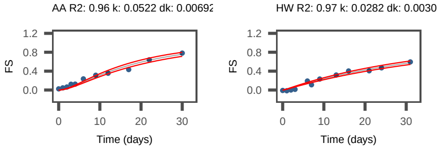

CLYBL − IQWAEELIAAFK_2

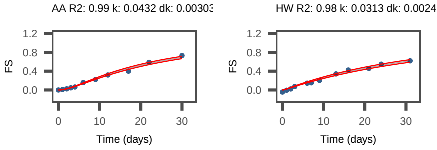

CH60 − ILQSSSEVGYDAMLGDFVNMVEK_2

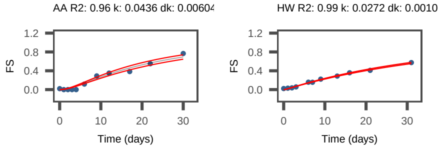

CISD1 − VVHAFDMEDLGDK_3

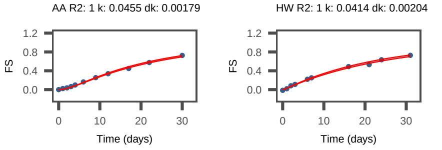

CMC1 − FGLYLPK_2

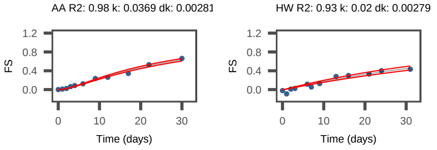

CH60 − ILQSSSEVGYDAMLGDFVNMVEK_3

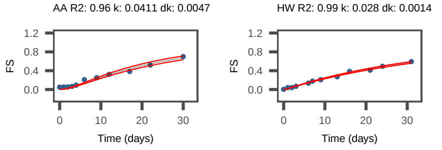

CISY − ALGFPLERPK_3

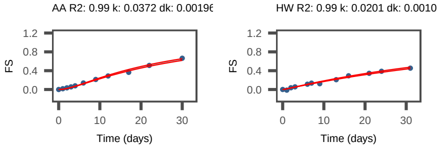

CMC1 − FTLGSVAGAVGATAVYPIDLVK_2

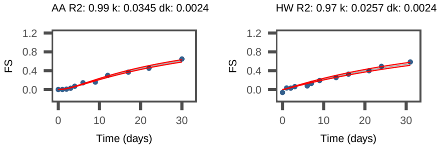

CH60 − NAGVEGSLIVEK_2

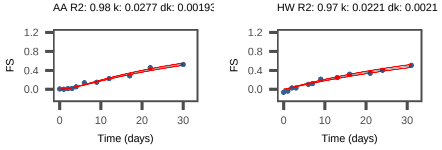

CISY − DVLSNLIPK_2

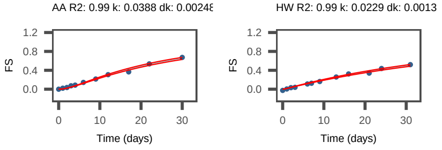

CMC1(Non−Unique) − GLIPQLIGVAPEK_2

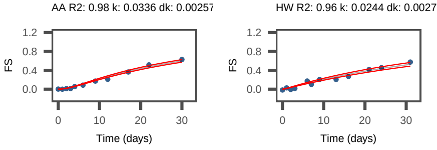

CH60 − VGEVIVTK_2

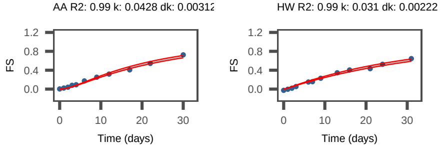

CLH1 − KDPELWGSVLLESNPYR_3

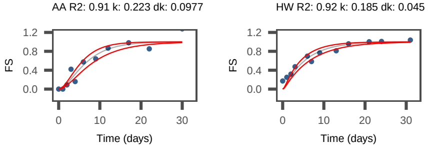

CMC1 − IVQLLAGVADQTK_2

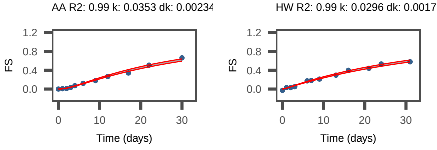

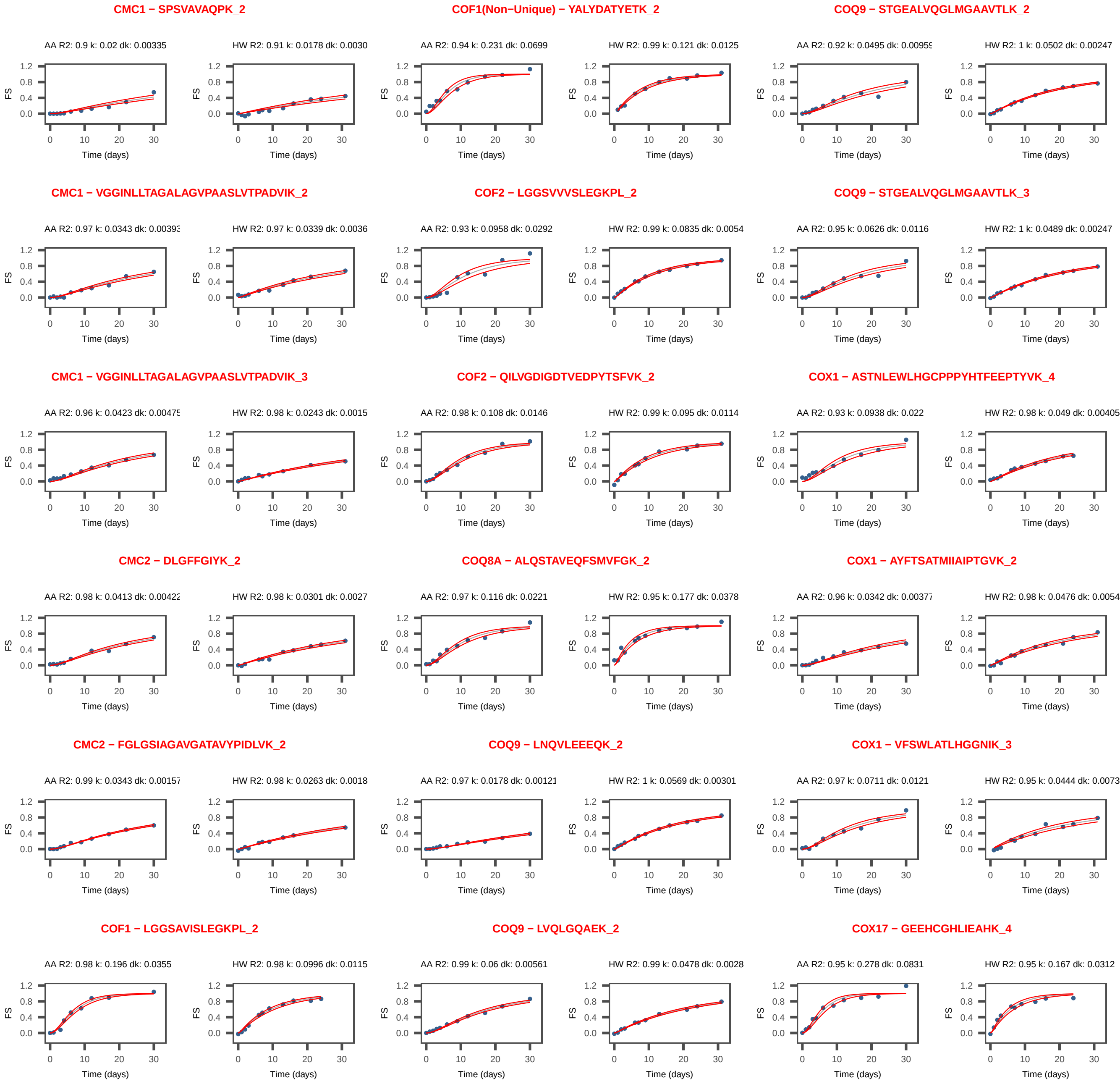
CMC1 − SPSVAVAQPK_2
AA R2: 0.9 k: 0.02 dk: 0.00335
HW R2: 0.91 k: 0.0178 dk: 0.0030
COF1(Non−Unique) − YALYDATYETK_2
AA R2: 0.94 k: 0.231 dk: 0.0699
HW R2: 0.99 k: 0.121 dk: 0.0125
COQ9 − STGEALVQGLMGAAVTLK_2
AA R2: 0.92 k: 0.0495 dk: 0.00959
HW R2: 1 k: 0.0502 dk: 0.00247
CMC1 − VGGINLLTAGALAGVPAASLVTPADVIK_2
AA R2: 0.97 k: 0.0343 dk: 0.00393
HW R2: 0.97 k: 0.0339 dk: 0.0036
COF2 − LGGSVVVSLEGKPL_2
AA R2: 0.93 k: 0.0958 dk: 0.0292
HW R2: 0.99 k: 0.0835 dk: 0.0054
COQ9 − STGEALVQGLMGAAVTLK_3
AA R2: 0.95 k: 0.0626 dk: 0.0116
HW R2: 1 k: 0.0489 dk: 0.00247
CMC1 − VGGINLLTAGALAGVPAASLVTPADVIK_3
AA R2: 0.96 k: 0.0423 dk: 0.00475
HW R2: 0.98 k: 0.0243 dk: 0.0015
COF2 − QILVGDIGDTVEDPYTSFVK_2
AA R2: 0.98 k: 0.108 dk: 0.0146
HW R2: 0.99 k: 0.095 dk: 0.0114
COX1 − ASTNLEWLHGCPPPYHTFEEPTYVK_4
AA R2: 0.93 k: 0.0938 dk: 0.022
HW R2: 0.98 k: 0.049 dk: 0.00405
CMC2 − DLGFFGIYK_2
AA R2: 0.98 k: 0.0413 dk: 0.00422
HW R2: 0.98 k: 0.0301 dk: 0.0027
COQ8A − ALQSTAVEQFSMVFGK_2
AA R2: 0.97 k: 0.116 dk: 0.0221
HW R2: 0.95 k: 0.177 dk: 0.0378
COX1 − AYFTSATMIIAIPTGVK_2
AA R2: 0.96 k: 0.0342 dk: 0.00377
HW R2: 0.98 k: 0.0476 dk: 0.0054
CMC2 − FGLGSIAGAVGATAVYPIDLVK_2
AA R2: 0.99 k: 0.0343 dk: 0.00157
HW R2: 0.98 k: 0.0263 dk: 0.0018
COQ9 − LNQVLEEEQK_2
AA R2: 0.97 k: 0.0178 dk: 0.00121
HW R2: 1 k: 0.0569 dk: 0.00301
COX1 − VFSWLATLHGGNIK_3
AA R2: 0.97 k: 0.0711 dk: 0.0121
HW R2: 0.95 k: 0.0444 dk: 0.0073
COF1 − LGGSAVISLEGKPL_2
AA R2: 0.98 k: 0.196 dk: 0.0355
HW R2: 0.98 k: 0.0996 dk: 0.0115
COQ9 − LVQLGQAEK_2
AA R2: 0.99 k: 0.06 dk: 0.00561
HW R2: 0.99 k: 0.0478 dk: 0.0028
COX17 − GEEHCGHLIEAHK_4
AA R2: 0.95 k: 0.278 dk: 0.0831
HW R2: 0.95 k: 0.167 dk: 0.0312
FS
Time (days)

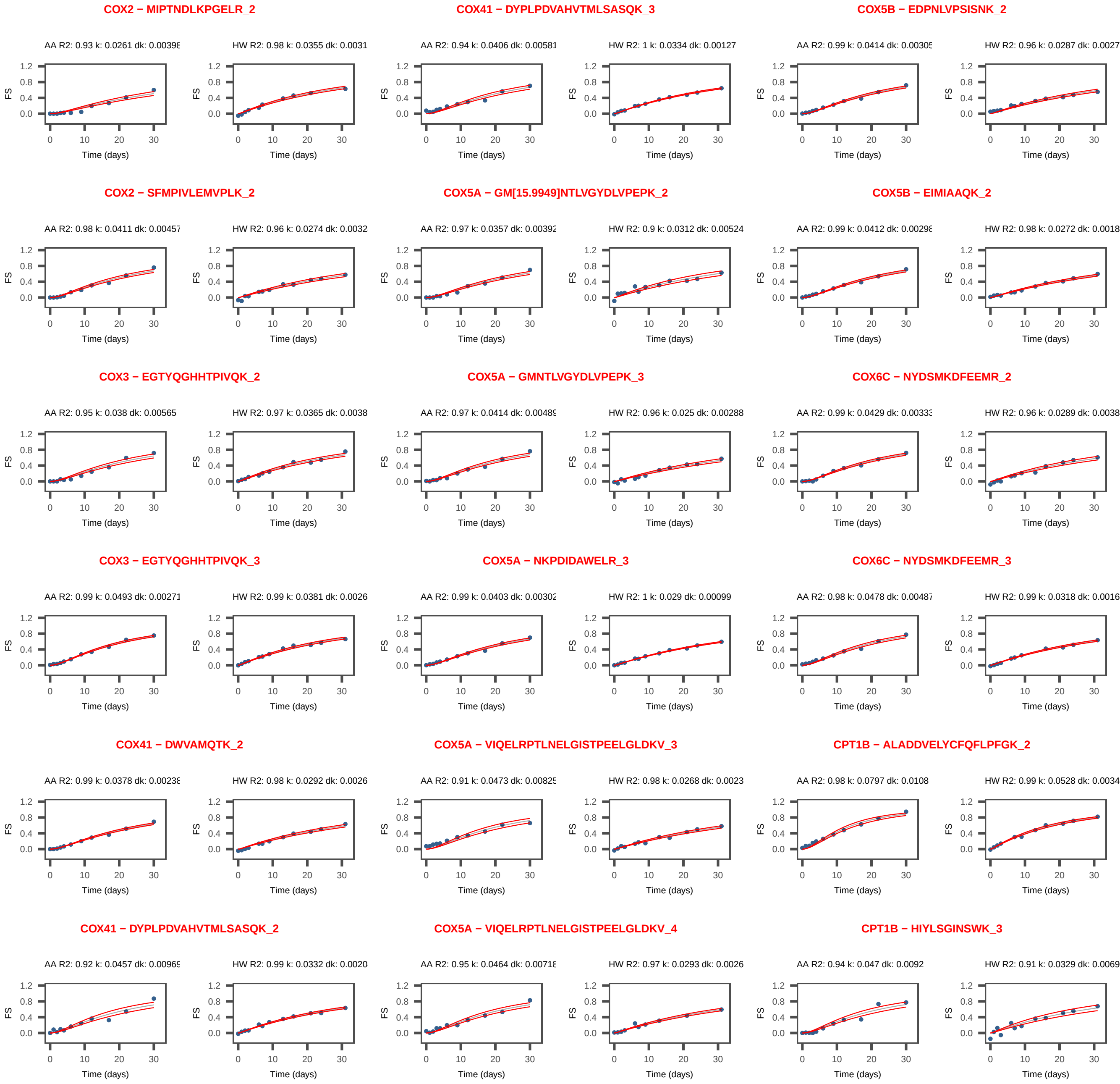
COX2 − MIPTNDLKPGELR_2
AA R2: 0.93 k: 0.0261 dk: 0.0039
HW R2: 0.98 k: 0.0355 dk: 0.0031
COX41 − DYPLPDVAHVTMLSASQK_3
AA R2: 0.94 k: 0.0406 dk: 0.00581
HW R2: 1 k: 0.0334 dk: 0.00127
COX5B − EDPNLVPSISNK_2
AA R2: 0.99 k: 0.0414 dk: 0.00305
HW R2: 0.96 k: 0.0287 dk: 0.0027
COX2 − SFMPIVLEMVPLK_2
AA R2: 0.98 k: 0.0411 dk: 0.00457
HW R2: 0.96 k: 0.0274 dk: 0.0032
COX5A − GM[15.9949]NTLVGYDLVPEPK_2
AA R2: 0.97 k: 0.0357 dk: 0.00392
HW R2: 0.9 k: 0.0312 dk: 0.00524
COX5B − EIMIAAQK_2
AA R2: 0.99 k: 0.0412 dk: 0.0029
HW R2: 0.98 k: 0.0272 dk: 0.0018
COX3 − EGTYQGHHTPIVQK_2
AA R2: 0.95 k: 0.038 dk: 0.00565
HW R2: 0.97 k: 0.0365 dk: 0.0038
COX5A − GMNTLVGYDLVPEPK_3
AA R2: 0.97 k: 0.0414 dk: 0.0048
HW R2: 0.96 k: 0.025 dk: 0.00288
COX6C − NYDSMKDFEEMR_2
AA R2: 0.99 k: 0.0429 dk: 0.0033
HW R2: 0.96 k: 0.0289 dk: 0.0038
COX3 − EGTYQGHHTPIVQK_3
AA R2: 0.99 k: 0.0493 dk: 0.00271
HW R2: 0.99 k: 0.0381 dk: 0.0026
COX5A − NKPDIDAWELR_3
AA R2: 0.99 k: 0.0403 dk: 0.00302
HW R2: 1 k: 0.029 dk: 0.00099
COX6C − NYDSMKDFEEMR_3
AA R2: 0.98 k: 0.0478 dk: 0.00487
HW R2: 0.99 k: 0.0318 dk: 0.0016
COX41 − DWVAMQTK_2
AA R2: 0.99 k: 0.0378 dk: 0.0023
HW R2: 0.98 k: 0.0292 dk: 0.0026
COX5A − VIQELRPTLNELGISTPEELGLDKV_3
AA R2: 0.91 k: 0.0473 dk: 0.00825
HW R2: 0.98 k: 0.0268 dk: 0.0023
CPT1B − ALADDVELYCFQFLPFGK_2
AA R2: 0.98 k: 0.0797 dk: 0.0108
HW R2: 0.99 k: 0.0528 dk: 0.0034
COX41 − DYPLPDVAHVTMLSASQK_2
AA R2: 0.92 k: 0.0457 dk: 0.0096
HW R2: 0.99 k: 0.0332 dk: 0.0020
COX5A − VIQELRPTLNELGISTPEELGLDKV_4
AA R2: 0.95 k: 0.0464 dk: 0.0071
HW R2: 0.97 k: 0.0293 dk: 0.0026
CPT1B − HIYLSGINSWK_3
AA R2: 0.94 k: 0.047 dk: 0.0092
HW R2: 0.91 k: 0.0329 dk: 0.0069
FS
Time (days)

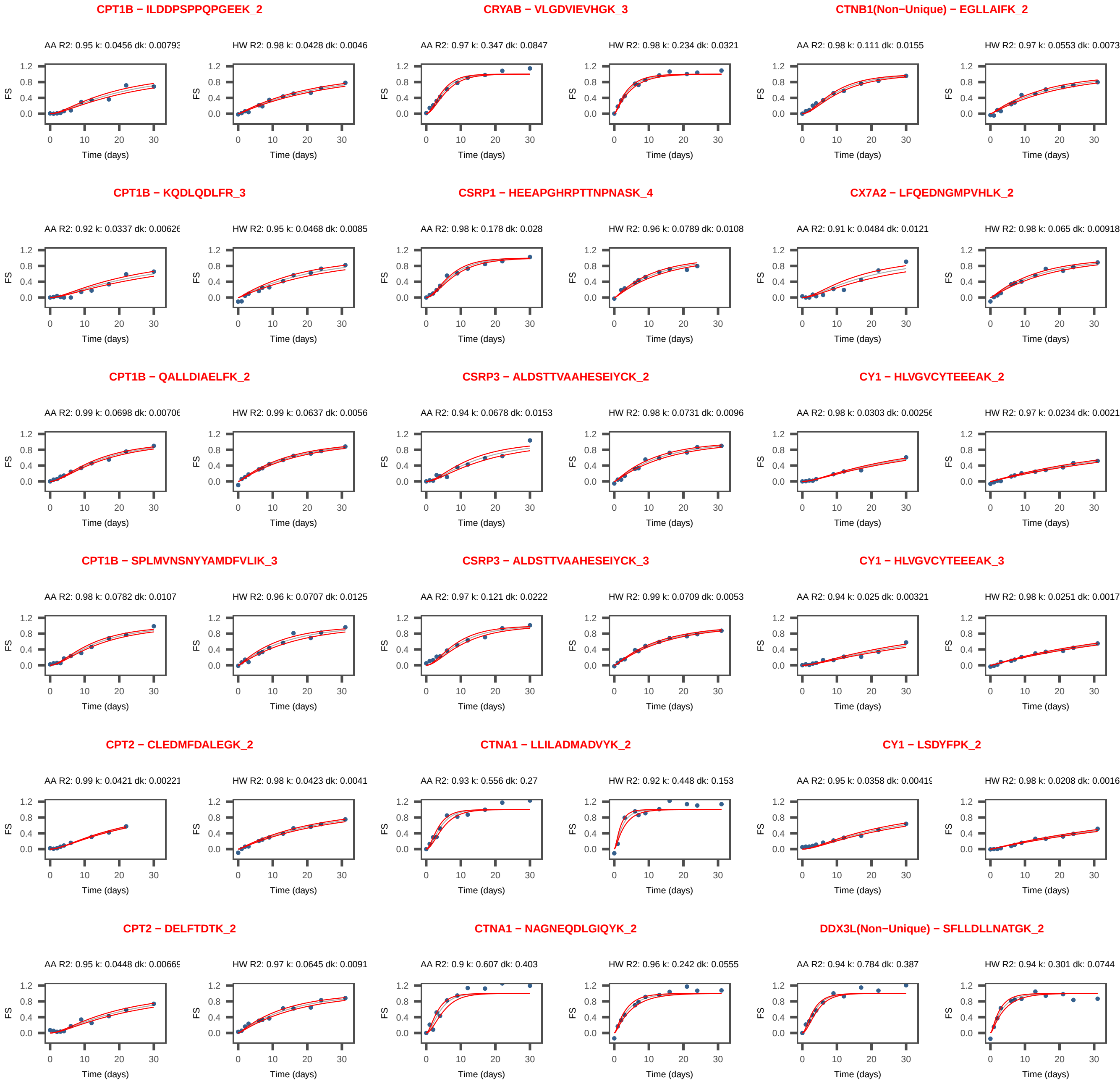
CPT1B − ILDDPSPPQPGEEK_2
AA R2: 0.95 k: 0.0456 dk: 0.00793
HW R2: 0.98 k: 0.0428 dk: 0.0046
CRYAB − VLGDVIEVHGK_3
AA R2: 0.97 k: 0.347 dk: 0.0847
HW R2: 0.98 k: 0.234 dk: 0.0321
CTNB1(Non−Unique) − EGLLAIFK_2
AA R2: 0.98 k: 0.111 dk: 0.0155
HW R2: 0.97 k: 0.0553 dk: 0.0073
CPT1B − KQDLQDLFR_3
AA R2: 0.92 k: 0.0337 dk: 0.00626
HW R2: 0.95 k: 0.0468 dk: 0.0085
CSRP1 − HEEAPGHRPTTNPNASK_4
AA R2: 0.98 k: 0.178 dk: 0.028
HW R2: 0.96 k: 0.0789 dk: 0.0108
CX7A2 − LFQEDNGMPVHLK_2
AA R2: 0.91 k: 0.0484 dk: 0.0121
HW R2: 0.98 k: 0.065 dk: 0.00918
CPT1B − QALLDIAELFK_2
AA R2: 0.99 k: 0.0698 dk: 0.00706
HW R2: 0.99 k: 0.0637 dk: 0.0056
CSRP3 − ALDSTTVAAHESEIYCK_2
AA R2: 0.94 k: 0.0678 dk: 0.0153
HW R2: 0.98 k: 0.0731 dk: 0.0096
CY1 − HLVGVCYTEEEAK_2
AA R2: 0.98 k: 0.0303 dk: 0.00256
HW R2: 0.97 k: 0.0234 dk: 0.0021
CPT1B − SPLMVNSNYYAMDFVLIK_3
AA R2: 0.98 k: 0.0782 dk: 0.0107
HW R2: 0.96 k: 0.0707 dk: 0.0125
CSRP3 − ALDSTTVAAHESEIYCK_3
AA R2: 0.97 k: 0.121 dk: 0.0222
HW R2: 0.99 k: 0.0709 dk: 0.0053
CY1 − HLVGVCYTEEEAK_3
AA R2: 0.94 k: 0.025 dk: 0.00321
HW R2: 0.98 k: 0.0251 dk: 0.0017
CPT2 − CLEDMFDALEGK_2
AA R2: 0.99 k: 0.0421 dk: 0.00221
HW R2: 0.98 k: 0.0423 dk: 0.0041
CTNA1 − LLILADMADVYK_2
AA R2: 0.93 k: 0.556 dk: 0.27
HW R2: 0.92 k: 0.448 dk: 0.153
CY1 − LSDYFPK_2
AA R2: 0.95 k: 0.0358 dk: 0.00419
HW R2: 0.98 k: 0.0208 dk: 0.0016
CPT2 − DELFTDTK_2
AA R2: 0.95 k: 0.0448 dk: 0.00669
HW R2: 0.97 k: 0.0645 dk: 0.0091
CTNA1 − NAGNEQDLGIQYK_2
AA R2: 0.9 k: 0.607 dk: 0.403
HW R2: 0.96 k: 0.242 dk: 0.0555
DDX3L(Non−Unique) − SFLLDLLNATGK_2
AA R2: 0.94 k: 0.784 dk: 0.387
HW R2: 0.94 k: 0.301 dk: 0.0744
FS
Time (days)

DECR − CDVRDPDMVHNTVLELIK_4

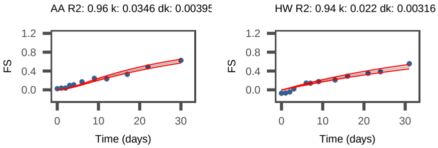

DESM − RIESLNEEIAFLK_3

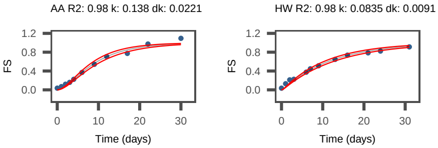

DESP − NLLLAEQGSTHHITVK_3

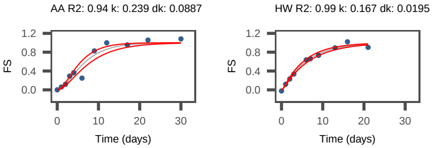

DECR − VAFITGGGTGLGK_2

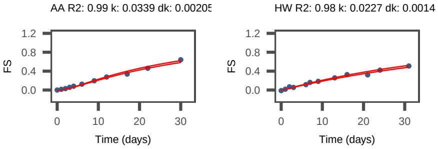

DESP − ALLQAILQTEDMLK_2

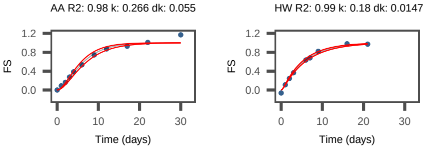

DESP − NMPLQHLLEQIK_3

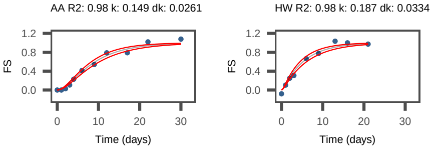

DECR − VTKEEWDIIEGLIR_3

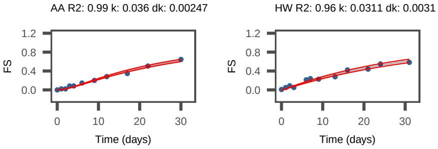

DESP − GFFDPNTEENLTYLQLK_2

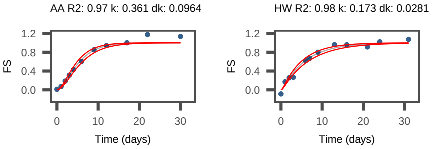

DESP − SVQNDSQALAEVLNQLK_3

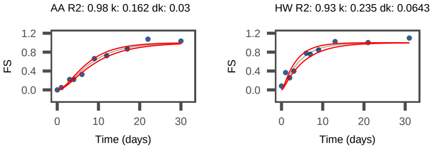

DESM − AQYETIAAK_2

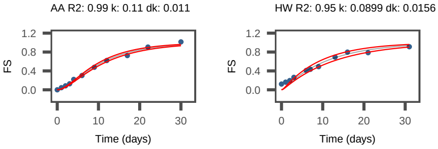

DESP − ITNLTQQLEQASIVK_2

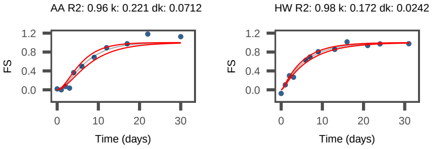

DEST − EILVGDVGATITDPFK_2

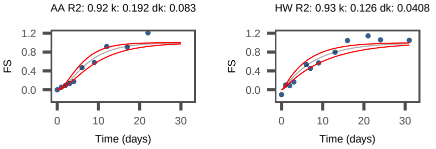

DESM − HQIQSYTCEIDALK_2

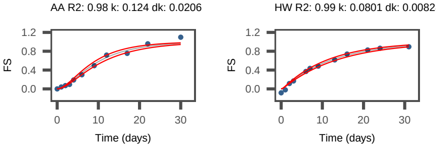

DESP − LLEAQACTGGIIHPTTGQK_3

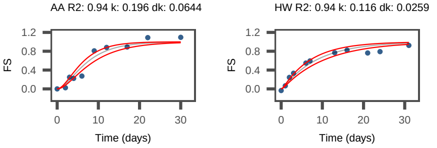

DHPR − GAVHQLCQSLAGK_2

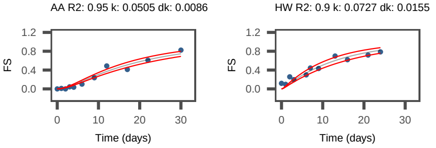

DESM − NISEAEEWYK_2

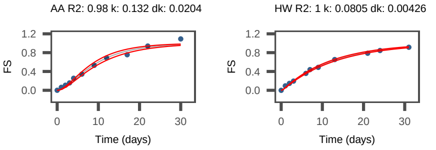

DESP − LLEAQLASGGVVDPVNSVFLPK_2

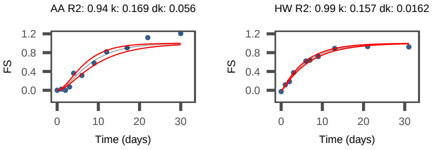

DLDH − ADQPIEADVTVIGSGPGGYVAAIK_2

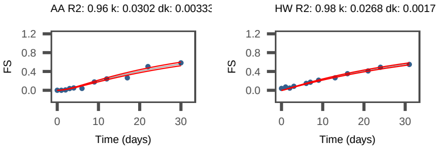

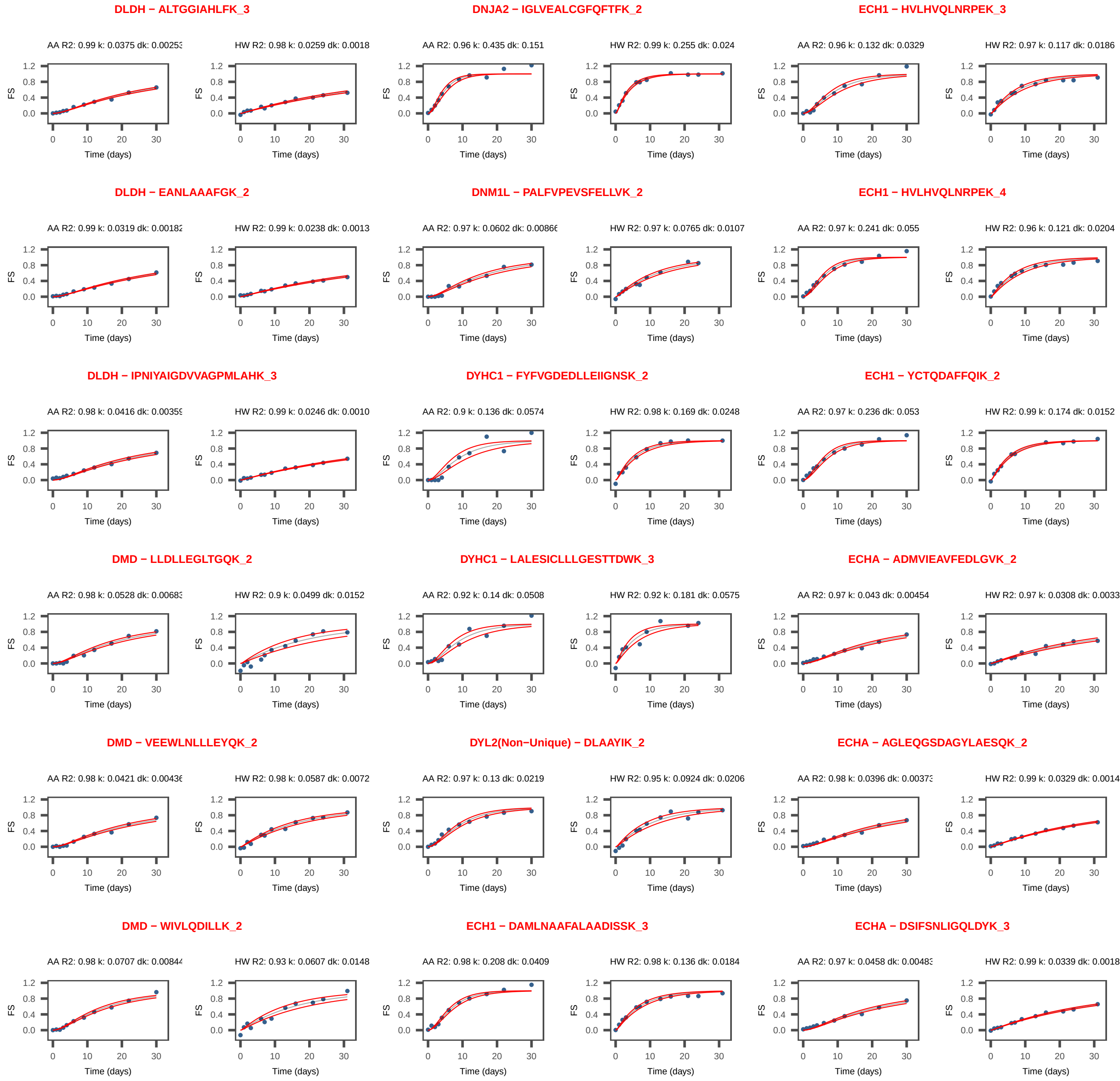
DLDH − ALTGGIAHLFK_3
AA R2: 0.99 k: 0.0375 dk: 0.00253
HW R2: 0.98 k: 0.0259 dk: 0.0018
DNJA2 − IGLVEALCGFQFTFK_2
AA R2: 0.96 k: 0.435 dk: 0.151
HW R2: 0.99 k: 0.255 dk: 0.024
ECH1 − HVLHVQLNRPEK_3
AA R2: 0.96 k: 0.132 dk: 0.0329
HW R2: 0.97 k: 0.117 dk: 0.0186
DLDH − EANLAAAFGK_2
AA R2: 0.99 k: 0.0319 dk: 0.00182
HW R2: 0.99 k: 0.0238 dk: 0.0013
DNM1L − PALFVPEVSFELLVK_2
AA R2: 0.97 k: 0.0602 dk: 0.00866
HW R2: 0.97 k: 0.0765 dk: 0.0107
ECH1 − HVLHVQLNRPEK_4
AA R2: 0.97 k: 0.241 dk: 0.055
HW R2: 0.96 k: 0.121 dk: 0.0204
DLDH − IPNIYAIGDVVAGPMLAHK_3
AA R2: 0.98 k: 0.0416 dk: 0.00359
HW R2: 0.99 k: 0.0246 dk: 0.0010
DYHC1 − FYFVGDEDLLEIIGNSK_2
AA R2: 0.9 k: 0.136 dk: 0.0574
HW R2: 0.98 k: 0.169 dk: 0.0248
ECH1 − YCTQDAFFQIK_2
AA R2: 0.97 k: 0.236 dk: 0.053
HW R2: 0.99 k: 0.174 dk: 0.0152
DMD − LLDLLEGLTGQK_2
AA R2: 0.98 k: 0.0528 dk: 0.00683
HW R2: 0.9 k: 0.0499 dk: 0.0152
DYHC1 − LALESICLLLGESTTDWK_3
AA R2: 0.92 k: 0.14 dk: 0.0508
HW R2: 0.92 k: 0.181 dk: 0.0575
ECHA − ADMVIEAVFEDLGVK_2
AA R2: 0.97 k: 0.043 dk: 0.00454
HW R2: 0.97 k: 0.0308 dk: 0.0033
DMD − VEEWLNLLLEYQK_2
AA R2: 0.98 k: 0.0421 dk: 0.00436
HW R2: 0.98 k: 0.0587 dk: 0.0072
DYL2(Non−Unique) − DLAAYIK_2
AA R2: 0.97 k: 0.13 dk: 0.0219
HW R2: 0.95 k: 0.0924 dk: 0.0206
ECHA − AGLEQGSDAGYLAESQK_2
AA R2: 0.98 k: 0.0396 dk: 0.00373
HW R2: 0.99 k: 0.0329 dk: 0.0014
DMD − WIVLQDILLK_2
AA R2: 0.98 k: 0.0707 dk: 0.00844
HW R2: 0.93 k: 0.0607 dk: 0.0148
ECH1 − DAMLNAAFALAADISSK_3
AA R2: 0.98 k: 0.208 dk: 0.0409
HW R2: 0.98 k: 0.136 dk: 0.0184
ECHA − DSIFSNLIGQLDYK_3
AA R2: 0.97 k: 0.0458 dk: 0.00483
HW R2: 0.99 k: 0.0339 dk: 0.0018
FS
Time (days)

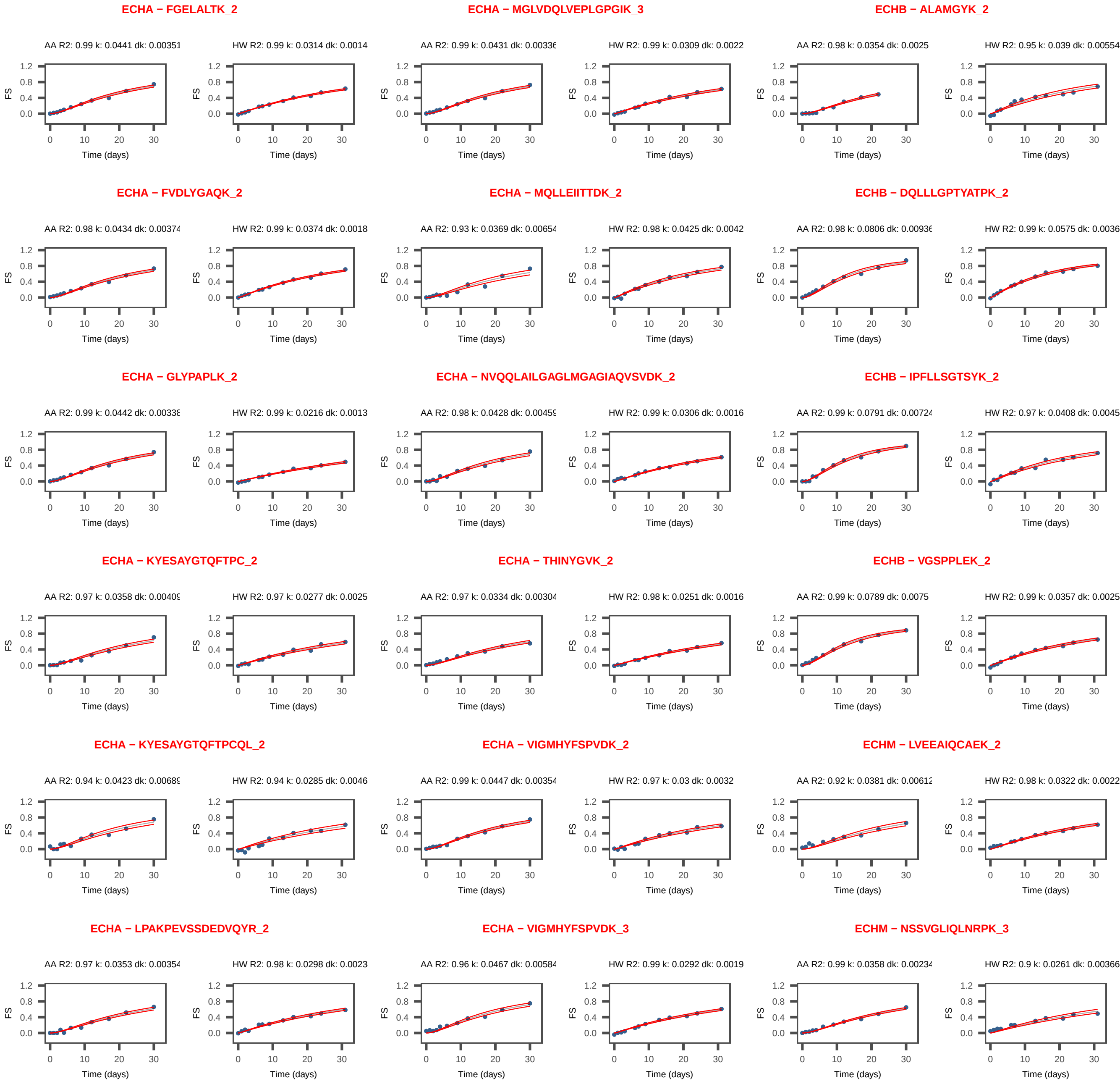
ECHA − FGELALTK_2
AA R2: 0.99 k: 0.0441 dk: 0.00351
HW R2: 0.99 k: 0.0314 dk: 0.0014
ECHA − MGLVDQLVEPLGPGIK_3
AA R2: 0.99 k: 0.0431 dk: 0.0033
HW R2: 0.99 k: 0.0309 dk: 0.0022
ECHB − ALAMGYK_2
AA R2: 0.98 k: 0.0354 dk: 0.0025
HW R2: 0.95 k: 0.039 dk: 0.00554
ECHA − FVDLYGAQK_2
AA R2: 0.98 k: 0.0434 dk: 0.00374
HW R2: 0.99 k: 0.0374 dk: 0.0018
ECHA − MQLLEIITTDK_2
AA R2: 0.93 k: 0.0369 dk: 0.00654
HW R2: 0.98 k: 0.0425 dk: 0.0042
ECHB − DQLLLGPTYATPK_2
AA R2: 0.98 k: 0.0806 dk: 0.0093
HW R2: 0.99 k: 0.0575 dk: 0.0036
ECHA − GLYPAPLK_2
AA R2: 0.99 k: 0.0442 dk: 0.0033
HW R2: 0.99 k: 0.0216 dk: 0.0013
ECHA − NVQQLAILGAGLMGAGIAQVSVDK_2
AA R2: 0.98 k: 0.0428 dk: 0.0045
HW R2: 0.99 k: 0.0306 dk: 0.0016
ECHB − IPFLLSGTSYK_2
AA R2: 0.99 k: 0.0791 dk: 0.00724
HW R2: 0.97 k: 0.0408 dk: 0.0045
ECHA − KYESAYGTQFTPC_2
AA R2: 0.97 k: 0.0358 dk: 0.0040
HW R2: 0.97 k: 0.0277 dk: 0.0025
ECHA − THINYGVK_2
AA R2: 0.97 k: 0.0334 dk: 0.00304
HW R2: 0.98 k: 0.0251 dk: 0.0016
ECHB − VGSPPLEK_2
AA R2: 0.99 k: 0.0789 dk: 0.0075
HW R2: 0.99 k: 0.0357 dk: 0.0025
ECHA − KYESAYGTQFTPCQL_2
AA R2: 0.94 k: 0.0423 dk: 0.0068
HW R2: 0.94 k: 0.0285 dk: 0.0046
ECHA − VIGMHYFSPVDK_2
AA R2: 0.99 k: 0.0447 dk: 0.00354
HW R2: 0.97 k: 0.03 dk: 0.0032
ECHM − LVEEAIQCAEK_2
AA R2: 0.92 k: 0.0381 dk: 0.00612
HW R2: 0.98 k: 0.0322 dk: 0.0022
ECHA − LPAKPEVSSDEDVQYR_2
AA R2: 0.97 k: 0.0353 dk: 0.00354
HW R2: 0.98 k: 0.0298 dk: 0.0023
ECHA − VIGMHYFSPVDK_3
AA R2: 0.96 k: 0.0467 dk: 0.00584
HW R2: 0.99 k: 0.0292 dk: 0.0019
ECHM − NSSVGLIQLNRPK_3
AA R2: 0.99 k: 0.0358 dk: 0.00234
HW R2: 0.9 k: 0.0261 dk: 0.00366
FS
Time (days)

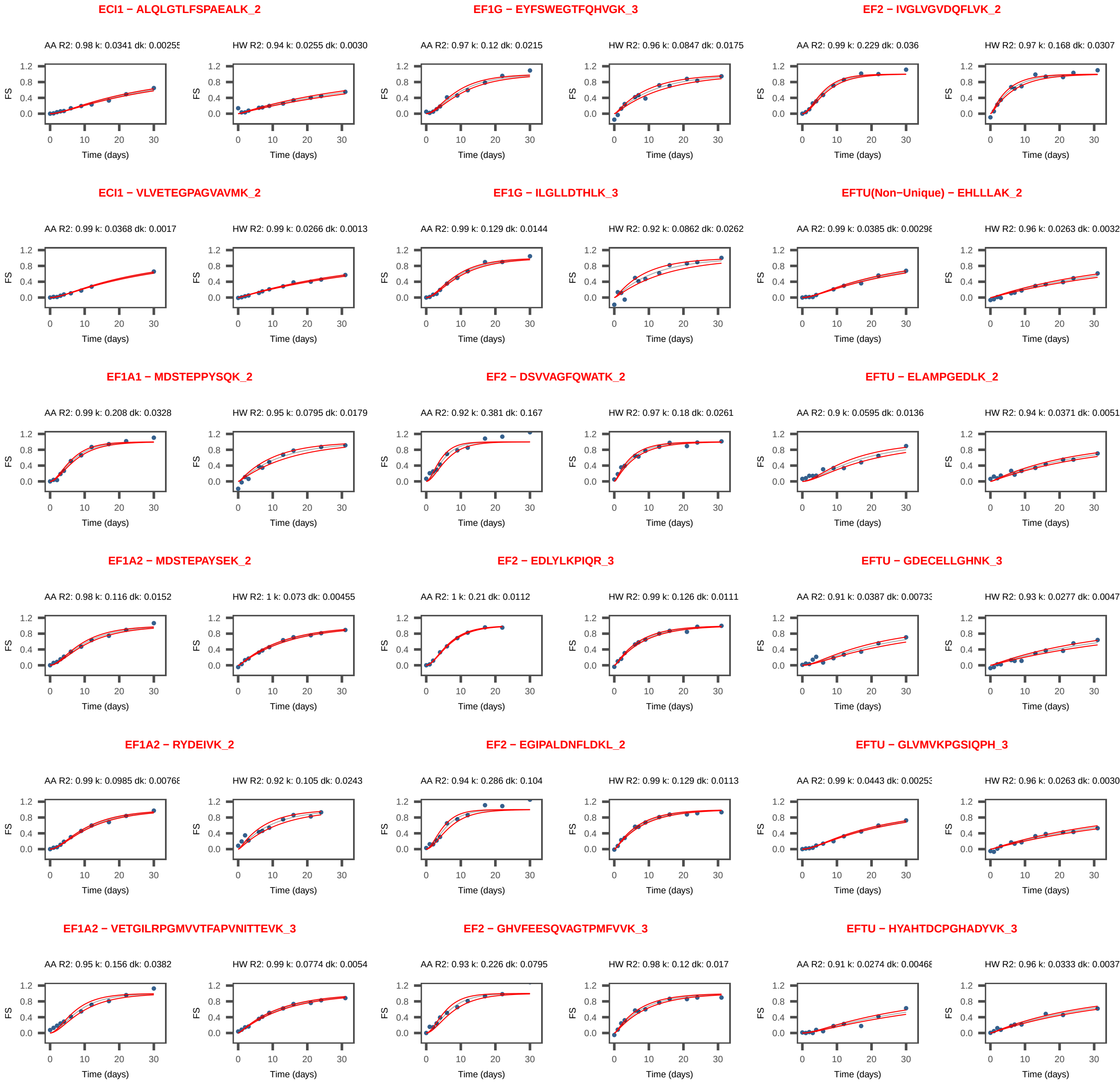
ECI1 − ALQLGTLFSPAEALK_2
AA R2: 0.98 k: 0.0341 dk: 0.00255
HW R2: 0.94 k: 0.0255 dk: 0.0030
EF1G − EYFSWEGTFQHVGK_3
AA R2: 0.97 k: 0.12 dk: 0.0215
HW R2: 0.96 k: 0.0847 dk: 0.0175
EF2 − IVGLVGVDQFLVK_2
AA R2: 0.99 k: 0.229 dk: 0.036
HW R2: 0.97 k: 0.168 dk: 0.0307
ECI1 − VLVETEGPAGVAVMK_2
AA R2: 0.99 k: 0.0368 dk: 0.0017
HW R2: 0.99 k: 0.0266 dk: 0.0013
EF1G − ILGLLDTHLK_3
AA R2: 0.99 k: 0.129 dk: 0.0144
HW R2: 0.92 k: 0.0862 dk: 0.0262
EFTU(Non−Unique) − EHLLLAK_2
AA R2: 0.99 k: 0.0385 dk: 0.00298
HW R2: 0.96 k: 0.0263 dk: 0.0032
EF1A1 − MDSTEPPYSQK_2
AA R2: 0.99 k: 0.208 dk: 0.0328
HW R2: 0.95 k: 0.0795 dk: 0.0179
EF2 − DSVVAGFQWATK_2
AA R2: 0.92 k: 0.381 dk: 0.167
HW R2: 0.97 k: 0.18 dk: 0.0261
EFTU − ELAMPGEDLK_2
AA R2: 0.9 k: 0.0595 dk: 0.0136
HW R2: 0.94 k: 0.0371 dk: 0.0051
EF1A2 − MDSTEPAYSEK_2
AA R2: 0.98 k: 0.116 dk: 0.0152
HW R2: 1 k: 0.073 dk: 0.00455
EF2 − EDLYLKPIQR_3
AA R2: 1 k: 0.21 dk: 0.0112
HW R2: 0.99 k: 0.126 dk: 0.0111
EFTU − GDECELLGHNK_3
AA R2: 0.91 k: 0.0387 dk: 0.00733
HW R2: 0.93 k: 0.0277 dk: 0.0047
EF1A2 − RYDEIVK_2
AA R2: 0.99 k: 0.0985 dk: 0.00768
HW R2: 0.92 k: 0.105 dk: 0.0243
EF2 − EGIPALDNFLDKL_2
AA R2: 0.94 k: 0.286 dk: 0.104
HW R2: 0.99 k: 0.129 dk: 0.0113
EFTU − GLVMVKPGSIQPH_3
AA R2: 0.99 k: 0.0443 dk: 0.00253
HW R2: 0.96 k: 0.0263 dk: 0.0030
EF1A2 − VETGILRPGMVVTFAPVNITTEVK_3
AA R2: 0.95 k: 0.156 dk: 0.0382
HW R2: 0.99 k: 0.0774 dk: 0.0054
EF2 − GHVFEESQVAGTPMFVVK_3
AA R2: 0.93 k: 0.226 dk: 0.0795
HW R2: 0.98 k: 0.12 dk: 0.017
EFTU − HYAHTDCPGHADYVK_3
AA R2: 0.91 k: 0.0274 dk: 0.00468
HW R2: 0.96 k: 0.0333 dk: 0.0037
FS
Time (days)

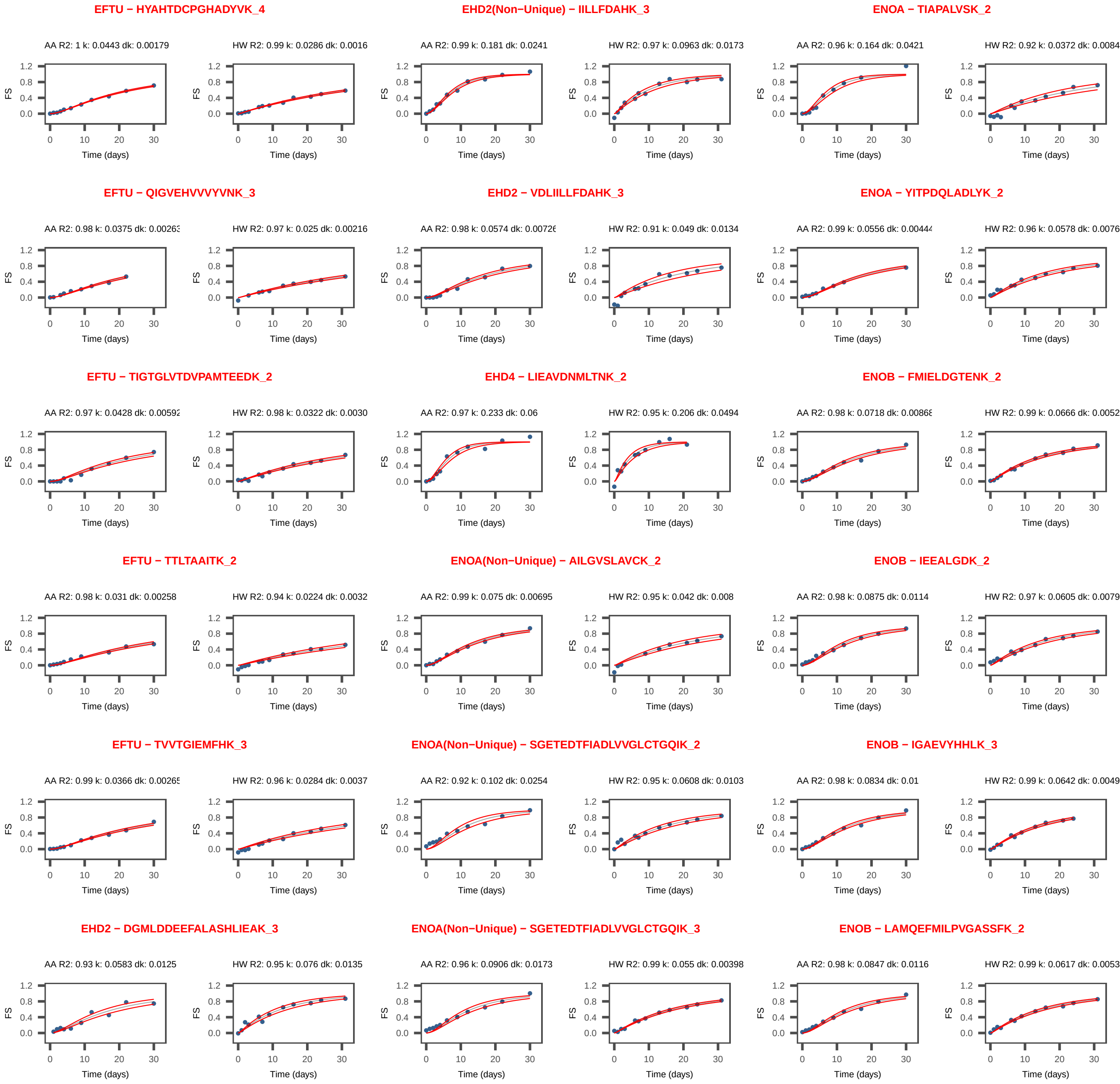
EFTU – HYAHTDCPGHADYVK_4
AA R2: 1 k: 0.0443 dk: 0.00179
HW R2: 0.99 k: 0.0286 dk: 0.0016
EHD2(Non–Unique) – IILLFDAHK_3
AA R2: 0.99 k: 0.181 dk: 0.0241
HW R2: 0.97 k: 0.0963 dk: 0.0173
ENOA – TIAPALVSK_2
AA R2: 0.96 k: 0.164 dk: 0.0421
HW R2: 0.92 k: 0.0372 dk: 0.0084
EFTU – QIGVEHVVVYVNK_3
AA R2: 0.98 k: 0.0375 dk: 0.00263
HW R2: 0.97 k: 0.025 dk: 0.00216
EHD2 – VDLIILLFDAHK_3
AA R2: 0.98 k: 0.0574 dk: 0.00726
HW R2: 0.91 k: 0.049 dk: 0.0134
ENOA – YITPDQLADLYK_2
AA R2: 0.99 k: 0.0556 dk: 0.00444
HW R2: 0.96 k: 0.0578 dk: 0.0076
EFTU – TIGTGLVTDVPAMTEEDK_2
AA R2: 0.97 k: 0.0428 dk: 0.00592
HW R2: 0.98 k: 0.0322 dk: 0.0030
EHD4 – LIEAVDNMLTNK_2
AA R2: 0.97 k: 0.233 dk: 0.06
HW R2: 0.95 k: 0.206 dk: 0.0494
ENOB – FMIELDGTENK_2
AA R2: 0.98 k: 0.0718 dk: 0.00868
HW R2: 0.99 k: 0.0666 dk: 0.0052
EFTU – TTLTAAITK_2
AA R2: 0.98 k: 0.031 dk: 0.00258
HW R2: 0.94 k: 0.0224 dk: 0.0032
ENOA(Non–Unique) – AILGVSLAVCK_2
AA R2: 0.99 k: 0.075 dk: 0.00695
HW R2: 0.95 k: 0.042 dk: 0.008
ENOB – IEEALGDK_2
AA R2: 0.98 k: 0.0875 dk: 0.0114
HW R2: 0.97 k: 0.0605 dk: 0.0079
EFTU – TVVTGIEMFHK_3
AA R2: 0.99 k: 0.0366 dk: 0.00265
HW R2: 0.96 k: 0.0284 dk: 0.0037
ENOA(Non–Unique) – SGETEDTFIADLVVGLCTGQIK_2
AA R2: 0.92 k: 0.102 dk: 0.0254
HW R2: 0.95 k: 0.0608 dk: 0.0103
ENOB – IGAEVYHHLK_3
AA R2: 0.98 k: 0.0834 dk: 0.01
HW R2: 0.99 k: 0.0642 dk: 0.0049
EHD2 – DGMLDDEEFALASHLIEAK_3
AA R2: 0.93 k: 0.0583 dk: 0.0125
HW R2: 0.95 k: 0.076 dk: 0.0135
ENOA(Non–Unique) – SGETEDTFIADLVVGLCTGQIK_3
AA R2: 0.96 k: 0.0906 dk: 0.0173
HW R2: 0.99 k: 0.055 dk: 0.00398
ENOB – LAMQEFMILPVGASSFK_2
AA R2: 0.98 k: 0.0847 dk: 0.0116
HW R2: 0.99 k: 0.0617 dk: 0.0053
FS
Time (days)

ENOB − LSGVDIQIVGDDLTVTNPK_2

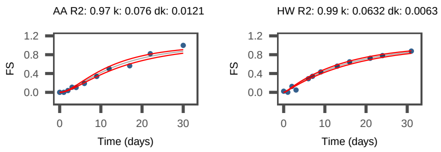

ETFA − DPEAPIFQVADYGIVADLFK_3

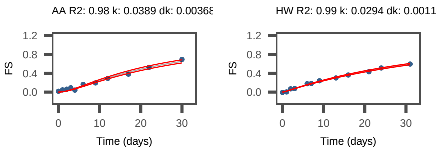

ETFA − SDRPELTGAK_2

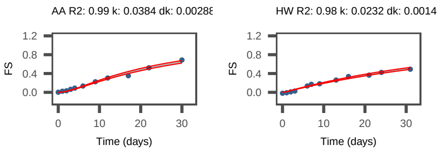

ENOB − TAIQAAGYPDK_2

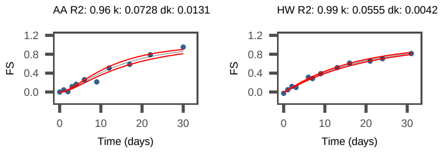

ETFA − GLLPEELTPLILETQK_3

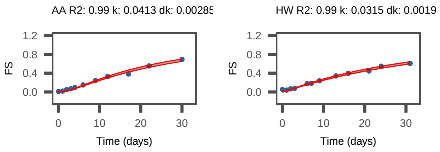

ETFA − SDRPELTGAK_3

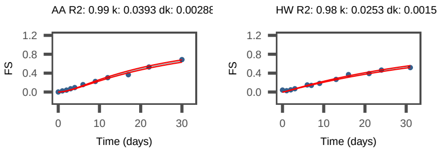

ENOB − TLGPALLEK_2

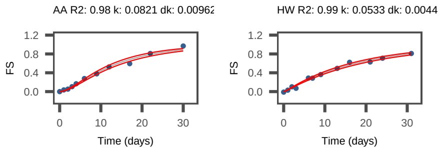

ETFA − GTSFEAAATSGGSASSEK_2

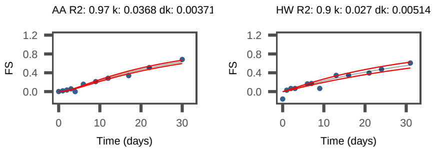

ETFA − TIVAINK_2

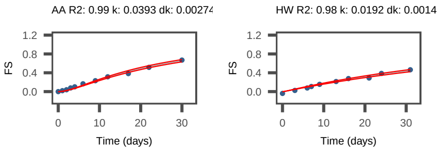

ENPL − EFEPLLNWMK_2

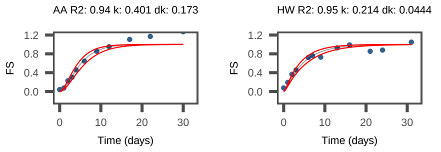

ETFA − GTSFEAAATSGGSASSEK_3

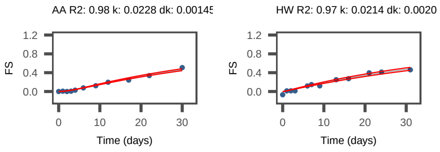

ETFA − VLVAQHDAYK_2

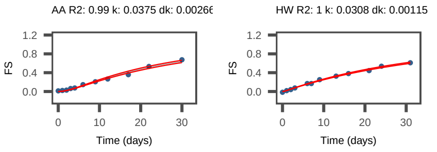

EST1D − AVIGDHGDEIFSVFGSPFLK_3

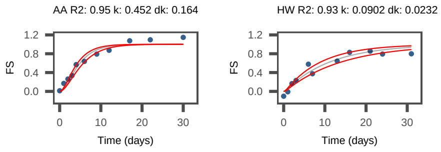

ETFA − IAVGISGAIQHLAGMK_3

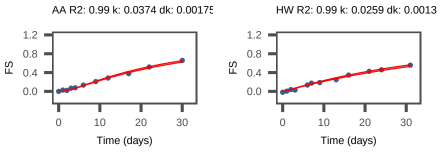

ETFA − VLVAQHDAYK_3

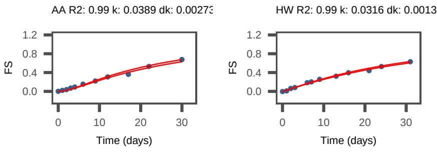

EST1D − ESYPFLPTVIDGVVLPK_2

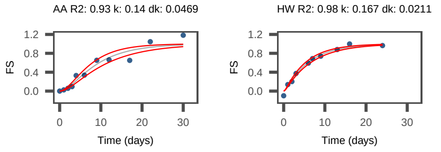

ETFA − LNVAPVSDIIEIK_2

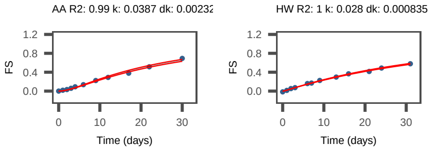

ETFA − VVPEMTEILK_2

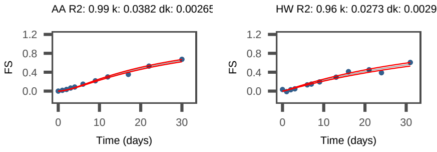

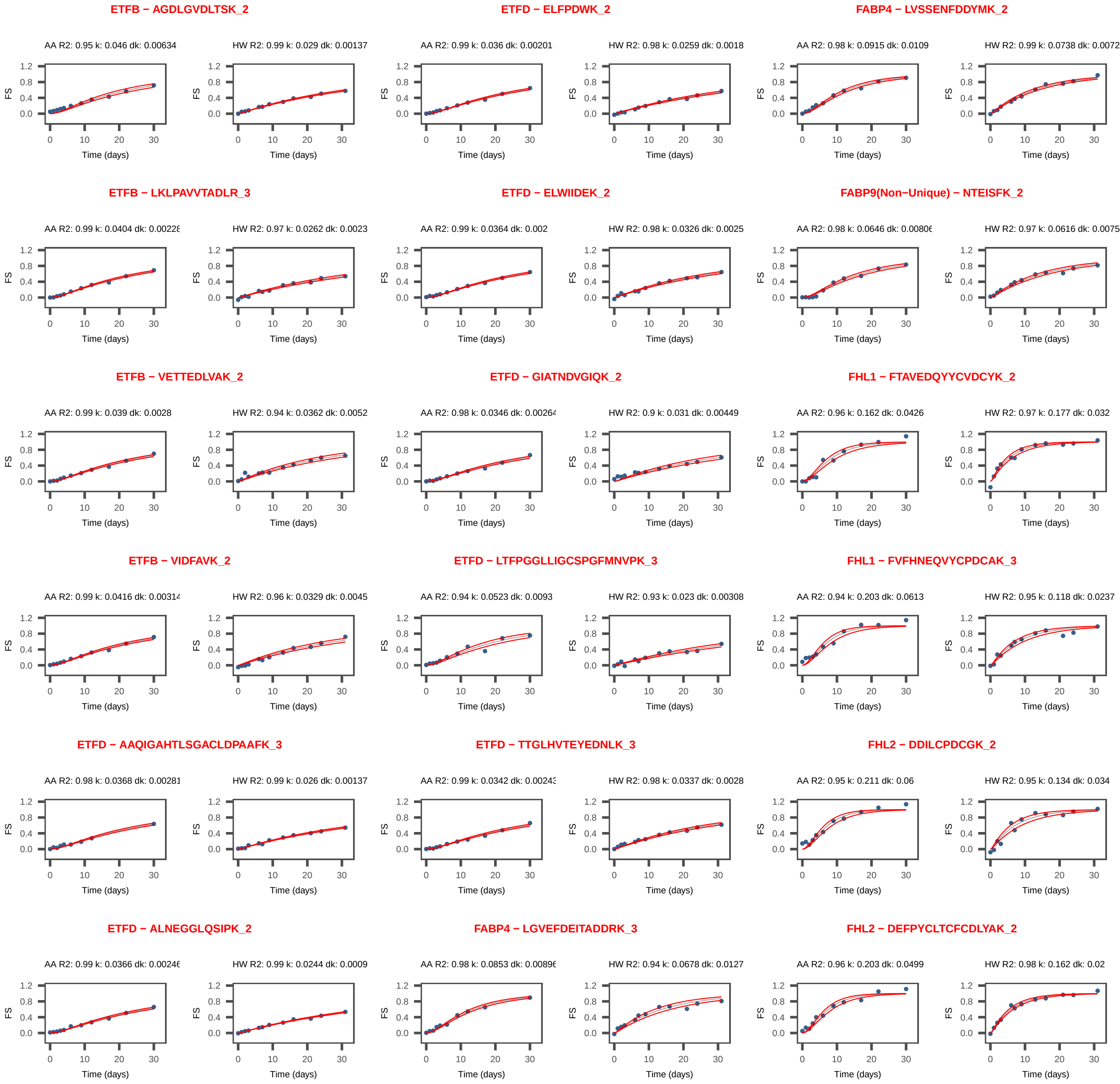
ETFB − AGDLGVDLTSK_2
AA R2: 0.95 k: 0.046 dk: 0.00634
HW R2: 0.99 k: 0.029 dk: 0.00137
ETFD − ELFPDWK_2
AA R2: 0.99 k: 0.036 dk: 0.00201
HW R2: 0.98 k: 0.0259 dk: 0.0018
FABP4 − LVSSENFDDYMK_2
AA R2: 0.98 k: 0.0915 dk: 0.0109
HW R2: 0.99 k: 0.0738 dk: 0.0072
ETFB − LKLPAVVTADLR_3
AA R2: 0.99 k: 0.0404 dk: 0.0022
HW R2: 0.97 k: 0.0262 dk: 0.0023
ETFD − ELWIIDEK_2
AA R2: 0.99 k: 0.0364 dk: 0.002
HW R2: 0.98 k: 0.0326 dk: 0.0025
FABP9(Non−Unique) − NTEISFK_2
AA R2: 0.98 k: 0.0646 dk: 0.0080
HW R2: 0.97 k: 0.0616 dk: 0.0075
ETFB − VETTEDLVAK_2
AA R2: 0.99 k: 0.039 dk: 0.0028
HW R2: 0.94 k: 0.0362 dk: 0.0052
ETFD − GIATNDVGIQK_2
AA R2: 0.98 k: 0.0346 dk: 0.0026
HW R2: 0.9 k: 0.031 dk: 0.00449
FHL1 − FTAVEDQYYCVDCYK_2
AA R2: 0.96 k: 0.162 dk: 0.0426
HW R2: 0.97 k: 0.177 dk: 0.032
ETFB − VIDFAVK_2
AA R2: 0.99 k: 0.0416 dk: 0.0031
HW R2: 0.96 k: 0.0329 dk: 0.0045
ETFD − LTFPGGLLIGCSPGFMNVPK_3
AA R2: 0.94 k: 0.0523 dk: 0.0093
HW R2: 0.93 k: 0.023 dk: 0.00308
FHL1 − FVFHNEQVYCPDCAK_3
AA R2: 0.94 k: 0.203 dk: 0.0613
HW R2: 0.95 k: 0.118 dk: 0.0237
ETFD − AAQIGAHTLSGACLDPAAFK_3
AA R2: 0.98 k: 0.0368 dk: 0.0028
HW R2: 0.99 k: 0.026 dk: 0.00137
ETFD − TTGLHVTEYEDNLK_3
AA R2: 0.99 k: 0.0342 dk: 0.0024
HW R2: 0.98 k: 0.0337 dk: 0.0028
FHL2 − DDILCPDCGK_2
AA R2: 0.95 k: 0.211 dk: 0.06
HW R2: 0.95 k: 0.134 dk: 0.034
ETFD − ALNEGGLQSIPK_2
AA R2: 0.99 k: 0.0366 dk: 0.0024
HW R2: 0.99 k: 0.0244 dk: 0.0009
FABP4 − LGVEFDEITADDRK_3
AA R2: 0.98 k: 0.0853 dk: 0.0089
HW R2: 0.94 k: 0.0678 dk: 0.0127
FHL2 − DEFPYCLTCFCDLYAK_2
AA R2: 0.96 k: 0.203 dk: 0.0499
HW R2: 0.98 k: 0.162 dk: 0.02
FS
Time (days)

FHL2 – ECFVCTACK_2

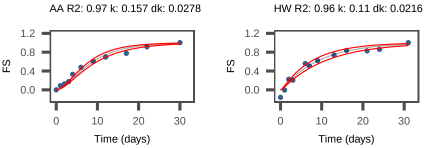

FIBA – EINLQDYEGHQK_3

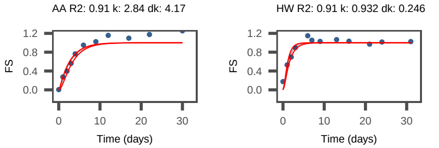

FUMH – AAAEVNQEYGLDPK_2

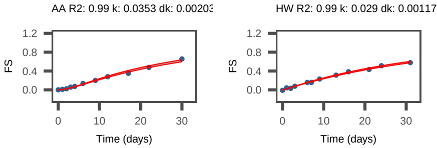

FHL2 – EEQLLCTDCYSNEYSSK_2

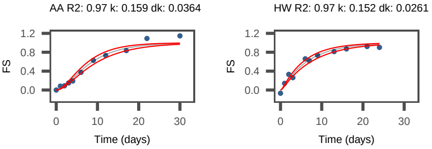

FIS1 – GIVLLEELLPK_2

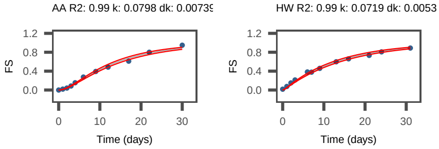

FUMH – HIAAAVEVHK_3

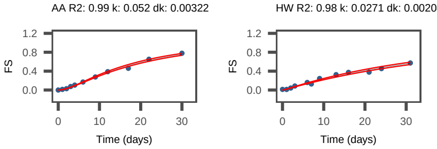

FHL2 – ENQNFCVPCYEK_2

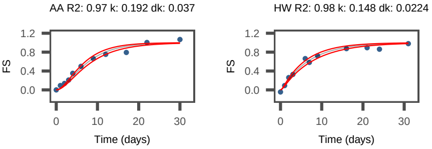

FLNB(Non–Unique) – LIALLEVLSQK_2

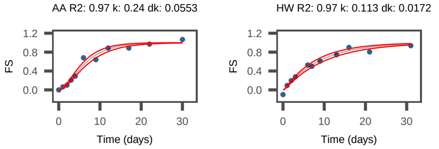

FUMH – MPIPVIQAFGILK_2

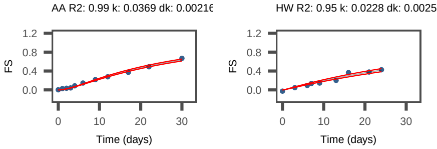

FHL2 – FDCHHCNESLYGK_3

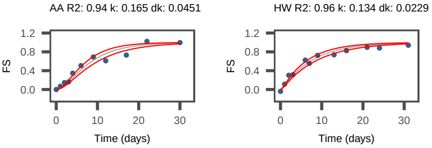

FLNC – FVPQEMGPHTVTVK_3

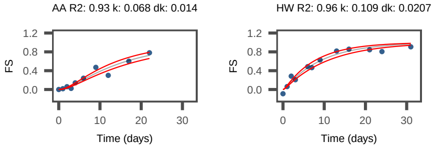

FUMH – SGLGELILPENEPGSSIMPGK_2

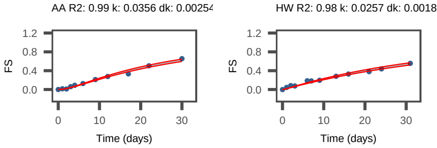

FHL2 – QWHNDCFNCK_2

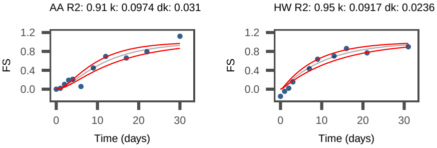

FMO1 – NLLPTPIVSWLISK_2

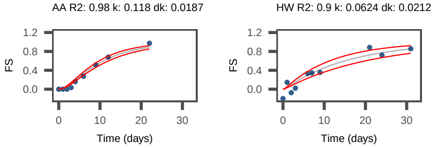

FUMH – SQSSNDTFPTAMHIAAAVEVHK_4

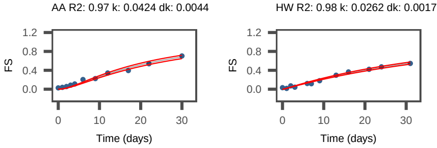

FHL2 – QYALQCVQCK_2

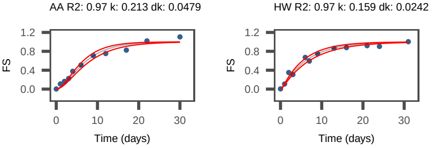

FRIH – IFLQDIK_2

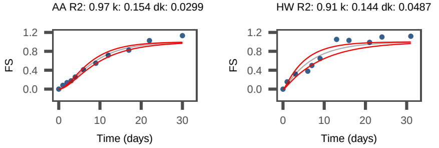

FUMH – VAALTGLPFVTAPNK_2

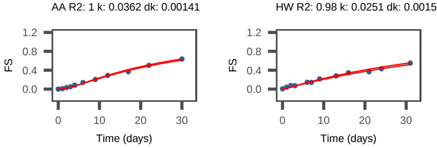

FUMH − VEFDTFGELK_2

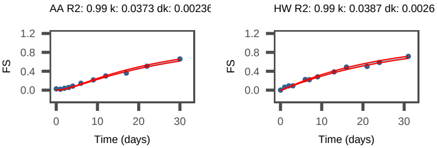

G3P − VIISAPSADAPMFVMGVNHEK_2

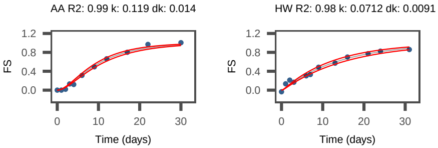

G6PI − HFVALSTNTAK_2

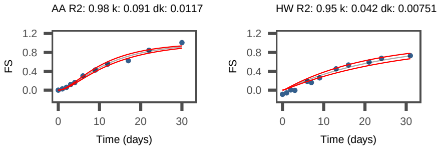

FUMH − VLLPGLQK_2

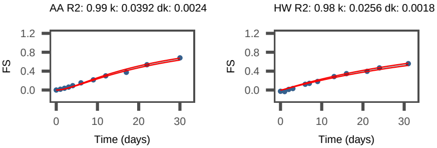

G3P − VIISAPSADAPMFVMGVNHEK_3

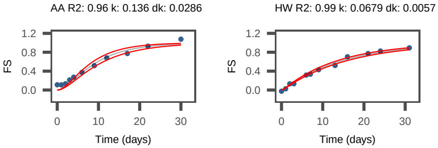

G6PI − ILGALIAMYEHK_3

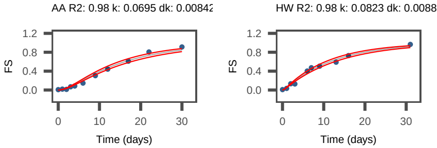

G3P(Non−Unique) − CLAPLAK_2

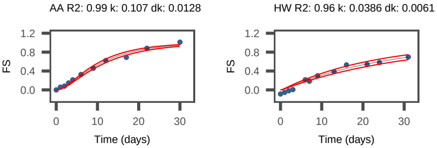

G3P − VIPELNGK_2

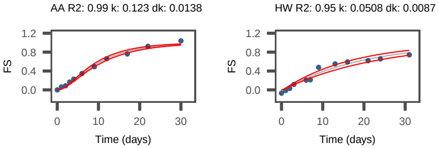

G6PI − ILLANFLAQTEALMK_2

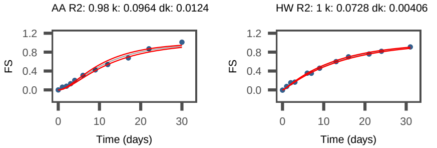

G3P − MFQYDSTHGK_2

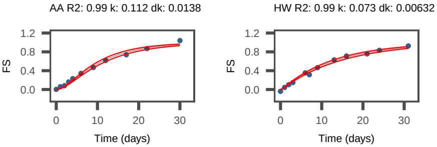

G3P − VVDLMAYMASK_2

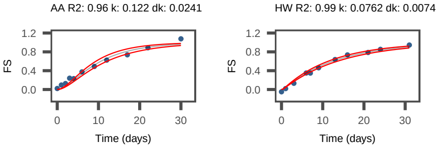

G6PI − ILLANFLAQTEALMK_3

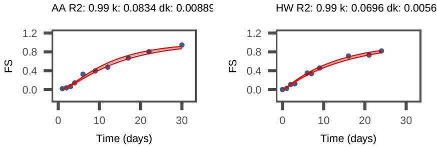

G3P − MFQYDSTHGK_3

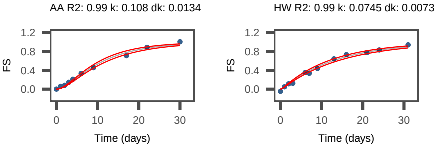

G6PI − EWFLEAAK_2

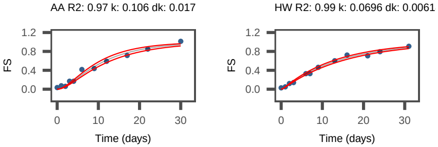

G6PI − SITDIINIGIGGSDLGPLMVTEALKPY_3

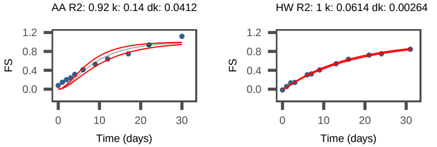

G3P − VIHDNFGIVEGLMTTVHAITATQK_3

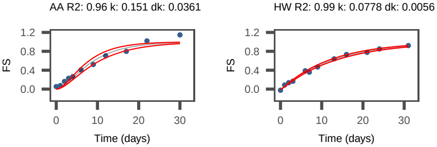

G6PI − FAAYFQQGDMESNGK_2

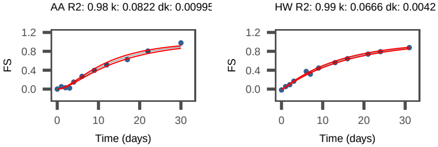

G6PI − TFTTQETITNAETAK_2

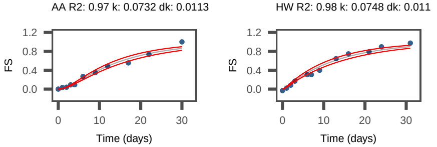

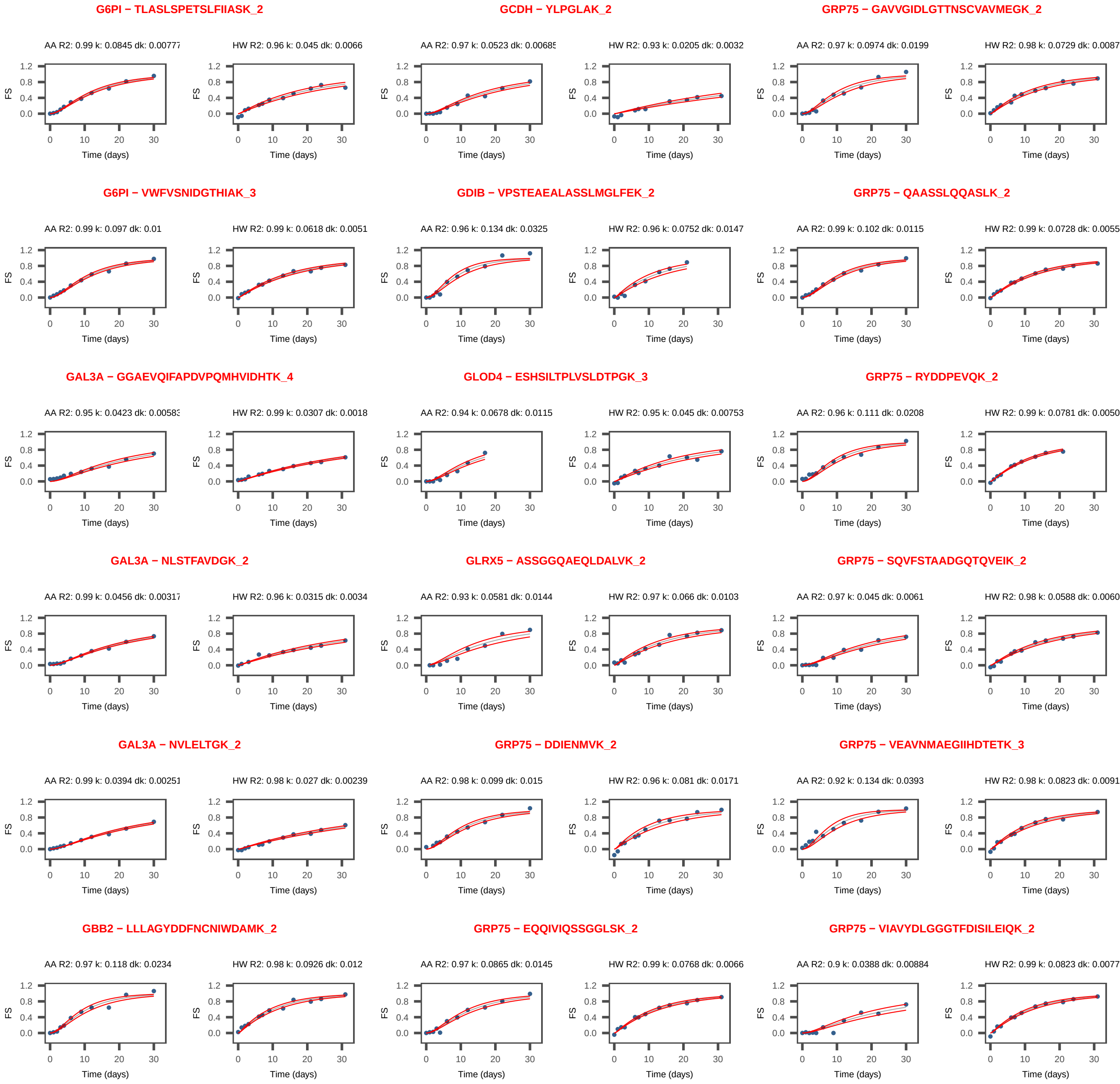
G6PI – TLASLSPETSLFIIASK_2
AA R2: 0.99 k: 0.0845 dk: 0.00777
HW R2: 0.96 k: 0.045 dk: 0.0066
GCDH – YLPGLAK_2
AA R2: 0.97 k: 0.0523 dk: 0.00685
HW R2: 0.93 k: 0.0205 dk: 0.0032
GRP75 – GAVVGIDLGTTNSCVAVMEGK_2
AA R2: 0.97 k: 0.0974 dk: 0.0199
HW R2: 0.98 k: 0.0729 dk: 0.0087
G6PI – VWFVSNIDGTHIAK_3
AA R2: 0.99 k: 0.097 dk: 0.01
HW R2: 0.99 k: 0.0618 dk: 0.0051
GDIB – VPSTEAEALASSLMGLFEK_2
AA R2: 0.96 k: 0.134 dk: 0.0325
HW R2: 0.96 k: 0.0752 dk: 0.0147
GRP75 – QAASSLQQASLK_2
AA R2: 0.99 k: 0.102 dk: 0.0115
HW R2: 0.99 k: 0.0728 dk: 0.0055
GAL3A – GGAEVQIFAPDVPQMHVIDHTK_4
AA R2: 0.95 k: 0.0423 dk: 0.00583
HW R2: 0.99 k: 0.0307 dk: 0.0018
GLOD4 – ESHSILTPLVSLDTPGK_3
AA R2: 0.94 k: 0.0678 dk: 0.0115
HW R2: 0.95 k: 0.045 dk: 0.00753
GRP75 – RYDDPEVQK_2
AA R2: 0.96 k: 0.111 dk: 0.0208
HW R2: 0.99 k: 0.0781 dk: 0.0050
GAL3A – NLSTFAVDGK_2
AA R2: 0.99 k: 0.0456 dk: 0.00317
HW R2: 0.96 k: 0.0315 dk: 0.0034
GLRX5 – ASSGGQAEQLDALVK_2
AA R2: 0.93 k: 0.0581 dk: 0.0144
HW R2: 0.97 k: 0.066 dk: 0.0103
GRP75 – SQVFSTAADGQTQVEIK_2
AA R2: 0.97 k: 0.045 dk: 0.0061
HW R2: 0.98 k: 0.0588 dk: 0.0060
GAL3A – NVLELTGK_2
AA R2: 0.99 k: 0.0394 dk: 0.00251
HW R2: 0.98 k: 0.027 dk: 0.00239
GRP75 – DDIENMVK_2
AA R2: 0.98 k: 0.099 dk: 0.015
HW R2: 0.96 k: 0.081 dk: 0.0171
GRP75 – VEAVNMAEGIIHDTETK_3
AA R2: 0.92 k: 0.134 dk: 0.0393
HW R2: 0.98 k: 0.0823 dk: 0.0091
GBB2 – LLLAGYDDFNCNIWDAMK_2
AA R2: 0.97 k: 0.118 dk: 0.0234
HW R2: 0.98 k: 0.0926 dk: 0.012
GRP75 – EQQIVIQSSGGLSK_2
AA R2: 0.97 k: 0.0865 dk: 0.0145
HW R2: 0.99 k: 0.0768 dk: 0.0066
GRP75 – VIAVYDLGGGTFDISILEIQK_2
AA R2: 0.9 k: 0.0388 dk: 0.00884
HW R2: 0.99 k: 0.0823 dk: 0.0077
FS
Time (days)

GRP75 − VINEPTAAALAYGLDK_2

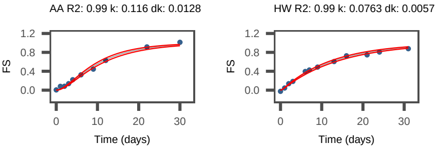

GSTO1(Non−Unique) − HEVININLK_2

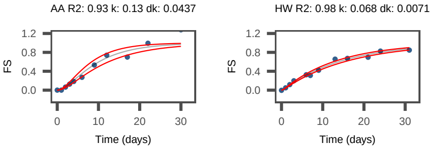

H2A1B(Non−Unique) − VTIAQGGVLPNIQAVLLPK_3

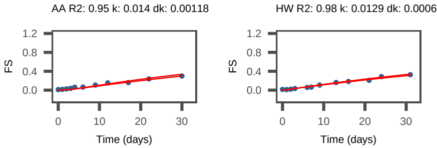

GSTA4 − KPPPDGPYVEVVR_3

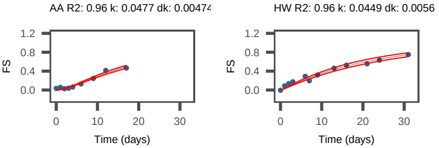

GSTO1 − LWMAAMQQDPVASSHK_3

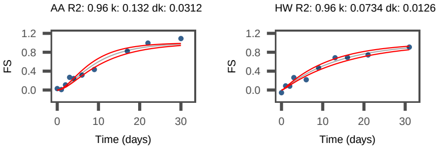

H2B1F(Non−Unique) − ESYSVYVYK_2

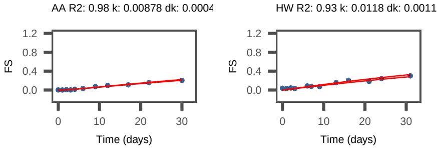

GSTK1 − LELLAYLLGEK_2

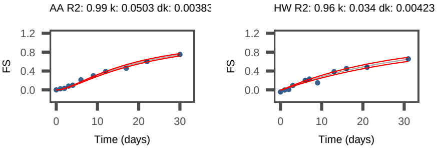

GSTP1(Non−Unique) − EEVVTIDTWMQGLLK_2

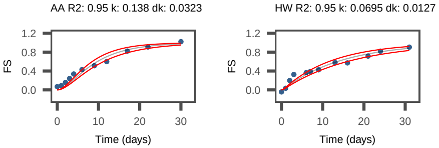

H2B1F(Non−Unique) − QVHPDTGISSK_3

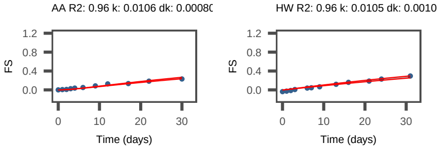

GSTM1(Non−Unique) − LGLDFPNLPYLIDGSHK_3

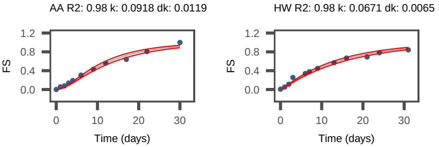

GSTP1_HUMAN,sp|P19157|GSTP1(Non−Unique) − MLLADQGQSWK_2

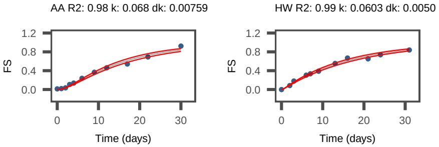

HBA − AAGHLDDLPGALSALSDLHAHK_4

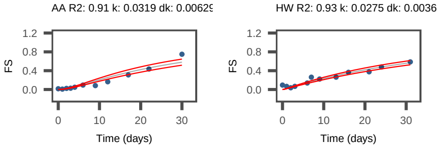

GSTM1 − MLLEYTDSSYDEK_2

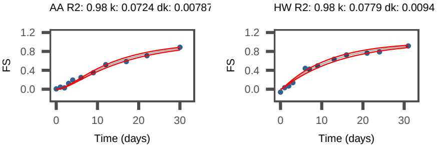

H12(Non−Unique) − ALAAAGYDVEK_2

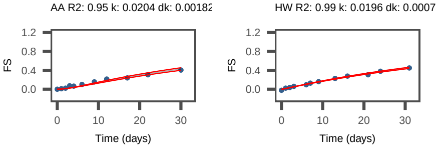

HBA − AAGHLDDLPGALSALSDLHAHK_5

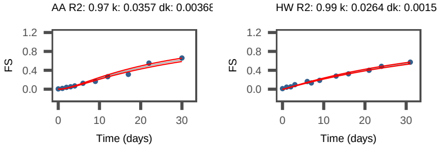

GSTM1 − MQLIMLCYNPDFEK_2

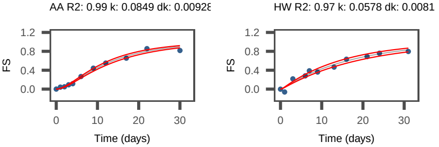

H12(Non−Unique) − ASGPPVSELITK_2

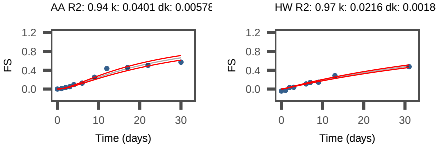

HBA − CLLVTLASHHPADFTPAVHASLDK_5

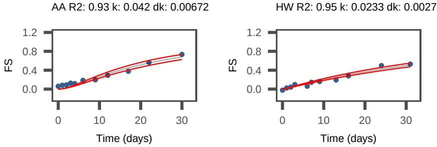

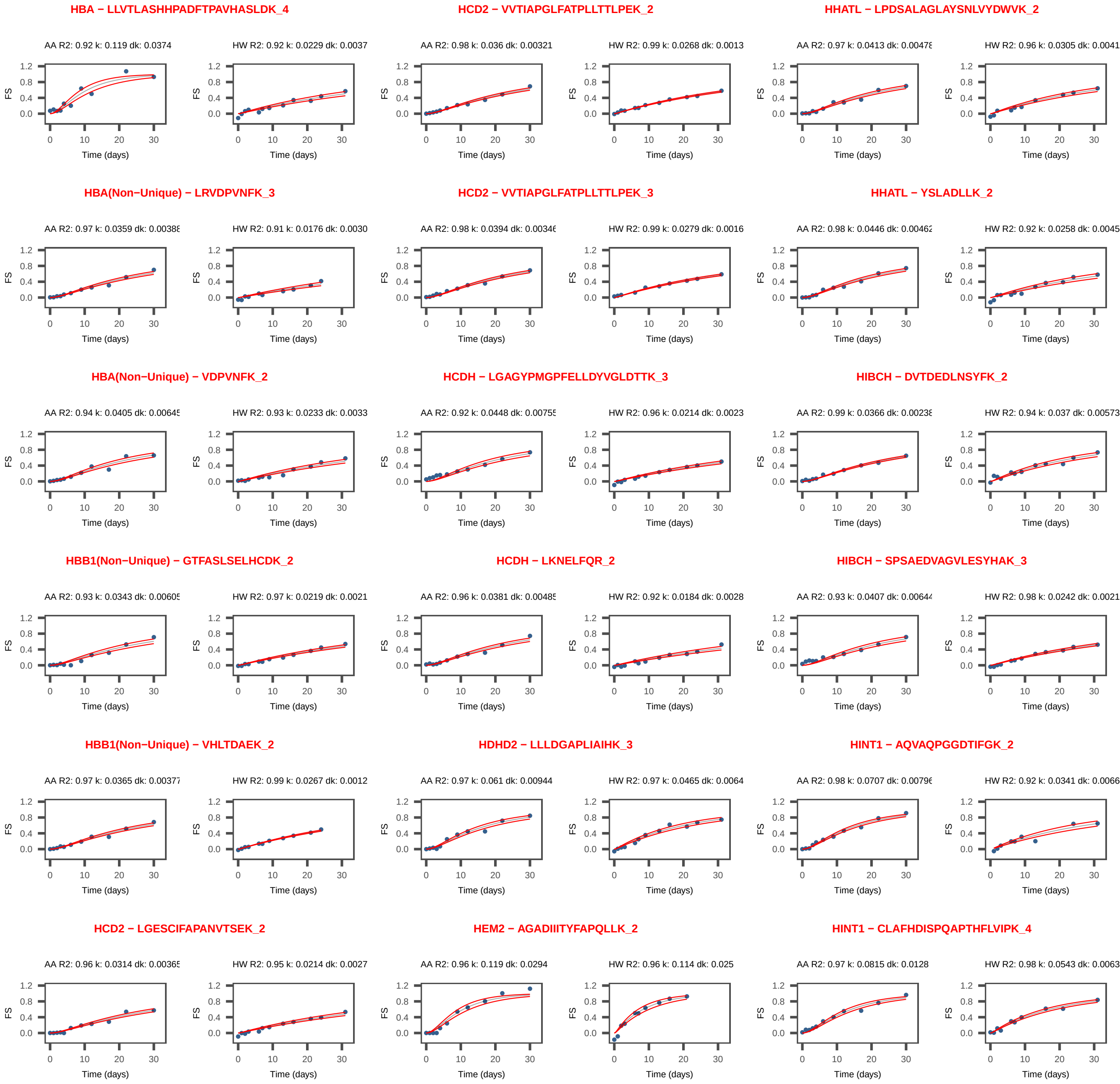
HBA – LLVTLASHHPADFTPAVHASLDK_4
AA R2: 0.92 k: 0.119 dk: 0.0374
HW R2: 0.92 k: 0.0229 dk: 0.0037
HCD2 – VVTIAPGLFATPLLTTLPEK_2
AA R2: 0.98 k: 0.036 dk: 0.00321
HW R2: 0.99 k: 0.0268 dk: 0.0013
HHATL – LPDSALAGLAYSNLVYDWVK_2
AA R2: 0.97 k: 0.0413 dk: 0.0047
HW R2: 0.96 k: 0.0305 dk: 0.0041
HBA(Non–Unique) – LRVDPVNFK_3
AA R2: 0.97 k: 0.0359 dk: 0.0038
HW R2: 0.91 k: 0.0176 dk: 0.0030
HCD2 – VVTIAPGLFATPLLTTLPEK_3
AA R2: 0.98 k: 0.0394 dk: 0.0034
HW R2: 0.99 k: 0.0279 dk: 0.0016
HHATL – YSLADLLK_2
AA R2: 0.98 k: 0.0446 dk: 0.0046
HW R2: 0.92 k: 0.0258 dk: 0.0045
HBA(Non–Unique) – VDPVNFK_2
AA R2: 0.94 k: 0.0405 dk: 0.0064
HW R2: 0.93 k: 0.0233 dk: 0.0033
HCDH – LGAGYPMGPFELLDYVGLDTTK_3
AA R2: 0.92 k: 0.0448 dk: 0.0075
HW R2: 0.96 k: 0.0214 dk: 0.0023
HIBCH – DVTDEDLNSYFK_2
AA R2: 0.99 k: 0.0366 dk: 0.0023
HW R2: 0.94 k: 0.037 dk: 0.00573
HBB1(Non–Unique) – GTFASLSELHCDK_2
AA R2: 0.93 k: 0.0343 dk: 0.0060
HW R2: 0.97 k: 0.0219 dk: 0.0021
HCDH – LKNELFQR_2
AA R2: 0.96 k: 0.0381 dk: 0.0048
HW R2: 0.92 k: 0.0184 dk: 0.0028
HIBCH – SPSAEDVAGVLESYHAK_3
AA R2: 0.93 k: 0.0407 dk: 0.0064
HW R2: 0.98 k: 0.0242 dk: 0.0021
HBB1(Non–Unique) – VHLTDAEK_2
AA R2: 0.97 k: 0.0365 dk: 0.0037
HW R2: 0.99 k: 0.0267 dk: 0.0012
HDHD2 – LLLDGAPLIAIHK_3
AA R2: 0.97 k: 0.061 dk: 0.00944
HW R2: 0.97 k: 0.0465 dk: 0.0064
HINT1 – AQVAQPGGDTIFGK_2
AA R2: 0.98 k: 0.0707 dk: 0.0079
HW R2: 0.92 k: 0.0341 dk: 0.0066
HCD2 – LGESCIFAPANVTSEK_2
AA R2: 0.96 k: 0.0314 dk: 0.0036
HW R2: 0.95 k: 0.0214 dk: 0.0027
HEM2 – AGADIIITYFAPQLLK_2
AA R2: 0.96 k: 0.119 dk: 0.0294
HW R2: 0.96 k: 0.114 dk: 0.025
HINT1 – CLAFHDISPQAPTHFLVIPK_4
AA R2: 0.97 k: 0.0815 dk: 0.0128
HW R2: 0.98 k: 0.0543 dk: 0.0063
FS
Time (days)

HINT2 − ISQAEEDDQQLLGHLLLVAK_3

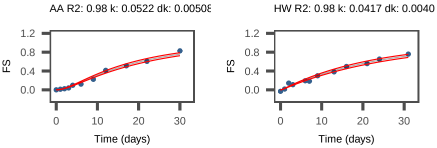

HS90A(Non−Unique) − YIDQEELNK_2

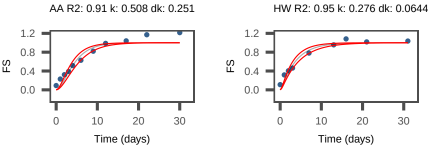

HSP72(Non−Unique) − LLQDFFNGK_2

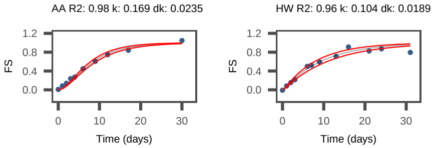

HNRPK − GSYGDLGGPIITTQVTIPK_2

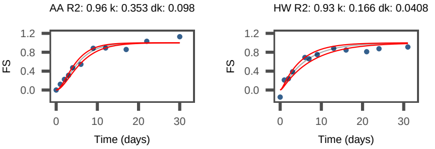

HS90B − DNSTMGYMMAK_2

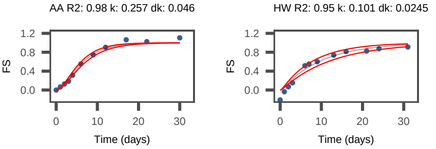

HSP74 − SNLAYDIVQLPTGLTGIK_2

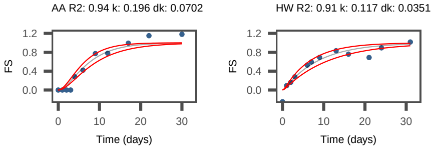

HNRPK − IITITGTQDQIQNAQYLLQNSVK_3

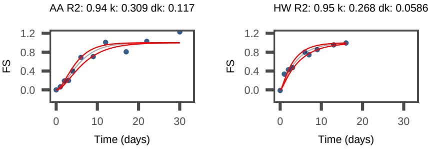

HS90B − GFEVVYMTEPIDEYCVQQLK_2

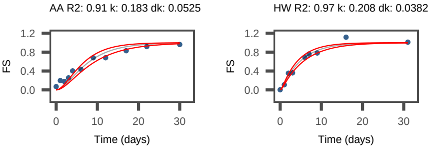

HSP7C − NQVAMNPTNTVFDAK_2

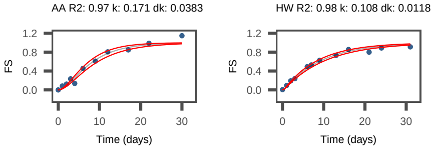

HS71L(Non−Unique) − IINEPTAAAIAYGLDK_2

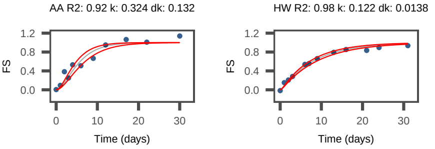

HSDL2 − DEQQINSAVEK_2

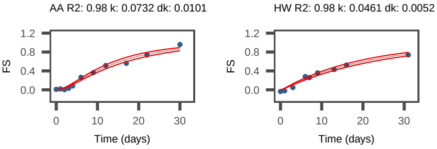

HSP7C − RFDDAVVQSDMK_3

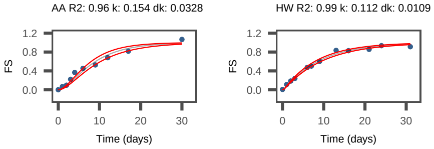

HS71L(Non−Unique) − IINEPTAAAIAYGLDK_3

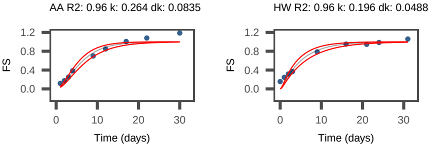

HSDL2 − LQLQEESQLQK_2

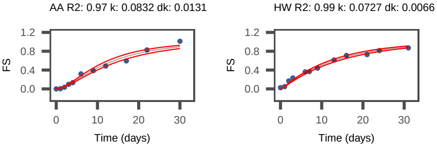

HSP7C − SINPDEAVAYGAAVQAAILSGDK_2

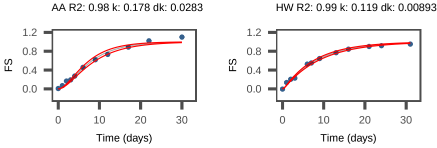

HS90A(Non−Unique) − ADLINNLGTIAK_2

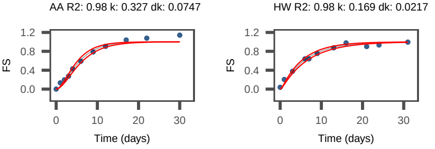

HSDL2 − VQSMLPQKPHFGAVEETFR_4

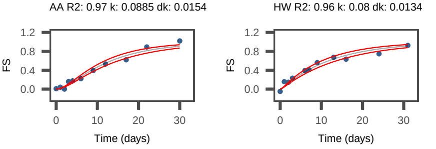

HSP7C − SINPDEAVAYGAAVQAAILSGDK_3

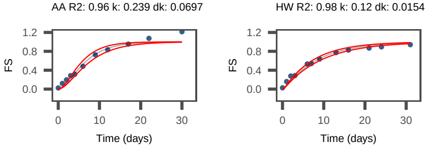

HSPB6 − APSVALPTAQVSTDSGYFSVLLDVK_2

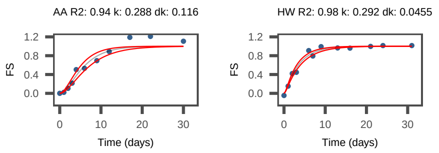

IDH3A − AGGVQTVTLIPGDGIGPEISASVMK_2

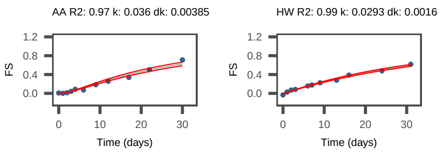

IDHP − GKLDGNQDLIR_2

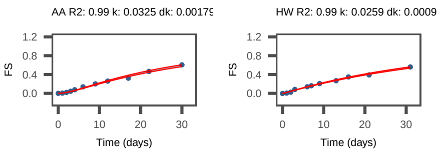

HXK2(Non−Unique) − NILIDFTK_2

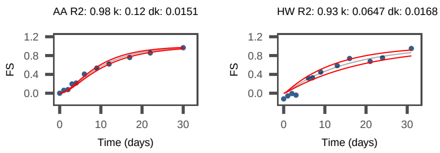

IDH3A − AGGVQTVTLIPGDGIGPEISASVMK_3

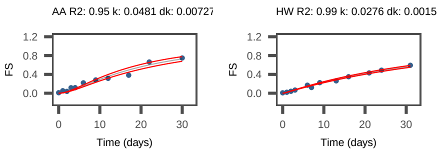

IDHP − LILPHVDVQLK_2

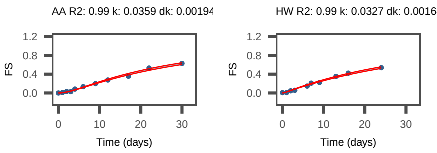

HYES − DFLLGAYQTEFPEGPTEQLMK_2

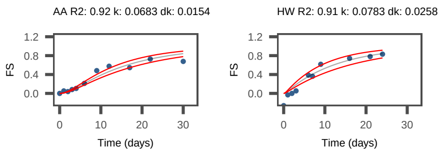

IDH3A − LITEEASKR_2

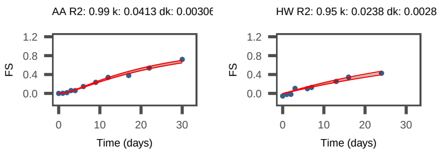

IDHP − LVPGWTKPITIGR_3

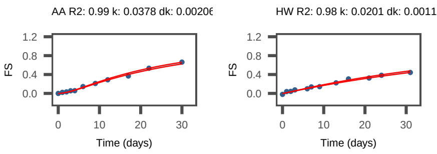

HYES − ILVPALMVTAEK_2

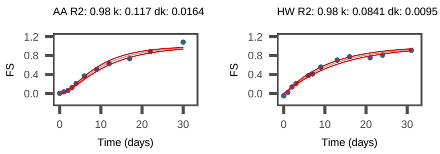

IDHP − DIFQEIFDK_2

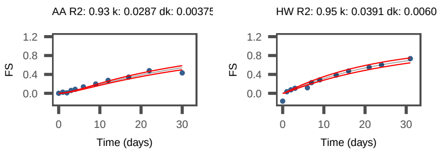

IDHP − SSGGFVWACK_2

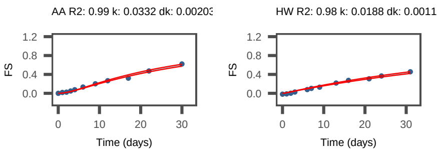

HYES − ITTEEEIEFYIQQFK_3

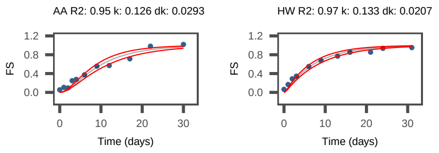

IDHP − DLAGCIHGLSNVK_2

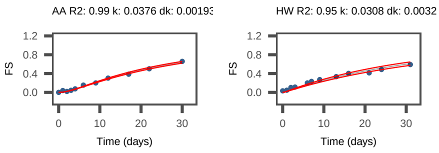

IDHP − VEKPVVEMDGDEMTR_2

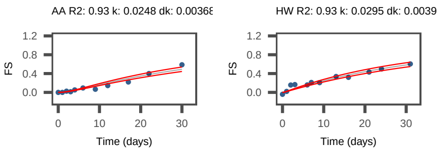

HYES − IYNFLLDTLK_2

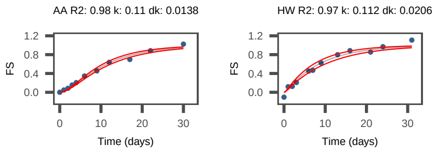

IDHP − DQTNDQVTIDSALATQK_3

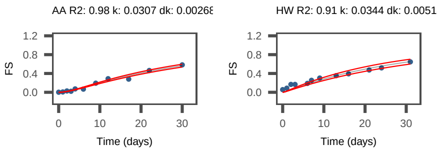

IDHP − VEKPVVEMDGDEMTR_3

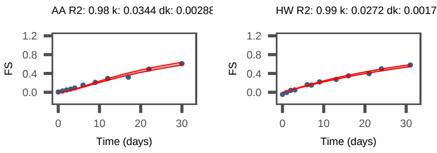

IGHM − LVESGFTTDPVTIENK_2

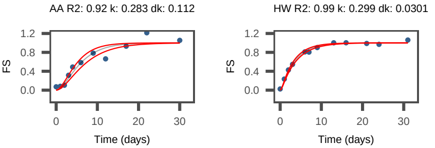

IVD − FLQENLAPK_2

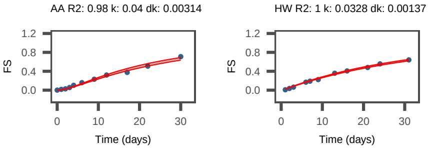

KCRB − LGFSEVELVQMVVDGVK_2

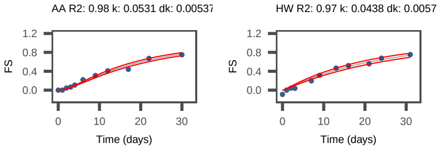

IGKC − DSTYSMSSTLTLTK_2

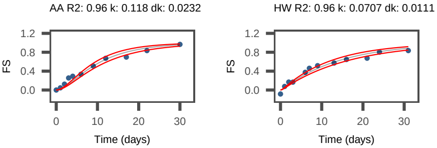

IVD − GSNTCELVFEDCK_2

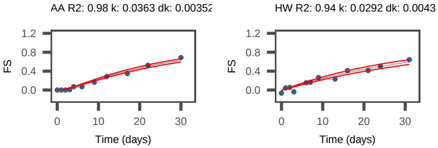

KCRB − LGFSEVELVQMVVDGVK_3

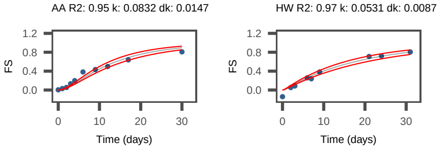

IPYR − DFAVDIIK_2

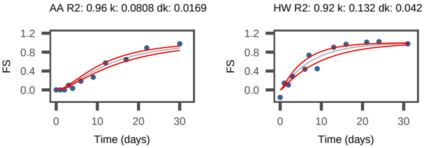

KAD1 − IIFVVGGPGSGK_2

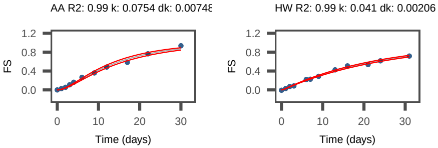

KCRM − DKETPSGFTLDDVIQTGVDNPGHPF_3

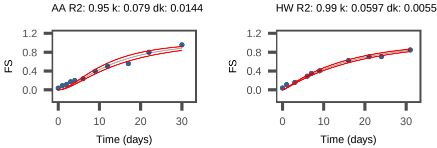

IPYR − LKPGYLEATVDWFR_3

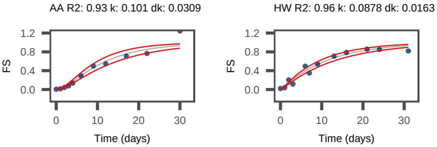

KAD1 − VNAEGTVDTVFSEVCTYLDSLK_2

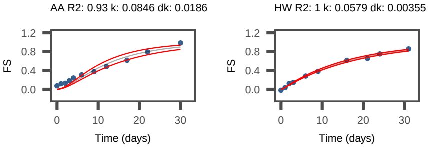

KCRM − IMTVGCVAGDEESYTVFK_2

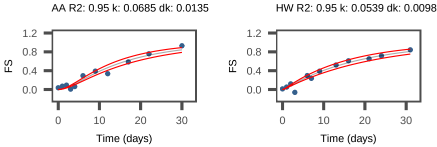

IPYR2 − HVAGHYISPFHDIPLK_4

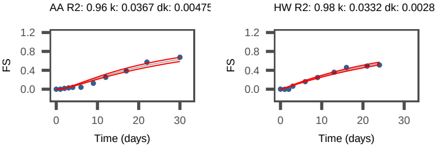

KAD1 − VNAEGTVDTVFSEVCTYLDSLK_3

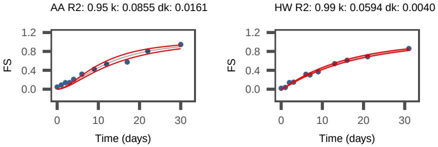

KCRM − TVGCVAGDEESYTVFK_2

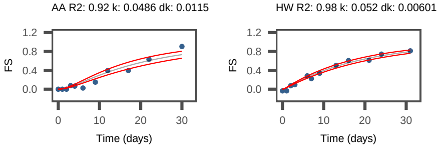

IVD − AQEIDQTNDFK_2

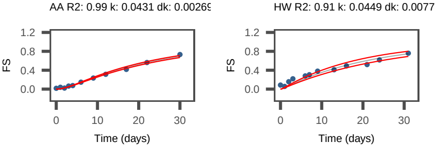

KAP2 − NLDQEQLSQVLDAMFEK_3

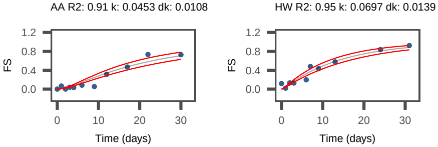

KCRM − VLTPDLYNK_2

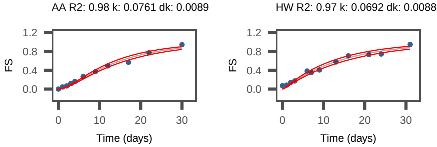

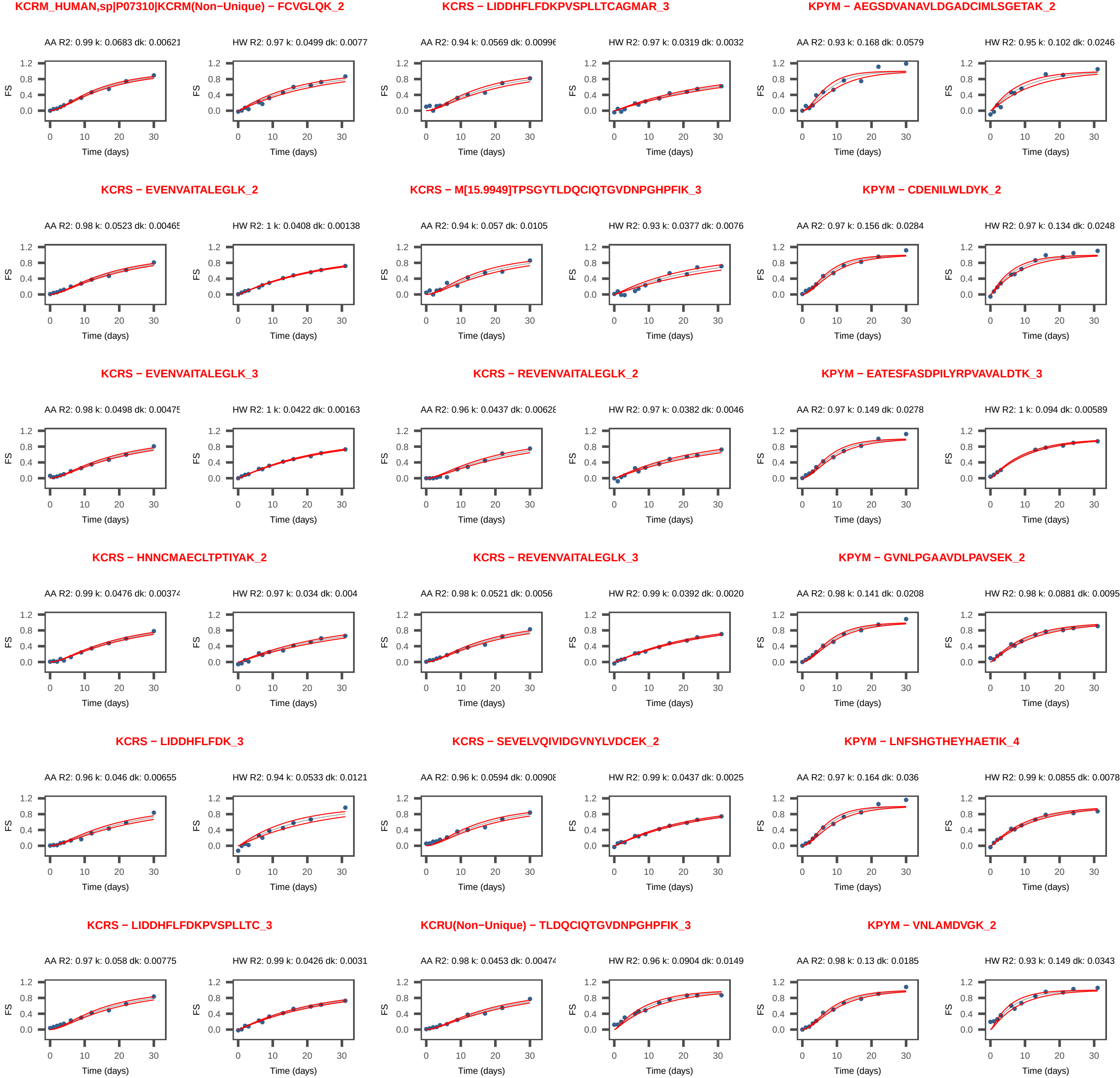
KCRM_HUMAN,sp|P07310|KCRM(Non−Unique) − FCVGLQK_2
AA R2: 0.99 k: 0.0683 dk: 0.00621
HW R2: 0.97 k: 0.0499 dk: 0.0077
KCRS − LIDDHFLFDKPVSPLLTCAGMAR_3
AA R2: 0.94 k: 0.0569 dk: 0.0099
HW R2: 0.97 k: 0.0319 dk: 0.0032
KPYM − AEGSDVANAVLDGADCIMLSGETAK_2
AA R2: 0.93 k: 0.168 dk: 0.0579
HW R2: 0.95 k: 0.102 dk: 0.0246
KCRS − EVENVAITALEGLK_2
AA R2: 0.98 k: 0.0523 dk: 0.00465
HW R2: 1 k: 0.0408 dk: 0.00138
KCRS − M[15.9949]TPSGYTLDQCIQTGVDNPGHPFIK_3
AA R2: 0.94 k: 0.057 dk: 0.0105
HW R2: 0.93 k: 0.0377 dk: 0.0076
KPYM − CDENILWLDYK_2
AA R2: 0.97 k: 0.156 dk: 0.0284
HW R2: 0.97 k: 0.134 dk: 0.0248
KCRS − EVENVAITALEGLK_3
AA R2: 0.98 k: 0.0498 dk: 0.00475
HW R2: 1 k: 0.0422 dk: 0.00163
KCRS − REVENVAITALEGLK_2
AA R2: 0.96 k: 0.0437 dk: 0.00628
HW R2: 0.97 k: 0.0382 dk: 0.0046
KPYM − EATESFASDPILYRPVAVALDTK_3
AA R2: 0.97 k: 0.149 dk: 0.0278
HW R2: 1 k: 0.094 dk: 0.00589
KCRS − HNNCMAECLTPTIYAK_2
AA R2: 0.99 k: 0.0476 dk: 0.00374
HW R2: 0.97 k: 0.034 dk: 0.004
KCRS − REVENVAITALEGLK_3
AA R2: 0.98 k: 0.0521 dk: 0.0056
HW R2: 0.99 k: 0.0392 dk: 0.0020
KPYM − GVNLPGAAVDLPAVSEK_2
AA R2: 0.98 k: 0.141 dk: 0.0208
HW R2: 0.98 k: 0.0881 dk: 0.0095
KCRS − LIDDHFLFDK_3
AA R2: 0.96 k: 0.046 dk: 0.00655
HW R2: 0.94 k: 0.0533 dk: 0.0121
KCRS − SEVELVQIVIDGVNYLVDCEK_2
AA R2: 0.96 k: 0.0594 dk: 0.00908
HW R2: 0.99 k: 0.0437 dk: 0.0025
KPYM − LNFSHGTHEYHAETIK_4
AA R2: 0.97 k: 0.164 dk: 0.036
HW R2: 0.99 k: 0.0855 dk: 0.0078
KCRS − LIDDHFLFDKPVSPLLTC_3
AA R2: 0.97 k: 0.058 dk: 0.00775
HW R2: 0.99 k: 0.0426 dk: 0.0031
KCRU(Non−Unique) − TLDQCIQTGVDNPGHPFIK_3
AA R2: 0.98 k: 0.0453 dk: 0.00474
HW R2: 0.96 k: 0.0904 dk: 0.0149
KPYM − VNLAMDVGK_2
AA R2: 0.98 k: 0.13 dk: 0.0185
HW R2: 0.93 k: 0.149 dk: 0.0343
FS
Time (days)

LAMB1 – DVLSALAEVEQLSK_2

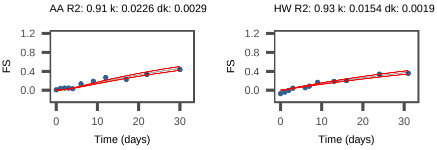

LDB3 – SASYNLSLTLQK_2

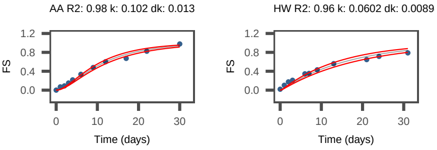

LDHA – LLIVSNPVDILTYVAWK_2

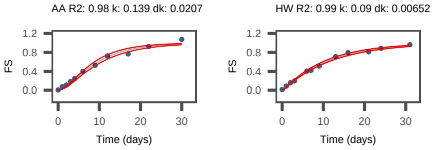

LAMB1 – QNAQDVLLK_2

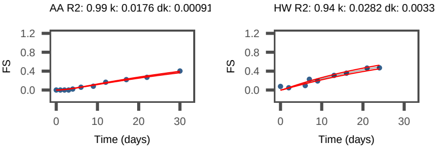

LDB3 – SLFHMEDGEPYCEK_3

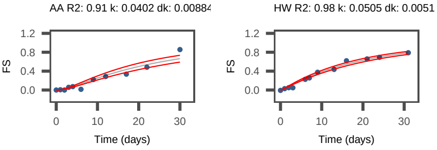

LDHA – LLIVSNPVDILTYVAWK_3

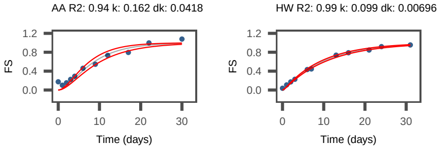

LDB3 – ASGAGLLGGSLPVK_2

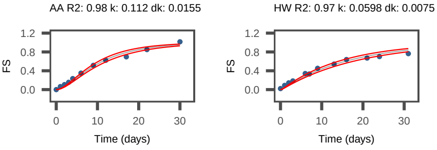

LDB3 – SWHPEEFNCAYCK_3

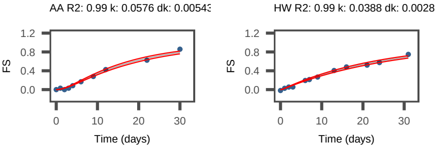

LDHA – QVVDSAYEVIK_2

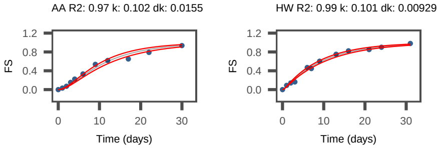

LDB3 – CHGCDFPVEAGDK_3

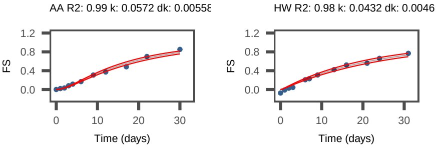

LDB3 – TQSKPEDEADEWAR_2

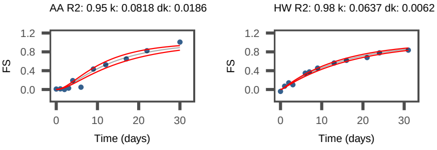

LDHB – FIIPQIVK_2

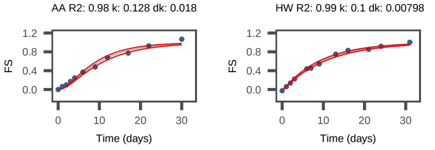

LDB3 – DLAVDSASPVYQAVIK_2

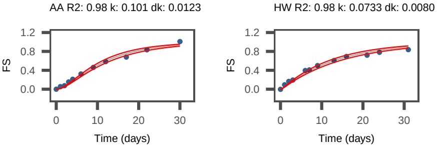

LDB3 – TQSKPEDEADEWAR_3

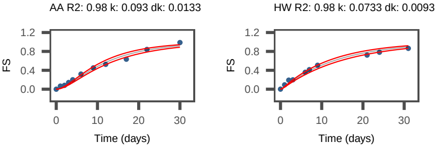

LDHB – GEMMDLQHGSLFLQTPK_3

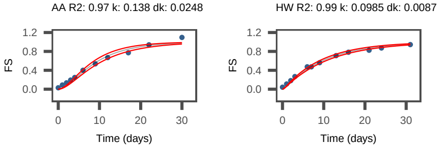

LDB3 – QTWHTTCFVCAACK_3

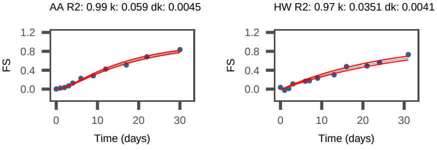

LDHA – DQLIVNLLK_2

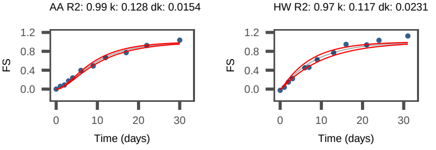

LDHB – GLTSVINQK_2

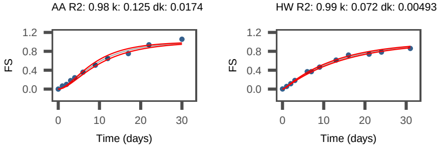

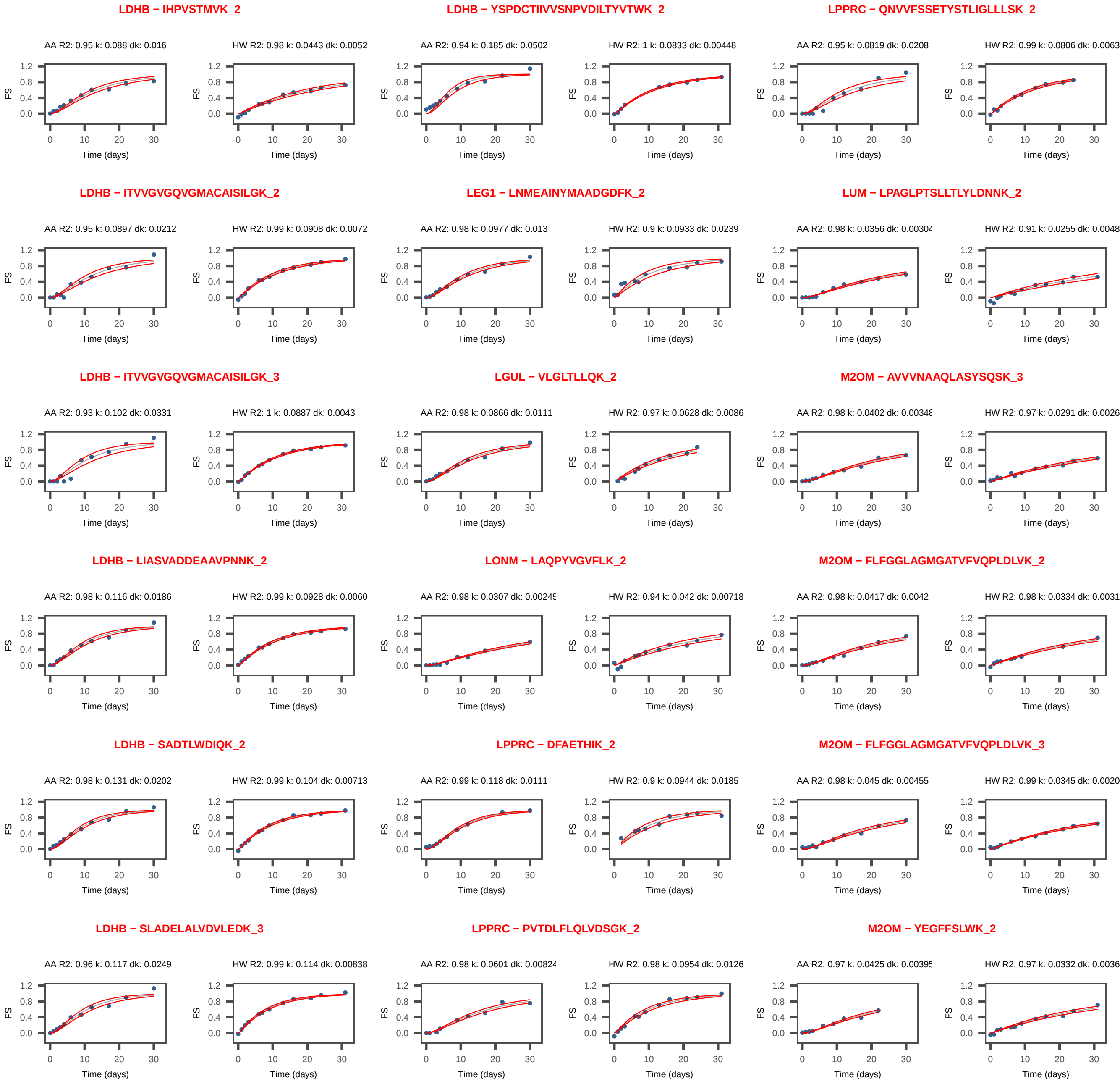

LDHB – IHPVSTMVK_2
AA R2: 0.95 k: 0.088 dk: 0.016
HW R2: 0.98 k: 0.0443 dk: 0.0052
LDHB – YSPDCTIIVVSNPVDILTYVTWK_2
AA R2: 0.94 k: 0.185 dk: 0.0502
HW R2: 1 k: 0.0833 dk: 0.00448
LPPRC – QNVVFSSETYSTLIGLLLSK_2
AA R2: 0.95 k: 0.0819 dk: 0.0208
HW R2: 0.99 k: 0.0806 dk: 0.0063
LDHB – ITVVGVGQVGMACAISILGK_2
AA R2: 0.95 k: 0.0897 dk: 0.0212
HW R2: 0.99 k: 0.0908 dk: 0.0072
LEG1 – LNMEAINYMAADGDFK_2
AA R2: 0.98 k: 0.0977 dk: 0.013
HW R2: 0.9 k: 0.0933 dk: 0.0239
LUM – LPAGLPTSLLTLYLDNNK_2
AA R2: 0.98 k: 0.0356 dk: 0.00304
HW R2: 0.91 k: 0.0255 dk: 0.0048
LDHB – ITVVGVGQVGMACAISILGK_3
AA R2: 0.93 k: 0.102 dk: 0.0331
HW R2: 1 k: 0.0887 dk: 0.0043
LGUL – VLGLTLLQK_2
AA R2: 0.98 k: 0.0866 dk: 0.0111
HW R2: 0.97 k: 0.0628 dk: 0.0086
M2OM – AVVVNAAQLASYSQSK_3
AA R2: 0.98 k: 0.0402 dk: 0.00348
HW R2: 0.97 k: 0.0291 dk: 0.0026
LDHB – LIASVADDEAAVPNNK_2
AA R2: 0.98 k: 0.116 dk: 0.0186
HW R2: 0.99 k: 0.0928 dk: 0.0060
LONM – LAQPYVGVFLK_2
AA R2: 0.98 k: 0.0307 dk: 0.00245
HW R2: 0.94 k: 0.042 dk: 0.00718
M2OM – FLFGGLAGMGATVFVQPLDLVK_2
AA R2: 0.98 k: 0.0417 dk: 0.0042
HW R2: 0.98 k: 0.0334 dk: 0.0031
LDHB – SADTLWDIQK_2
AA R2: 0.98 k: 0.131 dk: 0.0202
HW R2: 0.99 k: 0.104 dk: 0.00713
LPPRC – DFAETHIK_2
AA R2: 0.99 k: 0.118 dk: 0.0111
HW R2: 0.9 k: 0.0944 dk: 0.0185
M2OM – FLFGGLAGMGATVFVQPLDLVK_3
AA R2: 0.98 k: 0.045 dk: 0.00455
HW R2: 0.99 k: 0.0345 dk: 0.0020
LDHB – SLADELALVDVLEDK_3
AA R2: 0.96 k: 0.117 dk: 0.0249
HW R2: 0.99 k: 0.114 dk: 0.00838
LPPRC – PVTDLFLQLVDSGK_2
AA R2: 0.98 k: 0.0601 dk: 0.00824
HW R2: 0.98 k: 0.0954 dk: 0.0126
M2OM – YEGFFSLWK_2
AA R2: 0.97 k: 0.0425 dk: 0.00395
HW R2: 0.97 k: 0.0332 dk: 0.0036
FS
Time (days)

MAAI − MISDLIASGIQPLQNLSVLK_2

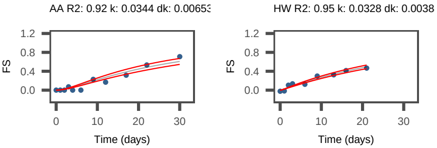

MDHM − GYLGPEQLPDCLK_2

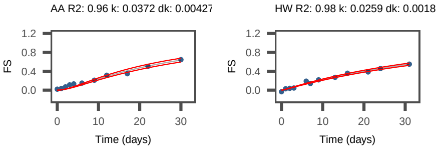

MGDP1 − TSEIQGANQLLELFDLGK_2

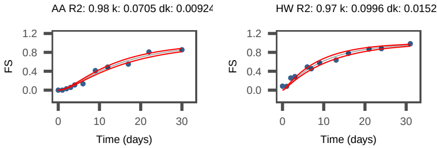

MCAT − DVPASGMYFMTYEWLK_2

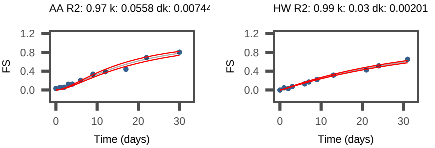

MDHM − IIANPVNSTIPITAEVFK_2

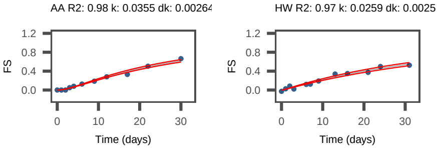

MIC13 − DSWNSGIISVMSALSVAPSK_3

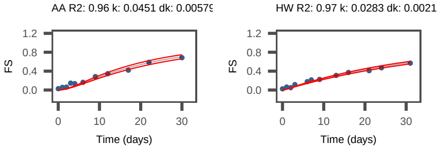

MDHC − HSSTQYPDVNHAK_3

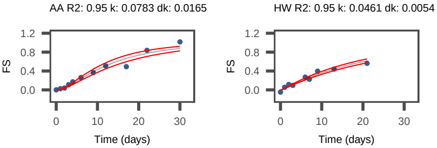

MDHM − IQEAGTEVVK_2

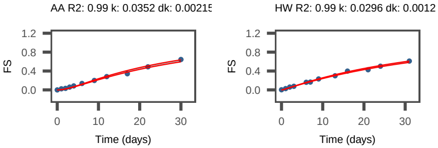

MIC26 − GYIVIEDLWK_2

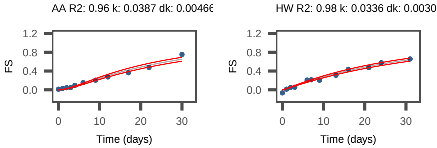

MDHC(Non−Unique) − SAPSIPK_2

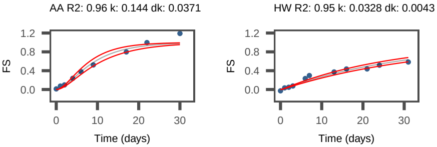

MDHM − VAVLGASGGIGQPLSLLLK_3

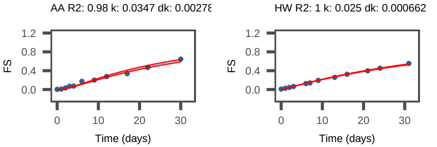

MIC27 − QLVRPDQLPIYTAPPLHSK_4

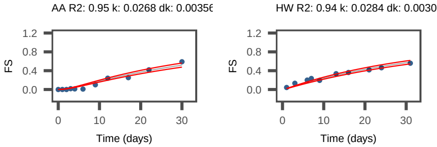

MDHC − SLLYSIGNGSVFGK_2

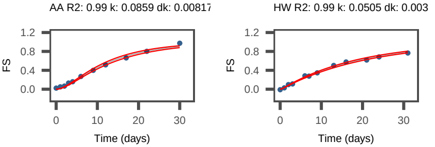

MDHM − VNVPVIGGHAGK_2

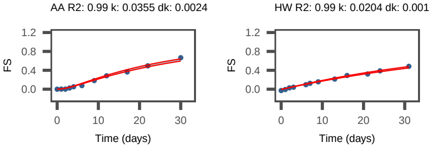

MIC60 − AHQLWLSVEALK_3

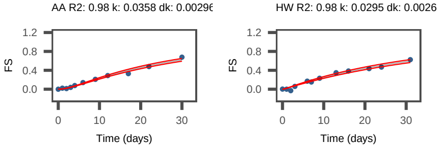

MDHM − ANTFVAELK_2

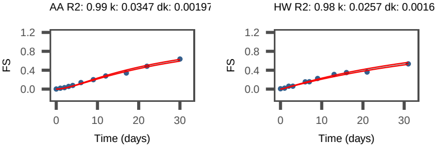

MDHM − VNVPVIGGHAGK_3

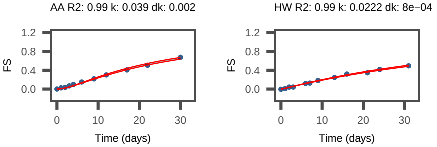

MIC60 − LFGMVLGSAPYTVPLPK_2

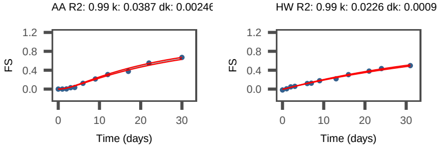

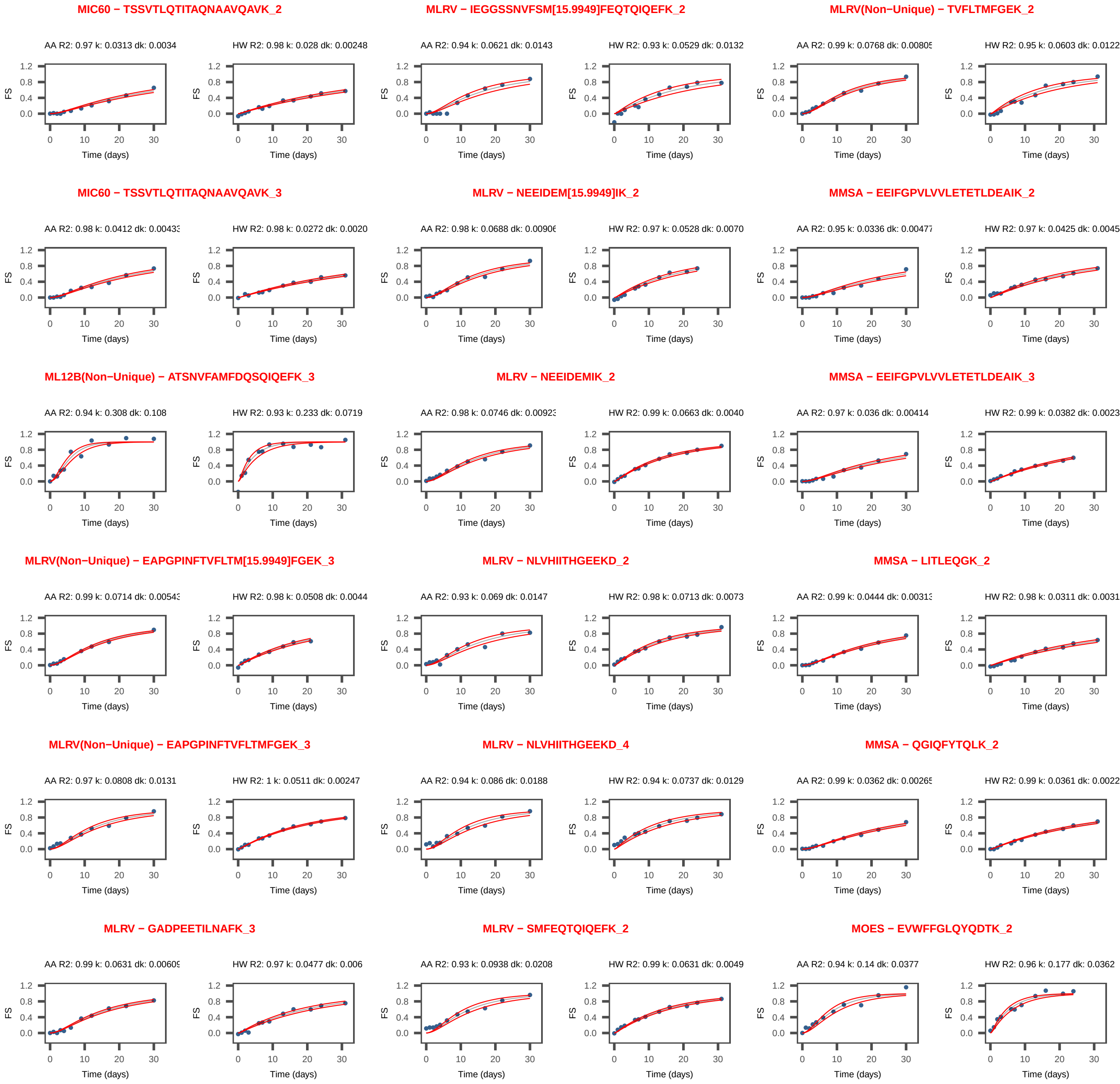
MIC60 − TSSVTLQTITAQNAAVQAVK_2
AA R2: 0.97 k: 0.0313 dk: 0.0034
HW R2: 0.98 k: 0.028 dk: 0.00248
MLRV − IEGGSSNVFSM[15.9949]FEQTQIQEFK_2
AA R2: 0.94 k: 0.0621 dk: 0.0143
HW R2: 0.93 k: 0.0529 dk: 0.0132
MLRV(Non−Unique) − TVFLTMFGEK_2
AA R2: 0.99 k: 0.0768 dk: 0.00805
HW R2: 0.95 k: 0.0603 dk: 0.0122
MIC60 − TSSVTLQTITAQNAAVQAVK_3
AA R2: 0.98 k: 0.0412 dk: 0.00433
HW R2: 0.98 k: 0.0272 dk: 0.0020
MLRV − NEEIDEM[15.9949]IK_2
AA R2: 0.98 k: 0.0688 dk: 0.00906
HW R2: 0.97 k: 0.0528 dk: 0.0070
MMSA − EEIFGPVLVVLETETLDEAIK_2
AA R2: 0.95 k: 0.0336 dk: 0.00477
HW R2: 0.97 k: 0.0425 dk: 0.0045
ML12B(Non−Unique) − ATSNVFAMFDQSQIQEFK_3
AA R2: 0.94 k: 0.308 dk: 0.108
HW R2: 0.93 k: 0.233 dk: 0.0719
MLRV − NEEIDEMIK_2
AA R2: 0.98 k: 0.0746 dk: 0.00923
HW R2: 0.99 k: 0.0663 dk: 0.0040
MMSA − EEIFGPVLVVLETETLDEAIK_3
AA R2: 0.97 k: 0.036 dk: 0.00414
HW R2: 0.99 k: 0.0382 dk: 0.0023
MLRV(Non−Unique) − EAPGPINFTVFLTM[15.9949]FGEK_3
AA R2: 0.99 k: 0.0714 dk: 0.00543
HW R2: 0.98 k: 0.0508 dk: 0.0044
MLRV − NLVHIITHGEEKD_2
AA R2: 0.93 k: 0.069 dk: 0.0147
HW R2: 0.98 k: 0.0713 dk: 0.0073
MMSA − LITLEQGK_2
AA R2: 0.99 k: 0.0444 dk: 0.00313
HW R2: 0.98 k: 0.0311 dk: 0.0031
MLRV(Non−Unique) − EAPGPINFTVFLTMFGEK_3
AA R2: 0.97 k: 0.0808 dk: 0.0131
HW R2: 1 k: 0.0511 dk: 0.00247
MLRV − NLVHIITHGEEKD_4
AA R2: 0.94 k: 0.086 dk: 0.0188
HW R2: 0.94 k: 0.0737 dk: 0.0129
MMSA − QGIQFYTQLK_2
AA R2: 0.99 k: 0.0362 dk: 0.00265
HW R2: 0.99 k: 0.0361 dk: 0.0022
MLRV − GADPEETILNAFK_3
AA R2: 0.99 k: 0.0631 dk: 0.00609
HW R2: 0.97 k: 0.0477 dk: 0.006
MLRV − SMFEQTQIQEFK_2
AA R2: 0.93 k: 0.0938 dk: 0.0208
HW R2: 0.99 k: 0.0631 dk: 0.0049
MOES − EVWFFGLQYQDTK_2
AA R2: 0.94 k: 0.14 dk: 0.0377
HW R2: 0.96 k: 0.177 dk: 0.0362
FS
Time (days)

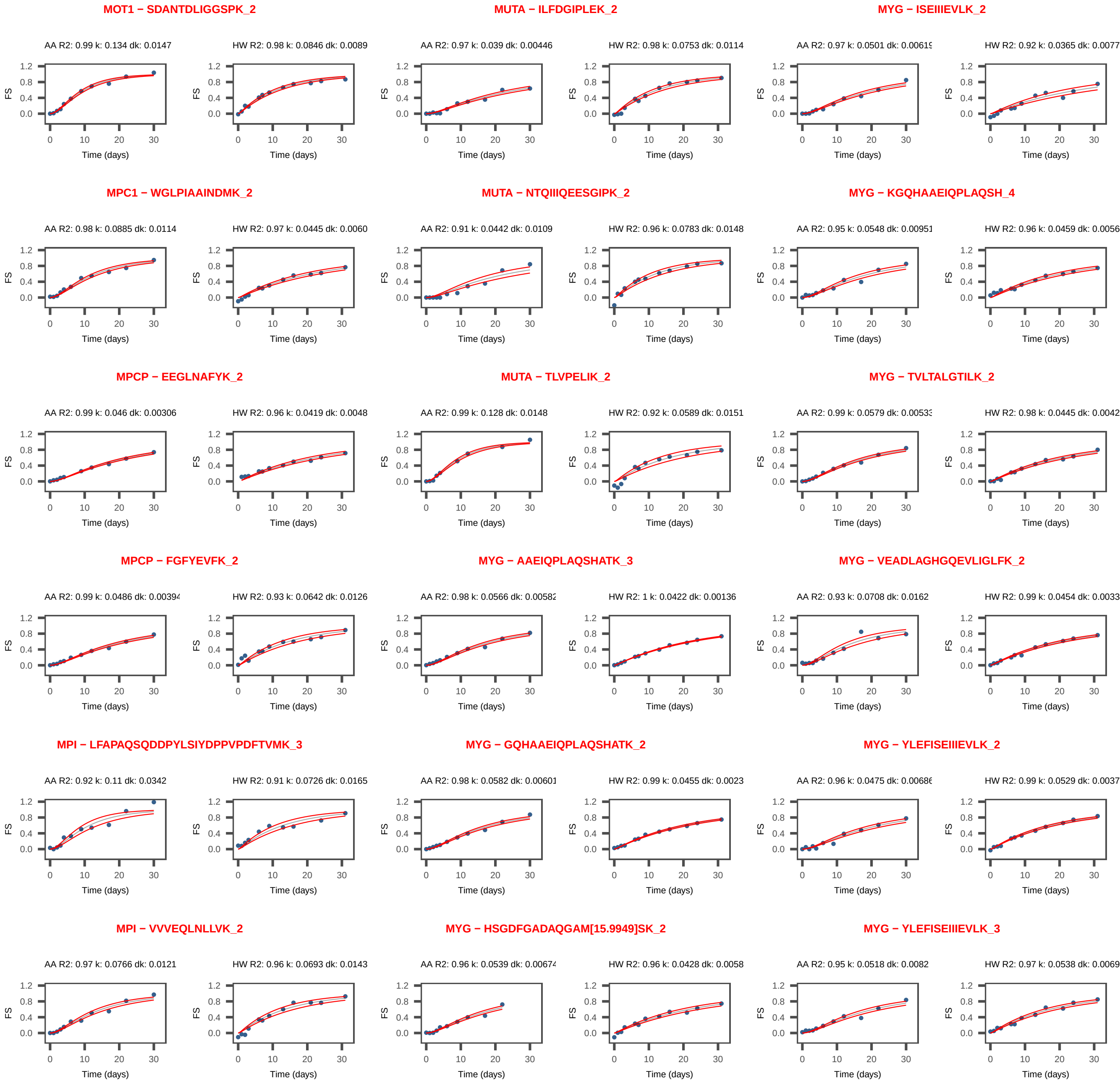
MOT1 – SDANTDLIGGSPK_2
AA R2: 0.99 k: 0.134 dk: 0.0147
HW R2: 0.98 k: 0.0846 dk: 0.0089
MUTA – ILFDGIPLEK_2
AA R2: 0.97 k: 0.039 dk: 0.00446
HW R2: 0.98 k: 0.0753 dk: 0.0114
MYG – ISEIIIEVLK_2
AA R2: 0.97 k: 0.0501 dk: 0.0061
HW R2: 0.92 k: 0.0365 dk: 0.0077
MPC1 – WGLPIAAINDMK_2
AA R2: 0.98 k: 0.0885 dk: 0.0114
HW R2: 0.97 k: 0.0445 dk: 0.0060
MUTA – NTQIIIQEESGIPK_2
AA R2: 0.91 k: 0.0442 dk: 0.0109
HW R2: 0.96 k: 0.0783 dk: 0.0148
MYG – KGQHAAEIQPLAQSH_4
AA R2: 0.95 k: 0.0548 dk: 0.00951
HW R2: 0.96 k: 0.0459 dk: 0.0056
MPCP – EEGLNAFYK_2
AA R2: 0.99 k: 0.046 dk: 0.00306
HW R2: 0.96 k: 0.0419 dk: 0.0048
MUTA – TLVPELIK_2
AA R2: 0.99 k: 0.128 dk: 0.0148
HW R2: 0.92 k: 0.0589 dk: 0.0151
MYG – TVLTALGTILK_2
AA R2: 0.99 k: 0.0579 dk: 0.0053
HW R2: 0.98 k: 0.0445 dk: 0.0042
MPCP – FGFYEVFK_2
AA R2: 0.99 k: 0.0486 dk: 0.0039
HW R2: 0.93 k: 0.0642 dk: 0.0126
MYG – AAEIQPLAQSHATK_3
AA R2: 0.98 k: 0.0566 dk: 0.0058
HW R2: 1 k: 0.0422 dk: 0.00136
MYG – VEADLAGHGQEVLIGLFK_2
AA R2: 0.93 k: 0.0708 dk: 0.0162
HW R2: 0.99 k: 0.0454 dk: 0.0033
MPI – LFAPAQSQDDPYLSIYDPPVPDFTVMK_3
AA R2: 0.92 k: 0.11 dk: 0.0342
HW R2: 0.91 k: 0.0726 dk: 0.0165
MYG – GQHAAEIQPLAQSHATK_2
AA R2: 0.98 k: 0.0582 dk: 0.00601
HW R2: 0.99 k: 0.0455 dk: 0.0023
MYG – YLEFISEIIIEVLK_2
AA R2: 0.96 k: 0.0475 dk: 0.0068
HW R2: 0.99 k: 0.0529 dk: 0.0037
MPI – VVVEQLNLLVK_2
AA R2: 0.97 k: 0.0766 dk: 0.0121
HW R2: 0.96 k: 0.0693 dk: 0.0143
MYG – HSGDFGADAQGAM[15.9949]SK_2
AA R2: 0.96 k: 0.0539 dk: 0.0067
HW R2: 0.96 k: 0.0428 dk: 0.0058
MYG – YLEFISEIIIEVLK_3
AA R2: 0.95 k: 0.0518 dk: 0.0082
HW R2: 0.97 k: 0.0538 dk: 0.0069
FS
Time (days)

MYG_HUMAN,sp|P04247|MYG(Non−Unique) − GLSDGEWQLVLNVWGK_3

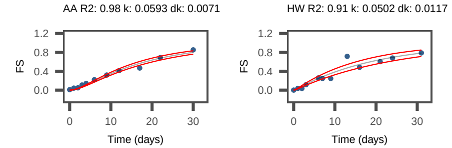

MYH3(Non−Unique) − PLNETVVGLYQK_2

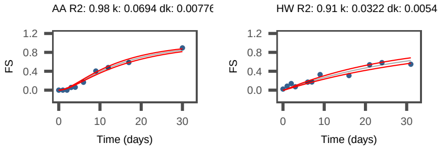

MYH6(Non−Unique) − EDQVM[15.9949]QQNPPK_2

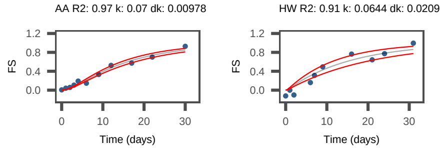

MYH11(Non−Unique) − NVHELEK_2

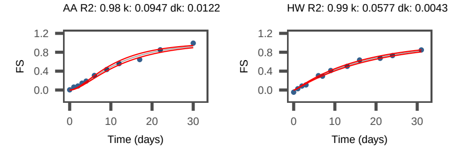

MYH3(Non−Unique) − TKYETDAIQR_2

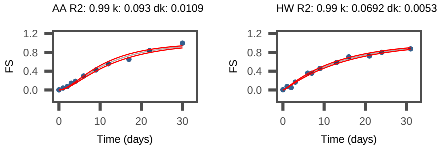

MYH6(Non−Unique) − EDQVMQQNPPK_3

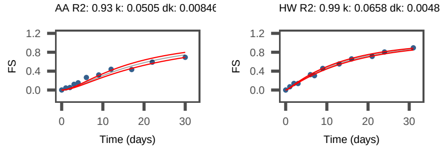

MYH3(Non−Unique) − ADIAESQVNK_2

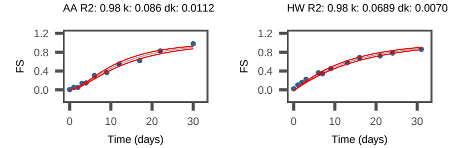

MYH3(Non−Unique) − TKYETDAIQR_3

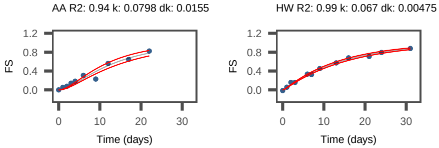

MYH6 − EFDISQQNSK_2

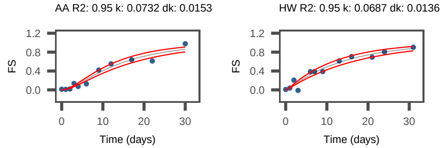

MYH3(Non−Unique) − LASADIETYLLEK_2

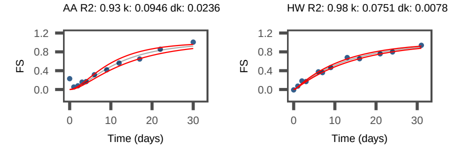

MYH6(Non−Unique) − AQLEFNQIK_2

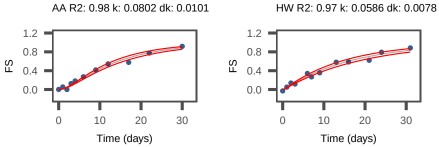

MYH6 − EMANMKEEFGR_2

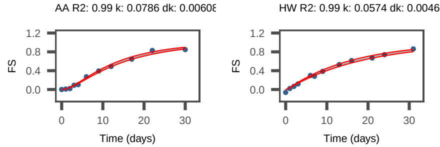

MYH3(Non−Unique) − LQDLVDK_2

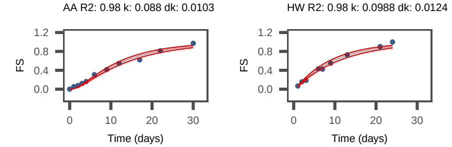

MYH6 − DTQLQLDDAVHANDDLK_2

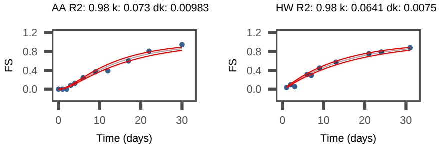

MYH6 − EMANMKEEFGR_3

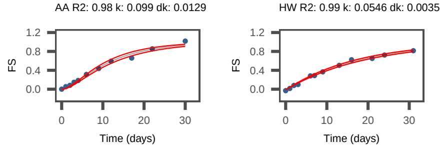

MYH3(Non−Unique) − LQQFFNHHMFVLEQEEYK_4

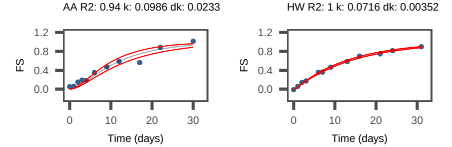

MYH6 − DTQLQLDDAVHANDDLKENIAIVER_3

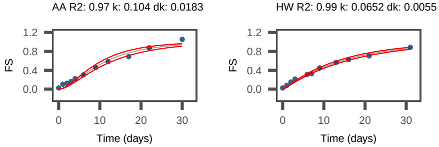

MYH6 − FSLVHYAGTVDYNIMGWLEK_3

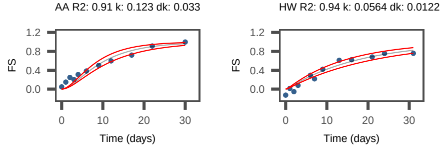

MYH6 − GQSVQQVYYSIGALAK_3

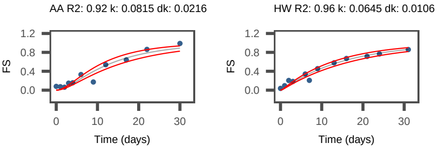

MYH6 − KAPGVMDNPLVMHQLR_3

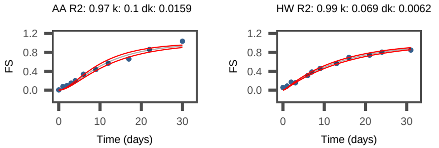

MYH6 − KMESDLTQLQTEVEEAVQECR_3

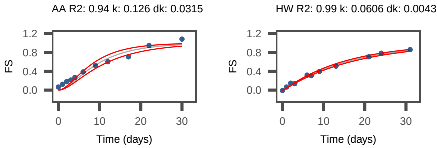

MYH6 − IEDEQALALQLQK_3

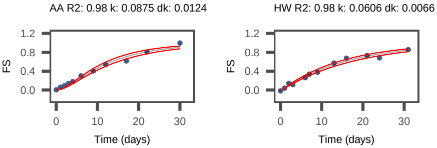

MYH6(Non−Unique) − KLAEQELIETSER_2

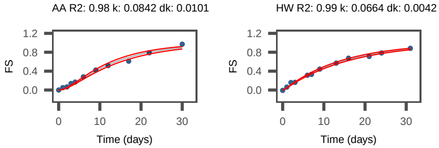

MYH6(Non−Unique) − KPELLDML_2

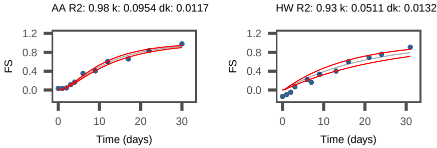

MYH6(Non−Unique) − IEDM[15.9949]AMLTFLHEPAVLYNLK_3

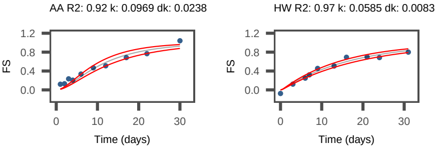

MYH6(Non−Unique) − KLAEQELIETSER_3

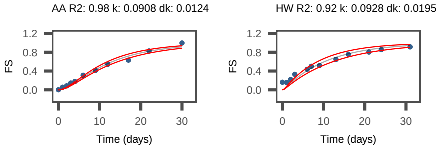

MYH6 − KPELLDMLLVTNNPYDYAF_2

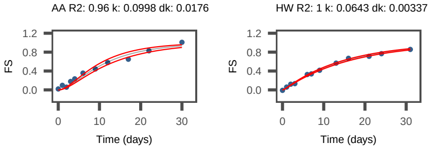

MYH6(Non−Unique) − IEDMAMLTFLHEPAVLYNLK_2

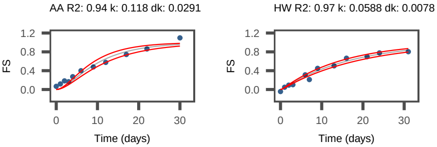

MYH6 − KMEGDLNEM[15.9949]EIQLSQANR_3

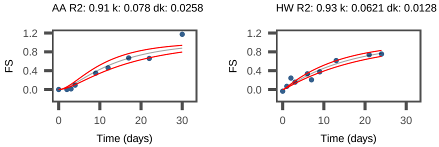

MYH6 − KPELLDMLLVTNNPYDYAF_3

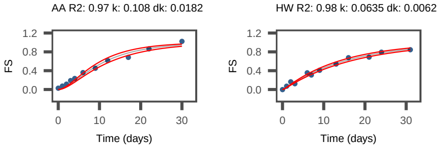

MYH6(Non−Unique) − IHFGATGK_2

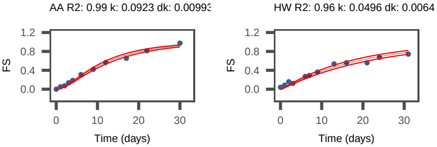

MYH6 − KMEGDLNEMEIQLSQANR_2

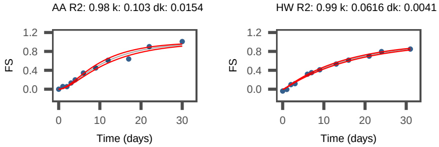

MYH6(Non−Unique) − KVQHELDEAEER_2

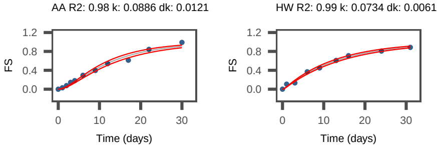

MYH6(Non−Unique) − INATLETK_2

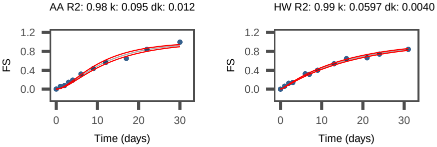

MYH6 − KMEGDLNEMEIQLSQANR_3

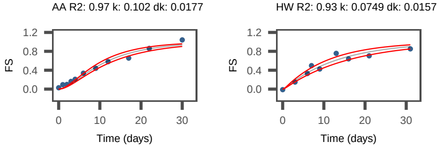

MYH6(Non−Unique) − KVQHELDEAEER_4

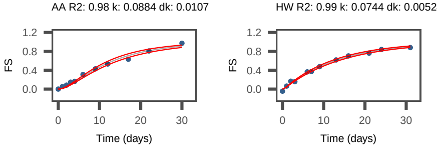

MYH6(Non−Unique) − LEDEEEM[15.9949]NAELTAK_2

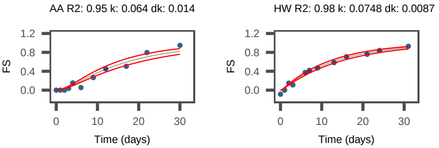

MYH6 − LMATLFSTYASADTGDSGK_3

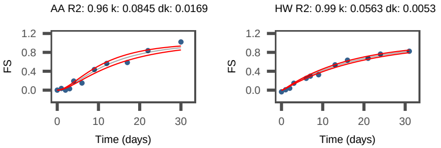

MYH6 − LTGAIMHYGNMK_3

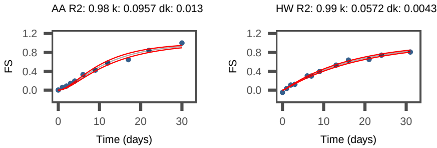

MYH6(Non−Unique) − LEEAGGATSVQIEM[15.9949]NK_2

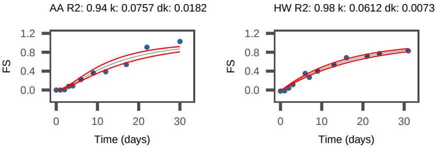

MYH6(Non−Unique) − LMGLNSADLLK_2

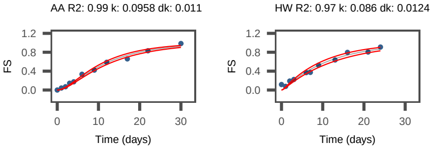

MYH6(Non−Unique) − LTQESIMDLENDK_2

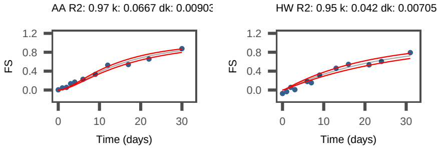

MYH6(Non−Unique) − LELDDVTSNMEQIIK_3

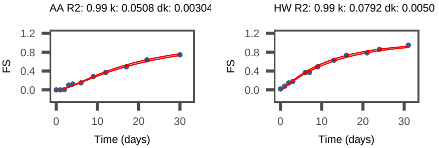

MYH6 − LSYTQQM[15.9949]EDLKR_3

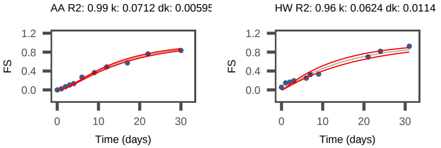

MYH6(Non−Unique) − LYDNHLGK_2

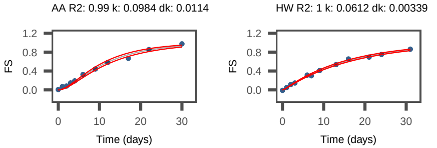

MYH6(Non−Unique) − LHEPAVLYNLK_2

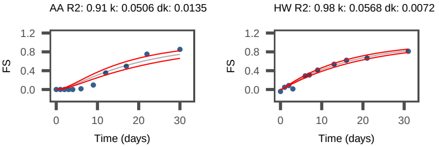

MYH6 − LSYTQQMEDLK_2

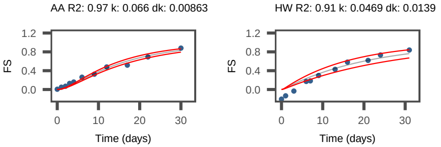

MYH6 − NLTEEMAGLDEIIAK_3

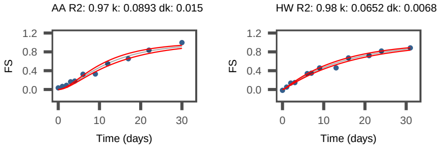

MYH6(Non−Unique) − LHEPAVLYNLK_3

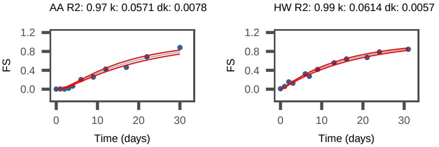

MYH6 − LTGAIM[15.9949]HYGNMK_2

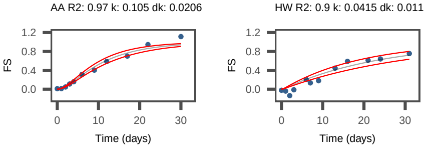

MYH6 − NYHIFYQILSNK_3

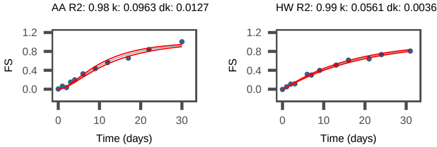

MYH6(Non−Unique) − LLGSLDIDHNQYK_2

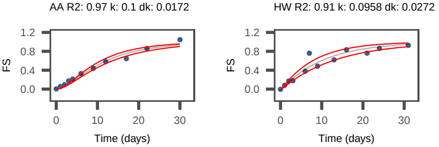

MYH6 − LTGAIMHYGNMK_2

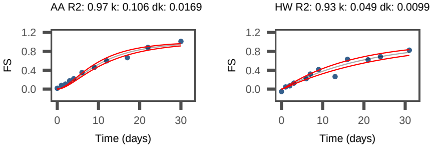

MYH6(Non−Unique) − PMGIMSILEEECMFPK_2

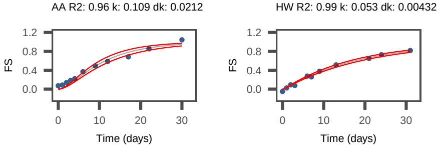

MYH6(Non−Unique) − PMGIMSILEEECMFPK_3

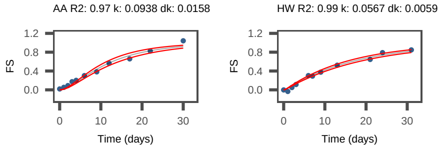

MYH6 − SVQQVYYSIGALAK_2

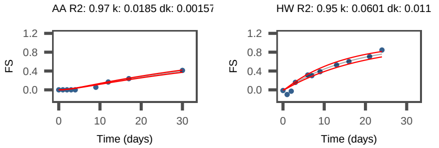

MYH7 − QEAHFSLVHYAGTVDYNILGWLQK_4

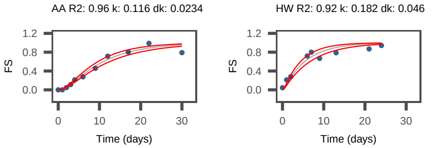

MYH6(Non−Unique) − QAEEAEEQANTNLSK_3

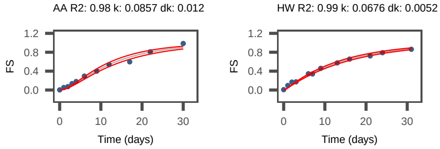

MYH6(Non−Unique) − VRELENELEAEQK_3

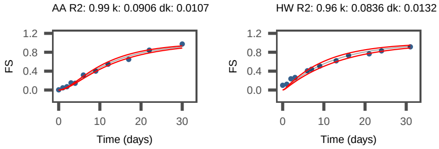

MYH7B(Non−Unique) − AITDAAMMAEELK_3

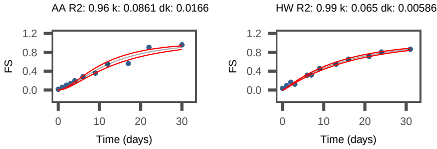

MYH6 − QEAHFSLVHYAGTVDYNIMGWLEK_3

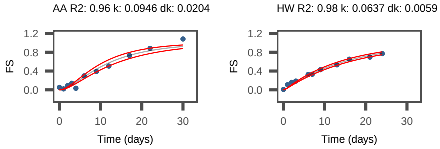

MYH6(Non−Unique) − VRELENELEAEQKR_4

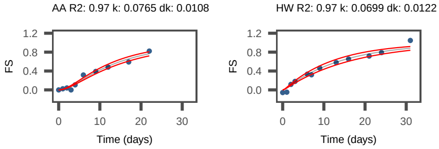

MYH7B(Non−Unique) − ILEEECMFPK_2

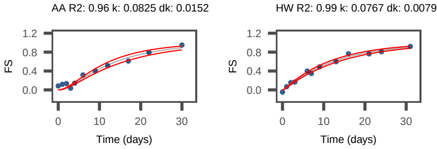

MYH6 − QREEQAEPDGTEDADK_2

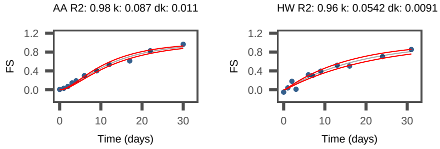

MYH6(Non−Unique) − YEESQSELESSQK_2

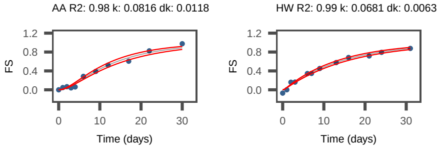

MYH7B(Non−Unique) − MFVLEQEEYK_2

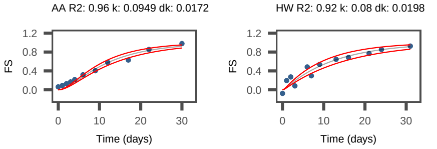

MYH6(Non−Unique) − RQAEEAEEQANTNLSK_3

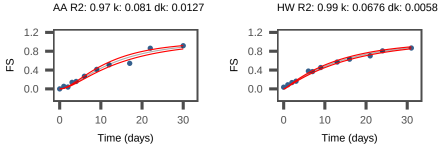

MYH6(Non−Unique) − YEESQSELESSQK_3

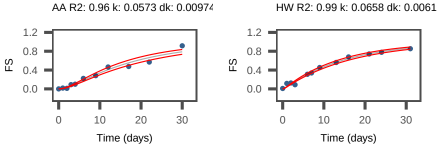

MYH8(Non−Unique) − LEQQVDDLEGSLEQEK_2

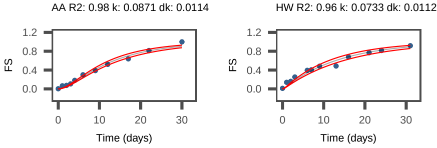

MYH6(Non−Unique) − SQNTSLINQK_2

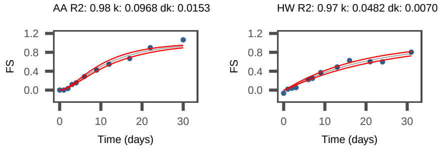

MYH6 − YSIGALAK_2

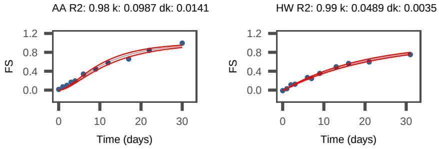

MYH9 − IIGLDQVAGMSETALPGAFK_2

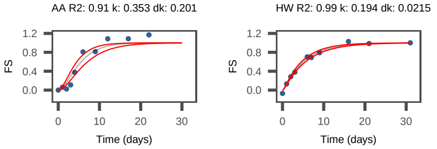

MYH9 – VISGVLQLGNIAFK_2

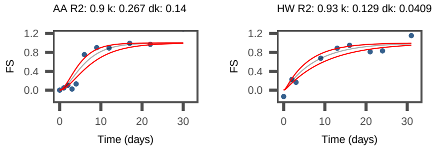

MYL3 – NKDTGTYEDFVEGLR_2

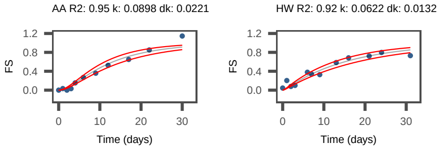

MYOM1 – DAGFYEVILK_2

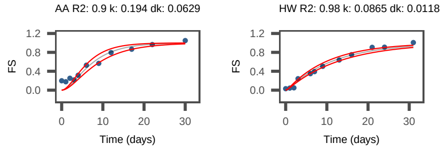

MYL3 – AAPAPAAAPAAAPAAAPEPERPK_2

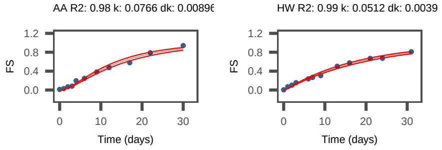

MYL3 – NKDTGTYEDFVEGLR_3

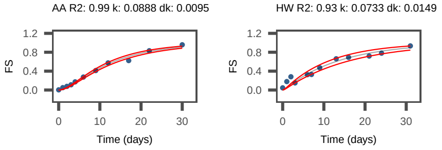

MYOM1 – DGICTLLITEFSK_2

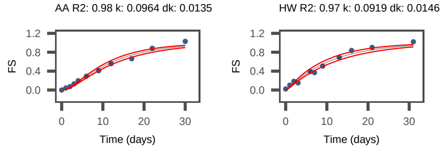

MYL3 – IEFTPEQIEEFKEAF_2

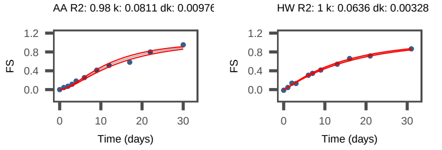

MYL6 – VLDFEHFLPMLQTVAK_3

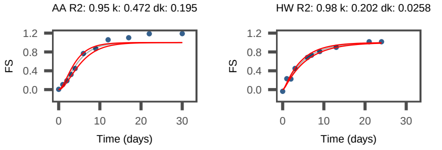

MYOM1 – DSMVLGWK_2

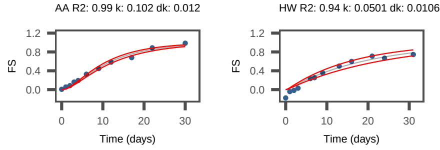

MYL3 – LTEDEVEK_2

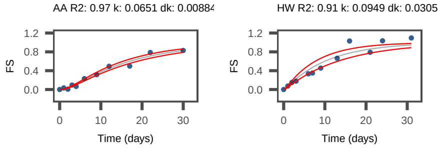

MYOM1 – ATGHSTLVLIGDVYK_3

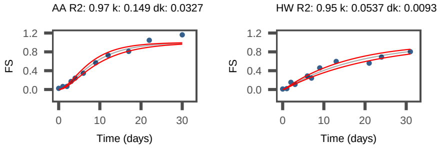

MYOM1 – FALFDLVEGK_2

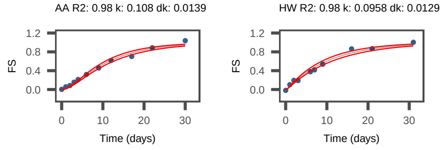

MYL3 – MMDFETFLPM[15.9949]LQHISK_3

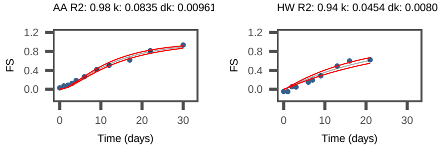

MYOM1 – AVNAAGLSEYSQDSEAIEVK_2

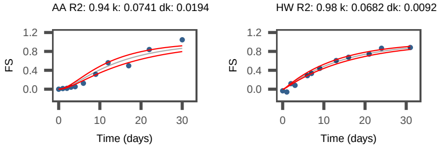

MYOM1 – LLALSQEHK_2

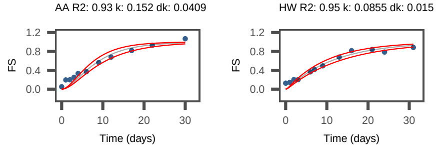

MYL3 – MMDFETFLPMLQHISK_2

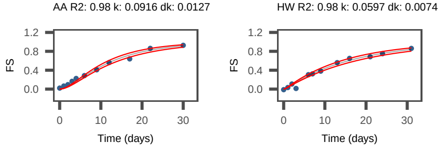

MYOM1 – CEVGTDTWSQCNDTPVK_2

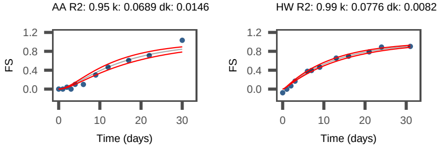

MYOM1 – LNEDHLLHAPEFIIKPR_5

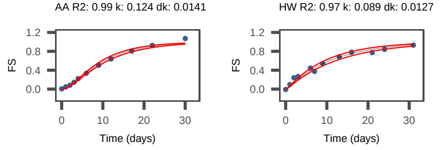

MYOM1 − LVDEAFQDLMTEVCK_2

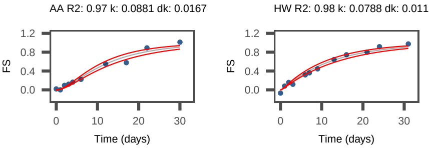

MYOM1 − STLVLIGDVYK_2

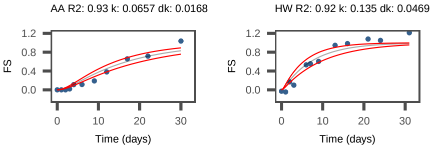

MYOM1 − YGMHTLEISK_3

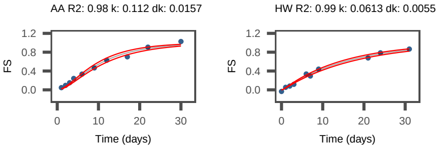

MYOM1 − LVDEAFQDLMTEVCK_3

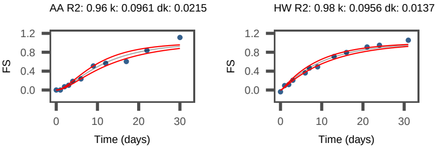

MYOM1 − TAFFTISGVSTADSGK_2

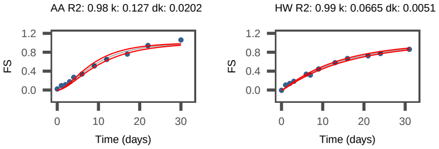

MYOM1 − YKGELDESLLR_2

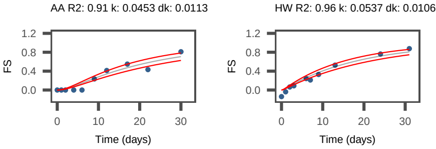

MYOM1 − NQVPINVHANPGK_3

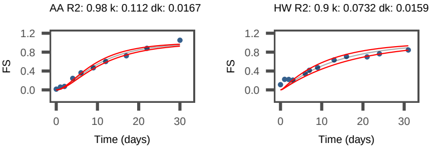

MYOM1 − TASAYDYGYSHGLTDSSLLLEDYSSK_3

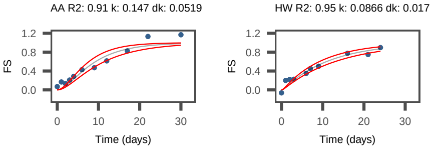

MYOZ2 − SPPNPENIAPGYSGPLK_2

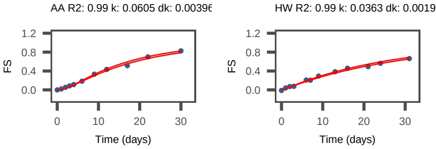

MYOM1 − NSLVLQWKPPVYSGR_3

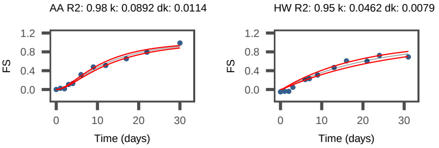

MYOM1 − TLEETQTYHGK_2

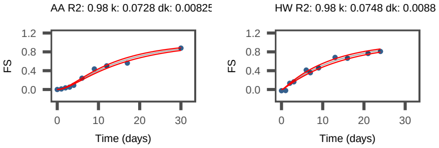

MYOZ2 − SPWEQAIGSDPELLEALYPK_2

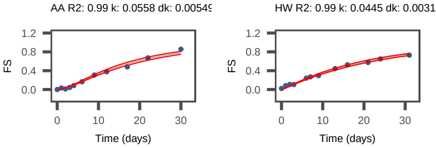

MYOM1 − PSDLAGPVVAETRPGTK_3

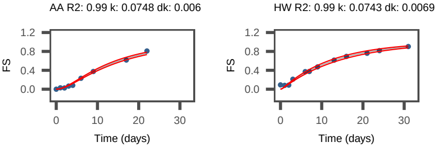

MYOM1 − VLGGLPDVVTIQEGK_2

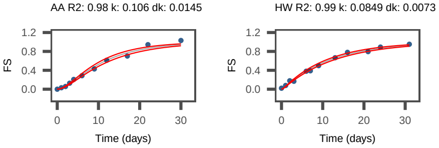

MYOZ2 − SPWEQAIGSDPELLEALYPK_3

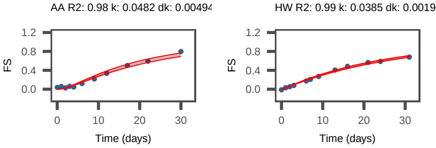

MYOM1 − SGVTGEQIWLQINEPTPNDK_2

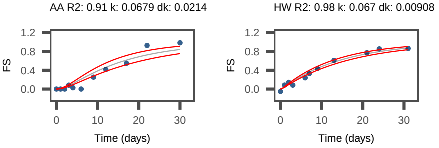

MYOM1 − VVITPEIK_2

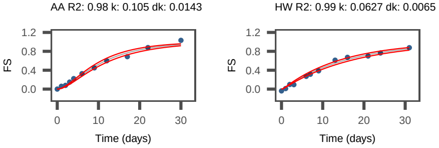

MYPC3 − AHNVAGPGGPIVTK_2

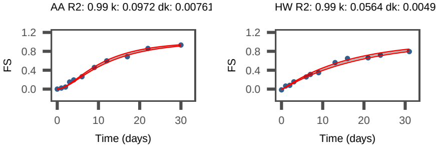

MYPC3 − AHNVAGPGGPIVTK_3

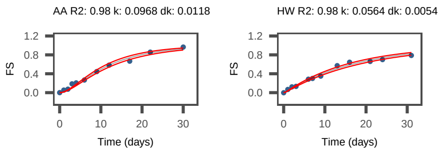

MYPC3 − KPCPYDGGVYVCR_3

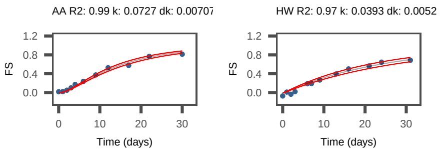

MYPC3 − SLADDAAYQCVVGGEK_2

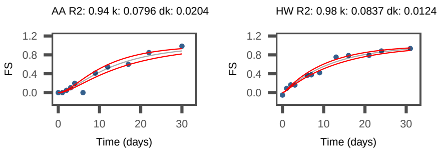

MYPC3 − ATLILQIVDKPSPPQDIR_3

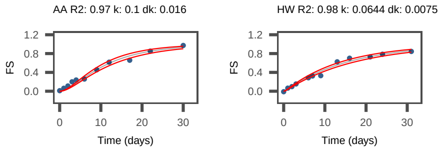

MYPC3 − LDVPISGDPAPTVVWQK_2

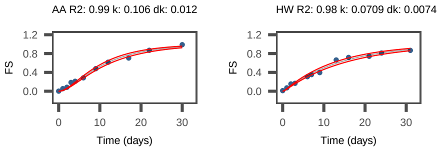

MYPC3 − TSGGQSLAELIVQEK_2

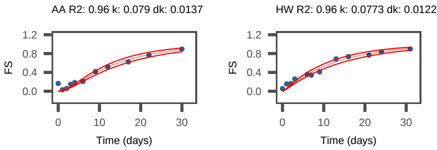

MYPC3 − DASPDDQGSYAVIAGSSK_2

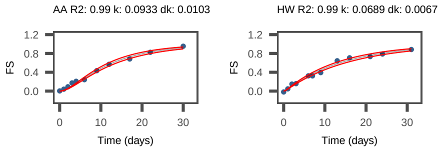

MYPC3 − LEVYQSIADLAVGAK_2

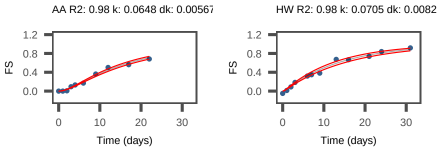

MYPC3 − VEFECEVSEEGAQVK_2

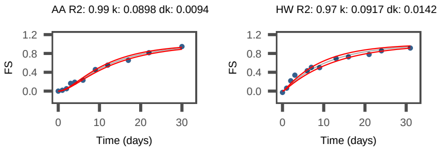

MYPC3 − DKFDSCNFNLTVHEAIGSGDLDLR_4

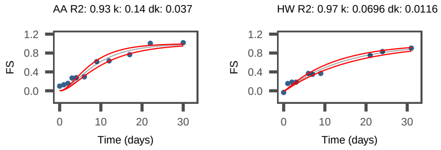

MYPC3 − LTVELADPDAEVK_2

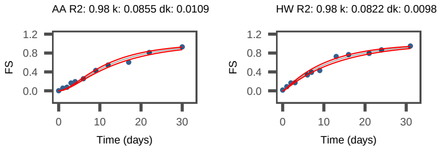

MYPC3 − VGEPVNLLIPFQGK_2

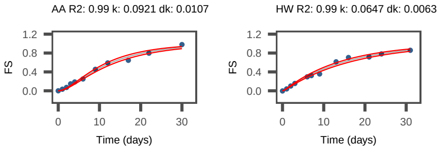

MYPC3 − EDEGVYTVTVK_2

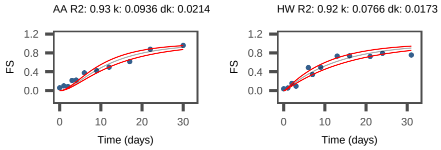

MYPC3 − NPVGEDQVNLTVK_2

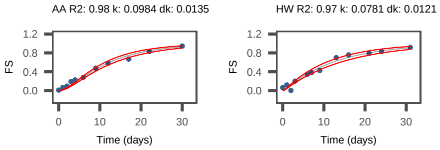

MYPC3 − VIDVPDAPAAPK_2

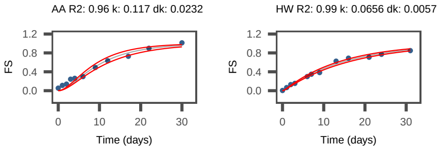

MYPC3 − KHHLIINEATLEDAGHYAVR_4

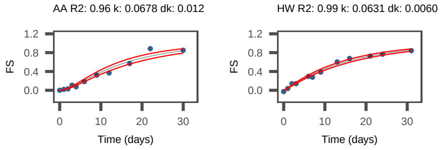

MYPC3 − PQVTWTK_2

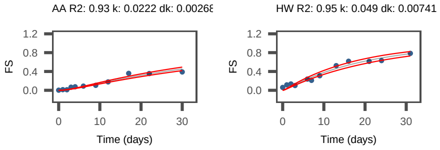

MYPC3 − YIFESVGAK_2

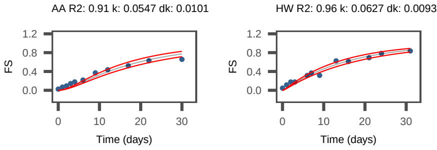

NAC1 − ALLLNELGGFTLTGK_2

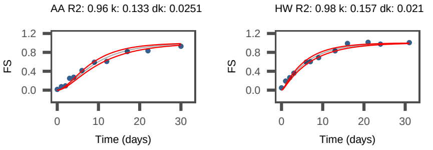

NDKA(Non−Unique) − GLVGEIIK_2

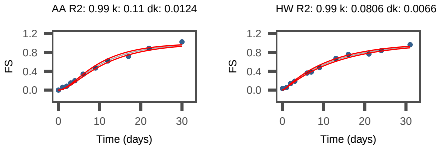

NDRG2 − GWMDWAAHK_3

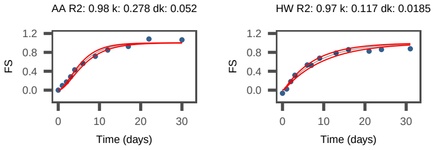

NAC1 − EIEQLIELANYQVLSQQQK_3

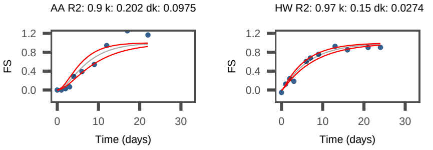

NDKA(Non−Unique) − TFIAIKPDGVQR_2

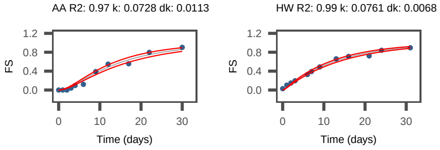

NDRG2 − LDPTQTSFLK_2

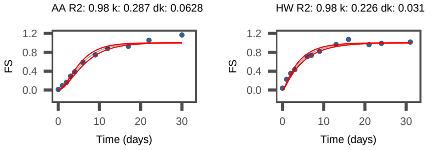

NACAM − GLPSAVALAPQTVPVEK_2

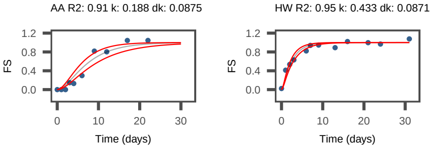

NDKA(Non−Unique) − TFIAIKPDGVQR_3

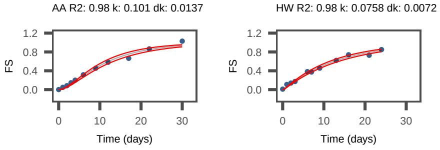

NDRG2 − MADSGGQPQLTQPGK_2

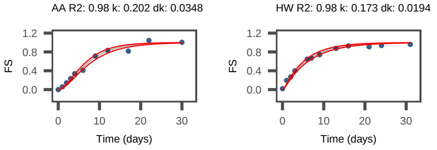

NACAM − QPLLESAPGSVLESPSK_2

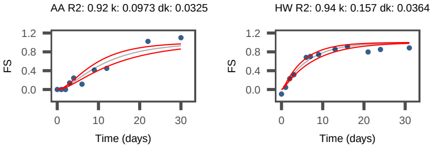

NDKA(Non−Unique) − VMLGETNPADSKPGTIR_3

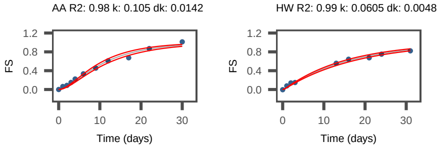

NDRG2 − RPAIFTYHDVGLNYK_3

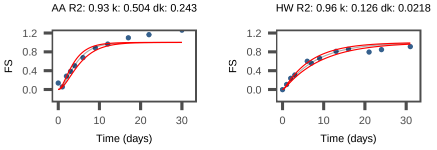

NACAM − TAAPEETSTTPSPQK_2

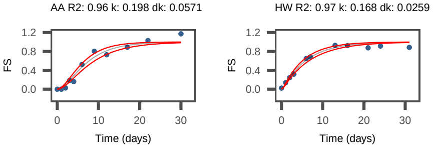

NDKB − DRPFFPGLVK_2

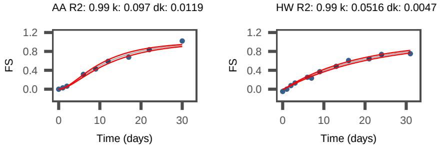

NDRG2 − RPAIFTYHDVGLNYK_4

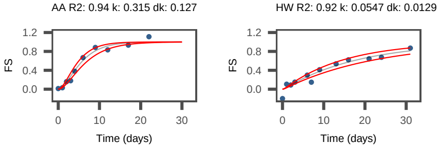

NAR3 − YLYNPDMDNQK_2

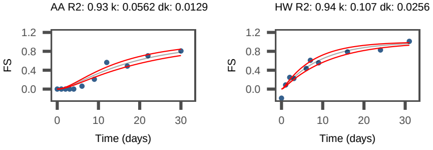

NDKB − DRPFFPGLVK_3

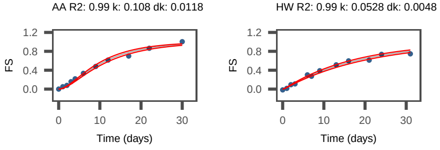

NDRG2 − YALNHPDTVEGLVLINIDPNAK_3

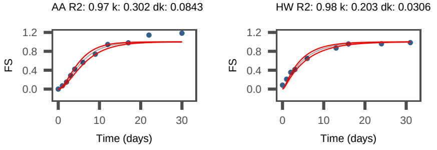

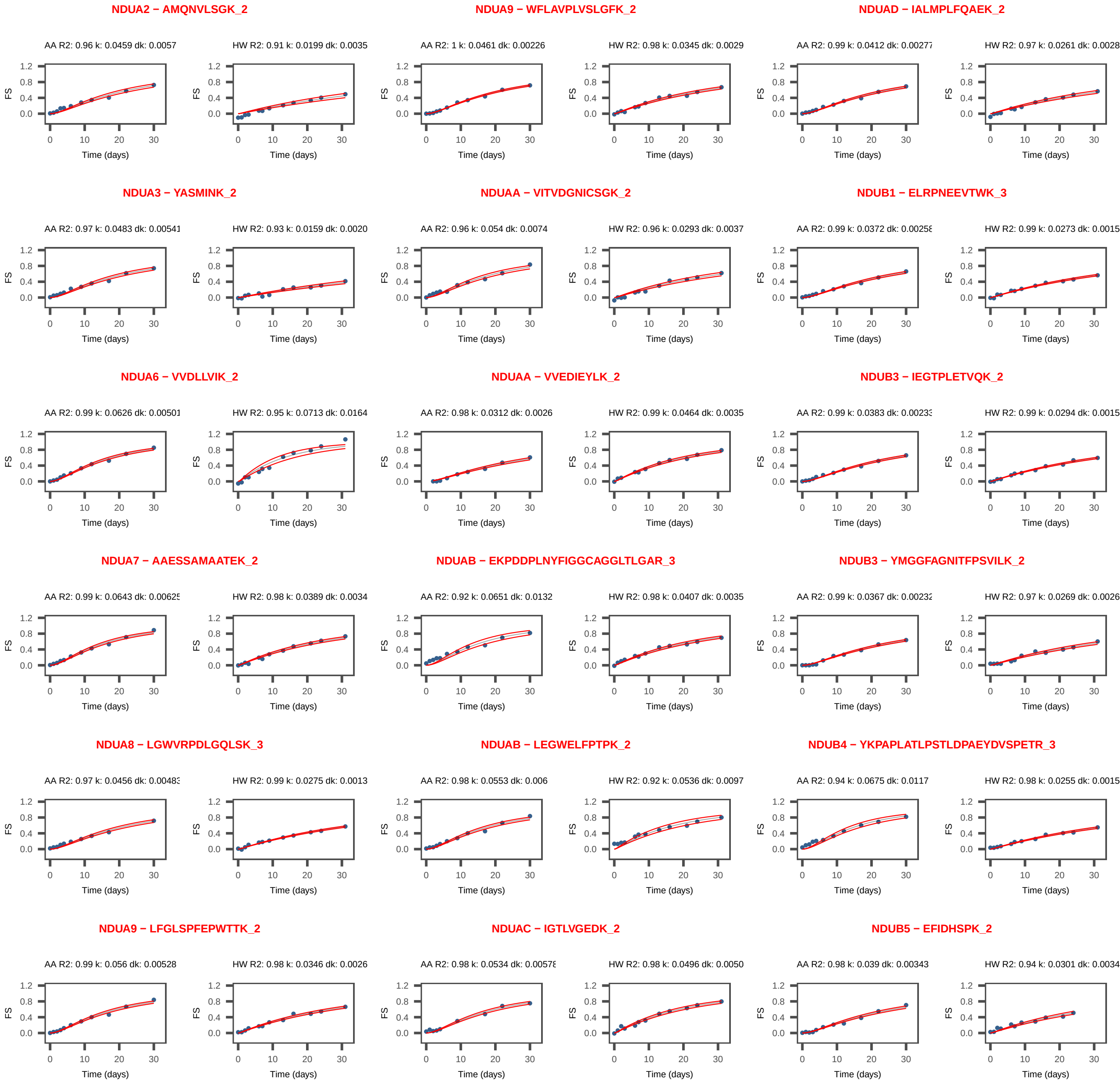
NDUA2 − AMQNVLSGK_2
AA R2: 0.96 k: 0.0459 dk: 0.0057
HW R2: 0.91 k: 0.0199 dk: 0.0035
NDUA9 − WFLAVPLVSLGFK_2
AA R2: 1 k: 0.0461 dk: 0.00226
HW R2: 0.98 k: 0.0345 dk: 0.0029
NDUAD − IALMPLFQAEK_2
AA R2: 0.99 k: 0.0412 dk: 0.00277
HW R2: 0.97 k: 0.0261 dk: 0.0028
NDUA3 − YASMINK_2
AA R2: 0.97 k: 0.0483 dk: 0.00541
HW R2: 0.93 k: 0.0159 dk: 0.0020
NDUAA − VITVDGNICSGK_2
AA R2: 0.96 k: 0.054 dk: 0.0074
HW R2: 0.96 k: 0.0293 dk: 0.0037
NDUB1 − ELRPNEEVTWK_3
AA R2: 0.99 k: 0.0372 dk: 0.00258
HW R2: 0.99 k: 0.0273 dk: 0.0015
NDUA6 − VVDLLVIK_2
AA R2: 0.99 k: 0.0626 dk: 0.00501
HW R2: 0.95 k: 0.0713 dk: 0.0164
NDUAA − VVEDIEYLK_2
AA R2: 0.98 k: 0.0312 dk: 0.0026
HW R2: 0.99 k: 0.0464 dk: 0.0035
NDUB3 − IEGTPLETVQK_2
AA R2: 0.99 k: 0.0383 dk: 0.00233
HW R2: 0.99 k: 0.0294 dk: 0.0015
NDUA7 − AAESSAMAATEK_2
AA R2: 0.99 k: 0.0643 dk: 0.00625
HW R2: 0.98 k: 0.0389 dk: 0.0034
NDUAB − EKPDDPLNYFIGGCAGGLTLGAR_3
AA R2: 0.92 k: 0.0651 dk: 0.0132
HW R2: 0.98 k: 0.0407 dk: 0.0035
NDUB3 − YMGGFAGNITFPSVILK_2
AA R2: 0.99 k: 0.0367 dk: 0.00232
HW R2: 0.97 k: 0.0269 dk: 0.0026
NDUA8 − LGWVRPDLGQLSK_3
AA R2: 0.97 k: 0.0456 dk: 0.00483
HW R2: 0.99 k: 0.0275 dk: 0.0013
NDUAB − LEGWELFPTPK_2
AA R2: 0.98 k: 0.0553 dk: 0.006
HW R2: 0.92 k: 0.0536 dk: 0.0097
NDUB4 − YKPAPLATLPSTLDPAEYDVSPETR_3
AA R2: 0.94 k: 0.0675 dk: 0.0117
HW R2: 0.98 k: 0.0255 dk: 0.0015
NDUA9 − LFGLSPFEPWTTK_2
AA R2: 0.99 k: 0.056 dk: 0.00528
HW R2: 0.98 k: 0.0346 dk: 0.0026
NDUAC − IGTLVGEDK_2
AA R2: 0.98 k: 0.0534 dk: 0.00578
HW R2: 0.98 k: 0.0496 dk: 0.0050
NDUB5 − EFIDHSPK_2
AA R2: 0.98 k: 0.039 dk: 0.00343
HW R2: 0.94 k: 0.0301 dk: 0.0034
FS
Time (days)

NDUB5 − LFVVKPSLYYDAR_3

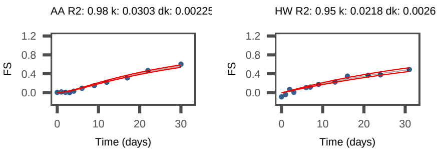

NDUBB − QREPTMQWQEDPEPEDENVYAK_3

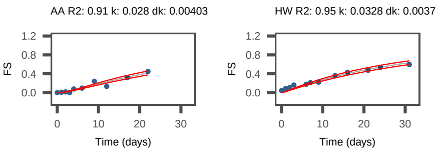

NDUS1 − LVNQEVLADPLVPPQLTIK_2

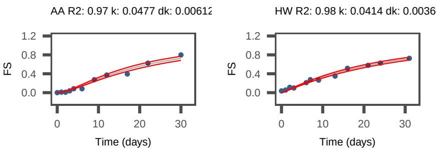

NDUB5 − NFYDGPEK_2

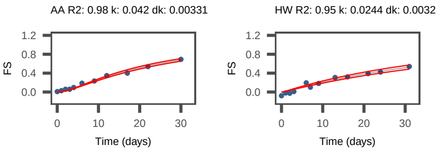

NDUF4 − ITVVEALTLLNNHK_3

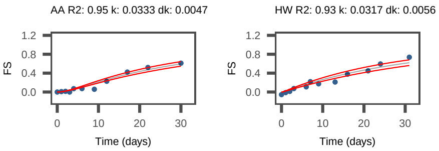

NDUS1 − LVNQEVLADPLVPPQLTIK_3

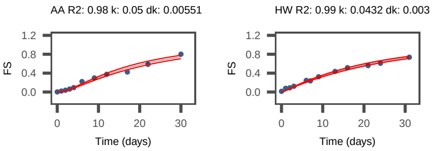

NDUB7 − HEQHDWDYCEHLDYVK_4

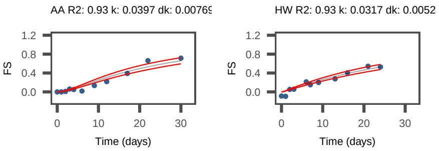

NDUS1 − DDGAAILVAVSNMVQK_3

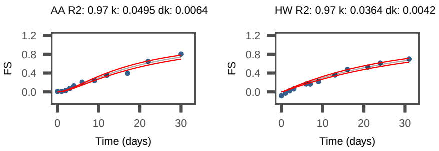

NDUS1 − MCLVEIEK_2

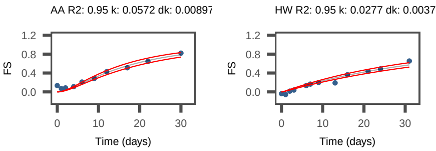

NDUB9 − AMYPDYFSK_2

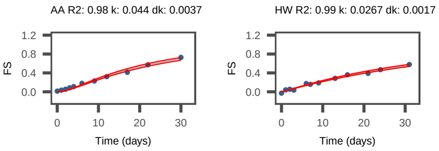

NDUS1 − GNDMQVGTYIEK_2

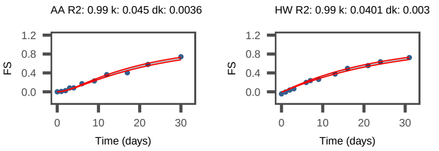

NDUS1 − MFMSELSGNVIDICPVGALTSK_2

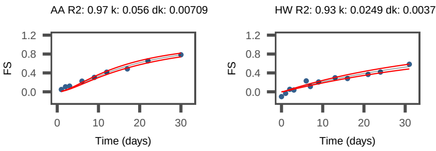

NDUB9 − VPEWCLDYWHPSEK_3

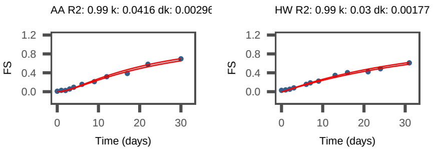

NDUS1 − IASQVAALDLGYKPGVEAIR_3

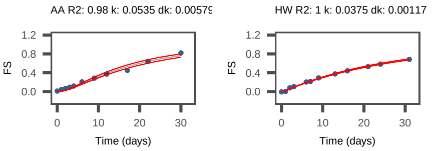

NDUS1 − MHEDINEEWISDK_3

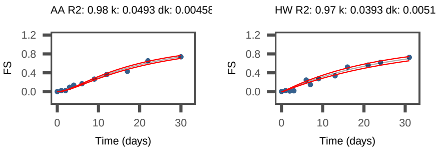

NDUBA − TPAPSPQTSLPNPITYLTK_2

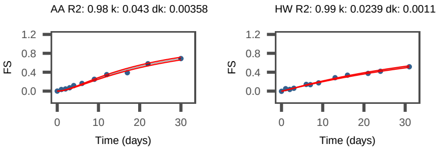

NDUS1 − KPMVVLGSSALQR_3

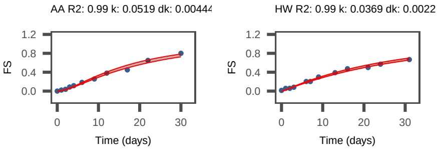

NDUS1 − YDHLGDSPK_2

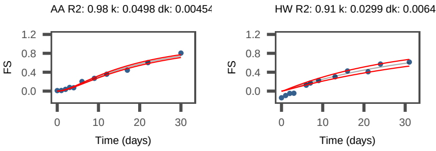

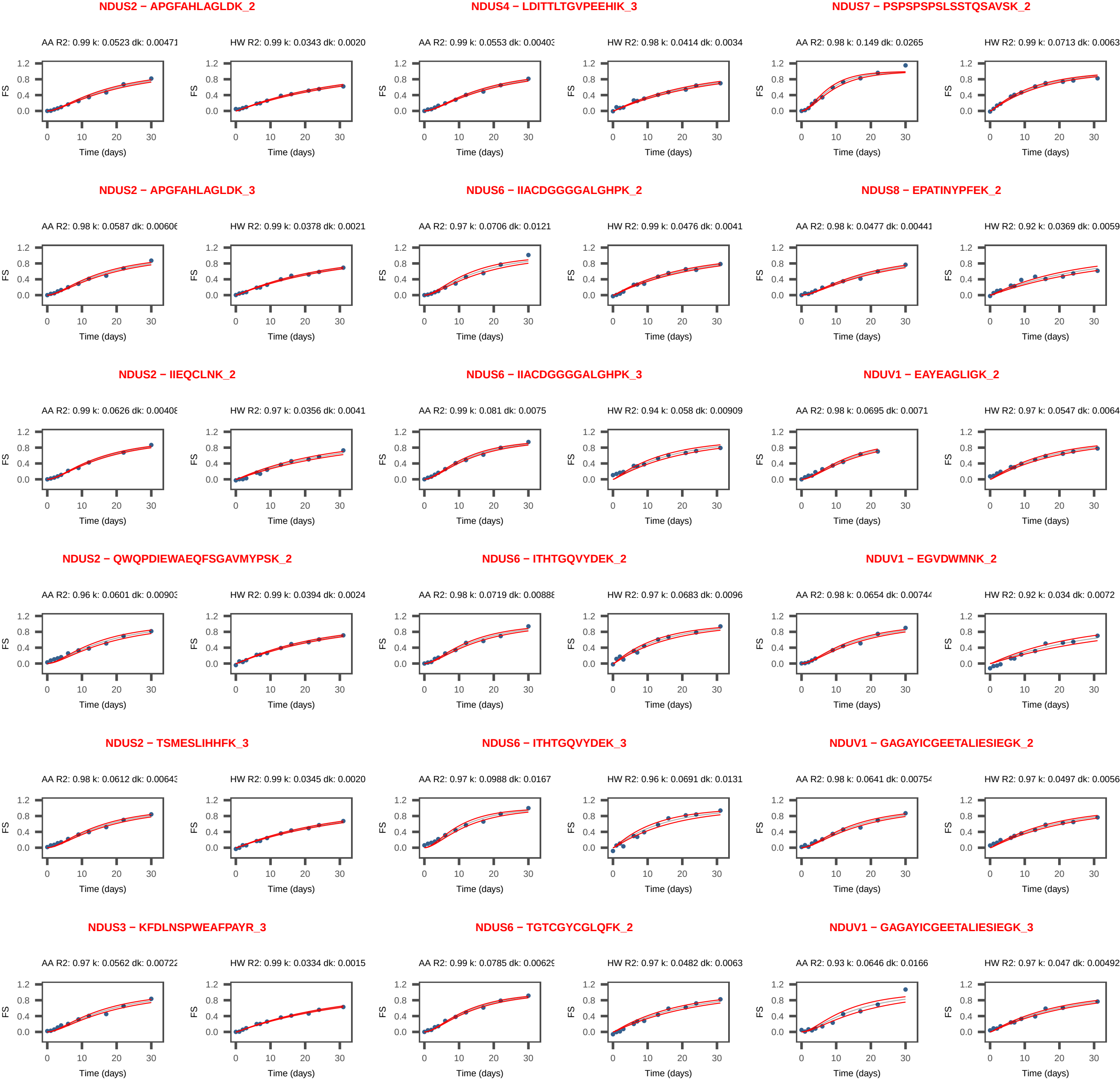
NDUS2 − APGFAHLAGLDK_2
AA R2: 0.99 k: 0.0523 dk: 0.00471
HW R2: 0.99 k: 0.0343 dk: 0.0020
NDUS4 − LDITTLTGVPEEHIK_3
AA R2: 0.99 k: 0.0553 dk: 0.00403
HW R2: 0.98 k: 0.0414 dk: 0.0034
NDUS7 − PSPSPSPSLSSTQSAVSK_2
AA R2: 0.98 k: 0.149 dk: 0.0265
HW R2: 0.99 k: 0.0713 dk: 0.0063
NDUS2 − APGFAHLAGLDK_3
AA R2: 0.98 k: 0.0587 dk: 0.00606
HW R2: 0.99 k: 0.0378 dk: 0.0021
NDUS6 − IIACDGGGGALGHPK_2
AA R2: 0.97 k: 0.0706 dk: 0.0121
HW R2: 0.99 k: 0.0476 dk: 0.0041
NDUS8 − EPATINYPFEK_2
AA R2: 0.98 k: 0.0477 dk: 0.00441
HW R2: 0.92 k: 0.0369 dk: 0.0059
NDUS2 − IIEQCLNK_2
AA R2: 0.99 k: 0.0626 dk: 0.00408
HW R2: 0.97 k: 0.0356 dk: 0.0041
NDUS6 − IIACDGGGGALGHPK_3
AA R2: 0.99 k: 0.081 dk: 0.0075
HW R2: 0.94 k: 0.058 dk: 0.00909
NDUV1 − EAYEAGLIGK_2
AA R2: 0.98 k: 0.0695 dk: 0.0071
HW R2: 0.97 k: 0.0547 dk: 0.0064
NDUS2 − QWQPDIEWAEQFSGAVMYPSK_2
AA R2: 0.96 k: 0.0601 dk: 0.00903
HW R2: 0.99 k: 0.0394 dk: 0.0024
NDUS6 − ITHTGQVYDEK_2
AA R2: 0.98 k: 0.0719 dk: 0.00888
HW R2: 0.97 k: 0.0683 dk: 0.0096
NDUV1 − EGVDWMNK_2
AA R2: 0.98 k: 0.0654 dk: 0.00744
HW R2: 0.92 k: 0.034 dk: 0.0072
NDUS2 − TSMESLIHHFK_3
AA R2: 0.98 k: 0.0612 dk: 0.00643
HW R2: 0.99 k: 0.0345 dk: 0.0020
NDUS6 − ITHTGQVYDEK_3
AA R2: 0.97 k: 0.0988 dk: 0.0167
HW R2: 0.96 k: 0.0691 dk: 0.0131
NDUV1 − GAGAYICGEETALIESIEGK_2
AA R2: 0.98 k: 0.0641 dk: 0.00754
HW R2: 0.97 k: 0.0497 dk: 0.0056
NDUS3 − KFDLNSPWEAFPAYR_3
AA R2: 0.97 k: 0.0562 dk: 0.00722
HW R2: 0.99 k: 0.0334 dk: 0.0015
NDUS6 − TGTCGYCGLQFK_2
AA R2: 0.99 k: 0.0785 dk: 0.00629
HW R2: 0.97 k: 0.0482 dk: 0.0063
NDUV1 − GAGAYICGEETALIESIEGK_3
AA R2: 0.93 k: 0.0646 dk: 0.0166
HW R2: 0.97 k: 0.047 dk: 0.00492
FS
Time (days)

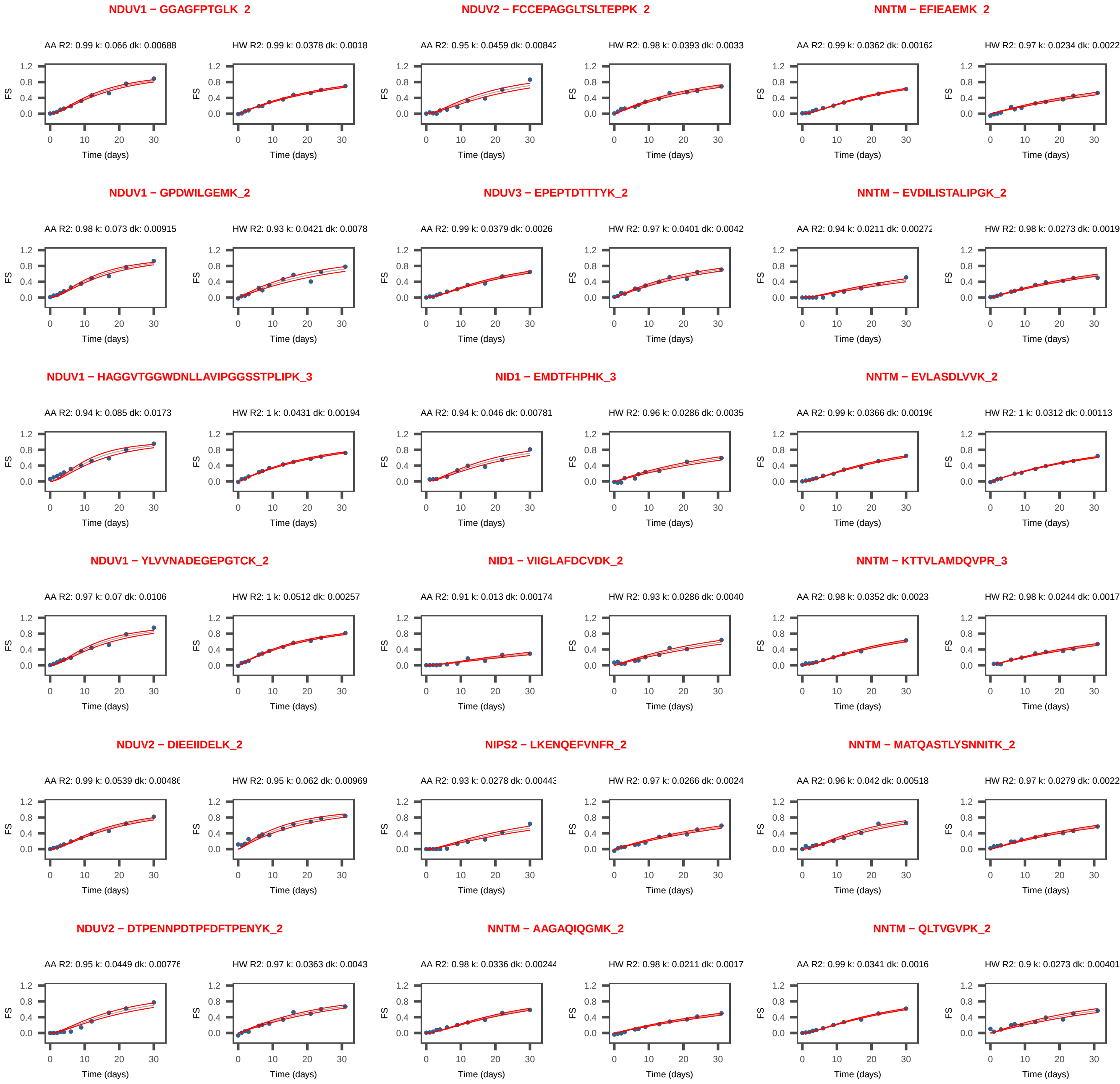
NDUV1 – GGAGFPTGLK_2
AA R2: 0.99 k: 0.066 dk: 0.00688
HW R2: 0.99 k: 0.0378 dk: 0.0018
NDUV2 – FCCEPAGGLTSLTEPPK_2
AA R2: 0.95 k: 0.0459 dk: 0.00842
HW R2: 0.98 k: 0.0393 dk: 0.0033
NNTM – EFIEAEMK_2
AA R2: 0.99 k: 0.0362 dk: 0.00162
HW R2: 0.97 k: 0.0234 dk: 0.0022
NDUV1 – GPDWILGEMK_2
AA R2: 0.98 k: 0.073 dk: 0.00915
HW R2: 0.93 k: 0.0421 dk: 0.0078
NDUV3 – EPEPTDTTTYK_2
AA R2: 0.99 k: 0.0379 dk: 0.0026
HW R2: 0.97 k: 0.0401 dk: 0.0042
NNTM – EVDILISTALIPGK_2
AA R2: 0.94 k: 0.0211 dk: 0.00272
HW R2: 0.98 k: 0.0273 dk: 0.0019
NDUV1 – HAGGVTGGWDNLLAVIPGGSSTPLIPK_3
AA R2: 0.94 k: 0.085 dk: 0.0173
HW R2: 1 k: 0.0431 dk: 0.00194
NID1 – EMDTFHPHK_3
AA R2: 0.94 k: 0.046 dk: 0.00781
HW R2: 0.96 k: 0.0286 dk: 0.0035
NNTM – EVLASDLVVK_2
AA R2: 0.99 k: 0.0366 dk: 0.0019
HW R2: 1 k: 0.0312 dk: 0.00113
NDUV1 – YLVVNADEGEPGTCK_2
AA R2: 0.97 k: 0.07 dk: 0.0106
HW R2: 1 k: 0.0512 dk: 0.00257
NID1 – VIIGLAFDCVDK_2
AA R2: 0.91 k: 0.013 dk: 0.00174
HW R2: 0.93 k: 0.0286 dk: 0.0040
NNTM – KTTVLAMDQVPR_3
AA R2: 0.98 k: 0.0352 dk: 0.0023
HW R2: 0.98 k: 0.0244 dk: 0.0017
NDUV2 – DIEEIIDELK_2
AA R2: 0.99 k: 0.0539 dk: 0.0048
HW R2: 0.95 k: 0.062 dk: 0.00969
NIPS2 – LKENQEFVNFR_2
AA R2: 0.93 k: 0.0278 dk: 0.0044
HW R2: 0.97 k: 0.0266 dk: 0.0024
NNTM – MATQASTLYSNNITK_2
AA R2: 0.96 k: 0.042 dk: 0.00518
HW R2: 0.97 k: 0.0279 dk: 0.0022
NDUV2 – DTPENNPDTPFDFTPENYK_2
AA R2: 0.95 k: 0.0449 dk: 0.0077
HW R2: 0.97 k: 0.0363 dk: 0.0043
NNTM – AAGAQIQGMK_2
AA R2: 0.98 k: 0.0336 dk: 0.0024
HW R2: 0.98 k: 0.0211 dk: 0.0017
NNTM – QLTVGVPK_2
AA R2: 0.99 k: 0.0341 dk: 0.0016
HW R2: 0.9 k: 0.0273 dk: 0.00401
FS
1.2
0.8
0.4
0.0
0
10
20
30
Time (days)

NNTM − SLANVILGGYGTTSTAGGK_3

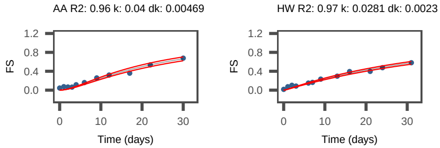

NU1M − YDQLMHLLWK_3

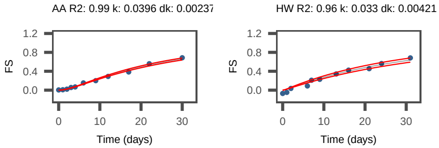

OBSCN − DAALSDAGEVVFSVLGLTSK_2

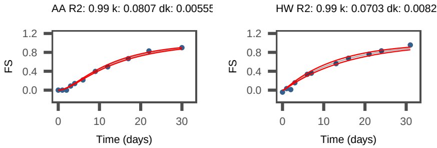

NNTM − TVAELEAEK_2

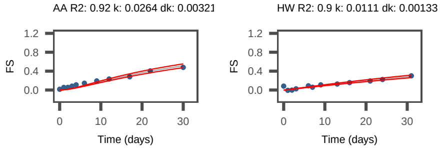

NU4M − AHVEAPIAGSMILAAILLK_3

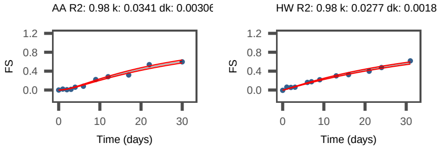

OBSCN − DAALSDAGEVVFSVLGLTSK_3

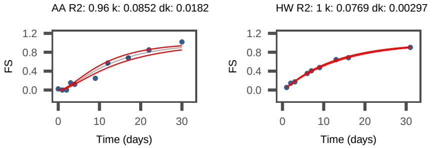

NNTM − VALSPAGVQALVK_2

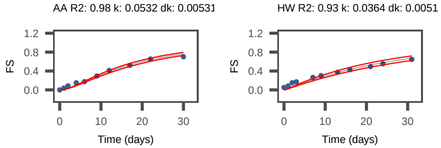

NU4M − ELTLMALHMIPLILLTTSPK_3

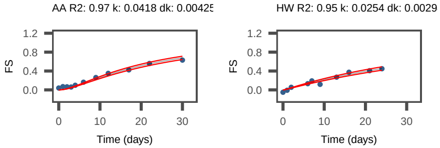

ODB2 − EDILSFLEK_2

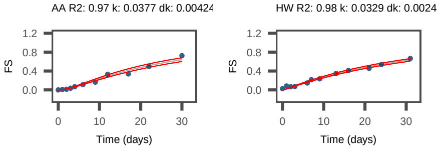

NNTM − VIFPAPTPK_2

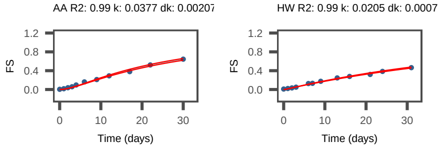

NU5M − TSLTLLDLIWLEK_2

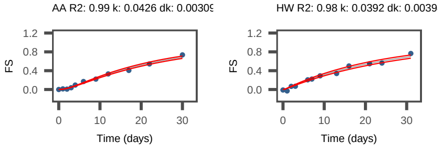

ODB2 − PVILPPEVAIGALGAIK_2

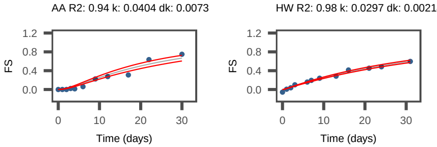

NNTM − VTIAQGYDALSSMANISGYK_2

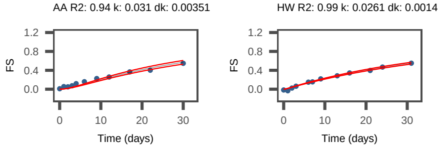

NUDT8 − VWGLTAVITELTLK_2

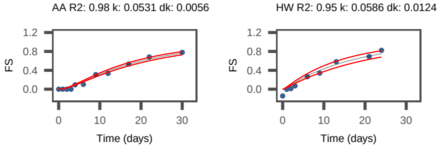

ODB2 − PVILPPEVAIGALGAIK_3

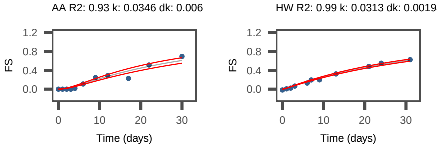

NU1M − GPNIVGPYGILQPFADAMK_2

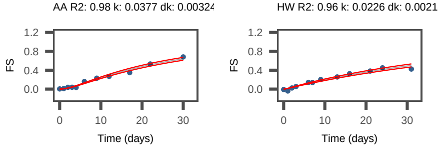

OAT − AFYNNVLGEYEEYITK_2

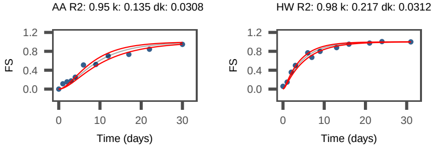

ODBB − MNLFQSITSALDNSLAK_3

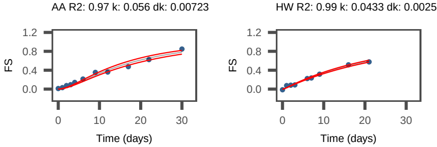

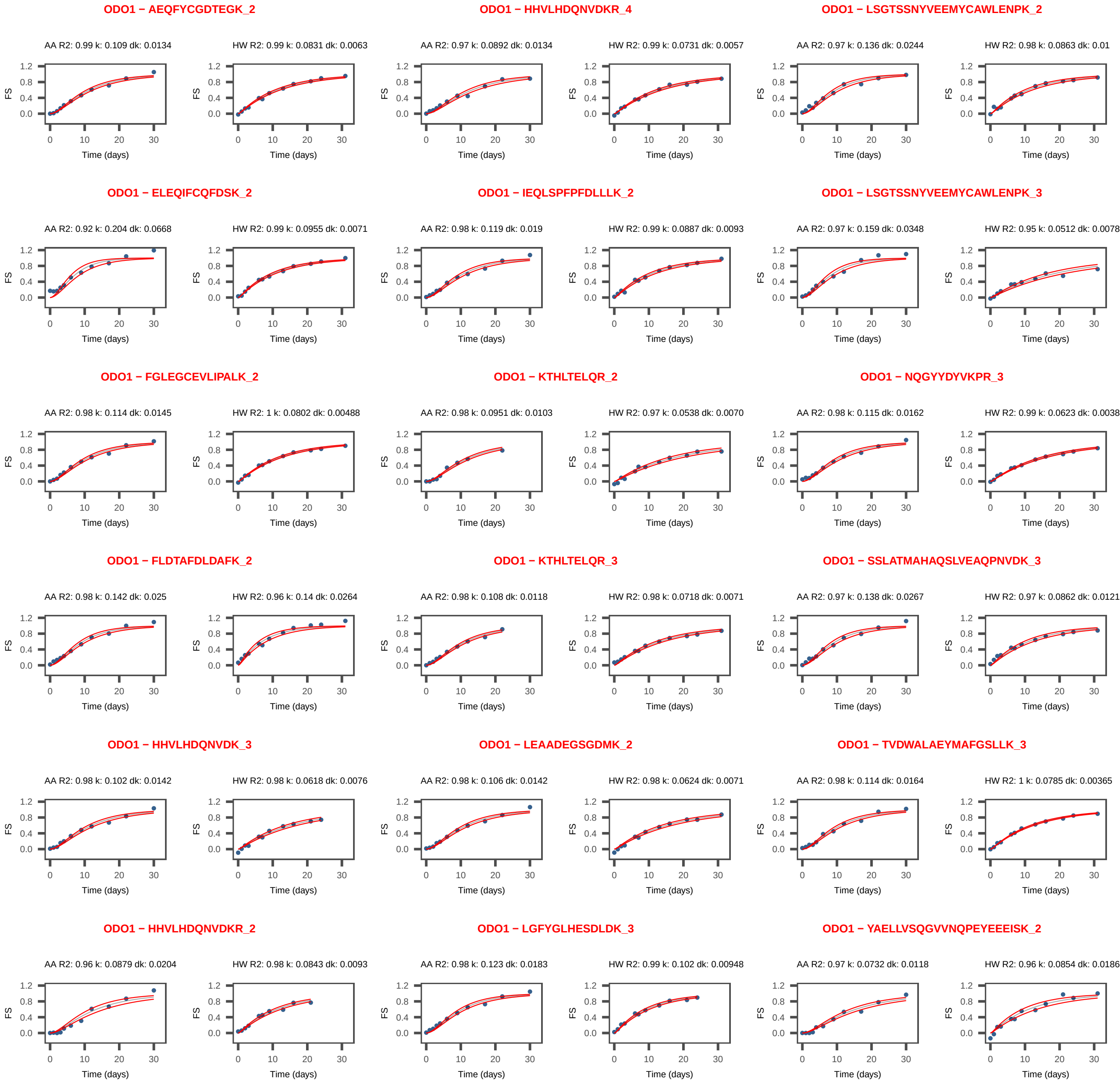
ODO1 − AEQFYCGDTEGK_2
AA R2: 0.99 k: 0.109 dk: 0.0134
HW R2: 0.99 k: 0.0831 dk: 0.0063
ODO1 − HHVLHDQNVDKR_4
AA R2: 0.97 k: 0.0892 dk: 0.0134
HW R2: 0.99 k: 0.0731 dk: 0.0057
ODO1 − LSGTSSNYVEEMYCAWLENPK_2
AA R2: 0.97 k: 0.136 dk: 0.0244
HW R2: 0.98 k: 0.0863 dk: 0.01
ODO1 − ELEQIFCQFDSK_2
AA R2: 0.92 k: 0.204 dk: 0.0668
HW R2: 0.99 k: 0.0955 dk: 0.0071
ODO1 − IEQLSPFPFDLLLK_2
AA R2: 0.98 k: 0.119 dk: 0.019
HW R2: 0.99 k: 0.0887 dk: 0.0093
ODO1 − LSGTSSNYVEEMYCAWLENPK_3
AA R2: 0.97 k: 0.159 dk: 0.0348
HW R2: 0.95 k: 0.0512 dk: 0.0078
ODO1 − FGLEGCEVLIPALK_2
AA R2: 0.98 k: 0.114 dk: 0.0145
HW R2: 1 k: 0.0802 dk: 0.00488
ODO1 − KTHLTELQR_2
AA R2: 0.98 k: 0.0951 dk: 0.0103
HW R2: 0.97 k: 0.0538 dk: 0.0070
ODO1 − NQGYYDYVKPR_3
AA R2: 0.98 k: 0.115 dk: 0.0162
HW R2: 0.99 k: 0.0623 dk: 0.0038
ODO1 − FLDTAFDLDAFK_2
AA R2: 0.98 k: 0.142 dk: 0.025
HW R2: 0.96 k: 0.14 dk: 0.0264
ODO1 − KTHLTELQR_3
AA R2: 0.98 k: 0.108 dk: 0.0118
HW R2: 0.98 k: 0.0718 dk: 0.0071
ODO1 − SSLATMAHAQSLVEAQPNVDK_3
AA R2: 0.97 k: 0.138 dk: 0.0267
HW R2: 0.97 k: 0.0862 dk: 0.0121
ODO1 − HHVLHDQNVDK_3
AA R2: 0.98 k: 0.102 dk: 0.0142
HW R2: 0.98 k: 0.0618 dk: 0.0076
ODO1 − LEAADEGSGDMK_2
AA R2: 0.98 k: 0.106 dk: 0.0142
HW R2: 0.98 k: 0.0624 dk: 0.0071
ODO1 − TVDWALAEYMAFGSLLK_3
AA R2: 0.98 k: 0.114 dk: 0.0164
HW R2: 1 k: 0.0785 dk: 0.00365
ODO1 − HHVLHDQNVDKR_2
AA R2: 0.96 k: 0.0879 dk: 0.0204
HW R2: 0.98 k: 0.0843 dk: 0.0093
ODO1 − LGFYGLHESDLDK_3
AA R2: 0.98 k: 0.123 dk: 0.0183
HW R2: 0.99 k: 0.102 dk: 0.00948
ODO1 − YAELLVSQGVVNQPEYEEEISK_2
AA R2: 0.97 k: 0.0732 dk: 0.0118
HW R2: 0.96 k: 0.0854 dk: 0.0186
FS
Time (days)

ODO1 – YPNAELAWCQEEHK_3

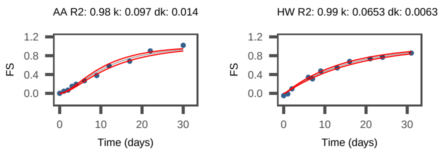

ODPB – AAWYGHCPGLK_3

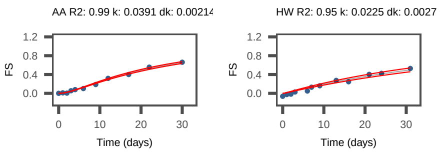

ODPX – STVPHAYATADCDLGAVLK_3

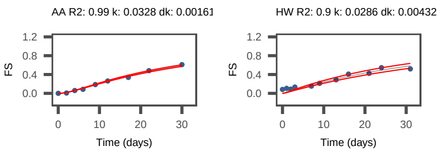

ODP2 – DVPLGAPLCIIVEK_2

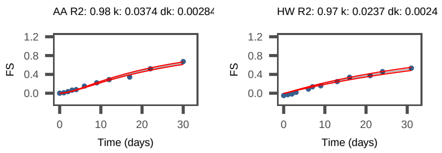

ODPB – DIIFAVK_2

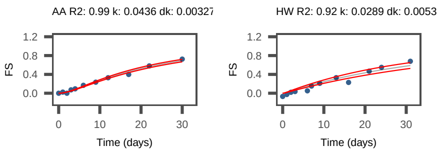

ODPX – VLMPSLSPTMEQGNIVK_2

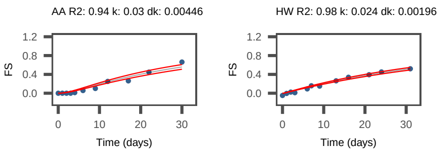

ODP2 – GLETIASDVVSLASK_3

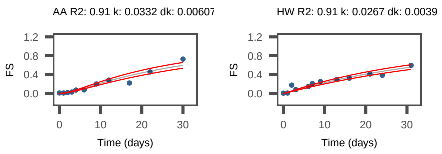

ODPB – EAINQGMDEELERDEK_3

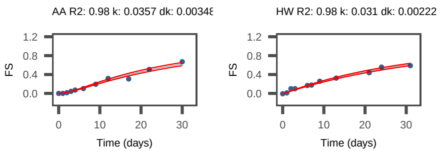

OLA1 – IPAFLNVVDIAGLVK_2

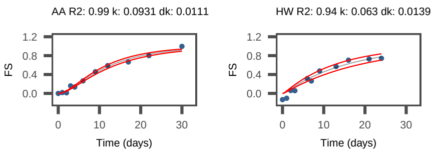

ODP2 – LQPHEFQGGTFTISNLGMFGIK_3

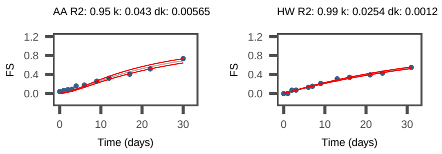

ODPB – FSMQAIDQVINSAAK_3

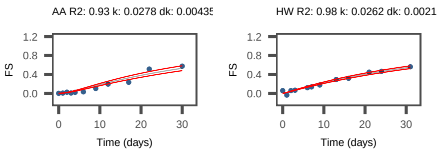

OPA1 – NEILDEVISLSQVTPK_3

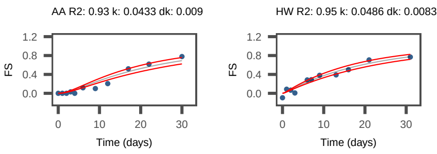

ODP2 – VVDGAVGAQWLAEFK_3

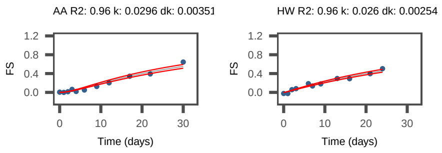

ODPB – VFLLGEEVAQYDGAYK_3

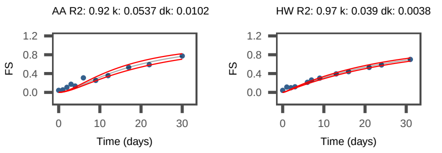

OPA1 – VTLSEGPHHVALFK_4

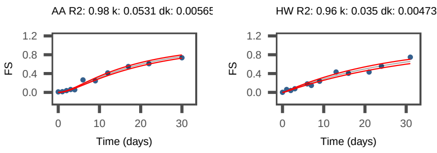

ODP2 – YLEKPITMLL_2

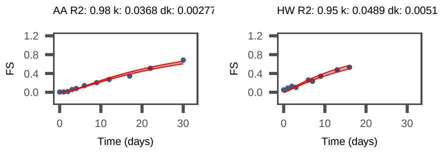

ODPB – VLEDNSVPQVK_2

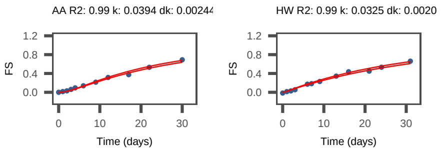

OTUB1 – LELSVLYK_2

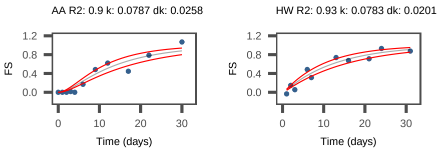

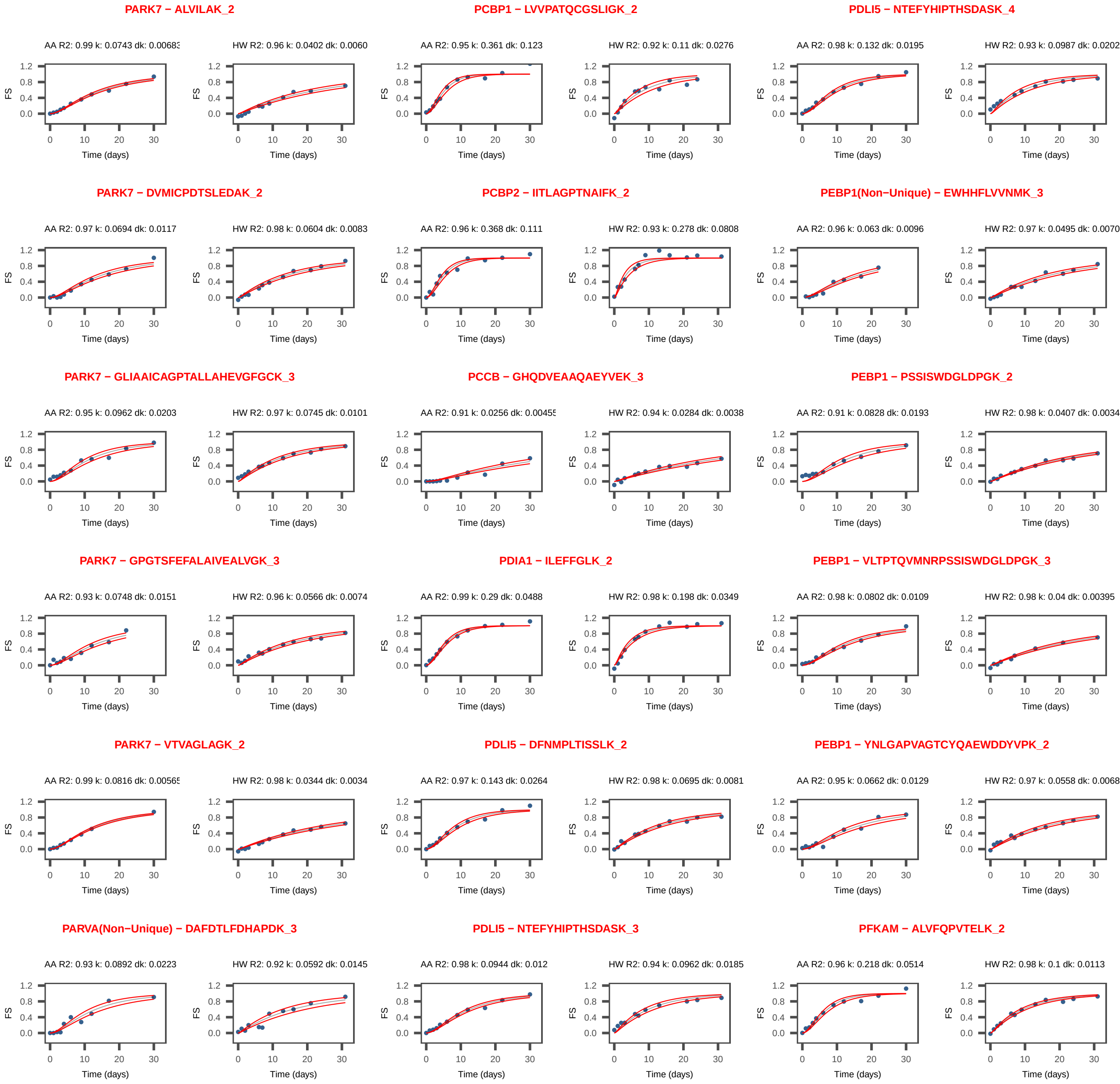
PARK7 − ALVILAK_2
AA R2: 0.99 k: 0.0743 dk: 0.00683
HW R2: 0.96 k: 0.0402 dk: 0.0060
PCBP1 − LVVPATQCGSLIGK_2
AA R2: 0.95 k: 0.361 dk: 0.123
HW R2: 0.92 k: 0.11 dk: 0.0276
PDLI5 − NTEFYHIPTHSDASK_4
AA R2: 0.98 k: 0.132 dk: 0.0195
HW R2: 0.93 k: 0.0987 dk: 0.0202
PARK7 − DVMICPDTSLEDAK_2
AA R2: 0.97 k: 0.0694 dk: 0.0117
HW R2: 0.98 k: 0.0604 dk: 0.0083
PCBP2 − IITLAGPTNAIFK_2
AA R2: 0.96 k: 0.368 dk: 0.111
HW R2: 0.93 k: 0.278 dk: 0.0808
PEBP1(Non−Unique) − EWHHFLVVNMK_3
AA R2: 0.96 k: 0.063 dk: 0.0096
HW R2: 0.97 k: 0.0495 dk: 0.0070
PARK7 − GLIAAICAGPTALLAHEVGFGCK_3
AA R2: 0.95 k: 0.0962 dk: 0.0203
HW R2: 0.97 k: 0.0745 dk: 0.0101
PCCB − GHQDVEAAQAEYVEK_3
AA R2: 0.91 k: 0.0256 dk: 0.00455
HW R2: 0.94 k: 0.0284 dk: 0.0038
PEBP1 − PSSISWDGLDPGK_2
AA R2: 0.91 k: 0.0828 dk: 0.0193
HW R2: 0.98 k: 0.0407 dk: 0.0034
PARK7 − GPGTSFEFALAIVEALVGK_3
AA R2: 0.93 k: 0.0748 dk: 0.0151
HW R2: 0.96 k: 0.0566 dk: 0.0074
PDIA1 − ILEFFGLK_2
AA R2: 0.99 k: 0.29 dk: 0.0488
HW R2: 0.98 k: 0.198 dk: 0.0349
PEBP1 − VLTPTQVMNRPSSISWDGLDPGK_3
AA R2: 0.98 k: 0.0802 dk: 0.0109
HW R2: 0.98 k: 0.04 dk: 0.00395
PARK7 − VTVAGLAGK_2
AA R2: 0.99 k: 0.0816 dk: 0.00565
HW R2: 0.98 k: 0.0344 dk: 0.0034
PDLI5 − DFNMPLTISSLK_2
AA R2: 0.97 k: 0.143 dk: 0.0264
HW R2: 0.98 k: 0.0695 dk: 0.0081
PEBP1 − YNLGAPVAGTCYQAEWDDYVPK_2
AA R2: 0.95 k: 0.0662 dk: 0.0129
HW R2: 0.97 k: 0.0558 dk: 0.0068
PARVA(Non−Unique) − DAFDTLFDHAPDK_3
AA R2: 0.93 k: 0.0892 dk: 0.0223
HW R2: 0.92 k: 0.0592 dk: 0.0145
PDLI5 − NTEFYHIPTHSDASK_3
AA R2: 0.98 k: 0.0944 dk: 0.012
HW R2: 0.94 k: 0.0962 dk: 0.0185
PFKAM − ALVFQPVTELK_2
AA R2: 0.96 k: 0.218 dk: 0.0514
HW R2: 0.98 k: 0.1 dk: 0.0113
FS
Time (days)

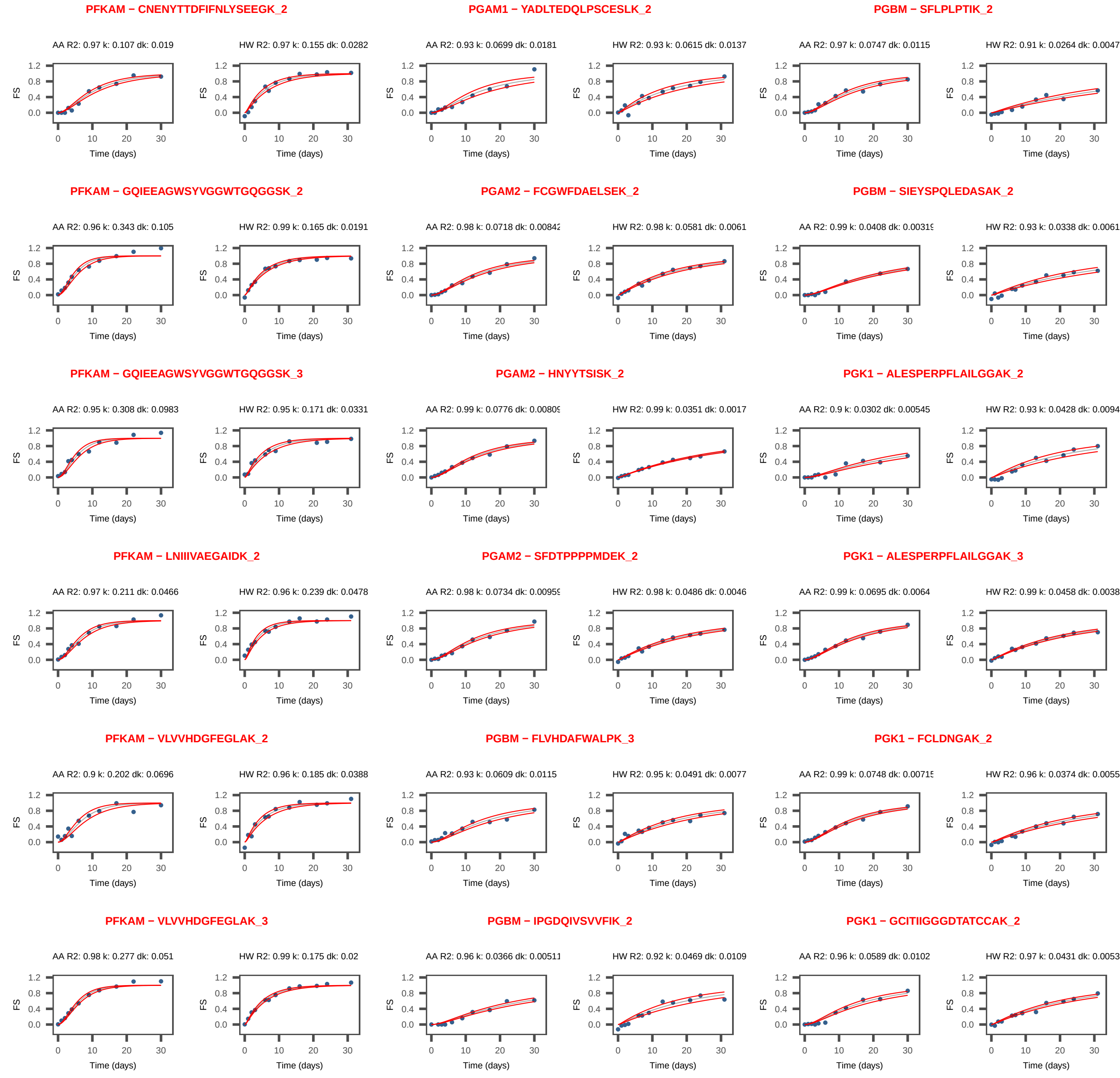
PFKAM − CNENYTTDFIFNLYSEEGK_2
AA R2: 0.97 k: 0.107 dk: 0.019
HW R2: 0.97 k: 0.155 dk: 0.0282
PGAM1 − YADLTEDQLPSCESLK_2
AA R2: 0.93 k: 0.0699 dk: 0.0181
HW R2: 0.93 k: 0.0615 dk: 0.0137
PGBM − SFLPLPTIK_2
AA R2: 0.97 k: 0.0747 dk: 0.0115
HW R2: 0.91 k: 0.0264 dk: 0.0047
PFKAM − GQIEEAGWSYVGGWTGQGGSK_2
AA R2: 0.96 k: 0.343 dk: 0.105
HW R2: 0.99 k: 0.165 dk: 0.0191
PGAM2 − FCGWFDAELSEK_2
AA R2: 0.98 k: 0.0718 dk: 0.00842
HW R2: 0.98 k: 0.0581 dk: 0.0061
PGBM − SIEYSPQLEDASAK_2
AA R2: 0.99 k: 0.0408 dk: 0.00319
HW R2: 0.93 k: 0.0338 dk: 0.0061
PFKAM − GQIEEAGWSYVGGWTGQGGSK_3
AA R2: 0.95 k: 0.308 dk: 0.0983
HW R2: 0.95 k: 0.171 dk: 0.0331
PGAM2 − HNYYTSISK_2
AA R2: 0.99 k: 0.0776 dk: 0.00809
HW R2: 0.99 k: 0.0351 dk: 0.0017
PGK1 − ALESPERPFLAILGGAK_2
AA R2: 0.9 k: 0.0302 dk: 0.00545
HW R2: 0.93 k: 0.0428 dk: 0.0094
PFKAM − LNIIIVAEGAIDK_2
AA R2: 0.97 k: 0.211 dk: 0.0466
HW R2: 0.96 k: 0.239 dk: 0.0478
PGAM2 − SFDTPPPPMDEK_2
AA R2: 0.98 k: 0.0734 dk: 0.00959
HW R2: 0.98 k: 0.0486 dk: 0.0046
PGK1 − ALESPERPFLAILGGAK_3
AA R2: 0.99 k: 0.0695 dk: 0.0064
HW R2: 0.99 k: 0.0458 dk: 0.0038
PFKAM − VLVVHDGFEGLAK_2
AA R2: 0.9 k: 0.202 dk: 0.0696
HW R2: 0.96 k: 0.185 dk: 0.0388
PGBM − FLVHDAFWALPK_3
AA R2: 0.93 k: 0.0609 dk: 0.0115
HW R2: 0.95 k: 0.0491 dk: 0.0077
PGK1 − FCLDNGAK_2
AA R2: 0.99 k: 0.0748 dk: 0.00715
HW R2: 0.96 k: 0.0374 dk: 0.0055
PFKAM − VLVVHDGFEGLAK_3
AA R2: 0.98 k: 0.277 dk: 0.051
HW R2: 0.99 k: 0.175 dk: 0.02
PGBM − IPGDQIVSVVFIK_2
AA R2: 0.96 k: 0.0366 dk: 0.00511
HW R2: 0.92 k: 0.0469 dk: 0.0109
PGK1 − GCITIIGGGDTATCCAK_2
AA R2: 0.96 k: 0.0589 dk: 0.0102
HW R2: 0.97 k: 0.0431 dk: 0.0053
FS
Time (days)

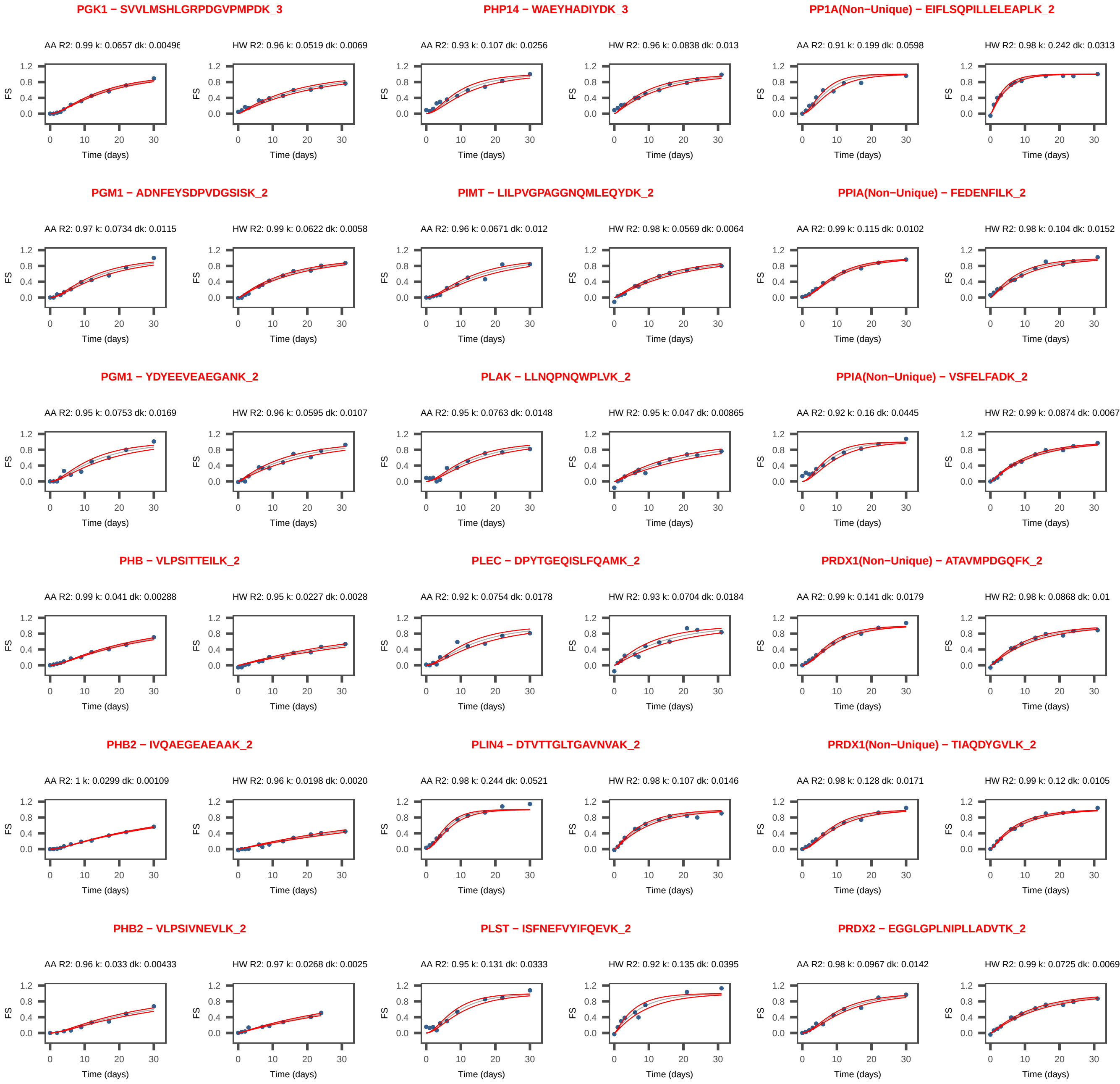
PGK1 − SVVLMSHLGRPDGVPMPDK_3
AA R2: 0.99 k: 0.0657 dk: 0.0049
HW R2: 0.96 k: 0.0519 dk: 0.0069
PHP14 − WAEYHADIYDK_3
AA R2: 0.93 k: 0.107 dk: 0.0256
HW R2: 0.96 k: 0.0838 dk: 0.013
PP1A(Non−Unique) − EIFLSQPILLELEAPLK_2
AA R2: 0.91 k: 0.199 dk: 0.0598
HW R2: 0.98 k: 0.242 dk: 0.0313
PGM1 − ADNFEYSDPVDGSISK_2
AA R2: 0.97 k: 0.0734 dk: 0.0115
HW R2: 0.99 k: 0.0622 dk: 0.0058
PIMT − LILPVGPAGGNQMLEQYDK_2
AA R2: 0.96 k: 0.0671 dk: 0.012
HW R2: 0.98 k: 0.0569 dk: 0.0064
PPIA(Non−Unique) − FEDENFILK_2
AA R2: 0.99 k: 0.115 dk: 0.0102
HW R2: 0.98 k: 0.104 dk: 0.0152
PGM1 − YDYEEVEAEGANK_2
AA R2: 0.95 k: 0.0753 dk: 0.0169
HW R2: 0.96 k: 0.0595 dk: 0.0107
PLAK − LLNQPNQWPLVK_2
AA R2: 0.95 k: 0.0763 dk: 0.0148
HW R2: 0.95 k: 0.047 dk: 0.00865
PPIA(Non−Unique) − VSFELFADK_2
AA R2: 0.92 k: 0.16 dk: 0.0445
HW R2: 0.99 k: 0.0874 dk: 0.0067
PHB − VLPSITTEILK_2
AA R2: 0.99 k: 0.041 dk: 0.00288
HW R2: 0.95 k: 0.0227 dk: 0.0028
PLEC − DPYTGEQISLFQAMK_2
AA R2: 0.92 k: 0.0754 dk: 0.0178
HW R2: 0.93 k: 0.0704 dk: 0.0184
PRDX1(Non−Unique) − ATAVMPDGQFK_2
AA R2: 0.99 k: 0.141 dk: 0.0179
HW R2: 0.98 k: 0.0868 dk: 0.01
PHB2 − IVQAEGEAEAAK_2
AA R2: 1 k: 0.0299 dk: 0.00109
HW R2: 0.96 k: 0.0198 dk: 0.0020
PLIN4 − DTVTTGLTGAVNVAK_2
AA R2: 0.98 k: 0.244 dk: 0.0521
HW R2: 0.98 k: 0.107 dk: 0.0146
PRDX1(Non−Unique) − TIAQDYGVLK_2
AA R2: 0.98 k: 0.128 dk: 0.0171
HW R2: 0.99 k: 0.12 dk: 0.0105
PHB2 − VLPSIVNEVLK_2
AA R2: 0.96 k: 0.033 dk: 0.00433
HW R2: 0.97 k: 0.0268 dk: 0.0025
PLST − ISFNEFVYIFQEVK_2
AA R2: 0.95 k: 0.131 dk: 0.0333
HW R2: 0.92 k: 0.135 dk: 0.0395
PRDX2 − EGGLGPLNIPLLADVTK_2
AA R2: 0.98 k: 0.0967 dk: 0.0142
HW R2: 0.99 k: 0.0725 dk: 0.0069
FS
Time (days)

PRDX2 − GLFIIDAK_2

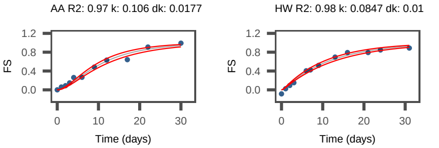

PRDX5 − VNLAELFK_2

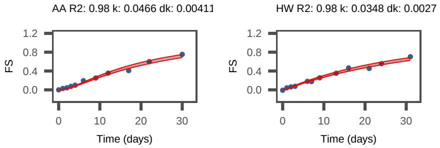

PSA3 − SNFGYNIPLK_2

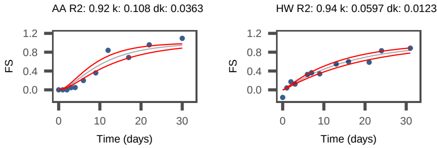

PRDX2 − SLSQNYGVLK_2

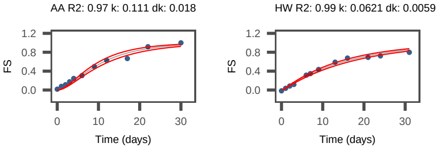

PRDX6 − DLAILLGMLDPVEK_2

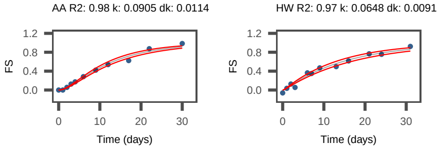

PSA4 − SALALAVK_2

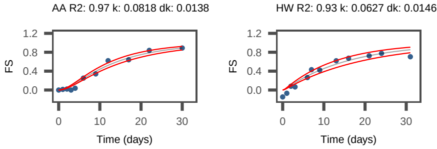

PRDX3 − ELSLDDFK_2

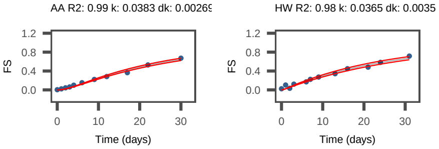

PRDX6 − LPFPIIDDK_2

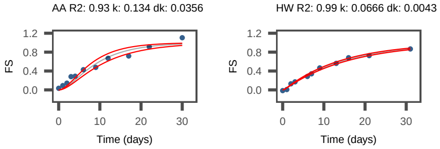

PSD11 − EASIDILHSIVK_3

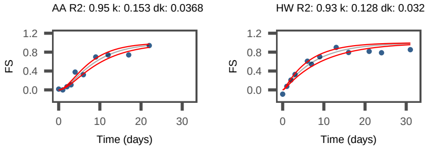

PRDX3 − PAVTQHAPYFK_3

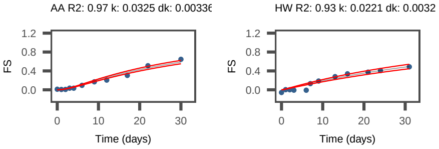

PRDX6 − VVFIFGPDK_2

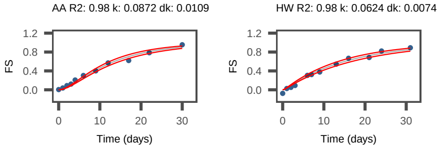

PSD12 − LQEVIETLLSLEK_2

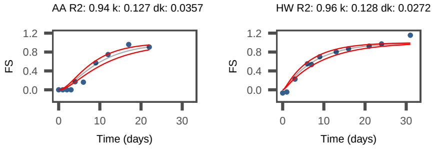

PRDX3 − TPAVTQHAPYFK_3

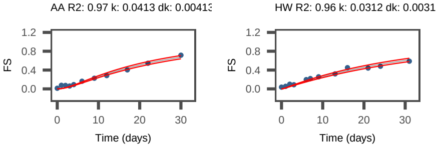

PROF1 − TFVSITPAEVGVLVGK_2

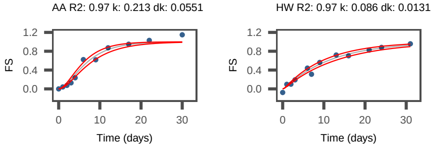

PSMD2 − EDVLTLLLPVMGDSK_2

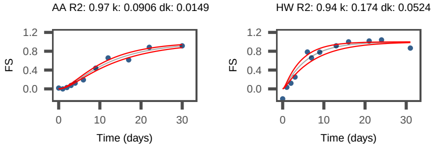

PRDX4(Non−Unique) − GLFIIDDK_2

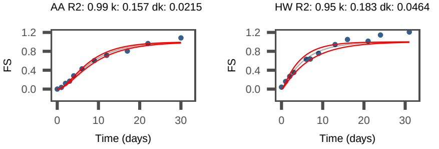

PSA2 − LVQIEYALAAVAGGAPSVGIK_3

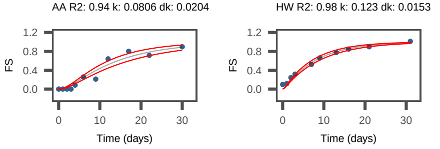

PSMD8 − LVLLELNFLPTTGTK_2

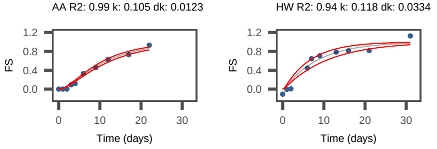

PTGR3 − FIGLESVFQAVDYMYTGK_3

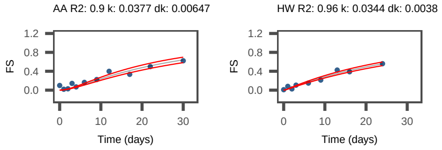

PYGB(Non−Unique) − YGNPWEK_2

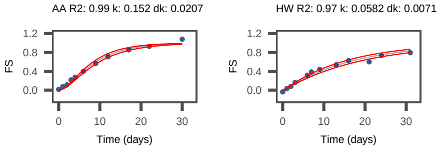

PYGM − QIIEQLSSGFFSPK_2

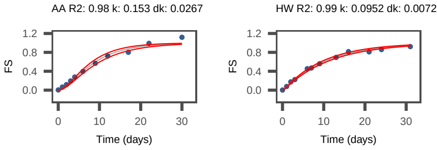

PURA1 − LDILDVLSEIK_2

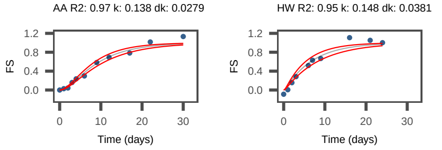

PYGM − DFYELEPHK_2

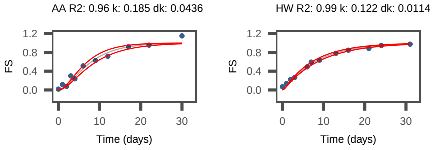

PYGM − SLFDVQVK_2

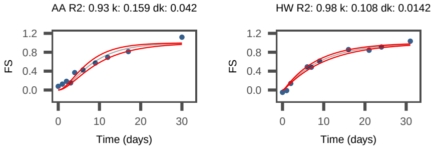

PYGB − INPASMFDVHVK_3

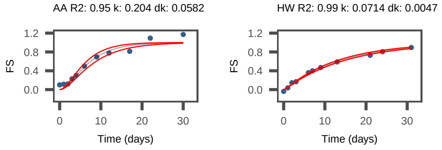

PYGM − DFYELEPHK_3

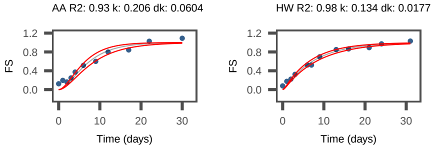

PYGM − TNFDAFPDK_2

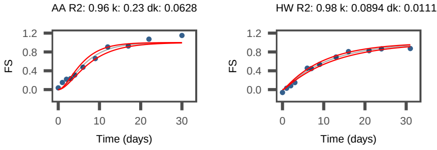

PYGB(Non−Unique) − LKQEYFVVAATLQDIIR_3

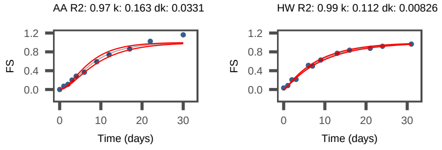

PYGM − GLAGVENVSELK_2

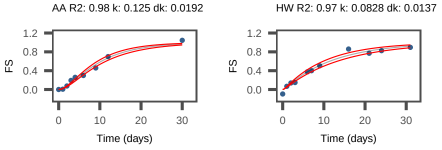

PYGM − VFADYEEYIK_2

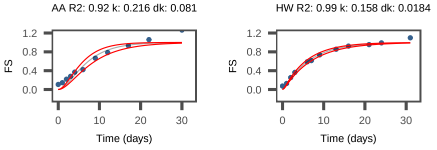

PYGB − QAVDQISSGFFSPK_2

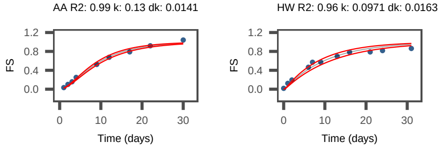

PYGM − IHSEILK_2

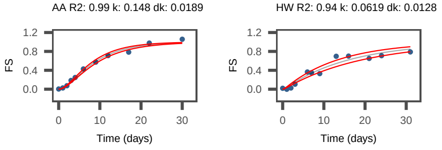

PZP − LSPQSIYNLLPGK_2

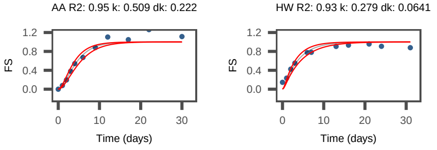

PYGB(Non−Unique) − VLYPNDNFFEGK_2

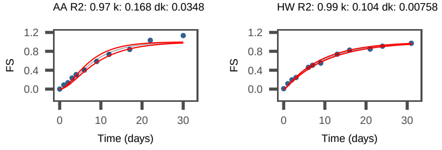

PYGM − MSLVEEGAVK_2

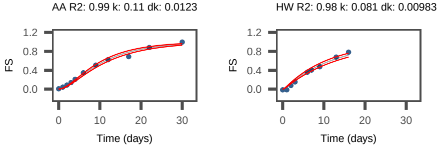

PZP − SVIVEPEGIEK_2

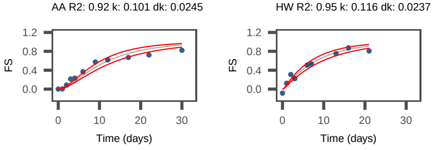

QCR1 − VVELLADIVQNSSLEDSQIEK_2

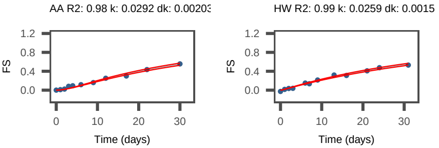

QCR7 − DDTLHETEDVK_2

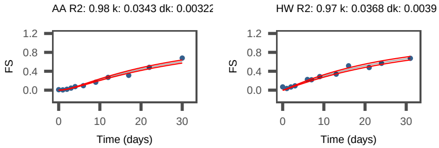

RB11B(Non−Unique) − DHADSNIVIMLVGNK_3

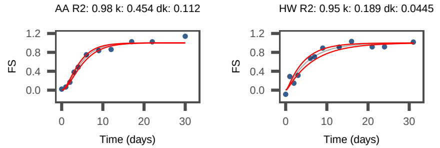

QCR10 − LILDWVPYINGK_2

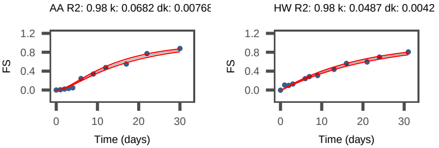

QCR7 − DDTLHETEDVK_3

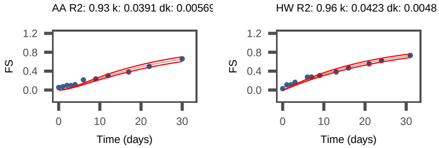

REEP5 − HESQVDSVVK_2

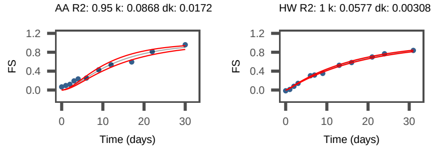

QCR2 − GGLGLAGAK_2

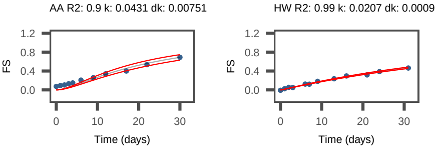

QCR7 − YEEDKFYLEPY_2

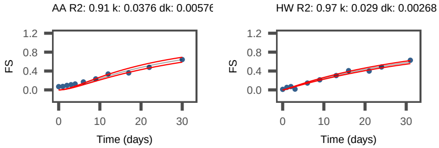

RL15 − FFEVILIDPFHK_3

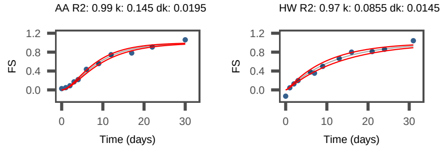

QCR2 − IIENLHDVAYK_3

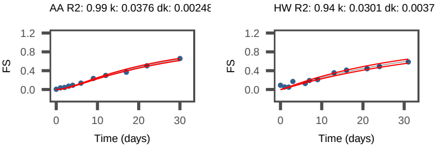

RAB1B − EFADSLGVPFLETSAK_2

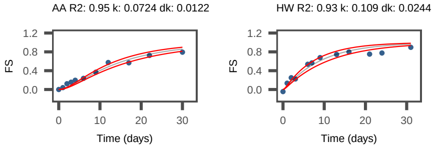

RL23 − ISLGLPVGAVINCADNTGAK_2

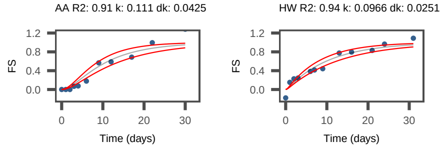

QCR2 − SVLQHLLGAGPHIK_4

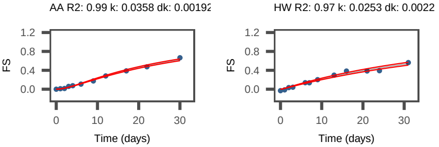

RACK1 − IWDLEGK_2

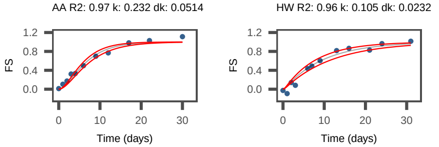

RL4 − NIPGITLLNVSK_2

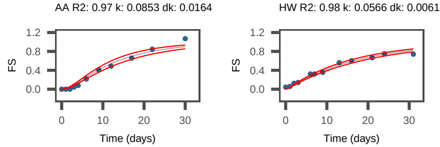

QCR2 − TSAAPGGVPLQPQDLEFTK_2

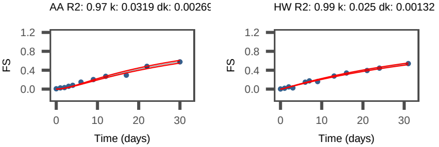

RAP1A(Non−Unique) − SALTVQFVQGIFVEK_2

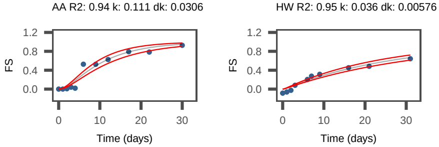

RL7A − AGVNTVTTLVENK_2

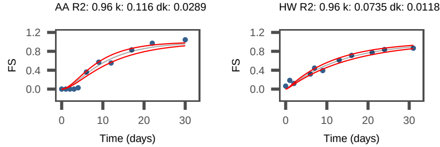

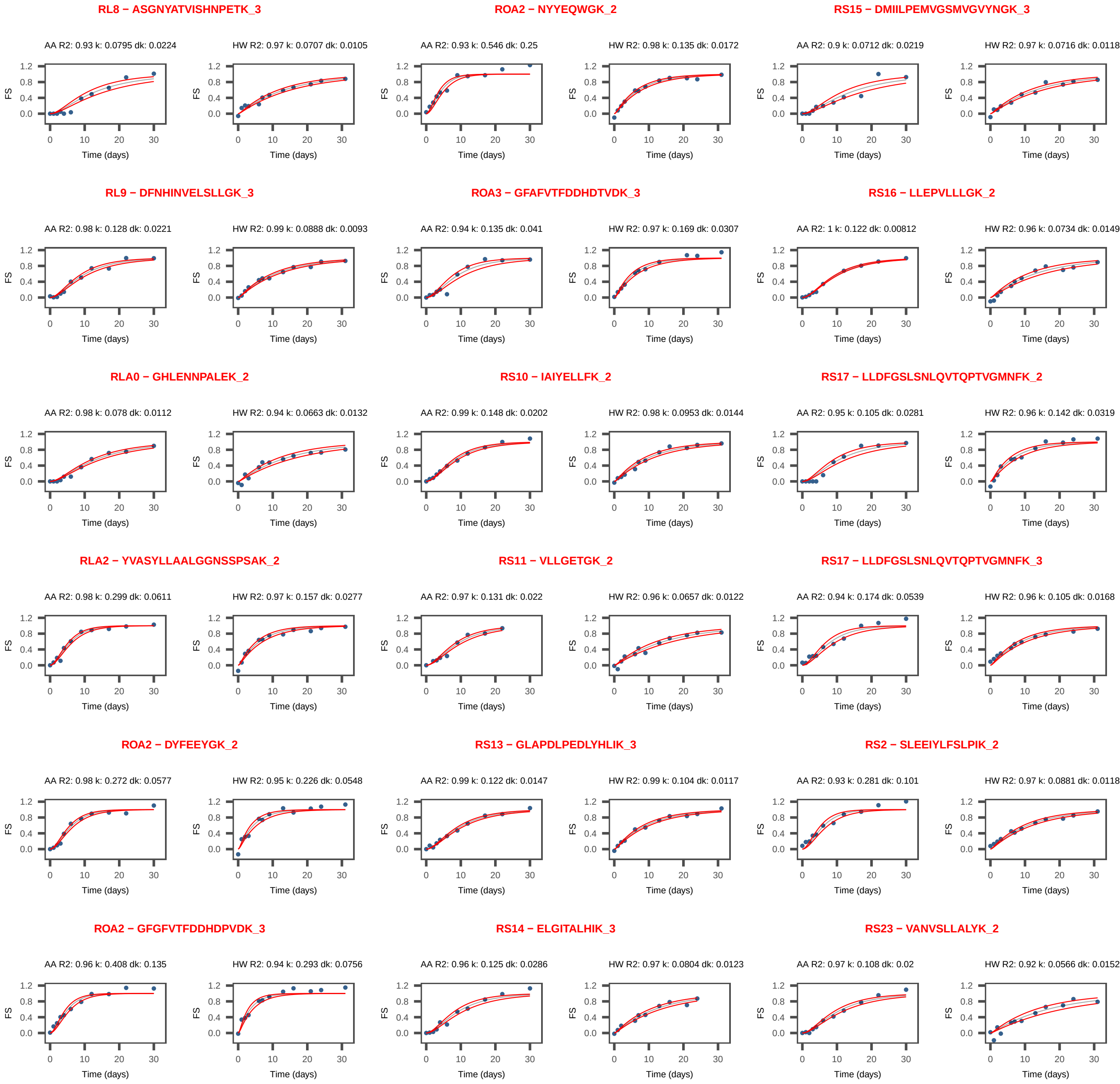
RL8 − ASGNYATVISHNPETK_3
AA R2: 0.93 k: 0.0795 dk: 0.0224
HW R2: 0.97 k: 0.0707 dk: 0.0105
ROA2 − NYYEQWGK_2
AA R2: 0.93 k: 0.546 dk: 0.25
HW R2: 0.98 k: 0.135 dk: 0.0172
RS15 − DMIILPEMVGSMVGVYNGK_3
AA R2: 0.9 k: 0.0712 dk: 0.0219
HW R2: 0.97 k: 0.0716 dk: 0.0118
RL9 − DFNHINVELSLLGK_3
AA R2: 0.98 k: 0.128 dk: 0.0221
HW R2: 0.99 k: 0.0888 dk: 0.0093
ROA3 − GFAFVTFDDHDTVDK_3
AA R2: 0.94 k: 0.135 dk: 0.041
HW R2: 0.97 k: 0.169 dk: 0.0307
RS16 − LLEPVLLLGK_2
AA R2: 1 k: 0.122 dk: 0.00812
HW R2: 0.96 k: 0.0734 dk: 0.0149
RLA0 − GHLENNPALEK_2
AA R2: 0.98 k: 0.078 dk: 0.0112
HW R2: 0.94 k: 0.0663 dk: 0.0132
RS10 − IAIYELLFK_2
AA R2: 0.99 k: 0.148 dk: 0.0202
HW R2: 0.98 k: 0.0953 dk: 0.0144
RS17 − LLDFGSLSNLQVTQPTVGMNFK_2
AA R2: 0.95 k: 0.105 dk: 0.0281
HW R2: 0.96 k: 0.142 dk: 0.0319
RLA2 − YVASYLLAALGGNSSPSAK_2
AA R2: 0.98 k: 0.299 dk: 0.0611
HW R2: 0.97 k: 0.157 dk: 0.0277
RS11 − VLLGETGK_2
AA R2: 0.97 k: 0.131 dk: 0.022
HW R2: 0.96 k: 0.0657 dk: 0.0122
RS17 − LLDFGSLSNLQVTQPTVGMNFK_3
AA R2: 0.94 k: 0.174 dk: 0.0539
HW R2: 0.96 k: 0.105 dk: 0.0168
ROA2 − DYFEEYGK_2
AA R2: 0.98 k: 0.272 dk: 0.0577
HW R2: 0.95 k: 0.226 dk: 0.0548
RS13 − GLAPDLPEDLYHLIK_3
AA R2: 0.99 k: 0.122 dk: 0.0147
HW R2: 0.99 k: 0.104 dk: 0.0117
RS2 − SLEEIYLFSLPIK_2
AA R2: 0.93 k: 0.281 dk: 0.101
HW R2: 0.97 k: 0.0881 dk: 0.0118
ROA2 − GFGFVTFDDHDPVDK_3
AA R2: 0.96 k: 0.408 dk: 0.135
HW R2: 0.94 k: 0.293 dk: 0.0756
RS14 − ELGITALHIK_3
AA R2: 0.96 k: 0.125 dk: 0.0286
HW R2: 0.97 k: 0.0804 dk: 0.0123
RS23 − VANVSLLALYK_2
AA R2: 0.97 k: 0.108 dk: 0.02
HW R2: 0.92 k: 0.0566 dk: 0.0152
FS
Time (days)

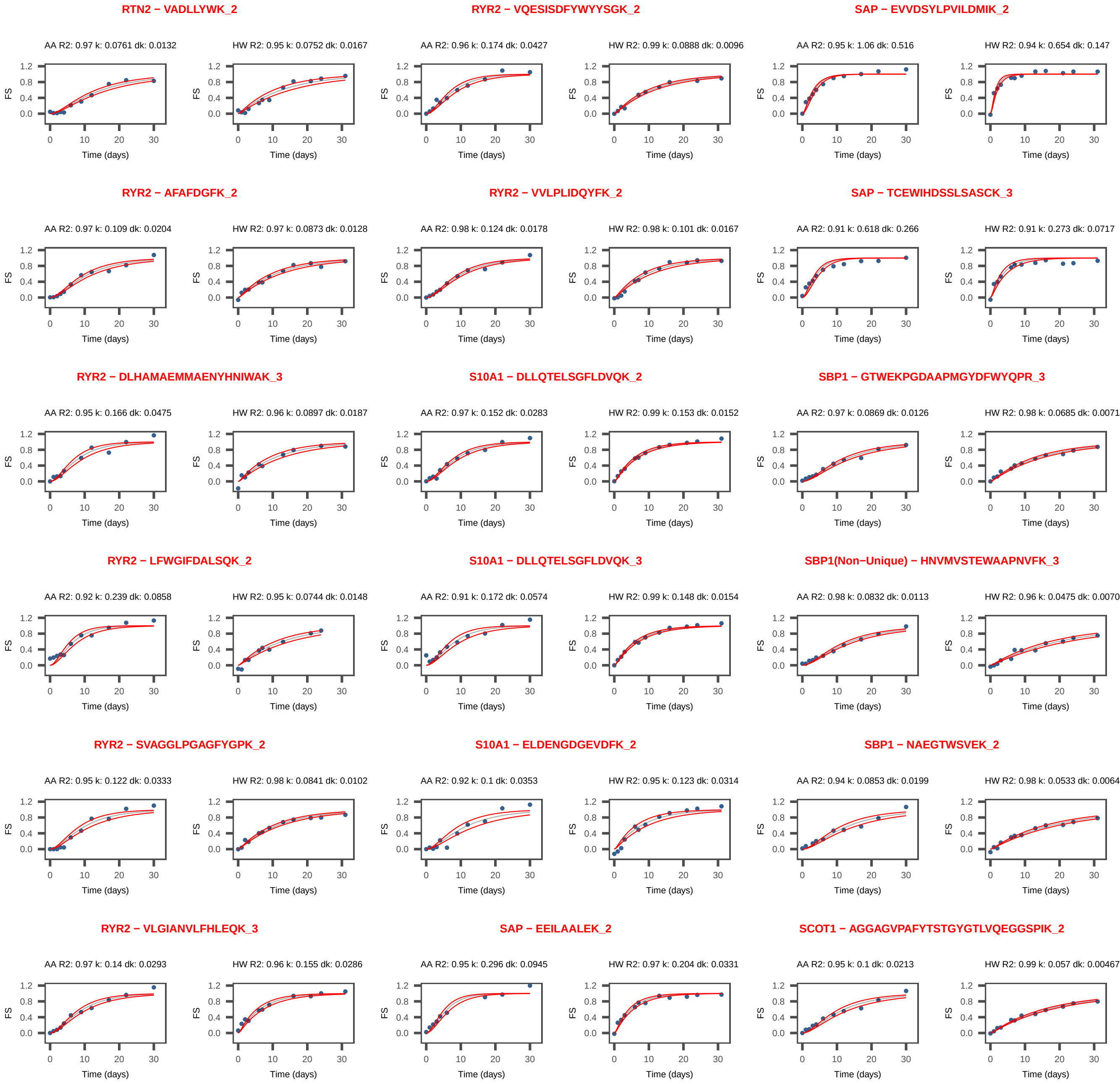
RTN2 − VADLLYWK_2
AA R2: 0.97 k: 0.0761 dk: 0.0132
HW R2: 0.95 k: 0.0752 dk: 0.0167
RYR2 − VQESISDFYWYYSGK_2
AA R2: 0.96 k: 0.174 dk: 0.0427
HW R2: 0.99 k: 0.0888 dk: 0.0096
SAP − EVVDSYLPVILDMIK_2
AA R2: 0.95 k: 1.06 dk: 0.516
HW R2: 0.94 k: 0.654 dk: 0.147
RYR2 − AFAFDGFK_2
AA R2: 0.97 k: 0.109 dk: 0.0204
HW R2: 0.97 k: 0.0873 dk: 0.0128
RYR2 − VVLPLIDQYFK_2
AA R2: 0.98 k: 0.124 dk: 0.0178
HW R2: 0.98 k: 0.101 dk: 0.0167
SAP − TCEWIHDSSLSASCK_3
AA R2: 0.91 k: 0.618 dk: 0.266
HW R2: 0.91 k: 0.273 dk: 0.0717
RYR2 − DLHAMAEMMAENYHNIWAK_3
AA R2: 0.95 k: 0.166 dk: 0.0475
HW R2: 0.96 k: 0.0897 dk: 0.0187
S10A1 − DLLQTELSGFLDVQK_2
AA R2: 0.97 k: 0.152 dk: 0.0283
HW R2: 0.99 k: 0.153 dk: 0.0152
SBP1 − GTWEKPGDAAPMGYDFWYQPR_3
AA R2: 0.97 k: 0.0869 dk: 0.0126
HW R2: 0.98 k: 0.0685 dk: 0.0071
RYR2 − LFWGIFDALSQK_2
AA R2: 0.92 k: 0.239 dk: 0.0858
HW R2: 0.95 k: 0.0744 dk: 0.0148
S10A1 − DLLQTELSGFLDVQK_3
AA R2: 0.91 k: 0.172 dk: 0.0574
HW R2: 0.99 k: 0.148 dk: 0.0154
SBP1(Non−Unique) − HNVMVSTEWAAPNVFK_3
AA R2: 0.98 k: 0.0832 dk: 0.0113
HW R2: 0.96 k: 0.0475 dk: 0.0070
RYR2 − SVAGGLPGAGFYGPK_2
AA R2: 0.95 k: 0.122 dk: 0.0333
HW R2: 0.98 k: 0.0841 dk: 0.0102
S10A1 − ELDENGDGEVDFK_2
AA R2: 0.92 k: 0.1 dk: 0.0353
HW R2: 0.95 k: 0.123 dk: 0.0314
SBP1 − NAEGTWSVEK_2
AA R2: 0.94 k: 0.0853 dk: 0.0199
HW R2: 0.98 k: 0.0533 dk: 0.0064
RYR2 − VLGIANVLFHLEQK_3
AA R2: 0.97 k: 0.14 dk: 0.0293
HW R2: 0.96 k: 0.155 dk: 0.0286
SAP − EEILAALEK_2
AA R2: 0.95 k: 0.296 dk: 0.0945
HW R2: 0.97 k: 0.204 dk: 0.0331
SCOT1 − AGGAGVPAFYTSTGYGTLVQEGGSPIK_2
AA R2: 0.95 k: 0.1 dk: 0.0213
HW R2: 0.99 k: 0.057 dk: 0.00467
FS
Time (days)

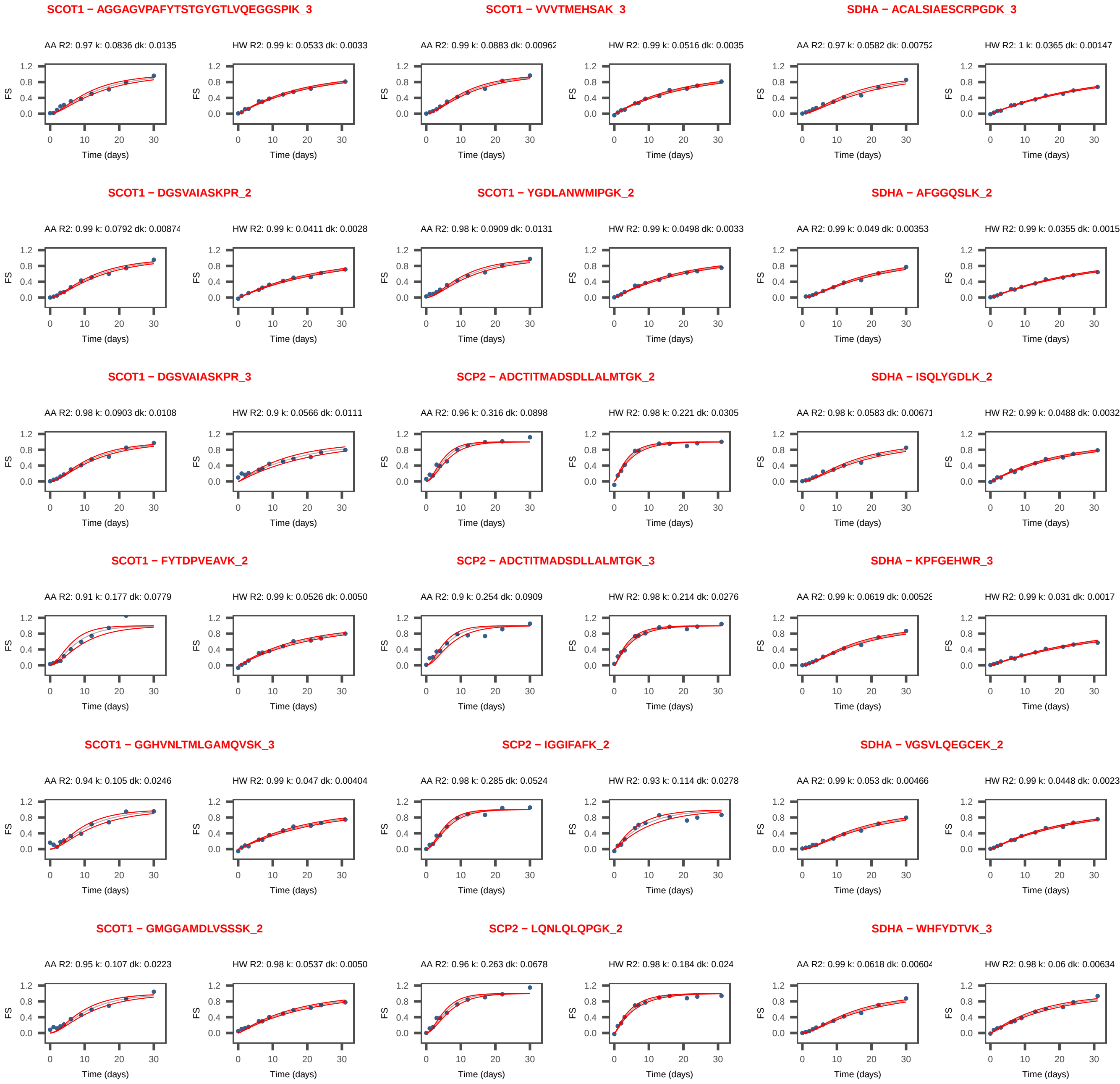
SCOT1 − AGGAGVPAFYTSTGYGTLVQEGGSPIK_3
AA R2: 0.97 k: 0.0836 dk: 0.0135
HW R2: 0.99 k: 0.0533 dk: 0.0033
SCOT1 − VVVTMEHSAK_3
AA R2: 0.99 k: 0.0883 dk: 0.00962
HW R2: 0.99 k: 0.0516 dk: 0.0035
SDHA − ACALSIAESCRPGDK_3
AA R2: 0.97 k: 0.0582 dk: 0.00752
HW R2: 1 k: 0.0365 dk: 0.00147
SCOT1 − DGSVAIASKPR_2
AA R2: 0.99 k: 0.0792 dk: 0.00874
HW R2: 0.99 k: 0.0411 dk: 0.0028
SCOT1 − YGDLANWMIPGK_2
AA R2: 0.98 k: 0.0909 dk: 0.0131
HW R2: 0.99 k: 0.0498 dk: 0.0033
SDHA − AFGGQSLK_2
AA R2: 0.99 k: 0.049 dk: 0.00353
HW R2: 0.99 k: 0.0355 dk: 0.0015
SCOT1 − DGSVAIASKPR_3
AA R2: 0.98 k: 0.0903 dk: 0.0108
HW R2: 0.9 k: 0.0566 dk: 0.0111
SCP2 − ADCTITMADSDLLALMTGK_2
AA R2: 0.96 k: 0.316 dk: 0.0898
HW R2: 0.98 k: 0.221 dk: 0.0305
SDHA − ISQLYGDLK_2
AA R2: 0.98 k: 0.0583 dk: 0.00671
HW R2: 0.99 k: 0.0488 dk: 0.0032
SCOT1 − FYTDPVEAVK_2
AA R2: 0.91 k: 0.177 dk: 0.0779
HW R2: 0.99 k: 0.0526 dk: 0.0050
SCP2 − ADCTITMADSDLLALMTGK_3
AA R2: 0.9 k: 0.254 dk: 0.0909
HW R2: 0.98 k: 0.214 dk: 0.0276
SDHA − KPFGEHWR_3
AA R2: 0.99 k: 0.0619 dk: 0.00528
HW R2: 0.99 k: 0.031 dk: 0.0017
SCOT1 − GGHVNLTMLGAMQVSK_3
AA R2: 0.94 k: 0.105 dk: 0.0246
HW R2: 0.99 k: 0.047 dk: 0.00404
SCP2 − IGGIFAFK_2
AA R2: 0.98 k: 0.285 dk: 0.0524
HW R2: 0.93 k: 0.114 dk: 0.0278
SDHA − VGSVLQEGCEK_2
AA R2: 0.99 k: 0.053 dk: 0.00466
HW R2: 0.99 k: 0.0448 dk: 0.0023
SCOT1 − GMGGAMDLVSSSK_2
AA R2: 0.95 k: 0.107 dk: 0.0223
HW R2: 0.98 k: 0.0537 dk: 0.0050
SCP2 − LQNLQLQPGK_2
AA R2: 0.96 k: 0.263 dk: 0.0678
HW R2: 0.98 k: 0.184 dk: 0.024
SDHA − WHFYDTVK_3
AA R2: 0.99 k: 0.0618 dk: 0.00604
HW R2: 0.98 k: 0.06 dk: 0.00634
FS
Time (days)

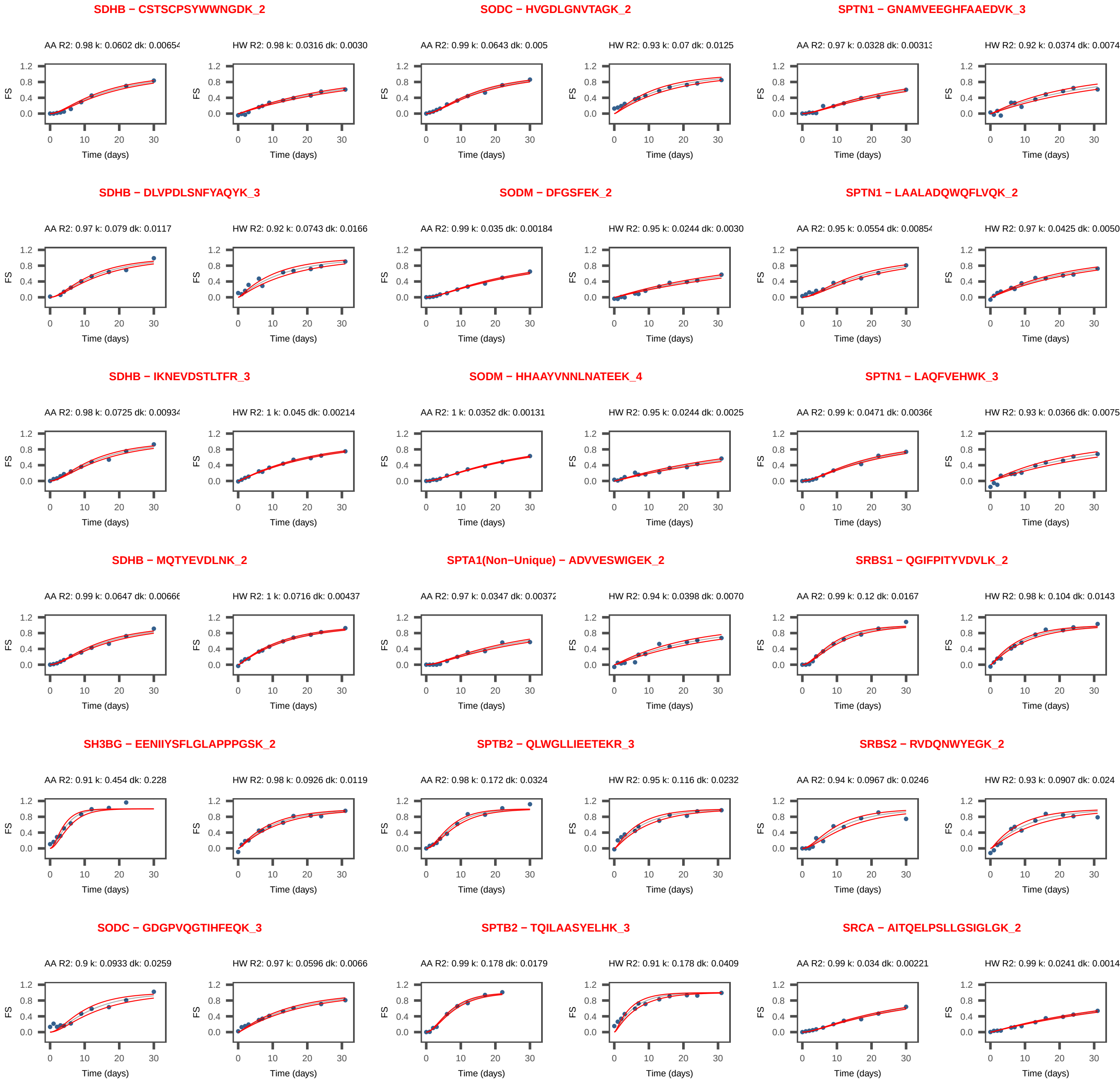
SDHB − CSTSCPSYWWNGDK_2
AA R2: 0.98 k: 0.0602 dk: 0.00654
HW R2: 0.98 k: 0.0316 dk: 0.0030
SODC − HVGDLGNVTAGK_2
AA R2: 0.99 k: 0.0643 dk: 0.005
HW R2: 0.93 k: 0.07 dk: 0.0125
SPTN1 − GNAMVEEGHFAAEDVK_3
AA R2: 0.97 k: 0.0328 dk: 0.00313
HW R2: 0.92 k: 0.0374 dk: 0.0074
SDHB − DLVPDLSNFYAQYK_3
AA R2: 0.97 k: 0.079 dk: 0.0117
HW R2: 0.92 k: 0.0743 dk: 0.0166
SODM − DFGSFEK_2
AA R2: 0.99 k: 0.035 dk: 0.00184
HW R2: 0.95 k: 0.0244 dk: 0.0030
SPTN1 − LAALADQWQFLVQK_2
AA R2: 0.95 k: 0.0554 dk: 0.00854
HW R2: 0.97 k: 0.0425 dk: 0.0050
SDHB − IKNEVDSTLTFR_3
AA R2: 0.98 k: 0.0725 dk: 0.00934
HW R2: 1 k: 0.045 dk: 0.00214
SODM − HHAAYVNNLNATEEK_4
AA R2: 1 k: 0.0352 dk: 0.00131
HW R2: 0.95 k: 0.0244 dk: 0.0025
SPTN1 − LAQFVEHWK_3
AA R2: 0.99 k: 0.0471 dk: 0.00366
HW R2: 0.93 k: 0.0366 dk: 0.0075
SDHB − MQTYEVDLNK_2
AA R2: 0.99 k: 0.0647 dk: 0.00666
HW R2: 1 k: 0.0716 dk: 0.00437
SPTA1(Non−Unique) − ADVVESWIGEK_2
AA R2: 0.97 k: 0.0347 dk: 0.00372
HW R2: 0.94 k: 0.0398 dk: 0.0070
SRBS1 − QGIFPITYVDVLK_2
AA R2: 0.99 k: 0.12 dk: 0.0167
HW R2: 0.98 k: 0.104 dk: 0.0143
SH3BG − EENIIYSFLGLAPPPGSK_2
AA R2: 0.91 k: 0.454 dk: 0.228
HW R2: 0.98 k: 0.0926 dk: 0.0119
SPTB2 − QLWGLLIEETEKR_3
AA R2: 0.98 k: 0.172 dk: 0.0324
HW R2: 0.95 k: 0.116 dk: 0.0232
SRBS2 − RVDQNWYEGK_2
AA R2: 0.94 k: 0.0967 dk: 0.0246
HW R2: 0.93 k: 0.0907 dk: 0.024
SODC − GDGPVQGTIHFEQK_3
AA R2: 0.9 k: 0.0933 dk: 0.0259
HW R2: 0.97 k: 0.0596 dk: 0.0066
SPTB2 − TQILAASYELHK_3
AA R2: 0.99 k: 0.178 dk: 0.0179
HW R2: 0.91 k: 0.178 dk: 0.0409
SRCA − AITQELPSLLGSIGLGK_2
AA R2: 0.99 k: 0.034 dk: 0.00221
HW R2: 0.99 k: 0.0241 dk: 0.0014
FS
Time (days)

SRCA − AITQELPSLLGSIGLGK_3

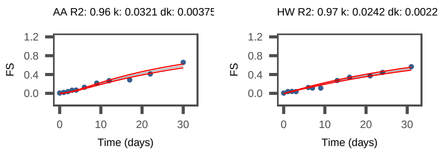

SUCA − HLGLPVFNTVK_2

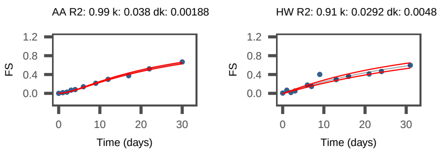

SUCA − MGHAGAIIAGGK_3

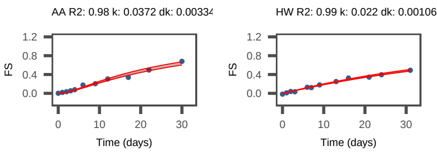

SRCA − ELFLKEEISLLEDLNQVIENR_3

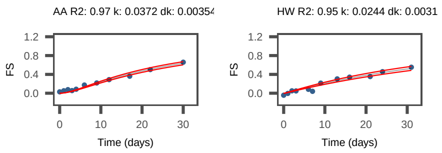

SUCA − HLGLPVFNTVK_3

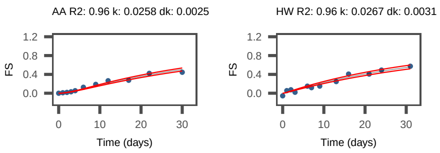

SUCA − QGTFHSQQALEYGTK_2

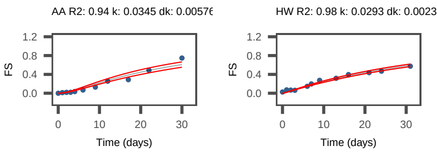

SRCA − FGQNFLEK_2

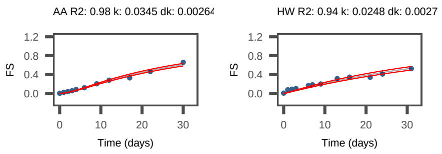

SUCA − IGIMPGHIHK_3

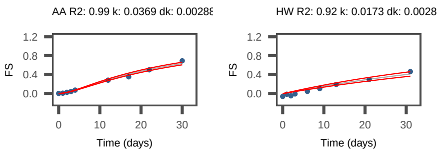

SUCA − QGTFHSQQALEYGTK_3

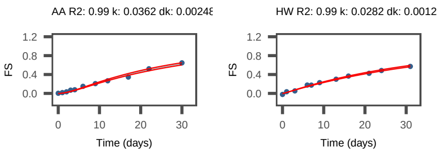

SRCA − VLFLGPWSVGK_2

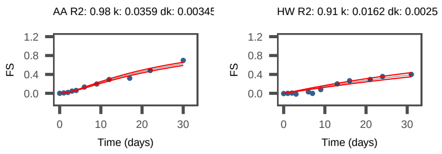

SUCA − IICQGFTGK_2

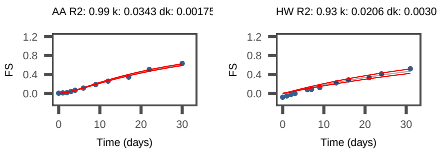

SUCB1 − ALIADSGLK_2

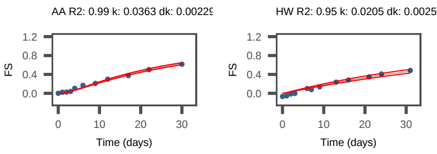

SRCA − VYVSSFWPQDYKPDTHR_3

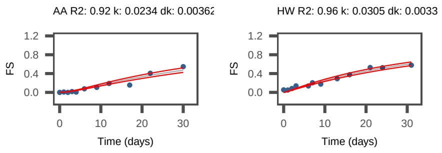

SUCA − ISALQSAGVVVSMSPAQLGTTIYK_2

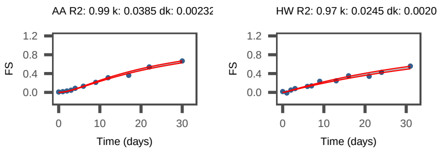

SUCB1 − CDVIAQGIVMAVK_2

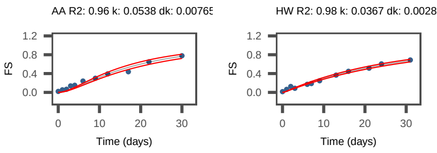

SUCA − AKPVVSFIAGITAPPGR_3

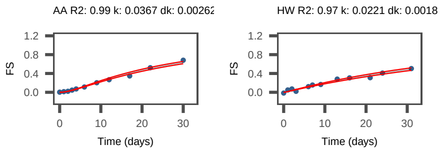

SUCA − LIGPNCPGVINPGECK_2

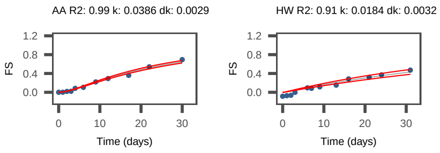

SUCB1 − EQAVTLAQK_2

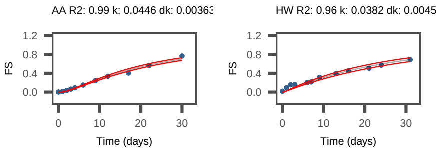

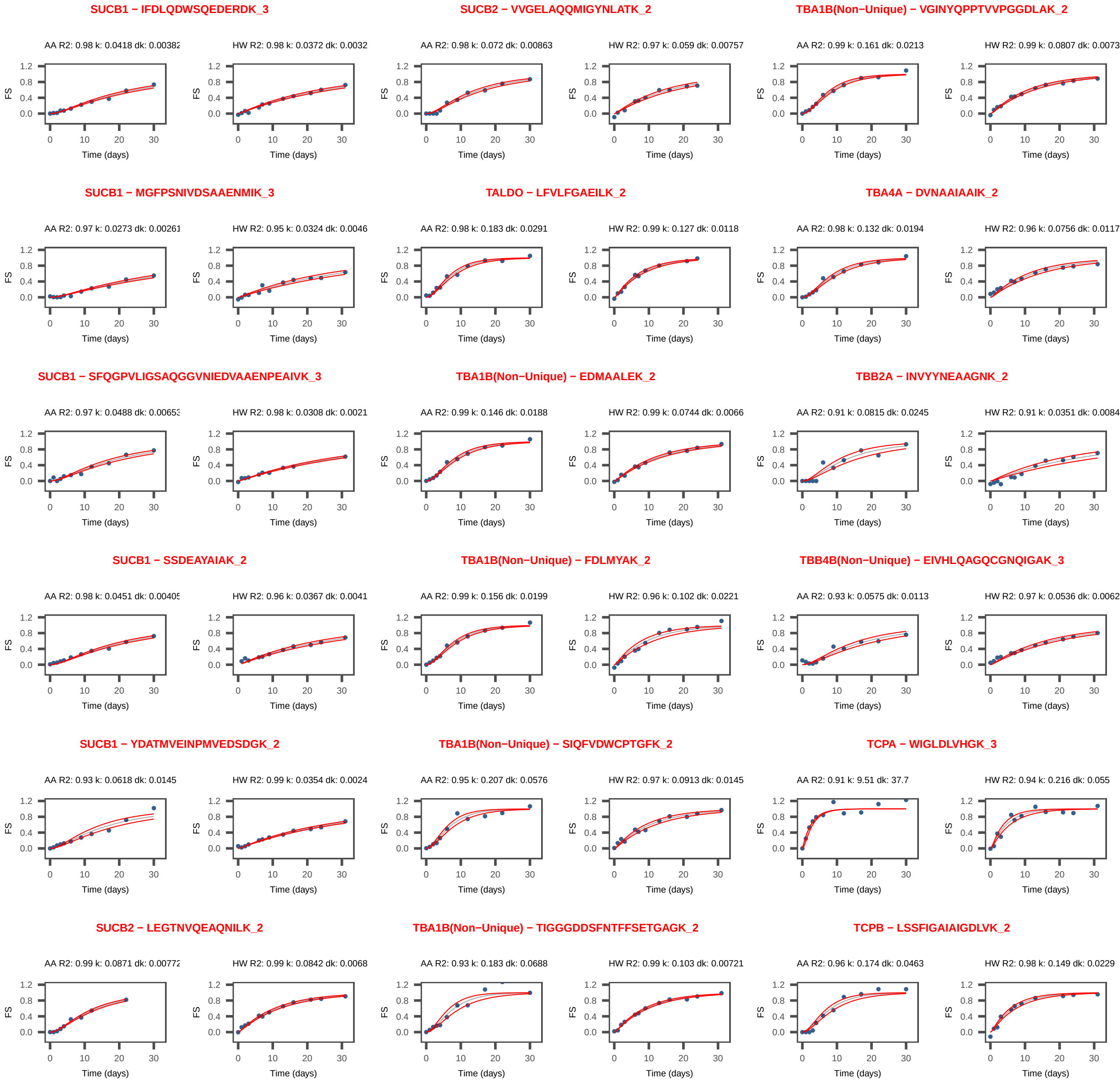
SUCB1 − IFDLQDWSQEDERDK_3
AA R2: 0.98 k: 0.0418 dk: 0.00382
HW R2: 0.98 k: 0.0372 dk: 0.0032
SUCB2 − VVGELAQQMIGYNLATK_2
AA R2: 0.98 k: 0.072 dk: 0.00863
HW R2: 0.97 k: 0.059 dk: 0.00757
TBA1B(Non−Unique) − VGINYQPPTVVPGGDLAK_2
AA R2: 0.99 k: 0.161 dk: 0.0213
HW R2: 0.99 k: 0.0807 dk: 0.0073
SUCB1 − MGFPSNIVDSAAENMIK_3
AA R2: 0.97 k: 0.0273 dk: 0.00261
HW R2: 0.95 k: 0.0324 dk: 0.0046
TALDO − LFVLFGAEILK_2
AA R2: 0.98 k: 0.183 dk: 0.0291
HW R2: 0.99 k: 0.127 dk: 0.0118
TBA4A − DVNAAIAAIK_2
AA R2: 0.98 k: 0.132 dk: 0.0194
HW R2: 0.96 k: 0.0756 dk: 0.0117
SUCB1 − SFQGPVLIGSAQGGVNIEDVAAENPEAIVK_3
AA R2: 0.97 k: 0.0488 dk: 0.00653
HW R2: 0.98 k: 0.0308 dk: 0.0021
TBA1B(Non−Unique) − EDMAALEK_2
AA R2: 0.99 k: 0.146 dk: 0.0188
HW R2: 0.99 k: 0.0744 dk: 0.0066
TBB2A − INVYYNEAAGNK_2
AA R2: 0.91 k: 0.0815 dk: 0.0245
HW R2: 0.91 k: 0.0351 dk: 0.0084
SUCB1 − SSDEAYAIAK_2
AA R2: 0.98 k: 0.0451 dk: 0.00405
HW R2: 0.96 k: 0.0367 dk: 0.0041
TBA1B(Non−Unique) − FDLMYAK_2
AA R2: 0.99 k: 0.156 dk: 0.0199
HW R2: 0.96 k: 0.102 dk: 0.0221
TBB4B(Non−Unique) − EIVHLQAGQCGNQIGAK_3
AA R2: 0.93 k: 0.0575 dk: 0.0113
HW R2: 0.97 k: 0.0536 dk: 0.0062
SUCB1 − YDATMVEINPMVEDSDGK_2
AA R2: 0.93 k: 0.0618 dk: 0.0145
HW R2: 0.99 k: 0.0354 dk: 0.0024
TBA1B(Non−Unique) − SIQFVDWCPTGFK_2
AA R2: 0.95 k: 0.207 dk: 0.0576
HW R2: 0.97 k: 0.0913 dk: 0.0145
TCPA − WIGLDLVHGK_3
AA R2: 0.91 k: 9.51 dk: 37.7
HW R2: 0.94 k: 0.216 dk: 0.055
SUCB2 − LEGTNVQEAQNILK_2
AA R2: 0.99 k: 0.0871 dk: 0.00772
HW R2: 0.99 k: 0.0842 dk: 0.0068
TBA1B(Non−Unique) − TIGGGDDSFNTFFSETGAGK_2
AA R2: 0.93 k: 0.183 dk: 0.0688
HW R2: 0.99 k: 0.103 dk: 0.00721
TCPB − LSSFIGAIAIGDLVK_2
AA R2: 0.96 k: 0.174 dk: 0.0463
HW R2: 0.98 k: 0.149 dk: 0.0229
FS
Time (days)

TCPD – DALSDLALHFLNK_3

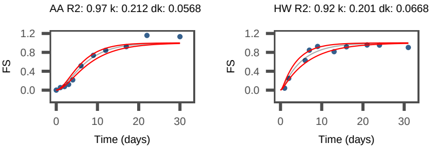

THIL – ASKPTLNEVVIVSAIR_3

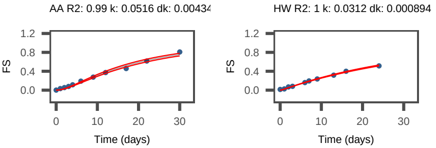

THIL – QATLGAGLPISTPCTTVNK_2

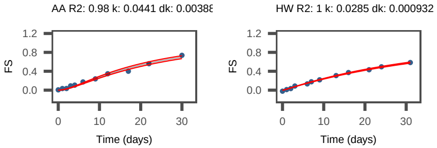

TCPQ – AIAGTGANVIVTGGK_2

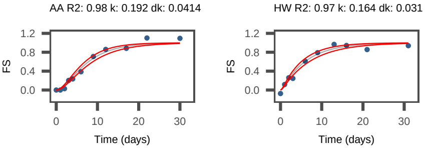

THIL – EDIAMWEVNEAFSVVVLANIK_3

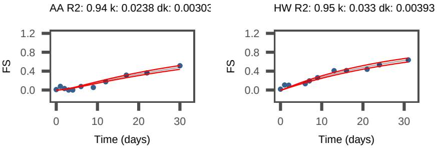

THIL – QATLGAGLPISTPCTTVNK_3

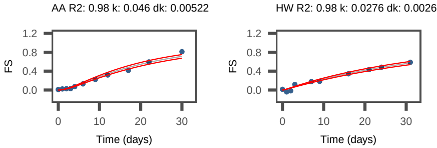

TELT – EAFWAEWK_2

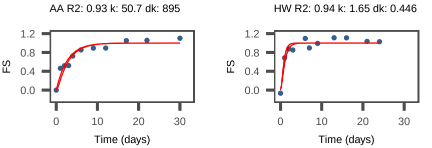

THIL – IAAFADAAVDPIDFPLAPAYAVPK_2

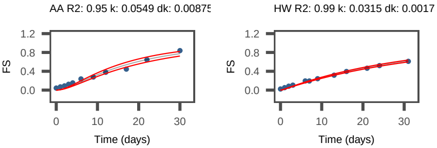

THIL – TPIGSFLGSLASQPATK_2

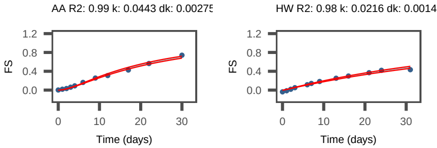

TERA – LEILQIHTK_3

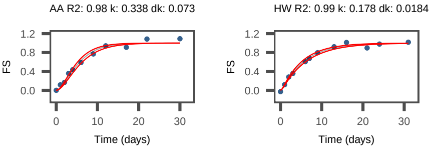

THIL – LEDLIVK_2

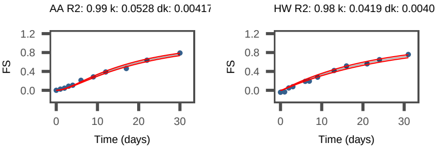

THIL – TPIGSFLGSLASQPATK_3

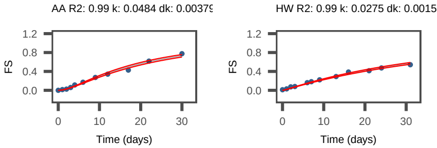

TERA – NAPAIIFIDELDAIAPK_2

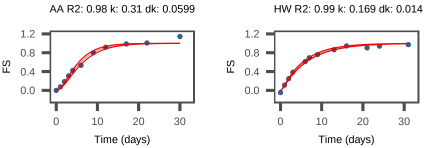

THIL – LGTAAIQGAIEK_2

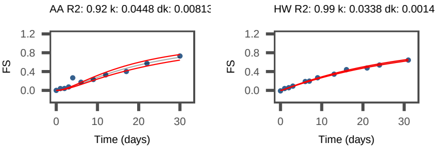

THIM – ALDLDPSK_2

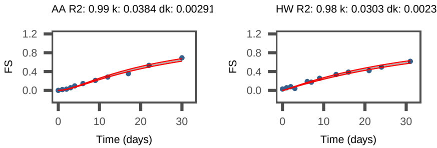

THIL – ASKPTLNEVVIVSAIR_2

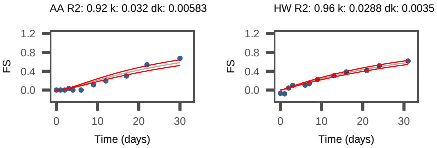

THIL – MLEIDPQK_2

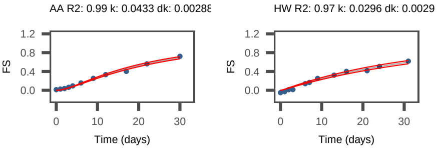

THIM – DGTVTAGNASGVSDGAGAVIIASEDAVK_2

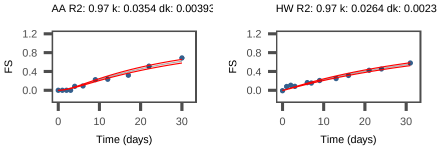

THIM – GVFIVAAK_2

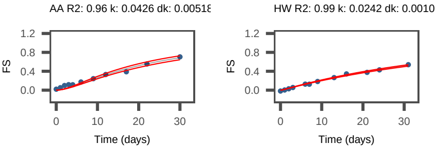

THIO – EAFQEALAAAGDK_2

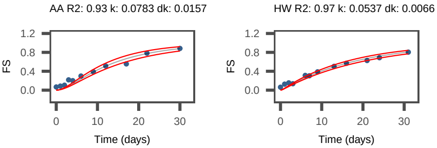

TITIN – ADTLLKPDQR_3

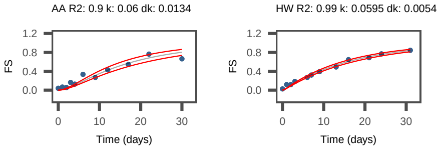

THIM – LCGSGFQSIVSGCQEICSK_2

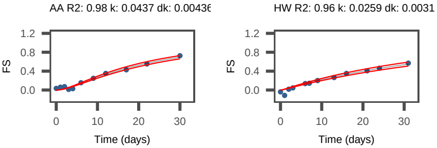

TIM50 – VLLDLSAFLK_2

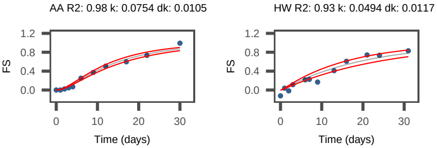

TITIN – AGDSIVLSAISILGK_2

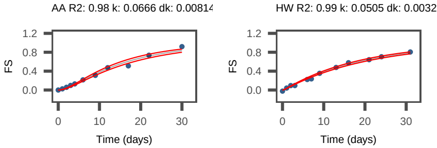

THIM – LCGSGFQSIVSGCQEICSK_3

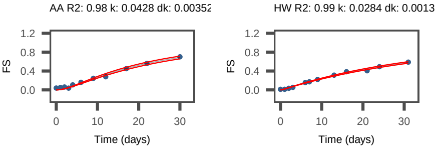

TITIN – AAADEWTTCTPPSGLQGK_2

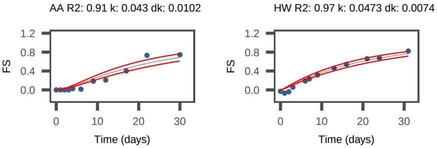

TITIN – AGDSIVLSAISILGK_3

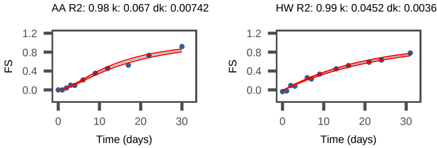

THIM – LEDTLWAGLTDQHVK_2

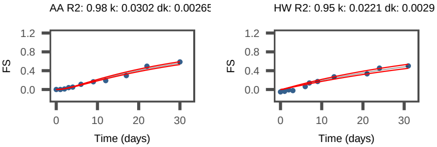

TITIN – AADPIDPPGPPAK_2

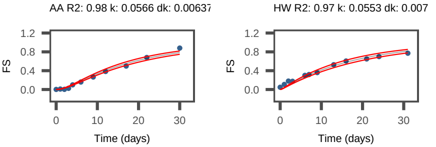

TITIN – AGEDVQLLIPFK_2

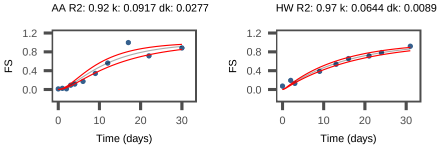

THIM – LPMGMTAENLAAK_2

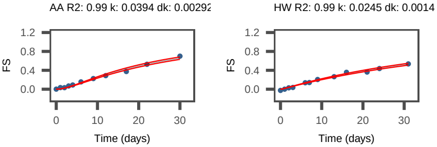

TITIN – AASFECEVSHFNVPSMWLK_3

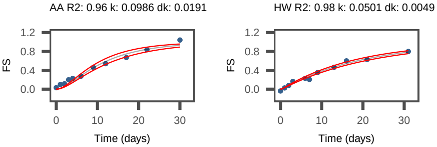

TITIN – AGSSPPSKPTEYVTAR_3

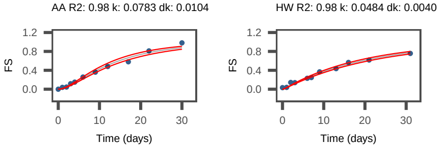

THIM – PQTTLEQLQK_2

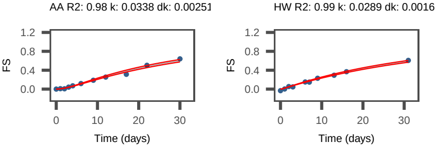

TITIN – ADSCEFTVTGLQK_2

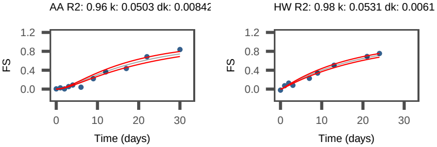

TITIN – AIIDNTESYSLLIVDK_2

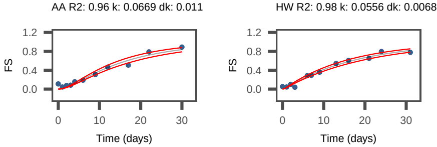

TITIN – ALDPFTTPSPPTSLEITSVTK_2

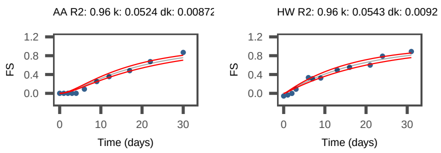

TITIN – ATLTWTPPLEDGGSPIK_2

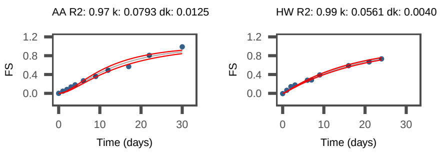

TITIN – AYATITNNCTK_2

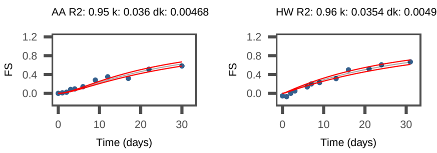

TITIN – ALVPGNIFK_2

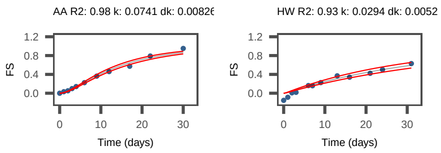

TITIN – ATSTAELVVQGEEVVPAK_2

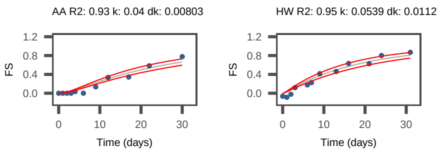

TITIN – AYSTVATNCHK_3

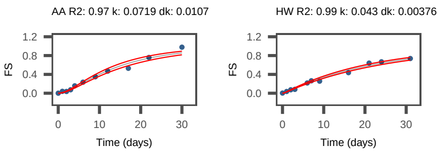

TITIN – AMIATSETHTELVIK_3

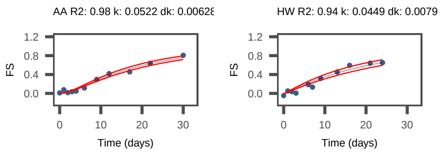

TITIN – ATSYTITSLIENQEYK_2

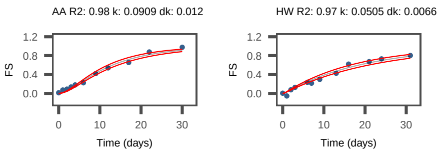

TITIN – CREPVNPPSAPSVVK_3

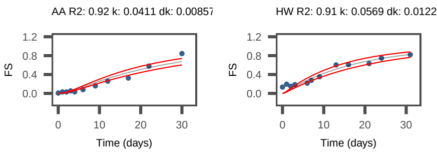

TITIN – APPIEPAPTPIAAPVTAPVVGK_2

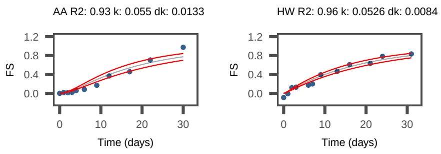

TITIN – AVFVNVK_2

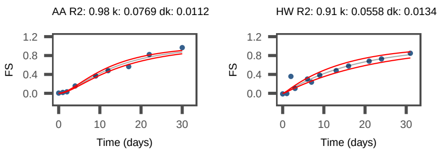

TITIN – CTLAWSPPLQDGGSDISHYVVEK_3

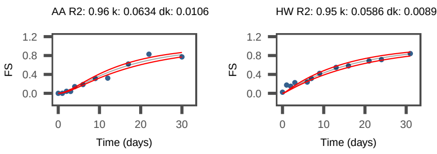

TITIN – AQIDVTPVGSK_2

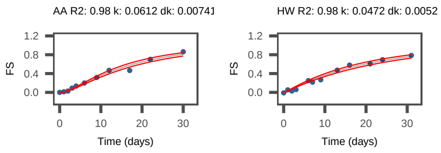

TITIN – AVYAQDPLYPPGPPAFPK_2

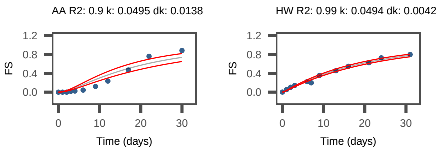

TITIN – DAHRPGWLPVSESVTRPTFK_4

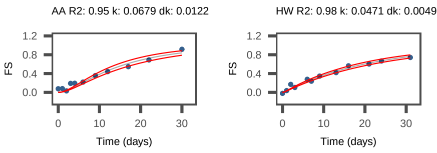

TITIN – ATGNPNPDIVWLK_2

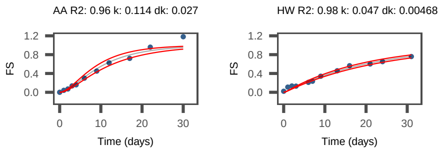

TITIN – AVYAQDPLYPPGPPAFPK_3

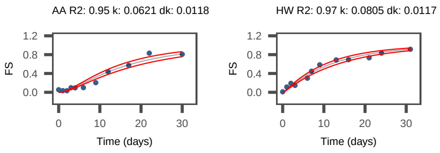

TITIN – DENVPPTVEFGPEYFDGLVIK_3

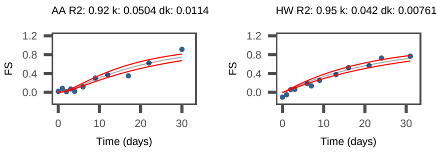

TITIN − DIRPSDIAQITSTPTSSMLTVK_3

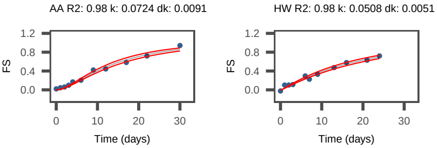

TITIN − DSLRPDHGQYMIK_3

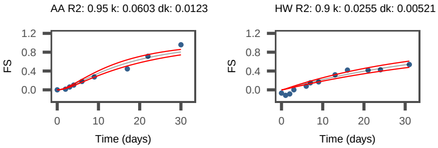

TITIN − EEQSEVVAVPTSQTSK_2

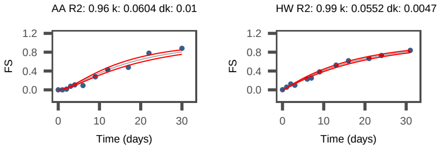

TITIN − DLPDLCYLAK_2

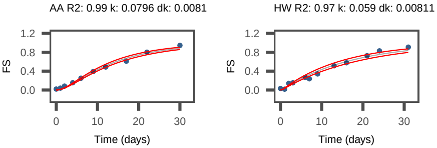

TITIN − DSMVLTWTRPVDDGGAEIEGYILEK_3

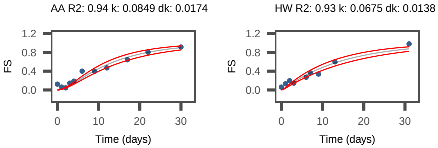

TITIN − EGQTATFVCELSHEK_3

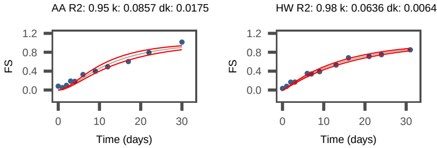

TITIN − DLSLIVTGLK_2

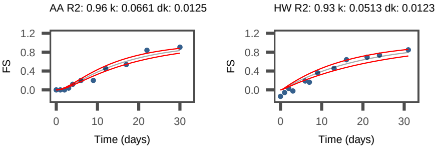

TITIN − DSPNIQTSFLDNIATLNIFK_3

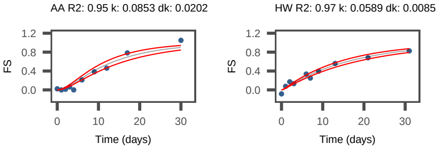

TITIN − ELLLPVLIK_2

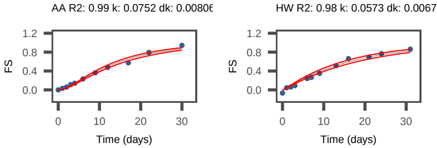

TITIN − DSAYVTWDPPIIDGGSPIINYVVEK_2

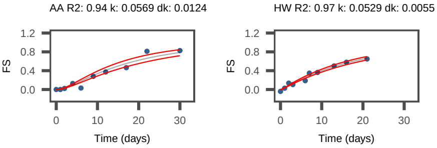

TITIN − DSVNLTWTEPASDGGSK_2

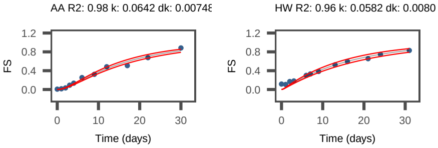

TITIN − ELPLIFITPLSDVK_2

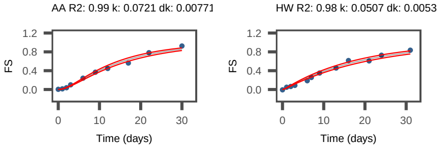

TITIN − DSAYVTWDPPIIDGGSPIINYVVEK_3

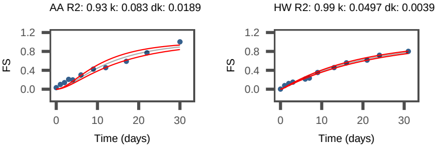

TITIN − DSVTLSWEPPLIDGGAK_2

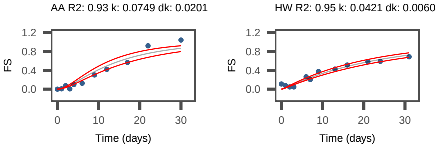

TITIN − ESLSTYEHYASAEMK_3

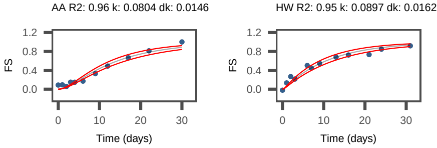

TITIN − DSGYYSLTAENSSGSDTQK_2

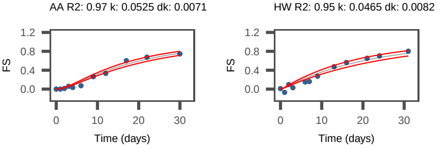

TITIN − DTGEYQLTVSNAAGTK_2

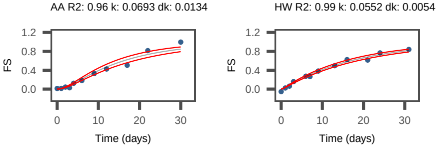

TITIN − ETAMLSWDVPENDGGAPVK_2

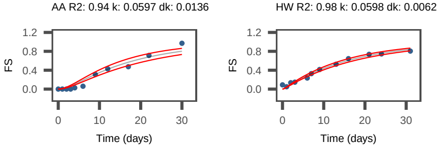

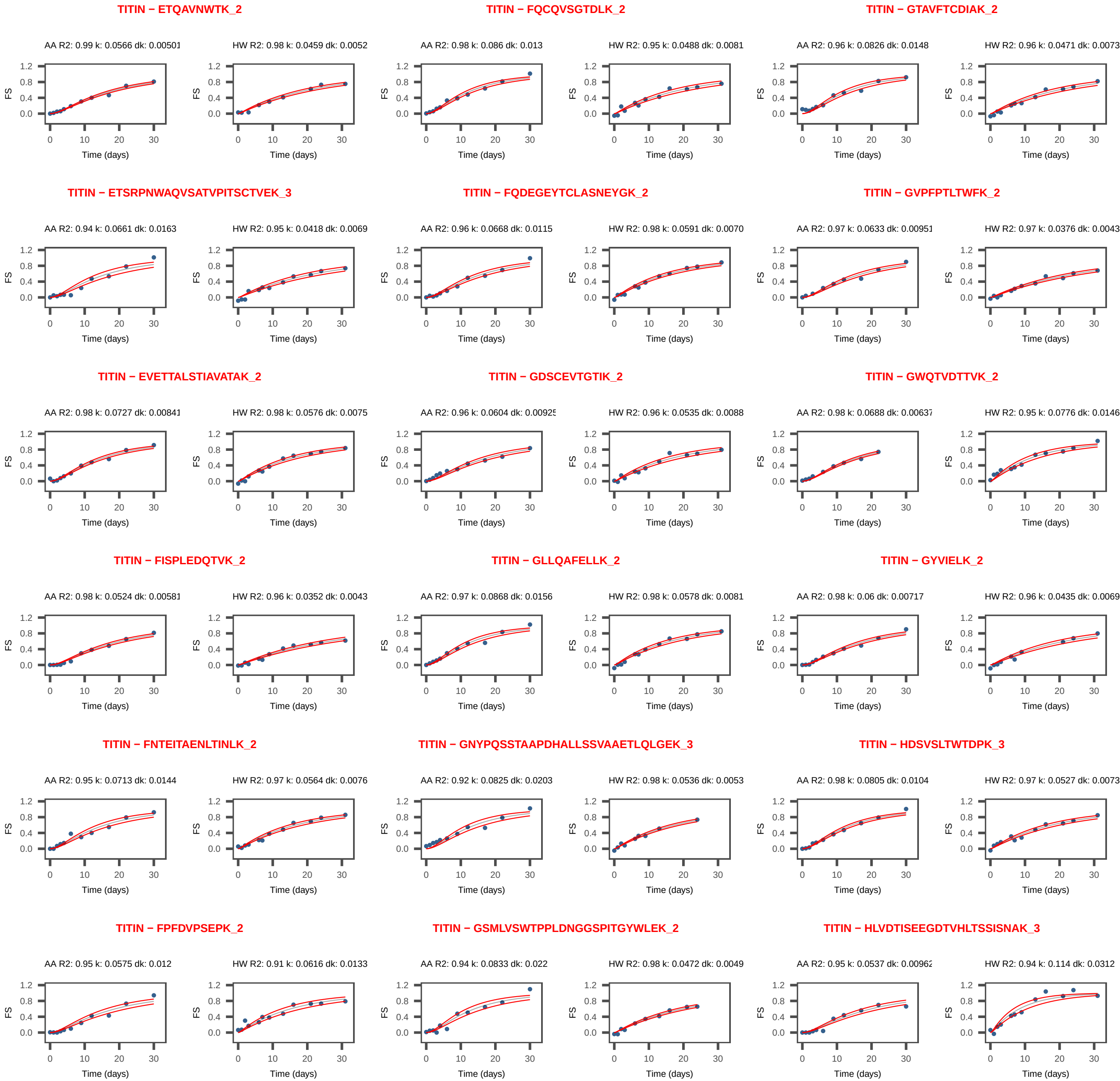
TITIN − ETQAVNWTK_2
AA R2: 0.99 k: 0.0566 dk: 0.00501
HW R2: 0.98 k: 0.0459 dk: 0.0052
TITIN − FQCQVSGTDLK_2
AA R2: 0.98 k: 0.086 dk: 0.013
HW R2: 0.95 k: 0.0488 dk: 0.0081
TITIN − GTAVFTCDIAK_2
AA R2: 0.96 k: 0.0826 dk: 0.0148
HW R2: 0.96 k: 0.0471 dk: 0.0073
TITIN − ETSRPNWAQVSATVPITSCTVEK_3
AA R2: 0.94 k: 0.0661 dk: 0.0163
HW R2: 0.95 k: 0.0418 dk: 0.0069
TITIN − FQDEGEYTCLASNEYGK_2
AA R2: 0.96 k: 0.0668 dk: 0.0115
HW R2: 0.98 k: 0.0591 dk: 0.0070
TITIN − GVPFPTLTWFK_2
AA R2: 0.97 k: 0.0633 dk: 0.00951
HW R2: 0.97 k: 0.0376 dk: 0.0043
TITIN − EVETTALSTIAVATAK_2
AA R2: 0.98 k: 0.0727 dk: 0.00841
HW R2: 0.98 k: 0.0576 dk: 0.0075
TITIN − GDSCEVTGTIK_2
AA R2: 0.96 k: 0.0604 dk: 0.00925
HW R2: 0.96 k: 0.0535 dk: 0.0088
TITIN − GWQTVDTTVK_2
AA R2: 0.98 k: 0.0688 dk: 0.00637
HW R2: 0.95 k: 0.0776 dk: 0.0146
TITIN − FISPLEDQTVK_2
AA R2: 0.98 k: 0.0524 dk: 0.00581
HW R2: 0.96 k: 0.0352 dk: 0.0043
TITIN − GLLQAFELLK_2
AA R2: 0.97 k: 0.0868 dk: 0.0156
HW R2: 0.98 k: 0.0578 dk: 0.0081
TITIN − GYVIELK_2
AA R2: 0.98 k: 0.06 dk: 0.00717
HW R2: 0.96 k: 0.0435 dk: 0.0069
TITIN − FNTEITAENLTINLK_2
AA R2: 0.95 k: 0.0713 dk: 0.0144
HW R2: 0.97 k: 0.0564 dk: 0.0076
TITIN − GNYPQSSTAAPDHALLSSVAAETLQLGEK_3
AA R2: 0.92 k: 0.0825 dk: 0.0203
HW R2: 0.98 k: 0.0536 dk: 0.0053
TITIN − HDSVSLTWTDPK_3
AA R2: 0.98 k: 0.0805 dk: 0.0104
HW R2: 0.97 k: 0.0527 dk: 0.0073
TITIN − FPFDVPSEPK_2
AA R2: 0.95 k: 0.0575 dk: 0.012
HW R2: 0.91 k: 0.0616 dk: 0.0133
TITIN − GSMLVSWTPPLDNGGSPITGYWLEK_2
AA R2: 0.94 k: 0.0833 dk: 0.022
HW R2: 0.98 k: 0.0472 dk: 0.0049
TITIN − HLVDTISEEGDTVHLTSSISNAK_3
AA R2: 0.95 k: 0.0537 dk: 0.00962
HW R2: 0.94 k: 0.114 dk: 0.0312
FS
Time (days)

TITIN − IDVPFIGRPPPAVTWHK_4

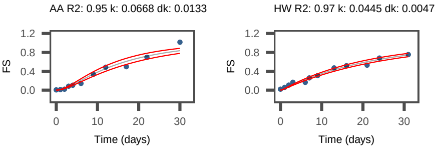

TITIN − INADIAGRPLPVISWAK_3

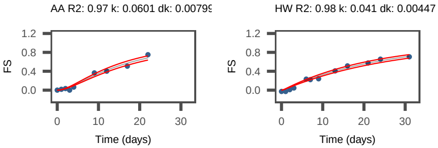

TITIN − KTPSPIEAER_3

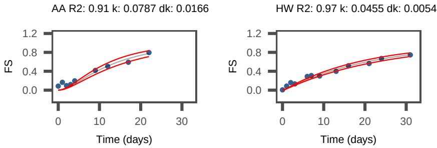

TITIN − IDWFKDEADVLEDDR_3

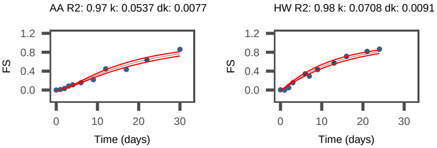

TITIN − ITIPTFKPER_3

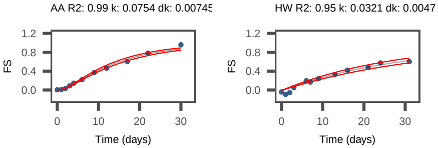

TITIN − LAWALIEDNCEALSYTAIK_2

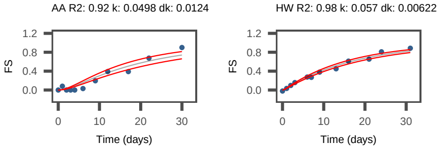

TITIN − IEPLEVALGHLAK_3

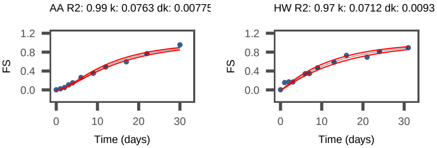

TITIN − ITNYVIEK_2

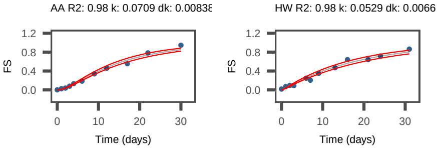

TITIN − LAWTNVATEVQVTK_2

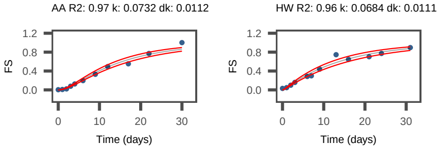

TITIN − IHEEDKIDVQGGR_3

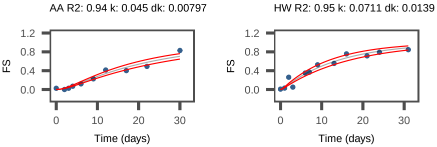

TITIN − IVGYWVEK_2

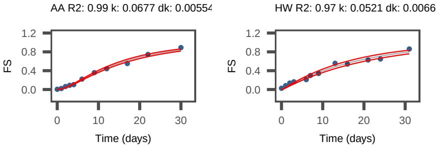

TITIN − LAWTVVASEVVTNSLK_3

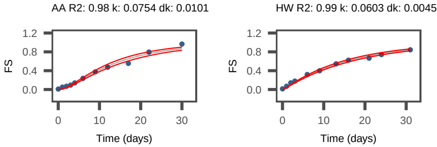

TITIN − IHNLSESDCGEYVCEVSGEGGTSK_3

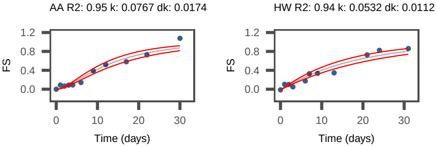

TITIN − KAEAVATVVAAVDQAR_3

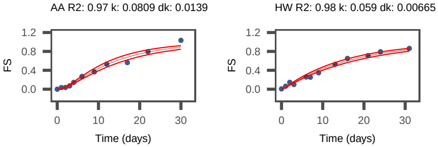

TITIN − LDQAGEVLYQACNAITTAILTVK_3

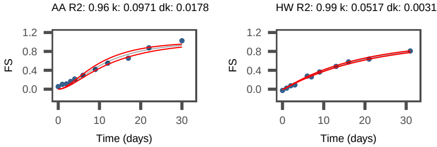

TITIN − IMAENAAGISAPSATSPFYK_2

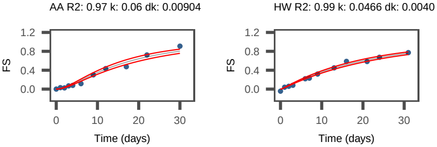

TITIN − KTPSPIEAER_2

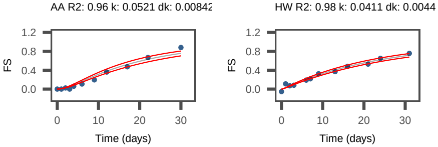

TITIN − LDQTGGVDFQAANVK_2

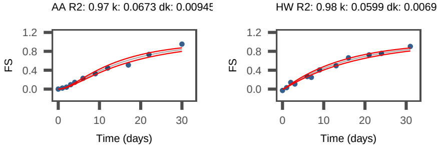

TITIN − LEADVHGKPLPTIEWLR_4

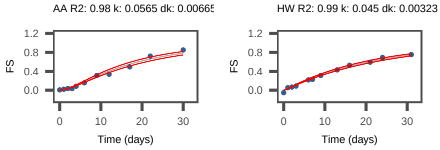

TITIN − LLERPPEFTLPLYNK_3

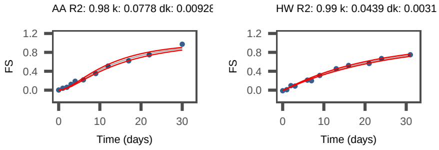

TITIN − LTWFSPEDDGGSPITNYVIQK_3

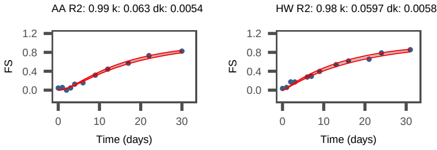

TITIN − LEDAGEVQLTAK_2

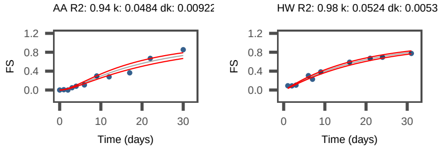

TITIN − LLLQAAPQFHPGYPLK_3

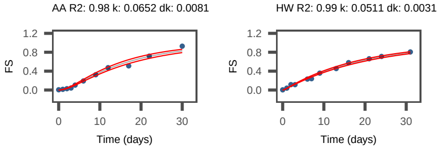

TITIN − LVVTGLK_2

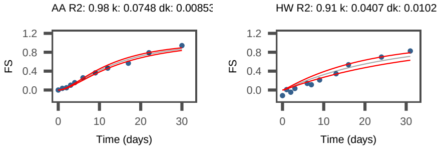

TITIN − LFVETLHITK_3

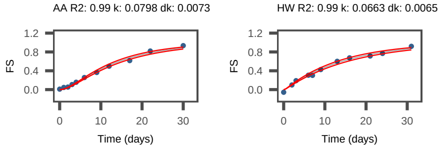

TITIN − LPFNTYSVQAGEDLK_2

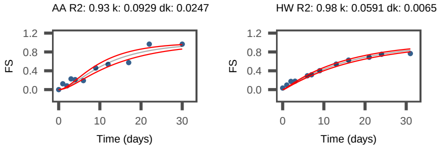

TITIN − MAHEGALTGVTTDQK_3

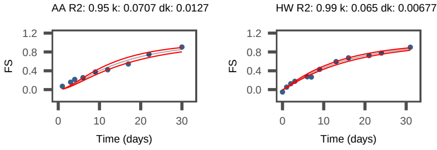

TITIN − LIEGLVVK_2

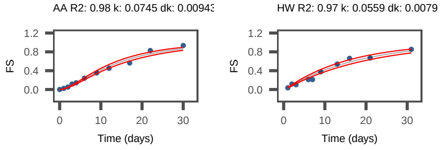

TITIN − LSGVLTVK_2

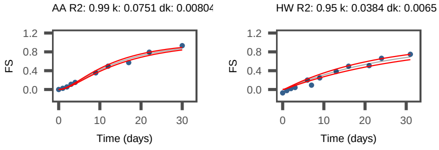

TITIN − MGVGPPLDSIPTVAK_2

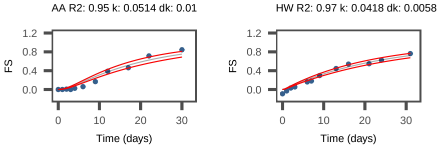

TITIN − LIEHQEYK_3

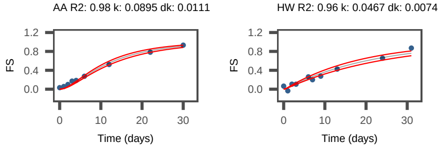

TITIN − LTPIGDPTMVVEWLHDGKPLEAANR_4

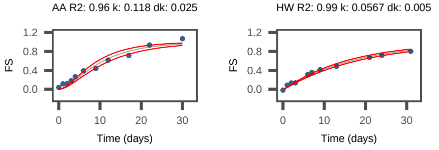

TITIN − MTASEALQHPWLK_3

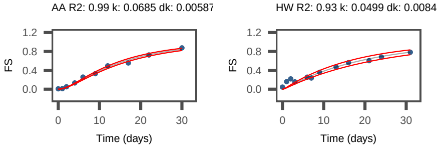

TITIN − LIVEGAVVEFVK_2

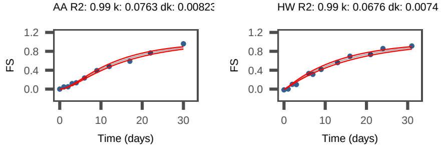

TITIN − LTWFSPEDDGGSPITNYVIQK_2

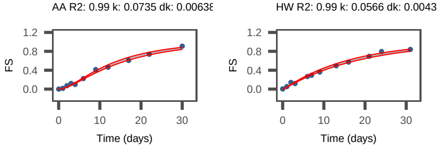

TITIN − MTLWWEAPLNDGCAPVTHYIIEK_3

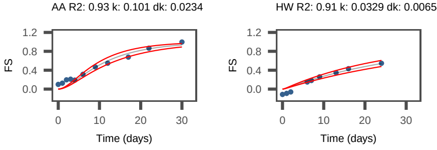

TITIN − NAADSVSEPSESTGPITVK_2

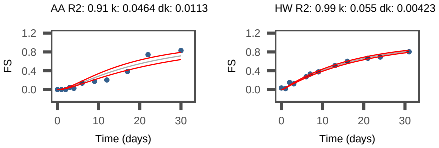

TITIN − NSFTIPSQPGIPEEVGAGK_2

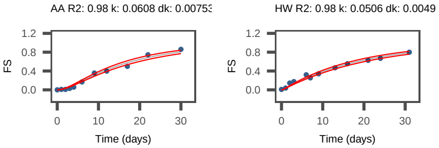

TITIN − PIYDGGSEILGYVVEICK_2

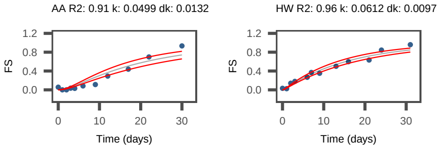

TITIN − NAAHEDGGIYSLTVENPAGTK_3

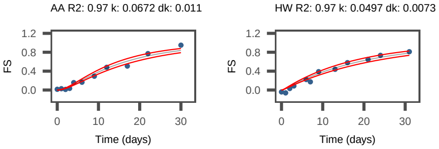

TITIN − NSVSLSWEKPEHDGGSR_3

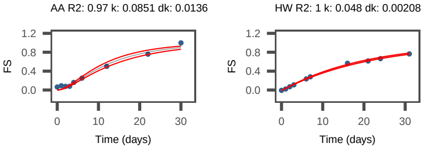

TITIN − PIYDGGSEILGYVVEICK_3

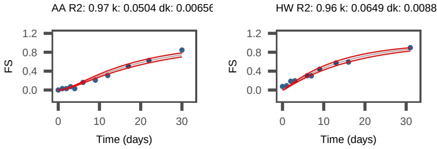

TITIN − NAFVTPGPPSIPEVTK_2

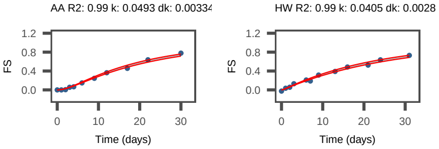

TITIN − NTAGAISAPSESTGTIICK_2

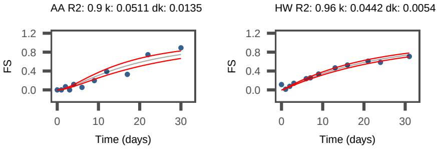

TITIN − PLPVISWAK_2

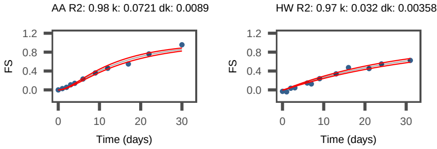

TITIN − NCAMADESVYGFK_2

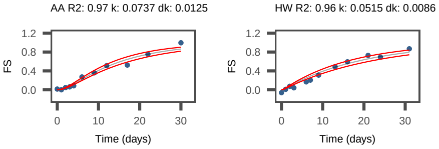

TITIN − NVHFGDAADYTFVAGK_3

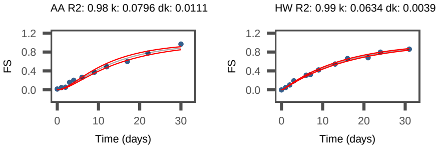

TITIN − PMYDGGTDIIGYVLEMQEK_2

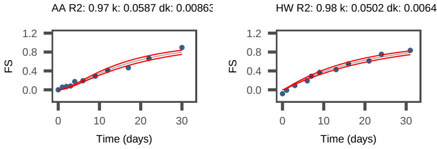

TITIN − NPFVVPDAPK_2

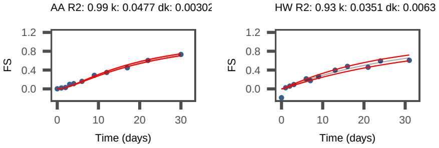

TITIN − PIAILQGLSDQK_2

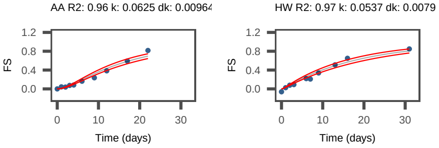

TITIN − PTPTAVWSKPDSNLSIR_3

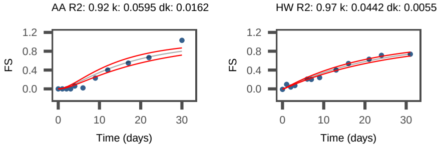

TITIN − NSDIIVPHK_2

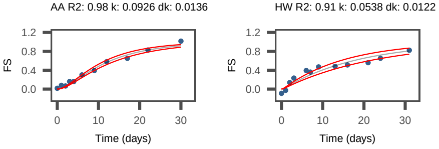

TITIN − PIYDGGCEIQGYIVEK_2

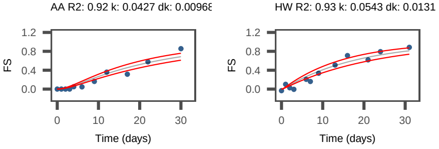

TITIN − QEAQFTLTVQK_2

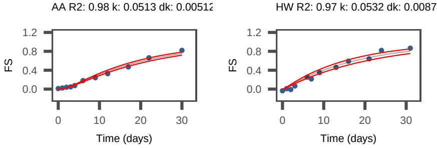

TITIN – QEASFSSFSSSSASSMTEMK_2

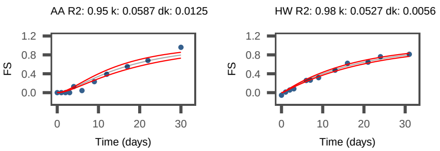

TITIN – RTEEGYYEAITAVELK_3

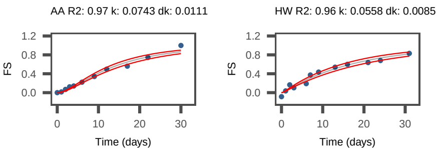

TITIN – SIHEQVSSISETTK_2

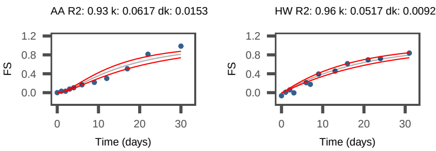

TITIN – QEHIQVTHGQVGVGK_4

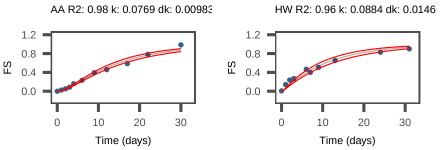

TITIN – SDIGQYTCDCGTDQTSGK_2

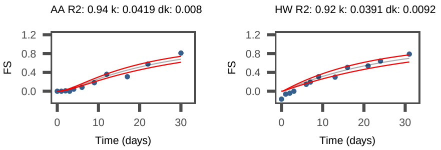

TITIN – SIHEQVSSISETTK_3

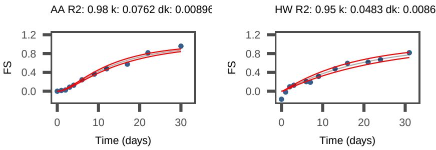

TITIN – QLITFTQELQDVVAK_2

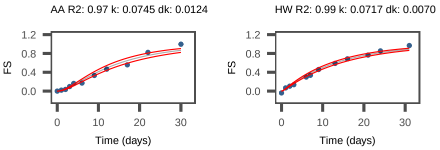

TITIN – SDTGLYSITAVNNLGTASK_2

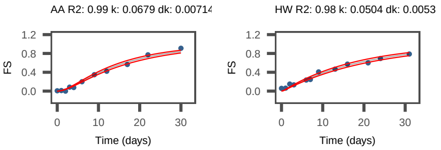

TITIN – SISGEINVNVIAPPSAPK_2

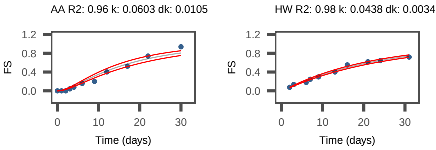

TITIN – QLSVPVIAK_2

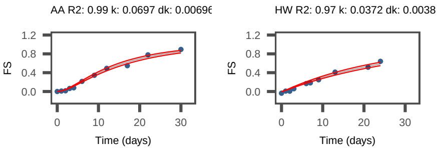

TITIN – SDVPIQAPHFK_3

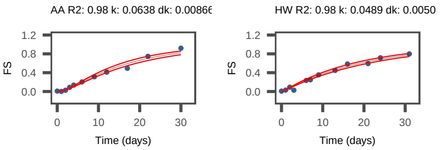

TITIN – SPTPPSIAAK_2

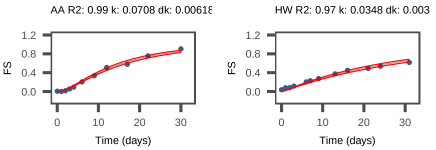

TITIN – RLDVVDTSK_2

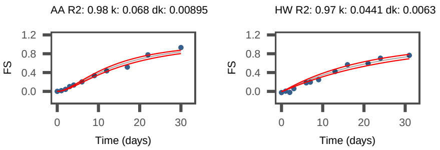

TITIN – SELDYMSK_2

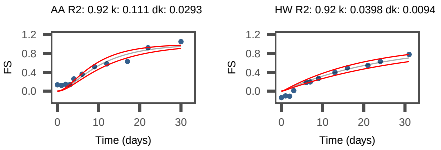

TITIN – SSAVLAWLKPDHDGGSR_3

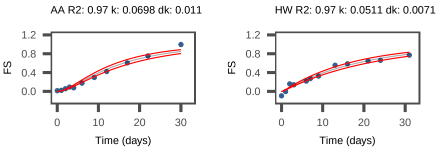

TITIN – RPGPPSTPEASAITK_3

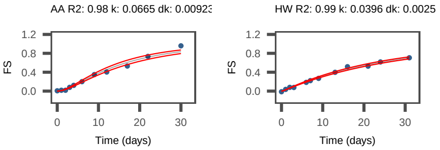

TITIN – SHQGVYSLLAK_3

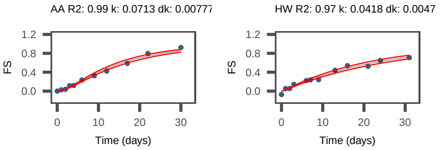

TITIN – SSVFLSWTK_2

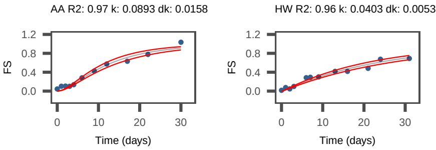

TITIN – SSWEFDGK_2

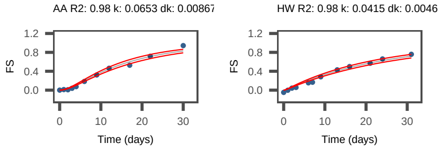

TITIN – TPILAINPIDRPGEPENLHIADK_4

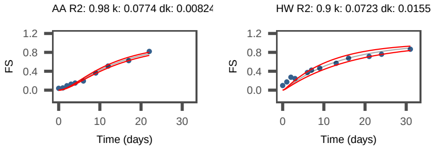

TITIN – VAAENAIGQSDYTEIGDSVLAK_2

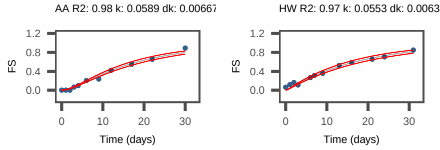

TITIN – STMVVSWQVPVNDGGSQVIGYHLEYK_3

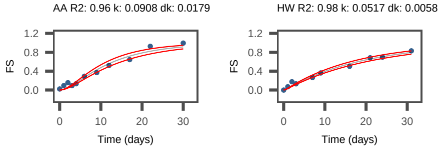

TITIN – TQVVDCGEYTCK_2

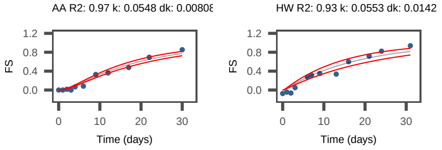

TITIN – VAAENMYGVGEPVQAAPIIAK_2

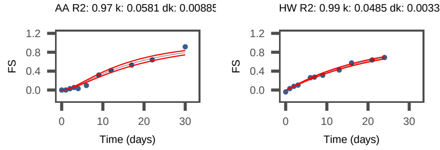

TITIN – SVDDSEVGTGVTIGAQK_2

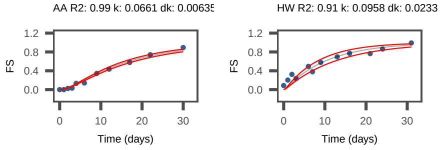

TITIN – TSCHVSWAPPENDGGSQVTHYIVEK_4

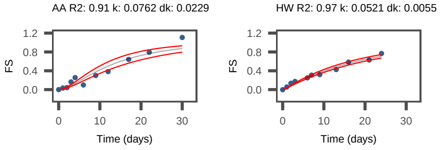

TITIN – VCAVNAAGVGPFSEPSDFYK_2

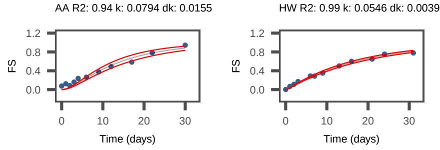

TITIN – TCEIEIGQLK_2

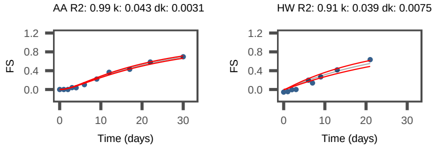

TITIN – TSSDVSLHGSVSSQSVQMSASK_3

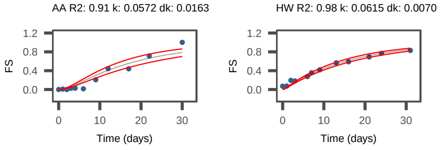

TITIN – VEKPLYGVEVFVGETAR_3

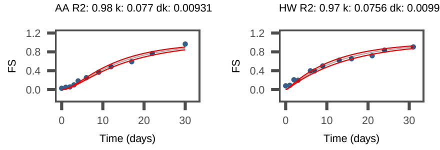

TITIN – TEAYAVSSFK_2

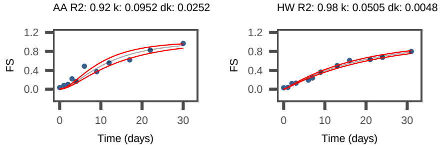

TITIN – TTPTTLALEK_2

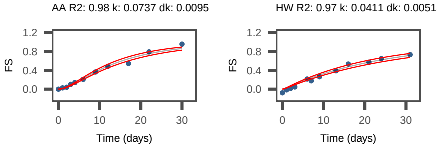

TITIN – VETSCNLSVEK_2

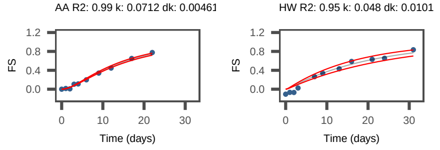

TITIN – TGGSPITGYHIEFK_3

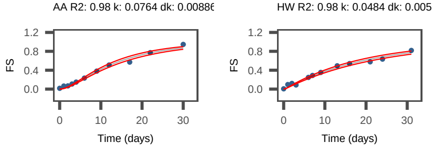

TITIN – TVLMSSEGK_2

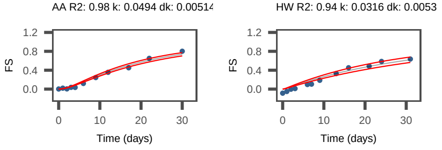

TITIN – VGVGPTIETK_2

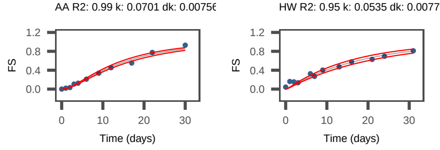

TITIN − VLACNAGGPGEPAEVPGTVK_2

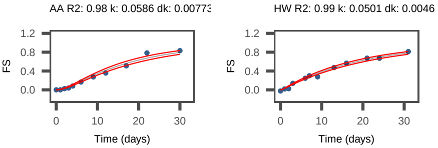

TITIN − VLDRPGPPEGPVQVTGVTAEK_3

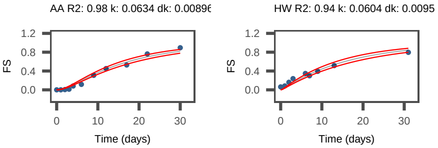

TITIN − VLGIPVIAK_2

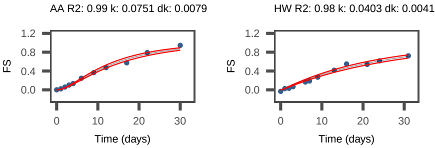

TITIN − VLAENEIGIGEPCETTEPVK_2

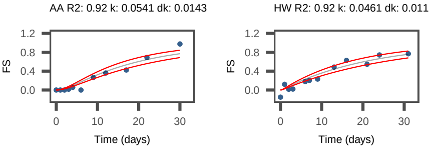

TITIN − VLDTPAACQK_2

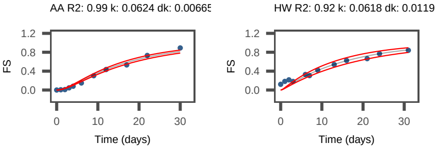

TITIN − VLIAEEVKR_3

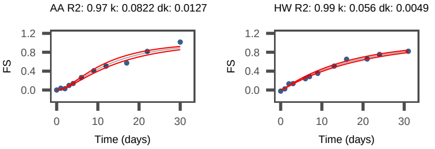

TITIN − VLAENEYGIGLPAETAESVK_2

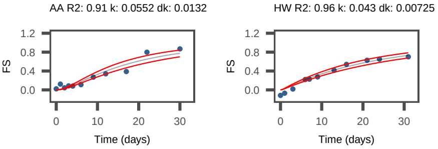

TITIN − VLDTPGPPQNLAVK_2

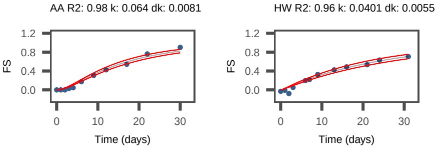

TITIN − VLPTIDLSTMPQK_2

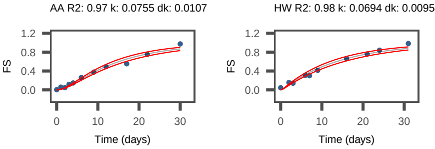

TITIN − VLASNEYGIGLPAETAEPVK_2

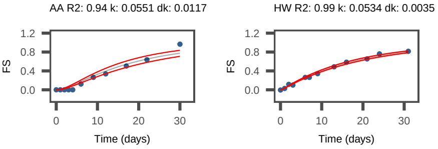

TITIN − VLDTPGPPQNLK_2

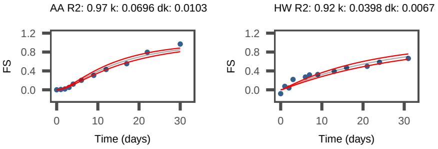

TITIN − VLVLDKPGPPR_3

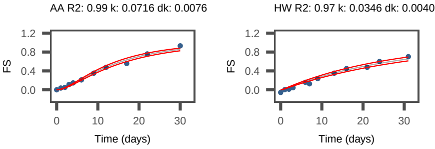

TITIN − VLDRPGPPEGPLAVSDVTSEK_3

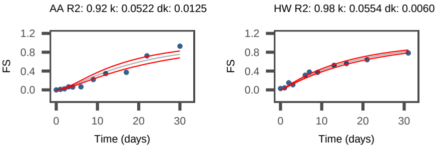

TITIN − VLDTPGPVLNLRPTDITK_3

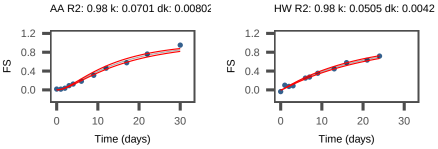

TITIN − VMAENEFGVGVPTETSDAVK_2

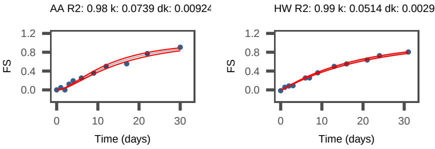

TITIN − VLDRPGPPEGPVAISGVTAEK_3

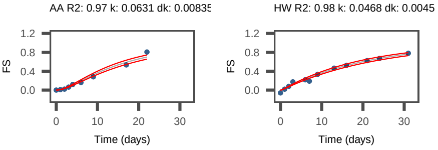

TITIN − VLDTPSPPVNLK_2

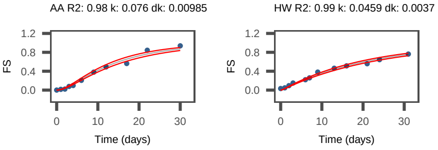

TITIN − VMDFVTDLEFTVPDLVQGK_2

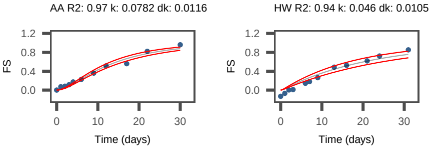

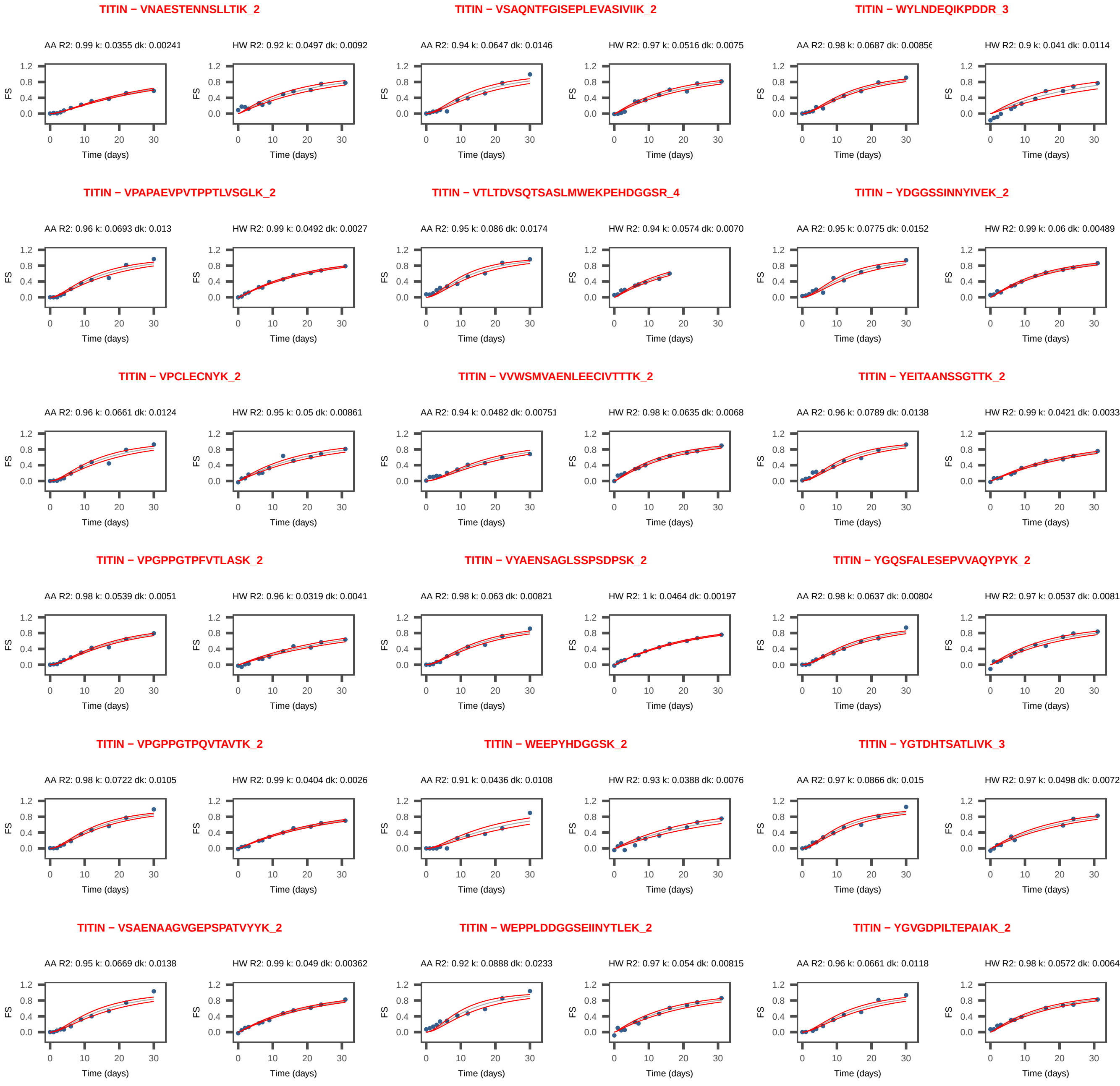
TITIN − VNAESTENNSLLTIK_2
AA R2: 0.99 k: 0.0355 dk: 0.00241
HW R2: 0.92 k: 0.0497 dk: 0.0092
TITIN − VSAQNTFGISEPLEVASIVIIK_2
AA R2: 0.94 k: 0.0647 dk: 0.0146
HW R2: 0.97 k: 0.0516 dk: 0.0075
TITIN − WYLNDEQIKPDDR_3
AA R2: 0.98 k: 0.0687 dk: 0.0085
HW R2: 0.9 k: 0.041 dk: 0.0114
TITIN − VPAPAEVPVTPPTLVSGLK_2
AA R2: 0.96 k: 0.0693 dk: 0.013
HW R2: 0.99 k: 0.0492 dk: 0.0027
TITIN − VTLTDVSQTSASLMWEKPEHDGGSR_4
AA R2: 0.95 k: 0.086 dk: 0.0174
HW R2: 0.94 k: 0.0574 dk: 0.0070
TITIN − YDGGSSINNYIVEK_2
AA R2: 0.95 k: 0.0775 dk: 0.0152
HW R2: 0.99 k: 0.06 dk: 0.00489
TITIN − VPCLECNYK_2
AA R2: 0.96 k: 0.0661 dk: 0.0124
HW R2: 0.95 k: 0.05 dk: 0.00861
TITIN − VVWSMVAENLEECIVTTTK_2
AA R2: 0.94 k: 0.0482 dk: 0.00751
HW R2: 0.98 k: 0.0635 dk: 0.0068
TITIN − YEITAANSSGTTK_2
AA R2: 0.96 k: 0.0789 dk: 0.0138
HW R2: 0.99 k: 0.0421 dk: 0.0033
TITIN − VPGPPGTPFVTLASK_2
AA R2: 0.98 k: 0.0539 dk: 0.0051
HW R2: 0.96 k: 0.0319 dk: 0.0041
TITIN − VYAENSAGLSSPSDPSK_2
AA R2: 0.98 k: 0.063 dk: 0.00821
HW R2: 1 k: 0.0464 dk: 0.00197
TITIN − YGQSFALESEPVVAQYPYK_2
AA R2: 0.98 k: 0.0637 dk: 0.00804
HW R2: 0.97 k: 0.0537 dk: 0.0081
TITIN − VPGPPGTPQVTAVTK_2
AA R2: 0.98 k: 0.0722 dk: 0.0105
HW R2: 0.99 k: 0.0404 dk: 0.0026
TITIN − WEEPYHDGGSK_2
AA R2: 0.91 k: 0.0436 dk: 0.0108
HW R2: 0.93 k: 0.0388 dk: 0.0076
TITIN − YGTDHTSATLIVK_3
AA R2: 0.97 k: 0.0866 dk: 0.015
HW R2: 0.97 k: 0.0498 dk: 0.0072
TITIN − VSAENAAGVGEPSPATVYYK_2
AA R2: 0.95 k: 0.0669 dk: 0.0138
HW R2: 0.99 k: 0.049 dk: 0.00362
TITIN − WEPPLDDGGSEIINYTLEK_2
AA R2: 0.92 k: 0.0888 dk: 0.0233
HW R2: 0.97 k: 0.054 dk: 0.00815
TITIN − YGVGDPILTEPAIAK_2
AA R2: 0.96 k: 0.0661 dk: 0.0118
HW R2: 0.98 k: 0.0572 dk: 0.0064
FS
Time (days)

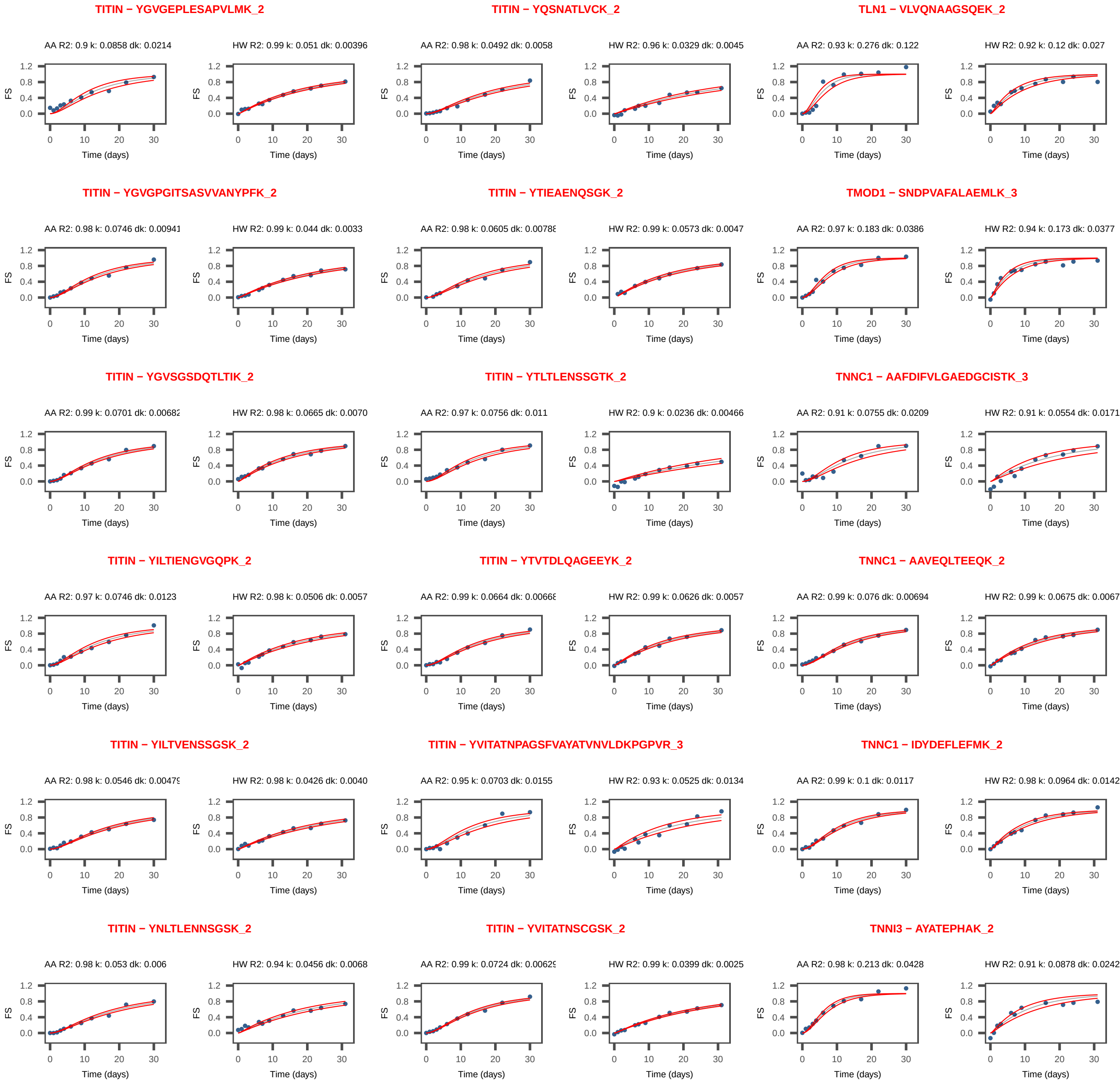
TITIN − YGVGEPLESAPVLMK_2
AA R2: 0.9 k: 0.0858 dk: 0.0214
HW R2: 0.99 k: 0.051 dk: 0.00396
TITIN − YQSNATLVCK_2
AA R2: 0.98 k: 0.0492 dk: 0.0058
HW R2: 0.96 k: 0.0329 dk: 0.0045
TLN1 − VLVQNAAGSQEK_2
AA R2: 0.93 k: 0.276 dk: 0.122
HW R2: 0.92 k: 0.12 dk: 0.027
TITIN − YGVGPGITSASVVANYPFK_2
AA R2: 0.98 k: 0.0746 dk: 0.00941
HW R2: 0.99 k: 0.044 dk: 0.0033
TITIN − YTIEAENQSGK_2
AA R2: 0.98 k: 0.0605 dk: 0.0078
HW R2: 0.99 k: 0.0573 dk: 0.0047
TMOD1 − SNDPVAFALAEMLK_3
AA R2: 0.97 k: 0.183 dk: 0.0386
HW R2: 0.94 k: 0.173 dk: 0.0377
TITIN − YGVSGSDQTLTIK_2
AA R2: 0.99 k: 0.0701 dk: 0.00682
HW R2: 0.98 k: 0.0665 dk: 0.0070
TITIN − YTLTLENSSGTK_2
AA R2: 0.97 k: 0.0756 dk: 0.011
HW R2: 0.9 k: 0.0236 dk: 0.00466
TNNC1 − AAFDIFVLGAEDGCISTK_3
AA R2: 0.91 k: 0.0755 dk: 0.0209
HW R2: 0.91 k: 0.0554 dk: 0.0171
TITIN − YILTIENGVGQPK_2
AA R2: 0.97 k: 0.0746 dk: 0.0123
HW R2: 0.98 k: 0.0506 dk: 0.0057
TITIN − YTVTDLQAGEEYK_2
AA R2: 0.99 k: 0.0664 dk: 0.0066
HW R2: 0.99 k: 0.0626 dk: 0.0057
TNNC1 − AAVEQLTEEQK_2
AA R2: 0.99 k: 0.076 dk: 0.00694
HW R2: 0.99 k: 0.0675 dk: 0.0067
TITIN − YILTVENSSGSK_2
AA R2: 0.98 k: 0.0546 dk: 0.0047
HW R2: 0.98 k: 0.0426 dk: 0.0040
TITIN − YVITATNPAGSFVAYATVNVLDKPGPVR_3
AA R2: 0.95 k: 0.0703 dk: 0.0155
HW R2: 0.93 k: 0.0525 dk: 0.0134
TNNC1 − IDYDEFLEFMK_2
AA R2: 0.99 k: 0.1 dk: 0.0117
HW R2: 0.98 k: 0.0964 dk: 0.0142
TITIN − YNLTLENNSGSK_2
AA R2: 0.98 k: 0.053 dk: 0.006
HW R2: 0.94 k: 0.0456 dk: 0.0068
TITIN − YVITATNSCGSK_2
AA R2: 0.99 k: 0.0724 dk: 0.0062
HW R2: 0.99 k: 0.0399 dk: 0.0025
TNNI3 − AYATEPHAK_2
AA R2: 0.98 k: 0.213 dk: 0.0428
HW R2: 0.91 k: 0.0878 dk: 0.0242
FS
Time (days)

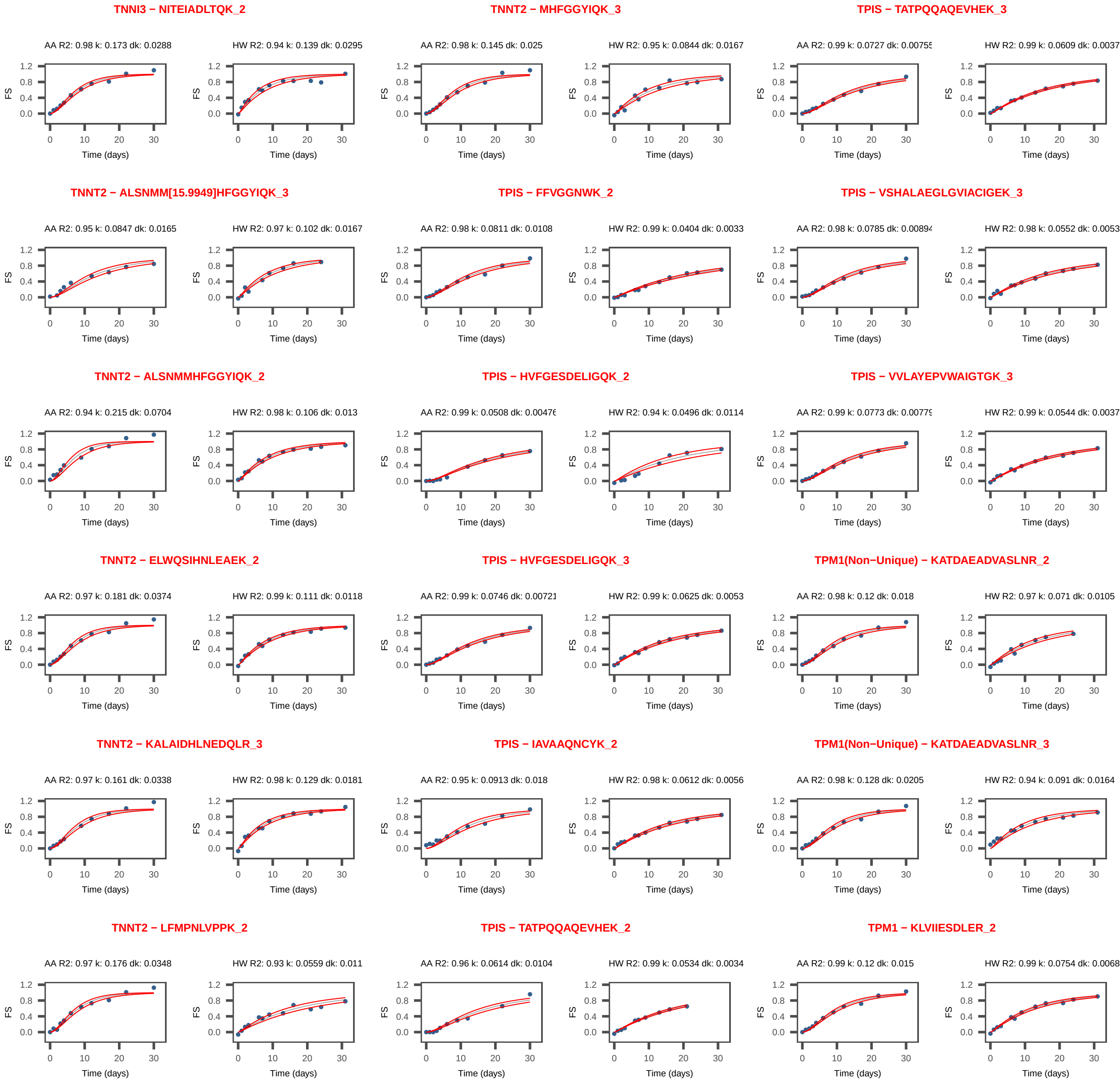
TNNI3 − NITEIADLTQK_2
AA R2: 0.98 k: 0.173 dk: 0.0288
HW R2: 0.94 k: 0.139 dk: 0.0295
TNNT2 − MHFGGYIQK_3
AA R2: 0.98 k: 0.145 dk: 0.025
HW R2: 0.95 k: 0.0844 dk: 0.0167
TPIS − TATPQQAQEVHEK_3
AA R2: 0.99 k: 0.0727 dk: 0.00755
HW R2: 0.99 k: 0.0609 dk: 0.0037
TNNT2 − ALSNMM[15.9949]HFGGYIQK_3
AA R2: 0.95 k: 0.0847 dk: 0.0165
HW R2: 0.97 k: 0.102 dk: 0.0167
TPIS − FFVGGNWK_2
AA R2: 0.98 k: 0.0811 dk: 0.0108
HW R2: 0.99 k: 0.0404 dk: 0.0033
TPIS − VSHALAEGLGVIACIGEK_3
AA R2: 0.98 k: 0.0785 dk: 0.00894
HW R2: 0.98 k: 0.0552 dk: 0.0053
TNNT2 − ALSNMMHFGGYIQK_2
AA R2: 0.94 k: 0.215 dk: 0.0704
HW R2: 0.98 k: 0.106 dk: 0.013
TPIS − HVFGESDELIGQK_2
AA R2: 0.99 k: 0.0508 dk: 0.00476
HW R2: 0.94 k: 0.0496 dk: 0.0114
TPIS − VVLAYEPVWAIGTGK_3
AA R2: 0.99 k: 0.0773 dk: 0.00779
HW R2: 0.99 k: 0.0544 dk: 0.0037
TNNT2 − ELWQSIHNLEAEK_2
AA R2: 0.97 k: 0.181 dk: 0.0374
HW R2: 0.99 k: 0.111 dk: 0.0118
TPIS − HVFGESDELIGQK_3
AA R2: 0.99 k: 0.0746 dk: 0.00721
HW R2: 0.99 k: 0.0625 dk: 0.0053
TPM1(Non−Unique) − KATDAEADVASLNR_2
AA R2: 0.98 k: 0.12 dk: 0.018
HW R2: 0.97 k: 0.071 dk: 0.0105
TNNT2 − KALAIDHLNEDQLR_3
AA R2: 0.97 k: 0.161 dk: 0.0338
HW R2: 0.98 k: 0.129 dk: 0.0181
TPIS − IAVAAQNCYK_2
AA R2: 0.95 k: 0.0913 dk: 0.018
HW R2: 0.98 k: 0.0612 dk: 0.0056
TPM1(Non−Unique) − KATDAEADVASLNR_3
AA R2: 0.98 k: 0.128 dk: 0.0205
HW R2: 0.94 k: 0.091 dk: 0.0164
TNNT2 − LFMPNLVPPK_2
AA R2: 0.97 k: 0.176 dk: 0.0348
HW R2: 0.93 k: 0.0559 dk: 0.011
TPIS − TATPQQAQEVHEK_2
AA R2: 0.96 k: 0.0614 dk: 0.0104
HW R2: 0.99 k: 0.0534 dk: 0.0034
TPM1 − KLVIIESDLER_2
AA R2: 0.99 k: 0.12 dk: 0.015
HW R2: 0.99 k: 0.0754 dk: 0.0068
FS
Time (days)

TPM1 − KLVIIESDLER_3

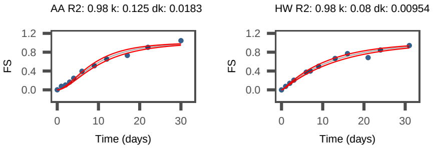

TPM3(Non−Unique) − LATALQK_2

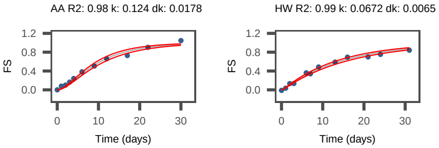

TRFE − SAGWVIPIGLLFCK_2

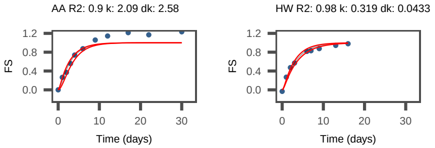

TPM1(Non−Unique) − LDKENALDR_2

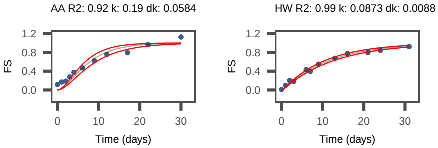

TPM3(Non−Unique) − MELQEIQLK_2

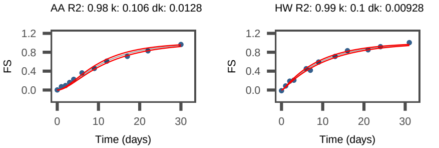

TRI72 − LPGPVYPIFDVCWHDK_3

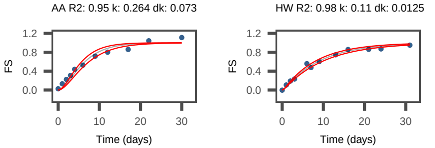

TPM1 − QLEDELVSLQK_2

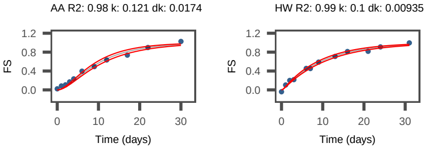

TRFE − ASDTSITWNNLK_2

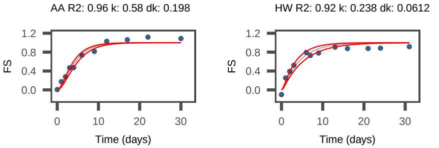

TTHY − TSEGSWEPFASGK_2

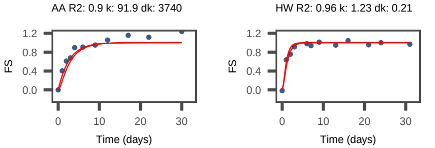

TPM1 − SIDDLEDELYAQK_2

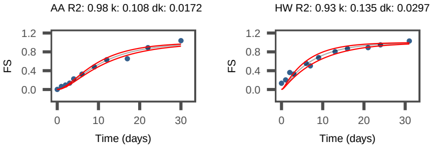

TRFE − EDLIWEILK_2

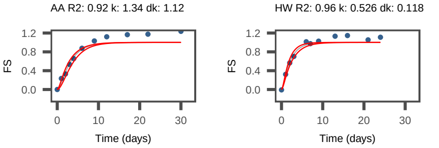

UBA1 − AAVASLLQSVQVPEFTPK_3

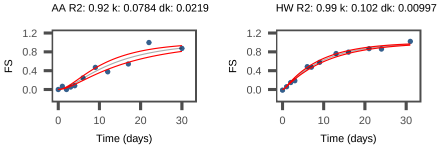

TPM1 − SIDDLEDELYAQK_3

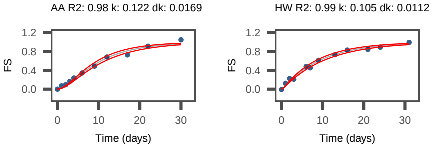

TRFE − GTDFQLNQLEGK_2

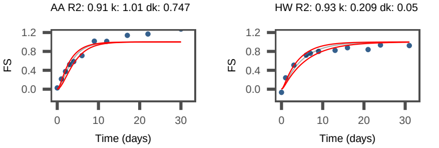

UBA1Y(Non−Unique) − DEFEGLFK_2

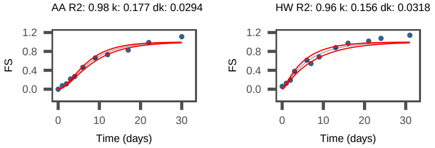

TPM3(Non−Unique) − KYEEVAR_2

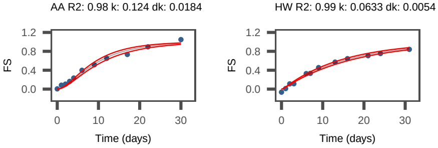

TRFE − GYYAVAVVK_2

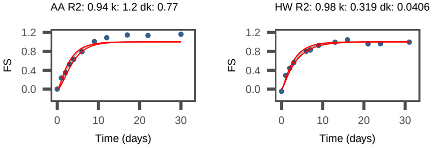

UBB(Non−Unique) − TITLEVEPSDTIENVK_2

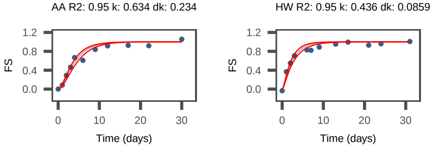

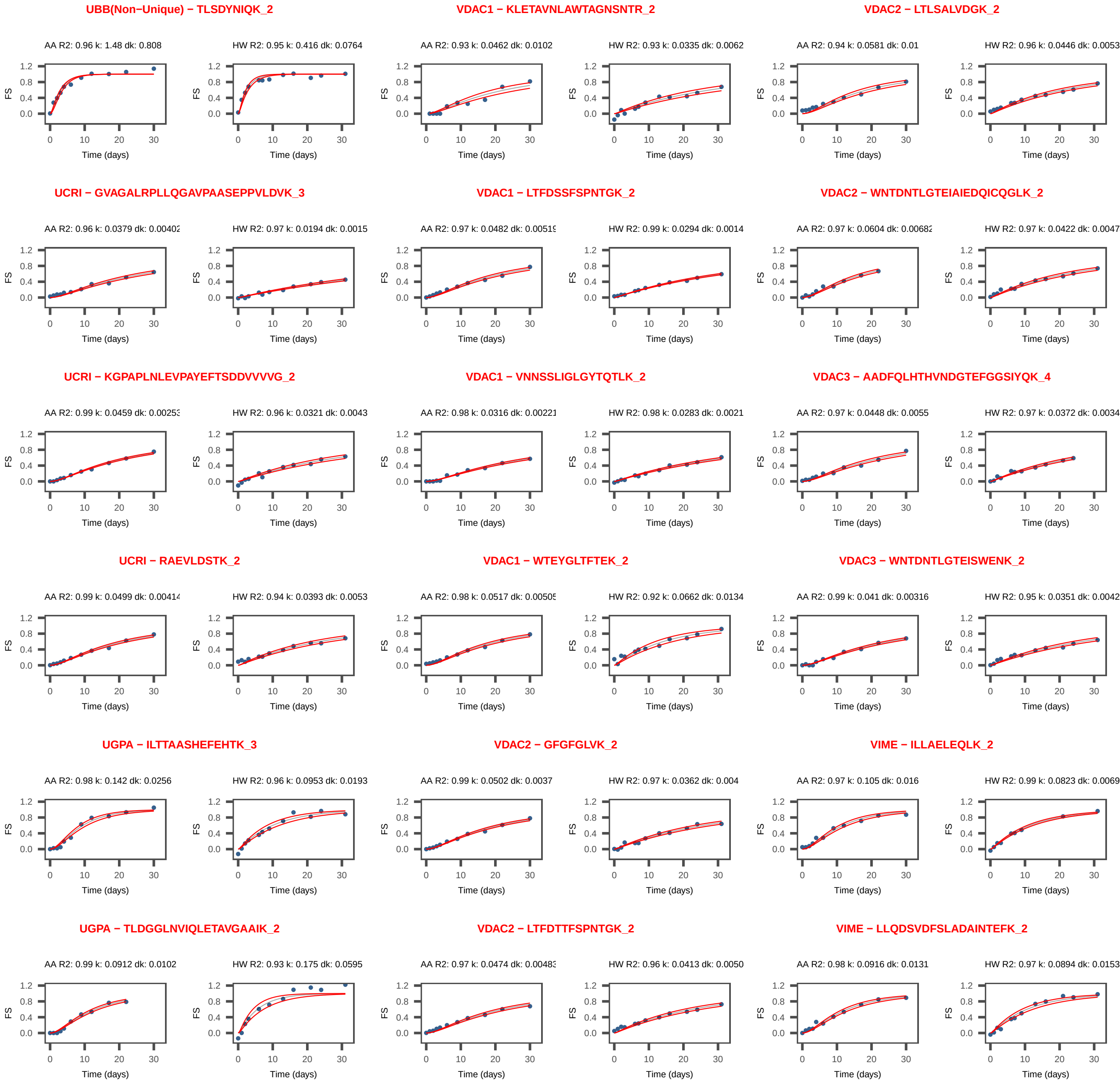
UBB(Non−Unique) − TLSDYNIQK_2
AA R2: 0.96 k: 1.48 dk: 0.808
HW R2: 0.95 k: 0.416 dk: 0.0764
VDAC1 − KLETAVNLAWTAGNSNTR_2
AA R2: 0.93 k: 0.0462 dk: 0.0102
HW R2: 0.93 k: 0.0335 dk: 0.0062
VDAC2 − LTLSALVDGK_2
AA R2: 0.94 k: 0.0581 dk: 0.01
HW R2: 0.96 k: 0.0446 dk: 0.0053
UCRI − GVAGALRPLLQGAVPAASEPPVLDVK_3
AA R2: 0.96 k: 0.0379 dk: 0.00402
HW R2: 0.97 k: 0.0194 dk: 0.0015
VDAC1 − LTFDSSFSPNTGK_2
AA R2: 0.97 k: 0.0482 dk: 0.00519
HW R2: 0.99 k: 0.0294 dk: 0.0014
VDAC2 − WNTDNTLGTEIAIEDQICQGLK_2
AA R2: 0.97 k: 0.0604 dk: 0.00682
HW R2: 0.97 k: 0.0422 dk: 0.0047
UCRI − KGPAPLNLEVPAYEFTSDDVVVVG_2
AA R2: 0.99 k: 0.0459 dk: 0.00253
HW R2: 0.96 k: 0.0321 dk: 0.0043
VDAC1 − VNNSSLIGLGYTQTLK_2
AA R2: 0.98 k: 0.0316 dk: 0.00221
HW R2: 0.98 k: 0.0283 dk: 0.0021
VDAC3 − AADFQLHTHVNDGTEFGGSIYQK_4
AA R2: 0.97 k: 0.0448 dk: 0.0055
HW R2: 0.97 k: 0.0372 dk: 0.0034
UCRI − RAEVLDSTK_2
AA R2: 0.99 k: 0.0499 dk: 0.00414
HW R2: 0.94 k: 0.0393 dk: 0.0053
VDAC1 − WTEYGLTFTEK_2
AA R2: 0.98 k: 0.0517 dk: 0.00505
HW R2: 0.92 k: 0.0662 dk: 0.0134
VDAC3 − WNTDNTLGTEISWENK_2
AA R2: 0.99 k: 0.041 dk: 0.00316
HW R2: 0.95 k: 0.0351 dk: 0.0042
UGPA − ILTTAASHEFEHTK_3
AA R2: 0.98 k: 0.142 dk: 0.0256
HW R2: 0.96 k: 0.0953 dk: 0.0193
VDAC2 − GFGFGLVK_2
AA R2: 0.99 k: 0.0502 dk: 0.0037
HW R2: 0.97 k: 0.0362 dk: 0.004
VIME − ILLAELEQLK_2
AA R2: 0.97 k: 0.105 dk: 0.016
HW R2: 0.99 k: 0.0823 dk: 0.0069
UGPA − TLDGGLNVIQLETAVGAAIK_2
AA R2: 0.99 k: 0.0912 dk: 0.0102
HW R2: 0.93 k: 0.175 dk: 0.0595
VDAC2 − LTFDTTFSPNTGK_2
AA R2: 0.97 k: 0.0474 dk: 0.00483
HW R2: 0.96 k: 0.0413 dk: 0.0050
VIME − LLQDSVDFSLADAINTEFK_2
AA R2: 0.98 k: 0.0916 dk: 0.0131
HW R2: 0.97 k: 0.0894 dk: 0.0153
FS
Time (days)

VIME − QVQSLTCEVDALK_2

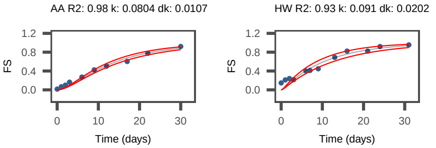

WDR1 − VINSVDIK_2

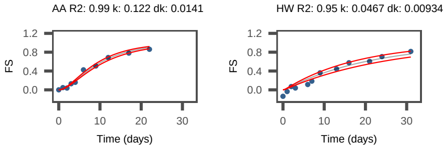

VINC − AQQVSQGLDVLTAK_2

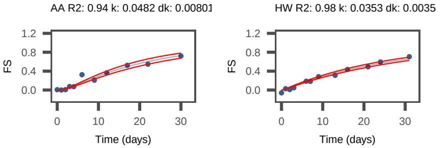

WDR1 − VYSILASTLK_2

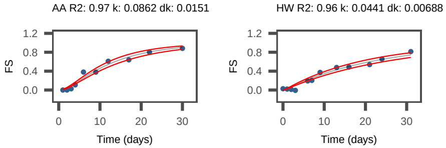

VINC − ELLPVLISAMK_2

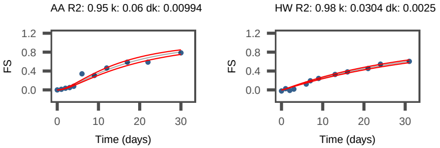

WDR1 − YAPSGFYIASGDISGK_2

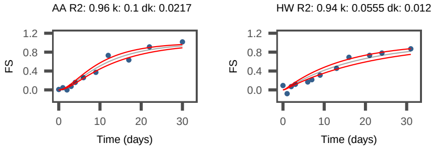

VINC − QVATALQNLQTK_2

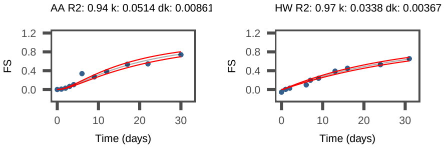

VINC − VLQLTSWDEDAWASK_2

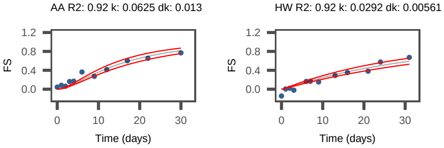

VWA8 − LLYSVGANVSAEK_2

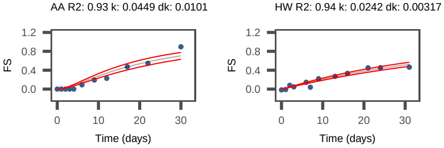

Supplement: Supplemental Data S1 [file mmc2.pdf]
