# Supplementary material for: Harmonizing Labeling and Analytical Strategies to Obtain Protein Turnover Rates in Intact Adult Animals
Source: Mol Cell Proteomics. 2022 May 28;21(7):100252. doi: 10.1016/j.mcpro.2022.100252 (PMC9249856; doi:10.1016/j.mcpro.2022.100252)
Supplement: Supplemental Data S2 [file mmc3.pdf]

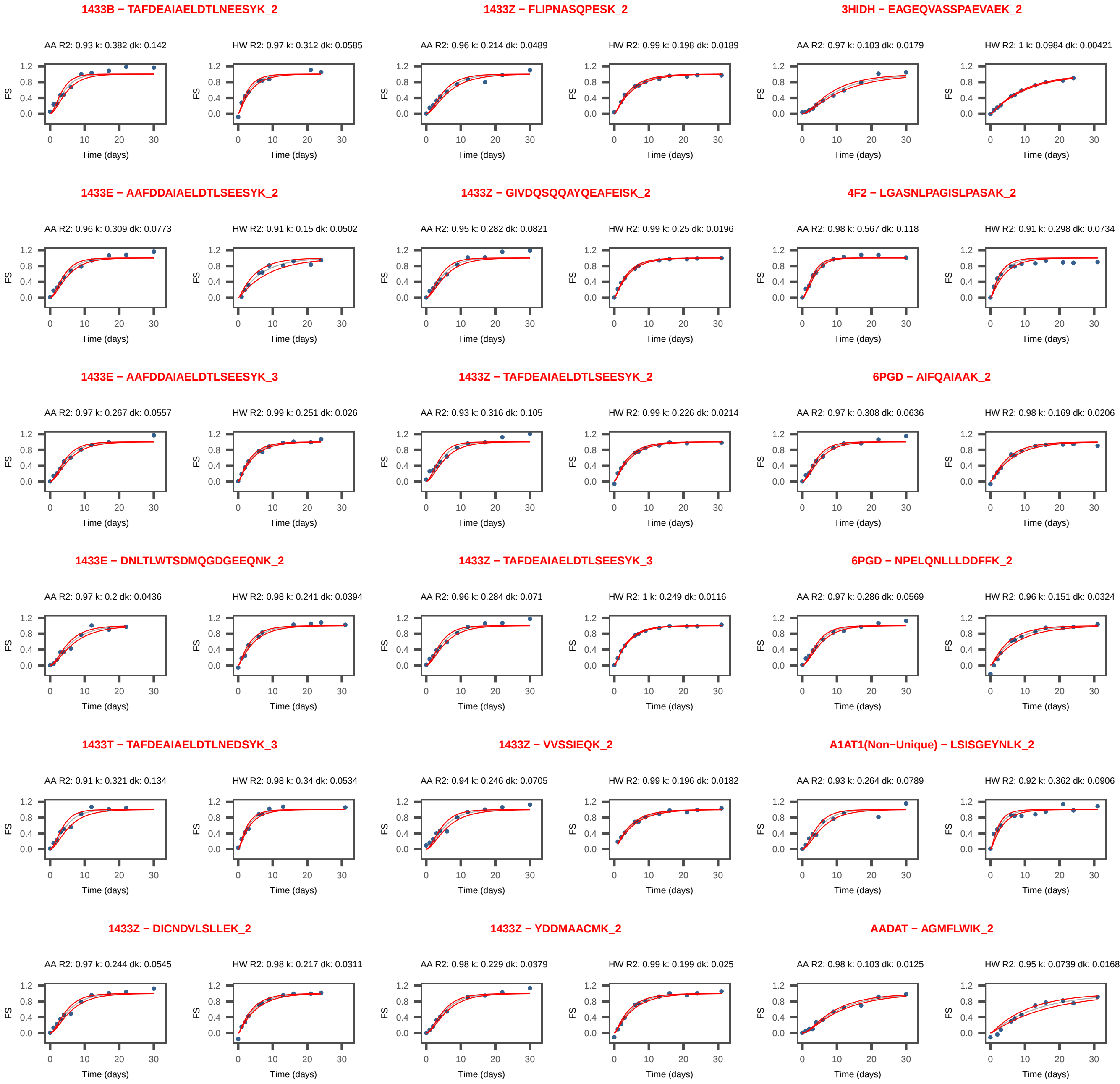
1433B − TAFDEAIAELDTLNEESYK_2
AA R2: 0.93 k: 0.382 dk: 0.142
HW R2: 0.97 k: 0.312 dk: 0.0585
1433Z − FLIPNASQPESK_2
AA R2: 0.96 k: 0.214 dk: 0.0489
HW R2: 0.99 k: 0.198 dk: 0.0189
3HIDH − EAGEQVASSPAEVAEK_2
AA R2: 0.97 k: 0.103 dk: 0.0179
HW R2: 1 k: 0.0984 dk: 0.00421
1433E − AAFDDAIAELDTLSEESYK_2
AA R2: 0.96 k: 0.309 dk: 0.0773
HW R2: 0.91 k: 0.15 dk: 0.0502
1433Z − GIVDQSQQAYQEAFEISK_2
AA R2: 0.95 k: 0.282 dk: 0.0821
HW R2: 0.99 k: 0.25 dk: 0.0196
4F2 − LGASNLPAGISLPASAK_2
AA R2: 0.98 k: 0.567 dk: 0.118
HW R2: 0.91 k: 0.298 dk: 0.0734
1433E − AAFDDAIAELDTLSEESYK_3
AA R2: 0.97 k: 0.267 dk: 0.0557
HW R2: 0.99 k: 0.251 dk: 0.026
1433Z − TAFDEAIAELDTLSEESYK_2
AA R2: 0.93 k: 0.316 dk: 0.105
HW R2: 0.99 k: 0.226 dk: 0.0214
6PGD − AIFQAIAAK_2
AA R2: 0.97 k: 0.308 dk: 0.0636
HW R2: 0.98 k: 0.169 dk: 0.0206
1433E − DNLTLWTSDMQGDGEEQNK_2
AA R2: 0.97 k: 0.2 dk: 0.0436
HW R2: 0.98 k: 0.241 dk: 0.0394
1433Z − TAFDEAIAELDTLSEESYK_3
AA R2: 0.96 k: 0.284 dk: 0.071
HW R2: 1 k: 0.249 dk: 0.0116
6PGD − NPELQNLLLDDFFK_2
AA R2: 0.97 k: 0.286 dk: 0.0569
HW R2: 0.96 k: 0.151 dk: 0.0324
1433T − TAFDEAIAELDTLNEDSYK_3
AA R2: 0.91 k: 0.321 dk: 0.134
HW R2: 0.98 k: 0.34 dk: 0.0534
1433Z − VVSSIEQK_2
AA R2: 0.94 k: 0.246 dk: 0.0705
HW R2: 0.99 k: 0.196 dk: 0.0182
A1AT1(Non−Unique) − LSISGEYNLK_2
AA R2: 0.93 k: 0.264 dk: 0.0789
HW R2: 0.92 k: 0.362 dk: 0.0906
1433Z − DICNDVLSLLEK_2
AA R2: 0.97 k: 0.244 dk: 0.0545
HW R2: 0.98 k: 0.217 dk: 0.0311
1433Z − YDDMAACMK_2
AA R2: 0.98 k: 0.229 dk: 0.0379
HW R2: 0.99 k: 0.199 dk: 0.025
AADAT − AGMFLWIK_2
AA R2: 0.98 k: 0.103 dk: 0.0125
HW R2: 0.95 k: 0.0739 dk: 0.0168
FS
Time (days)

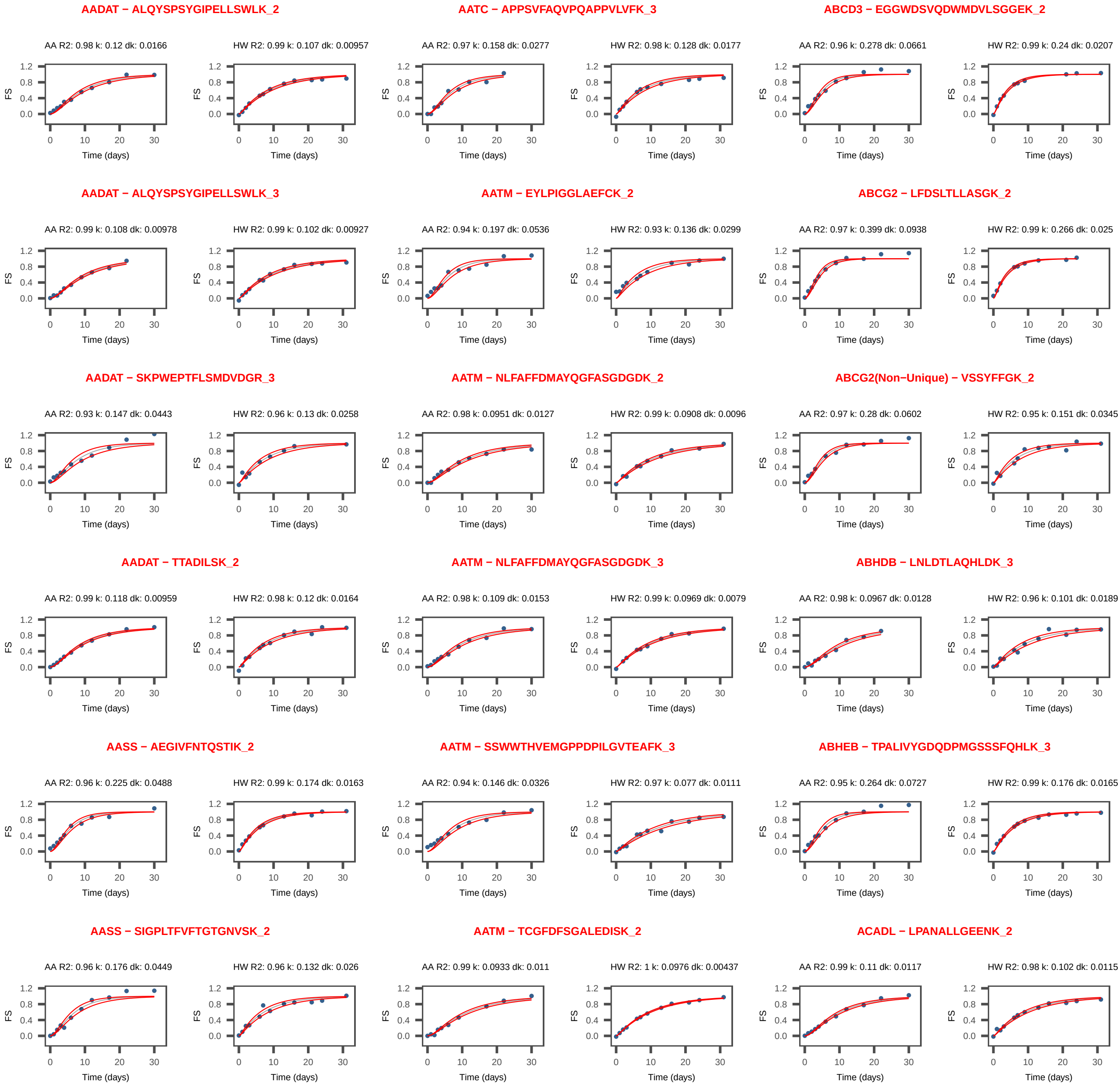
AADAT − ALQYSPSYGIPELLSWLK_2
AA R2: 0.98 k: 0.12 dk: 0.0166
HW R2: 0.99 k: 0.107 dk: 0.00957
AATC − APPSVFAQVPQAPPVLVFK_3
AA R2: 0.97 k: 0.158 dk: 0.0277
HW R2: 0.98 k: 0.128 dk: 0.0177
ABCD3 − EGGWDSVQDWMDVLSGGEK_2
AA R2: 0.96 k: 0.278 dk: 0.0661
HW R2: 0.99 k: 0.24 dk: 0.0207
AADAT − ALQYSPSYGIPELLSWLK_3
AA R2: 0.99 k: 0.108 dk: 0.00978
HW R2: 0.99 k: 0.102 dk: 0.00927
AATM − EYLPIGGLAEFCK_2
AA R2: 0.94 k: 0.197 dk: 0.0536
HW R2: 0.93 k: 0.136 dk: 0.0299
ABCG2 − LFDSLTLLASGK_2
AA R2: 0.97 k: 0.399 dk: 0.0938
HW R2: 0.99 k: 0.266 dk: 0.025
AADAT − SKPWEPTFLSMDVDGR_3
AA R2: 0.93 k: 0.147 dk: 0.0443
HW R2: 0.96 k: 0.13 dk: 0.0258
AATM − NLFAFFDMAYQGFASGDGDK_2
AA R2: 0.98 k: 0.0951 dk: 0.0127
HW R2: 0.99 k: 0.0908 dk: 0.0096
ABCG2(Non−Unique) − VSSYFFGK_2
AA R2: 0.97 k: 0.28 dk: 0.0602
HW R2: 0.95 k: 0.151 dk: 0.0345
AADAT − TTADILSK_2
AA R2: 0.99 k: 0.118 dk: 0.00959
HW R2: 0.98 k: 0.12 dk: 0.0164
AATM − NLFAFFDMAYQGFASGDGDK_3
AA R2: 0.98 k: 0.109 dk: 0.0153
HW R2: 0.99 k: 0.0969 dk: 0.0079
ABHDB − LNLDTLAQHLDK_3
AA R2: 0.98 k: 0.0967 dk: 0.0128
HW R2: 0.96 k: 0.101 dk: 0.0189
AASS − AEGIVFNTQSTIK_2
AA R2: 0.96 k: 0.225 dk: 0.0488
HW R2: 0.99 k: 0.174 dk: 0.0163
AATM − SSWWTHVEMGPPDPILGVTEAFK_3
AA R2: 0.94 k: 0.146 dk: 0.0326
HW R2: 0.97 k: 0.077 dk: 0.0111
ABHEB − TPALIVYGDQDPMGSSSFQHLK_3
AA R2: 0.95 k: 0.264 dk: 0.0727
HW R2: 0.99 k: 0.176 dk: 0.0165
AASS − SIGPLTFVFTGTGNVSK_2
AA R2: 0.96 k: 0.176 dk: 0.0449
HW R2: 0.96 k: 0.132 dk: 0.026
AATM − TCGFDFSGALEDISK_2
AA R2: 0.99 k: 0.0933 dk: 0.011
HW R2: 1 k: 0.0976 dk: 0.00437
ACADL − LPANALLGEENK_2
AA R2: 0.99 k: 0.11 dk: 0.0117
HW R2: 0.98 k: 0.102 dk: 0.0115
FS
Time (days)

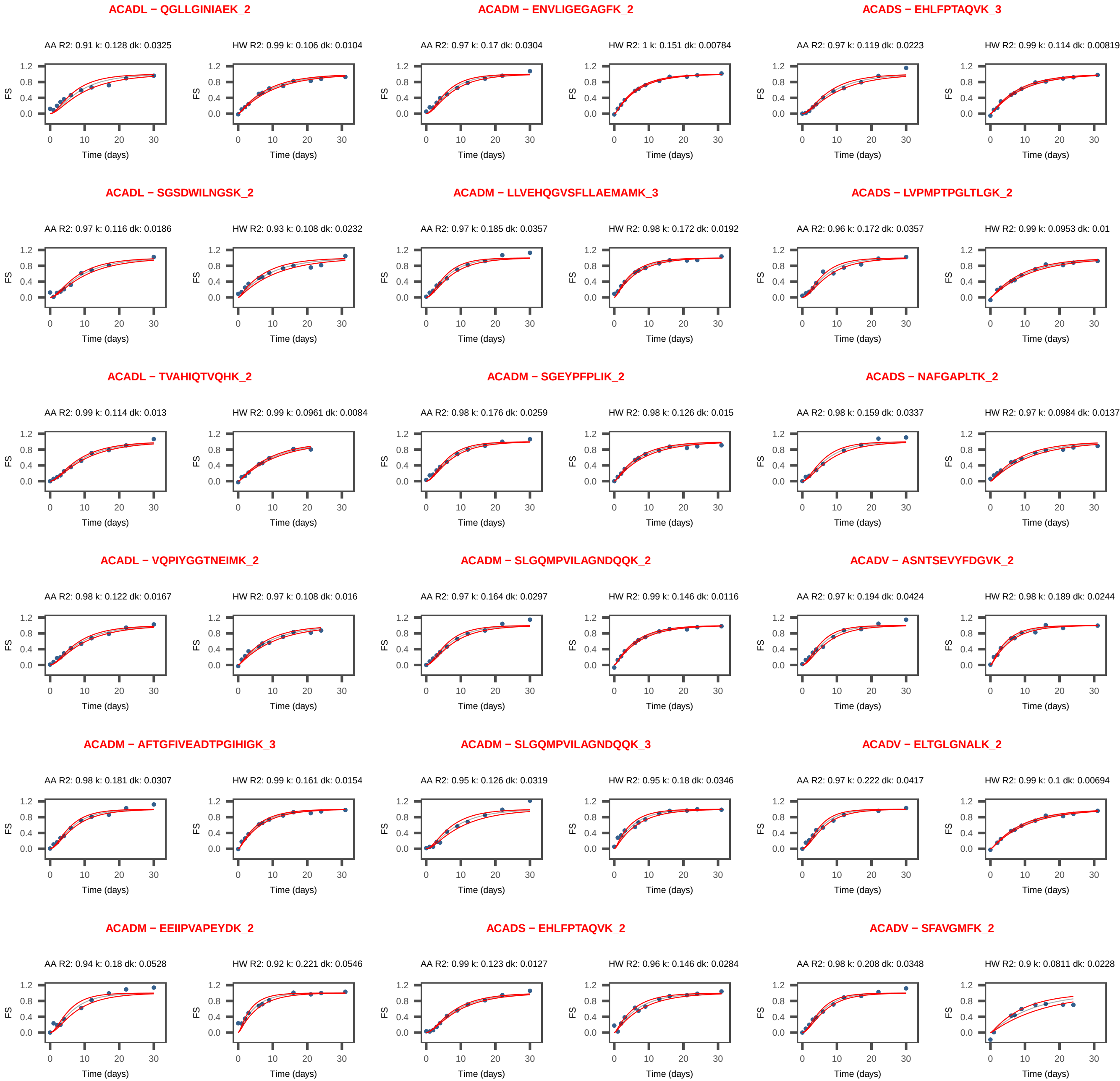
ACADL − QGLLGINIAEK_2
AA R2: 0.91 k: 0.128 dk: 0.0325
HW R2: 0.99 k: 0.106 dk: 0.0104
ACADM − ENVLIGEGAGFK_2
AA R2: 0.97 k: 0.17 dk: 0.0304
HW R2: 1 k: 0.151 dk: 0.00784
ACADS − EHLFPTAQVK_3
AA R2: 0.97 k: 0.119 dk: 0.0223
HW R2: 0.99 k: 0.114 dk: 0.00819
ACADL − SGSDWILNGSK_2
AA R2: 0.97 k: 0.116 dk: 0.0186
HW R2: 0.93 k: 0.108 dk: 0.0232
ACADM − LLVEHQGVSFLLAEMAMK_3
AA R2: 0.97 k: 0.185 dk: 0.0357
HW R2: 0.98 k: 0.172 dk: 0.0192
ACADS − LVPMPTPGLTLGK_2
AA R2: 0.96 k: 0.172 dk: 0.0357
HW R2: 0.99 k: 0.0953 dk: 0.01
ACADL − TVAHIQTVQHK_2
AA R2: 0.99 k: 0.114 dk: 0.013
HW R2: 0.99 k: 0.0961 dk: 0.0084
ACADM − SGEYPFPLIK_2
AA R2: 0.98 k: 0.176 dk: 0.0259
HW R2: 0.98 k: 0.126 dk: 0.015
ACADS − NAFGAPLTK_2
AA R2: 0.98 k: 0.159 dk: 0.0337
HW R2: 0.97 k: 0.0984 dk: 0.0137
ACADL − VQPIYGGTNEIMK_2
AA R2: 0.98 k: 0.122 dk: 0.0167
HW R2: 0.97 k: 0.108 dk: 0.016
ACADM − SLGQMPVILAGNDQQK_2
AA R2: 0.97 k: 0.164 dk: 0.0297
HW R2: 0.99 k: 0.146 dk: 0.0116
ACADV − ASNTSEVYFDGVK_2
AA R2: 0.97 k: 0.194 dk: 0.0424
HW R2: 0.98 k: 0.189 dk: 0.0244
ACADM − AFTGFIVEADTPGIHIGK_3
AA R2: 0.98 k: 0.181 dk: 0.0307
HW R2: 0.99 k: 0.161 dk: 0.0154
ACADM − SLGQMPVILAGNDQQK_3
AA R2: 0.95 k: 0.126 dk: 0.0319
HW R2: 0.95 k: 0.18 dk: 0.0346
ACADV − ELTGLGNALK_2
AA R2: 0.97 k: 0.222 dk: 0.0417
HW R2: 0.99 k: 0.1 dk: 0.00694
ACADM − EEIIPVAPEYDK_2
AA R2: 0.94 k: 0.18 dk: 0.0528
HW R2: 0.92 k: 0.221 dk: 0.0546
ACADS − EHLFPTAQVK_2
AA R2: 0.99 k: 0.123 dk: 0.0127
HW R2: 0.96 k: 0.146 dk: 0.0284
ACADV − SFAVGMFK_2
AA R2: 0.98 k: 0.208 dk: 0.0348
HW R2: 0.9 k: 0.0811 dk: 0.0228
FS
Time (days)

ACBP − QATVGDVNTDRPGLLDLK_3

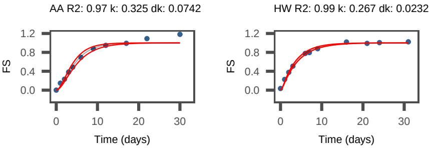

ACDSB − IGTIYEGASNIQLNTIAK_2

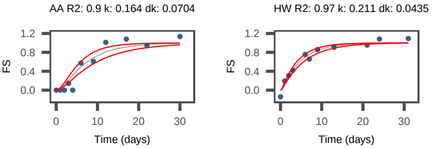

ACON − DINQEVYNFLATAGAK_2

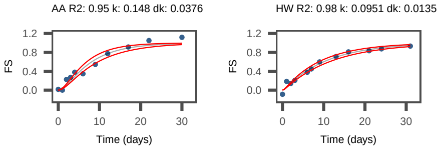

ACBP − TQPTDEEMLFIYSHFK_3

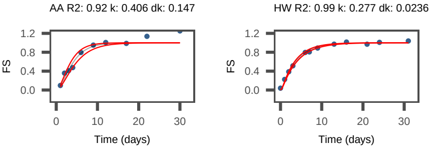

ACOC − GPFLLGIK_2

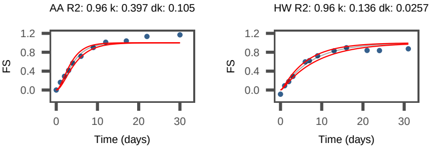

ACON − DINQEVYNFLATAGAK_3

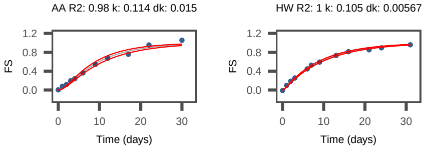

ACD10 − FADGPDEVHQLTVAK_3

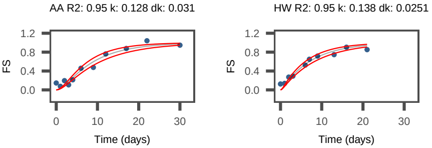

ACOC − PHPLVTSTDIVLTITK_3

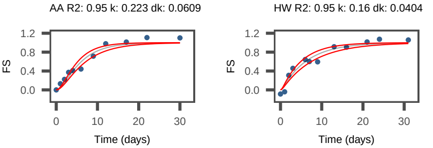

ACON − DLEDLQILIK_2

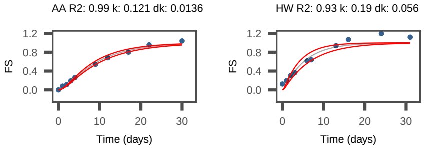

ACD10 − LTESMAELAWDFATK_3

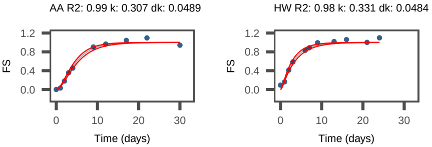

ACOC − SPPFFESLTLDLQPPK_3

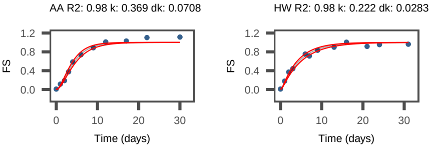

ACON − FKLEAPDADELPR_3

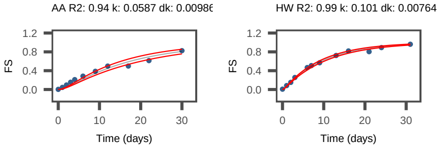

ACD11 − ALFSIGFPVAK_2

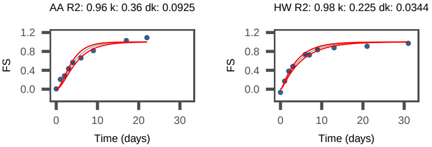

ACOC − YQQAGLPLIVLAGK_2

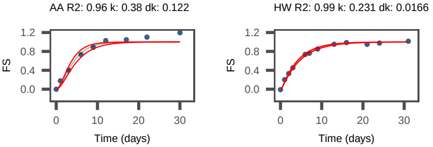

ACON − FNPETDFLTGK_2

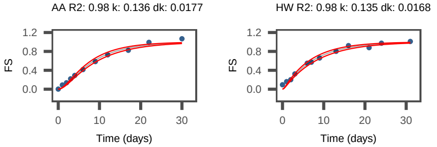

ACD11 − LDNIVFHPK_3

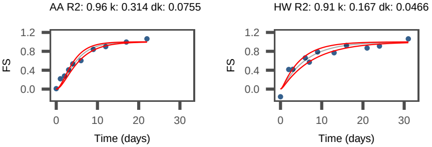

ACOC − YTINIPEDLKPR_3

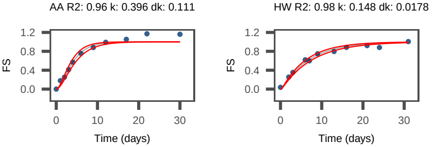

ACON − LNRPLTLSEK_2

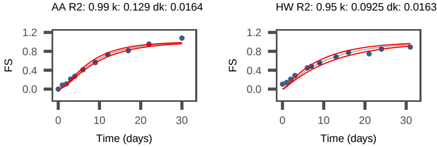

ACON − LNRPLTLSEK_3

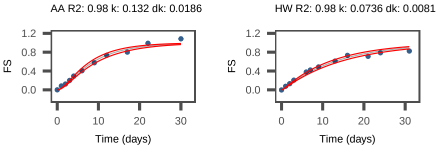

ACOT9 − NIIHELFLTTLDPK_3

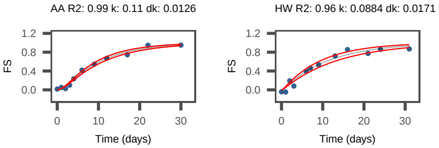

ACOX1 − SEPEPQILDFQTQQYK_3

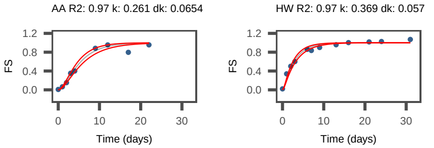

ACON − LTGSLSGWTSPK_2

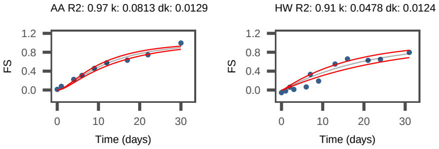

ACOX1 − ASEAHCHYVTVK_2

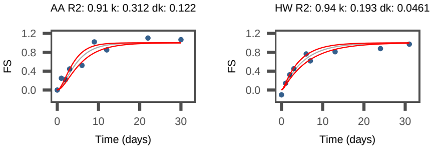

ACOX1 − TEVHESYYK_3

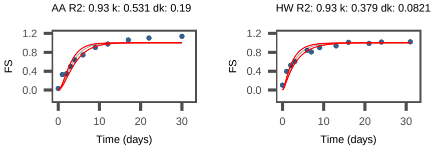

ACON − SDFDPGQDTYQHPPK_3

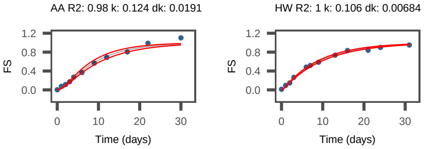

ACOX1 − EFGIADPEEIMWFK_2

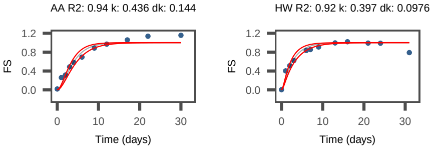

ACOX1 − TQEFILNSPTVTSIK_2

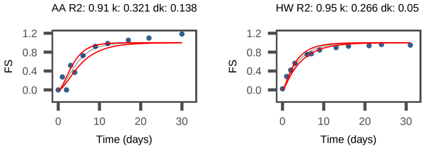

ACON − VAVPSTIHCDHLIEAQVGGEK_4

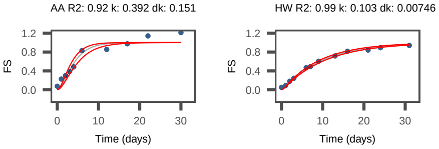

ACOX1 − ILELLTVTRPNAVALVDAFDFK_3

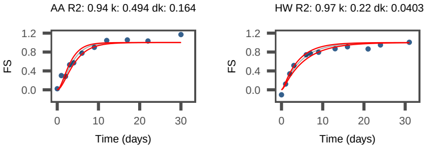

ACOX1 − YDGNVYENLFEWAK_2

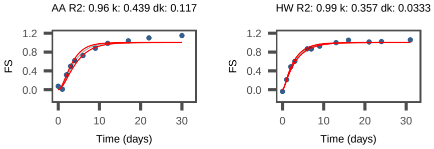

ACOT1(Non−Unique) − SDTTFLFLVGQDDHNWK_3

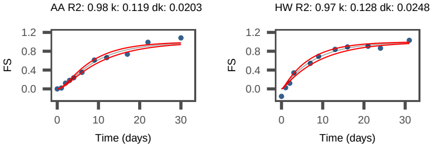

ACOX1 − LVEIAAK_2

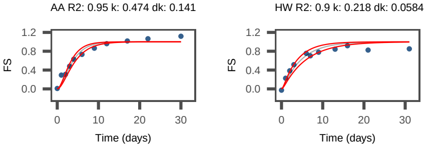

ACOX3 − TVDFLEAYPGILGQK_3

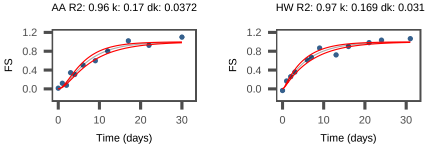

ACOT1(Non−Unique) − SEFYADEISK_2

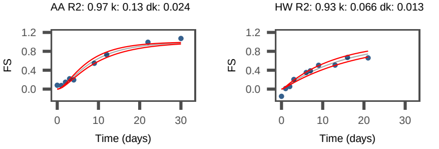

ACOX1 − SEPEPQILDFQTQQYK_2

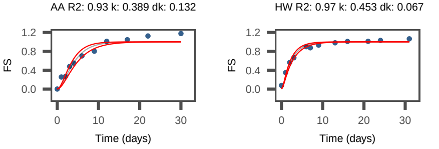

ACPM − LMCPQEIVDYIADK_2

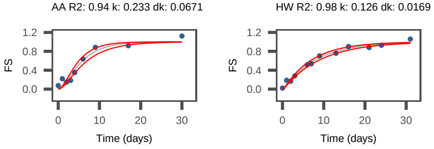

ACSA − AELGMNDSPSQSPPVK_2

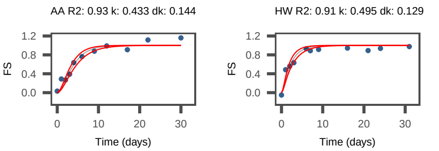

ACSM2 − EGWLNFK_2

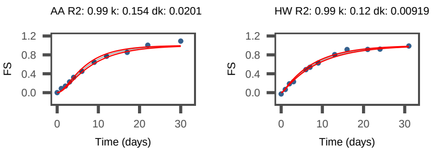

ACSM2(Non−Unique) − SVTAPYK_2

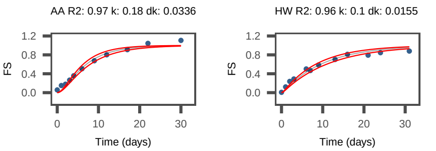

ACSM1 − AFIVLNPEFLSHDQEQLIK_3

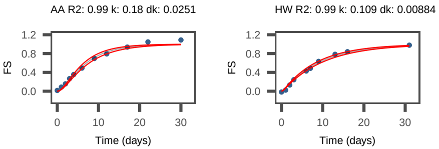

ACSM2 − FDSQTVLK_2

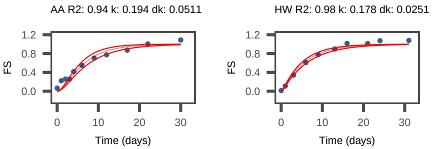

ACSM2 − VKPIWPIGMF_2

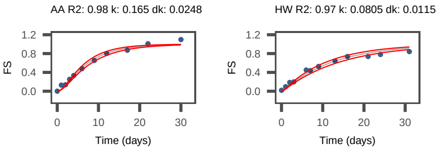

ACSM1 − TGIIFMPGTTQLK_2

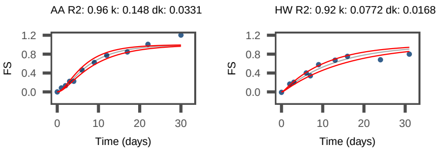

ACSM2 − FNFASDVIDHWASVEK_2

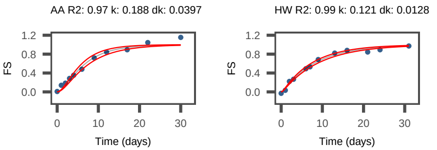

ACSM2 − VLQEHVK_2

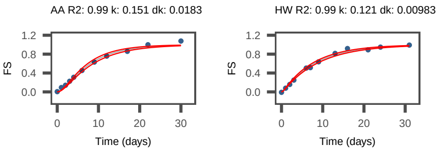

ACSM1 − VIVEVLFK_2

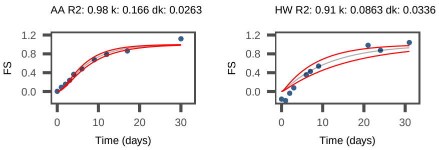

ACSM2 − MAEHSHCSLGIK_3

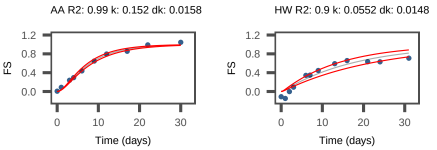

ACSM3 − HDEMMAIYFTSGTTGPPK_3

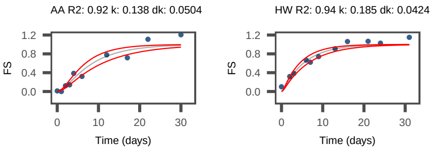

ACSM2 − AHYDVQVIDEQGNVLPPGK_3

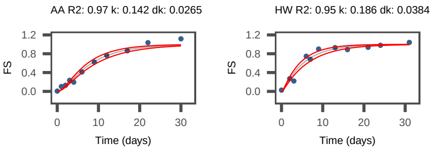

ACSM2 − PIQWGHQEAPAK_3

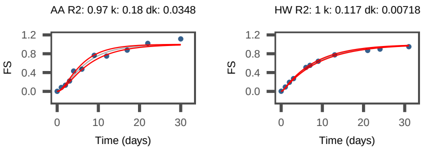

ACSM3 − MLVQNDMSSYK_2

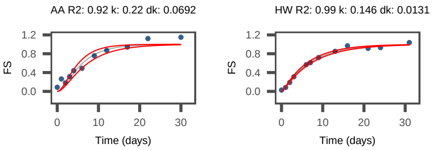

ACSM2 − AIVAGDEVAQEVDAVAPDCSFLK_2

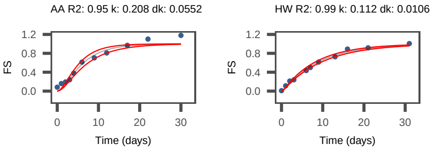

ACSM2 − PIWPIGMFSGYVDNPK_2

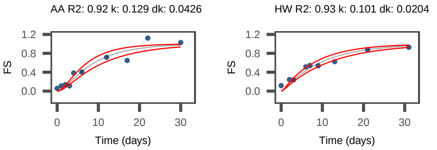

ACSM3 − TGTVLIPGTTQLTQK_2

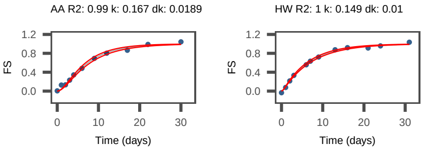

ACSM3 − YASDSHTCVDTK_2

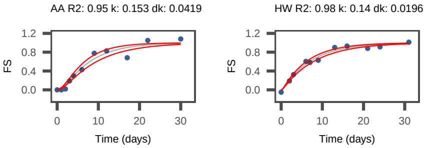

ACTB(Non−Unique) − KDLYANTVLSGGTTMYPGIADR_2

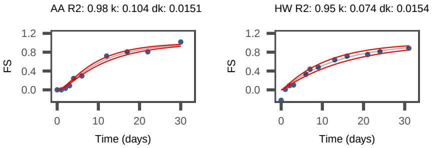

ACTN4 − EAILAIHK_2

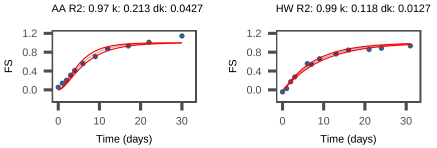

ACTA(Non−Unique) − YPIEHGIITNWDDM[15.9949]EK_3

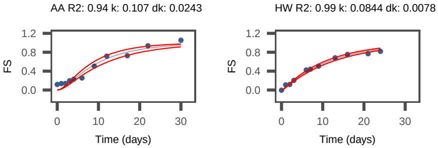

ACTB(Non−Unique) − VALDFEQEMATAASSSSLEK_2

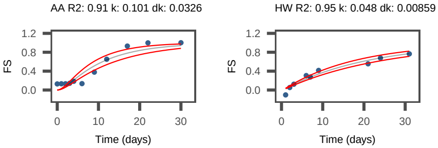

ACTN4 − ETTDTDTADQVIASFK_2

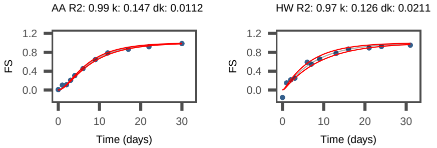

ACTA(Non−Unique) − YPIEHGIITNWDDMEK_2

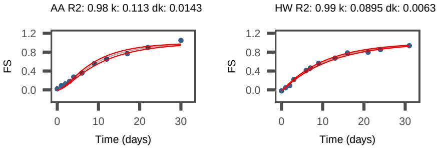

ACTB(Non−Unique) − VALDFEQEMATAASSSSLEK_3

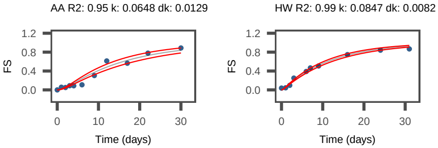

ACTN4 − HRPELIEYDK_3

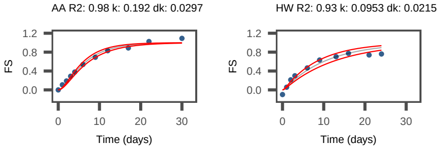

ACTB(Non−Unique) − DLTDYLM[15.9949]K_2

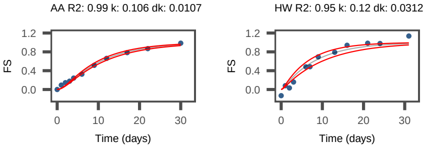

ACTB(Non−Unique) − VAPEEHPVLLTEAPLNPK_2

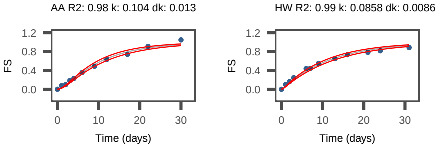

ACTN4 − MAPYQGPDAAPGALDYK_2

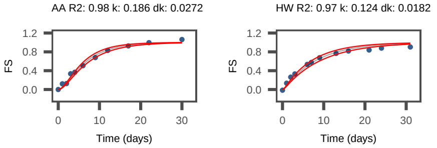

ACTB(Non−Unique) − DSYVGDEAQSK_2

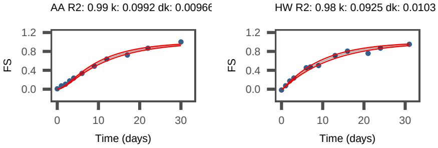

ACTB(Non−Unique) − VAPEEHPVLLTEAPLNPK_3

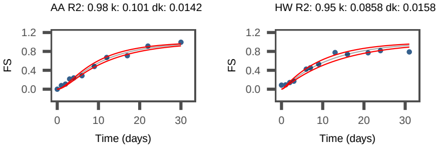

ACTN4 − RDHALLEEQSK_2

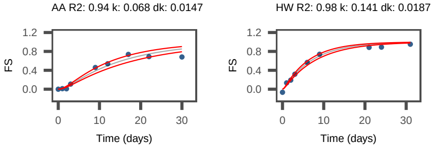

ACTB(Non−Unique) − HQGVM[15.9949]VGMGQK_3

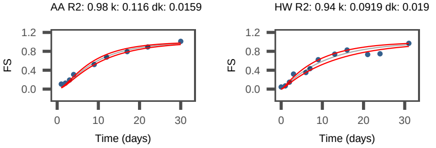

ACTN1 − LLETIDQLYLEYAK_2

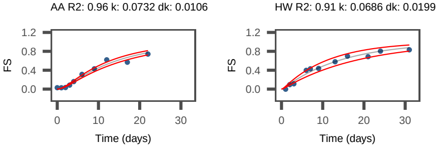

ACY3 − ELNQLLGPK_2

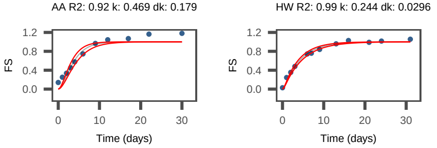

ACY3 – LFLYEPAGTETFSVESISK_3

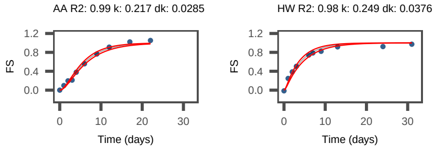

ADH1 – GAIFGGFK_2

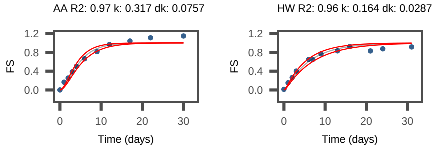

ADH1 – IIAVDINK_2

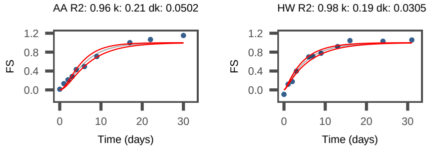

ADH1 – LVADFMAK_2

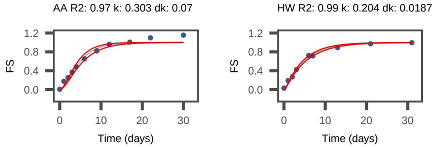

ADT1 – DEGANAFFK_2

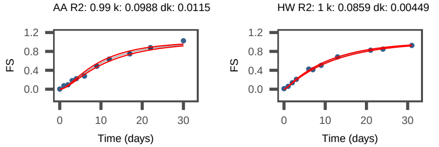

ADT1 – DFLAGGIAAAVSK_2

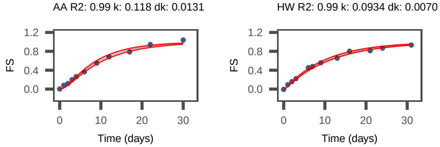

ADT1(Non–Unique) – LLLQVQHASK_2

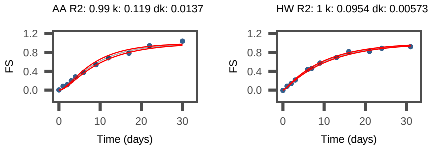

ADT1(Non–Unique) – LLLQVQHASK_3

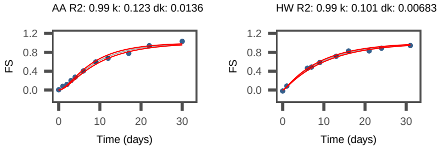

ADT2 – AAYFGIYDTAK_2

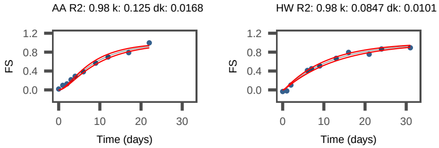

ADT2 – DFLAGGVAAAISK_3

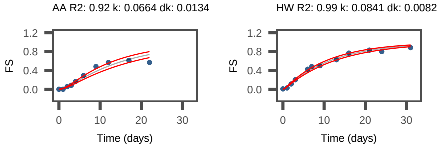

AFG1L – QLFENLFK_2

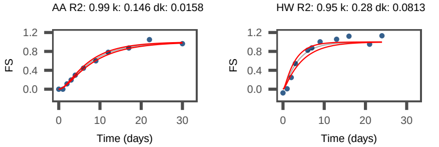

AGT2 – LGSHFWGFQTHDVLPDIVTMAK_4

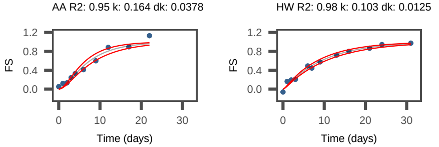

AGT2 – LSALLPEPLK_2

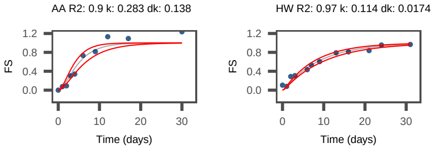

AK1A1 – ALEVLVAK_2

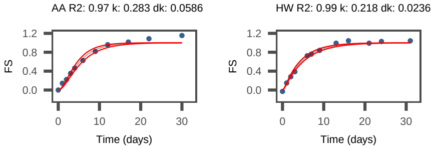

AK1A1 – EELFVTSK_2

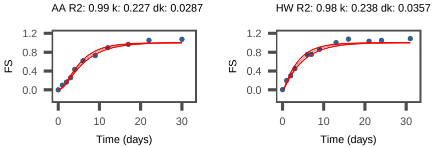

AK1A1 – GDNPFPK_2

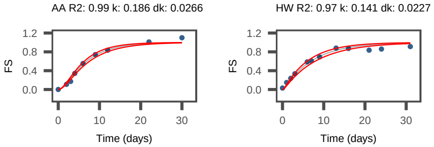

AK1A1 – HIDCASVYGNETEIGEALK_2

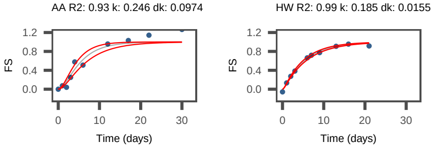

AK1A1 – HIDCASVYGNETEIGEALK_3

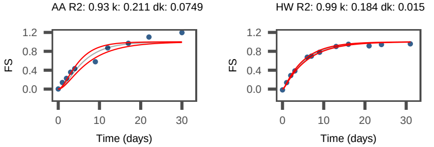

AK1A1 − ILQNIQVFDFTFSPEEMK_2

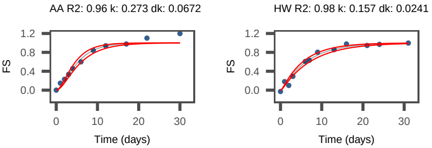

AL1L1 − GQALPEVVAK_2

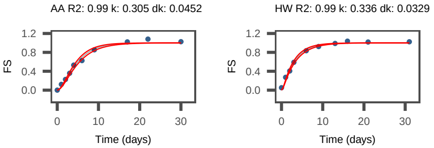

AL4A1 − SAFEYGGQK_2

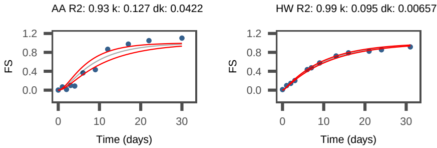

AK1A1 − MPLIGLGTWK_2

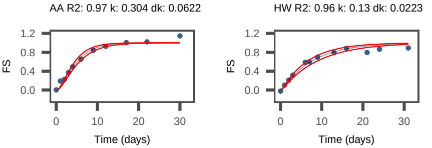

AL1L1 − LQAGTVFVNTYNK_2

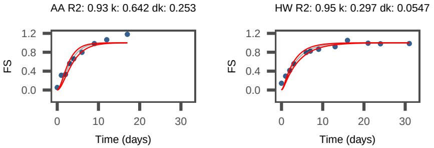

AL4A1 − VGDPAEDFGTFFSAVIDAK_2

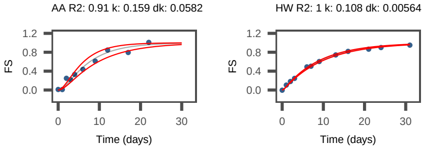

AK1CL − GIVVLNTSLK_2

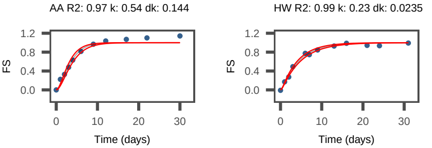

AL1L1 − MMPASQFFK_2

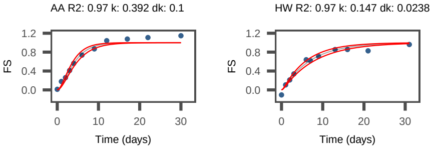

AL4A1 − VGDPAEDFGTFFSAVIDAK_3

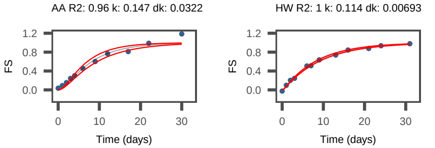

AK1CL − YGGWVDQNSPVLLDEPVLGSMAK_3

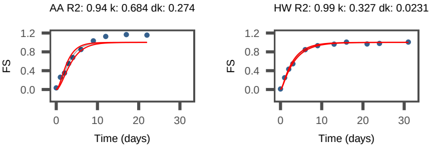

AL3A2 − VMQEEIFGPILPIVSVK_2

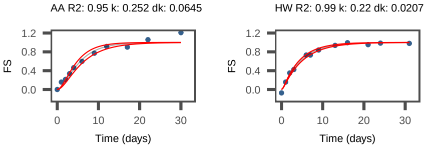

AL7A1 − AWNIWADIPAPK_2

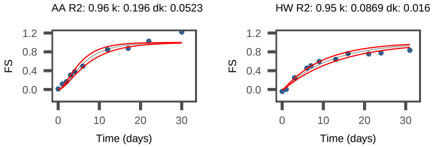

AK1CL − YIPAAIFK_2

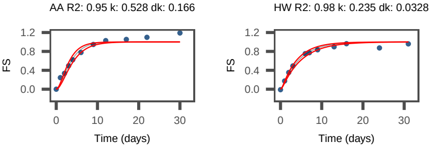

AL3A2 − VMQEEIFGPILPIVSVK_3

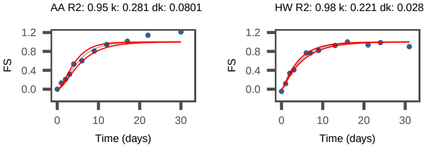

AL7A1 − VNLLSFTGSTQVGK_2

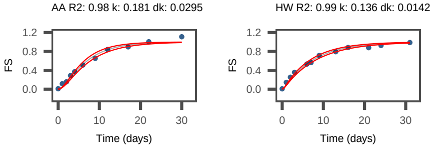

AL1A7(Non−Unique) − VTLELGGK_2

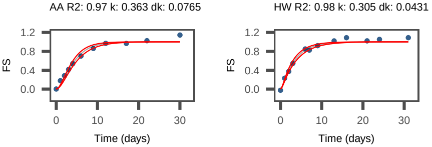

AL3A2 − YLAPTILTDVDPNSK_2

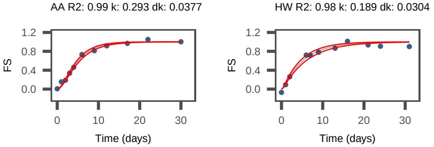

AL8A1 − DSYDFFTEIK_2

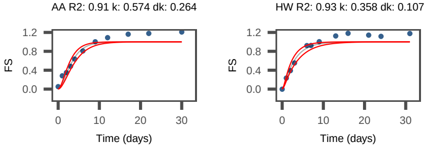

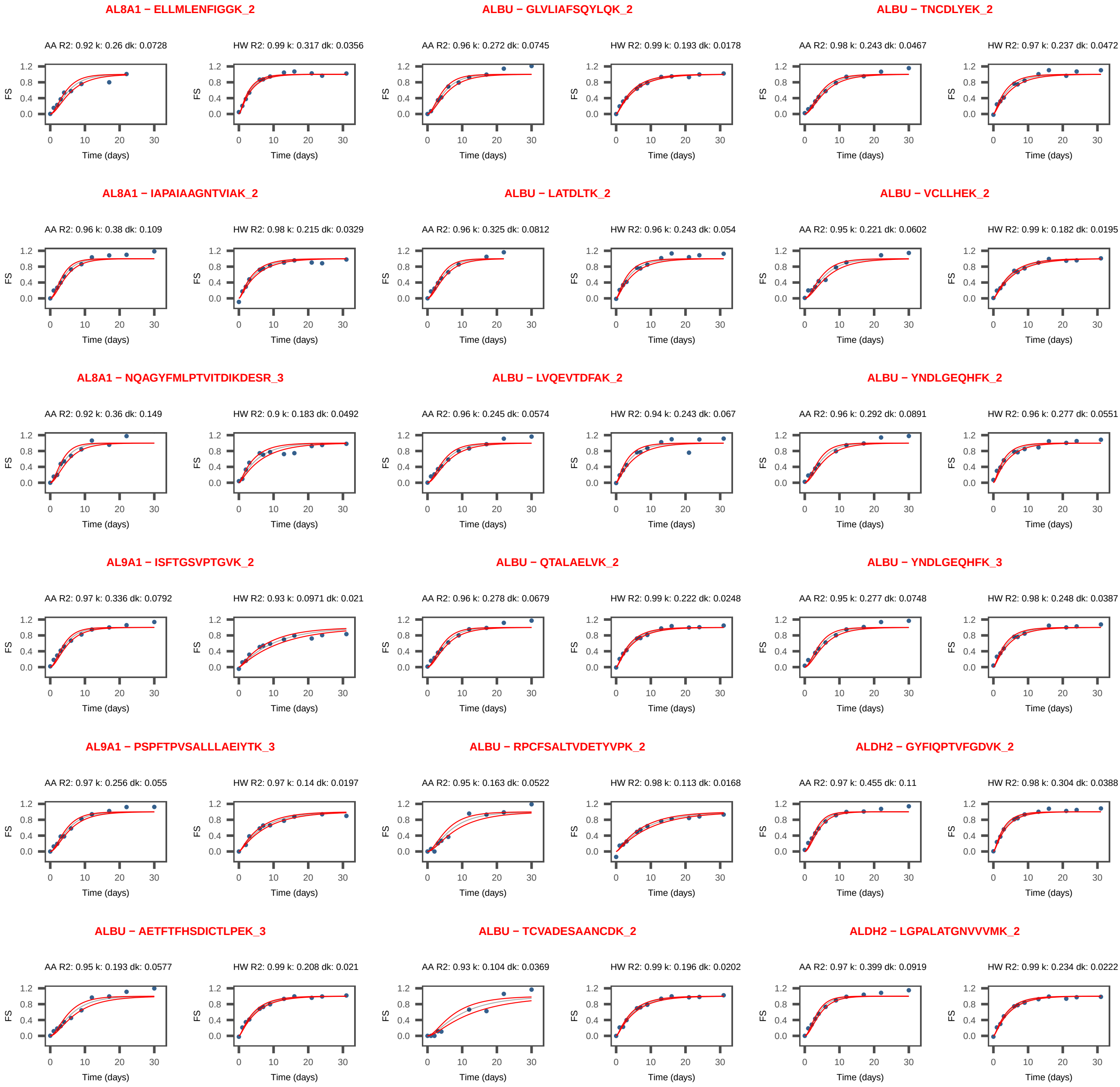
AL8A1 − ELLMLENFIGGK_2
AA R2: 0.92 k: 0.26 dk: 0.0728
HW R2: 0.99 k: 0.317 dk: 0.0356
ALBU − GLVLIAFSQYLQK_2
AA R2: 0.96 k: 0.272 dk: 0.0745
HW R2: 0.99 k: 0.193 dk: 0.0178
ALBU − TNCDLYEK_2
AA R2: 0.98 k: 0.243 dk: 0.0467
HW R2: 0.97 k: 0.237 dk: 0.0472
AL8A1 − IAPAIAAGNTVIAK_2
AA R2: 0.96 k: 0.38 dk: 0.109
HW R2: 0.98 k: 0.215 dk: 0.0329
ALBU − LATDLTK_2
AA R2: 0.96 k: 0.325 dk: 0.0812
HW R2: 0.96 k: 0.243 dk: 0.054
ALBU − VCLLHEK_2
AA R2: 0.95 k: 0.221 dk: 0.0602
HW R2: 0.99 k: 0.182 dk: 0.0195
AL8A1 − NQAGYFMLPTVITDIKDESR_3
AA R2: 0.92 k: 0.36 dk: 0.149
HW R2: 0.9 k: 0.183 dk: 0.0492
ALBU − LVQEVTDFAK_2
AA R2: 0.96 k: 0.245 dk: 0.0574
HW R2: 0.94 k: 0.243 dk: 0.067
ALBU − YNDLGEQHFK_2
AA R2: 0.96 k: 0.292 dk: 0.0891
HW R2: 0.96 k: 0.277 dk: 0.0551
AL9A1 − ISFTGSVPTGVK_2
AA R2: 0.97 k: 0.336 dk: 0.0792
HW R2: 0.93 k: 0.0971 dk: 0.021
ALBU − QTALAELVK_2
AA R2: 0.96 k: 0.278 dk: 0.0679
HW R2: 0.99 k: 0.222 dk: 0.0248
ALBU − YNDLGEQHFK_3
AA R2: 0.95 k: 0.277 dk: 0.0748
HW R2: 0.98 k: 0.248 dk: 0.0387
AL9A1 − PSPFTPVSALLLAEIYTK_3
AA R2: 0.97 k: 0.256 dk: 0.055
HW R2: 0.97 k: 0.14 dk: 0.0197
ALBU − RPCFSALTVDETYVPK_2
AA R2: 0.95 k: 0.163 dk: 0.0522
HW R2: 0.98 k: 0.113 dk: 0.0168
ALDH2 − GYFIQPTVFGDVK_2
AA R2: 0.97 k: 0.455 dk: 0.11
HW R2: 0.98 k: 0.304 dk: 0.0388
ALBU − AETFTFHSDICTLPEK_3
AA R2: 0.95 k: 0.193 dk: 0.0577
HW R2: 0.99 k: 0.208 dk: 0.021
ALBU − TCVADESAANCDK_2
AA R2: 0.93 k: 0.104 dk: 0.0369
HW R2: 0.99 k: 0.196 dk: 0.0202
ALDH2 − LGPALATGNVVVMK_2
AA R2: 0.97 k: 0.399 dk: 0.0919
HW R2: 0.99 k: 0.234 dk: 0.0222
FS
Time (days)

ALDH2 − TEQGPQVDETQFK_2

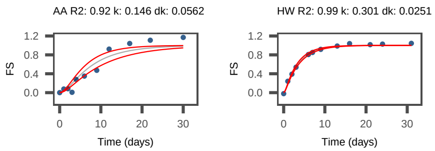

ALDH2 − VAEQTPLTALYVANLIK_2

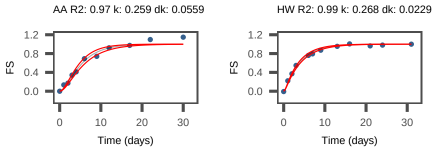

ALDH2 − VAFTGSTEVGHLIQVAAGSSNLK_2

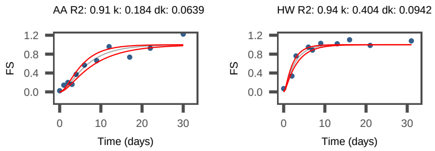

ALDH2 − YGLAAAVFTK_2

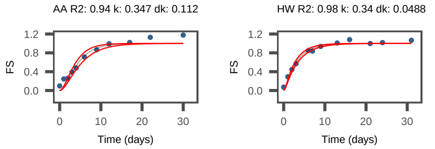

ALDOA_RABIT,sp|P05063|ALDOC(Non−Unique) − VLAAVYK_2

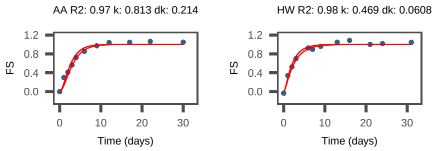

ALDOA_RABIT,sp|P05064|ALDOA(Non−Unique) − GILAADESTGSIAK_2

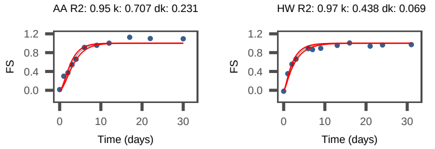

ALDOB − ALNDHHVYLEGTLLKPN_2

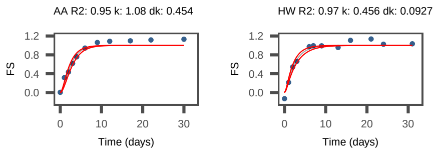

ALDOB − ALNDHHVYLEGTLLKPN_4

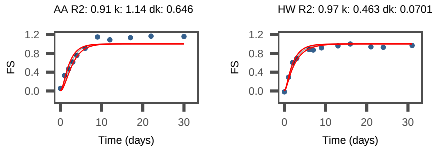

ALDOB − ATQEAFMK_2

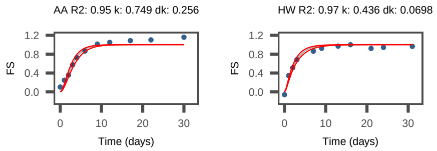

ALDOB − IKVENTEENR_2

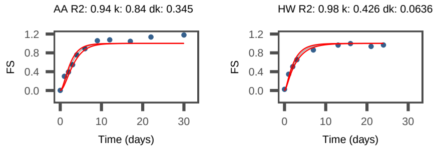

ALDOB − IKVENTEENR_3

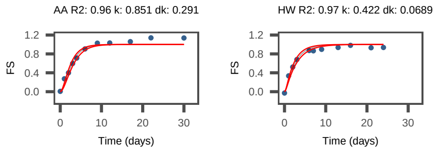

ALDOB − LDQGGAPLAGTNK_2

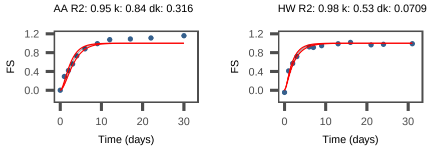

AMACR − GLGLESEELPSQMSSADWPEMK_2

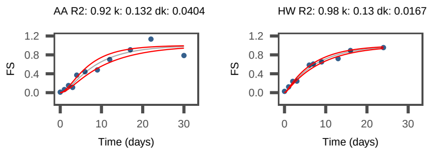

AMPE − EYSALSNMPEEK_2

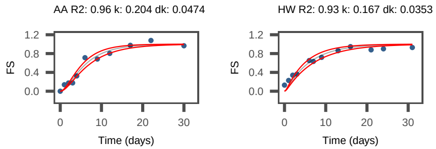

AMPL − EMLNISGPPLK_2

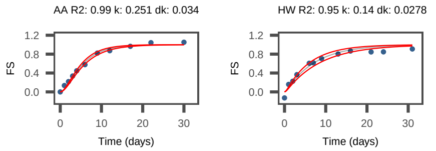

AMPL − GLVLGIYAK_2

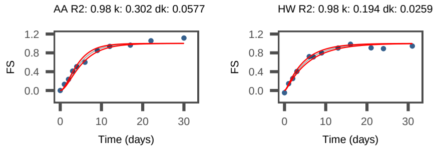

AMPL − LHGSGDLEAWEK_3

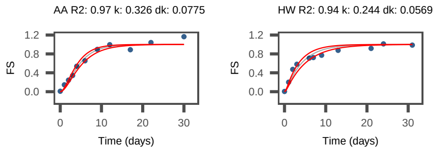

AMPL − LNLPINIIGLAPLCENMPSGK_2

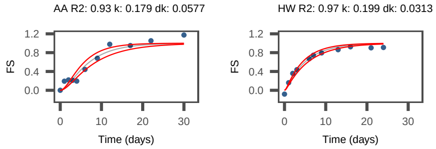

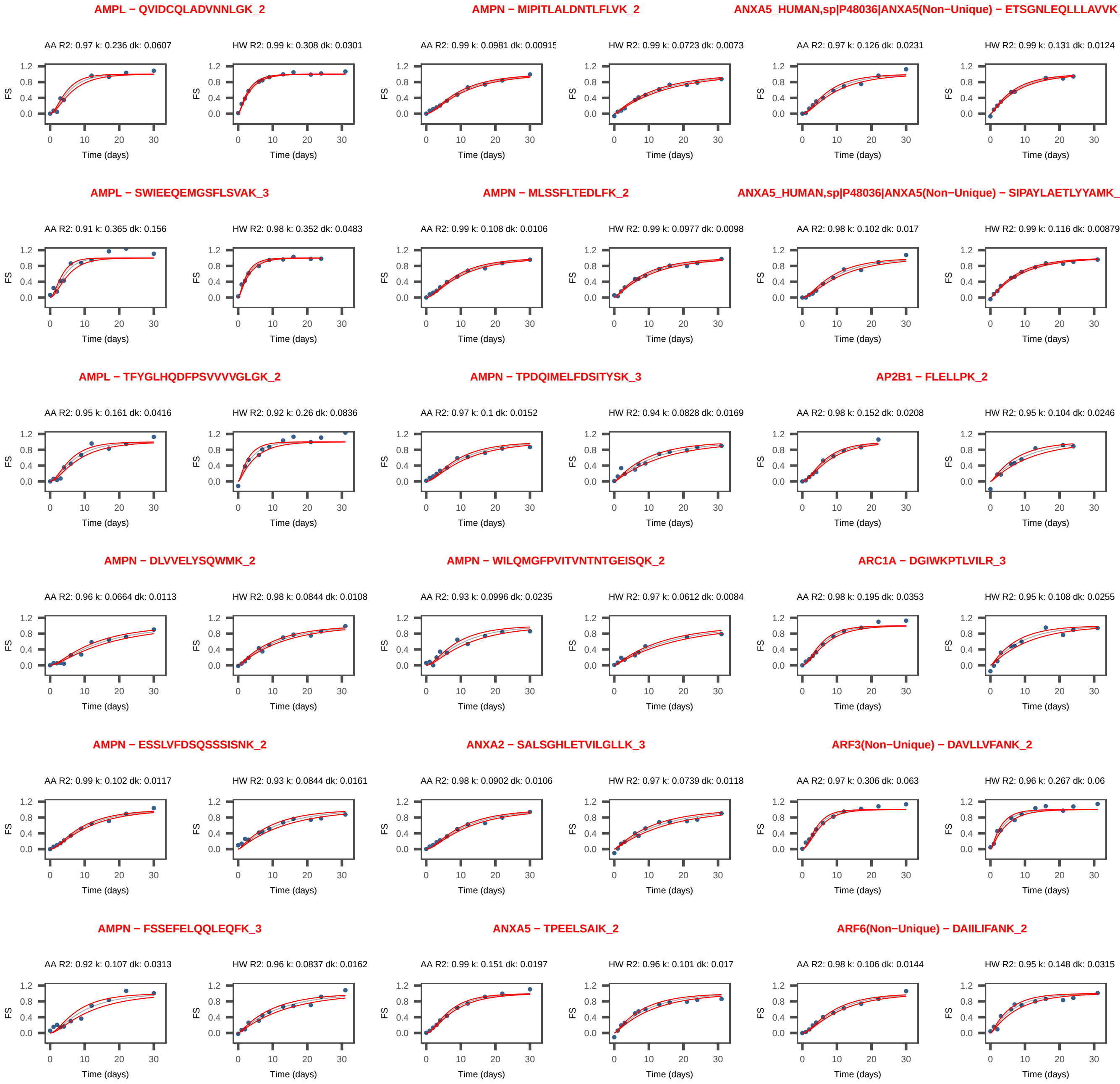
AMPL − QVIDCQLADVNNLGK_2
AA R2: 0.97 k: 0.236 dk: 0.0607
HW R2: 0.99 k: 0.308 dk: 0.0301
AMPN − MIPITLALDNTLFLVK_2
AA R2: 0.99 k: 0.0981 dk: 0.00915
HW R2: 0.99 k: 0.0723 dk: 0.0073
ANXA5_HUMAN,sp|P48036|ANXA5(Non−Unique) − ETSGNLEQLLLAVVK_
AA R2: 0.97 k: 0.126 dk: 0.0231
HW R2: 0.99 k: 0.131 dk: 0.0124
AMPL − SWIEEQEMGSFLSVAK_3
AA R2: 0.91 k: 0.365 dk: 0.156
HW R2: 0.98 k: 0.352 dk: 0.0483
AMPN − MLSSFLTEDLFK_2
AA R2: 0.99 k: 0.108 dk: 0.0106
HW R2: 0.99 k: 0.0977 dk: 0.0098
ANXA5_HUMAN,sp|P48036|ANXA5(Non−Unique) − SIPAYLAETLYYAMK_
AA R2: 0.98 k: 0.102 dk: 0.017
HW R2: 0.99 k: 0.116 dk: 0.00879
AMPL − TFYGLHQDFPSVVVVGLGK_2
AA R2: 0.95 k: 0.161 dk: 0.0416
HW R2: 0.92 k: 0.26 dk: 0.0836
AMPN − TPDQIMELFDSITYSK_3
AA R2: 0.97 k: 0.1 dk: 0.0152
HW R2: 0.94 k: 0.0828 dk: 0.0169
AP2B1 − FLELLPK_2
AA R2: 0.98 k: 0.152 dk: 0.0208
HW R2: 0.95 k: 0.104 dk: 0.0246
AMPN − DLVVELYSQWMK_2
AA R2: 0.96 k: 0.0664 dk: 0.0113
HW R2: 0.98 k: 0.0844 dk: 0.0108
AMPN − WILQMGFPVITVNTNTGEISQK_2
AA R2: 0.93 k: 0.0996 dk: 0.0235
HW R2: 0.97 k: 0.0612 dk: 0.0084
ARC1A − DGIWKPTLVILR_3
AA R2: 0.98 k: 0.195 dk: 0.0353
HW R2: 0.95 k: 0.108 dk: 0.0255
AMPN − ESSLVFDSQSSSISNK_2
AA R2: 0.99 k: 0.102 dk: 0.0117
HW R2: 0.93 k: 0.0844 dk: 0.0161
ANXA2 − SALSGHLETVILGLLK_3
AA R2: 0.98 k: 0.0902 dk: 0.0106
HW R2: 0.97 k: 0.0739 dk: 0.0118
ARF3(Non−Unique) − DAVLLVFANK_2
AA R2: 0.97 k: 0.306 dk: 0.063
HW R2: 0.96 k: 0.267 dk: 0.06
AMPN − FSSEFELQQLEQFK_3
AA R2: 0.92 k: 0.107 dk: 0.0313
HW R2: 0.96 k: 0.0837 dk: 0.0162
ANXA5 − TPEELSAIK_2
AA R2: 0.99 k: 0.151 dk: 0.0197
HW R2: 0.96 k: 0.101 dk: 0.017
ARF6(Non−Unique) − DAIILIFANK_2
AA R2: 0.98 k: 0.106 dk: 0.0144
HW R2: 0.95 k: 0.148 dk: 0.0315
FS
Time (days)

ARLY − EFSFVQLSDAYSTGSSLMPQK_2

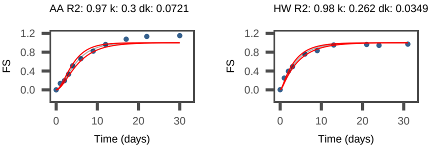

ASSY − VFIEDVSK_2

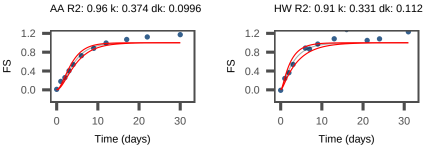

AT1A1 − YQLSIHK_2

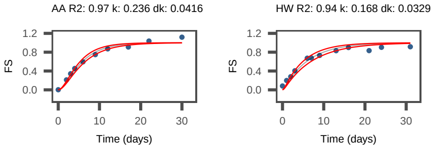

ARPC4 − HNKPEVEVR_2

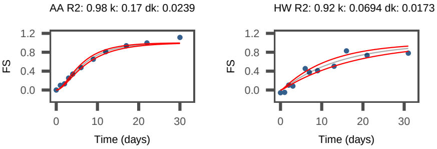

AT1A1 − ADIGVAMGIVGSDVSK_2

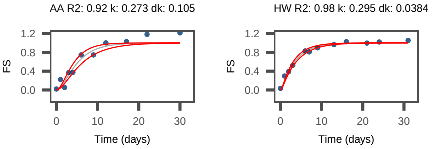

AT1A3(Non−Unique) − AAVPDAVGK_2

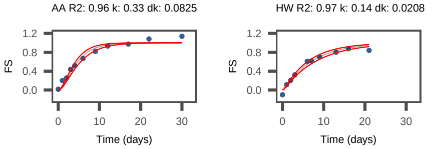

ASSY − NQAPPGLYTK_2

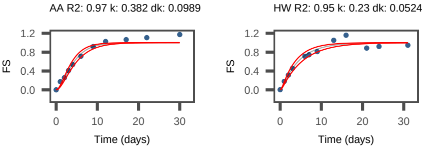

AT1A1 − ADIGVAMGIVGSDVSK_3

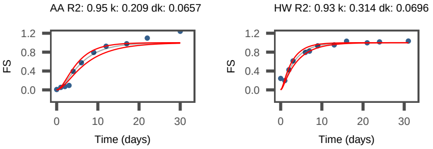

AT1B1 − AYGENIGYSEK_2

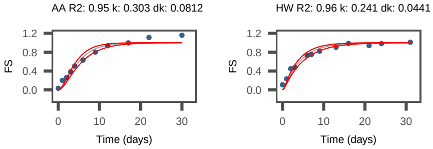

ASSY − QHGIPIPVTPK_2

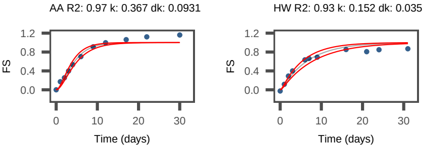

AT1A1 − AVAGDASESALLK_2

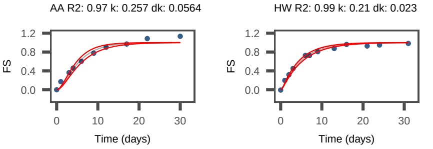

AT1B1 − FIWNSEK_2

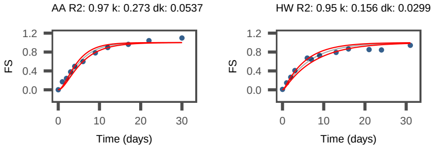

ASSY − QHGIPIPVTPK_3

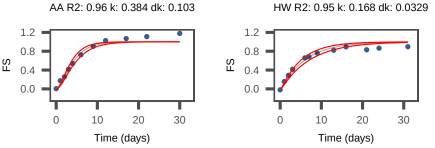

AT1A1 − RPGGWVEK_2

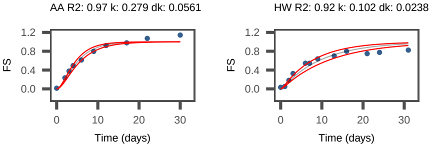

AT1B1 − TEISFRPNDPK_2

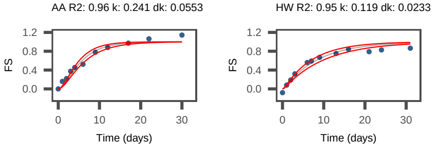

ASSY − TTSLELFMYLNEVAGK_3

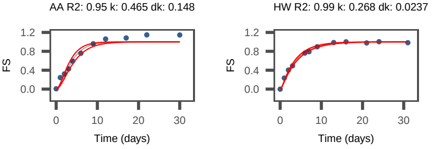

AT1A1 − YEPAAVSEHGDK_3

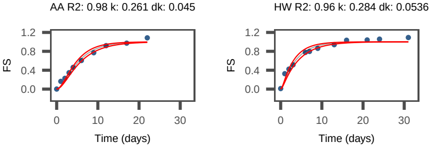

AT1B1 − VAPPGLTQIPQIQK_2

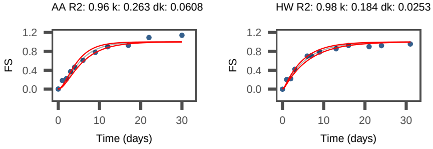

AT1B1 − VAPPGLTQIPQIQK_3

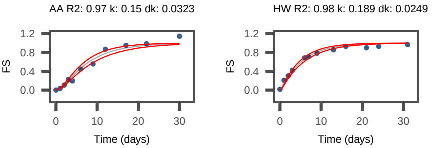

ATNG − AGEISDLSANSGGSAK_2

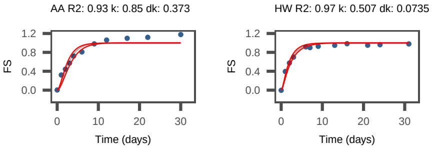

ATP5L − LATFWHYAK_3

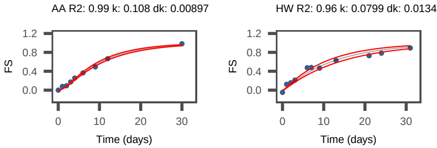

AT2A2(Non−Unique) − VDQSILTGESVSVIK_2

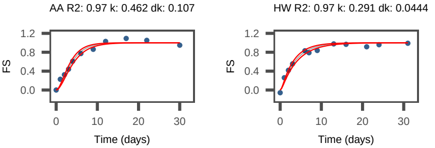

ATP4A(Non−Unique) − VIMVTGDHPITAK_3

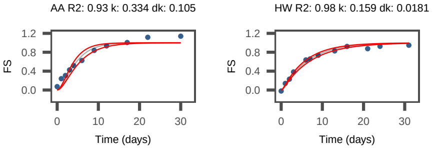

ATP5L − VELVPPTPAEIPTAIQSVK_2

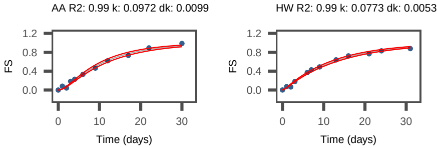

AT5F1 − IAQLEEVK_2

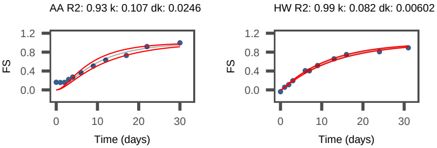

ATP5H − NIIPFDQMTIDDLNEIFPETK_2

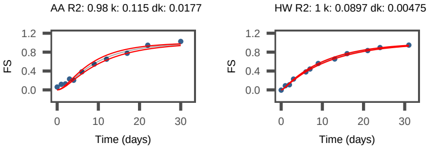

ATPA − GMSLNLEPDNVGVVVFGNDK_3

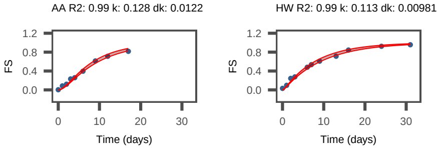

AT5F1 − LGLIPEEFFQFLYPK_2

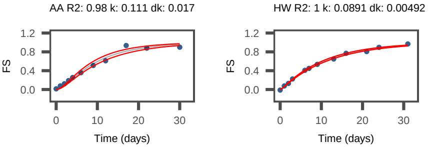

ATP5H − TIDWVSFVEVMPQNQK_3

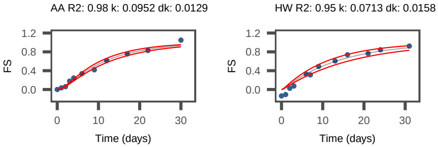

ATPA − NVQAEEMVEFSSGLK_3

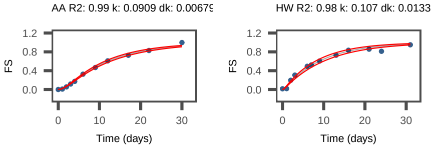

AT5F1 − LGLIPEEFFQFLYPK_3

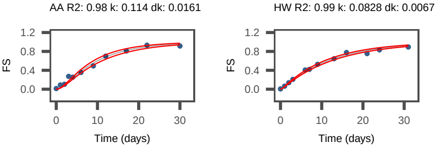

ATP5I − ELAEAQDDSILK_2

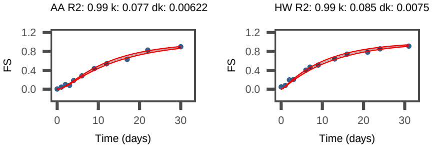

ATPA − TSIAIDTIINQK_2

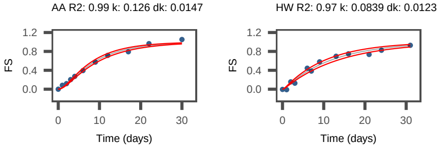

AT5F1 − PLPPLPEYGGK_2

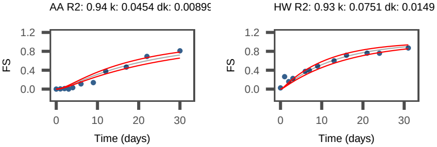

ATP5I − VPPVQVSPLIK_2

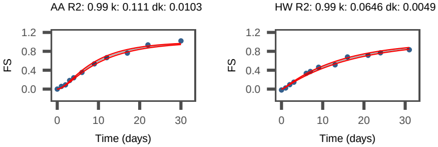

ATPA − TSIAIDTIINQK_3

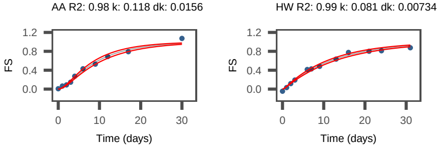

ATPB – SLQDIIAILGMDELSEEDKLTVSR_2

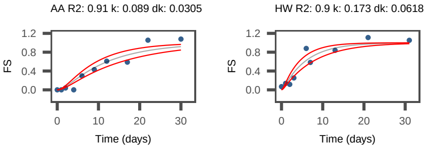

ATPG – VYGTGSLALYEK_2

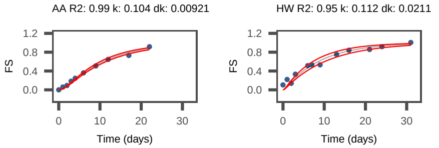

BIP – IEWLESHQDADIEDFK_3

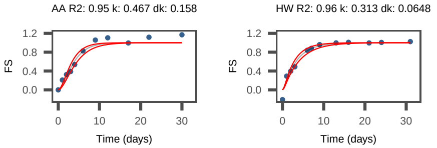

ATPB – TVLIMELINNVAK_2

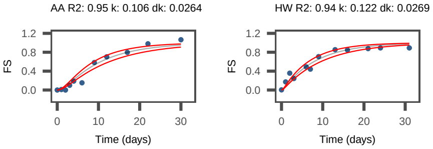

ATPO – GEVPCTVTTASPLDDAVLSELK_2

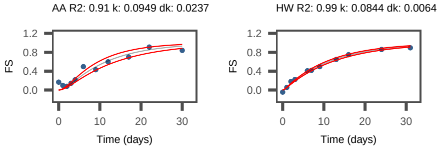

BIP – SQIFSTASDNQPTVTIK_2

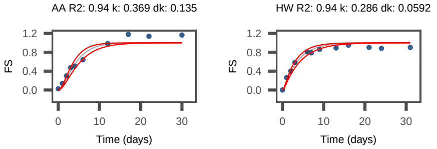

ATPD – IEANEALVK_2

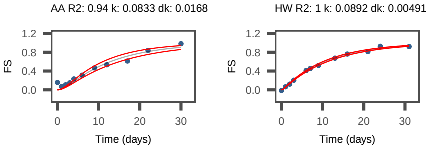

ATPO – SFLSPNQILK_2

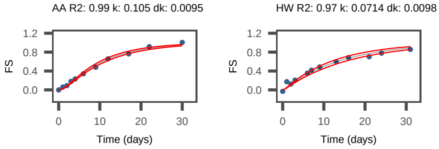

BPHL – QVSLLGWSDGGITALIAAAK_2

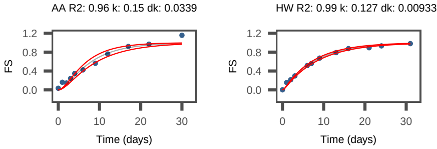

ATPD – PGLVVVHTEDGTTTK_3

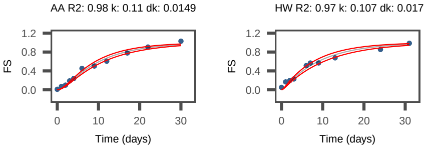

AUHM – DRLEGLLAFK_3

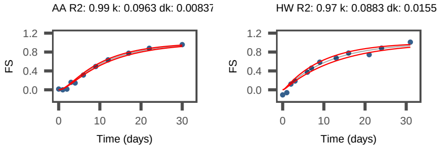

BPHL – TDFAPQLQSLNK_2

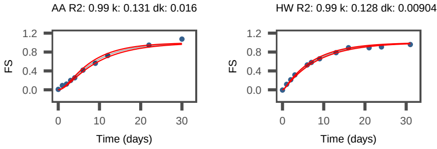

ATPG – THSDQFLVSFK_2

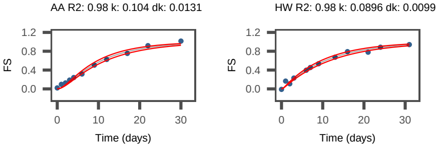

BDH – MQIMTHFPGAISDK_3

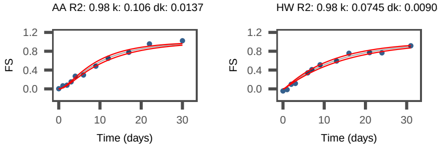

BPHL – VGEGEHAILLLPGMLGSGK_2

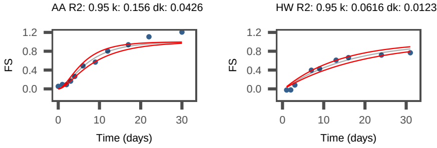

ATPG – THSDQFLVSFK_3

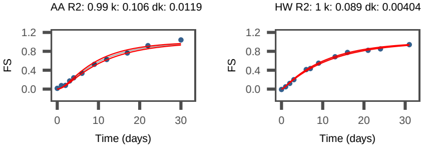

BDH2 – AAVIGLTK_2

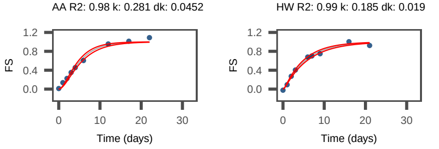

BPHL – VGEGEHAILLLPGMLGSGK_3

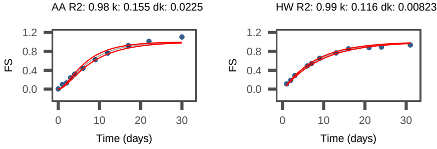

BPNT1 − EYTEGLLDNVTVLIGIAYEGK_2

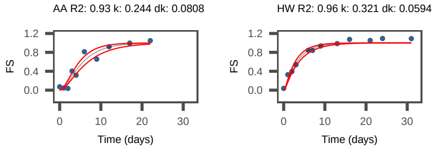

C1TC − THLSLSHNPEQK_2

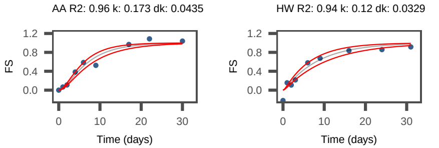

CAH2 − IGPASQGLQK_2

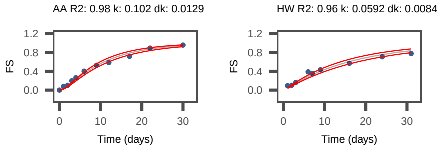

BPNT1 − IIQLIEGK_2

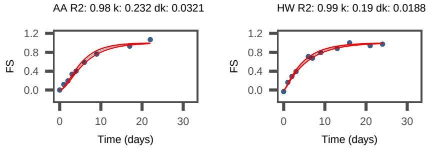

C560 − HLLWDLGK_2

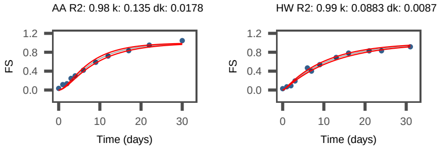

CAH2_HUMAN,sp|P00920|CAH2(Non−Unique) − AVQQPDGLAVLGIFLK_[illegible]

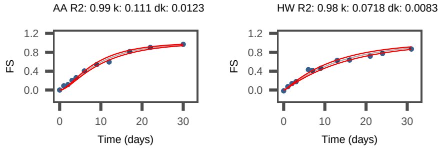

BPNT1 − LTDIHGNALQYNK_2

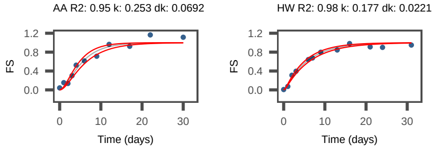

C560 − NTSSNRPLSPHLTIYK_3

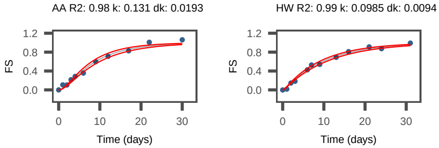

CALB1(Non−Unique) − LLPVQENFLLK_2

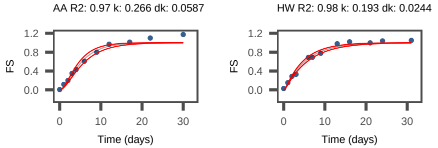

BPNT1 − LVASAYSIAQK_2

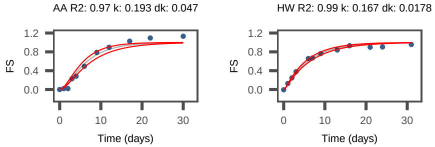

CACP − MENWLSEWWLK_2

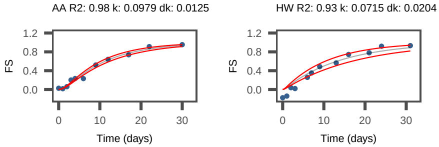

CAND1 − ADVFHAYLSLLK_3

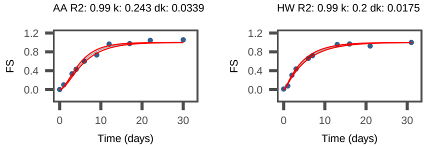

BPNT1 − NYEYYASHVPESVK_3

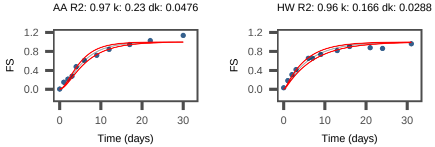

CAH2 − HNGPENWHK_2

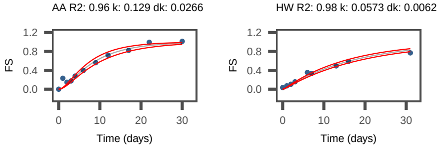

CAND1 − LGTLSALDILIK_2

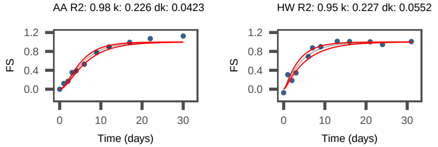

C1TC − MFGVPVVVAVNVFK_2

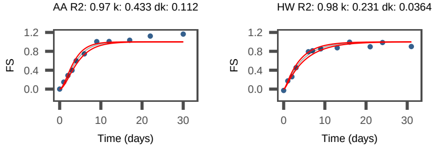

CAH2 − HNGPENWHK_3

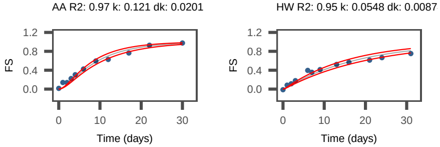

CAP1 − VENQENVSNLVIDDTELK_2

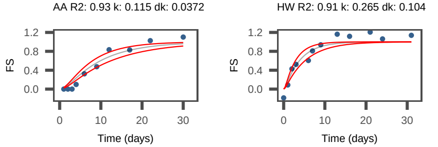

CATA − ASQRPDVLTTGGGNPIGDK_2

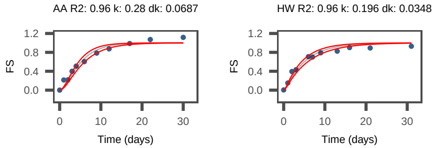

CATA − DAILFPSFIHSQK_2

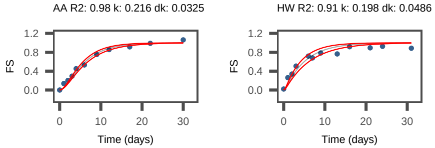

CATA − DAILFPSFIHSQK_3

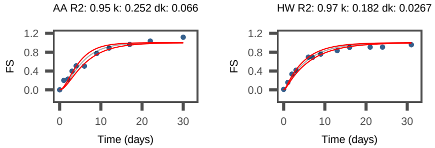

CATA − EAETFPFNPFDLTK_2

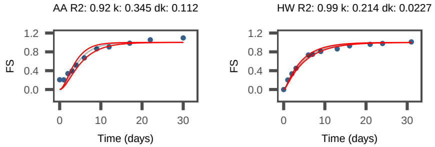

CATA − LVNADGEAVYCK_2

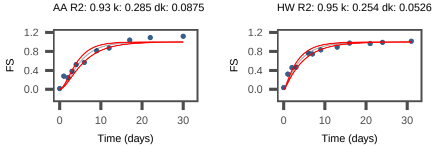

CATA_HUMAN,sp|P24270|CATA(Non−Unique) − DYPLIPVGK_2

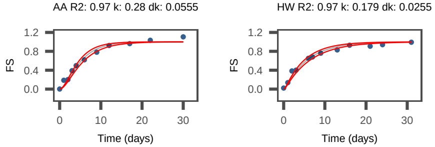

CBR1 − VNDDTPFHIQAEVTMK_3

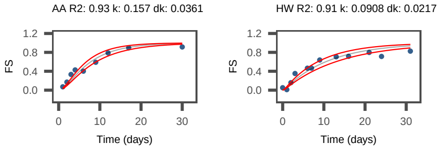

CBR4 − TMIQQGGSIVNVGSIIGLK_2

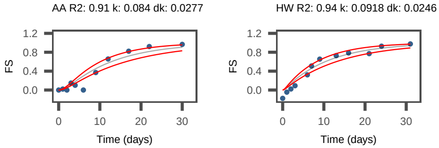

CDC42 − NVFDEAILAALEPPEPK_2

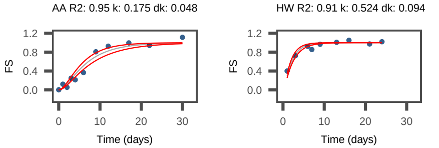

CDC42 − NVFDEAILAALEPPEPK_3

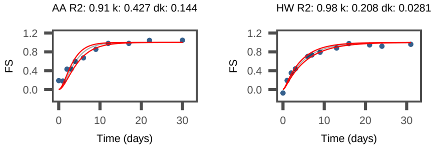

CENPV − AGAGGGGGAVGPQPSAK_2

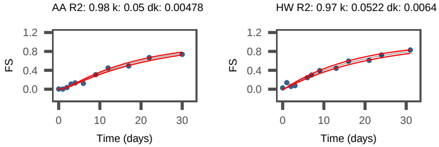

CGL − AVVLPISLATTFK_2

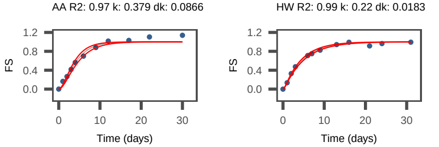

CH10 − VLQATVVAVGSGGK_2

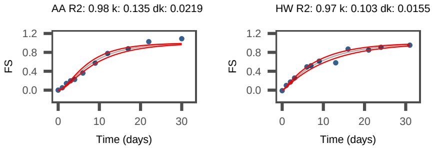

CH10 − VVLDDKDYFLFR_3

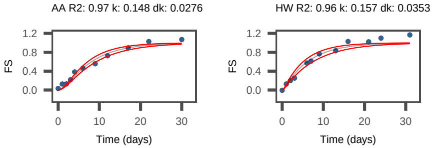

CH60(Non−Unique) − GIIDPTK_2

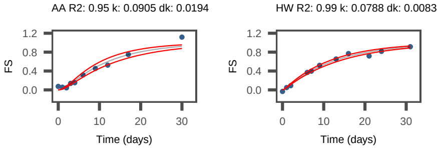

CH60 − GVMLAVDAVIAELK_2

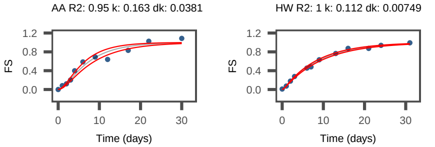

CH60 − GVMLAVDAVIAELK_3

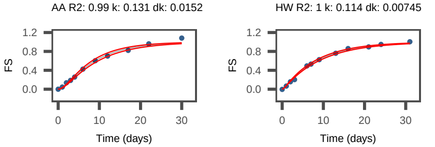

CH60 − IGIEIIK_2

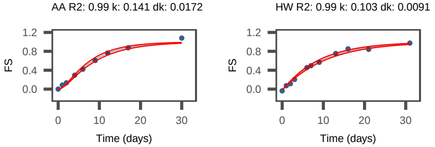

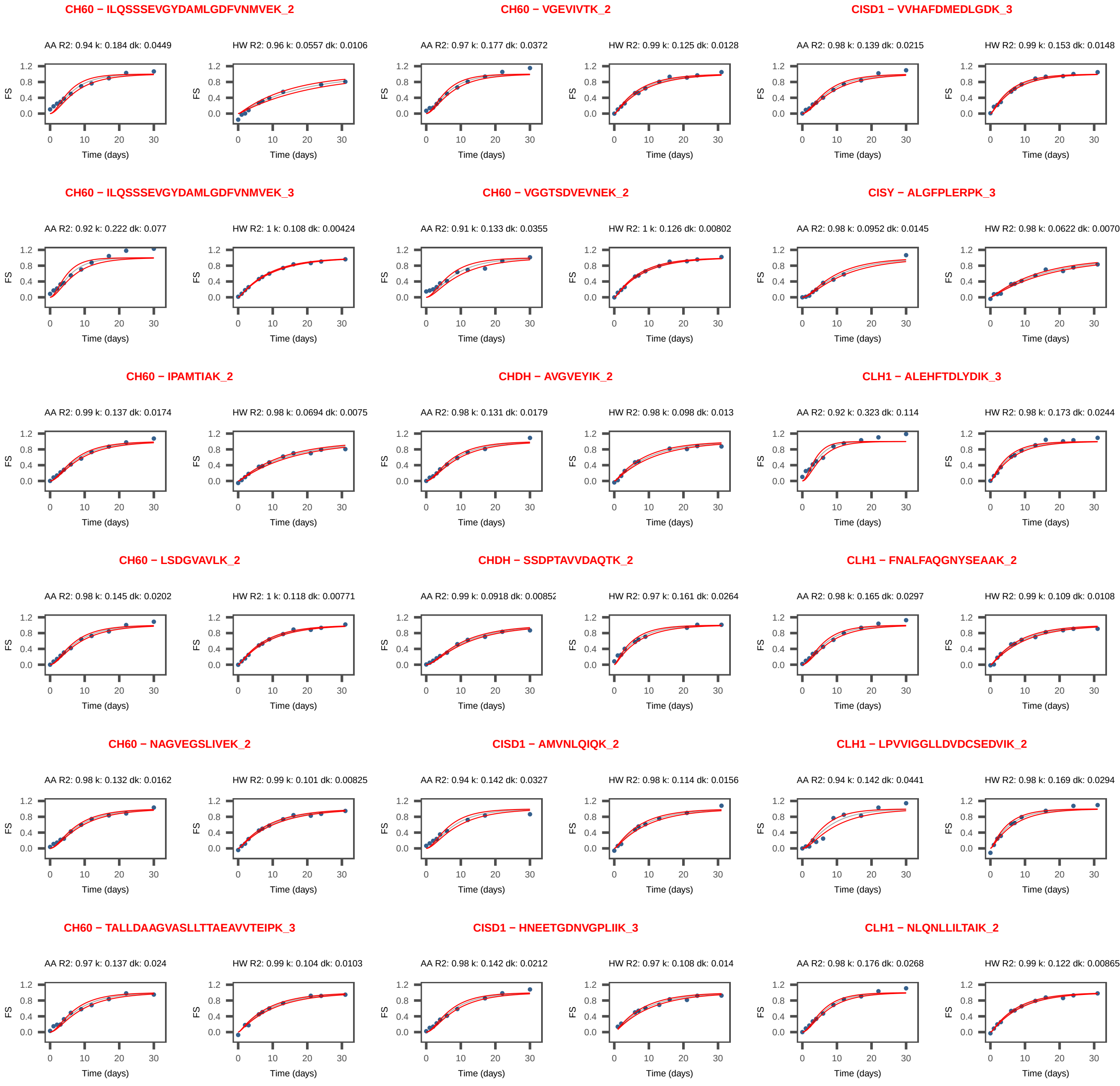
CH60 − ILQSSSEVGYDAMLGDFVNMVEK_2
AA R2: 0.94 k: 0.184 dk: 0.0449
HW R2: 0.96 k: 0.0557 dk: 0.0106
CH60 − VGEVIVTK_2
AA R2: 0.97 k: 0.177 dk: 0.0372
HW R2: 0.99 k: 0.125 dk: 0.0128
CISD1 − VVHAFDMEDLGDK_3
AA R2: 0.98 k: 0.139 dk: 0.0215
HW R2: 0.99 k: 0.153 dk: 0.0148
CH60 − ILQSSSEVGYDAMLGDFVNMVEK_3
AA R2: 0.92 k: 0.222 dk: 0.077
HW R2: 1 k: 0.108 dk: 0.00424
CH60 − VGGTSDVEVNEK_2
AA R2: 0.91 k: 0.133 dk: 0.0355
HW R2: 1 k: 0.126 dk: 0.00802
CISY − ALGFPLERPK_3
AA R2: 0.98 k: 0.0952 dk: 0.0145
HW R2: 0.98 k: 0.0622 dk: 0.0070
CH60 − IPAMTIAK_2
AA R2: 0.99 k: 0.137 dk: 0.0174
HW R2: 0.98 k: 0.0694 dk: 0.0075
CHDH − AVGVEYIK_2
AA R2: 0.98 k: 0.131 dk: 0.0179
HW R2: 0.98 k: 0.098 dk: 0.013
CLH1 − ALEHFTDLYDIK_3
AA R2: 0.92 k: 0.323 dk: 0.114
HW R2: 0.98 k: 0.173 dk: 0.0244
CH60 − LSDGVAVLK_2
AA R2: 0.98 k: 0.145 dk: 0.0202
HW R2: 1 k: 0.118 dk: 0.00771
CHDH − SSDPTAVVDAQTK_2
AA R2: 0.99 k: 0.0918 dk: 0.00852
HW R2: 0.97 k: 0.161 dk: 0.0264
CLH1 − FNALFAQGNYSEAAK_2
AA R2: 0.98 k: 0.165 dk: 0.0297
HW R2: 0.99 k: 0.109 dk: 0.0108
CH60 − NAGVEGSLIVEK_2
AA R2: 0.98 k: 0.132 dk: 0.0162
HW R2: 0.99 k: 0.101 dk: 0.00825
CISD1 − AMVNLQIQK_2
AA R2: 0.94 k: 0.142 dk: 0.0327
HW R2: 0.98 k: 0.114 dk: 0.0156
CLH1 − LPVVIGGLLDVDCSEDVIK_2
AA R2: 0.94 k: 0.142 dk: 0.0441
HW R2: 0.98 k: 0.169 dk: 0.0294
CH60 − TALLDAAGVASLLTTAEAVVTEIPK_3
AA R2: 0.97 k: 0.137 dk: 0.024
HW R2: 0.99 k: 0.104 dk: 0.0103
CISD1 − HNEETGDNVGPLIIK_3
AA R2: 0.98 k: 0.142 dk: 0.0212
HW R2: 0.97 k: 0.108 dk: 0.014
CLH1 − NLQNLLILTAIK_2
AA R2: 0.98 k: 0.176 dk: 0.0268
HW R2: 0.99 k: 0.122 dk: 0.00865
FS
Time (days)

CLH1 − NLQNLLILTAIK_3

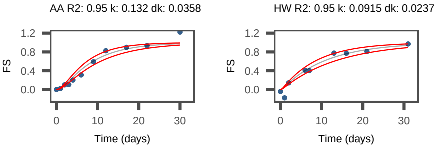

CLIC1 − LAALNPESNTSGLDIFAK_2

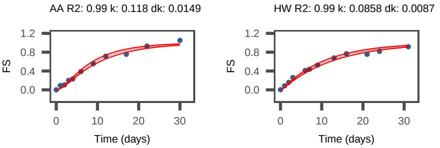

CMBL − NLIEWLNK_2

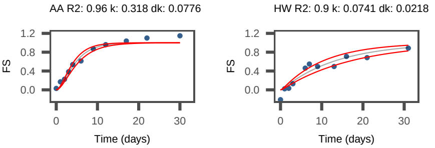

CLH1 − RPISADSAIMNPASK_2

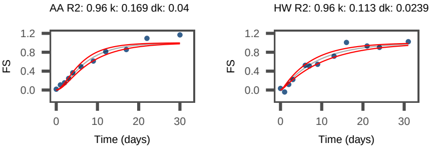

CLTRN − INSAFFLDDHTLEFLK_3

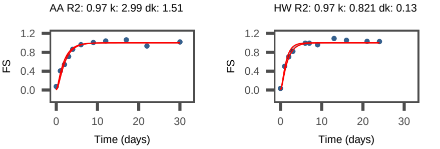

CMC1 − FTLGSVAGAVGATAVYPIDLVK_3

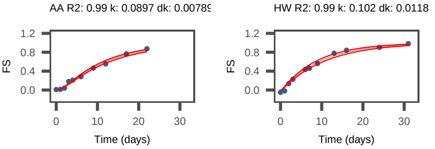

CLH1 − RPISADSAIMNPASK_3

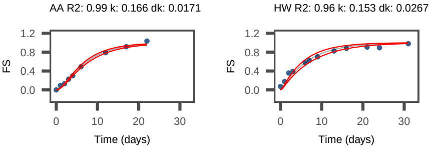

CLYBL − GSMIDMPLLK_2

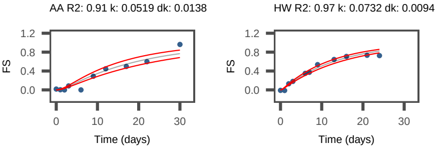

CMC1(Non−Unique) − GLIPQLIGVAPEK_2

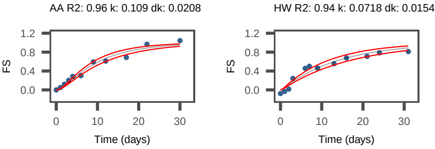

CLH1 − RPLIDQVVQTALSETQDPEEVSVTVK_3

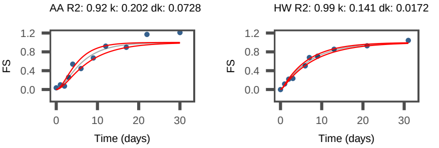

CLYBL − IQWAEELIAAFK_2

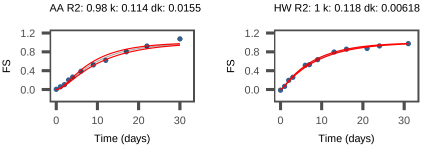

CMC1 − IVQLLAGVADQTK_2

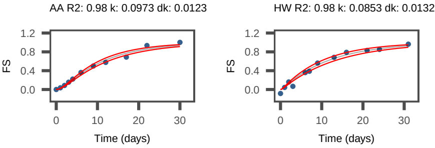

CLH1 − TLQIFNIEMK_2

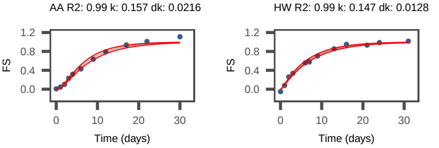

CLYBL − QAQNIVTLATSIK_2

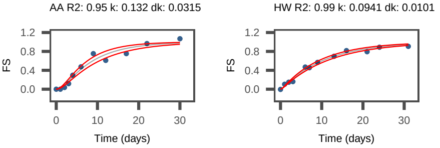

CMC1 − VGGINLLTAGALAGVPAASLVTPADVIK_3

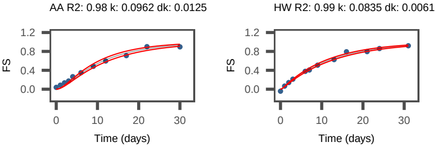

CLH1 − YHEQLSTQSLIELFESFK_3

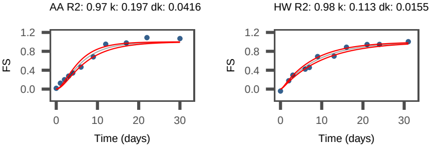

CLYBL − QIAVVQEQFTPTPEK_2

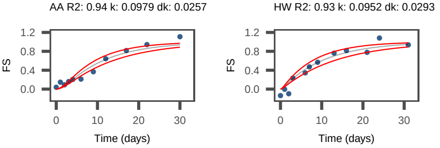

CMC2 − DLGFFGIYK_2

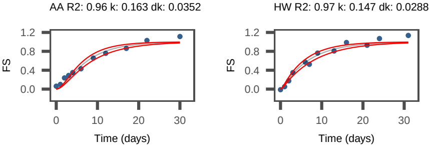

CMC2 − FGLGSIAGAVGATAVYPIDLVK_2
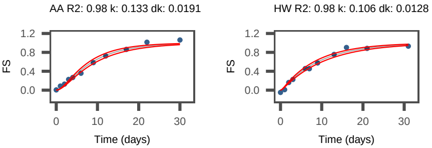

CNDP2 − LVPDMIPEVVSEQVSSYLSK_3
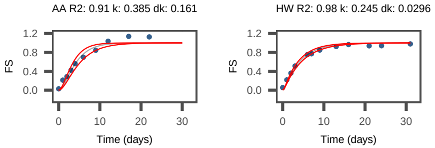

COMT − VSILIGASQDLIPQLK_2
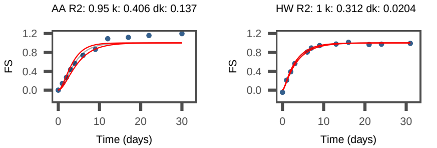

CMC2 − FGLGSIAGAVGATAVYPIDLVK_3
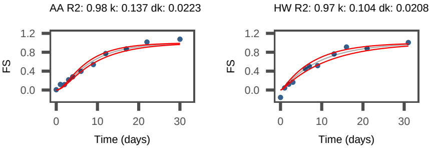

CNDP2 − MLAAYLYEVSQLK_2
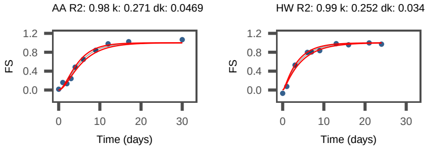

COPB2 − HSEVQQANLK_2
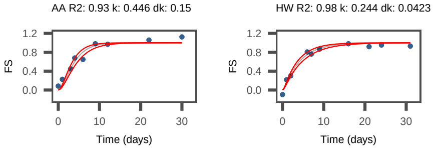

CMC2 − ITLPAPNPDHVGGYK_2
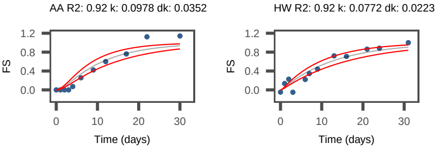

CNDP2 − MLAAYLYEVSQLK_3
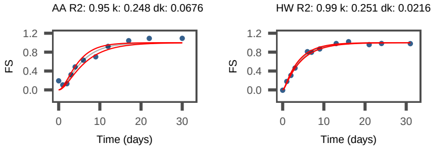

COPB2 − VWQLGSSSPNFTLEGHEK_3
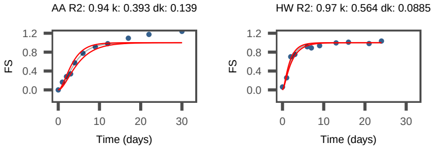

CMC2 − ITLPAPNPDHVGGYK_3
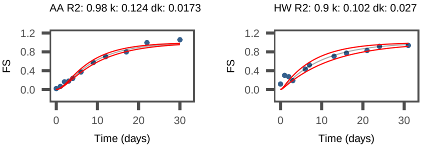

CNDP2 − NVMLLPVGSADDGAHSQNEK_3
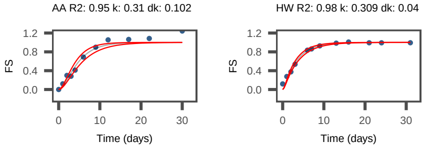

COPG2(Non−Unique) − SIATLAITTLLK_2
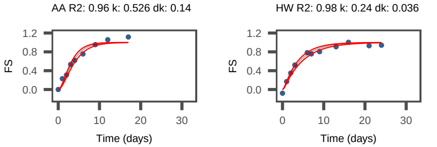

CNDP2 − LGGSVELVDIGK_2
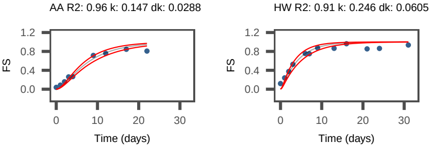

COASY − AYAPGGPAYQPVVEAFGTDILHK_3
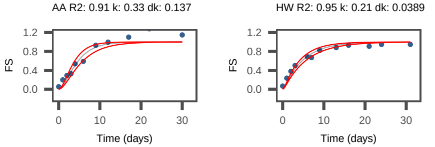

COQ9 − LVQLGQAEK_2
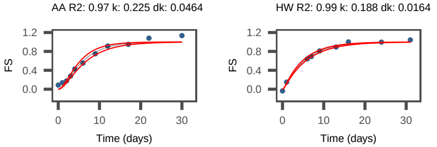

CNDP2 − LVPDMIPEVVSEQVSSYLSK_2
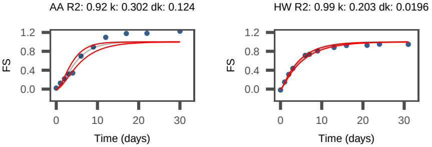

COASY − ILTDIVWPVIAK_2
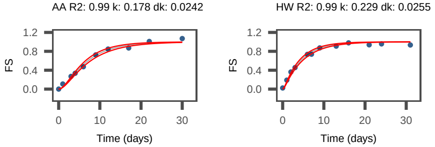

COX2 − MLISSEDVLHSWAVPSLGLK_2
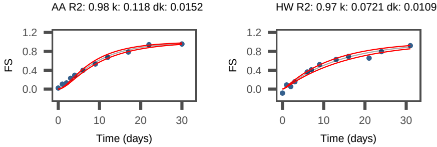

COX41 − DYPLPDVAHVTMLSASQK_2

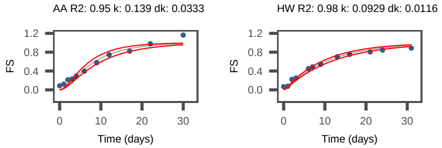

COX5B − EIMIAAQK_2

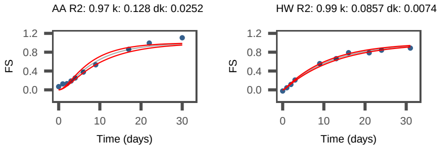

CP4B1 − NCIGQQFAMNEMK_2

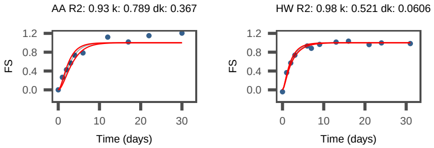

COX41 − DYPLPDVAHVTMLSASQK_3

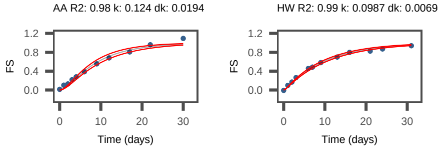

COX6C − AGIFQSAK_2

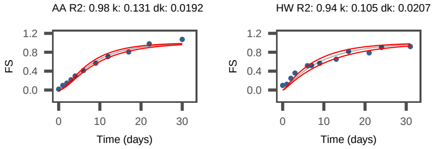

CP4B1 − QLSKPVTFVDGR_3

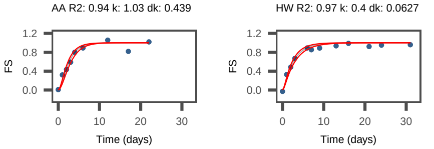

COX5A − GMNTLVGYDLVPEPK_2

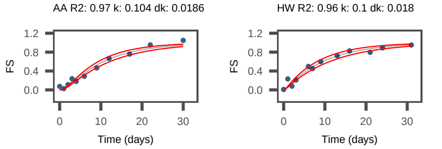

CP2A4(Non−Unique) − GEQATFDWLFK_2

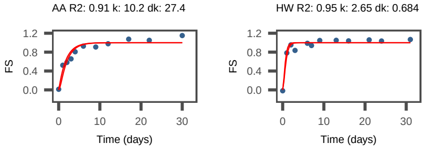

CP51A − EPAEDILQTLLDSTYK_3

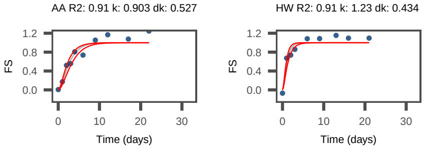

COX5A − NKPDIDAWELR_3

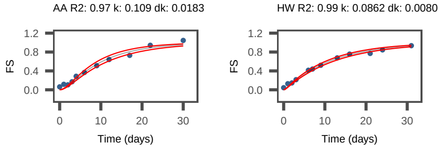

CP4B1 − FEFSPDPSK_2

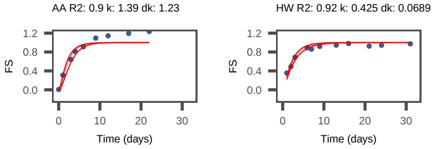

CPT1A − IPGEETDTIQHVK_3

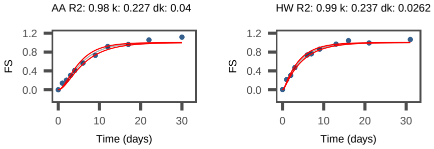

COX5A − VIQELRPTLNELGISTPEELGLDKV_3

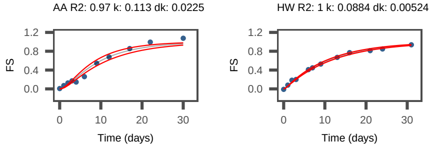

CP4B1 − GLLVLEGPK_2

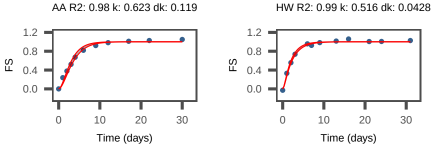

CPT1A − LSTSQTPQQQVELFDFEK_2

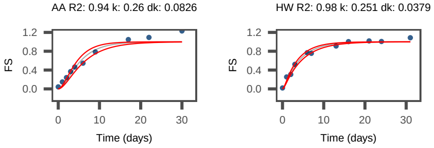

COX5B − EDPNLVPSISNK_2

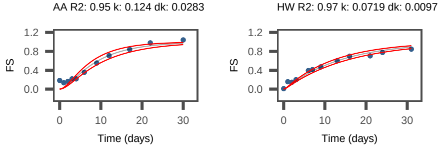

CP4B1 − HWLFGHALEIQK_3

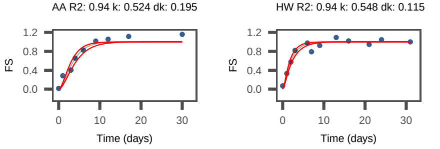

CPT1A − SITFVVFK_2

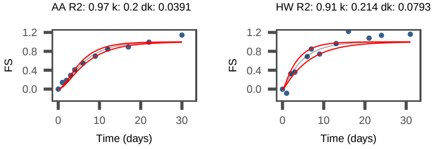

CPT2 − SFNLIVAK_2

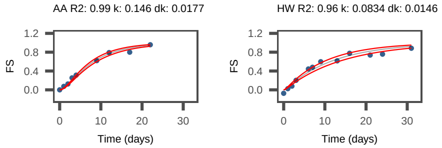

CY1 − HLVGVCYTEEEAK_2

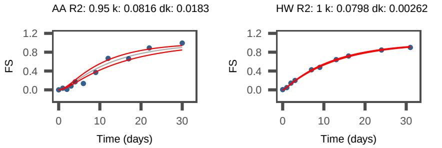

DCXR − AVIQVSQIVAK_2

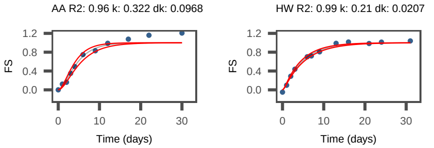

CRYL1 − SLEQSGSLK_2

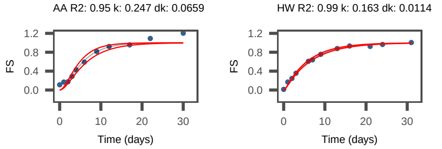

CYC(Non−Unique) − ADLIAYLK_2

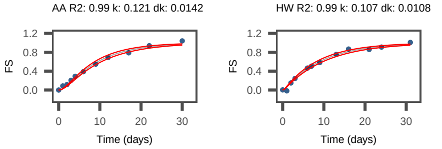

DDX1 − DLGLAFEIPAHIK_3

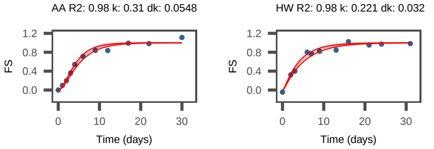

CSRP1 − HEEAPGHRPTTNPNASK_4

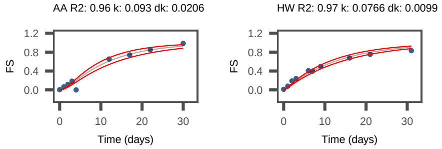

CYC − GITWGEDTLMEYLENPK_3

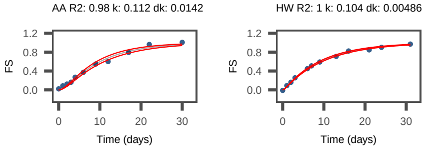

DECR − DPDMVHNTVLELIK_3

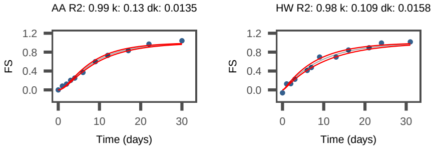

CTND1 − GIPILVGLLDHPK_3

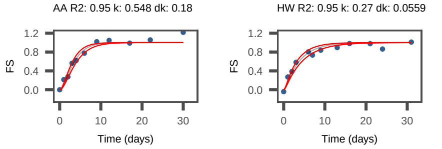

CYC − TGQAAGFSYTDANK_2

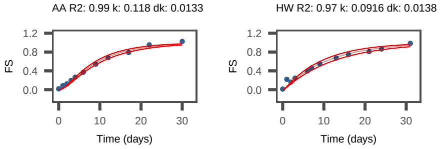

DECR − FDGGEEVFLSGEFNSLK_2

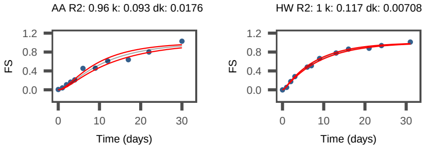

CX6B1 − IAEGTFPGK_2

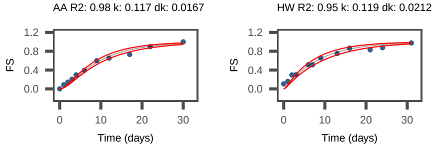

CYTB − QIVAGTNLFIK_2

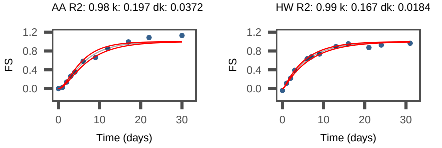

DECR − VAFITGGGTGLGK_2

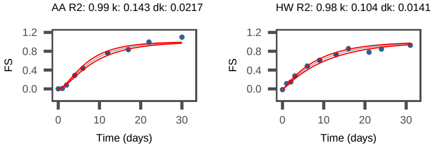

CY1 − ALAEEVEVQDGPNDDGEMFMRPGK_3

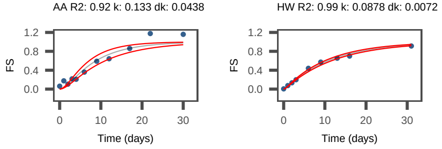

D39U1 − EYDEDSPGGNFDFFSNLVTK_2

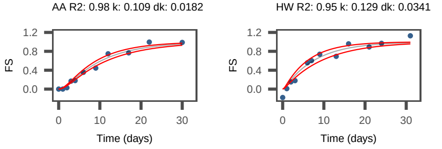

DHB4 − LPSFSSSYTELQSIMYALGVGASVK_3

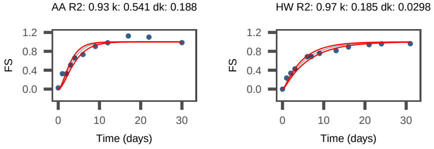

DHB5(Non−Unique) − REDIFYTSK_2

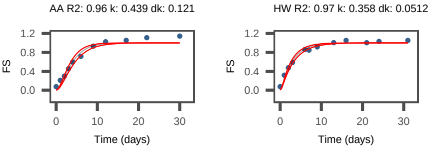

DHPR − MTDSFTEQADQVTADVGK_2

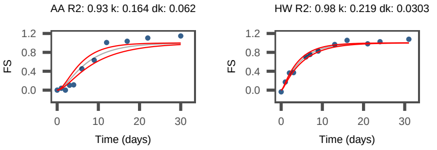

DHSO − LENYPIPELGPNDVLLK_2

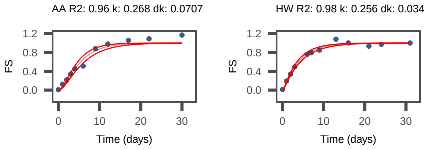

DHE3 − LQHGSILGFPK_3

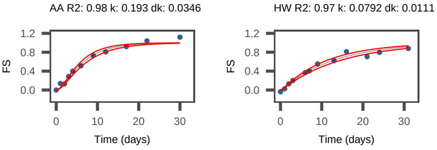

DHRS4 − FGNLMDVTEEVWDK_2

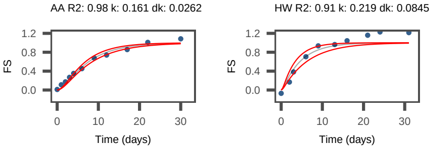

DHTK1 − YGGEGAESMMGFFHELLK_3

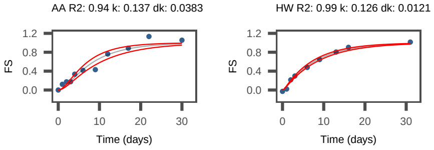

DHE3 − VYEGSILEADCDILIPAASEK_2

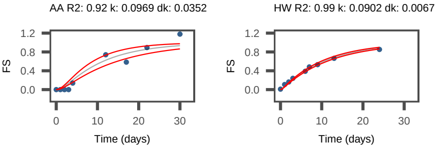

DHRS4 − TALLGLTK_2

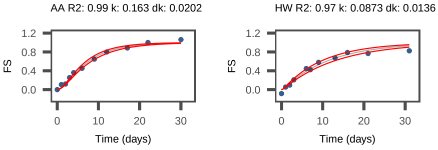

DIC − GEYQGVFHCAMETAK_3

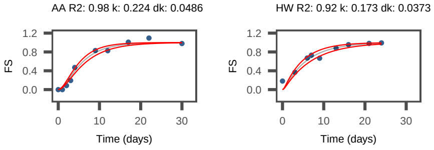

DHE3_BOVIN,sp|P26443|DHE3(Non−Unique) − NYTDNELEK_2

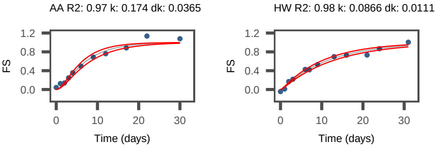

DHRS4 − VNCLAPGLIK_2

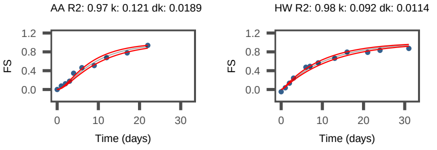

DIC − VHLQTQQEVK_2

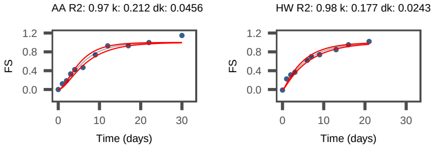

DHE3_BOVIN,sp|P26443|DHE3(Non−Unique) − SEAAADREDDPNFFK_2

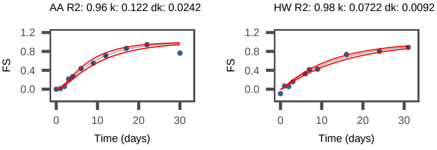

DHSO − AVEAFETAK_2

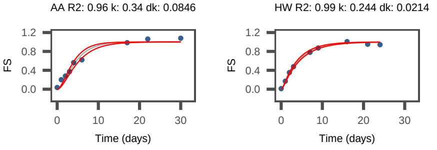

DIC − VHLQTQQEVK_3

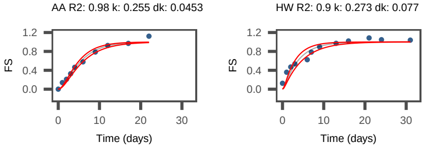

DHE3_BOVIN,sp|P26443|DHE3(Non−Unique) − TAAYVNAIEK_2

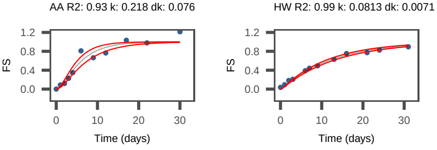

DHSO − ETPQEIASK_2

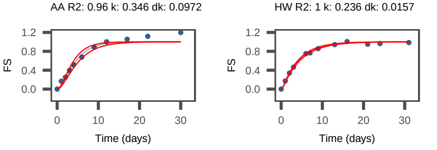

DLDH − ADGSTQVIDTK_2

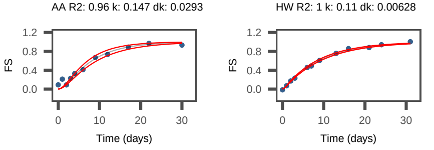

DLDH – ADQPIEADVTVIGSGPGGYVAAIK_2

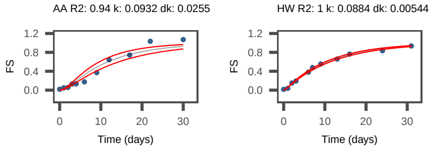

DPEP1 – LAQTHTNIPK_2

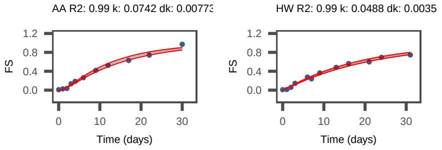

ECHA – LPAKPEVSSDEDVQYR_3

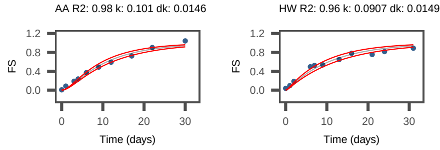

DLDH – ADQPIEADVTVIGSGPGGYVAAIK_3

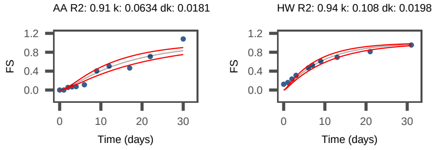

DPEP1 – LAQTHTNIPK_3

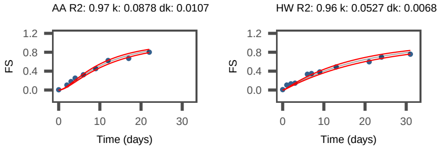

ECHA – MGLVDQLVEPLGPGIK_2

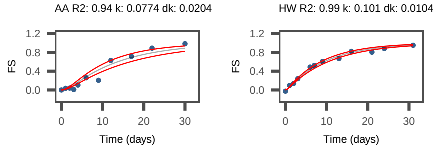

DLDH – ALTGGIAHLFK_2

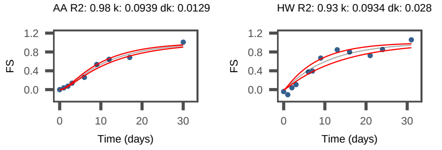

DX39A(Non–Unique) – DFLLKPELLR_3

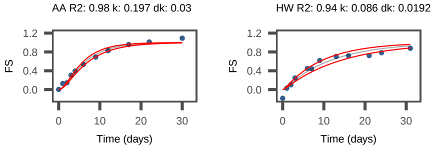

ECHA – MGLVDQLVEPLGPGIK_3

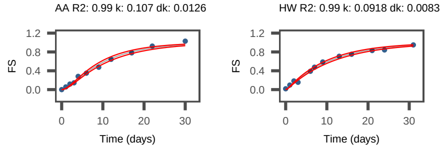

DLDH – ALTGGIAHLFK_3

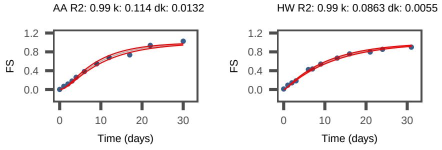

ECHA – ADMVIEAVFEDLGVK_2

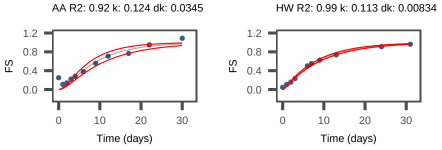

ECHA – MQLLEIITTDK_2

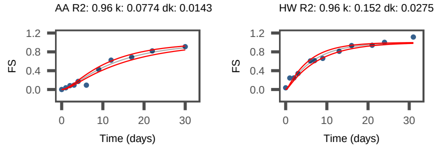

DLDH – IPNIYAIGDVVAGPMLAHK_3

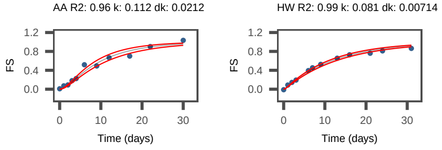

ECHA – AGLEQGSDAGYLAESQK_2

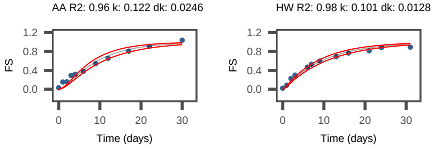

ECHA – NVQQLAILGAGLMGAGIAQVSVDK_2

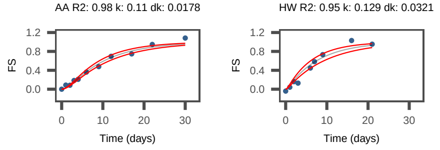

DLDH – RPFTQNLGLEELGIELDPK_3

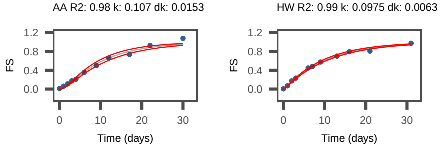

ECHA – FGELALTK_2

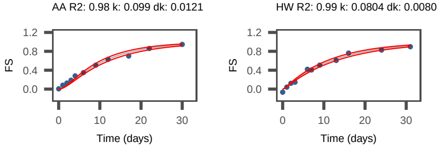

ECHA – NVQQLAILGAGLMGAGIAQVSVDK_3

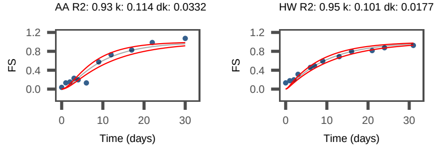

ECHA − TIEYLEEVAVNFAK_3

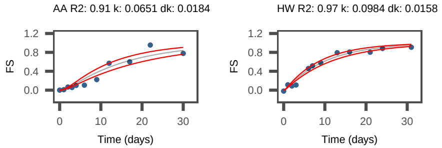

ECHM − IVVAMAK_2

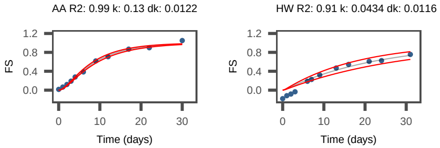

ECHP − LVAQGSPPLK_2

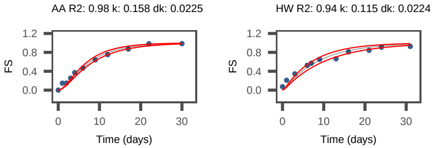

ECHB − DQLLLGPTYATPK_2

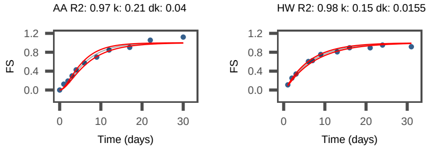

ECHM − NSSVGLIQLNRPK_2

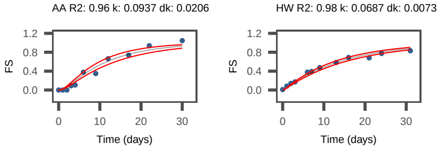

ECHP − SGQASAKPNLR_2

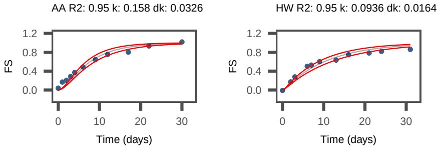

ECHB − DVVDYIIFGTVIQEVK_2

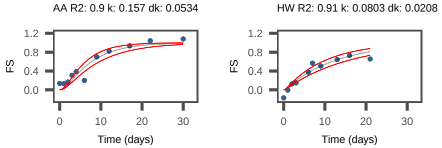

ECHP − ELSSVDLVIEAVFEDMNLK_2

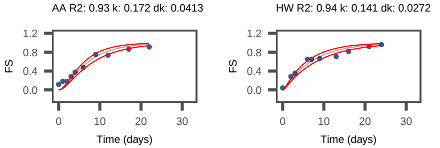

ECHP − SGQASAKPNLR_3

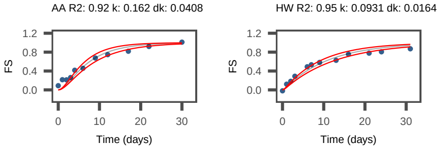

ECHB − DVVDYIIFGTVIQEVK_3

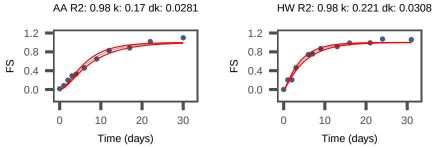

ECHP − GWYQYDKPLGR_3

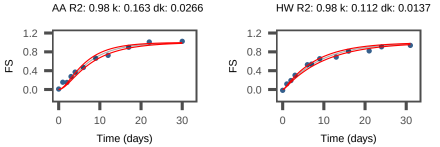

ECHP − VGIPVVAVESDPK_2

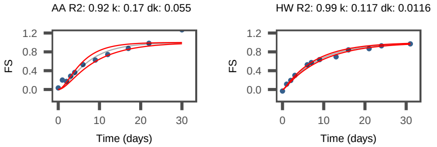

ECHB − EAALGAGFSDK_2

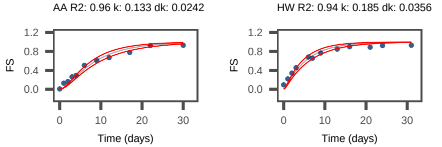

ECHP − IHKPDPWLSEFLSQYR_4

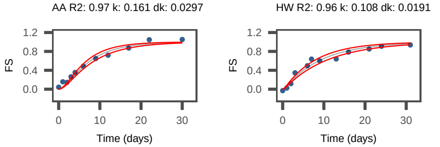

ECHP − VSDLAGLDVGWK_2

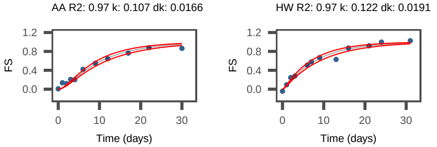

ECHM − AFAAGADIK_2

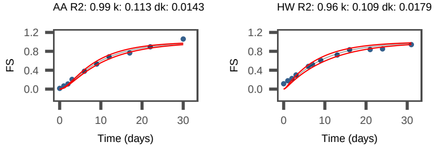

ECHP − LGILDVVVK_2

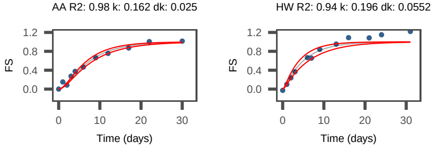

ECHP − YSSPTTIATVMSLSK_2

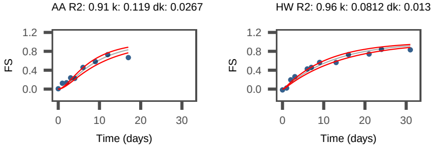

ECI1 − ALQLGTLFSPAEALK_2

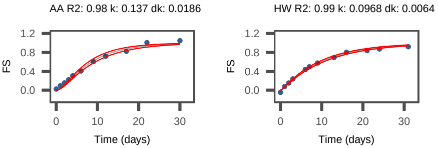

EF1A1(Non−Unique) − STTTGHLIYK_2

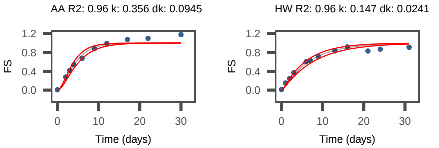

EFTU − TTLTAAITK_2

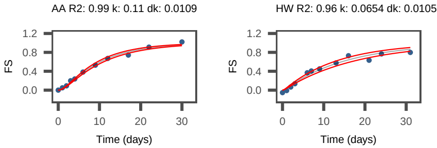

ECI1 − VLVETEGPAGVAVMK_2

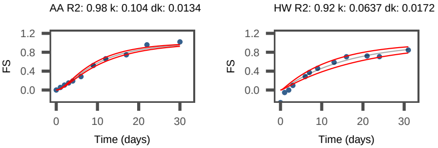

EF1G − ILGLLDTHLK_3

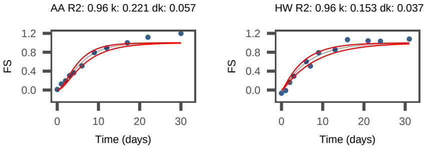

EM55 − FVTGDIIQIINK_2

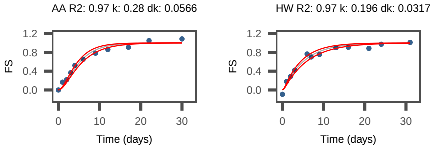

ECSIT − DLSVYNLLLDVFPK_2

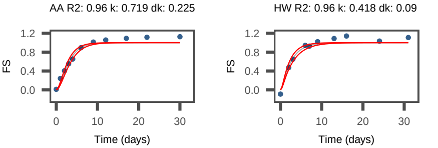

EF2 − VFSGVVSTGLK_2

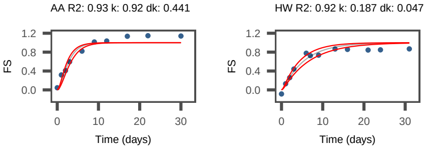

EM55 − IAILDIEPQTLK_2

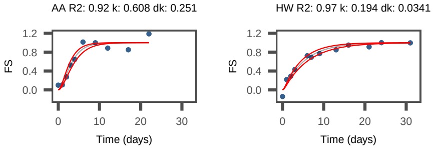

EF1A1 − MDSTEPPYSQK_2

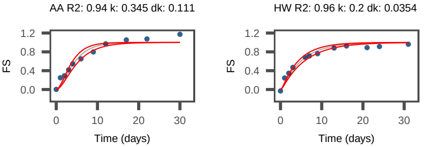

EFTU − DLDKPFLLPVESVYSIPGR_3

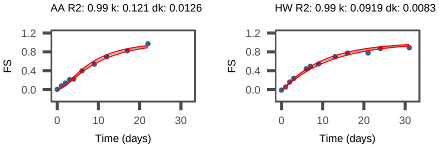

ENOA(Non−Unique) − AILGVSLAVCK_2

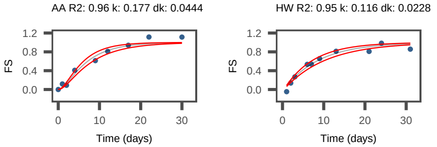

EF1A1(Non−Unique) − QLIVGVNK_2

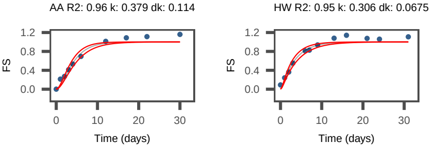

EFTU − ELAMPGEDLK_2

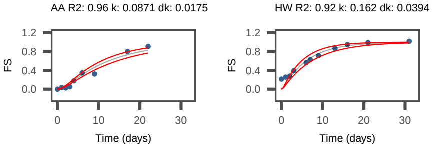

ENOA − FTASAGIQVVGDDLTVTNPK_3

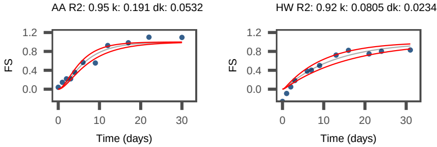

EF1A1 − RYEEIVK_2

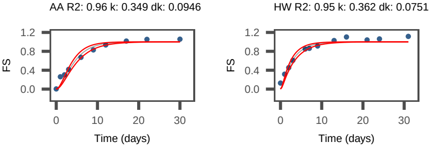

EFTU − QIGVEHVVVYVNK_2

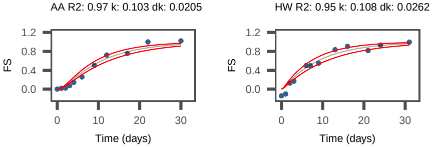

ENOA(Non−Unique) − GNPTVEVDLYTAK_2

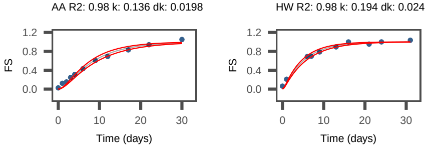

ENOA − GVSQAVEHINK_2

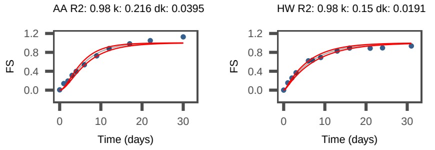

ENPL − TVWDWELMNDIKPIWQR_3

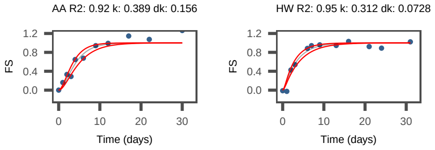

EST1D − ESYPFLPTVIDGVVLPK_2

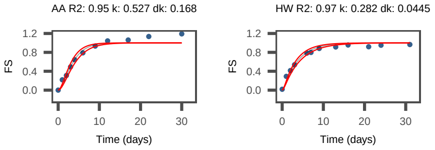

ENOA − SFVQNYPVVSIEDPFDQDDWGAWQK_2

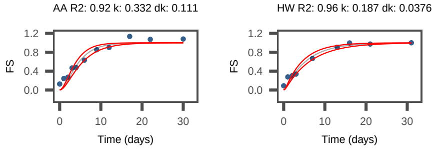

ENPP3 − ITTLLQWLDLPK_2

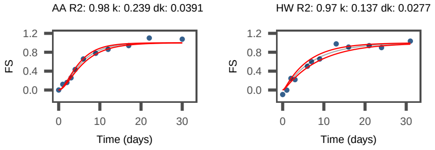

EST1D − QEFGWIIPTLMGYPLAEGK_2

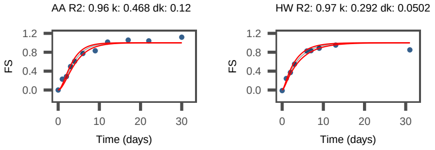

ENOA − SFVQNYPVVSIEDPFDQDDWGAWQK_3

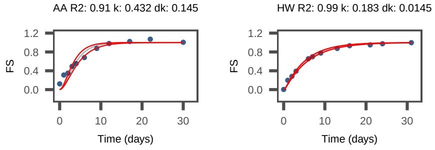

ENTP5 − AQALLLEVEEIFK_2

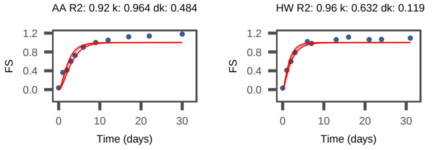

EST1D − QEFGWIIPTLMGYPLAEGK_3

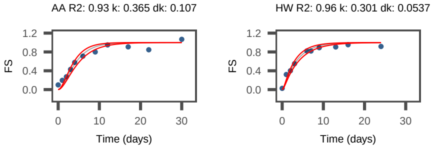

ENOA(Non−Unique) − SGETEDTFIADLVVGLCTGQIK_3

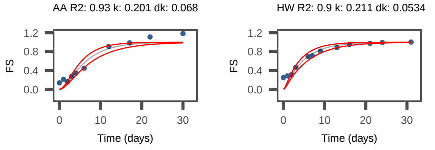

ENTP5 − WLEAEWIFGGVK_2

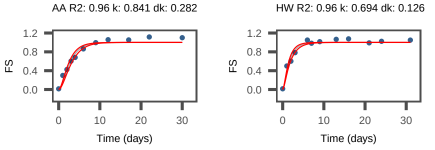

EST1E(Non−Unique) − FAPPQPAEPWSSVK_2

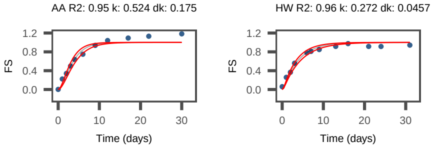

ENOA(Non−Unique) − VNQIGSVTESLQACK_2

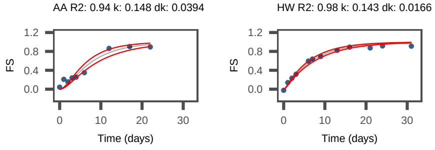

EST1(Non−Unique) − EGASEEEINLSK_2

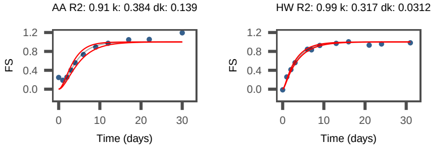

EST1F − AIALLEQFASMTGIPEDIIPVAVEK_2

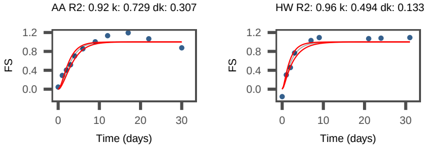

ENOA − YITPDQLADLYK_2

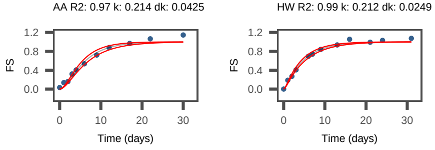

EST1D − AVIGDHGDEIFSVFGSPFLK_3

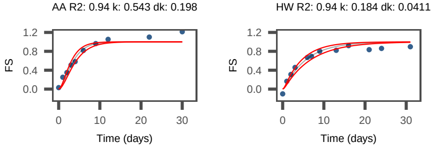

EST1F − AIALLEQFASMTGIPEDIIPVAVEK_3

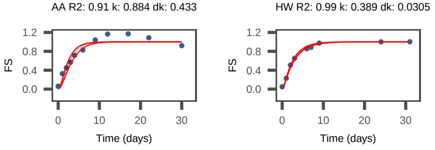

EST1F − AISESGVAFIPGMFTK_2

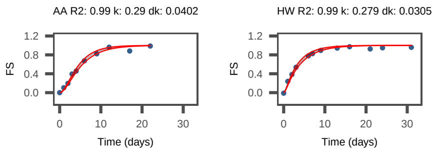

ETFA − DPEAPIFQVADYGIVADLFK_3

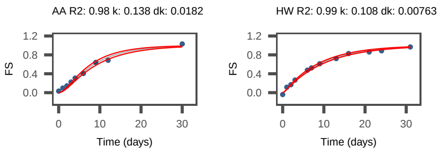

ETFA − VLVAQHDAYK_2

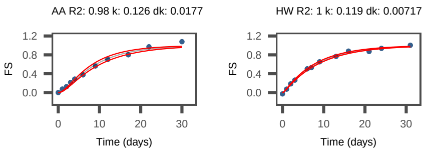

EST1F − GNPSSPPVVDTAHGK_3

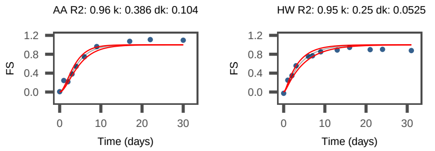

ETFA − GLLPEELTPLILETQK_3

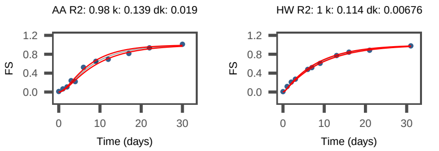

ETFA − VLVAQHDAYK_3

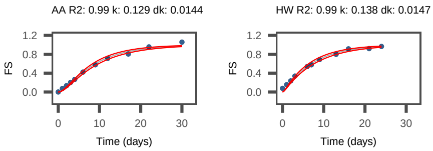

EST1F − LPVMVWIHGGGLK_3

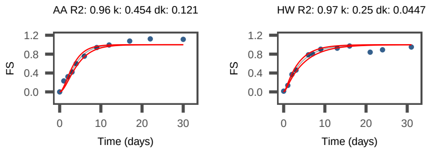

ETFA − GTSFEAAATSGGSASSEK_2

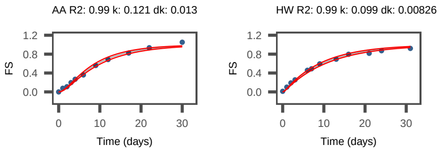

ETFA − VVPEMTEILK_2

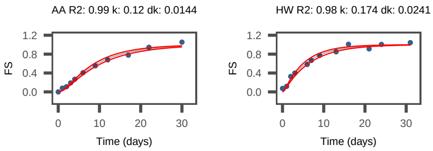

EST2C − SHAPVYFYEFQHPPSYFK_4

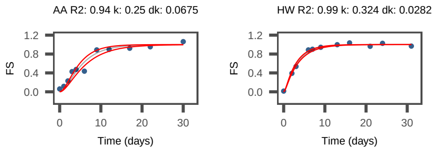

ETFA − IVAPELYIAVGISGAIQHLAGMK_3

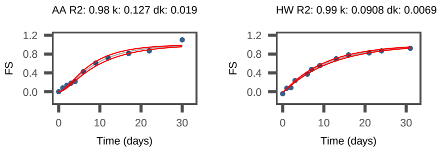

ETFB − SGVVTDGVK_2

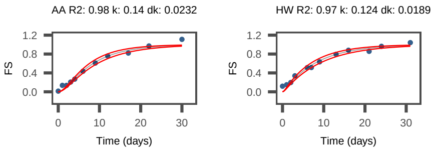

ESTD − VFEHSSVELK_2

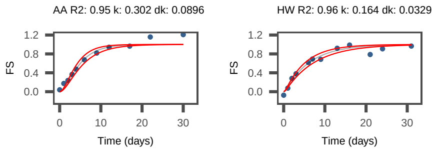

ETFA − LNVAPVSDIIEIK_2

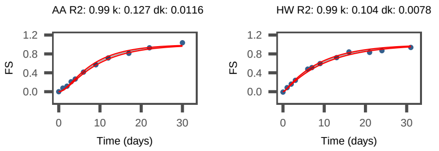

ETFB − VDLLFLGK_2

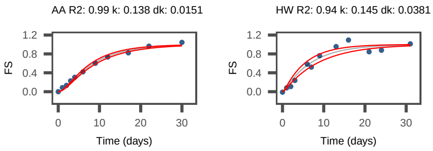

ETFA − DPEAPIFQVADYGIVADLFK_2

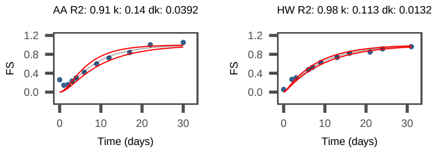

ETFA − SDRPELTGAK_3

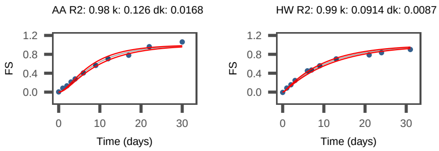

ETFB − VIDFAVK_2

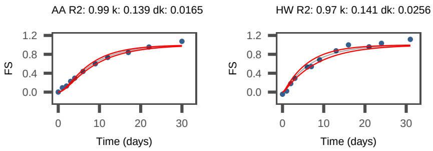

ETFD – FAILTEK_2

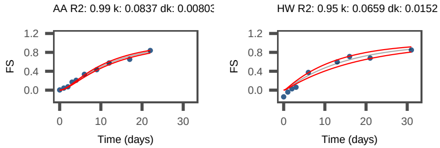

EZRI – SQEQLAAELAEYTAK_2

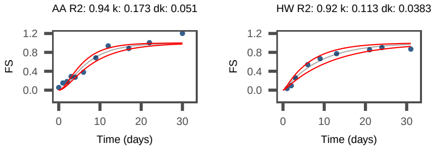

FAAA – DIQQWEYVPLGPFLGK_2

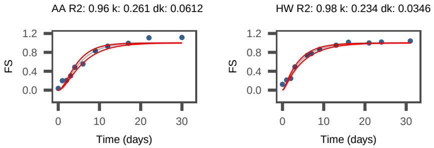

ETFD – HHPSIQPTLEGGK_3

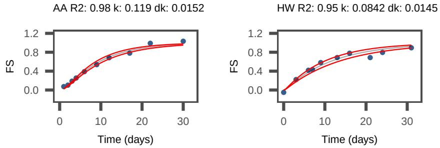

F16P1 – GNIYSLNEGYAK_2

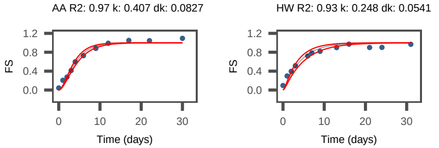

FAAA – DIQQWEYVPLGPFLGK_3

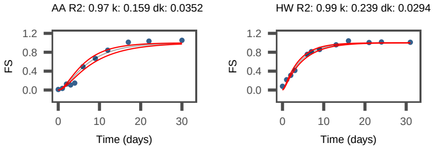

EZRI – EVWYFGLQYVDNK_2

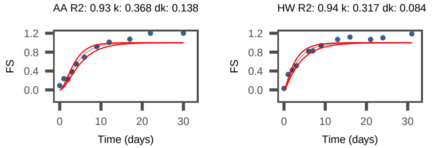

F16P1 – LDILSNDLVINMLK_2

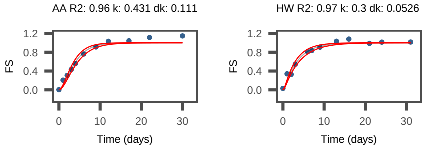

FAAA – HLFTGPALSK_2

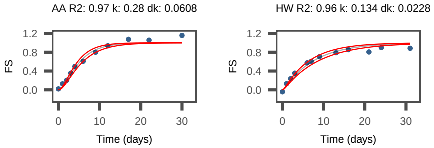

EZRI – FYPEDVAEELIQDITQK_3

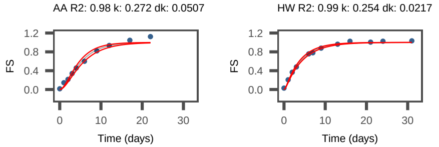

F16P1 – LDILSNDLVINMLK_3

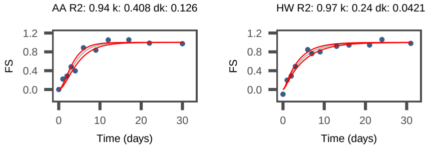

FAAA – HQHVFDETTLNNFMGLGQAAWK_4

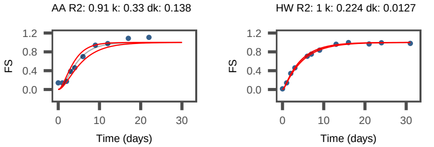

EZRI – GTDLWLGVDALGLNIYEK_2

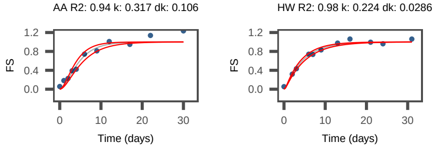

F16P1 – QAGIAQLYGIAGSTNVTGDQVK_2

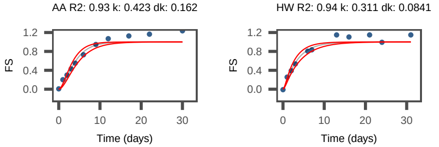

FABP4 – LVSSENFDDYMK_2

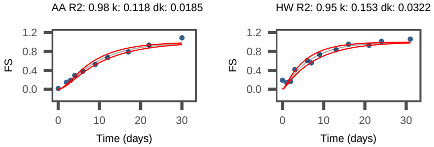

EZRI – RKEDEVEEWQHR_4

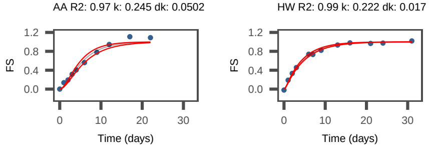

F16P1 – TLVYGGIFLYPANK_2

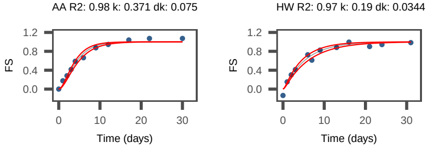

FAHD1 – IITLEEGDLILTGTPK_2

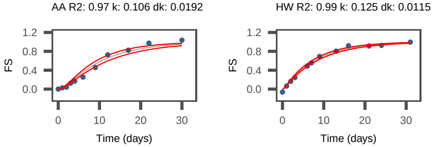

FAHD2 − FSSSIVGPYDEIILPPESK_2

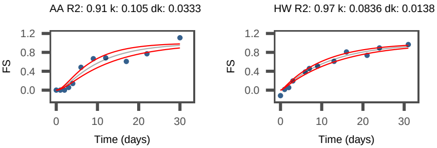

FIS1 − GLLQTEPQNNQAK_2

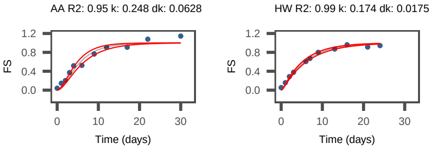

FLNB(Non−Unique) − LIALLEVLSQK_3

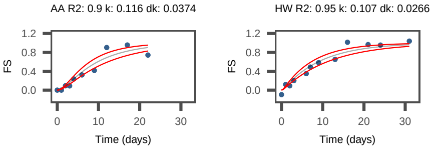

FERM2 − TMADSSYNLEVQNILSFLK_2

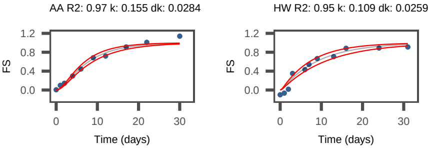

FLNA − GAGTGGLGLAVEGPSEAK_2

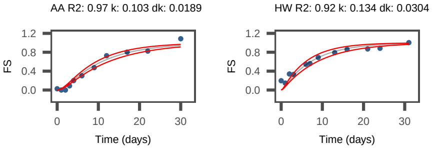

FMO1 − NLLPTPIVSWLISK_2

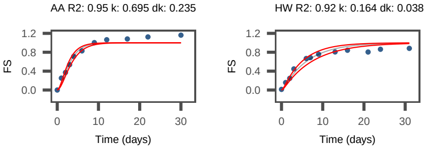

FHL1 − FTAVEDQYYCVDCYK_2

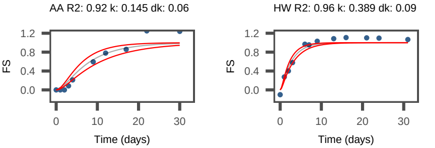

FLNA − VTAQGPGLEPSGNIANK_2

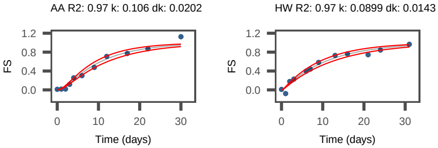

FMO1 − VAIVGAGVSGLASIK_2

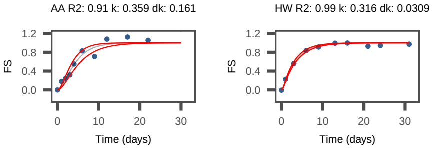

FIBA − QLQQVIAK_2

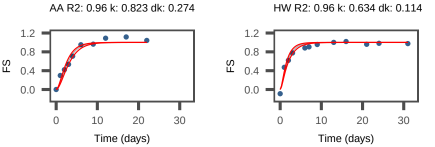

FLNB(Non−Unique) − EAGAGGLSIAVEGPSK_2

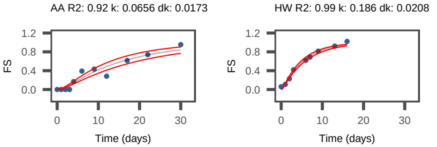

FMO4 − VAVIGAGVSGLSSIK_2

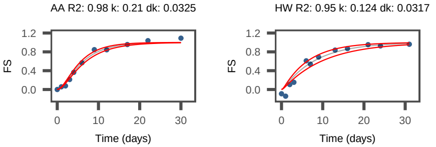

FIBG − SSTTNGFDDGIIWATWK_2

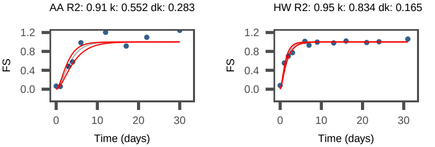

FLNB − GAGIGGLGITVEGPSESK_2

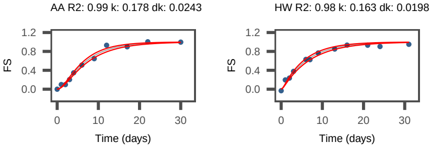

FMO5 − IIAGLVK_2

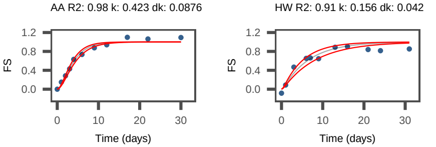

FIS1 − GIVLLEELLPK_2

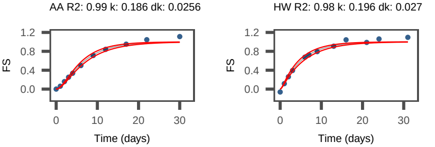

FLNB(Non−Unique) − LIALLEVLSQK_2

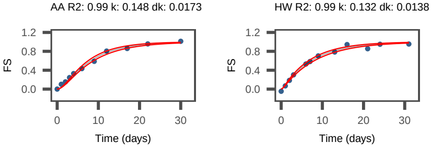

FOLH1 − GMILYSDPADYFVPAVK_2

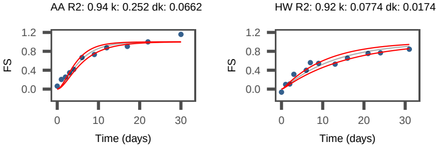

FOLH1 − YAGESFPGIYDALFDISSK_2

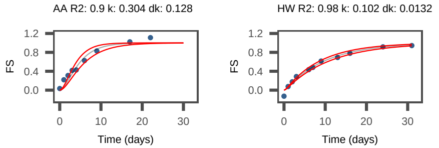

FUMH − VEFDTFGELK_2

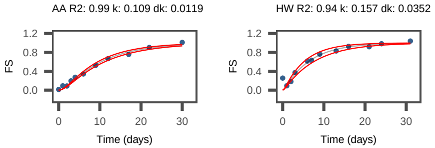

G3P − VIHDNFGIVEGLMTTVHAITATQK_4

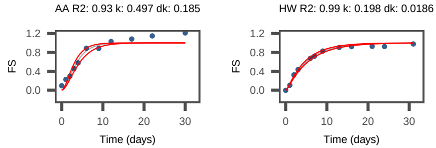

FUMH − AAAEVNQEYGLDPK_2

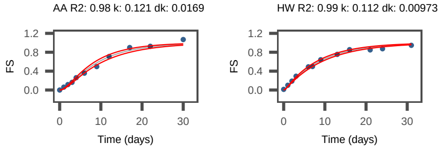

FUMH − VLLPGLQK_2

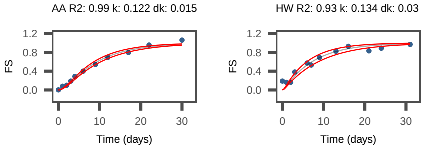

G3P − VIISAPSADAPMFVMGVNHEK_2

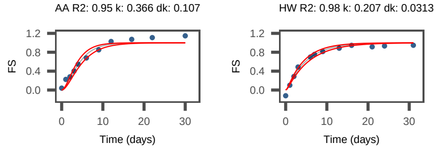

FUMH − FEALAAHDALVELSGAMNTAACSLMK_3

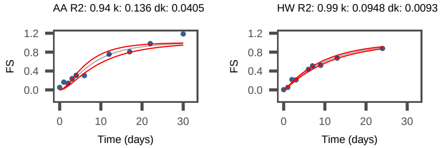

G3P − GAAQNIIPASTGAAK_2

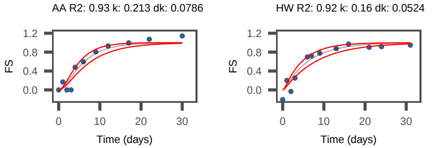

G3P − WGEAGAEYVVESTGVFTTMEK_2

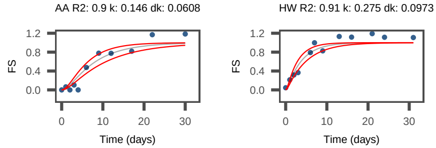

FUMH − LHDALSAK_2

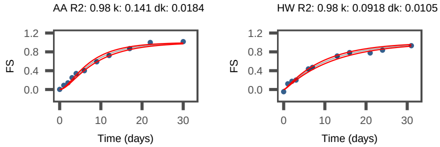

G3P − MFQYDSTHGK_2

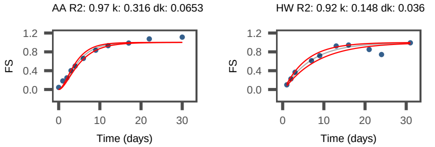

G6PI − EVMQMLVELAK_2

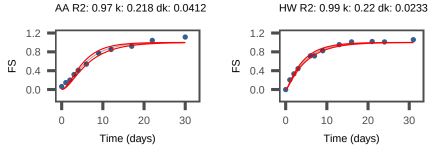

FUMH − SQSSNDTFPTAMHIAAAVEVHK_4

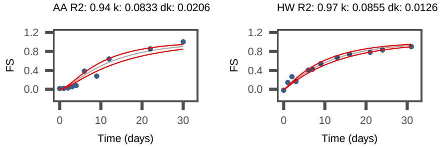

G3P − MFQYDSTHGK_3

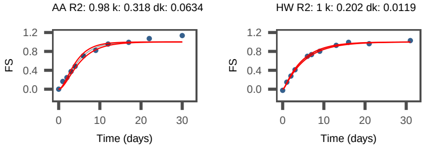

G6PI − EWFLEAAK_2

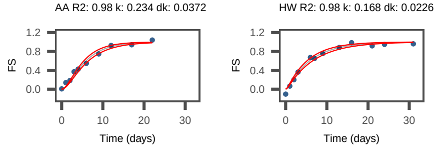

FUMH − VAALTGLPFVTAPNK_2

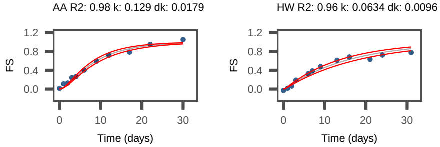

G3P − SSTFDAGAGIALNDNFVK_2

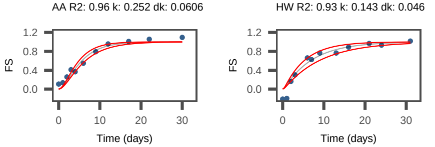

G6PI − FAAYFQQGDMESNGK_2

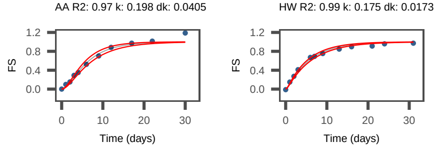

G6PI − HFVALSTNTAK_2

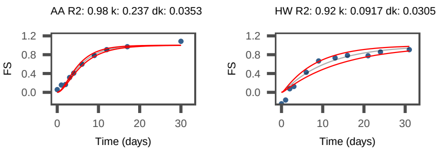

GABT − NLLLAEVINIIK_2

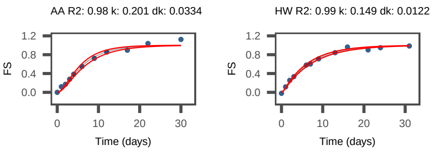

GAS2 − EDLALWLTNLLGK_2

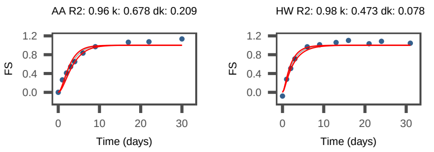

G6PI − ILGALIAMYEHK_3

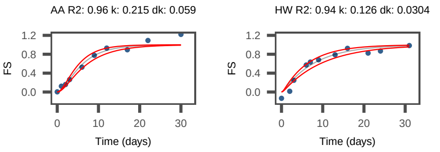

GABT − NLLLAEVINIIK_3

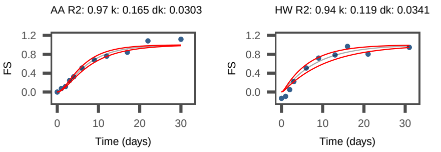

GATM − VMVDANEVPIQK_2

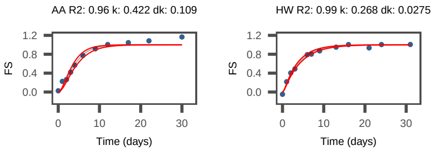

G6PI − ILLANFLAQTEALMK_2

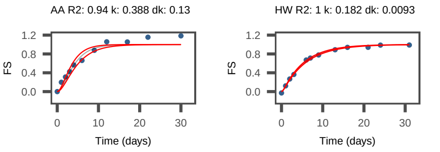

GAL3A − GGAEVQIFAPDVPQMHVIDHTK_4

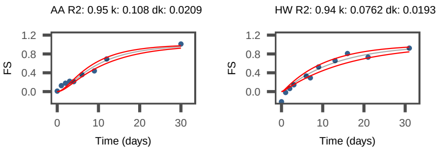

GCDH − DIVYEMGELGVLGPTIK_2

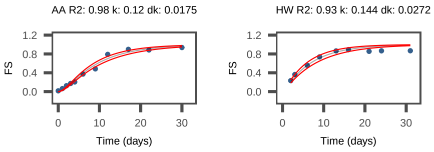

G6PI − TLASLSPETSLFIIASK_2

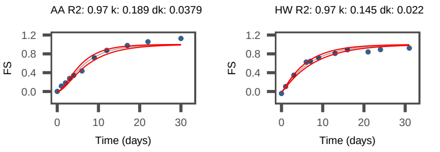

GAL3A − GVEVTVGHEQEEGGK_3

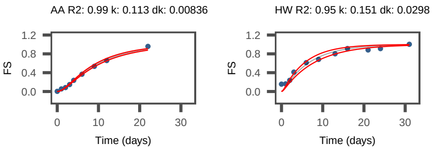

GCDH − HNPSNQSYTLSGTK_2

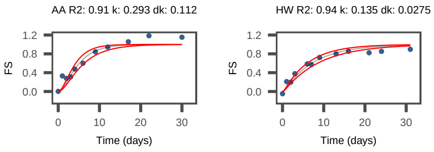

G6PI − VFEGNRPTNSIVFTK_2

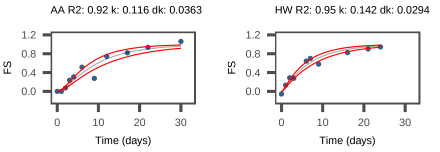

GALM − ASDVVLGFAELEGYLQK_3

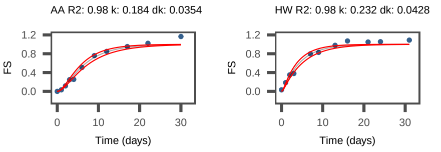

GDIA − NPYYGGESSSITPLEELYK_2

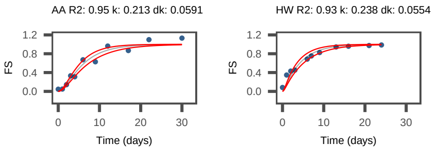

G6PI − VWFVSNIDGTHIAK_3

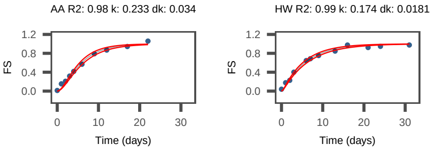

GALNS − ILSLLQNLGISK_2

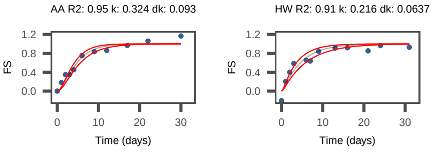

GDIB − FVSISDLFVPK_2

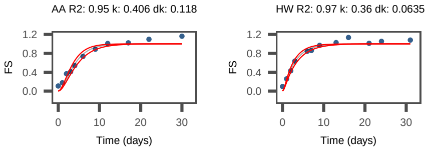

GDIB − VPSTEAEALASSLMGLFEK_3

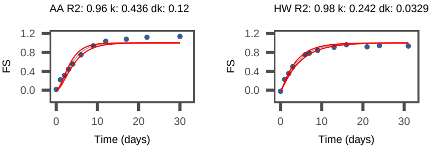

GLO2 − VTHLSTLQVGSLSVK_3

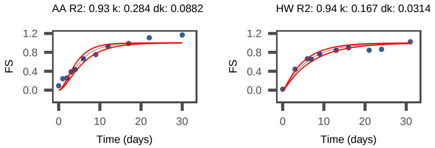

GLYAT − HSENILYVSSETIK_2

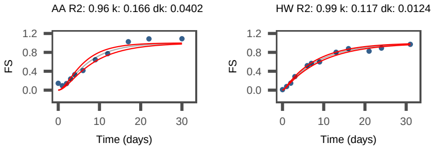

GDIB − YIAIVSTTVETK_2

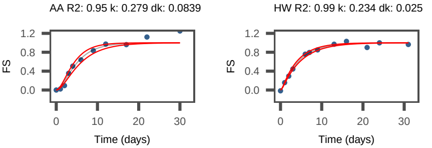

GLPK − TAELLSHHQVEIK_2

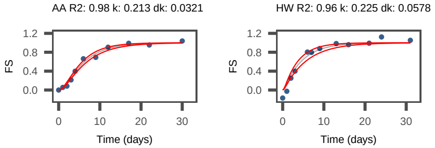

GLYAT − HSENILYVSSETIK_3

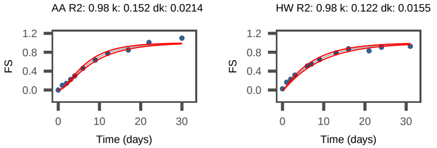

GDIR1 − AEEYEFLTPMEEAPK_2

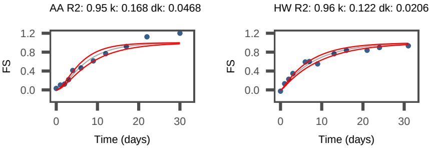

GLPK(Non−Unique) − TGLPLSTYFSAVK_2

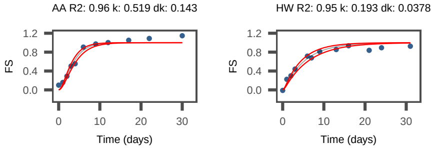

GLYAT − IVPLQGAQMLQMLEK_2

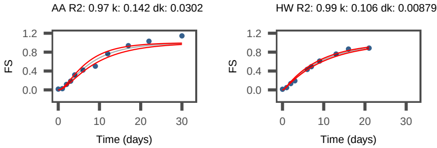

GGT1 − LADTLQILAQEGAK_2

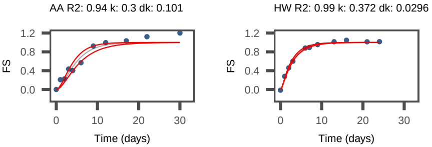

GLUCM − AGEQSWIQALPSVAK_2

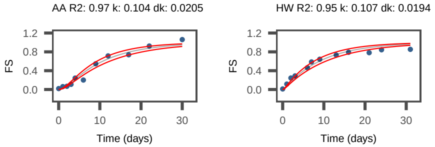

GLYAT − LFPSLLDTK_2

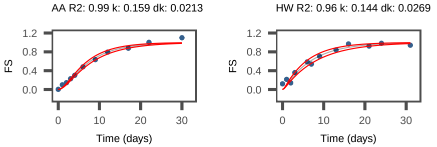

GGT1 − VLQEGETVTMPK_2

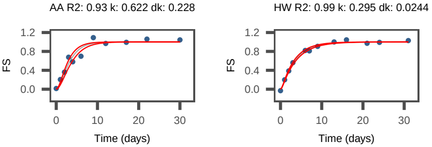

GLYAL(Non−Unique) − YLPESLK_2

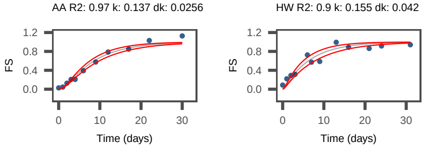

GLYAT − QHLQIQSSQSHLNK_3

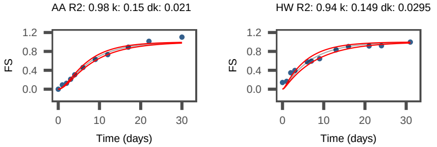

GLO2 − FYEGTADEMYK_2

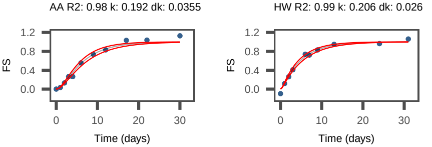

GLYAT − GYPVYSHTEK_3

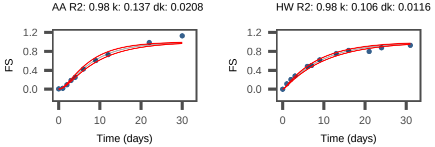

GLYAT − QHLQIQSSQSHLNK_4

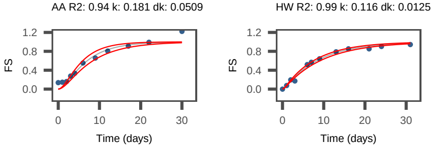

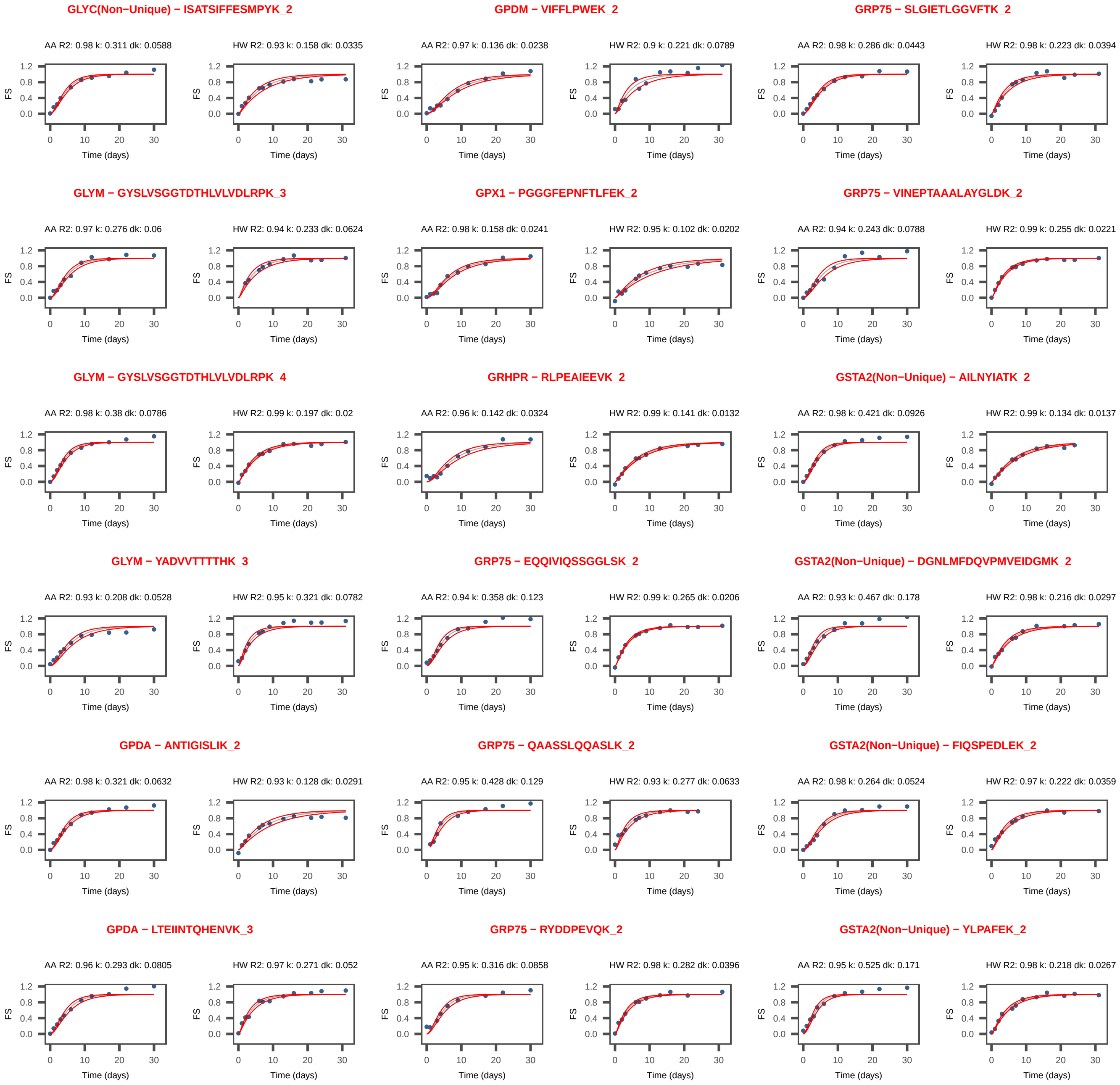
GLYC(Non−Unique) − ISATSIFFESMPYK_2
AA R2: 0.98 k: 0.311 dk: 0.0588
HW R2: 0.93 k: 0.158 dk: 0.0335
GPDM − VIFFLPWEK_2
AA R2: 0.97 k: 0.136 dk: 0.0238
HW R2: 0.9 k: 0.221 dk: 0.0789
GRP75 − SLGIETLGGVFTK_2
AA R2: 0.98 k: 0.286 dk: 0.0443
HW R2: 0.98 k: 0.223 dk: 0.0394
GLYM − GYSLVSGGTDTHLVLVDLRPK_3
AA R2: 0.97 k: 0.276 dk: 0.06
HW R2: 0.94 k: 0.233 dk: 0.0624
GPX1 − PGGGFEPNFTLFEK_2
AA R2: 0.98 k: 0.158 dk: 0.0241
HW R2: 0.95 k: 0.102 dk: 0.0202
GRP75 − VINEPTAAALAYGLDK_2
AA R2: 0.94 k: 0.243 dk: 0.0788
HW R2: 0.99 k: 0.255 dk: 0.0221
GLYM − GYSLVSGGTDTHLVLVDLRPK_4
AA R2: 0.98 k: 0.38 dk: 0.0786
HW R2: 0.99 k: 0.197 dk: 0.02
GRHPR − RLPEAIEEVK_2
AA R2: 0.96 k: 0.142 dk: 0.0324
HW R2: 0.99 k: 0.141 dk: 0.0132
GSTA2(Non−Unique) − AILNYIATK_2
AA R2: 0.98 k: 0.421 dk: 0.0926
HW R2: 0.99 k: 0.134 dk: 0.0137
GLYM − YADVVTTTTHK_3
AA R2: 0.93 k: 0.208 dk: 0.0528
HW R2: 0.95 k: 0.321 dk: 0.0782
GRP75 − EQQIVIQSSGGLSK_2
AA R2: 0.94 k: 0.358 dk: 0.123
HW R2: 0.99 k: 0.265 dk: 0.0206
GSTA2(Non−Unique) − DGNLMFDQVPMVEIDGMK_2
AA R2: 0.93 k: 0.467 dk: 0.178
HW R2: 0.98 k: 0.216 dk: 0.0297
GPDA − ANTIGISLIK_2
AA R2: 0.98 k: 0.321 dk: 0.0632
HW R2: 0.93 k: 0.128 dk: 0.0291
GRP75 − QAASSLQQASLK_2
AA R2: 0.95 k: 0.428 dk: 0.129
HW R2: 0.93 k: 0.277 dk: 0.0633
GSTA2(Non−Unique) − FIQSPEDLEK_2
AA R2: 0.98 k: 0.264 dk: 0.0524
HW R2: 0.97 k: 0.222 dk: 0.0359
GPDA − LTEIINTQHENVK_3
AA R2: 0.96 k: 0.293 dk: 0.0805
HW R2: 0.97 k: 0.271 dk: 0.052
GRP75 − RYDDPEVQK_2
AA R2: 0.95 k: 0.316 dk: 0.0858
HW R2: 0.98 k: 0.282 dk: 0.0396
GSTA2(Non−Unique) − YLPAFEK_2
AA R2: 0.95 k: 0.525 dk: 0.171
HW R2: 0.98 k: 0.218 dk: 0.0267
FS
Time (days)

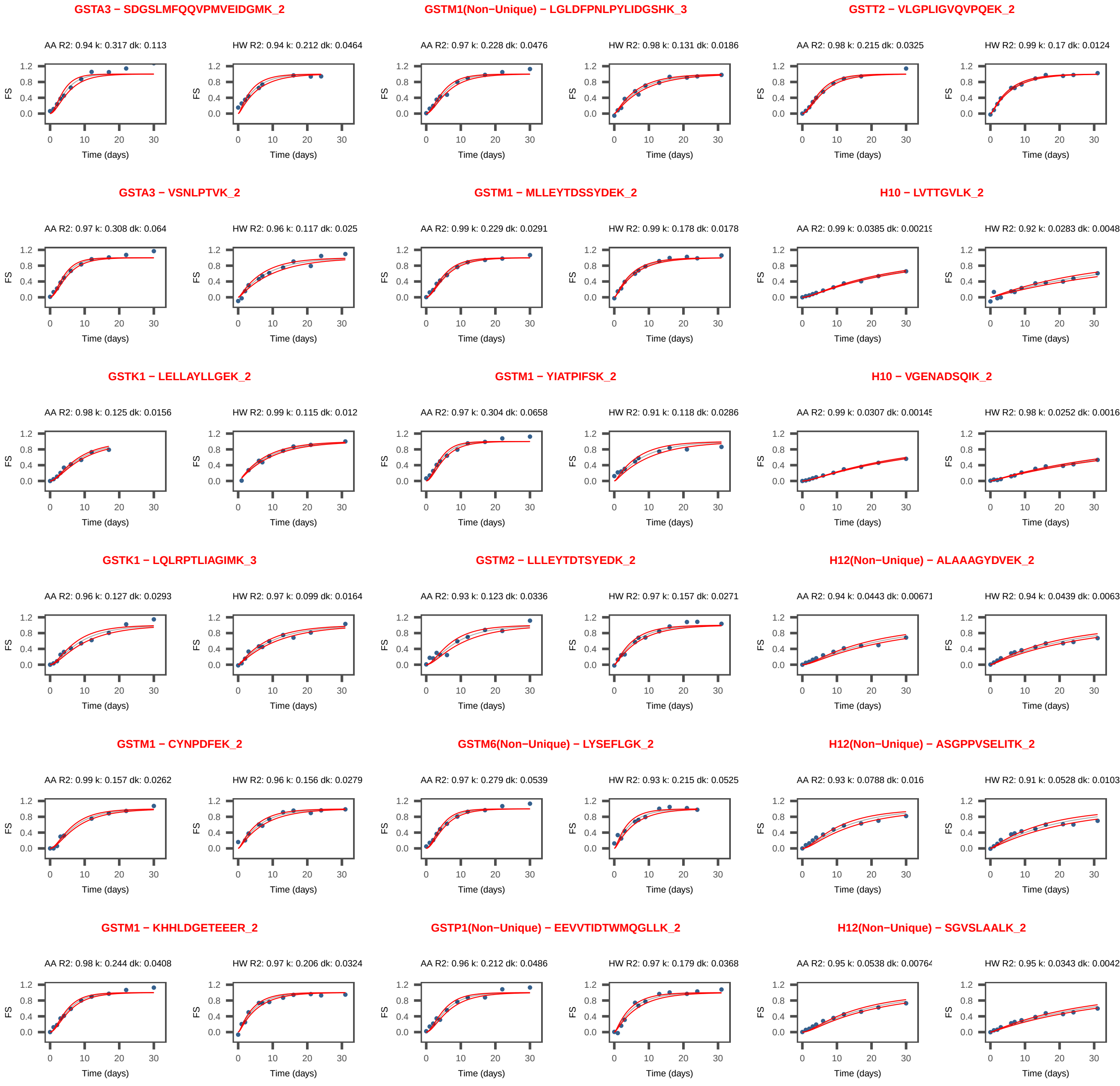
GSTA3 − SDGSLMFQQVPMVEIDGMK_2
AA R2: 0.94 k: 0.317 dk: 0.113
HW R2: 0.94 k: 0.212 dk: 0.0464
GSTM1(Non−Unique) − LGLDFPNLPYLIDGSHK_3
AA R2: 0.97 k: 0.228 dk: 0.0476
HW R2: 0.98 k: 0.131 dk: 0.0186
GSTT2 − VLGPLIGVQVPQEK_2
AA R2: 0.98 k: 0.215 dk: 0.0325
HW R2: 0.99 k: 0.17 dk: 0.0124
GSTA3 − VSNLPTVK_2
AA R2: 0.97 k: 0.308 dk: 0.064
HW R2: 0.96 k: 0.117 dk: 0.025
GSTM1 − MLLEYTDSSYDEK_2
AA R2: 0.99 k: 0.229 dk: 0.0291
HW R2: 0.99 k: 0.178 dk: 0.0178
H10 − LVTTGVLK_2
AA R2: 0.99 k: 0.0385 dk: 0.0021
HW R2: 0.92 k: 0.0283 dk: 0.0048
GSTK1 − LELLAYLLGEK_2
AA R2: 0.98 k: 0.125 dk: 0.0156
HW R2: 0.99 k: 0.115 dk: 0.012
GSTM1 − YIATPIFSK_2
AA R2: 0.97 k: 0.304 dk: 0.0658
HW R2: 0.91 k: 0.118 dk: 0.0286
H10 − VGENADSQIK_2
AA R2: 0.99 k: 0.0307 dk: 0.0014
HW R2: 0.98 k: 0.0252 dk: 0.0016
GSTK1 − LQLRPTLIAGIMK_3
AA R2: 0.96 k: 0.127 dk: 0.0293
HW R2: 0.97 k: 0.099 dk: 0.0164
GSTM2 − LLLEYTDTSYEDK_2
AA R2: 0.93 k: 0.123 dk: 0.0336
HW R2: 0.97 k: 0.157 dk: 0.0271
H12(Non−Unique) − ALAAAGYDVEK_2
AA R2: 0.94 k: 0.0443 dk: 0.00671
HW R2: 0.94 k: 0.0439 dk: 0.0063
GSTM1 − CYNPDFEK_2
AA R2: 0.99 k: 0.157 dk: 0.0262
HW R2: 0.96 k: 0.156 dk: 0.0279
GSTM6(Non−Unique) − LYSEFLGK_2
AA R2: 0.97 k: 0.279 dk: 0.0539
HW R2: 0.93 k: 0.215 dk: 0.0525
H12(Non−Unique) − ASGPPVSELITK_2
AA R2: 0.93 k: 0.0788 dk: 0.016
HW R2: 0.91 k: 0.0528 dk: 0.0103
GSTM1 − KHHLDGETEEER_2
AA R2: 0.98 k: 0.244 dk: 0.0408
HW R2: 0.97 k: 0.206 dk: 0.0324
GSTP1(Non−Unique) − EEVVTIDTWMQGLLK_2
AA R2: 0.96 k: 0.212 dk: 0.0486
HW R2: 0.97 k: 0.179 dk: 0.0368
H12(Non−Unique) − SGVSLAALK_2
AA R2: 0.95 k: 0.0538 dk: 0.0076
HW R2: 0.95 k: 0.0343 dk: 0.0042
FS
Time (days)

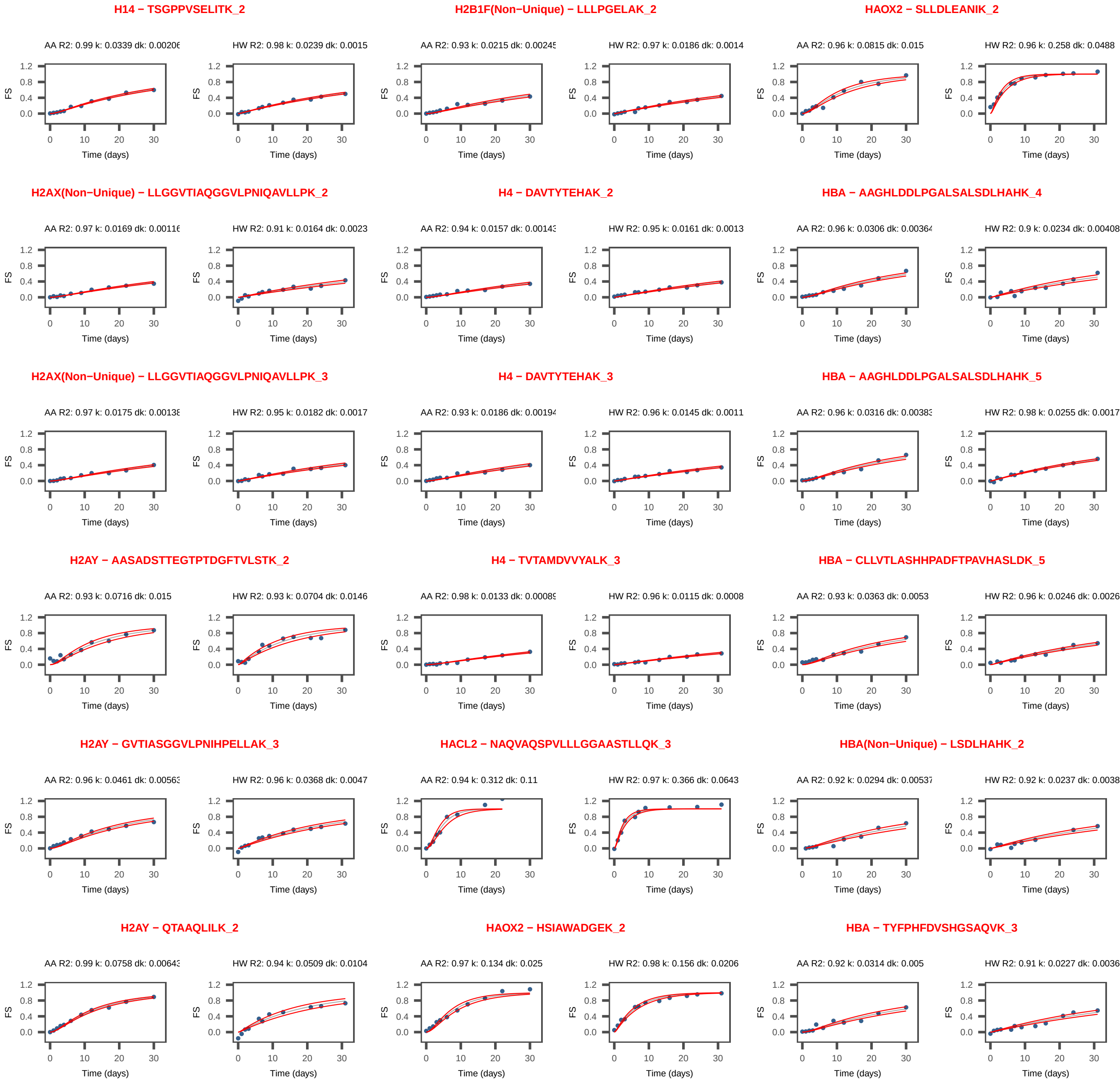
H14 – TSGPPVSELITK_2
AA R2: 0.99 k: 0.0339 dk: 0.00206
HW R2: 0.98 k: 0.0239 dk: 0.0015
H2B1F(Non–Unique) – LLLPGELAK_2
AA R2: 0.93 k: 0.0215 dk: 0.00245
HW R2: 0.97 k: 0.0186 dk: 0.0014
HAOX2 – SLLDLEANIK_2
AA R2: 0.96 k: 0.0815 dk: 0.015
HW R2: 0.96 k: 0.258 dk: 0.0488
H2AX(Non–Unique) – LLGGVTIAQGGVLPNIQAVLLPK_2
AA R2: 0.97 k: 0.0169 dk: 0.00116
HW R2: 0.91 k: 0.0164 dk: 0.0023
H4 – DAVTYTEHAK_2
AA R2: 0.94 k: 0.0157 dk: 0.00143
HW R2: 0.95 k: 0.0161 dk: 0.0013
HBA – AAGHLDDLPGALSALSDLHAHK_4
AA R2: 0.96 k: 0.0306 dk: 0.00364
HW R2: 0.9 k: 0.0234 dk: 0.00408
H2AX(Non–Unique) – LLGGVTIAQGGVLPNIQAVLLPK_3
AA R2: 0.97 k: 0.0175 dk: 0.00138
HW R2: 0.95 k: 0.0182 dk: 0.0017
H4 – DAVTYTEHAK_3
AA R2: 0.93 k: 0.0186 dk: 0.00194
HW R2: 0.96 k: 0.0145 dk: 0.0011
HBA – AAGHLDDLPGALSALSDLHAHK_5
AA R2: 0.96 k: 0.0316 dk: 0.00383
HW R2: 0.98 k: 0.0255 dk: 0.0017
H2AY – AASADSTTEGTPTDGFTVLSTK_2
AA R2: 0.93 k: 0.0716 dk: 0.015
HW R2: 0.93 k: 0.0704 dk: 0.0146
H4 – TVTAMDVVYALK_3
AA R2: 0.98 k: 0.0133 dk: 0.00089
HW R2: 0.96 k: 0.0115 dk: 0.0008
HBA – CLLVTLASHHPADFTPAVHASLDK_5
AA R2: 0.93 k: 0.0363 dk: 0.0053
HW R2: 0.96 k: 0.0246 dk: 0.0026
H2AY – GVTIASGGVLPNIHPELLAK_3
AA R2: 0.96 k: 0.0461 dk: 0.00563
HW R2: 0.96 k: 0.0368 dk: 0.0047
HACL2 – NAQVAQSPVLLLGGAASTLLQK_3
AA R2: 0.94 k: 0.312 dk: 0.11
HW R2: 0.97 k: 0.366 dk: 0.0643
HBA(Non–Unique) – LSDLHAHK_2
AA R2: 0.92 k: 0.0294 dk: 0.00537
HW R2: 0.92 k: 0.0237 dk: 0.0038
H2AY – QTAAQLILK_2
AA R2: 0.99 k: 0.0758 dk: 0.00643
HW R2: 0.94 k: 0.0509 dk: 0.0104
HAOX2 – HSIAWADGEK_2
AA R2: 0.97 k: 0.134 dk: 0.025
HW R2: 0.98 k: 0.156 dk: 0.0206
HBA – TYFPHFDVSHGSAQVK_3
AA R2: 0.92 k: 0.0314 dk: 0.005
HW R2: 0.91 k: 0.0227 dk: 0.0036
FS
Time (days)

HCD2 − VVTIAPGLFATPLLTTLPEK_2

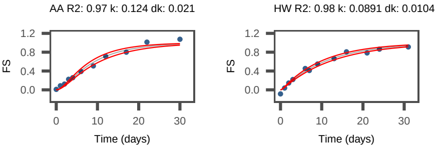

HEM2 − AGADIIITYFAPQLLK_3

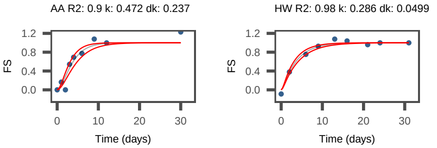

HNRPD(Non−Unique) − GFGFVLFK_2

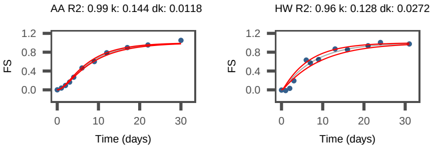

HCD2 − VVTIAPGLFATPLLTTLPEK_3

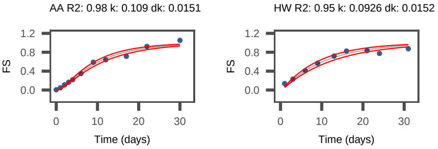

HEMH − VGPVPWLGPQTDEAIK_2

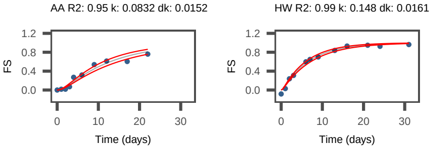

HNRPK − GSYGDLGGPIITTQVTIPK_2

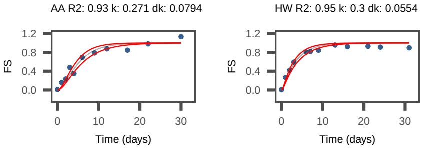

HCDH − LGAGYPMGPFELLDYVGLDTTK_2

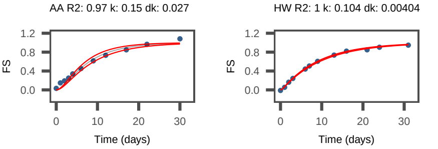

HIBCH − VLEEELLALK_2

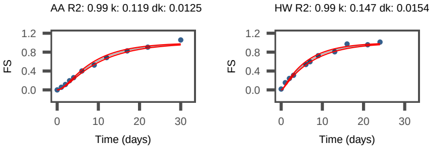

HNRPL − SDALETLGFLNHYQMK_3

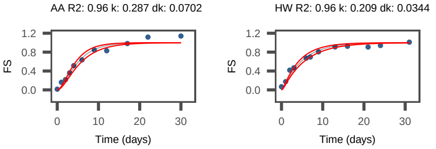

HCDH − LGAGYPMGPFELLDYVGLDTTK_3

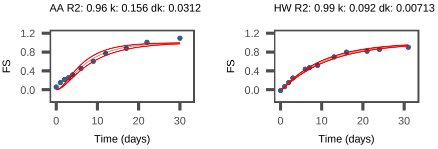

HINT1 − AQVAQPGGDTIFGK_2

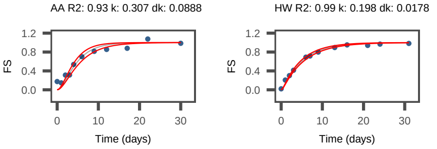

HOGA1 − FGIPGLK_2

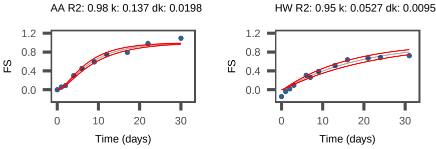

HCDH − TLSCLSTSTDAASVVHSTDLVVEAIVENLK_3

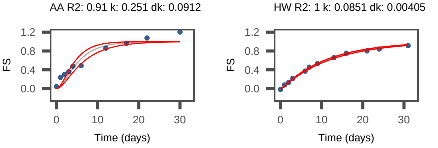

HINT2 − ISQAEEDDQQLLGHLLLVAK_3

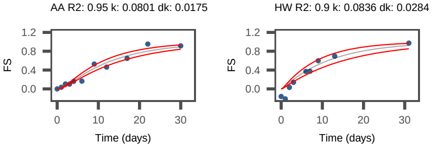

HS90A − ELHINLIPSK_3

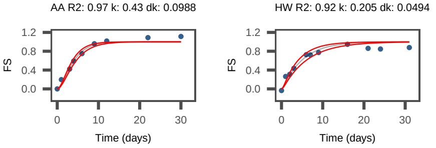

HEM2 − AGADIIITYFAPQLLK_2

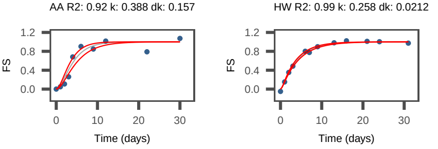

HMGCL − LIDMLSEAGLPVIEATSFVSPK_2

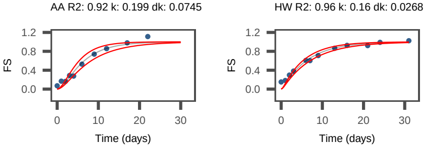

HS90A − NPDDITNEEYGEFYK_2

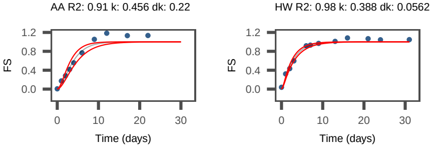

HS90A − YYTSASGDEMVSLK_2

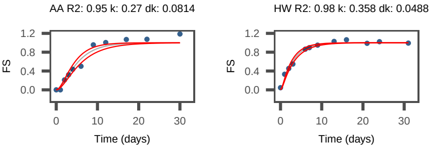

HYES − ASDETGFIAVHK_2

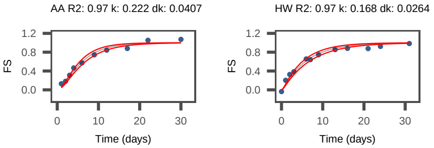

IAH1 − DCGTDVLDLWTLMQK_2

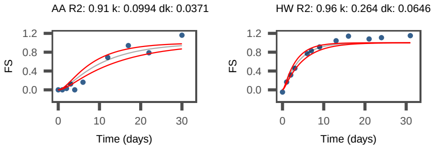

HS90B − APFDLFENK_2

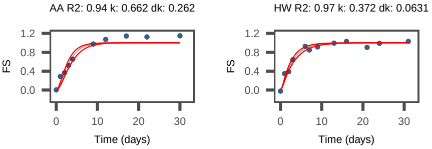

HYES − ATEIGGILVNTPEDPNLSK_2

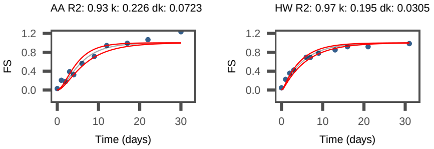

IDHC(Non−Unique) − IIWELIK_2

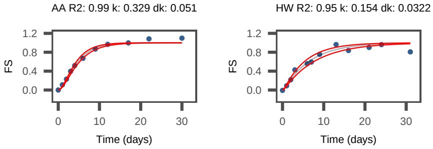

HS90B − NPDDITQEEYGEFYK_2

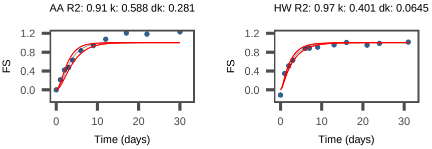

HYES − AVASLNTPFMPPDPDVSPMK_2

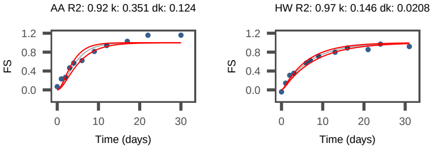

IDHC − LDNNTELSFFAK_2

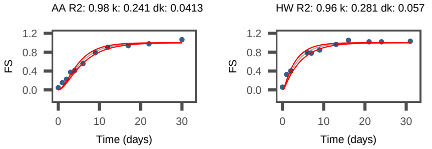

HSP7C − SINPDEAVAYGAAVQAAILSGDK_3

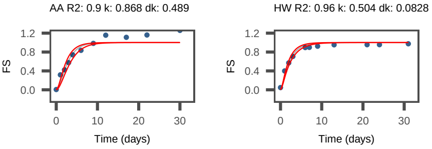

HYES − ITTEEEIEFYIQQFK_2

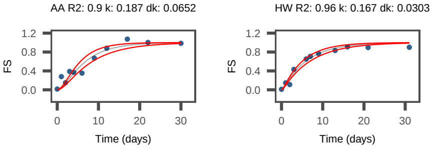

IDHC − SDYLNTFEFMDK_2

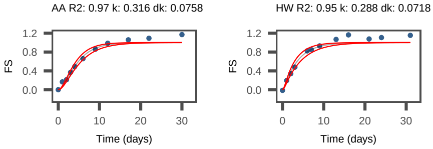

HXK2(Non−Unique) − NILIDFTK_2

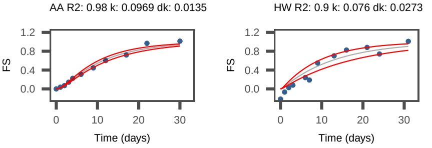

HYES − ITTEEEIEFYIQQFK_3

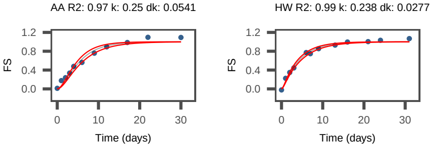

IDHG1 − ENTEGEYSSLEHESVAGVVESLK_3

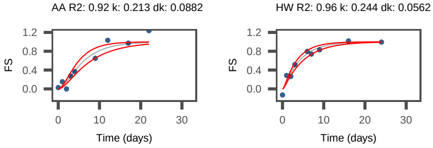

HYEP − IIPLLTDPK_2

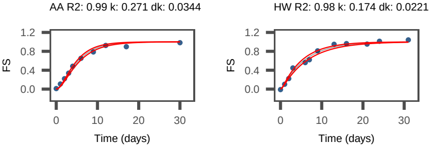

HYKK − ENFIWNLK_2

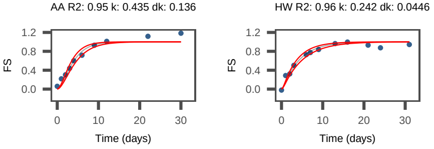

IDHG1 − HTVTMIPGDGIGPELMLHVK_4

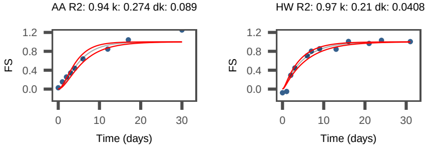

IDHP – DIFQEIFDK_2

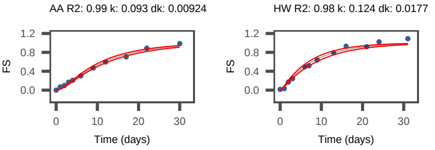

IDHP – VCVQTVESGAMTK_2

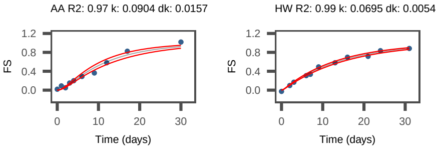

ISC2A – ILPESSILFLCDLQEK_2

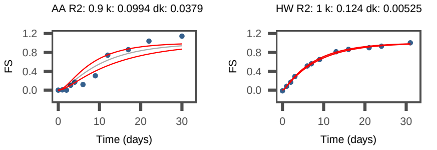

IDHP – FAQTLEK_2

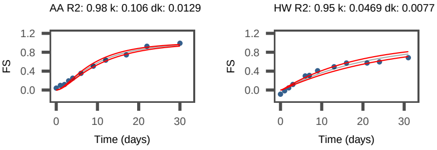

IF5A1(Non−Unique) – VHLVGIDIFTGK_3

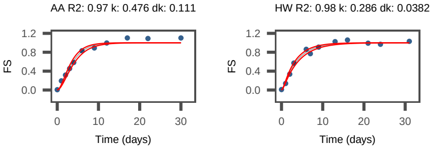

ISC2A – ILPESSILFLCDLQEK_3

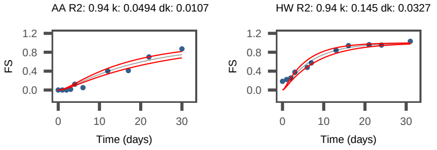

IDHP – LIDDMVAQVLK_2

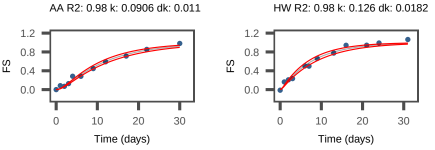

INMT – DYLTTYYSFHSGPVAEQEIVK_2

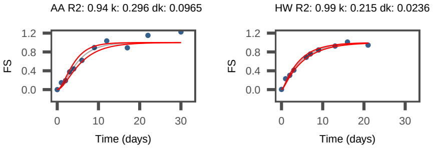

IVD – IGQFQLMQGK_2

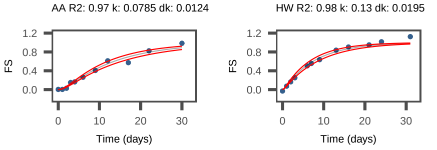

IDHP – LILPHVDVQLK_3

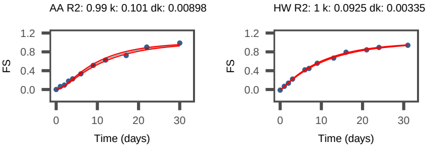

IPYR2 – ILGTLALIDQSETDWK_2

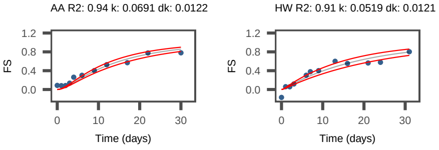

IVD – VPAANVLSQESK_2

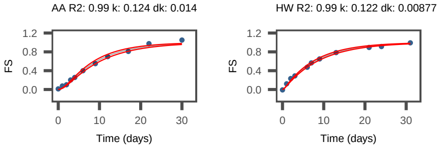

IDHP – LVPGWTKPITIGR_3

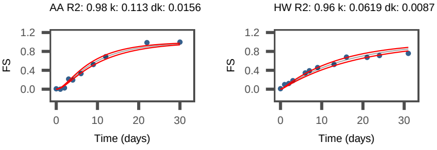

IQGA1 – SWVNQMESQTGEASK_2

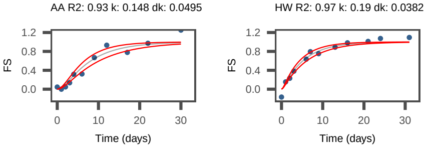

KAD2 – AVLLGPPGAGK_2

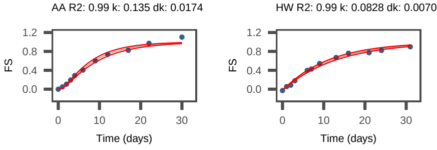

IDHP – SSGGFVWACK_2

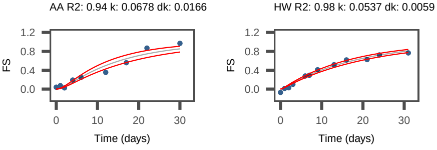

IRGM1 – LLELVYGIK_2

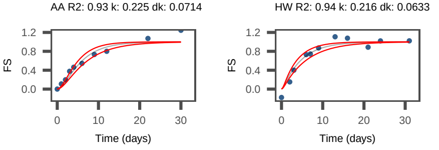

KAD3 – VYQIDTVINLNVPFEVIK_2

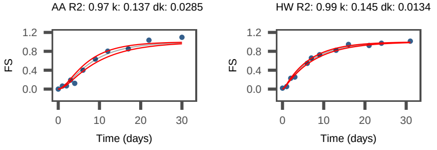

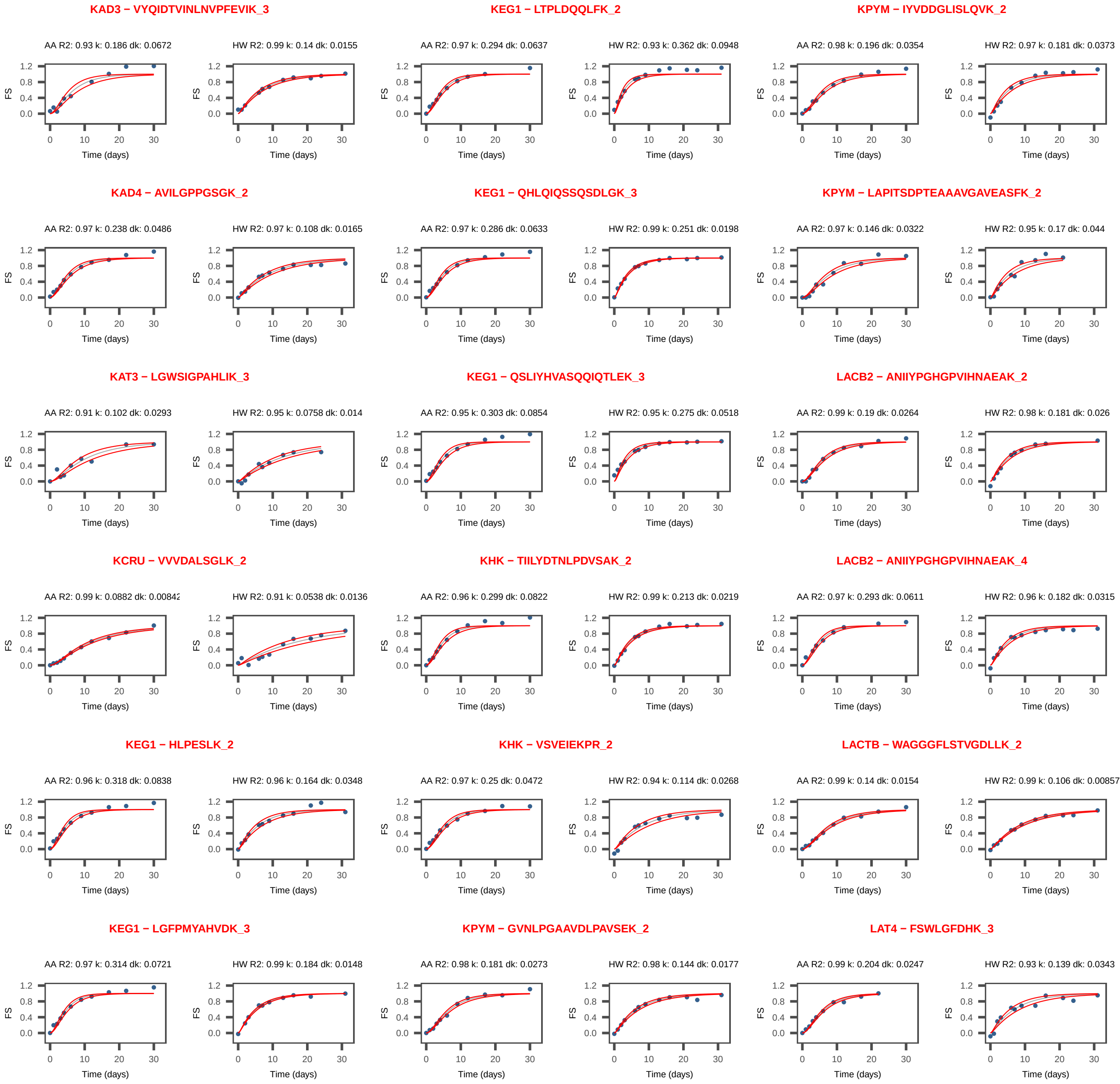
KAD3 – VYQIDTVINLNVPFEVIK_3
AA R2: 0.93 k: 0.186 dk: 0.0672
HW R2: 0.99 k: 0.14 dk: 0.0155
KEG1 – LTPLDQQLFK_2
AA R2: 0.97 k: 0.294 dk: 0.0637
HW R2: 0.93 k: 0.362 dk: 0.0948
KPYM – IYVDDGLISLQVK_2
AA R2: 0.98 k: 0.196 dk: 0.0354
HW R2: 0.97 k: 0.181 dk: 0.0373
KAD4 – AVILGPPGSGK_2
AA R2: 0.97 k: 0.238 dk: 0.0486
HW R2: 0.97 k: 0.108 dk: 0.0165
KEG1 – QHLQIQSSQSDLGK_3
AA R2: 0.97 k: 0.286 dk: 0.0633
HW R2: 0.99 k: 0.251 dk: 0.0198
KPYM – LAPITSDPTEAAAVGAVEASFK_2
AA R2: 0.97 k: 0.146 dk: 0.0322
HW R2: 0.95 k: 0.17 dk: 0.044
KAT3 – LGWSIGPAHLIK_3
AA R2: 0.91 k: 0.102 dk: 0.0293
HW R2: 0.95 k: 0.0758 dk: 0.014
KEG1 – QSLIYHVASQQIQTLEK_3
AA R2: 0.95 k: 0.303 dk: 0.0854
HW R2: 0.95 k: 0.275 dk: 0.0518
LACB2 – ANIIYPGHGPVIHNAEAK_2
AA R2: 0.99 k: 0.19 dk: 0.0264
HW R2: 0.98 k: 0.181 dk: 0.026
KCRU – VVVDALSGLK_2
AA R2: 0.99 k: 0.0882 dk: 0.00842
HW R2: 0.91 k: 0.0538 dk: 0.0136
KHK – TIILYDTNLPDVSAK_2
AA R2: 0.96 k: 0.299 dk: 0.0822
HW R2: 0.99 k: 0.213 dk: 0.0219
LACB2 – ANIIYPGHGPVIHNAEAK_4
AA R2: 0.97 k: 0.293 dk: 0.0611
HW R2: 0.96 k: 0.182 dk: 0.0315
KEG1 – HLPESLK_2
AA R2: 0.96 k: 0.318 dk: 0.0838
HW R2: 0.96 k: 0.164 dk: 0.0348
KHK – VSVEIEKPR_2
AA R2: 0.97 k: 0.25 dk: 0.0472
HW R2: 0.94 k: 0.114 dk: 0.0268
LACTB – WAGGGFLSTVGDLLK_2
AA R2: 0.99 k: 0.14 dk: 0.0154
HW R2: 0.99 k: 0.106 dk: 0.00857
KEG1 – LGFPMYAHVDK_3
AA R2: 0.97 k: 0.314 dk: 0.0721
HW R2: 0.99 k: 0.184 dk: 0.0148
KPYM – GVNLPGAAVDLPAVSEK_2
AA R2: 0.98 k: 0.181 dk: 0.0273
HW R2: 0.98 k: 0.144 dk: 0.0177
LAT4 – FSWLGFDHK_3
AA R2: 0.99 k: 0.204 dk: 0.0247
HW R2: 0.93 k: 0.139 dk: 0.0343

LDHA − DQLIVNLLK_2

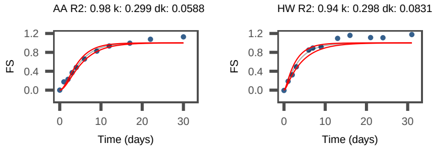

LDHB − GEMMDLQHGSLFLQTPK_2

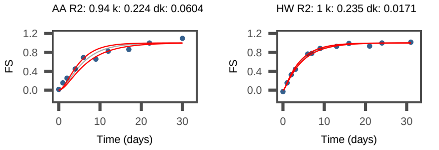

LDHB − MVVDSAYEVIK_2

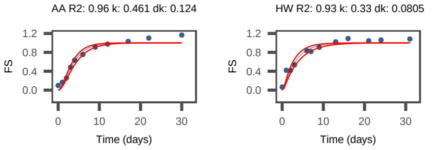

LDHA − GEMMDLQHGSLFLK_3

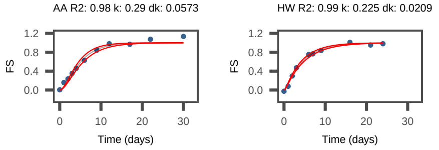

LDHB − GEMMDLQHGSLFLQTPK_3

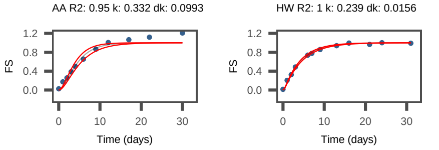

LDHB − SADTLWDIQK_2

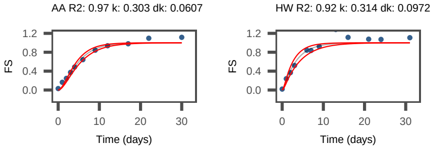

LDHA − LLIVSNPVDILTYVAWK_2

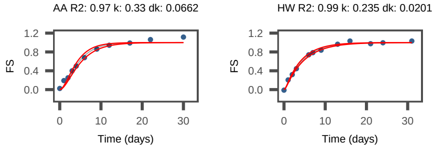

LDHB − GLTSVINQK_2

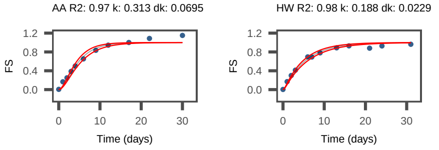

LDHB − SLADELALVDVLEDK_3

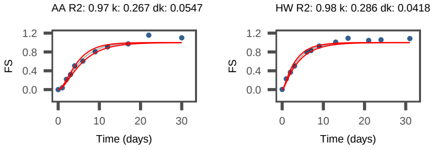

LDHA − LLIVSNPVDILTYVAWK_3

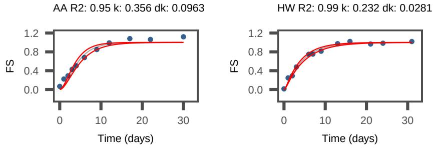

LDHB − ITVVGVGQVGMACAISILGK_2

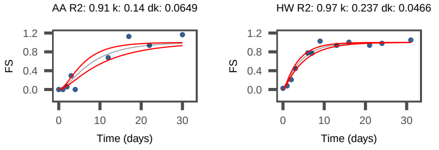

LDHD − GSQGGLSQDFVEALK_2

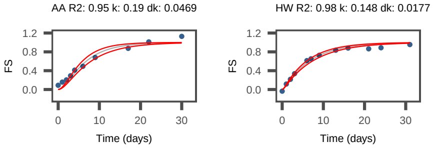

LDHA − QVVDSAYEVIK_2

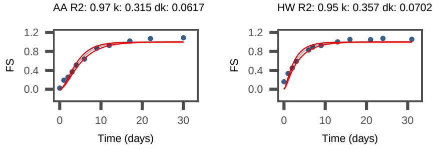

LDHB − LIASVADDEAAVPNNK_2

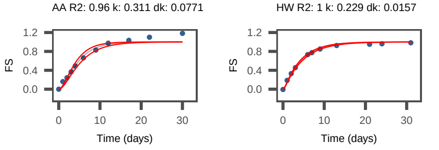

LDHD − LPEILVETK_2

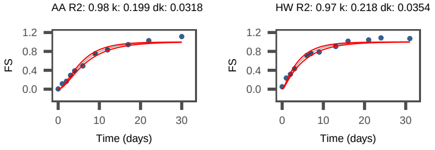

LDHB − FIIPQIVK_2

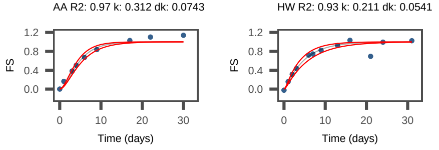

LDHB − LKDDEVAQLR_3

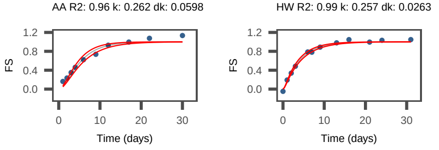

LETM1 − STLQTLPEIVAK_2

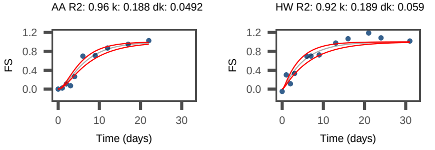

LETM1 – VQQMIGQIDGLITQLETTQQDGK_3

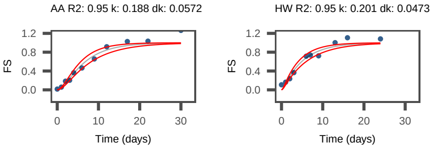

LRP2 – GIALDPTVGYLFFSDWGSLSGQPK_2

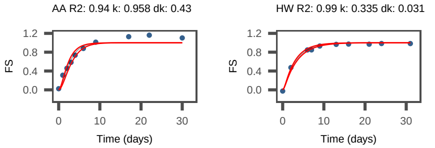

LRP2 – LGWPAGITLDLVSK_2

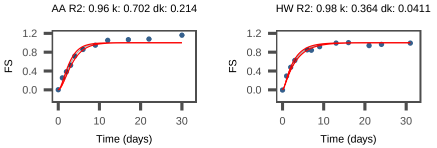

LKHA4 – SHDQAVHTYQEHK_4

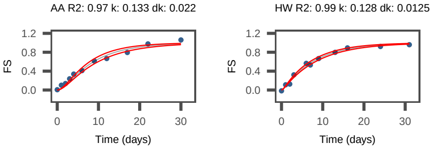

LRP2 – GIALDPTVGYLFFSDWGSLSGQPK_3

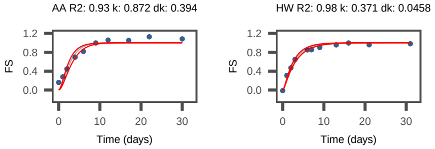

LRP2 – LPTQPSGISTVVK_2

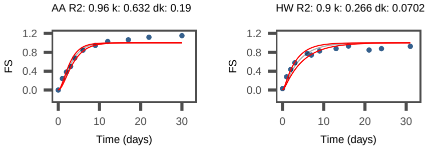

LPPRC – LDASAILDTAK_2

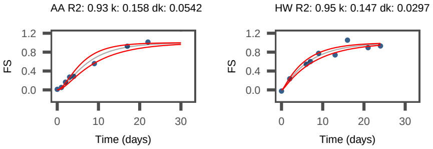

LRP2 – GISLFEEHVFFTDWTK_3

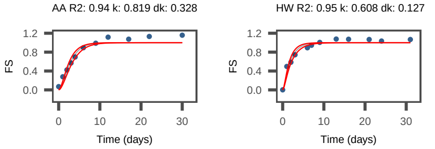

LRP2 – SMAMNEQFVMEVGK_2

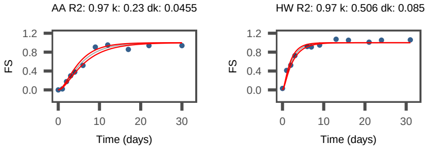

LPPRC – LEDTAFQVLLALPLSK_2

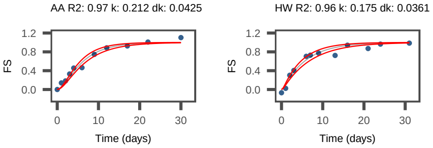

LRP2 – GIVMGVDFHYQK_2

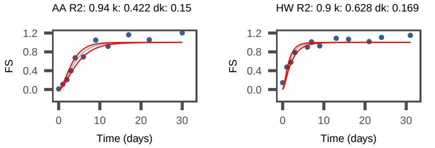

LRP2 – WLITTQLDQPAAIAVNPK_2

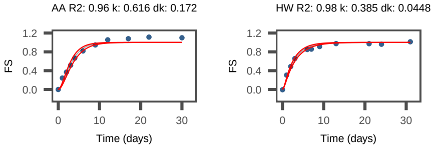

LRC59 – LVTLPVSFAQLK_2

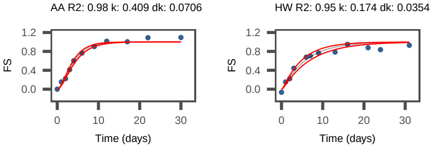

LRP2 – GYMYWTDWGTNAK_2

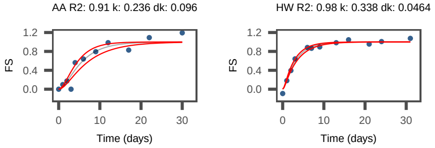

M2OM – LTGADGTPPGFLLK_2

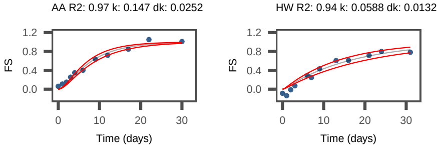

LRP2 – ENVIFGISLDPEVK_2

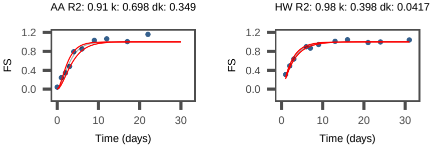

LRP2 – LGHVDQMTHPFGLTVFK_4

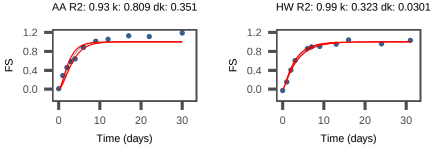

M2OM – TSFHALTSILK_3

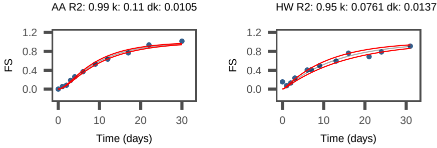

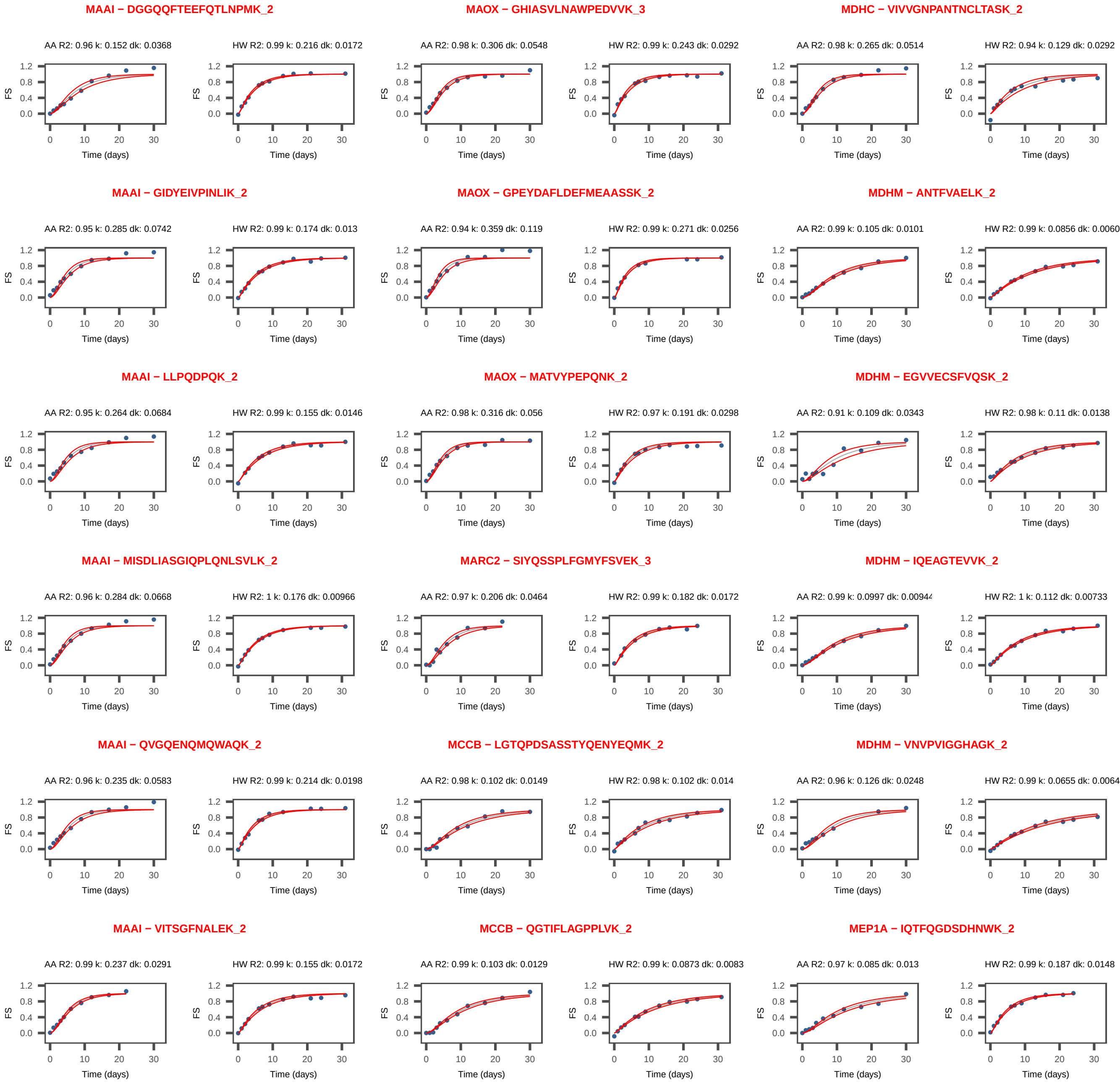
MAAI – DGGQQFTEEFQTLNPMK_2
AA R2: 0.96 k: 0.152 dk: 0.0368
HW R2: 0.99 k: 0.216 dk: 0.0172
MAOX – GHIASVLNAWPEDVVK_3
AA R2: 0.98 k: 0.306 dk: 0.0548
HW R2: 0.99 k: 0.243 dk: 0.0292
MDHC – VIVVGNPANTNCLTASK_2
AA R2: 0.98 k: 0.265 dk: 0.0514
HW R2: 0.94 k: 0.129 dk: 0.0292
MAAI – GIDYEIVPINLIK_2
AA R2: 0.95 k: 0.285 dk: 0.0742
HW R2: 0.99 k: 0.174 dk: 0.013
MAOX – GPEYDAFLDEFMEAASSK_2
AA R2: 0.94 k: 0.359 dk: 0.119
HW R2: 0.99 k: 0.271 dk: 0.0256
MDHM – ANTFVAELK_2
AA R2: 0.99 k: 0.105 dk: 0.0101
HW R2: 0.99 k: 0.0856 dk: 0.0060
MAAI – LLPQDPQK_2
AA R2: 0.95 k: 0.264 dk: 0.0684
HW R2: 0.99 k: 0.155 dk: 0.0146
MAOX – MATVYPEPQNK_2
AA R2: 0.98 k: 0.316 dk: 0.056
HW R2: 0.97 k: 0.191 dk: 0.0298
MDHM – EGVVECSFVQSK_2
AA R2: 0.91 k: 0.109 dk: 0.0343
HW R2: 0.98 k: 0.11 dk: 0.0138
MAAI – MISDLIASGIQPLQNLSVLK_2
AA R2: 0.96 k: 0.284 dk: 0.0668
HW R2: 1 k: 0.176 dk: 0.00966
MARC2 – SIYQSSPLFGMYFSVEK_3
AA R2: 0.97 k: 0.206 dk: 0.0464
HW R2: 0.99 k: 0.182 dk: 0.0172
MDHM – IQEAGTEVVK_2
AA R2: 0.99 k: 0.0997 dk: 0.00944
HW R2: 1 k: 0.112 dk: 0.00733
MAAI – QVGQENQMQWAQK_2
AA R2: 0.96 k: 0.235 dk: 0.0583
HW R2: 0.99 k: 0.214 dk: 0.0198
MCCB – LGTQPDSASSTYQENYEQMK_2
AA R2: 0.98 k: 0.102 dk: 0.0149
HW R2: 0.98 k: 0.102 dk: 0.014
MDHM – VNVPVIGGHAGK_2
AA R2: 0.96 k: 0.126 dk: 0.0248
HW R2: 0.99 k: 0.0655 dk: 0.0064
MAAI – VITSGFNALEK_2
AA R2: 0.99 k: 0.237 dk: 0.0291
HW R2: 0.99 k: 0.155 dk: 0.0172
MCCB – QGTIFLAGPPLVK_2
AA R2: 0.99 k: 0.103 dk: 0.0129
HW R2: 0.99 k: 0.0873 dk: 0.0083
MEP1A – IQTFQGDSDHNWK_2
AA R2: 0.97 k: 0.085 dk: 0.013
HW R2: 0.99 k: 0.187 dk: 0.0148
FS
Time (days)

MEP1B − MDFSDYDLLK_2

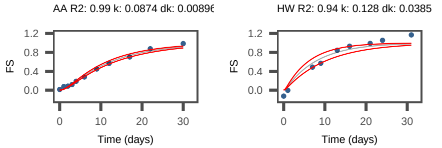

MIC26 − IDELSLYSVPEGQSK_2

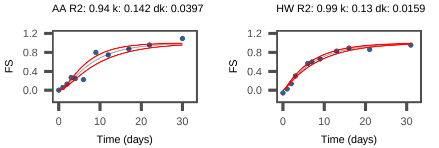

MLEC − YNEETFGYEVPVK_2

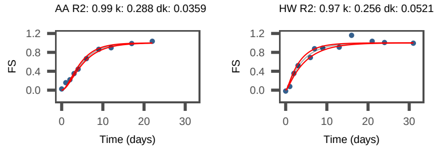

MEP1B − NSIIGDHK_2

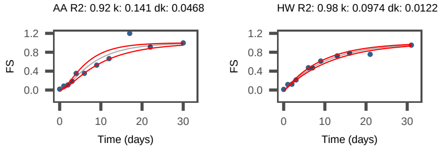

MIC60 − TAMDNSEIAGEK_2

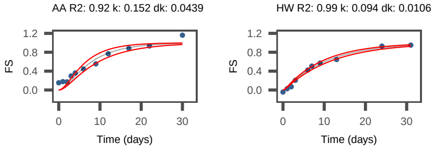

MMSA − EEIFGPVLVVLETETLDEAIK_2

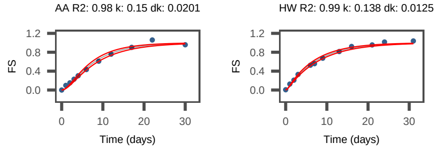

MEP1B − VSQVLSGPESDHSK_2

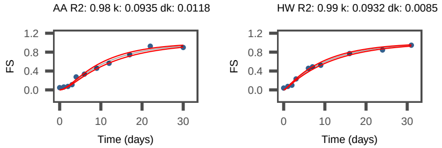

MIC60 − TSSVTLQTITAQNAAVQAVK_2

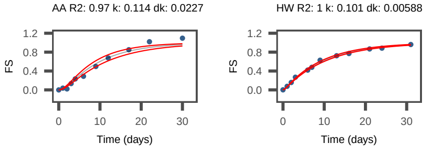

MMSA − EEIFGPVLVVLETETLDEAIK_3

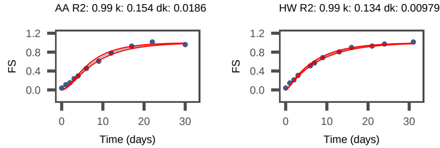

MIC13 − DSWNSGIISVMSALSVAPSK_2

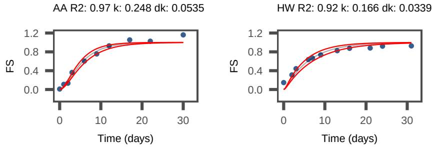

MIF − ASVPEGFLSELTQQLAQATGK_2

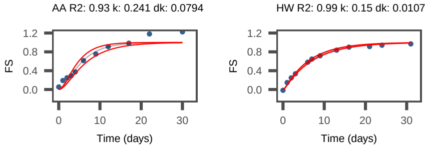

MMSA − LITLEQGK_2

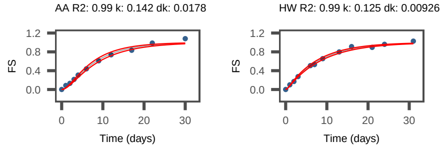

MIC13 − DSWNSGIISVMSALSVAPSK_3

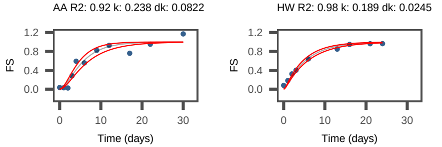

MIF − ASVPEGFLSELTQQLAQATGK_3

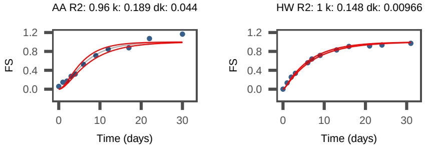

MMSA − MALSTAILVGEAK_2

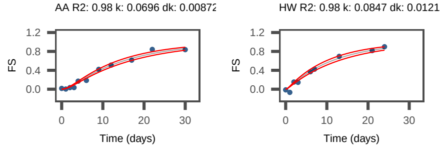

MIC19 − YSSVYGASVSDEDLK_2

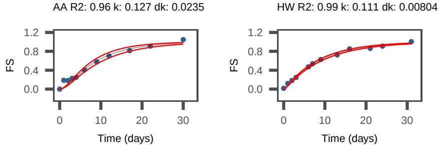

MLEC − LYIEFVK_2

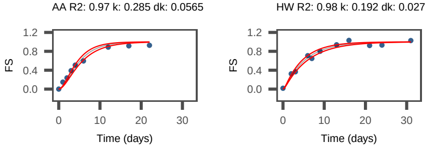

MMSA − NHGVVMPDANK_2

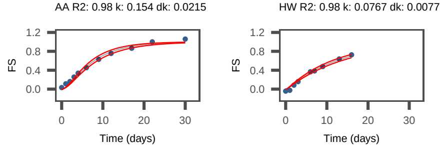

MMSA − VNAGDQPGADLGPLITPQAK_2

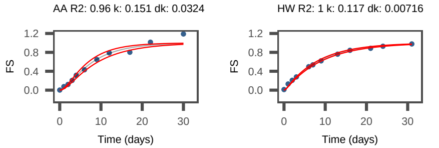

MPCP − EEGLNAFYK_2

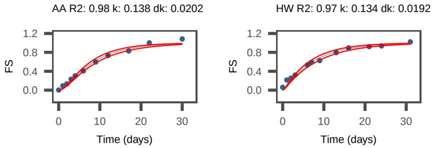

MYH9 − FVSELWK_2

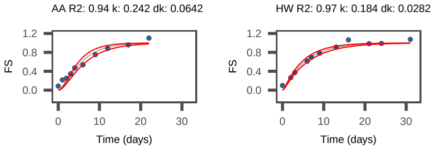

MMSA − VNAGDQPGADLGPLITPQAK_3

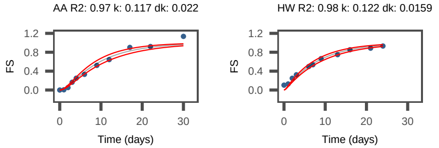

MPCP − TAVVPLDLVK_2

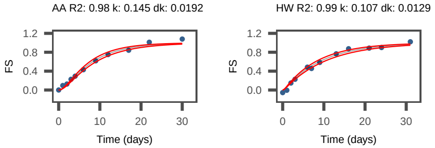

MYH9 − IIGLDQVAGMSETALPGAFK_2

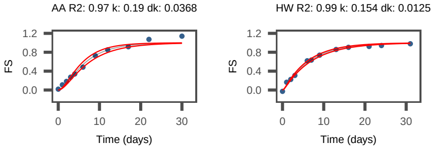

MMSA − VPGATMLLAK_2

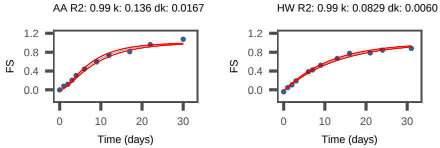

MPU1 − GLLVPILLPEK_2

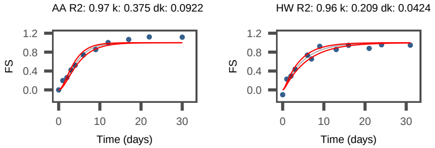

MYH9 − LTEMETMQSQLMAEK_2

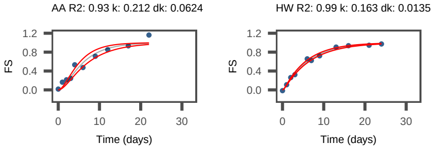

MOES − ESEAVEWQQK_2

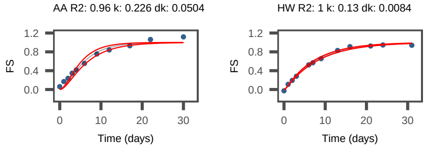

MSRA − TEPIPVTAK_2

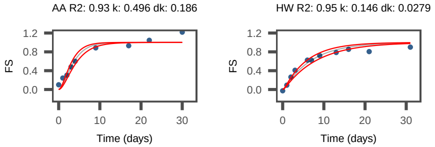

MYH9 − QIATLHAQVTDMK_3

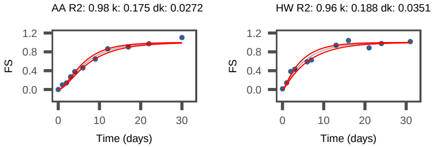

MOES − EVWFFGLQYQDTK_2

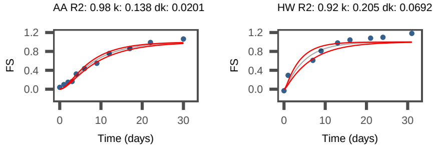

MTX2 − VPFIHVGNQVVSELGPIVQFVK_3

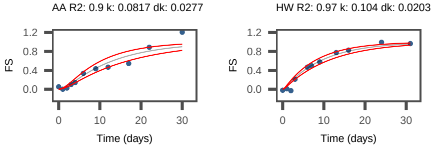

MYH9 − TEMEDLMSSK_2

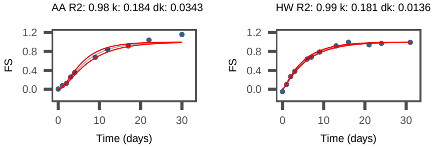

MPCP − AVEEYSCEFGSMK_2

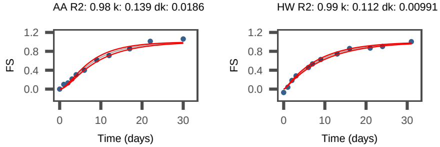

MYH9 − ALEQQVEEMK_2

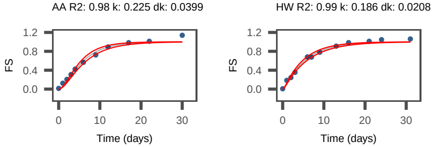

MYH9 − VEAQLQELQVK_2

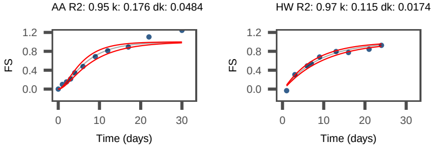

MYH9 – VISGVLQLGNIAFK_2

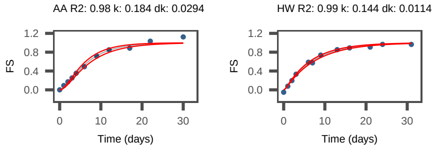

NDKA – DRPFFTGLVK_3

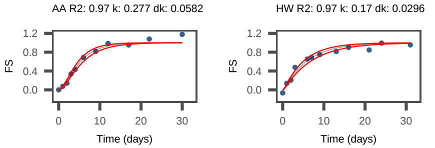

NDUAA – VTSAYLQDIENAYK_2

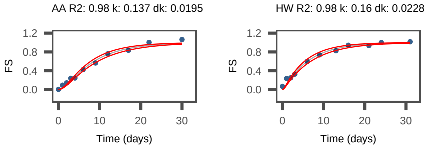

MYL6 – VLDFEHFLPMLQTVAK_3

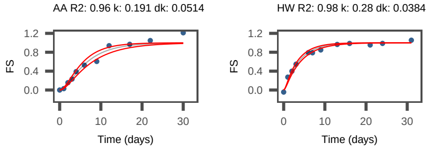

NDKA – FLQASEDLLK_2

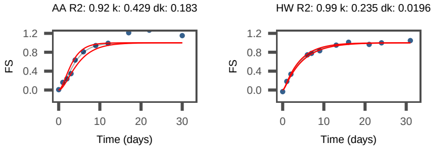

NDUAA – YGLLAAILGDK_2

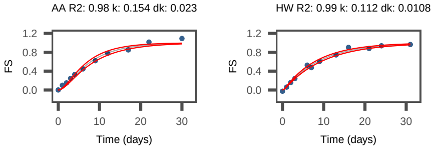

MYO1C – QLLLTPSAVVIVEDAK_2

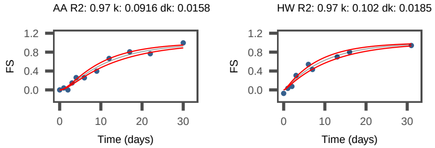

NDKB – DRPFFPGLVK_3

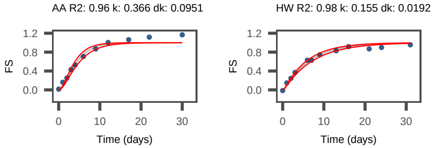

NDUAD – IALMPLFQAEK_2

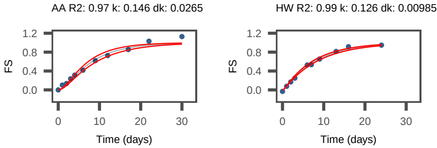

NB5R3 – STPAITLENPDIK_2

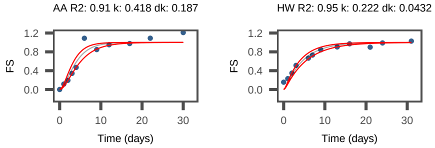

NDRG1 – ISGWTQALPDMVVSHLFGK_3

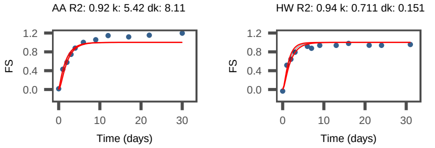

NDUB3 – IEGTPLETVQK_2

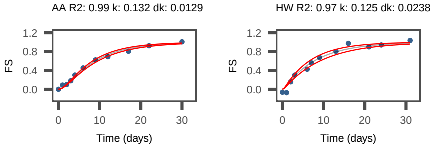

NCEH1 – LDWTSLLPSSIK_2

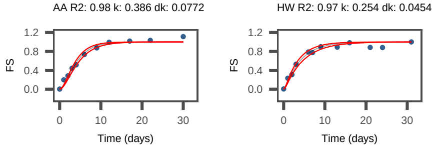

NDUA2 – YAFGQEK_2

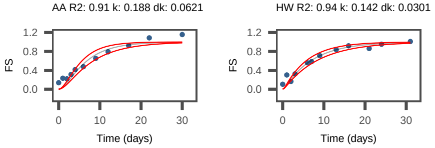

NDUB5 – NFYDGPEK_2

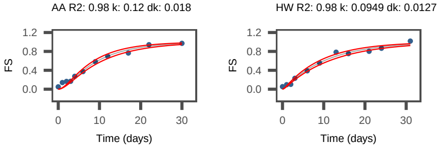

NCPR – TYEHFNAMGK_3

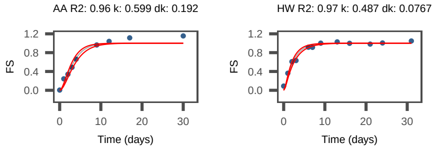

NDUA4 – FYSVNVDYSK_2

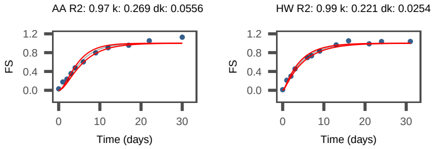

NDUB9 – AMYPDYFSK_2

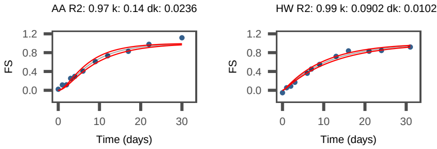

NDUC2 − DHDMFGYIK_2

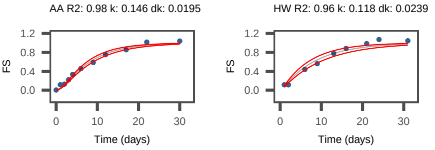

NDUS1 − YDHLGDSPK_2

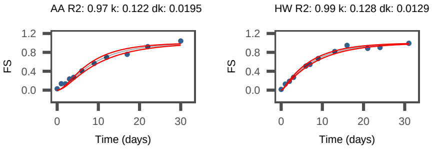

NDUV1 − EAYEAGLIGK_2

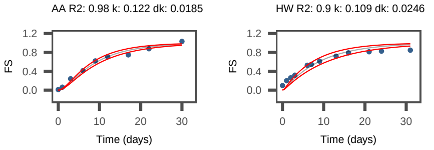

NDUC2 − DHDMFGYIK_3

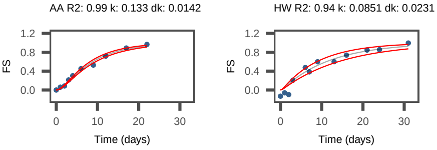

NDUS2 − APGFAHLAGLDK_3

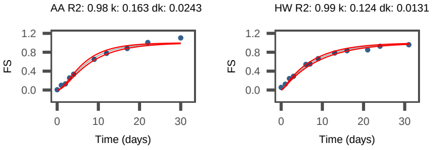

NDUV1 − EGVDWMNK_2

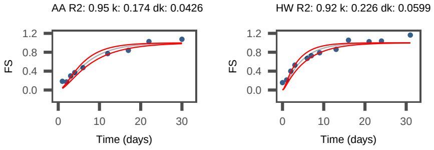

NDUS1 − IASQVAALDLGYKPGVEAIR_3

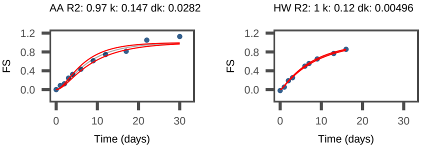

NDUS2 − PGGVHQDLPLGLLDDIYEFSK_3

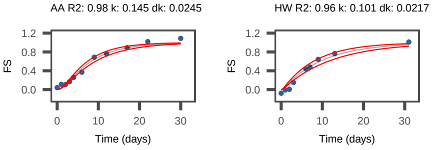

NDUV1 − GPDWILGEMK_2

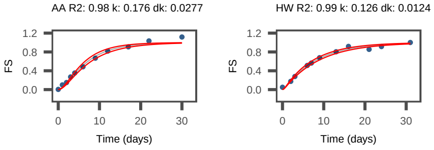

NDUS1 − KPMVVLGSSALQR_3

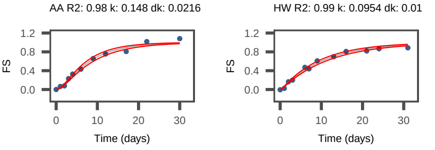

NDUS2 − QWQPDIEWAEQFSGAVMYPSK_2

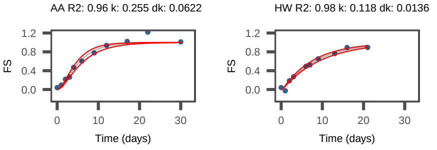

NDUV1 − HAGGVTGGWDNLLAVIPGGSSTPLIPK_3

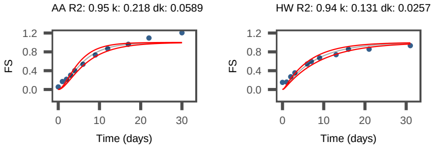

NDUS1 − LVNQEVLADPLVPPQLTIK_2

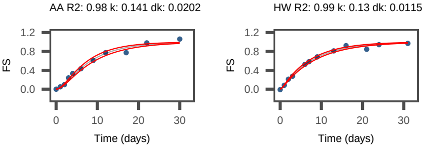

NDUS4 − LDITTLTGVPEEHIK_3

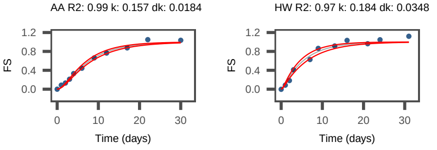

NDUV2 − DIEEIIDELK_2

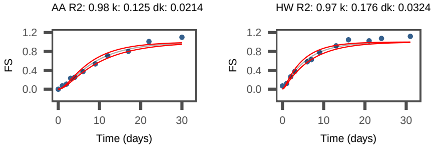

NDUS1 − LVNQEVLADPLVPPQLTIK_3

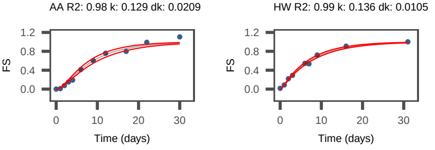

NDUS4 − SYGANFSWNK_2

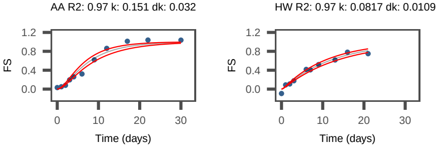

NEP − EVFIQTLDDLTWMDAETK_2

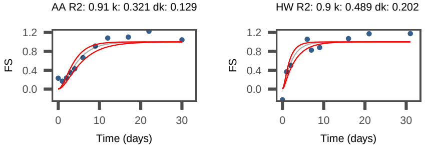

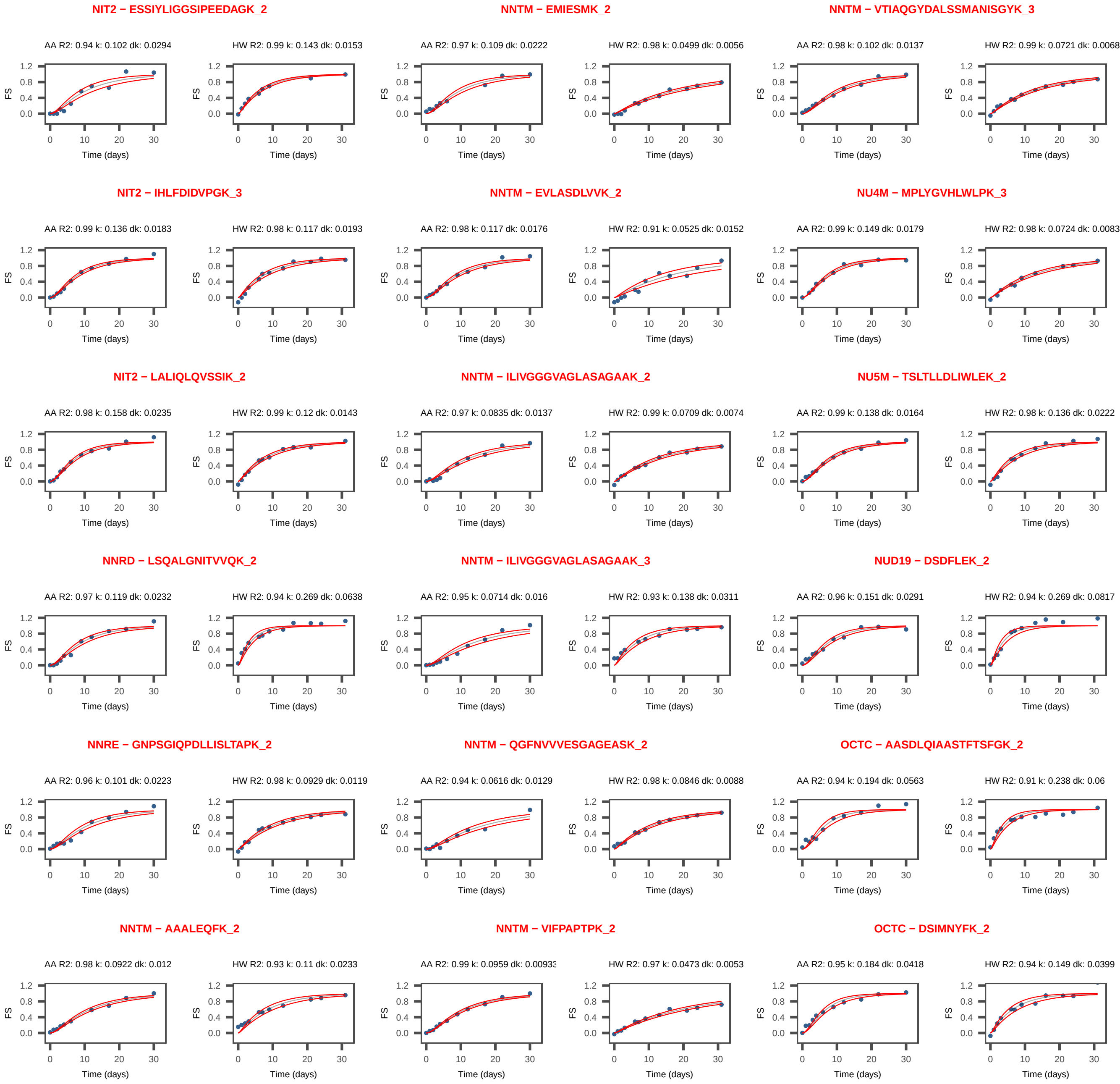
NIT2 – ESSIYLIGGSIPEEDAGK_2
AA R2: 0.94 k: 0.102 dk: 0.0294
HW R2: 0.99 k: 0.143 dk: 0.0153
NNTM – EMIESMK_2
AA R2: 0.97 k: 0.109 dk: 0.0222
HW R2: 0.98 k: 0.0499 dk: 0.0056
NNTM – VTIAQGYDALSSMANISGYK_3
AA R2: 0.98 k: 0.102 dk: 0.0137
HW R2: 0.99 k: 0.0721 dk: 0.0068
NIT2 – IHLFDIDVPGK_3
AA R2: 0.99 k: 0.136 dk: 0.0183
HW R2: 0.98 k: 0.117 dk: 0.0193
NNTM – EVLASDLVVK_2
AA R2: 0.98 k: 0.117 dk: 0.0176
HW R2: 0.91 k: 0.0525 dk: 0.0152
NU4M – MPLYGVHLWLPK_3
AA R2: 0.99 k: 0.149 dk: 0.0179
HW R2: 0.98 k: 0.0724 dk: 0.0083
NIT2 – LALIQLQVSSIK_2
AA R2: 0.98 k: 0.158 dk: 0.0235
HW R2: 0.99 k: 0.12 dk: 0.0143
NNTM – ILIVGGGVAGLASAGAAK_2
AA R2: 0.97 k: 0.0835 dk: 0.0137
HW R2: 0.99 k: 0.0709 dk: 0.0074
NU5M – TSLTLLLDLIWLEK_2
AA R2: 0.99 k: 0.138 dk: 0.0164
HW R2: 0.98 k: 0.136 dk: 0.0222
NNRD – LSQALGNITVVQK_2
AA R2: 0.97 k: 0.119 dk: 0.0232
HW R2: 0.94 k: 0.269 dk: 0.0638
NNTM – ILIVGGGVAGLASAGAAK_3
AA R2: 0.95 k: 0.0714 dk: 0.016
HW R2: 0.93 k: 0.138 dk: 0.0311
NUD19 – DSDFLEK_2
AA R2: 0.96 k: 0.151 dk: 0.0291
HW R2: 0.94 k: 0.269 dk: 0.0817
NNRE – GNPSGIQPDLLISLTAPK_2
AA R2: 0.96 k: 0.101 dk: 0.0223
HW R2: 0.98 k: 0.0929 dk: 0.0119
NNTM – QGFNVVVESGAGEASK_2
AA R2: 0.94 k: 0.0616 dk: 0.0129
HW R2: 0.98 k: 0.0846 dk: 0.0088
OCTC – AASDLQIAASTFTSFGK_2
AA R2: 0.94 k: 0.194 dk: 0.0563
HW R2: 0.91 k: 0.238 dk: 0.06
NNTM – AAALEQFK_2
AA R2: 0.98 k: 0.0922 dk: 0.012
HW R2: 0.93 k: 0.11 dk: 0.0233
NNTM – VIFPAPTPK_2
AA R2: 0.99 k: 0.0959 dk: 0.0093
HW R2: 0.97 k: 0.0473 dk: 0.0053
OCTC – DSIMNYFK_2
AA R2: 0.95 k: 0.184 dk: 0.0418
HW R2: 0.94 k: 0.149 dk: 0.0399
FS
Time (days)

ODB2 − PVILPPEVAIGALGAIK_3

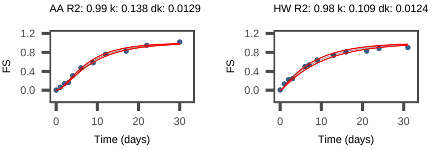

ODO1 − IEQLSPFPFDLLLK_2

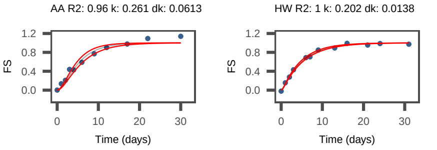

ODO1 − VIPENGPAAQDPHK_2

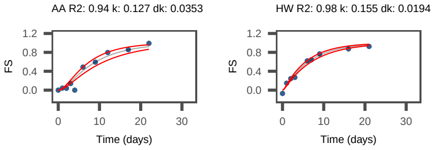

ODB2 − SEITPPPPQPK_2

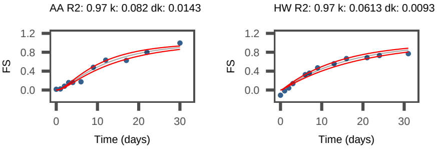

ODO1 − KTHLTELQR_2

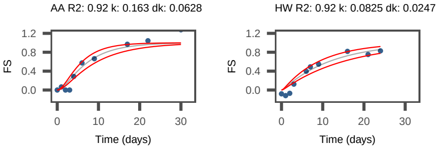

ODO1 − VIPENGPAAQDPHK_3

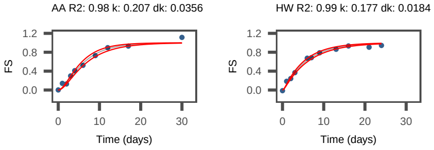

ODBA − LKPNPSLLFSDVYQEMPAQLR_3

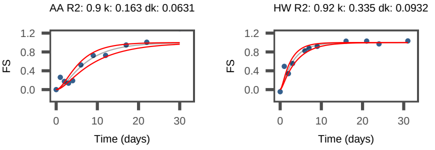

ODO1 − LGFYGLHESDLDK_3

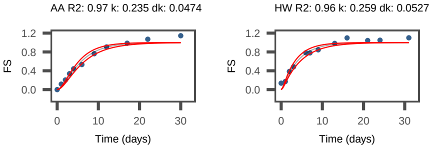

ODPA − MVNSNLASVEELK_2

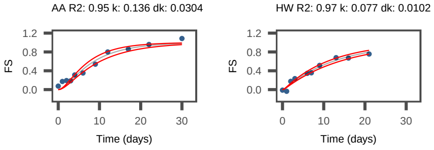

ODBB − MNLFQSITSALDNSLAK_3

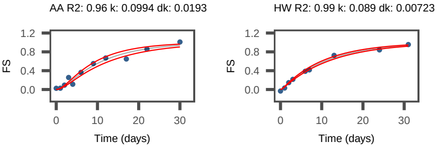

ODO1 − NGHNEMDEPMFTQPLMYK_3

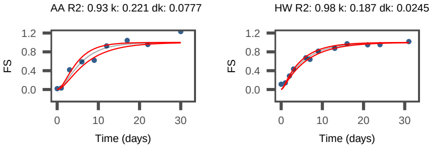

ORNT1 − IAASQNTVWSVVK_2

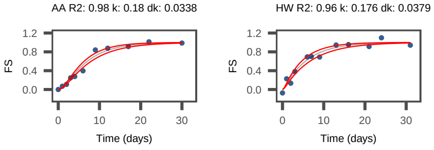

ODC − IQGPQPVPGEIK_2

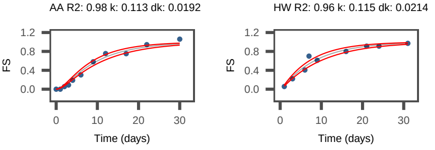

ODO1 − NITLSLVANPSHLEAADPVVMGK_3

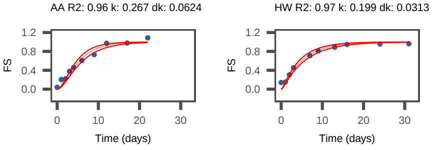

OXDA − HFILTHDPSLGIYNSPYIIPGSK_4

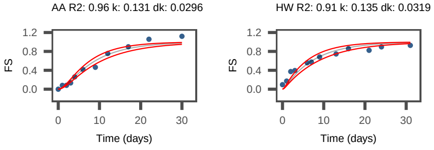

ODO1 − FGLEGCEVLIPALK_2

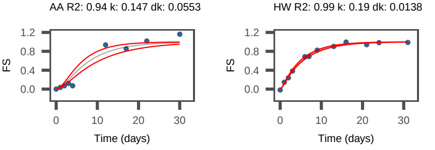

ODO1 − TVDWALAEYMAFGSLLK_3

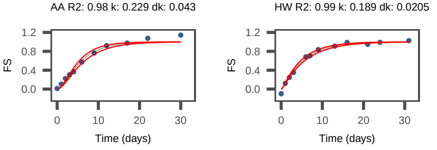

PA2G4 − TIIQNPTDQQK_2

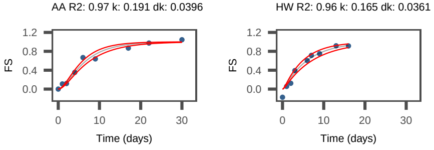

PARK7 − TQGPYDVVVLPGGNLGAQNLSESPMVK_3

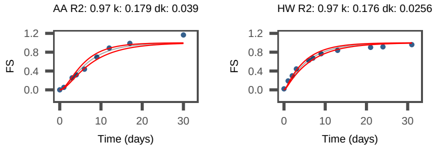

PCKGC − EIISFGSGYGGNSLLGK_2

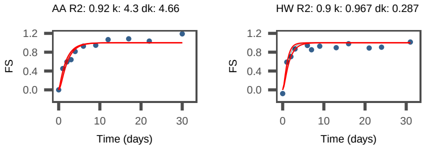

PEBP1 − VDYAGVTVDELGK_2

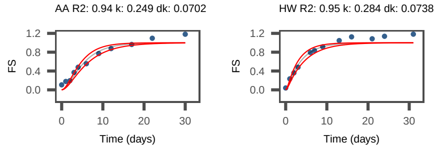

PCCA − AQAVHPGYGFLSENK_3

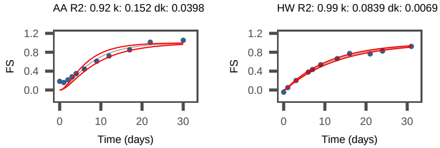

PDIA1 − DHENIIIAK_2

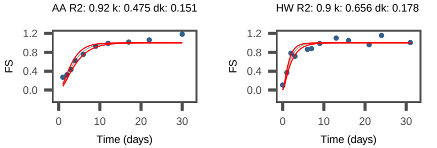

PECR − EGVYNLTK_2

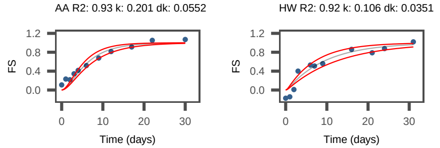

PCCA − VDSGIQPGSDISIYYDPMISK_2

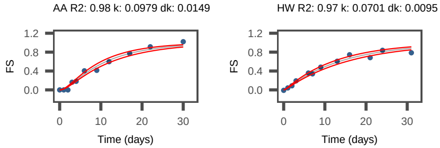

PDIA1 − ILEFFGLK_2

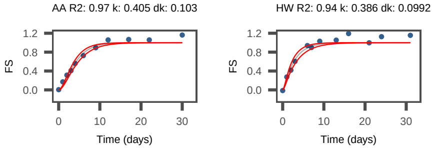

PECR − SIPDHDNWPVGAGDLSIVK_3

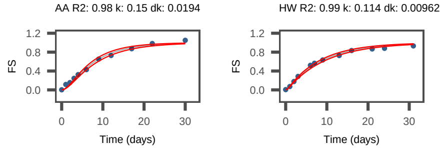

PCCB − AYGGAYDVMSSK_2

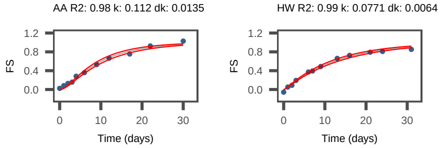

PDIA1 − THILLFLPK_3

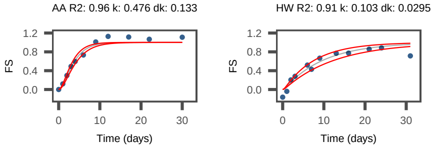

PGAM1 − FSGWYDADLSPAGHEEAK_3

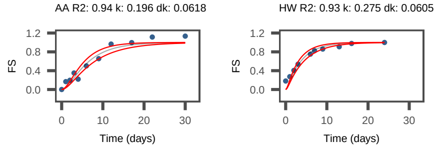

PCCB − DTSYLFITGPEVVK_2

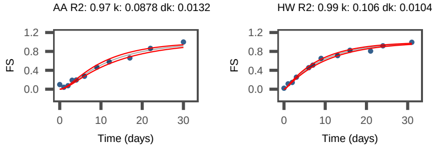

PDIA3 − DLLTAYYDVDYEK_2

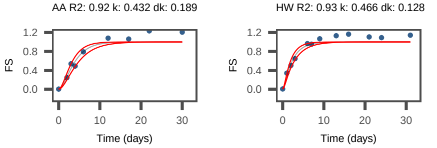

PGAM1 − YADLTEDQLPSCESLK_2

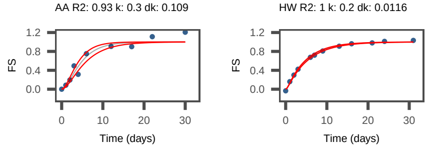

PCCB − SVTNEDVTQEQLGGAK_2

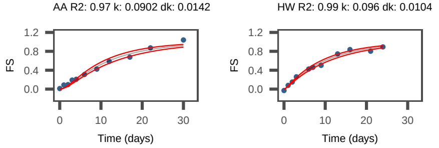

PDIA3 − SEPIPESNEGPVK_2

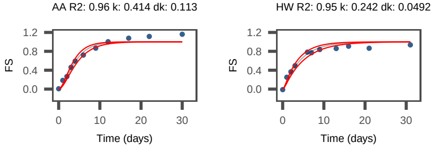

PGAM2 − SFDTPPPPMDEK_2

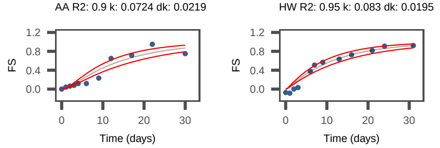

PGBM − IPGDQIVSVVFIK_2

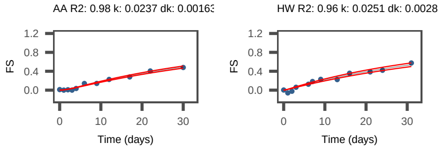

PHB − VLPSITTEILK_2

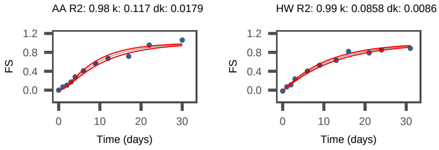

PLSI(Non−Unique) − QFVTPADVVSGNPK_2

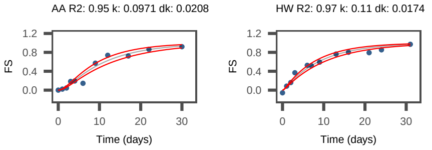

PGK1 − AGGFLMK_2

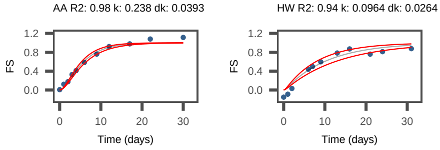

PHB2 − IVQAEGEAEAAK_2

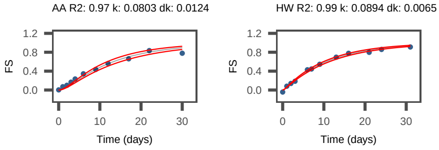

PLSI − VTDDIIIK_2

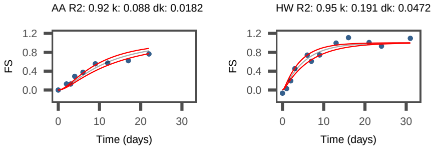

PGK1 − AHSSMVGVNLPQK_3

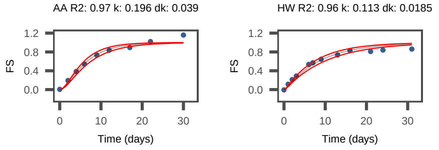

PHB2 − VLPSIVNEVLK_2

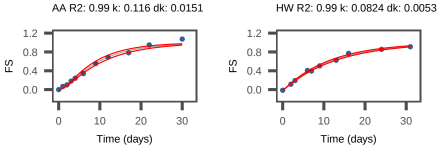

PLST − ISFNEFVYIFQEVK_2

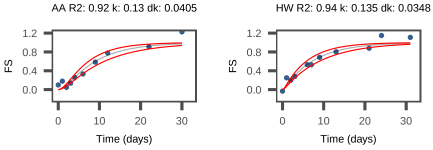

PGK1 − ALESPERPFLAILGGAK_3

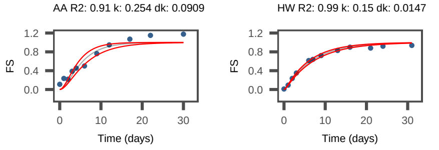

PICAL − ITAAQHSVTGSAVSK_2

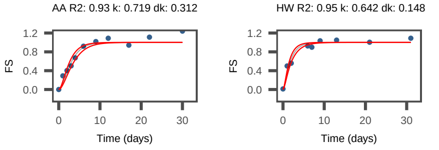

PP1A(Non−Unique) − EIFLSQPILLELEAPLK_2

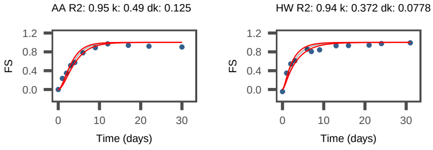

PGK1 − SVVLMSHLGRPDGVPMPDK_3

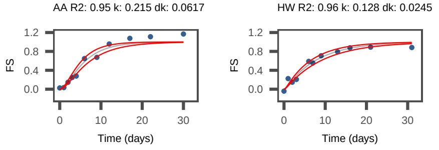

PICAL − ITAAQHSVTGSAVSK_3

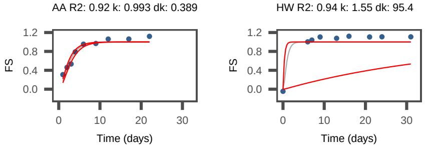

PPIA(Non−Unique) − FEDENFILK_2

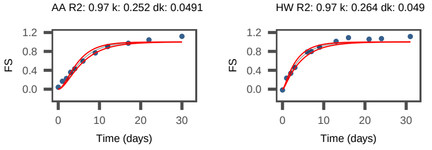

PHB − AATFGLILDDVSLTHLTFGK_3

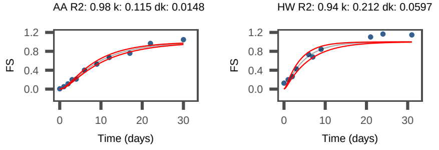

PLSI − NEALIALLK_2

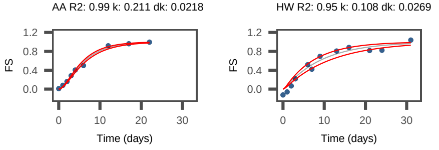

PPIA(Non−Unique) − TEWLDGK_2

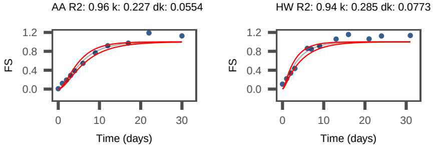

PPIA(Non−Unique) − VSFELFADK_2

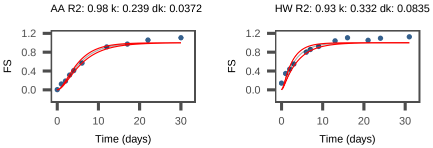

PRDX1(Non−Unique) − LVQAFQFTDK_2

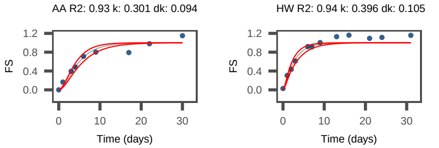

PRDX5 − THLPGFVEQAGALK_3

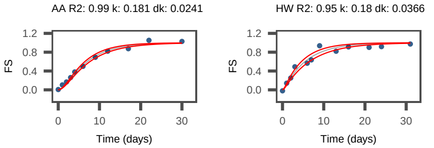

PPID − IVLELFADIVPK_2

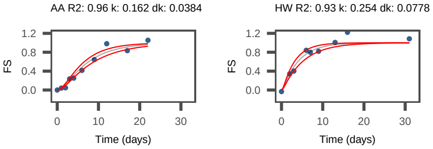

PRDX1 − QGGLGPMNIPLISDPK_2

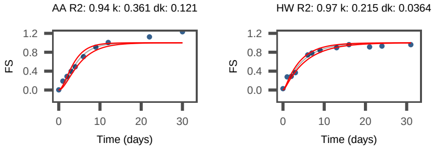

PRDX5 − VGDAIPSVEVFEGEPGK_2

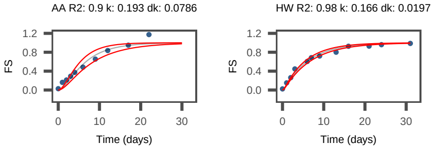

PRD16 − VADPVGVLK_2

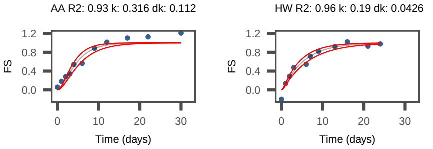

PRDX1(Non−Unique) − TIAQDYGVLK_2

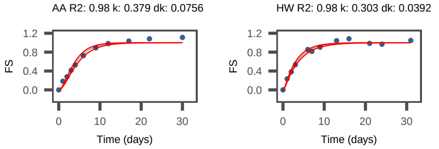

PRDX5 − VNLAELFK_2

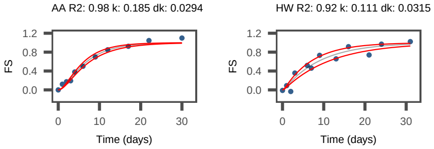

PRDX1(Non−Unique) − ATAVMPDGQFK_2

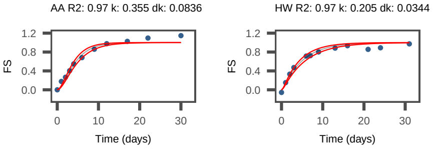

PRDX2 − EGGLGPLNIPLLADVTK_2

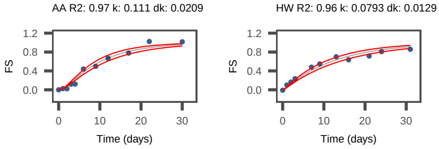

PRDX6 − DLAILLGMLDPVEK_2

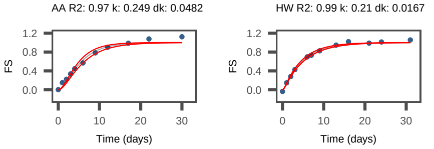

PRDX1 − DISLSEYK_2

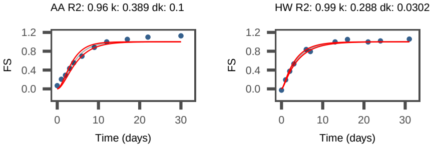

PRDX5 − FSMVIDNGIVK_2

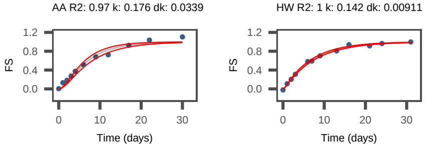

PRDX6 − VVFIFGPDK_2

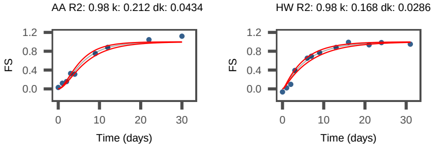

PRDX1 − IGYPAPNFK_2

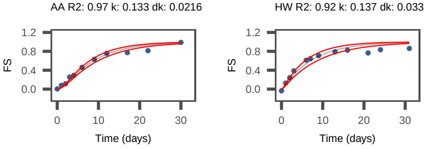

PRDX5 − THLPGFVEQAGALK_2

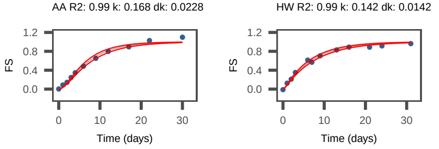

PROF1 − TFVSITPAEVGVLVGK_2

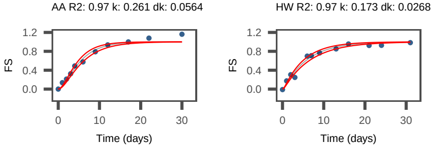

PSA2 – LVQIEYALAAVAGGAPSVGIK_2

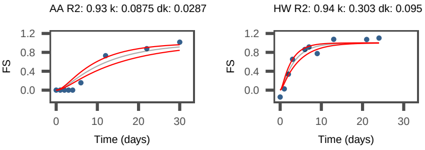

PSB3 – DAVSGMGVIVHVIEK_3

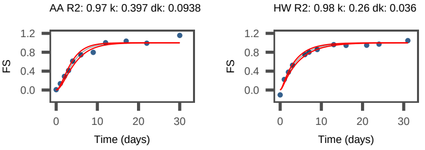

PSB4 – VNDSTMLGASGDYADFQYLK_2

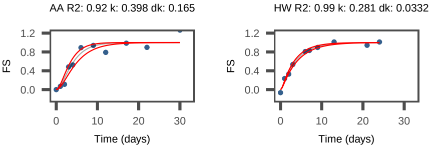

PTER – ILQEAGADISK_2

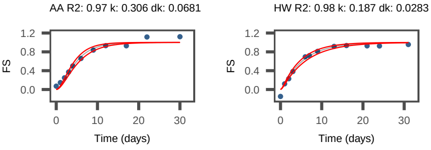

PUR8 – VLSQQAAAVVK_2

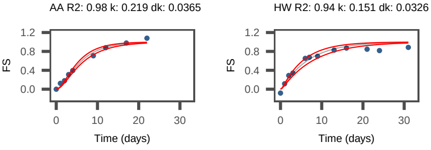

PXL2A – VNPLSVLEAVK_2

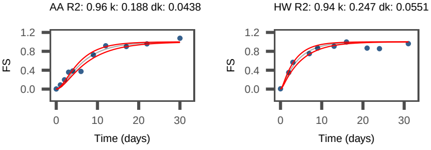

PXMP2 – ALAQYLLLLK_2

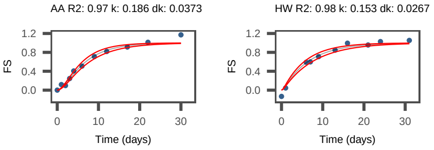

PYC – AYVEANQMLGDLIK_2

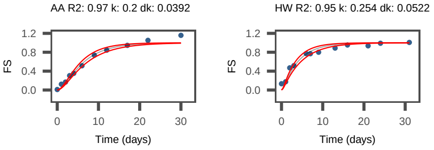

PYC – GLAPVQAYLHIPDIIK_2

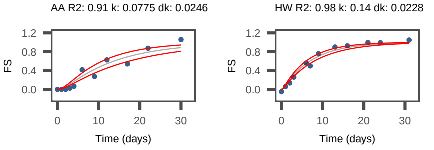

PYC – GLAPVQAYLHIPDIIK_3

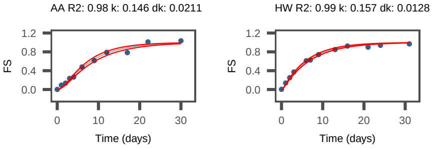

PYC – HGEEVTPEDVLSAAMYPDVFAQFK_3

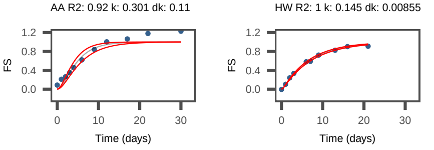

PYC – LDNASAFQGAVISPHYDSLLVK_2

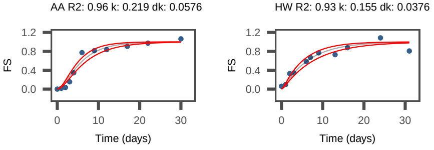

PYC – PGASLPPLNLK_2

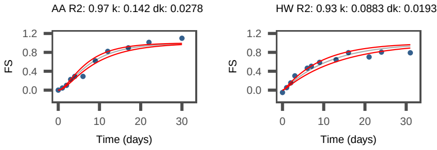

QCR2 – IIENLHDVAYK_2

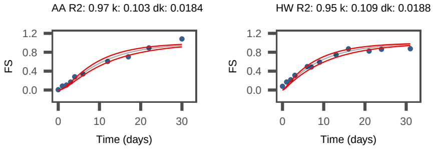

QCR2 – LASSLTTK_2

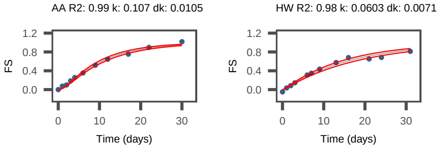

QCR2 – TSAAPGGVPLQPQDLEFTK_2

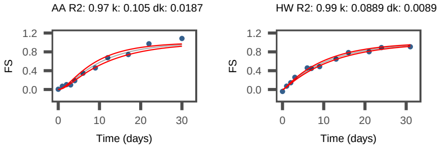

QCR7 – WYYNAAGFNK_2

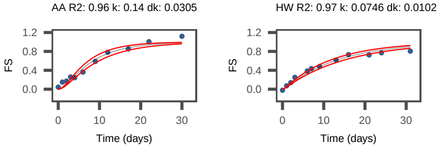

QCR8 – NPAMYENDK_2

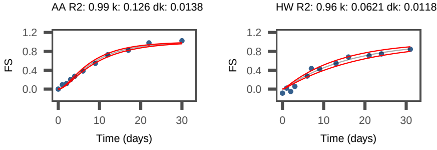

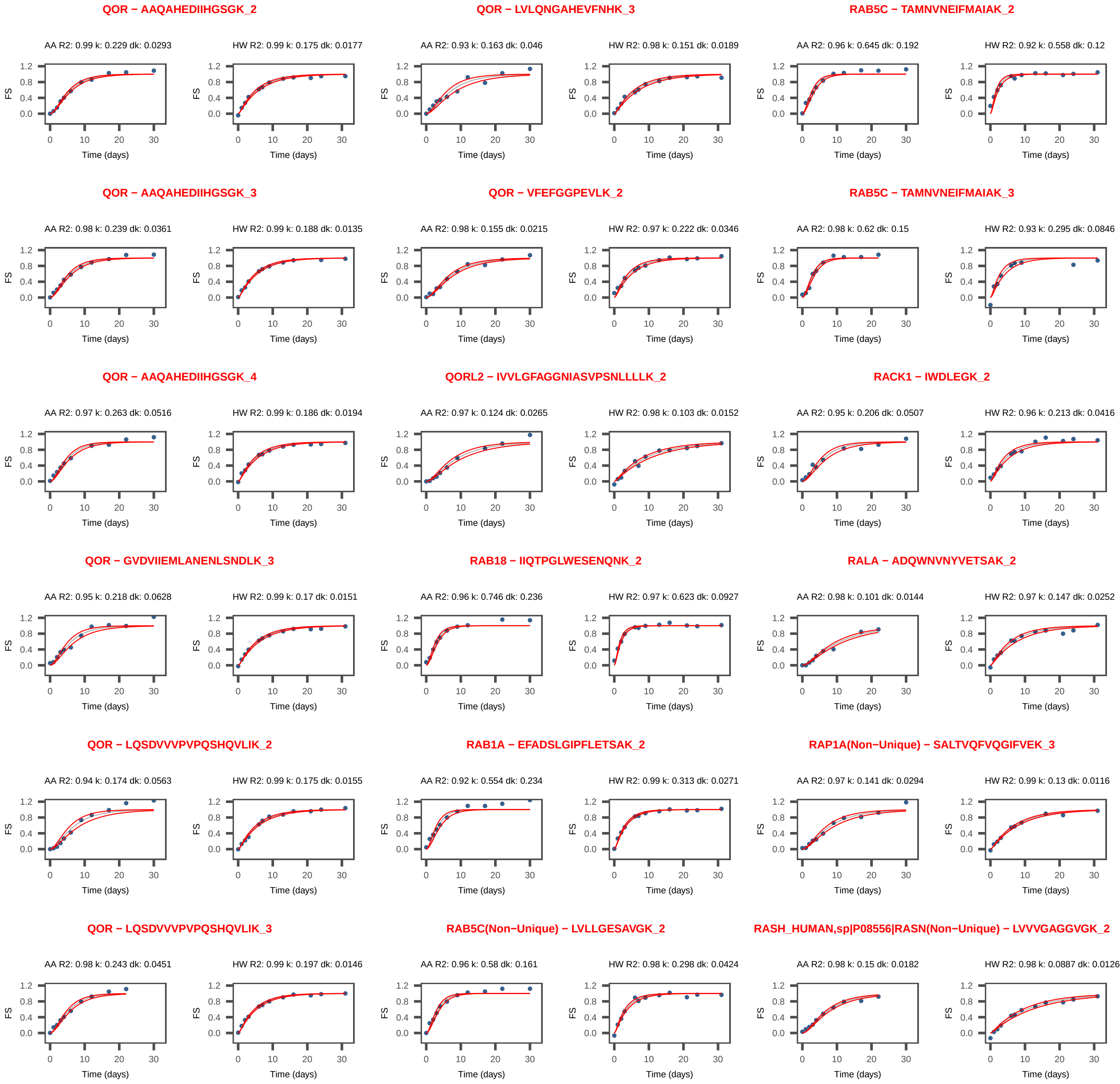
QOR − AAQAHEDIIHGSGK_2
AA R2: 0.99 k: 0.229 dk: 0.0293
HW R2: 0.99 k: 0.175 dk: 0.0177
QOR − LVLQNGAHEVFNHK_3
AA R2: 0.93 k: 0.163 dk: 0.046
HW R2: 0.98 k: 0.151 dk: 0.0189
RAB5C − TAMNVNEIFMAIAK_2
AA R2: 0.96 k: 0.645 dk: 0.192
HW R2: 0.92 k: 0.558 dk: 0.12
QOR − AAQAHEDIIHGSGK_3
AA R2: 0.98 k: 0.239 dk: 0.0361
HW R2: 0.99 k: 0.188 dk: 0.0135
QOR − VFEFGGPEVLK_2
AA R2: 0.98 k: 0.155 dk: 0.0215
HW R2: 0.97 k: 0.222 dk: 0.0346
RAB5C − TAMNVNEIFMAIAK_3
AA R2: 0.98 k: 0.62 dk: 0.15
HW R2: 0.93 k: 0.295 dk: 0.0846
QOR − AAQAHEDIIHGSGK_4
AA R2: 0.97 k: 0.263 dk: 0.0516
HW R2: 0.99 k: 0.186 dk: 0.0194
QORL2 − IVVLGFAGGNIASVPSNLLLLK_2
AA R2: 0.97 k: 0.124 dk: 0.0265
HW R2: 0.98 k: 0.103 dk: 0.0152
RACK1 − IWDLEGK_2
AA R2: 0.95 k: 0.206 dk: 0.0507
HW R2: 0.96 k: 0.213 dk: 0.0416
QOR − GVDVIIEMLANENLSNDLK_3
AA R2: 0.95 k: 0.218 dk: 0.0628
HW R2: 0.99 k: 0.17 dk: 0.0151
RAB18 − IIQTPGLWESENQNK_2
AA R2: 0.96 k: 0.746 dk: 0.236
HW R2: 0.97 k: 0.623 dk: 0.0927
RALA − ADQWNVNYVETSAK_2
AA R2: 0.98 k: 0.101 dk: 0.0144
HW R2: 0.97 k: 0.147 dk: 0.0252
QOR − LQSDVVVPVPQSHQVLIK_2
AA R2: 0.94 k: 0.174 dk: 0.0563
HW R2: 0.99 k: 0.175 dk: 0.0155
RAB1A − EFADSLGIPFLETSAK_2
AA R2: 0.92 k: 0.554 dk: 0.234
HW R2: 0.99 k: 0.313 dk: 0.0271
RAP1A(Non−Unique) − SALTVQFVQGIFVEK_3
AA R2: 0.97 k: 0.141 dk: 0.0294
HW R2: 0.99 k: 0.13 dk: 0.0116
QOR − LQSDVVVPVPQSHQVLIK_3
AA R2: 0.98 k: 0.243 dk: 0.0451
HW R2: 0.99 k: 0.197 dk: 0.0146
RAB5C(Non−Unique) − LVLLGESAVGK_2
AA R2: 0.96 k: 0.58 dk: 0.161
HW R2: 0.98 k: 0.298 dk: 0.0424
RASH_HUMAN,sp|P08556|RASN(Non−Unique) − LVVVGAGGVGK_2
AA R2: 0.98 k: 0.15 dk: 0.0182
HW R2: 0.98 k: 0.0887 dk: 0.0126
FS
Time (days)

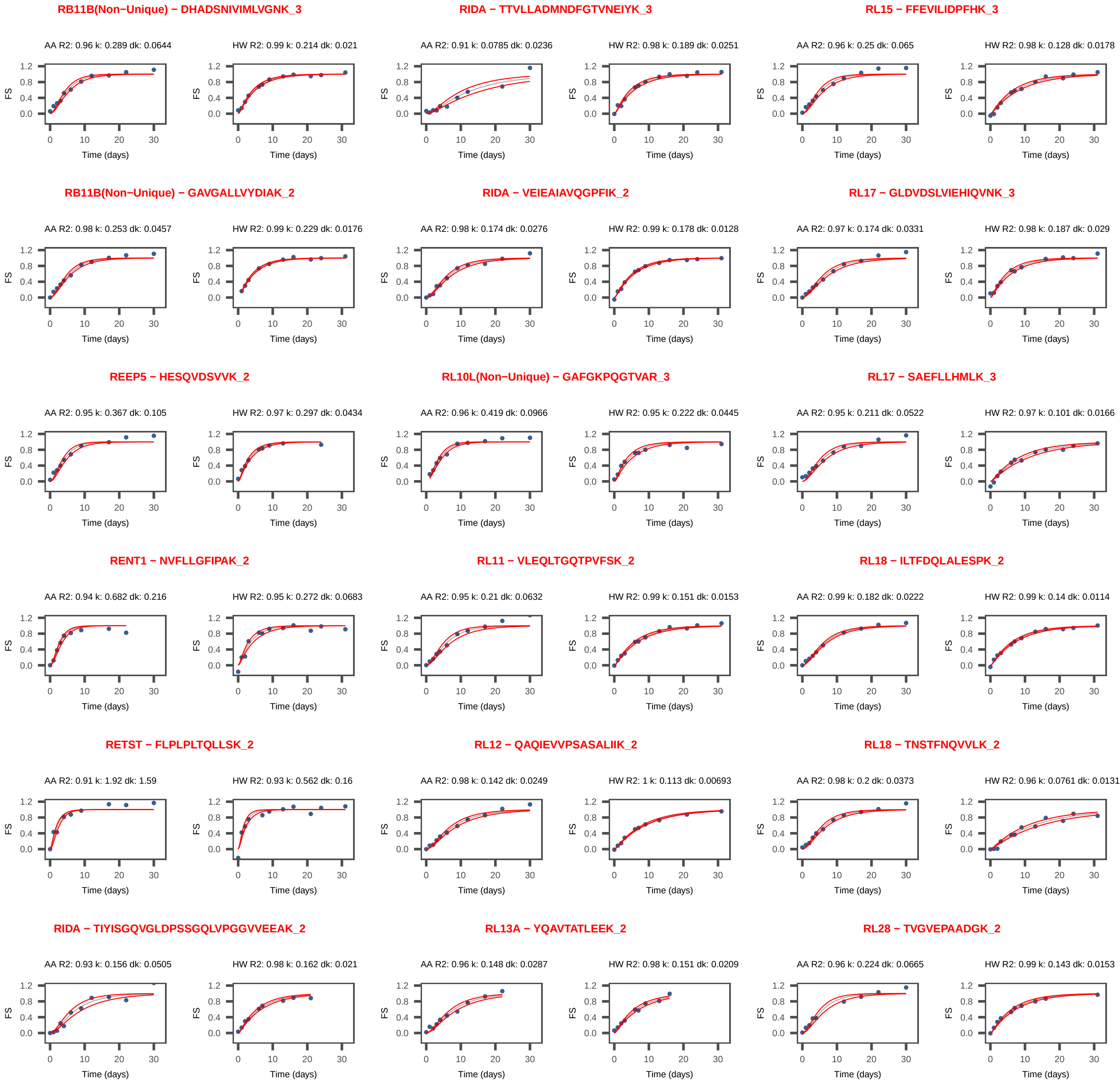
RB11B(Non−Unique) − DHADSNIVIMLVGNK_3
AA R2: 0.96 k: 0.289 dk: 0.0644
HW R2: 0.99 k: 0.214 dk: 0.021
RIDA − TTVLLADMNDFGTVNEIYK_3
AA R2: 0.91 k: 0.0785 dk: 0.0236
HW R2: 0.98 k: 0.189 dk: 0.0251
RL15 − FFEVILIDPFHK_3
AA R2: 0.96 k: 0.25 dk: 0.065
HW R2: 0.98 k: 0.128 dk: 0.0178
RB11B(Non−Unique) − GAVGALLVYDIAK_2
AA R2: 0.98 k: 0.253 dk: 0.0457
HW R2: 0.99 k: 0.229 dk: 0.0176
RIDA − VEIEAIAVQGPFIK_2
AA R2: 0.98 k: 0.174 dk: 0.0276
HW R2: 0.99 k: 0.178 dk: 0.0128
RL17 − GLDVDSLVIEHIQVNK_3
AA R2: 0.97 k: 0.174 dk: 0.0331
HW R2: 0.98 k: 0.187 dk: 0.029
REEP5 − HESQVDSVVK_2
AA R2: 0.95 k: 0.367 dk: 0.105
HW R2: 0.97 k: 0.297 dk: 0.0434
RL10L(Non−Unique) − GAFGKPQGTVAR_3
AA R2: 0.96 k: 0.419 dk: 0.0966
HW R2: 0.95 k: 0.222 dk: 0.0445
RL17 − SAEFLLHMLK_3
AA R2: 0.95 k: 0.211 dk: 0.0522
HW R2: 0.97 k: 0.101 dk: 0.0166
RENT1 − NVFLLGFIPAK_2
AA R2: 0.94 k: 0.682 dk: 0.216
HW R2: 0.95 k: 0.272 dk: 0.0683
RL11 − VLEQLTGQTPVFSK_2
AA R2: 0.95 k: 0.21 dk: 0.0632
HW R2: 0.99 k: 0.151 dk: 0.0153
RL18 − ILTFDQLALESPK_2
AA R2: 0.99 k: 0.182 dk: 0.0222
HW R2: 0.99 k: 0.14 dk: 0.0114
RETST − FLPLPLTQLLSK_2
AA R2: 0.91 k: 1.92 dk: 1.59
HW R2: 0.93 k: 0.562 dk: 0.16
RL12 − QAQIEVVPSASALIIK_2
AA R2: 0.98 k: 0.142 dk: 0.0249
HW R2: 1 k: 0.113 dk: 0.00693
RL18 − TNSTFNQVVLK_2
AA R2: 0.98 k: 0.2 dk: 0.0373
HW R2: 0.96 k: 0.0761 dk: 0.0131
RIDA − TIYISGQVGLDPSSGQLVPGGVVEEAK_2
AA R2: 0.93 k: 0.156 dk: 0.0505
HW R2: 0.98 k: 0.162 dk: 0.021
RL13A − YQAVTATLEEK_2
AA R2: 0.96 k: 0.148 dk: 0.0287
HW R2: 0.98 k: 0.151 dk: 0.0209
RL28 − TVGVEPAADGK_2
AA R2: 0.96 k: 0.224 dk: 0.0665
HW R2: 0.99 k: 0.143 dk: 0.0153
FS
Time (days)

RL3 – NNASTDYDLSDK_2

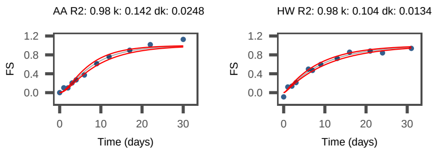

RL7 – VATVPGTLK_2

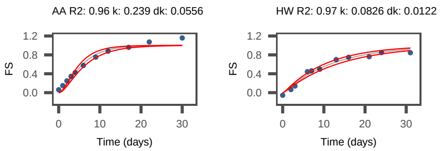

ROA1 – DYFEQYGK_2

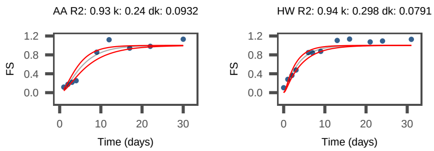

RL3 – SINPLGGFVHYGEVTNDFIMLK_3

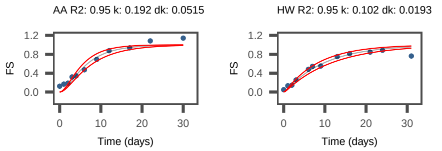

RL9 – DFNHINVELSLLGK_3

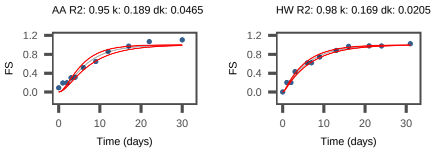

ROA2 – DYFEEYGK_2

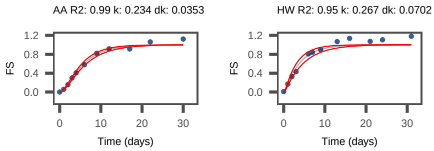

RL31 – LYTLVTYVPVTTFK_2

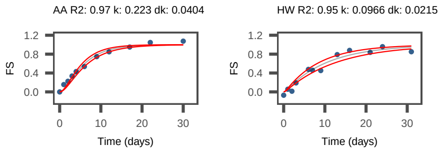

RLA0 – GHLENNPALEK_2

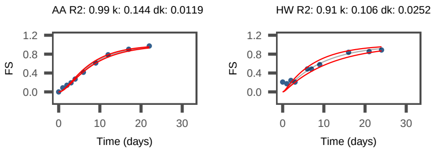

RPN1 – FPLFGGWK_2

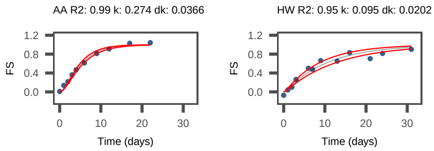

RL4 – NVTLPAVFK_2

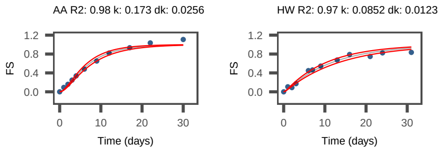

RLA0 – GTIEILSDVQLIK_2

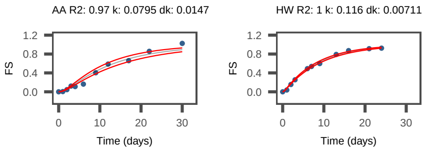

RPN1 – LPVALDPGSK_2

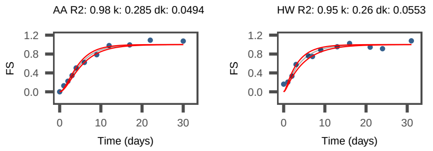

RL5 – GAVDGGLSIPHSTK_3

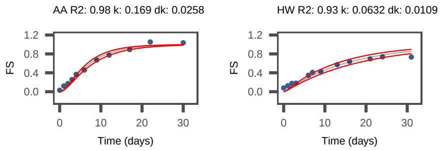

RLA0 – IIQLLDDYPK_2

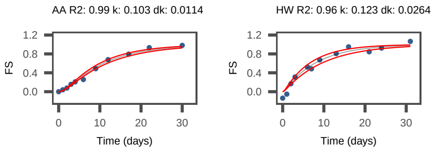

RPN2 – FPEEEAPSTVLSQSLFTPK_2

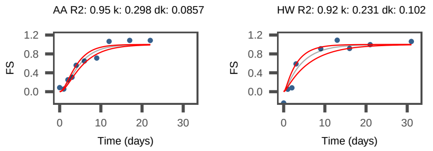

RL7 – FGIICMEDLIHEIYTVGK_3

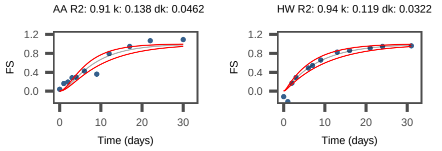

RLA1 – AAGVSVEPFWPGLFAK_2

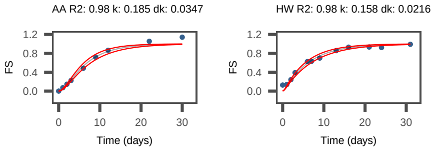

RS10 – IAIYELLFK_2

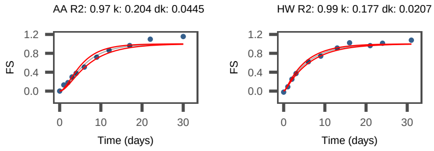

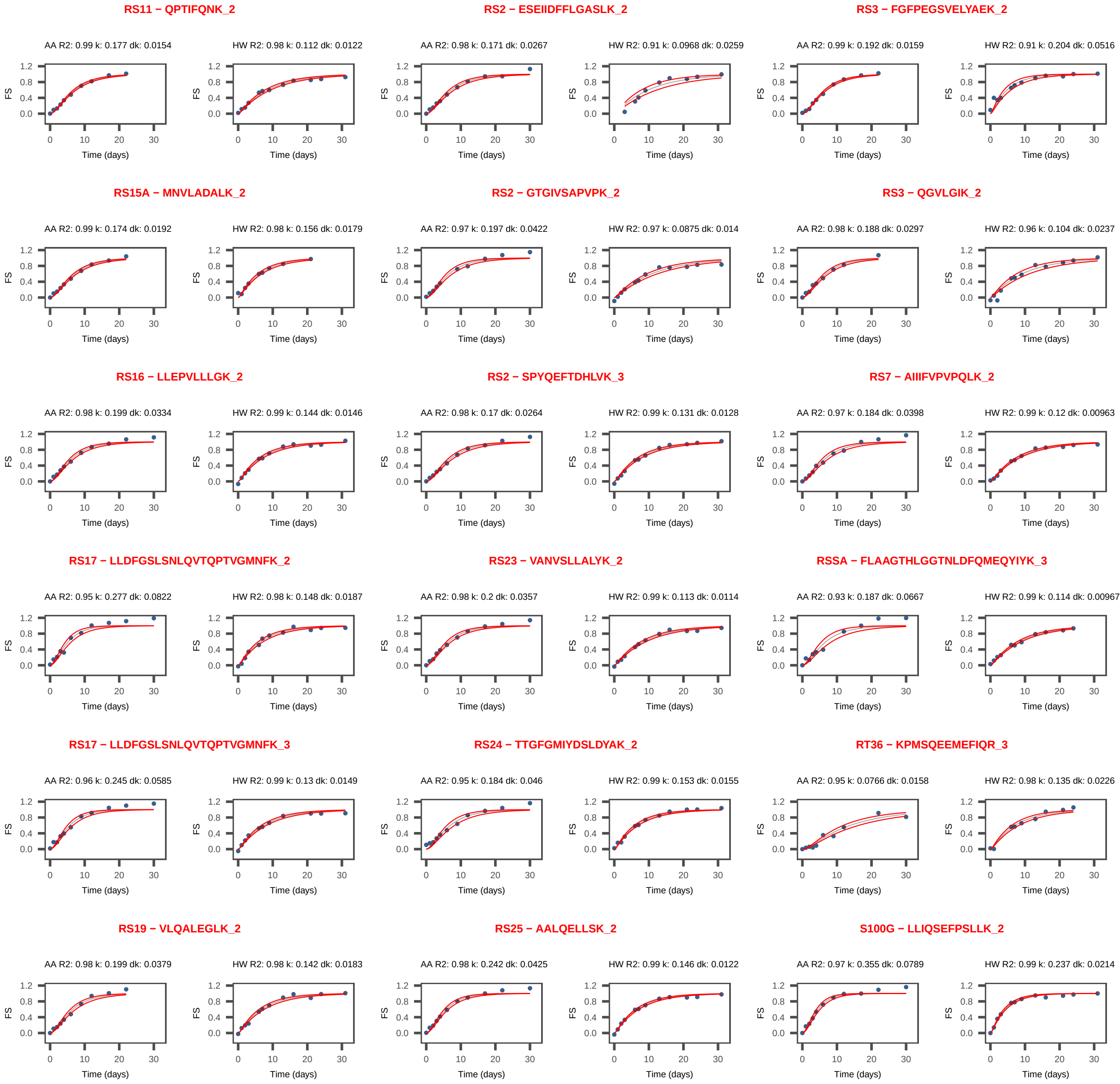
RS11 − QPTIFQNK_2
AA R2: 0.99 k: 0.177 dk: 0.0154
HW R2: 0.98 k: 0.112 dk: 0.0122
RS2 − ESEIIDFFLGASLK_2
AA R2: 0.98 k: 0.171 dk: 0.0267
HW R2: 0.91 k: 0.0968 dk: 0.0259
RS3 − FGFPEGSVELYAEK_2
AA R2: 0.99 k: 0.192 dk: 0.0159
HW R2: 0.91 k: 0.204 dk: 0.0516
RS15A − MNVLADALK_2
AA R2: 0.99 k: 0.174 dk: 0.0192
HW R2: 0.98 k: 0.156 dk: 0.0179
RS2 − GTGIVSAPVPK_2
AA R2: 0.97 k: 0.197 dk: 0.0422
HW R2: 0.97 k: 0.0875 dk: 0.014
RS3 − QGVLGIK_2
AA R2: 0.98 k: 0.188 dk: 0.0297
HW R2: 0.96 k: 0.104 dk: 0.0237
RS16 − LLEPVLLLGK_2
AA R2: 0.98 k: 0.199 dk: 0.0334
HW R2: 0.99 k: 0.144 dk: 0.0146
RS2 − SPYQEFTDHLVK_3
AA R2: 0.98 k: 0.17 dk: 0.0264
HW R2: 0.99 k: 0.131 dk: 0.0128
RS7 − AIIIFVPVPQLK_2
AA R2: 0.97 k: 0.184 dk: 0.0398
HW R2: 0.99 k: 0.12 dk: 0.00963
RS17 − LLDFGSLSNLQVTQPTVGMNFK_2
AA R2: 0.95 k: 0.277 dk: 0.0822
HW R2: 0.98 k: 0.148 dk: 0.0187
RS23 − VANVSLLALYK_2
AA R2: 0.98 k: 0.2 dk: 0.0357
HW R2: 0.99 k: 0.113 dk: 0.0114
RSSA − FLAAGTHLGGTNLDFQMEQYIYK_3
AA R2: 0.93 k: 0.187 dk: 0.0667
HW R2: 0.99 k: 0.114 dk: 0.00967
RS17 − LLDFGSLSNLQVTQPTVGMNFK_3
AA R2: 0.96 k: 0.245 dk: 0.0585
HW R2: 0.99 k: 0.13 dk: 0.0149
RS24 − TTGFGMIYDSLDYAK_2
AA R2: 0.95 k: 0.184 dk: 0.046
HW R2: 0.99 k: 0.153 dk: 0.0155
RT36 − KPMSQEEMEFIQR_3
AA R2: 0.95 k: 0.0766 dk: 0.0158
HW R2: 0.98 k: 0.135 dk: 0.0226
RS19 − VLQALEGLK_2
AA R2: 0.98 k: 0.199 dk: 0.0379
HW R2: 0.98 k: 0.142 dk: 0.0183
RS25 − AALQELLSK_2
AA R2: 0.98 k: 0.242 dk: 0.0425
HW R2: 0.99 k: 0.146 dk: 0.0122
S100G − LLIQSEFPSLLK_2
AA R2: 0.97 k: 0.355 dk: 0.0789
HW R2: 0.99 k: 0.237 dk: 0.0214
FS
Time (days)

S10A1 − DLLQTELSGFLDVQK_2

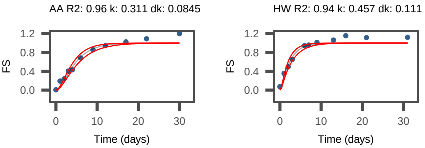

S27A2 − ENYEFNGK_2

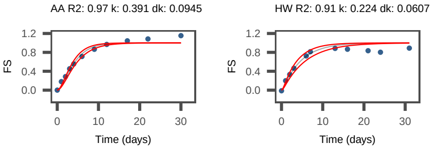

S27A2 − VTLMEEGFNPTVIK_2

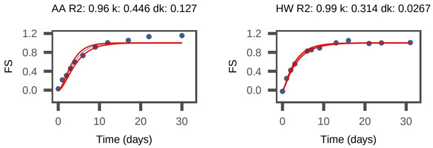

S10A1 − DLLQTELSGFLDVQK_3

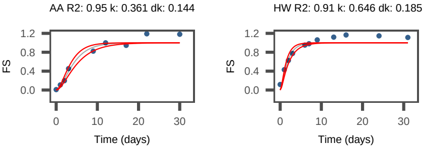

S27A2 − IQDTIEITGTFK_2

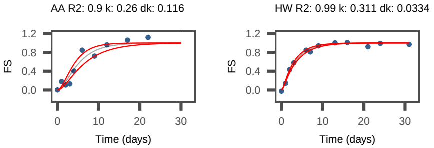

S27A2 − YDVEKDEPVR_2

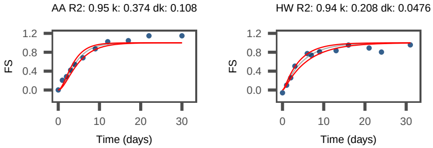

S22A6 − DGGLEAWLPLDK_2

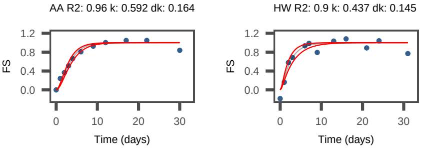

S27A2 − SEVTFTTPAVYIYTSGTTGLPK_2

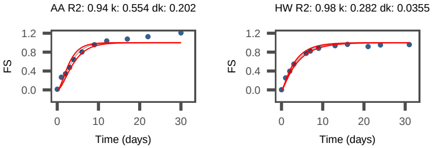

S27A2 − YDVEKDEPVR_3

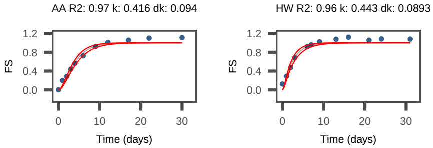

S22A6 − LSIEVLQTSLQK_2

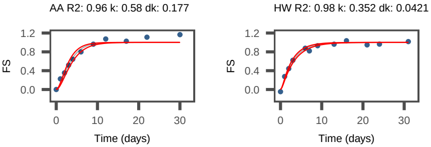

S27A2 − TFVPMTENIYNAIIDK_3

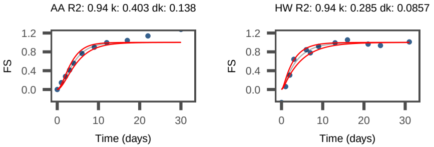

S27A2 − YLCNTPQKPNDR_3

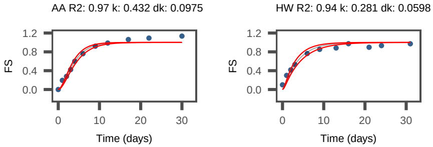

S22AI − VLPVFLVK_2

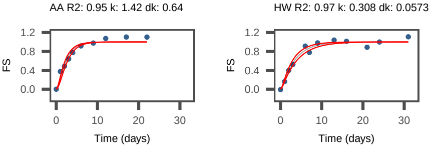

S27A2 − VLLASPDLQEAVEEVLPTLK_2

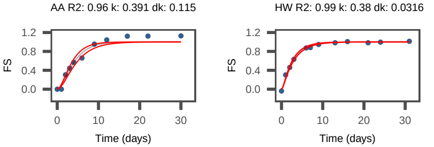

S4A4 − FLFILLGPK_2

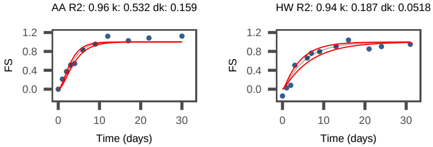

S23A1 − SYDFPFGMGMVK_2

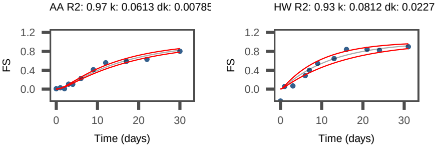

S27A2 − VLLASPDLQEAVEEVLPTLK_3

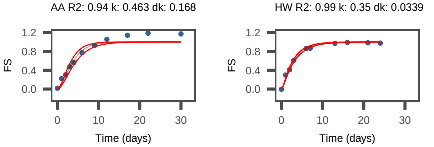

S4A4 − LADYYPINSDFK_2

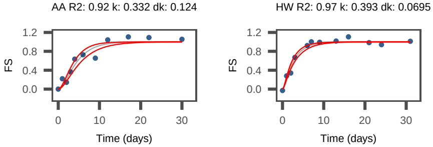

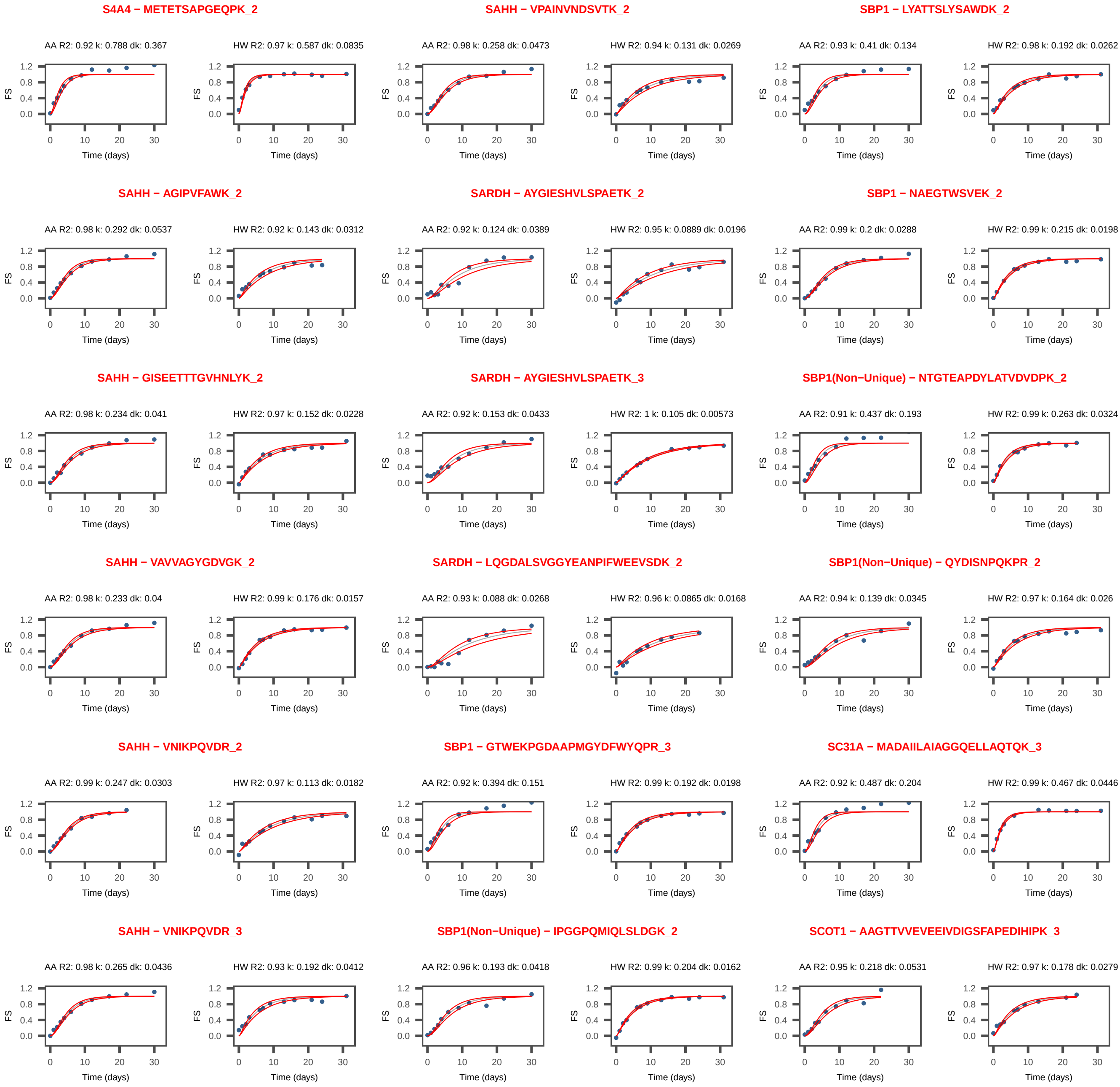
S4A4 − METETSAPGEQPK_2
AA R2: 0.92 k: 0.788 dk: 0.367
HW R2: 0.97 k: 0.587 dk: 0.0835
SAHH − VPAINVNDSVTK_2
AA R2: 0.98 k: 0.258 dk: 0.0473
HW R2: 0.94 k: 0.131 dk: 0.0269
SBP1 − LYATTSLYSAWDK_2
AA R2: 0.93 k: 0.41 dk: 0.134
HW R2: 0.98 k: 0.192 dk: 0.0262
SAHH − AGIPVFAWK_2
AA R2: 0.98 k: 0.292 dk: 0.0537
HW R2: 0.92 k: 0.143 dk: 0.0312
SARDH − AYGIESHVLSPAETK_2
AA R2: 0.92 k: 0.124 dk: 0.0389
HW R2: 0.95 k: 0.0889 dk: 0.0196
SBP1 − NAEGTWSVEK_2
AA R2: 0.99 k: 0.2 dk: 0.0288
HW R2: 0.99 k: 0.215 dk: 0.0198
SAHH − GISEETTTGVHNLYK_2
AA R2: 0.98 k: 0.234 dk: 0.041
HW R2: 0.97 k: 0.152 dk: 0.0228
SARDH − AYGIESHVLSPAETK_3
AA R2: 0.92 k: 0.153 dk: 0.0433
HW R2: 1 k: 0.105 dk: 0.00573
SBP1(Non−Unique) − NTGTEAPDYLATVDVDPK_2
AA R2: 0.91 k: 0.437 dk: 0.193
HW R2: 0.99 k: 0.263 dk: 0.0324
SAHH − VAVVAGYGDVGK_2
AA R2: 0.98 k: 0.233 dk: 0.04
HW R2: 0.99 k: 0.176 dk: 0.0157
SARDH − LQGDALSVGGYEANPIFWEEVSDK_2
AA R2: 0.93 k: 0.088 dk: 0.0268
HW R2: 0.96 k: 0.0865 dk: 0.0168
SBP1(Non−Unique) − QYDISNPQKPR_2
AA R2: 0.94 k: 0.139 dk: 0.0345
HW R2: 0.97 k: 0.164 dk: 0.026
SAHH − VNIKPQVDR_2
AA R2: 0.99 k: 0.247 dk: 0.0303
HW R2: 0.97 k: 0.113 dk: 0.0182
SBP1 − GTWEKPGDAAPMGYDFWYQPR_3
AA R2: 0.92 k: 0.394 dk: 0.151
HW R2: 0.99 k: 0.192 dk: 0.0198
SC31A − MADAIILAIAGGQELLAQTQK_3
AA R2: 0.92 k: 0.487 dk: 0.204
HW R2: 0.99 k: 0.467 dk: 0.0446
SAHH − VNIKPQVDR_3
AA R2: 0.98 k: 0.265 dk: 0.0436
HW R2: 0.93 k: 0.192 dk: 0.0412
SBP1(Non−Unique) − IPGGPQMIQLSLDGK_2
AA R2: 0.96 k: 0.193 dk: 0.0418
HW R2: 0.99 k: 0.204 dk: 0.0162
SCOT1 − AAGTTVVEVEEIVDIGSFAPEDIHIPK_3
AA R2: 0.95 k: 0.218 dk: 0.0531
HW R2: 0.97 k: 0.178 dk: 0.0279
FS
Time (days)

SCOT1 – FYTDPVEAVK_2

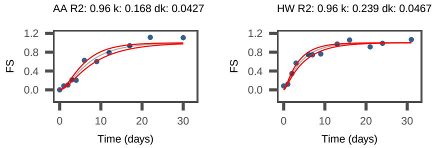

SCP2 – GSVLPNSDK_2

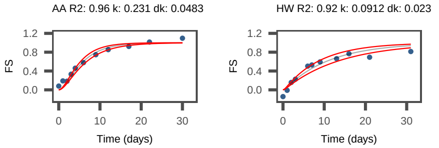

SDHA – WHFYDTVK_3

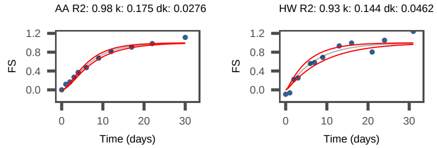

SCOT1 – GMGGAMDLVSSSK_2

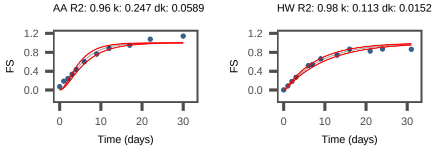

SCP2 – IGGIFAFK_2

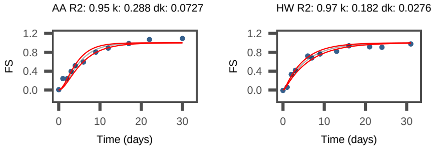

SDHB – DLVPDLSNFYAQYK_2

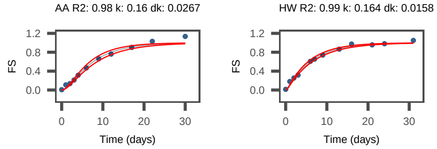

SCOT1 – VVVTMEHSAK_2

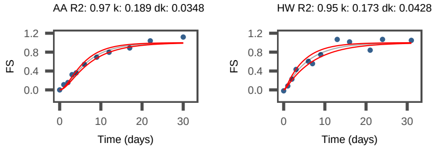

SCP2 – LQNLQLQPGK_2

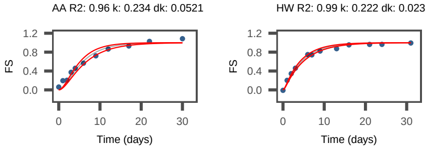

SFXN1 – YAYDSAFHPDTGEK_3

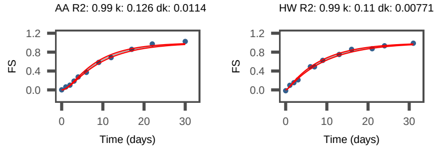

SCOT1 – YGDLANWMIPGK_2

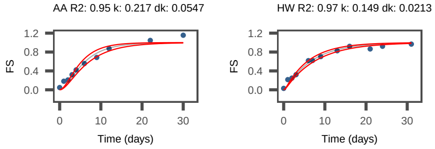

SDHA – ANAGEESVMNLDK_2

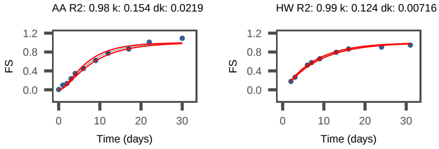

SLC31 – DFENLVAAIHDK_3

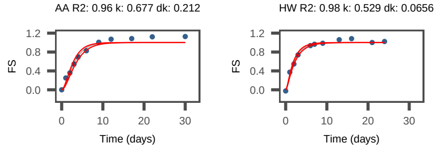

SCP2 – ADCTITMADSDLLALMTGK_2

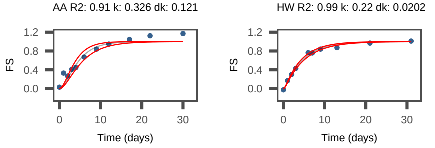

SDHA – KPFGEHWR_3

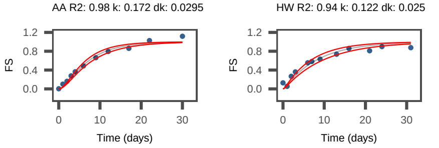

SLC31 – TMMYYGLPFIQEADFPFNK_2

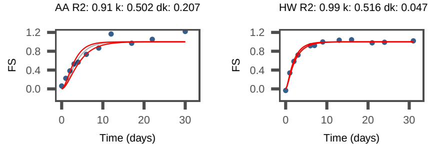

SCP2 – ADCTITMADSDLLALMTGK_3

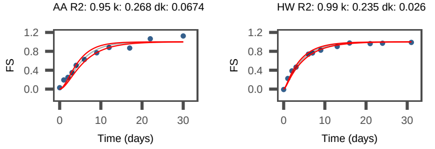

SDHA – VGSVLQEGCEK_2

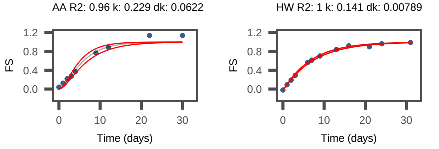

SMD3 – FLILPDMLK_2

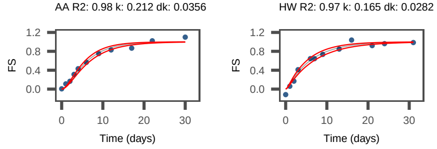

SND1 − VITEYLNAQESAK_2

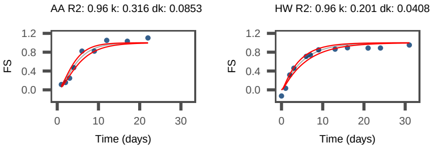

SODM − HHAAYVNNLNATEEK_4

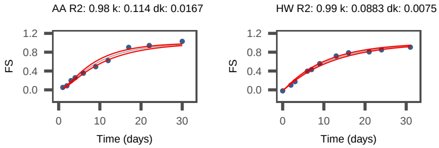

SPTN1 − DLSSVQTLLTK_2

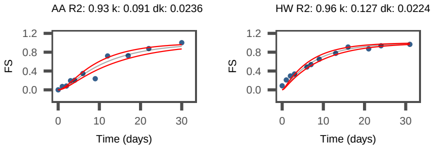

SODC − GDGPVQGTIHFEQK_2

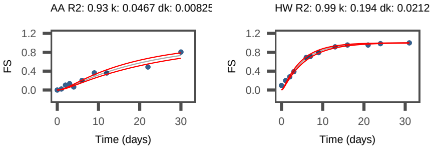

SODM − YHEALAK_2

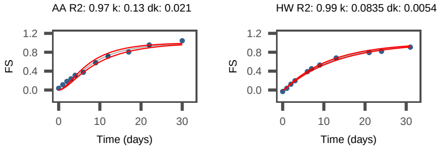

SPTN1 − DMDDEESWIK_2

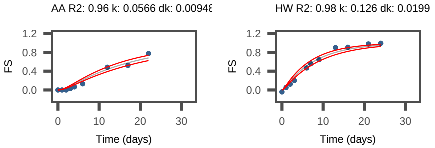

SODC − GDGPVQGTIHFEQK_3

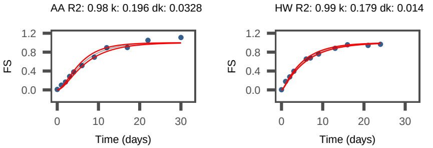

SPA3K(Non−Unique) − DLQILAEFHEK_3

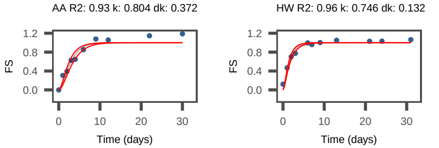

SPTN1 − EKEPIAASTNR_3

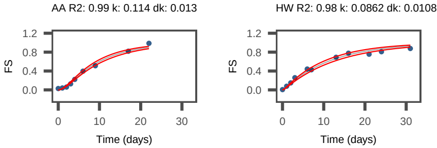

SODC − HVGDLGNVTAGK_2

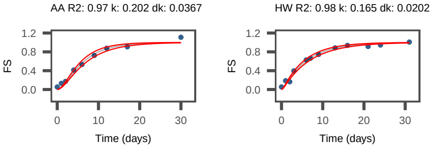

SPTB2 − HLLGVEDLLQK_3

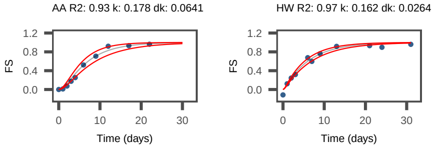

SPTN1 − IAALQAFADQLIAVDHYAK_3

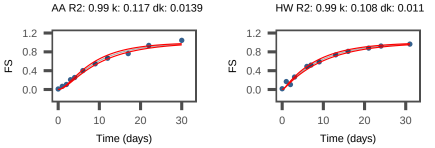

SODM − GDVTTQVALQPALK_2

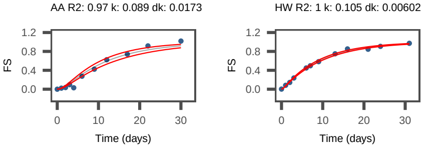

SPTB2 − LQALDTGWNELHK_3

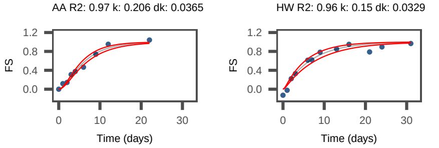

SPTN1 − KQEALVAR_2

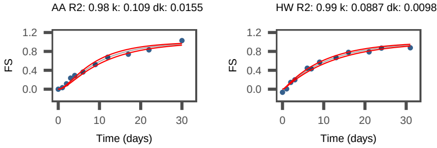

SODM − GELLEAIK_2

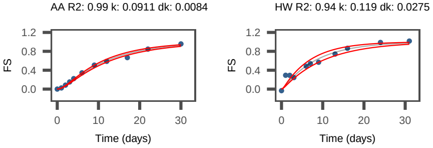

SPTB2 − NEIDNYEEDYQK_2

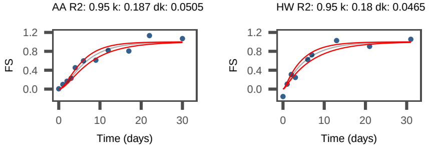

SPTN1 − LQQSHPLSASQIQVK_3

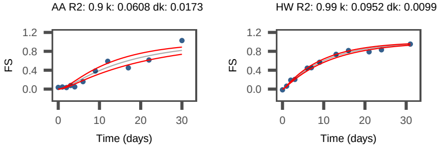

SQOR − ANIIFNTALGTIFGVK_2

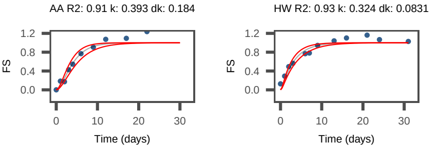

ST1D1 − THLPVQLLPSSFWK_3

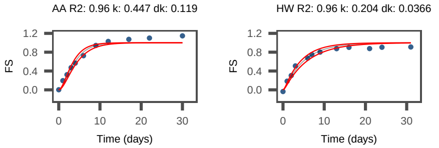

SUCA − MGHAGAIIAGGK_3

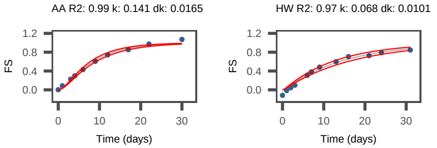

SQOR − ANIIFNTALGTIFGVK_3

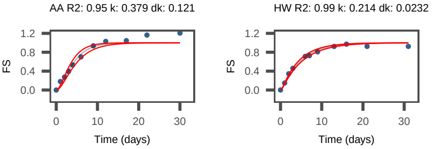

STIP1 − LLEFQLALK_2

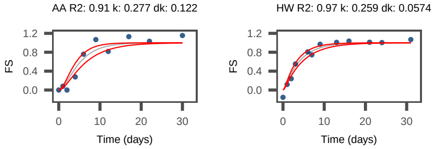

SUCA − QGTFHSQQALEYGTK_2

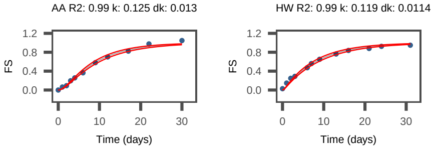

ST1C2 − VSWGSWFDHVK_3

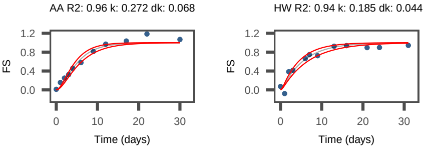

SUCA − AKPVVSFIAGITAPPGR_3

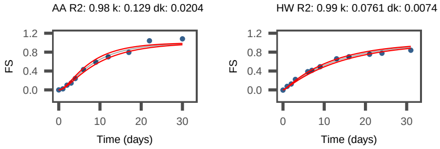

SUCA − QGTFHSQQALEYGTK_3

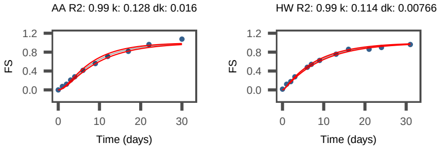

ST1D1 − FEEDYVK_2

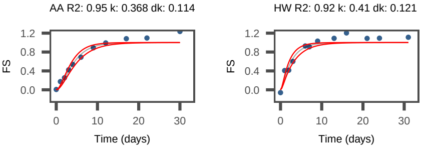

SUCA − HLGLPVFNTVK_3

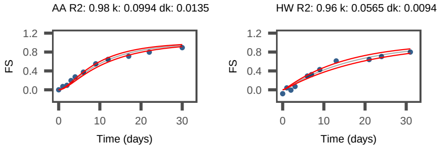

SUCB1 − ALIADSGLK_2

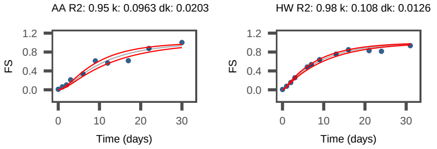

ST1D1 − FMAGQVSFGPWYDHVK_3

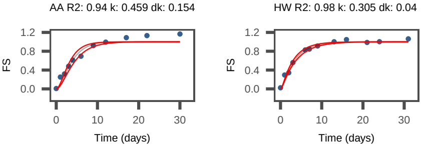

SUCA − ISALQSAGVVVSMSPAQLGTTIYK_2

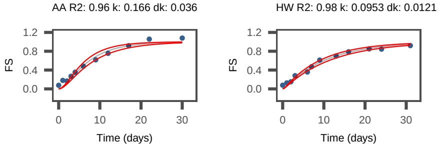

SUCB1 − EQAVTLAQK_2

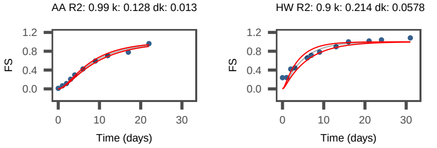

ST1D1(Non−Unique) − ILYLFYEDMK_2

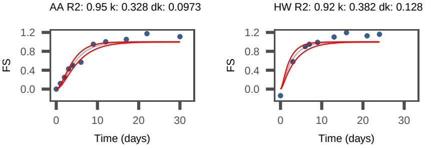

SUCA − MGHAGAIIAGGK_2

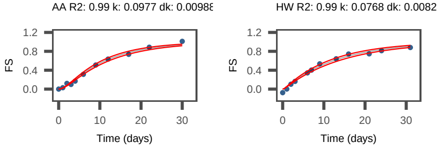

SUCB1 − LHGGTPANFLDVGGGATVQQVTEAFK_3

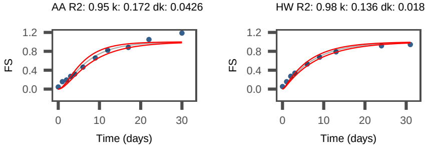

SUCB1 − LSEIVTLAK_2

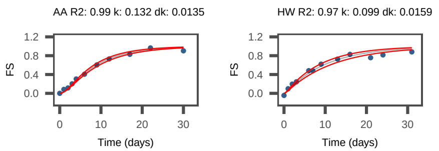

SUCB1 − LYNLFLK_2

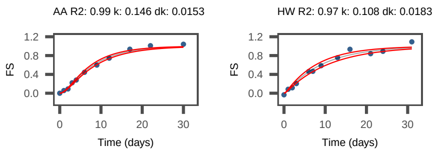

SUCB2 − EAQVYEAFK_2

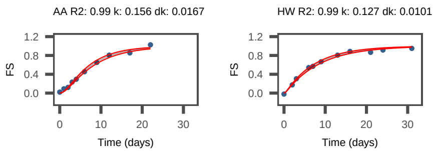

SUCB2 − SSGLPITSAVDLEDAAK_2

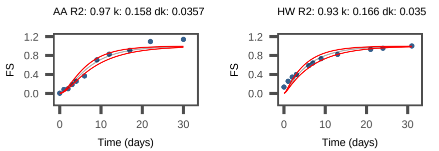

SUCB2 − VVGELAQQMIGYNLATK_2

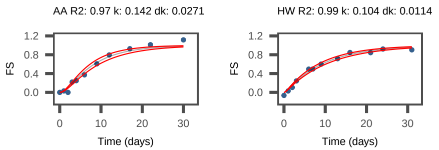

SUSD2 − GMFLSVAAQDK_2

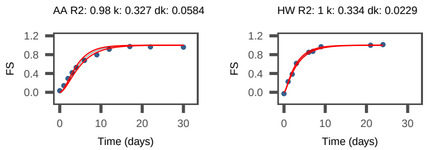

SUSD2 − VLSFTEQNWMDLK_2

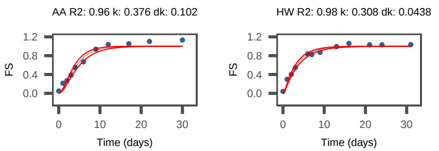

SYEP − SQGSGLSSGGAGEGQGPK_2

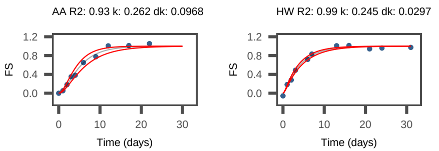

SYFA − SIPLEGLVQSELMHLPSGK_3

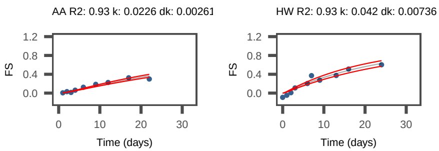

TADBP − TSDLIVLGLPWK_2

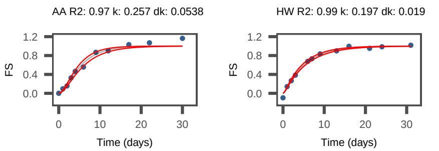

TAGL2 − DDGLFSGDPNWFPK_2

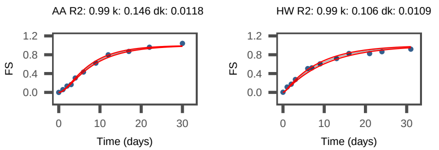

TAGL2 − YGINTTDIFQTVDLWEGK_2

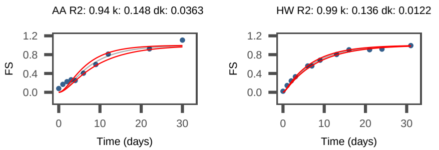

TALDO − LFVLFGAEILK_2

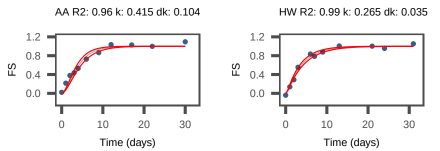

TALDO − LGGPQEEQIK_2

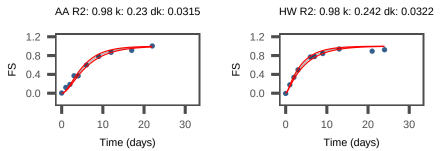

TALDO − SYEPQEDPGVK_2

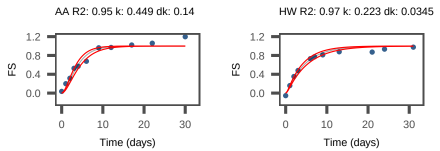

TALDO − WLHNEDQMAVEK_3

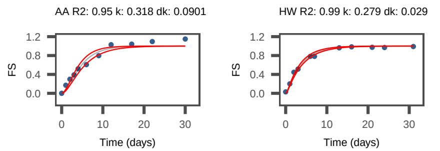

TBA1B(Non−Unique) − IHFPLATYAPVISAEK_2

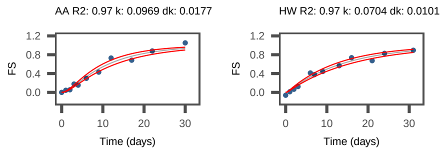

TBA1B(Non−Unique) − IHFPLATYAPVISAEK_3

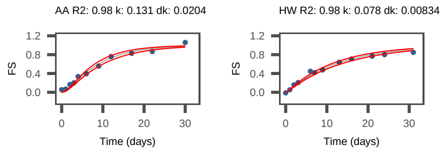

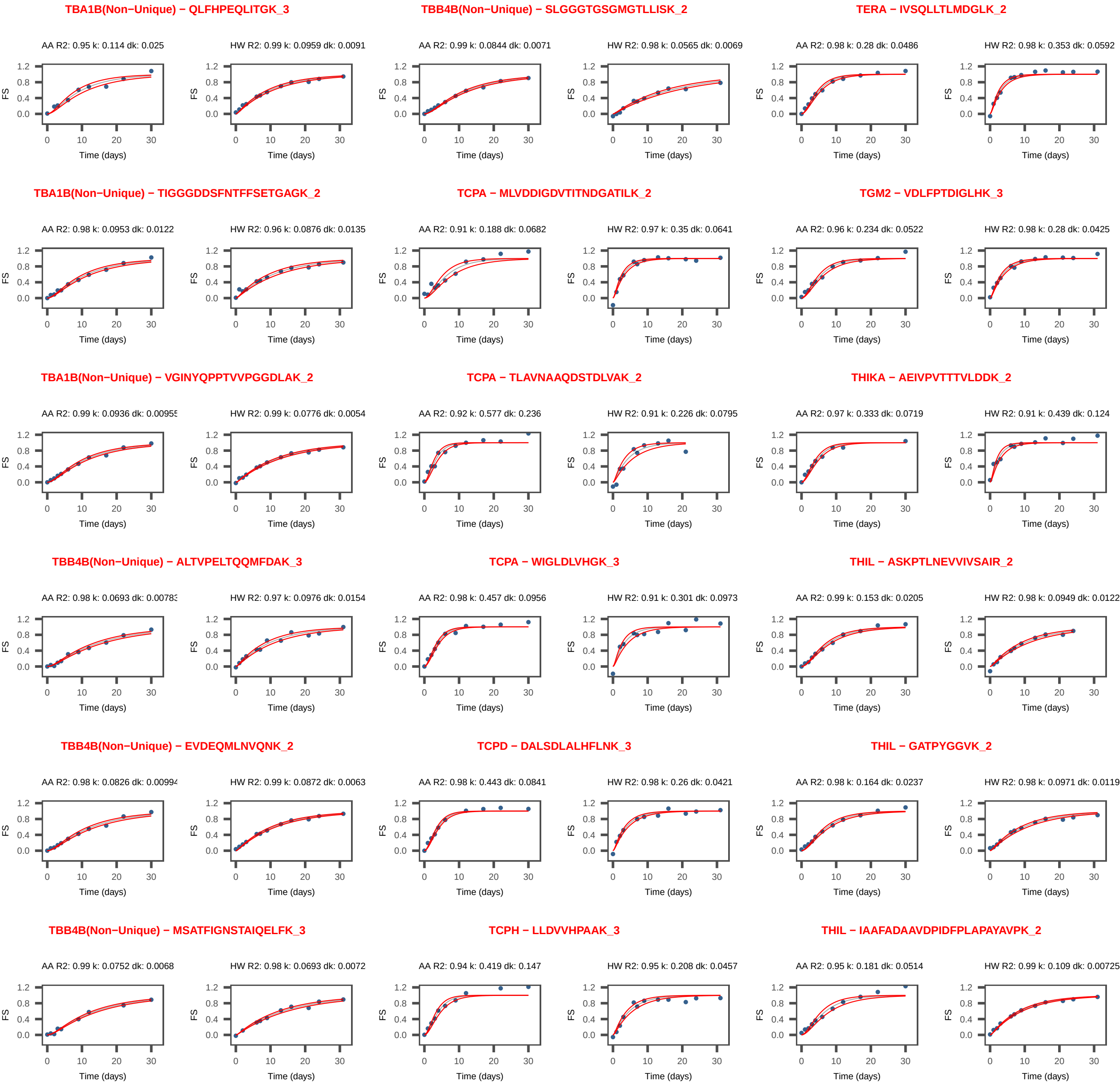
TBA1B(Non−Unique) − QLFHPEQLITGK_3
AA R2: 0.95 k: 0.114 dk: 0.025
HW R2: 0.99 k: 0.0959 dk: 0.0091
TBB4B(Non−Unique) − SLGGGTGSGMGTLLISK_2
AA R2: 0.99 k: 0.0844 dk: 0.0071
HW R2: 0.98 k: 0.0565 dk: 0.0069
TERA − IVSQLLTLMDGLK_2
AA R2: 0.98 k: 0.28 dk: 0.0486
HW R2: 0.98 k: 0.353 dk: 0.0592
TBA1B(Non−Unique) − TIGGGDDSFNTFFSETGAGK_2
AA R2: 0.98 k: 0.0953 dk: 0.0122
HW R2: 0.96 k: 0.0876 dk: 0.0135
TCPA − MLVDDIGDVTITNDGATILK_2
AA R2: 0.91 k: 0.188 dk: 0.0682
HW R2: 0.97 k: 0.35 dk: 0.0641
TGM2 − VDLFPTDIGLHK_3
AA R2: 0.96 k: 0.234 dk: 0.0522
HW R2: 0.98 k: 0.28 dk: 0.0425
TBA1B(Non−Unique) − VGINYQPPTVVPGGDLAK_2
AA R2: 0.99 k: 0.0936 dk: 0.00955
HW R2: 0.99 k: 0.0776 dk: 0.0054
TCPA − TLAVNAAQDSTDLVAK_2
AA R2: 0.92 k: 0.577 dk: 0.236
HW R2: 0.91 k: 0.226 dk: 0.0795
THIKA − AEIVPVTTTVLDDK_2
AA R2: 0.97 k: 0.333 dk: 0.0719
HW R2: 0.91 k: 0.439 dk: 0.124
TBB4B(Non−Unique) − ALTVPELTQQMFDAK_3
AA R2: 0.98 k: 0.0693 dk: 0.00783
HW R2: 0.97 k: 0.0976 dk: 0.0154
TCPA − WIGLDLVHGK_3
AA R2: 0.98 k: 0.457 dk: 0.0956
HW R2: 0.91 k: 0.301 dk: 0.0973
THIL − ASKPTLNEVVIVSAIR_2
AA R2: 0.99 k: 0.153 dk: 0.0205
HW R2: 0.98 k: 0.0949 dk: 0.0122
TBB4B(Non−Unique) − EVDEQMLNVQNK_2
AA R2: 0.98 k: 0.0826 dk: 0.00994
HW R2: 0.99 k: 0.0872 dk: 0.0063
TCPD − DALSDLALHFLNK_3
AA R2: 0.98 k: 0.443 dk: 0.0841
HW R2: 0.98 k: 0.26 dk: 0.0421
THIL − GATPYGGVK_2
AA R2: 0.98 k: 0.164 dk: 0.0237
HW R2: 0.98 k: 0.0971 dk: 0.0119
TBB4B(Non−Unique) − MSATFIGNSTAIQELFK_3
AA R2: 0.99 k: 0.0752 dk: 0.0068
HW R2: 0.98 k: 0.0693 dk: 0.0072
TCPH − LLDVVHPAAK_3
AA R2: 0.94 k: 0.419 dk: 0.147
HW R2: 0.95 k: 0.208 dk: 0.0457
THIL − IAAFADAAVDPIDFPLAPAYAVPK_2
AA R2: 0.95 k: 0.181 dk: 0.0514
HW R2: 0.99 k: 0.109 dk: 0.00725
FS
Time (days)

THIL − IAAFADAAVDPIDFPLAPAYAVPK_3

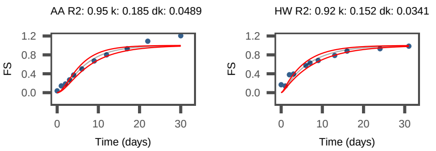

THIL − TPIGSFLGSLASQPATK_3

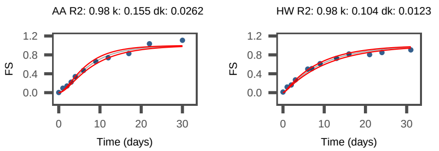

TIM50 − VLLDLSAFLK_2

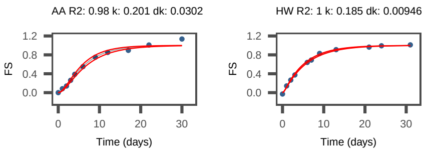

THIL − LGTAAIQGAIEK_2

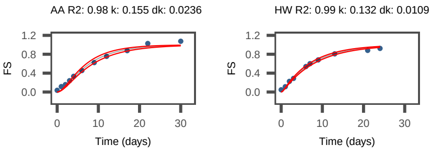

THIM − DMDLIDVNEAFAPQFLSVQK_2

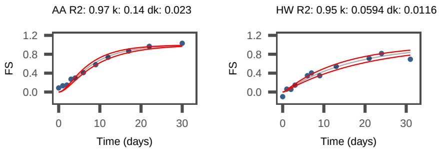

TKFC − AVAQAGTVGTLLIVK_2

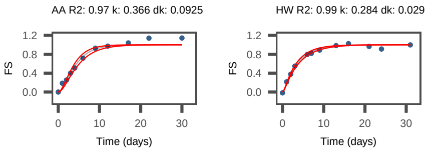

THIL − LNVKPLAR_2

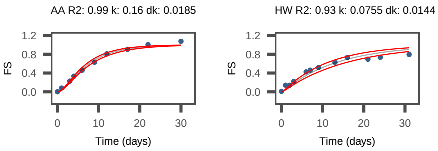

THIM − DMDLIDVNEAFAPQFLSVQK_3

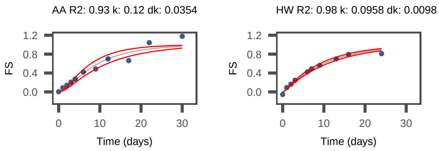

TKFC − MGGSSGALYGLFLTAAAQPLK_2

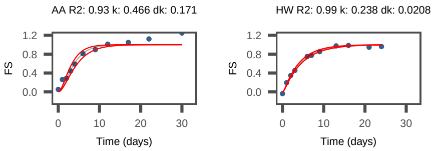

THIL − MLEIDPQK_2

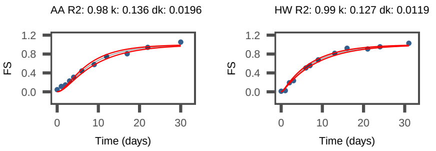

THIM − LEDTLWAGLTDQHVK_3

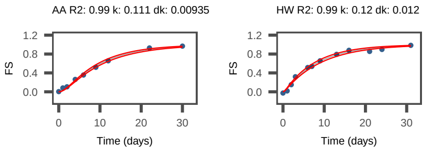

TKFC − TMLDSLWAAAQEFQAWK_3

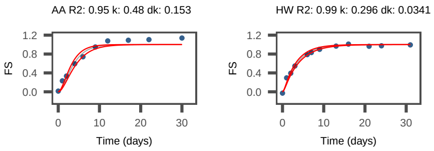

THIL − QATLGAGLPISTPCTTVNK_2

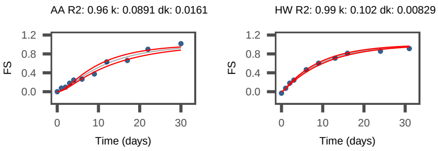

THTR − ATLNLSLLK_2

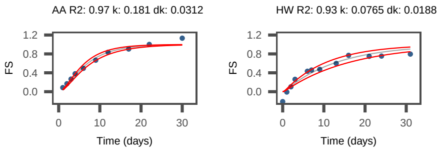

TKT − AVELAANTK_2

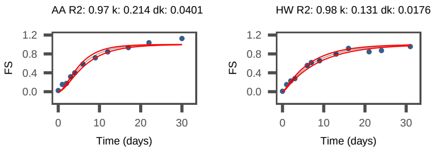

THIL − TPIGSFLGSLASQPATK_2

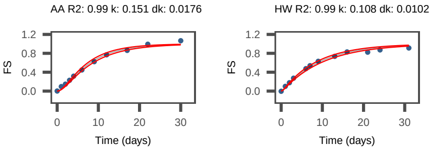

THTR − GSVNMPFMDFLTK_2

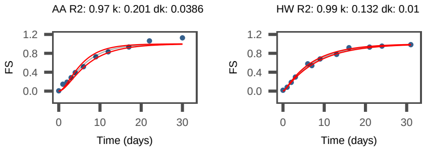

TKT − GITGIEDK_2

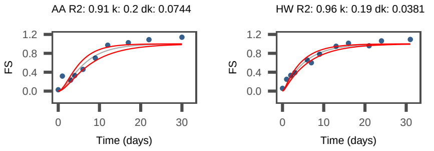

TKT – HQPTAIIAK_2

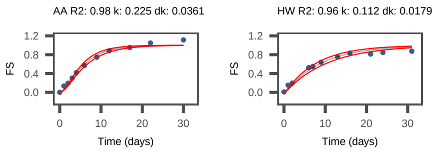

TMM19 – TGLVVSSPTQETK_2

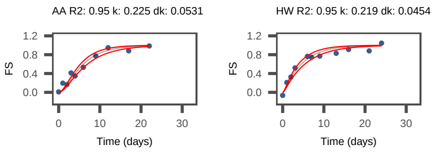

TPMT – EFQYLVAVLSYDPTK_2

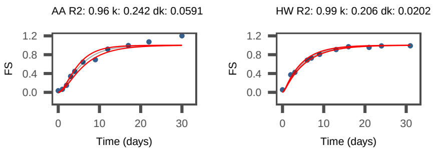

TKT – IIALDGDTK_2

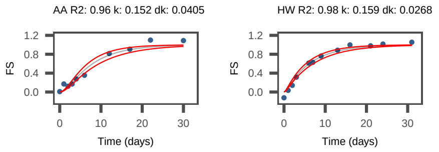

TOM40 – MQDTSASFGYQLDLPK_2

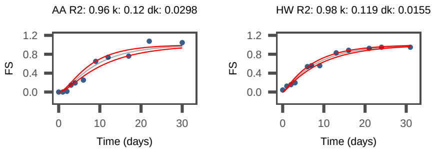

TRAP1 – AFLEALQNQAETSSK_2

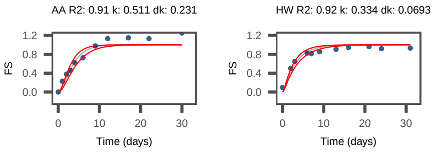

TKT – LGQSDPAPLQHQVDIYQK_2

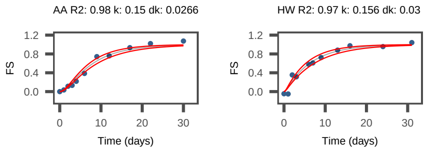

TPIS – FFVGGNWK_2

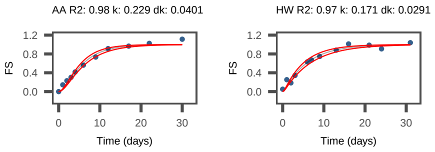

TRFE – DFQLFSSPLGK_2

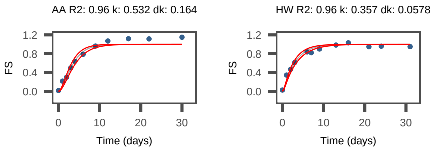

TKT – LGQSDPAPLQHQVDIYQK_3

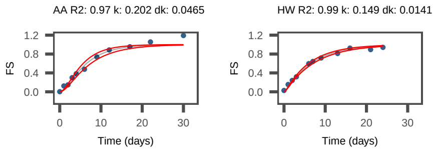

TPIS – TATPQQAQEVHEK_2

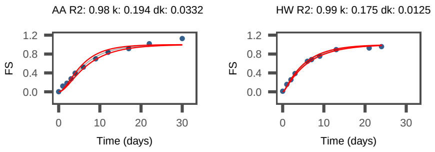

TRFE – EDLIWEILK_2

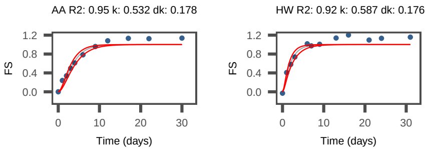

TKT – NSTFSELFK_2

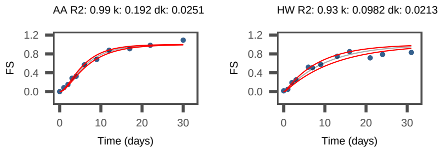

TPIS – TATPQQAQEVHEK_3

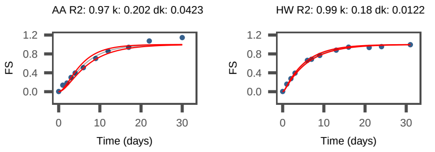

TRFE – HTTIFEVLPEK_2

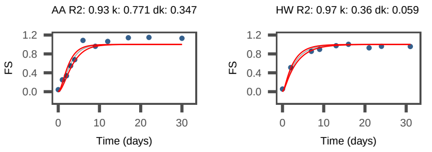

TLN1 – GVGAAATAVTQALNELLQHVK_3

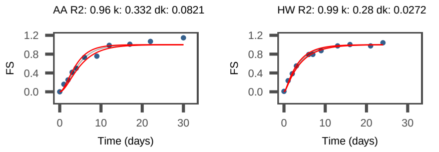

TPIS – VVLAYEPVWAIGTGK_3

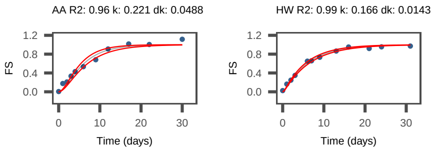

TRFE – LPEGTTPEK_2

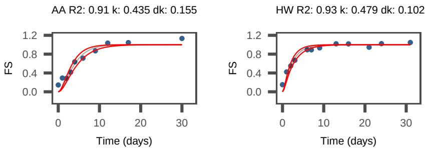

TXTP – FIHDQTSSNPK_2

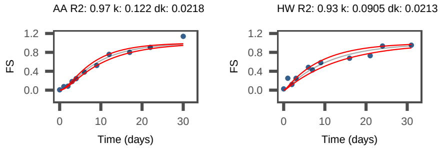

UCRI – RAEVLDSTK_2

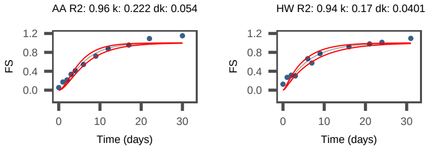

VA0D1 – LHLQSTDYGNFLANEASPLTVSVIDDK_3

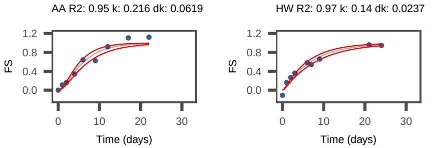

UBA1 – AAVASLLQSVQVPEFTPK_2

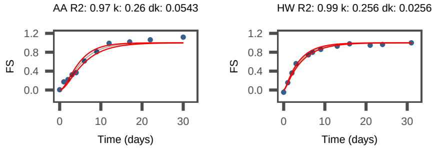

UD12(Non–Unique) – GAGVTLNVLEMTADDLENALK_3

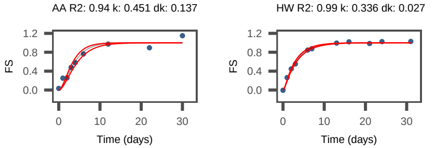

VAPB(Non–Unique) – GPFTDVVTTNLK_2

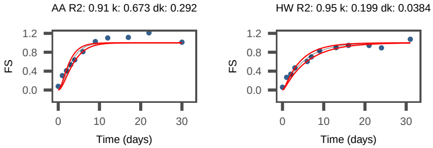

UBA1 – QPAENVNQYLTDSK_2

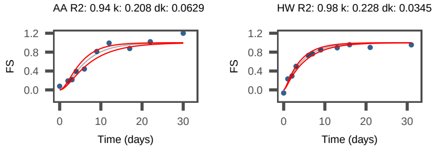

UD17 – IFIDAQWK_2

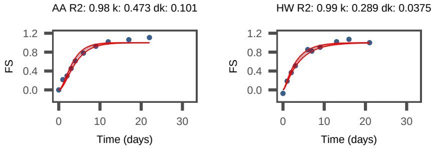

VATA – EILQEEEDLAEIVQLVGK_3

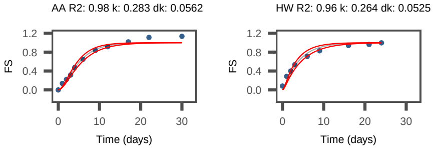

UCRI – GVAGALRPLLQGAVPAASEPPVLDVK_3

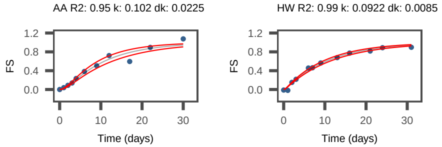

UD17 – PLNFVVK_2

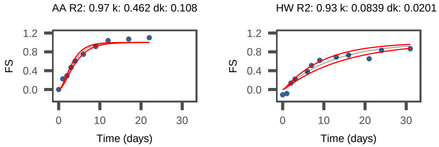

VATA – TGKPLSVELGPGIMGAIFDGIQR_3

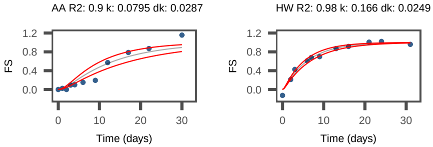

UCRI – KGPAPLNLEVPAYEFTSDDVVVVG_2

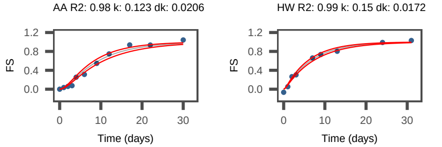

UD17 – SQQEGGILPLLDSPAK_2

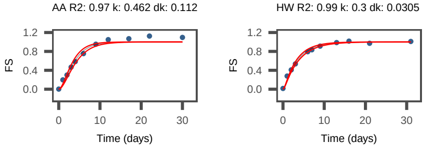

VATA – TVISQSLSK_2

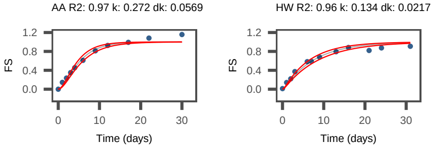

UCRI – NVVSQFVSSMSASADVLAMSK_3

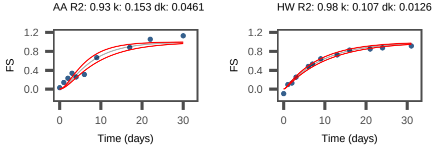

UD3A1(Non–Unique) – NLGVSIQLQTLK_2

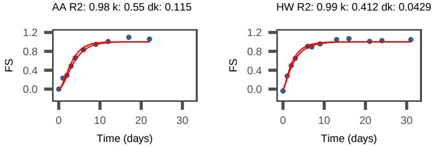

VATA – WEFIPSK_2

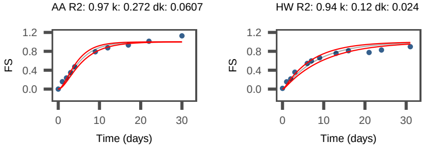

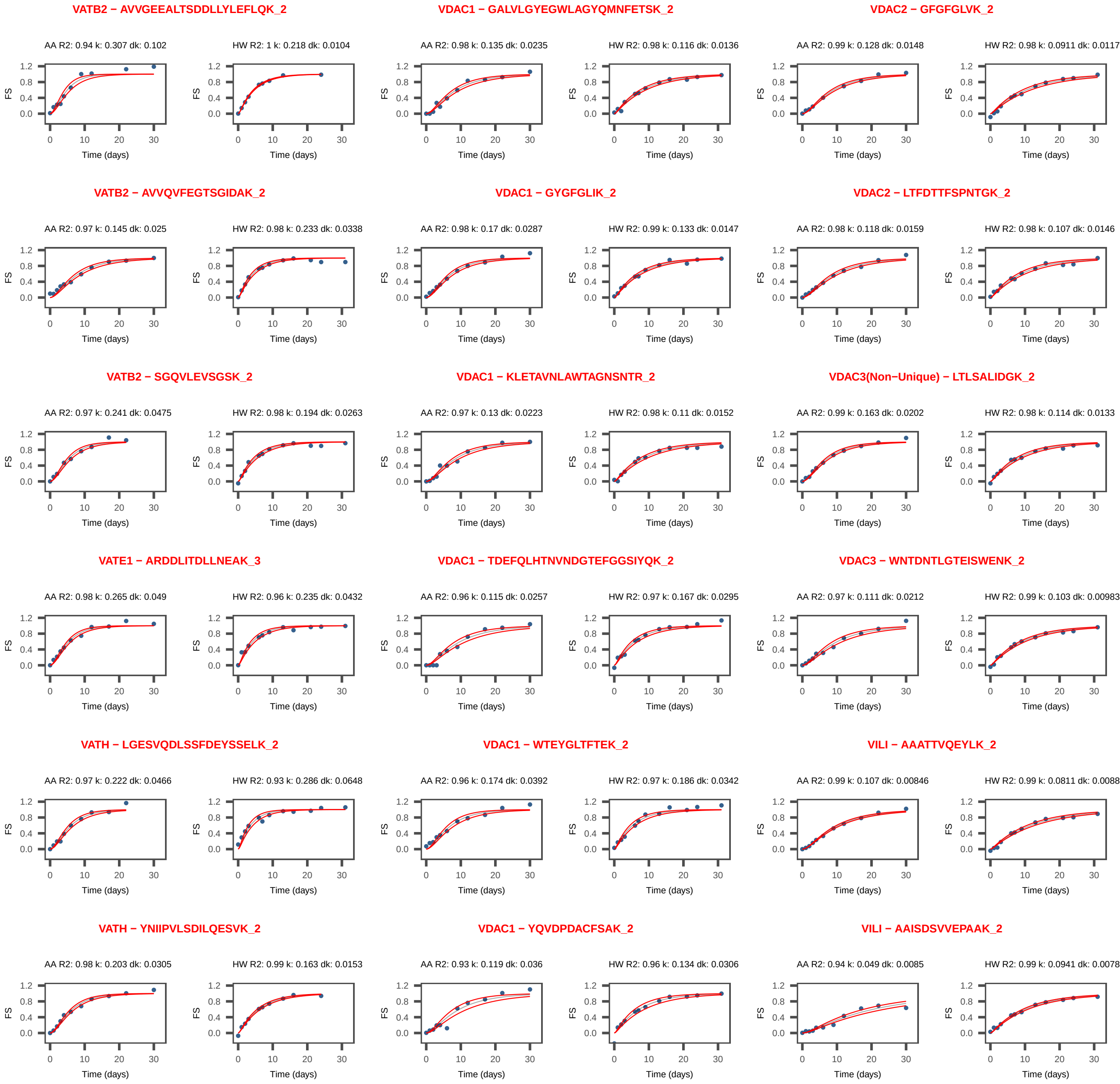

VATB2 − AVVGEEALTSDDLLYLEFLQK_2
AA R2: 0.94 k: 0.307 dk: 0.102
HW R2: 1 k: 0.218 dk: 0.0104
VDAC1 − GALVLGYEGWLAGYQMNFETSK_2
AA R2: 0.98 k: 0.135 dk: 0.0235
HW R2: 0.98 k: 0.116 dk: 0.0136
VDAC2 − GFGFGLVK_2
AA R2: 0.99 k: 0.128 dk: 0.0148
HW R2: 0.98 k: 0.0911 dk: 0.0117
VATB2 − AVVQVFEGTSGIDAK_2
AA R2: 0.97 k: 0.145 dk: 0.025
HW R2: 0.98 k: 0.233 dk: 0.0338
VDAC1 − GYGFGLIK_2
AA R2: 0.98 k: 0.17 dk: 0.0287
HW R2: 0.99 k: 0.133 dk: 0.0147
VDAC2 − LTFDTTFSPNTGK_2
AA R2: 0.98 k: 0.118 dk: 0.0159
HW R2: 0.98 k: 0.107 dk: 0.0146
VATB2 − SGQVLEVSGSK_2
AA R2: 0.97 k: 0.241 dk: 0.0475
HW R2: 0.98 k: 0.194 dk: 0.0263
VDAC1 − KLETAVNLAWTAGNSNTR_2
AA R2: 0.97 k: 0.13 dk: 0.0223
HW R2: 0.98 k: 0.11 dk: 0.0152
VDAC3(Non−Unique) − LTLSALIDGK_2
AA R2: 0.99 k: 0.163 dk: 0.0202
HW R2: 0.98 k: 0.114 dk: 0.0133
VATE1 − ARDDLITDLLNEAK_3
AA R2: 0.98 k: 0.265 dk: 0.049
HW R2: 0.96 k: 0.235 dk: 0.0432
VDAC1 − TDEFQLHTNVNDGTEFGGSIYQK_2
AA R2: 0.96 k: 0.115 dk: 0.0257
HW R2: 0.97 k: 0.167 dk: 0.0295
VDAC3 − WNTDNTLGTEISWENK_2
AA R2: 0.97 k: 0.111 dk: 0.0212
HW R2: 0.99 k: 0.103 dk: 0.00983
VATH − LGESVQDLSSFDEYSSELK_2
AA R2: 0.97 k: 0.222 dk: 0.0466
HW R2: 0.93 k: 0.286 dk: 0.0648
VDAC1 − WTEYGLTFTEK_2
AA R2: 0.96 k: 0.174 dk: 0.0392
HW R2: 0.97 k: 0.186 dk: 0.0342
VILI − AAATTVQEYLK_2
AA R2: 0.99 k: 0.107 dk: 0.00846
HW R2: 0.99 k: 0.0811 dk: 0.0088
VATH − YNIIPVLSDILQESVK_2
AA R2: 0.98 k: 0.203 dk: 0.0305
HW R2: 0.99 k: 0.163 dk: 0.0153
VDAC1 − YQVDPDACFSAK_2
AA R2: 0.93 k: 0.119 dk: 0.036
HW R2: 0.96 k: 0.134 dk: 0.0306
VILI − AAISDSVVEPAAK_2
AA R2: 0.94 k: 0.049 dk: 0.0085
HW R2: 0.99 k: 0.0941 dk: 0.0078
FS
Time (days)

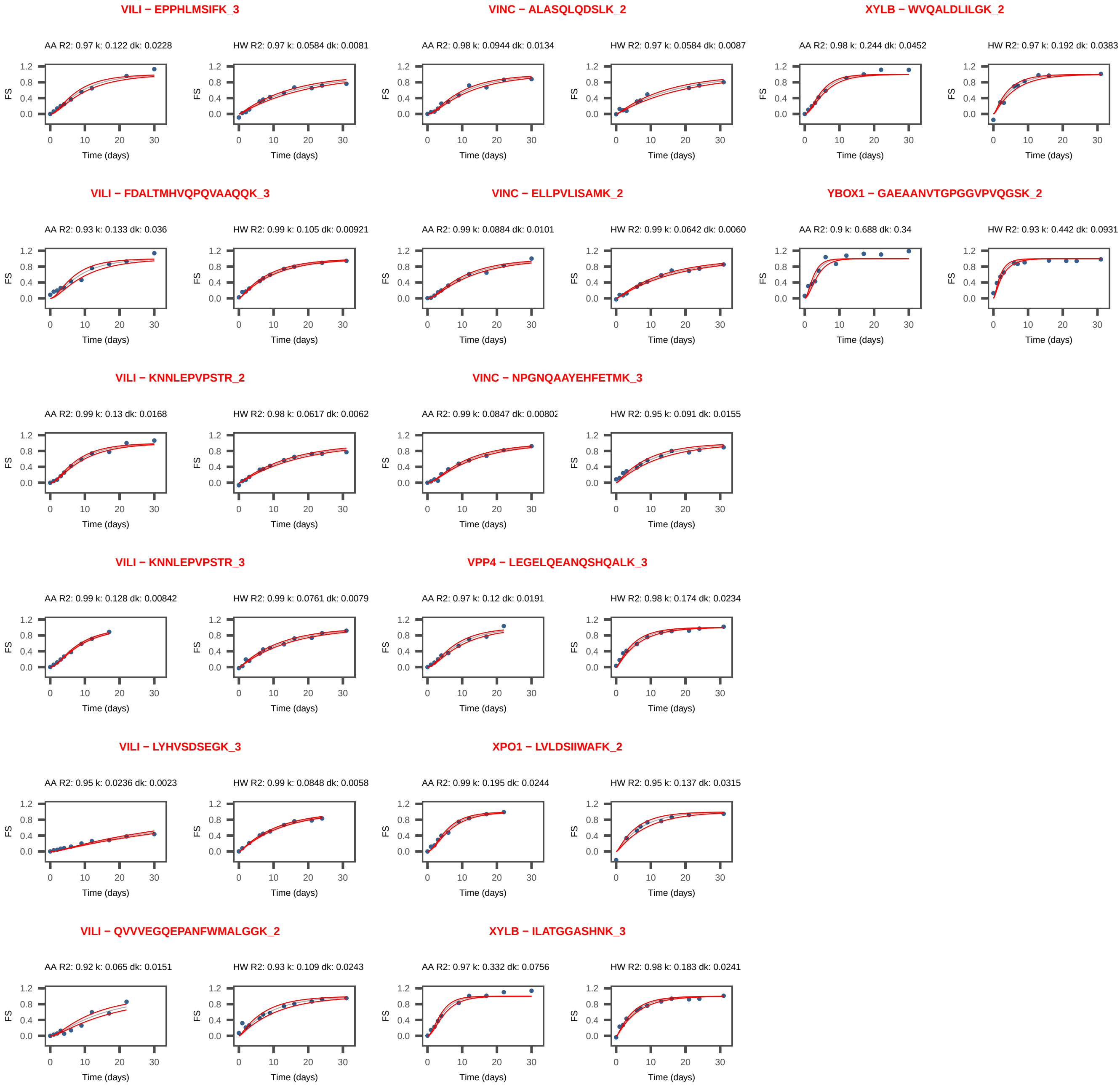
VILI – EPPHLMSIFK_3
AA R2: 0.97 k: 0.122 dk: 0.0228
HW R2: 0.97 k: 0.0584 dk: 0.0081
VINC – ALASQLQDSLK_2
AA R2: 0.98 k: 0.0944 dk: 0.0134
HW R2: 0.97 k: 0.0584 dk: 0.0087
XYLB – WVQALDLILGK_2
AA R2: 0.98 k: 0.244 dk: 0.0452
HW R2: 0.97 k: 0.192 dk: 0.0383
VILI – FDALTMHVQPQVAAQQK_3
AA R2: 0.93 k: 0.133 dk: 0.036
HW R2: 0.99 k: 0.105 dk: 0.00921
VINC – ELLPVLISAMK_2
AA R2: 0.99 k: 0.0884 dk: 0.0101
HW R2: 0.99 k: 0.0642 dk: 0.0060
YBOX1 – GAEAANVTGPGGVPVQGSK_2
AA R2: 0.9 k: 0.688 dk: 0.34
HW R2: 0.93 k: 0.442 dk: 0.0931
VILI – KNNLEPVPSTR_2
AA R2: 0.99 k: 0.13 dk: 0.0168
HW R2: 0.98 k: 0.0617 dk: 0.0062
VINC – NPGNQAAYEHFETMK_3
AA R2: 0.99 k: 0.0847 dk: 0.00802
HW R2: 0.95 k: 0.091 dk: 0.0155
VILI – KNNLEPVPSTR_3
AA R2: 0.99 k: 0.128 dk: 0.00842
HW R2: 0.99 k: 0.0761 dk: 0.0079
VPP4 – LEGELQEANQSHQALK_3
AA R2: 0.97 k: 0.12 dk: 0.0191
HW R2: 0.98 k: 0.174 dk: 0.0234
VILI – LYHVSDSEGK_3
AA R2: 0.95 k: 0.0236 dk: 0.0023
HW R2: 0.99 k: 0.0848 dk: 0.0058
XPO1 – LVLDSIIWAFK_2
AA R2: 0.99 k: 0.195 dk: 0.0244
HW R2: 0.95 k: 0.137 dk: 0.0315
VILI – QVVVEGQEPANFWMALGGK_2
AA R2: 0.92 k: 0.065 dk: 0.0151
HW R2: 0.93 k: 0.109 dk: 0.0243
XYLB – ILATGGASHNK_3
AA R2: 0.97 k: 0.332 dk: 0.0756
HW R2: 0.98 k: 0.183 dk: 0.0241
FS
Time (days)
