# Supplementary material for: Harmonizing Labeling and Analytical Strategies to Obtain Protein Turnover Rates in Intact Adult Animals
Source: Mol Cell Proteomics. 2022 May 28;21(7):100252. doi: 10.1016/j.mcpro.2022.100252 (PMC9249856; doi:10.1016/j.mcpro.2022.100252)

1433B − TAFDEAIAELDTLNEESYK_2

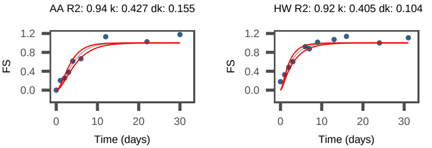

1433G − TAFDDAIAELDTLNEDSYK_2

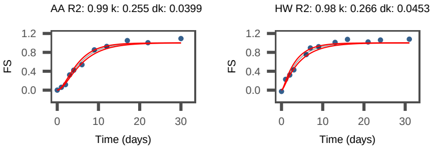

1433Z − TAFDEAIAELDTLSEESYK_2

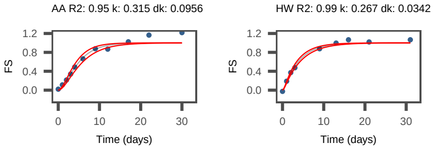

1433E − AAFDDAIAELDTLSEESYK_3

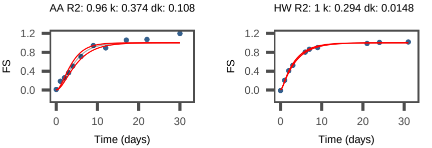

1433S(Non−Unique) − NLLSVAYK_2

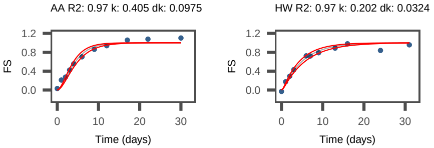

1433Z − TAFDEAIAELDTLSEESYK_3

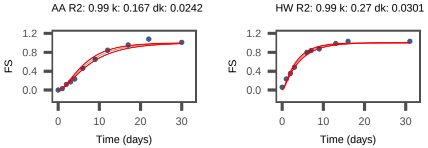

1433E − LICCDILDVLDK_2

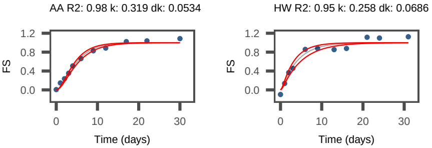

1433T − SICTTVLELLDK_2

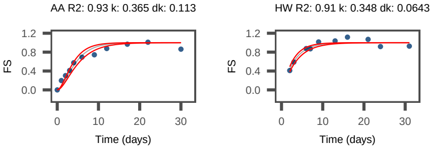

1433Z − YDDMAACMK_2

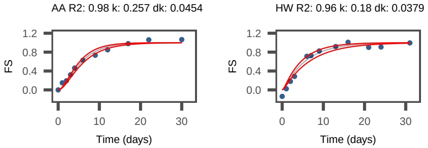

1433E − YDEMVESMK_2

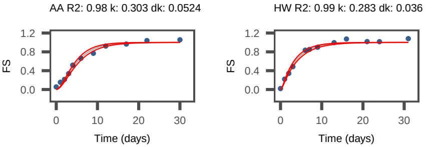

1433T − YDDMATCMK_2

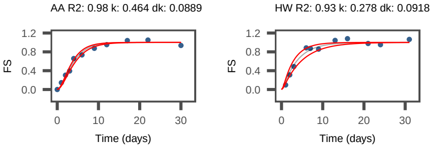

2AAA − DNTIEHLLPLFLAQLK_3

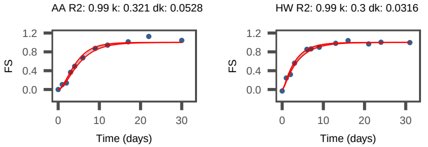

1433G − AYSEAHEISK_2

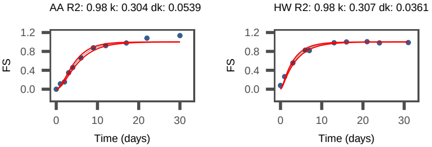

1433Z − FLIPNASQPESK_2

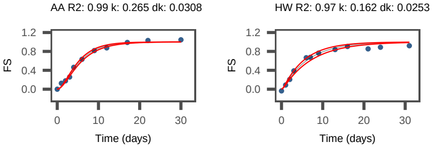

2AAA − SALASVIMGLSPILGK_2

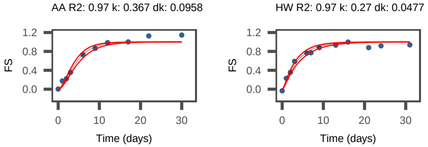

1433G − AYSEAHEISK_3

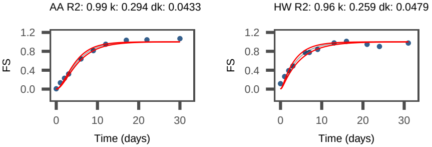

1433Z − GIVDQSQQAYQEAFEISK_2

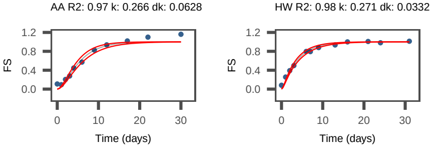

3HAO − YYVGDTEDVLFEK_2

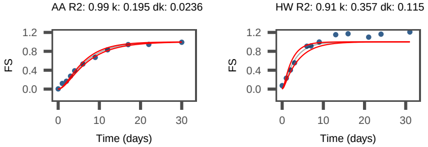

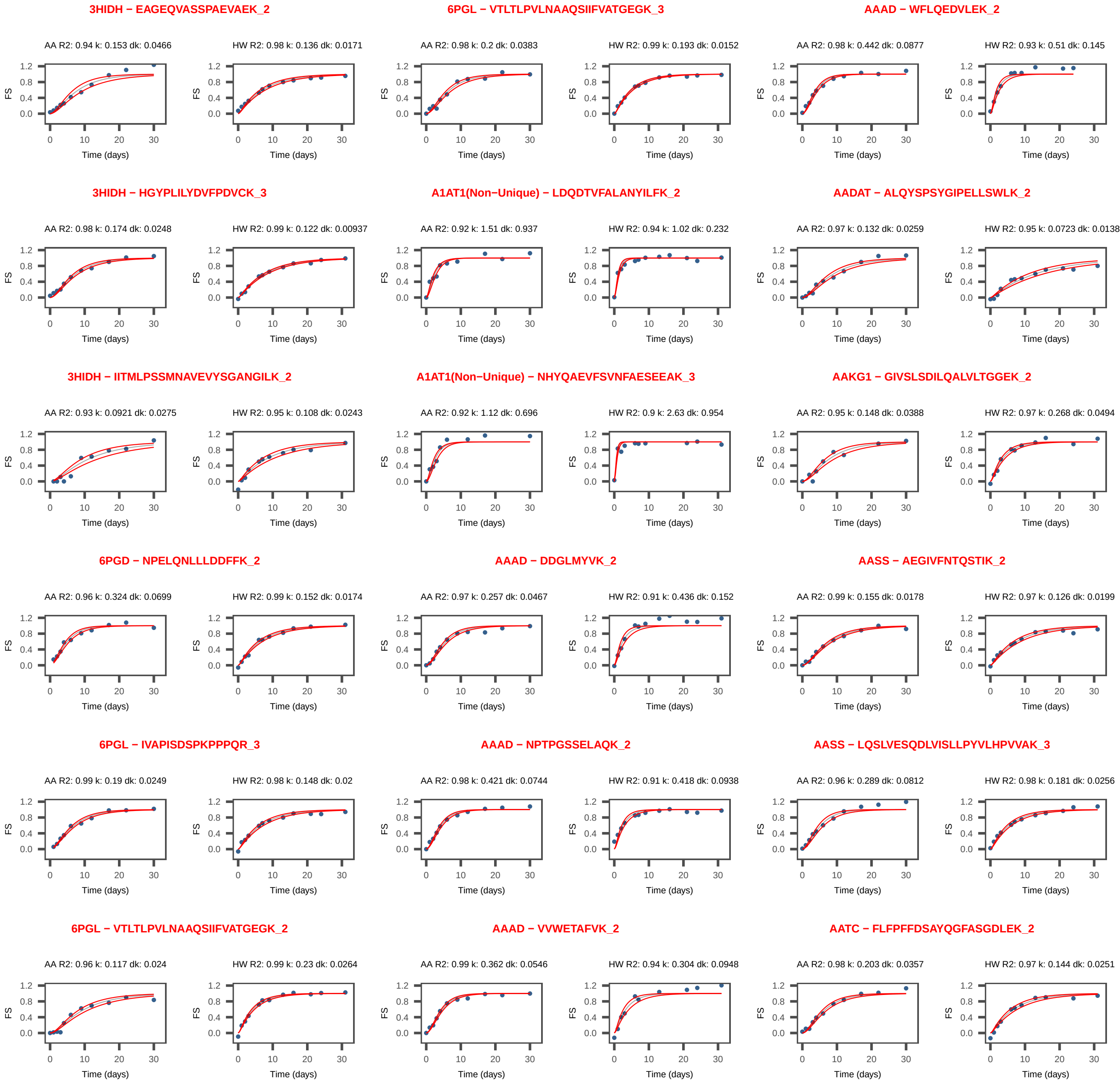
3HIDH − EAGEQVASSPAEVAEK_2
AA R2: 0.94 k: 0.153 dk: 0.0466
HW R2: 0.98 k: 0.136 dk: 0.0171
6PGL − VTLTLPVLNAAQSIIFVATGEGK_3
AA R2: 0.98 k: 0.2 dk: 0.0383
HW R2: 0.99 k: 0.193 dk: 0.0152
AAAD − WFLQEDVLEK_2
AA R2: 0.98 k: 0.442 dk: 0.0877
HW R2: 0.93 k: 0.51 dk: 0.145
3HIDH − HGYPLILYDVFPDVCK_3
AA R2: 0.98 k: 0.174 dk: 0.0248
HW R2: 0.99 k: 0.122 dk: 0.00937
A1AT1(Non−Unique) − LDQDTVFALANYILFK_2
AA R2: 0.92 k: 1.51 dk: 0.937
HW R2: 0.94 k: 1.02 dk: 0.232
AADAT − ALQYSPSYGIPELLSWLK_2
AA R2: 0.97 k: 0.132 dk: 0.0259
HW R2: 0.95 k: 0.0723 dk: 0.0138
3HIDH − IITMLPSSMNAVEVYSGANGILK_2
AA R2: 0.93 k: 0.0921 dk: 0.0275
HW R2: 0.95 k: 0.108 dk: 0.0243
A1AT1(Non−Unique) − NHYQAEVFSVNFAESEEAK_3
AA R2: 0.92 k: 1.12 dk: 0.696
HW R2: 0.9 k: 2.63 dk: 0.954
AAKG1 − GIVSLSDILQALVLTGGEK_2
AA R2: 0.95 k: 0.148 dk: 0.0388
HW R2: 0.97 k: 0.268 dk: 0.0494
6PGD − NPELQNLLLDDFFK_2
AA R2: 0.96 k: 0.324 dk: 0.0699
HW R2: 0.99 k: 0.152 dk: 0.0174
AAAD − DDGLMYVK_2
AA R2: 0.97 k: 0.257 dk: 0.0467
HW R2: 0.91 k: 0.436 dk: 0.152
AASS − AEGIVFNTQSTIK_2
AA R2: 0.99 k: 0.155 dk: 0.0178
HW R2: 0.97 k: 0.126 dk: 0.0199
6PGL − IVAPISDSPKPPPQR_3
AA R2: 0.99 k: 0.19 dk: 0.0249
HW R2: 0.98 k: 0.148 dk: 0.02
AAAD − NPTPGSSELAQK_2
AA R2: 0.98 k: 0.421 dk: 0.0744
HW R2: 0.91 k: 0.418 dk: 0.0938
AASS − LQSLVESQDLVISLLPYVLHPVVAK_3
AA R2: 0.96 k: 0.289 dk: 0.0812
HW R2: 0.98 k: 0.181 dk: 0.0256
6PGL − VTLTLPVLNAAQSIIFVATGEGK_2
AA R2: 0.96 k: 0.117 dk: 0.024
HW R2: 0.99 k: 0.23 dk: 0.0264
AAAD − VVWETAFVK_2
AA R2: 0.99 k: 0.362 dk: 0.0546
HW R2: 0.94 k: 0.304 dk: 0.0948
AATC − FLFPFFDSAYQGFASGDLEK_2
AA R2: 0.98 k: 0.203 dk: 0.0357
HW R2: 0.97 k: 0.144 dk: 0.0251
FS
Time (days)

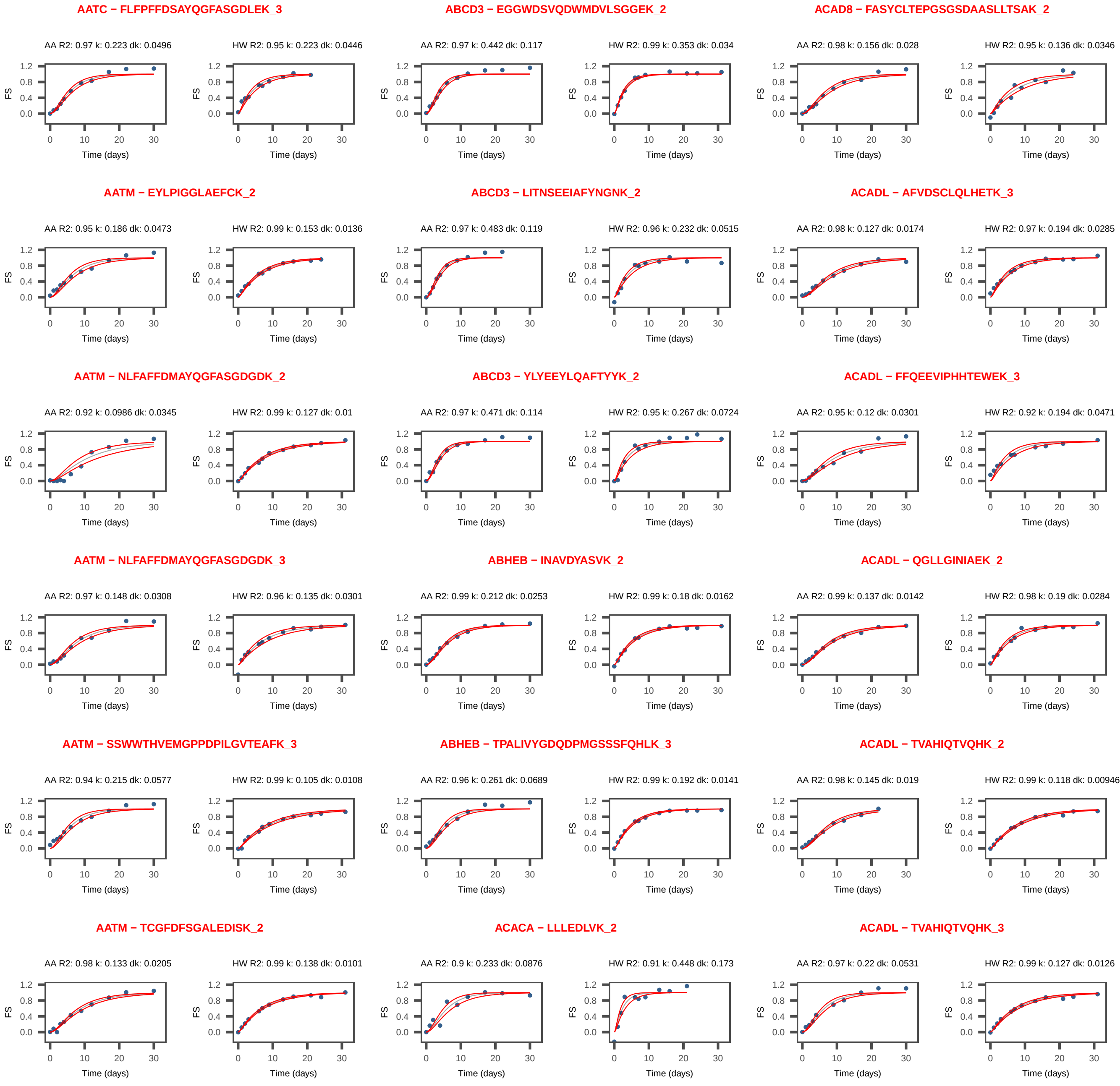
AATC – FLFPFFDSAYQGFASGDLEK_3
AA R2: 0.97 k: 0.223 dk: 0.0496
HW R2: 0.95 k: 0.223 dk: 0.0446
ABCD3 – EGGWDSVQDWMDVLSGGEK_2
AA R2: 0.97 k: 0.442 dk: 0.117
HW R2: 0.99 k: 0.353 dk: 0.034
ACAD8 – FASYCLTEPGSGSDAASLLTSAK_2
AA R2: 0.98 k: 0.156 dk: 0.028
HW R2: 0.95 k: 0.136 dk: 0.0346
AATM – EYLPIGGLAEFCK_2
AA R2: 0.95 k: 0.186 dk: 0.0473
HW R2: 0.99 k: 0.153 dk: 0.0136
ABCD3 – LITNSEEIAFYNGNK_2
AA R2: 0.97 k: 0.483 dk: 0.119
HW R2: 0.96 k: 0.232 dk: 0.0515
ACADL – AFVDSCLQLHETK_3
AA R2: 0.98 k: 0.127 dk: 0.0174
HW R2: 0.97 k: 0.194 dk: 0.0285
AATM – NLFAFFDMAYQGFASGDGDK_2
AA R2: 0.92 k: 0.0986 dk: 0.0345
HW R2: 0.99 k: 0.127 dk: 0.01
ABCD3 – YLYEEYLQAFTYYK_2
AA R2: 0.97 k: 0.471 dk: 0.114
HW R2: 0.95 k: 0.267 dk: 0.0724
ACADL – FFQEEVIPHHTEWEK_3
AA R2: 0.95 k: 0.12 dk: 0.0301
HW R2: 0.92 k: 0.194 dk: 0.0471
AATM – NLFAFFDMAYQGFASGDGDK_3
AA R2: 0.97 k: 0.148 dk: 0.0308
HW R2: 0.96 k: 0.135 dk: 0.0301
ABHEB – INAVDYASVK_2
AA R2: 0.99 k: 0.212 dk: 0.0253
HW R2: 0.99 k: 0.18 dk: 0.0162
ACADL – QGLLGINIAEK_2
AA R2: 0.99 k: 0.137 dk: 0.0142
HW R2: 0.98 k: 0.19 dk: 0.0284
AATM – SSWWTHVEMGPPDPILGVTEAFK_3
AA R2: 0.94 k: 0.215 dk: 0.0577
HW R2: 0.99 k: 0.105 dk: 0.0108
ABHEB – TPALIVYGDQDPMGSSSFQHLK_3
AA R2: 0.96 k: 0.261 dk: 0.0689
HW R2: 0.99 k: 0.192 dk: 0.0141
ACADL – TVAHIQTVQHK_2
AA R2: 0.98 k: 0.145 dk: 0.019
HW R2: 0.99 k: 0.118 dk: 0.00946
AATM – TCGFDFSGALEDISK_2
AA R2: 0.98 k: 0.133 dk: 0.0205
HW R2: 0.99 k: 0.138 dk: 0.0101
ACACA – LLLEDLVK_2
AA R2: 0.9 k: 0.233 dk: 0.0876
HW R2: 0.91 k: 0.448 dk: 0.173
ACADL – TVAHIQTVQHK_3
AA R2: 0.97 k: 0.22 dk: 0.0531
HW R2: 0.99 k: 0.127 dk: 0.0126
FS
Time (days)

ACADM − AFTGFIVEADTPGIHIGK_3

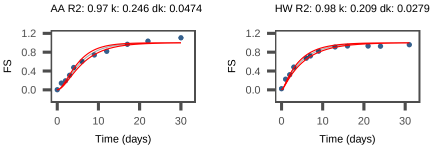

ACADV − EATQAVLDKPETLSSDASTR_2

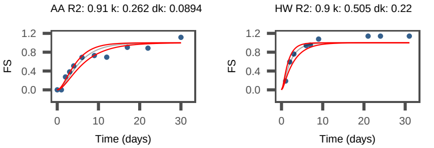

ACD11 − SGQSNPTFFLQK_2

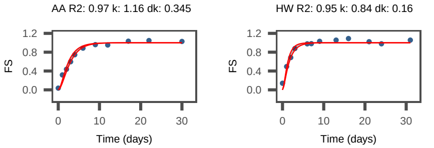

ACADM − ENVLIGEGAGFK_2

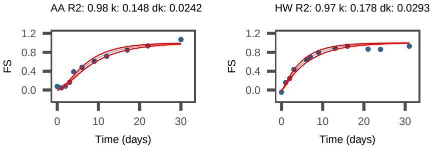

ACADV − SFAVGMFK_2

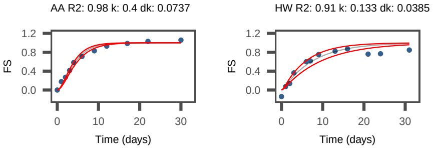

ACDSB − VPETNILGK_2

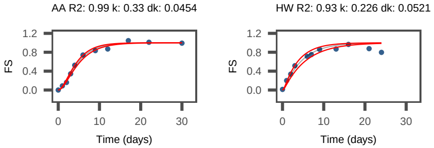

ACADM − QEPGLGFSFELTEQQK_2

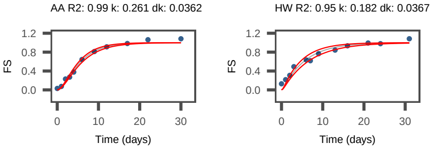

ACADV − SLSEGYPTAQHEK_3

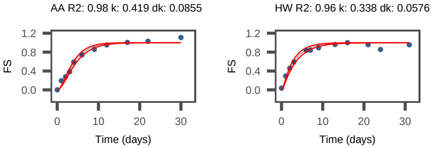

ACLY − EILIPVFK_2

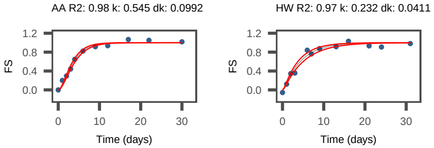

ACADM − SGEYPFPLIK_2

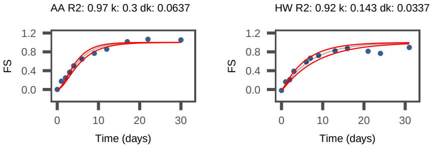

ACBP − QATVGDVNTDRPGLLDLK_2

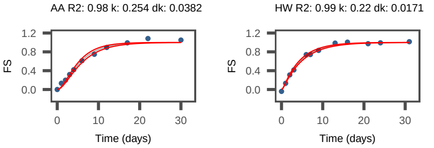

ACLY − LGLVGVNLSLDGVK_2

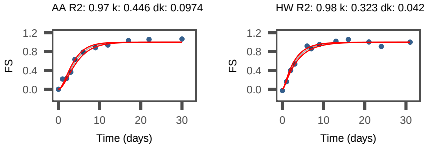

ACADS − EHLFPTAQVK_2

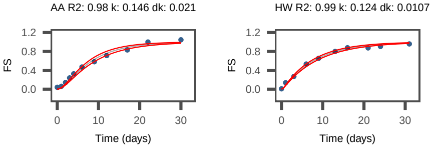

ACBP − TQPTDEEMLFIYSHFK_2

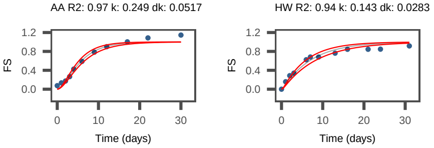

ACOC − DFESCLGAK_2

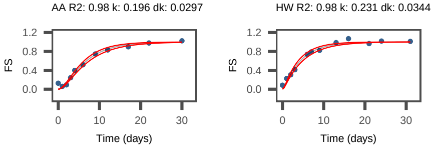

ACADS − IGIASQALGIAQASLDCAVK_3

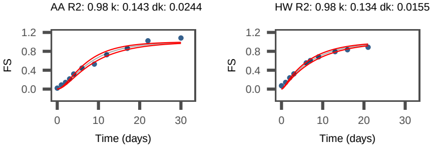

ACBP − TQPTDEEMLFIYSHFK_3

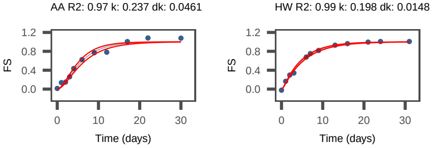

ACOC − GPFLLGIK_2

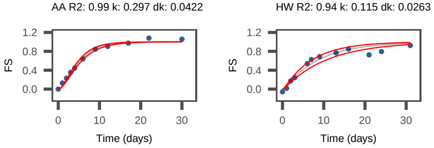

ACOC − NIEVPFKPAR_3

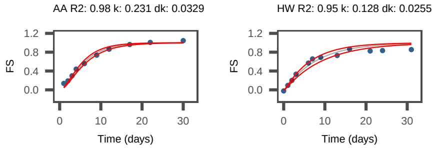

ACON − LNRPLTLSEK_2

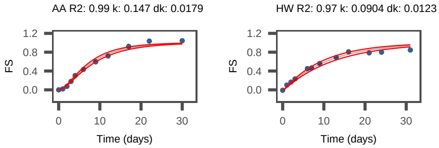

ACOX1 − ASEAHCHYVTVK_2

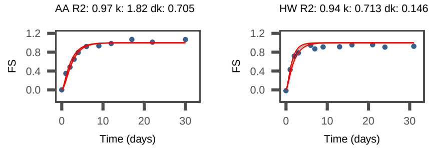

ACOC − SPPFFESLTLDLQPPK_3

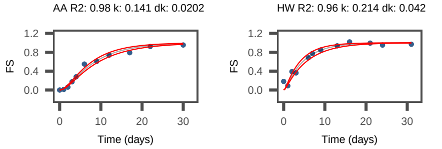

ACON(Non−Unique) − LTIQGIK_2

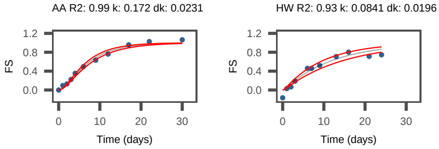

ACOX1 − ASEAHCHYVTVK_3

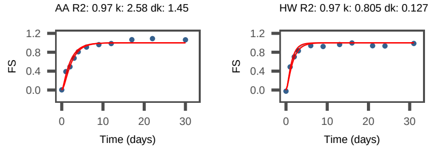

ACOC − YQQAGLPLIVLAGK_2

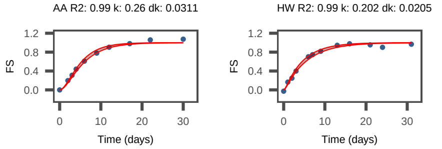

ACON − SDFDPGQDTYQHPPK_3

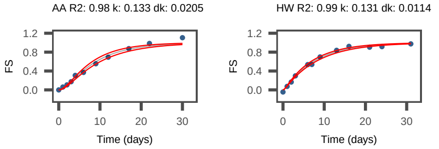

ACOX1 − EFGIADPEEIMWFK_2

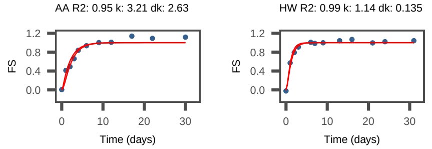

ACON − CTTDHISAAGPWLK_3

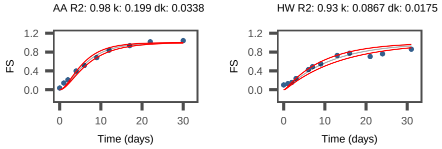

ACON − VAGILTVK_2

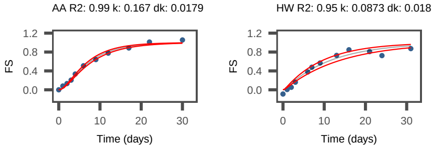

ACOX1 − FGYEEMDNGYLK_2

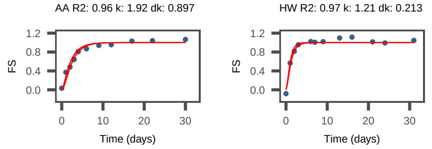

ACON − DINQEVYNFLATAGAK_3

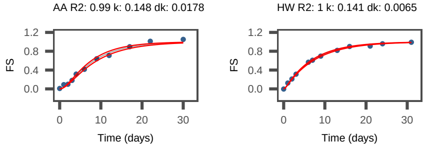

ACON − VAVPSTIHCDHLIEAQVGGEK_3

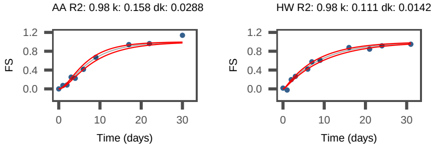

ACOX1 − INESIGQGDLSELPELHALTAGLK_3

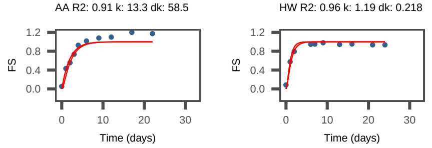

ACON − FNPETDFLTGK_2

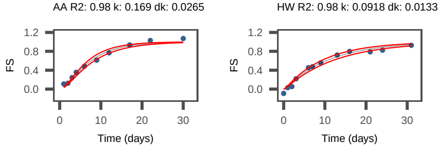

ACOT1(Non−Unique) − SDTTFLFLVGQDDHNWK_3

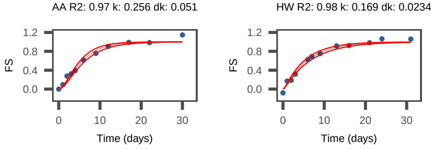

ACOX1 − NLCLLYSLYGISQK_3

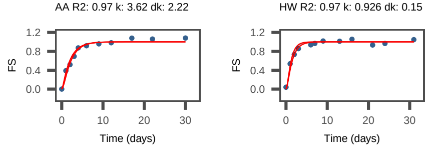

ACOX1 − TEVHESYYK_2

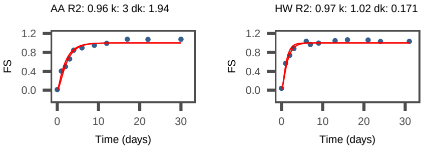

ACSA − IGPIATPDYIQNAPGLPK_2

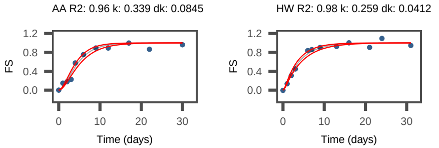

ACSF2 − TQQYYDILK_2

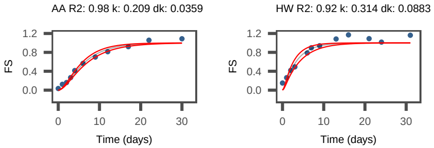

ACOX1 − TEVHESYYK_3

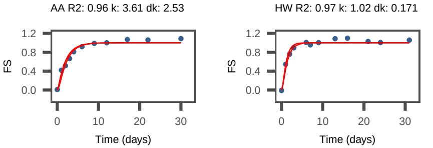

ACSF2 − FLSCYDPINIQFTSGTTGNPK_2

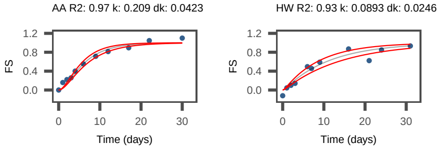

ACSL1(Non−Unique) − DGWLHTGDIGK_3

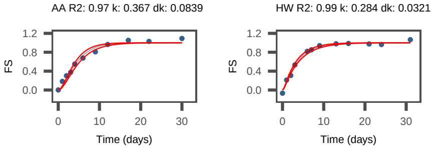

ACOX1 − TQEFILNSPTVTSIK_2

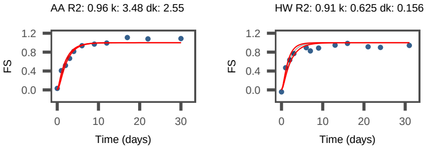

ACSF2 − HPQVQEAQVVGVK_2

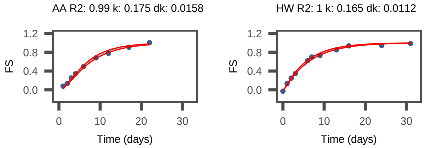

ACSL1 − EVAELAECIGSGLIQK_3

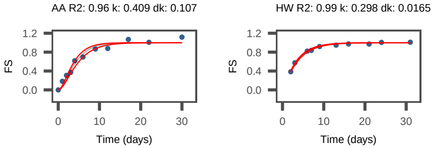

ACOX1 − YDGNVYENLFEWAK_2

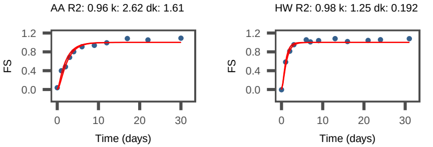

ACSF2 − HPQVQEAQVVGVK_3

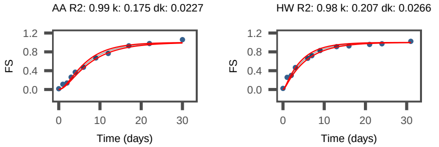

ACSL1(Non−Unique) − LAQGEYIAPEK_2

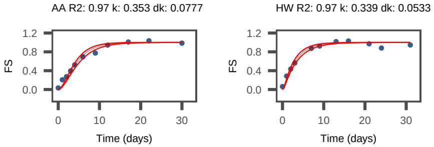

ACPM − SDAPPLTLDGIK_2

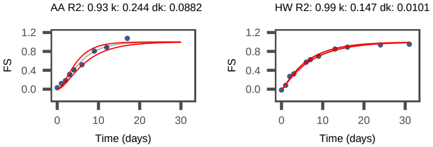

ACSF2 − HPQVQEAQVVGVKDER_3

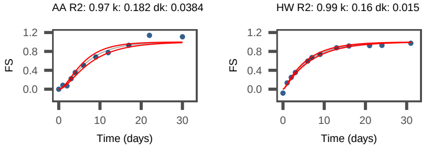

ACSL1 − LIAVVVPDVESLPSWAQK_2

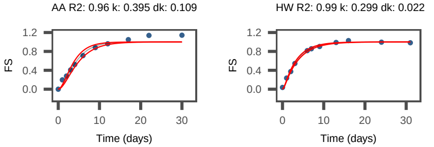

ACSA − AELGMNDSPSQSPPVK_2

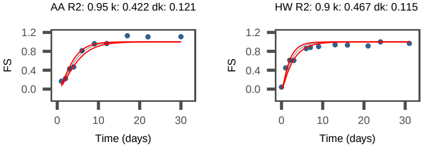

ACSF2 − LPDLTTVISVDAPLPGTLLLDDIVAAGGK_3

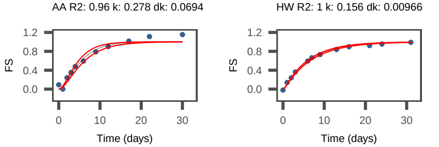

ACSL1 − SAVLEDDKLLVYYYDDVR_3

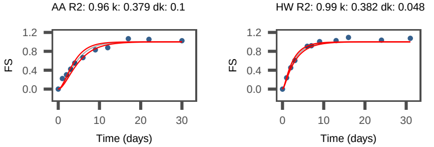

ACSL1 − WLLDFASK_2
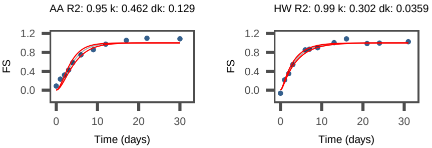

ACSM1 − AIVTTASLVPEVESVASECPDLK_2
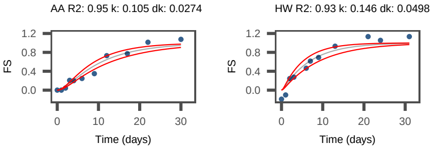

ACTB(Non−Unique) − EITALAPSTMK_2
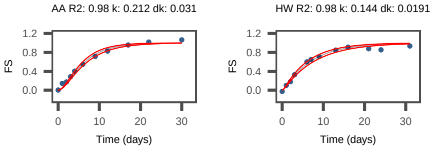

ACSL5 − ADIPVVICDTPQK_2
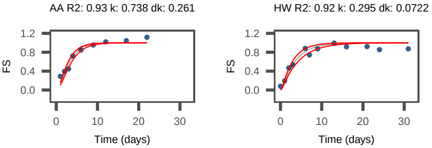

ACSM1 − VIVEVLFK_2
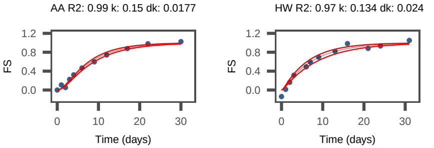

ACTB(Non−Unique) − HQGVM[15.9949]VGMGQK_2
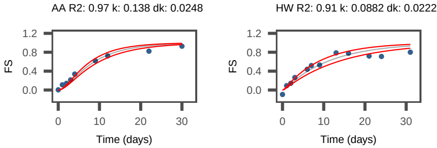

ACSL5 − CGVEMLSLHDAENIGK_3
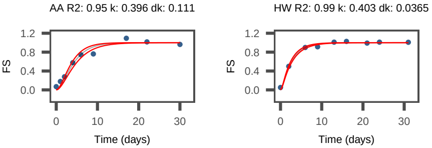

ACSS3 − KVEYIPLLEEALR_3
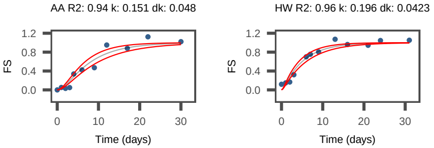

ACTB(Non−Unique) − HQGVMVGMGQK_2
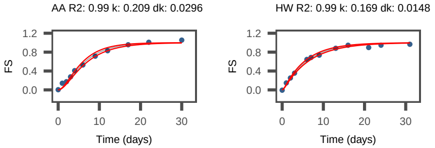

ACSL5 − SFLIGVVVPDPDSLPSFAAK_2
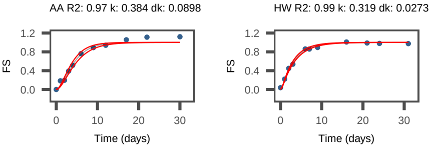

ACTA(Non−Unique) − YPIEHGIITNWDDMEK_2
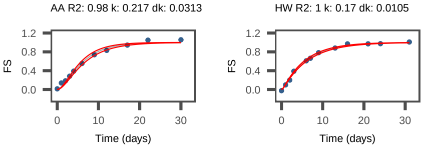

ACTB(Non−Unique) − HQGVMVGMGQK_3
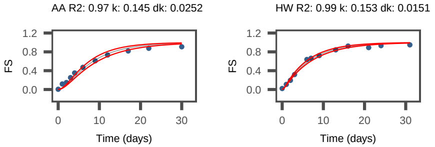

ACSM1 − AFIVLNPEFLSHDQEQLIK_3
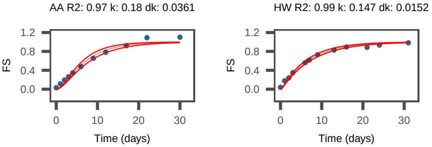

ACTA(Non−Unique) − YPIEHGIITNWDDMEK_3
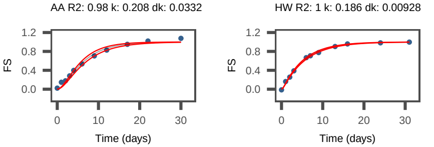

ACTB(Non−Unique) − KDLYANTVLSGGTTMYPGIADR_2
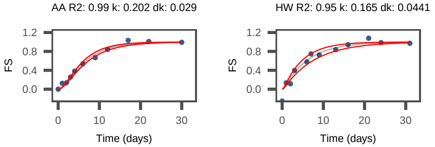

ACSM1 − AILPFDLQIIDEK_2
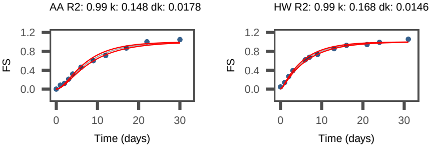

ACTB(Non−Unique) − DSYVGDEAQSK_2
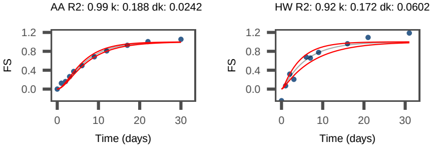

ACTB(Non−Unique) − KDLYANTVLSGGTTMYPGIADR_3
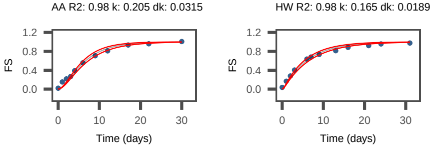

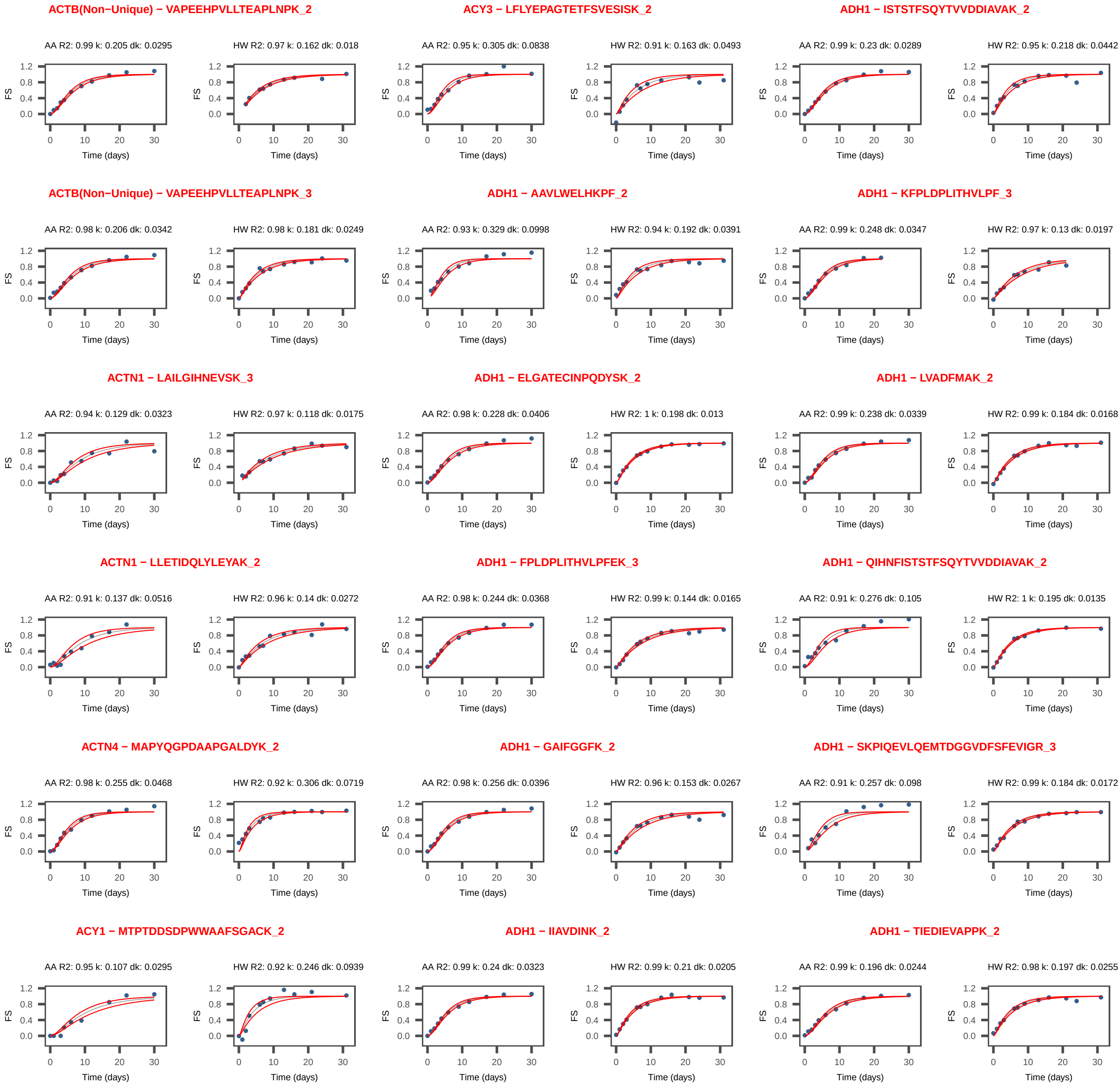
ACTB(Non−Unique) − VAPEEHPVLLTEAPLNPK_2
AA R2: 0.99 k: 0.205 dk: 0.0295
HW R2: 0.97 k: 0.162 dk: 0.018
ACY3 − LFLYEPAGTETFSVESISK_2
AA R2: 0.95 k: 0.305 dk: 0.0838
HW R2: 0.91 k: 0.163 dk: 0.0493
ADH1 − ISTSTFSQYTVVDDIAVAK_2
AA R2: 0.99 k: 0.23 dk: 0.0289
HW R2: 0.95 k: 0.218 dk: 0.0442
ACTB(Non−Unique) − VAPEEHPVLLTEAPLNPK_3
AA R2: 0.98 k: 0.206 dk: 0.0342
HW R2: 0.98 k: 0.181 dk: 0.0249
ADH1 − AAVLWELHKPF_2
AA R2: 0.93 k: 0.329 dk: 0.0998
HW R2: 0.94 k: 0.192 dk: 0.0391
ADH1 − KFPLDPLITHVLPF_3
AA R2: 0.99 k: 0.248 dk: 0.0347
HW R2: 0.97 k: 0.13 dk: 0.0197
ACTN1 − LAILGIHNEVSK_3
AA R2: 0.94 k: 0.129 dk: 0.0323
HW R2: 0.97 k: 0.118 dk: 0.0175
ADH1 − ELGATECINPQDYSK_2
AA R2: 0.98 k: 0.228 dk: 0.0406
HW R2: 1 k: 0.198 dk: 0.013
ADH1 − LVADFMAK_2
AA R2: 0.99 k: 0.238 dk: 0.0339
HW R2: 0.99 k: 0.184 dk: 0.0168
ACTN1 − LLETIDQLYLEYAK_2
AA R2: 0.91 k: 0.137 dk: 0.0516
HW R2: 0.96 k: 0.14 dk: 0.0272
ADH1 − FPLDPLITHVLPFEK_3
AA R2: 0.98 k: 0.244 dk: 0.0368
HW R2: 0.99 k: 0.144 dk: 0.0165
ADH1 − QIHNFISTSTFSQYTVVDDIAVAK_2
AA R2: 0.91 k: 0.276 dk: 0.105
HW R2: 1 k: 0.195 dk: 0.0135
ACTN4 − MAPYQGPDAAPGALDYK_2
AA R2: 0.98 k: 0.255 dk: 0.0468
HW R2: 0.92 k: 0.306 dk: 0.0719
ADH1 − GAIFGGFK_2
AA R2: 0.98 k: 0.256 dk: 0.0396
HW R2: 0.96 k: 0.153 dk: 0.0267
ADH1 − SKPIQEVLQEMTDGGVDFSFEVIGR_3
AA R2: 0.91 k: 0.257 dk: 0.098
HW R2: 0.99 k: 0.184 dk: 0.0172
ACY1 − MTPTDDSDPWWAAFSGACK_2
AA R2: 0.95 k: 0.107 dk: 0.0295
HW R2: 0.92 k: 0.246 dk: 0.0939
ADH1 − IIAVDINK_2
AA R2: 0.99 k: 0.24 dk: 0.0323
HW R2: 0.99 k: 0.21 dk: 0.0205
ADH1 − TIEDIEVAPPK_2
AA R2: 0.99 k: 0.196 dk: 0.0244
HW R2: 0.98 k: 0.197 dk: 0.0255

ADH1 − TVVDDIAVAK_2

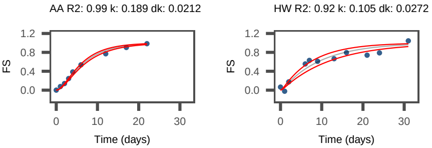

ADK − ELFDELVK_2

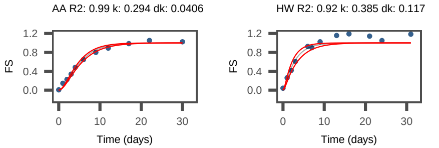

ADK − VEYHAGGSTQNSMK_2

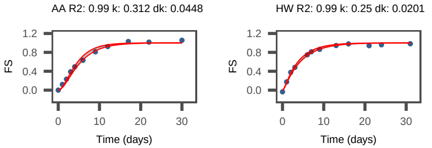

ADH1 − VTPGSTCAVFGLGGVGLSVIIGCK_3

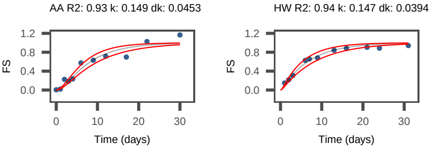

ADK − LSQLVSDKPLTECIR_3

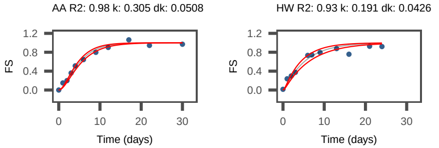

ADT1(Non−Unique) − LLLQVQHASK_2

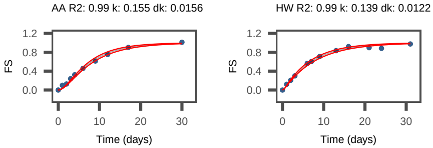

ADHX − EFGASECISPQDFSK_2

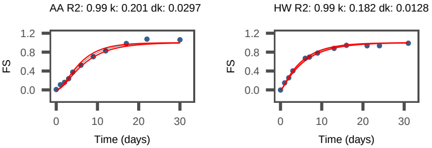

ADK − SLVANLAAANCYK_2

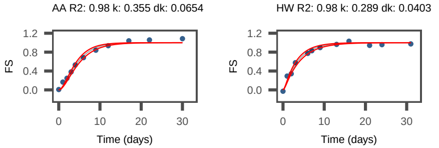

AGT2 − LGSHFWGFQTHDVLPDIVTMAK_3

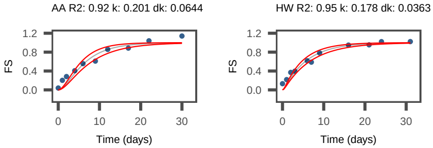

ADHX − VCLLGCGISTGYGAAVNTAK_2

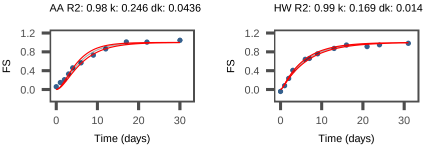

ADK − TGCTFPEKPDFH_3

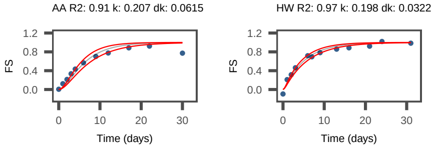

AGT2 − LGSHFWGFQTHDVLPDIVTMAK_4

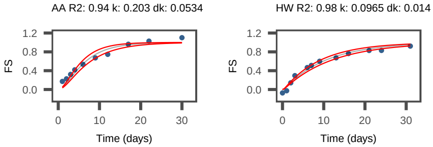

ADHX − YIPQCGECK_2

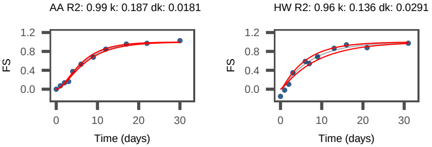

ADK − VAQWLIQEPHK_2

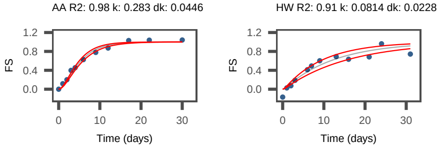

AIFM1 − ELWFSDDPNVTK_2

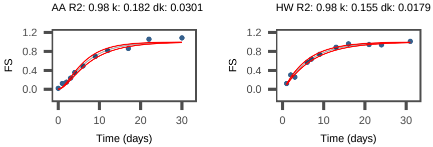

ADK − AATFFGCIGIDK_2

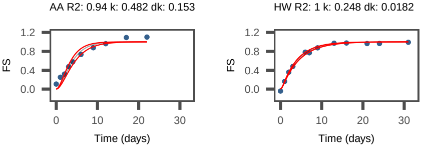

ADK − VAQWLIQEPHK_3

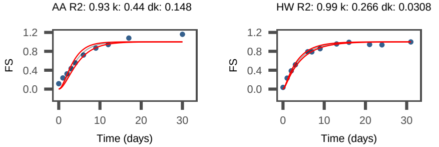

AIMP2 − VLSTVHTHSSVK_2

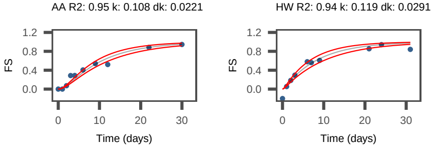

AK1A1 – ALEVLVAK_2

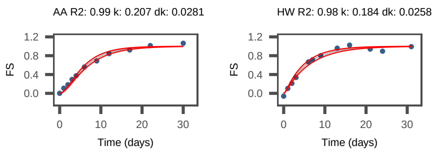

AK1CD – SGDNDFPVNEQGK_2

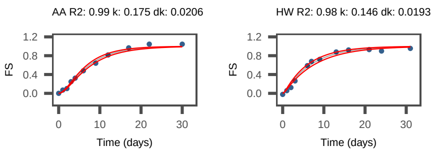

AL1A1 – IFINNEWHNSVSGK_2

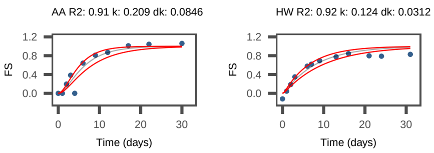

AK1A1 – HIDCASVYGNETEIGEALK_2

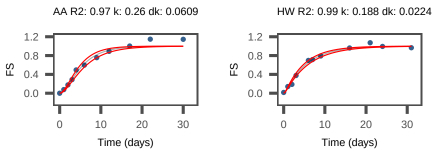

AK1CD – YKPVCNQVECHLYLNQR_3

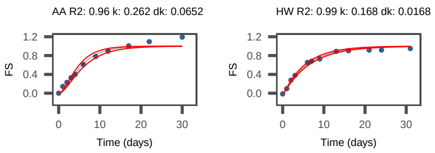

AL1A1 – PAEQTPLTALHLASLIK_2

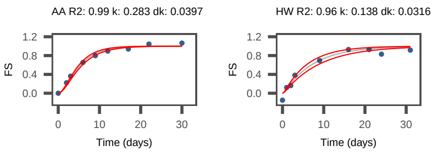

AK1A1 – ILQNIQVFDFTFSPEEMK_2

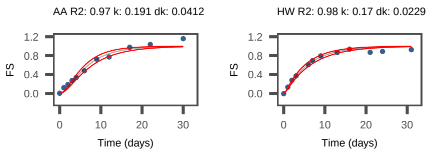

AK1CD – YKPVCNQVECHLYLNQR_4

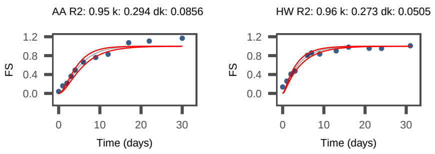

AL1A1 – PAEQTPLTALHLASLIK_3

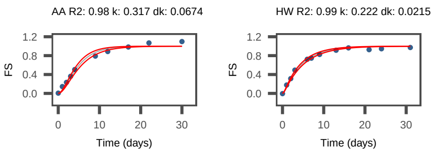

AK1A1 – YIVPMITVDGK_2

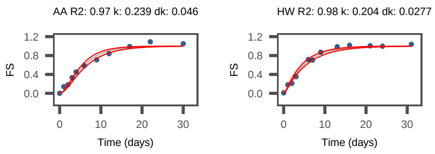

AK1D1 – LDYIDLYIIELPMAFK_2

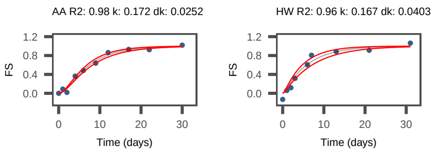

AL1A1 – VAFTGSTQVGK_2

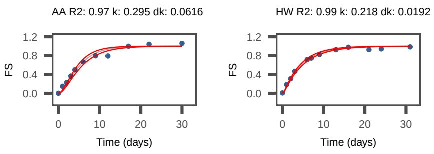

AK1CD – ENLQVFGFQLSPEDMK_2

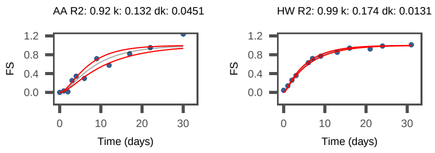

AK1D1 – TNLCATWEALEACK_2

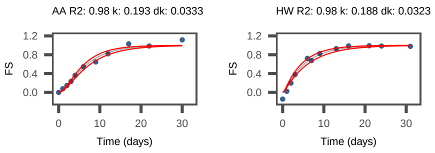

AL1A1 – VFANAYLSDLGGCIK_3

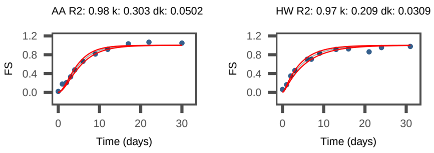

AK1CD – GIVPLAQSFK_2

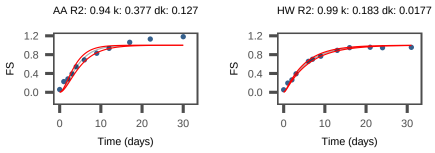

AL1A1 – EAGFPPGVVNIVPGYGPTAGAAISSHMDVDK_3

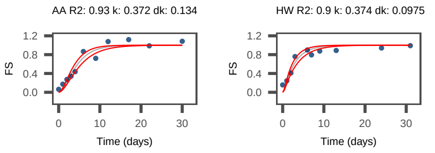

AL1A1 – YCAGWADK_2

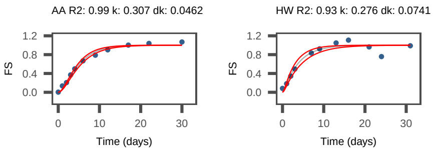

AL1A7(Non−Unique) − EEIFGPVQQIMK_2

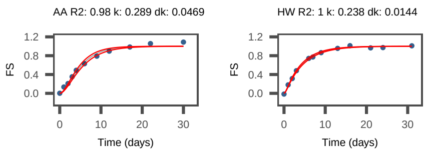

AL1A7 − TVAMQISQK_2

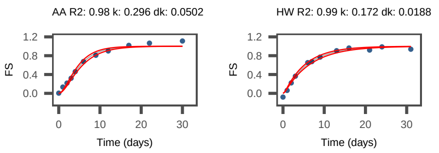

AL1L1 − FAELTLK_2

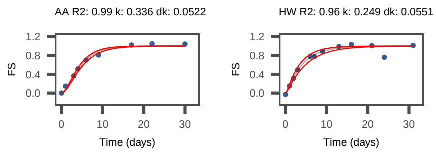

AL1A7(Non−Unique) − ELGEHGLYEYTELK_2

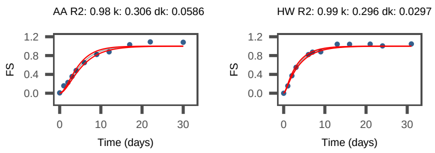

AL1A7(Non−Unique) − VTLELGGK_2

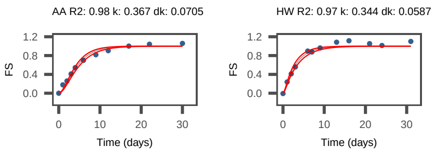

AL1L1 − GASAINWTLIHGDK_3

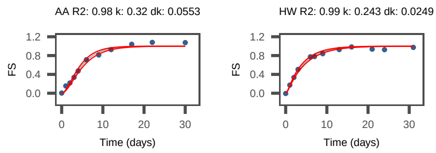

AL1A7 − IFINNEWHDSVSSK_2

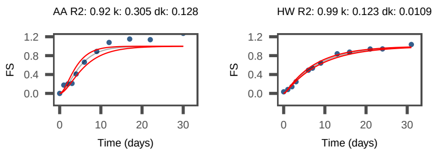

AL1B1 − KIEEVIQR_2

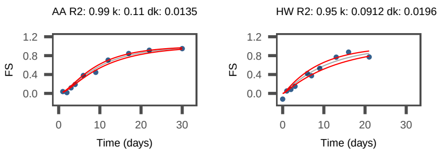

AL1L1 − GQALPEVVAK_2

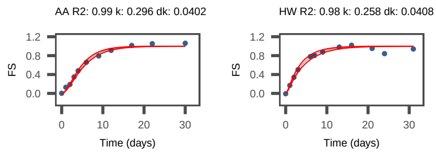

AL1A7 − ILGLIESGK_2

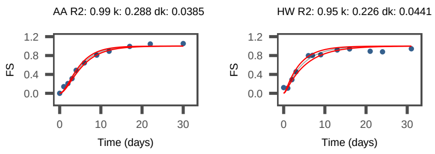

AL1B1 − LAPALATGNTVVMK_2

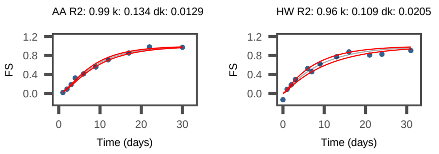

AL1L1 − KLVEYCQR_2

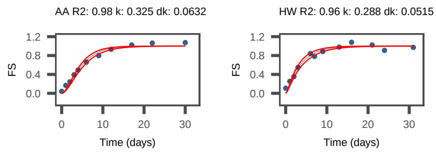

AL1A7 − LLLATMESMNAGK_2

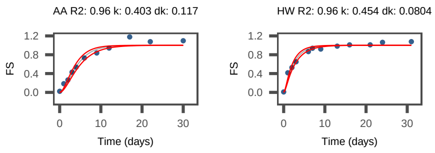

AL1B1 − LFINNEWHDAVSK_3

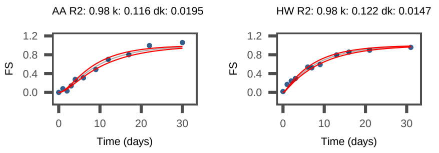

AL1L1 − LFVEDSIHDQFVQK_3

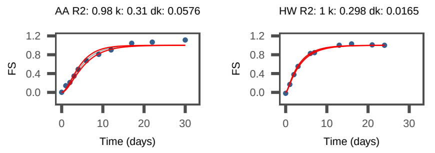

AL1A7 − PAEQTPLTALHMASLIK_3

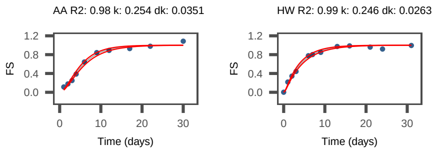

AL1B1 − VAFTGSTEVGHLIQK_3

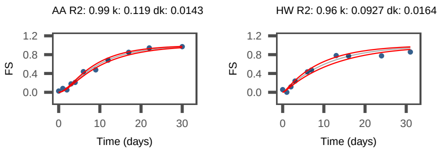

AL1L1 − LQAGTVFVNTYNK_2

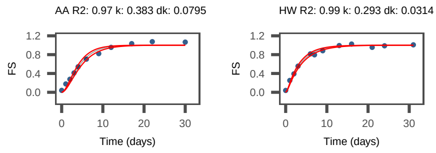

AL1L1 − LRGEDGESECVINYVEK_2

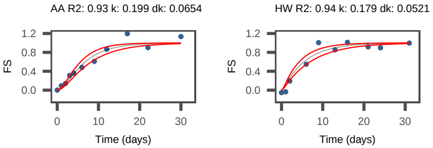

AL1L2(Non−Unique) − YFAGWCDK_2

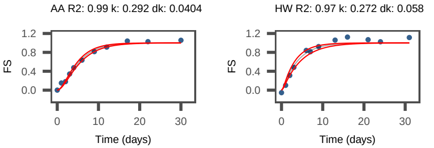

AL3A2 − YLAPTILTDVDPNSK_2

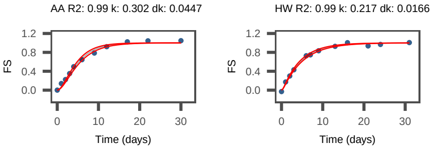

AL1L1 − MMPASQFFK_2

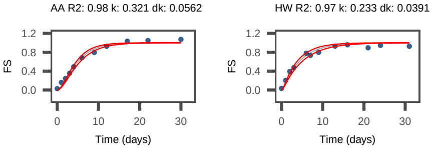

AL3A2 − EILAAIAADLSK_2

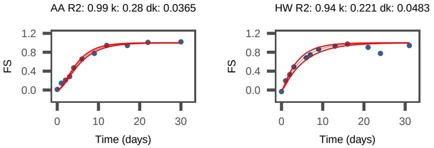

AL4A1 − ETLQLVDSTTSYGLTGAVFAQDK_2

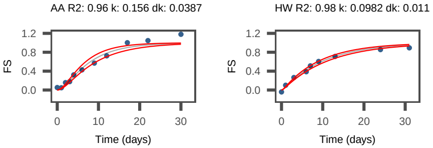

AL1L1 − SPLIIFADCDLNK_3

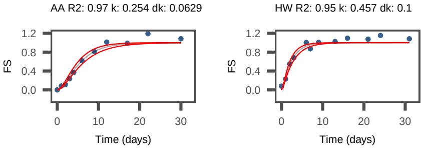

AL3A2 − SLPFGGVGASGMGAYHGK_2

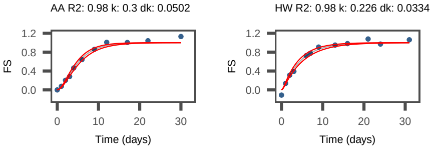

AL4A1 − SAFEYGGQK_2

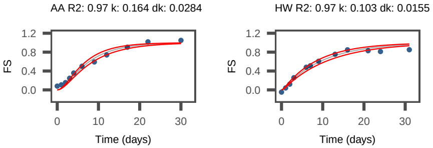

AL1L1 − TSGLVAQGEALPIPGAHRPGLVTK_3

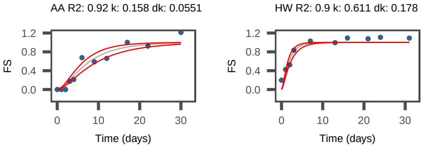

AL3A2 − SLPFGGVGASGMGAYHGK_3

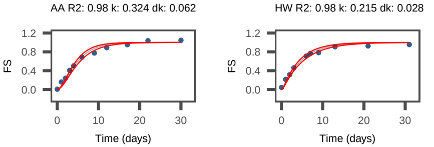

AL4A1 − WTSPQVIK_2

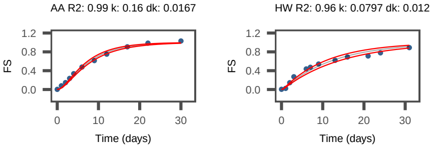

AL1L1 − TYSTINPTDGSVICQVSLAQVSDVDK_2

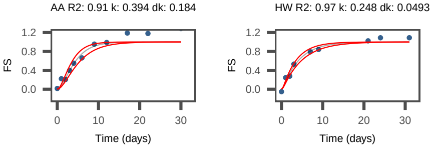

AL3A2 − VMQEEIFGPILPIVSVK_2

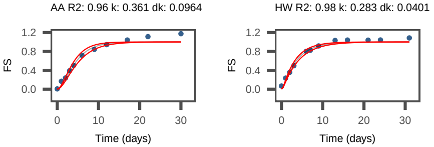

AL7A1 − FQDEEEVFEWNNEVK_2

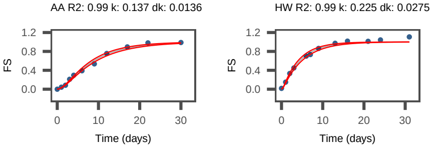

AL1L1 − WADDGLDTGDLLLQK_2

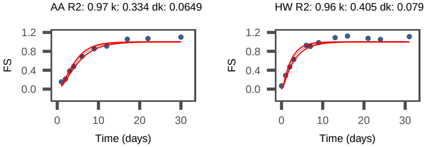

AL3A2 − VMQEEIFGPILPIVSVK_3

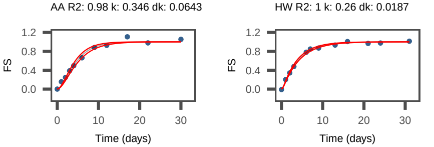

AL7A1 − GAPTTSLVSVAVTK_2

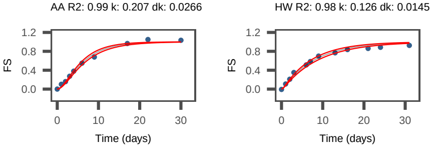

AL7A1 − GSDCGIVNVNIPTSGAEIGGAFGGEK_3

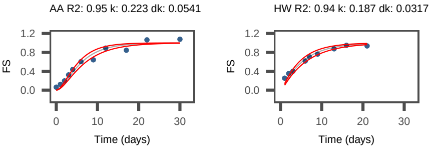

AL8A1 − IAPAIAAGNTVIAK_2

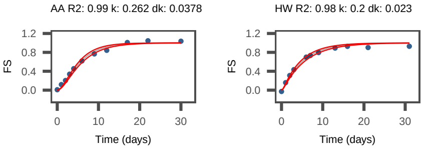

ALAT1 − VLTEMGPPYATQQELASFHSVSK_3

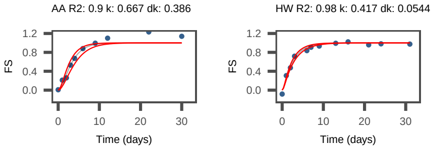

AL7A1 − QGLSSSIFTK_2

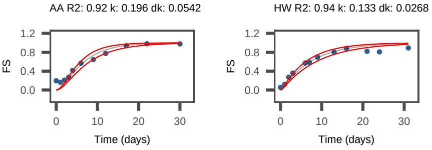

AL8A1 − VGVPSDPSANMGALISK_2

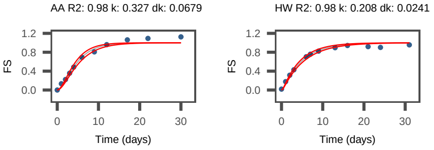

ALAT2 − GGYMEVINLHPEIK_3

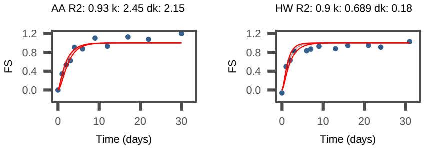

AL7A1 − VGNPWDPNILYGPLHTK_3

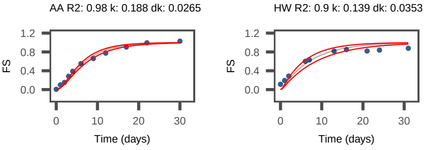

AL9A1 − EPLGVCVGIGAWNYPFQIACWK_3

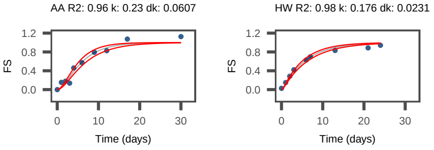

ALBU − AADKDTCFSTEGPNLVTR_3

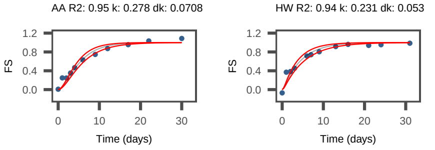

AL7A1 − VNLLSFTGSTQVGK_2

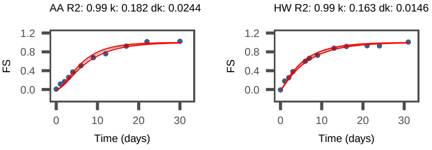

AL9A1 − FINEVVK_2

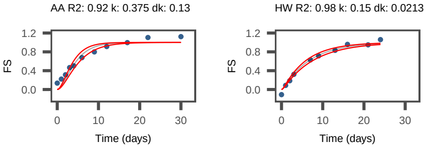

ALBU − AETFTFHSDICTLPEK_3

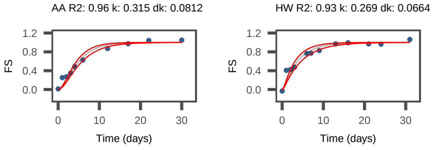

AL8A1 − ELLMLENFIGGK_2

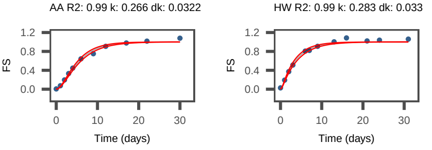

AL9A1 − ISFTGSVPTGVK_2

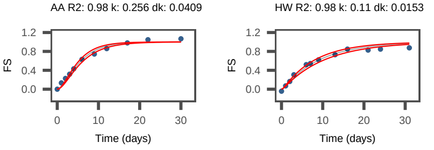

ALBU − ENYGELADCCTK_2

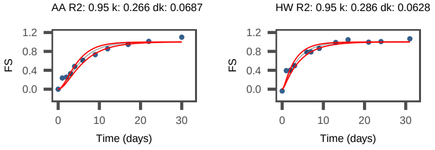

AL8A1 − FLPCNSYIDSYDPSTGEVYCK_2

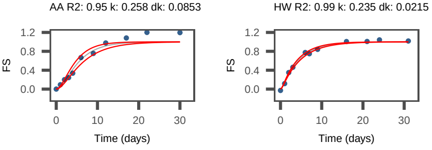

ALAT1 − CNPVQGAMYSFPQIQLPLK_2

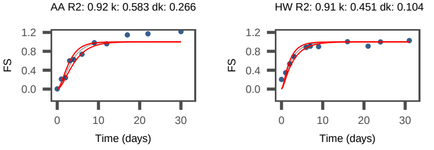

ALBU − GLVLIAFSQYLQK_2

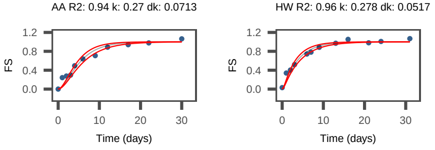

ALBU − QTALAELVK_2

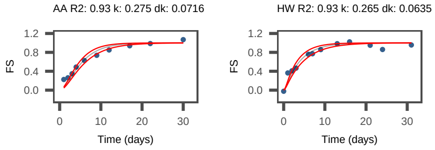

ALBU − YMCENQATISSK_2

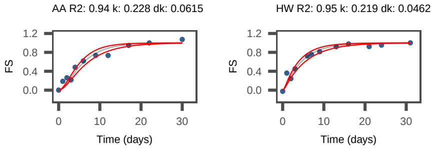

ALDH2 − TYLAALETLDNGKPYVISY_2

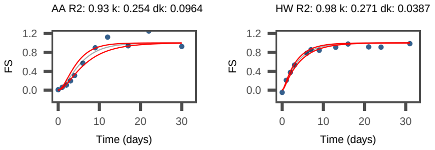

ALBU − RPCFSALTVDETYVPK_2

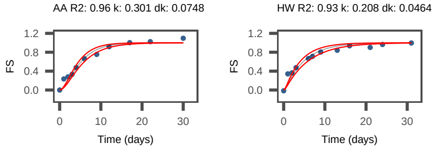

ALBU_HUMAN,sp|P07724|ALBU(Non−Unique) − NECFLQHK_3

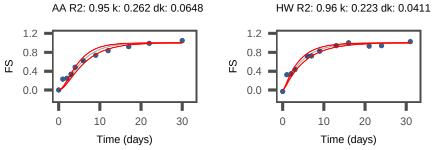

ALDH2 − VAEQTPLTALYVANLIK_2

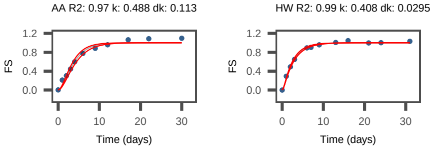

ALBU − RPCFSALTVDETYVPK_3

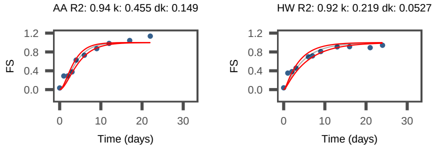

ALDH2 − CYDVFGAQSPFGGYK_2

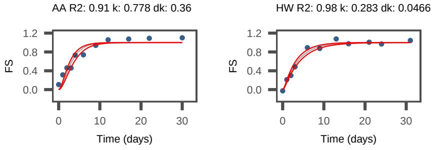

ALDH2 − VAEQTPLTALYVANLIK_3

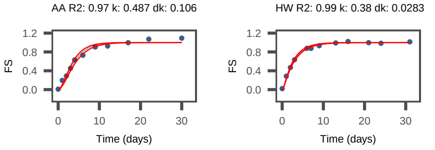

ALBU − TCVADESAANCDK_2

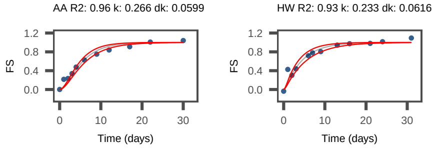

ALDH2 − EEIFGPVMQILK_2

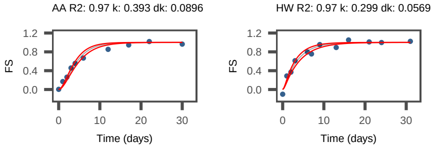

ALDH2 − VAFTGSTEVGHLIQVAAGSSNLK_2

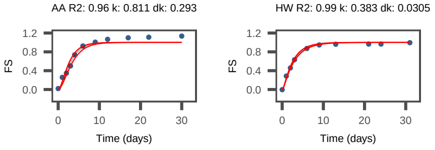

ALBU − TVMDDFAQFLDTCCK_2

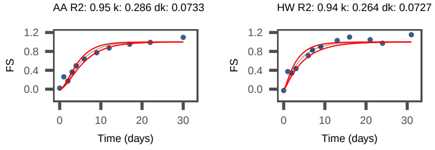

ALDH2 − IIPWNFPLLMQAWK_2

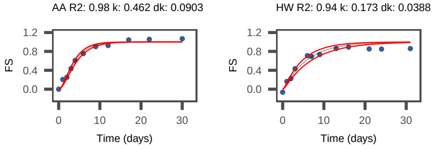

ALDH2 − VAFTGSTEVGHLIQVAAGSSNLK_3

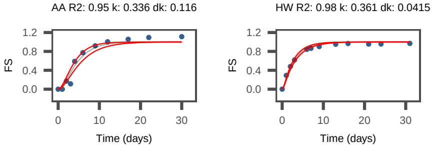

ALBU − TVMDDFAQFLDTCCK_3

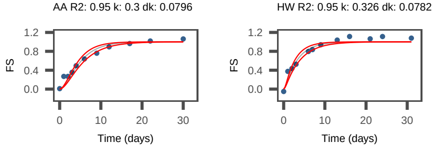

ALDH2 − LGPALATGNVVVMK_2

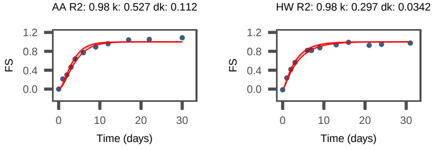

ALDH2 − YGLAAAVFTK_2

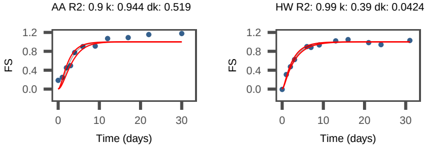

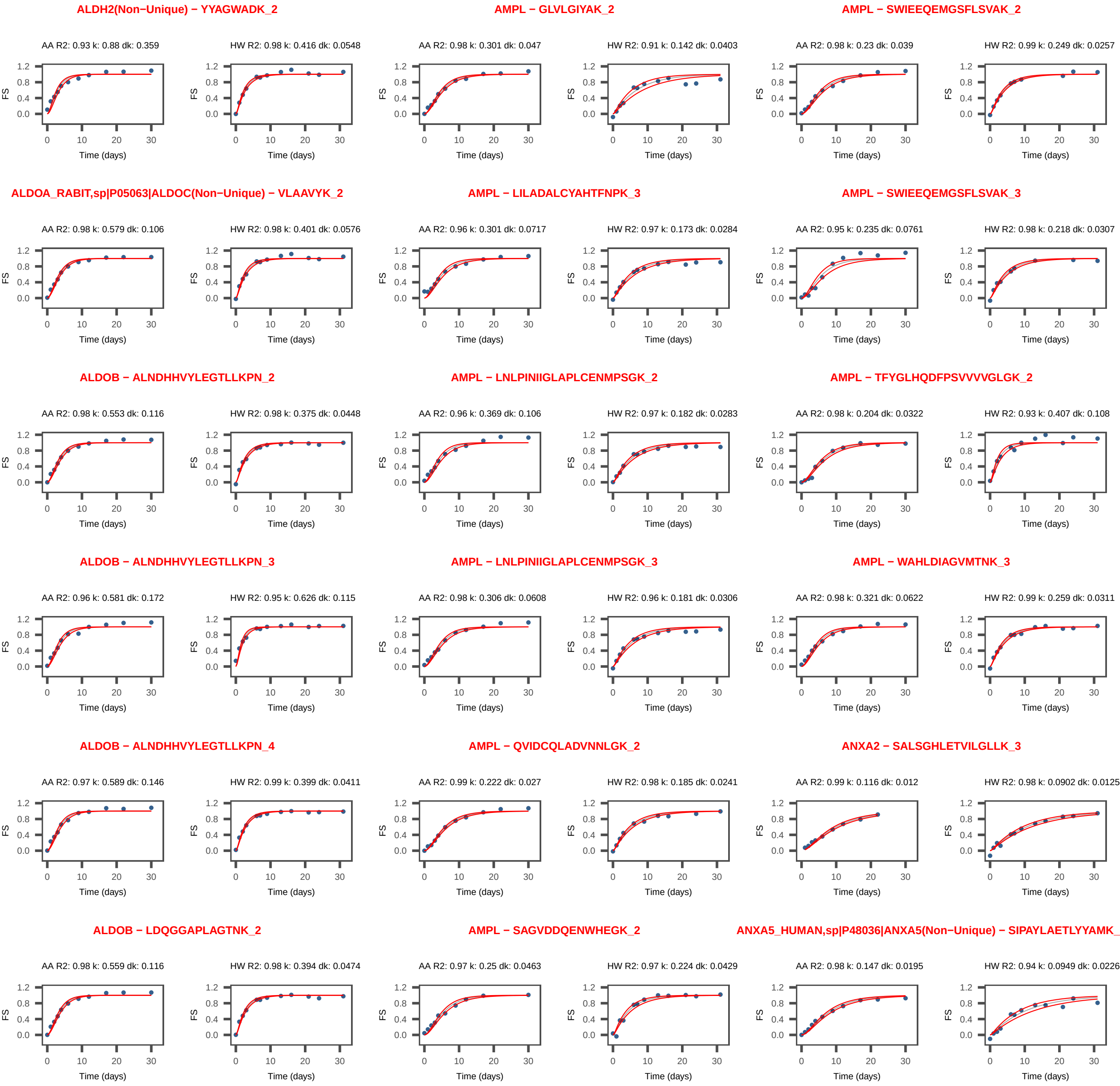
ALDH2(Non−Unique) − YYAGWADK_2
AA R2: 0.93 k: 0.88 dk: 0.359
HW R2: 0.98 k: 0.416 dk: 0.0548
AMPL − GLVLGIYAK_2
AA R2: 0.98 k: 0.301 dk: 0.047
HW R2: 0.91 k: 0.142 dk: 0.0403
AMPL − SWIEEQEMGSFLSVAK_2
AA R2: 0.98 k: 0.23 dk: 0.039
HW R2: 0.99 k: 0.249 dk: 0.0257
ALDOA_RABIT,sp|P05063|ALDOC(Non−Unique) − VLAAVYK_2
AA R2: 0.98 k: 0.579 dk: 0.106
HW R2: 0.98 k: 0.401 dk: 0.0576
AMPL − LILADALCYAHTFNPK_3
AA R2: 0.96 k: 0.301 dk: 0.0717
HW R2: 0.97 k: 0.173 dk: 0.0284
AMPL − SWIEEQEMGSFLSVAK_3
AA R2: 0.95 k: 0.235 dk: 0.0761
HW R2: 0.98 k: 0.218 dk: 0.0307
ALDOB − ALNDHHVYLEGTLLKPN_2
AA R2: 0.98 k: 0.553 dk: 0.116
HW R2: 0.98 k: 0.375 dk: 0.0448
AMPL − LNLPINIIGLAPLCENMPSGK_2
AA R2: 0.96 k: 0.369 dk: 0.106
HW R2: 0.97 k: 0.182 dk: 0.0283
AMPL − TFYGLHQDFPSVVVVGLGK_2
AA R2: 0.98 k: 0.204 dk: 0.0322
HW R2: 0.93 k: 0.407 dk: 0.108
ALDOB − ALNDHHVYLEGTLLKPN_3
AA R2: 0.96 k: 0.581 dk: 0.172
HW R2: 0.95 k: 0.626 dk: 0.115
AMPL − LNLPINIIGLAPLCENMPSGK_3
AA R2: 0.98 k: 0.306 dk: 0.0608
HW R2: 0.96 k: 0.181 dk: 0.0306
AMPL − WAHLDIAGVMTNK_3
AA R2: 0.98 k: 0.321 dk: 0.0622
HW R2: 0.99 k: 0.259 dk: 0.0311
ALDOB − ALNDHHVYLEGTLLKPN_4
AA R2: 0.97 k: 0.589 dk: 0.146
HW R2: 0.99 k: 0.399 dk: 0.0411
AMPL − QVIDCQLADVNNLGK_2
AA R2: 0.99 k: 0.222 dk: 0.027
HW R2: 0.98 k: 0.185 dk: 0.0241
ANXA2 − SALSGHLETVILGLLK_3
AA R2: 0.99 k: 0.116 dk: 0.012
HW R2: 0.98 k: 0.0902 dk: 0.0125
ALDOB − LDQGGAPLAGTNK_2
AA R2: 0.98 k: 0.559 dk: 0.116
HW R2: 0.98 k: 0.394 dk: 0.0474
AMPL − SAGVDDQENWHEGK_2
AA R2: 0.97 k: 0.25 dk: 0.0463
HW R2: 0.97 k: 0.224 dk: 0.0429
ANXA5_HUMAN,sp|P48036|ANXA5(Non−Unique) − SIPAYLAETLYYAMK_
AA R2: 0.98 k: 0.147 dk: 0.0195
HW R2: 0.94 k: 0.0949 dk: 0.0226
FS
Time (days)

ANXA5_HUMAN,sp|P48036|ANXA5(Non−Unique) − SIPAYLAETLYYAMK_3

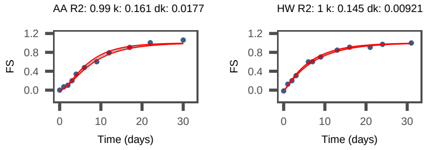

AOFB − YVISAIPPALGMK_2

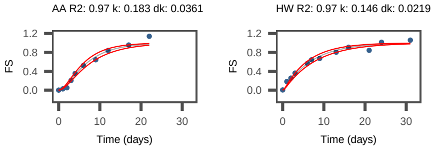

AP2B1 − APEVSQYIYQVYDSILK_3

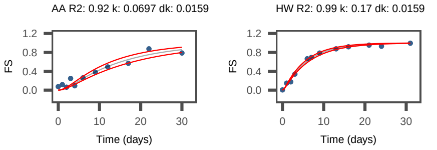

ANXA6 − DAFVAIVQSVK_2

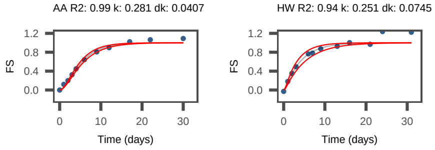

AOXC − GYQADMDWEK_2

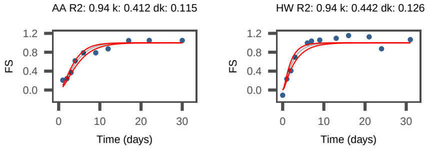

APEH − SFNLSALEK_2

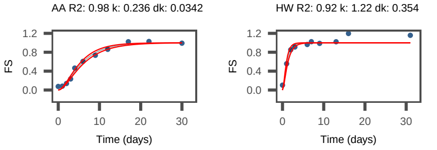

ANXA6 − GTVCAANDFNPDADAK_2

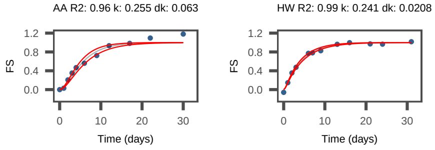

AOXC − ILELFVVTNTK_2

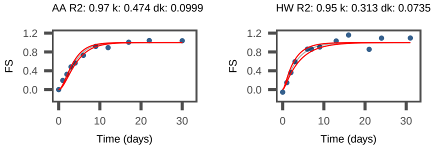

ARF3(Non−Unique) − DAVLLVFANK_2

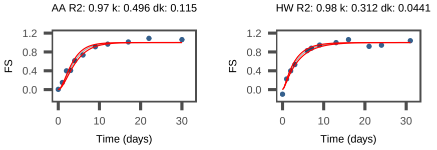

ANXA6 − SLHQAIEGDTSGDFMK_2

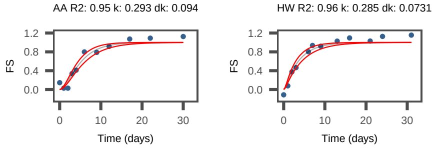

AOXC − MACEDQFTNLVPQTDSK_2

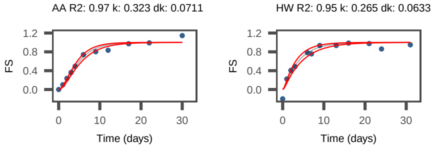

ARF3(Non−Unique) − LGEIVTTIPTIGFNVETVEYK_2

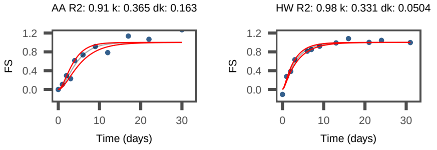

AOFB − APLAEEWDYMTMK_2

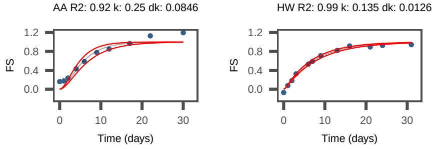

AOXC − TTWIAPGTLNDLLELK_2

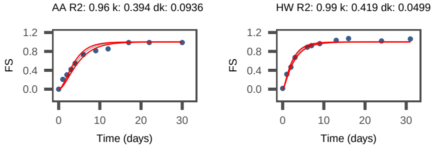

ARF3(Non−Unique) − QDLPNAMNAAEITDK_2

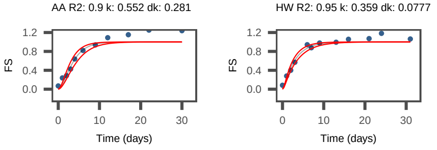

AOFB − TMDEMGQEIPSDAPWK_2

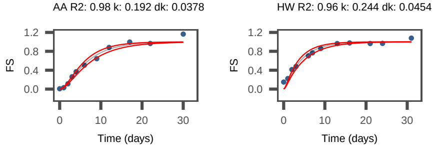

AP2A1(Non−Unique) − YLALESMCTLASSEFSHEAVK_3

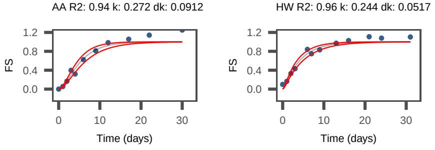

ARGI1 − ANEELAGVVAEVQK_2

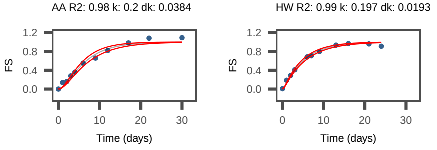

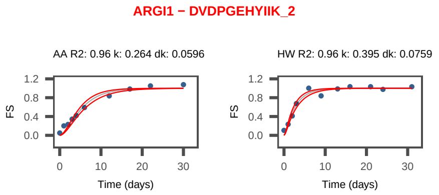
ARGI1 − DVDPGEHYIIK_2
AA R2: 0.96 k: 0.264 dk: 0.0596
HW R2: 0.96 k: 0.395 dk: 0.0759
FS
Time (days)

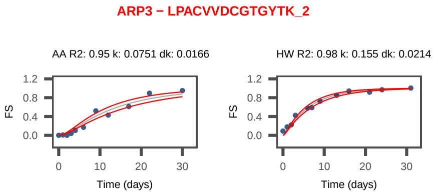
ARP3 − LPACVVDCGTGYTK_2
AA R2: 0.95 k: 0.0751 dk: 0.0166
HW R2: 0.98 k: 0.155 dk: 0.0214
FS
Time (days)

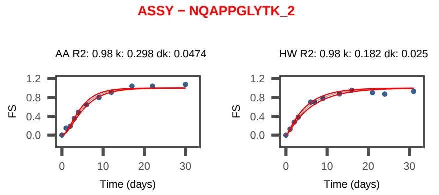
ASSY − NQAPPGLYTK_2
AA R2: 0.98 k: 0.298 dk: 0.0474
HW R2: 0.98 k: 0.182 dk: 0.025
FS
Time (days)

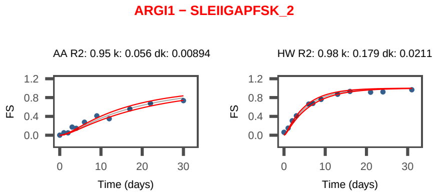
ARGI1 − SLEIIGAPFSK_2
AA R2: 0.95 k: 0.056 dk: 0.00894
HW R2: 0.98 k: 0.179 dk: 0.0211
FS
Time (days)

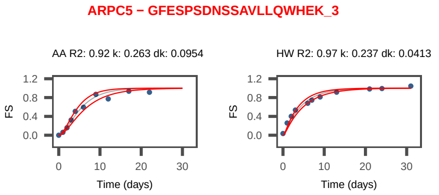
ARPC5 − GFESPSDNSSAVLLQWHEK_3
AA R2: 0.92 k: 0.263 dk: 0.0954
HW R2: 0.97 k: 0.237 dk: 0.0413
FS
Time (days)

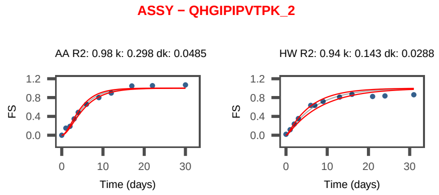
ASSY − QHGIPIPVTPK_2
AA R2: 0.98 k: 0.298 dk: 0.0485
HW R2: 0.94 k: 0.143 dk: 0.0288
FS
Time (days)

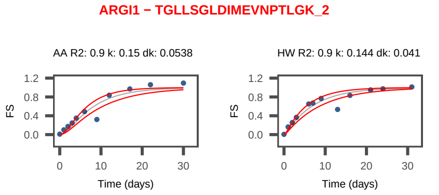
ARGI1 − TGLLSGLDIMEVNPTLGK_2
AA R2: 0.9 k: 0.15 dk: 0.0538
HW R2: 0.9 k: 0.144 dk: 0.041
FS
Time (days)

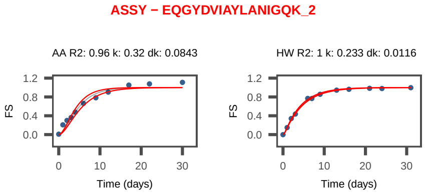
ASSY − EQGYDVIAYLANIGQK_2
AA R2: 0.96 k: 0.32 dk: 0.0843
HW R2: 1 k: 0.233 dk: 0.0116
FS
Time (days)

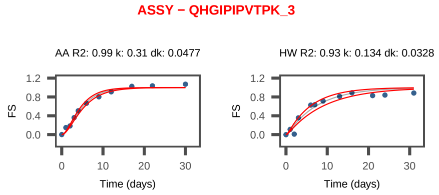
ASSY − QHGIPIPVTPK_3
AA R2: 0.99 k: 0.31 dk: 0.0477
HW R2: 0.93 k: 0.134 dk: 0.0328
FS
Time (days)

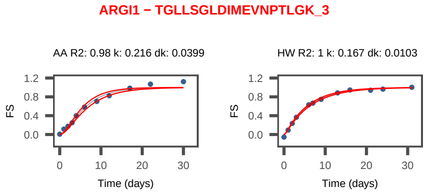
ARGI1 − TGLLSGLDIMEVNPTLGK_3
AA R2: 0.98 k: 0.216 dk: 0.0399
HW R2: 1 k: 0.167 dk: 0.0103
FS
Time (days)

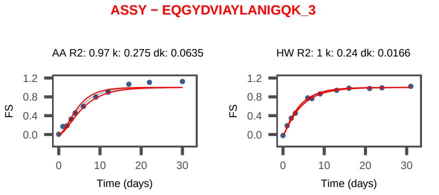
ASSY − EQGYDVIAYLANIGQK_3
AA R2: 0.97 k: 0.275 dk: 0.0635
HW R2: 1 k: 0.24 dk: 0.0166
FS
Time (days)

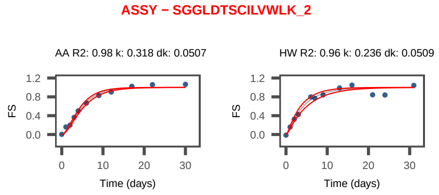
ASSY − SGGLDTSCILVWLK_2
AA R2: 0.98 k: 0.318 dk: 0.0507
HW R2: 0.96 k: 0.236 dk: 0.0509
FS
Time (days)

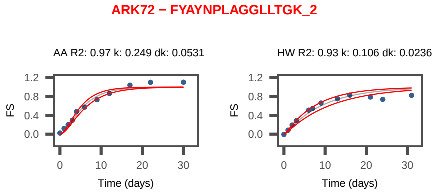
ARK72 − FYAYNPLAGGLLTGK_2
AA R2: 0.97 k: 0.249 dk: 0.0531
HW R2: 0.93 k: 0.106 dk: 0.0236
FS
Time (days)

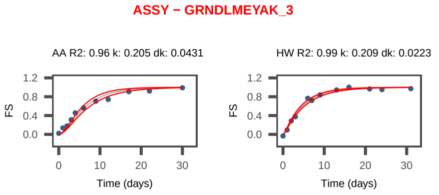
ASSY − GRNDLMEYAK_3
AA R2: 0.96 k: 0.205 dk: 0.0431
HW R2: 0.99 k: 0.209 dk: 0.0223
FS
Time (days)

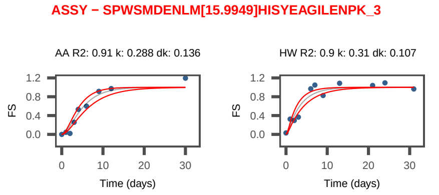
ASSY − SPWSMDENLM[15.9949]HISYEAGILENPK_3
AA R2: 0.91 k: 0.288 dk: 0.136
HW R2: 0.9 k: 0.31 dk: 0.107
FS
Time (days)

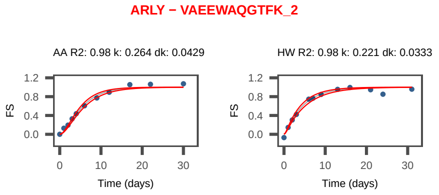
ARLY − VAEEWAQGTFK_2
AA R2: 0.98 k: 0.264 dk: 0.0429
HW R2: 0.98 k: 0.221 dk: 0.0333
FS
Time (days)

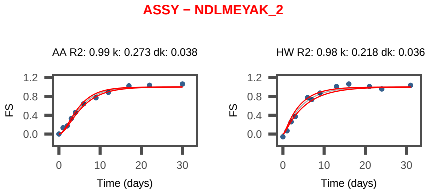
ASSY − NDLMEYAK_2
AA R2: 0.99 k: 0.273 dk: 0.038
HW R2: 0.98 k: 0.218 dk: 0.036
FS
Time (days)

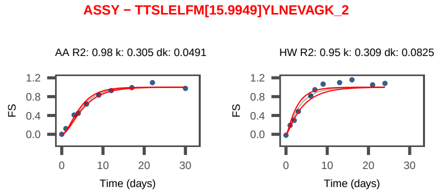
ASSY − TTSLELFM[15.9949]YLNEVAGK_2
AA R2: 0.98 k: 0.305 dk: 0.0491
HW R2: 0.95 k: 0.309 dk: 0.0825
FS
Time (days)

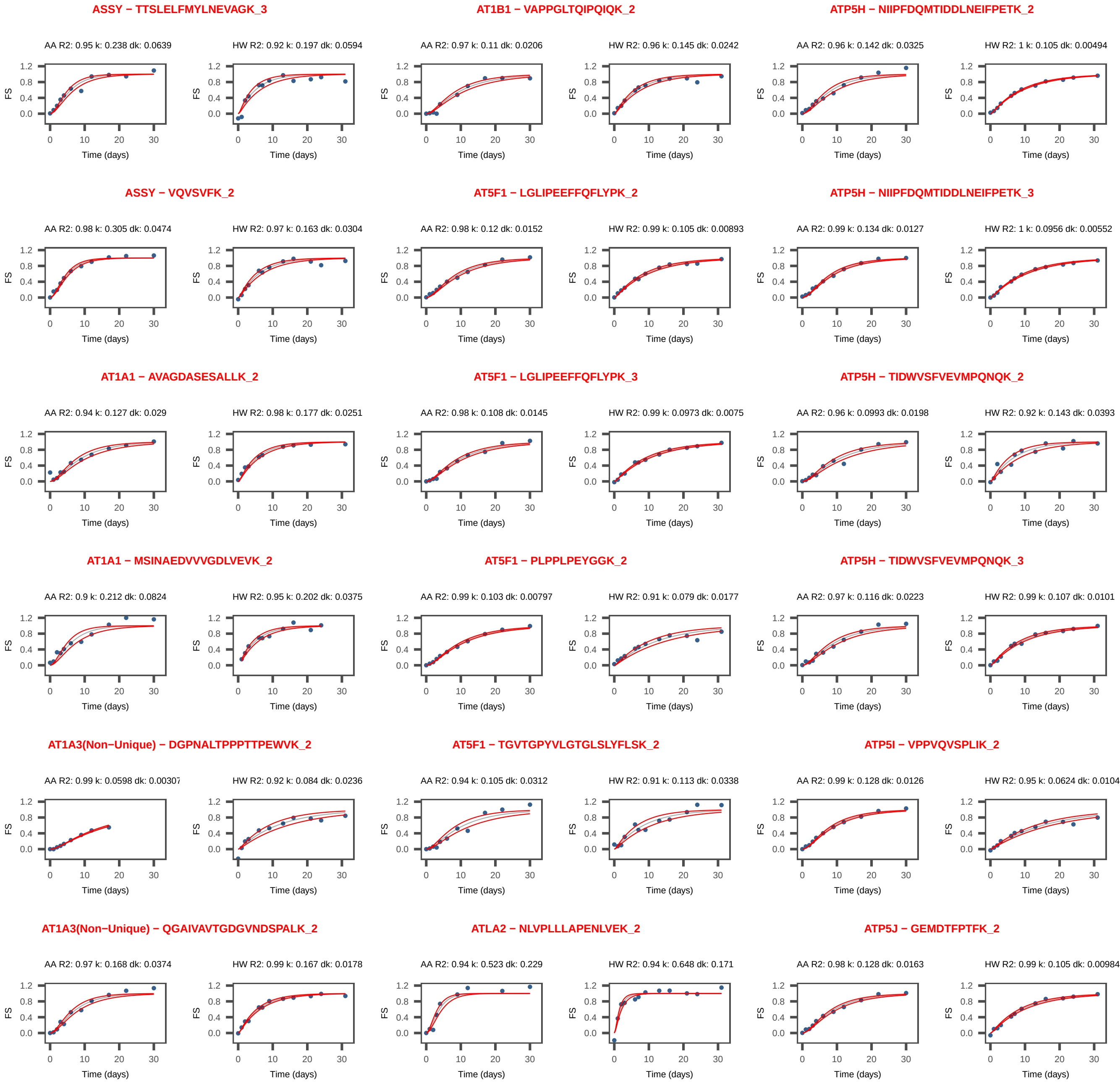
ASSY − TTSLELFMYLNEVAGK_3
AA R2: 0.95 k: 0.238 dk: 0.0639
HW R2: 0.92 k: 0.197 dk: 0.0594
AT1B1 − VAPPGLTQIPQIQK_2
AA R2: 0.97 k: 0.11 dk: 0.0206
HW R2: 0.96 k: 0.145 dk: 0.0242
ATP5H − NIIPFDQMTIDDLNEIFPETK_2
AA R2: 0.96 k: 0.142 dk: 0.0325
HW R2: 1 k: 0.105 dk: 0.00494
ASSY − VQVSVFK_2
AA R2: 0.98 k: 0.305 dk: 0.0474
HW R2: 0.97 k: 0.163 dk: 0.0304
AT5F1 − LGLIPEEFFQFLYPK_2
AA R2: 0.98 k: 0.12 dk: 0.0152
HW R2: 0.99 k: 0.105 dk: 0.00893
ATP5H − NIIPFDQMTIDDLNEIFPETK_3
AA R2: 0.99 k: 0.134 dk: 0.0127
HW R2: 1 k: 0.0956 dk: 0.00552
AT1A1 − AVAGDASESALLK_2
AA R2: 0.94 k: 0.127 dk: 0.029
HW R2: 0.98 k: 0.177 dk: 0.0251
AT5F1 − LGLIPEEFFQFLYPK_3
AA R2: 0.98 k: 0.108 dk: 0.0145
HW R2: 0.99 k: 0.0973 dk: 0.0075
ATP5H − TIDWVSFVEVMPQNQK_2
AA R2: 0.96 k: 0.0993 dk: 0.0198
HW R2: 0.92 k: 0.143 dk: 0.0393
AT1A1 − MSINAEDVVVGDLVEVK_2
AA R2: 0.9 k: 0.212 dk: 0.0824
HW R2: 0.95 k: 0.202 dk: 0.0375
AT5F1 − PLPPLPEYGGK_2
AA R2: 0.99 k: 0.103 dk: 0.00797
HW R2: 0.91 k: 0.079 dk: 0.0177
ATP5H − TIDWVSFVEVMPQNQK_3
AA R2: 0.97 k: 0.116 dk: 0.0223
HW R2: 0.99 k: 0.107 dk: 0.0101
AT1A3(Non−Unique) − DGPNALTPPPTTPEWVK_2
AA R2: 0.99 k: 0.0598 dk: 0.00307
HW R2: 0.92 k: 0.084 dk: 0.0236
AT5F1 − TGVTGPYVLGTGLSLYFLSK_2
AA R2: 0.94 k: 0.105 dk: 0.0312
HW R2: 0.91 k: 0.113 dk: 0.0338
ATP5I − VPPVQVSPLIK_2
AA R2: 0.99 k: 0.128 dk: 0.0126
HW R2: 0.95 k: 0.0624 dk: 0.0104
AT1A3(Non−Unique) − QGAIVAVTGDGVNDSPALK_2
AA R2: 0.97 k: 0.168 dk: 0.0374
HW R2: 0.99 k: 0.167 dk: 0.0178
ATLA2 − NLVPLLLAPENLVEK_2
AA R2: 0.94 k: 0.523 dk: 0.229
HW R2: 0.94 k: 0.648 dk: 0.171
ATP5J − GEMDTFPTFK_2
AA R2: 0.98 k: 0.128 dk: 0.0163
HW R2: 0.99 k: 0.105 dk: 0.00984
FS
Time (days)

ATP5L − APSMVAAAVTYSK_2

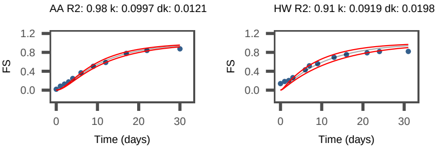

ATPA − TSIAIDTIINQK_2

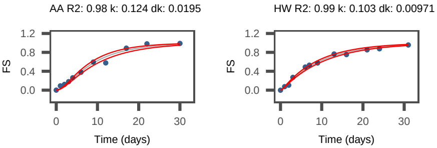

ATPB − TVLIMELINNVAK_2

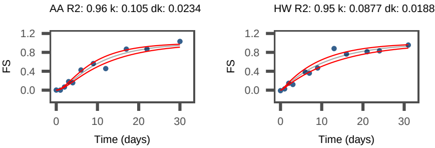

ATP5L − LATFWHYAK_3

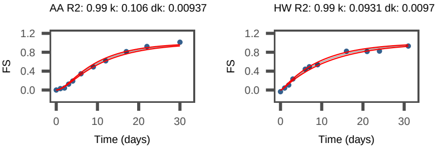

ATPA − VVDALGNAIDGK_2

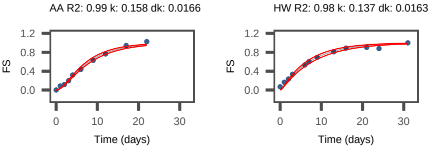

ATPB − VVDLLAPYAK_2

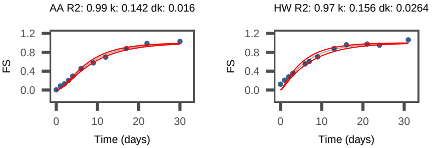

ATP5L − VELVPPTPAEIPTAIQSVK_2

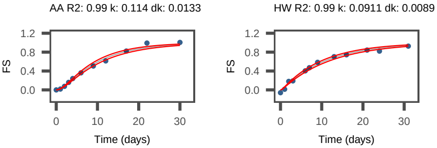

ATPB − IGLFGGAGVGK_2

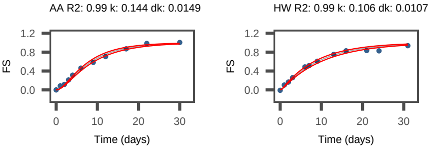

ATPD − SFTFASPTQVFFDSANVK_2

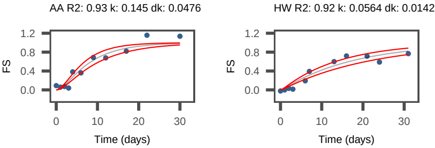

ATPA − GMSLNLEPDNVGVVVFGNDK_2

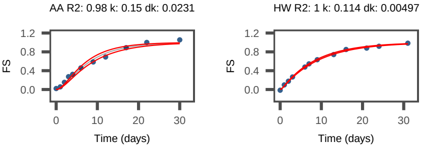

ATPB − QVAEVFTGHMGK_2

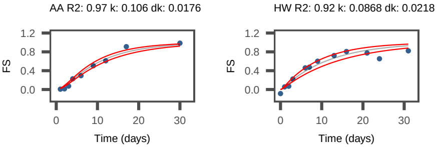

ATPD − TFASPTQVFFDSANVK_2

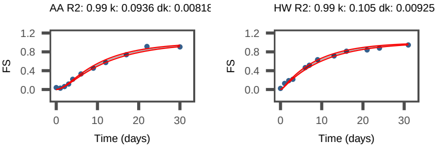

ATPA − LYCIYVAIGQK_2

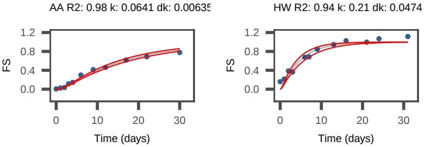

ATPB − QVAEVFTGHMGK_3

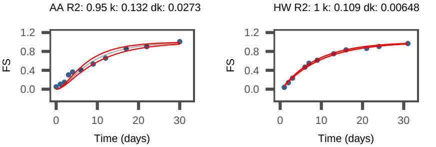

ATPG − VYGTGSLALYEK_2

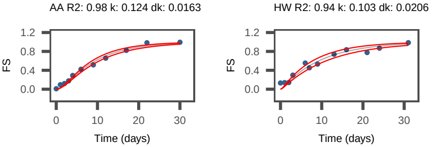

ATPA − RLTDADAMK_2

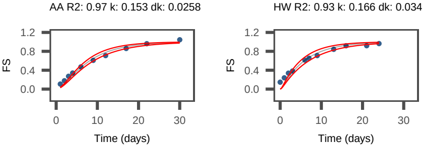

ATPB − SLQDIIAILGMDELSEEDK_3

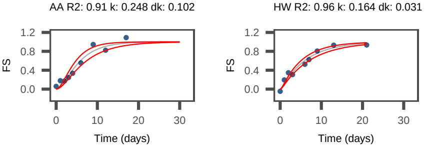

ATPO − YATALYSAASK_2

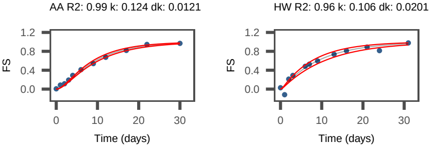

B2MG − VEMSDMSFSK_2

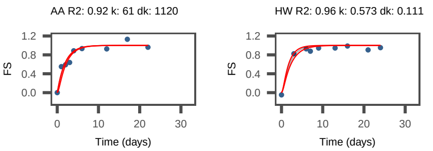

BHMT1(Non−Unique) − GFLPPASEK_2

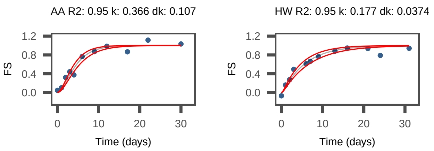

BHMT1 − PYNPSMSRPDAWGVTK_3

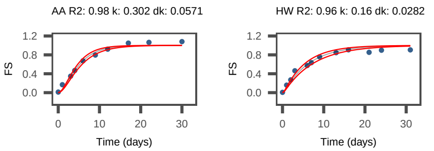

BDH − TIQLNVCNSEEVEK_2

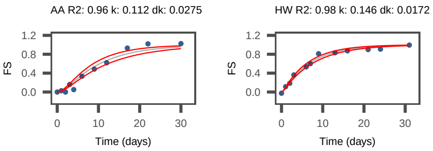

BHMT1 − HGSWGSGLDMHTK_2

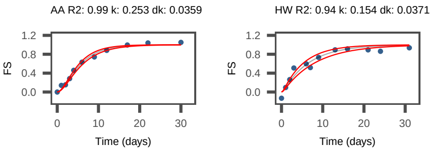

BHMT1 − QVADEGDALVAGGVSQTPSYLSCK_3

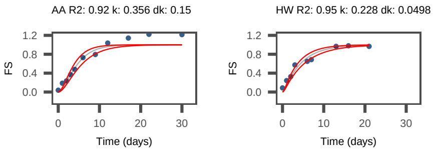

BHMT1 − AGASIVGVNCHFDPSVSLQTVK_2

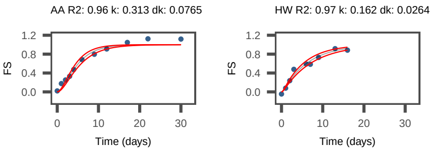

BHMT1 − HGSWGSGLDMHTK_3

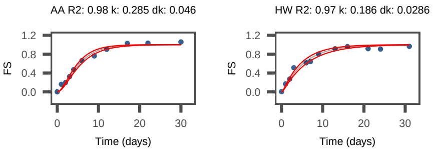

BHMT1 − SRPDAWGVTK_2

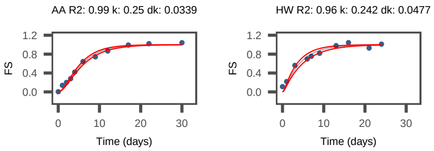

BHMT1 − AGSNVMQTFTFYASEDKLENR_2

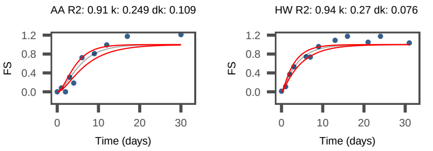

BHMT1 − HGSWGSGLDMHTKPW_3

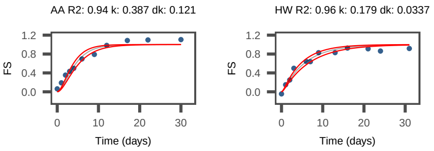

BHMT1 − TFYASEDKLENR_2

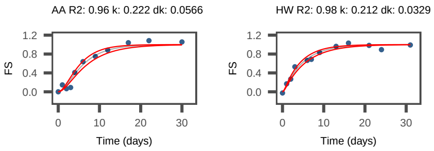

BHMT1 − AGSNVMQTFTFYASEDKLENR_3

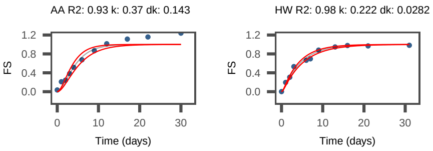

BHMT1 − KEYWQNLR_2

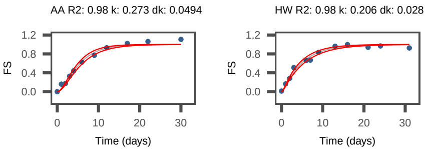

BHMT1 − TFYASEDKLENR_3

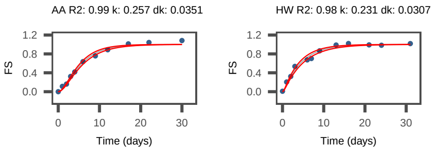

BHMT1 − GAAELMQQK_2

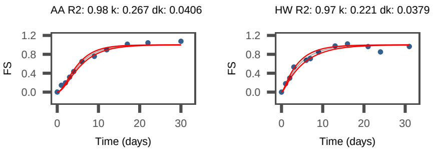

BHMT1 − PYNPSMSRPDAWGVTK_2

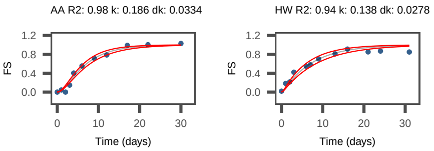

BIP − AKFEELNMDLFR_3

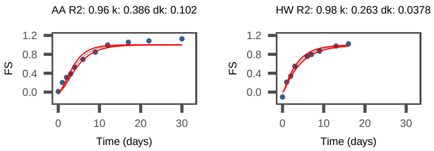

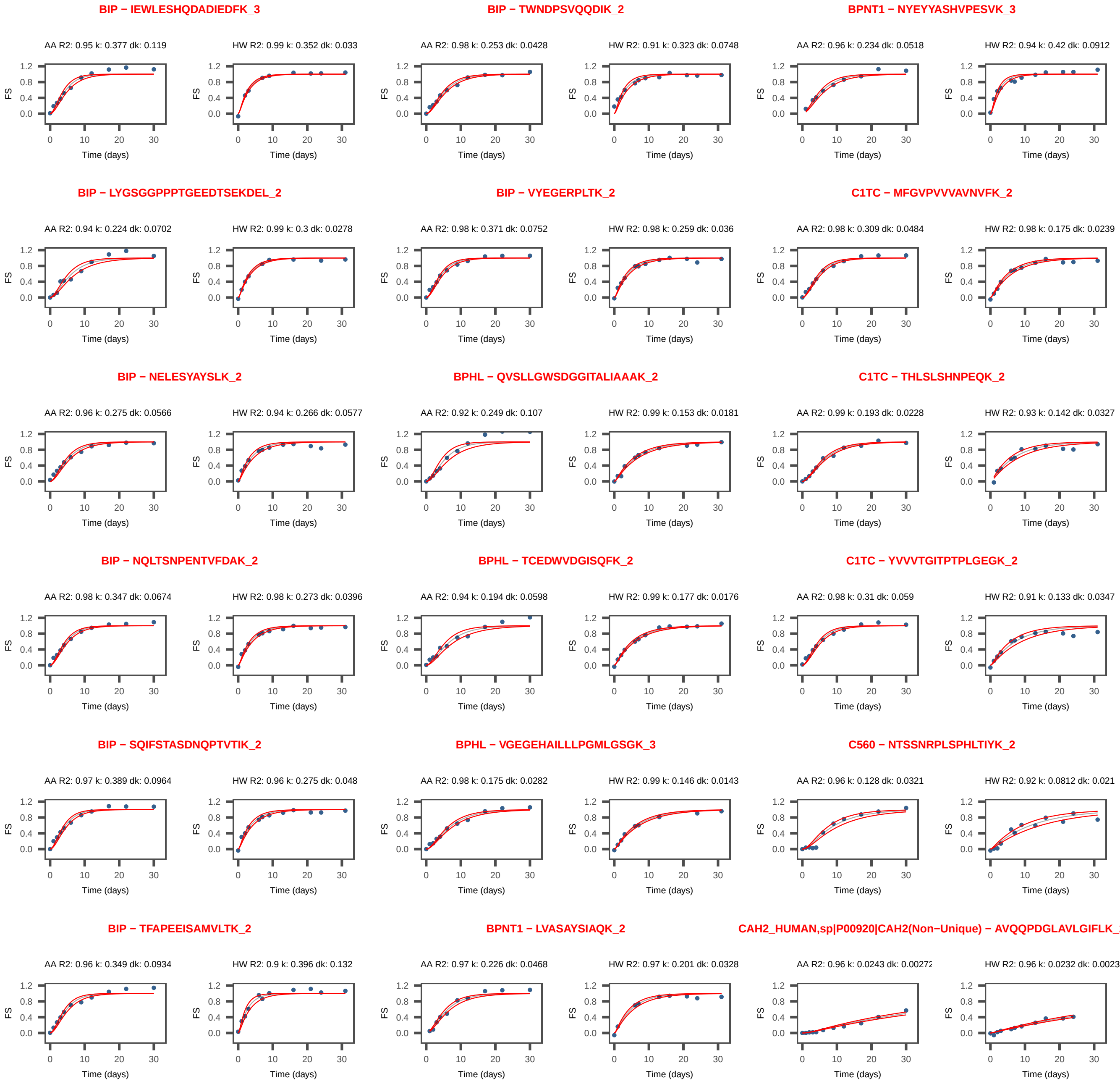
BIP − IEWLESHQDADIEDFK_3
AA R2: 0.95 k: 0.377 dk: 0.119
HW R2: 0.99 k: 0.352 dk: 0.033
BIP − TWNDPSVQQDIK_2
AA R2: 0.98 k: 0.253 dk: 0.0428
HW R2: 0.91 k: 0.323 dk: 0.0748
BPNT1 − NYEYYASHVPESVK_3
AA R2: 0.96 k: 0.234 dk: 0.0518
HW R2: 0.94 k: 0.42 dk: 0.0912
BIP − LYGSGGPPPTGEEDTSEKDEL_2
AA R2: 0.94 k: 0.224 dk: 0.0702
HW R2: 0.99 k: 0.3 dk: 0.0278
BIP − VYEGERPLTK_2
AA R2: 0.98 k: 0.371 dk: 0.0752
HW R2: 0.98 k: 0.259 dk: 0.036
C1TC − MFGVPVVVAVNVFK_2
AA R2: 0.98 k: 0.309 dk: 0.0484
HW R2: 0.98 k: 0.175 dk: 0.0239
BIP − NELESYAYSLK_2
AA R2: 0.96 k: 0.275 dk: 0.0566
HW R2: 0.94 k: 0.266 dk: 0.0577
BPHL − QVSLLGWSDGGITALIAAAK_2
AA R2: 0.92 k: 0.249 dk: 0.107
HW R2: 0.99 k: 0.153 dk: 0.0181
C1TC − THLSLSHNPEQK_2
AA R2: 0.99 k: 0.193 dk: 0.0228
HW R2: 0.93 k: 0.142 dk: 0.0327
BIP − NQLTSNPENTVFDAK_2
AA R2: 0.98 k: 0.347 dk: 0.0674
HW R2: 0.98 k: 0.273 dk: 0.0396
BPHL − TCEDWVDGISQFK_2
AA R2: 0.94 k: 0.194 dk: 0.0598
HW R2: 0.99 k: 0.177 dk: 0.0176
C1TC − YVVVTGITPTPLGEGK_2
AA R2: 0.98 k: 0.31 dk: 0.059
HW R2: 0.91 k: 0.133 dk: 0.0347
BIP − SQIFSTASDNQPTVTIK_2
AA R2: 0.97 k: 0.389 dk: 0.0964
HW R2: 0.96 k: 0.275 dk: 0.048
BPHL − VGEGEHAILLLPGMLGSGK_3
AA R2: 0.98 k: 0.175 dk: 0.0282
HW R2: 0.99 k: 0.146 dk: 0.0143
C560 − NTSSNRPLSPHLTIYK_2
AA R2: 0.96 k: 0.128 dk: 0.0321
HW R2: 0.92 k: 0.0812 dk: 0.021
BIP − TFAPEEISAMVLTK_2
AA R2: 0.96 k: 0.349 dk: 0.0934
HW R2: 0.9 k: 0.396 dk: 0.132
BPNT1 − LVASAYSIAQK_2
AA R2: 0.97 k: 0.226 dk: 0.0468
HW R2: 0.97 k: 0.201 dk: 0.0328
CAH2_HUMAN,sp|P00920|CAH2(Non−Unique) − AVQQPDGLAVLGIFLK_
AA R2: 0.96 k: 0.0243 dk: 0.00272
HW R2: 0.96 k: 0.0232 dk: 0.0023

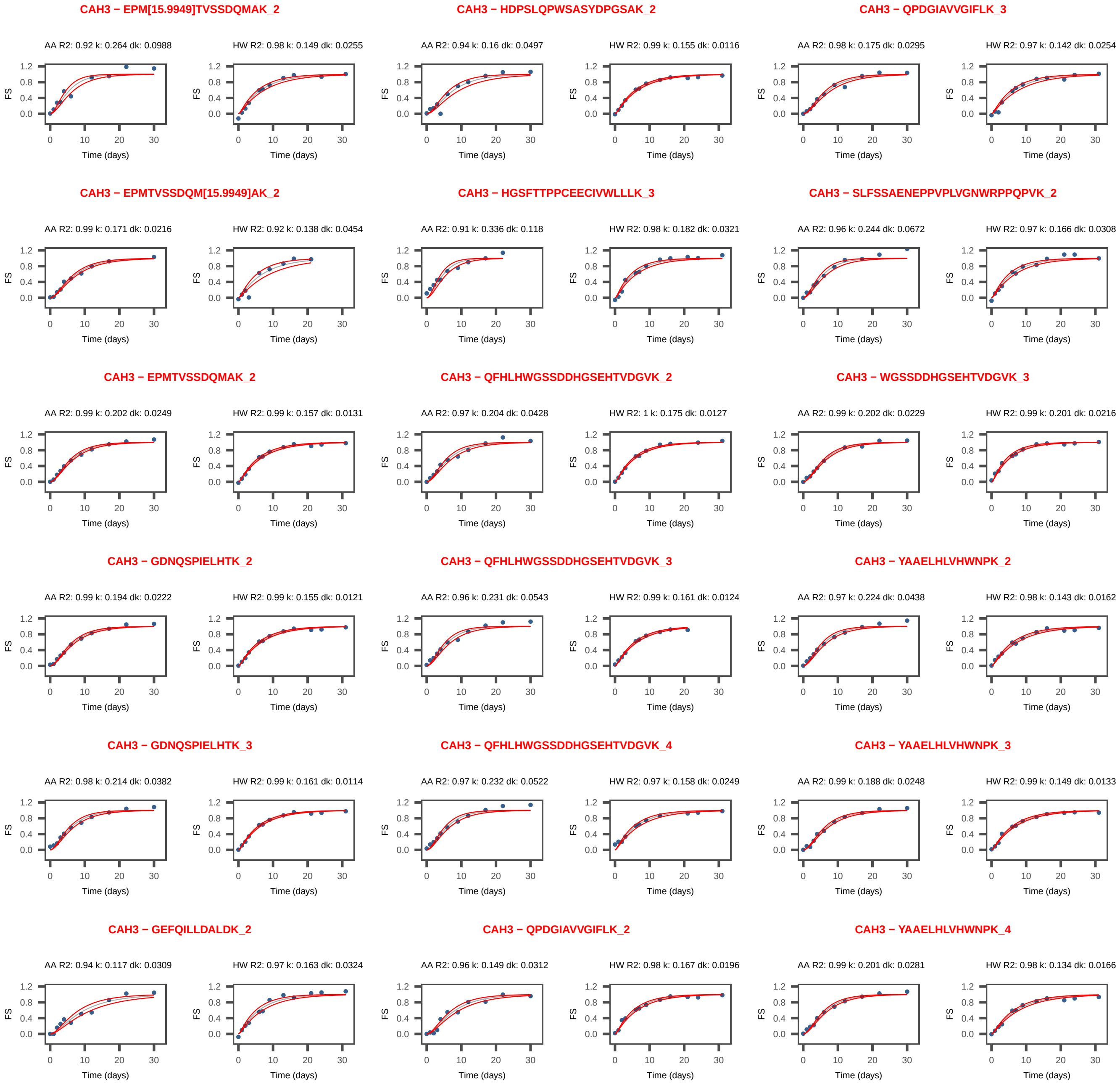
CAH3 − EPM[15.9949]TVSSDQMAK_2
AA R2: 0.92 k: 0.264 dk: 0.0988
HW R2: 0.98 k: 0.149 dk: 0.0255
CAH3 − HDPSLQPWSASYDPGSAK_2
AA R2: 0.94 k: 0.16 dk: 0.0497
HW R2: 0.99 k: 0.155 dk: 0.0116
CAH3 − QPDGIAVVGIFLK_3
AA R2: 0.98 k: 0.175 dk: 0.0295
HW R2: 0.97 k: 0.142 dk: 0.0254
CAH3 − EPMTVSSDQM[15.9949]AK_2
AA R2: 0.99 k: 0.171 dk: 0.0216
HW R2: 0.92 k: 0.138 dk: 0.0454
CAH3 − HGSFTTPPCEECIVWLLLK_3
AA R2: 0.91 k: 0.336 dk: 0.118
HW R2: 0.98 k: 0.182 dk: 0.0321
CAH3 − SLFSSAENEPPVPLVGNWRPPQPVK_2
AA R2: 0.96 k: 0.244 dk: 0.0672
HW R2: 0.97 k: 0.166 dk: 0.0308
CAH3 − EPMTVSSDQMAK_2
AA R2: 0.99 k: 0.202 dk: 0.0249
HW R2: 0.99 k: 0.157 dk: 0.0131
CAH3 − QFHLHWGSSDDHGSEHTVDGVK_2
AA R2: 0.97 k: 0.204 dk: 0.0428
HW R2: 1 k: 0.175 dk: 0.0127
CAH3 − WGSSDDHGSEHTVDGVK_3
AA R2: 0.99 k: 0.202 dk: 0.0229
HW R2: 0.99 k: 0.201 dk: 0.0216
CAH3 − GDNQSPIELHTK_2
AA R2: 0.99 k: 0.194 dk: 0.0222
HW R2: 0.99 k: 0.155 dk: 0.0121
CAH3 − QFHLHWGSSDDHGSEHTVDGVK_3
AA R2: 0.96 k: 0.231 dk: 0.0543
HW R2: 0.99 k: 0.161 dk: 0.0124
CAH3 − YAAELHLVHWNPK_2
AA R2: 0.97 k: 0.224 dk: 0.0438
HW R2: 0.98 k: 0.143 dk: 0.0162
CAH3 − GDNQSPIELHTK_3
AA R2: 0.98 k: 0.214 dk: 0.0382
HW R2: 0.99 k: 0.161 dk: 0.0114
CAH3 − QFHLHWGSSDDHGSEHTVDGVK_4
AA R2: 0.97 k: 0.232 dk: 0.0522
HW R2: 0.97 k: 0.158 dk: 0.0249
CAH3 − YAAELHLVHWNPK_3
AA R2: 0.99 k: 0.188 dk: 0.0248
HW R2: 0.99 k: 0.149 dk: 0.0133
CAH3 − GEFQILLDALDK_2
AA R2: 0.94 k: 0.117 dk: 0.0309
HW R2: 0.97 k: 0.163 dk: 0.0324
CAH3 − QPDGIAVVGIFLK_2
AA R2: 0.96 k: 0.149 dk: 0.0312
HW R2: 0.98 k: 0.167 dk: 0.0196
CAH3 − YAAELHLVHWNPK_4
AA R2: 0.99 k: 0.201 dk: 0.0281
HW R2: 0.98 k: 0.134 dk: 0.0166
FS
Time (days)

CAH3 − YNTFGEALK_2

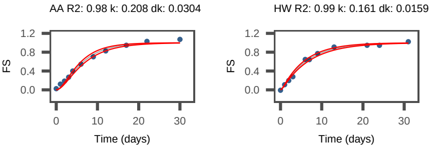

CALR − HEQNIDCGGGYVK_3

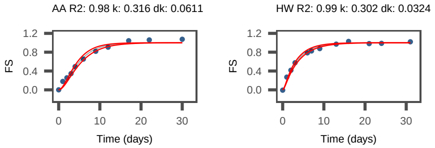

CAP1 − LEAVSHTSDMHCGYGDSPSK_3

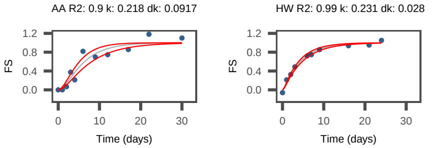

CALM1(Non−Unique) − EAFSLFDK_2

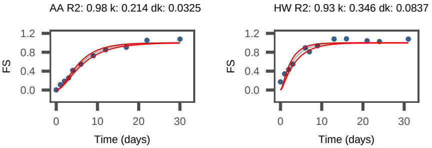

CALR − VHVIFNYK_2

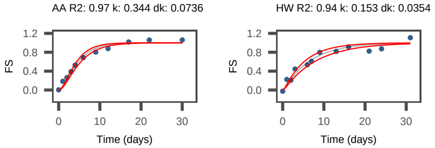

CAP1 − VQAFDSLLANPVAEYLK_2

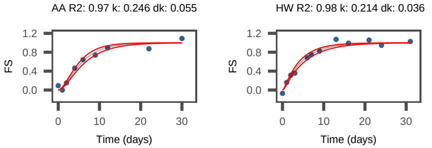

CALM1(Non−Unique) − MKDTDSEEEIR_3

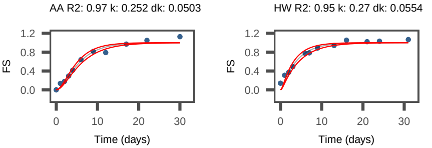

CALU − VHHEPQLSDK_3

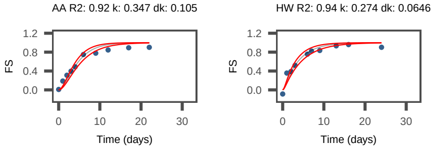

CATA − ASQRPDVLTTGGGNPIGDK_2

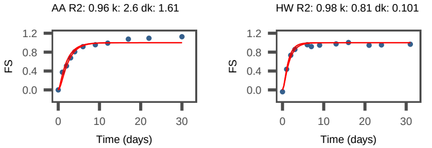

CALR − DMHGDSEYNIMFGPDICGPGTK_3

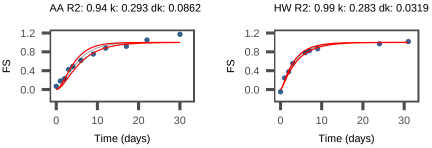

CAND1 − DLLDSVLPHLYNETK_3

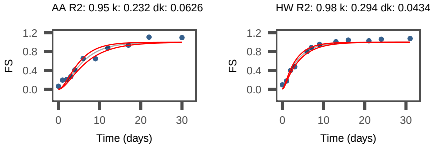

CATA − ASQRPDVLTTGGGNPIGDK_3

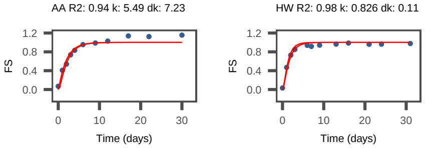

CALR − GQTLVVQFTVK_2

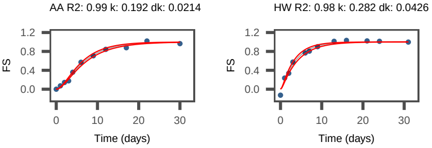

CAP1 − HAEMVHTGLK_2

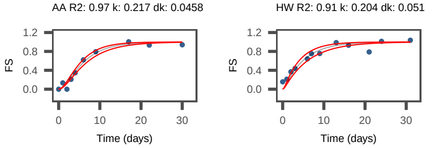

CATA − DAILFPSFIHSQK_3

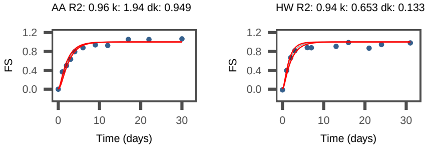

CALR − HEQNIDCGGGYVK_2

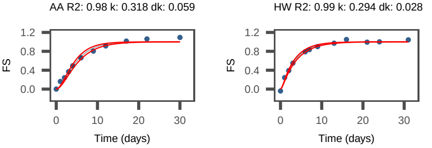

CAP1 − HAEMVHTGLK_3

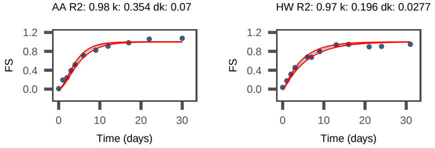

CATA − EAETFPFNPFDLTK_2

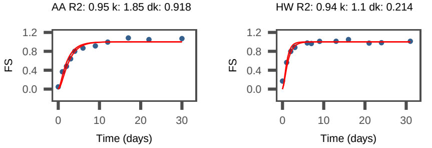

CATA − NAIHTYTQAGSHMAAK_2

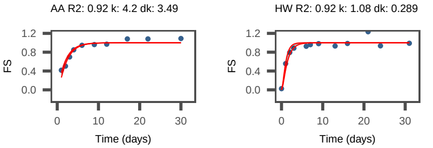

CATD − ILDIACWVHHK_3

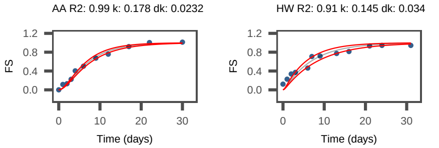

CBPQ − IIVYNQPYTGYEK_2

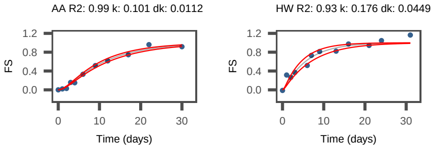

CATA − SALEHSVQCAVDVK_2

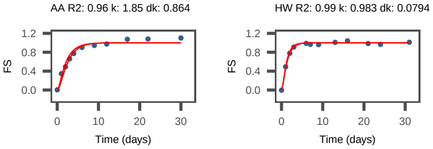

CATD_HUMAN,sp|P18242|CATD(Non−Unique) − AIGAVPLIQGEYMIPCEK_2

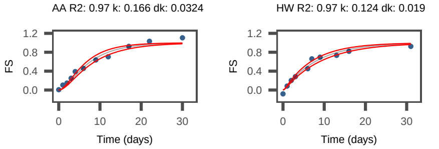

CBR1 − GVHAEEGWPNSAYGVTK_2

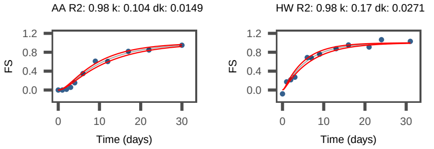

CATA − SALEHSVQCAVDVK_3

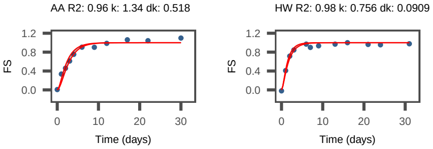

CATD_HUMAN,sp|P18242|CATD(Non−Unique) − ISVNNVLPVFDNLMQQK_3

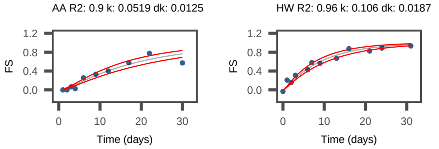

CBR4 − TMIQQGGSIVNVGSIIGLK_2

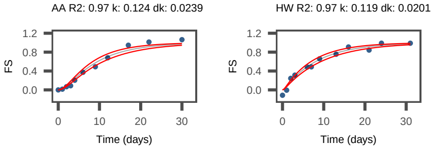

CATB − SCEAGYSPSYK_2

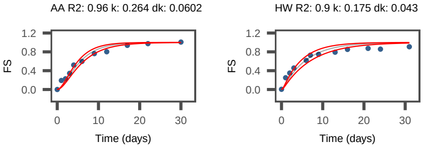

CATF − ACLGGLPSNAYAAIK_2

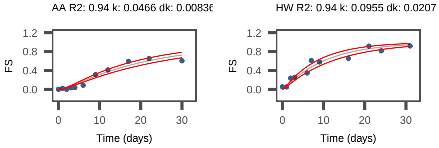

CGL − AVVLPISLATTFK_2

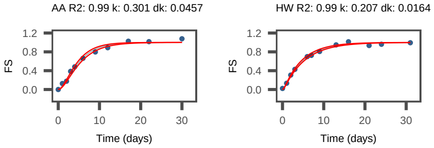

CATB − SWNLDWGDNGFFK_2

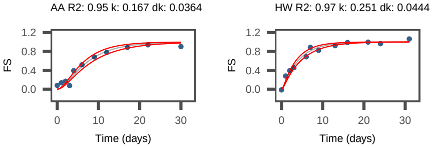

CATZ − HGIPDETCNNYQAK_2

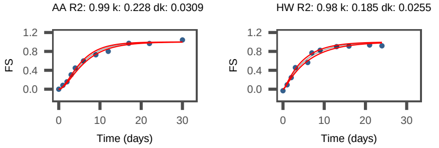

CGL − LLEAAITPQTK_2

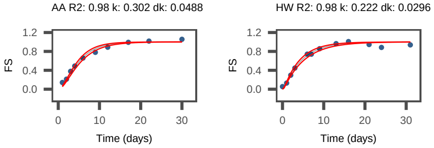

CATD − DPEGQPGGELMLGGTDSK_2

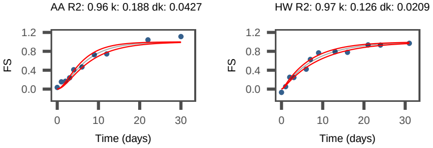

CATZ − HGIPDETCNNYQAK_3

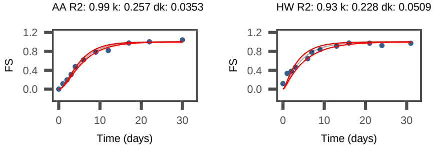

CGL − VVYPGLPSHPQHELAK_2

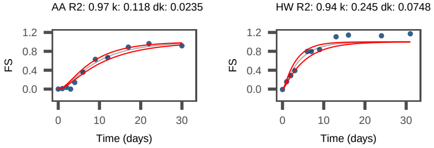

CGL − VVYPGLPSHPQHELAK_4

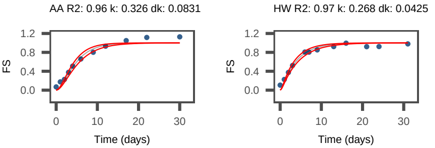

CH60 − GVMLAVDAVIAELK_3

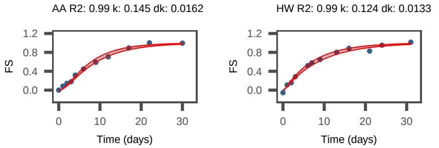

CH60 − VGGTSDVEVNEK_2

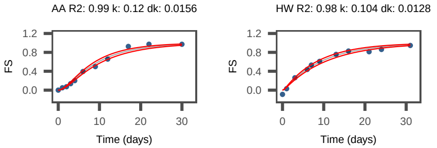

CH10 − VLLPEYGGTK_2

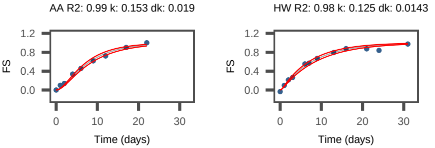

CH60 − ILQSSSEVGYDAMLGDFVNMVEK_2

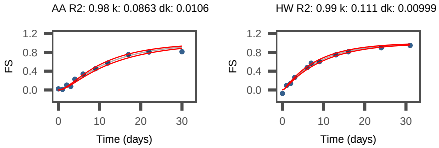

CHDH − ELQPGSHVQSDK_2

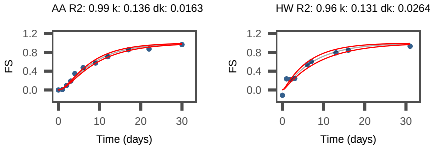

CH10 − VVLDDKDYFLFR_3

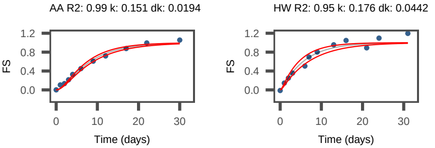

CH60 − ILQSSSEVGYDAMLGDFVNMVEK_3

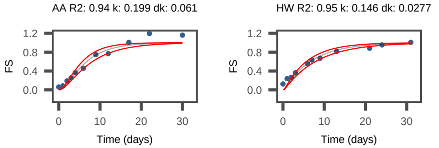

CHDH − KPTQQEAYQVHVGTMR_3

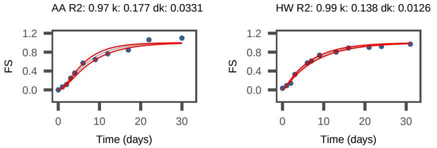

CH60 − CEFQDAYVLLSEK_2

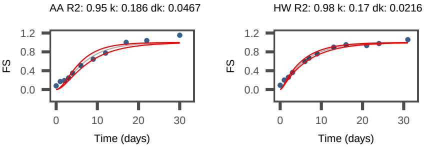

CH60 − NAGVEGSLIVEK_2

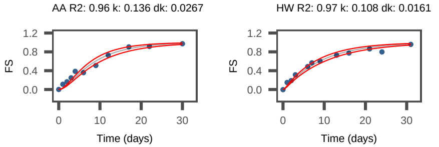

CHDH − SSDPTAVVDAQTK_2

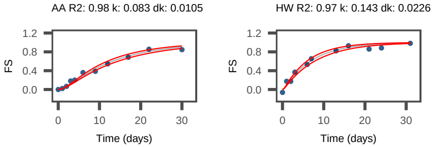

CH60 − CEFQDAYVLLSEK_3

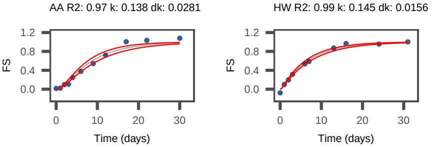

CH60 − TALLDAAGVASLLTTAEAVVTEIPK_2

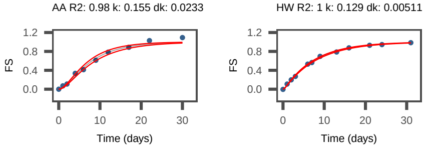

CISD1 − VVHAFDMEDLGDK_3

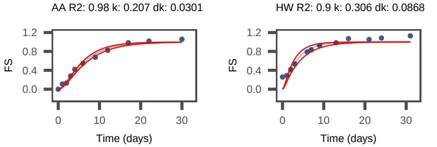

CH60 − DIGNIISDAMK_2

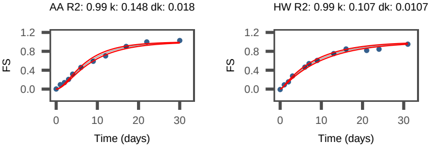

CH60 − VGEVIVTK_2

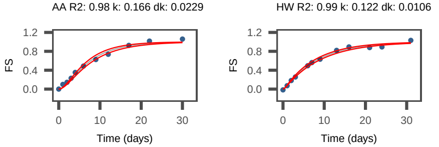

CISY − ALGFPLERPK_3

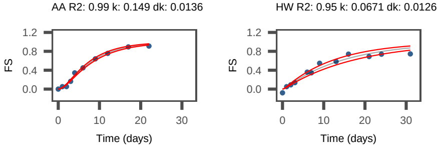

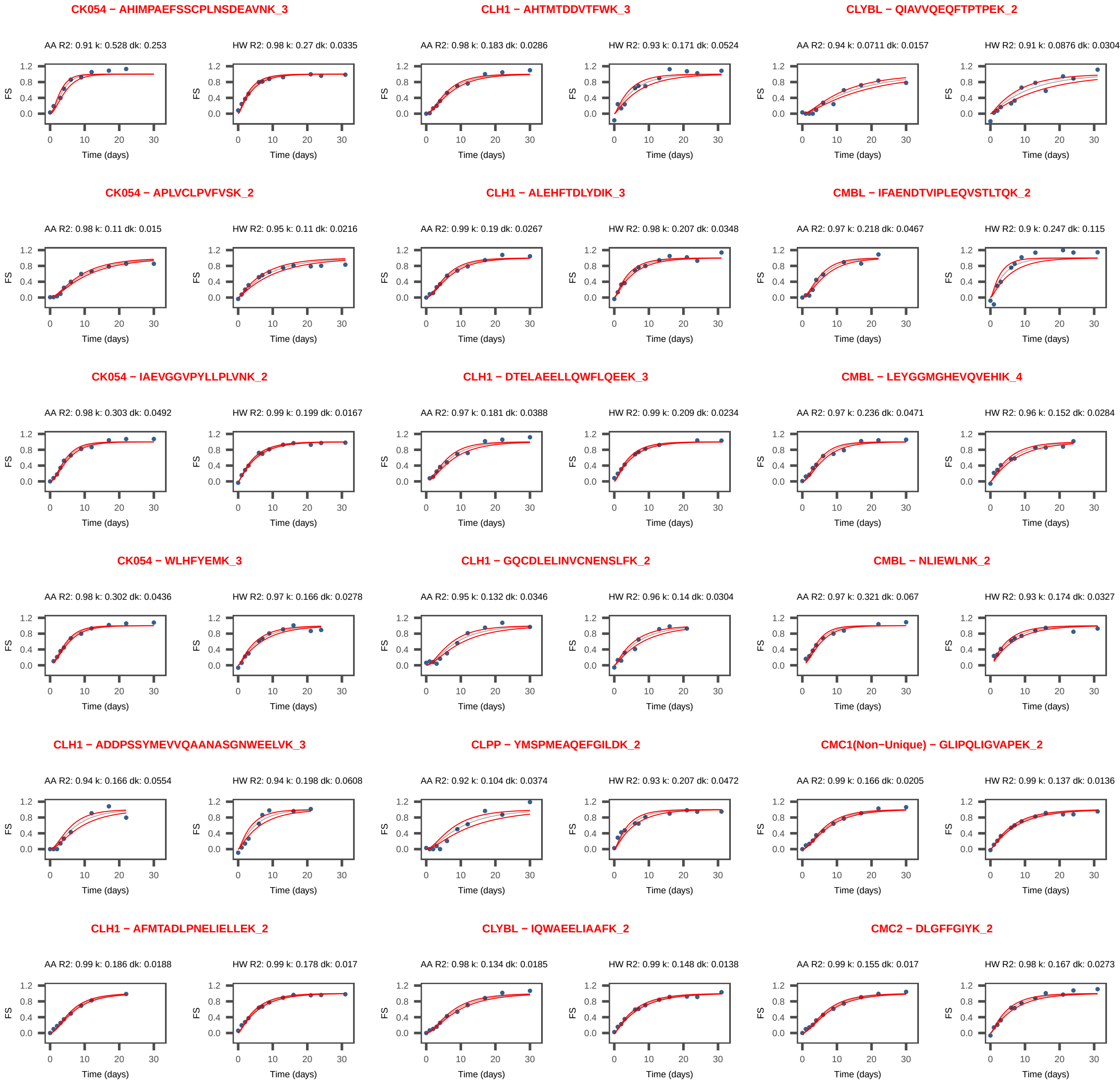
CK054 − AHIMPAEFSSCPLNSDEAVNK_3
AA R2: 0.91 k: 0.528 dk: 0.253
HW R2: 0.98 k: 0.27 dk: 0.0335
CLH1 − AHTMTDDVTFWK_3
AA R2: 0.98 k: 0.183 dk: 0.0286
HW R2: 0.93 k: 0.171 dk: 0.0524
CLYBL − QIAVVQEQFTPTPEK_2
AA R2: 0.94 k: 0.0711 dk: 0.0157
HW R2: 0.91 k: 0.0876 dk: 0.0304
CK054 − APLVCLPVFVSK_2
AA R2: 0.98 k: 0.11 dk: 0.015
HW R2: 0.95 k: 0.11 dk: 0.0216
CLH1 − ALEHFTDLYDIK_3
AA R2: 0.99 k: 0.19 dk: 0.0267
HW R2: 0.98 k: 0.207 dk: 0.0348
CMBL − IFAENDTVIPLEQVSTLTQK_2
AA R2: 0.97 k: 0.218 dk: 0.0467
HW R2: 0.9 k: 0.247 dk: 0.115
CK054 − IAEVGGVPYLLPLVNK_2
AA R2: 0.98 k: 0.303 dk: 0.0492
HW R2: 0.99 k: 0.199 dk: 0.0167
CLH1 − DTELAEELLQWFLQEEK_3
AA R2: 0.97 k: 0.181 dk: 0.0388
HW R2: 0.99 k: 0.209 dk: 0.0234
CMBL − LEYGGMGHEVQVEHIK_4
AA R2: 0.97 k: 0.236 dk: 0.0471
HW R2: 0.96 k: 0.152 dk: 0.0284
CK054 − WLHFYEMK_3
AA R2: 0.98 k: 0.302 dk: 0.0436
HW R2: 0.97 k: 0.166 dk: 0.0278
CLH1 − GQCDLELINVCNENSLFK_2
AA R2: 0.95 k: 0.132 dk: 0.0346
HW R2: 0.96 k: 0.14 dk: 0.0304
CMBL − NLIEWLNK_2
AA R2: 0.97 k: 0.321 dk: 0.067
HW R2: 0.93 k: 0.174 dk: 0.0327
CLH1 − ADDPSSYMEVVQAANASGNWEELVK_3
AA R2: 0.94 k: 0.166 dk: 0.0554
HW R2: 0.94 k: 0.198 dk: 0.0608
CLPP − YMSPMEAQEFGILDK_2
AA R2: 0.92 k: 0.104 dk: 0.0374
HW R2: 0.93 k: 0.207 dk: 0.0472
CMC1(Non−Unique) − GLIPQLIGVAPEK_2
AA R2: 0.99 k: 0.166 dk: 0.0205
HW R2: 0.99 k: 0.137 dk: 0.0136
CLH1 − AFMTADLPNELIELLEK_2
AA R2: 0.99 k: 0.186 dk: 0.0188
HW R2: 0.99 k: 0.178 dk: 0.017
CLYBL − IQWAEELIAAFK_2
AA R2: 0.98 k: 0.134 dk: 0.0185
HW R2: 0.99 k: 0.148 dk: 0.0138
CMC2 − DLGFFGIYK_2
AA R2: 0.99 k: 0.155 dk: 0.017
HW R2: 0.98 k: 0.167 dk: 0.0273
FS
Time (days)

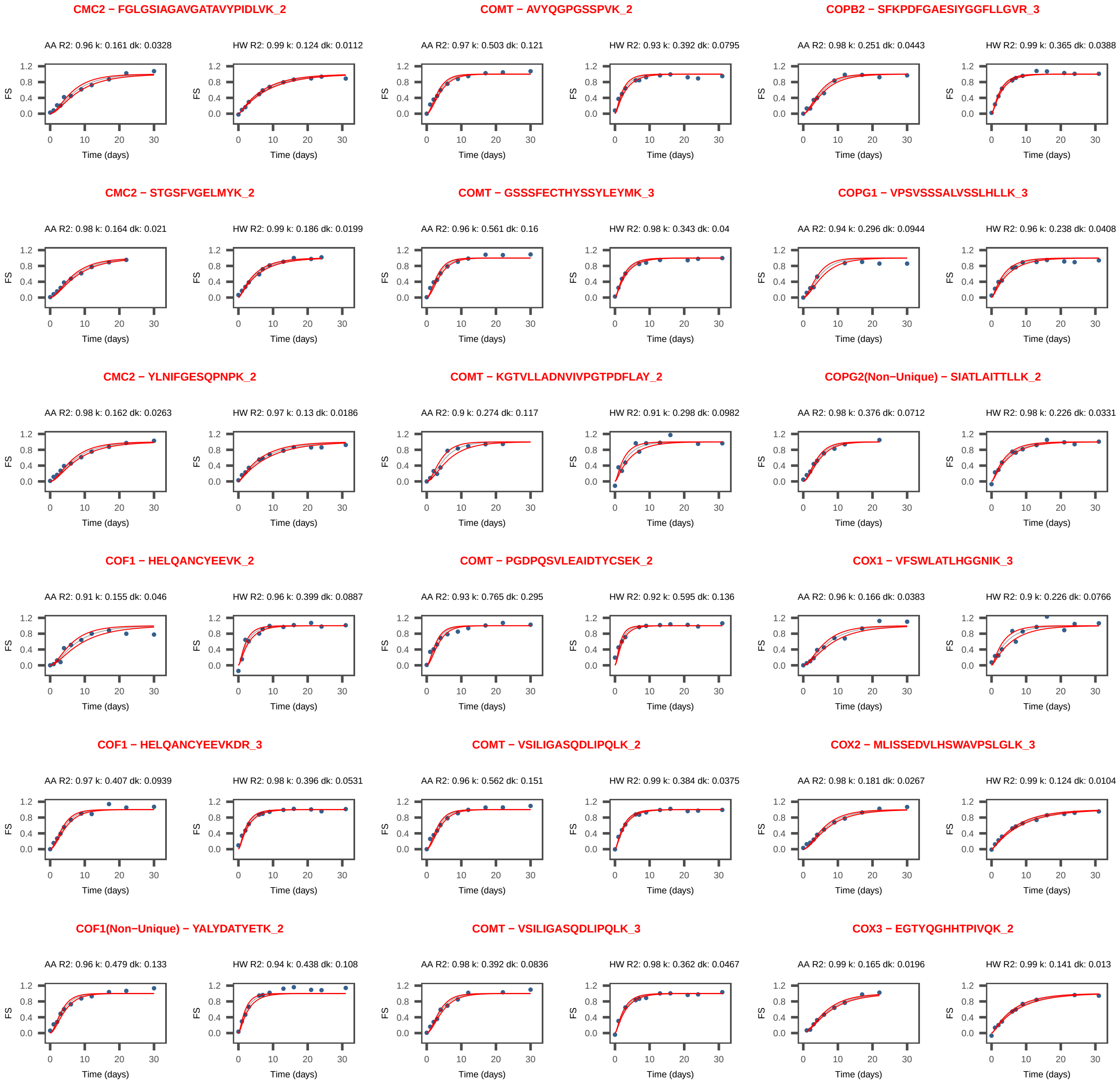
CMC2 − FGLGSIAGAVGATAVYPIDLVK_2
AA R2: 0.96 k: 0.161 dk: 0.0328
HW R2: 0.99 k: 0.124 dk: 0.0112
COMT − AVYQGPGSSPVK_2
AA R2: 0.97 k: 0.503 dk: 0.121
HW R2: 0.93 k: 0.392 dk: 0.0795
COPB2 − SFKPDFGAESIYGGFLLGVR_3
AA R2: 0.98 k: 0.251 dk: 0.0443
HW R2: 0.99 k: 0.365 dk: 0.0388
CMC2 − STGSFVGELMYK_2
AA R2: 0.98 k: 0.164 dk: 0.021
HW R2: 0.99 k: 0.186 dk: 0.0199
COMT − GSSSFECTHYSSYLEYMK_3
AA R2: 0.96 k: 0.561 dk: 0.16
HW R2: 0.98 k: 0.343 dk: 0.04
COPG1 − VPSVSSSALVSSLHLLK_3
AA R2: 0.94 k: 0.296 dk: 0.0944
HW R2: 0.96 k: 0.238 dk: 0.0408
CMC2 − YLNIFGESQPNPK_2
AA R2: 0.98 k: 0.162 dk: 0.0263
HW R2: 0.97 k: 0.13 dk: 0.0186
COMT − KGTVLLADNVIVPGTPDFLAY_2
AA R2: 0.9 k: 0.274 dk: 0.117
HW R2: 0.91 k: 0.298 dk: 0.0982
COPG2(Non−Unique) − SIATLAITTLLK_2
AA R2: 0.98 k: 0.376 dk: 0.0712
HW R2: 0.98 k: 0.226 dk: 0.0331
COF1 − HELQANCYEEVK_2
AA R2: 0.91 k: 0.155 dk: 0.046
HW R2: 0.96 k: 0.399 dk: 0.0887
COMT − PGDPQSVLEAIDTYCSEK_2
AA R2: 0.93 k: 0.765 dk: 0.295
HW R2: 0.92 k: 0.595 dk: 0.136
COX1 − VFSWLATLHGGNIK_3
AA R2: 0.96 k: 0.166 dk: 0.0383
HW R2: 0.9 k: 0.226 dk: 0.0766
COF1 − HELQANCYEEVKDR_3
AA R2: 0.97 k: 0.407 dk: 0.0939
HW R2: 0.98 k: 0.396 dk: 0.0531
COMT − VSILIGASQDLIPQLK_2
AA R2: 0.96 k: 0.562 dk: 0.151
HW R2: 0.99 k: 0.384 dk: 0.0375
COX2 − MLISSEDVLHSWAVPSLGLK_3
AA R2: 0.98 k: 0.181 dk: 0.0267
HW R2: 0.99 k: 0.124 dk: 0.0104
COF1(Non−Unique) − YALYDATYETK_2
AA R2: 0.96 k: 0.479 dk: 0.133
HW R2: 0.94 k: 0.438 dk: 0.108
COMT − VSILIGASQDLIPQLK_3
AA R2: 0.98 k: 0.392 dk: 0.0836
HW R2: 0.98 k: 0.362 dk: 0.0467
COX3 − EGTYQGHHTPIVQK_2
AA R2: 0.99 k: 0.165 dk: 0.0196
HW R2: 0.99 k: 0.141 dk: 0.013
FS
Time (days)

COX41 − DYPLPDVAHVTMLSASQK_2

AA R2: 0.92 k: 0.236 dk: 0.0789 HW R2: 0.98 k: 0.0566 dk: 0.0058

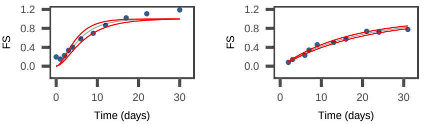

COX41 − DYPLPDVAHVTMLSASQK_3

AA R2: 0.98 k: 0.177 dk: 0.0277 HW R2: 0.99 k: 0.122 dk: 0.01

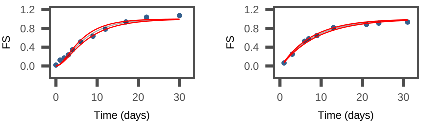

COX5A − VIQELRPTLNELGISTPEELGLDKV_3

AA R2: 0.92 k: 0.243 dk: 0.0946 HW R2: 0.99 k: 0.129 dk: 0.00953

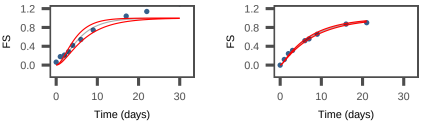

COX5A − WVTYFNKPDIDAWELR_3

AA R2: 0.95 k: 0.147 dk: 0.0391 HW R2: 0.94 k: 0.15 dk: 0.0305

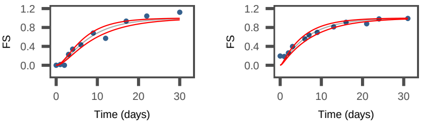

COX6C − NYDSMKDFEEMR_2

AA R2: 0.97 k: 0.113 dk: 0.02 HW R2: 0.94 k: 0.139 dk: 0.0415

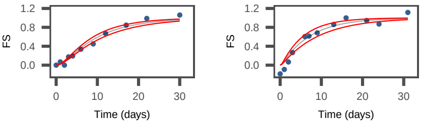

COX6C − NYDSMKDFEEMR_3

AA R2: 0.98 k: 0.142 dk: 0.023 HW R2: 0.99 k: 0.141 dk: 0.015

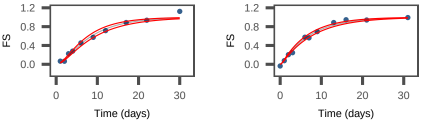

CP1A1(Non−Unique) − IGSTPVVVLSGLNTIK_2

AA R2: 0.91 k: 3.29 dk: 2.26 HW R2: 0.92 k: 0.554 dk: 0.132

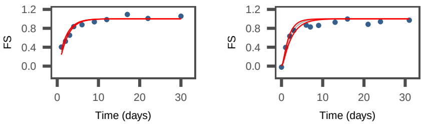

CP237(Non−Unique) − DFLNLMEK_2

AA R2: 0.96 k: 1.67 dk: 0.718 HW R2: 0.98 k: 0.604 dk: 0.0902

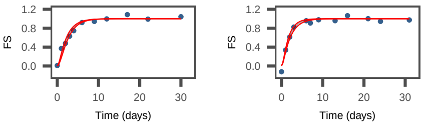

CP237(Non−Unique) − DRDFLNLMEK_3

AA R2: 0.92 k: 0.997 dk: 0.449 HW R2: 0.92 k: 0.815 dk: 0.256

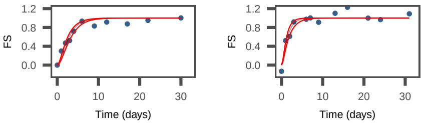

CP237(Non−Unique) − LPPGPTPLPIIGNILQIDVK_3

AA R2: 0.94 k: 0.496 dk: 0.217 HW R2: 0.95 k: 0.443 dk: 0.085

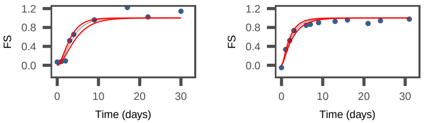

CP254 − DRDFLNLLEK_3

AA R2: 0.97 k: 0.43 dk: 0.101 HW R2: 0.93 k: 0.454 dk: 0.15

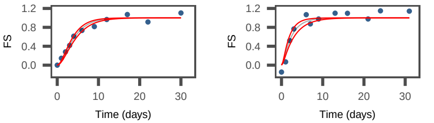

CP27A − DFAHMPLLK_3

AA R2: 0.98 k: 0.411 dk: 0.0895 HW R2: 0.92 k: 0.241 dk: 0.0671

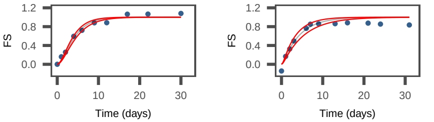

CP27A − DHMDQWK_2

AA R2: 0.98 k: 0.263 dk: 0.0422 HW R2: 0.96 k: 0.323 dk: 0.0752

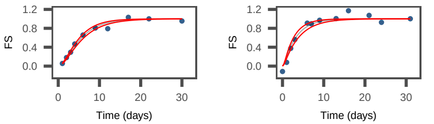

CP27A − SPEIQEALHK_3

AA R2: 0.99 k: 0.49 dk: 0.0703 HW R2: 0.94 k: 0.306 dk: 0.0622

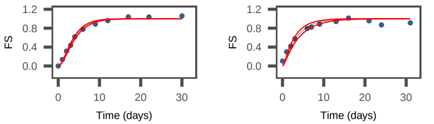

CP2AC − DFIDSFLIHMQK_3

AA R2: 0.95 k: 1.3 dk: 0.551 HW R2: 0.96 k: 0.713 dk: 0.13

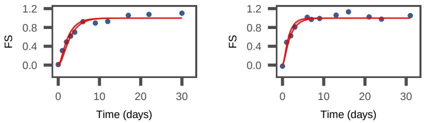

CP2AC − FAASPTGQLYDMFHSVMK_3

AA R2: 0.94 k: 1.51 dk: 0.747 HW R2: 0.92 k: 0.492 dk: 0.119

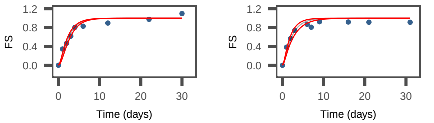

CP2CT − FIDLLPTSLPHAVTCDIK_3

AA R2: 0.96 k: 1.49 dk: 0.663 HW R2: 0.98 k: 0.744 dk: 0.0874

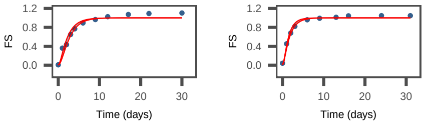

CP2CT − GTTVITSLSSVLHDSK_3

AA R2: 0.96 k: 1.51 dk: 0.608 HW R2: 0.97 k: 0.585 dk: 0.0837

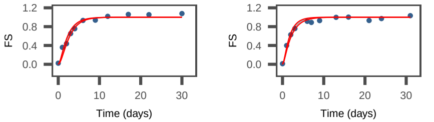

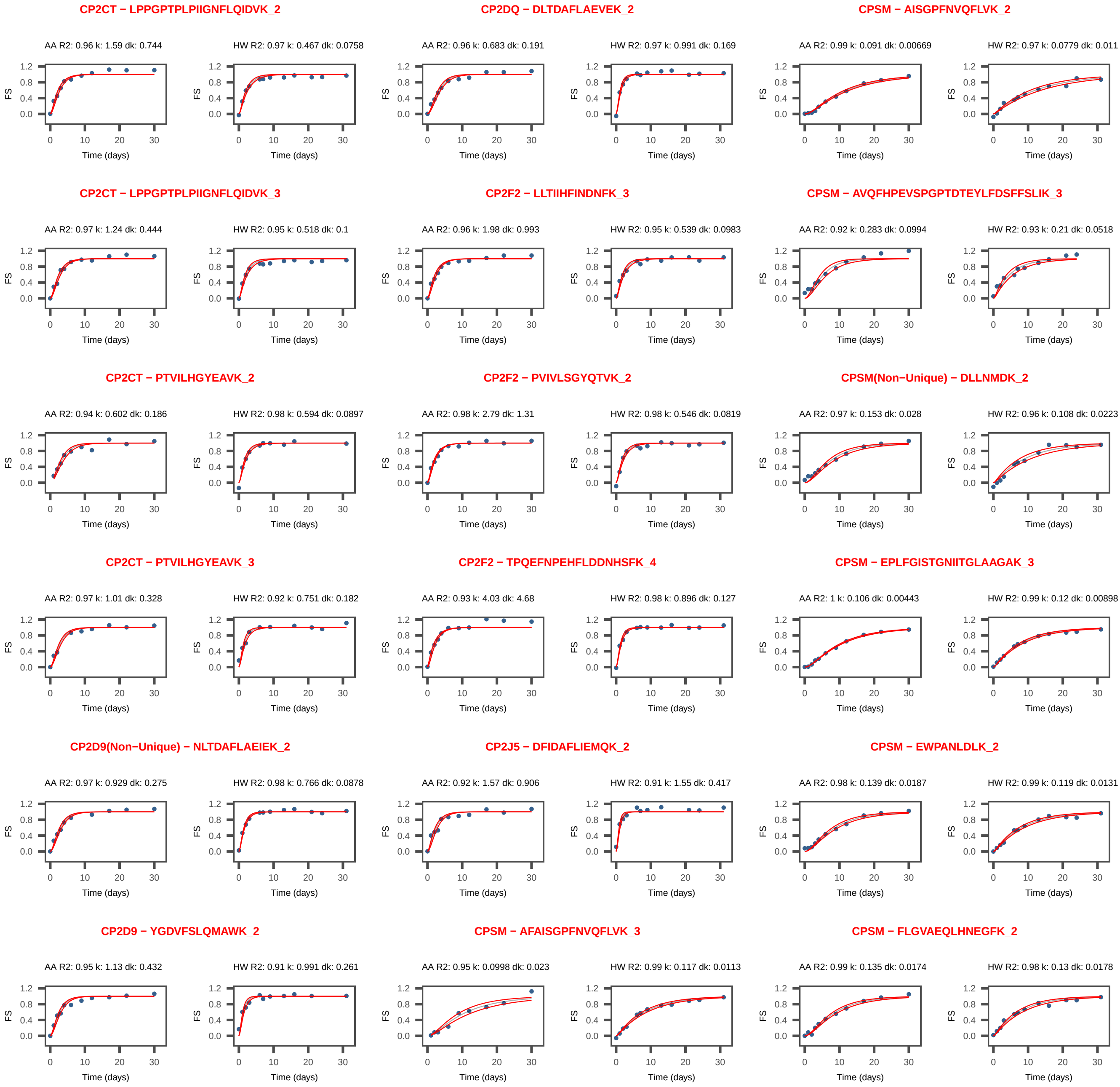
CP2CT − LPPGPTPLPIIGNFLQIDVK_2
AA R2: 0.96 k: 1.59 dk: 0.744
HW R2: 0.97 k: 0.467 dk: 0.0758
CP2DQ − DLTDAFLAEVEK_2
AA R2: 0.96 k: 0.683 dk: 0.191
HW R2: 0.97 k: 0.991 dk: 0.169
CPSM − AISGPFNVQFLVK_2
AA R2: 0.99 k: 0.091 dk: 0.00669
HW R2: 0.97 k: 0.0779 dk: 0.011
CP2CT − LPPGPTPLPIIGNFLQIDVK_3
AA R2: 0.97 k: 1.24 dk: 0.444
HW R2: 0.95 k: 0.518 dk: 0.1
CP2F2 − LLTIIHFINDNFK_3
AA R2: 0.96 k: 1.98 dk: 0.993
HW R2: 0.95 k: 0.539 dk: 0.0983
CPSM − AVQFHPEVSPGPTDTEYLFDSFFSLIK_3
AA R2: 0.92 k: 0.283 dk: 0.0994
HW R2: 0.93 k: 0.21 dk: 0.0518
CP2CT − PTVILHGYEAVK_2
AA R2: 0.94 k: 0.602 dk: 0.186
HW R2: 0.98 k: 0.594 dk: 0.0897
CP2F2 − PVIVLSGYQTVK_2
AA R2: 0.98 k: 2.79 dk: 1.31
HW R2: 0.98 k: 0.546 dk: 0.0819
CPSM(Non−Unique) − DLLNMDK_2
AA R2: 0.97 k: 0.153 dk: 0.028
HW R2: 0.96 k: 0.108 dk: 0.0223
CP2CT − PTVILHGYEAVK_3
AA R2: 0.97 k: 1.01 dk: 0.328
HW R2: 0.92 k: 0.751 dk: 0.182
CP2F2 − TPQEFNPEHFLDDNHSFK_4
AA R2: 0.93 k: 4.03 dk: 4.68
HW R2: 0.98 k: 0.896 dk: 0.127
CPSM − EPLFGISTGNIITGLAAGAK_3
AA R2: 1 k: 0.106 dk: 0.00443
HW R2: 0.99 k: 0.12 dk: 0.00898
CP2D9(Non−Unique) − NLTDAFLAEIEK_2
AA R2: 0.97 k: 0.929 dk: 0.275
HW R2: 0.98 k: 0.766 dk: 0.0878
CP2J5 − DFIDAFLIEMQK_2
AA R2: 0.92 k: 1.57 dk: 0.906
HW R2: 0.91 k: 1.55 dk: 0.417
CPSM − EWPANLDLK_2
AA R2: 0.98 k: 0.139 dk: 0.0187
HW R2: 0.99 k: 0.119 dk: 0.0131
CP2D9 − YGDVFSLQMAWK_2
AA R2: 0.95 k: 1.13 dk: 0.432
HW R2: 0.91 k: 0.991 dk: 0.261
CPSM − AFAISGPFNVQFLVK_3
AA R2: 0.95 k: 0.0998 dk: 0.023
HW R2: 0.99 k: 0.117 dk: 0.0113
CPSM − FLGVAEQLHNEGFK_2
AA R2: 0.99 k: 0.135 dk: 0.0174
HW R2: 0.98 k: 0.13 dk: 0.0178
FS
Time (days)

CPSM − FLPITPQFVTEVIK_2

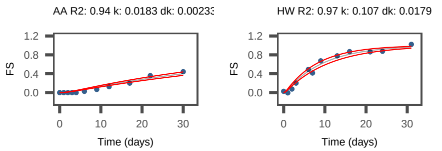

CPSM − NLAVPLYK_2

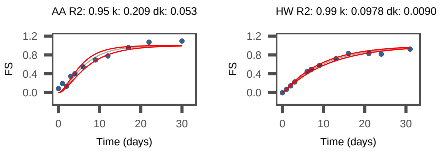

CPSM − RLPTLEQPIIPSDYVAIK_2

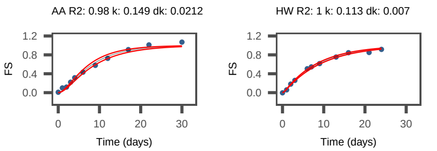

CPSM − GTTITSVLPK_2

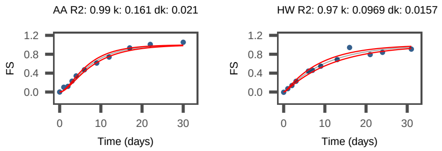

CPSM − PSYVLSGSAMNVVFSEDEMK_2

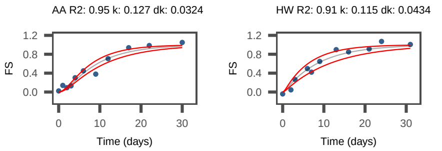

CPSM − RLPTLEQPIIPSDYVAIK_3

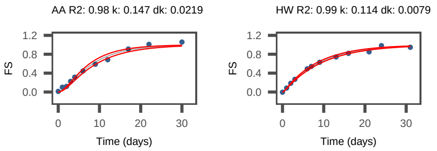

CPSM − IALGIPLPEIK_2

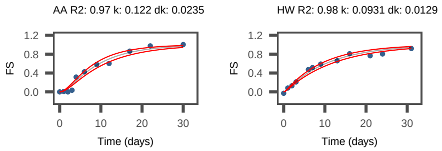

CPSM − PSYVLSGSAMNVVFSEDEMKR_2

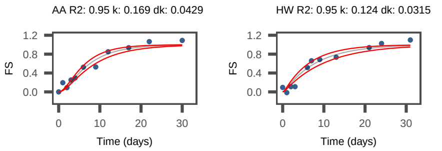

CPSM − SIGQAGEFDYSGSQAVK_2

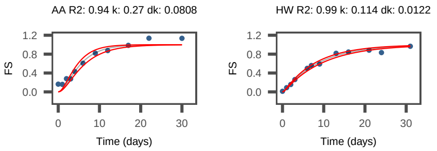

CPSM − IAPSFAVESMEDALK_3

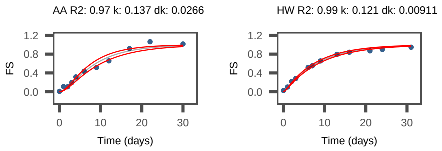

CPSM − PSYVLSGSAMNVVFSEDEMKR_3

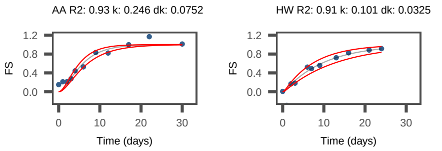

CPSM − SLGQWLQEEK_2

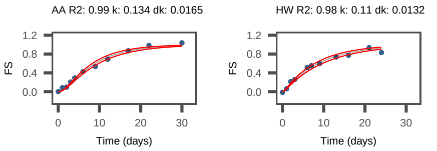

CPSM − IASVQTNEVGLK_2

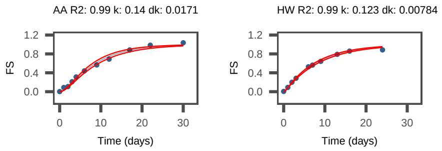

CPSM − QAFITAQNHGYALDNTLPAGWK_2

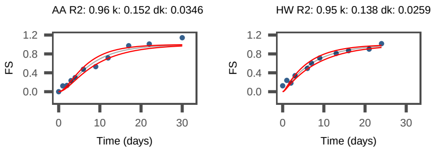

CPSM − TAHIVLEDGTK_3

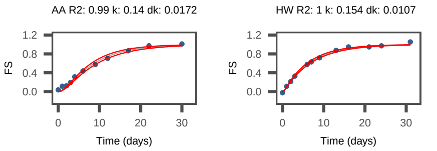

CPSM − IEFEGQSVDFVDPNK_2

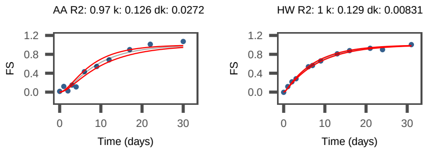

CPSM − QNLIAEVSTK_2

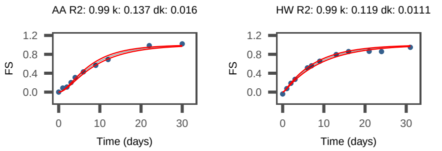

CPSM − TAVDSGIALLTNFQVTK_2

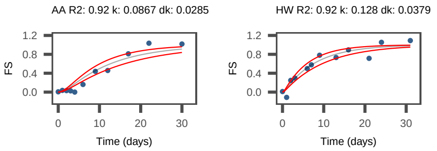

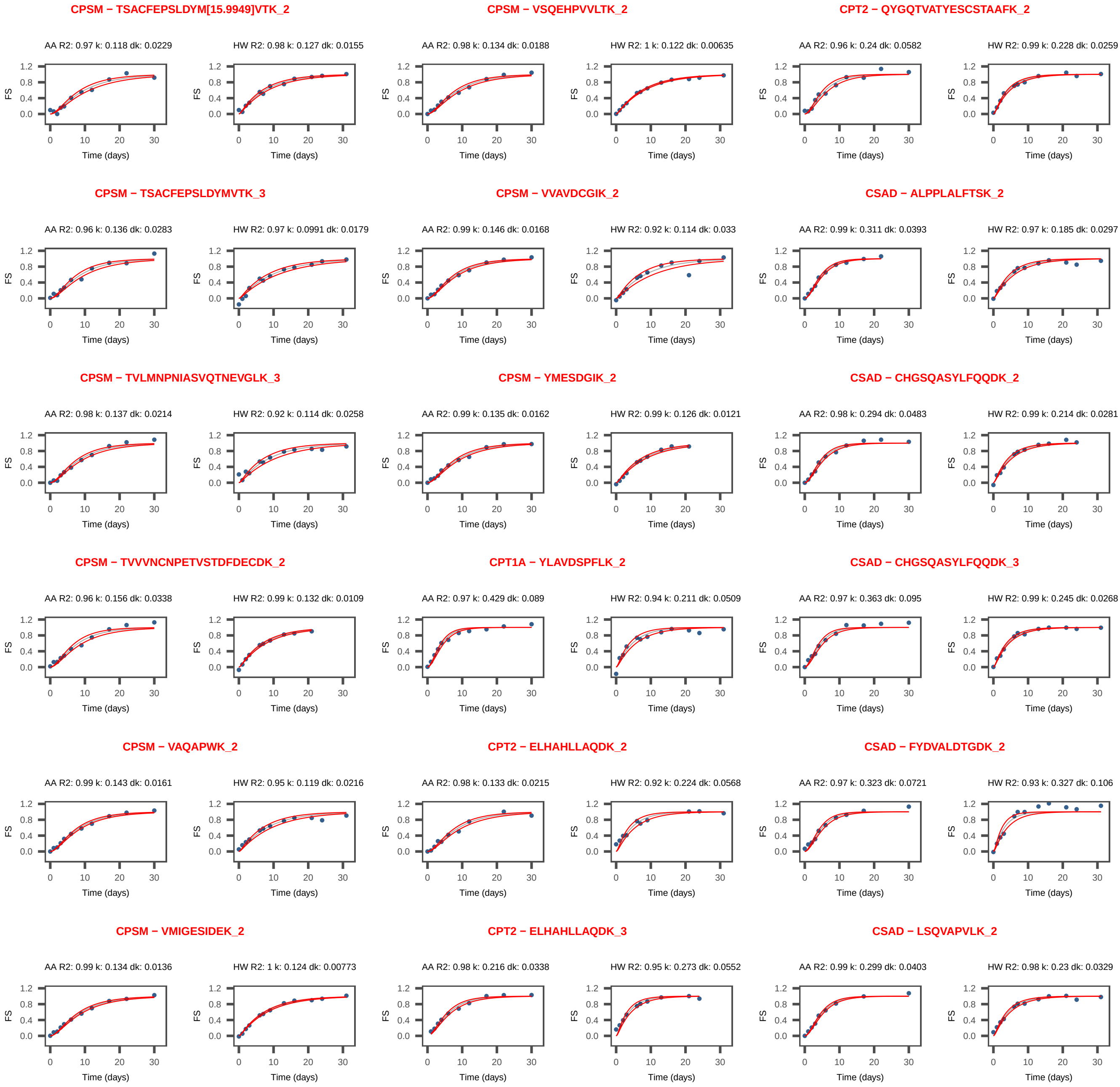
CPSM – TSACFEPSLDYM[15.9949]VTK_2
AA R2: 0.97 k: 0.118 dk: 0.0229
HW R2: 0.98 k: 0.127 dk: 0.0155
CPSM – VSQEHPVVLTK_2
AA R2: 0.98 k: 0.134 dk: 0.0188
HW R2: 1 k: 0.122 dk: 0.00635
CPT2 – QYGQTVATYESCSTAAFK_2
AA R2: 0.96 k: 0.24 dk: 0.0582
HW R2: 0.99 k: 0.228 dk: 0.0259
CPSM – TSACFEPSLDYMVTK_3
AA R2: 0.96 k: 0.136 dk: 0.0283
HW R2: 0.97 k: 0.0991 dk: 0.0179
CPSM – VVAVDCGIK_2
AA R2: 0.99 k: 0.146 dk: 0.0168
HW R2: 0.92 k: 0.114 dk: 0.033
CSAD – ALPPLALFTSK_2
AA R2: 0.99 k: 0.311 dk: 0.0393
HW R2: 0.97 k: 0.185 dk: 0.0297
CPSM – TVLMNPNIASVQTNEVGLK_3
AA R2: 0.98 k: 0.137 dk: 0.0214
HW R2: 0.92 k: 0.114 dk: 0.0258
CPSM – YMESDGIK_2
AA R2: 0.99 k: 0.135 dk: 0.0162
HW R2: 0.99 k: 0.126 dk: 0.0121
CSAD – CHGSQASYLFQQDK_2
AA R2: 0.98 k: 0.294 dk: 0.0483
HW R2: 0.99 k: 0.214 dk: 0.0281
CPSM – TVVVNCNPETVSTDFDECDK_2
AA R2: 0.96 k: 0.156 dk: 0.0338
HW R2: 0.99 k: 0.132 dk: 0.0109
CPT1A – YLAVDSPFLK_2
AA R2: 0.97 k: 0.429 dk: 0.089
HW R2: 0.94 k: 0.211 dk: 0.0509
CSAD – CHGSQASYLFQQDK_3
AA R2: 0.97 k: 0.363 dk: 0.095
HW R2: 0.99 k: 0.245 dk: 0.0268
CPSM – VAQAPWK_2
AA R2: 0.99 k: 0.143 dk: 0.0161
HW R2: 0.95 k: 0.119 dk: 0.0216
CPT2 – ELHAHLLAQDK_2
AA R2: 0.98 k: 0.133 dk: 0.0215
HW R2: 0.92 k: 0.224 dk: 0.0568
CSAD – FYDVALDTGDK_2
AA R2: 0.97 k: 0.323 dk: 0.0721
HW R2: 0.93 k: 0.327 dk: 0.106
CPSM – VMIGESIDEK_2
AA R2: 0.99 k: 0.134 dk: 0.0136
HW R2: 1 k: 0.124 dk: 0.00773
CPT2 – ELHAHLLAQDK_3
AA R2: 0.98 k: 0.216 dk: 0.0338
HW R2: 0.95 k: 0.273 dk: 0.0552
CSAD – LSQVAPVLK_2
AA R2: 0.99 k: 0.299 dk: 0.0403
HW R2: 0.98 k: 0.23 dk: 0.0329
FS
Time (days)

CSN2 − AHTDFFEAFK_3

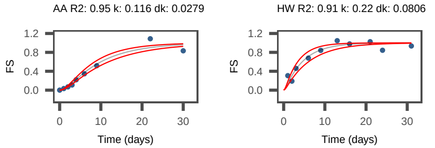

CSN4 − LYNNITFEELGALLEIPAAK_2

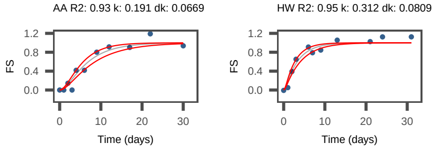

CSRP1 − HEEAPGHRPTTNPNASK_4

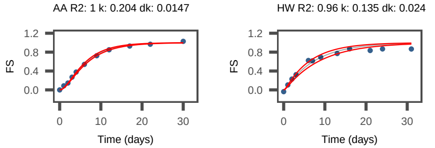

CTND1 − DMDLTEVITGTLWNLSSHDSIK_3

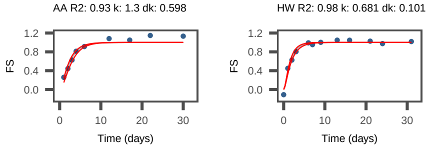

CTND1 − GIPILVGLLDHPK_3

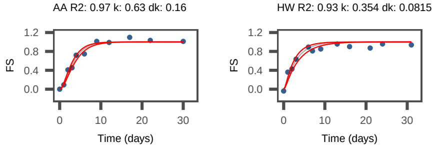

CX6A1 − VALPGVGVSMLNVFLK_2

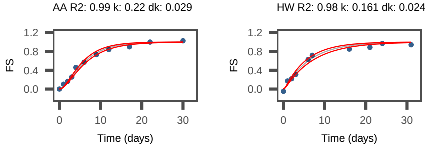

CY1 − ALAEEVEVQDGPNDDGEMFMRPGK_3

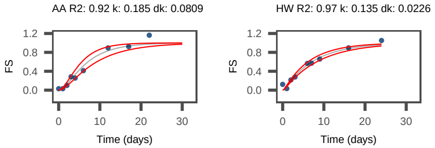

CY1 − HLVGVCYTEEEAK_2

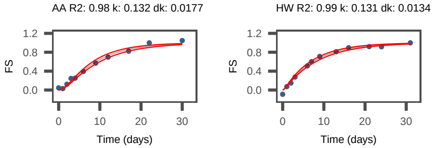

CYB5 − STWVILHHK_2

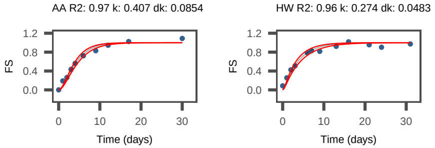

CYB5 − STWVILHHK_3

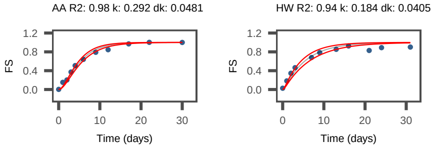

CYC − GITWGEDTLMEYLENPK_2

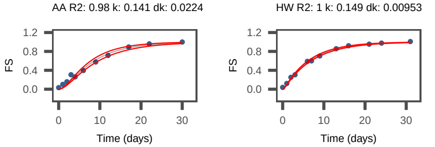

CYC − GITWGEDTLMEYLENPK_3

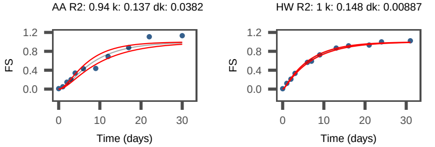

CYC − TGQAAGFSYTDANK_2

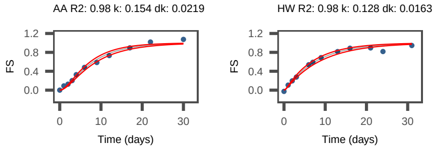

CYC_HORSE,sp|P00015|CYC2(Non−Unique) − MIFAGIK_2

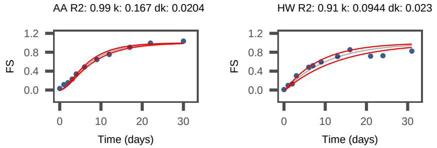

DCXR − AVIQVSQIVAK_2

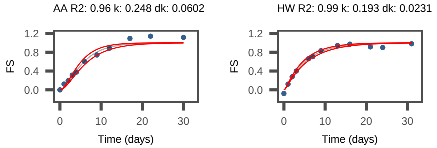

DCXR − MMALELGPHK_3

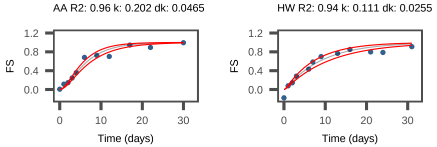

DDAH1 − SFCSMAGPNLIAIGSSESAQK_2

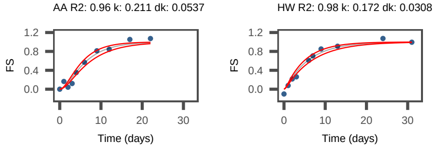

DDB1 − DLLFILTAK_2

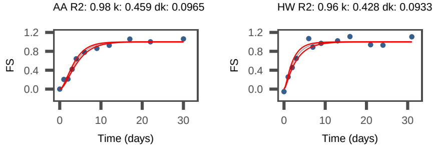

DDX1 − DLGLAFEIPAHIK_3

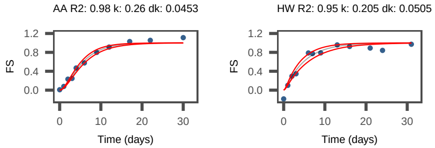

DDX3L(Non−Unique) − SFLLDLLNATGK_2

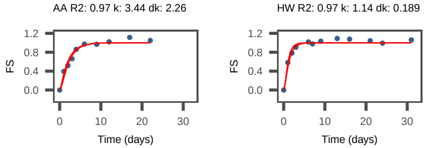

DECR − FDGGEEVFLSGEFNSLK_2

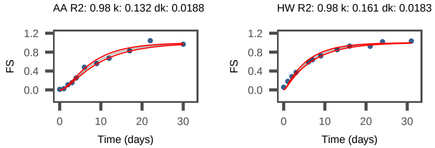

DECR − VAFITGGGTGLGK_2

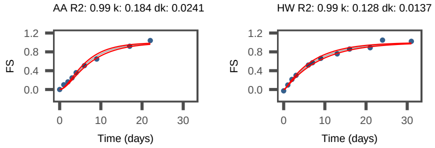

DECR − VTKEEWDIIEGLIR_3

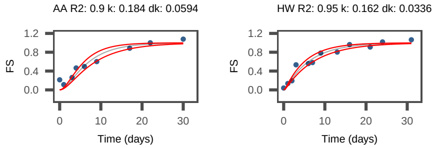

DEST − EILVGDVGATITDPFK_2

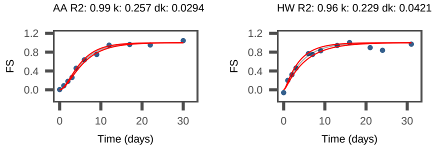

DEST − HFVGMLPEK_2

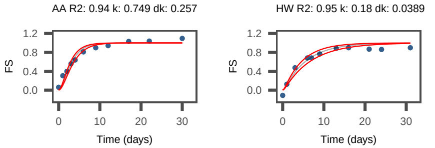

DHB12 − AFVDFFSQCLHEEYK_3

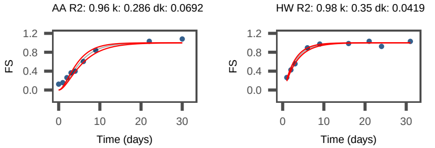

DHB13 − LVLWDINKR_3

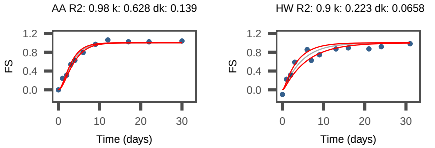

DHB4 − AVANYDSVEAGEK_2

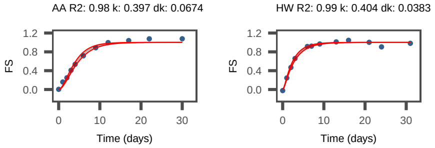

DHB4 − FVYEGSADFSCLPTFGVIVAQK_2

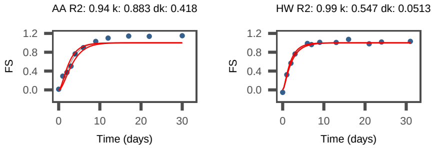

DHB4 − PQTIQESTGGIVEVLHK_3

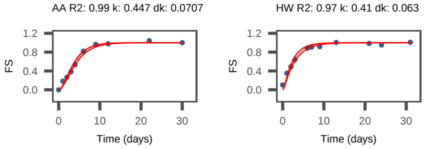

DHB4 − PVYPGQTLQTEMWK_2

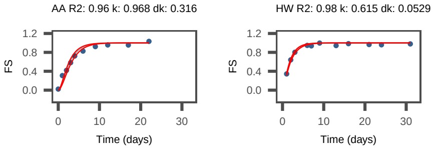

DHB4 − TSHAAPAATSGFVGAVGHK_2

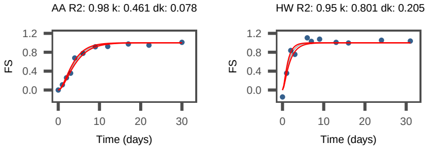

DHB4 − TSHAAPAATSGFVGAVGHK_3

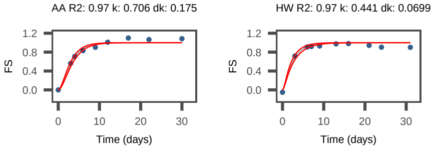

DHB5(Non−Unique) − EDIFYTSK_2

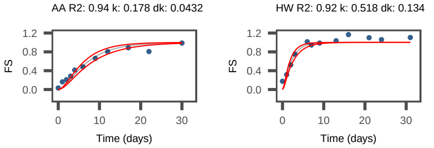

DHB5 − ENMQVFEFQLTSEDMK_2

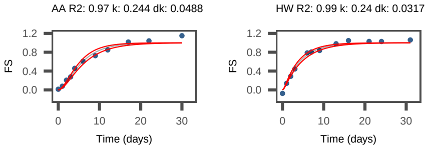

DHB5 − HIDSASMYQNEK_2

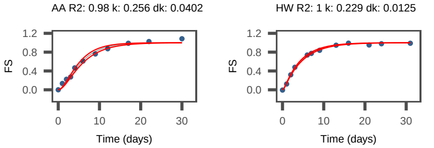

DHB5 − HIDSASMYQNEK_3

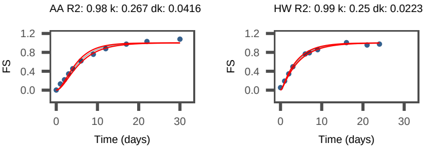

DHB5(Non−Unique) − YKPVCNQVECHPY_2

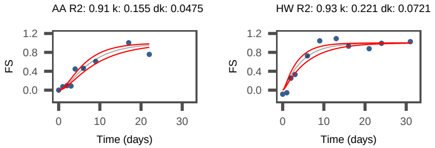

DHE3_BOVIN,sp|P26443|DHE3(Non−Unique) − FTMELAK_2

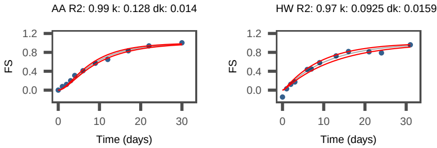

DHB5 − LSDGHFIPILGFGTYAPQEVPK_2

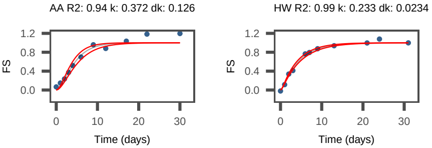

DHCR7 − AIECSYTSADGLK_2

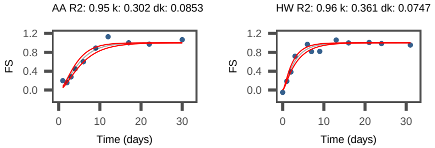

DHE3_BOVIN,sp|P26443|DHE3(Non−Unique) − NYTDNELEK_2

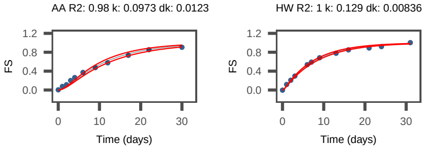

DHB5 − LSDGHFIPILGFGTYAPQEVPK_3

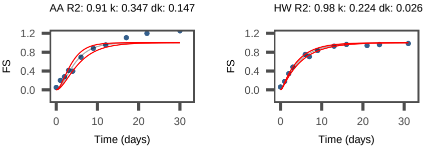

DHDH − AVLCEKPMGVNAAEVR_3

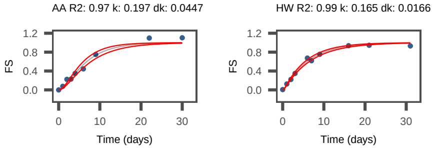

DHE3_BOVIN,sp|P26443|DHE3(Non−Unique) − SEAAADREDDPNFFK_2

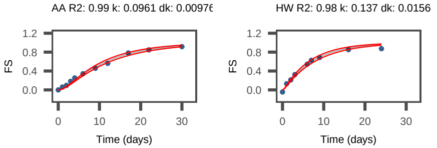

DHB5 − QLQLDYVDLYLIHFPMAMK_3

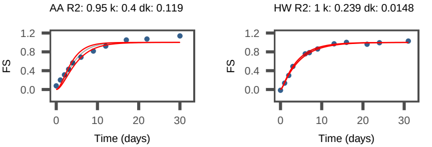

DHE3 − LQHGSILGFPK_3

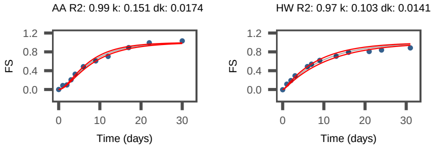

DHE3_BOVIN,sp|P26443|DHE3(Non−Unique) − VTGKPISQGGIHGR_4

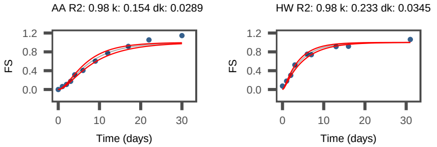

DHB5(Non−Unique) − REDIFYTSK_2

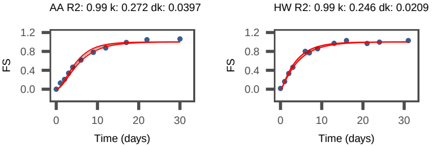

DHE3 − VYEGSILEADCDILIPAASEK_2

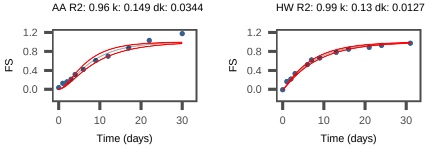

DHI1 − EISGIINAQASPK_2

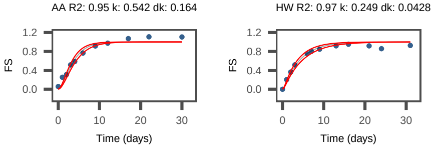

DHB5 − VCLEQSLK_2

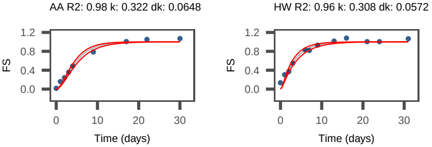

DHE3_BOVIN,sp|P26443|DHE3(Non−Unique) − CAVVDVPFGGAK_2

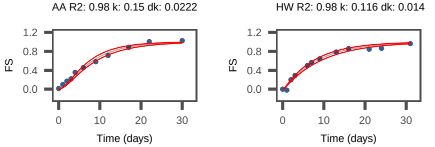

DHPR − AALDGTPGMIGYGMAK_2

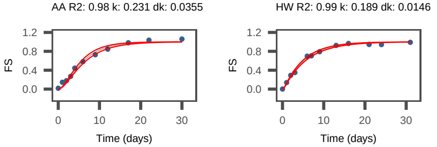

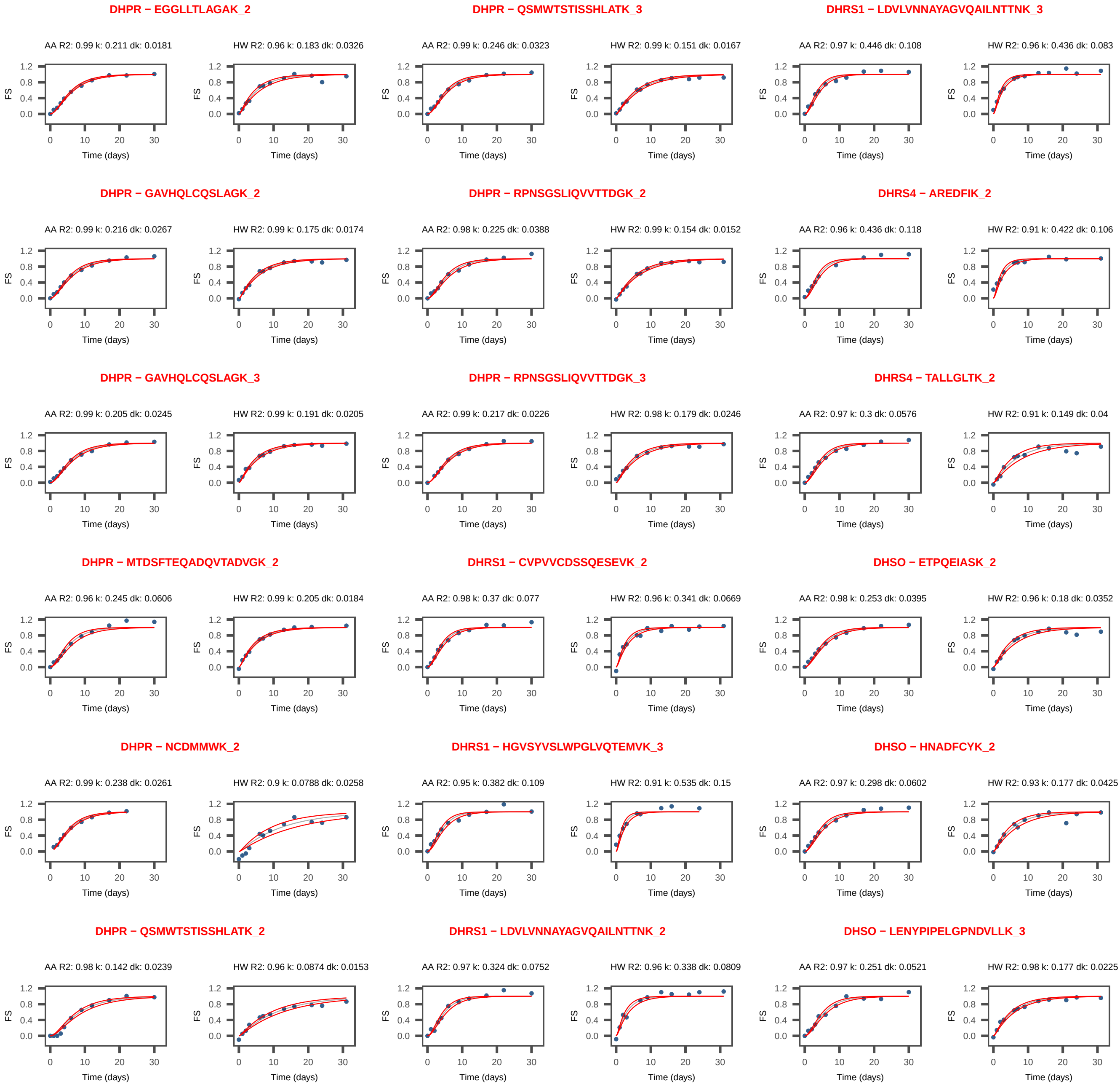
DHPR – EGGLLTLAGAK_2
AA R2: 0.99 k: 0.211 dk: 0.0181
HW R2: 0.96 k: 0.183 dk: 0.0326
DHPR – QSMWTSTISSHLATK_3
AA R2: 0.99 k: 0.246 dk: 0.0323
HW R2: 0.99 k: 0.151 dk: 0.0167
DHRS1 – LDVLVNNAYAGVQAILNTTNK_3
AA R2: 0.97 k: 0.446 dk: 0.108
HW R2: 0.96 k: 0.436 dk: 0.083
DHPR – GAVHQLCQSLAGK_2
AA R2: 0.99 k: 0.216 dk: 0.0267
HW R2: 0.99 k: 0.175 dk: 0.0174
DHPR – RPNSGSLIQVVTTDGK_2
AA R2: 0.98 k: 0.225 dk: 0.0388
HW R2: 0.99 k: 0.154 dk: 0.0152
DHRS4 – AREDFIK_2
AA R2: 0.96 k: 0.436 dk: 0.118
HW R2: 0.91 k: 0.422 dk: 0.106
DHPR – GAVHQLCQSLAGK_3
AA R2: 0.99 k: 0.205 dk: 0.0245
HW R2: 0.99 k: 0.191 dk: 0.0205
DHPR – RPNSGSLIQVVTTDGK_3
AA R2: 0.99 k: 0.217 dk: 0.0226
HW R2: 0.98 k: 0.179 dk: 0.0246
DHRS4 – TALLGLTK_2
AA R2: 0.97 k: 0.3 dk: 0.0576
HW R2: 0.91 k: 0.149 dk: 0.04
DHPR – MTDSFTEQADQVTADVGK_2
AA R2: 0.96 k: 0.245 dk: 0.0606
HW R2: 0.99 k: 0.205 dk: 0.0184
DHRS1 – CVPVVCDSSQESEVK_2
AA R2: 0.98 k: 0.37 dk: 0.077
HW R2: 0.96 k: 0.341 dk: 0.0669
DHSO – ETPQEIASK_2
AA R2: 0.98 k: 0.253 dk: 0.0395
HW R2: 0.96 k: 0.18 dk: 0.0352
DHPR – NCDMMWK_2
AA R2: 0.99 k: 0.238 dk: 0.0261
HW R2: 0.9 k: 0.0788 dk: 0.0258
DHRS1 – HGVSYVSLWPGLVQTEMVK_3
AA R2: 0.95 k: 0.382 dk: 0.109
HW R2: 0.91 k: 0.535 dk: 0.15
DHSO – HNADFCYK_2
AA R2: 0.97 k: 0.298 dk: 0.0602
HW R2: 0.93 k: 0.177 dk: 0.0425
DHPR – QSMWTSTISSHLATK_2
AA R2: 0.98 k: 0.142 dk: 0.0239
HW R2: 0.96 k: 0.0874 dk: 0.0153
DHRS1 – LDVLVNNAYAGVQAILNTTNK_2
AA R2: 0.97 k: 0.324 dk: 0.0752
HW R2: 0.96 k: 0.338 dk: 0.0809
DHSO – LENYPIPELGPNDVLLK_3
AA R2: 0.97 k: 0.251 dk: 0.0521
HW R2: 0.98 k: 0.177 dk: 0.0225
FS
Time (days)

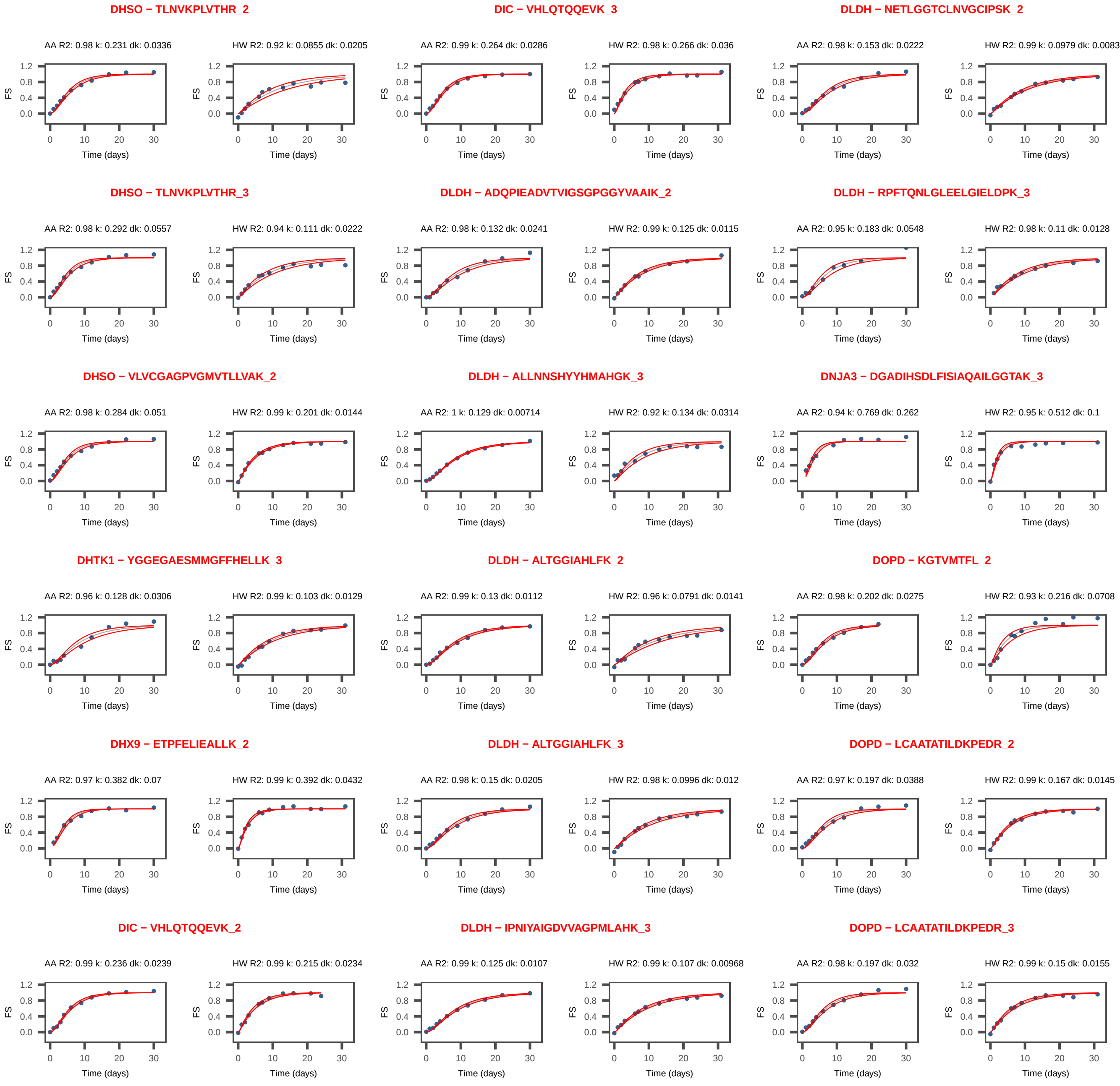
DHSO – TLNVKPLVTHR_2
AA R2: 0.98 k: 0.231 dk: 0.0336
HW R2: 0.92 k: 0.0855 dk: 0.0205
DIC – VHLQTQQEVK_3
AA R2: 0.99 k: 0.264 dk: 0.0286
HW R2: 0.98 k: 0.266 dk: 0.036
DLDH – NETLGGTCLNVGCIPSK_2
AA R2: 0.98 k: 0.153 dk: 0.0222
HW R2: 0.99 k: 0.0979 dk: 0.0083
DHSO – TLNVKPLVTHR_3
AA R2: 0.98 k: 0.292 dk: 0.0557
HW R2: 0.94 k: 0.111 dk: 0.0222
DLDH – ADQPIEADVTVIGSGPGGYVAAIK_2
AA R2: 0.98 k: 0.132 dk: 0.0241
HW R2: 0.99 k: 0.125 dk: 0.0115
DLDH – RPFTQNLGLEELGIELDPK_3
AA R2: 0.95 k: 0.183 dk: 0.0548
HW R2: 0.98 k: 0.11 dk: 0.0128
DHSO – VLVCGAGPVGMVTLLVAK_2
AA R2: 0.98 k: 0.284 dk: 0.051
HW R2: 0.99 k: 0.201 dk: 0.0144
DLDH – ALLNNSHYYHMAHGK_3
AA R2: 1 k: 0.129 dk: 0.00714
HW R2: 0.92 k: 0.134 dk: 0.0314
DNJA3 – DGADIHSDLFISIAQAILGGTAK_3
AA R2: 0.94 k: 0.769 dk: 0.262
HW R2: 0.95 k: 0.512 dk: 0.1
DHTK1 – YGGEGAESMMGFFHELLK_3
AA R2: 0.96 k: 0.128 dk: 0.0306
HW R2: 0.99 k: 0.103 dk: 0.0129
DLDH – ALTGGIAHLFK_2
AA R2: 0.99 k: 0.13 dk: 0.0112
HW R2: 0.96 k: 0.0791 dk: 0.0141
DOPD – KGTVMTFL_2
AA R2: 0.98 k: 0.202 dk: 0.0275
HW R2: 0.93 k: 0.216 dk: 0.0708
DHX9 – ETPFELIEALLK_2
AA R2: 0.97 k: 0.382 dk: 0.07
HW R2: 0.99 k: 0.392 dk: 0.0432
DLDH – ALTGGIAHLFK_3
AA R2: 0.98 k: 0.15 dk: 0.0205
HW R2: 0.98 k: 0.0996 dk: 0.012
DOPD – LCAATATILDKPEDR_2
AA R2: 0.97 k: 0.197 dk: 0.0388
HW R2: 0.99 k: 0.167 dk: 0.0145
DIC – VHLQTQQEVK_2
AA R2: 0.99 k: 0.236 dk: 0.0239
HW R2: 0.99 k: 0.215 dk: 0.0234
DLDH – IPNIYAIGDVVAGPMLAHK_3
AA R2: 0.99 k: 0.125 dk: 0.0107
HW R2: 0.99 k: 0.107 dk: 0.00968
DOPD – LCAATATILDKPEDR_3
AA R2: 0.98 k: 0.197 dk: 0.032
HW R2: 0.99 k: 0.15 dk: 0.0155
FS
Time (days)

DPYD – DFLPLVAK_2

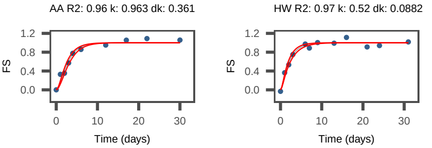

EBP – HLTSAQSVLDSK_2

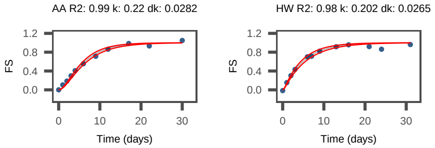

ECHA – FGELALTK_2

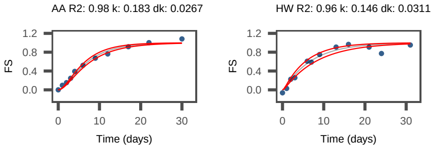

DPYS – DQTCTPVPVK_2

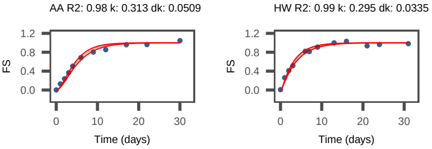

ECH1 – HVLHVQLNRPEK_4

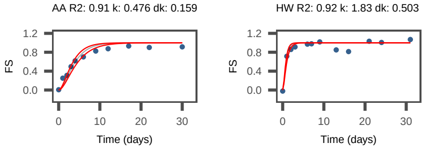

ECHA – FVDLYGAQK_2

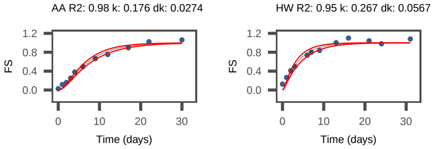

DPYS – MGSQSVDDFYQGTK_2

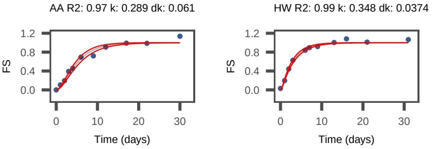

ECHA – ADMVIEAVFEDLGVK_2

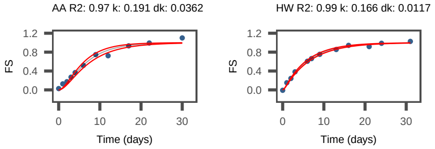

ECHA – GLYPAPLK_2

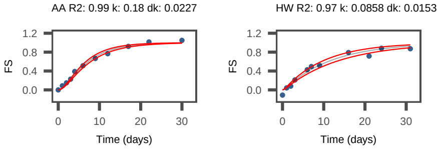

DPYS – VVYEAGVFNVTAGHGK_2

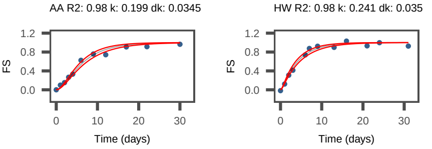

ECHA – ADMVIEAVFEDLGVK_3

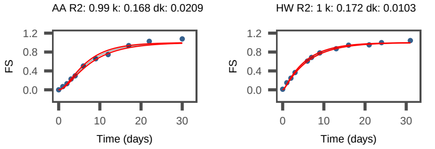

ECHA – ILQEGVDPK_2

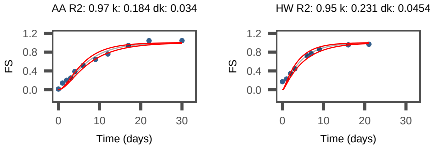

DPYS – VVYEAGVFNVTAGHGK_3

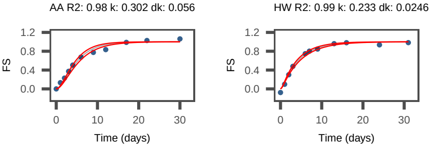

ECHA – AGLEQGSDAGYLAESQK_2

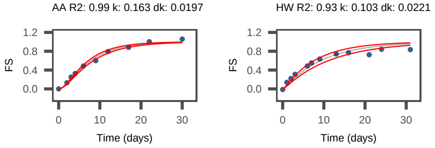

ECHA – KYESAYGTQFTPC_2

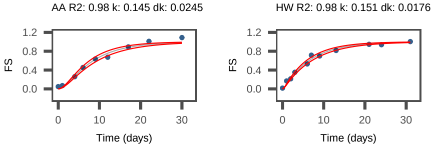

DX39A(Non–Unique) – DFLLKPELLR_3

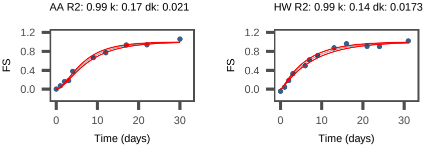

ECHA – DSIFSNLIGQLDYK_3

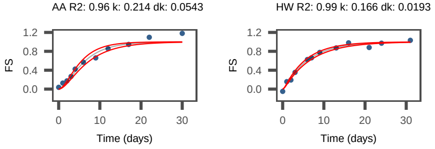

ECHA – LPAKPEVSSDEDVQYR_2

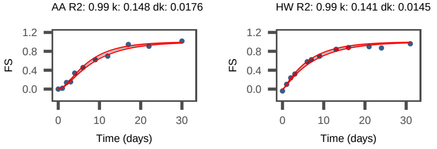

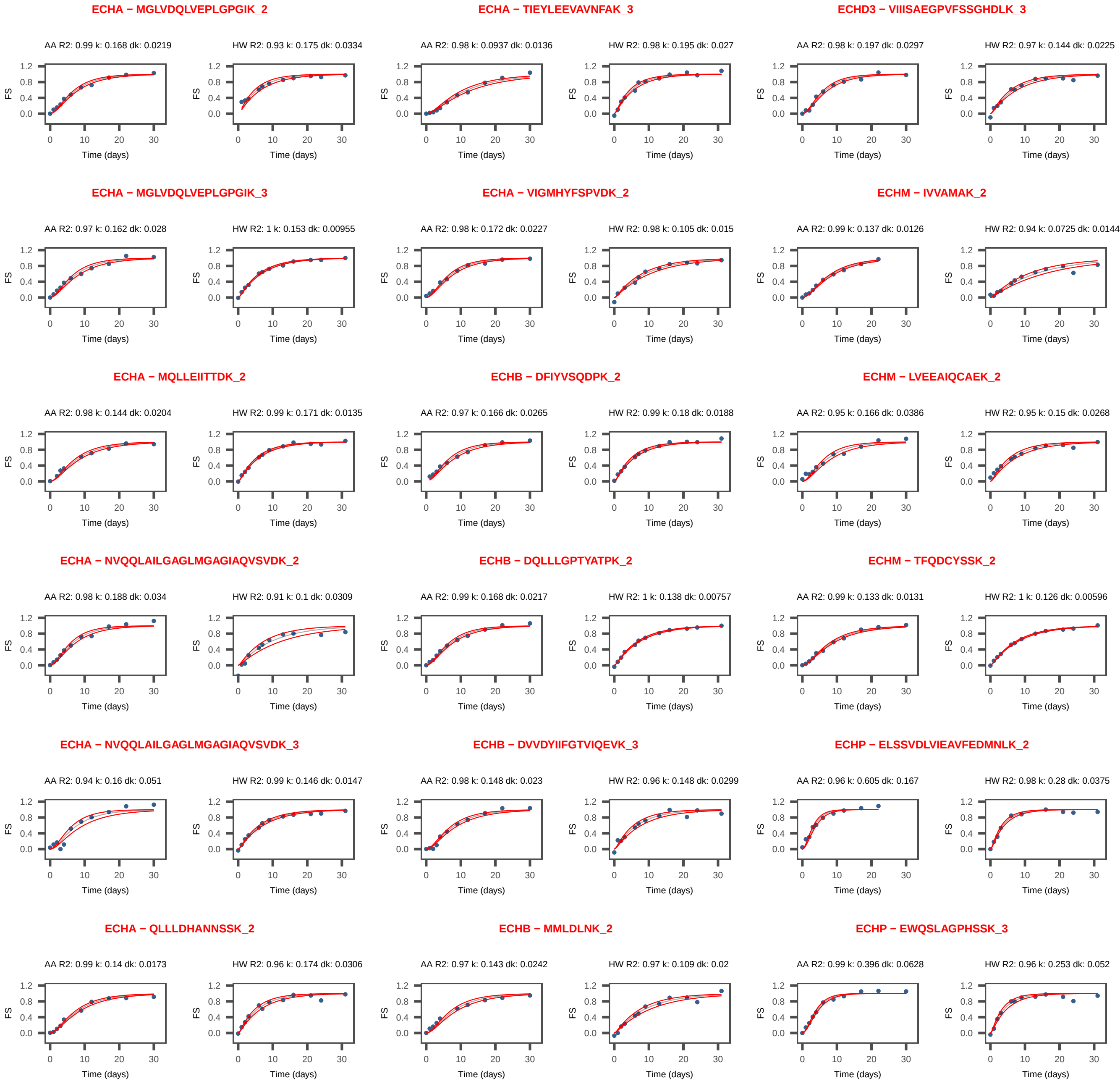
ECHA – MGLVDQLVEPLGPGIK_2
AA R2: 0.99 k: 0.168 dk: 0.0219
HW R2: 0.93 k: 0.175 dk: 0.0334
ECHA – TIEYLEEVAVNFAK_3
AA R2: 0.98 k: 0.0937 dk: 0.0136
HW R2: 0.98 k: 0.195 dk: 0.027
ECHD3 – VIIISAEGPVFSSGHDLK_3
AA R2: 0.98 k: 0.197 dk: 0.0297
HW R2: 0.97 k: 0.144 dk: 0.0225
ECHA – MGLVDQLVEPLGPGIK_3
AA R2: 0.97 k: 0.162 dk: 0.028
HW R2: 1 k: 0.153 dk: 0.00955
ECHA – VIGMHYFSPVDK_2
AA R2: 0.98 k: 0.172 dk: 0.0227
HW R2: 0.98 k: 0.105 dk: 0.015
ECHM – IVVAMAK_2
AA R2: 0.99 k: 0.137 dk: 0.0126
HW R2: 0.94 k: 0.0725 dk: 0.0144
ECHA – MQLLEIITTDK_2
AA R2: 0.98 k: 0.144 dk: 0.0204
HW R2: 0.99 k: 0.171 dk: 0.0135
ECHB – DFIYVSQDPK_2
AA R2: 0.97 k: 0.166 dk: 0.0265
HW R2: 0.99 k: 0.18 dk: 0.0188
ECHM – LVEEAIQCAEK_2
AA R2: 0.95 k: 0.166 dk: 0.0386
HW R2: 0.95 k: 0.15 dk: 0.0268
ECHA – NVQQLAILGAGLMGAGIAQVSVDK_2
AA R2: 0.98 k: 0.188 dk: 0.034
HW R2: 0.91 k: 0.1 dk: 0.0309
ECHB – DQLLLGPTYATPK_2
AA R2: 0.99 k: 0.168 dk: 0.0217
HW R2: 1 k: 0.138 dk: 0.00757
ECHM – TFQDCYSSK_2
AA R2: 0.99 k: 0.133 dk: 0.0131
HW R2: 1 k: 0.126 dk: 0.00596
ECHA – NVQQLAILGAGLMGAGIAQVSVDK_3
AA R2: 0.94 k: 0.16 dk: 0.051
HW R2: 0.99 k: 0.146 dk: 0.0147
ECHB – DVVDYIIFGTVIQEVK_3
AA R2: 0.98 k: 0.148 dk: 0.023
HW R2: 0.96 k: 0.148 dk: 0.0299
ECHP – ELSSVDLVIEAVFEDMNLK_2
AA R2: 0.96 k: 0.605 dk: 0.167
HW R2: 0.98 k: 0.28 dk: 0.0375
ECHA – QLLLDHANNSSK_2
AA R2: 0.99 k: 0.14 dk: 0.0173
HW R2: 0.96 k: 0.174 dk: 0.0306
ECHB – MMLDLNK_2
AA R2: 0.97 k: 0.143 dk: 0.0242
HW R2: 0.97 k: 0.109 dk: 0.02
ECHP – EWQSLAGPHSSK_3
AA R2: 0.99 k: 0.396 dk: 0.0628
HW R2: 0.96 k: 0.253 dk: 0.052
FS
Time (days)

ECHP − GWYQYDKPLGR_3

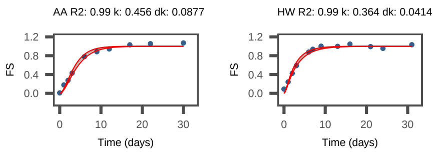

ECI1 − NPPVNSLSLECLTEFTISLEK_2

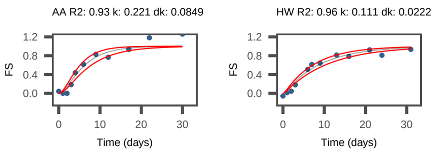

EF1A1 − MDSTEPPYSQK_2

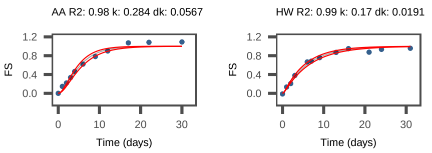

ECHP − HISTDEALK_2

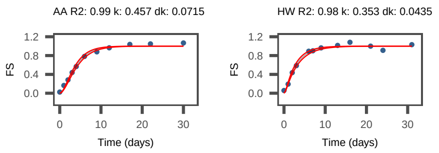

ECI1 − VLVETEGPAGVAVMK_2

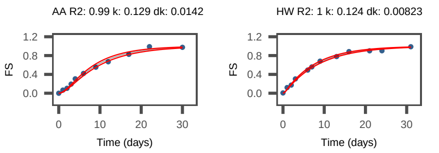

EF1A1(Non−Unique) − QLIVGVNK_2

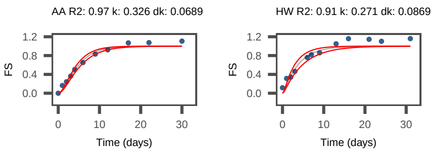

ECHP − IHKPDPWLSEFLSQYR_4

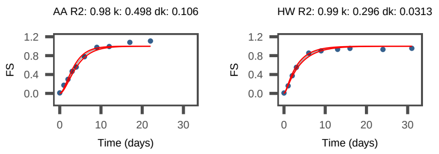

ECI1 − YTIGLNESLLGIVAPFWFK_2

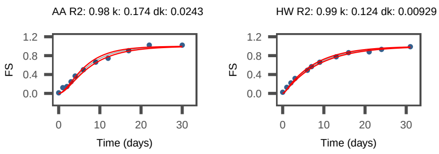

EF1A1(Non−Unique) − QTVAVGVIK_2

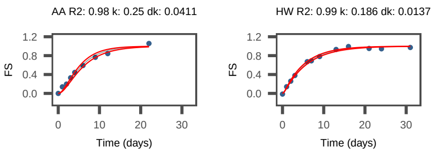

ECHP − LGILDVVVK_2

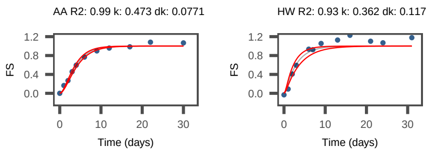

ECI2 − QNYVDLVSSLSSSSEAPSQGK_2

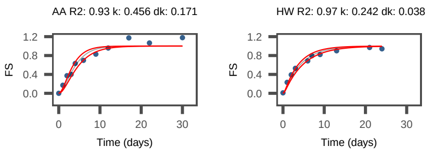

EF1A1 − RYEEIVK_2

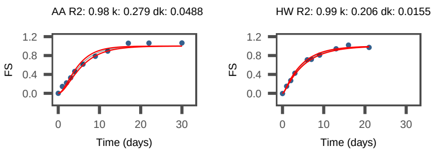

ECI1 − ALQLGTLFSPAEALK_2

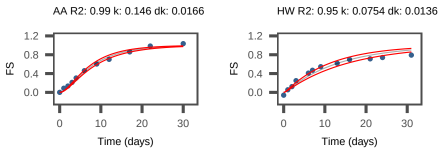

ECI2 − SQLGQSPEACSSYTFPK_2

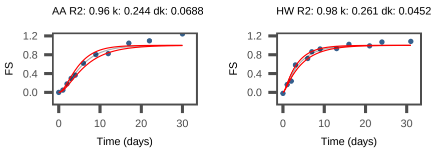

EF1A1(Non−Unique) − STTTGHLIYK_2

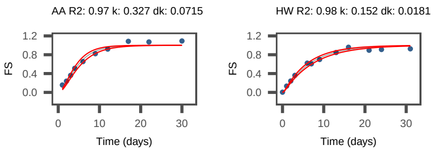

ECI1 − NPAHYAEYWK_2

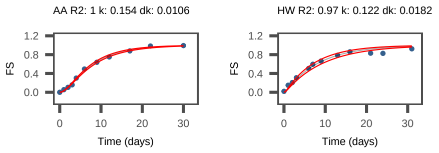

EF1A1(Non−Unique) − EHALLAYTLGVK_2

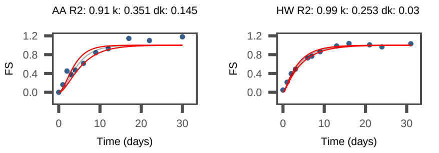

EF1A1(Non−Unique) − THINIVVIGHVDSGK_4

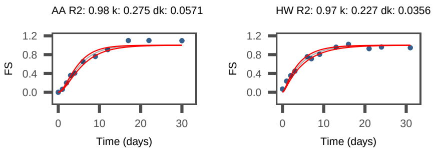

EF1G – EYFSWEGTFQHVGK_3

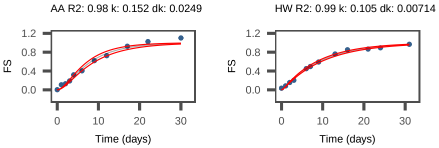

EF1G – ILGLLDTHLK_2

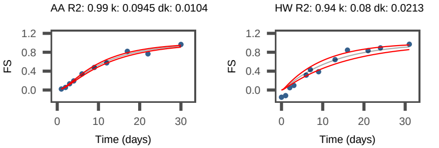

EF1G – ILGLLDTHLK_3

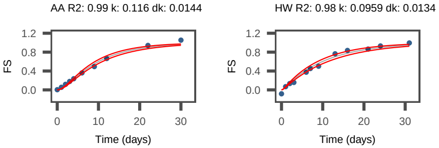

EF1G – STFVLDEFKR_3

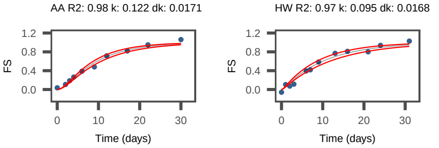

EF1G – YSNEDTLSVALPYFWEHFDK_3

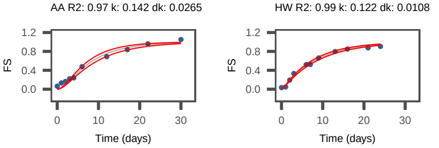

EF2 – EGIPALDNFLDKL_2

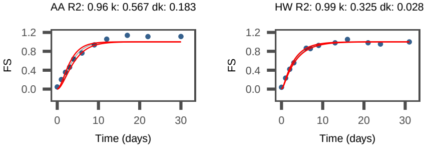

EF2 – ETVSEESNVLCLSK_2

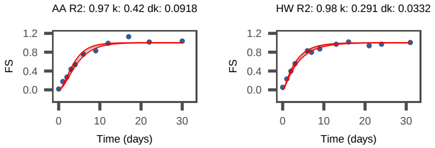

EF2 – GHVFEESQVAGTPMFVVK_3

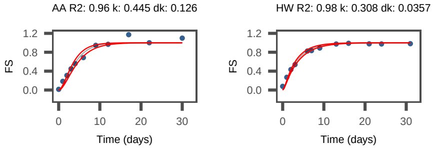

EF2 – IWCFGPDGTGPNILTDITK_2

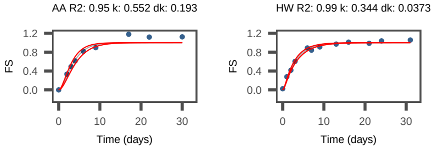

EF2 – NMSVIAHVDHGK_3

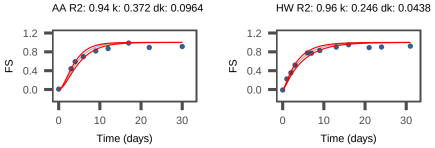

EF2 – STAISLFYELSENDLNFIK_2

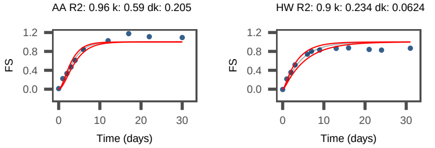

EF2 – VFSGVVSTGLK_2

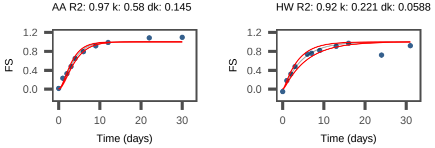

EF2 – WLPAGDALLQMITIHLPSPVTAQK_3

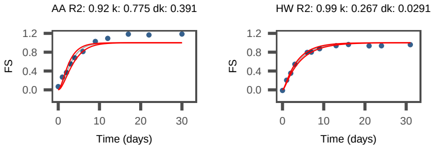

EFTU – DLDKPFLLPVESVYSIPGR_3

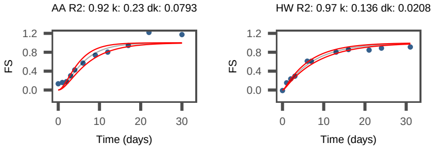

EFTU – GLVMVKPGSIQPH_2

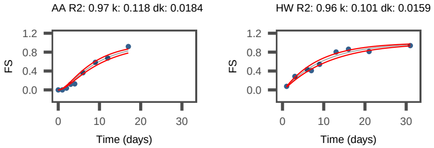

EFTU – HYAHTDCPGHADYVK_2

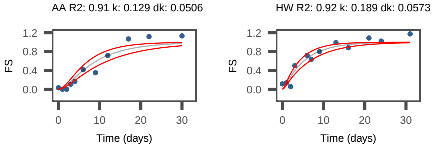

EFTU – HYAHTDCPGHADYVK_3

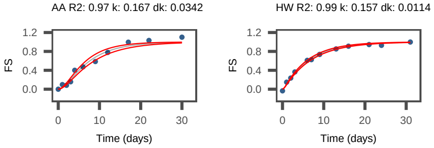

EFTU – HYAHTDCPGHADYVK_4

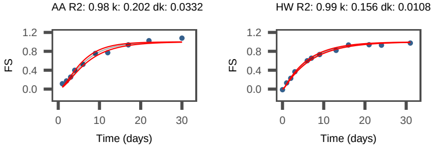

EFTU − TIGTGLVTDVPAMTEEDK_2

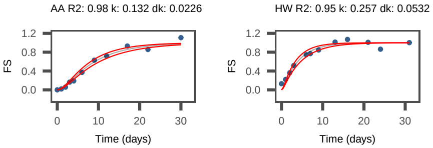

ENOA − YITPDQLADLYK_2

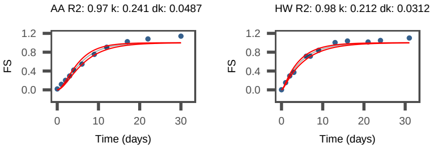

ENPL − TVWDWELMNDIKPIWQR_3

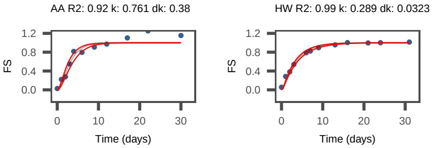

EIF3E − HLVFPLLEFLSVK_3

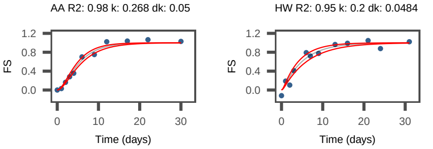

ENPL − DISTNYYASQK_2

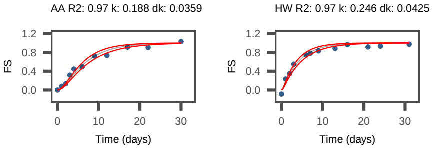

ENPL − VFITDDFHDMMPK_3

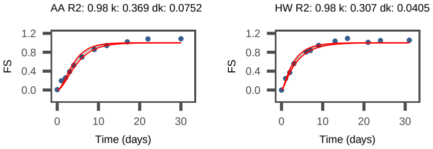

ENOA − DATNVGDEGGFAPNILENK_2

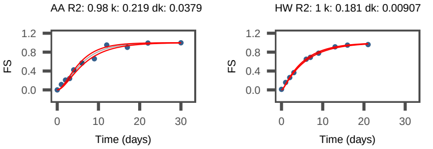

ENPL − EFEPLLNWMK_2

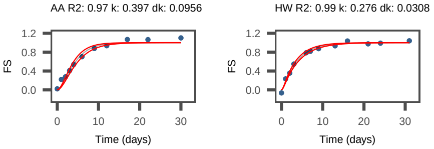

ENTP5 − AQALLLEVEEIFK_2

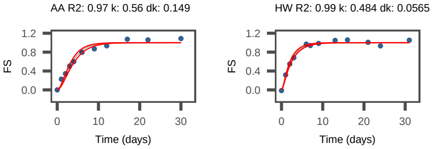

ENOA(Non−Unique) − GNPTVEVDLYTAK_2

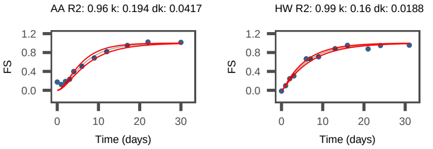

ENPL − GTTITLVLK_2

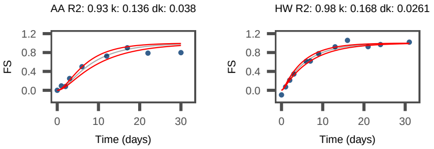

ENTP5 − QGAETVQELLEVAK_2

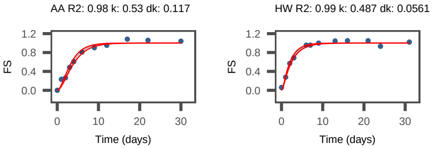

ENOA − LMIEMDGTENK_2

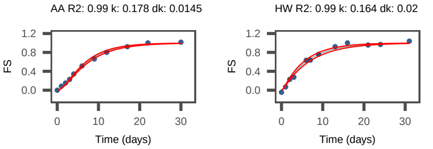

ENPL − LIINSLYK_2

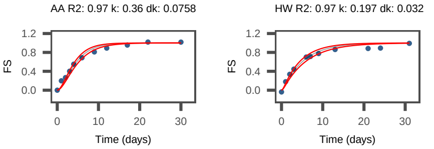

ENTP5 − WLEAEWIFGGVK_2

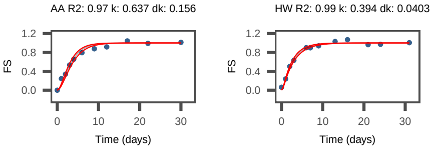

ENOA − TIAPALVSK_2

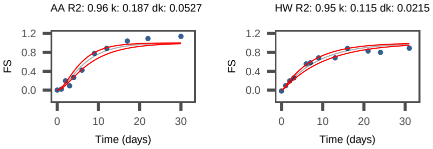

ENPL − SGYLLPDTK_2

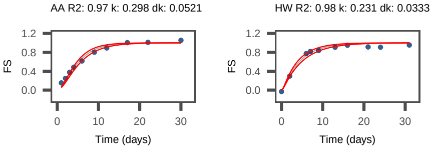

ERAP1 − TDVLILPEAVQWIK_2

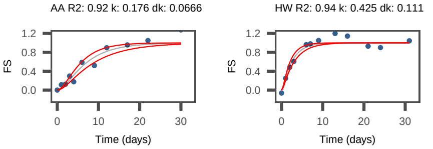

EST1 − ESYPFLPTVIDGVLLPK_2

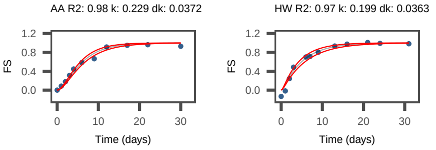

EST1D − DKEVSFWAELR_3

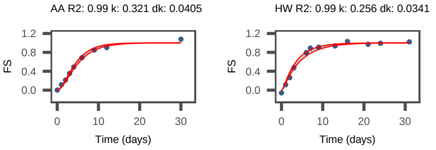

EST1D − TANSLLWK_2

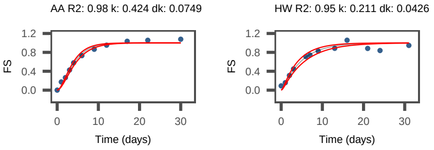

EST1 − HPSLPPVVHTVHGK_4

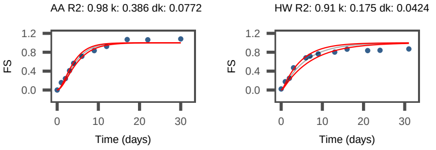

EST1D − ENIPLQFSEDCLYLNIYTPADLTK_3

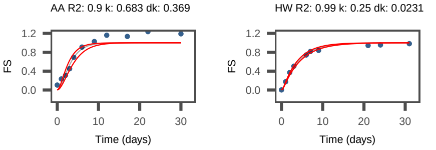

EST1E(Non−Unique) − FAPPQPAEPWSSVK_2

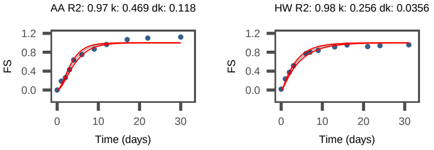

EST1C(Non−Unique) − EVAFWTELLAK_2

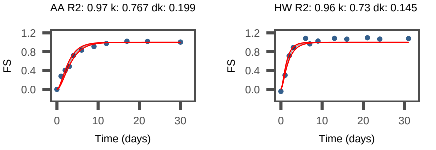

EST1D − ESYPFLPTVIDGVVLPK_2

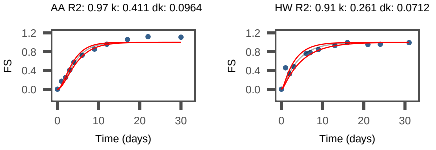

EST1F − GNPSSPPVVDTAHGK_2

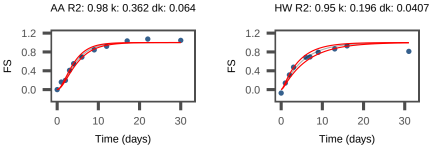

EST1C(Non−Unique) − FAPPQPAEPWSFVK_2

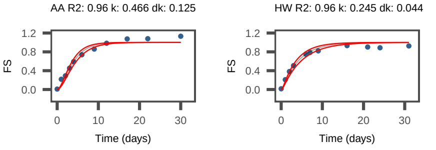

EST1D − ISENMIPVVAEK_2

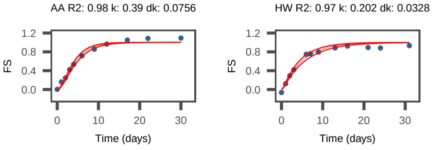

EST1F − LPVMVWIHGGGLK_3

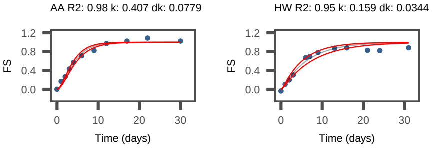

EST1D − AVIGDHGDEIFSVFGSPFLK_3

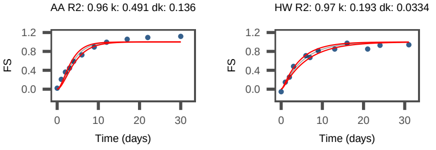

EST1D − QEFGWIIPTLMGYPLAEGK_3

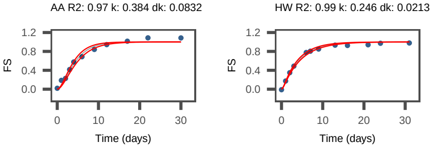

EST2A − QEILAINQVFK_2

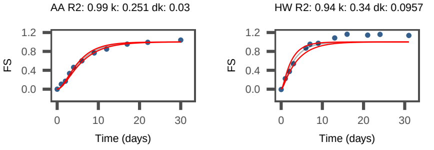

EST1D − DGASEEETNLSK_2

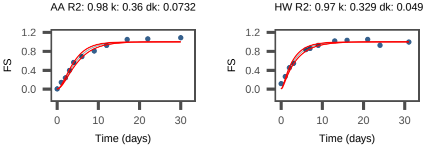

EST1D − SFSTVPYIVGINK_2

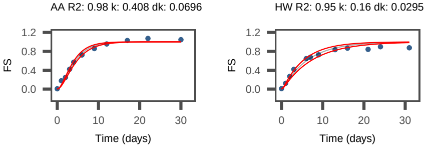

EST2C − ADHADEIPFVFASFFWGMK_3

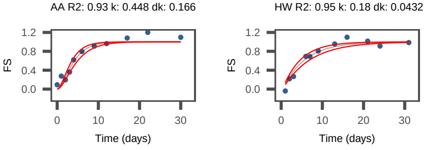

EST2E − HPQELMASK_2

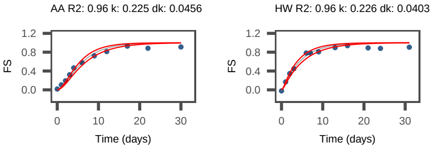

EST3B − DASINPPMCLQDVEK_2

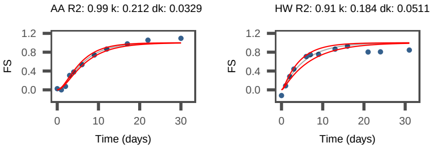

ETFA − APSSSSVGISEWLDQK_2

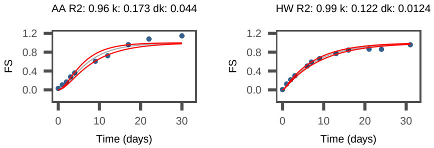

EST3A − AISQSGVVISK_2

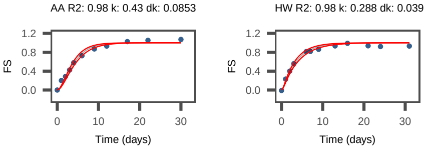

ESTD − AFSGYLGPDESK_2

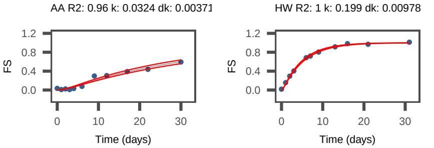

ETFA − GLLPEELTPLILETQK_2

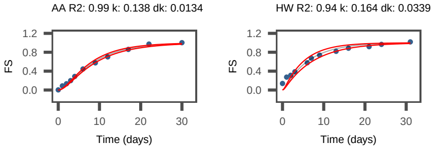

EST3A(Non−Unique) − FWNILDK_2

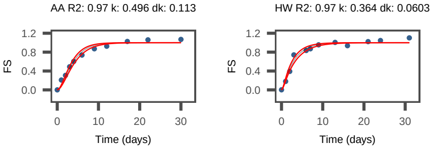

ESTD − AYDATCLVK_2

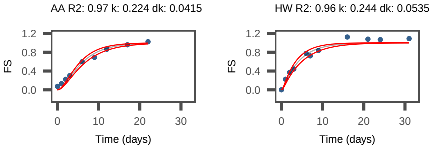

ETFA − GLLPEELTPLILETQK_3

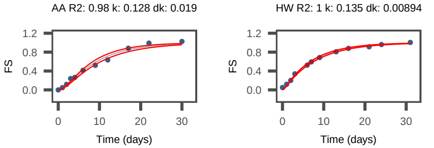

EST3A(Non−Unique) − LGIFGFLSTGDK_2

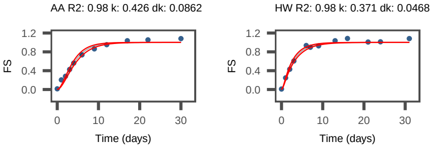

ESTD − CPALYWLSGLTCTEQNFISK_2

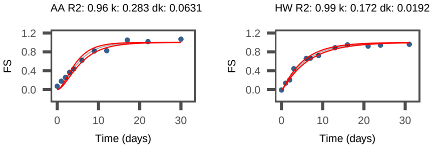

ETFA − GTSFEAAATSGGSASSEK_2

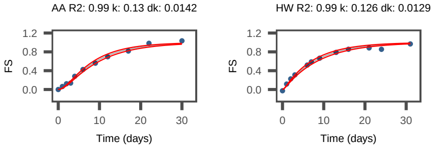

EST3A(Non−Unique) − QFPTVPYLLGVTNHEFGWLLLK_3

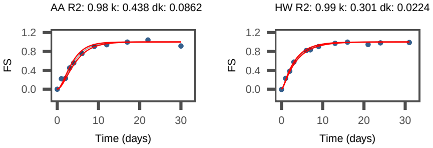

ESTD − CPALYWLSGLTCTEQNFISK_3

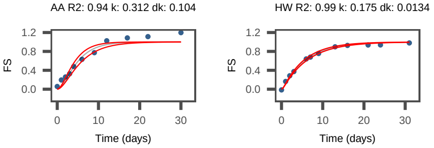

ETFA − IVAPELYIAVGISGAIQHLAGMK_3

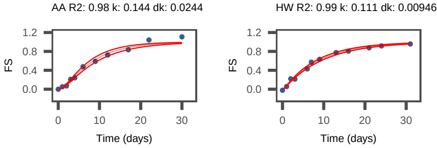

EST3A − YALQELLGDITLVIPTLIFSK_3

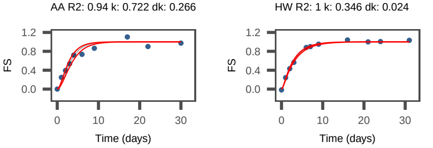

ESTD − SVSAFAPICNPVLCSWGK_2

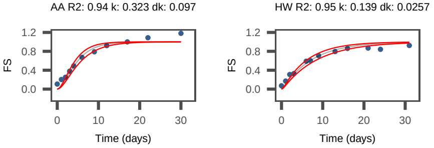

ETFA − LNVAPVSDIIEIK_2

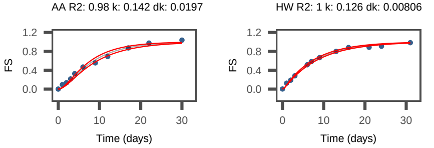

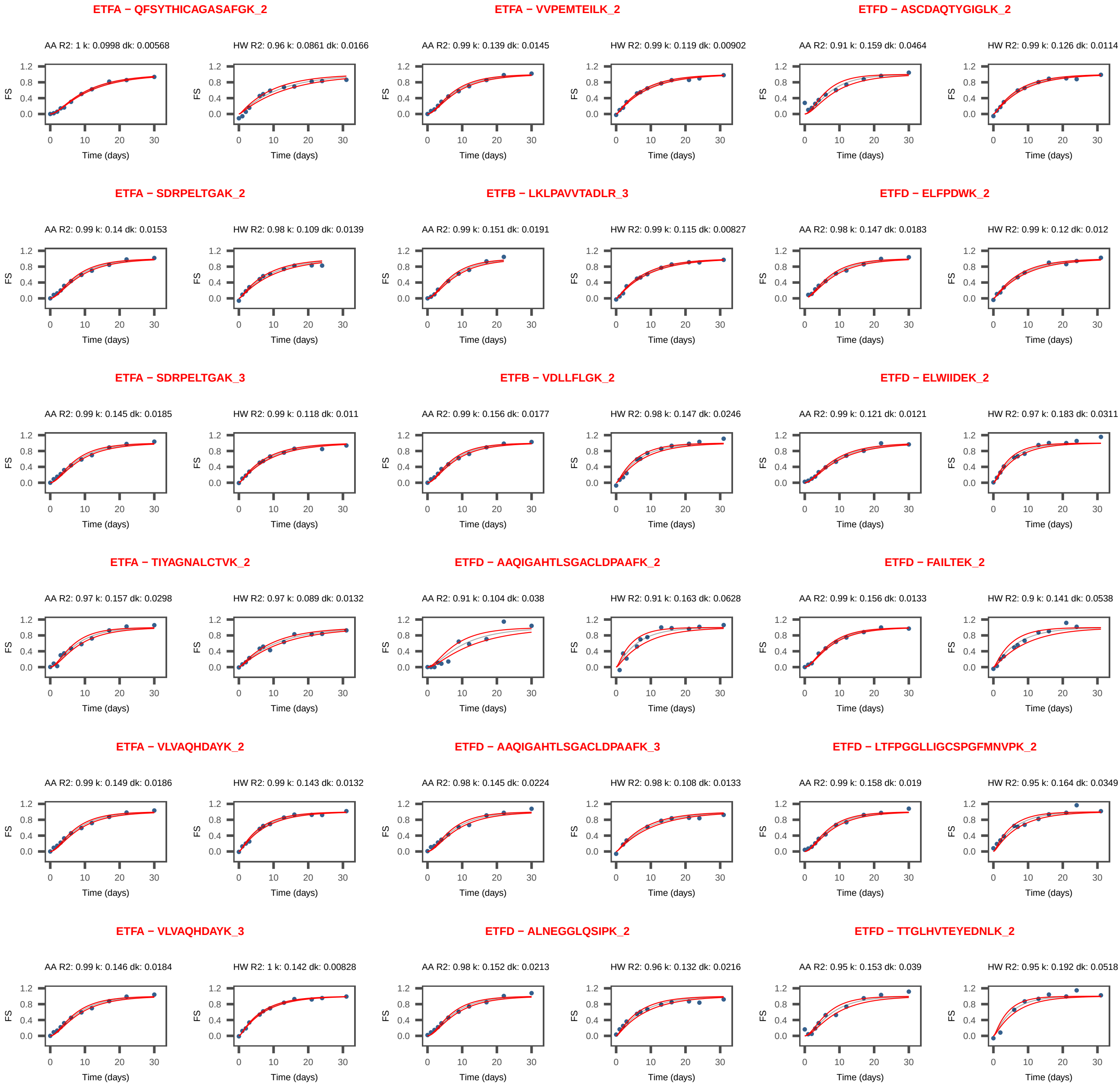
ETFA − QFSYTHICAGASAFGK_2
AA R2: 1 k: 0.0998 dk: 0.00568
HW R2: 0.96 k: 0.0861 dk: 0.0166
ETFA − VVPEMTEILK_2
AA R2: 0.99 k: 0.139 dk: 0.0145
HW R2: 0.99 k: 0.119 dk: 0.00902
ETFD − ASCDAQTYGIGLK_2
AA R2: 0.91 k: 0.159 dk: 0.0464
HW R2: 0.99 k: 0.126 dk: 0.0114
ETFA − SDRPELTGAK_2
AA R2: 0.99 k: 0.14 dk: 0.0153
HW R2: 0.98 k: 0.109 dk: 0.0139
ETFB − LKLPAVVTADLR_3
AA R2: 0.99 k: 0.151 dk: 0.0191
HW R2: 0.99 k: 0.115 dk: 0.00827
ETFD − ELFPDWK_2
AA R2: 0.98 k: 0.147 dk: 0.0183
HW R2: 0.99 k: 0.12 dk: 0.012
ETFA − SDRPELTGAK_3
AA R2: 0.99 k: 0.145 dk: 0.0185
HW R2: 0.99 k: 0.118 dk: 0.011
ETFB − VDLLFLGK_2
AA R2: 0.99 k: 0.156 dk: 0.0177
HW R2: 0.98 k: 0.147 dk: 0.0246
ETFD − ELWIIDEK_2
AA R2: 0.99 k: 0.121 dk: 0.0121
HW R2: 0.97 k: 0.183 dk: 0.0311
ETFA − TIYAGNALCTVK_2
AA R2: 0.97 k: 0.157 dk: 0.0298
HW R2: 0.97 k: 0.089 dk: 0.0132
ETFD − AAQIGAHTLSGACLDPAAFK_2
AA R2: 0.91 k: 0.104 dk: 0.038
HW R2: 0.91 k: 0.163 dk: 0.0628
ETFD − FAILTEK_2
AA R2: 0.99 k: 0.156 dk: 0.0133
HW R2: 0.9 k: 0.141 dk: 0.0538
ETFA − VLVAQHDAYK_2
AA R2: 0.99 k: 0.149 dk: 0.0186
HW R2: 0.99 k: 0.143 dk: 0.0132
ETFD − AAQIGAHTLSGACLDPAAFK_3
AA R2: 0.98 k: 0.145 dk: 0.0224
HW R2: 0.98 k: 0.108 dk: 0.0133
ETFD − LTFPGGLLIGCSPGFMNVPK_2
AA R2: 0.99 k: 0.158 dk: 0.019
HW R2: 0.95 k: 0.164 dk: 0.0349
ETFA − VLVAQHDAYK_3
AA R2: 0.99 k: 0.146 dk: 0.0184
HW R2: 1 k: 0.142 dk: 0.00828
ETFD − ALNEGGLQSIPK_2
AA R2: 0.98 k: 0.152 dk: 0.0213
HW R2: 0.96 k: 0.132 dk: 0.0216
ETFD − TTGLHVTEYEDNLK_2
AA R2: 0.95 k: 0.153 dk: 0.039
HW R2: 0.95 k: 0.192 dk: 0.0518
FS
Time (days)

F16P1 – AQGTGELTQLLNSLCTAIK_3

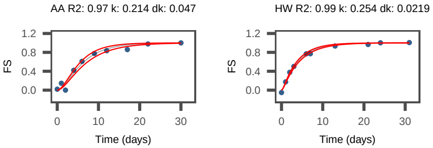

F16P1 – STDEPSEKDALQPGR_3

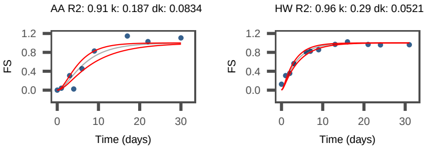

FAAA – IGVAIGDQILDLSVIK_2

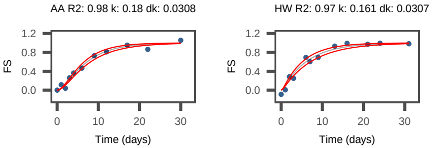

F16P1 – DILDIVPTEIHQK_3

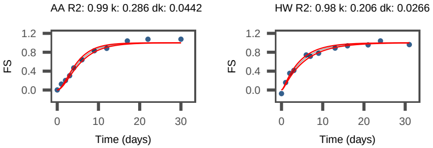

F16P1 – TLVYGGIFLYPANK_2

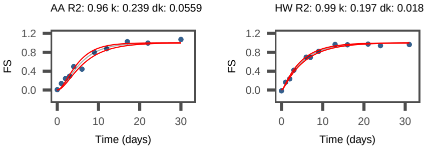

FAAA – M[15.9949]RPDNSKPPVYGACR_3

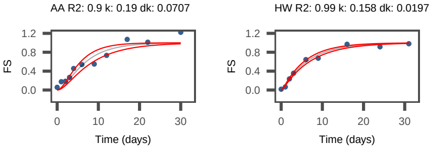

F16P1 – GNIYSLNEGYAK_2

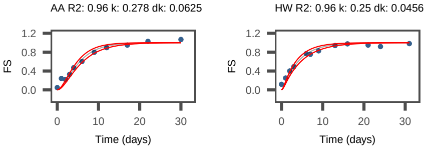

FAAA – DIQQWEYVPLGPFLGK_2

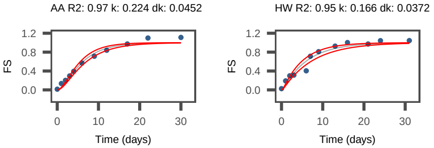

FAAA – MRPDNSKPPVYGACR_4

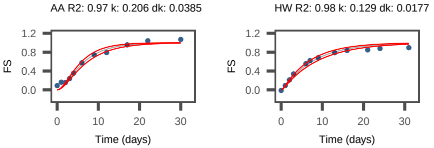

F16P1 – LDILSNDLVINMLK_3

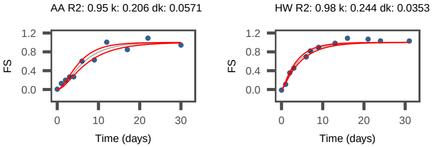

FAAA – DIQQWEYVPLGPFLGK_3

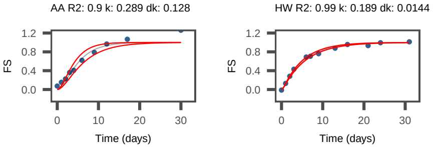

FAAA – PGDLLASGTISGSDPESFGSMLELSWK_2

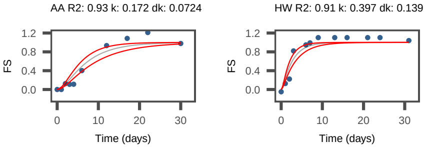

F16P1 – LLYECNPIAYVMEK_2

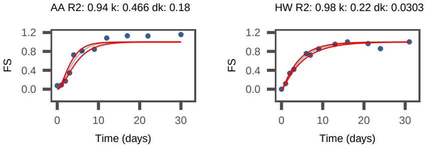

FAAA – HLFTGPALSK_2

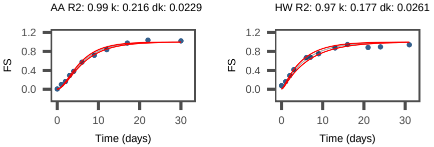

FABPI – EVSGNELIQTYTYEGVEAK_2

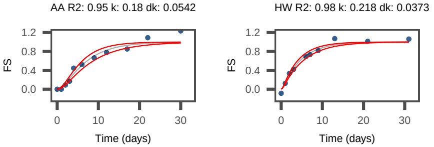

F16P1 – QAGIAQLYGIAGSTNVTGDQVK_2

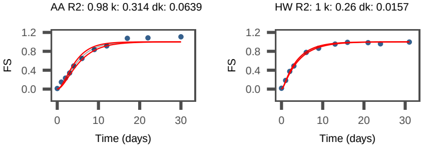

FAAA – HQHVFDETTLNNFMGLGQAAWK_2

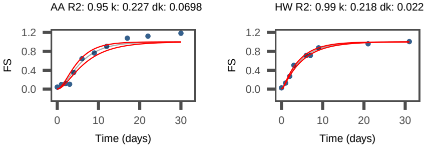

FABPI – LTITQDGNK_2

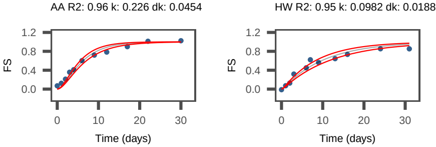

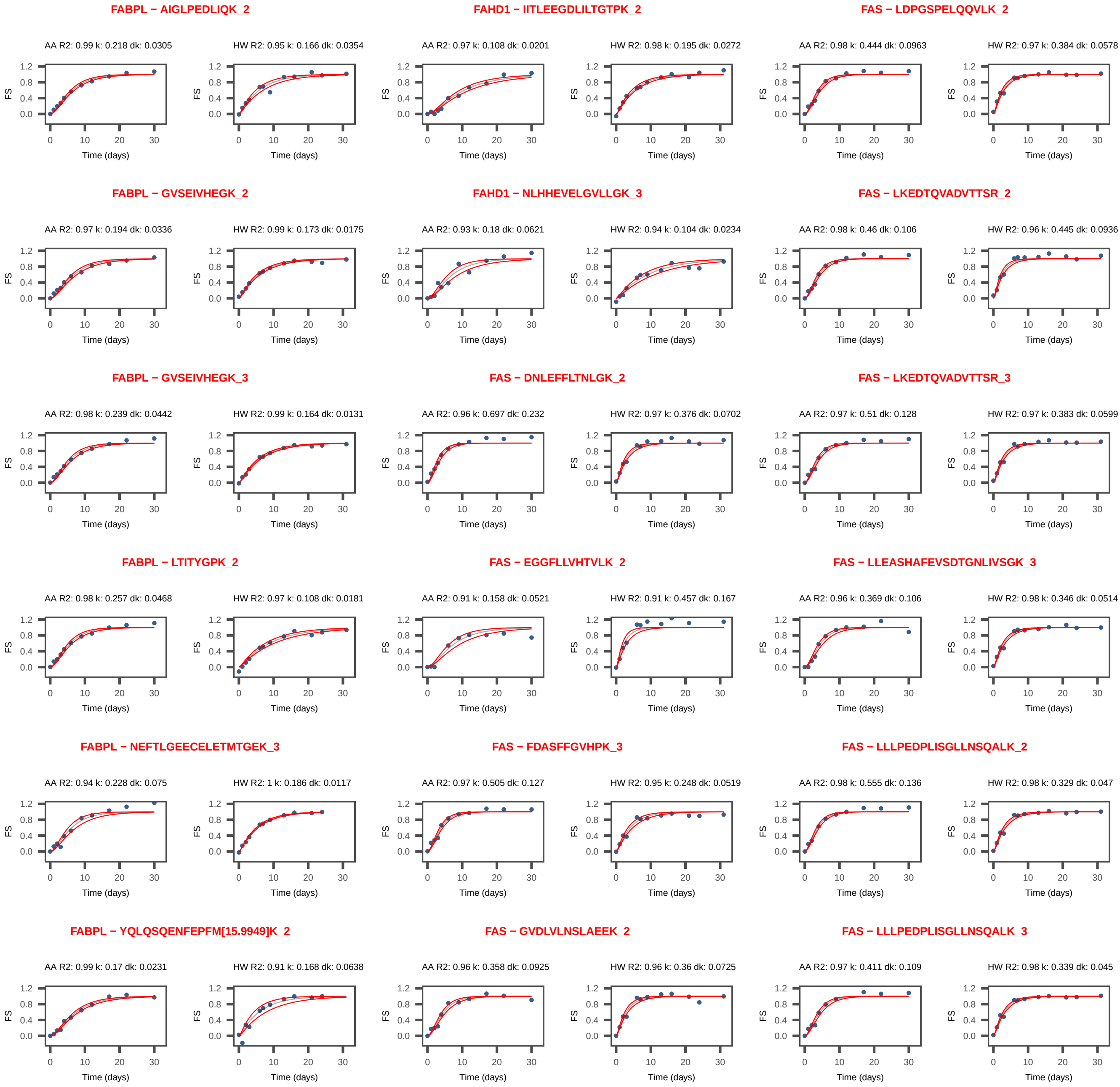
FABPL – AIGLPEDLIQK_2
AA R2: 0.99 k: 0.218 dk: 0.0305
HW R2: 0.95 k: 0.166 dk: 0.0354
FAHD1 – IITLEEGDLILTGTPK_2
AA R2: 0.97 k: 0.108 dk: 0.0201
HW R2: 0.98 k: 0.195 dk: 0.0272
FAS – LDPGSPELQQVLK_2
AA R2: 0.98 k: 0.444 dk: 0.0963
HW R2: 0.97 k: 0.384 dk: 0.0578
FABPL – GVSEIVHEGK_2
AA R2: 0.97 k: 0.194 dk: 0.0336
HW R2: 0.99 k: 0.173 dk: 0.0175
FAHD1 – NLHHEVELGVLLGK_3
AA R2: 0.93 k: 0.18 dk: 0.0621
HW R2: 0.94 k: 0.104 dk: 0.0234
FAS – LKEDTQVADVTTSR_2
AA R2: 0.98 k: 0.46 dk: 0.106
HW R2: 0.96 k: 0.445 dk: 0.0936
FABPL – GVSEIVHEGK_3
AA R2: 0.98 k: 0.239 dk: 0.0442
HW R2: 0.99 k: 0.164 dk: 0.0131
FAS – DNLEFFLTNLGK_2
AA R2: 0.96 k: 0.697 dk: 0.232
HW R2: 0.97 k: 0.376 dk: 0.0702
FAS – LKEDTQVADVTTSR_3
AA R2: 0.97 k: 0.51 dk: 0.128
HW R2: 0.97 k: 0.383 dk: 0.0599
FABPL – LTITYGPK_2
AA R2: 0.98 k: 0.257 dk: 0.0468
HW R2: 0.97 k: 0.108 dk: 0.0181
FAS – EGGFLLVHTVLK_2
AA R2: 0.91 k: 0.158 dk: 0.0521
HW R2: 0.91 k: 0.457 dk: 0.167
FAS – LLEASHAFEVSDTGNLIVSGK_3
AA R2: 0.96 k: 0.369 dk: 0.106
HW R2: 0.98 k: 0.346 dk: 0.0514
FABPL – NEFTLGEECELETMTGEK_3
AA R2: 0.94 k: 0.228 dk: 0.075
HW R2: 1 k: 0.186 dk: 0.0117
FAS – FDASFFGVHPK_3
AA R2: 0.97 k: 0.505 dk: 0.127
HW R2: 0.95 k: 0.248 dk: 0.0519
FAS – LLLPEDPLISGLLNSQALK_2
AA R2: 0.98 k: 0.555 dk: 0.136
HW R2: 0.98 k: 0.329 dk: 0.047
FABPL – YQLQSQENFEPFM[15.9949]K_2
AA R2: 0.99 k: 0.17 dk: 0.0231
HW R2: 0.91 k: 0.168 dk: 0.0638
FAS – GVDLVLNSLAEEK_2
AA R2: 0.96 k: 0.358 dk: 0.0925
HW R2: 0.96 k: 0.36 dk: 0.0725
FAS – LLLPEDPLISGLLNSQALK_3
AA R2: 0.97 k: 0.411 dk: 0.109
HW R2: 0.98 k: 0.339 dk: 0.045

FAS − LSFFFDFK_2

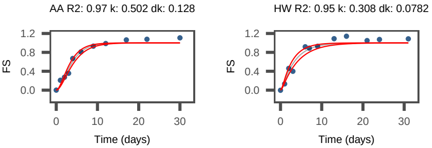

FAS − LTQGEVYK_2

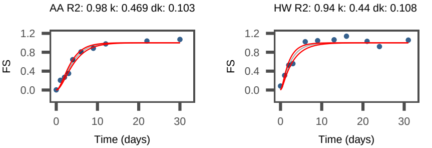

FAS − SNMGHPEPASGLAALTK_2

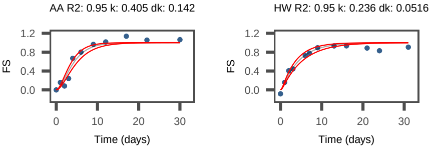

FAS − SNMGHPEPASGLAALTK_3

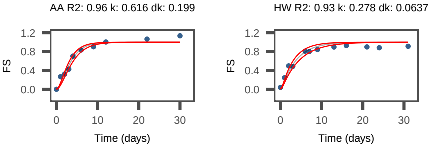

FAS − TGGLAFHSYFMEGIAPTLLQALK_3

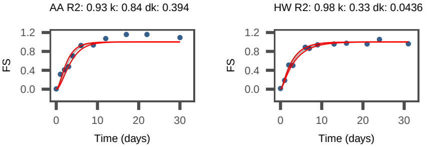

FAS − VLEALLPLK_2

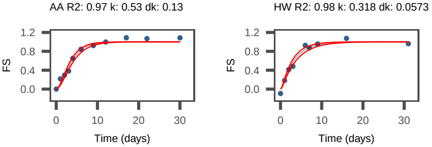

FAS − VQEVQQVSTNK_2

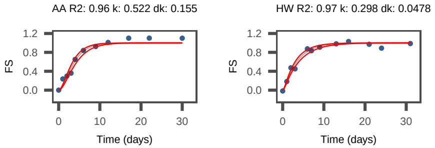

FGGY − AGHFFDLPDFLSWK_3

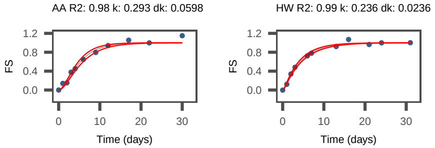

FIS1 − GIVLLEELLPK_2

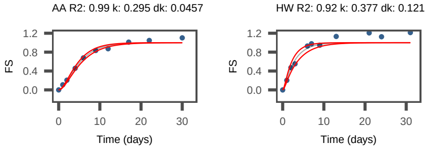

FLNB − AQITNPSGASTECFVK_2

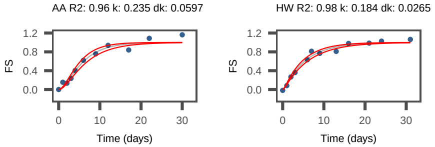

FMO1 − ALQTDYITYIDDLLTSINAK_2

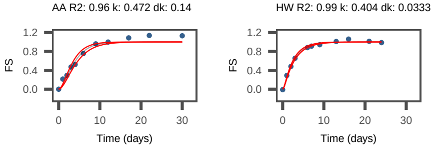

FMO1 − ALQTDYITYIDDLLTSINAK_3

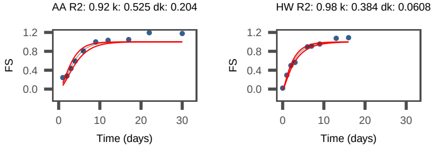

FMO1 − WVVQVLK_2

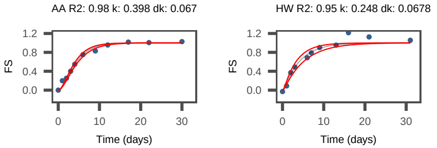

FMO5 − IAVIGAGASGLTCIK_2

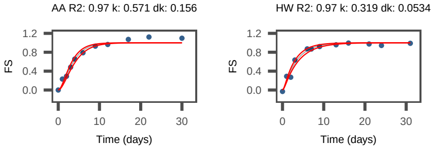

FMO5 − IIAGLVK_2

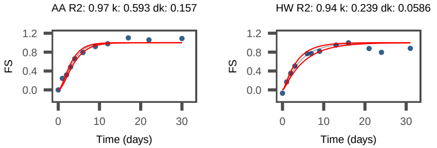

FMO5 − VIVIGIGNSGGDLAVEISHTAK_3

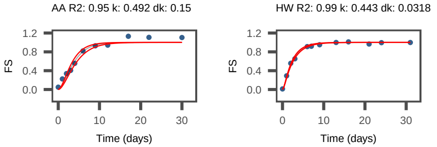

FPPS − ALYEALDLQSAFFK_2

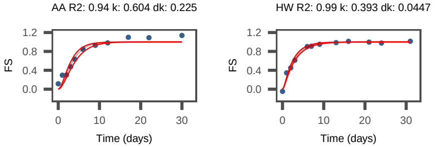

FPPS − ALYEALDLQSAFFK_3

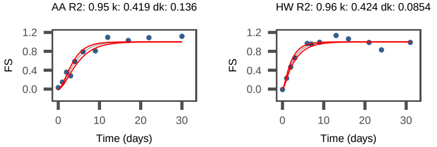

FPPS − GLTVVQAFQELVEPK_2

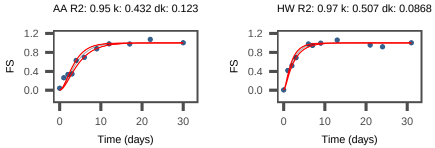

FTCD − AFAACLEAIK_2

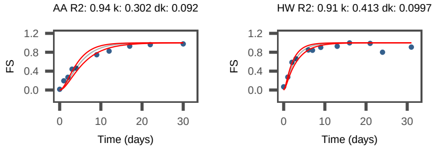

FTCD − EAQELNLPVVGSQLVGLVPLK_2

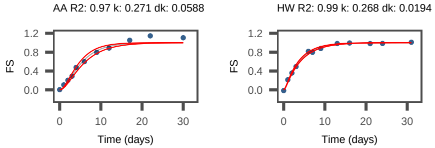

FTCD − EAQELNLPVVGSQLVGLVPLK_3

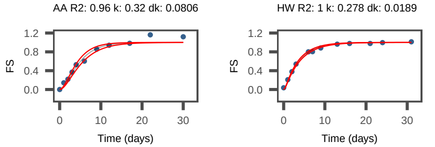

FTCD − GVSMEECVLCAK_2

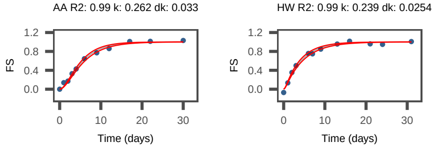

FUMH − AAAEVNQEYGLDPK_2

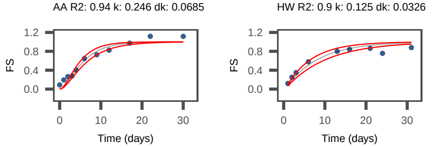

FUMH − HIAAAVEVHK_3

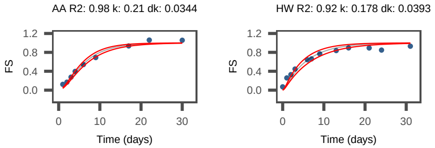

FUMH − MPIPVIQAFGILK_2

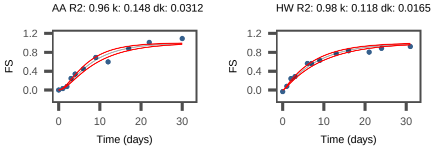

FUMH − VAALTGLPFVTAPNK_2

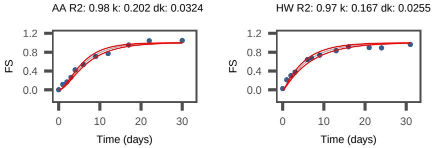

FUMH − VEFDTFGELK_2

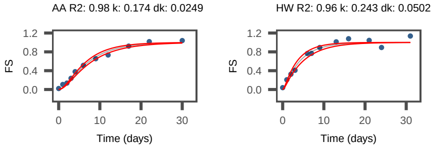

FUMH − VLLPGLQK_2

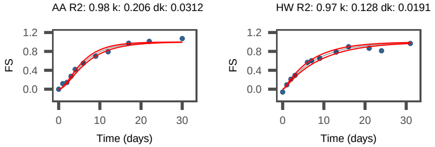

FUS − APKPDGPGGGPGGSHMGGNYGDDR_4

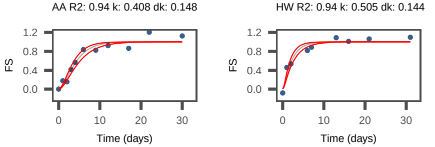

G3P(Non−Unique) − CLAPLAK_2

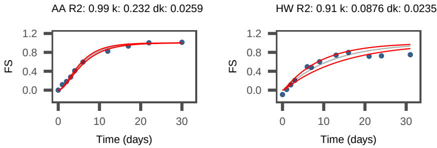

G3P − FQYDSTHGK_2

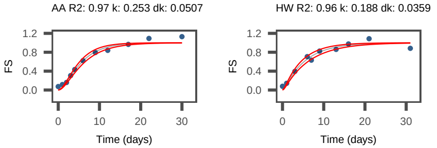

G3P − FQYDSTHGK_3

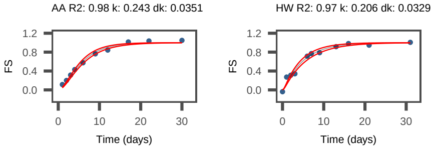

G3P − GAAQNIIPASTGAAK_2

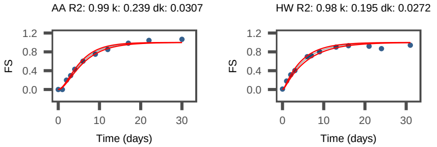

G3P − MFQYDSTHGK_3

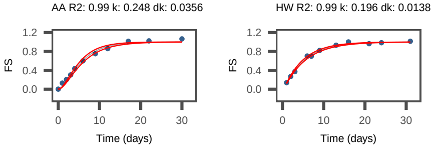

G3P − RVIISAPSADAPMFVMGVNHEK_4

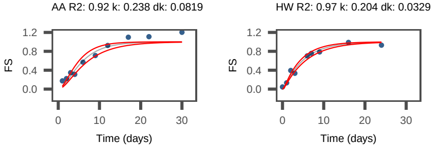

G3P − TTVHAITATQK_2

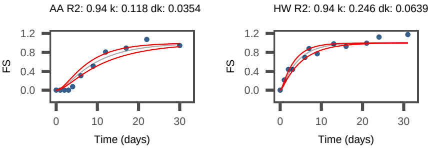

G6PE − QSPLITAWPEELISK_2

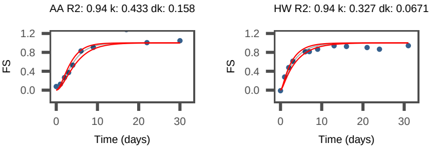

G6PI − TLASLSPETSLFIIASK_2

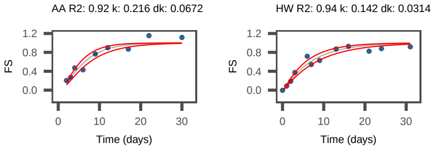

G3P − VIHDNFGIVEGLMTTVHAITATQK_3

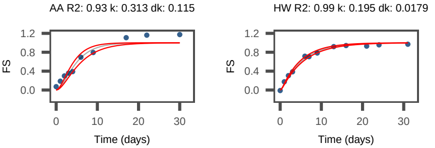

G6PI − FAAYFQQGDMESNGK_2

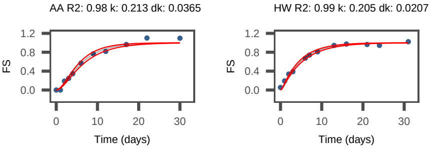

G6PI − VFEGNRPTNSIVFTK_2

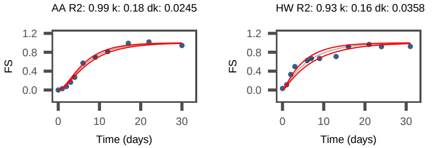

G3P − VIISAPSADAPMFVMGVNHEK_2

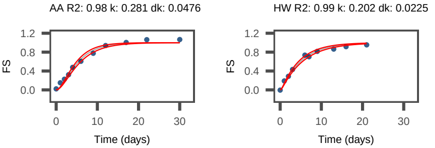

G6PI − ILGALIAMYEHK_3

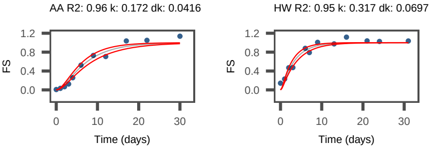

GABT − NLLLAEVINIIK_2

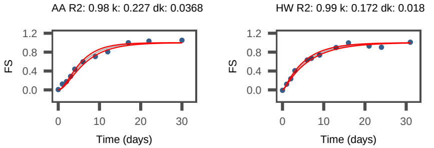

G3P − VIISAPSADAPMFVMGVNHEK_3

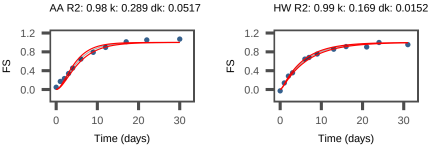

G6PI − ILLANFLAQTEALMK_2

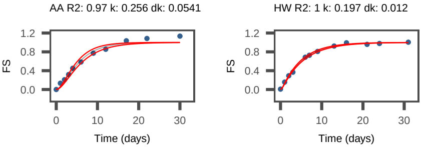

GADL1(Non−Unique) − ADSVAWNPHK_3

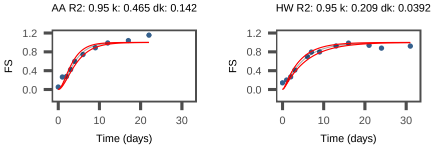

G3P − VIPELNGK_2

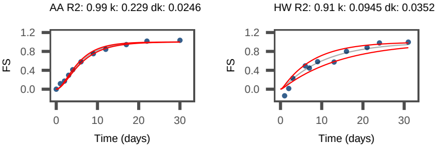

G6PI − SITDIINIGIGGSDLGPLMVTEALKPY_2

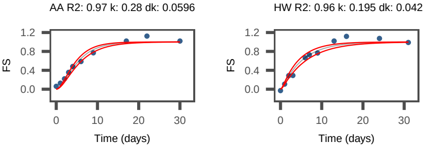

GAL3A − ITSLAQLNAANHDAAIFPGGFGAAK_3

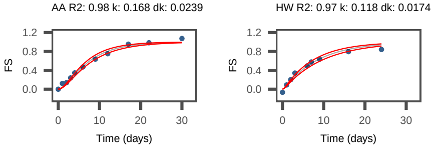

G6PC1 − WCERPEWVHLDTTPFASLFK_4

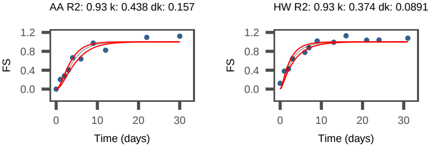

G6PI − SITDIINIGIGGSDLGPLMVTEALKPY_3

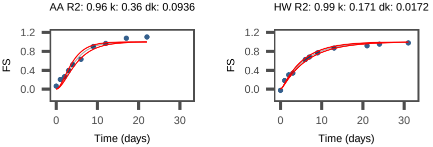

GALK1 − TDGLVSLLTTSK_2

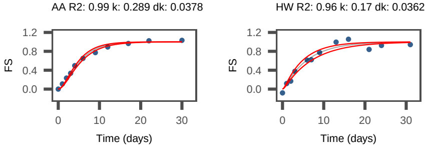

GALM − ILEVYTTQPGVQFYTGNFLDGTLK_3

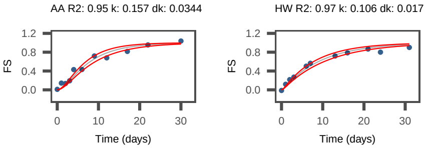

GCKR − ILLETLLLAAHK_3

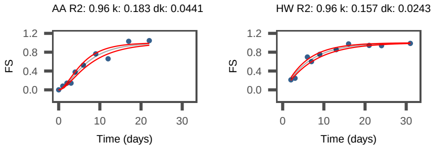

GLNA − ACLYAGVK_2

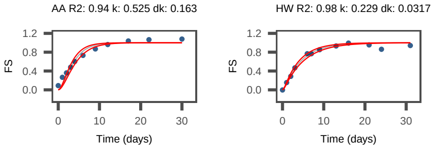

GAMT − TGGVLTYCNLTSWGELMK_2

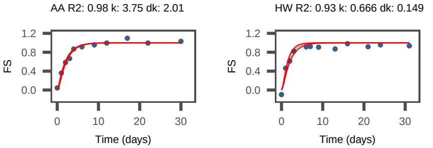

GCSH − SCYEDGWLIK_2

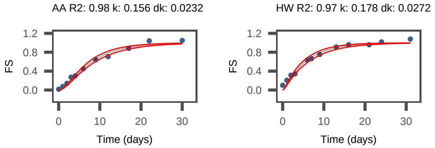

GLNA − LVLCEVFK_2

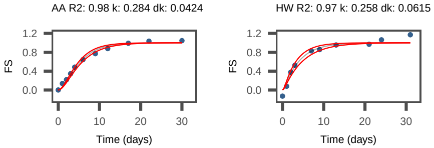

GBB2 − LLLAGYDDFNCNIWDAMK_2

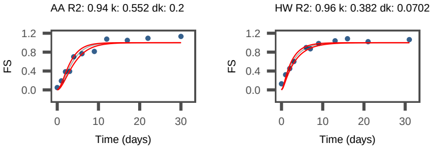

GDIB − FVSISDLFVPK_2

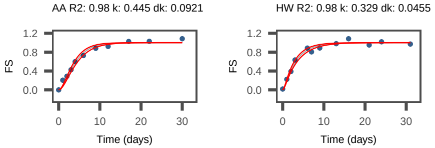

GLNA − QMYMSLPQGEK_2

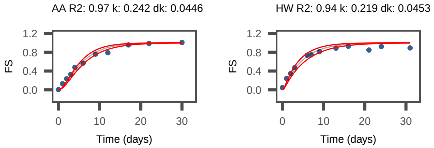

GCDH − DIVYEMGELGVLGPTIK_2

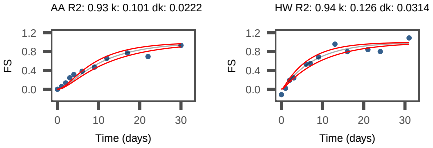

GDIB − VLHMDQNPYYGGESASITPLEDLYK_3

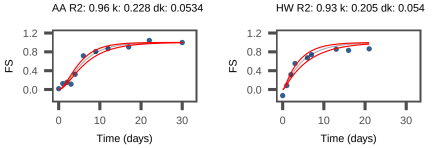

GLNA − TCLLNETGDEPFQYK_2

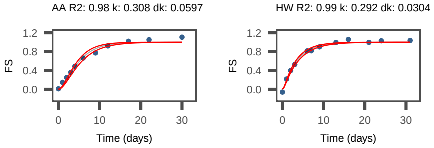

GCDH − DIVYEMGELGVLGPTIK_3

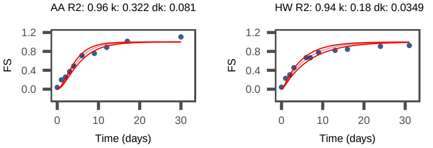

GDIB − VPSTEAEALASSLMGLFEK_3

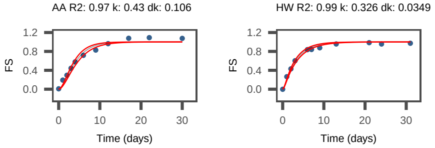

GLNA − TCLLNETGDEPFQYKN_2

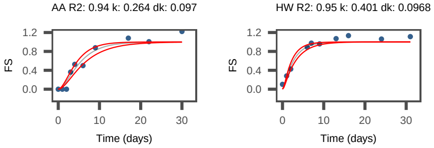

GCDH − HNPSNQSYTLSGTK_2

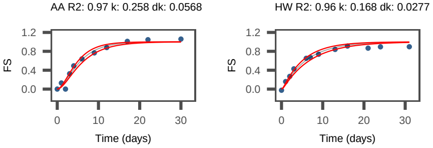

GLGB − EFKDEDWNMGNIVYTLTNR_3

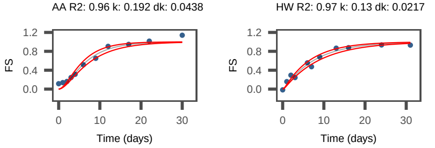

GLO2 − FYEGTADEMYK_2

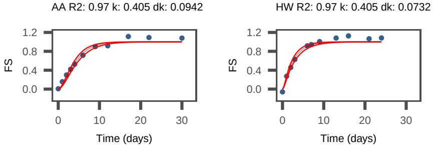

GLO2 − VTHLSTLQVGSLSVK_2

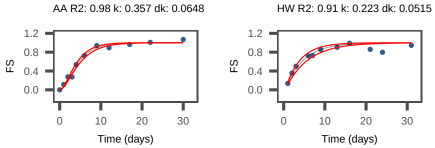

GLYAL − VYGTVFHMNQGNPFK_3

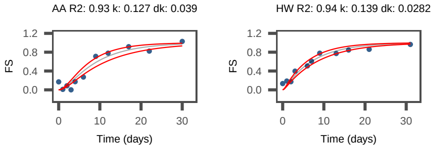

GLYAT − TIQNLASIQSFQIK_2

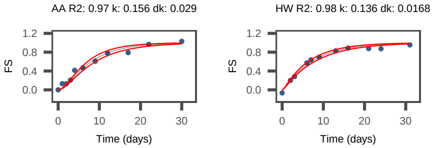

GLOD4 − ELPDLEDLMK_2

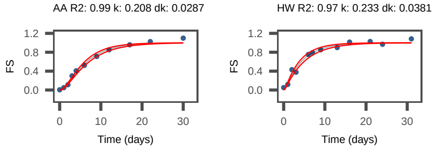

GLYAT − DPQNCQEFLESSEVINWK_2

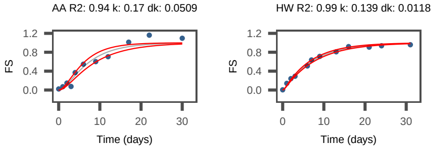

GLYC − AVLEALGSCLNNK_2

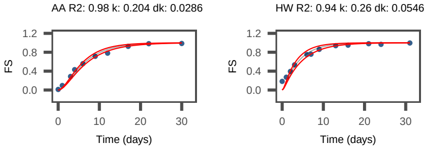

GLSL − MAGNEFMGFSNATFQSEK_2

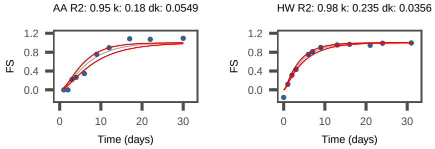

GLYAT − IVPLQGAQMLQMLEK_3

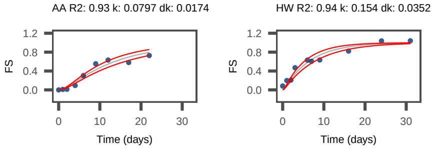

GLYC(Non−Unique) − ISATSIFFESMPYK_2

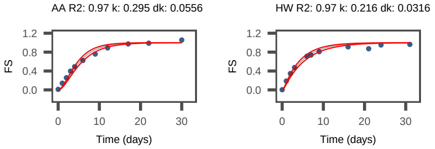

GLSL − VAAYIPHLAK_3

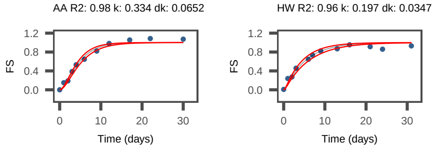

GLYAT − LSSLDVVHAALVNK_3

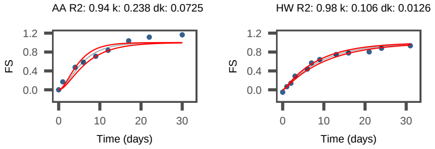

GLYC − VYPETGYINYDQLEENASLFHPK_3

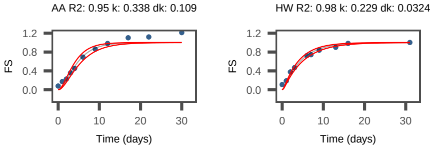

GLYAL − EQEMGDDLDQHTNTYQIYSK_3

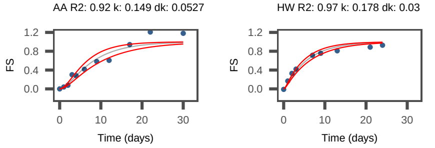

GLYAT − QHLQIQSSQSHLNK_3

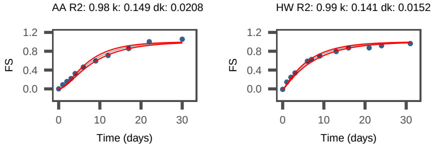

GLYC − YYGGTEFIDELEMLCQK_2

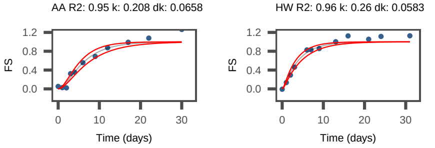

GLYAL − GYPVYNHTEQTNK_3

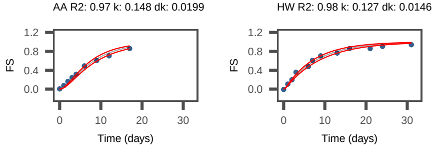

GLYAT − QHLQIQSSQSHLNK_4

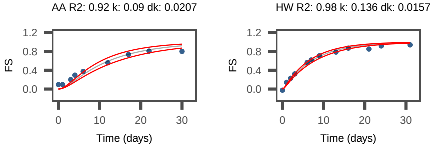

GLYM − GYSLVSGGTDTHLVLVDLRPK_4

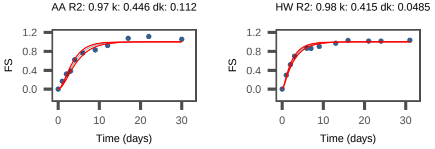

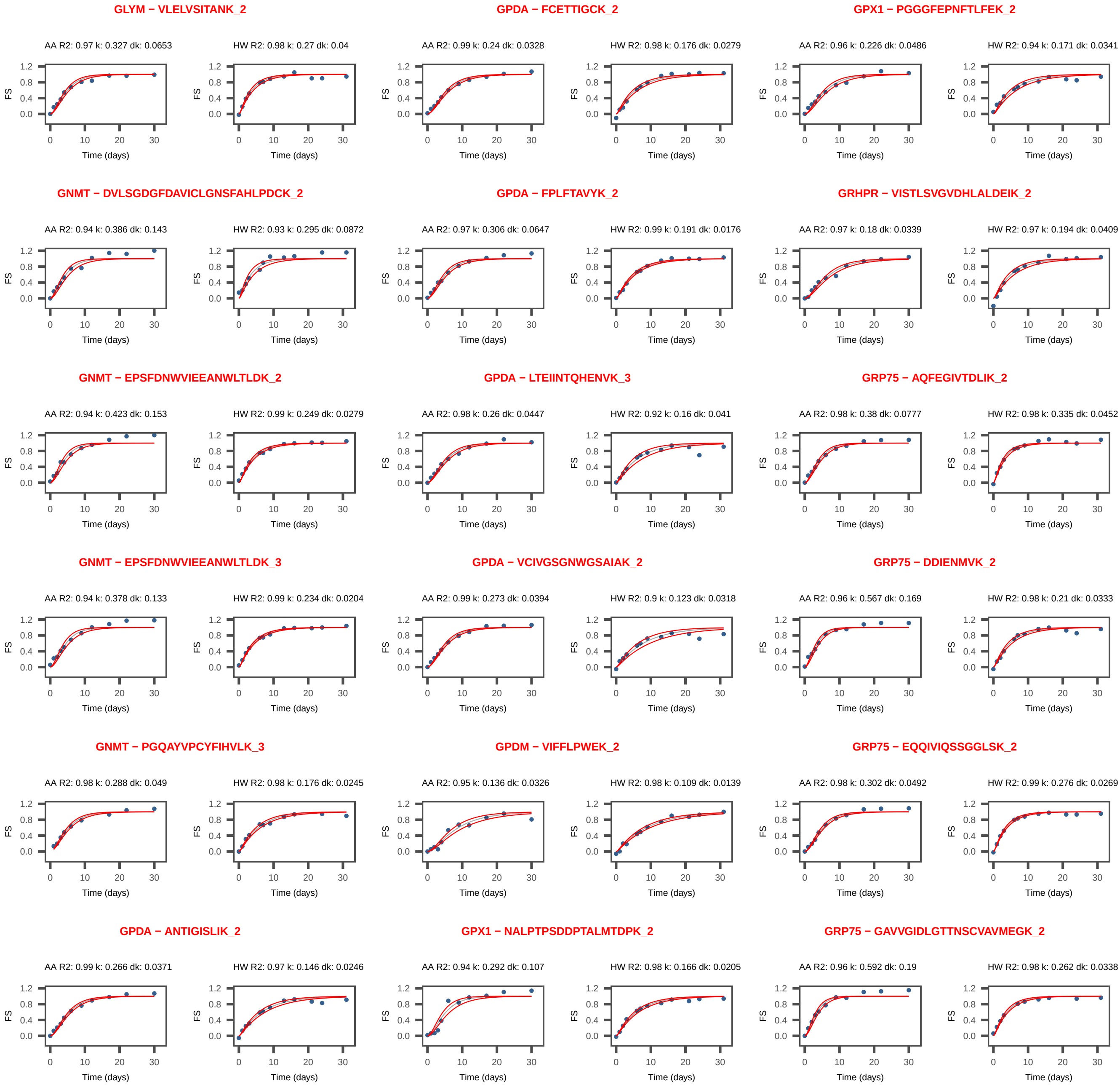
GLYM − VLELVSITANK_2
AA R2: 0.97 k: 0.327 dk: 0.0653
HW R2: 0.98 k: 0.27 dk: 0.04
GPDA − FCETTIGCK_2
AA R2: 0.99 k: 0.24 dk: 0.0328
HW R2: 0.98 k: 0.176 dk: 0.0279
GPX1 − PGGGFEPNFTLFEK_2
AA R2: 0.96 k: 0.226 dk: 0.0486
HW R2: 0.94 k: 0.171 dk: 0.0341
GNMT − DVLSGDGFDAVICLGNSFAHLPDCK_2
AA R2: 0.94 k: 0.386 dk: 0.143
HW R2: 0.93 k: 0.295 dk: 0.0872
GPDA − FPLFTAVYK_2
AA R2: 0.97 k: 0.306 dk: 0.0647
HW R2: 0.99 k: 0.191 dk: 0.0176
GRHPR − VISTLSVGVDHLALDEIK_2
AA R2: 0.97 k: 0.18 dk: 0.0339
HW R2: 0.97 k: 0.194 dk: 0.0409
GNMT − EPSFDNWVIEEANWLTLDK_2
AA R2: 0.94 k: 0.423 dk: 0.153
HW R2: 0.99 k: 0.249 dk: 0.0279
GPDA − LTEIINTQHENVK_3
AA R2: 0.98 k: 0.26 dk: 0.0447
HW R2: 0.92 k: 0.16 dk: 0.041
GRP75 − AQFEGIVTDLIK_2
AA R2: 0.98 k: 0.38 dk: 0.0777
HW R2: 0.98 k: 0.335 dk: 0.0452
GNMT − EPSFDNWVIEEANWLTLDK_3
AA R2: 0.94 k: 0.378 dk: 0.133
HW R2: 0.99 k: 0.234 dk: 0.0204
GPDA − VCIVGSGNWGSAIAK_2
AA R2: 0.99 k: 0.273 dk: 0.0394
HW R2: 0.9 k: 0.123 dk: 0.0318
GRP75 − DDIENMVK_2
AA R2: 0.96 k: 0.567 dk: 0.169
HW R2: 0.98 k: 0.21 dk: 0.0333
GNMT − PGQAYVPCYFIHVLK_3
AA R2: 0.98 k: 0.288 dk: 0.049
HW R2: 0.98 k: 0.176 dk: 0.0245
GPDM − VIFFLPWEK_2
AA R2: 0.95 k: 0.136 dk: 0.0326
HW R2: 0.98 k: 0.109 dk: 0.0139
GRP75 − EQQIVIQSSGGLSK_2
AA R2: 0.98 k: 0.302 dk: 0.0492
HW R2: 0.99 k: 0.276 dk: 0.0269
GPDA − ANTIGISLIK_2
AA R2: 0.99 k: 0.266 dk: 0.0371
HW R2: 0.97 k: 0.146 dk: 0.0246
GPX1 − NALPTPSDDPTALMTDPK_2
AA R2: 0.94 k: 0.292 dk: 0.107
HW R2: 0.98 k: 0.166 dk: 0.0205
GRP75 − GAVVGIDLGTTNSCVAVMEGK_2
AA R2: 0.96 k: 0.592 dk: 0.19
HW R2: 0.98 k: 0.262 dk: 0.0338
FS
Time (days)

GRP75 – QAASSLQQASLK_2

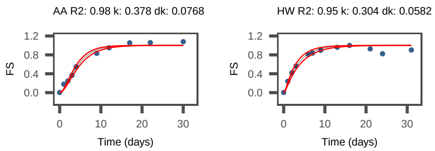

GSH0 – EFPDVLECTMSHAVEK_3

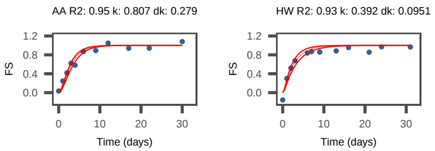

GSTA4 – AILSYLAAK_2

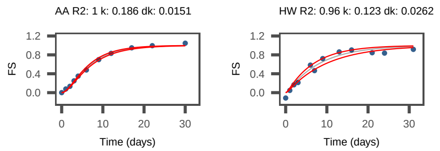

GRP75 – RYDDPEVQK_2

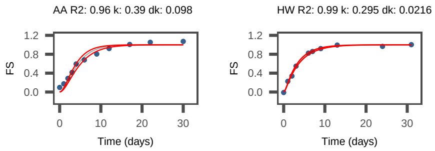

GSH1 – LDFLIPLSK_2

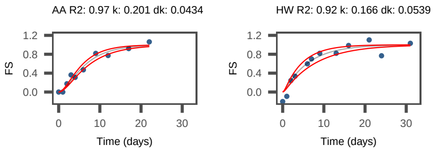

GSTA4 – EKEESYDLILSR_3

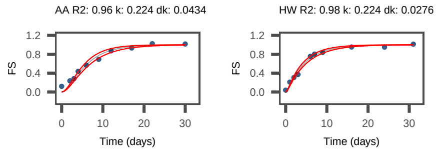

GRP75 – SQVFSTAADGQTQVEIK_2

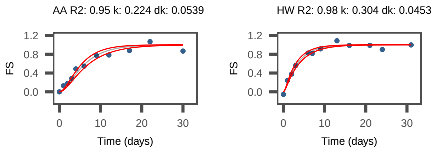

GSTA1_HUMAN,sp|P10648|GSTA2(Non–Unique) – WLLAAAGVEFEEK_2

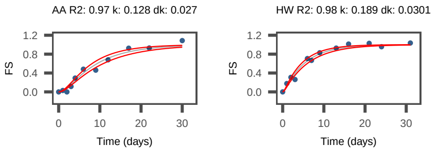

GSTA4 – KPPPDGPYVEVVR_2

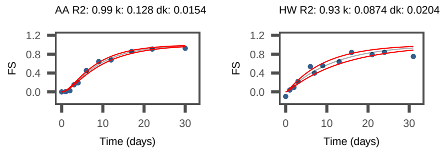

GRP75 – VIAVYDLGGGTFDISILEIQK_2

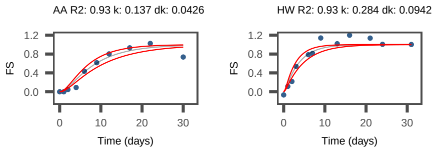

GSTA1_HUMAN,sp|P30115|GSTA3(Non–Unique) – YFPAFEK_2

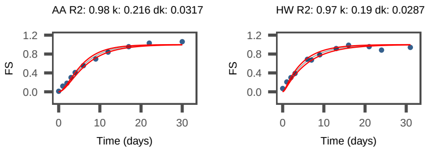

GSTA4 – YFPVFEK_2

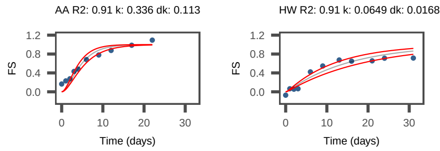

GRP75 – VINEPTAAALAYGLDK_2

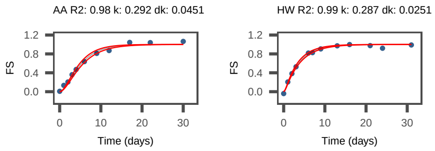

GSTA2(Non–Unique) – YLPAFEK_2

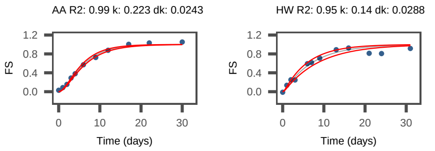

GSTK1 – LELLAYLLGEK_2

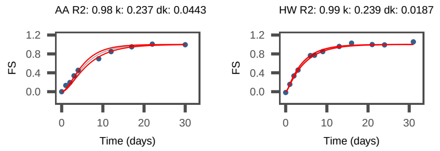

GRPE1 – DLLEVADILEK_2

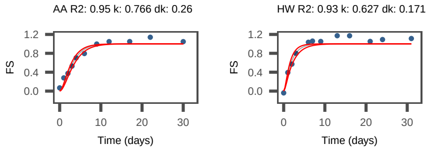

GSTA3 – VSNLPTVK_2

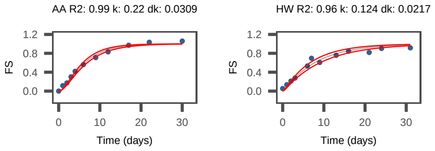

GSTK1 – QFFQVPLNIPK_2

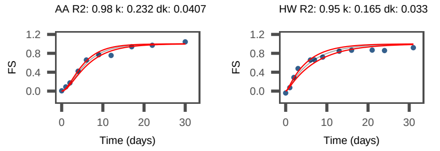

GSTK1 − YGAFGLPTTVAHVDGK_3

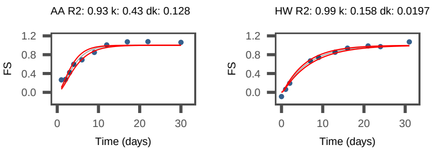

GSTM1(Non−Unique) − LGLDFPNLPYLIDGSHK_3

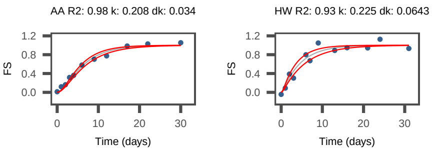

GSTM6 − LDLDFPNLPYLIDGSHK_3

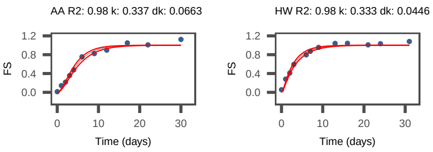

GSTM1 − CYNPDFEK_2

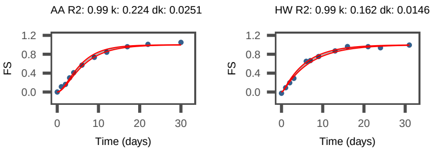

GSTM1 − MLLEYTDSSYDEK_2

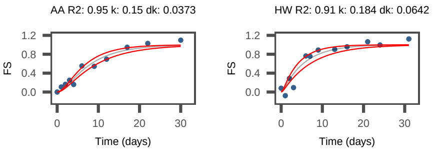

GSTM7 − YTMGDAPDYDQSQWLNEK_2

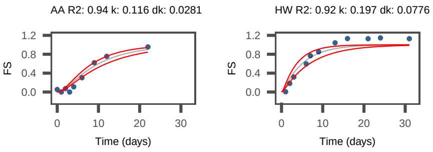

GSTM1 − KHHLDGETEEER_2

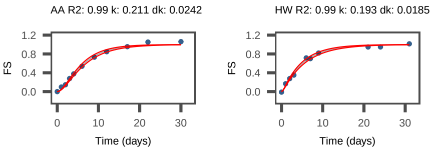

GSTM1 − MLLEYTDSSYDEKR_3

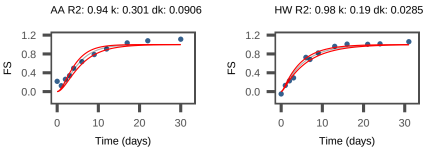

GSTP1(Non−Unique) − AFLSSPEHVNRPINGNGK_3

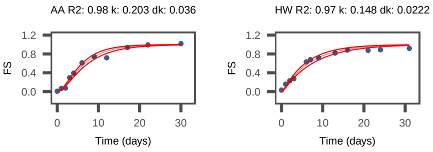

GSTM1 − KHHLDGETEEER_3

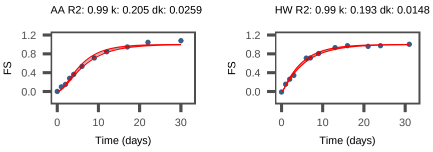

GSTM1(Non−Unique) − RPWFAGDK_2

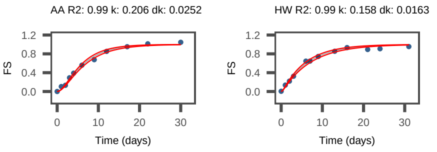

GSTP1(Non−Unique) − EEVVTIDTWMQGLLK_3

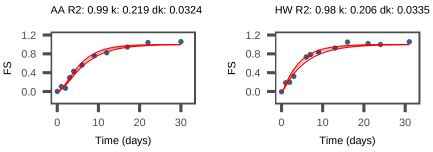

GSTM1 − KHHLDGETEEER_4

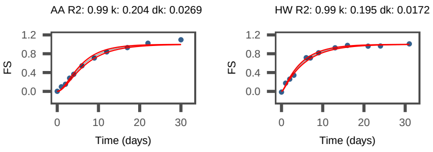

GSTM1 − YIATPIFSK_2

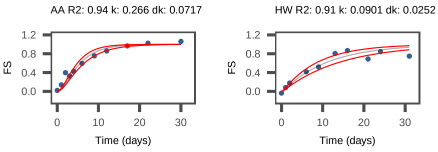

GSTP1(Non−Unique) − PTCLYGQLPK_2

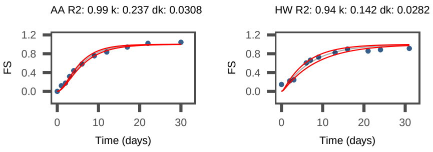

GSTM1 − LCYNPDFEK_2

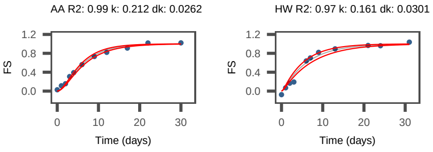

GSTM3 − FNLGLDFPNLPYLIDGSHK_3

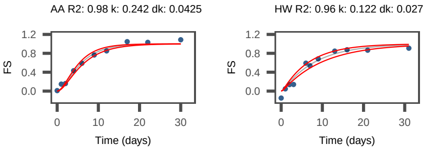

GSTP1(Non−Unique) − SLGLYGK_2

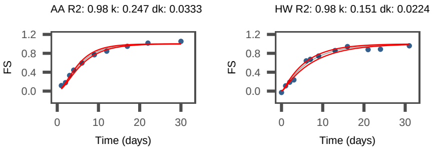

GSTP1_HUMAN,sp|P19157|GSTP1(Non−Unique) − ETLLSQNQGGK_2

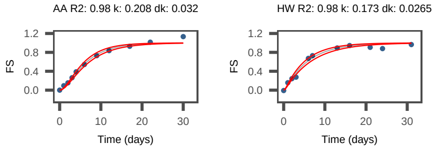

H12(Non−Unique) − SGVSLAALK_2

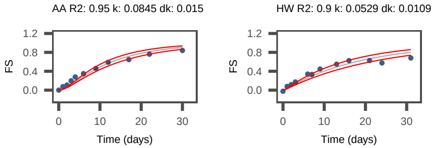

H2AZ(Non−Unique) − ATIAGGGVIPHIHK_2

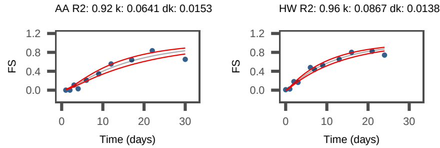

GSTP1_HUMAN,sp|P19157|GSTP1(Non−Unique) − MLLADQGQSWK_2

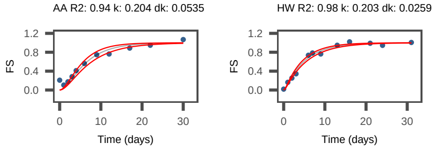

H14 − TSGPPVSELITK_2

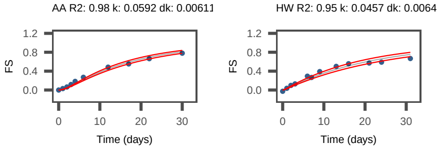

H2AZ(Non−Unique) − SAAILEYLTAEVLELAGNASK_3

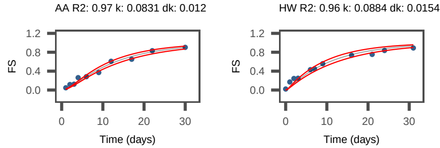

GSTP1_HUMAN,sp|P19157|GSTP1(Non−Unique) − PFETLLSQNQGGK_2

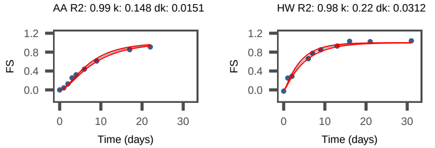

H2A1B(Non−Unique) − VTIAQGGVLPNIQAVLLPK_2

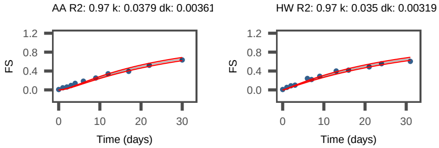

H2B1F(Non−Unique) − QVHPDTGISSK_2

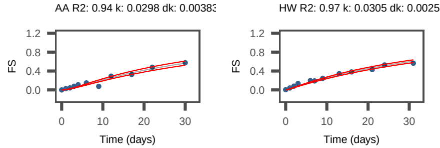

GSTT2 − GQHMSEQFSQVNCLNK_2

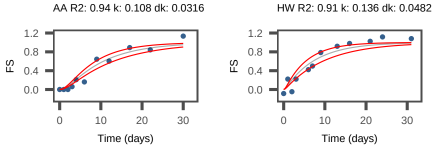

H2A1B(Non−Unique) − VTIAQGGVLPNIQAVLLPK_3

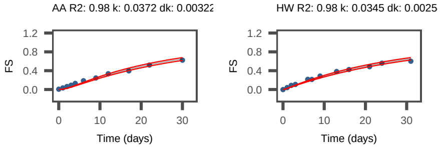

H2B1F(Non−Unique) − QVHPDTGISSK_3

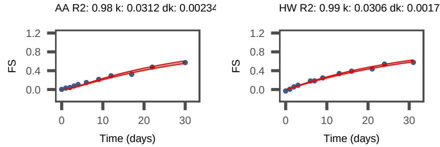

GSTT2 − VLGPLIGVQVPQEK_2

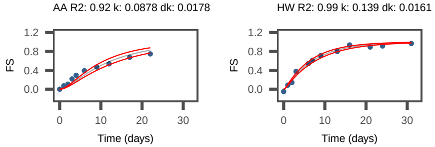

H2AX(Non−Unique) − LLGGVTIAQGGVLPNIQAVLLPK_3

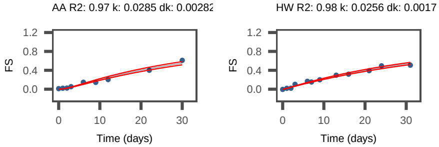

H4 − DAVTYTEHAK_3

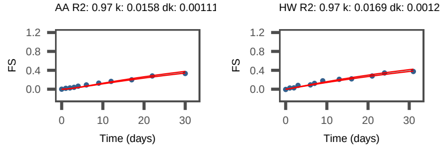

GYS2 − SPFSLNHFPK_3

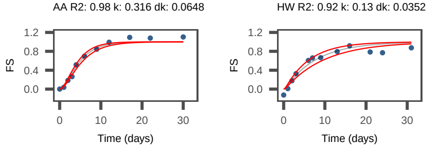

H2AY − GVTIASGGVLPNIHPELLAK_3

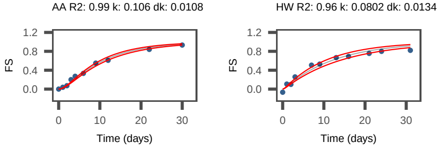

HACL1 − LDAGSFGTMGVGLGFAIAAALVAK_3

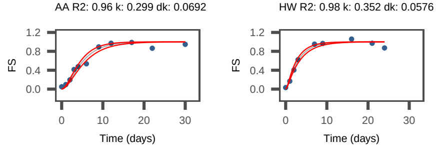

HACL1 − LVEQCSLPFLPTPMGK_2

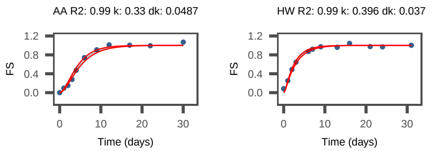

HBA − MFASFPTTK_2

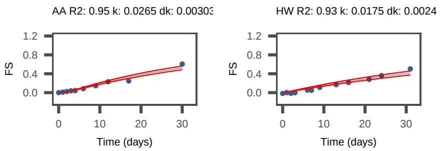

HCD2 − VVTIAPGLFATPLLTTLPEK_3

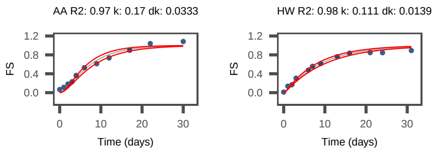

HACL1 − PGACYIDIPADFVTLQANVTSIK_2

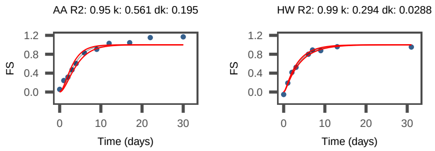

HBA − TYFPHFDVSHGSAQVK_3

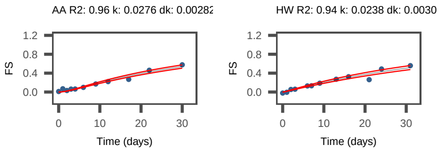

HCDH − LGAGYPMGPFELLDYVGLDTTK_2

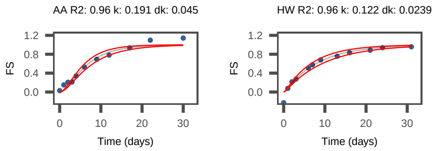

HACL1 − PGACYIDIPADFVTLQANVTSIK_3

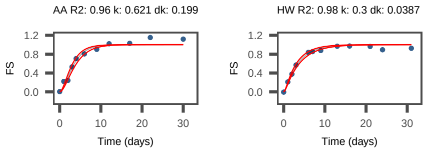

HBB1(Non−Unique) − GTFASLSELHCDK_3

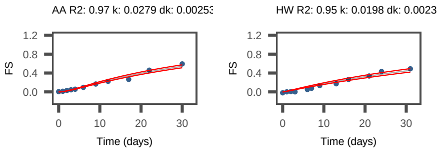

HCDH − LGAGYPMGPFELLDYVGLDTTK_3

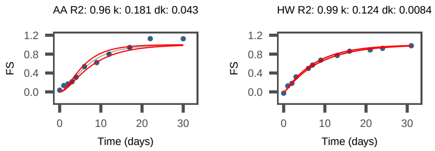

HACL1 − QPLLIIGK_2

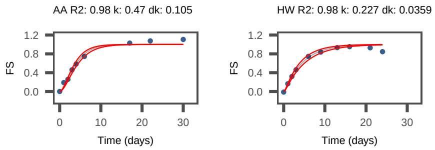

HCD2 − GVIINTASVAAFEGQVGQAAYSASK_2

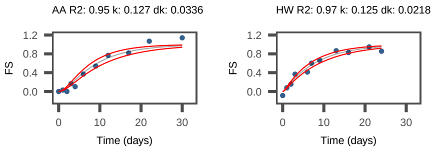

HCDH − TFESLVDFCK_2

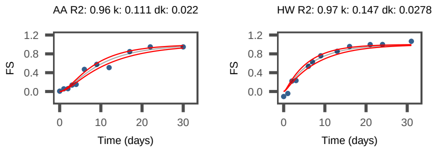

HACL1 − TPWQCPPDSQWWK_2

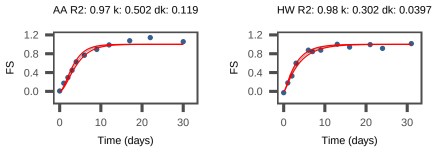

HCD2 − LGESCIFAPANVTSEK_2

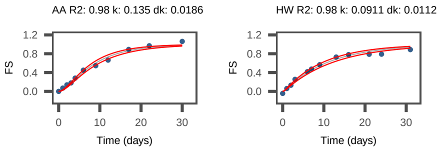

HCDH − TVVLVDQTEDILAK_2

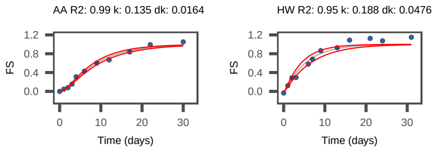

HBA(Non−Unique) − LRVDPVNFK_3

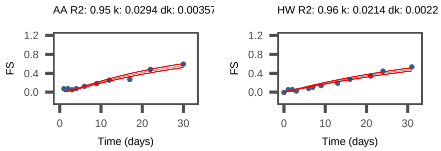

HCD2 − VVTIAPGLFATPLLTTLPEK_2

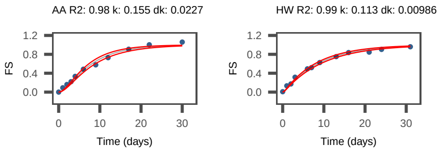

HDHD2 − LLLDGAPLIAIHK_3

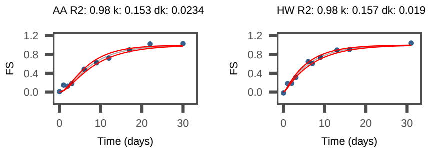

HEM2 − AGADIIITYFAPQLLK_2

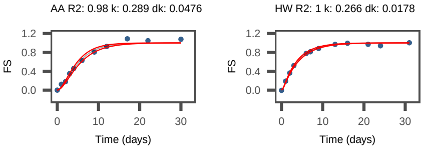

HEM2 − AGADIIITYFAPQLLK_3

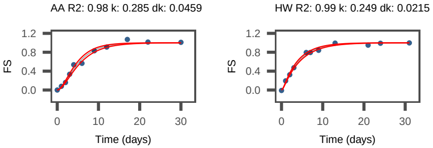

HGD − GPDADCFEK_2

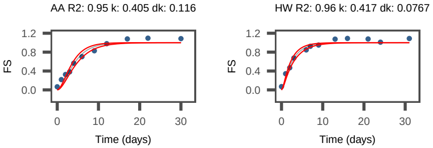

HGD − MVINAVAFDHADPSIFTVLTAK_3

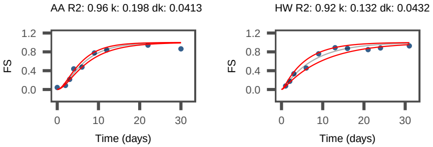

HGD − TCSCLDENYYK_2

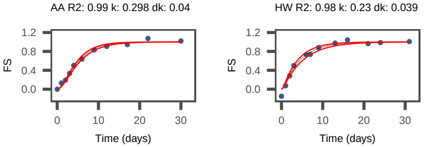

HIBCH − AFCAGGDIK_2

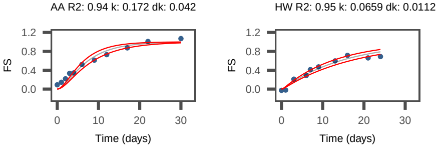

HIBCH − AGIATHFVDSEK_2

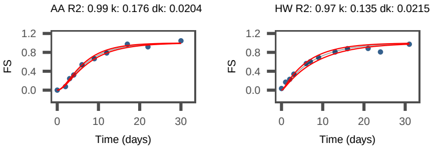

HIBCH − VLEEELLALK_2

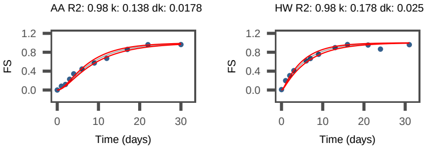

HINT1 − AQVAQPGGDTIFGK_2

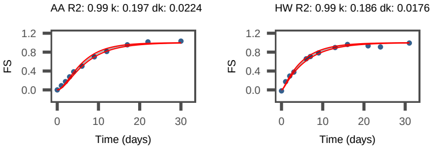

HINT1 − HISQISVADDDDESLLGHLMIVGK_3

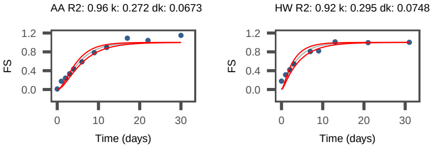

HIUH − GQESFYPYVEVVFTITK_2

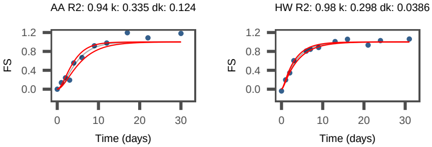

HIUH − GQESFYPYVEVVFTITK_3

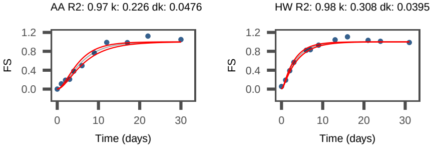

HMCS2 − ASLDMFNQK_2

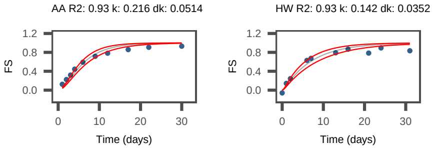

HMCS2 − DVGILALEVYFPAQYVDQTDLEK_2

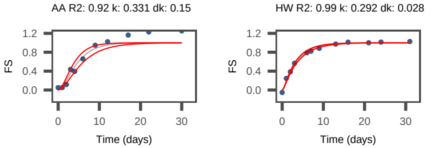

HMCS2 − GTHMENAYDFYKPN_2

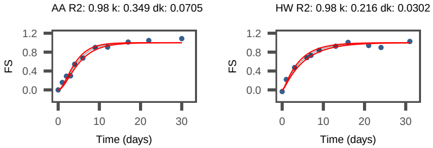

HMCS2 − GTHMENAYDFYKPN_3

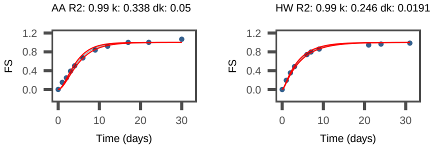

HMCS2 − LASEYPLVDGK_2

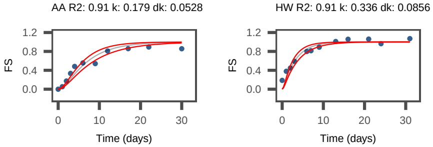

HMCS2(Non−Unique) − LEVGTETIIDK_2

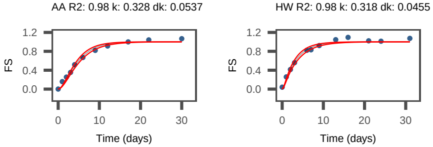

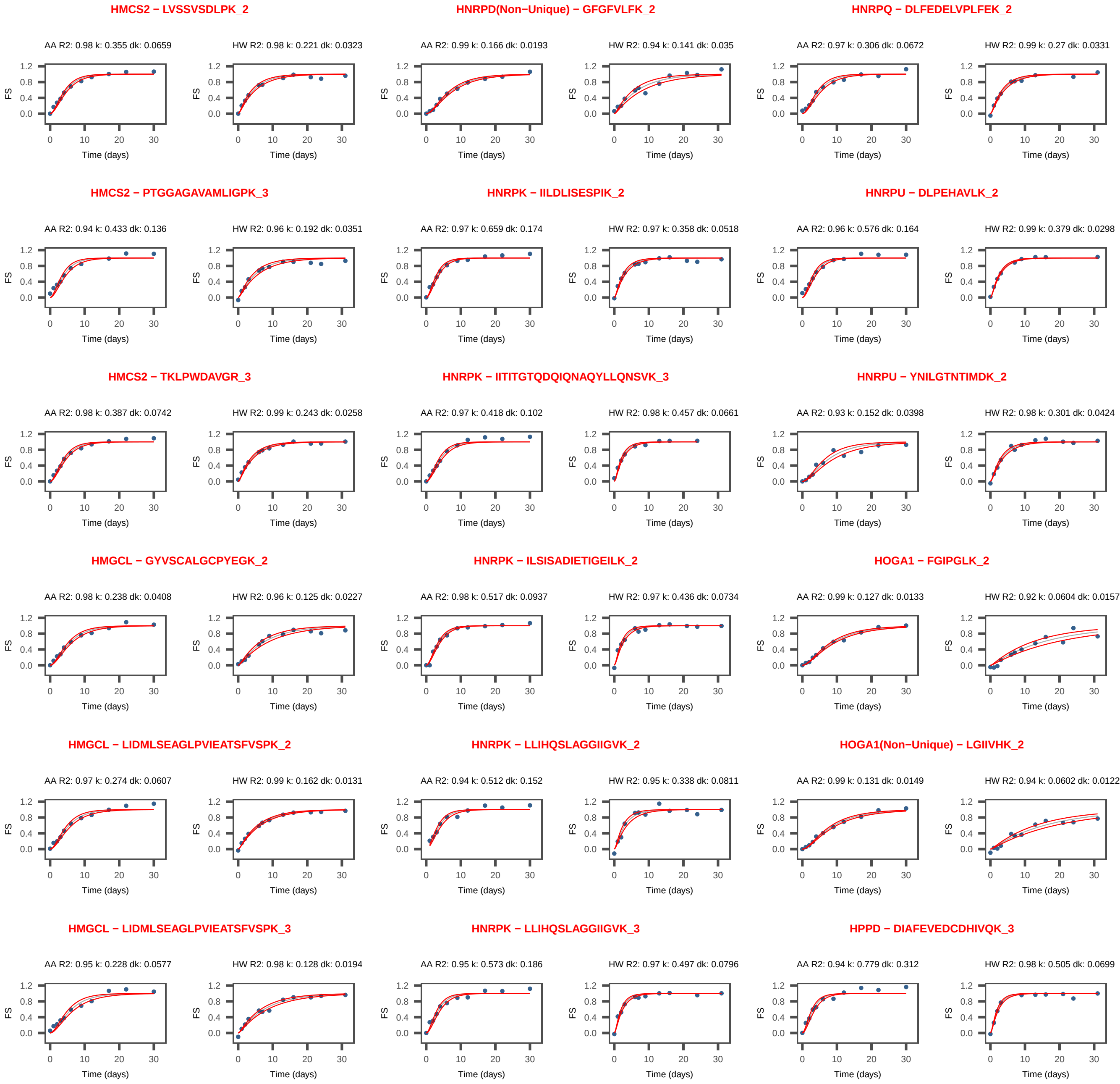
HMCS2 − LVSSVSDLPK_2
AA R2: 0.98 k: 0.355 dk: 0.0659
HW R2: 0.98 k: 0.221 dk: 0.0323
HNRPD(Non−Unique) − GFGFVLFK_2
AA R2: 0.99 k: 0.166 dk: 0.0193
HW R2: 0.94 k: 0.141 dk: 0.035
HNRPQ − DLFEDELVPLFEK_2
AA R2: 0.97 k: 0.306 dk: 0.0672
HW R2: 0.99 k: 0.27 dk: 0.0331
HMCS2 − PTGGAGAVAMLIGPK_3
AA R2: 0.94 k: 0.433 dk: 0.136
HW R2: 0.96 k: 0.192 dk: 0.0351
HNRPK − IILDLISESPIK_2
AA R2: 0.97 k: 0.659 dk: 0.174
HW R2: 0.97 k: 0.358 dk: 0.0518
HNRPU − DLPEHAVLK_2
AA R2: 0.96 k: 0.576 dk: 0.164
HW R2: 0.99 k: 0.379 dk: 0.0298
HMCS2 − TKLPWDAVGR_3
AA R2: 0.98 k: 0.387 dk: 0.0742
HW R2: 0.99 k: 0.243 dk: 0.0258
HNRPK − IITITGTQDQIQNAQYLLQNSVK_3
AA R2: 0.97 k: 0.418 dk: 0.102
HW R2: 0.98 k: 0.457 dk: 0.0661
HNRPU − YNILGTNTIMDK_2
AA R2: 0.93 k: 0.152 dk: 0.0398
HW R2: 0.98 k: 0.301 dk: 0.0424
HMGCL − GYVSCALGCPYEGK_2
AA R2: 0.98 k: 0.238 dk: 0.0408
HW R2: 0.96 k: 0.125 dk: 0.0227
HNRPK − ILSISADIETIGEILK_2
AA R2: 0.98 k: 0.517 dk: 0.0937
HW R2: 0.97 k: 0.436 dk: 0.0734
HOGA1 − FGIPGLK_2
AA R2: 0.99 k: 0.127 dk: 0.0133
HW R2: 0.92 k: 0.0604 dk: 0.0157
HMGCL − LIDMLSEAGLPVIEATSFVSPK_2
AA R2: 0.97 k: 0.274 dk: 0.0607
HW R2: 0.99 k: 0.162 dk: 0.0131
HNRPK − LLIHQSLAGGIIGVK_2
AA R2: 0.94 k: 0.512 dk: 0.152
HW R2: 0.95 k: 0.338 dk: 0.0811
HOGA1(Non−Unique) − LGIIVHK_2
AA R2: 0.99 k: 0.131 dk: 0.0149
HW R2: 0.94 k: 0.0602 dk: 0.0122
HMGCL − LIDMLSEAGLPVIEATSFVSPK_3
AA R2: 0.95 k: 0.228 dk: 0.0577
HW R2: 0.98 k: 0.128 dk: 0.0194
HNRPK − LLIHQSLAGGIIGVK_3
AA R2: 0.95 k: 0.573 dk: 0.186
HW R2: 0.97 k: 0.497 dk: 0.0796
HPPD − DIAFEVEDCDHIVQK_3
AA R2: 0.94 k: 0.779 dk: 0.312
HW R2: 0.98 k: 0.505 dk: 0.0699
FS
Time (days)

HPPD − EVVSHVIK_2

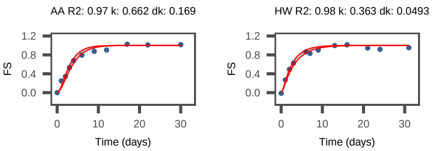

HS90A(Non−Unique) − ADLINNLGTIAK_2

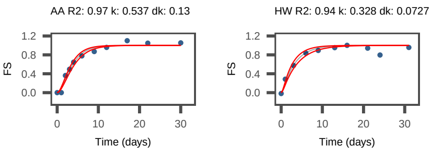

HS90B − NPDDITQEEYGEFYK_2

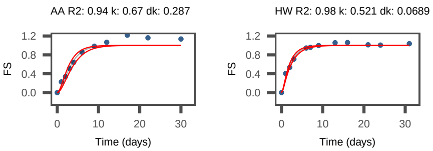

HPPD − GYLLQIFTK_2

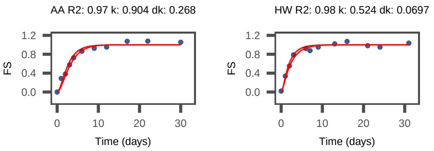

HS90A − ELHINLIPSK_3

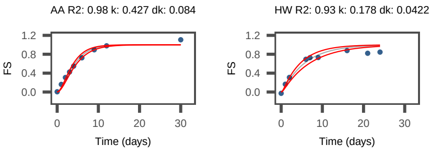

HSDL2 − DEQQINSAVEK_2

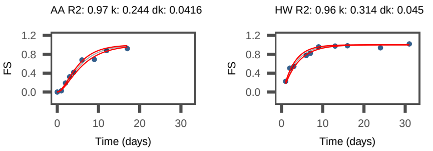

HPPD − SIVVTNYEESIK_2

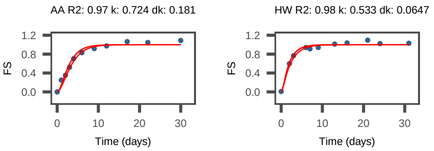

HS90A − NPDDITNEEYGEFYK_2

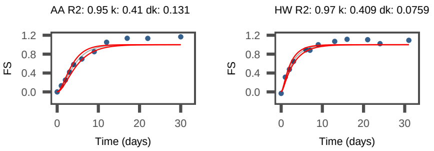

HSDL2 − LQLQEESQLQK_2

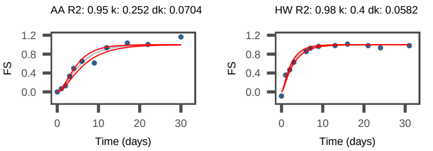

HPRT − FFADLLDYIK_2

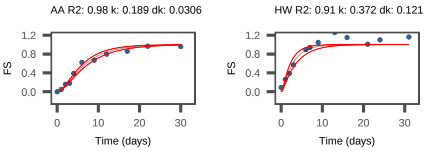

HS90A(Non−Unique) − YESLTDPSK_2

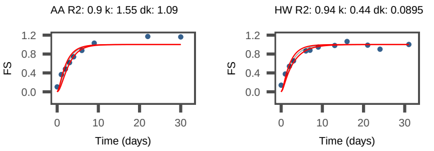

HSP74 − STNEAMEWMNSK_2

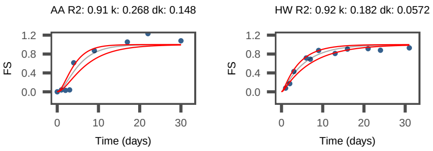

HS71L(Non−Unique) − IINEPTAAAIAYGLDK_2

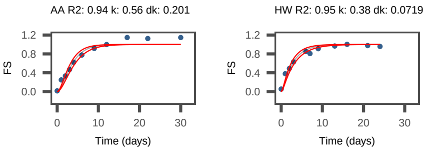

HS90A − YYTSASGDEMVSLK_2

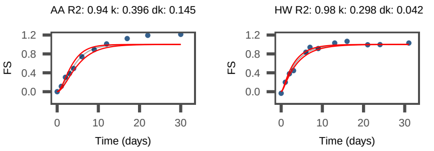

HSP7C − GPAVGIDLGTTYSCVGVFQHGK_3

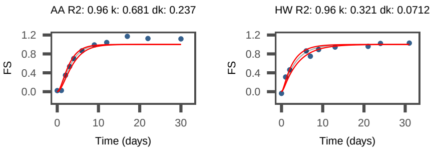

HS74L(Non−Unique) − LMNETTAVALAYGIYK_2

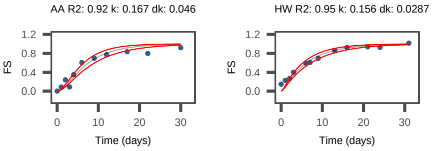

HS90B − DNSTMGYMMAK_2

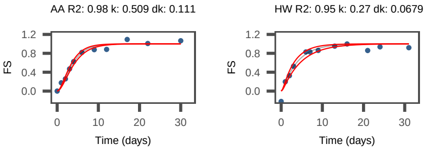

HSP7C − SINPDEAVAYGAAVQAAILSGDK_2

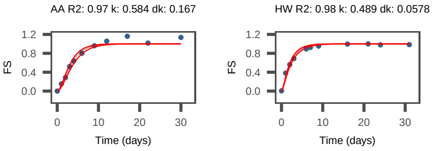

HSP7C − SINPDEAVAYGAAVQAAILSGDK_3

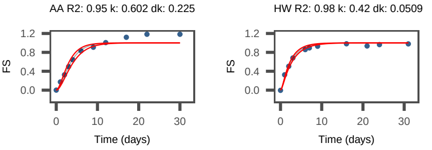

HUTI − SLAVLEGASVVVGTDGLIK_2

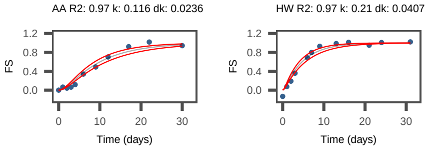

HYEP − SAFPSEILHAPEK_3

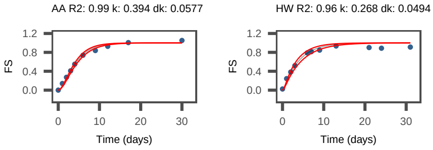

HUTH − CTAAALVSESK_2

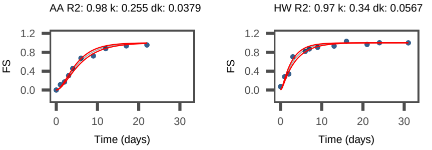

HUTU − VAIAVAINQAIASGK_2

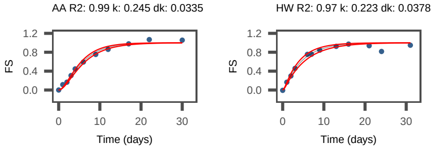

HYEP − VETSDEEIKDLHQR_4

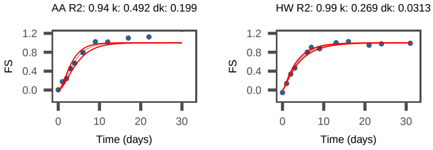

HUTH − GETISGGNFHGEYPAK_2

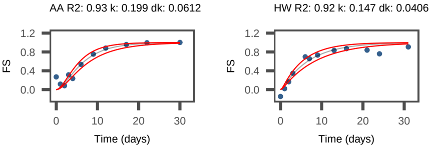

HUTU − VFVTSGLGGMSGAQAK_2

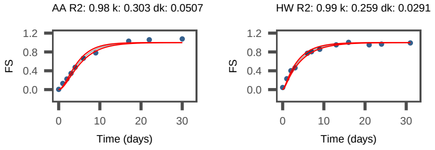

HYES − ASDETGFIAVHK_2

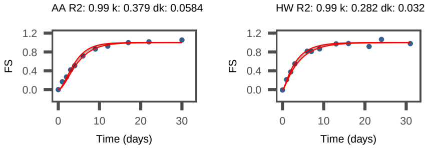

HUTH − QADIVAALTLEVLK_2

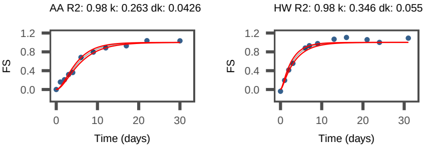

HYEP − IEGLDIHFIHVKPPQLPSGR_4

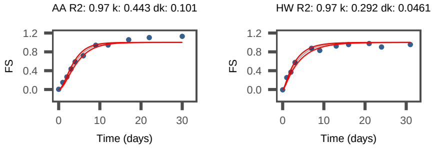

HYES − ASDETGFIAVHK_3

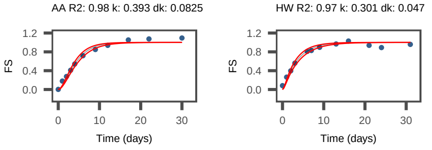

HUTI − AVGPAAVIQK_2

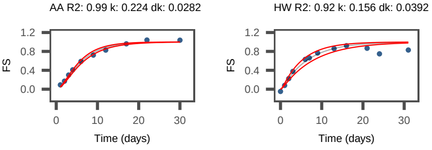

HYEP − IEGLDIHFIHVKPPQLPSGR_5

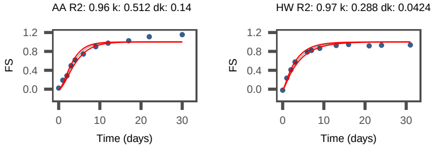

HYES − AVASLNTPFMPPDPDVSPMK_2

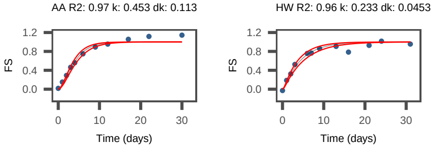

HUTI − MSMPEALAAATINAAYALGK_3

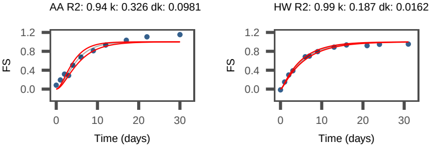

HYEP − IIPLLTDPK_2

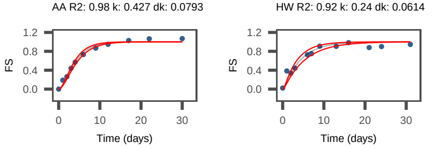

HYES − AVASLNTPFMPPDPDVSPMK_3

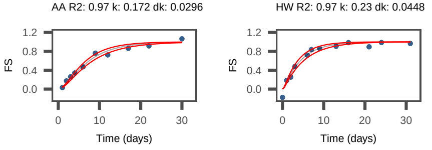

HYES − DFLLGAYQTEFPEGPTEQLMK_2

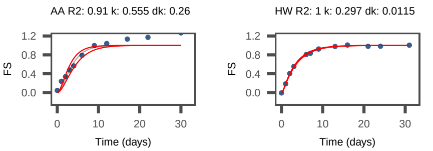

HYES − SINRPMLQAAIALK_3

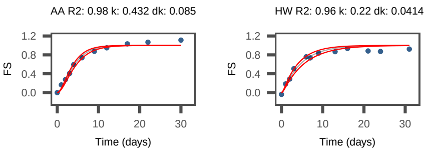

IDHC − ALEDVCIETIEAGFMTK_3

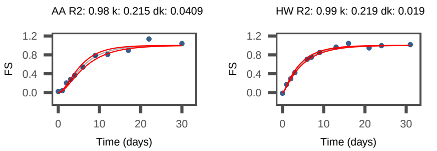

HYES − EMVTFLDK_2

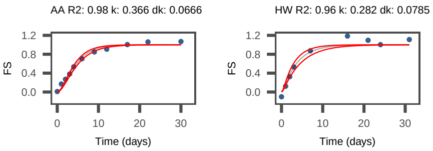

HYES − WLQTEVQNPSVTSK_2

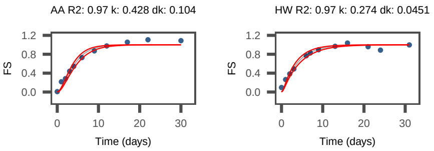

IDHC − GWPLYLSTK_2

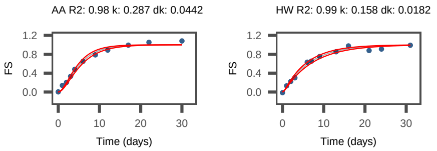

HYES − GFTTCIVTNNWLDDGDKR_3

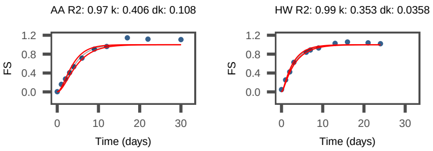

IAH1 − DCGTDVLDLWTLMQK_2

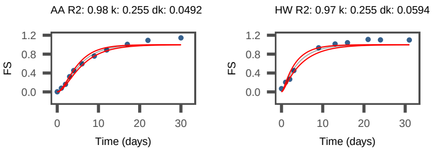

IDHC(Non−Unique) − IIWELIK_2

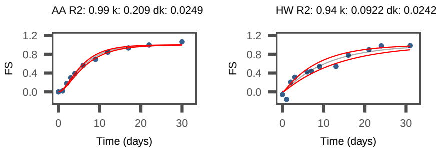

HYES − ILVPALMVTAEK_2

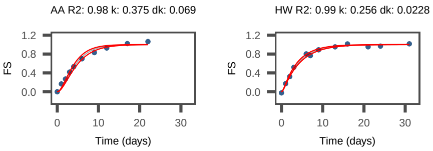

IAH1 − VILITPPPLCEAAWEK_2

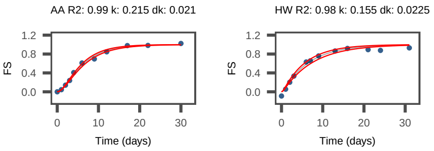

IDHC − LIDDMVAQAMK_2

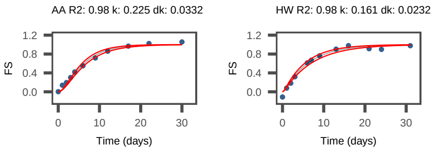

HYES − ITTEEEIEFYIQQFK_3

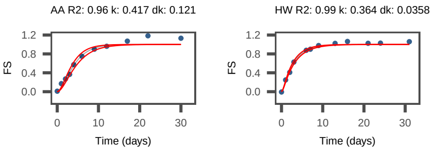

IAH1 − VSSLPWLLPYWK_2

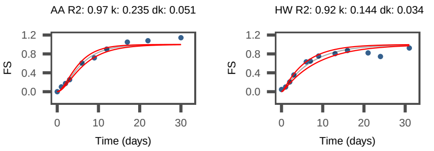

IDHC − LVTGWVKPIIIGR_3

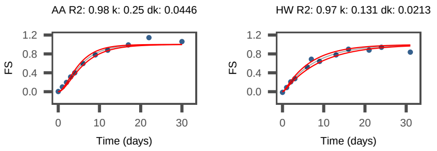

HYES − IYNFLLDTLK_2

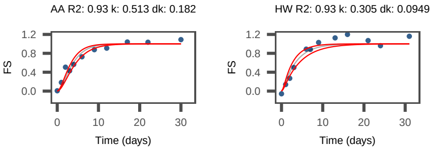

IDHC − ALEDVCIETIEAGFMTK_2

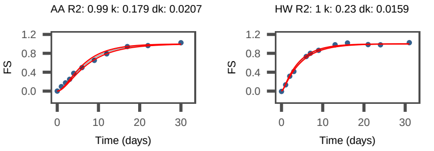

IDHC − SDYLNTFEFMDK_2

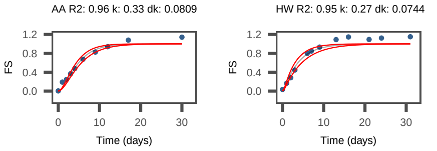

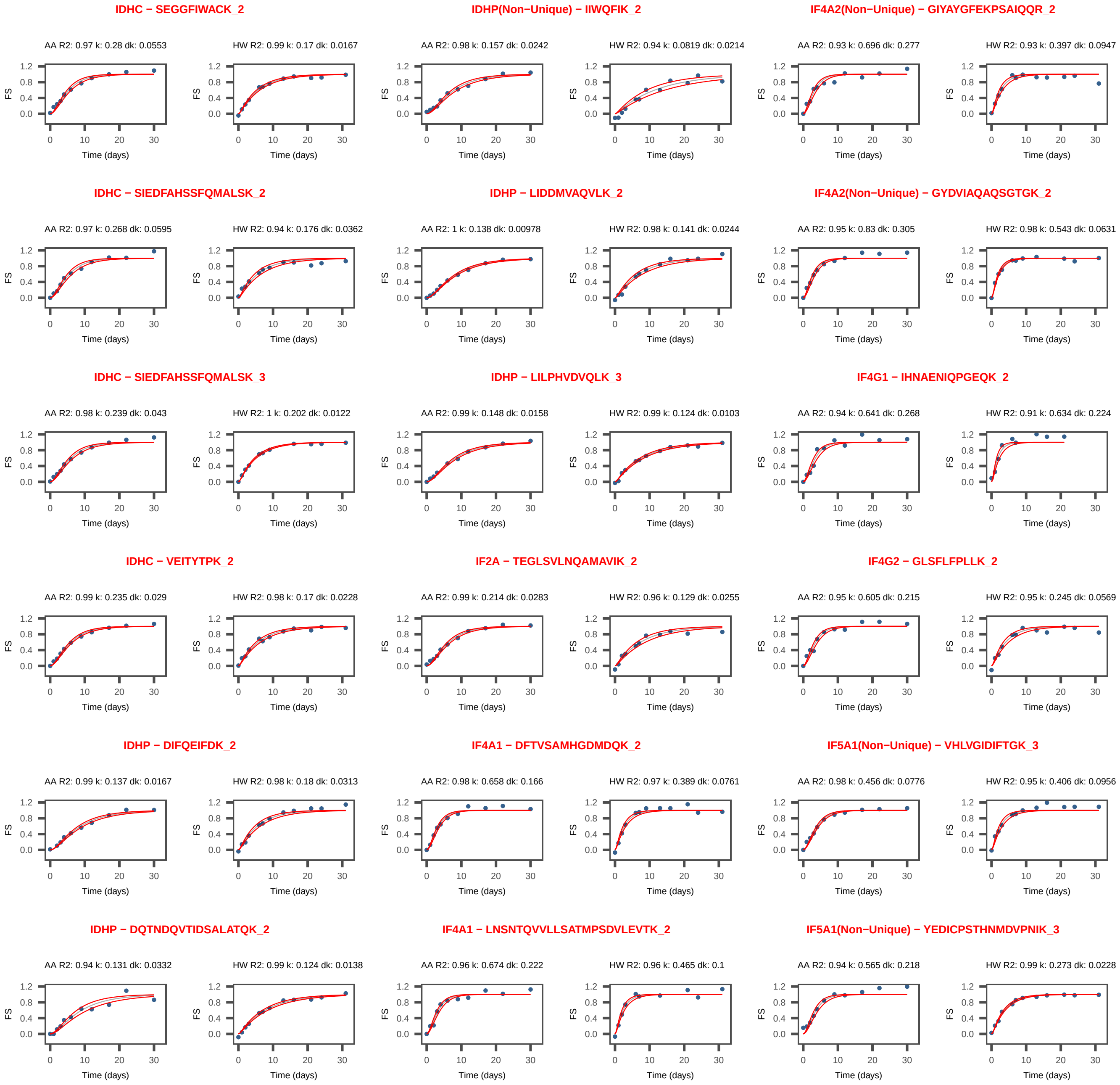
IDHC − SEGGFIWACK_2
AA R2: 0.97 k: 0.28 dk: 0.0553
HW R2: 0.99 k: 0.17 dk: 0.0167
IDHP(Non−Unique) − IIWQFIK_2
AA R2: 0.98 k: 0.157 dk: 0.0242
HW R2: 0.94 k: 0.0819 dk: 0.0214
IF4A2(Non−Unique) − GIYAYGFEKPSAIQQR_2
AA R2: 0.93 k: 0.696 dk: 0.277
HW R2: 0.93 k: 0.397 dk: 0.0947
IDHC − SIEDFAHSSFQMALSK_2
AA R2: 0.97 k: 0.268 dk: 0.0595
HW R2: 0.94 k: 0.176 dk: 0.0362
IDHP − LIDDMVAQVLK_2
AA R2: 1 k: 0.138 dk: 0.00978
HW R2: 0.98 k: 0.141 dk: 0.0244
IF4A2(Non−Unique) − GYDVIAQAQSGTGK_2
AA R2: 0.95 k: 0.83 dk: 0.305
HW R2: 0.98 k: 0.543 dk: 0.0631
IDHC − SIEDFAHSSFQMALSK_3
AA R2: 0.98 k: 0.239 dk: 0.043
HW R2: 1 k: 0.202 dk: 0.0122
IDHP − LILPHVDVQLK_3
AA R2: 0.99 k: 0.148 dk: 0.0158
HW R2: 0.99 k: 0.124 dk: 0.0103
IF4G1 − IHNAENIQPGEQK_2
AA R2: 0.94 k: 0.641 dk: 0.268
HW R2: 0.91 k: 0.634 dk: 0.224
IDHC − VEITYTPK_2
AA R2: 0.99 k: 0.235 dk: 0.029
HW R2: 0.98 k: 0.17 dk: 0.0228
IF2A − TEGLSVLNQAMAVIK_2
AA R2: 0.99 k: 0.214 dk: 0.0283
HW R2: 0.96 k: 0.129 dk: 0.0255
IF4G2 − GLSFLFPLLK_2
AA R2: 0.95 k: 0.605 dk: 0.215
HW R2: 0.95 k: 0.245 dk: 0.0569
IDHP − DIFQEIFDK_2
AA R2: 0.99 k: 0.137 dk: 0.0167
HW R2: 0.98 k: 0.18 dk: 0.0313
IF4A1 − DFTVSAMHGDMDQK_2
AA R2: 0.98 k: 0.658 dk: 0.166
HW R2: 0.97 k: 0.389 dk: 0.0761
IF5A1(Non−Unique) − VHLVGIDIFTGK_3
AA R2: 0.98 k: 0.456 dk: 0.0776
HW R2: 0.95 k: 0.406 dk: 0.0956
IDHP − DQTNDQVTIDSALATQK_2
AA R2: 0.94 k: 0.131 dk: 0.0332
HW R2: 0.99 k: 0.124 dk: 0.0138
IF4A1 − LNSNTQVVLLSATMPSDVLEVTK_2
AA R2: 0.96 k: 0.674 dk: 0.222
HW R2: 0.96 k: 0.465 dk: 0.1
IF5A1(Non−Unique) − YEDICPSTHNMDVPNIK_3
AA R2: 0.94 k: 0.565 dk: 0.218
HW R2: 0.99 k: 0.273 dk: 0.0228
FS
Time (days)

IMB1 − GALQYLVPILTQTLTK_3

AA R2: 0.91 k: 0.277 dk: 0.0891

HW R2: 0.97 k: 0.313 dk: 0.0563

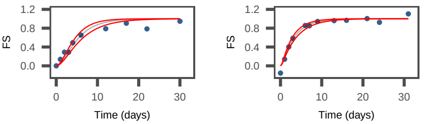

INMT − VYIGGEDYEK_2

AA R2: 0.99 k: 0.205 dk: 0.0268

HW R2: 0.97 k: 0.221 dk: 0.0458

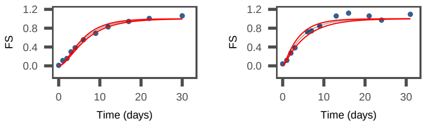

IPYR − GYIWNYGAIPQTWEDPGHSDK_3

AA R2: 0.92 k: 0.405 dk: 0.159

HW R2: 0.99 k: 0.208 dk: 0.0202

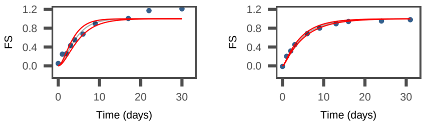

INMT − AIQDAGCQVLK_2

AA R2: 0.99 k: 0.198 dk: 0.0251

HW R2: 0.99 k: 0.181 dk: 0.0139

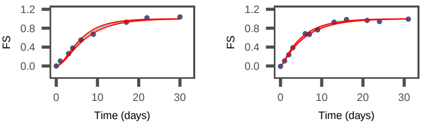

INPP − TLADVLVQEVIK_2

AA R2: 0.95 k: 0.09 dk: 0.0181

HW R2: 0.92 k: 0.144 dk: 0.0366

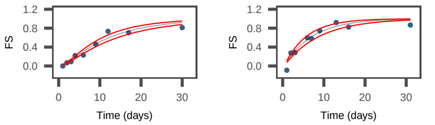

IPYR − LKPGYLEATVDWFR_3

AA R2: 0.98 k: 0.246 dk: 0.0362

HW R2: 0.99 k: 0.213 dk: 0.0159

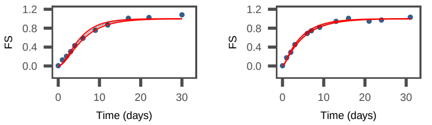

INMT − DYLTTYYSFHSGPVAEQEIVK_2

AA R2: 0.97 k: 0.21 dk: 0.0475

HW R2: 1 k: 0.173 dk: 0.0118

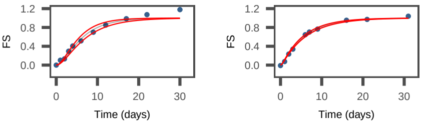

IPYR − AIVDALPPPCESACSLPTDVDK_2

AA R2: 0.93 k: 0.142 dk: 0.0337

HW R2: 0.94 k: 0.337 dk: 0.106

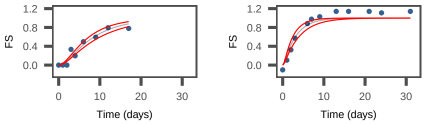

IPYR − VIAINVDDPDAANYK_2

AA R2: 0.97 k: 0.274 dk: 0.0579

HW R2: 0.99 k: 0.245 dk: 0.0266

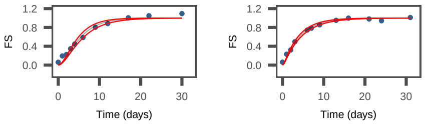

INMT − FSGVYLEK_2

AA R2: 0.96 k: 0.318 dk: 0.0675

HW R2: 0.98 k: 0.18 dk: 0.0246

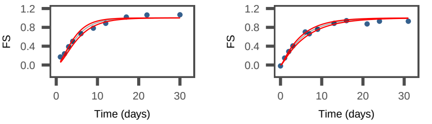

IPYR − DFAVDIIK_2

AA R2: 0.99 k: 0.245 dk: 0.0324

HW R2: 0.97 k: 0.196 dk: 0.0394

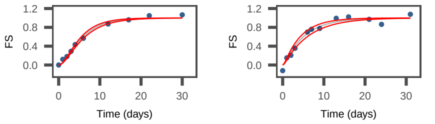

IPYR − VLGILAMIDEGETDWK_2

AA R2: 0.96 k: 0.372 dk: 0.107

HW R2: 0.99 k: 0.236 dk: 0.0268

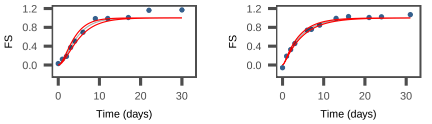

INMT − KEPGAYDWSSIVQH_3

AA R2: 0.93 k: 0.227 dk: 0.0634

HW R2: 0.97 k: 0.141 dk: 0.0216

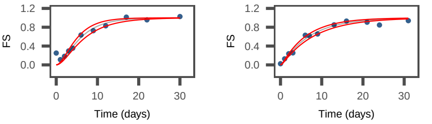

IPYR − GISCMNTTVSESPFK_2

AA R2: 0.96 k: 0.298 dk: 0.0739

HW R2: 0.98 k: 0.161 dk: 0.0185

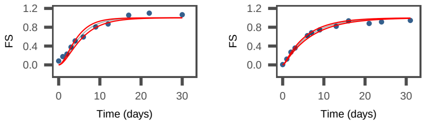

IPYR2 − ADCKEEHDIPR_3

AA R2: 0.96 k: 0.205 dk: 0.046

HW R2: 0.97 k: 0.166 dk: 0.0236

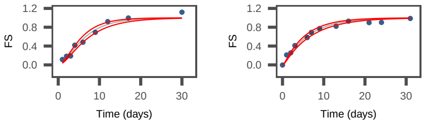

INMT − KEPGAYDWSSIVQHACELEGDR_3

AA R2: 0.95 k: 0.252 dk: 0.0699

HW R2: 0.96 k: 0.216 dk: 0.0487

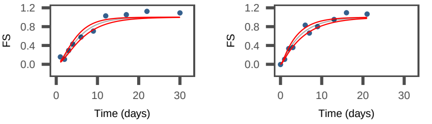

IPYR − GQYISPFHDVPIYADK_3

AA R2: 0.9 k: 0.612 dk: 0.33

HW R2: 0.92 k: 0.235 dk: 0.055

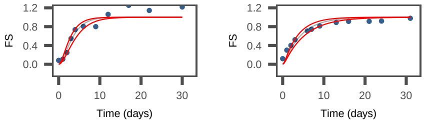

IQGA2 − LGIAPQIQDLLGK_2

AA R2: 0.97 k: 0.315 dk: 0.0741

HW R2: 0.97 k: 0.352 dk: 0.0556

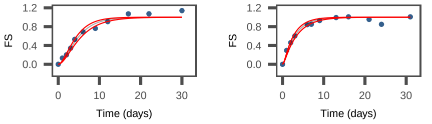

ISC2A − ILPESSILFLCDLQEK_2

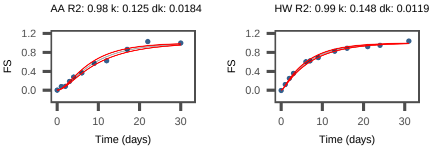

K1C18 − VKYETELAMR_3

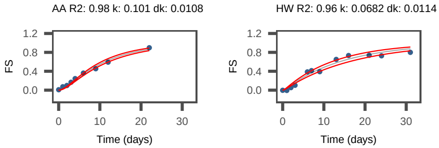

K2C8 − TKTEISEMNR_2

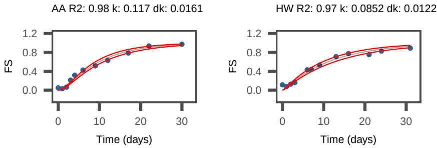

ISOC1 − GLGSTVQEIDLTGVK_2

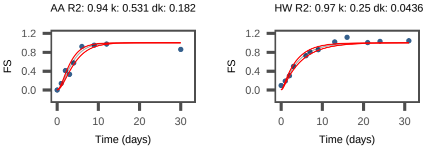

K2C8 − AEAETMYQIK_2

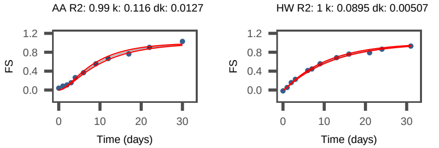

K2C8 − YEELQTLAGK_2

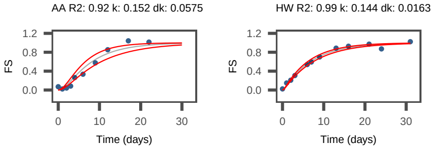

K1C18 − AQYEALAQK_2

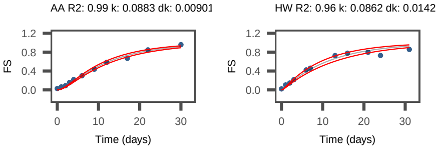

K2C8 − DVDEAYMNK_2

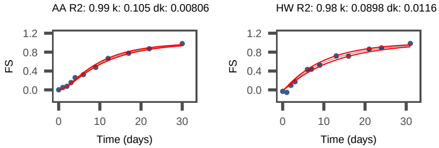

KAD2 − AVLLGPPGAGK_2

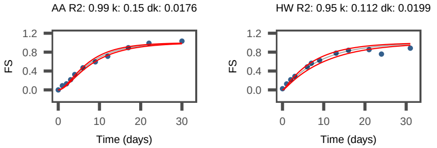

K1C18 − QAQEYEALLNIK_2

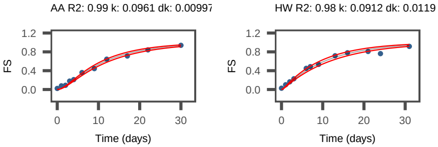

K2C8 − GSMGTGVGLGGFGGAGVGGITAVTVNQSLLSPLK_2

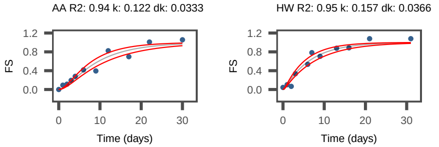

KAD2 − GIHCAIDASQTPDIVFASILAAFSK_3

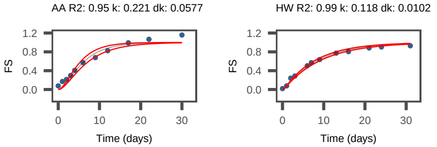

K1C18 − SVWGGSVGSAGLAGMGGIQTEK_3

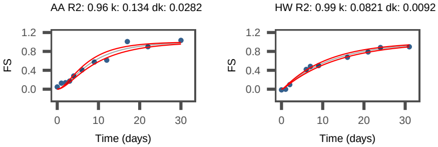

K2C8 − LEAELGNMQGLVEDFK_2

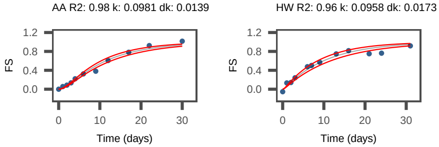

KAD3 − VYQIDTVINLNVPFEVIK_2

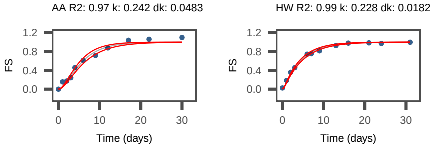

K1C18 − VKLEAEIATYR_2

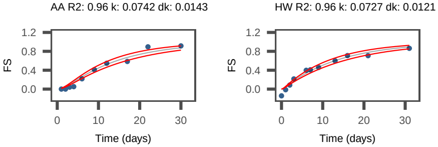

K2C8 − LVSESSDVVSK_2

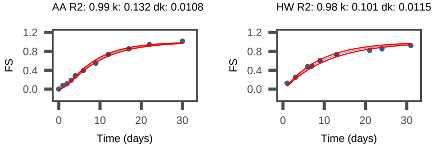

KAD3 − VYQIDTVINLNVPFEVIK_3

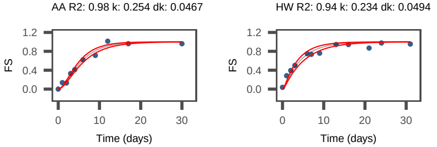

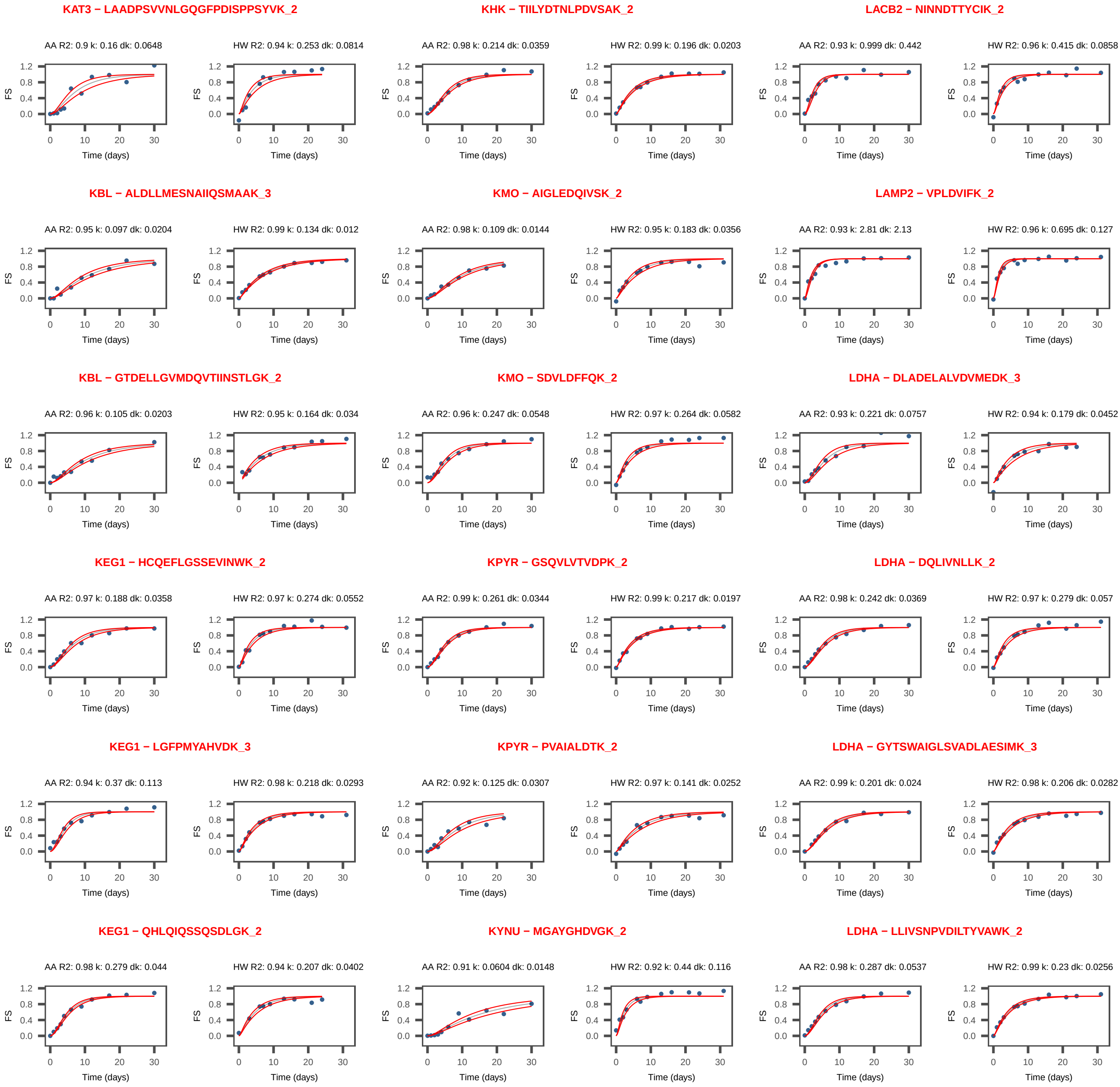
KAT3 − LAADPSVVNLGQGFPDISPPSYVK_2
AA R2: 0.9 k: 0.16 dk: 0.0648
HW R2: 0.94 k: 0.253 dk: 0.0814
KHK − TIILYDTNLPDVSAK_2
AA R2: 0.98 k: 0.214 dk: 0.0359
HW R2: 0.99 k: 0.196 dk: 0.0203
LACB2 − NINNDTTYCIK_2
AA R2: 0.93 k: 0.999 dk: 0.442
HW R2: 0.96 k: 0.415 dk: 0.0858
KBL − ALDLLMESNAIIQSMAAK_3
AA R2: 0.95 k: 0.097 dk: 0.0204
HW R2: 0.99 k: 0.134 dk: 0.012
KMO − AIGLEDQIVSK_2
AA R2: 0.98 k: 0.109 dk: 0.0144
HW R2: 0.95 k: 0.183 dk: 0.0356
LAMP2 − VPLDVIFK_2
AA R2: 0.93 k: 2.81 dk: 2.13
HW R2: 0.96 k: 0.695 dk: 0.127
KBL − GTDELLGVMDQVTIINSTLGK_2
AA R2: 0.96 k: 0.105 dk: 0.0203
HW R2: 0.95 k: 0.164 dk: 0.034
KMO − SDVLDFFQK_2
AA R2: 0.96 k: 0.247 dk: 0.0548
HW R2: 0.97 k: 0.264 dk: 0.0582
LDHA − DLADELALVDVMEDK_3
AA R2: 0.93 k: 0.221 dk: 0.0757
HW R2: 0.94 k: 0.179 dk: 0.0452
KEG1 − HCQEFLGSSEVINWK_2
AA R2: 0.97 k: 0.188 dk: 0.0358
HW R2: 0.97 k: 0.274 dk: 0.0552
KPYR − GSQVLVTVDPK_2
AA R2: 0.99 k: 0.261 dk: 0.0344
HW R2: 0.99 k: 0.217 dk: 0.0197
LDHA − DQLIVNLLK_2
AA R2: 0.98 k: 0.242 dk: 0.0369
HW R2: 0.97 k: 0.279 dk: 0.057
KEG1 − LGFPMYAHVDK_3
AA R2: 0.94 k: 0.37 dk: 0.113
HW R2: 0.98 k: 0.218 dk: 0.0293
KPYR − PVAIALDTK_2
AA R2: 0.92 k: 0.125 dk: 0.0307
HW R2: 0.97 k: 0.141 dk: 0.0252
LDHA − GYTSWAIGLSVADLAESIMK_3
AA R2: 0.99 k: 0.201 dk: 0.024
HW R2: 0.98 k: 0.206 dk: 0.0282
KEG1 − QHLQIQSSQSDLGK_2
AA R2: 0.98 k: 0.279 dk: 0.044
HW R2: 0.94 k: 0.207 dk: 0.0402
KYNU − MGAYGHDVGK_2
AA R2: 0.91 k: 0.0604 dk: 0.0148
HW R2: 0.92 k: 0.44 dk: 0.116
LDHA − LLIVSNPVDILTYVAWK_2
AA R2: 0.98 k: 0.287 dk: 0.0537
HW R2: 0.99 k: 0.23 dk: 0.0256
FS
Time (days)

LDHA − LLIVSNPVDILTYVAWK_3

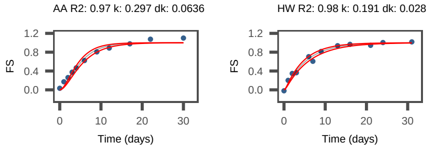

LGUL − GFGHIGIAVPDVYSACK_3

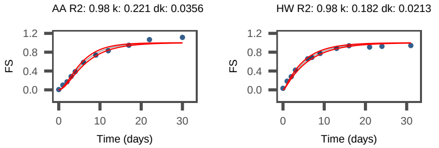

LONM − AQLSAAVLTLLIK_2

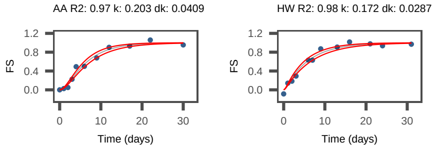

LDHA − QVVDSAYEVIK_2

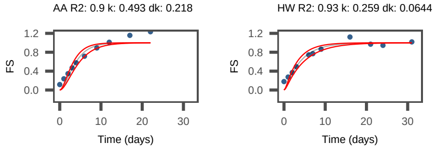

LGUL − GLAFIQDPDGYWIEILNPNK_2

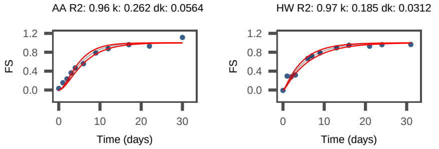

LPPRC − LIQALALK_2

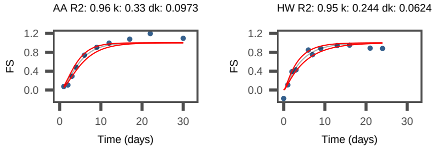

LDHA − VHPISTMIK_2

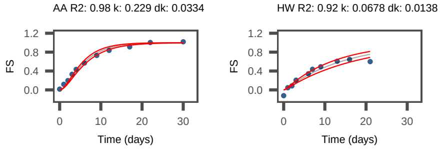

LGUL − GLAFIQDPDGYWIEILNPNK_3

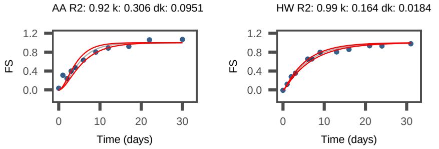

LRC59 − LVTLPVSFAQLK_2

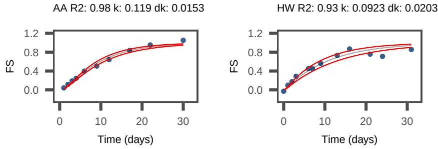

LEG1 − FNAHGDANTIVCNTK_3

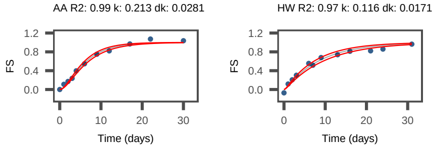

LGUL − VLGLTLLQK_2

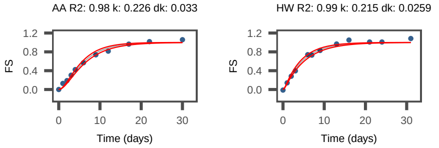

LYAG − QLLWGPALLITPVLEPGK_2

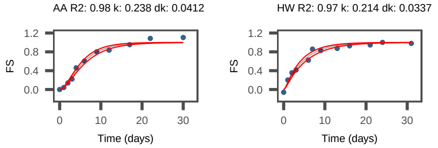

LEG9 − FEEGGYVVCNTK_2

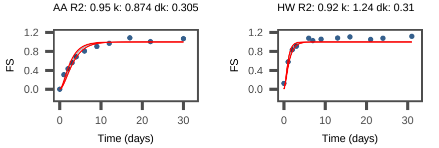

LHPP − AFQVLMELENPVLISLGK_2

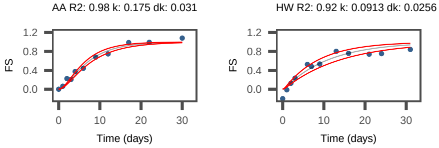

LYPA1 − ATAAVIFLHGLGDTGHGWAEAFAGIK_4

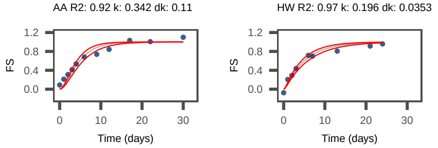

LGUL − GFGHIGIAVPDVYSACK_2

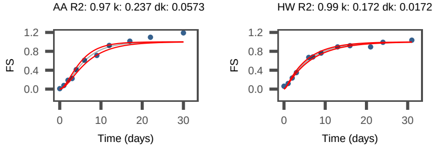

LICH − LYDEIISLMK_2

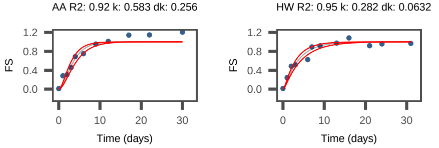

M2GD − AWGSEMNCDTNPLEAGLEYFVK_2

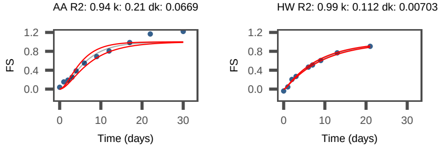

M2GD – AYVPVQLSEVGQQVEVELLGK_2

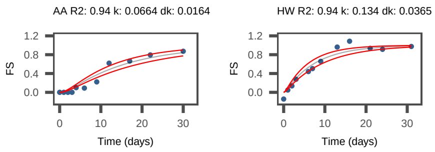

M2GD – TNWFEPVGSEYK_2

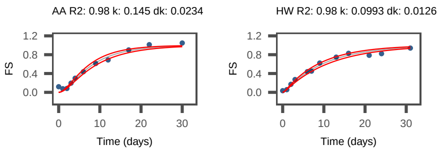

MAAI – LLPQDPQKR_2

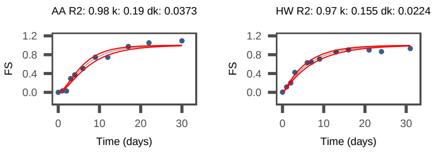

M2GD – DGLLFGPYESQEK_2

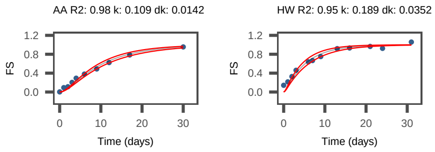

M2GD – VGVIDLSPFGK_2

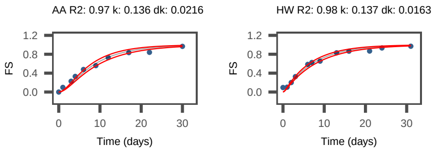

MAAI – MISDLIASGIQPLQNLSVLK_2

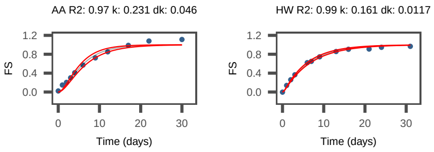

M2GD – DSTQLLDHLFANVIPK_2

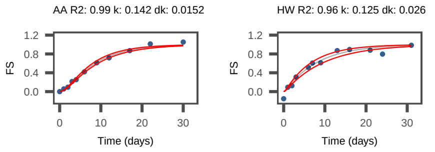

M2GD – YPAPVTSLKPR_3

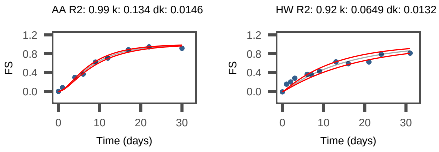

MAAI – MISDLIASGIQPLQNLSVLK_3

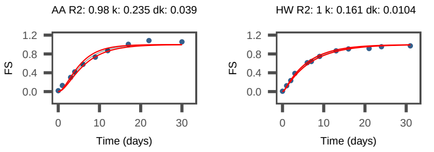

M2GD – IHELFPLLNMNK_3

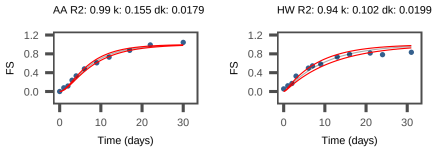

MAAI – DGGQQFTEEFQTLNPMK_2

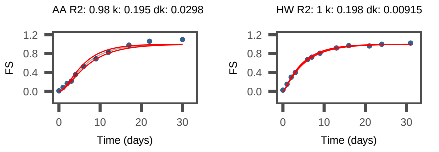

MAAI – QVGQENQMQWAQK_2

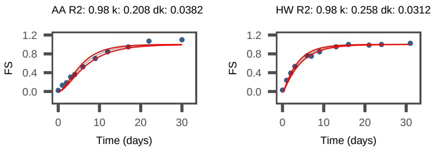

M2GD – LQASWVTHGVPPGFGK_3

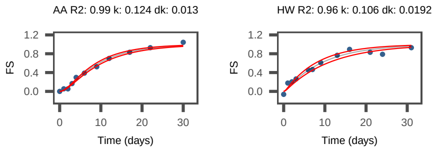

MAAI – GIDYEIVPINLIK_2

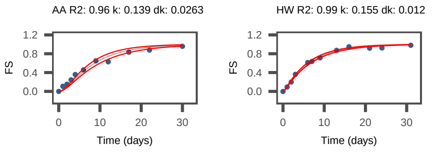

MAAI – VDLSPYPTISHINK_2

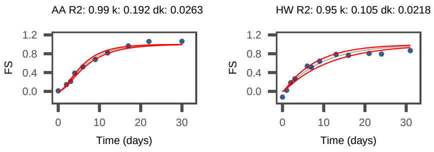

M2GD – SLAFAYVPVQLSEVGQQVEVELLGK_3

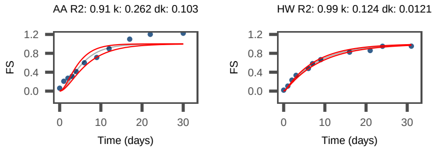

MAAI – LLPQDPQK_2

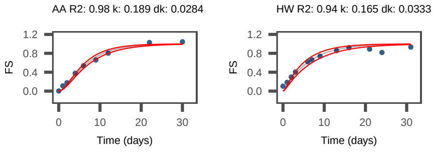

MAAI – VDLSPYPTISHINK_3

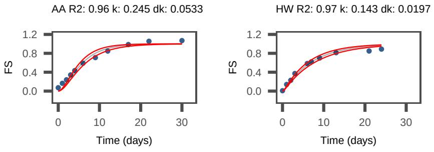

MAAI − VITSGFNALEK_2

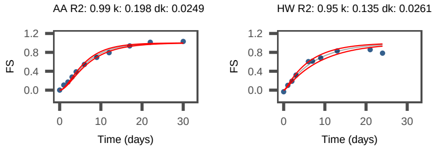

MAOX − MATVYPEPQNK_2

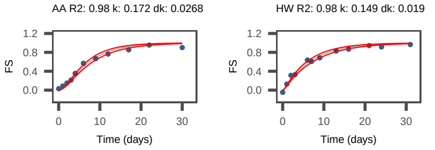

MARC2 − DCGDEVAQWFTNYLK_2

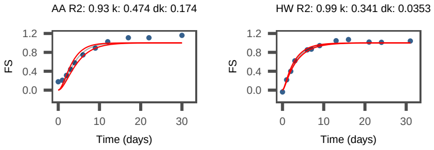

MAOX − EVFAHEHEEMK_3

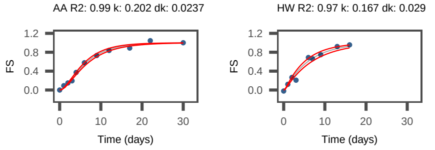

MAOX(Non−Unique) − NIEALVQK_2

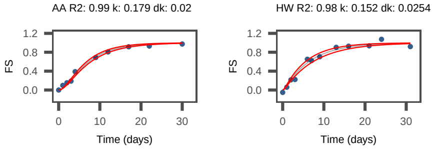

MARC2 − DCGDEVAQWFTNYLK_3

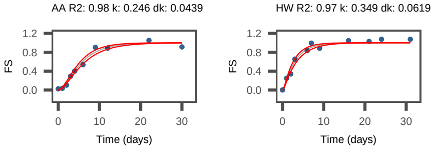

MAOX − GHIASVLNAWPEDVVK_2

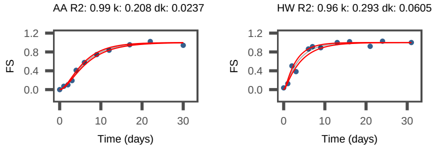

MAOX − VFLTTAEVISQQVSDK_2

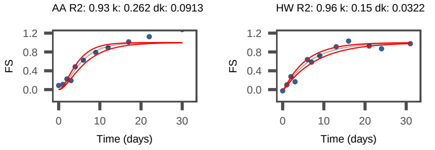

MARC2 − QLQQVGTVSK_2

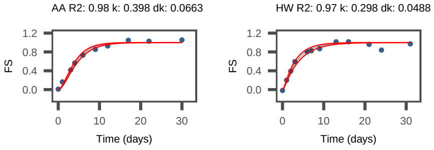

MAOX − GPEYDAFLDEFMEAASSK_3

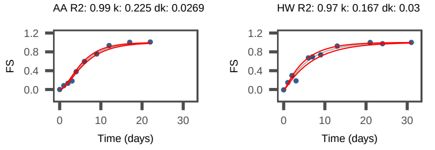

MARC1 − DCGEDAAQWVSSFLK_2

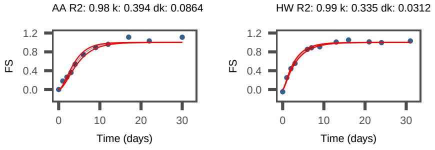

MATR3 − YQLLQLVEPFGVISNHLILNK_3

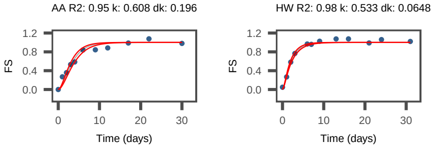

MAOX − IGVAAIGGAFTEQILK_2

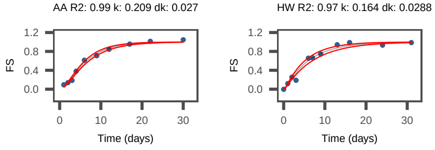

MARC1 − LCDPSEQALYGK_2

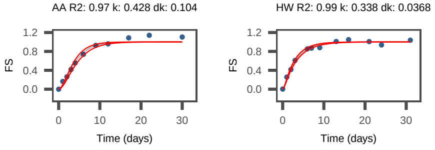

MCAT − DVPASGMYFMTYEWLK_2

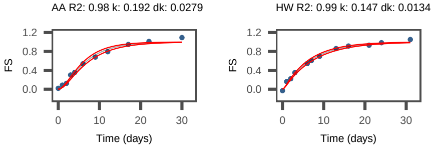

MAOX − IWLVDSK_2

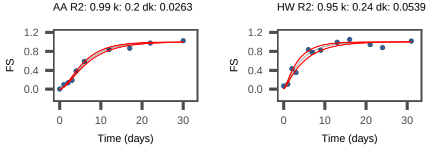

MARC1 − LQQVGTVAQLWIYPIK_2

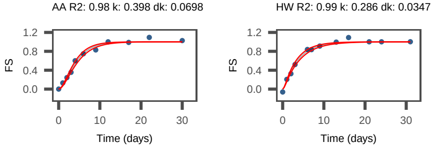

MCCB − QGTIFLAGPPLVK_2

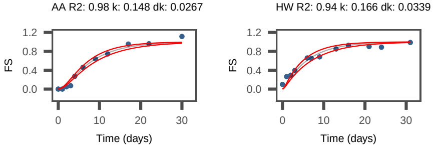

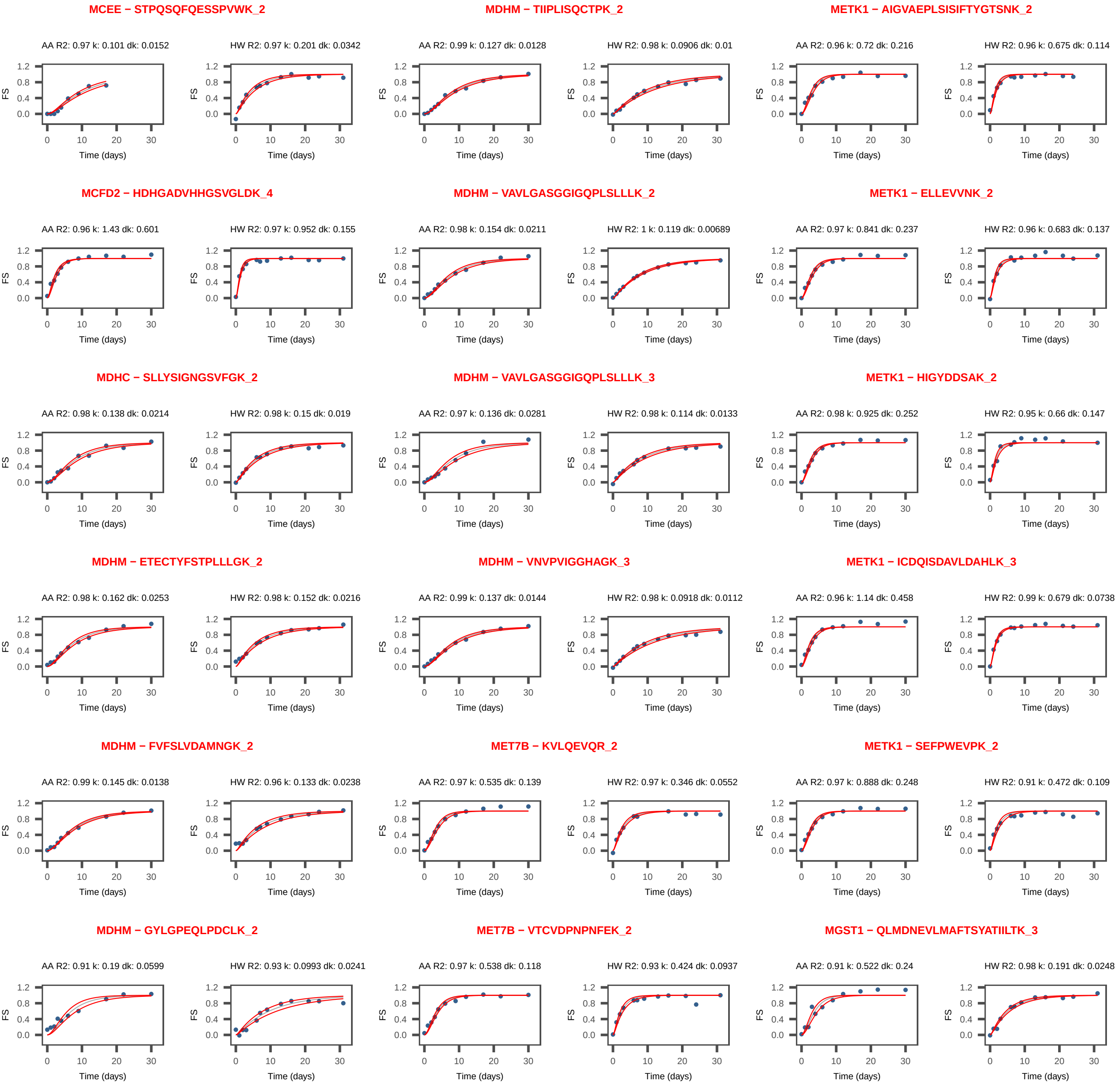
MCEE – STPQSQFQESSPVWK_2
AA R2: 0.97 k: 0.101 dk: 0.0152
HW R2: 0.97 k: 0.201 dk: 0.0342
MDHM – TIIPLISQCTPK_2
AA R2: 0.99 k: 0.127 dk: 0.0128
HW R2: 0.98 k: 0.0906 dk: 0.01
METK1 – AIGVAEPLSISIFTYGTSNK_2
AA R2: 0.96 k: 0.72 dk: 0.216
HW R2: 0.96 k: 0.675 dk: 0.114
MCFD2 – HDHGADVHHGSVGLDK_4
AA R2: 0.96 k: 1.43 dk: 0.601
HW R2: 0.97 k: 0.952 dk: 0.155
MDHM – VAVLGASGGIGQPLSLLLK_2
AA R2: 0.98 k: 0.154 dk: 0.0211
HW R2: 1 k: 0.119 dk: 0.00689
METK1 – ELLEVVNK_2
AA R2: 0.97 k: 0.841 dk: 0.237
HW R2: 0.96 k: 0.683 dk: 0.137
MDHC – SLLYSIGNGSVFGK_2
AA R2: 0.98 k: 0.138 dk: 0.0214
HW R2: 0.98 k: 0.15 dk: 0.019
MDHM – VAVLGASGGIGQPLSLLLK_3
AA R2: 0.97 k: 0.136 dk: 0.0281
HW R2: 0.98 k: 0.114 dk: 0.0133
METK1 – HIGYDDSAK_2
AA R2: 0.98 k: 0.925 dk: 0.252
HW R2: 0.95 k: 0.66 dk: 0.147
MDHM – ETECTYFSTPLLLGK_2
AA R2: 0.98 k: 0.162 dk: 0.0253
HW R2: 0.98 k: 0.152 dk: 0.0216
MDHM – VNVPVIGGHAGK_3
AA R2: 0.99 k: 0.137 dk: 0.0144
HW R2: 0.98 k: 0.0918 dk: 0.0112
METK1 – ICDQISDAVLDAHLK_3
AA R2: 0.96 k: 1.14 dk: 0.458
HW R2: 0.99 k: 0.679 dk: 0.0738
MDHM – FVFSLVDAMNGK_2
AA R2: 0.99 k: 0.145 dk: 0.0138
HW R2: 0.96 k: 0.133 dk: 0.0238
MET7B – KVLQEVQR_2
AA R2: 0.97 k: 0.535 dk: 0.139
HW R2: 0.97 k: 0.346 dk: 0.0552
METK1 – SEFPWEVPK_2
AA R2: 0.97 k: 0.888 dk: 0.248
HW R2: 0.91 k: 0.472 dk: 0.109
MDHM – GYLGPEQLPDCLK_2
AA R2: 0.91 k: 0.19 dk: 0.0599
HW R2: 0.93 k: 0.0993 dk: 0.0241
MET7B – VTCVDPNPNFEK_2
AA R2: 0.97 k: 0.538 dk: 0.118
HW R2: 0.93 k: 0.424 dk: 0.0937
MGST1 – QLMDNEVLMAFTSYATIILTK_3
AA R2: 0.91 k: 0.522 dk: 0.24
HW R2: 0.98 k: 0.191 dk: 0.0248
FS
Time (days)

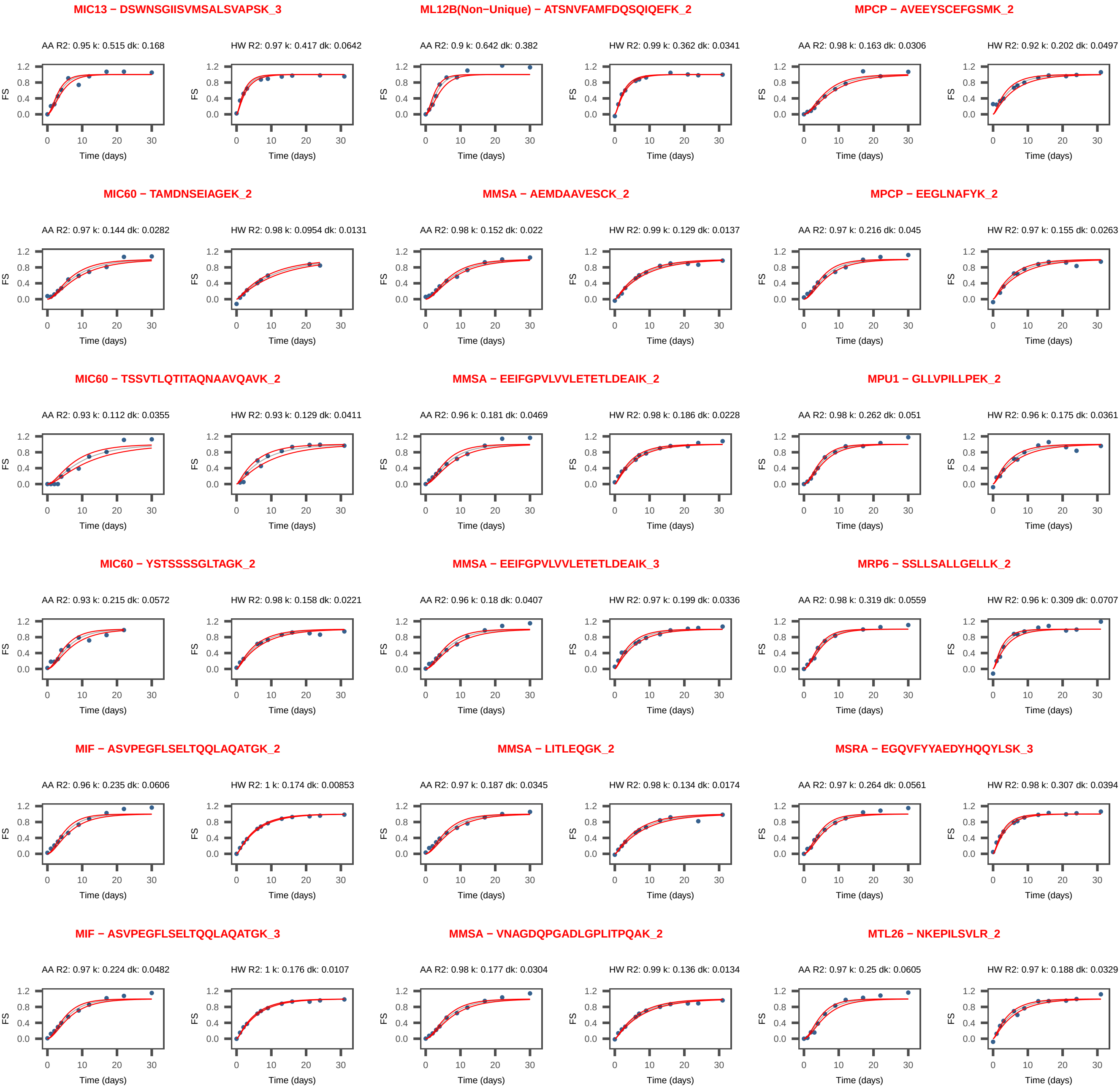
MIC13 − DSWNSGIISVMSALSVAPSK_3
AA R2: 0.95 k: 0.515 dk: 0.168
HW R2: 0.97 k: 0.417 dk: 0.0642
ML12B(Non−Unique) − ATSNVFAMFDQSQIQEFK_2
AA R2: 0.9 k: 0.642 dk: 0.382
HW R2: 0.99 k: 0.362 dk: 0.0341
MPCP − AVEEYSCEFGSMK_2
AA R2: 0.98 k: 0.163 dk: 0.0306
HW R2: 0.92 k: 0.202 dk: 0.0497
MIC60 − TAMDNSEIAGEK_2
AA R2: 0.97 k: 0.144 dk: 0.0282
HW R2: 0.98 k: 0.0954 dk: 0.0131
MMSA − AEMDAAVESCK_2
AA R2: 0.98 k: 0.152 dk: 0.022
HW R2: 0.99 k: 0.129 dk: 0.0137
MPCP − EEGLNAFYK_2
AA R2: 0.97 k: 0.216 dk: 0.045
HW R2: 0.97 k: 0.155 dk: 0.0263
MIC60 − TSSVTLQTITAQNAAVQAVK_2
AA R2: 0.93 k: 0.112 dk: 0.0355
HW R2: 0.93 k: 0.129 dk: 0.0411
MMSA − EEIFGPVLVVLETETLDEAIK_2
AA R2: 0.96 k: 0.181 dk: 0.0469
HW R2: 0.98 k: 0.186 dk: 0.0228
MPU1 − GLLVPILLPEK_2
AA R2: 0.98 k: 0.262 dk: 0.051
HW R2: 0.96 k: 0.175 dk: 0.0361
MIC60 − YSTSSSSGLTAGK_2
AA R2: 0.93 k: 0.215 dk: 0.0572
HW R2: 0.98 k: 0.158 dk: 0.0221
MMSA − EEIFGPVLVVLETETLDEAIK_3
AA R2: 0.96 k: 0.18 dk: 0.0407
HW R2: 0.97 k: 0.199 dk: 0.0336
MRP6 − SSLLSALLGELLK_2
AA R2: 0.98 k: 0.319 dk: 0.0559
HW R2: 0.96 k: 0.309 dk: 0.0707
MIF − ASVPEGFLSELTQQLAQATGK_2
AA R2: 0.96 k: 0.235 dk: 0.0606
HW R2: 1 k: 0.174 dk: 0.00853
MMSA − LITLEQGK_2
AA R2: 0.97 k: 0.187 dk: 0.0345
HW R2: 0.98 k: 0.134 dk: 0.0174
MSRA − EGQVFYYAEDYHQQYLSK_3
AA R2: 0.97 k: 0.264 dk: 0.0561
HW R2: 0.98 k: 0.307 dk: 0.0394
MIF − ASVPEGFLSELTQQLAQATGK_3
AA R2: 0.97 k: 0.224 dk: 0.0482
HW R2: 1 k: 0.176 dk: 0.0107
MMSA − VNAGDQPGADLGPLITPQAK_2
AA R2: 0.98 k: 0.177 dk: 0.0304
HW R2: 0.99 k: 0.136 dk: 0.0134
MTL26 − NKEPILSVLR_2
AA R2: 0.97 k: 0.25 dk: 0.0605
HW R2: 0.97 k: 0.188 dk: 0.0329
FS
Time (days)

MTP − MLSASGDPVSVVK_2

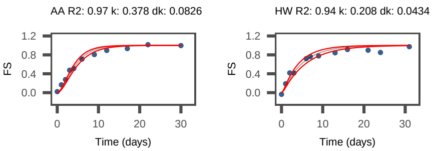

MYDGF − SYLYFTQFK_2

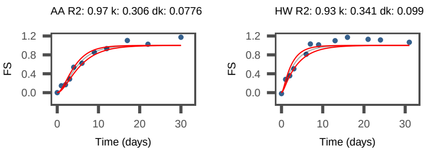

NCPR − TYEHFNAMGK_3

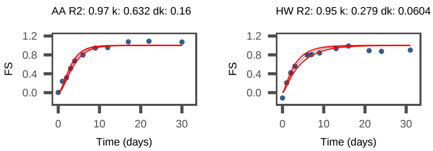

MTP − NALLPEGIPLLLK_2

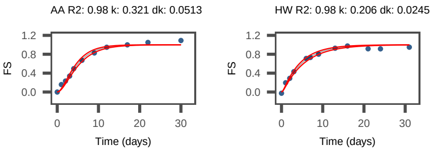

MYH11(Non−Unique) − EDQSILCTGESGAGK_2

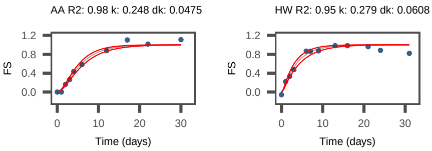

NDKA − FLQASEDLLK_2

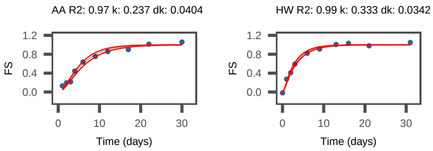

MTP − NILLSIGELPK_2

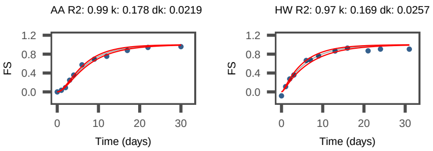

MYH11(Non−Unique) − NTDQASMPDNTAAQK_2

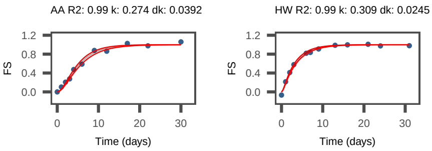

NDKA(Non−Unique) − TFIAIKPDGVQR_2

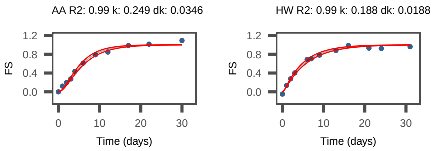

MTP − YMLTVVQDILHFEMPASK_3

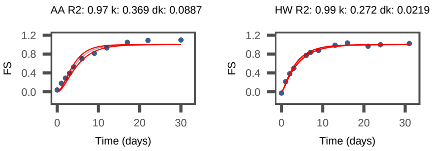

MYL6 − VLDFEHFLPMLQTVAK_3

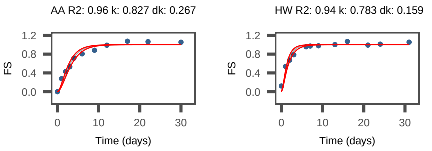

NDKA(Non−Unique) − TFIAIKPDGVQR_3

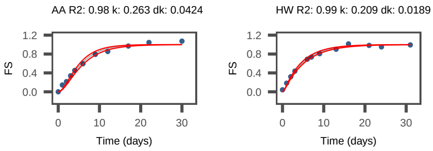

MTX2 − VPFIHVGNQVVSELGPIVQFVK_3

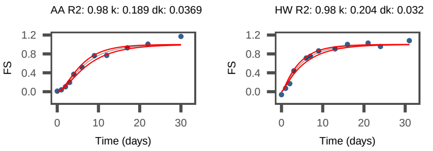

NACAM(Non−Unique) − IEDLSQQAQLAAAEK_2

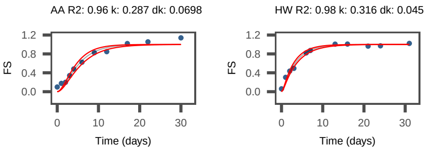

NDKA(Non−Unique) − VMLGETNPADSKPGTIR_3

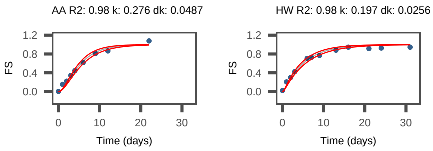

MVP − QAIPLDQNEGIYVQDVK_2

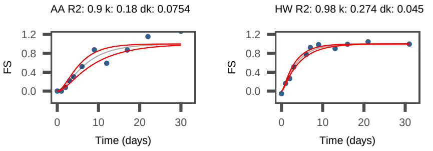

NCPR − FAVFGLGNK_2

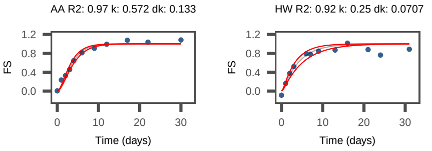

NDKA − YMHSGPVVAMVWEGLNVVK_3

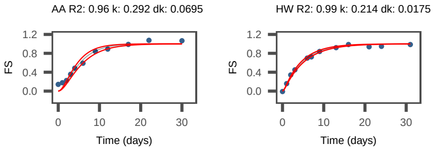

NDKB – DRPFFPGLVK_2

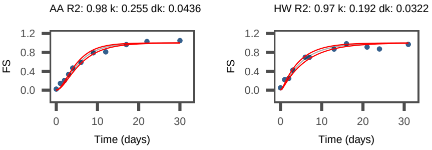

NDUA4 – LGPNEQYK_2

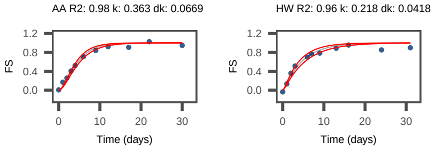

NDUS2 – IRPGGVHQDLPLGLLDDIYEFSK_4

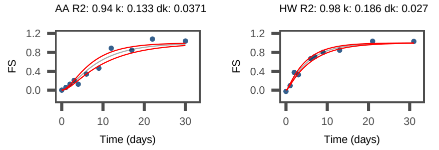

NDKB – DRPFFPGLVK_3

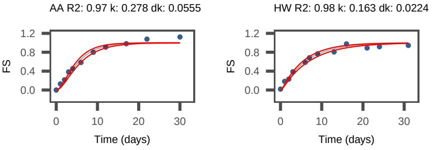

NDUAA – YGLLAAILGDK_2

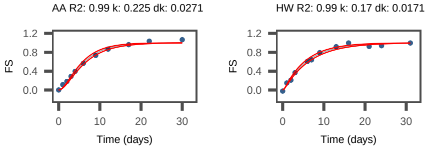

NDUS2 – TSMESLIHHFK_3

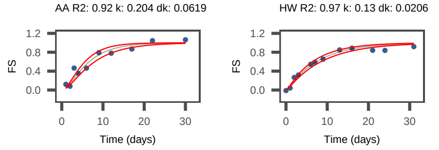

NDRG2 – GWMDWAAHK_3

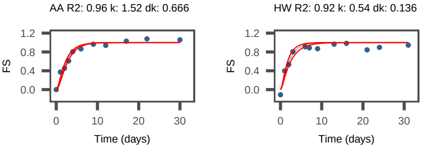

NDUAD – IALMPLFQAEK_2

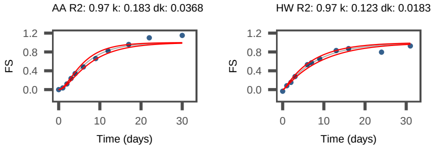

NDUS6 – IIACDGGGGALGHPK_2

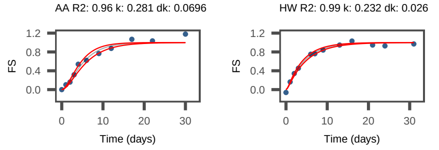

NDRG2 – ILLDQGQTHSVETPYGSVTFTVYGTPK_3

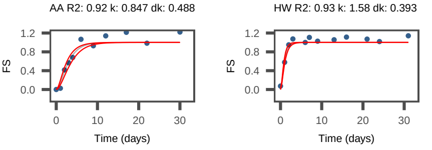

NDUB3 – IEGTPLETVQK_2

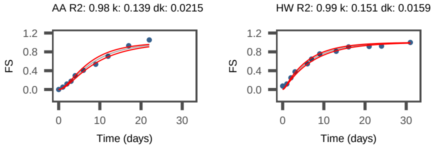

NDUS6 – ITHTGQVYDEK_2

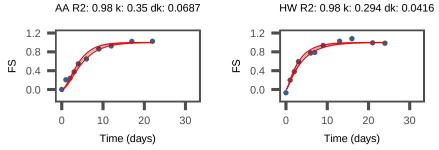

NDRG2 – MADSGGQPQLTQPGK_2

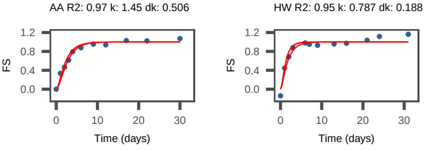

NDUS1 – FAYDGLK_2

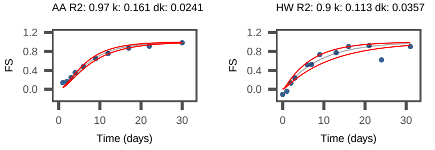

NDUS6 – ITHTGQVYDEK_3

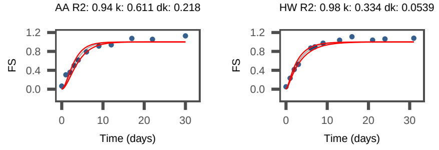

NDUA4 – FYSVNVDYSK_2

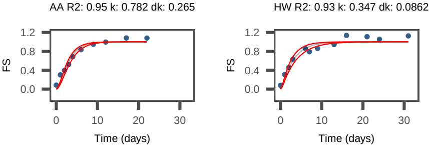

NDUS1 – MFMSELSGNVIDICPVGALTSK_2

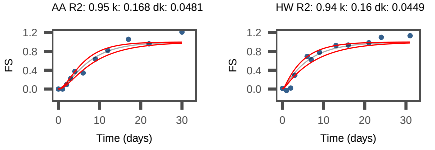

NDUV1 – GPDWILGEMK_2

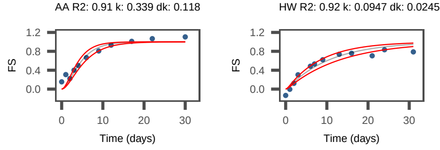

NDUV1 − HAGGVTGGWDNLLAVIPGGSSTPLIPK_3

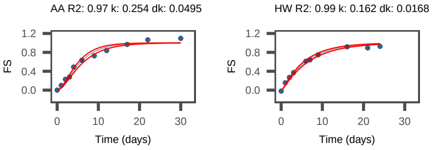

NIT2 − ASYVAWGHSTVVDPWGQVLTK_2

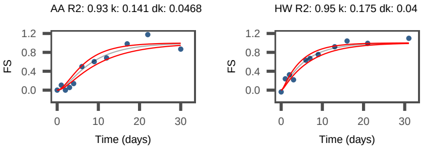

NNRE − GNPSGIQPDLLISLTAPK_2

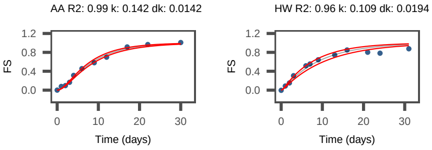

NDUV1 − YLVVNADEGEPGTCK_2

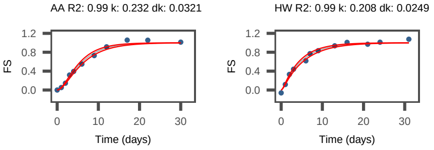

NIT2 − ASYVAWGHSTVVDPWGQVLTK_3

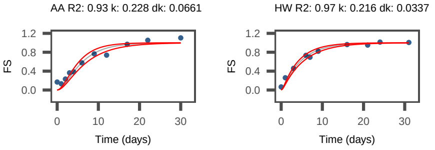

NNTM − ILIVGGGVAGLASAGAAK_2

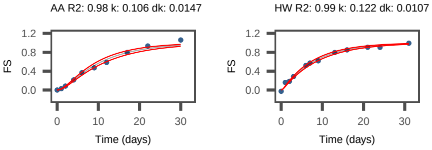

NDUV2 − DIEEIIDELK_2

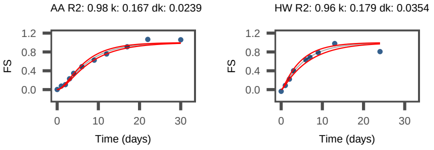

NIT2 − IHLFDIDVPGK_3

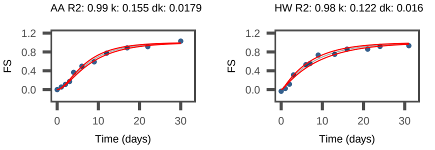

NPM − VTLATLK_2

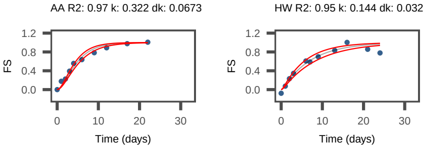

NDUV2 − FCCEPAGGLTSLTEPPK_2

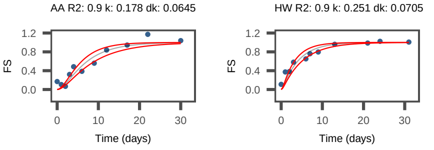

NIT2 − LYNTCSVFGPDGSLLVK_2

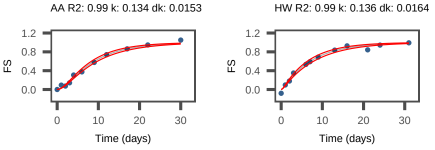

NQO2 − GFAFDIPGFYDSGFLK_2

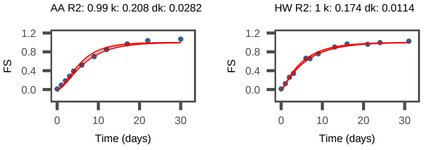

NIPS1 − FSGGYPALMDCMNK_2

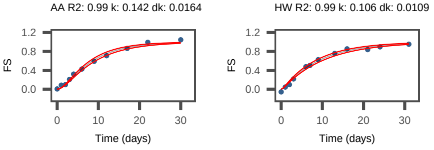

NIT2 − TLSPGDSFSTFDTPYCK_2

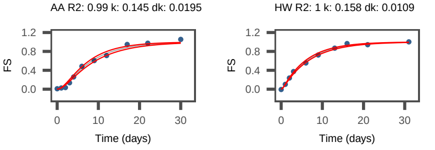

NQO2_HUMAN,sp|Q9JI75|NQO2(Non−Unique) − VLIVYAHQEPK_2

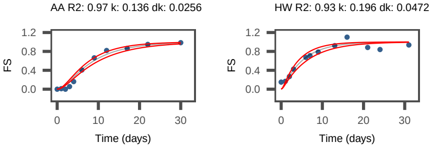

NIPS1 − LKPGTMIEWGNNWAR_2

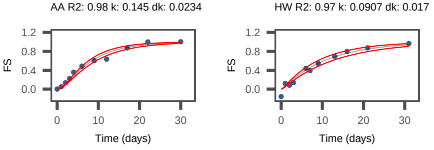

NNRD − LSQALGNITVVQK_2

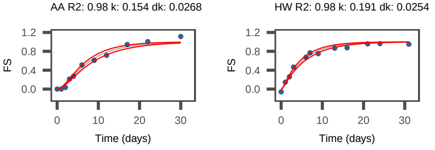

NQO2_HUMAN,sp|Q9JI75|NQO2(Non−Unique) − VLIVYAHQEPK_3

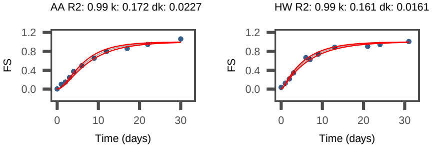

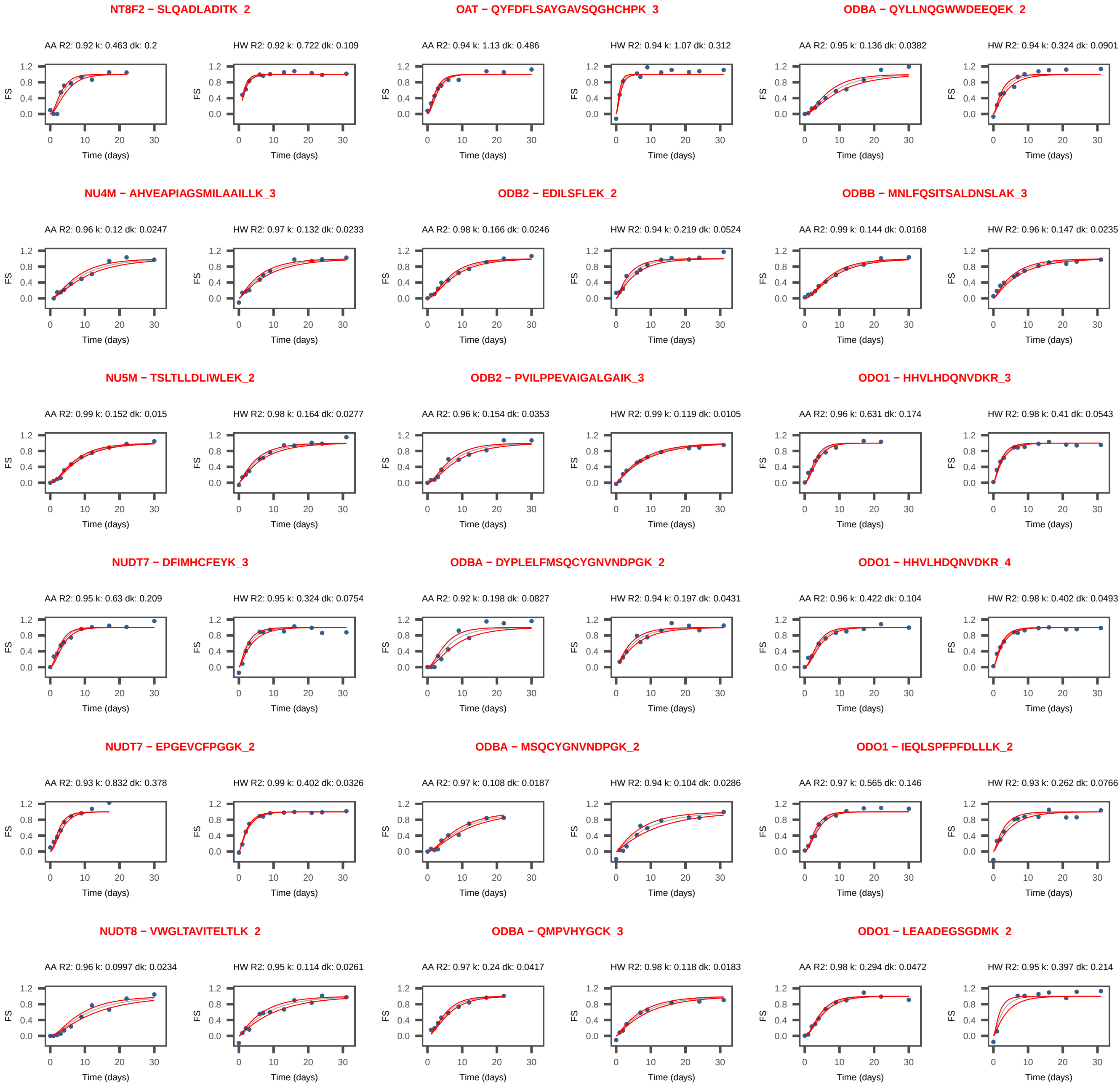
NT8F2 – SLQADLADITK_2
AA R2: 0.92 k: 0.463 dk: 0.2
HW R2: 0.92 k: 0.722 dk: 0.109
OAT – QYFDFLSAYGAVSQGHCHPK_3
AA R2: 0.94 k: 1.13 dk: 0.486
HW R2: 0.94 k: 1.07 dk: 0.312
ODBA – QYLLNQGWWDEEQEK_2
AA R2: 0.95 k: 0.136 dk: 0.0382
HW R2: 0.94 k: 0.324 dk: 0.0901
NU4M – AHVEAPIAGSMILAAILLK_3
AA R2: 0.96 k: 0.12 dk: 0.0247
HW R2: 0.97 k: 0.132 dk: 0.0233
ODB2 – EDILSFLEK_2
AA R2: 0.98 k: 0.166 dk: 0.0246
HW R2: 0.94 k: 0.219 dk: 0.0524
ODBB – MNLFQSITSALDNSLAK_3
AA R2: 0.99 k: 0.144 dk: 0.0168
HW R2: 0.96 k: 0.147 dk: 0.0235
NU5M – TSLTLLDLIWLEK_2
AA R2: 0.99 k: 0.152 dk: 0.015
HW R2: 0.98 k: 0.164 dk: 0.0277
ODB2 – PVILPPEVAIGALGAIK_3
AA R2: 0.96 k: 0.154 dk: 0.0353
HW R2: 0.99 k: 0.119 dk: 0.0105
ODO1 – HHVLHDQNVDKR_3
AA R2: 0.96 k: 0.631 dk: 0.174
HW R2: 0.98 k: 0.41 dk: 0.0543
NUDT7 – DFIMHCFEYK_3
AA R2: 0.95 k: 0.63 dk: 0.209
HW R2: 0.95 k: 0.324 dk: 0.0754
ODBA – DYPLELFMSQCYGNVNDPGK_2
AA R2: 0.92 k: 0.198 dk: 0.0827
HW R2: 0.94 k: 0.197 dk: 0.0431
ODO1 – HHVLHDQNVDKR_4
AA R2: 0.96 k: 0.422 dk: 0.104
HW R2: 0.98 k: 0.402 dk: 0.0493
NUDT7 – EPGEVCFPGGK_2
AA R2: 0.93 k: 0.832 dk: 0.378
HW R2: 0.99 k: 0.402 dk: 0.0326
ODBA – MSQCYGNVNDPGK_2
AA R2: 0.97 k: 0.108 dk: 0.0187
HW R2: 0.94 k: 0.104 dk: 0.0286
ODO1 – IEQLSPFPFDLLLK_2
AA R2: 0.97 k: 0.565 dk: 0.146
HW R2: 0.93 k: 0.262 dk: 0.0766
NUDT8 – VWGLTAVITELTLK_2
AA R2: 0.96 k: 0.0997 dk: 0.0234
HW R2: 0.95 k: 0.114 dk: 0.0261
ODBA – QMPVHYGCK_3
AA R2: 0.97 k: 0.24 dk: 0.0417
HW R2: 0.98 k: 0.118 dk: 0.0183
ODO1 – LEAADEGSGDMK_2
AA R2: 0.98 k: 0.294 dk: 0.0472
HW R2: 0.95 k: 0.397 dk: 0.214
FS
Time (days)

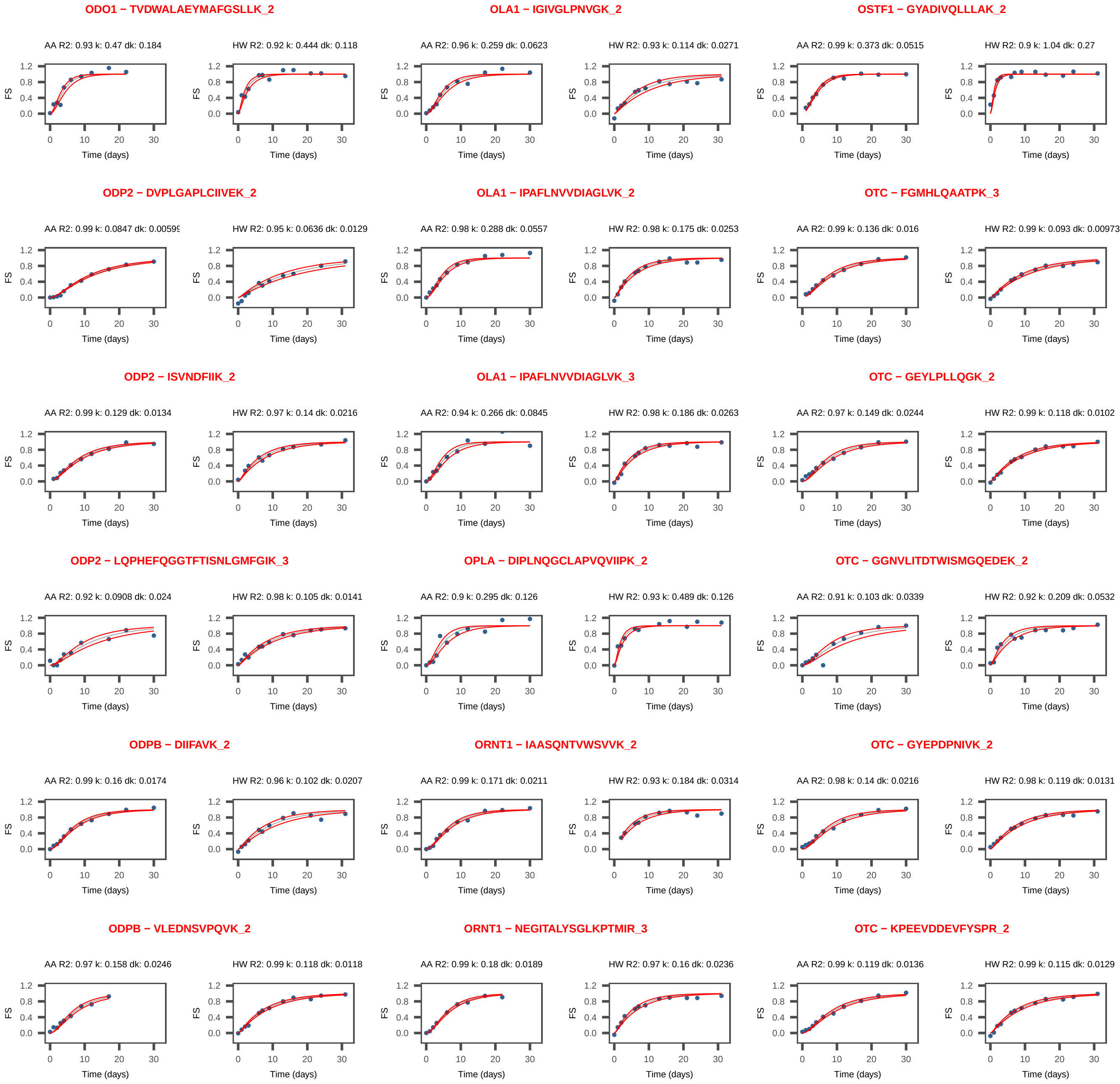
ODO1 – TVDWALAEYMAFGSLLK_2
AA R2: 0.93 k: 0.47 dk: 0.184
HW R2: 0.92 k: 0.444 dk: 0.118
OLA1 – IGIVGLPNVGK_2
AA R2: 0.96 k: 0.259 dk: 0.0623
HW R2: 0.93 k: 0.114 dk: 0.0271
OSTF1 – GYADIVQLLLAK_2
AA R2: 0.99 k: 0.373 dk: 0.0515
HW R2: 0.9 k: 1.04 dk: 0.27
ODP2 – DVPLGAPLCIIVEK_2
AA R2: 0.99 k: 0.0847 dk: 0.0059
HW R2: 0.95 k: 0.0636 dk: 0.0129
OLA1 – IPAFLNVVDIAGLVK_2
AA R2: 0.98 k: 0.288 dk: 0.0557
HW R2: 0.98 k: 0.175 dk: 0.0253
OTC – FGMHLQAATPK_3
AA R2: 0.99 k: 0.136 dk: 0.016
HW R2: 0.99 k: 0.093 dk: 0.00973
ODP2 – ISVNDFIIK_2
AA R2: 0.99 k: 0.129 dk: 0.0134
HW R2: 0.97 k: 0.14 dk: 0.0216
OLA1 – IPAFLNVVDIAGLVK_3
AA R2: 0.94 k: 0.266 dk: 0.0845
HW R2: 0.98 k: 0.186 dk: 0.0263
OTC – GEYLPLLQGK_2
AA R2: 0.97 k: 0.149 dk: 0.0244
HW R2: 0.99 k: 0.118 dk: 0.0102
ODP2 – LQPHEFQGGTFTISNLGMFGIK_3
AA R2: 0.92 k: 0.0908 dk: 0.024
HW R2: 0.98 k: 0.105 dk: 0.0141
OPLA – DIPLNQGCLAPVQVIIPK_2
AA R2: 0.9 k: 0.295 dk: 0.126
HW R2: 0.93 k: 0.489 dk: 0.126
OTC – GGNVLITDTWISMGQEDEK_2
AA R2: 0.91 k: 0.103 dk: 0.0339
HW R2: 0.92 k: 0.209 dk: 0.0532
ODPB – DIIFAVK_2
AA R2: 0.99 k: 0.16 dk: 0.0174
HW R2: 0.96 k: 0.102 dk: 0.0207
ORNT1 – IAASQNTVWSVVK_2
AA R2: 0.99 k: 0.171 dk: 0.0211
HW R2: 0.93 k: 0.184 dk: 0.0314
OTC – GYEPDPNIVK_2
AA R2: 0.98 k: 0.14 dk: 0.0216
HW R2: 0.98 k: 0.119 dk: 0.0131
ODPB – VLEDNSVPQVK_2
AA R2: 0.97 k: 0.158 dk: 0.0246
HW R2: 0.99 k: 0.118 dk: 0.0118
ORNT1 – NEGITALYSGLKPTMIR_3
AA R2: 0.99 k: 0.18 dk: 0.0189
HW R2: 0.97 k: 0.16 dk: 0.0236
OTC – KPEEVDDEVFYSPR_2
AA R2: 0.99 k: 0.119 dk: 0.0136
HW R2: 0.99 k: 0.115 dk: 0.0129
FS
Time (days)

OTC − KPEEVDDEVFYSPR_3

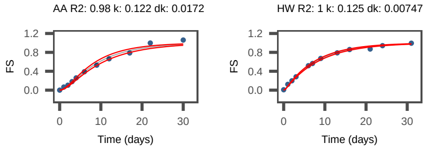

OTC − WIGDGNNILHSIMMSAAK_3

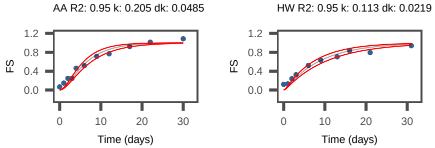

OTUB1 − AFGFSHLEALLDDSK_3

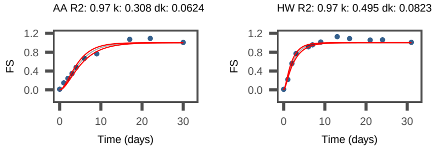

PA2G4 − SEMEVQDAELK_2

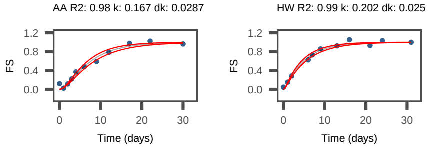

PA2G4 − TIIQNPTDQQK_2

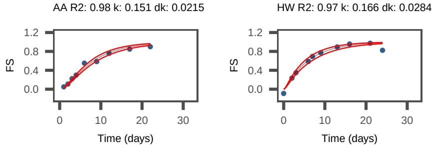

PABP1 − LFPLIQAMHPSLAGK_3

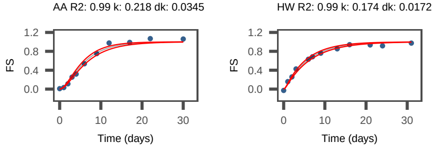

PAHX − YCLLPEILK_2

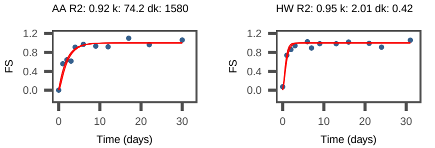

PAPS2 − LDLQWVQILSEGWATPLK_3

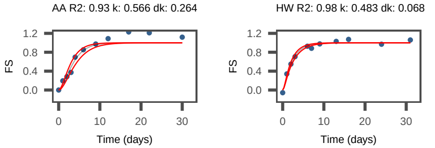

PARK7 − ALAIVEALVGK_2

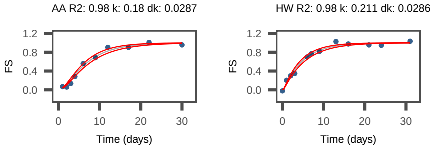

PARK7 − ALVILAK_2

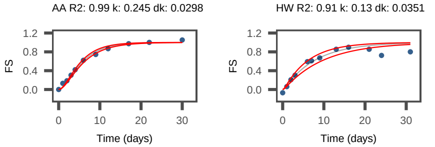

PARK7 − GLIAAICAGPTALLAHEVGFGCK_3

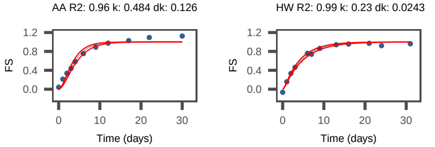

PARK7 − GPGTSFEFALAIVEALVGK_2

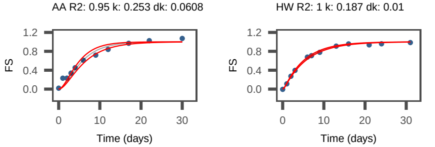

PARK7 − TQGPYDVVVLPGGNLGAQNLSESPMVK_3

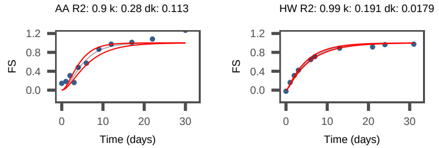

PARK7 − VTVAGLAGK_2

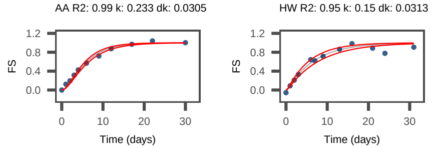

PBLD2(Non−Unique) − GLILTVK_2

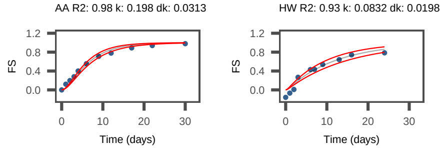

PBLD2(Non−Unique) − VNTEPLPAIEK_2

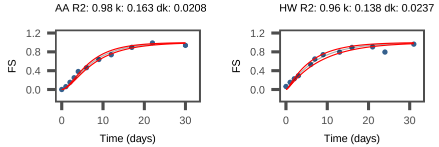

PCBP1 − LVVPATQCGSLIGK_2

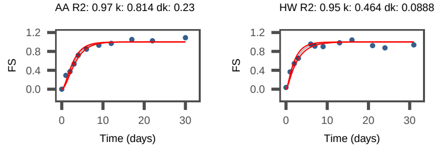

PCCB − LVYVFSQDFTVFGGSLSGAHAQK_3

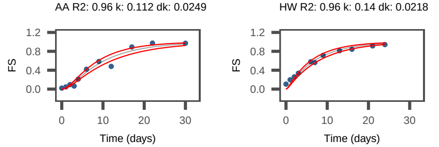

PCKGC – EIISFGSGYGGNSLLGK_2

AA R2: 0.91 k: 0.828 dk: 0.407 | HW R2: 0.94 k: 2.03 dk: 0.441

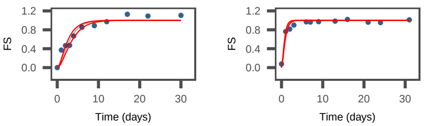

PDIA1 – MDSTANEVEAVK_2

AA R2: 0.97 k: 0.367 dk: 0.0892 | HW R2: 0.99 k: 0.292 dk: 0.0224

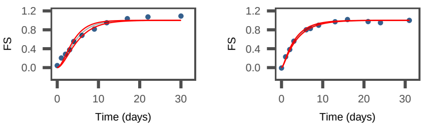

PDIA3 – GFPTIYFSPANK_2

AA R2: 0.95 k: 0.512 dk: 0.151 | HW R2: 0.92 k: 0.165 dk: 0.0382

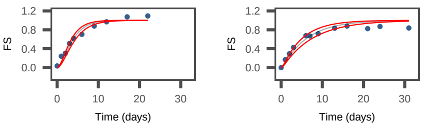

PCKGC – GLGGVNVEELFGISK_2

AA R2: 0.92 k: 14.8 dk: 60.7 | HW R2: 0.94 k: 2.18 dk: 0.504

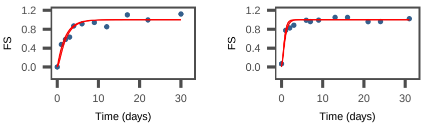

PDIA1 – NNFEGEITK_2

AA R2: 0.95 k: 0.415 dk: 0.116 | HW R2: 0.99 k: 0.323 dk: 0.0318

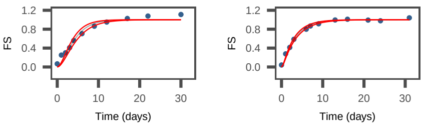

PDIA3 – MDATANDVPSPYEVK_2

AA R2: 0.98 k: 0.368 dk: 0.0748 | HW R2: 0.98 k: 0.33 dk: 0.0435

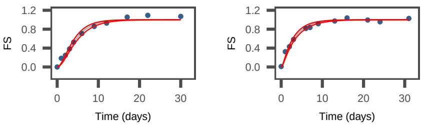

PCY2 – YVSEVVIGAPYSVTAELLNHFK_3

AA R2: 0.96 k: 0.565 dk: 0.184 | HW R2: 0.95 k: 0.428 dk: 0.0793

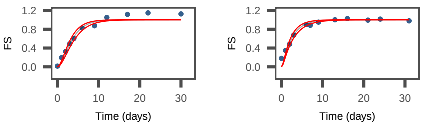

PDIA1 – SNFEEALAAHK_3

AA R2: 0.96 k: 0.41 dk: 0.113 | HW R2: 0.96 k: 0.358 dk: 0.0746

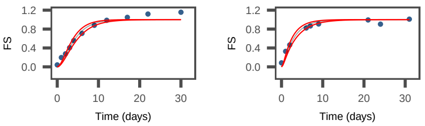

PDIA3 – SEPIPESNEGPVK_2

AA R2: 0.98 k: 0.297 dk: 0.0544 | HW R2: 0.95 k: 0.239 dk: 0.0464

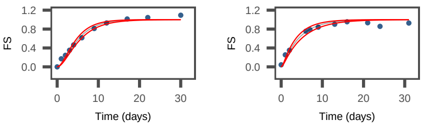

PDIA1 – DGVVLFK_2

AA R2: 0.98 k: 0.343 dk: 0.066 | HW R2: 0.92 k: 0.237 dk: 0.0835

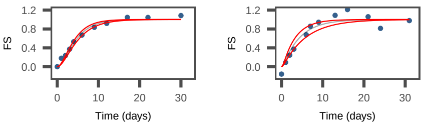

PDIA1 – THILLFLPK_2

AA R2: 0.96 k: 0.468 dk: 0.12 | HW R2: 0.91 k: 0.135 dk: 0.0347

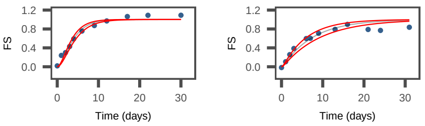

PDIA4 – EVSQPDWTPPPEVTLSLTK_2

AA R2: 0.91 k: 0.051 dk: 0.0108 | HW R2: 0.94 k: 0.298 dk: 0.0635

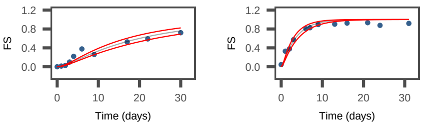

PDIA1 – DHENIIIAK_2

AA R2: 0.95 k: 0.412 dk: 0.12 | HW R2: 0.98 k: 0.271 dk: 0.0375

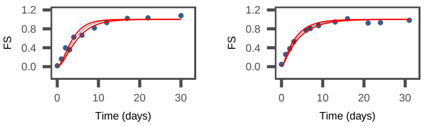

PDIA1 – THILLFLPK_3

AA R2: 0.99 k: 0.372 dk: 0.0573 | HW R2: 0.92 k: 0.0999 dk: 0.0271

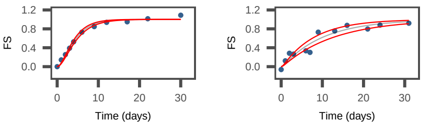

PDIA5 – FLFPYENYGSTAEDIVEWLK_2

AA R2: 0.96 k: 0.153 dk: 0.0297 | HW R2: 0.99 k: 0.114 dk: 0.00913

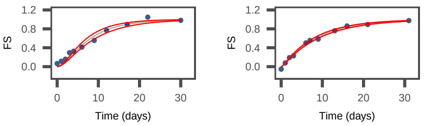

PDIA1 – HNQLPLVIEFTEQTAPK_3

AA R2: 0.96 k: 0.354 dk: 0.0989 | HW R2: 0.98 k: 0.282 dk: 0.0333

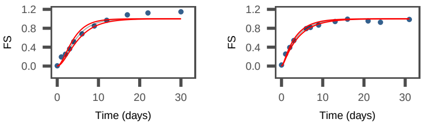

PDIA3 – DLFSDGHSEFLK_3

AA R2: 0.97 k: 0.329 dk: 0.0689 | HW R2: 0.98 k: 0.354 dk: 0.0487

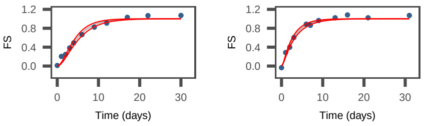

PDIA6 – HQSLGGQYGVQGFPTIK_2

AA R2: 0.98 k: 0.29 dk: 0.0475 | HW R2: 0.95 k: 0.484 dk: 0.11

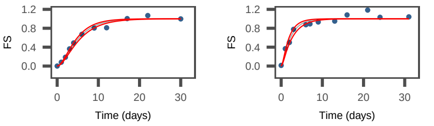

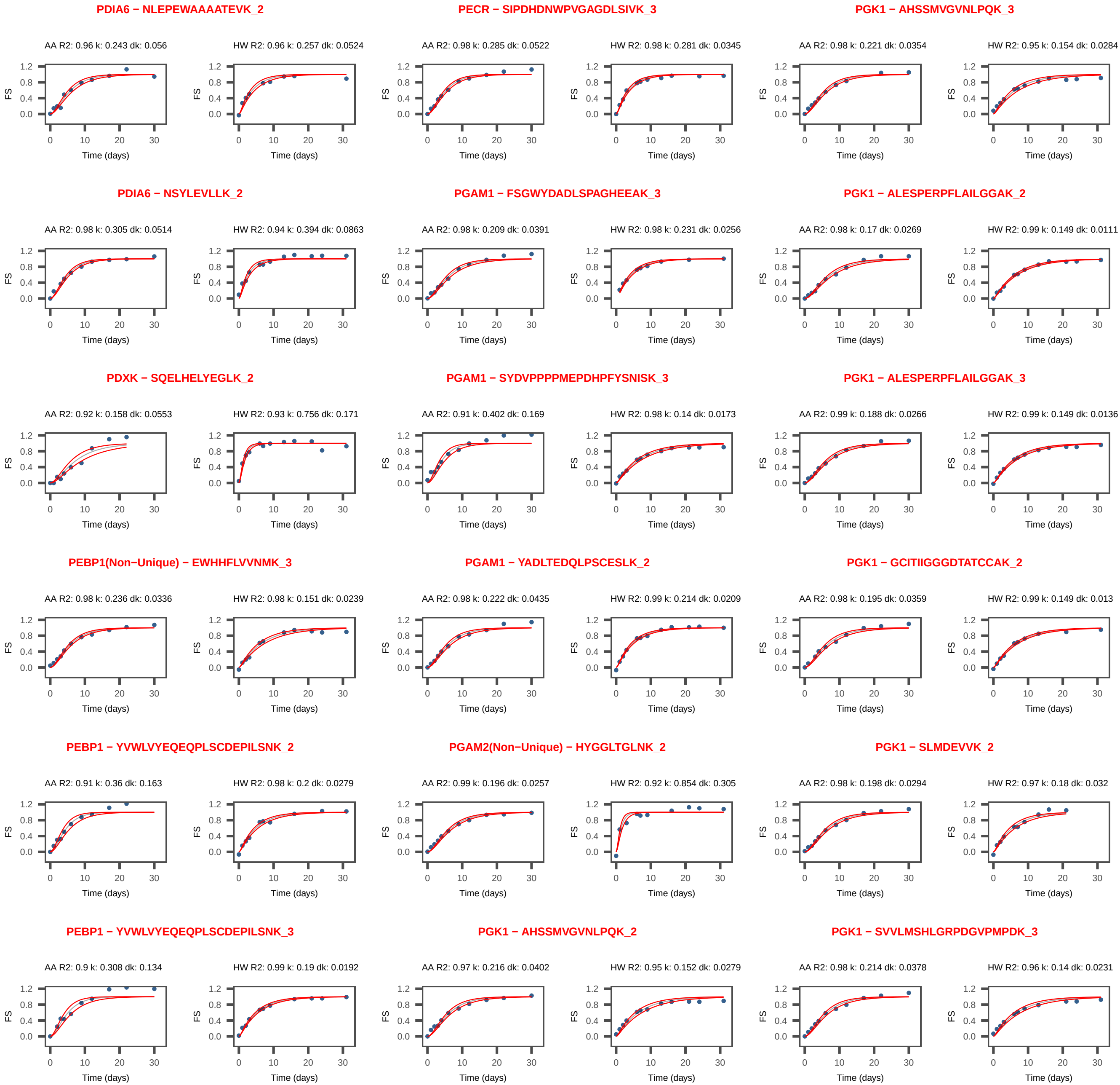
PDIA6 − NLEPEWAAAATEVK_2
AA R2: 0.96 k: 0.243 dk: 0.056
HW R2: 0.96 k: 0.257 dk: 0.0524
PECR − SIPDHDNWPVGAGDLSIVK_3
AA R2: 0.98 k: 0.285 dk: 0.0522
HW R2: 0.98 k: 0.281 dk: 0.0345
PGK1 − AHSSMVGVNLPQK_3
AA R2: 0.98 k: 0.221 dk: 0.0354
HW R2: 0.95 k: 0.154 dk: 0.0284
PDIA6 − NSYLEVLLK_2
AA R2: 0.98 k: 0.305 dk: 0.0514
HW R2: 0.94 k: 0.394 dk: 0.0863
PGAM1 − FSGWYDADLSPAGHEEAK_3
AA R2: 0.98 k: 0.209 dk: 0.0391
HW R2: 0.98 k: 0.231 dk: 0.0256
PGK1 − ALESPERPFLAILGGAK_2
AA R2: 0.98 k: 0.17 dk: 0.0269
HW R2: 0.99 k: 0.149 dk: 0.0111
PDXK − SQELHELYEGLK_2
AA R2: 0.92 k: 0.158 dk: 0.0553
HW R2: 0.93 k: 0.756 dk: 0.171
PGAM1 − SYDVPPPPMEPDHPFYSNISK_3
AA R2: 0.91 k: 0.402 dk: 0.169
HW R2: 0.98 k: 0.14 dk: 0.0173
PGK1 − ALESPERPFLAILGGAK_3
AA R2: 0.99 k: 0.188 dk: 0.0266
HW R2: 0.99 k: 0.149 dk: 0.0136
PEBP1(Non−Unique) − EWHHFLVVNMK_3
AA R2: 0.98 k: 0.236 dk: 0.0336
HW R2: 0.98 k: 0.151 dk: 0.0239
PGAM1 − YADLTEDQLPSCESLK_2
AA R2: 0.98 k: 0.222 dk: 0.0435
HW R2: 0.99 k: 0.214 dk: 0.0209
PGK1 − GCITIIGGGDTATCCAK_2
AA R2: 0.98 k: 0.195 dk: 0.0359
HW R2: 0.99 k: 0.149 dk: 0.013
PEBP1 − YVWLVYEQEQPLSCDEPILSNK_2
AA R2: 0.91 k: 0.36 dk: 0.163
HW R2: 0.98 k: 0.2 dk: 0.0279
PGAM2(Non−Unique) − HYGGLTGLNK_2
AA R2: 0.99 k: 0.196 dk: 0.0257
HW R2: 0.92 k: 0.854 dk: 0.305
PGK1 − SLMDEVVK_2
AA R2: 0.98 k: 0.198 dk: 0.0294
HW R2: 0.97 k: 0.18 dk: 0.032
PEBP1 − YVWLVYEQEQPLSCDEPILSNK_3
AA R2: 0.9 k: 0.308 dk: 0.134
HW R2: 0.99 k: 0.19 dk: 0.0192
PGK1 − AHSSMVGVNLPQK_2
AA R2: 0.97 k: 0.216 dk: 0.0402
HW R2: 0.95 k: 0.152 dk: 0.0279
PGK1 − SVVLMSHLGRPDGVPMPDK_3
AA R2: 0.98 k: 0.214 dk: 0.0378
HW R2: 0.96 k: 0.14 dk: 0.0231
FS
Time (days)

PGK1 − TGQATVASGIPAGWMGLDCGTESSK_2

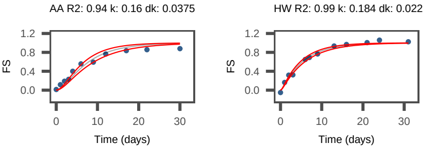

PGM1 − IDAMHGVVGPYVK_3

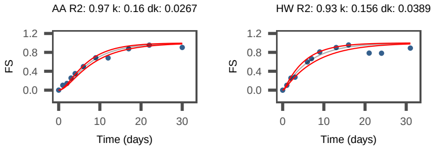

PHB − AATFGLILDDVSLTHLTFGK_3

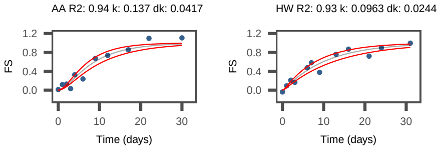

PGK1 − VLNNMEIGTSLYDEEGAK_2

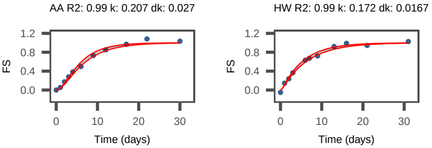

PGM1 − INQDPQVMLAPLISIALK_3

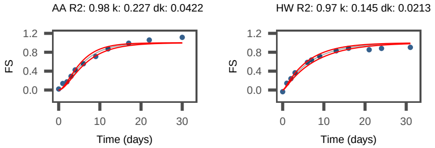

PHB − GVQDIVVGEGTHFLIPWVQK_3

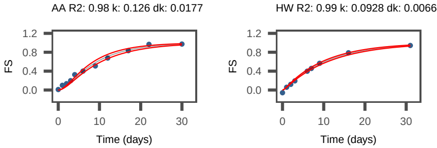

PGK2(Non−Unique) − VSHVSTGGGASLELLEGK_2

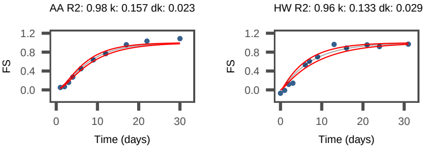

PGM1 − VDLGVLGK_2

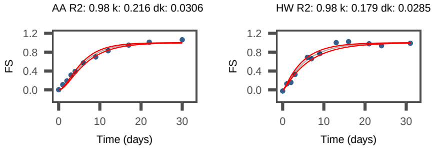

PHB − KLEAAEDIAYQLSR_2

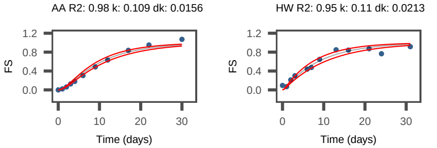

PGK2(Non−Unique) − VSHVSTGGGASLELLEGK_3

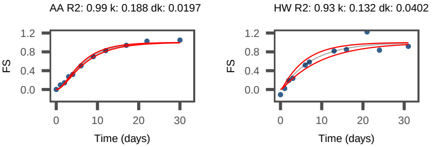

PGM1 − YDYEEVEAEGANK_2

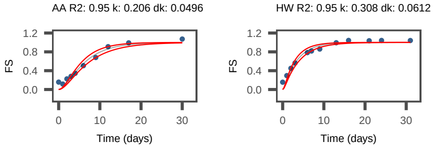

PHB − KLEAAEDIAYQLSR_3

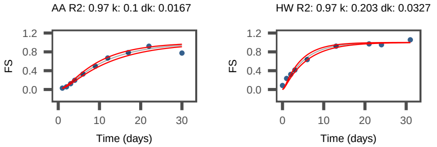

PGM1 − ADNFEYSDPVDGSISK_2

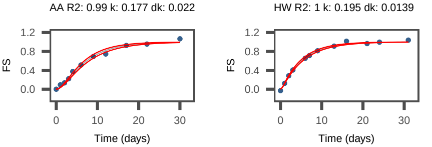

PGRC1(Non−Unique) − GLATFCLDK_2

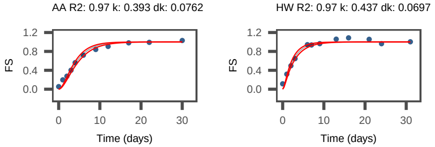

PHB − NVPVITGSK_2

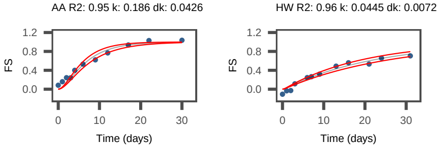

PGM1 − FFGNLMDASK_2

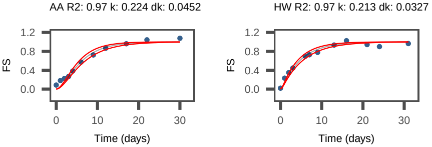

PHB − AATFGLILDDVSLTHLTFGK_2

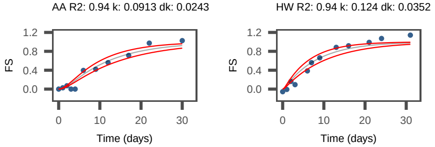

PHB − VLPSITTEILK_2

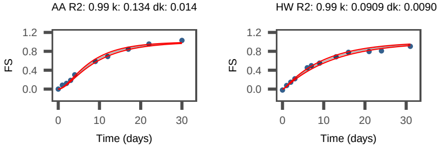

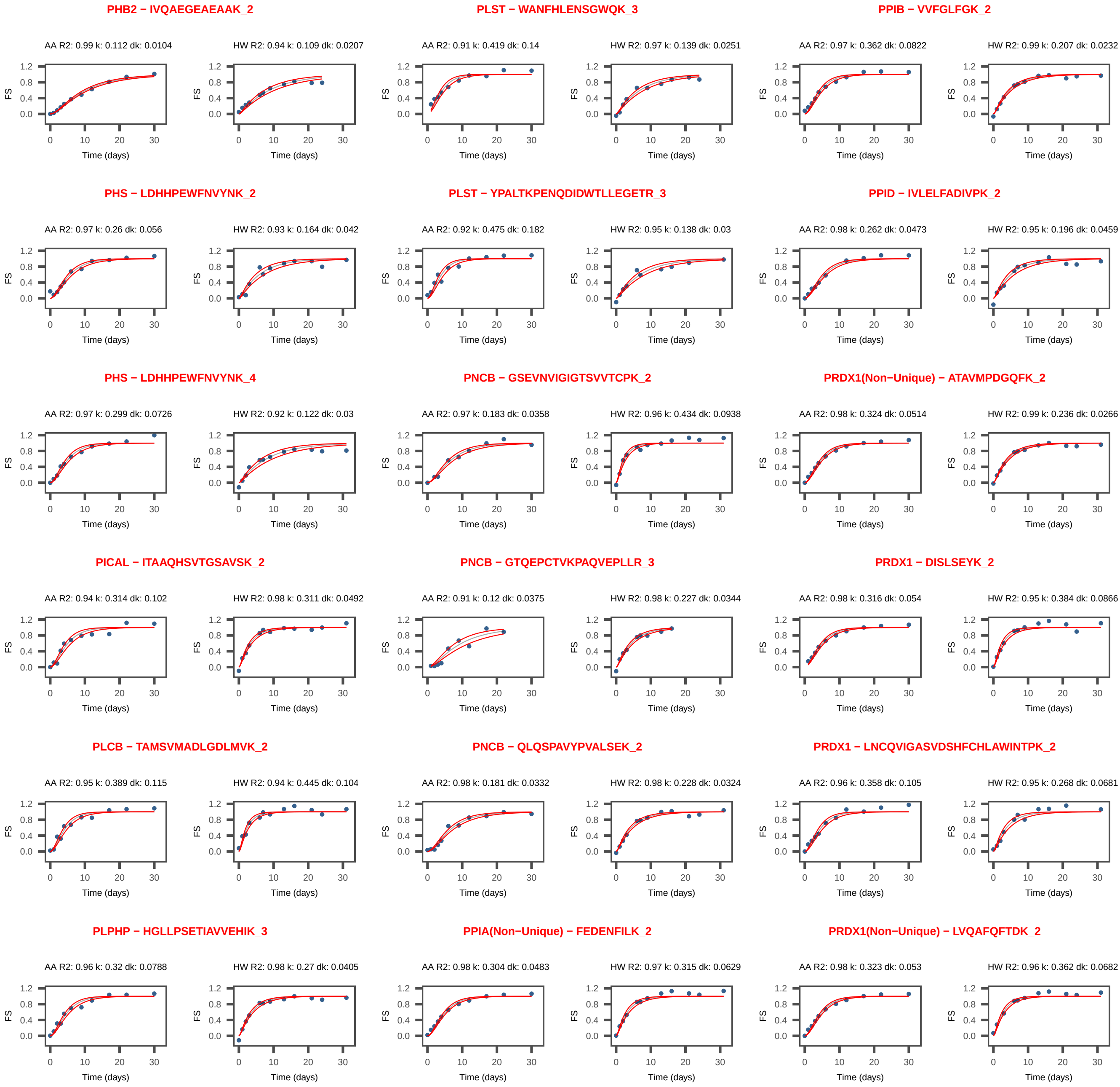
PHB2 − IVQAEGEAEAAK_2
AA R2: 0.99 k: 0.112 dk: 0.0104
HW R2: 0.94 k: 0.109 dk: 0.0207
PLST − WANFHLENSGWQK_3
AA R2: 0.91 k: 0.419 dk: 0.14
HW R2: 0.97 k: 0.139 dk: 0.0251
PPIB − VVFGLFGK_2
AA R2: 0.97 k: 0.362 dk: 0.0822
HW R2: 0.99 k: 0.207 dk: 0.0232
PHS − LDHHPEWFNVYNK_2
AA R2: 0.97 k: 0.26 dk: 0.056
HW R2: 0.93 k: 0.164 dk: 0.042
PLST − YPALTKPENQDIDWTLLEGETR_3
AA R2: 0.92 k: 0.475 dk: 0.182
HW R2: 0.95 k: 0.138 dk: 0.03
PPID − IVLELFADIVPK_2
AA R2: 0.98 k: 0.262 dk: 0.0473
HW R2: 0.95 k: 0.196 dk: 0.0459
PHS − LDHHPEWFNVYNK_4
AA R2: 0.97 k: 0.299 dk: 0.0726
HW R2: 0.92 k: 0.122 dk: 0.03
PNCB − GSEVNVIGIGTSVVTCPK_2
AA R2: 0.97 k: 0.183 dk: 0.0358
HW R2: 0.96 k: 0.434 dk: 0.0938
PRDX1(Non−Unique) − ATAVMPDGQFK_2
AA R2: 0.98 k: 0.324 dk: 0.0514
HW R2: 0.99 k: 0.236 dk: 0.0266
PICAL − ITAAQHSVTGSAVSK_2
AA R2: 0.94 k: 0.314 dk: 0.102
HW R2: 0.98 k: 0.311 dk: 0.0492
PNCB − GTQEPCTVKPAQVEPLLR_3
AA R2: 0.91 k: 0.12 dk: 0.0375
HW R2: 0.98 k: 0.227 dk: 0.0344
PRDX1 − DISLSEYK_2
AA R2: 0.98 k: 0.316 dk: 0.054
HW R2: 0.95 k: 0.384 dk: 0.0866
PLCB − TAMSVMADLGDLMVK_2
AA R2: 0.95 k: 0.389 dk: 0.115
HW R2: 0.94 k: 0.445 dk: 0.104
PNCB − QLQSPAVYPVALSEK_2
AA R2: 0.98 k: 0.181 dk: 0.0332
HW R2: 0.98 k: 0.228 dk: 0.0324
PRDX1 − LNCQVIGASVDSHFCHLAWINTPK_2
AA R2: 0.96 k: 0.358 dk: 0.105
HW R2: 0.95 k: 0.268 dk: 0.0681
PLPHP − HGLLPSETIAVVEHIK_3
AA R2: 0.96 k: 0.32 dk: 0.0788
HW R2: 0.98 k: 0.27 dk: 0.0405
PPIA(Non−Unique) − FEDENFILK_2
AA R2: 0.98 k: 0.304 dk: 0.0483
HW R2: 0.97 k: 0.315 dk: 0.0629
PRDX1(Non−Unique) − LVQAFQFTDK_2
AA R2: 0.98 k: 0.323 dk: 0.053
HW R2: 0.96 k: 0.362 dk: 0.0682
FS
Time (days)

PRDX1(Non−Unique) − TIAQDYGVLK_2

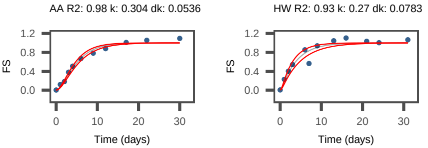

PRDX5 − VNLAELFK_2

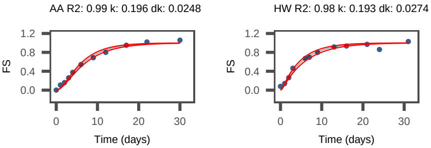

PRDX6 − LPFPIIDDK_2

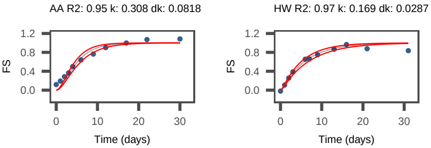

PRDX2 − EGGLGPLNIPLLADVTK_2

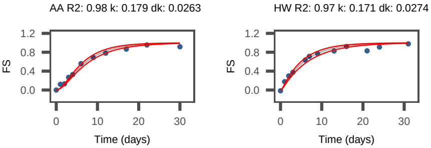

PRDX6 − DLAILLGMLDPVEK_2

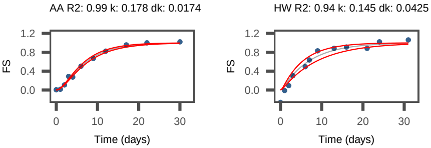

PRDX6 − VVFIFGPDK_2

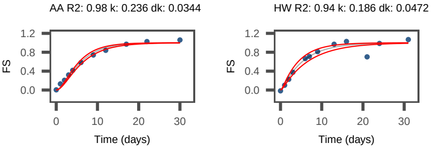

PRDX2 − GLFIIDAK_2

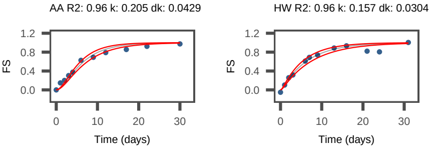

PRDX6 − DLAILLGMLDPVEK_3

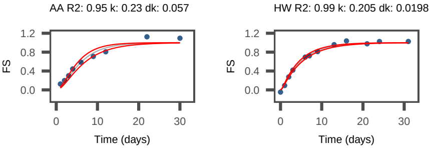

PROF1 − TFVSITPAEVGVLVGK_2

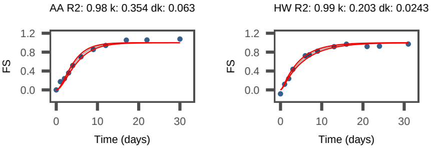

PRDX5 − GVLFGVPGAFTPGCSK_2

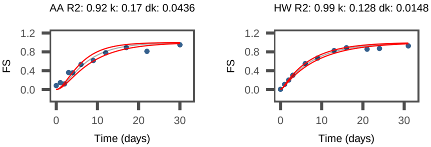

PRDX6 − LAPEFAK_2

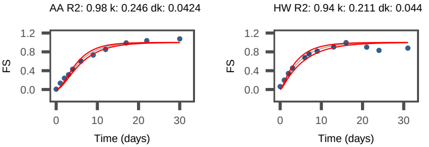

PSA2 − LVQIEYALAAVAGGAPSVGIK_2

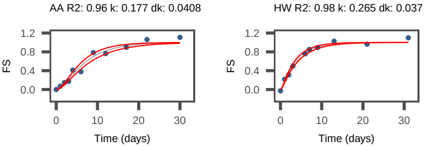

PRDX5 − LLADPTGAFGK_2

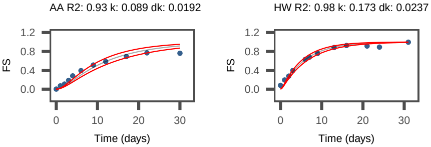

PRDX6 − LIALSIDSVEDHLAWSK_2

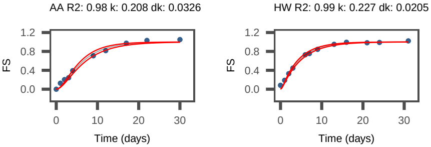

PSA2 − LVQIEYALAAVAGGAPSVGIK_3

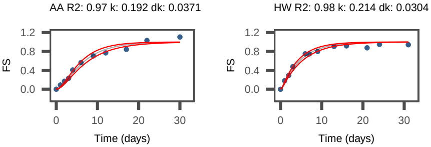

PRDX5 − THLPGFVEQAGALK_3

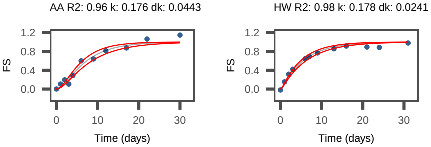

PRDX6 − LIALSIDSVEDHLAWSK_3

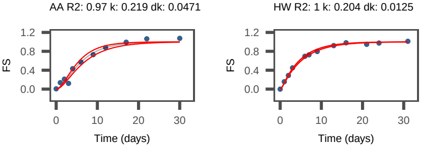

PSA6 − CDPAGYYCGFK_2

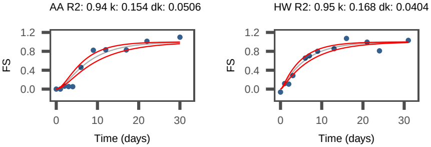

PSB4 − TQNPMVTGTSVLGVK_2

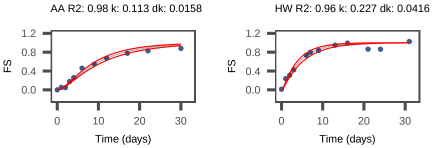

PURA − FFFDVGSNK_2

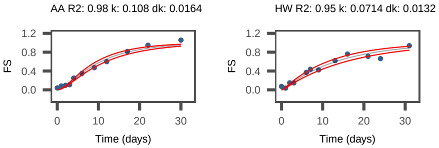

PYC − HGEEVTPEDVLSAAMYPDVFAQFK_3

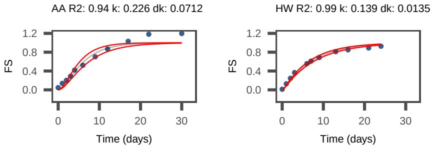

PSB5 − ATAGAYIASQTVK_2

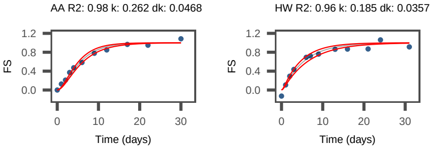

PXMP2 − ALAQYLLLLK_2

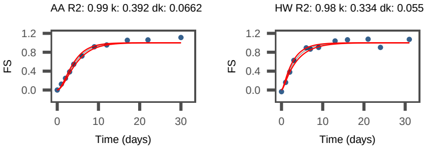

PYC − IAPYVAHNFNK_2

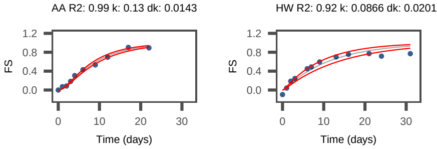

PSD11 − ALLVEVQLLESK_2

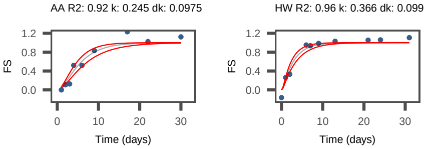

PXMP2 − AVSSGILSALGNLLAQTIEK_2

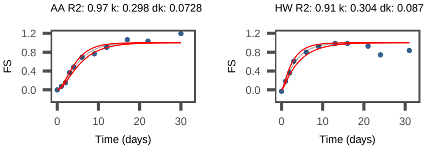

PYC − PGASLPPLNLK_2

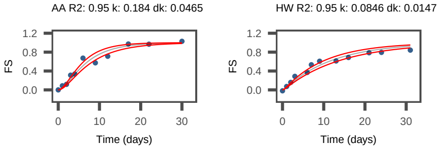

PSMD2 − LAQGLTHLGK_2

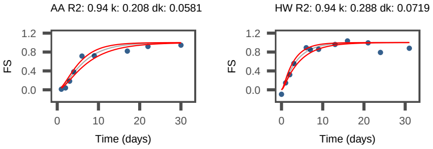

PXMP2 − AVSSGILSALGNLLAQTIEK_3

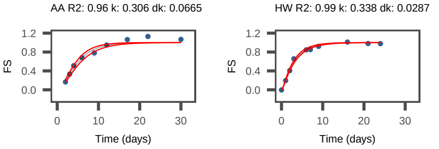

PYC − QVGYENAGTVEFLVDK_2

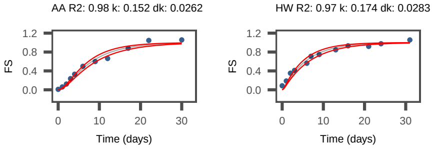

PTBP1 − IIVENLFYPVTLDVLHQIFSK_3

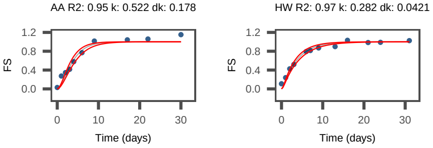

PYC − GLAPVQAYLHIPDIIK_2

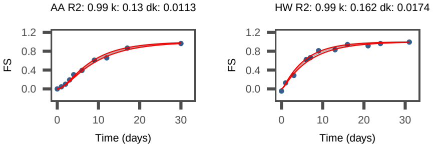

PYGB(Non−Unique) − VLYPNDNFFEGK_2

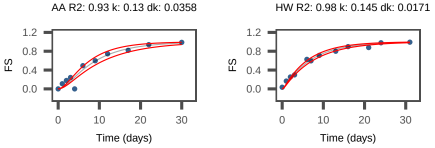

PTH2 − APDEDTLIQLLTHAK_3

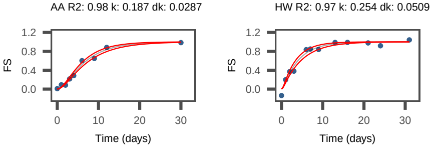

PYC − GLAPVQAYLHIPDIIK_3

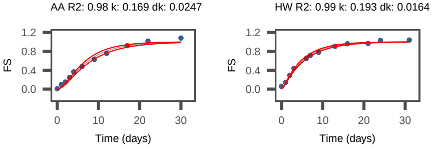

PYGL − HLEIIYEINQK_2

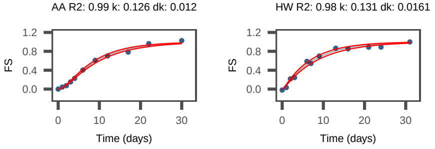

PYGL − HLEIIYEINQK_3

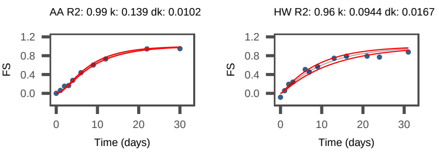

PYGL − VIPATDLSEQISTAGTEASGTGNMK_2

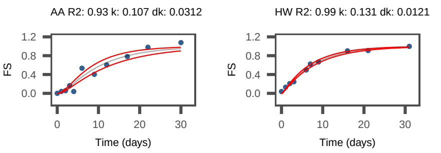

QCR10 − LILDWVPYINGK_2

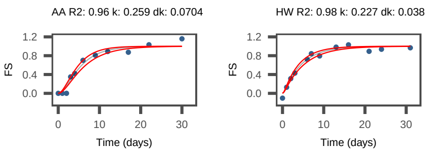

PYGL − INPSSMFDVHVK_3

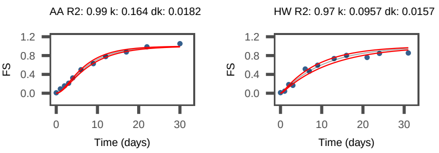

PYGL − VSQLYMNQK_2

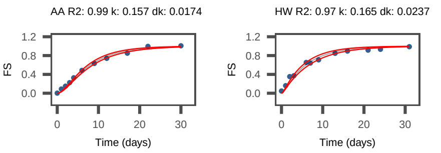

QCR2 − AVAQGNLSSADVQAAK_2

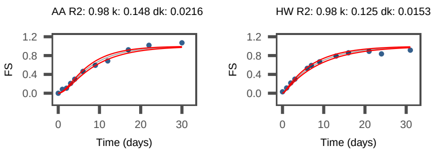

PYGL − LITSVAEVVNNDPMVGSK_2

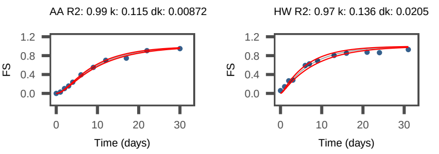

PYGL − WLLLCNPGLADLIAEK_2

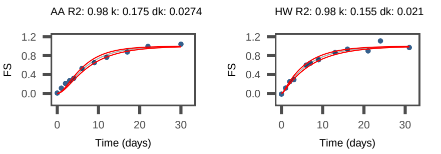

QCR2 − TSAAPGGVPLQPQDLEFTK_2

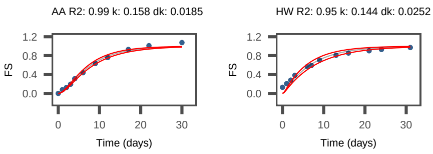

PYGL − LKQEYFVVAATLQDVIR_3

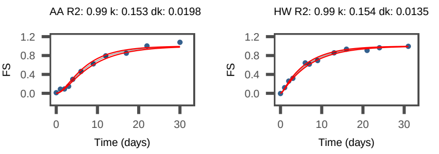

PYR1(Non−Unique) − AMLSTGFK_2

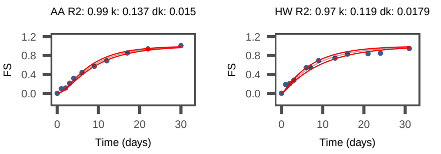

QOR − LQSDVVVPVPQSHQVLIK_3

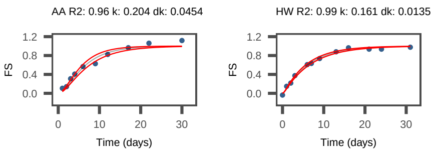

PYGL(Non−Unique) − LVAIFPK_2

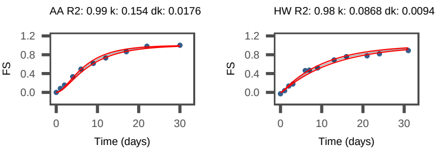

PYR1(Non−Unique) − SFPFVSK_2

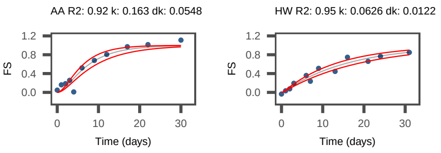

RAB18 − IIQTPGLWESENQNK_2

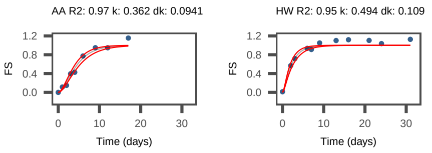

PYGL − MSLIEEEGGK_2

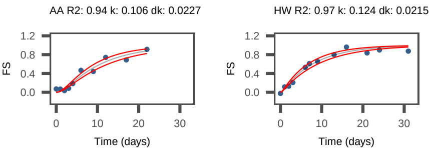

QCR1 − VVELLADIVQNSSLEDSQIEK_2

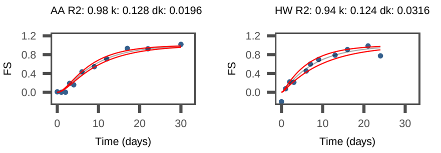

RACK1 − TNHIGHTGYLNTVTVSPDGSLCASGGK_3

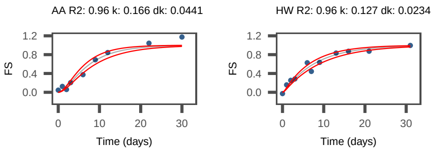

RAP1A(Non−Unique) − SALTVQFVQGIFVEK_2

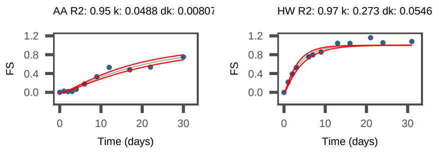

RDH7 − VSLCGGGYCISK_2

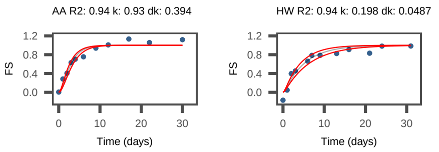

RIDA − TIYISGQVGLDPSSGQLVPGGVVEEAK_3

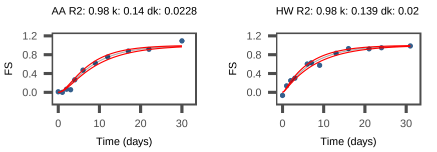

RDH16(Non−Unique) − LETVILDVTK_2

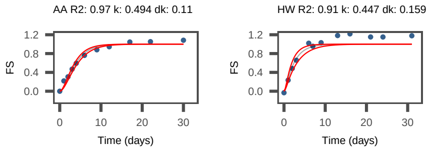

REEP5 − HESQVDSVVK_2

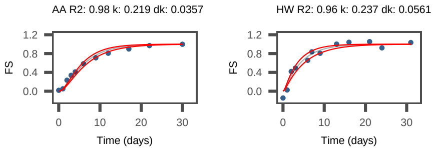

RIDA − VEIEAIAVQGPFIK_2

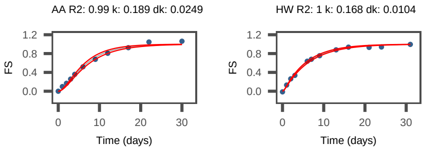

RDH16(Non−Unique) − QVVSHLQDK_2

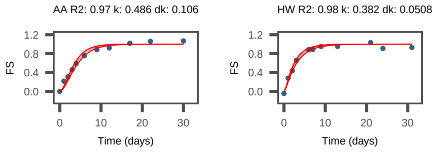

RGN − DEQIPDGMCIDAEGK_2

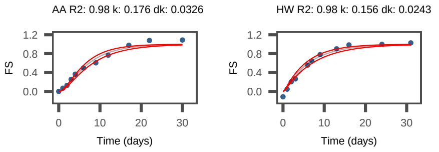

RISC − SYENLAFYWILK_2

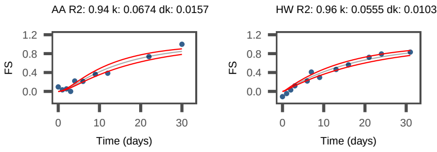

RDH16(Non−Unique) − QVVSHLQDK_3

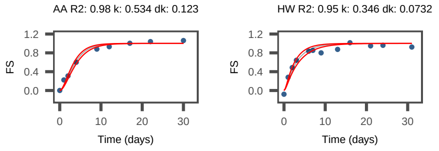

RGN − HQGSLYSLFPDHSVK_2

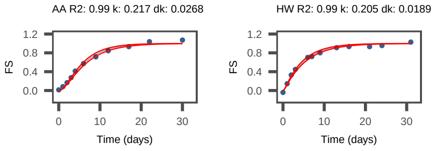

RL10 − FNADEFEDMVAEK_2

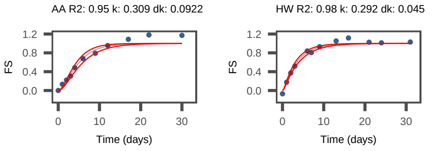

RDH7 − NFLDSYIK_2

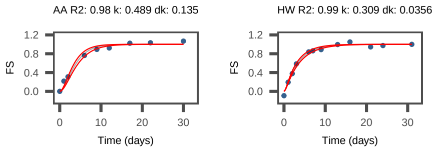

RIDA − AAGCDFNNVVK_2

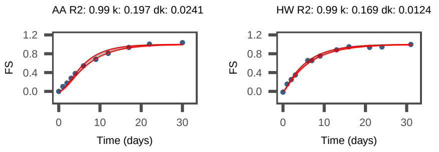

RL10L(Non−Unique) − GAFGKPQGTVAR_3

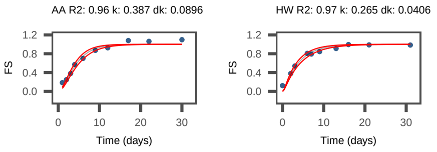

RDH7(Non−Unique) − TESIVAATQWVK_2

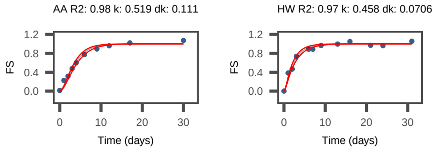

RIDA − TIYISGQVGLDPSSGQLVPGGVVEEAK_2

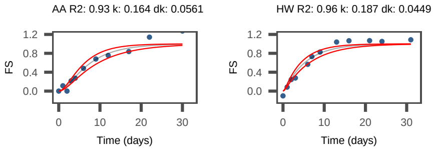

RL11 − YDGIILPGK_2

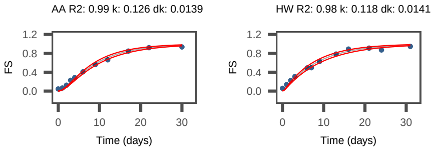

RL12 – CTGGEVGATSALAPK_2
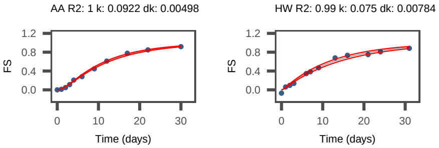

RL18 – TNSTFNQVVLK_2
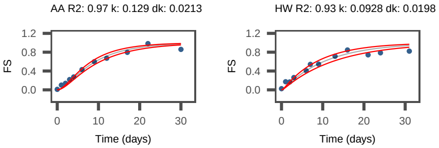

RL3 – SINPLGGFVHYGEVTNDFIMLK_3
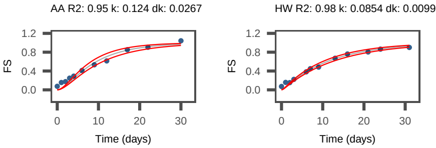

RL14 – AAIAAAAAAAAAK_2
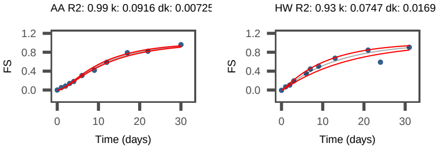

RL23 – ISLGLPVGAVINCADNTGAK_2
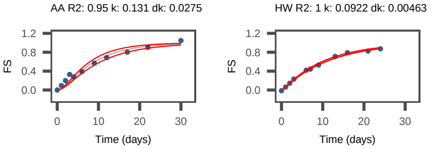

RL30 – TGVHHYSGNNIELGTACGK_2
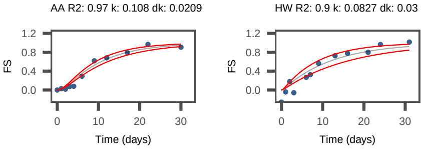

RL15 – FFEVILIDPFHK_3
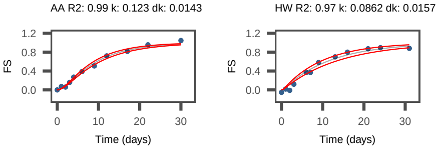

RL23A – VNTLIRPDGEK_2
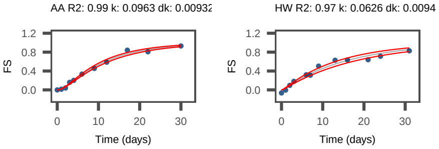

RL30 – TGVHHYSGNNIELGTACGK_3
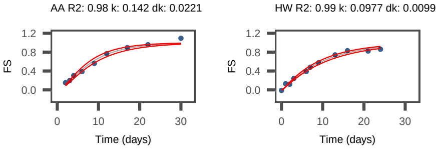

RL15 – PVHHGVNQLK_2
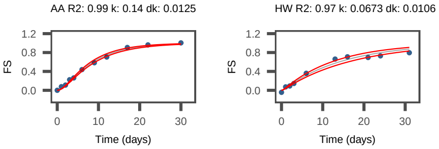

RL26 – KDDEVQVVR_2
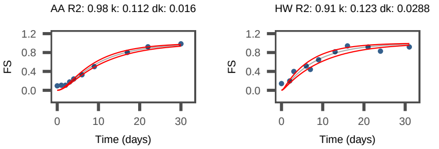

RL30 – TGVHHYSGNNIELGTACGK_4
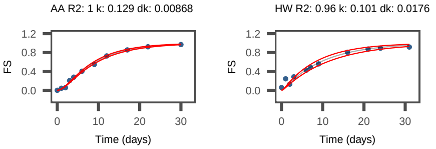

RL15 – VLNSYWVGEDSTYK_2
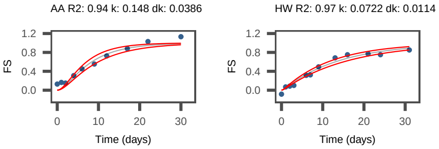

RL27A – NQSFCPTVNLDK_2
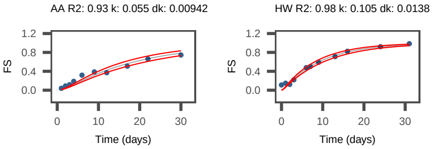

RL31 – LYTLVTYVPVTTFK_2
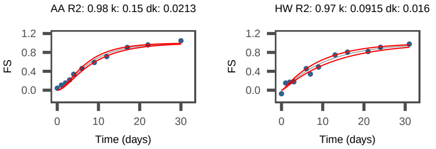

RL17 – GLDVDSLVIEHIQVNK_2
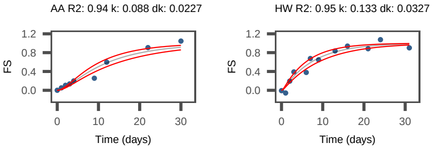

RL3 – NNASTDYDLSDK_2
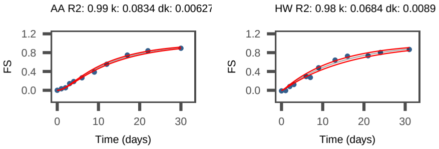

RL32 – SYCAEIAHNVSSK_2
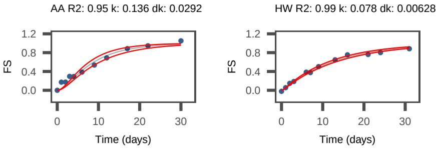

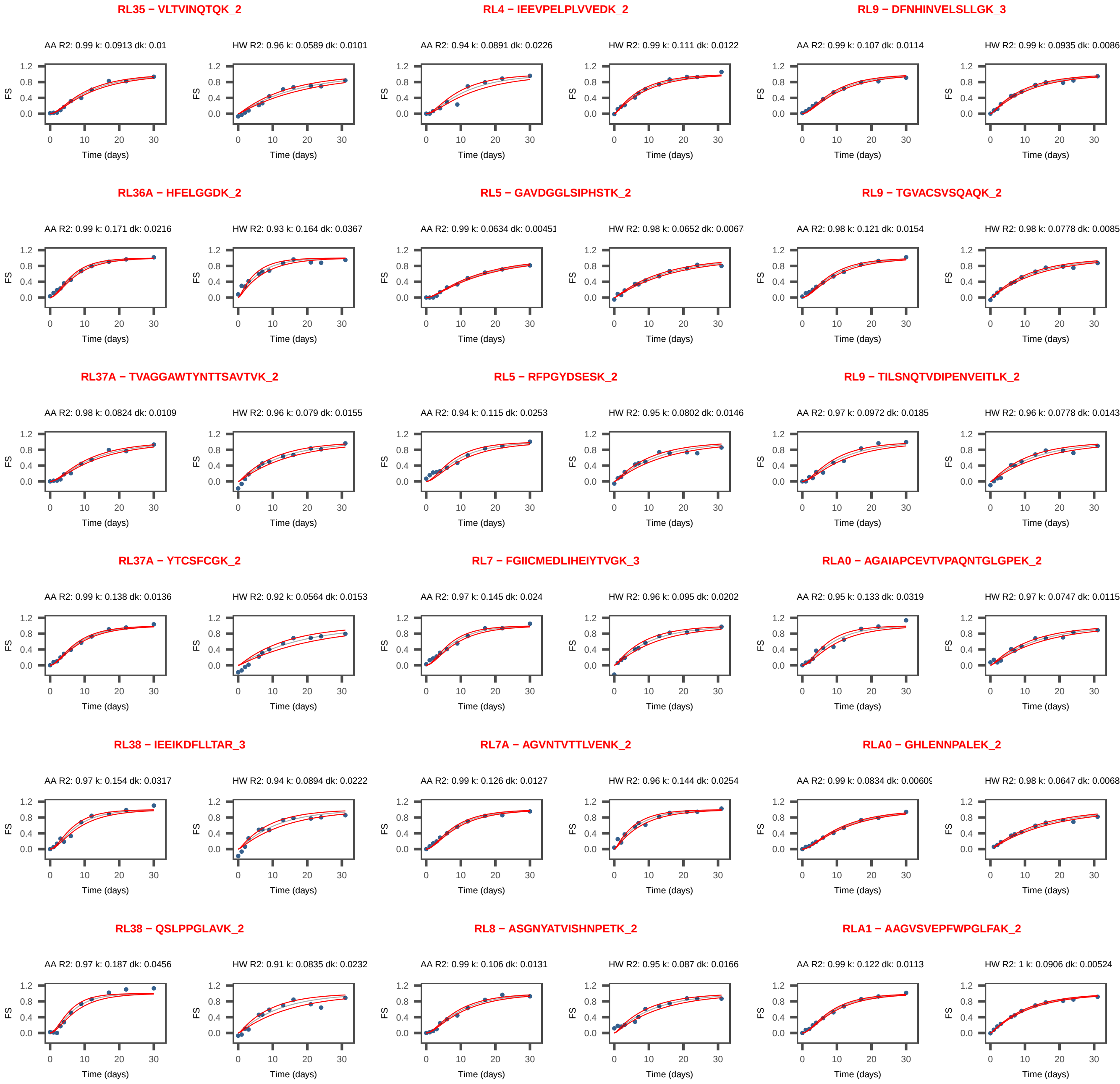
RL35 − VLTVINQTQK_2
AA R2: 0.99 k: 0.0913 dk: 0.01
HW R2: 0.96 k: 0.0589 dk: 0.0101
RL4 − IEEVPELPLVVEDK_2
AA R2: 0.94 k: 0.0891 dk: 0.0226
HW R2: 0.99 k: 0.111 dk: 0.0122
RL9 − DFNHINVELSLLGK_3
AA R2: 0.99 k: 0.107 dk: 0.0114
HW R2: 0.99 k: 0.0935 dk: 0.0086
RL36A − HFELGGDK_2
AA R2: 0.99 k: 0.171 dk: 0.0216
HW R2: 0.93 k: 0.164 dk: 0.0367
RL5 − GAVDGGLSIPHSTK_2
AA R2: 0.99 k: 0.0634 dk: 0.00451
HW R2: 0.98 k: 0.0652 dk: 0.0067
RL9 − TGVACSVSQAQK_2
AA R2: 0.98 k: 0.121 dk: 0.0154
HW R2: 0.98 k: 0.0778 dk: 0.0085
RL37A − TVAGGAWTYNTTSAVTVK_2
AA R2: 0.98 k: 0.0824 dk: 0.0109
HW R2: 0.96 k: 0.079 dk: 0.0155
RL5 − RFPGYDSESK_2
AA R2: 0.94 k: 0.115 dk: 0.0253
HW R2: 0.95 k: 0.0802 dk: 0.0146
RL9 − TILSNQTVDIPENVEITLK_2
AA R2: 0.97 k: 0.0972 dk: 0.0185
HW R2: 0.96 k: 0.0778 dk: 0.0143
RL37A − YTCSFCGK_2
AA R2: 0.99 k: 0.138 dk: 0.0136
HW R2: 0.92 k: 0.0564 dk: 0.0153
RL7 − FGIICMEDLIHEIYTVGK_3
AA R2: 0.97 k: 0.145 dk: 0.024
HW R2: 0.96 k: 0.095 dk: 0.0202
RLA0 − AGAIAPCEVTVPAQNTGLGPEK_2
AA R2: 0.95 k: 0.133 dk: 0.0319
HW R2: 0.97 k: 0.0747 dk: 0.0115
RL38 − IEEIKDFLLTAR_3
AA R2: 0.97 k: 0.154 dk: 0.0317
HW R2: 0.94 k: 0.0894 dk: 0.0222
RL7A − AGVNTVTTLVENK_2
AA R2: 0.99 k: 0.126 dk: 0.0127
HW R2: 0.96 k: 0.144 dk: 0.0254
RLA0 − GHLENNPALEK_2
AA R2: 0.99 k: 0.0834 dk: 0.0060
HW R2: 0.98 k: 0.0647 dk: 0.0068
RL38 − QSLPPGLAVK_2
AA R2: 0.97 k: 0.187 dk: 0.0456
HW R2: 0.91 k: 0.0835 dk: 0.0232
RL8 − ASGNYATVISHNPETK_2
AA R2: 0.99 k: 0.106 dk: 0.0131
HW R2: 0.95 k: 0.087 dk: 0.0166
RLA1 − AAGVSVEPFWPGLFAK_2
AA R2: 0.99 k: 0.122 dk: 0.0113
HW R2: 1 k: 0.0906 dk: 0.00524

RLA2 − ILDSVGIEADDDRLNK_3

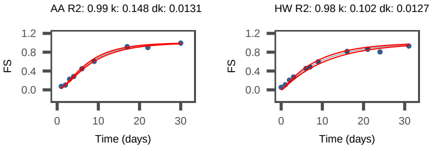

ROA3 − GFAFVTFDDHDTVDK_3

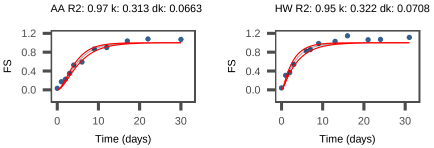

RS10 − IAIYELLFK_2

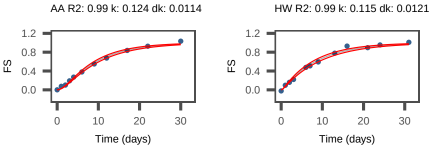

RLA2 − NIEDVIAQGVGK_2

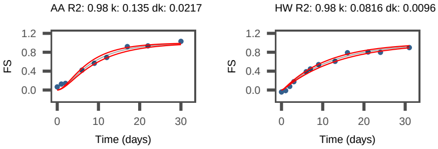

RPN1 − NLVEQHIQDIVVHYTFNK_3

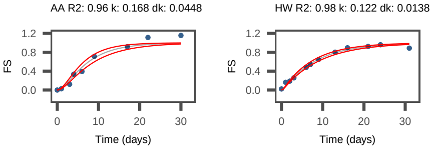

RS12 − QAHLCVLASNCDEPMYVK_3

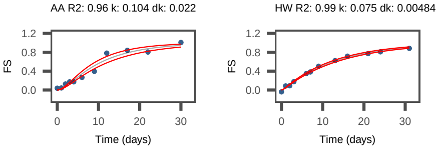

RMD3 − TATALFESPLSATVQDALQSFLK_3

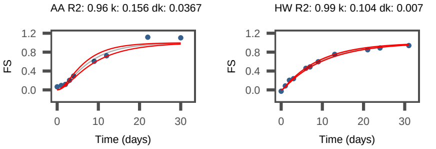

RPN1 − NLVEQHIQDIVVHYTFNK_4

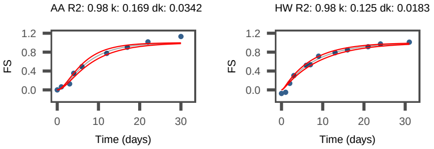

RS13 − GLAPDLPEDLYHLIK_3

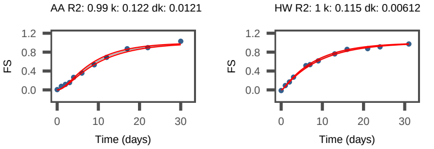

ROA2 − DYFEEYGK_2

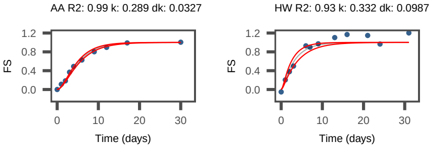

RPN1 − VACITEQVLTLVNK_2

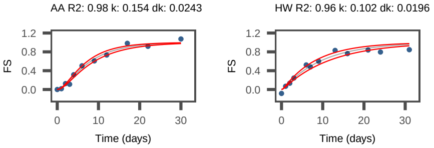

RS15 − DMIILPEMVGSMVGVYNGK_2

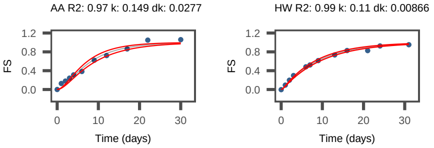

ROA2 − GFGFVTFDDHDPVDK_3

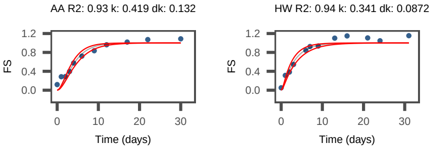

RPN2 − NPILWNVADVVIK_2

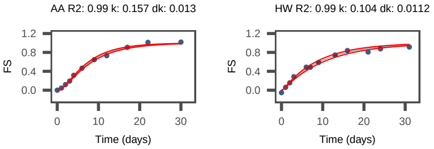

RS16 − LLEPVLLLGK_2

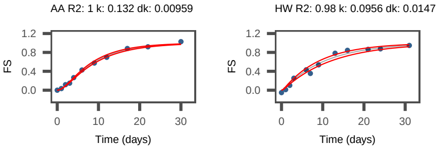

ROA2 − NYYEQWGK_2

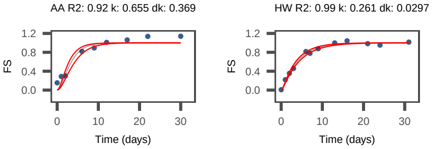

RRBP1 − HMAAASAECQNYAK_3

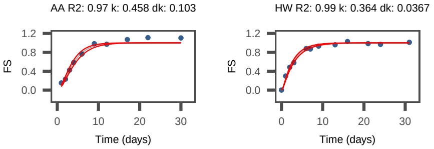

RS18 − IAFAITAIK_2

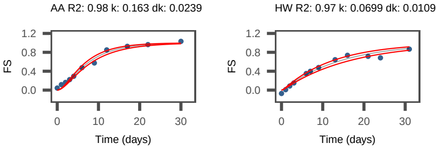

RS19 − ALAAFLK_2

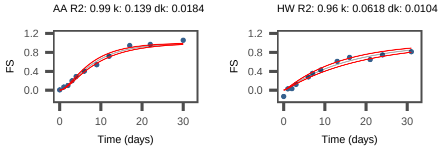

RS23 − AHLGTALK_2

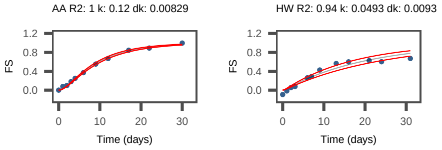

RS4X − ERHPGSFDVVHVK_4

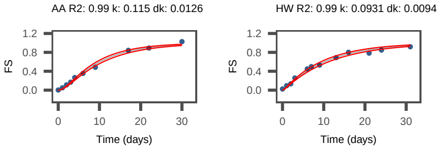

RS2 − ESEIIDFFLGASLK_2

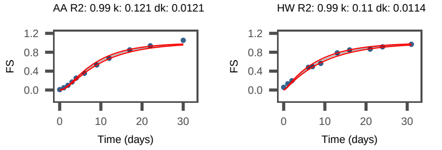

RS25 − LNNLVLFDK_2

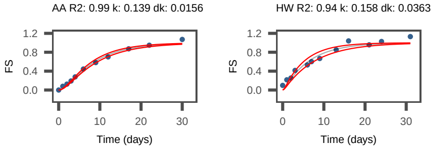

RS4X − HPGSFDVVHVK_2

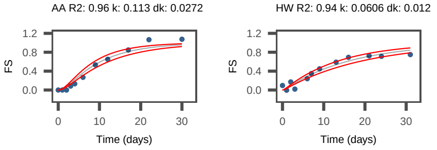

RS2 − ESEIIDFFLGASLK_3

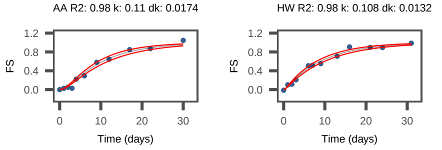

RS26 − DISEASVFDAYVLPK_2

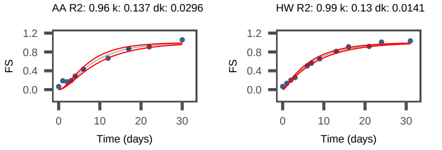

RS4X − LSNIFVIGK_2

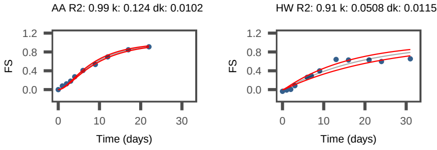

RS2 − SPYQEFTDHLVK_2

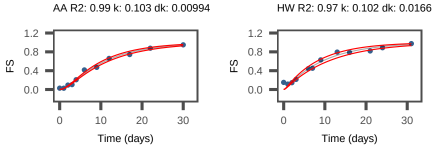

RS27(Non−Unique) − LVQSPNSYFMDVK_2

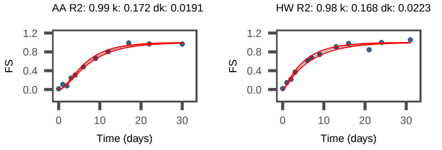

RS5 − WSTDDVQINDISLQDYIAVK_2

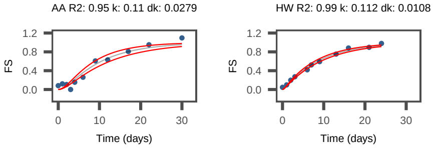

RS2 − SPYQEFTDHLVK_3

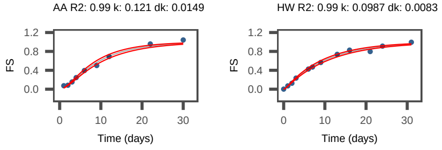

RS3 − FGFPEGSVELYAEK_2

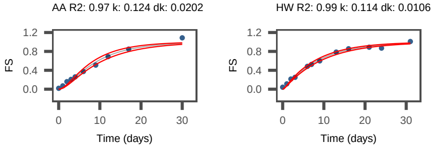

RS6 − GCIVDANLSVLNLVIVK_2

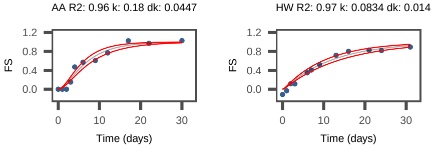

RS20 − LIDLHSPSEIVK_3

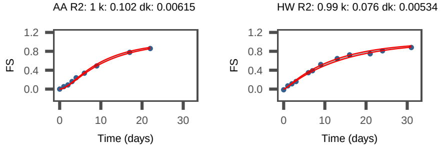

RS3 − QGVLGIK_2

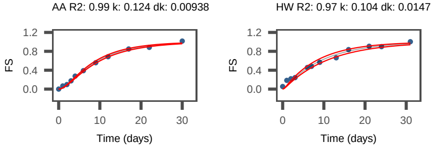

RS7 − AIIIFVPVPQLK_2

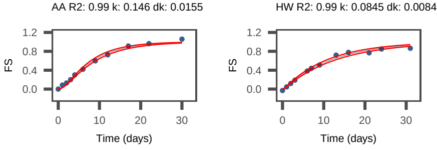

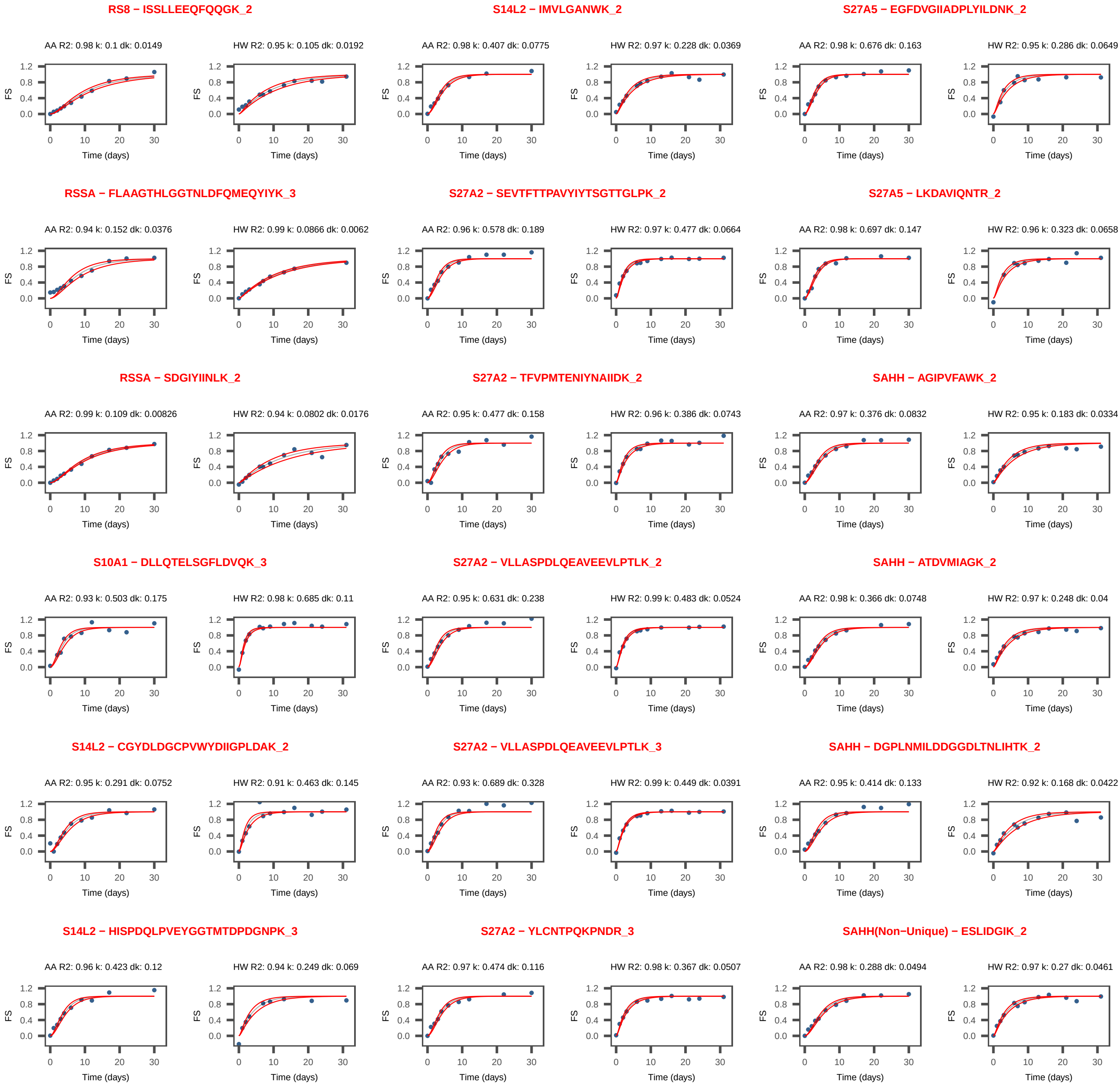
RS8 − ISSLLEEQFQQGK_2
AA R2: 0.98 k: 0.1 dk: 0.0149
HW R2: 0.95 k: 0.105 dk: 0.0192
S14L2 − IMVLGANWK_2
AA R2: 0.98 k: 0.407 dk: 0.0775
HW R2: 0.97 k: 0.228 dk: 0.0369
S27A5 − EGFDVGIIADPLYILDNK_2
AA R2: 0.98 k: 0.676 dk: 0.163
HW R2: 0.95 k: 0.286 dk: 0.0649
RSSA − FLAAGTHLGGTNLDFQMEQYIYK_3
AA R2: 0.94 k: 0.152 dk: 0.0376
HW R2: 0.99 k: 0.0866 dk: 0.0062
S27A2 − SEVTFTTPAVYIYTSGTTGLPK_2
AA R2: 0.96 k: 0.578 dk: 0.189
HW R2: 0.97 k: 0.477 dk: 0.0664
S27A5 − LKDAVIQNTR_2
AA R2: 0.98 k: 0.697 dk: 0.147
HW R2: 0.96 k: 0.323 dk: 0.0658
RSSA − SDGIYIINLK_2
AA R2: 0.99 k: 0.109 dk: 0.00826
HW R2: 0.94 k: 0.0802 dk: 0.0176
S27A2 − TFVPMTENIYNAIIDK_2
AA R2: 0.95 k: 0.477 dk: 0.158
HW R2: 0.96 k: 0.386 dk: 0.0743
SAHH − AGIPVFAWK_2
AA R2: 0.97 k: 0.376 dk: 0.0832
HW R2: 0.95 k: 0.183 dk: 0.0334
S10A1 − DLLQTELSGFLDVQK_3
AA R2: 0.93 k: 0.503 dk: 0.175
HW R2: 0.98 k: 0.685 dk: 0.11
S27A2 − VLLASPDLQEAVEEVLPTLK_2
AA R2: 0.95 k: 0.631 dk: 0.238
HW R2: 0.99 k: 0.483 dk: 0.0524
SAHH − ATDVMIAGK_2
AA R2: 0.98 k: 0.366 dk: 0.0748
HW R2: 0.97 k: 0.248 dk: 0.04
S14L2 − CGYDLDGCPVWYDIIGPLDAK_2
AA R2: 0.95 k: 0.291 dk: 0.0752
HW R2: 0.91 k: 0.463 dk: 0.145
S27A2 − VLLASPDLQEAVEEVLPTLK_3
AA R2: 0.93 k: 0.689 dk: 0.328
HW R2: 0.99 k: 0.449 dk: 0.0391
SAHH − DGPLNMILDDGGDLTNLIHTK_2
AA R2: 0.95 k: 0.414 dk: 0.133
HW R2: 0.92 k: 0.168 dk: 0.0422
S14L2 − HISPDQLPVEYGGTMTDPDGNPK_3
AA R2: 0.96 k: 0.423 dk: 0.12
HW R2: 0.94 k: 0.249 dk: 0.069
S27A2 − YLCNTPQKPNDR_3
AA R2: 0.97 k: 0.474 dk: 0.116
HW R2: 0.98 k: 0.367 dk: 0.0507
SAHH(Non−Unique) − ESLIDGIK_2
AA R2: 0.98 k: 0.288 dk: 0.0494
HW R2: 0.97 k: 0.27 dk: 0.0461
FS
Time (days)

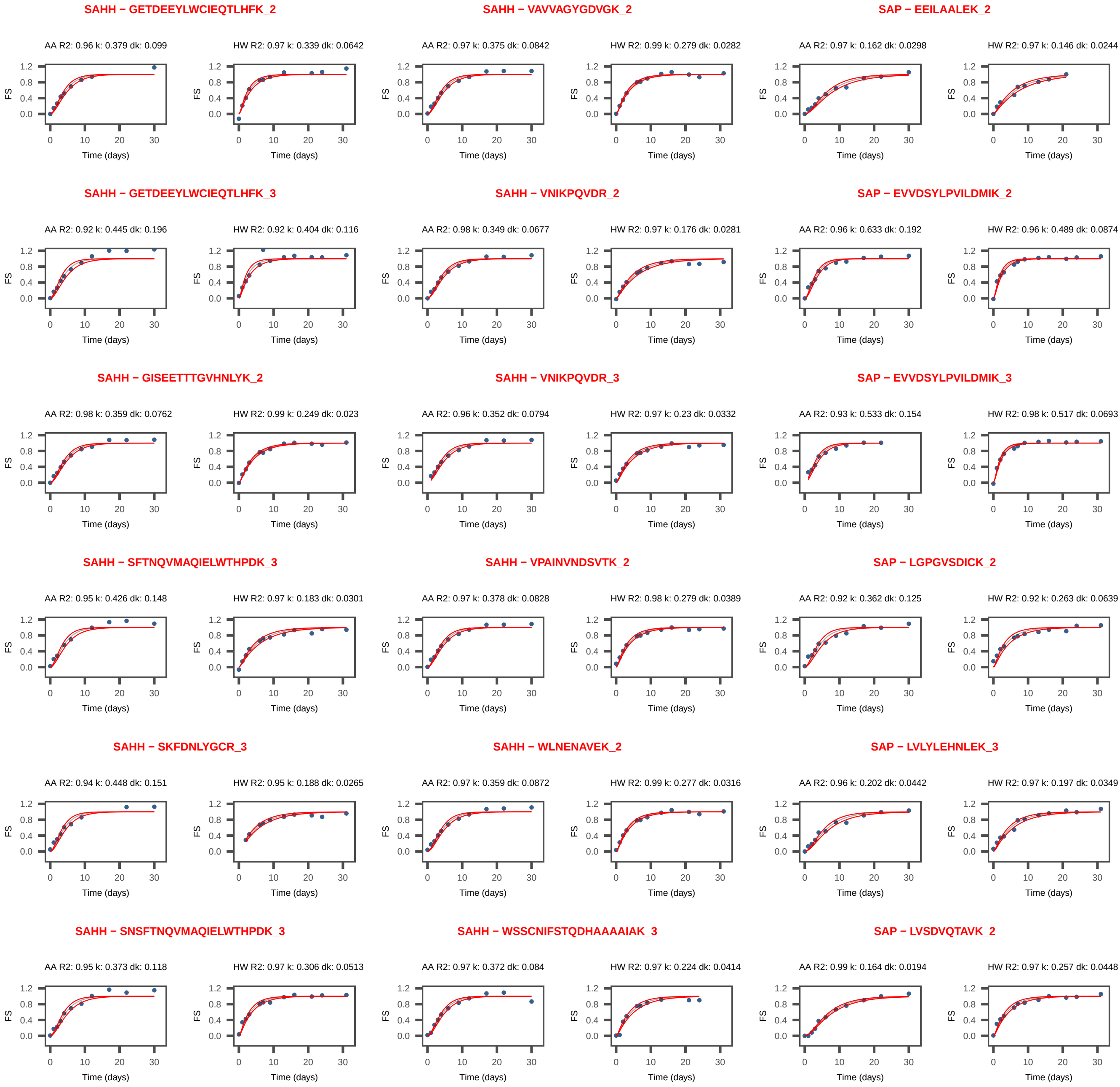
SAHH – GETDEEYLWCIEQTLHFK_2
AA R2: 0.96 k: 0.379 dk: 0.099
HW R2: 0.97 k: 0.339 dk: 0.0642
SAHH – VAVVAGYGDVGK_2
AA R2: 0.97 k: 0.375 dk: 0.0842
HW R2: 0.99 k: 0.279 dk: 0.0282
SAP – EEILAALEK_2
AA R2: 0.97 k: 0.162 dk: 0.0298
HW R2: 0.97 k: 0.146 dk: 0.0244
SAHH – GETDEEYLWCIEQTLHFK_3
AA R2: 0.92 k: 0.445 dk: 0.196
HW R2: 0.92 k: 0.404 dk: 0.116
SAHH – VNIKPQVDR_2
AA R2: 0.98 k: 0.349 dk: 0.0677
HW R2: 0.97 k: 0.176 dk: 0.0281
SAP – EVVDSYLPVILDMIK_2
AA R2: 0.96 k: 0.633 dk: 0.192
HW R2: 0.96 k: 0.489 dk: 0.0874
SAHH – GISEETTTGVHNLYK_2
AA R2: 0.98 k: 0.359 dk: 0.0762
HW R2: 0.99 k: 0.249 dk: 0.023
SAHH – VNIKPQVDR_3
AA R2: 0.96 k: 0.352 dk: 0.0794
HW R2: 0.97 k: 0.23 dk: 0.0332
SAP – EVVDSYLPVILDMIK_3
AA R2: 0.93 k: 0.533 dk: 0.154
HW R2: 0.98 k: 0.517 dk: 0.0693
SAHH – SFTNQVMAQIELWTHPDK_3
AA R2: 0.95 k: 0.426 dk: 0.148
HW R2: 0.97 k: 0.183 dk: 0.0301
SAHH – VPAINVNDSVTK_2
AA R2: 0.97 k: 0.378 dk: 0.0828
HW R2: 0.98 k: 0.279 dk: 0.0389
SAP – LGPGVSDICK_2
AA R2: 0.92 k: 0.362 dk: 0.125
HW R2: 0.92 k: 0.263 dk: 0.0639
SAHH – SKFDNLYGCR_3
AA R2: 0.94 k: 0.448 dk: 0.151
HW R2: 0.95 k: 0.188 dk: 0.0265
SAHH – WLNENAVEK_2
AA R2: 0.97 k: 0.359 dk: 0.0872
HW R2: 0.99 k: 0.277 dk: 0.0316
SAP – LVLYLEHNLEK_3
AA R2: 0.96 k: 0.202 dk: 0.0442
HW R2: 0.97 k: 0.197 dk: 0.0349
SAHH – SNSFTNQVMAQIELWTHPDK_3
AA R2: 0.95 k: 0.373 dk: 0.118
HW R2: 0.97 k: 0.306 dk: 0.0513
SAHH – WSSCNIFSTQDHAAAAIAK_3
AA R2: 0.97 k: 0.372 dk: 0.084
HW R2: 0.97 k: 0.224 dk: 0.0414
SAP – LVSDVQTAVK_2
AA R2: 0.99 k: 0.164 dk: 0.0194
HW R2: 0.97 k: 0.257 dk: 0.0448
FS
Time (days)

SAP − TCEWIHDSSLSASCK_2

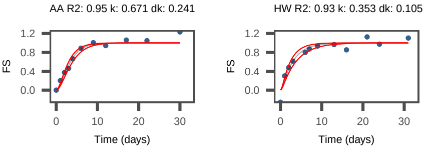

SARDH − GAAAVFNMSYFGK_2

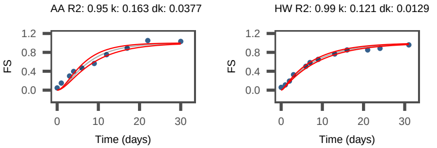

SBP1 − CGPGYSTPLEAMK_2

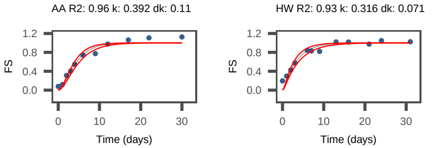

SAP − TVVTEAGNLLK_2

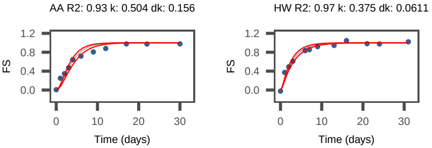

SARDH − LGGGCGQELAHWIVHGRPEK_4

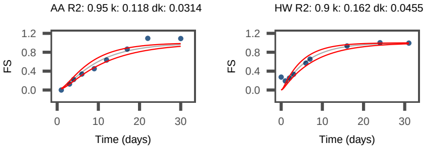

SBP1(Non−Unique) − DELHHSGWNTCSSCFGDSTK_3

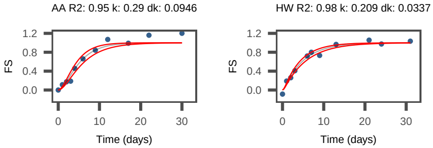

SARDH − AIDSLSIEK_2

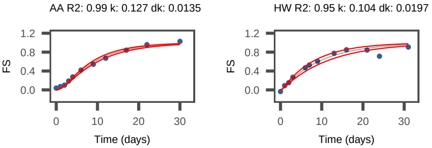

SARDH − PGWFNPQETAQVLDYDYYGAYGNQAHK_4

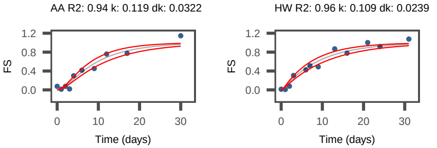

SBP1 − EGSMMLQIDVDTVNGGLK_2

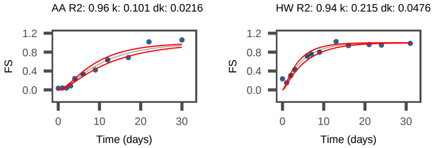

SARDH − AYGIESHVLSPAETK_2

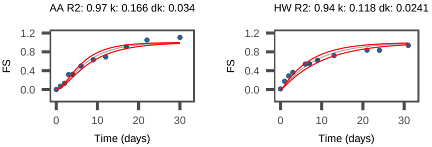

SARDH − SPFDPDNKR_2

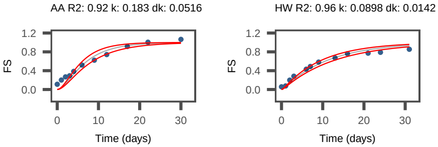

SBP1(Non−Unique) − FLVDFGKEPLGPALAHELR_4

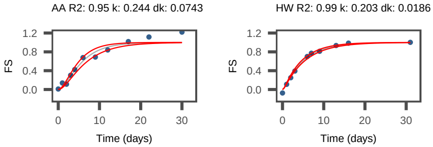

SARDH − AYGIESHVLSPAETK_3

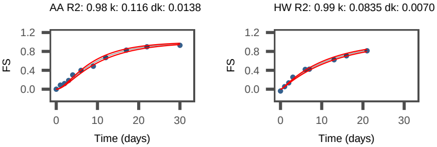

SARDH − STVCGPESFTPDHKPLMGEAPELR_4

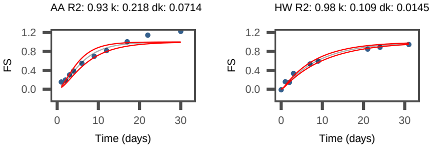

SBP1(Non−Unique) − GSFVLLDGETFEVK_2

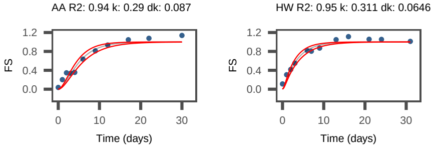

SARDH − DPSGGPVSLDFVK_2

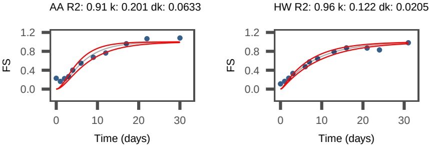

SARDH − VAAVETEHGSIQTPCVVNCAGVWASK_3

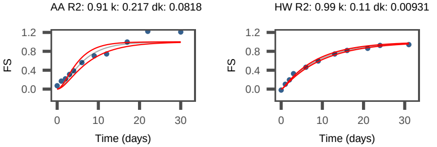

SBP1 − GTWEKPGDAAPMGYDFWYQPR_2

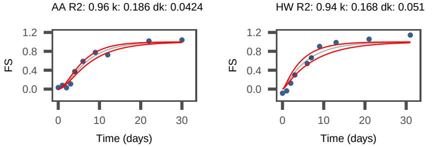

SBP1 − GTWEKPGDAAPMGYDFWYQPR_3

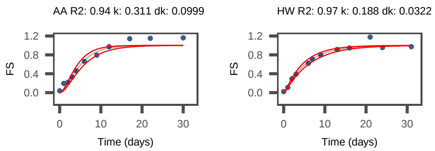

SBP1(Non−Unique) − QYDISNPQKPR_2

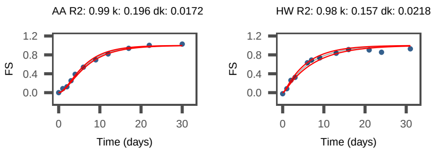

SCP2 − HIEVLIDK_2

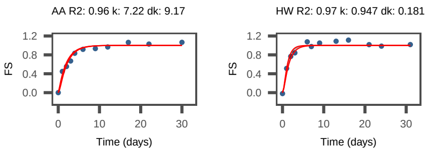

SBP1(Non−Unique) − HNVMVSTEWAAPNVFK_2

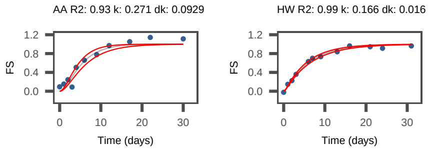

SBP1(Non−Unique) − VIEASEIQAK_2

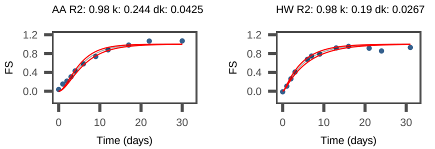

SCP2 − IAGNMGLAMK_2

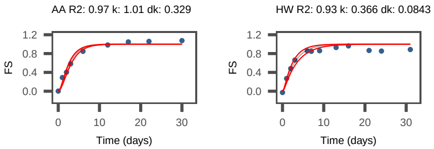

SBP1(Non−Unique) − IPGGPQMIQLSLDGK_2

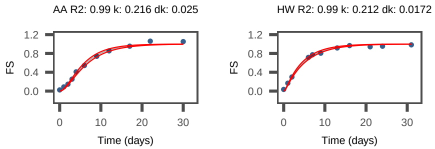

SC23A − MVVPVAALFTPLK_2

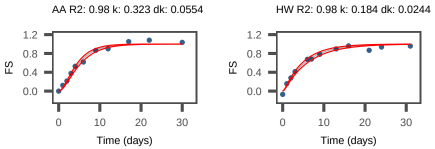

SCP2 − IGGIFAFK_2

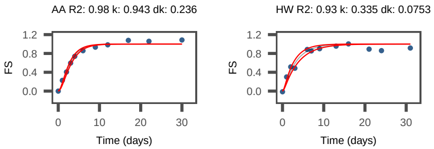

SBP1 − LYATTSLYSAWDK_2

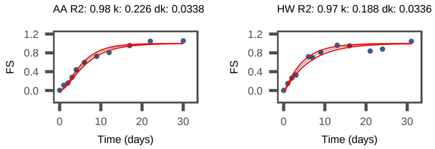

SCP2 − ADCTITMADSDLLALMTGK_2

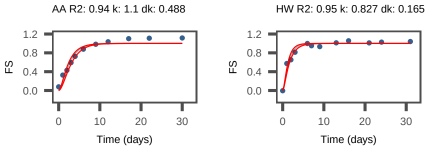

SCP2 − MNPQSAFFQGK_2

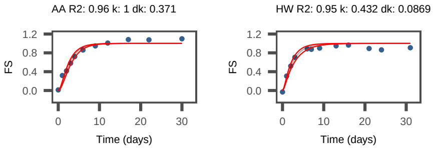

SBP1 − NAEGTWSVEK_2

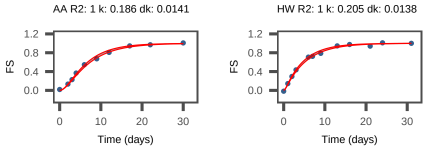

SCP2 − ADCTITMADSDLLALMTGK_3

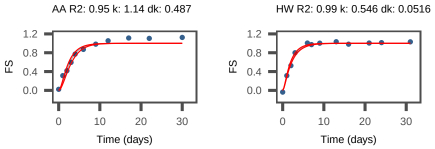

SCP2 − QLIQGGLANCVLALGFEK_2

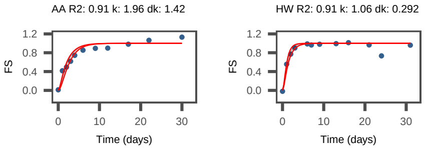

SBP1(Non−Unique) − NTGTEAPDYLATVDVDPK_2

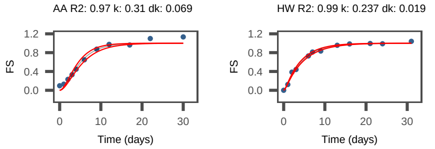

SCP2 − EATWVVDVK_2

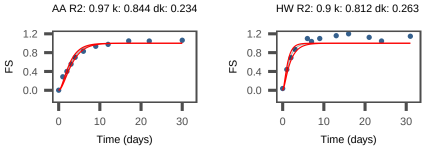

SCP2 − THQVSAAPTSSAGDGFK_2

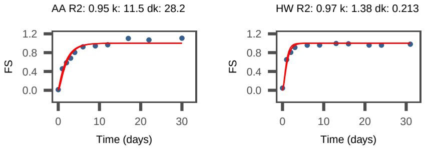

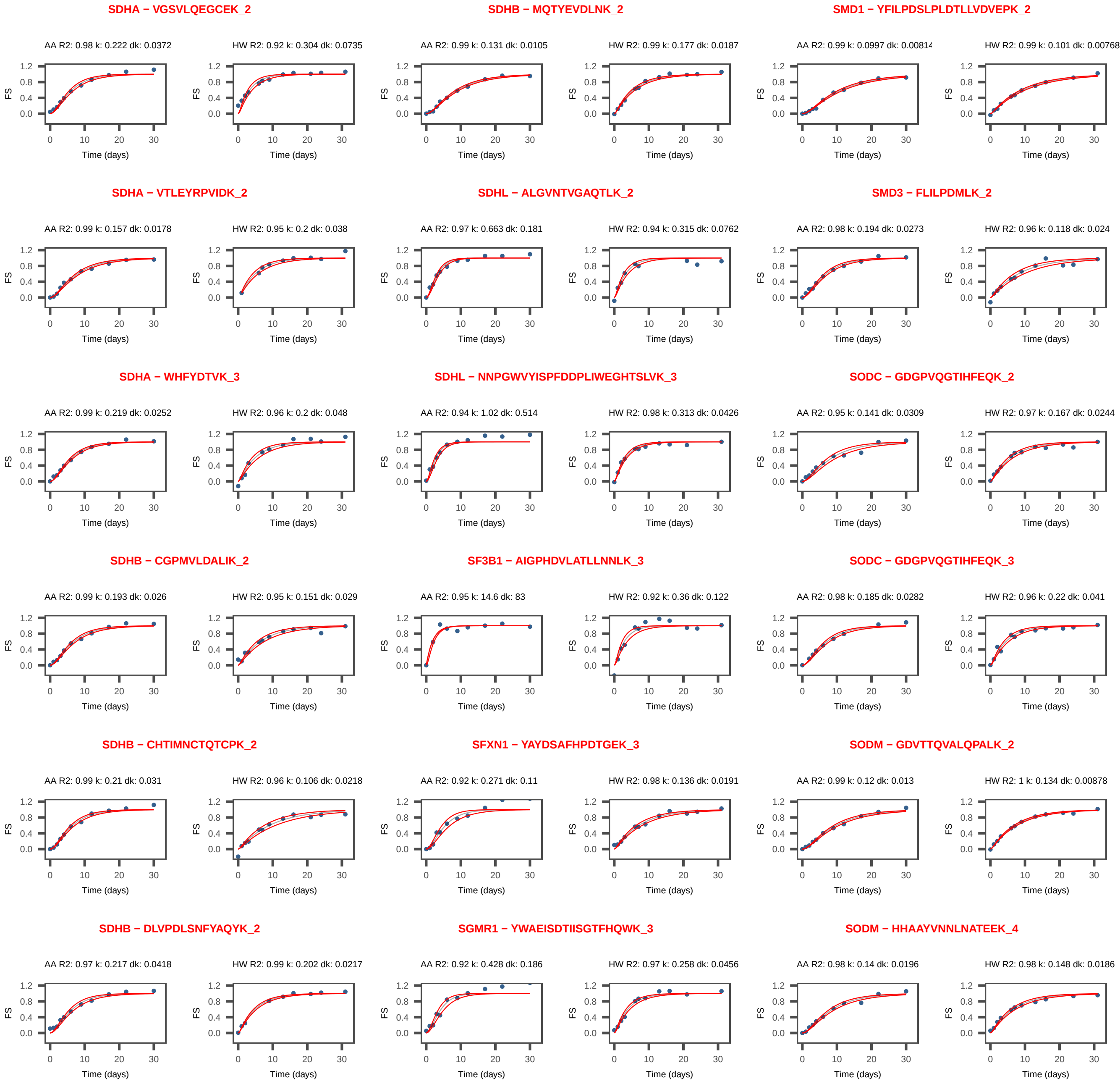
SDHA − VGSVLQEGCEK_2
AA R2: 0.98 k: 0.222 dk: 0.0372
HW R2: 0.92 k: 0.304 dk: 0.0735
SDHB − MQTYEVDLNK_2
AA R2: 0.99 k: 0.131 dk: 0.0105
HW R2: 0.99 k: 0.177 dk: 0.0187
SMD1 − YFILPDSLPLDTLLVDVEPK_2
AA R2: 0.99 k: 0.0997 dk: 0.00814
HW R2: 0.99 k: 0.101 dk: 0.00768
SDHA − VTLEYRPVIDK_2
AA R2: 0.99 k: 0.157 dk: 0.0178
HW R2: 0.95 k: 0.2 dk: 0.038
SDHL − ALGVNTVGAQTLK_2
AA R2: 0.97 k: 0.663 dk: 0.181
HW R2: 0.94 k: 0.315 dk: 0.0762
SMD3 − FLILPDMLK_2
AA R2: 0.98 k: 0.194 dk: 0.0273
HW R2: 0.96 k: 0.118 dk: 0.024
SDHA − WHFYDTVK_3
AA R2: 0.99 k: 0.219 dk: 0.0252
HW R2: 0.96 k: 0.2 dk: 0.048
SDHL − NNPGWVYISPFDDPLIWEGHTSLVK_3
AA R2: 0.94 k: 1.02 dk: 0.514
HW R2: 0.98 k: 0.313 dk: 0.0426
SODC − GDGPVQGTIHFEQK_2
AA R2: 0.95 k: 0.141 dk: 0.0309
HW R2: 0.97 k: 0.167 dk: 0.0244
SDHB − CGPMVLDALIK_2
AA R2: 0.99 k: 0.193 dk: 0.026
HW R2: 0.95 k: 0.151 dk: 0.029
SF3B1 − AIGPHDVLATLLNNLK_3
AA R2: 0.95 k: 14.6 dk: 83
HW R2: 0.92 k: 0.36 dk: 0.122
SODC − GDGPVQGTIHFEQK_3
AA R2: 0.98 k: 0.185 dk: 0.0282
HW R2: 0.96 k: 0.22 dk: 0.041
SDHB − CHTIMNCTQTCPK_2
AA R2: 0.99 k: 0.21 dk: 0.031
HW R2: 0.96 k: 0.106 dk: 0.0218
SFXN1 − YAYDSAFHPDTGEK_3
AA R2: 0.92 k: 0.271 dk: 0.11
HW R2: 0.98 k: 0.136 dk: 0.0191
SODM − GDVTTQVALQPALK_2
AA R2: 0.99 k: 0.12 dk: 0.013
HW R2: 1 k: 0.134 dk: 0.00878
SDHB − DLVPDLSNFYAQYK_2
AA R2: 0.97 k: 0.217 dk: 0.0418
HW R2: 0.99 k: 0.202 dk: 0.0217
SGMR1 − YWAEISDTIISGTFHQWK_3
AA R2: 0.92 k: 0.428 dk: 0.186
HW R2: 0.97 k: 0.258 dk: 0.0456
SODM − HHAAYVNNLNATEEK_4
AA R2: 0.98 k: 0.14 dk: 0.0196
HW R2: 0.98 k: 0.148 dk: 0.0186
FS
Time (days)

SPA3K(Non−Unique) − NIVFSPLSISAALALVSLGAK_2

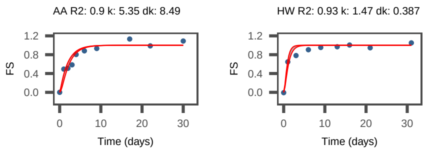

ST1A1 − IPFLEFSCPGVPPGLETLK_2

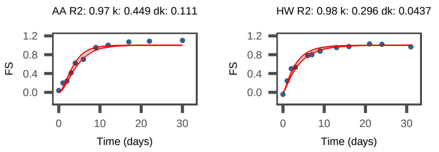

ST1D1 − THLPVQLLPSSFWK_3

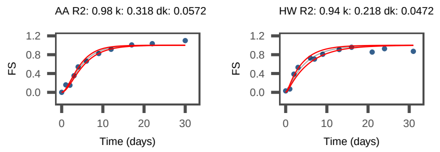

SPEB − EHGPVGLVHVGAHTNTTDKPR_4

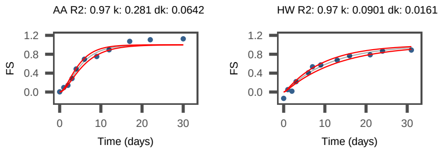

ST1A1 − LHPDPGTWESFLENFMDGK_3

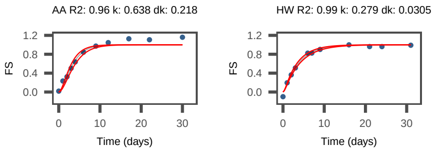

STA10 − AVSIQTGYLIQSTGPK_2

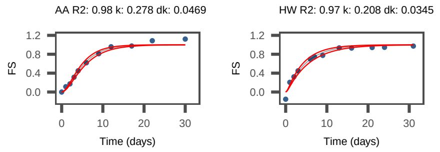

SPRE − LLLINNAATLGDVSK_2

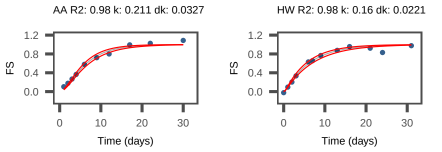

ST1A1 − THLPLSLLPQSLLDQK_2

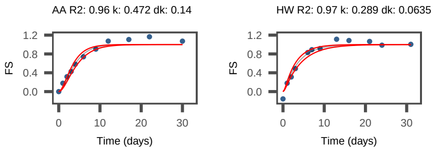

STIP1 − LLEFQLALK_2

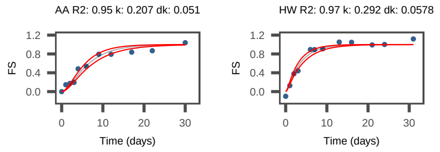

SPRE − SDGALVDCGTSAQK_2

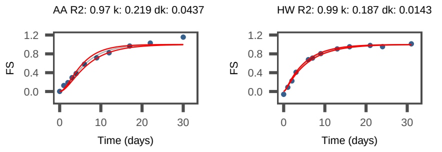

ST1A1 − THLPLSLLPQSLLDQK_3

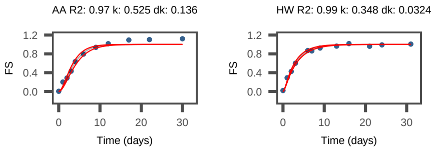

SUCA − AKPVVSFIAGITAPPGR_2

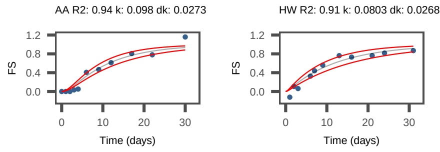

SPRE − TVVNISSLCALQPYK_2

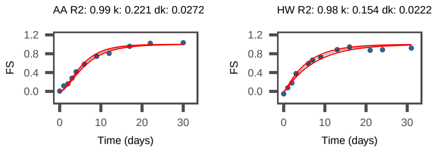

ST1D1 − ENPSANYTTMMK_2

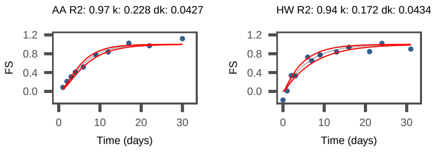

SUCA − AKPVVSFIAGITAPPGR_3

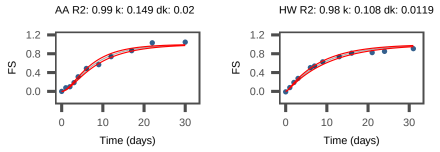

SSDH − ILLHHAANSVK_2

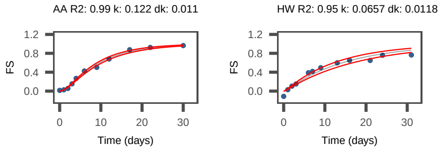

ST1D1 − FMAGQVSFGPWYDHVK_3

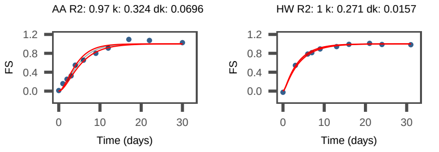

SUCA − HLGLPVFNTVK_2

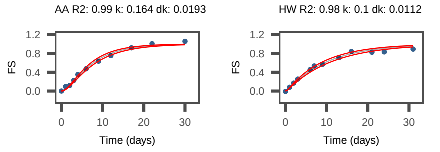

SUCA − IICQGFTGK_2

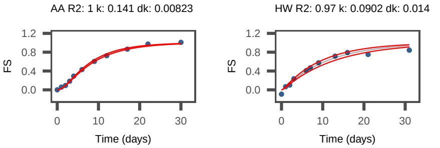

SUCB1 − MGFPSNIVDSAAENMIK_2

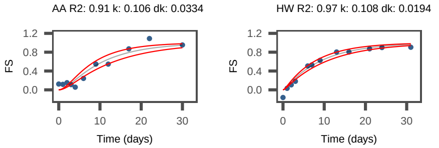

SYEP − SQGSGLSSGGAGEGQGPK_2

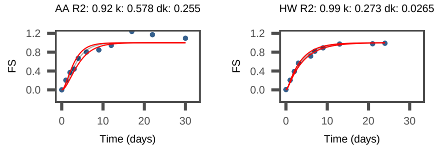

SUCA − ISALQSAGVVVSMSPAQLGTTIYK_2

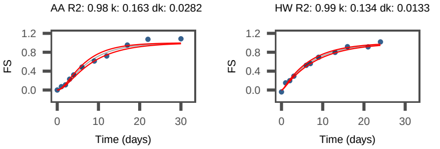

SUCB2 − EAQVYEAFK_2

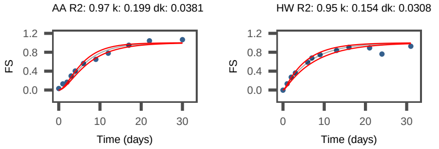

SYQ − NEVDMQVLHLLGPK_3

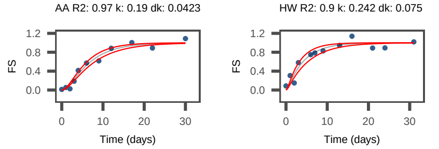

SUCA − LIGPNCPGVINPGECK_2

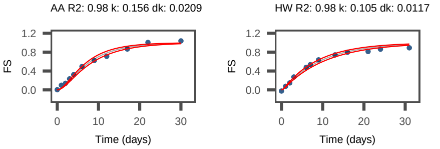

SUCB2 − IDATQVEVNPFGETPEGQVVCFDAK_2

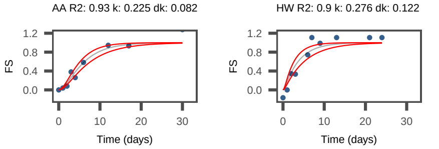

SYTC − DQELYFFHELSPGSCFFLPK_3

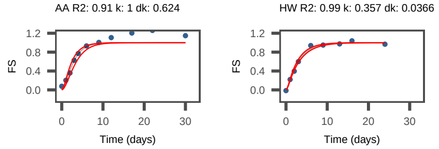

SUCA − MGHAGAIIAGGK_3

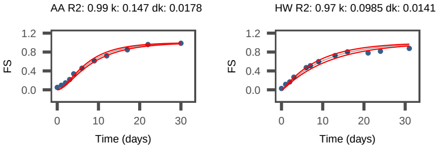

SUCB2 − KLMSEHGVR_3

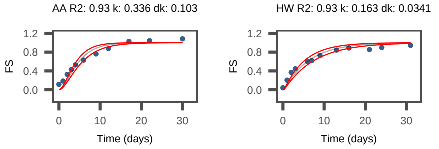

SYTC − FLGDIEIWNQAEK_2

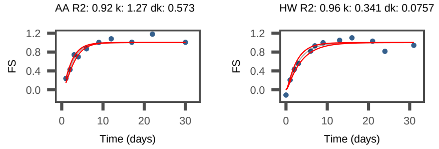

SUCA − QGTFHSQQALEYGTK_2

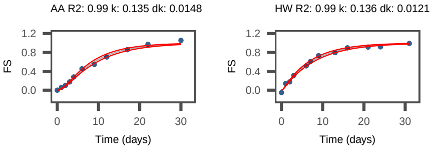

SUCB2 − VVGELAQQMIGYNLATK_2

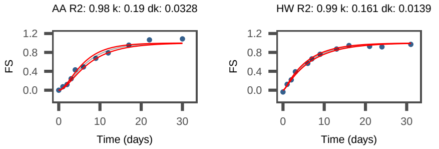

TADBP − TSDLIVLGLPWK_2

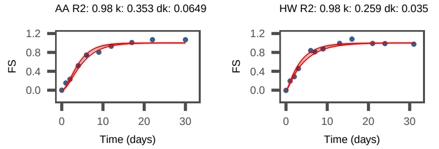

SUCA − QGTFHSQQALEYGTK_3

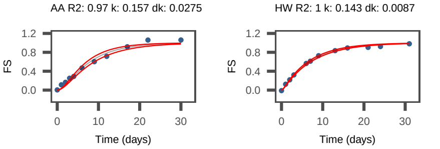

SUOX − LHVVGAPGGQSLSLSLDDLHK_4

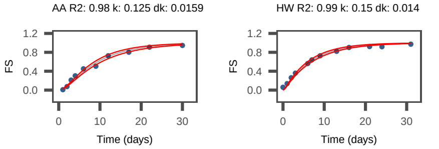

TALDO − LFVLFGAEILK_2

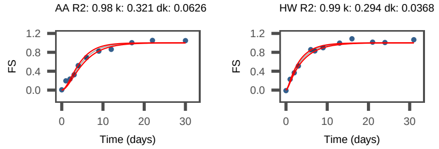

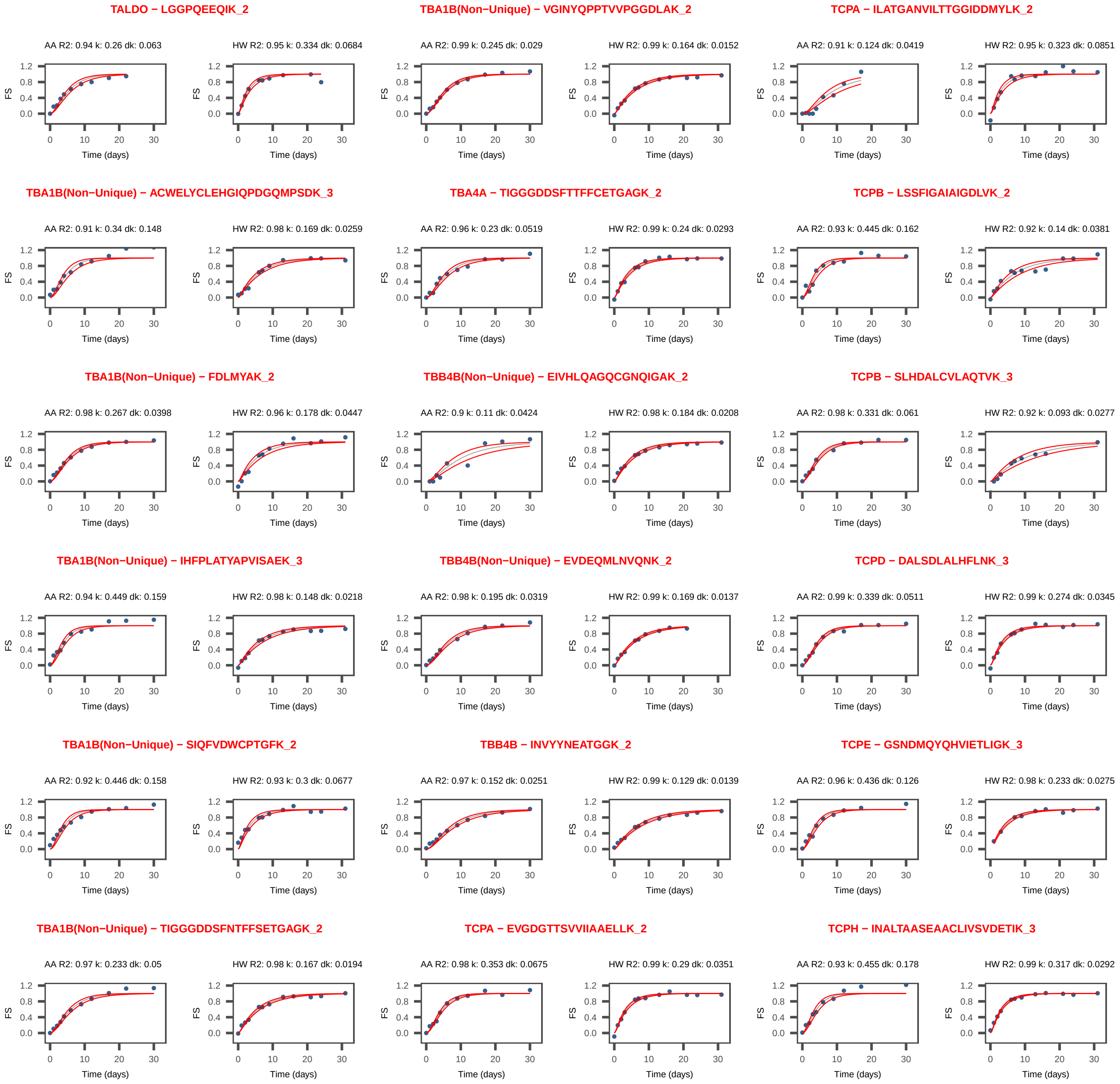
TALDO − LGGPQEEQIK_2
AA R2: 0.94 k: 0.26 dk: 0.063
HW R2: 0.95 k: 0.334 dk: 0.0684
TBA1B(Non−Unique) − VGINYQPPTVVPGGDLAK_2
AA R2: 0.99 k: 0.245 dk: 0.029
HW R2: 0.99 k: 0.164 dk: 0.0152
TCPA − ILATGANVILTTGGIDDMYLK_2
AA R2: 0.91 k: 0.124 dk: 0.0419
HW R2: 0.95 k: 0.323 dk: 0.0851
TBA1B(Non−Unique) − ACWELYCLEHGIQPDGQMPSDK_3
AA R2: 0.91 k: 0.34 dk: 0.148
HW R2: 0.98 k: 0.169 dk: 0.0259
TBA4A − TIGGGDDSFTTFFCETGAGK_2
AA R2: 0.96 k: 0.23 dk: 0.0519
HW R2: 0.99 k: 0.24 dk: 0.0293
TCPB − LSSFIGAIAIGDLVK_2
AA R2: 0.93 k: 0.445 dk: 0.162
HW R2: 0.92 k: 0.14 dk: 0.0381
TBA1B(Non−Unique) − FDLMYAK_2
AA R2: 0.98 k: 0.267 dk: 0.0398
HW R2: 0.96 k: 0.178 dk: 0.0447
TBB4B(Non−Unique) − EIVHLQAGQCGNQIGAK_2
AA R2: 0.9 k: 0.11 dk: 0.0424
HW R2: 0.98 k: 0.184 dk: 0.0208
TCPB − SLHDALCVLAQTVK_3
AA R2: 0.98 k: 0.331 dk: 0.061
HW R2: 0.92 k: 0.093 dk: 0.0277
TBA1B(Non−Unique) − IHFPLATYAPVISAEK_3
AA R2: 0.94 k: 0.449 dk: 0.159
HW R2: 0.98 k: 0.148 dk: 0.0218
TBB4B(Non−Unique) − EVDEQMLNVQNK_2
AA R2: 0.98 k: 0.195 dk: 0.0319
HW R2: 0.99 k: 0.169 dk: 0.0137
TCPD − DALSDLALHFLNK_3
AA R2: 0.99 k: 0.339 dk: 0.0511
HW R2: 0.99 k: 0.274 dk: 0.0345
TBA1B(Non−Unique) − SIQFVDWCPTGFK_2
AA R2: 0.92 k: 0.446 dk: 0.158
HW R2: 0.93 k: 0.3 dk: 0.0677
TBB4B − INVYYNEATGGK_2
AA R2: 0.97 k: 0.152 dk: 0.0251
HW R2: 0.99 k: 0.129 dk: 0.0139
TCPE − GSNDMQYQHVIETLIGK_3
AA R2: 0.96 k: 0.436 dk: 0.126
HW R2: 0.98 k: 0.233 dk: 0.0275
TBA1B(Non−Unique) − TIGGGDDSFNTFFSETGAGK_2
AA R2: 0.97 k: 0.233 dk: 0.05
HW R2: 0.98 k: 0.167 dk: 0.0194
TCPA − EVGDGTTSVVIIAAELLK_2
AA R2: 0.98 k: 0.353 dk: 0.0675
HW R2: 0.99 k: 0.29 dk: 0.0351
TCPH − INALTAASEAACLIVSVDETIK_3
AA R2: 0.93 k: 0.455 dk: 0.178
HW R2: 0.99 k: 0.317 dk: 0.0292
FS
Time (days)

TCPQ − QITSYGETCPGLEQYAIK_2

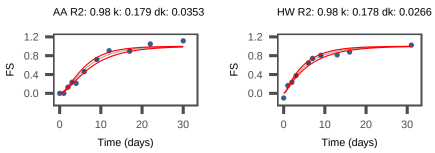

TERA − LEILQIHTK_3

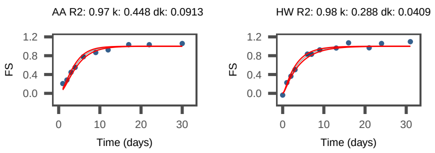

THIC − IVSWSQAGVEPSVMGVGPIPAIK_3

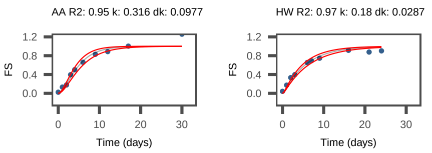

TCPZ − AQLGVQAFADALLIIPK_2

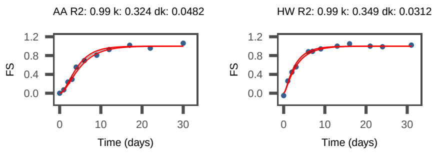

TERA − NAPAIIFIDELDAIAPK_2

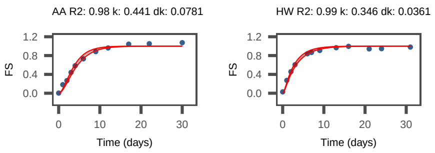

THIKB − DTTPDELLSAVLTAVLQDVK_2

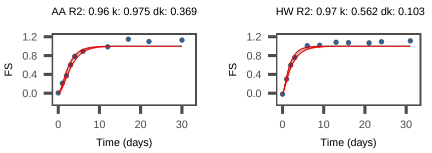

TERA − AVANETGAFFFLINGPEIMSK_3

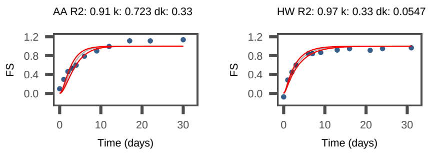

TERA − QAAPCVLFFDELDSIAK_2

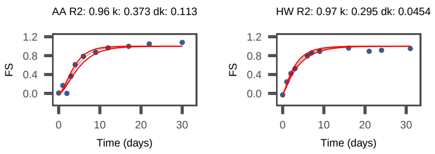

THIKB − DTTPDELLSAVLTAVLQDVK_3

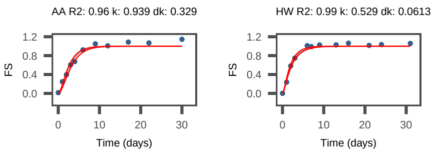

TERA(Non−Unique) − GILLYGPPGTGK_2

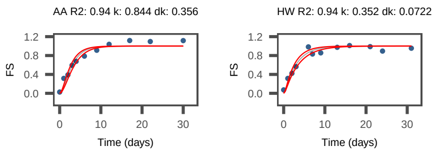

TERA − QAAPCVLFFDELDSIAK_3

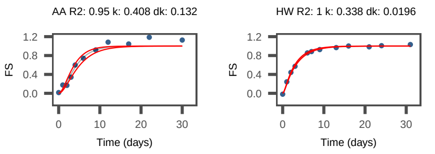

THIKB − GCFHAEIVPVTTTVLNDK_2

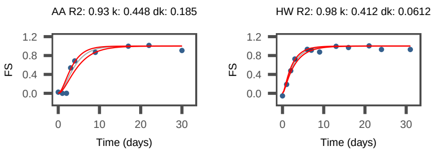

TERA − IVSQLLTLMDGLK_2

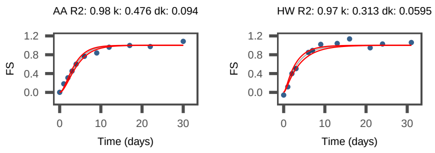

TGM2 − LAEKEETGVAMR_2

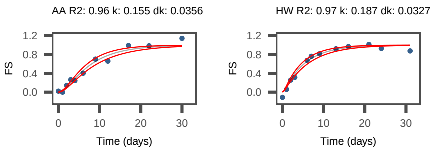

THIKB(Non−Unique) − QVVTLLNELK_2

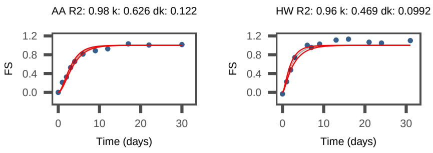

TERA − IVSQLLTLMDGLK_3

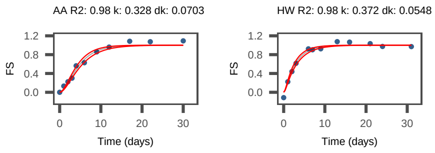

TGM2 − VDLFPTDIGLHK_3

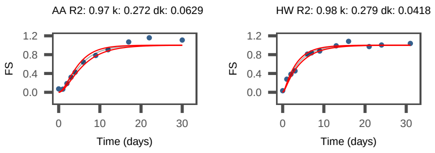

THIKB(Non−Unique) − SKAEELGLPILGVLR_3

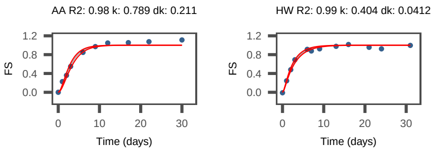

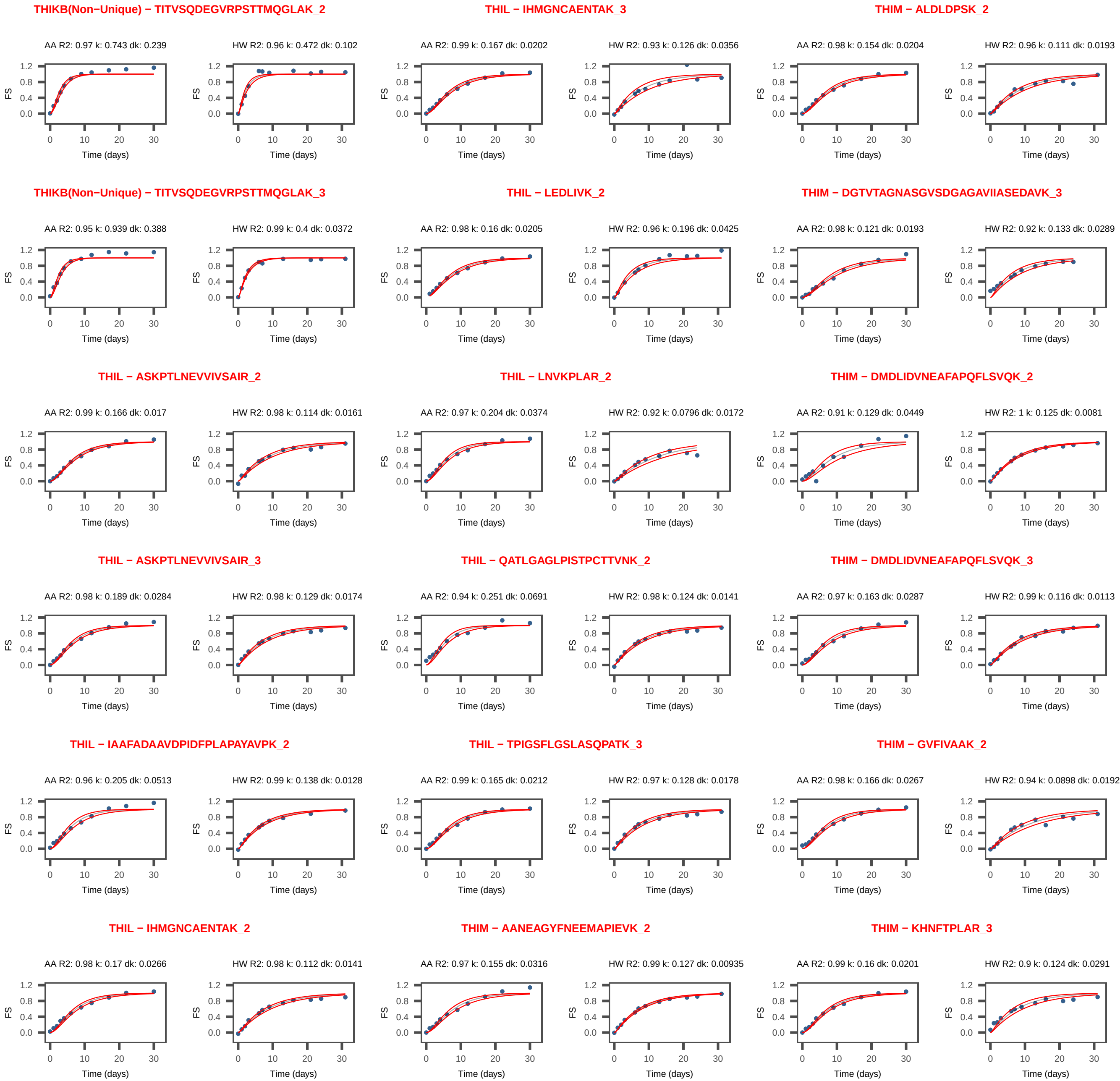
THIKB(Non−Unique) − TITVSQDEGVRPSTTMQGLAK_2
AA R2: 0.97 k: 0.743 dk: 0.239
HW R2: 0.96 k: 0.472 dk: 0.102
THIL − IHMGNCAENTAK_3
AA R2: 0.99 k: 0.167 dk: 0.0202
HW R2: 0.93 k: 0.126 dk: 0.0356
THIM − ALDLDPSK_2
AA R2: 0.98 k: 0.154 dk: 0.0204
HW R2: 0.96 k: 0.111 dk: 0.0193
THIKB(Non−Unique) − TITVSQDEGVRPSTTMQGLAK_3
AA R2: 0.95 k: 0.939 dk: 0.388
HW R2: 0.99 k: 0.4 dk: 0.0372
THIL − LEDLIVK_2
AA R2: 0.98 k: 0.16 dk: 0.0205
HW R2: 0.96 k: 0.196 dk: 0.0425
THIM − DGTVTAGNASGVSDGAGAVIIASEDAVK_3
AA R2: 0.98 k: 0.121 dk: 0.0193
HW R2: 0.92 k: 0.133 dk: 0.0289
THIL − ASKPTLNEVVIVSAIR_2
AA R2: 0.99 k: 0.166 dk: 0.017
HW R2: 0.98 k: 0.114 dk: 0.0161
THIL − LNVKPLAR_2
AA R2: 0.97 k: 0.204 dk: 0.0374
HW R2: 0.92 k: 0.0796 dk: 0.0172
THIM − DMDLIDVNEAFAPQFLSVQK_2
AA R2: 0.91 k: 0.129 dk: 0.0449
HW R2: 1 k: 0.125 dk: 0.0081
THIL − ASKPTLNEVVIVSAIR_3
AA R2: 0.98 k: 0.189 dk: 0.0284
HW R2: 0.98 k: 0.129 dk: 0.0174
THIL − QATLGAGLPISTPCTTVNK_2
AA R2: 0.94 k: 0.251 dk: 0.0691
HW R2: 0.98 k: 0.124 dk: 0.0141
THIM − DMDLIDVNEAFAPQFLSVQK_3
AA R2: 0.97 k: 0.163 dk: 0.0287
HW R2: 0.99 k: 0.116 dk: 0.0113
THIL − IAAFADAAVDPIDFPLAPAYAVPK_2
AA R2: 0.96 k: 0.205 dk: 0.0513
HW R2: 0.99 k: 0.138 dk: 0.0128
THIL − TPIGSFLGSLASQPATK_3
AA R2: 0.99 k: 0.165 dk: 0.0212
HW R2: 0.97 k: 0.128 dk: 0.0178
THIM − GVFIVAAK_2
AA R2: 0.98 k: 0.166 dk: 0.0267
HW R2: 0.94 k: 0.0898 dk: 0.0192
THIL − IHMGNCAENTAK_2
AA R2: 0.98 k: 0.17 dk: 0.0266
HW R2: 0.98 k: 0.112 dk: 0.0141
THIM − AANEAGYFNEEMAPIEVK_2
AA R2: 0.97 k: 0.155 dk: 0.0316
HW R2: 0.99 k: 0.127 dk: 0.00935
THIM − KHNFTPLAR_3
AA R2: 0.99 k: 0.16 dk: 0.0201
HW R2: 0.9 k: 0.124 dk: 0.0291
FS
Time (days)

THIM − LCGSGFQSIVSGCQEICSK_2

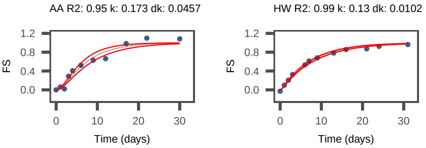

THIO − EAFQEALAAAGDK_2

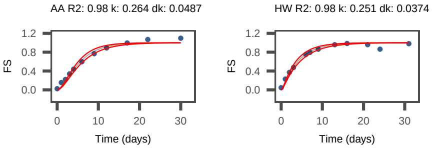

TKFC − AAPTEPPEAPEATAAGGVTSK_2

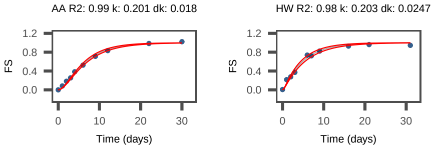

THIM − LCGSGFQSIVSGCQEICSK_3

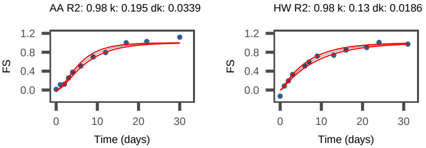

THTR − ATLNLSLLK_2

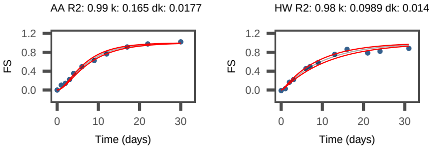

TKFC − AVAQAGTVGTLLIVK_2

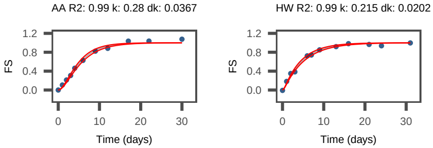

THIM − LEDTLWAGLTDQHVK_3

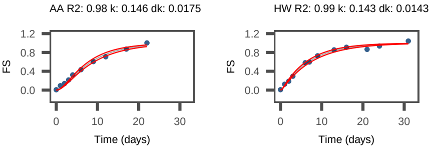

THTR − EGHPVTSEPSRPEPAVFK_3

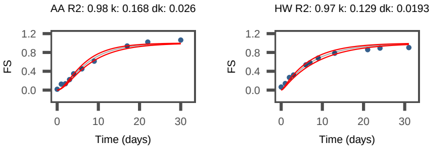

TKFC − MGGSSGALYGLFLTAAAQPLK_2

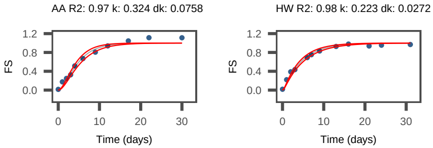

THIM − LPM[15.9949]GMTAENLAAK_2

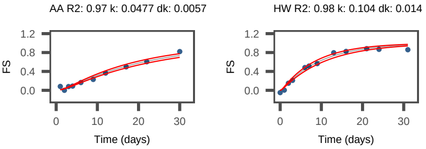

THTR − GSVNMPFMDFLTK_2

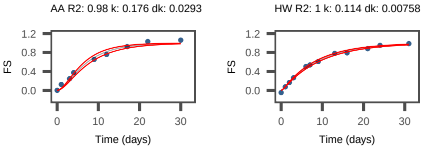

TKFC − MGGSSGALYGLFLTAAAQPLK_3

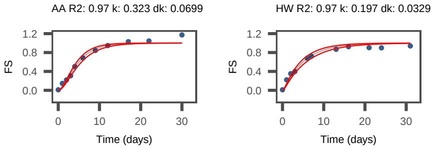

THIM − LPMGMTAENLAAK_2

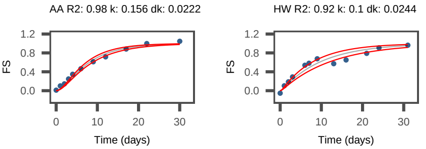

THTR − LCGKPDVAVYDGSWSEWFR_3

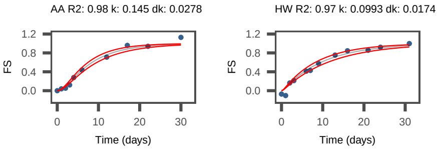

TKFC − MSALEMPGVSLTLMLVDEPVLK_2

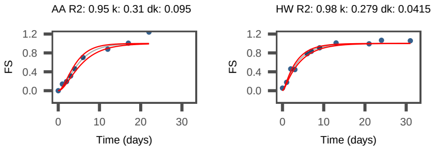

THIM − PQTTLEQLQK_2

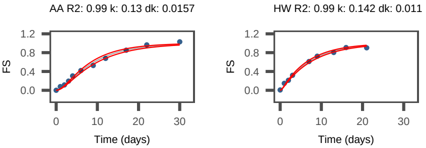

TIM50 − VLLDLSAFLK_2

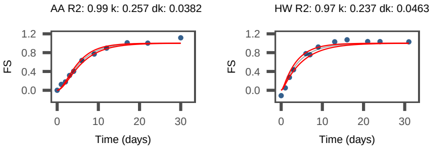

TKFC − SPGASLLPVLTK_2

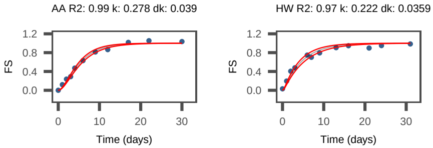

TKFC – TMLDSLWAAAQEFQAWK_2

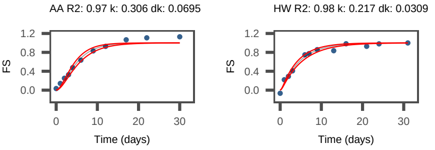

TKT – LGQSDPAPLQHQVDIYQK_3

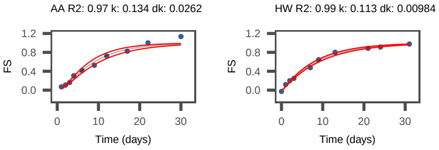

TPIS – HVFGESDELIGQK_3

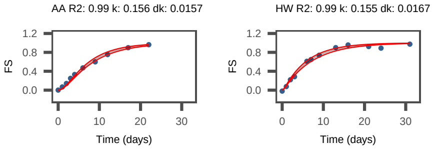

TKFC – TMLDSLWAAAQEFQAWK_3

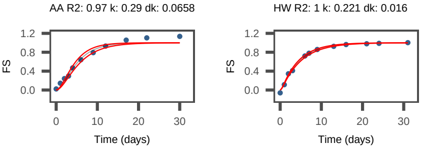

TKT – MPTPPSYK_2

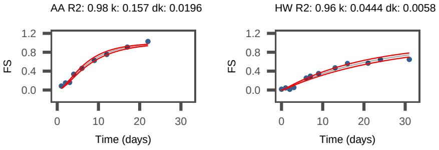

TPIS – IAVAAQNCYK_2

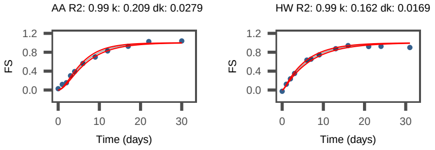

TKFC – VALLSGGGSGHEPAHAGFIGK_2

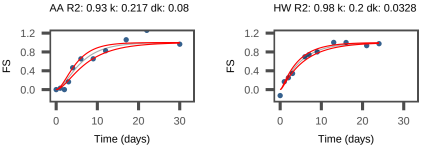

TKT – NSTFSELFK_2

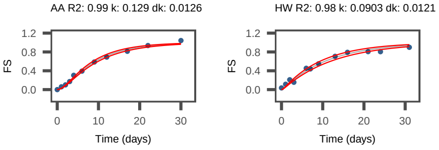

TPIS – TATPQQAQEVHEK_2

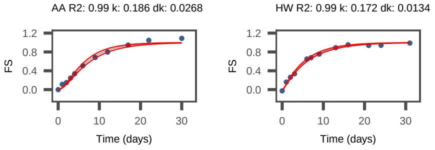

TKFC – VALLSGGGSGHEPAHAGFIGK_3

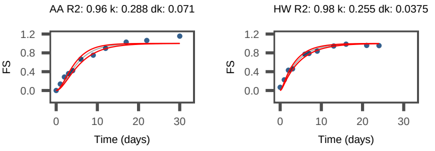

TKT – TSRPENAIIYSNNEDFQVGQAK_3

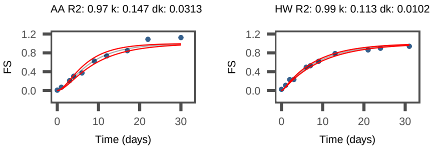

TPIS – TATPQQAQEVHEK_3

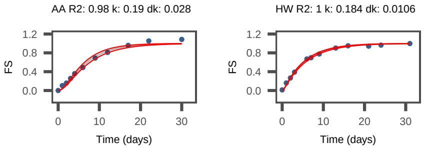

TKT(Non–Unique) – AYGLALAK_2

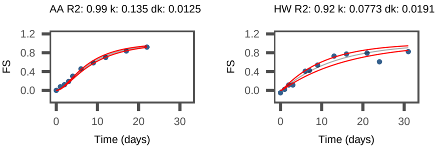

TLN1 – GVGAAATAVTQALNELLQHVK_3

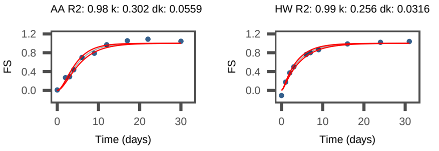

TPIS – VSHALAEGLGVIACIGEK_3

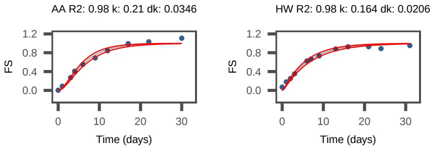

TKT – HQPTAIIAK_2

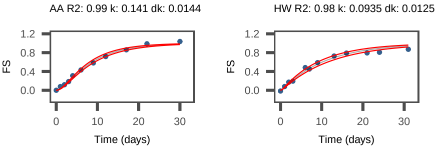

TPIS – HVFGESDELIGQK_2

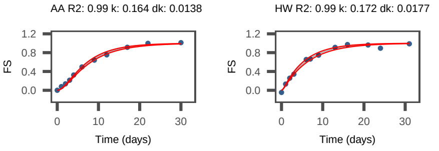

TPIS – VVLAYEPVWAIGTGK_3

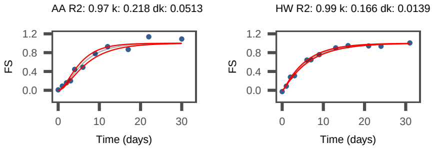

TPMT – AWGLDYLFEK_2

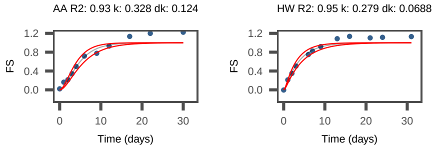

TTPA – EFSMEDICQEWTNFIMK_2

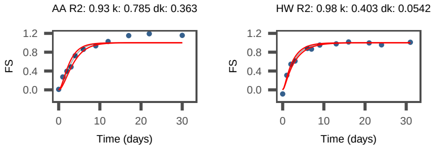

UBA1 – IIPAIATTTAAVVGLVCLELYK_3

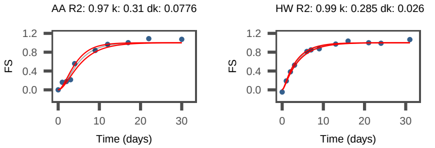

TRAP1 – INTLQAIWMMDPK_2

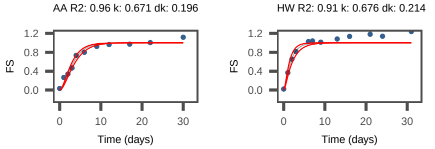

TXND5 – SFEDTIAQGITFVK_2

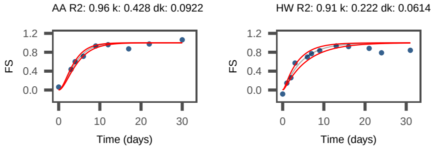

UBA1 – QPAENVNQYLTDSK_2

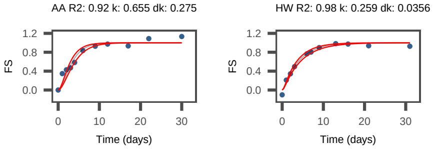

TRAP1 – YIAQAYDKPR_2

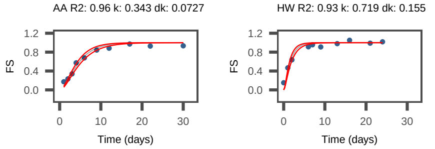

TXTP – FIHDQTSSNPK_2

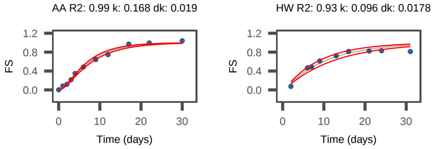

UBB(Non–Unique) – TLSDYNIQK_2

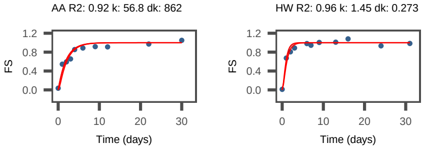

TRFE – KPVDQYEDCYLAR_2

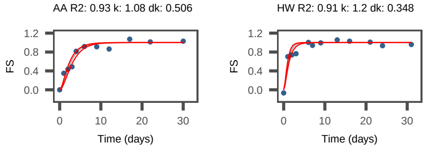

TXTP – FIHDQTSSNPK_3

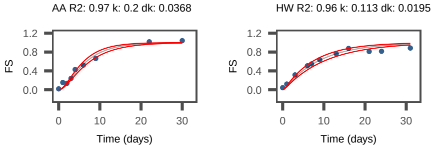

UBE2N – YFHVVIAGPQDSPFEGGTFK_3

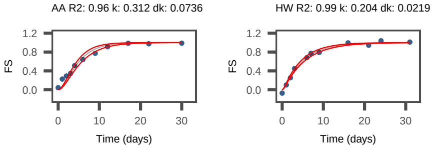

TTC38 – DALKPNSPLTER_2

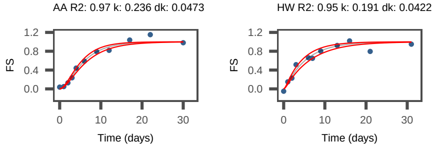

UBA1 – AAVASLLQSVQVPEFTPK_2

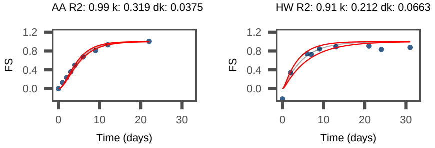

UCRI – KGPAPLNLEVPAYEFTSDDVVVVG_2

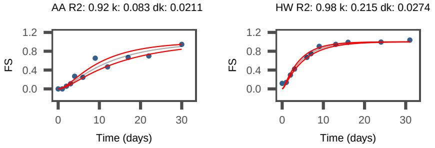

TTC38 – NAGLPLSTTSNEACK_2

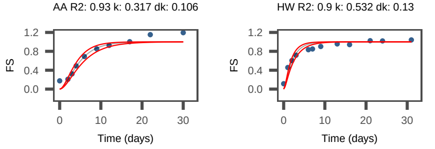

UBA1 – AAVASLLQSVQVPEFTPK_3

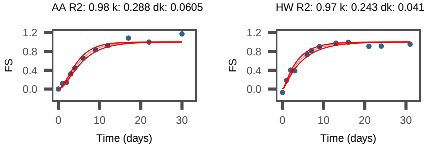

UD11 – LPCSLDSEATQCPVPLSYVPK_2

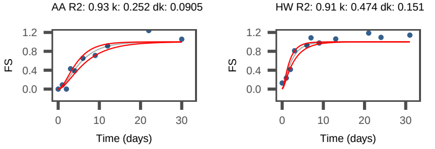

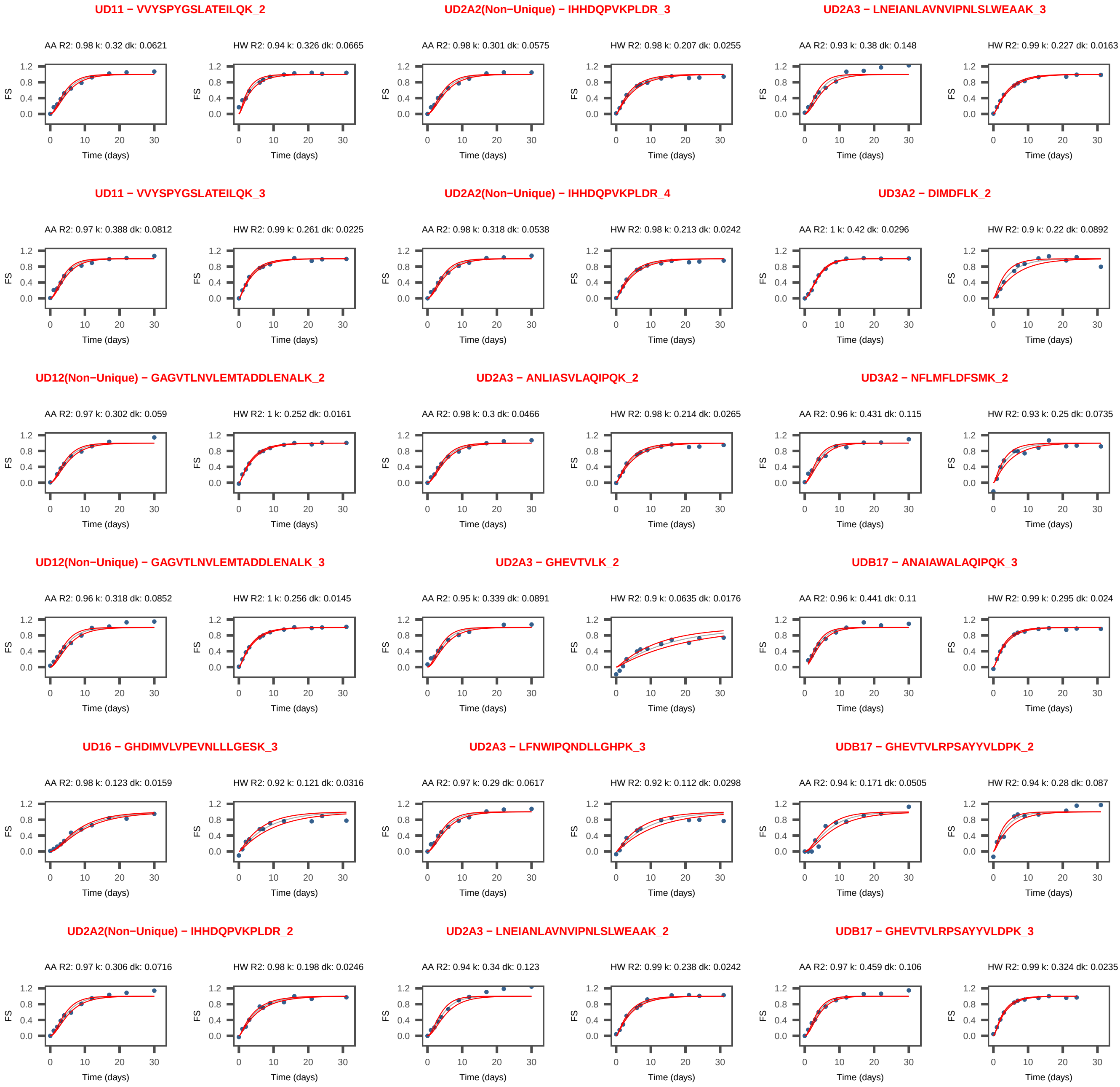
UD11 − VVYSPYGSLATEILQK_2
AA R2: 0.98 k: 0.32 dk: 0.0621
HW R2: 0.94 k: 0.326 dk: 0.0665
UD2A2(Non−Unique) − IHHDQPVKPLDR_3
AA R2: 0.98 k: 0.301 dk: 0.0575
HW R2: 0.98 k: 0.207 dk: 0.0255
UD2A3 − LNEIANLAVNVIPNLSLWEAAK_3
AA R2: 0.93 k: 0.38 dk: 0.148
HW R2: 0.99 k: 0.227 dk: 0.0163
UD11 − VVYSPYGSLATEILQK_3
AA R2: 0.97 k: 0.388 dk: 0.0812
HW R2: 0.99 k: 0.261 dk: 0.0225
UD2A2(Non−Unique) − IHHDQPVKPLDR_4
AA R2: 0.98 k: 0.318 dk: 0.0538
HW R2: 0.98 k: 0.213 dk: 0.0242
UD3A2 − DIMDFLK_2
AA R2: 1 k: 0.42 dk: 0.0296
HW R2: 0.9 k: 0.22 dk: 0.0892
UD12(Non−Unique) − GAGVTLNVLEMTADDLENALK_2
AA R2: 0.97 k: 0.302 dk: 0.059
HW R2: 1 k: 0.252 dk: 0.0161
UD2A3 − ANLIASVLAQIPQK_2
AA R2: 0.98 k: 0.3 dk: 0.0466
HW R2: 0.98 k: 0.214 dk: 0.0265
UD3A2 − NFLMFLDFSMK_2
AA R2: 0.96 k: 0.431 dk: 0.115
HW R2: 0.93 k: 0.25 dk: 0.0735
UD12(Non−Unique) − GAGVTLNVLEMTADDLENALK_3
AA R2: 0.96 k: 0.318 dk: 0.0852
HW R2: 1 k: 0.256 dk: 0.0145
UD2A3 − GHEVTVLK_2
AA R2: 0.95 k: 0.339 dk: 0.0891
HW R2: 0.9 k: 0.0635 dk: 0.0176
UDB17 − ANAIAWALAQIPQK_3
AA R2: 0.96 k: 0.441 dk: 0.11
HW R2: 0.99 k: 0.295 dk: 0.024
UD16 − GHDIMVLVPEVNLLLGESK_3
AA R2: 0.98 k: 0.123 dk: 0.0159
HW R2: 0.92 k: 0.121 dk: 0.0316
UD2A3 − LFNWIPQNDLLGHPK_3
AA R2: 0.97 k: 0.29 dk: 0.0617
HW R2: 0.92 k: 0.112 dk: 0.0298
UDB17 − GHEVTVLRPSAYYVLDPK_2
AA R2: 0.94 k: 0.171 dk: 0.0505
HW R2: 0.94 k: 0.28 dk: 0.087
UD2A2(Non−Unique) − IHHDQPVKPLDR_2
AA R2: 0.97 k: 0.306 dk: 0.0716
HW R2: 0.98 k: 0.198 dk: 0.0246
UD2A3 − LNEIANLAVNVIPNLSLWEAAK_2
AA R2: 0.94 k: 0.34 dk: 0.123
HW R2: 0.99 k: 0.238 dk: 0.0242
UDB17 − GHEVTVLRPSAYYVLDPK_3
AA R2: 0.97 k: 0.459 dk: 0.106
HW R2: 0.99 k: 0.324 dk: 0.0235
FS
Time (days)

UDB17 − GHEVTVLRPSAYYVLDPK_4

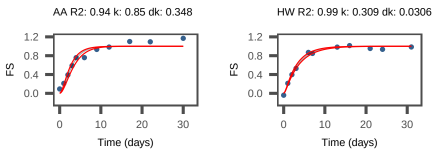

UDB17 − SDVLNALEEVIENPFYK_3

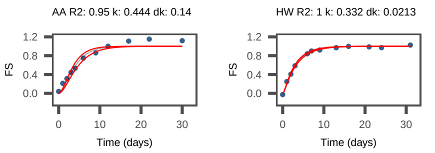

UGDH − ILTTNTWSSELSK_2

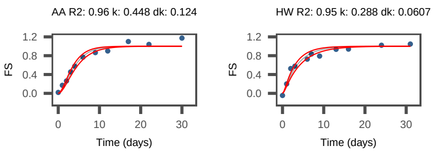

UDB17 − IHHDQPM[15.9949]KPLDR_4

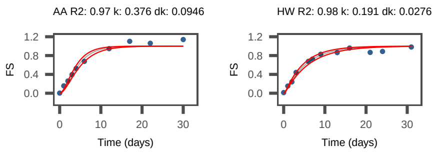

UDB17 − SNWDLEFPHPTLPNVDYVGGLHCK_4

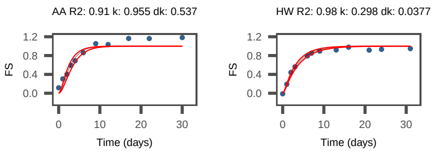

UGDH − INAWNSPTLPIYEPGLK_2

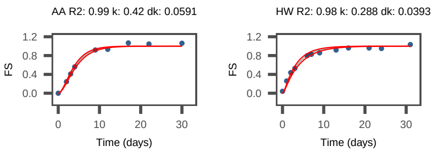

UDB17 − IHHDQPMKPLDR_2

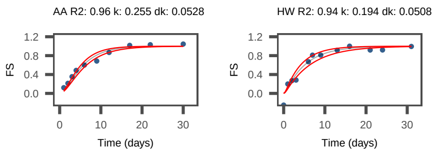

UDB17 − VLVWPMEFSHWMNIK_3

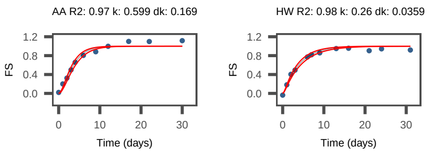

UGPA − EFPTVPLVK_2

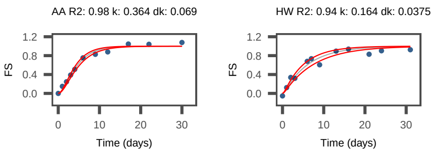

UDB17 − IHHDQPMKPLDR_3

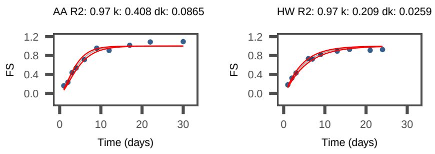

UGDH − ALVICTEWDMFK_2

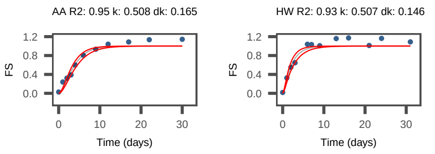

UGPA − ILTTAASHEFEHTK_3

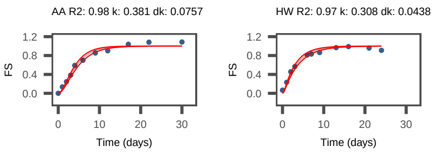

UDB17 − IHHDQPMKPLDR_4

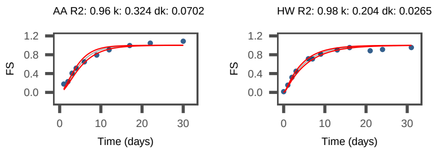

UGDH − ASVGFGGSCFQK_2

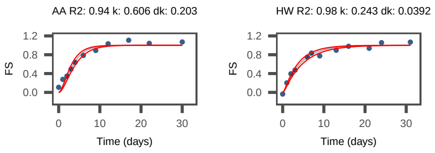

UGPA − IQRPPEDSIQPYEK_3

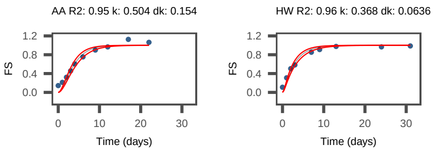

UDB17 − KWDSFYSEYLGR_3

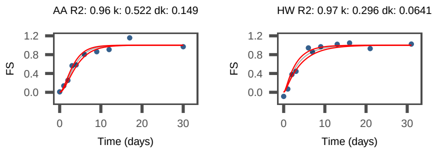

UGDH − IAILGFAFK_2

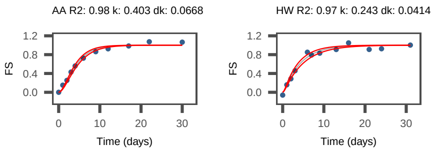

UGPA − LVEIAQVPK_2

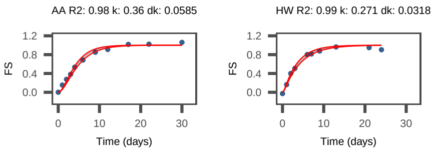

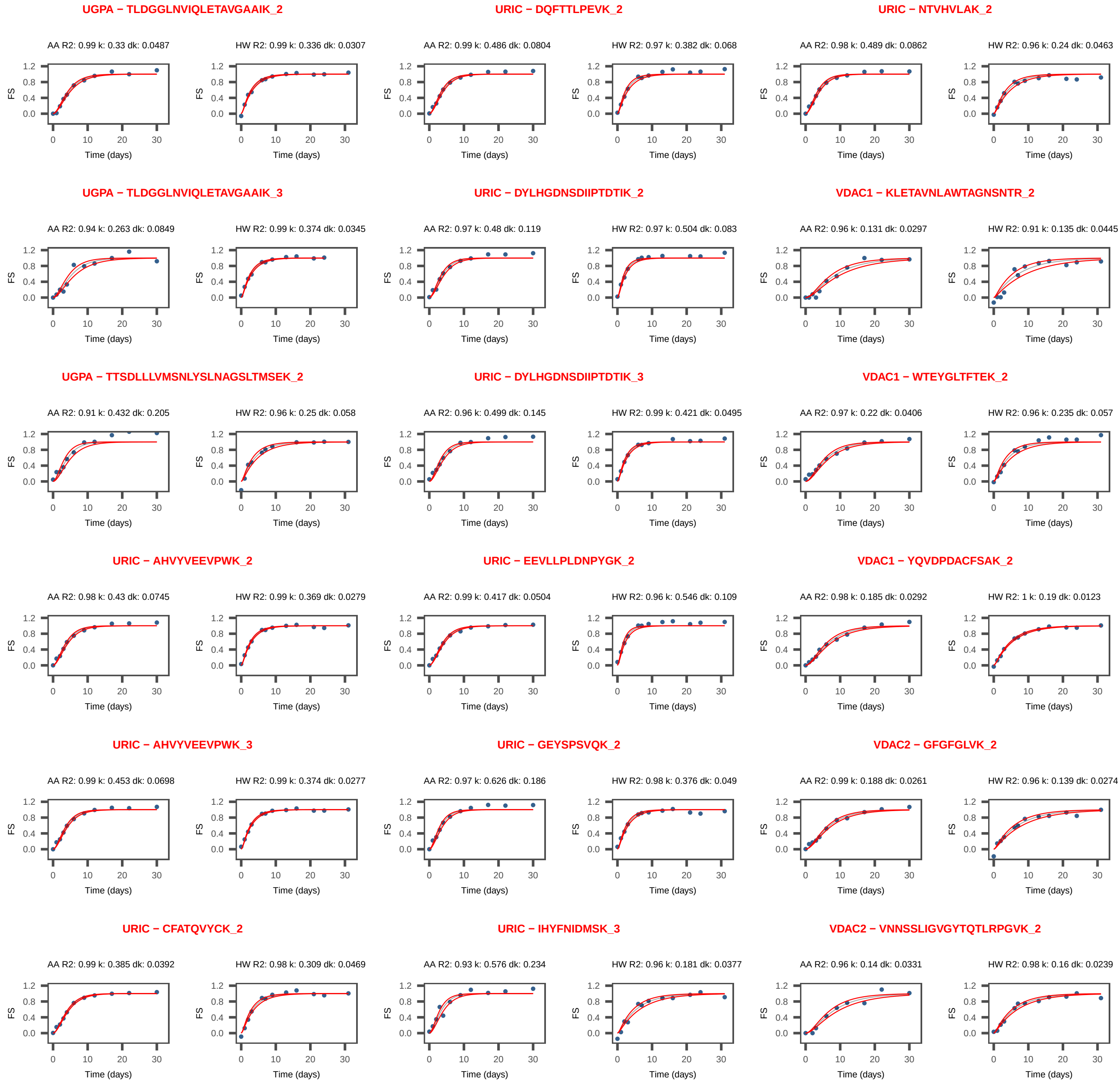
UGPA − TLDGGLNVIQLETAVGAAIK_2
AA R2: 0.99 k: 0.33 dk: 0.0487
HW R2: 0.99 k: 0.336 dk: 0.0307
URIC − DQFTTLPEVK_2
AA R2: 0.99 k: 0.486 dk: 0.0804
HW R2: 0.97 k: 0.382 dk: 0.068
URIC − NTVHVLAK_2
AA R2: 0.98 k: 0.489 dk: 0.0862
HW R2: 0.96 k: 0.24 dk: 0.0463
UGPA − TLDGGLNVIQLETAVGAAIK_3
AA R2: 0.94 k: 0.263 dk: 0.0849
HW R2: 0.99 k: 0.374 dk: 0.0345
URIC − DYLHGDNSDIIPTDTIK_2
AA R2: 0.97 k: 0.48 dk: 0.119
HW R2: 0.97 k: 0.504 dk: 0.083
VDAC1 − KLETAVNLAWTAGNSNTR_2
AA R2: 0.96 k: 0.131 dk: 0.0297
HW R2: 0.91 k: 0.135 dk: 0.0445
UGPA − TTSDLLLVMSNLYSLNAGSLTMSEK_2
AA R2: 0.91 k: 0.432 dk: 0.205
HW R2: 0.96 k: 0.25 dk: 0.058
URIC − DYLHGDNSDIIPTDTIK_3
AA R2: 0.96 k: 0.499 dk: 0.145
HW R2: 0.99 k: 0.421 dk: 0.0495
VDAC1 − WTEYGLTFTEK_2
AA R2: 0.97 k: 0.22 dk: 0.0406
HW R2: 0.96 k: 0.235 dk: 0.057
URIC − AHVYVEEVPWK_2
AA R2: 0.98 k: 0.43 dk: 0.0745
HW R2: 0.99 k: 0.369 dk: 0.0279
URIC − EEVLLPLDNPYGK_2
AA R2: 0.99 k: 0.417 dk: 0.0504
HW R2: 0.96 k: 0.546 dk: 0.109
VDAC1 − YQVDPDACFSAK_2
AA R2: 0.98 k: 0.185 dk: 0.0292
HW R2: 1 k: 0.19 dk: 0.0123
URIC − AHVYVEEVPWK_3
AA R2: 0.99 k: 0.453 dk: 0.0698
HW R2: 0.99 k: 0.374 dk: 0.0277
URIC − GEYSPSVQK_2
AA R2: 0.97 k: 0.626 dk: 0.186
HW R2: 0.98 k: 0.376 dk: 0.049
VDAC2 − GFGFGLVK_2
AA R2: 0.99 k: 0.188 dk: 0.0261
HW R2: 0.96 k: 0.139 dk: 0.0274
URIC − CFATQVYCK_2
AA R2: 0.99 k: 0.385 dk: 0.0392
HW R2: 0.98 k: 0.309 dk: 0.0469
URIC − IHYFNIDMSK_3
AA R2: 0.93 k: 0.576 dk: 0.234
HW R2: 0.96 k: 0.181 dk: 0.0377
VDAC2 − VNNSSLIGVGYTQTLRPGVK_2
AA R2: 0.96 k: 0.14 dk: 0.0331
HW R2: 0.98 k: 0.16 dk: 0.0239
FS
Time (days)
0
10
20
30
0.0
0.4
0.8
1.2

VDAC2 − WCEYGLTFTEK_2

AA R2: 0.98 k: 0.23 dk: 0.039 | HW R2: 0.97 k: 0.208 dk: 0.042

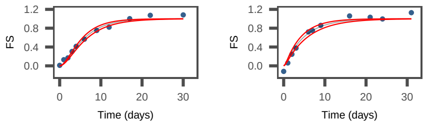

VINC − QVATALQNLQTK_2

AA R2: 0.99 k: 0.142 dk: 0.0161 | HW R2: 0.91 k: 0.257 dk: 0.0668

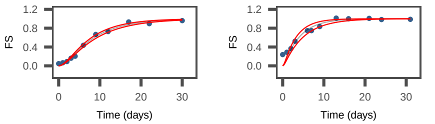

VDAC3(Non−Unique) − LTLSALIDGK_2

AA R2: 0.98 k: 0.214 dk: 0.0354 | HW R2: 0.97 k: 0.198 dk: 0.035

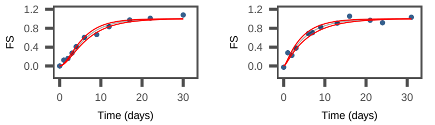

WDR1 − YAPSGFYIASGDISGK_2

AA R2: 0.93 k: 0.157 dk: 0.0468 | HW R2: 0.94 k: 0.163 dk: 0.0357

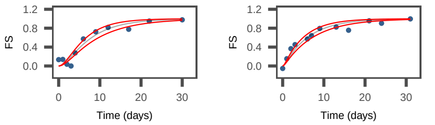

VDAC3 − SCSGVEFSTSGHAYTDTGK_3

AA R2: 0.9 k: 0.0959 dk: 0.0244 | HW R2: 0.98 k: 0.191 dk: 0.0259

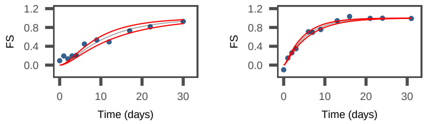

XDH − AVGEPPLFLASSIFFAIK_2

AA R2: 0.98 k: 0.341 dk: 0.0675 | HW R2: 0.97 k: 0.169 dk: 0.0302

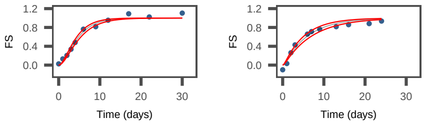

VDAC3 − WNTDNTLGTEISWENK_2

AA R2: 0.98 k: 0.188 dk: 0.0306 | HW R2: 0.98 k: 0.181 dk: 0.0261

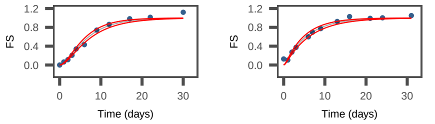

XDH − ITYEDLPAIITIQDAIK_2

AA R2: 0.96 k: 0.196 dk: 0.0462 | HW R2: 0.98 k: 0.32 dk: 0.0394

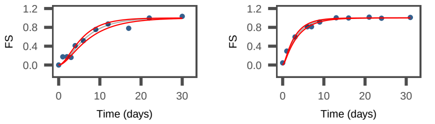

VIGLN − AACLESAQEPAGAWSNK_2

AA R2: 0.93 k: 1.09 dk: 0.598 | HW R2: 0.99 k: 0.682 dk: 0.0706

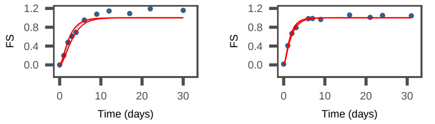

XDH − ITYEDLPAIITIQDAIK_3

AA R2: 0.95 k: 0.197 dk: 0.0545 | HW R2: 0.99 k: 0.299 dk: 0.0333

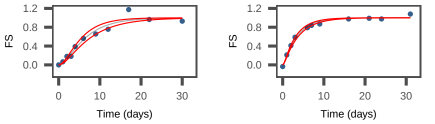

VIGLN − IEGDPQGVQQAK_2

AA R2: 0.98 k: 0.793 dk: 0.19 | HW R2: 0.97 k: 0.613 dk: 0.0863

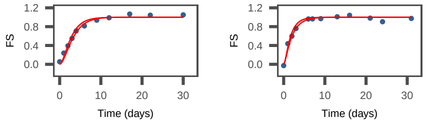

Supplement: Supplemental Data S3 [file mmc4.pdf]
