# Supplementary material for: Harmonizing Labeling and Analytical Strategies to Obtain Protein Turnover Rates in Intact Adult Animals
Source: Mol Cell Proteomics. 2022 May 28;21(7):100252. doi: 10.1016/j.mcpro.2022.100252 (PMC9249856; doi:10.1016/j.mcpro.2022.100252)

1433E − AAFDDAIAELDTLSEESYK_2

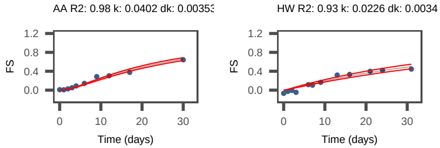

AATM − TCGFDFSGALEDISK_2

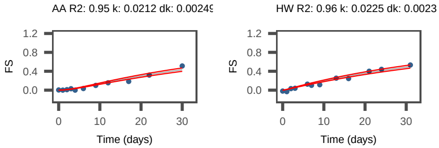

ACADV − SLSEGYPTAQHEK_3

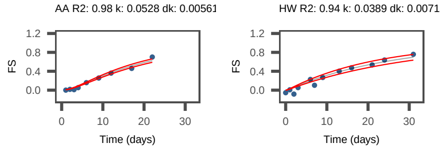

1433E − AAFDDAIAELDTLSEESYK_3

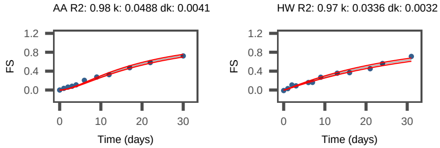

ABEC2 − LFMWEEPEVQAALK_2

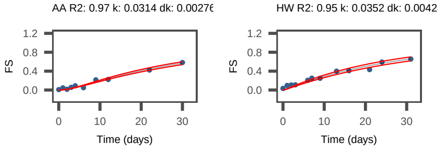

ACADV − VPSENVLGEVGDGFK_2

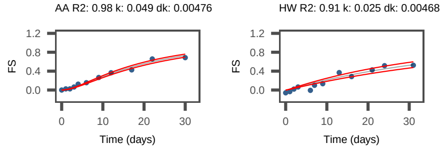

1433E − LICCDILDVLDK_2

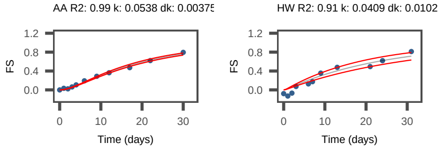

ACADL − FFQEEVIPHHTEWEK_4

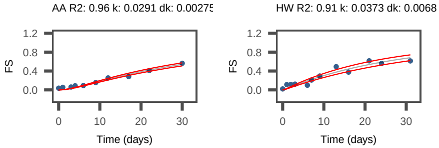

ACBP − TQPTDEEMLFIYSHFK_3

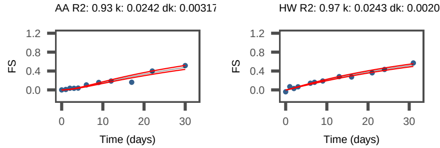

1433G(Non−Unique) − YDDMAAAMK_2

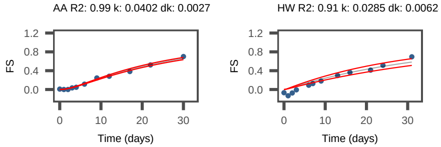

ACADL − LPANALLGEENK_2

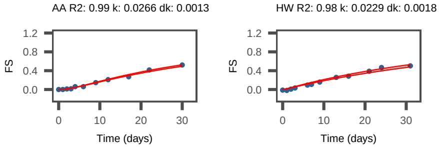

ACO13 − VTLVSAAPEK_2

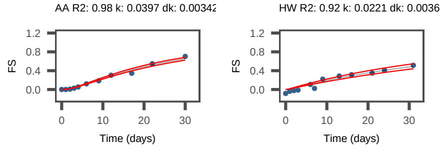

1433G − YLAEVATGEK_2

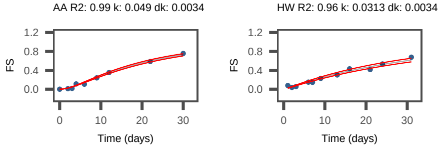

ACADM − LLVEHQGVSFLLAEMAMK_3

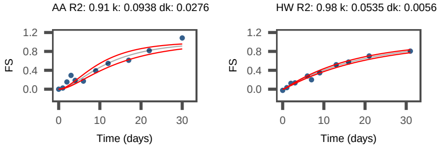

ACON − CTTDHISAAGPWLK_3

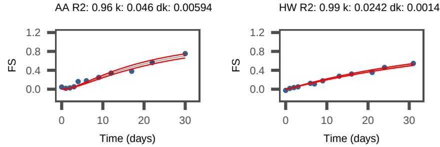

2AAA − SALASVIMGLSPILGK_2

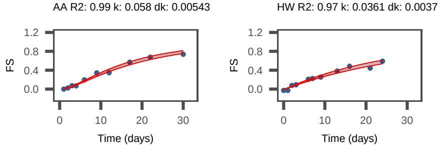

ACADV − EATQAVLDKPETLSSDASTR_3

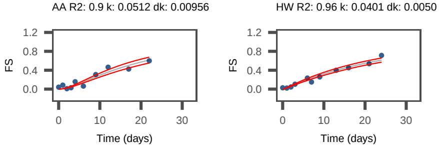

ACON − DINQEVYNFLATAGAK_2

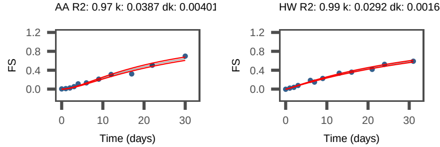

ACON − DINQEVYNFLATAGAK_3

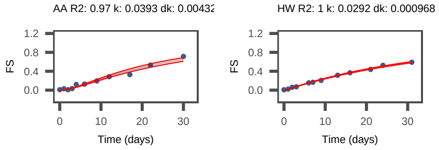

ACON − SDFDPGQDTYQHPPK_3

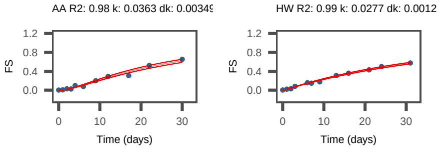

ACTN2 − LLETIDQLHLEFAK_3

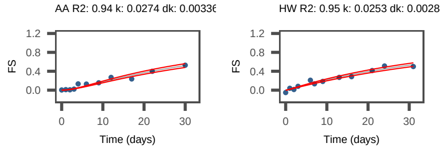

ACON − DLEDLQILIK_2

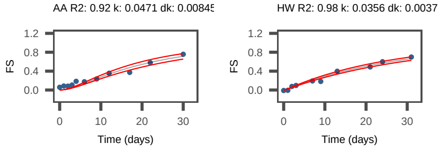

ACON − VAGILTVK_2

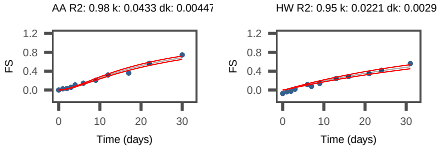

ACTN2 − QSILAIQNEVEK_2

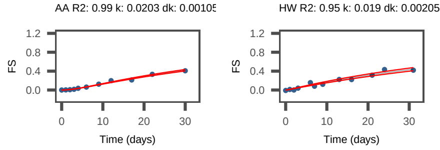

ACON − FNPETDFLTGK_2

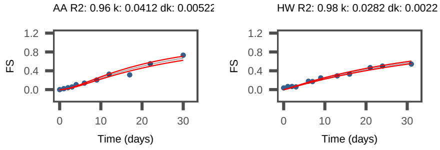

ACON − VAVPSTIHCDHLIEAQVGGEK_3

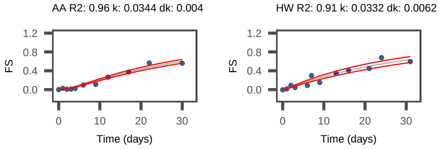

ACTN2 − SSIQITGALEDQMNQLK_3

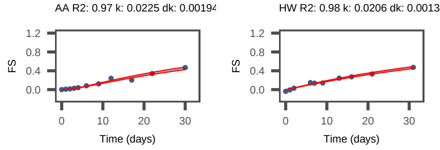

ACON − LNRPLTLSEK_3

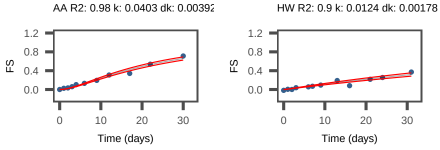

ACTN2 − AIGPWIQNK_2

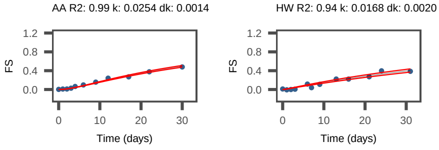

ACTN3 − CQAICDQWDNLGTLTQK_3

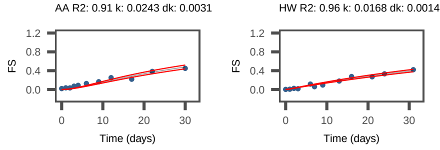

ACON − LQLLEPFDK_2

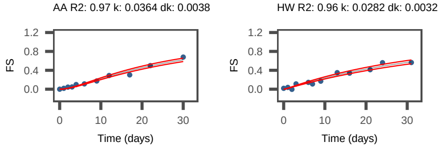

ACTN2 − ASTHETWAYGK_3

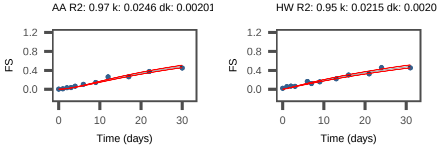

ACTN3(Non−Unique) − CQLEINFNTLQTK_3

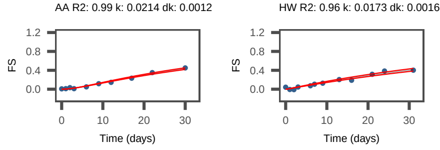

ACON − LTGSLSGWTSPK_2

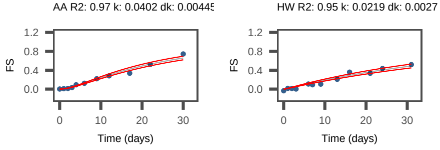

ACTN2 − ILASDKPYILAEELR_3

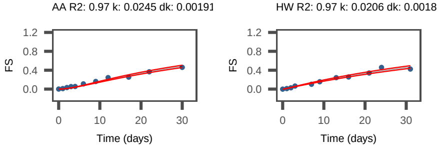

ACTN3 − DDPIGNLNTAFEVAEK_2

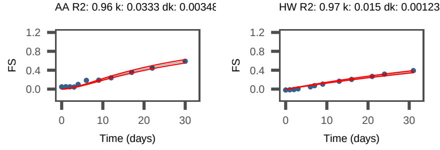

ACTN3 − ETAETDTAEQVVASFK_3

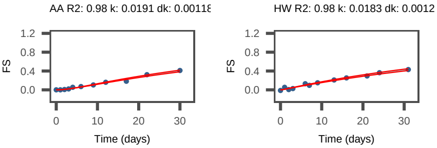

ACTN3 − QQEQNIINYK_2

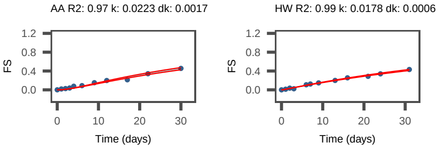

ALBU − AETFTFHSDICTLPEK_3

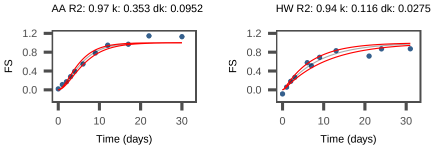

ACTN3(Non−Unique) − FAIQDISVEETSAK_2

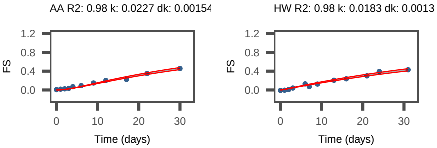

ACTN3 − VLAVNQENEK_2

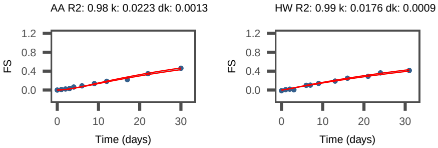

ALBU − ENYGELADCCTK_2

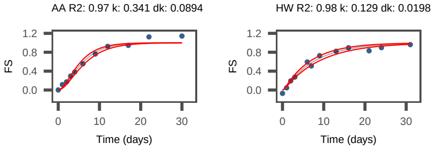

ACTN3 − GAILGIQGEIQK_2

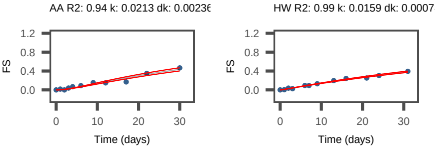

ACTN4(Non−Unique) − KHEAFESDLAAHQDR_4

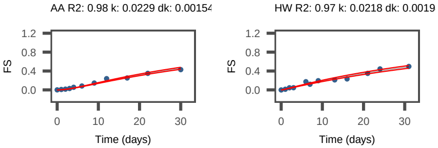

ALBU − GLVLIAFSQYLQK_2

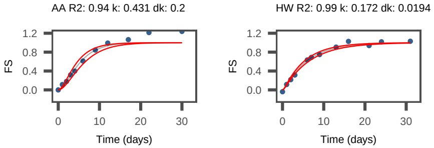

ACTN3 − LMEEYEK_2

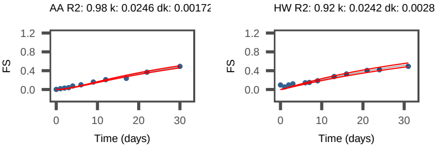

ADT1 − DEGANAFFK_2

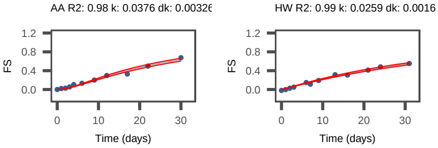

ALBU − LATDLTK_2

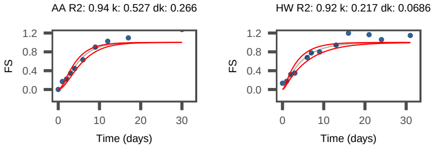

ACTN3 − NVNVQNFHTSWK_3

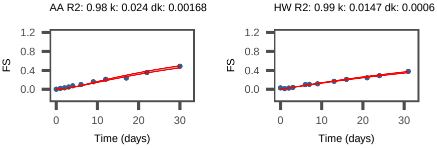

ADT1(Non−Unique) − LLLQVQHASK_2

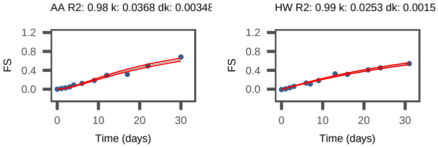

ALBU − LSQTFPNADFAEITK_2

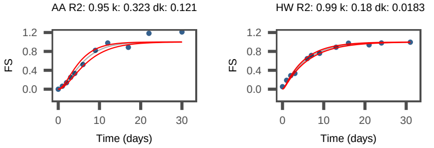

ACTN3(Non−Unique) − PAFMPSEGK_2

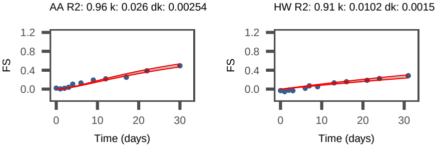

ADT1(Non−Unique) − LLLQVQHASK_3

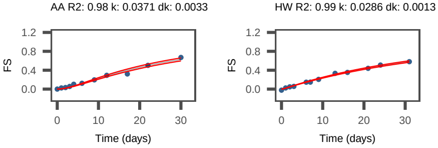

ALBU − LVQEVTDFAK_2

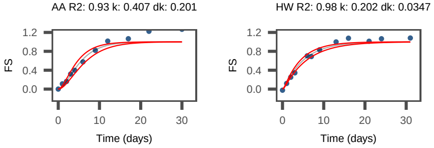

ALBU − QTALAELVK_2

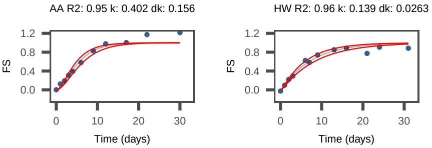

ALBU − YMCENQATISSK_2

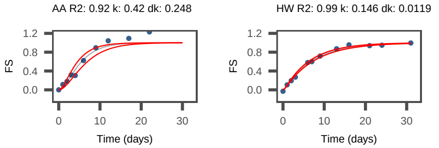

ALDOA − PHPYPALTPEQK_2

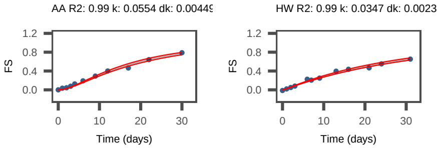

ALBU − RPCFSALTVDETYVPK_2

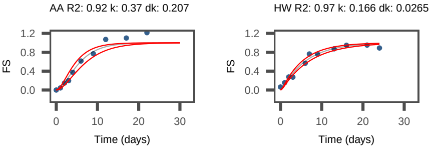

ALBU_HUMAN,sp|P07724|ALBU(Non−Unique) − NECFLQHK_3

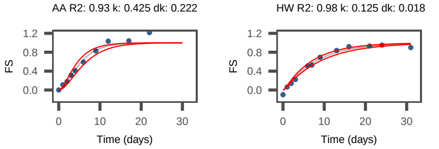

ALDOA − PHPYPALTPEQK_3

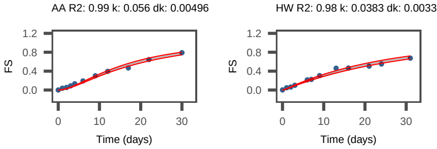

ALBU − TCVADESAANCDK_2

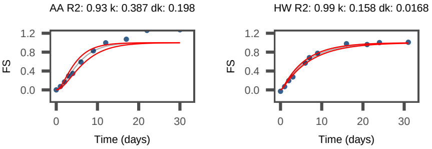

ALBU_HUMAN,sp|P07724|ALBU(Non−Unique) − SLHTLFGDK_2

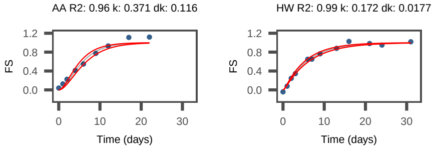

ALDOA_RABIT,sp|P05063|ALDOC(Non−Unique) − VLAAVYK_2

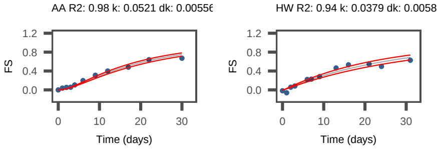

ALBU − TNCDLYEK_2

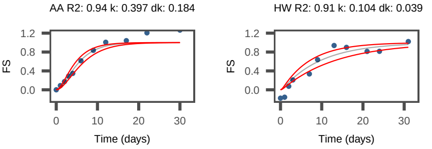

ALDH2 − LGPALATGNVVVMK_2

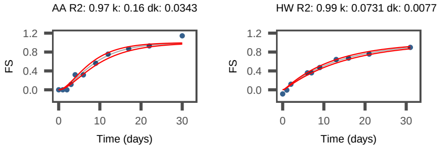

ALDOA_RABIT,sp|P05064|ALDOA(Non−Unique) − ADDGRPFPQVIK_2

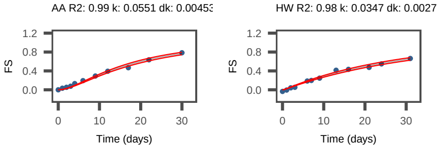

ALBU − TVMDDFAQFLDTCCK_2

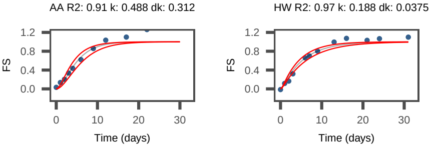

ALDOA − AAQEEYIK_2

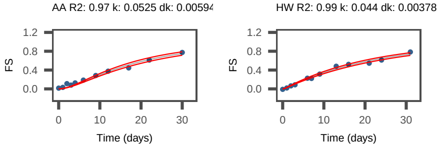

ALDOA_RABIT,sp|P05064|ALDOA(Non−Unique) − ALQASALK_2

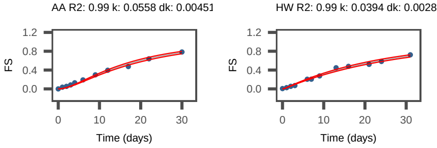

ALBU − TVMDDFAQFLDTCCK_3

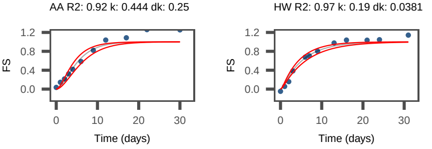

ALDOA − AAQEEYIKR_3

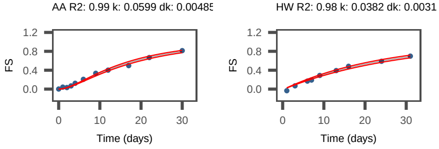

ALDOA_RABIT,sp|P05064|ALDOA(Non−Unique) − CPLLKPW_2

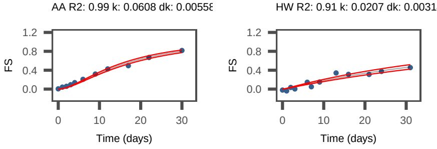

ALDOA_RABIT,sp|P05064|ALDOA(Non−Unique) − CPLLKPWAL[illegible]

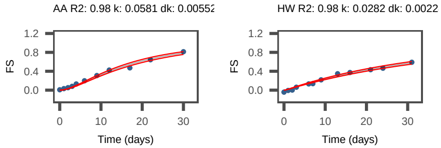

ALDOA_RABIT,sp|P05064|ALDOA(Non−Unique) − LSGGQSEEEASINLNAINK_2

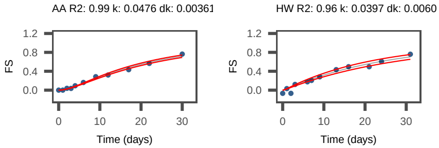

ALDOC(Non−Unique) − ALSDHHVYLEGTLLKPN_4

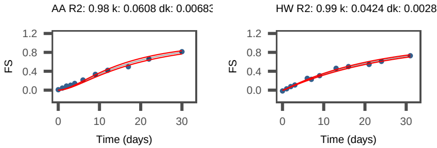

ALDOA_RABIT,sp|P05064|ALDOA(Non−Unique) − CPLLKPWALTF_2

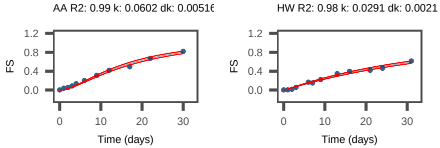

ALDOA_RABIT,sp|P05064|ALDOA(Non−Unique) − MVTPGHACTQK_2

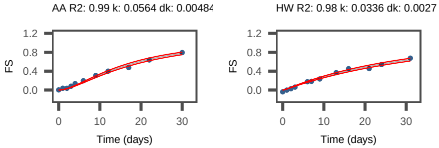

ALDR − LIEYCHSK_3

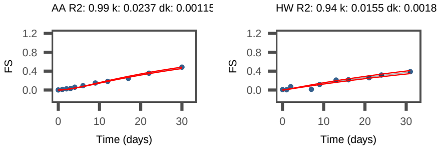

ALDOA_RABIT,sp|P05064|ALDOA(Non−Unique) − GILAADESTGSIAK_2

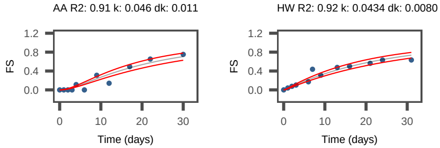

ALDOA_RABIT,sp|P05064|ALDOA(Non−Unique) − MVTPGHACTQK_3

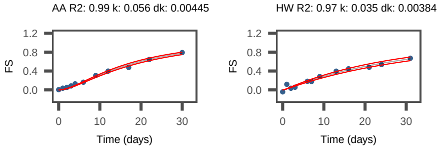

AMPD1 − NIDGEALVGNESFYPVFTPPPK_2

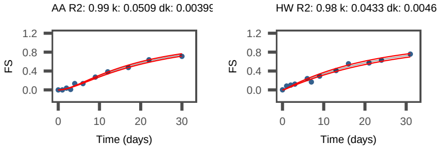

ALDOA_RABIT,sp|P05064|ALDOA(Non−Unique) − IGGVILFHE[illegible]

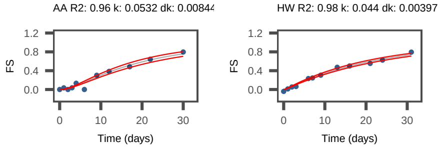

ALDOA_RABIT,sp|P05064|ALDOA(Non−Unique) − TVPPAVTGVTFLSGGQSEEEASINLNAINK_4

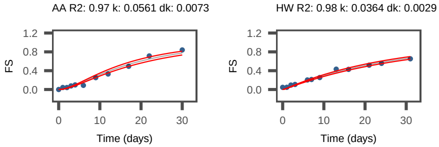

AMPD1 − NPFLDFLQK_2

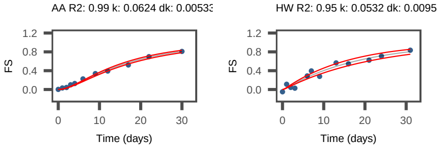

ALDOA_RABIT,sp|P05064|ALDOA(Non−Unique) − IGGVILFHETLYQK_[illegible]

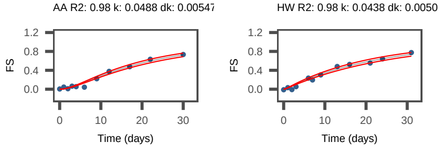

ALDOA_RABIT,sp|P05064|ALDOA(Non−Unique) − VNPCIGGVILFHETLYQK_2

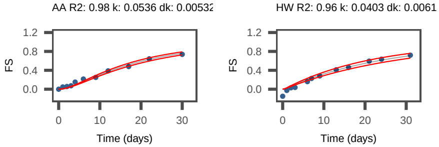

AMPD1 − TEDLPANLGYHLK_3

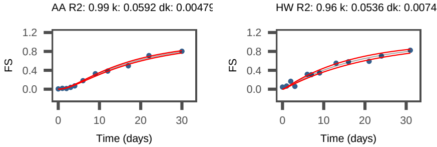

ALDOA_RABIT,sp|P05064|ALDOA(Non−Unique) − KELSDIAHR_3

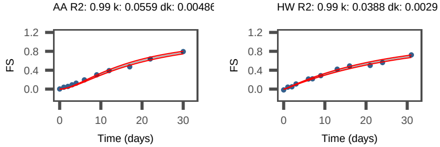

ALDOC(Non−Unique) − ALSDHHVYLEGTLLKPN_2

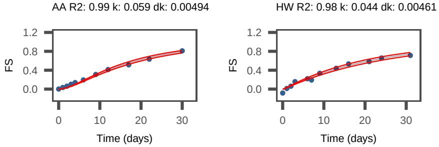

AMPD1 − TVNLSIPQSETSSTK_2

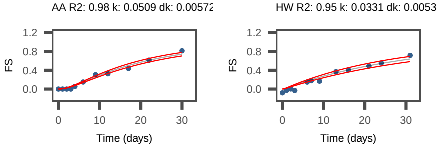

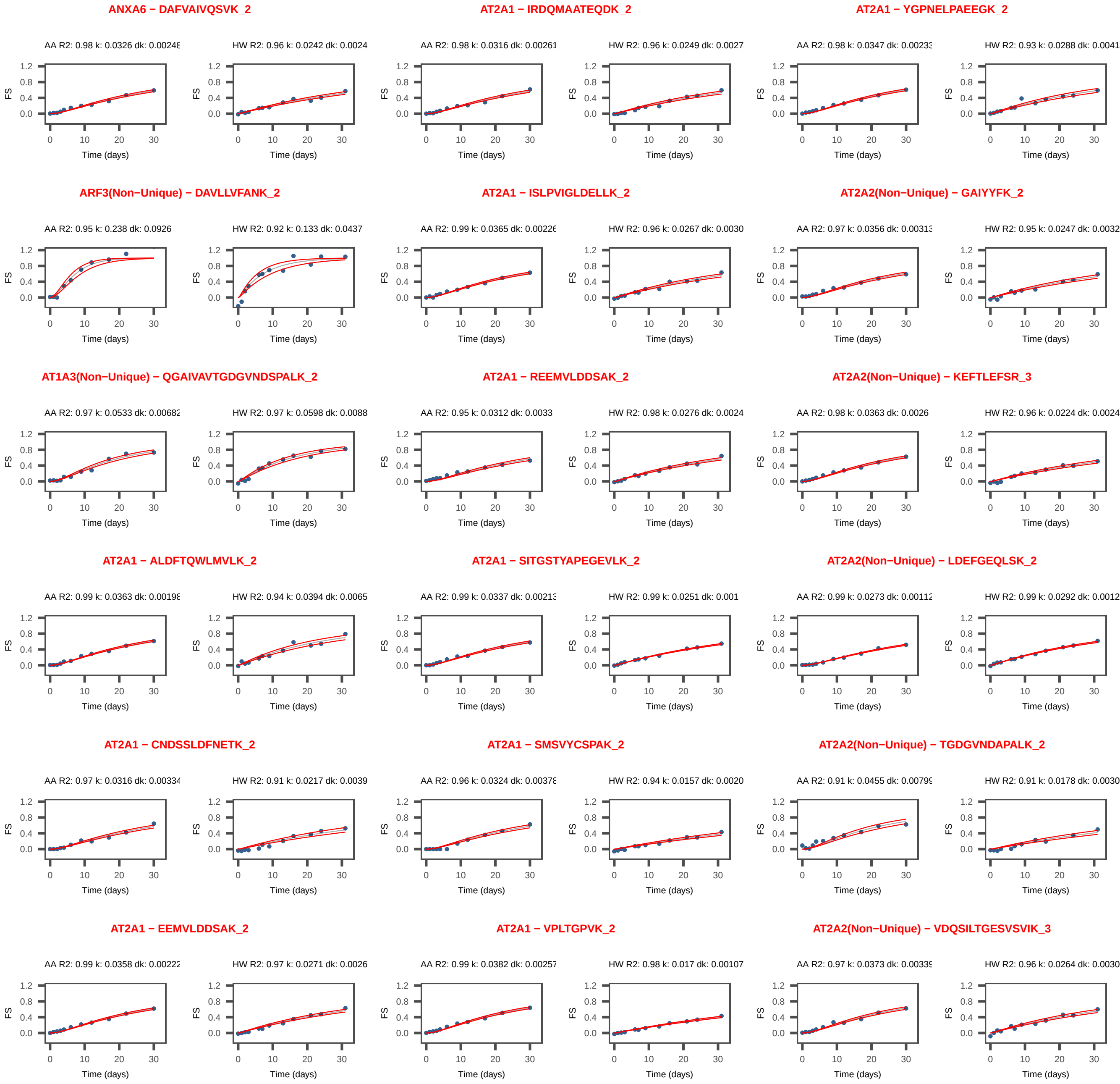
ANXA6 − DAFVAIVQSVK_2
AA R2: 0.98 k: 0.0326 dk: 0.00248
HW R2: 0.96 k: 0.0242 dk: 0.0024
AT2A1 − IRDQMAATEQDK_2
AA R2: 0.98 k: 0.0316 dk: 0.00261
HW R2: 0.96 k: 0.0249 dk: 0.0027
AT2A1 − YGPNELPAEEGK_2
AA R2: 0.98 k: 0.0347 dk: 0.00233
HW R2: 0.93 k: 0.0288 dk: 0.0041
ARF3(Non−Unique) − DAVLLVFANK_2
AA R2: 0.95 k: 0.238 dk: 0.0926
HW R2: 0.92 k: 0.133 dk: 0.0437
AT2A1 − ISLPVIGLDELLK_2
AA R2: 0.99 k: 0.0365 dk: 0.00226
HW R2: 0.96 k: 0.0267 dk: 0.0030
AT2A2(Non−Unique) − GAIYYFK_2
AA R2: 0.97 k: 0.0356 dk: 0.00313
HW R2: 0.95 k: 0.0247 dk: 0.0032
AT1A3(Non−Unique) − QGAIVAVTGDGVNDSPALK_2
AA R2: 0.97 k: 0.0533 dk: 0.00682
HW R2: 0.97 k: 0.0598 dk: 0.0088
AT2A1 − REEMVLDDSAK_2
AA R2: 0.95 k: 0.0312 dk: 0.0033
HW R2: 0.98 k: 0.0276 dk: 0.0024
AT2A2(Non−Unique) − KEFTLEFSR_3
AA R2: 0.98 k: 0.0363 dk: 0.0026
HW R2: 0.96 k: 0.0224 dk: 0.0024
AT2A1 − ALDFTQWLMVLK_2
AA R2: 0.99 k: 0.0363 dk: 0.00198
HW R2: 0.94 k: 0.0394 dk: 0.0065
AT2A1 − SITGSTYAPEGEVLK_2
AA R2: 0.99 k: 0.0337 dk: 0.00213
HW R2: 0.99 k: 0.0251 dk: 0.001
AT2A2(Non−Unique) − LDEFGEQLSK_2
AA R2: 0.99 k: 0.0273 dk: 0.00112
HW R2: 0.99 k: 0.0292 dk: 0.0012
AT2A1 − CNDSSLDFNETK_2
AA R2: 0.97 k: 0.0316 dk: 0.00334
HW R2: 0.91 k: 0.0217 dk: 0.0039
AT2A1 − SMSVYCSPAK_2
AA R2: 0.96 k: 0.0324 dk: 0.00378
HW R2: 0.94 k: 0.0157 dk: 0.0020
AT2A2(Non−Unique) − TGDGVNDAPALK_2
AA R2: 0.91 k: 0.0455 dk: 0.00799
HW R2: 0.91 k: 0.0178 dk: 0.0030
AT2A1 − EEMVLDDSAK_2
AA R2: 0.99 k: 0.0358 dk: 0.00222
HW R2: 0.97 k: 0.0271 dk: 0.0026
AT2A1 − VPLTGPVK_2
AA R2: 0.99 k: 0.0382 dk: 0.00257
HW R2: 0.98 k: 0.017 dk: 0.00107
AT2A2(Non−Unique) − VDQSILTGESVSVIK_3
AA R2: 0.97 k: 0.0373 dk: 0.00339
HW R2: 0.96 k: 0.0264 dk: 0.0030
FS
Time (days)

AT2A3(Non−Unique) − DIVPGDIVEVAVGDKVPADLR_2

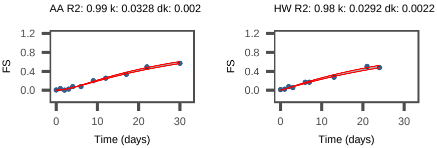

ATPA − GMSLNLEPDNVGVVVFGNDK_3

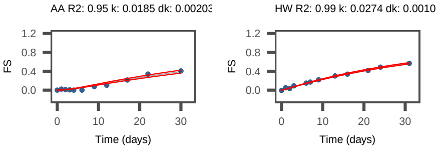

ATPB − FLSQPFQVAEVFTGHMGK_3

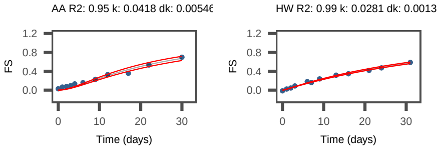

AT2A3(Non−Unique) − DIVPGDIVEVAVGDKVPADLR_3

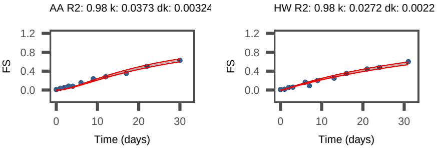

ATPA − HALIIYDDLSK_2

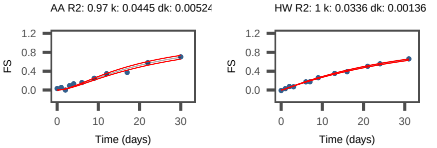

ATPB − IGLFGGAGVGK_2

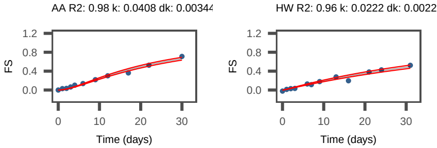

AT5F1 − PLPPLPEYGGK_2

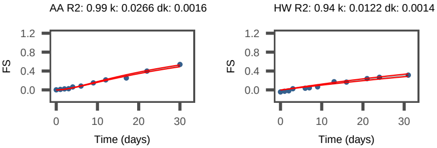

ATPA − LKEIVTNFLAGFEP_2

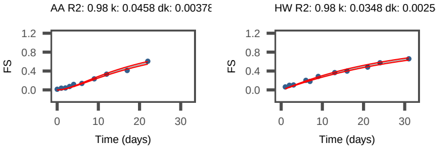

ATPB − TVLIMELINNVAK_2

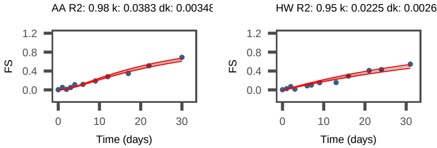

ATP5H − TIDWVSFVEVMPQNQK_3

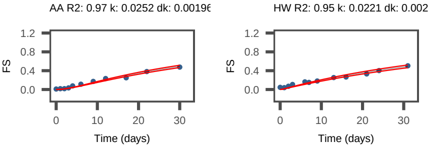

ATPA − NVQAEEMVEFSSGLK_3

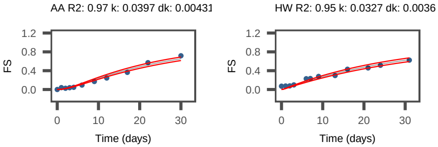

ATPD − IEANEALVK_2

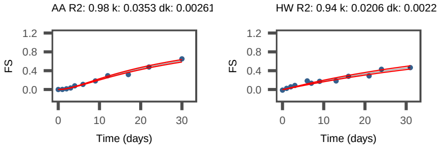

ATP5J − FEVIDKPQS_2

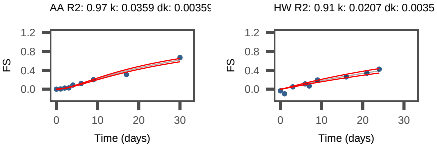

ATPA − TSIAIDTIINQK_3

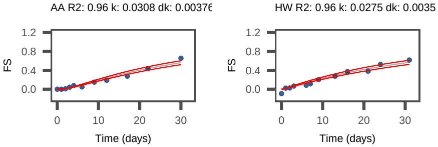

ATPG − THSDQFLVSFK_3

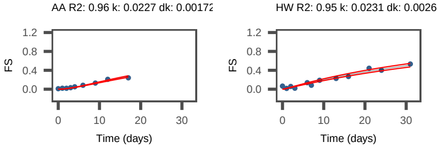

ATPA − GMSLNLEPDNVGVVVFGNDK_2

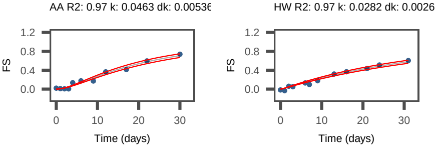

ATPA − VVDALGNAIDGK_2

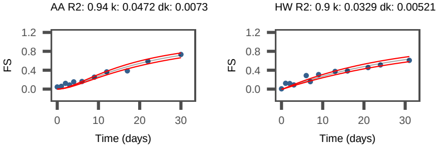

ATPO − YATALYSAASK_2

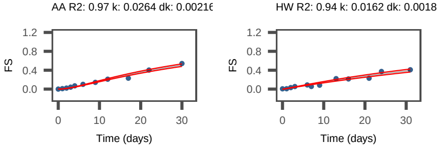

BIN1 − HHYESLQTAK_3

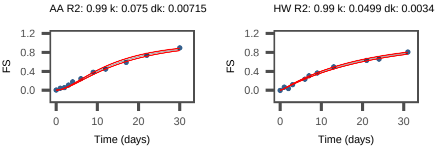

CAH3 − HDPSLQPWSASYDPGSAK_2

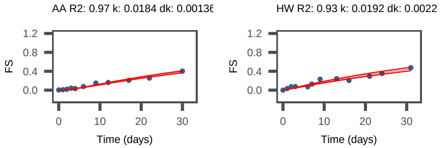

CH60 − ALMLQGVDLLADAVAVTMGPK_3

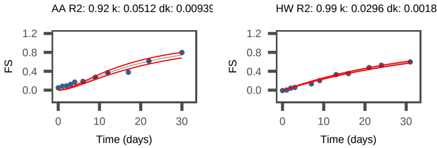

BIN1 − IAENNDLLWMDYHQK_3

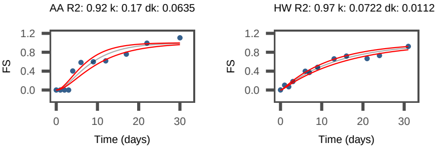

CAH3 − YAAELHLVHWNPK_3

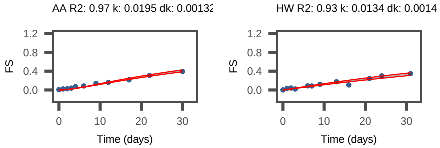

CMC1 − IVQLLAGVADQTK_2

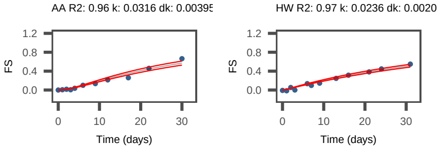

BIN1 − LDLPPGFMFK_2

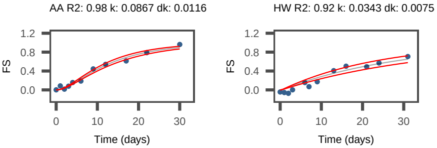

CAH3 − YAAELHLVHWNPK_4

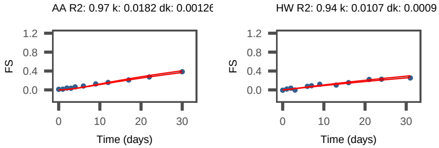

CMC1 − YLGLYNDPNSNPK_2

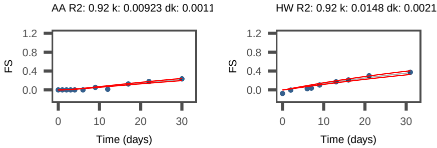

BIN1 − LNQNLNDVLVSLEK_2

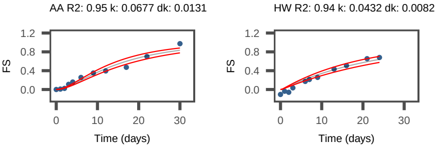

CALM1(Non−Unique) − VFDKDGNGYISAAELR_3

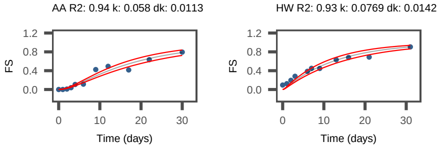

COF1(Non−Unique) − YALYDATYETK_2

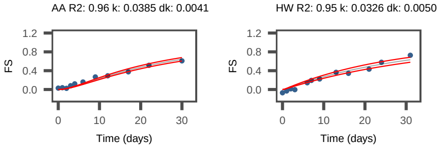

CAH3 − EPMTVSSDQMAK_2

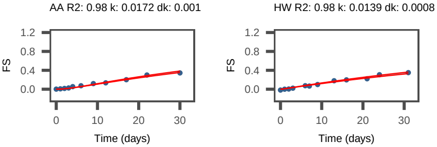

CH10 − VLQATVVAVGSGGK_2

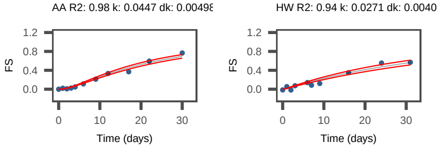

COF2 − LGGSVVVSLEGKPL_2

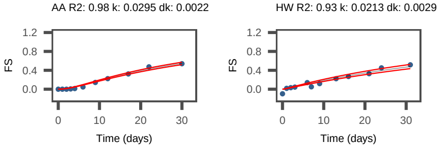

CAH3 − GDNQSPIELHTK_2

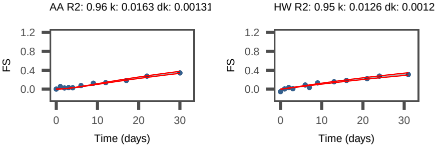

CH10 − VVLDDKDYFLFR_3

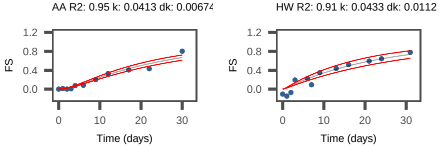

COQ9 − LVQLGQAEK_2

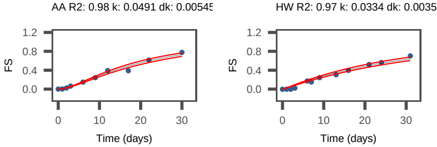

COQ9 − STGEALVQGLMGAAVTLK_2

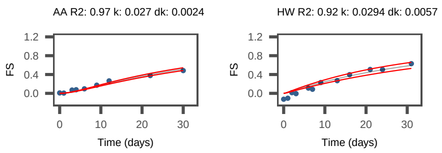

COQ9 − STGEALVQGLMGAAVTLK_3

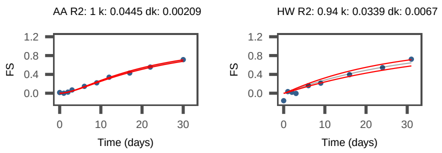

COX41 − DWVAMQTK_2

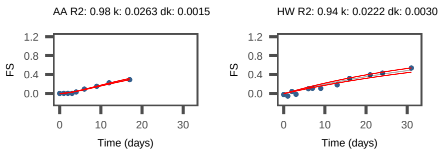

COX41 − DYPLPDVAHVTMLSASQK_3

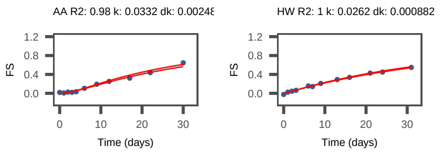

COX5A − GMNTLVGYDLVPEPK_2

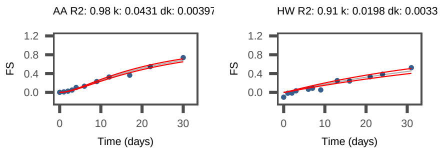

COX5A − NKPDIDAWELR_3

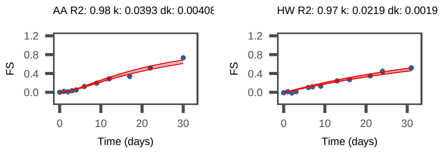

COX5B − EDPNLVPSISNK_2

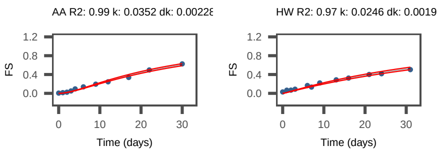

COX6C − NYDSMKDFEEMR_3

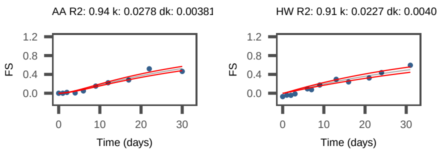

CPT1B − QALLDIAELFK_2

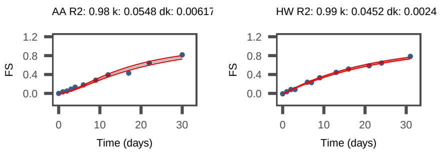

CPT1B − SPLMVNSNYYAMDFVLIK_2

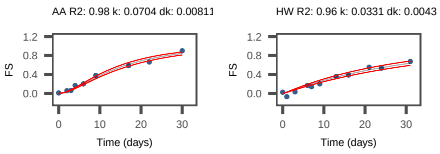

CRYAB − VLGDVIEVHGK_3

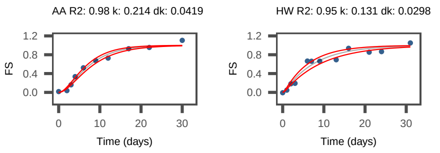

CYC − TGQAAGFSYTDANK_2

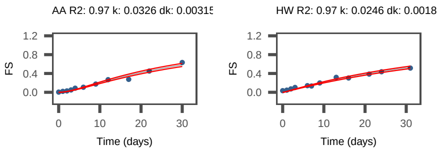

DDX3L(Non−Unique) − SFLLDLLNATGK_2

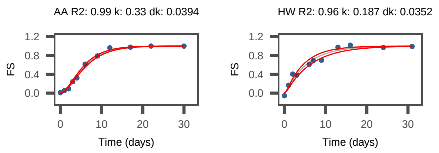

DESM − HQIQSYTCEIDALK_3

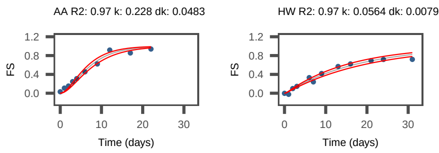

DESM − VSDLTQAANK_2

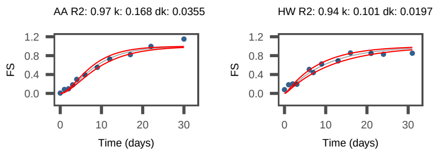

DLDH − ALTGGIAHLFK_3

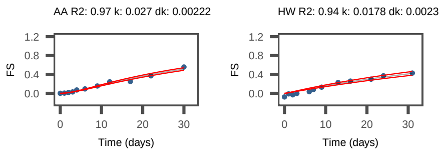

DLDH − EANLAAAFGK_2

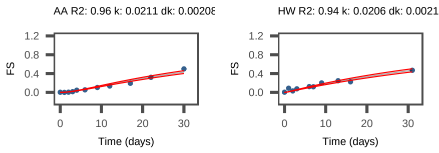

DLDH − IPNIYAIGDVVAGPMLAHK_3

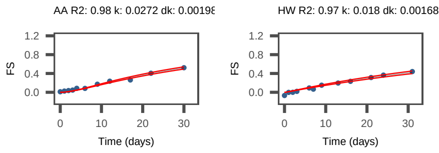

ECHA − MGLVDQLVEPLGPGIK_2

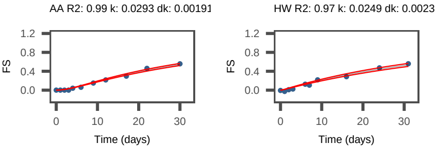

ECHA − MGLVDQLVEPLGPGIK_3

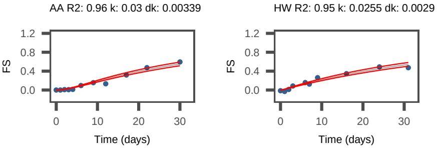

ECHA − MQLLEIITTDK_2

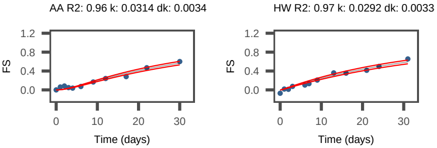

ECHA − NVQQLAILGAGLMGAGIAQVSVDK_3

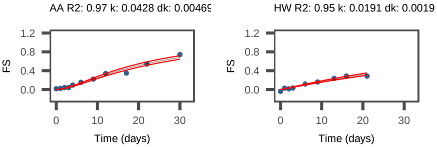

ECHB − AMIVEAYPK_2

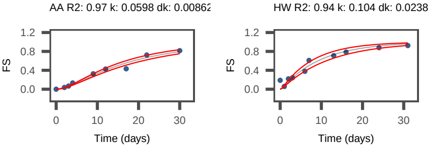

ECHB − DQLLLGPTYATPK_2

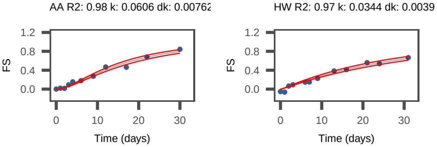

ECHB − DVVDYIIFGTVIQEVK_2

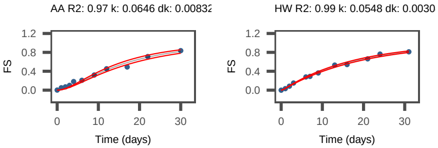

ECHB − DVVDYIIFGTVIQEVK_3

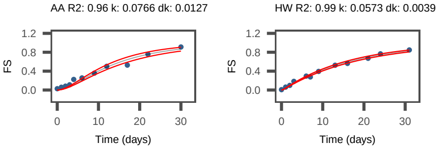

ECI1 − ALQLGTLFSPAEALK_2

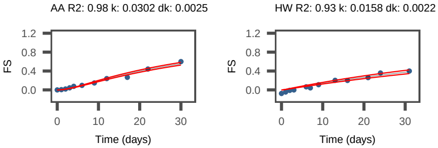

EF1A1(Non−Unique) − LPLQDVYK_2

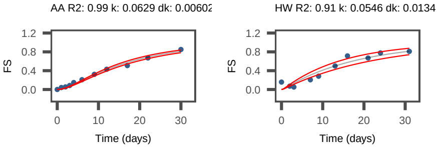

EF1A1(Non−Unique) − STTTGHLIYK_2

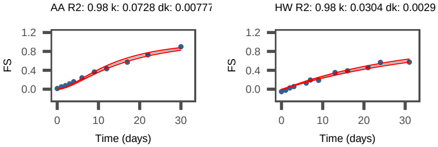

EF1A2 − MDSTEPAYSEK_2

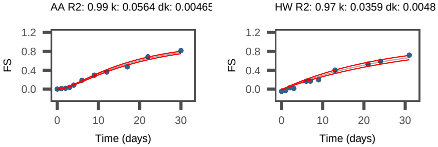

EF1A2 − PGMVVTFAPVNITTEVK_2

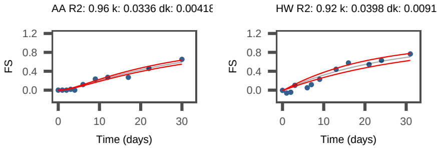

EF1A2 − VETGILRPGMVVTFAPVNITTEVK_3

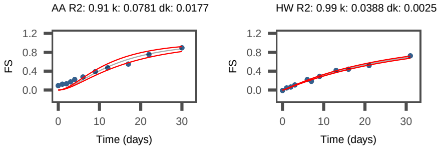

EF2 − DSVVAGFQWATK_2

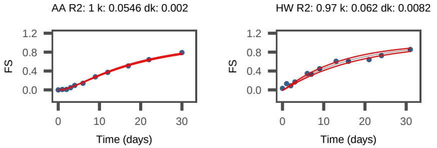

EF2 − EGIPALDNFLDKL_2

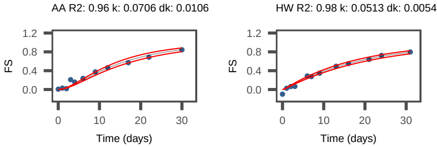

EF2 − IVGLVGVDQFLVK_2

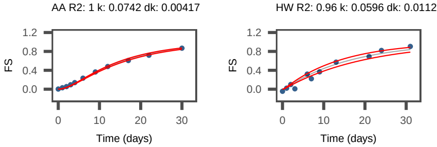

EF2 − IWCFGPDGTGPNILTDITK_2

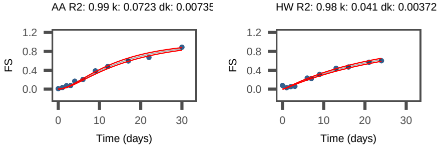

EFTU − DLDKPFLLPVESVYSIPGR_3

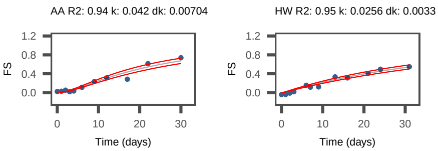

ENOB − IGAEVYHHLK_2

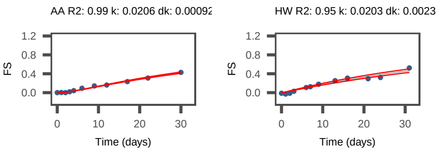

ENPL(Non−Unique) − ELISNASDALDK_2

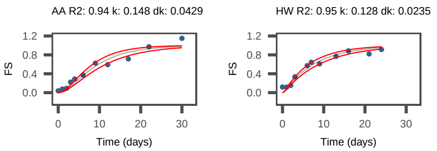

ENOA(Non−Unique) − AILGVSLAVCK_2

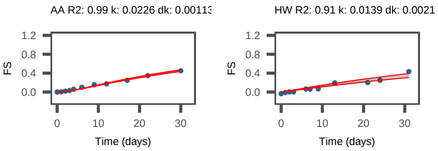

ENOB − IGAEVYHHLK_3

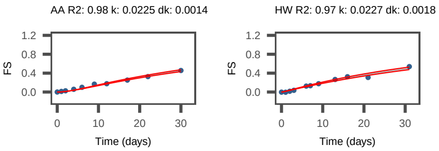

ETFA − GTSFEAAATSGGSASSEK_2

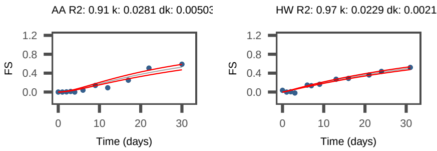

ENOA(Non−Unique) − SGETEDTFIADLVVGLCTGQIK_2

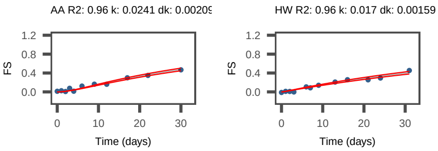

ENOB − ILPVGASSFK_2

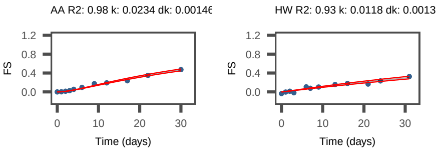

ETFA − LNVAPVSDIIEIK_2

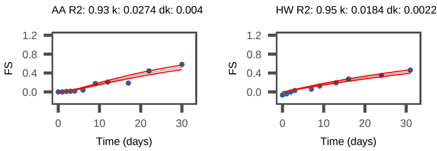

ENOB − GNPTVEVDLHTAK_2

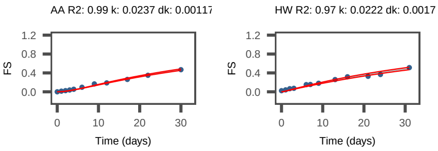

ENOB − LAMQEFMILPVGASSFK_3

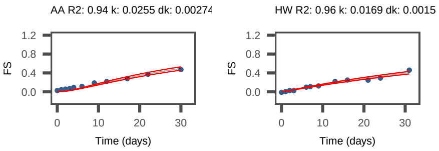

ETFA − SDRPELTGAK_3

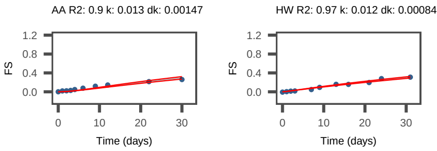

ENOB − GNPTVEVDLHTAK_3

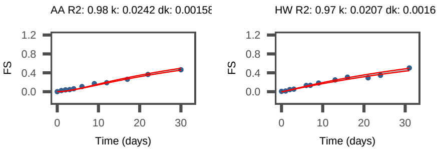

ENOB − LSGVDIQIVGDDLTVTNPK_2

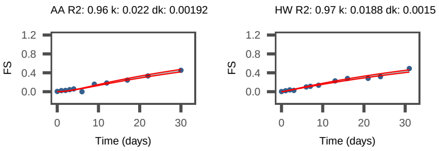

ETFD − ALNEGGLQSIPK_2

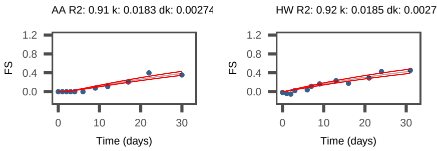

ENOB − IEEALGDK_2

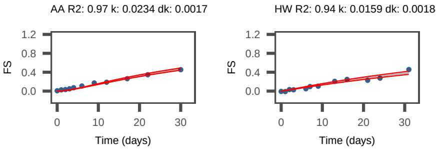

ENOB − LSVVDQEK_2

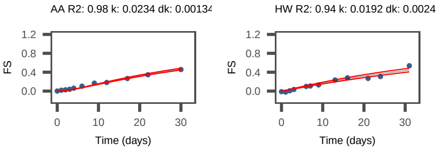

FABP4 − LVSSENFDDYMK_2

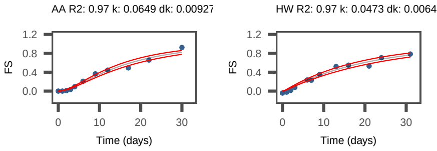

FHL1 – AIVAGDQNVEYK_2

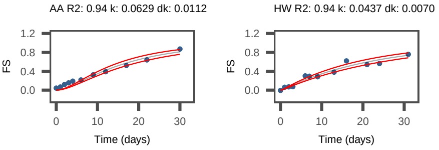

FLNB(Non–Unique) – LLGWIQNK_2

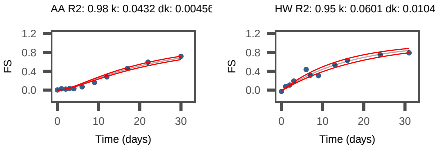

G3P – IIPASTGAAK_2

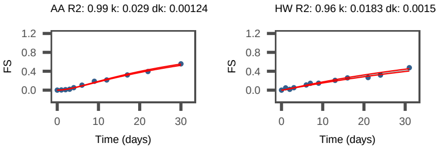

FHL1 – CLHPLASETFVSK_3

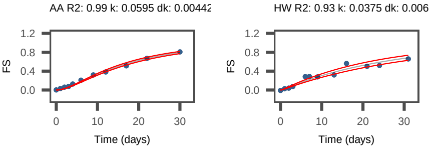

FLNC – GPGLSQAFVGQK_2

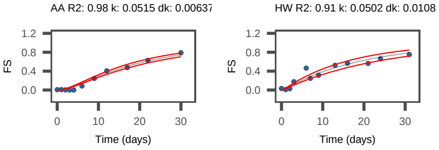

G3P – MFQYDSTHGK_2

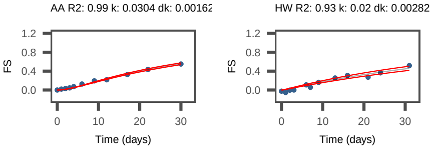

FHL1 – FTAVEDQYYCVDCYK_2

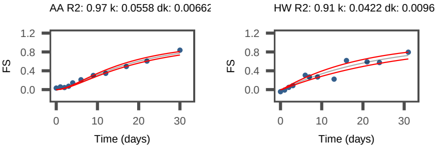

FLNC – SPFVVNVAPPLDLSK_2

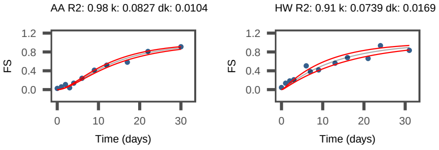

G3P – MFQYDSTHGK_3

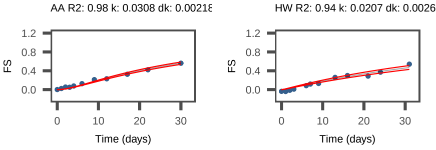

FHL1 – FVFHNEQVYCPDCAK_2

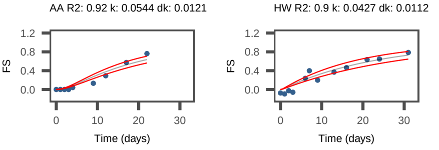

FLNC – TPCEEVYVK_2

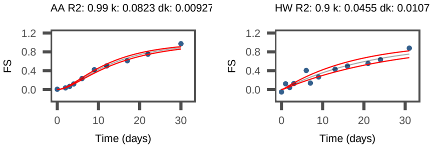

G3P – VIISAPSADAPM[15.9949]FVMGVNHEK_3

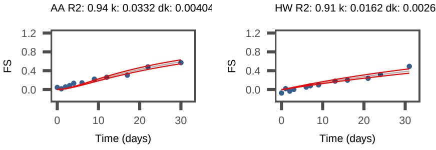

FHL1 – FVFHNEQVYCPDCAK_3

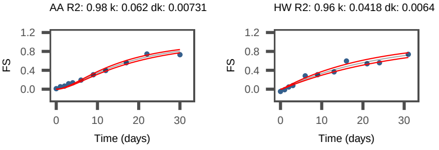

FLNC – TSQLNVGTSTDVSLK_2

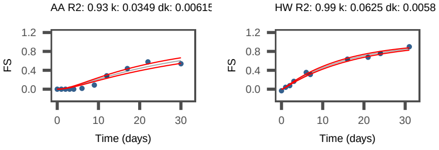

G3P – VIISAPSADAPMFVMGVNHEK_2

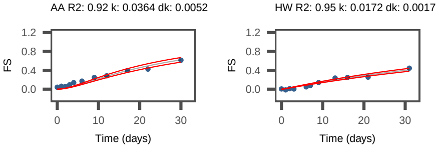

FLNB(Non–Unique) – DLAEDAPWK_2

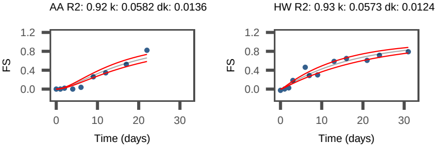

FUMH – AAAEVNQEYGLDPK_2

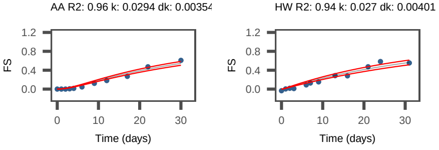

G3P – VIPELNGK_2

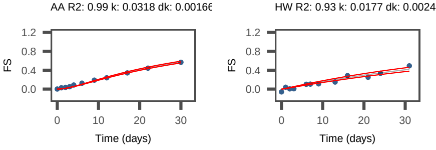

G6PI − EVMQMLVELAK_2

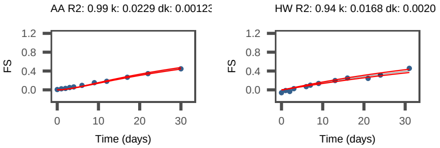

GPDA − LTEIINTQHENVK_3

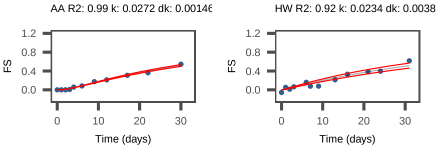

GYS1 − LYESLLVGSLPDMNK_2

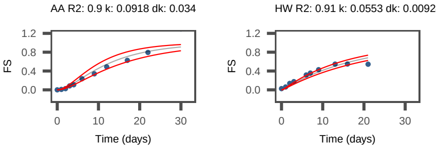

G6PI − EWFLEAAK_2

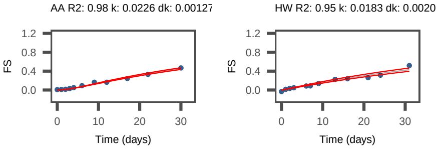

GRP75 − EQQIVIQSSGGLSK_2

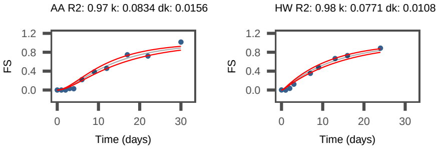

GYS1 − VGGIYTVLQTK_2

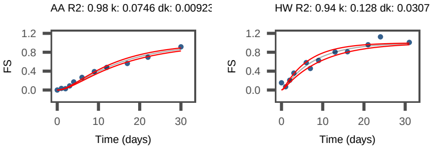

G6PI − ILLANFLAQTEALMK_2

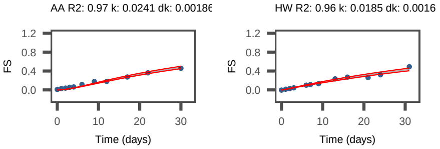

GRP75 − QAASSLQQASLK_2

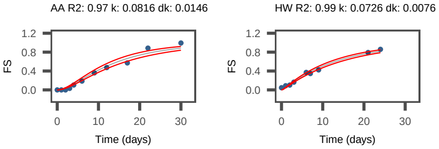

H12(Non−Unique) − ALAAAGYDVEK_2

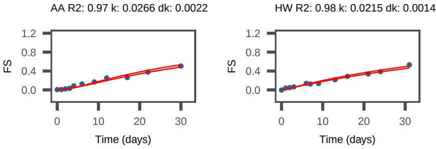

G6PI − TLASLSPETSLFIIASK_2

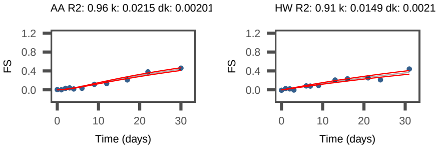

GSTM1(Non−Unique) − LGLDFPNLPYLIDGSHK_3

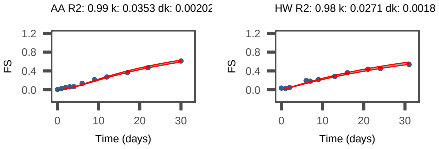

H12(Non−Unique) − ASGPPVSELITK_2

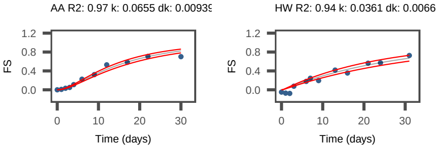

G6PI − VWFVSNIDGTHIAK_3

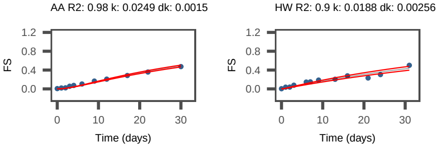

GSTM1 − YIATPIFSK_2

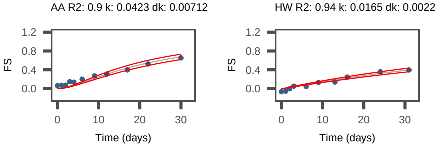

H2A1B(Non−Unique) − VTIAQGGVLPNIQAVLLPK_2

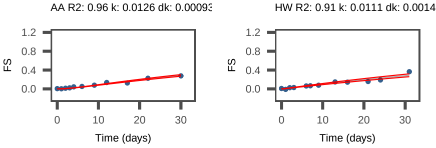

GLYG − WEQGQADYMGADSFDNIK_2

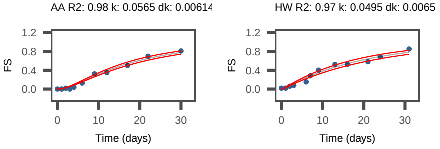

GSTP1_HUMAN,sp|P19157|GSTP1(Non−Unique) − MLLADQGQSWK_2

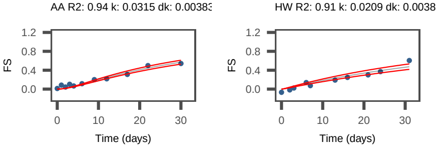

HBA − AAGHLDDLPGALSALSDLHAHK_5

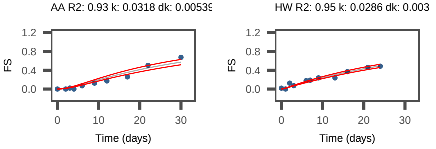

HBA − TYFPHFDVSHGSAQVK_4

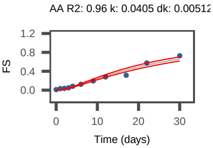

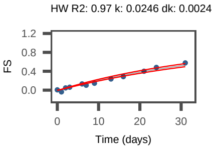

HSP72(Non−Unique) − LLQDFFNGK_2

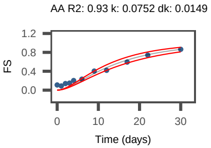

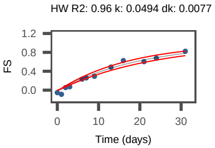

IDH3A − AGGVQTVTLIPGDGIGPEISASVMK_3

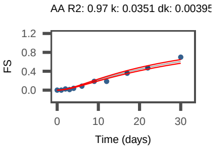

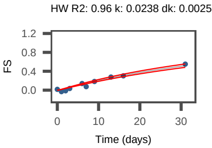

HBB1(Non−Unique) − GTFASLSELHCDK_2

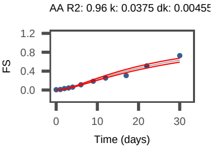

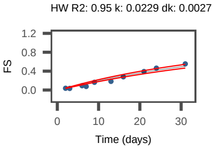

HSP7C − CNEIISWLDK_2

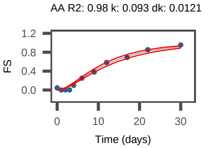

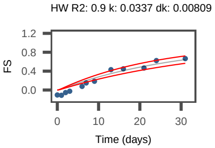

IDH3A − ENTEGEYSGIEHVIVDGVVQSIK_3

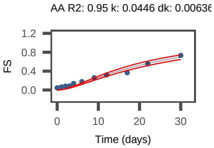

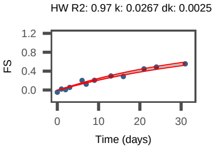

HBB1(Non−Unique) − VHLTDAEK_2

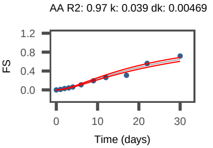

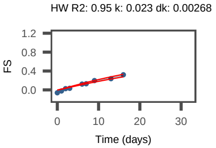

HSP7C − RFDDAVVQSDMK_3

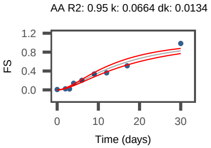

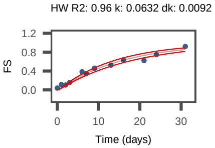

IDHG1 − NIANPTATLLASCMMLDHLK_3

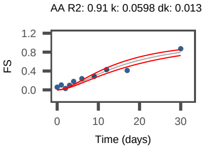

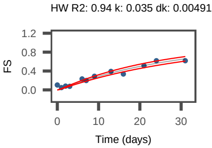

HS90A(Non−Unique) − ADLINNLGTIAK_2

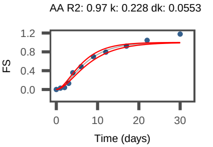

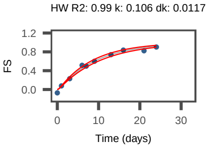

HSP7C − SINPDEAVAYGAAVQAAILSGDK_3

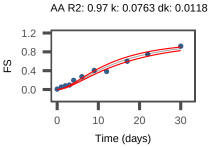

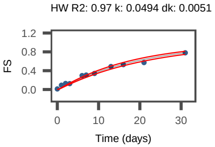

IDHP − DLAGCIHGLSNVK_3

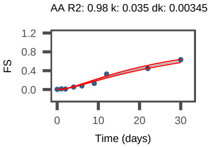

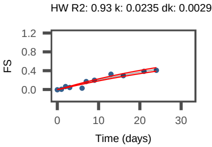

HS90B − APFDLFENK_2

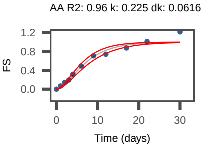

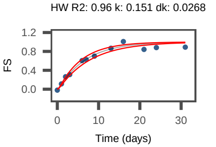

HSPB6 − APSVALPTAQVSTDSGYFSVLLDVK_3

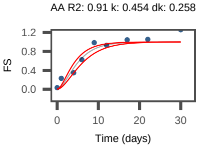

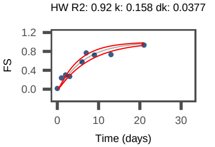

IDHP − DQTNDQVTIDSALATQK_2

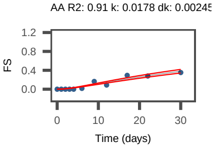

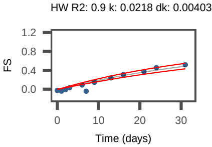

HS90B − NPDDITQEEYGEFYK_2

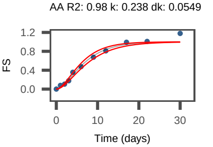

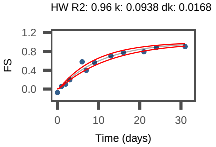

HXK2 − LSPELLTTGSFETK_2

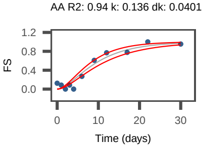

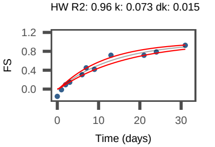

IDHP − LILPHVDVQLK_3

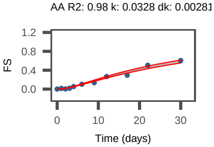

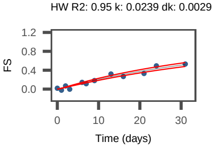

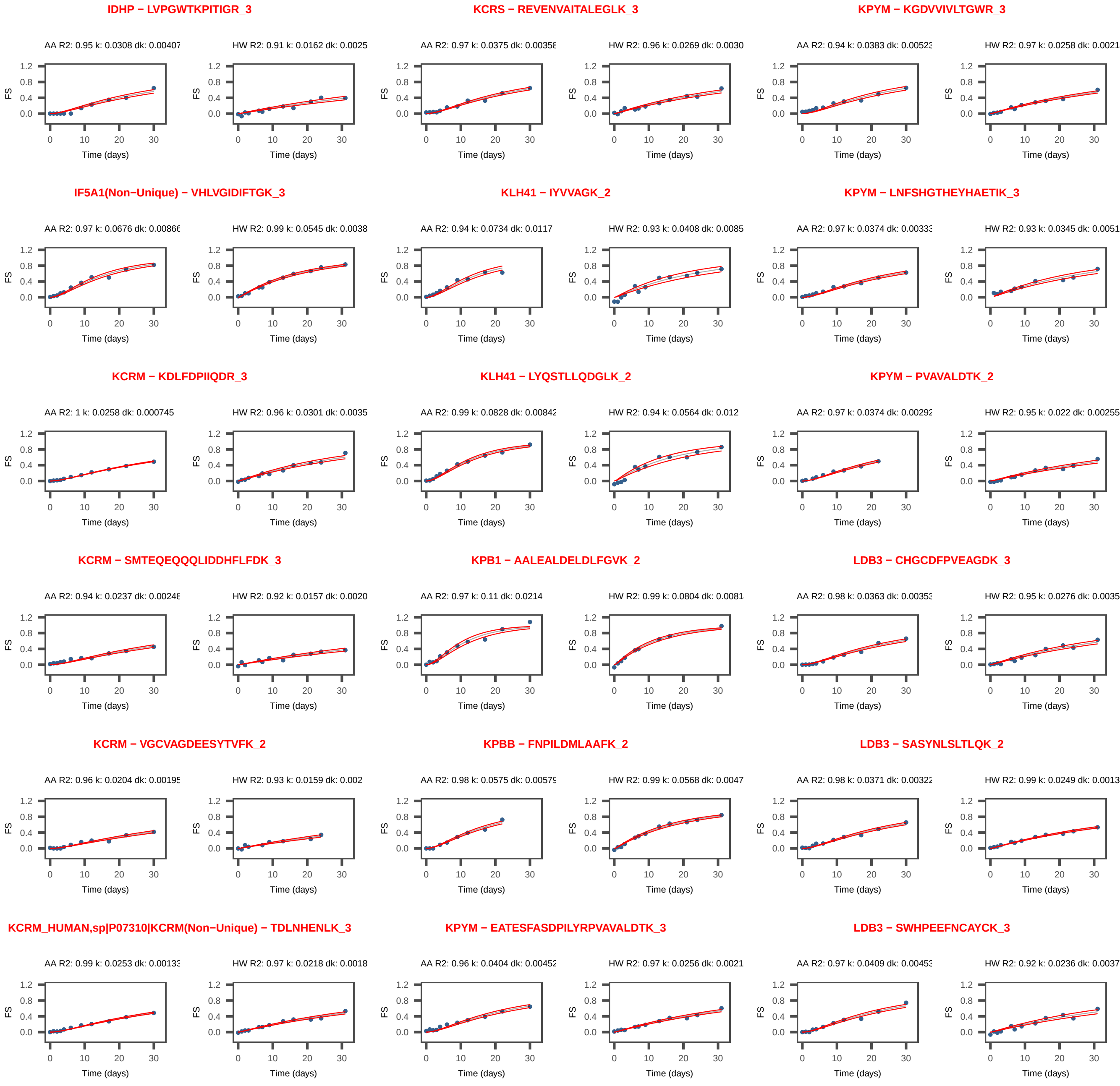
IDHP − LVPGWTKPITIGR_3
AA R2: 0.95 k: 0.0308 dk: 0.00407
HW R2: 0.91 k: 0.0162 dk: 0.0025
KCRS − REVENVAITALEGLK_3
AA R2: 0.97 k: 0.0375 dk: 0.00358
HW R2: 0.96 k: 0.0269 dk: 0.0030
KPYM − KGDVVIVLTGWR_3
AA R2: 0.94 k: 0.0383 dk: 0.00523
HW R2: 0.97 k: 0.0258 dk: 0.0021
IF5A1(Non−Unique) − VHLVGIDIFTGK_3
AA R2: 0.97 k: 0.0676 dk: 0.00866
HW R2: 0.99 k: 0.0545 dk: 0.0038
KLH41 − IYVVAGK_2
AA R2: 0.94 k: 0.0734 dk: 0.0117
HW R2: 0.93 k: 0.0408 dk: 0.0085
KPYM − LNFSHGTHEYHAETIK_3
AA R2: 0.97 k: 0.0374 dk: 0.00333
HW R2: 0.93 k: 0.0345 dk: 0.0051
KCRM − KDLFDPIIQDR_3
AA R2: 1 k: 0.0258 dk: 0.000745
HW R2: 0.96 k: 0.0301 dk: 0.0035
KLH41 − LYQSTLLQDGLK_2
AA R2: 0.99 k: 0.0828 dk: 0.00842
HW R2: 0.94 k: 0.0564 dk: 0.012
KPYM − PVAVALDTK_2
AA R2: 0.97 k: 0.0374 dk: 0.00292
HW R2: 0.95 k: 0.022 dk: 0.00255
KCRM − SMTEQEQQQLIDDHFLFDK_3
AA R2: 0.94 k: 0.0237 dk: 0.00248
HW R2: 0.92 k: 0.0157 dk: 0.0020
KPB1 − AALEALDELDLFGVK_2
AA R2: 0.97 k: 0.11 dk: 0.0214
HW R2: 0.99 k: 0.0804 dk: 0.0081
LDB3 − CHGCDFPVEAGDK_3
AA R2: 0.98 k: 0.0363 dk: 0.00353
HW R2: 0.95 k: 0.0276 dk: 0.0035
KCRM − VGCVAGDEESYTVFK_2
AA R2: 0.96 k: 0.0204 dk: 0.00195
HW R2: 0.93 k: 0.0159 dk: 0.002
KPBB − FNPILDMLAAFK_2
AA R2: 0.98 k: 0.0575 dk: 0.00579
HW R2: 0.99 k: 0.0568 dk: 0.0047
LDB3 − SASYNLSLTLQK_2
AA R2: 0.98 k: 0.0371 dk: 0.00322
HW R2: 0.99 k: 0.0249 dk: 0.0013
KCRM_HUMAN,sp|P07310|KCRM(Non−Unique) − TDLNHENLK_3
AA R2: 0.99 k: 0.0253 dk: 0.00133
HW R2: 0.97 k: 0.0218 dk: 0.0018
KPYM − EATESFASDPILYRPVAVALDTK_3
AA R2: 0.96 k: 0.0404 dk: 0.00452
HW R2: 0.97 k: 0.0256 dk: 0.0021
LDB3 − SWHPEEFNCAYCK_3
AA R2: 0.97 k: 0.0409 dk: 0.00453
HW R2: 0.92 k: 0.0236 dk: 0.0037
FS
Time (days)

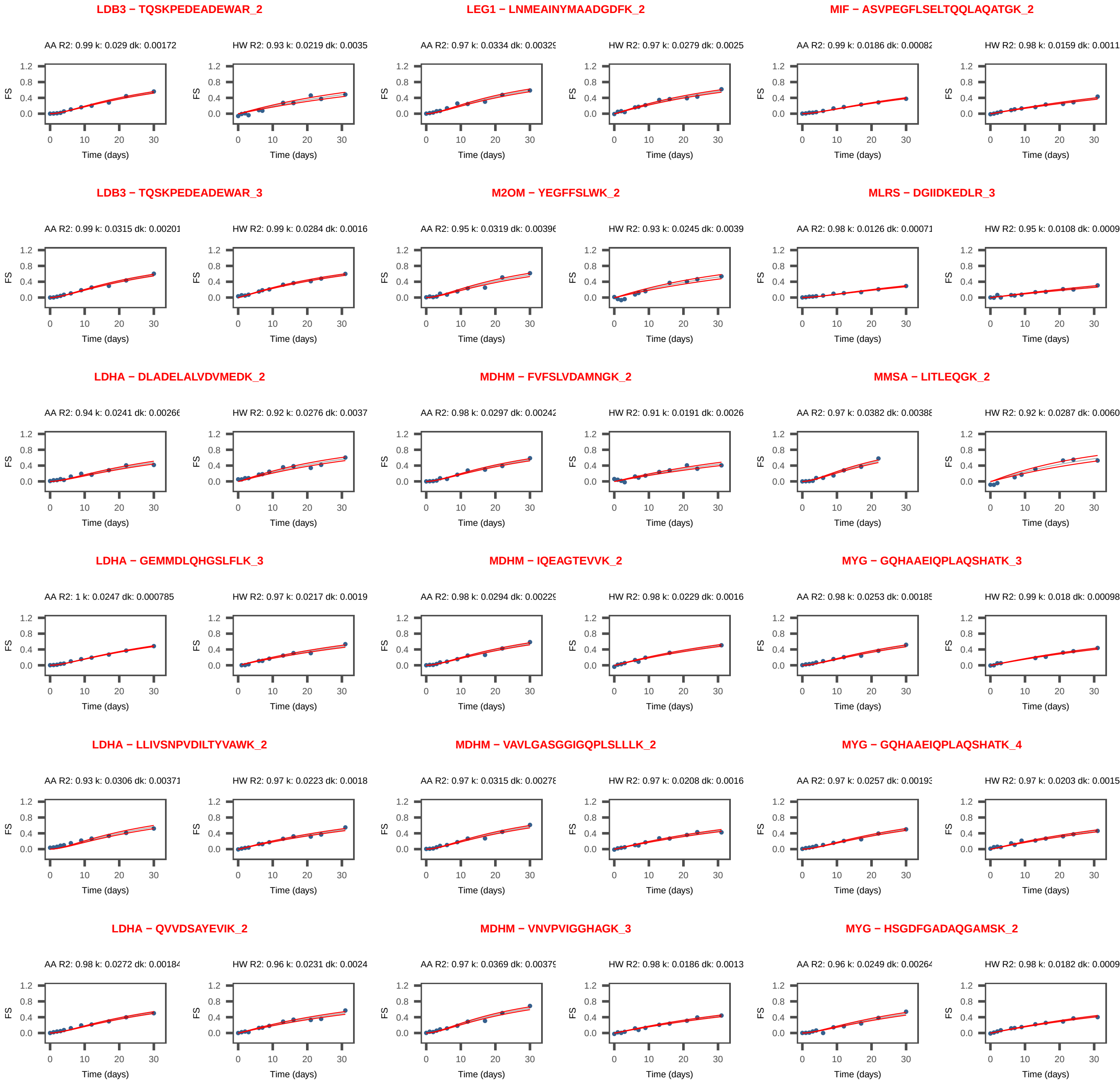
LDB3 − TQSKPEDEADEWAR_2
AA R2: 0.99 k: 0.029 dk: 0.00172
HW R2: 0.93 k: 0.0219 dk: 0.0035
LEG1 − LNMEAINYMAADGDFK_2
AA R2: 0.97 k: 0.0334 dk: 0.00329
HW R2: 0.97 k: 0.0279 dk: 0.0025
MIF − ASVPEGFLSELTQQLAQATGK_2
AA R2: 0.99 k: 0.0186 dk: 0.00082
HW R2: 0.98 k: 0.0159 dk: 0.0011
LDB3 − TQSKPEDEADEWAR_3
AA R2: 0.99 k: 0.0315 dk: 0.00201
HW R2: 0.99 k: 0.0284 dk: 0.0016
M2OM − YEGFFSLWK_2
AA R2: 0.95 k: 0.0319 dk: 0.00396
HW R2: 0.93 k: 0.0245 dk: 0.0039
MLRS − DGIIDKEDLR_3
AA R2: 0.98 k: 0.0126 dk: 0.00071
HW R2: 0.95 k: 0.0108 dk: 0.0009
LDHA − DLADELALVDVMEDK_2
AA R2: 0.94 k: 0.0241 dk: 0.00266
HW R2: 0.92 k: 0.0276 dk: 0.0037
MDHM − FVFSLVDAMNGK_2
AA R2: 0.98 k: 0.0297 dk: 0.00242
HW R2: 0.91 k: 0.0191 dk: 0.0026
MMSA − LITLEQGK_2
AA R2: 0.97 k: 0.0382 dk: 0.00388
HW R2: 0.92 k: 0.0287 dk: 0.0060
LDHA − GEMMDLQHGSLFLK_3
AA R2: 1 k: 0.0247 dk: 0.000785
HW R2: 0.97 k: 0.0217 dk: 0.0019
MDHM − IQEAGTEVVK_2
AA R2: 0.98 k: 0.0294 dk: 0.00229
HW R2: 0.98 k: 0.0229 dk: 0.0016
MYG − GQHAAEIQPLAQSHATK_3
AA R2: 0.98 k: 0.0253 dk: 0.00185
HW R2: 0.99 k: 0.018 dk: 0.00098
LDHA − LLIVSNPVDILTYVAWK_2
AA R2: 0.93 k: 0.0306 dk: 0.00371
HW R2: 0.97 k: 0.0223 dk: 0.0018
MDHM − VAVLGASGGIGQPLSLLLK_2
AA R2: 0.97 k: 0.0315 dk: 0.00278
HW R2: 0.97 k: 0.0208 dk: 0.0016
MYG − GQHAAEIQPLAQSHATK_4
AA R2: 0.97 k: 0.0257 dk: 0.00193
HW R2: 0.97 k: 0.0203 dk: 0.0015
LDHA − QVVDSAYEVIK_2
AA R2: 0.98 k: 0.0272 dk: 0.00184
HW R2: 0.96 k: 0.0231 dk: 0.0024
MDHM − VNVPVIGGHAGK_3
AA R2: 0.97 k: 0.0369 dk: 0.00379
HW R2: 0.98 k: 0.0186 dk: 0.0013
MYG − HSGDFGADAQGAMSK_2
AA R2: 0.96 k: 0.0249 dk: 0.00264
HW R2: 0.98 k: 0.0182 dk: 0.0009
FS
Time (days)

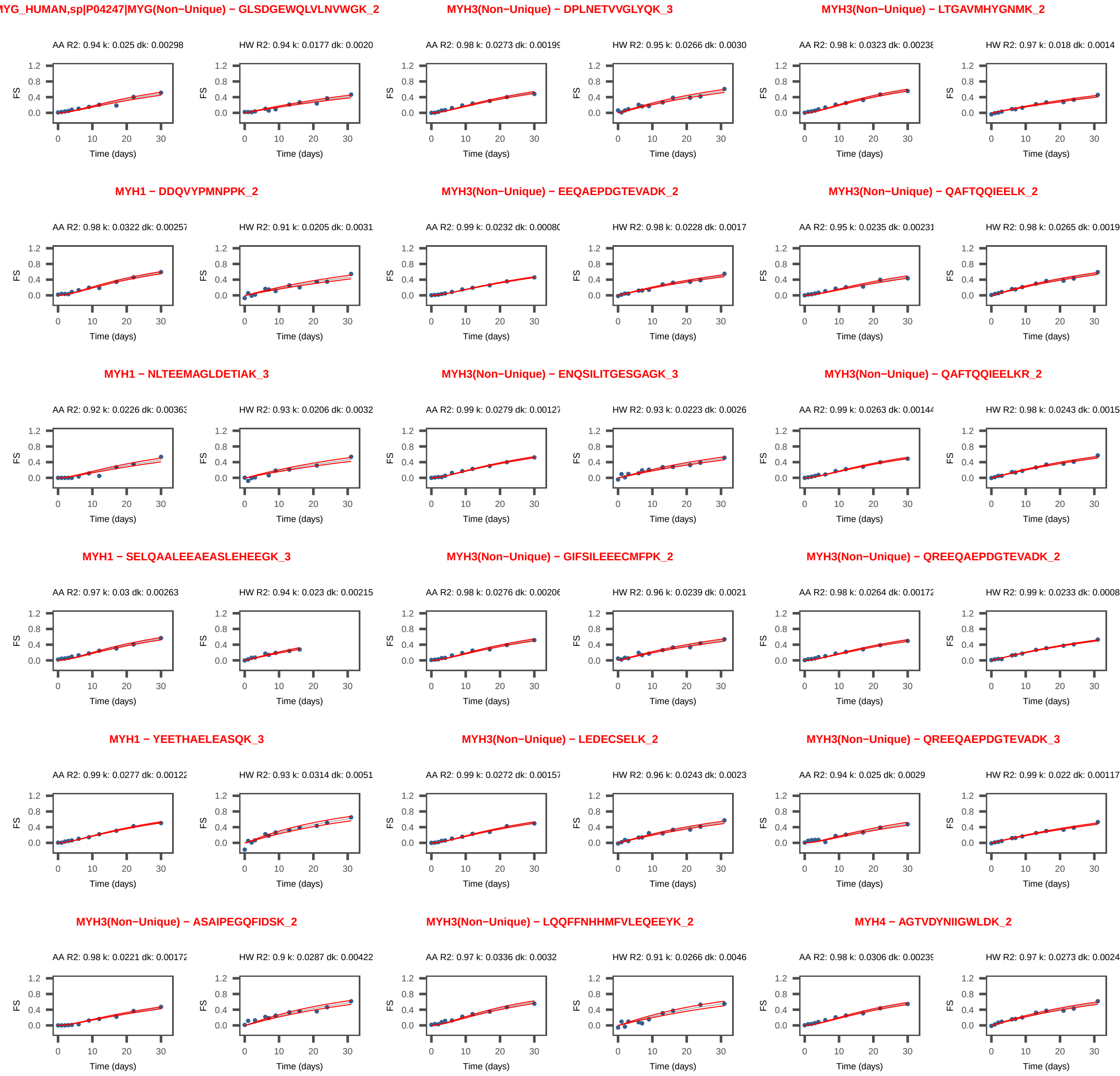
YG_HUMAN,sp|P04247|MYG(Non−Unique) − GLSDGEWQLVLNVWGK_2
AA R2: 0.94 k: 0.025 dk: 0.00298
HW R2: 0.94 k: 0.0177 dk: 0.0020
MYH3(Non−Unique) − DPLNETVVGLYQK_3
AA R2: 0.98 k: 0.0273 dk: 0.0019
HW R2: 0.95 k: 0.0266 dk: 0.0030
MYH3(Non−Unique) − LTGAVMHYGNMK_2
AA R2: 0.98 k: 0.0323 dk: 0.0023
HW R2: 0.97 k: 0.018 dk: 0.0014
MYH1 − DDQVYPMNPPK_2
AA R2: 0.98 k: 0.0322 dk: 0.00257
HW R2: 0.91 k: 0.0205 dk: 0.0031
MYH3(Non−Unique) − EEQAEPDGTEVADK_2
AA R2: 0.99 k: 0.0232 dk: 0.0008
HW R2: 0.98 k: 0.0228 dk: 0.0017
MYH3(Non−Unique) − QAFTQQIEELK_2
AA R2: 0.95 k: 0.0235 dk: 0.00231
HW R2: 0.98 k: 0.0265 dk: 0.0019
MYH1 − NLTEEMAGLDETIAK_3
AA R2: 0.92 k: 0.0226 dk: 0.00363
HW R2: 0.93 k: 0.0206 dk: 0.0032
MYH3(Non−Unique) − ENQSILITGESGAGK_3
AA R2: 0.99 k: 0.0279 dk: 0.00127
HW R2: 0.93 k: 0.0223 dk: 0.0026
MYH3(Non−Unique) − QAFTQQIEELKR_2
AA R2: 0.99 k: 0.0263 dk: 0.0014
HW R2: 0.98 k: 0.0243 dk: 0.0015
MYH1 − SELQAALEEAEASLEHEEGK_3
AA R2: 0.97 k: 0.03 dk: 0.00263
HW R2: 0.94 k: 0.023 dk: 0.00215
MYH3(Non−Unique) − GIFSILEEECMFPK_2
AA R2: 0.98 k: 0.0276 dk: 0.0020
HW R2: 0.96 k: 0.0239 dk: 0.0021
MYH3(Non−Unique) − QREEQAEPDGTEVADK_2
AA R2: 0.98 k: 0.0264 dk: 0.0017
HW R2: 0.99 k: 0.0233 dk: 0.0008
MYH1 − YEETHAELEASQK_3
AA R2: 0.99 k: 0.0277 dk: 0.00122
HW R2: 0.93 k: 0.0314 dk: 0.0051
MYH3(Non−Unique) − LEDECSELK_2
AA R2: 0.99 k: 0.0272 dk: 0.00157
HW R2: 0.96 k: 0.0243 dk: 0.0023
MYH3(Non−Unique) − QREEQAEPDGTEVADK_3
AA R2: 0.94 k: 0.025 dk: 0.0029
HW R2: 0.99 k: 0.022 dk: 0.00117
MYH3(Non−Unique) − ASAIPEGQFIDSK_2
AA R2: 0.98 k: 0.0221 dk: 0.00172
HW R2: 0.9 k: 0.0287 dk: 0.00422
MYH3(Non−Unique) − LQQFFNHHMFVLEQEEYK_2
AA R2: 0.97 k: 0.0336 dk: 0.0032
HW R2: 0.91 k: 0.0266 dk: 0.0046
MYH4 − AGTVDYNIIGWLDK_2
AA R2: 0.98 k: 0.0306 dk: 0.0023
HW R2: 0.97 k: 0.0273 dk: 0.0024
FS
Time (days)

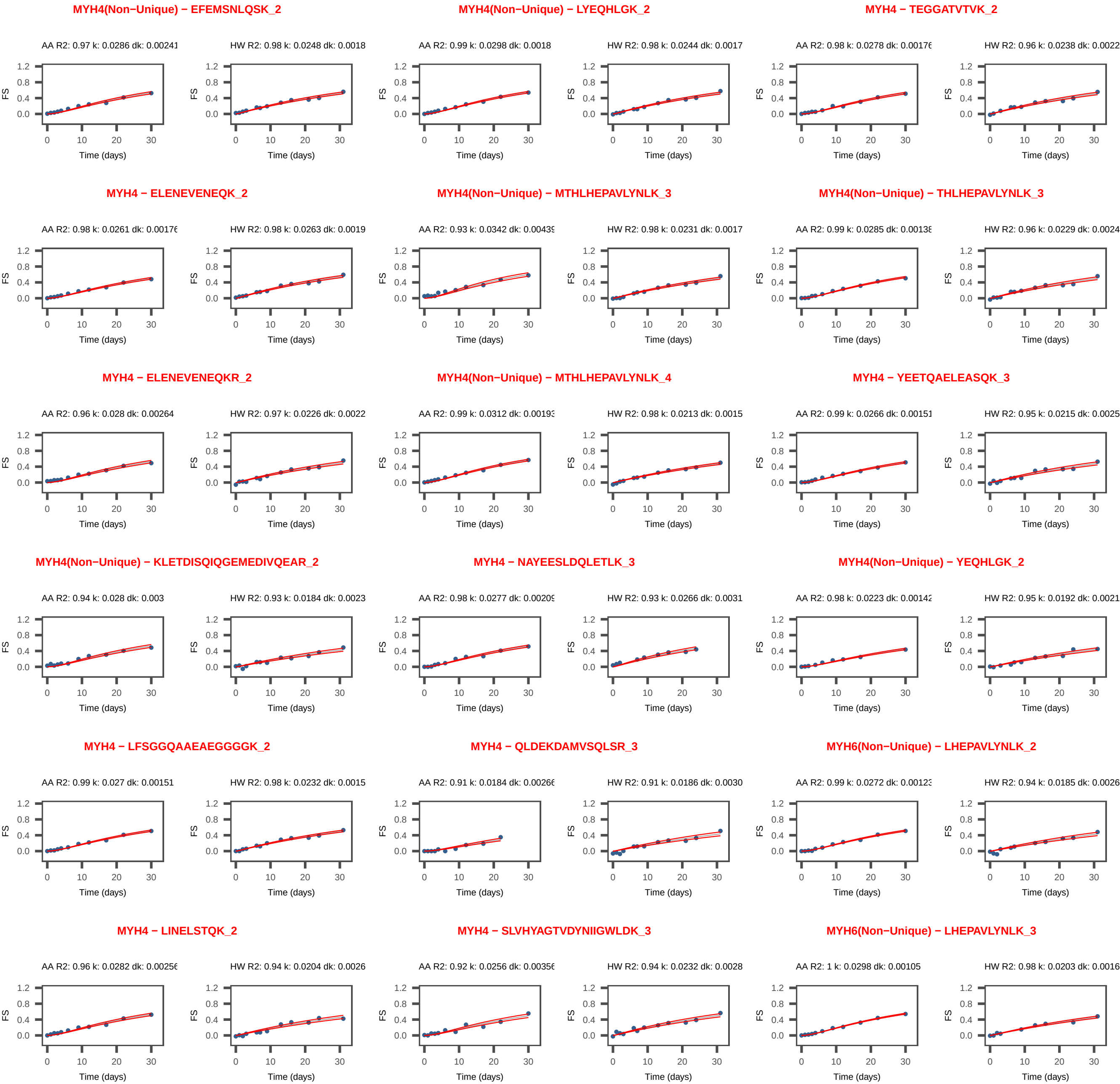

MYH4(Non−Unique) − EFEMSNLQSK_2
AA R2: 0.97 k: 0.0286 dk: 0.00241
HW R2: 0.98 k: 0.0248 dk: 0.0018
MYH4(Non−Unique) − LYEQHLGK_2
AA R2: 0.99 k: 0.0298 dk: 0.0018
HW R2: 0.98 k: 0.0244 dk: 0.0017
MYH4 − TEGGATVTVK_2
AA R2: 0.98 k: 0.0278 dk: 0.00176
HW R2: 0.96 k: 0.0238 dk: 0.0022
MYH4 − ELENEVENEQK_2
AA R2: 0.98 k: 0.0261 dk: 0.00176
HW R2: 0.98 k: 0.0263 dk: 0.0019
MYH4(Non−Unique) − MTHLHEPAVLYNLK_3
AA R2: 0.93 k: 0.0342 dk: 0.00439
HW R2: 0.98 k: 0.0231 dk: 0.0017
MYH4(Non−Unique) − THLHEPAVLYNLK_3
AA R2: 0.99 k: 0.0285 dk: 0.00138
HW R2: 0.96 k: 0.0229 dk: 0.0024
MYH4 − ELENEVENEQKR_2
AA R2: 0.96 k: 0.028 dk: 0.00264
HW R2: 0.97 k: 0.0226 dk: 0.0022
MYH4(Non−Unique) − MTHLHEPAVLYNLK_4
AA R2: 0.99 k: 0.0312 dk: 0.00193
HW R2: 0.98 k: 0.0213 dk: 0.0015
MYH4 − YEETQAELEASQK_3
AA R2: 0.99 k: 0.0266 dk: 0.00151
HW R2: 0.95 k: 0.0215 dk: 0.0025
MYH4(Non−Unique) − KLETDISQIQGEMEDIVQEAR_2
AA R2: 0.94 k: 0.028 dk: 0.003
HW R2: 0.93 k: 0.0184 dk: 0.0023
MYH4 − NAYEESLDQLETLK_3
AA R2: 0.98 k: 0.0277 dk: 0.00209
HW R2: 0.93 k: 0.0266 dk: 0.0031
MYH4(Non−Unique) − YEQHLGK_2
AA R2: 0.98 k: 0.0223 dk: 0.00142
HW R2: 0.95 k: 0.0192 dk: 0.0021
MYH4 − LFSGGQAAEAEGGGGK_2
AA R2: 0.99 k: 0.027 dk: 0.00151
HW R2: 0.98 k: 0.0232 dk: 0.0015
MYH4 − QLDEKDAMVSQLSR_3
AA R2: 0.91 k: 0.0184 dk: 0.00266
HW R2: 0.91 k: 0.0186 dk: 0.0030
MYH6(Non−Unique) − LHEPAVLYNLK_2
AA R2: 0.99 k: 0.0272 dk: 0.00123
HW R2: 0.94 k: 0.0185 dk: 0.0026
MYH4 − LINELSTQK_2
AA R2: 0.96 k: 0.0282 dk: 0.00256
HW R2: 0.94 k: 0.0204 dk: 0.0026
MYH4 − SLVHYAGTVDYNIIGWLDK_3
AA R2: 0.92 k: 0.0256 dk: 0.00356
HW R2: 0.94 k: 0.0232 dk: 0.0028
MYH6(Non−Unique) − LHEPAVLYNLK_3
AA R2: 1 k: 0.0298 dk: 0.00105
HW R2: 0.98 k: 0.0203 dk: 0.0016
FS
Time (days)

MYH7B(Non−Unique) − AITDAAMMAEELK_2

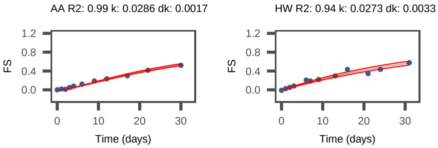

MYH8(Non−Unique) − ELEGEVENEQKR_2

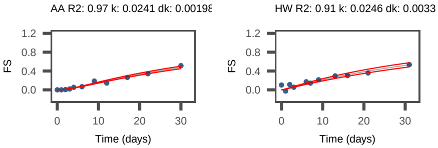

MYH8 − TSVFVAEPK_2

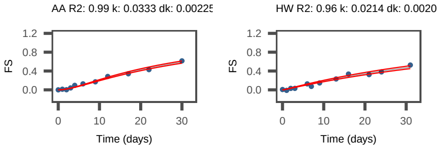

MYH7B(Non−Unique) − AITDAAMMAEELK_3

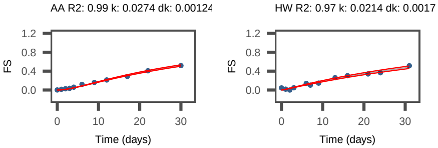

MYH8(Non−Unique) − IQLELNQVK_2

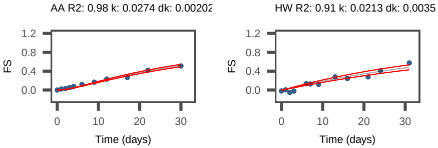

MYH8(Non−Unique) − YDKIEDMAM_2

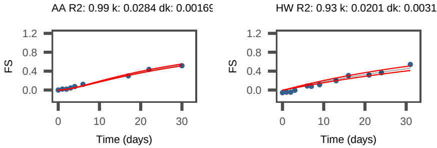

MYH7B(Non−Unique) − KEQDTSAHLER_3

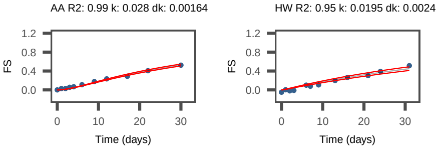

MYH8(Non−Unique) − KIAEQELLDASER_2

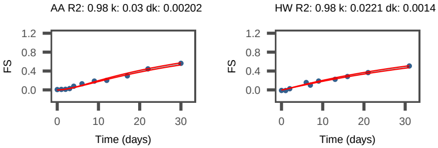

MYL1(Non−Unique) − IDLSAIK_2

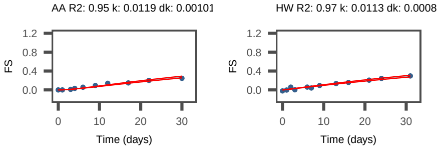

MYH7B(Non−Unique) − MFVLEQEEYK_2

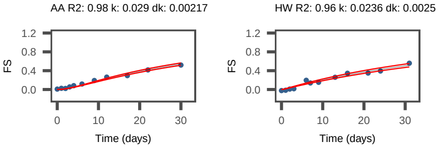

MYH8(Non−Unique) − KLQHELEEAEER_2

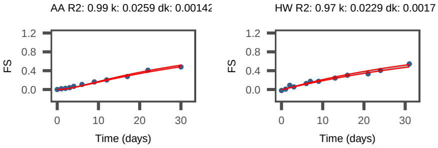

MYL1 − KIEFEQFLPMMQ_2

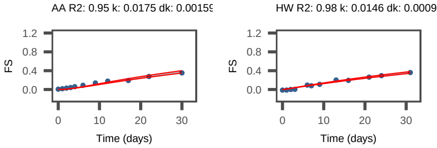

MYH7B(Non−Unique) − SILEEECMFPK_2

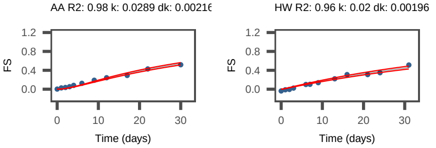

MYH8(Non−Unique) − KLQHELEEAEER_3

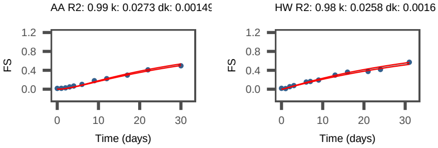

MYL1 − KIEFEQFLPMMQAISN_2

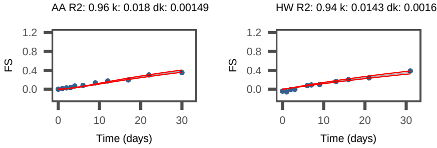

MYH8(Non−Unique) − DEEIDQLKR_3

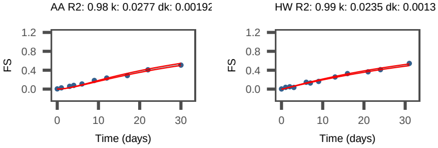

MYH8(Non−Unique) − LLGSIDIDHTQYK_2

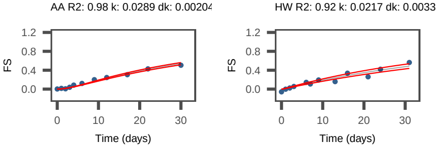

MYOM1 − DAGFYEVILK_2

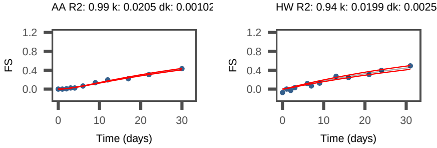

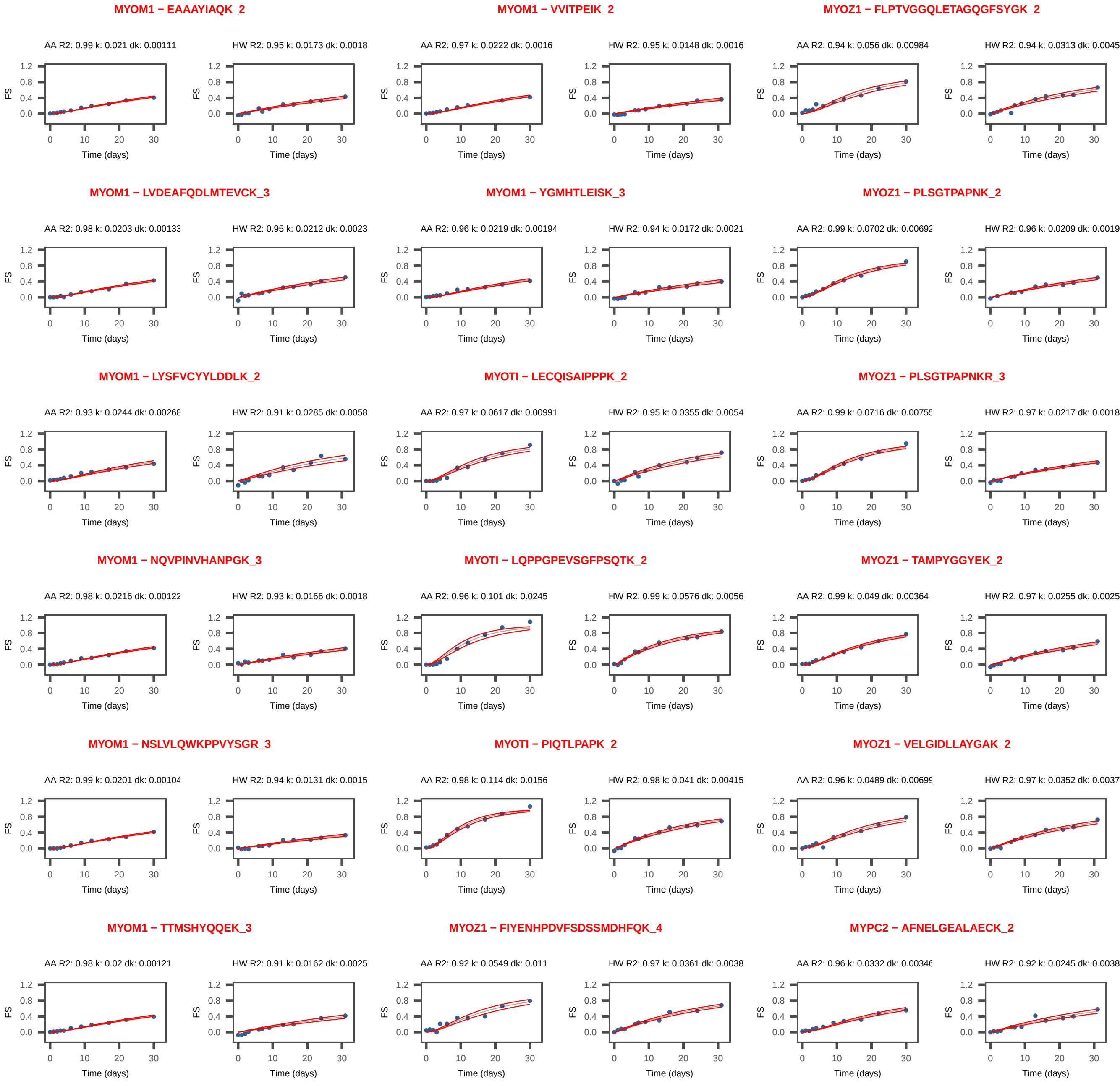
MYOM1 – EAAAYIAQK_2
AA R2: 0.99 k: 0.021 dk: 0.00111
HW R2: 0.95 k: 0.0173 dk: 0.0018
MYOM1 – VVITPEIK_2
AA R2: 0.97 k: 0.0222 dk: 0.0016
HW R2: 0.95 k: 0.0148 dk: 0.0016
MYOZ1 – FLPTVGGQLETAGQGFSYGK_2
AA R2: 0.94 k: 0.056 dk: 0.00984
HW R2: 0.94 k: 0.0313 dk: 0.0045
MYOM1 – LVDEAFQDLMTEVCK_3
AA R2: 0.98 k: 0.0203 dk: 0.0013
HW R2: 0.95 k: 0.0212 dk: 0.0023
MYOM1 – YGMHTLEISK_3
AA R2: 0.96 k: 0.0219 dk: 0.0019
HW R2: 0.94 k: 0.0172 dk: 0.0021
MYOZ1 – PLSGTPAPNK_2
AA R2: 0.99 k: 0.0702 dk: 0.0069
HW R2: 0.96 k: 0.0209 dk: 0.0019
MYOM1 – LYSFVCYYLDDLK_2
AA R2: 0.93 k: 0.0244 dk: 0.0026
HW R2: 0.91 k: 0.0285 dk: 0.0058
MYOTI – LECQISAIPPPK_2
AA R2: 0.97 k: 0.0617 dk: 0.0099
HW R2: 0.95 k: 0.0355 dk: 0.0054
MYOZ1 – PLSGTPAPNKR_3
AA R2: 0.99 k: 0.0716 dk: 0.0075
HW R2: 0.97 k: 0.0217 dk: 0.0018
MYOM1 – NQVPINVHANPGK_3
AA R2: 0.98 k: 0.0216 dk: 0.0012
HW R2: 0.93 k: 0.0166 dk: 0.0018
MYOTI – LQPPGPEVSGFPSQTK_2
AA R2: 0.96 k: 0.101 dk: 0.0245
HW R2: 0.99 k: 0.0576 dk: 0.0056
MYOZ1 – TAMPYGGYEK_2
AA R2: 0.99 k: 0.049 dk: 0.00364
HW R2: 0.97 k: 0.0255 dk: 0.0025
MYOM1 – NSLVLQWKPPVYSGR_3
AA R2: 0.99 k: 0.0201 dk: 0.0010
HW R2: 0.94 k: 0.0131 dk: 0.0015
MYOTI – PIQTLPAPK_2
AA R2: 0.98 k: 0.114 dk: 0.0156
HW R2: 0.98 k: 0.041 dk: 0.00415
MYOZ1 – VELGIDLLAYGAK_2
AA R2: 0.96 k: 0.0489 dk: 0.0069
HW R2: 0.97 k: 0.0352 dk: 0.0037
MYOM1 – TTMSHYQQEK_3
AA R2: 0.98 k: 0.02 dk: 0.00121
HW R2: 0.91 k: 0.0162 dk: 0.0025
MYOZ1 – FIYENHPDVFSDSSMDHFQK_4
AA R2: 0.92 k: 0.0549 dk: 0.011
HW R2: 0.97 k: 0.0361 dk: 0.0038
MYPC2 – AFNELGEALAECK_2
AA R2: 0.96 k: 0.0332 dk: 0.0034
HW R2: 0.92 k: 0.0245 dk: 0.0038
FS
Time (days)

MYPC2 − AFNELGEALAECK_3

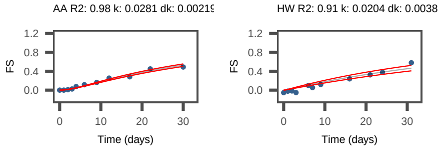

MYPC2 − QLEVLQDIADLTVK_2

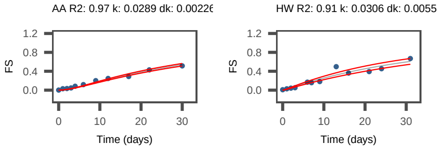

NDKB − DRPFFPGLVK_3

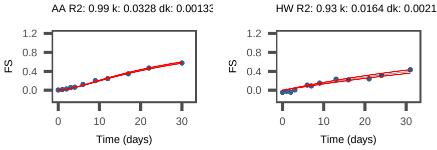

MYPC2 − AGPAENVMVK_2

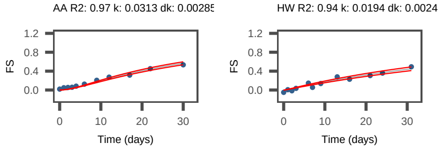

MYPC2 − VFAVNAIGVSQPSMNTK_2

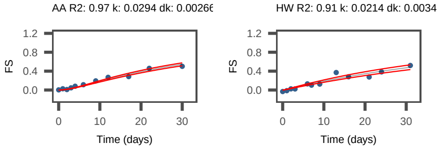

NDRG2 − GWMDWAAHK_3

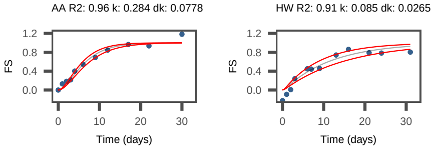

MYPC2 − DDDDLGIPPEIWELLK_2

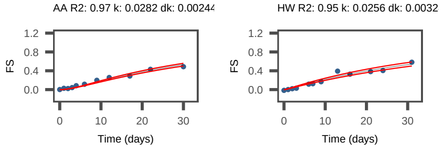

MYPC2 − VGEALNLVIPFQGK_2

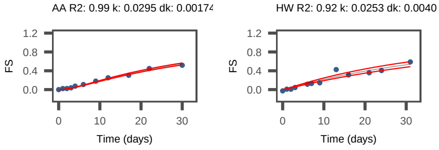

NDRG2 − MADSGGQPQLTQPGK_2

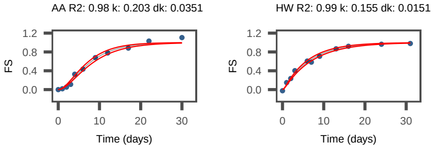

MYPC2 − ESHDSTSNVYTVELHIGK_3

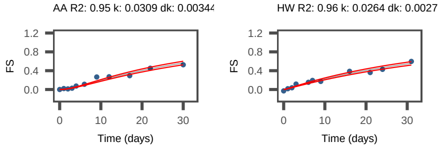

NACAM − AIETLLVSPAK_2

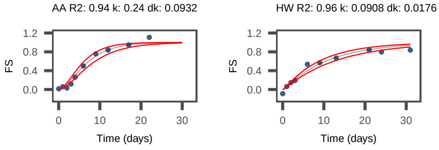

NDRG2 − YALNHPDTVEGLVLINIDPNAK_3

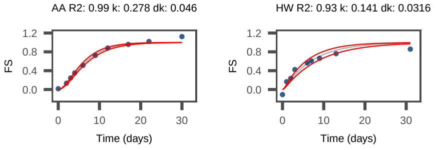

MYPC2 − IFSENICGLSDSPGVSK_2

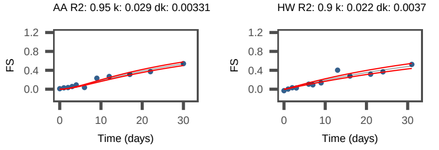

NACAM − ENLAAPAVLPVSSK_2

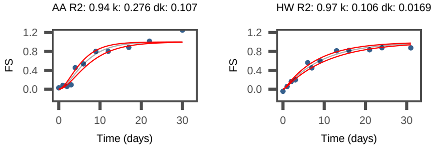

NDUA7 − AAESSAMAATEK_2

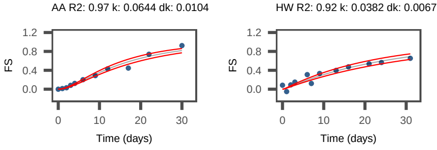

MYPC2 − PQVVWTK_2

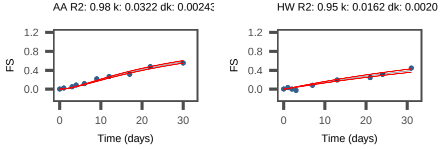

NDKA(Non−Unique) − GLVGEIIK_2

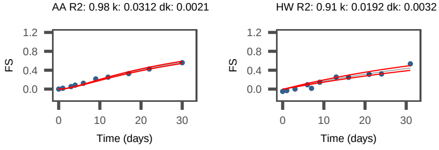

NDUA9 − LFGLSPFEPWTTK_2

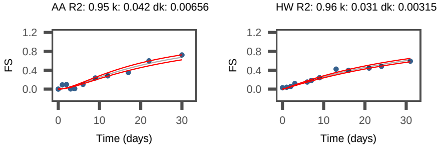

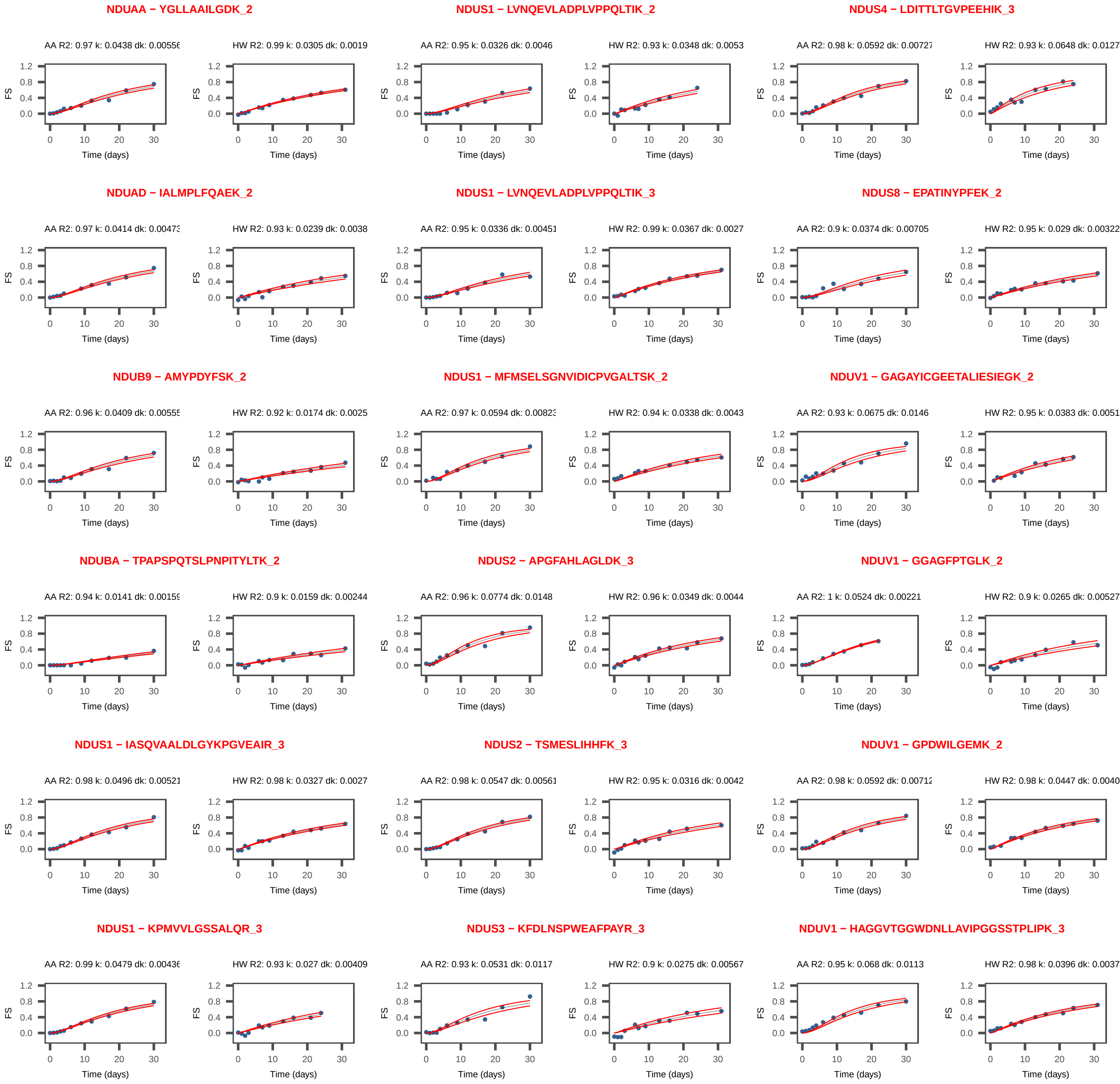
NDUAA – YGLLAAILGDK_2
AA R2: 0.97 k: 0.0438 dk: 0.0055
HW R2: 0.99 k: 0.0305 dk: 0.0019
NDUS1 – LVNQEVLADPLVPPQLTIK_2
AA R2: 0.95 k: 0.0326 dk: 0.0046
HW R2: 0.93 k: 0.0348 dk: 0.0053
NDUS4 – LDITTLTGVPEEHIK_3
AA R2: 0.98 k: 0.0592 dk: 0.0072
HW R2: 0.93 k: 0.0648 dk: 0.0127
NDUAD – IALMPLFQAEK_2
AA R2: 0.97 k: 0.0414 dk: 0.0047
HW R2: 0.93 k: 0.0239 dk: 0.0038
NDUS1 – LVNQEVLADPLVPPQLTIK_3
AA R2: 0.95 k: 0.0336 dk: 0.00451
HW R2: 0.99 k: 0.0367 dk: 0.0027
NDUS8 – EPATINYPFEK_2
AA R2: 0.9 k: 0.0374 dk: 0.00705
HW R2: 0.95 k: 0.029 dk: 0.00322
NDUB9 – AMYPDYFSK_2
AA R2: 0.96 k: 0.0409 dk: 0.0055
HW R2: 0.92 k: 0.0174 dk: 0.0025
NDUS1 – MFMSELSGNVIDICPVGALTSK_2
AA R2: 0.97 k: 0.0594 dk: 0.0082
HW R2: 0.94 k: 0.0338 dk: 0.0043
NDUV1 – GAGAYICGEETALIESIEGK_2
AA R2: 0.93 k: 0.0675 dk: 0.0146
HW R2: 0.95 k: 0.0383 dk: 0.0051
NDUBA – TPAPSPQTSLPNPITYLTK_2
AA R2: 0.94 k: 0.0141 dk: 0.0015
HW R2: 0.9 k: 0.0159 dk: 0.00244
NDUS2 – APGFAHLAGLDK_3
AA R2: 0.96 k: 0.0774 dk: 0.0148
HW R2: 0.96 k: 0.0349 dk: 0.0044
NDUV1 – GGAGFPTGLK_2
AA R2: 1 k: 0.0524 dk: 0.00221
HW R2: 0.9 k: 0.0265 dk: 0.00527
NDUS1 – IASQVAALDLGYKPGVEAIR_3
AA R2: 0.98 k: 0.0496 dk: 0.00521
HW R2: 0.98 k: 0.0327 dk: 0.0027
NDUS2 – TSMESLIHHFK_3
AA R2: 0.98 k: 0.0547 dk: 0.00561
HW R2: 0.95 k: 0.0316 dk: 0.0042
NDUV1 – GPDWILGEMK_2
AA R2: 0.98 k: 0.0592 dk: 0.0071
HW R2: 0.98 k: 0.0447 dk: 0.0040
NDUS1 – KPMVVLGSSALQR_3
AA R2: 0.99 k: 0.0479 dk: 0.0043
HW R2: 0.93 k: 0.027 dk: 0.00409
NDUS3 – KFDLNSPWEAFPAYR_3
AA R2: 0.93 k: 0.0531 dk: 0.0117
HW R2: 0.9 k: 0.0275 dk: 0.00567
NDUV1 – HAGGVTGGWDNLLAVIPGGSSTPLIPK_3
AA R2: 0.95 k: 0.068 dk: 0.0113
HW R2: 0.98 k: 0.0396 dk: 0.0037

OBSCN – ADAGEYSCEAGGQK_2

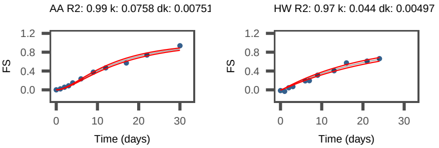

ODPB – VFLLGEEVAQYDGAYK_2

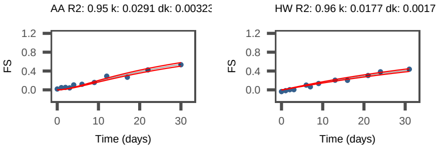

PFKAM – LNIIIVAEGAIDK_2

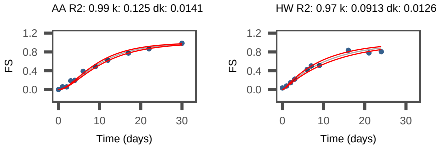

OBSCN – IAPADYGPVHWFLDK_3

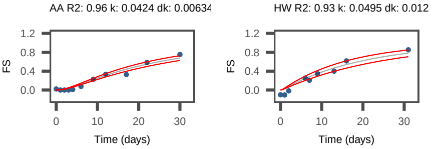

ODPB – VLEDNSVPQVK_2

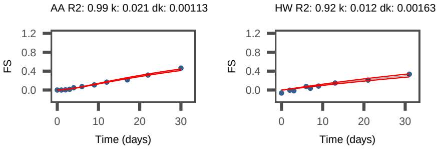

PFKAM – RFDEAIK_2

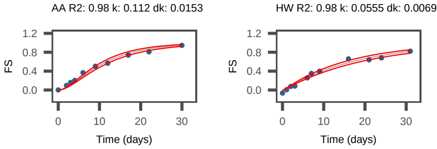

ODO1 – FGLEGCEVLIPALK_2

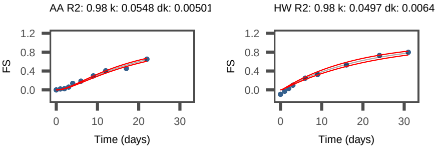

PDLI5 – DFNMPLTISSLK_2

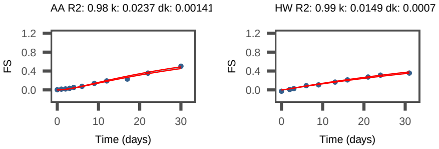

PFKAM – VLVVHDGFEGLAK_2

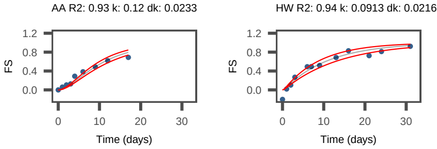

ODO1 – IEQLSPFPFDLLLK_2

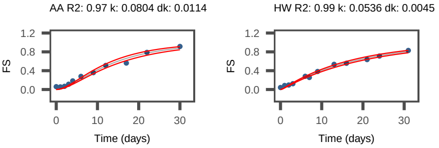

PFKAM – ALVFQPVTELK_2

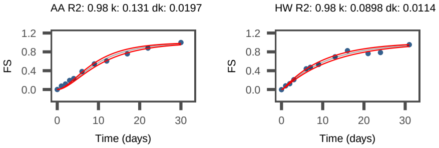

PFKAM – VLVVHDGFEGLAK_3

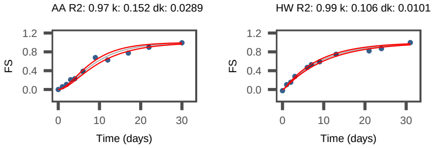

ODO1 – VIPENGPAAQDPHK_3

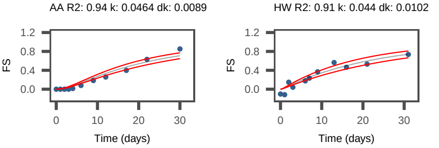

PFKAM – DLQVNVEHLVQK_3

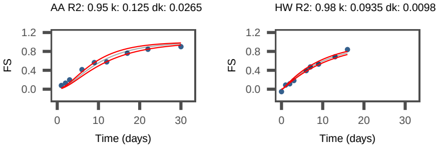

PGAM2 – SFDTPPPPMDEK_2

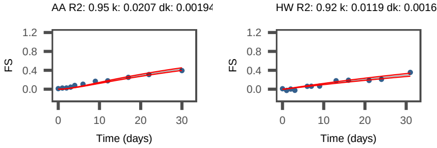

ODP2 – GLETIASDVVSLASK_2

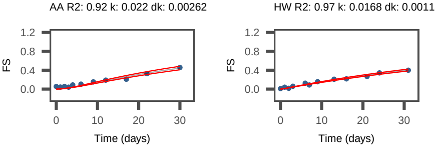

PFKAM – GQIEEAGWSYVGGWTGQGGSK_2

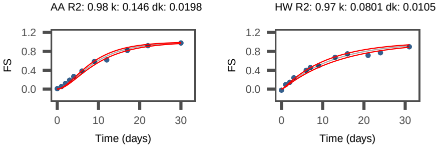

PGK1 – ALESPERPFLAILGGAK_3

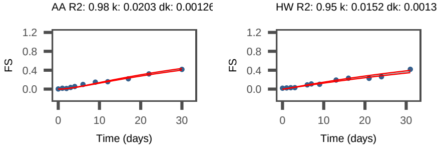

PGK1 − IQLINNMLDK_2

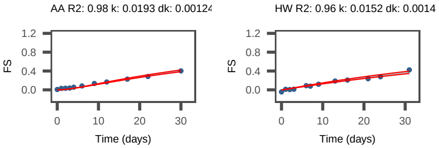

PGS2 − DLHTLILVNNK_3

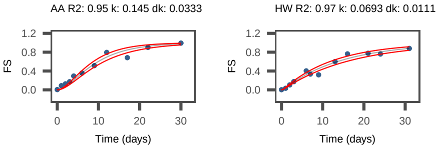

PRDX6 − DLAILLGMLDPVEK_2

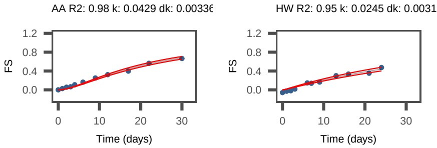

PGM1 − ADNFEYSDPVDGSISK_2

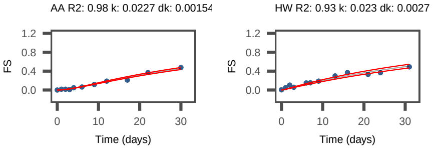

PLEC − LLLEAQAATGFLLDPVK_2

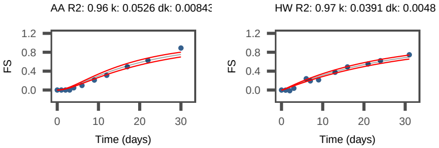

PROF1 − TFVSITPAEVGVLVGK_2

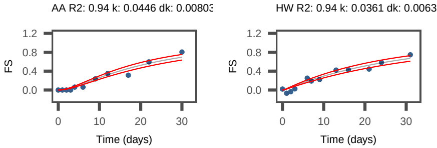

PGM1 − INQDPQVMLAPLISIALK_2

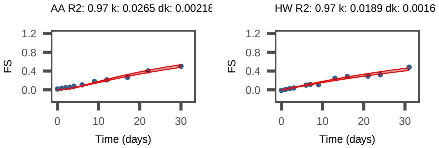

PP1A(Non−Unique) − AHQVVEDGYEFFAK_3

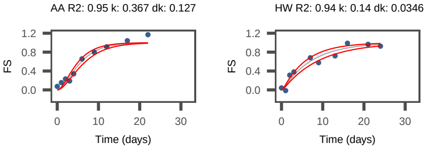

PSA5 − LFQVEYAIEAIK_2

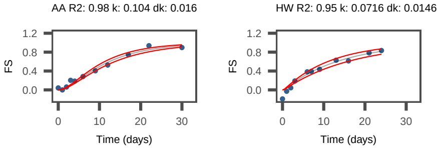

PGM1 − INQDPQVMLAPLISIALK_3

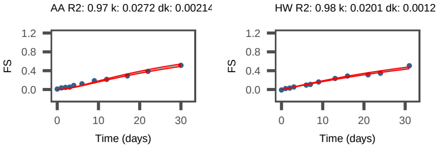

PRDX2 − SLSQNYGVLK_2

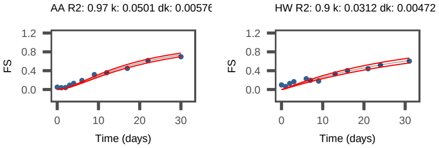

PURA1 − DGVYFMYEALHGPPK_3

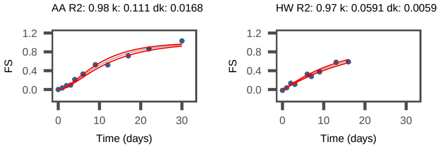

PGM1 − YDYEEVEAEGANK_2

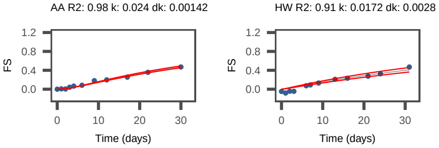

PRDX3 − GLFIIDPNGVVK_2

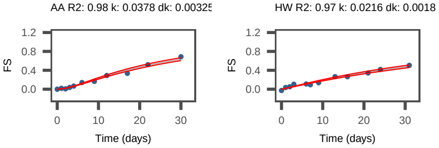

PYGB(Non−Unique) − YEFGIFNQK_2

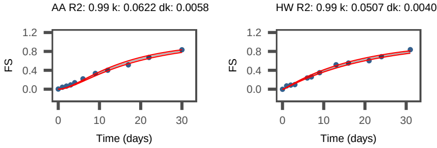

PGM5(Non−Unique) − NIFDFNAIK_2

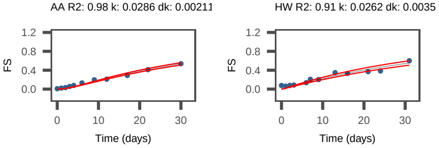

PRDX4(Non−Unique) − GLFIIDDK_2

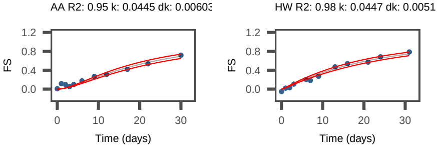

PYGB(Non−Unique) − YGNPWEK_2

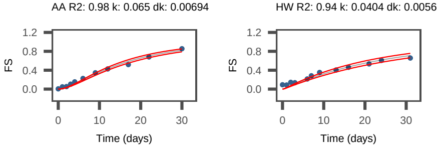

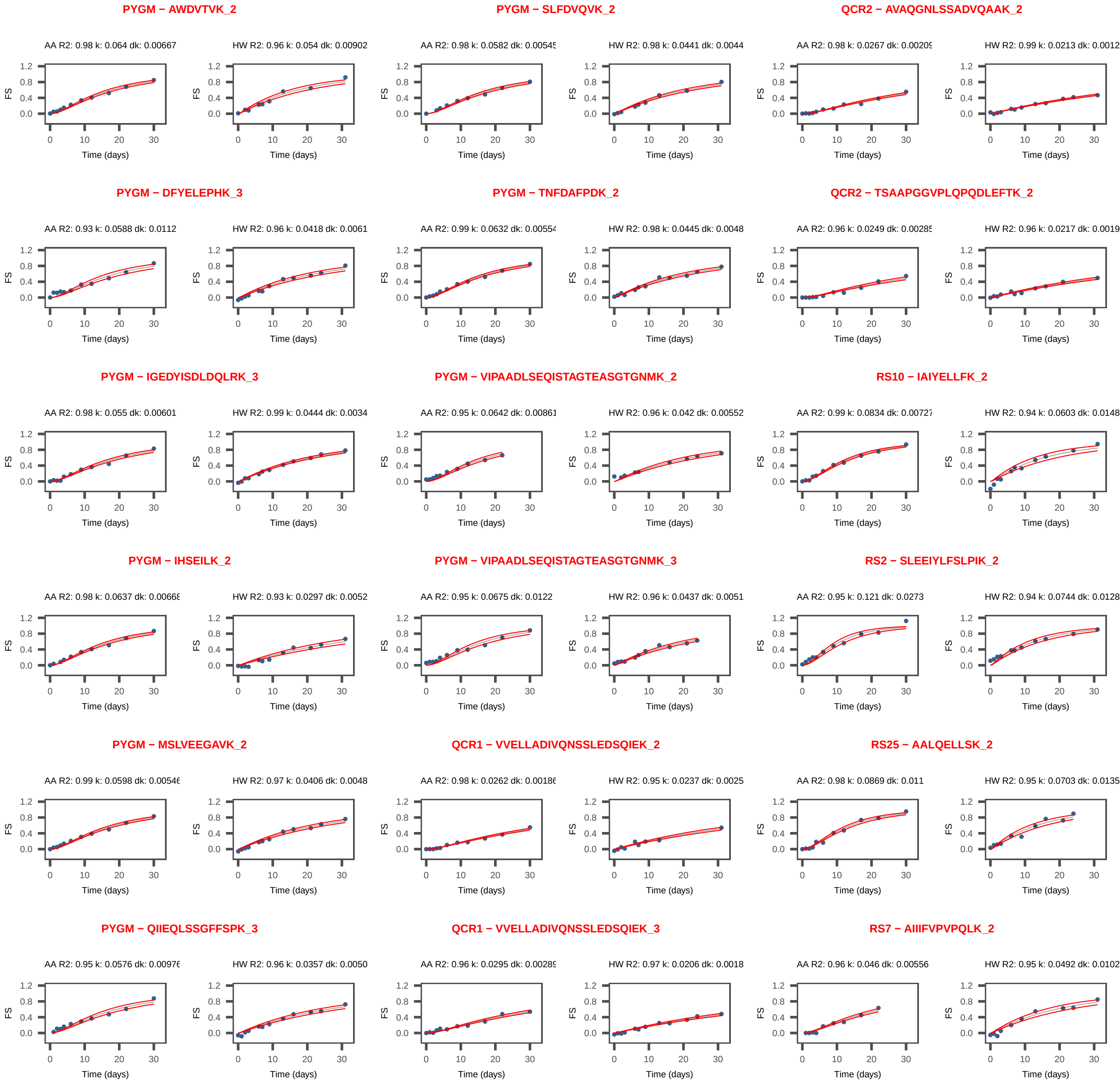
PYGM − AWDVTVK_2
AA R2: 0.98 k: 0.064 dk: 0.00667
HW R2: 0.96 k: 0.054 dk: 0.00902
PYGM − SLFDVQVK_2
AA R2: 0.98 k: 0.0582 dk: 0.00545
HW R2: 0.98 k: 0.0441 dk: 0.0044
QCR2 − AVAQGNLSSADVQAAK_2
AA R2: 0.98 k: 0.0267 dk: 0.00209
HW R2: 0.99 k: 0.0213 dk: 0.0012
PYGM − DFYELEPHK_3
AA R2: 0.93 k: 0.0588 dk: 0.0112
HW R2: 0.96 k: 0.0418 dk: 0.0061
PYGM − TNFDAFPDK_2
AA R2: 0.99 k: 0.0632 dk: 0.00554
HW R2: 0.98 k: 0.0445 dk: 0.0048
QCR2 − TSAAPGGVPLQPQDLEFTK_2
AA R2: 0.96 k: 0.0249 dk: 0.00285
HW R2: 0.96 k: 0.0217 dk: 0.0019
PYGM − IGEDYISDLDQLRK_3
AA R2: 0.98 k: 0.055 dk: 0.00601
HW R2: 0.99 k: 0.0444 dk: 0.0034
PYGM − VIPAADLSEQISTAGTEASGTGNMK_2
AA R2: 0.95 k: 0.0642 dk: 0.00861
HW R2: 0.96 k: 0.042 dk: 0.00552
RS10 − IAIYELLFK_2
AA R2: 0.99 k: 0.0834 dk: 0.00727
HW R2: 0.94 k: 0.0603 dk: 0.0148
PYGM − IHSEILK_2
AA R2: 0.98 k: 0.0637 dk: 0.00668
HW R2: 0.93 k: 0.0297 dk: 0.0052
PYGM − VIPAADLSEQISTAGTEASGTGNMK_3
AA R2: 0.95 k: 0.0675 dk: 0.0122
HW R2: 0.96 k: 0.0437 dk: 0.0051
RS2 − SLEEIYLFSLPIK_2
AA R2: 0.95 k: 0.121 dk: 0.0273
HW R2: 0.94 k: 0.0744 dk: 0.0128
PYGM − MSLVEEGAVK_2
AA R2: 0.99 k: 0.0598 dk: 0.00546
HW R2: 0.97 k: 0.0406 dk: 0.0048
QCR1 − VVELLADIVQNSSLEDSQIEK_2
AA R2: 0.98 k: 0.0262 dk: 0.00186
HW R2: 0.95 k: 0.0237 dk: 0.0025
RS25 − AALQELLSK_2
AA R2: 0.98 k: 0.0869 dk: 0.011
HW R2: 0.95 k: 0.0703 dk: 0.0135
PYGM − QIIEQLSSGFFSPK_3
AA R2: 0.95 k: 0.0576 dk: 0.00976
HW R2: 0.96 k: 0.0357 dk: 0.0050
QCR1 − VVELLADIVQNSSLEDSQIEK_3
AA R2: 0.96 k: 0.0295 dk: 0.00289
HW R2: 0.97 k: 0.0206 dk: 0.0018
RS7 − AIIIFVPVPQLK_2
AA R2: 0.96 k: 0.046 dk: 0.00556
HW R2: 0.95 k: 0.0492 dk: 0.0102
FS
Time (days)

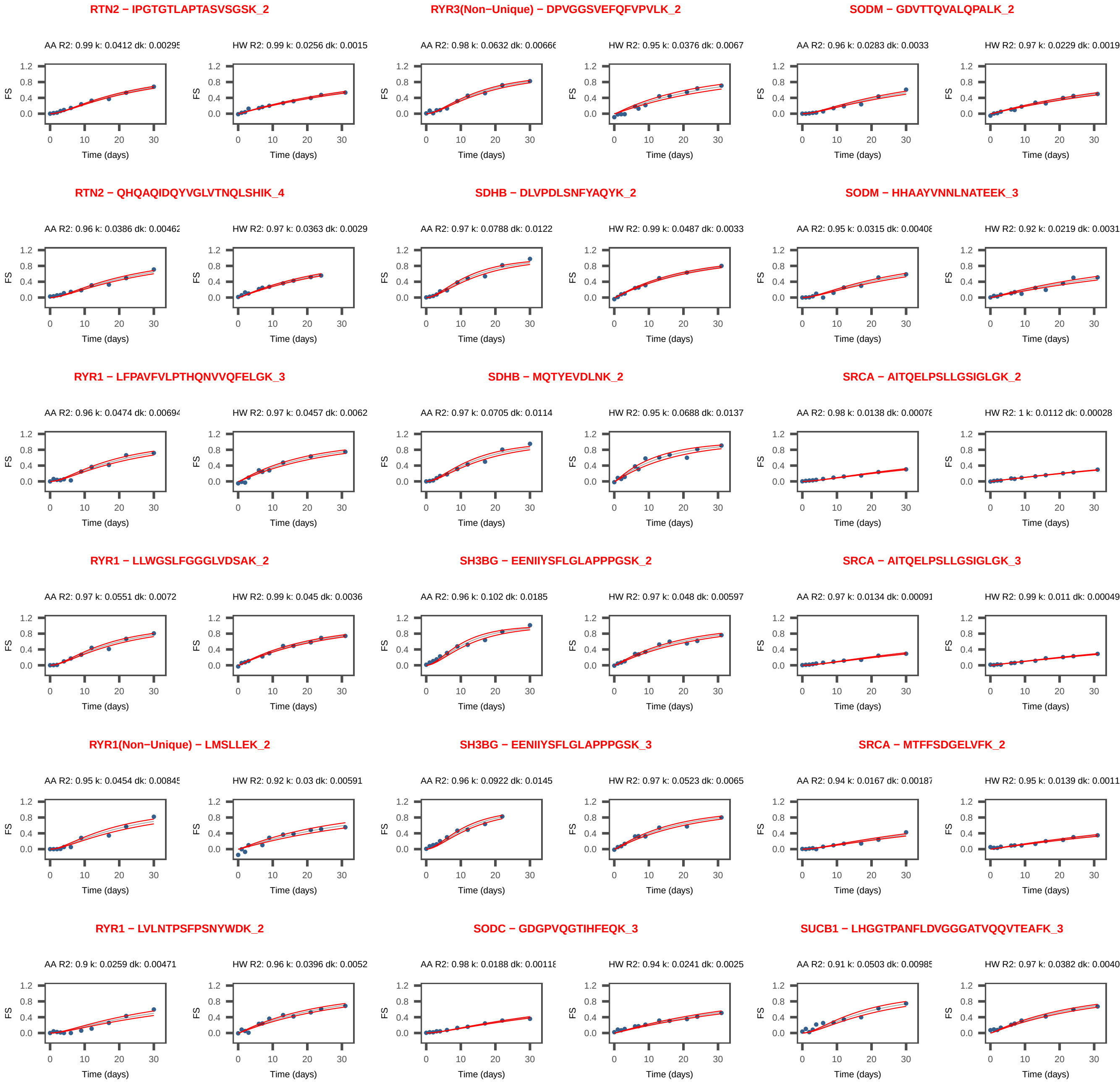
RTN2 − IPGTGTLAPTASVSGSK_2
AA R2: 0.99 k: 0.0412 dk: 0.00295
HW R2: 0.99 k: 0.0256 dk: 0.0015
RYR3(Non−Unique) − DPVGGSVEFQFVPVLK_2
AA R2: 0.98 k: 0.0632 dk: 0.0066
HW R2: 0.95 k: 0.0376 dk: 0.0067
SODM − GDVTTQVALQPALK_2
AA R2: 0.96 k: 0.0283 dk: 0.0033
HW R2: 0.97 k: 0.0229 dk: 0.0019
RTN2 − QHQAQIDQYVGLVTNQLSHIK_4
AA R2: 0.96 k: 0.0386 dk: 0.00462
HW R2: 0.97 k: 0.0363 dk: 0.0029
SDHB − DLVPDLSNFYAQYK_2
AA R2: 0.97 k: 0.0788 dk: 0.0122
HW R2: 0.99 k: 0.0487 dk: 0.0033
SODM − HHAAYVNNLNATEEK_3
AA R2: 0.95 k: 0.0315 dk: 0.0040
HW R2: 0.92 k: 0.0219 dk: 0.0031
RYR1 − LFPAVFVLPTHQNVVQFELGK_3
AA R2: 0.96 k: 0.0474 dk: 0.00694
HW R2: 0.97 k: 0.0457 dk: 0.0062
SDHB − MQTYEVDLNK_2
AA R2: 0.97 k: 0.0705 dk: 0.0114
HW R2: 0.95 k: 0.0688 dk: 0.0137
SRCA − AITQELPSLLGSIGLGK_2
AA R2: 0.98 k: 0.0138 dk: 0.0007
HW R2: 1 k: 0.0112 dk: 0.00028
RYR1 − LLWGSLFGGGLVDSAK_2
AA R2: 0.97 k: 0.0551 dk: 0.0072
HW R2: 0.99 k: 0.045 dk: 0.0036
SH3BG − EENIIYSFLGLAPPPGSK_2
AA R2: 0.96 k: 0.102 dk: 0.0185
HW R2: 0.97 k: 0.048 dk: 0.00597
SRCA − AITQELPSLLGSIGLGK_3
AA R2: 0.97 k: 0.0134 dk: 0.00091
HW R2: 0.99 k: 0.011 dk: 0.00049
RYR1(Non−Unique) − LMSLLEK_2
AA R2: 0.95 k: 0.0454 dk: 0.0084
HW R2: 0.92 k: 0.03 dk: 0.00591
SH3BG − EENIIYSFLGLAPPPGSK_3
AA R2: 0.96 k: 0.0922 dk: 0.0145
HW R2: 0.97 k: 0.0523 dk: 0.0065
SRCA − MTFFSDGELVFK_2
AA R2: 0.94 k: 0.0167 dk: 0.00187
HW R2: 0.95 k: 0.0139 dk: 0.0011
RYR1 − LVLNTPSFPSNYWDK_2
AA R2: 0.9 k: 0.0259 dk: 0.00471
HW R2: 0.96 k: 0.0396 dk: 0.0052
SODC − GDGPVQGTIHFEQK_3
AA R2: 0.98 k: 0.0188 dk: 0.0011
HW R2: 0.94 k: 0.0241 dk: 0.0025
SUCB1 − LHGGTPANFLDVGGGATVQQVTEAFK_3
AA R2: 0.91 k: 0.0503 dk: 0.0098
HW R2: 0.97 k: 0.0382 dk: 0.0040

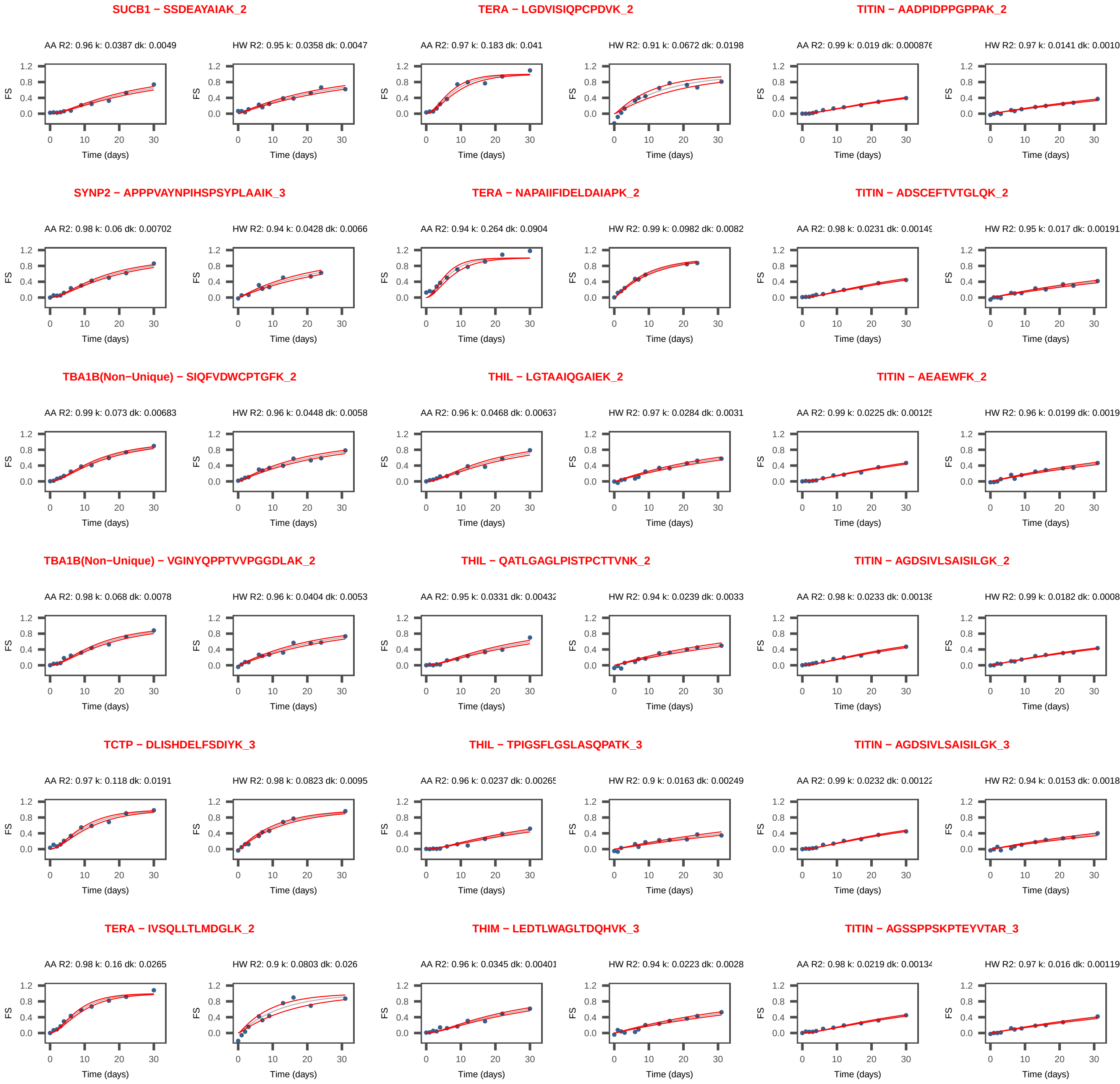
SUCB1 − SSDEAYAIAK_2
AA R2: 0.96 k: 0.0387 dk: 0.0049
HW R2: 0.95 k: 0.0358 dk: 0.0047
TERA − LGDVISIQPCPDVK_2
AA R2: 0.97 k: 0.183 dk: 0.041
HW R2: 0.91 k: 0.0672 dk: 0.0198
TITIN − AADPIDPPGPPAK_2
AA R2: 0.99 k: 0.019 dk: 0.00087
HW R2: 0.97 k: 0.0141 dk: 0.0010
SYNP2 − APPPVAYNPIHSPSYPLAAIK_3
AA R2: 0.98 k: 0.06 dk: 0.00702
HW R2: 0.94 k: 0.0428 dk: 0.0066
TERA − NAPAIIFIDELDAIAPK_2
AA R2: 0.94 k: 0.264 dk: 0.0904
HW R2: 0.99 k: 0.0982 dk: 0.0082
TITIN − ADSCEFTVTGLQK_2
AA R2: 0.98 k: 0.0231 dk: 0.0014
HW R2: 0.95 k: 0.017 dk: 0.00191
TBA1B(Non−Unique) − SIQFVDWCPTGFK_2
AA R2: 0.99 k: 0.073 dk: 0.00683
HW R2: 0.96 k: 0.0448 dk: 0.0058
THIL − LGTAAIQGAIEK_2
AA R2: 0.96 k: 0.0468 dk: 0.00637
HW R2: 0.97 k: 0.0284 dk: 0.0031
TITIN − AEAEWFK_2
AA R2: 0.99 k: 0.0225 dk: 0.00125
HW R2: 0.96 k: 0.0199 dk: 0.0019
TBA1B(Non−Unique) − VGINYQPPTVVPGGDLAK_2
AA R2: 0.98 k: 0.068 dk: 0.0078
HW R2: 0.96 k: 0.0404 dk: 0.0053
THIL − QATLGAGLPISTPCTTVNK_2
AA R2: 0.95 k: 0.0331 dk: 0.00432
HW R2: 0.94 k: 0.0239 dk: 0.0033
TITIN − AGDSIVLSAISILGK_2
AA R2: 0.98 k: 0.0233 dk: 0.00138
HW R2: 0.99 k: 0.0182 dk: 0.0008
TCTP − DLISHDELFSDIYK_3
AA R2: 0.97 k: 0.118 dk: 0.0191
HW R2: 0.98 k: 0.0823 dk: 0.0095
THIL − TPIGSFLGSLASQPATK_3
AA R2: 0.96 k: 0.0237 dk: 0.00265
HW R2: 0.9 k: 0.0163 dk: 0.00249
TITIN − AGDSIVLSAISILGK_3
AA R2: 0.99 k: 0.0232 dk: 0.00122
HW R2: 0.94 k: 0.0153 dk: 0.0018
TERA − IVSQLLTLMDGLK_2
AA R2: 0.98 k: 0.16 dk: 0.0265
HW R2: 0.9 k: 0.0803 dk: 0.026
THIM − LEDTLWAGLTDQHVK_3
AA R2: 0.96 k: 0.0345 dk: 0.00401
HW R2: 0.94 k: 0.0223 dk: 0.0028
TITIN − AGSSPPSKPTEYVTAR_3
AA R2: 0.98 k: 0.0219 dk: 0.00134
HW R2: 0.97 k: 0.016 dk: 0.00119
FS
Time (days)

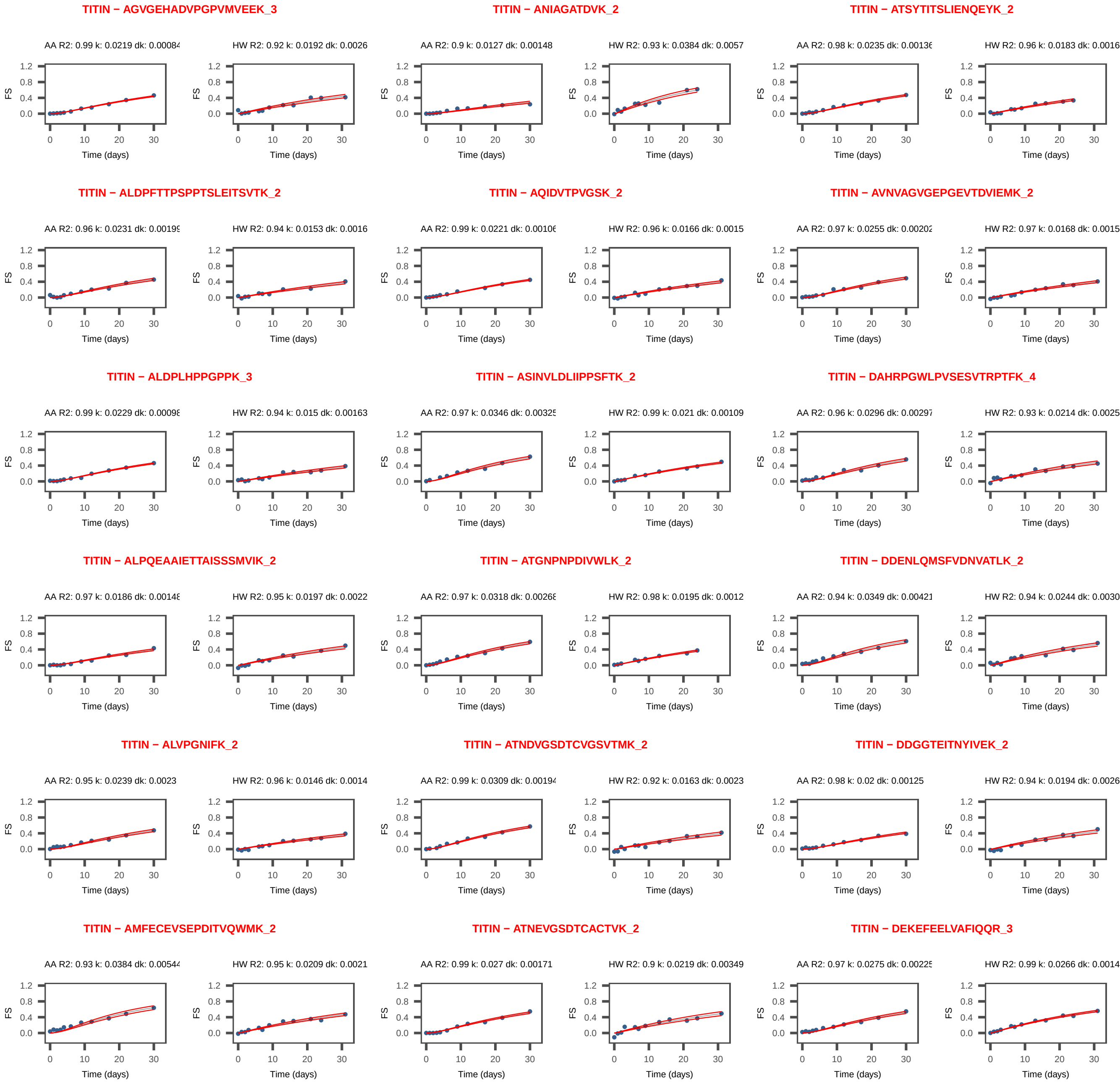
TITIN − AGVGEHADVPGPVMVEEK_3
AA R2: 0.99 k: 0.0219 dk: 0.00084
HW R2: 0.92 k: 0.0192 dk: 0.0026
TITIN − ANIAGATDVK_2
AA R2: 0.9 k: 0.0127 dk: 0.00148
HW R2: 0.93 k: 0.0384 dk: 0.0057
TITIN − ATSYTITSLIENQEYK_2
AA R2: 0.98 k: 0.0235 dk: 0.0013
HW R2: 0.96 k: 0.0183 dk: 0.0016
TITIN − ALDPFTTPSPPTSLEITSVTK_2
AA R2: 0.96 k: 0.0231 dk: 0.0019
HW R2: 0.94 k: 0.0153 dk: 0.0016
TITIN − AQIDVTPVGSK_2
AA R2: 0.99 k: 0.0221 dk: 0.0010
HW R2: 0.96 k: 0.0166 dk: 0.0015
TITIN − AVNVAGVGEPGEVTDVIEMK_2
AA R2: 0.97 k: 0.0255 dk: 0.0020
HW R2: 0.97 k: 0.0168 dk: 0.0015
TITIN − ALDPLHPPGPPK_3
AA R2: 0.99 k: 0.0229 dk: 0.0009
HW R2: 0.94 k: 0.015 dk: 0.00163
TITIN − ASINVLDLIIPPSFTK_2
AA R2: 0.97 k: 0.0346 dk: 0.0032
HW R2: 0.99 k: 0.021 dk: 0.00109
TITIN − DAHRPGWLPVSESVTRPTFK_4
AA R2: 0.96 k: 0.0296 dk: 0.0029
HW R2: 0.93 k: 0.0214 dk: 0.0025
TITIN − ALPQEAAIETTAISSSMVIK_2
AA R2: 0.97 k: 0.0186 dk: 0.0014
HW R2: 0.95 k: 0.0197 dk: 0.0022
TITIN − ATGNPNPDIVWLK_2
AA R2: 0.97 k: 0.0318 dk: 0.0026
HW R2: 0.98 k: 0.0195 dk: 0.0012
TITIN − DDENLQMSFVDNVATLK_2
AA R2: 0.94 k: 0.0349 dk: 0.00421
HW R2: 0.94 k: 0.0244 dk: 0.0030
TITIN − ALVPGNIFK_2
AA R2: 0.95 k: 0.0239 dk: 0.0023
HW R2: 0.96 k: 0.0146 dk: 0.0014
TITIN − ATNDVGSDTCVGSVTMK_2
AA R2: 0.99 k: 0.0309 dk: 0.0019
HW R2: 0.92 k: 0.0163 dk: 0.0023
TITIN − DDGGTEITNYIVEK_2
AA R2: 0.98 k: 0.02 dk: 0.00125
HW R2: 0.94 k: 0.0194 dk: 0.0026
TITIN − AMFECEVSEPDITVQWMK_2
AA R2: 0.93 k: 0.0384 dk: 0.0054
HW R2: 0.95 k: 0.0209 dk: 0.0021
TITIN − ATNEVGSDTCACTVK_2
AA R2: 0.99 k: 0.027 dk: 0.00171
HW R2: 0.9 k: 0.0219 dk: 0.00349
TITIN − DEKEFEELVAFIQQR_3
AA R2: 0.97 k: 0.0275 dk: 0.0022
HW R2: 0.99 k: 0.0266 dk: 0.0014
FS
Time (days)

TITIN − DENVPPTVEFGPEYFDGLVIK_2

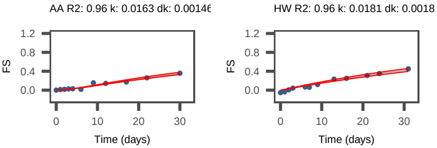

TITIN − DSVHLTWEPPDDDGGSPLTGYVVEK_3

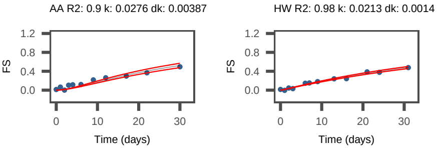

TITIN − EEQLLSTGFK_2

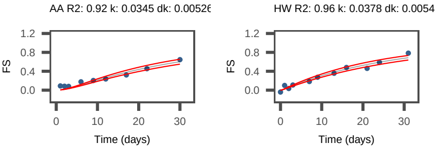

TITIN − DENVPPTVEFGPEYFDGLVIK_3

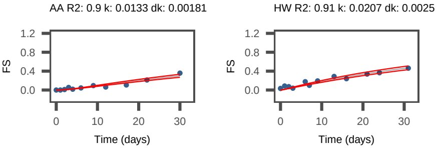

TITIN − DSVLLVWEPPIIDGGAK_2

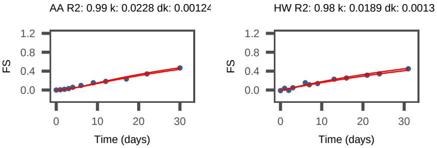

TITIN − EIELDFAVPLK_2

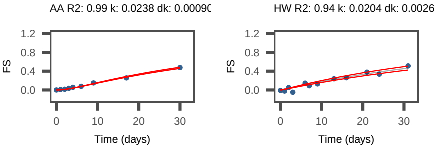

TITIN − DGLEVIVPNPIK_2

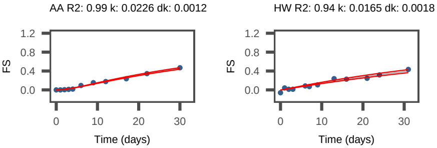

TITIN − DSVNLTWTEPASDGGSK_2

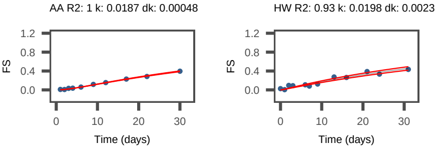

TITIN − ELAESVIAK_2

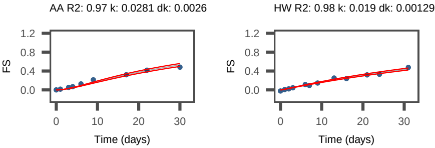

TITIN − DIRPSDIAQITSTPTSSMLTVK_3

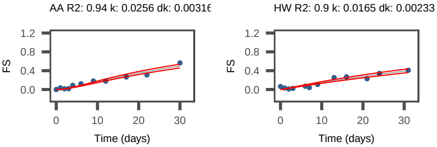

TITIN − DSVSLSWLKPEHDGGSR_4

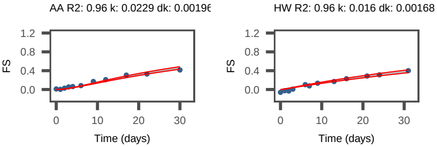

TITIN − ELLLPVLIK_2

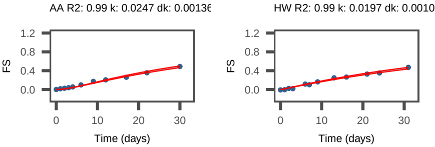

TITIN − DQMLVQWHEPVNDGGSK_2

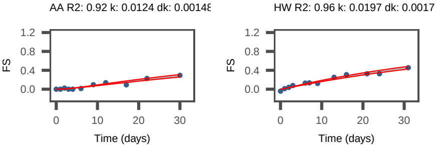

TITIN − EAFSSVIIK_2

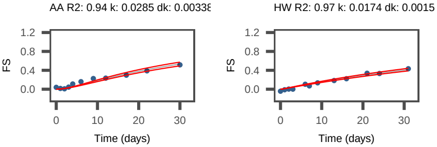

TITIN − ELPLIFITPLSDVK_2

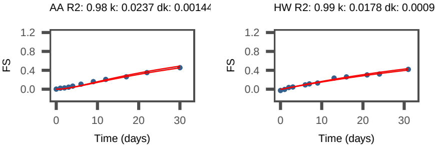

TITIN − DSGDYTITAENSSGSK_2

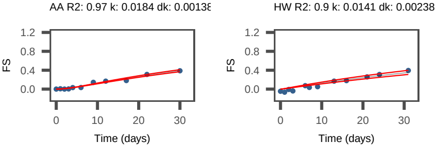

TITIN − EDAGTYTFQVQNNVGK_2

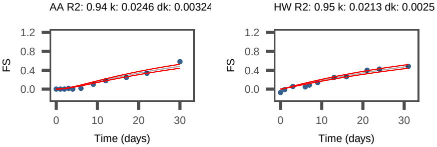

TITIN − ENMATLTVLEPAVIIEK_2

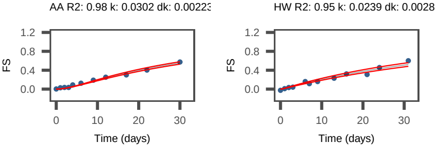

TITIN – EPPVFSSFPPIVETLK_2

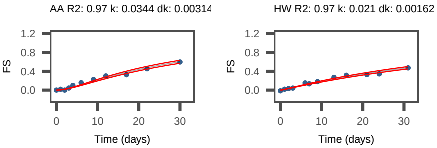

TITIN – EVQLQTTIEGAEPISVAWFK_2

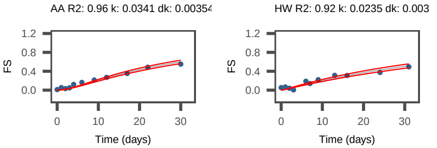

TITIN – GLLQAFELLK_2

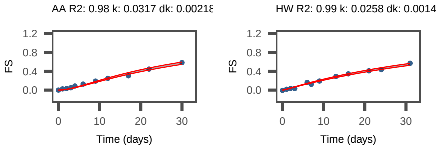

TITIN – EPQIEPTADLTGITNQLITCK_2

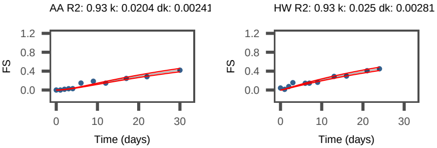

TITIN – FETEISEDDIHANWK_3

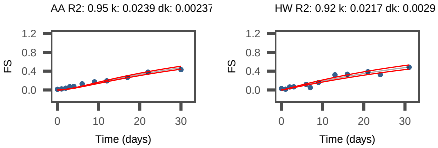

TITIN – GQPLYLSCELNK_2

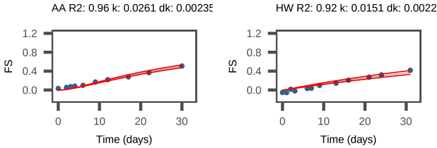

TITIN – EPTHVEESHSQQTTLEYGYK_4

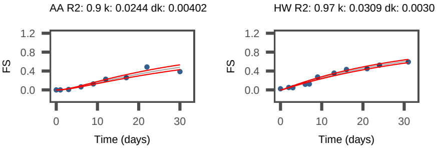

TITIN – FGISDHIDSVCVVVK_3

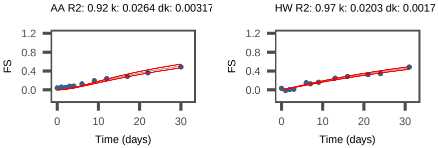

TITIN – GSPPFTVSWFK_2

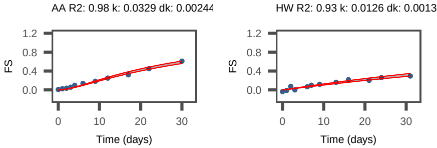

TITIN – ESFEVQWK_2

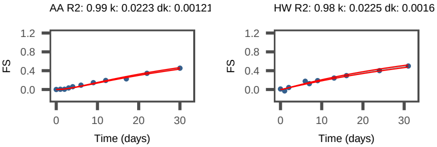

TITIN – FNTEITAENLTINLK_2

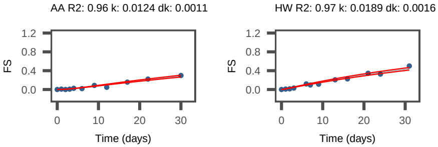

TITIN – GVPFPTLTWFK_2

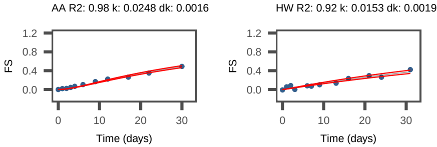

TITIN – ESGTTAWQLINSSVK_2

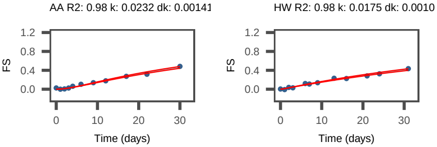

TITIN – GDAGQYTCYASNVAGK_2

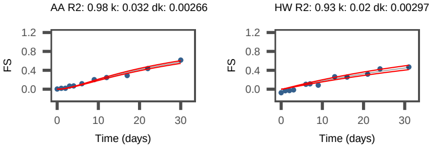

TITIN – IADFSTHLINK_3

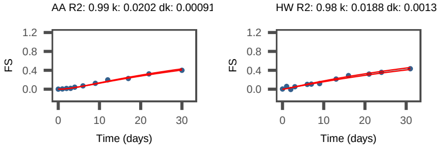

TITIN – ETSESSLELYAVK_2

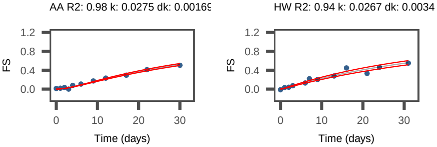

TITIN – GDSGQYTCQATNDVGK_2

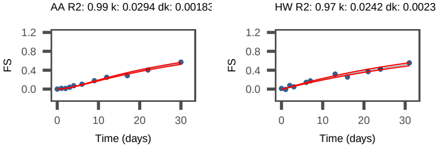

TITIN – IASLEIPLAK_2

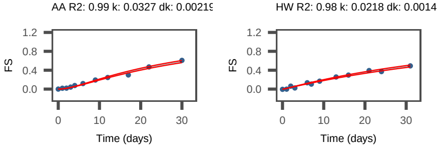

TITIN − IDSIISQDSAWYTATAINK_2

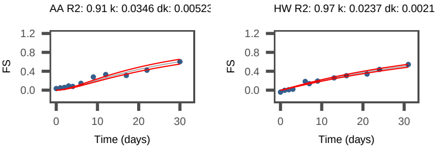

TITIN − INADIAGRPLPVISWAK_3

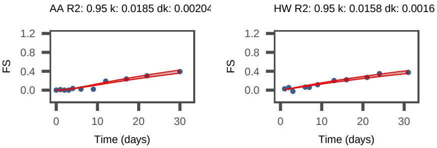

TITIN − ITDQYRPK_2

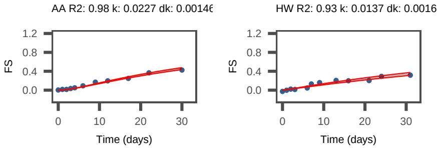

TITIN − IDVPFIGRPPPAVTWHK_4

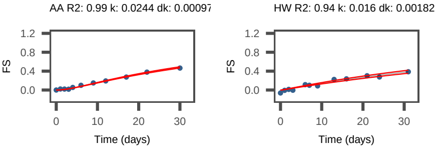

TITIN − INVTDSLDLTTLSIK_2

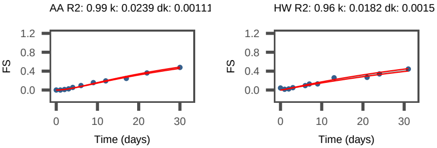

TITIN − ITGYVVDFLTEENK_2

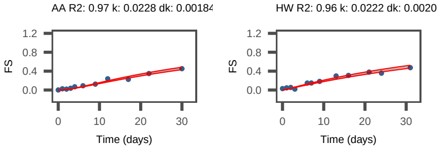

TITIN − IEPLEVALGHLAK_3

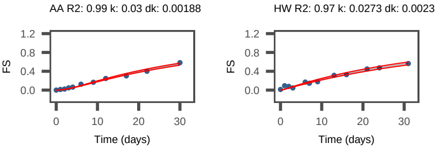

TITIN − IPVVLPEDEGIYTAFASNIK_2

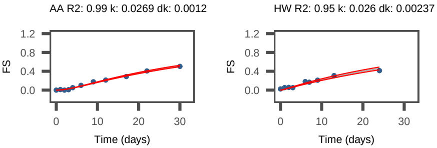

TITIN − ITQFVVPDLQTK_2

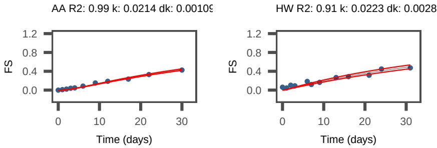

TITIN − IHLECQVDEDRK_3

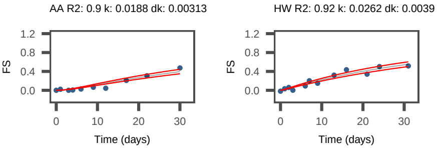

TITIN − ISAINDAGVGEPAVIPNVEIVEK_2

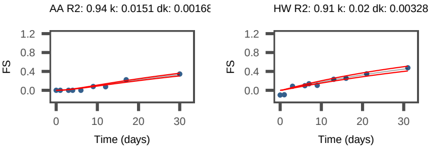

TITIN − KTPSPIEAER_2

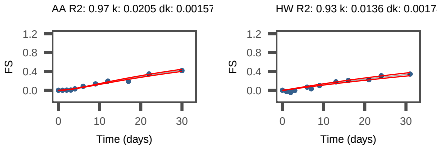

TITIN − IIHYVVEALEK_3

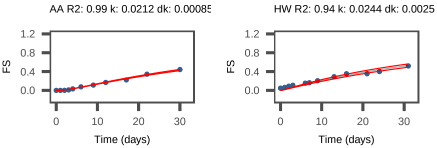

TITIN − ISFVDNIATLQLGSPEASQSGK_2

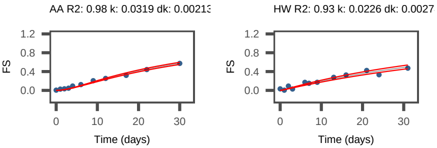

TITIN − KTPSPIEAER_3

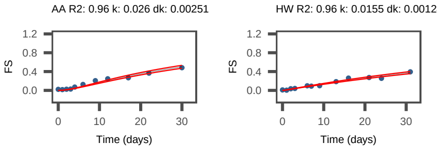

TITIN − IMAENAAGISAPSATSPFYK_2

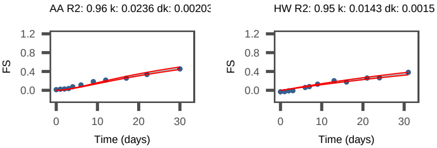

TITIN − ISFVNNVATLQFAK_2

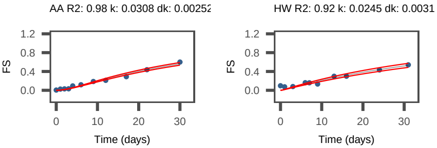

TITIN − LAWTVVASEVVTNSLK_3

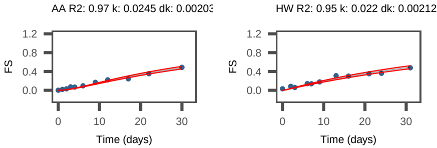

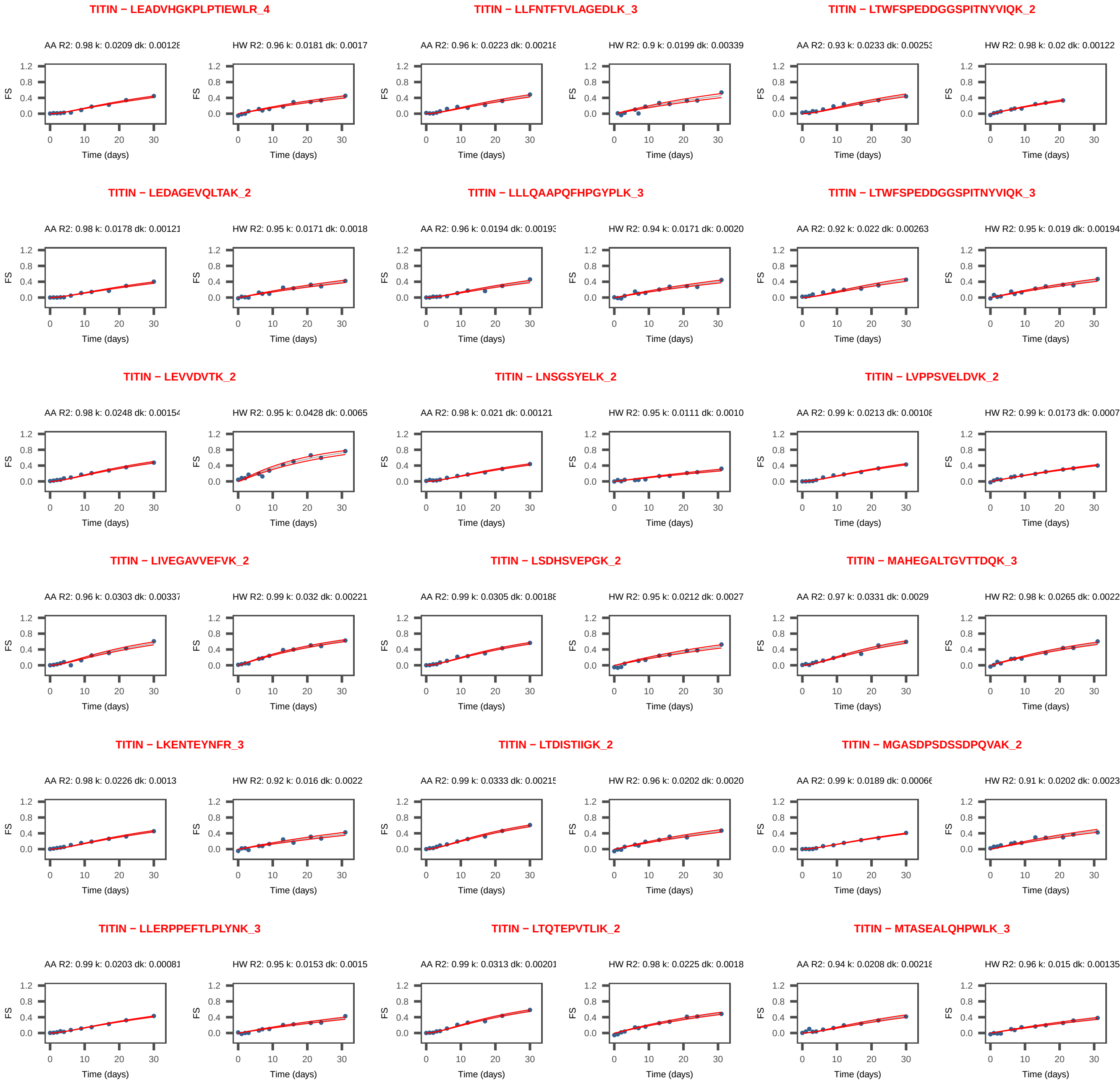
TITIN – LEADVHGKPLPTIEWLR_4
AA R2: 0.98 k: 0.0209 dk: 0.0012
HW R2: 0.96 k: 0.0181 dk: 0.0017
TITIN – LLFNTFTVLAGEDLK_3
AA R2: 0.96 k: 0.0223 dk: 0.0021
HW R2: 0.9 k: 0.0199 dk: 0.00339
TITIN – LTWFSPEDDGGSPITNYVIQK_2
AA R2: 0.93 k: 0.0233 dk: 0.0025
HW R2: 0.98 k: 0.02 dk: 0.00122
TITIN – LEDAGEVQLTAK_2
AA R2: 0.98 k: 0.0178 dk: 0.00121
HW R2: 0.95 k: 0.0171 dk: 0.0018
TITIN – LLLQAAPQFHPGYPLK_3
AA R2: 0.96 k: 0.0194 dk: 0.0019
HW R2: 0.94 k: 0.0171 dk: 0.0020
TITIN – LTWFSPEDDGGSPITNYVIQK_3
AA R2: 0.92 k: 0.022 dk: 0.00263
HW R2: 0.95 k: 0.019 dk: 0.00194
TITIN – LEVVDVTK_2
AA R2: 0.98 k: 0.0248 dk: 0.0015
HW R2: 0.95 k: 0.0428 dk: 0.0065
TITIN – LNSGSYELK_2
AA R2: 0.98 k: 0.021 dk: 0.00121
HW R2: 0.95 k: 0.0111 dk: 0.0010
TITIN – LVPPSVELDVK_2
AA R2: 0.99 k: 0.0213 dk: 0.0010
HW R2: 0.99 k: 0.0173 dk: 0.0007
TITIN – LIVEGAVVEFVK_2
AA R2: 0.96 k: 0.0303 dk: 0.0033
HW R2: 0.99 k: 0.032 dk: 0.00221
TITIN – LSDHSVEPGK_2
AA R2: 0.99 k: 0.0305 dk: 0.0018
HW R2: 0.95 k: 0.0212 dk: 0.0027
TITIN – MAHEGALTGVTTDQK_3
AA R2: 0.97 k: 0.0331 dk: 0.0029
HW R2: 0.98 k: 0.0265 dk: 0.0022
TITIN – LKENTEYNFR_3
AA R2: 0.98 k: 0.0226 dk: 0.0013
HW R2: 0.92 k: 0.016 dk: 0.0022
TITIN – LTDISTIIGK_2
AA R2: 0.99 k: 0.0333 dk: 0.0021
HW R2: 0.96 k: 0.0202 dk: 0.0020
TITIN – MGASDPSDSSDPQVAK_2
AA R2: 0.99 k: 0.0189 dk: 0.0006
HW R2: 0.91 k: 0.0202 dk: 0.0023
TITIN – LLERPPEFTLPLYNK_3
AA R2: 0.99 k: 0.0203 dk: 0.00081
HW R2: 0.95 k: 0.0153 dk: 0.0015
TITIN – LTQTEPVTLIK_2
AA R2: 0.99 k: 0.0313 dk: 0.00201
HW R2: 0.98 k: 0.0225 dk: 0.0018
TITIN – MTASEALQHPWLK_3
AA R2: 0.94 k: 0.0208 dk: 0.0021
HW R2: 0.96 k: 0.015 dk: 0.00135

TITIN − NAAHEDGGIYSLTVENPAGTK_3

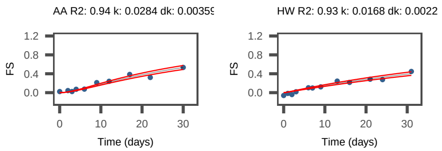

TITIN − NSVTLIWTEPK_2

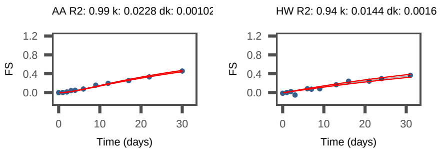

TITIN − QLITFTQELQDVVAK_2

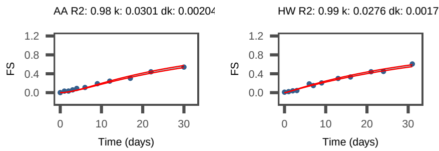

TITIN − NAFVTPGPPSIPEVTK_2

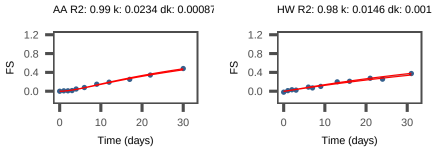

TITIN − NVTGTTSETIK_2

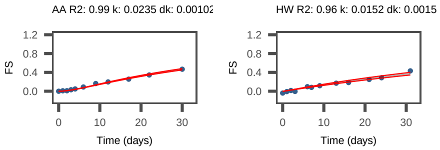

TITIN − QLSVPVIAK_2

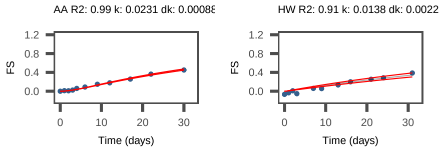

TITIN − NCAMADESVYGFK_2

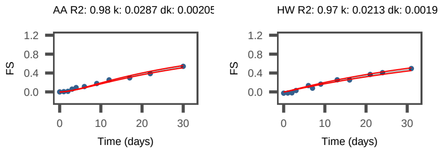

TITIN − PGPPSTPEASAITK_2

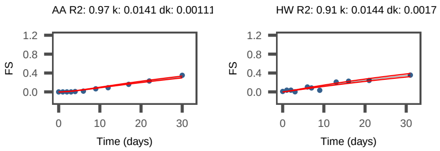

TITIN − QNATVQGLIQGK_2

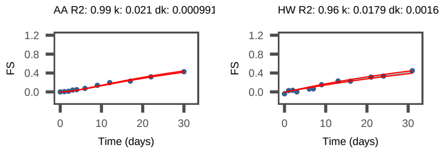

TITIN − NSFTIPSQPGIPEEVGAGK_2

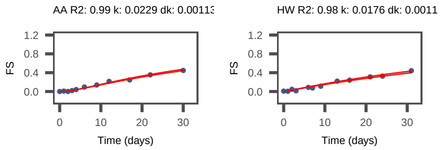

TITIN − PIAILQGLSDQK_2

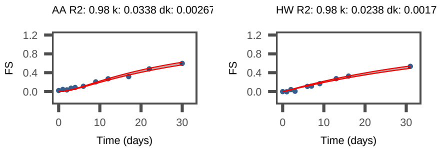

TITIN − RIEPLEVALGHLAK_3

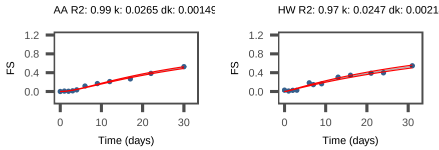

TITIN − NSVSLSWEKPEHDGGSR_3

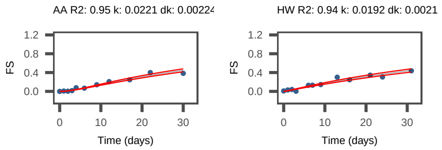

TITIN − PLPVISWAK_2

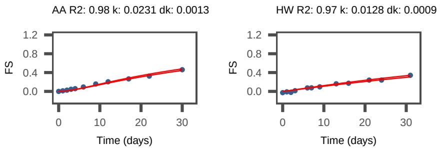

TITIN − RTEEGYYEAITAVELK_3

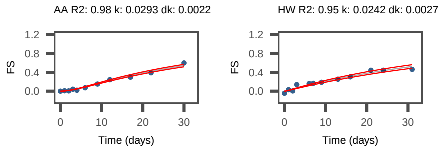

TITIN − NSVSLSWEKPEHDGGSR_4

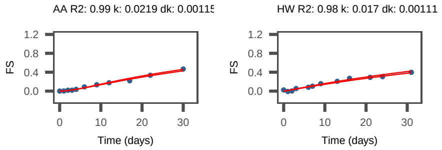

TITIN − QLGVPVIAK_2

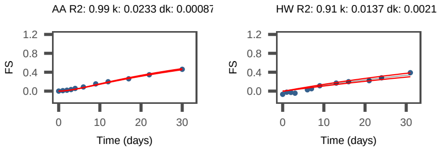

TITIN − SAFQGSAPLTIK_2

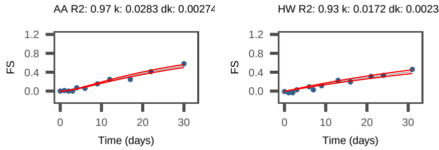

TITIN − SDIGQYTCDCGTDQTSGK_2

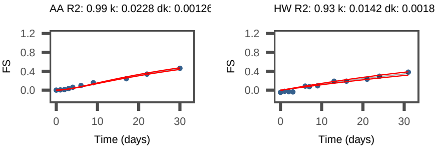

TITIN − SISLAWTK_2

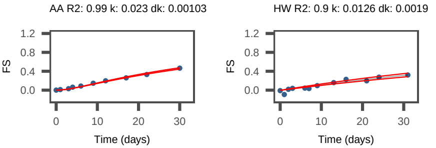

TITIN − STAELYLTTK_2

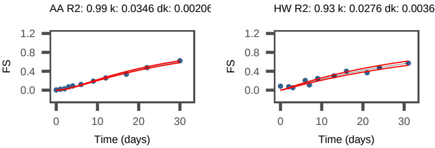

TITIN − SDVPIQAPHFK_3

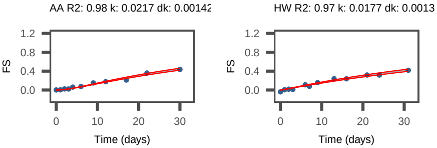

TITIN − SLAVPIIAK_2

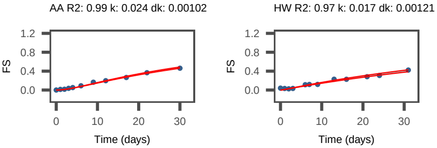

TITIN − TCILEILSSTK_2

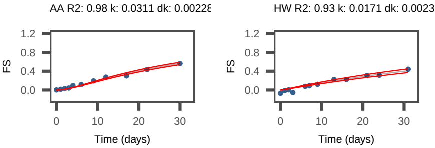

TITIN − SGGNYQISYLENSAHLTIVK_3

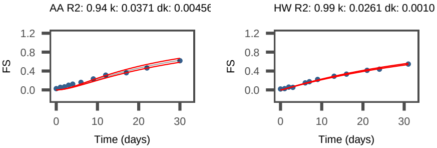

TITIN − SSATFQSTVAGSPPISITWLK_2

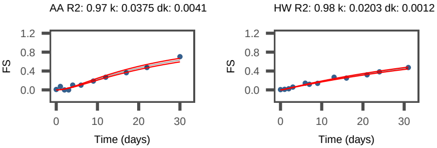

TITIN − TEAYAVSSFK_2

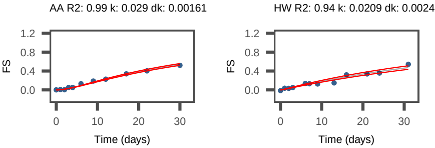

TITIN − SIHEQVSSISETTK_3

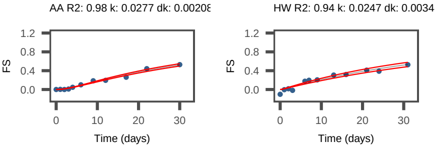

TITIN − SSAVLAWLKPDHDGGSR_3

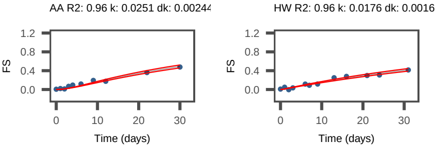

TITIN − TFLQDQLVSLQVLK_2

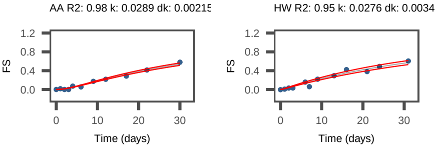

TITIN − SIILEGTYTGTLPISVTWK_2

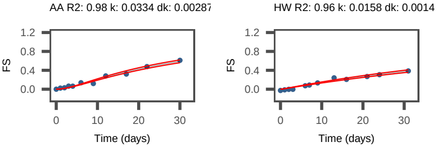

TITIN − SSVFLSWTK_2

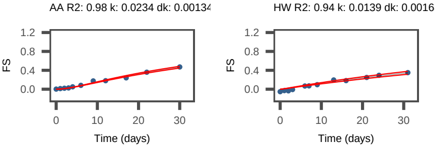

TITIN − TKPDSDWIVITSTLR_3

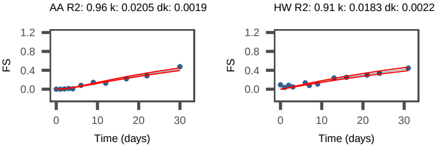

TITIN − SISGEINVNVIAPPSAPK_2

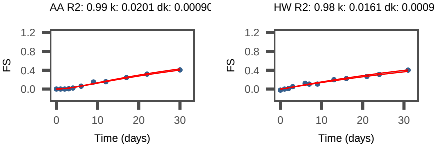

TITIN − SSWEFDGK_2

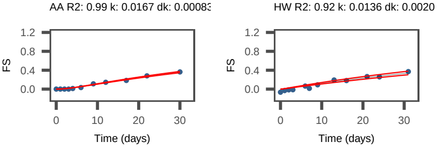

TITIN − TSVSLAWSVPEDEGGSK_2

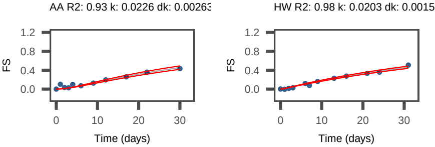

TITIN − TSVTLTWEPPLLDGGSK_2

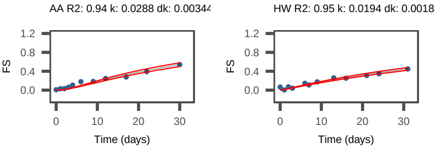

TITIN − VETSCNLSVEK_2

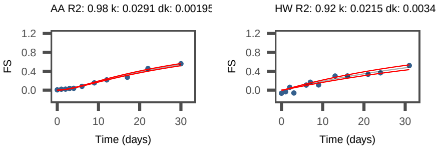

TITIN − VLASNEYGIGLPAETAEPVK_2

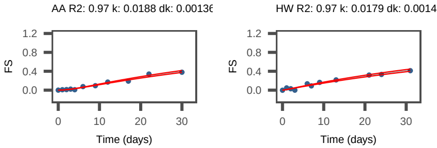

TITIN − VAAENAIGQSDYTEIGDSVLAK_2

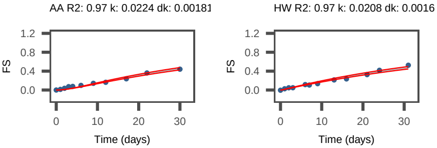

TITIN − VEVFDVPGPVLDLKPVVTNR_3

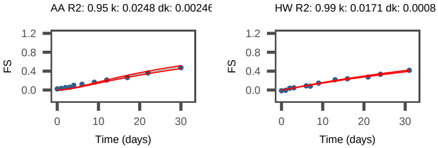

TITIN − VLDRPGPPEGPLAVSDVTSEK_3

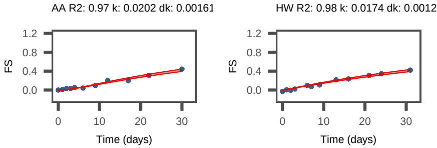

TITIN − VAAENMYGVGEPVQAAPIIAK_2

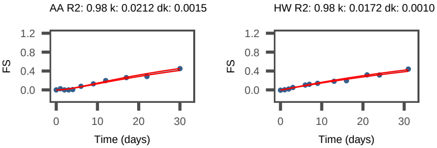

TITIN − VGVGPTIETK_2

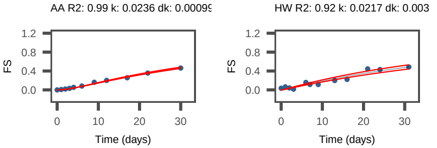

TITIN − VLDRPGPPEGPVAISGVTAEK_3

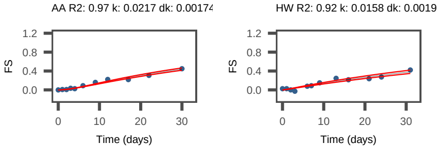

TITIN − VAGSSPISIAWFHEK_3

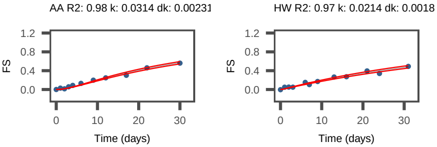

TITIN − VIIENVGTK_2

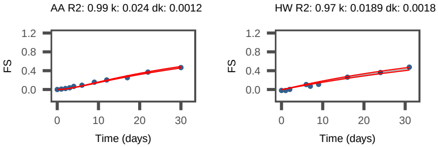

TITIN − VLDRPGPPEGPVQVTGVTAEK_3

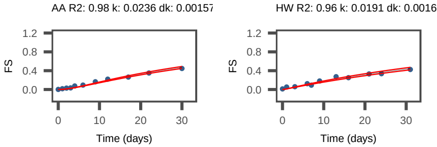

TITIN − VCAVNAAGVGPFSEPSDFYK_2

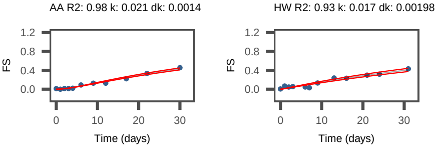

TITIN − VLACNAGGPGEPAEVPGTVK_2

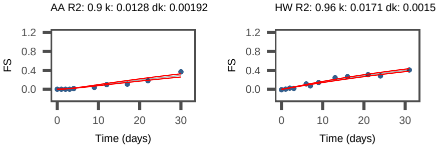

TITIN − VLDTPGPPQNLAVK_2

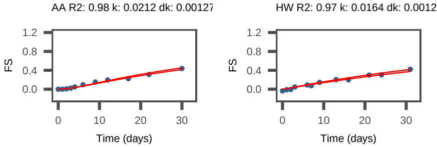

TITIN − VEKPLYGVEVFVGETAR_3

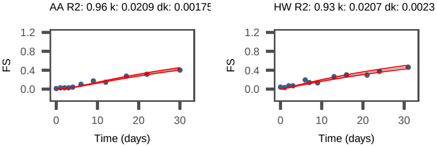

TITIN − VLAENEIGIGEPCETTEPVK_2

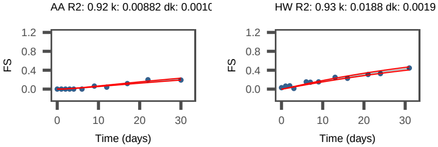

TITIN − VLDTPGPVLNLRPTDITK_3

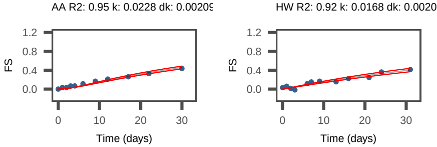

TITIN – VLDTPSPPVNLK_2

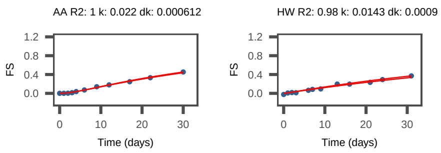

TITIN – VNVEETATSTILHIK_3

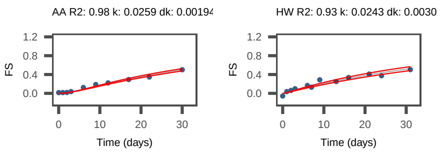

TITIN – VSAQNTFGISEPLEVASIVIIK_3

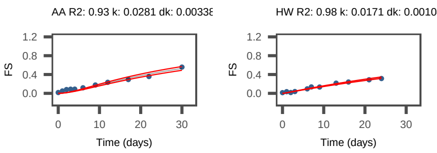

TITIN – VLGIPVIAK_2

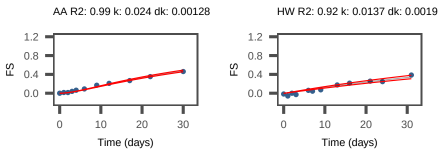

TITIN – VPAPAEVPVTPPTLVSGLK_2

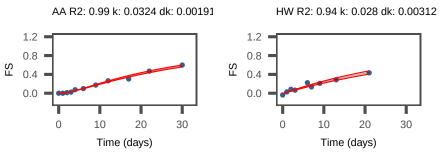

TITIN – VSAVNIVGQGK_2

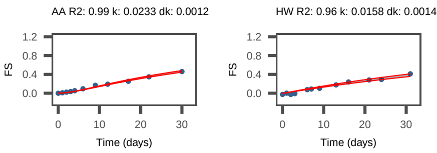

TITIN – VLPTIDLSTMPQK_2

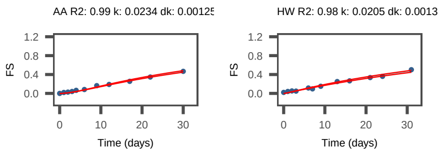

TITIN – VPGPPGTPFVTLASK_2

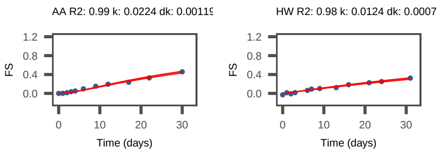

TITIN – VSGIPAPTLK_2

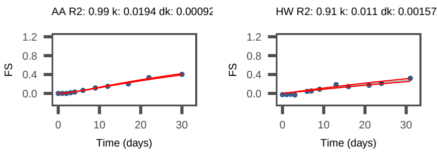

TITIN – VLVLDKPGPPR_3

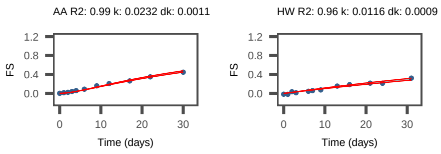

TITIN – VPGPPGTPQVTAVTK_2

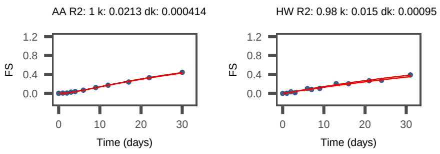

TITIN – VSGSAPISVGWFLDGNEIISSPK_3

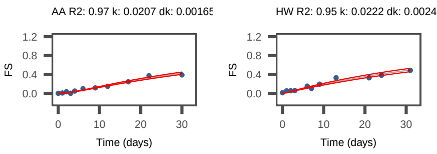

TITIN – VMAENEFGVGVPTETSDAVK_2

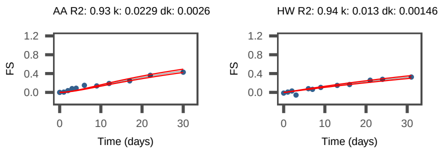

TITIN – VSAENAAGVGEPSPATVYYK_2

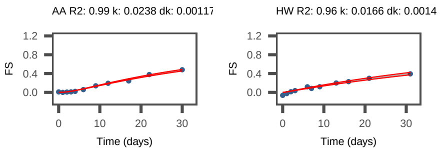

TITIN – VTAENPEGVTEHK_3

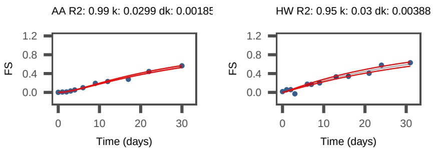

TITIN – VNAESTENNSLLTIK_2

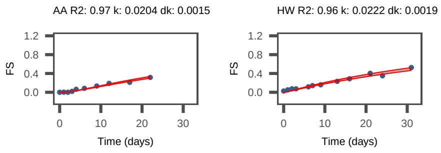

TITIN – VSAQNTFGISEPLEVASIVIIK_2

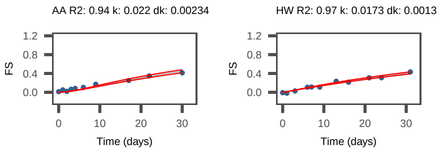

TITIN – VYAENIAGIGK_2

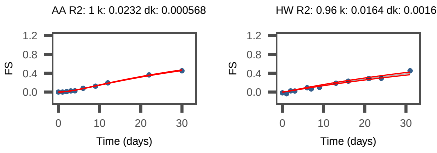

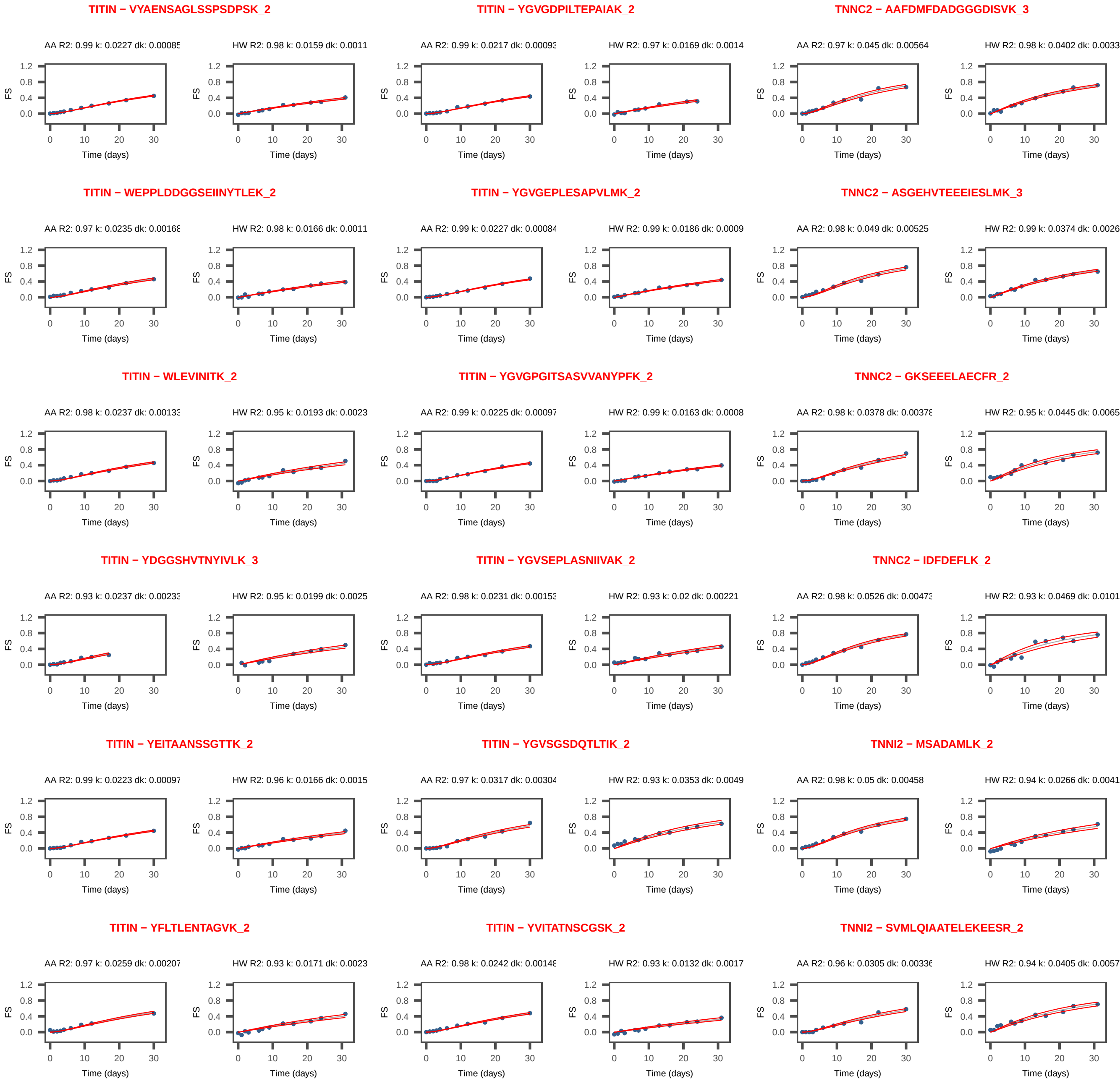
TITIN − VYAENSAGLSSPSDPSK_2
AA R2: 0.99 k: 0.0227 dk: 0.00085
HW R2: 0.98 k: 0.0159 dk: 0.0011
TITIN − YGVGDPILTEPAIAK_2
AA R2: 0.99 k: 0.0217 dk: 0.00093
HW R2: 0.97 k: 0.0169 dk: 0.0014
TNNC2 − AAFDMFDADGGGDISVK_3
AA R2: 0.97 k: 0.045 dk: 0.00564
HW R2: 0.98 k: 0.0402 dk: 0.0033
TITIN − WEPPLDDGGSEIINYTLEK_2
AA R2: 0.97 k: 0.0235 dk: 0.0016
HW R2: 0.98 k: 0.0166 dk: 0.0011
TITIN − YGVGEPLESAPVLMK_2
AA R2: 0.99 k: 0.0227 dk: 0.00084
HW R2: 0.99 k: 0.0186 dk: 0.0009
TNNC2 − ASGEHVTEEEIESLMK_3
AA R2: 0.98 k: 0.049 dk: 0.00525
HW R2: 0.99 k: 0.0374 dk: 0.0026
TITIN − WLEVINITK_2
AA R2: 0.98 k: 0.0237 dk: 0.0013
HW R2: 0.95 k: 0.0193 dk: 0.0023
TITIN − YGVGPGITSASVVANYPFK_2
AA R2: 0.99 k: 0.0225 dk: 0.00097
HW R2: 0.99 k: 0.0163 dk: 0.0008
TNNC2 − GKSEEELAECFR_2
AA R2: 0.98 k: 0.0378 dk: 0.0037
HW R2: 0.95 k: 0.0445 dk: 0.0065
TITIN − YDGGSHVTNYIVLK_3
AA R2: 0.93 k: 0.0237 dk: 0.0023
HW R2: 0.95 k: 0.0199 dk: 0.0025
TITIN − YGVSEPLASNIIVAK_2
AA R2: 0.98 k: 0.0231 dk: 0.0015
HW R2: 0.93 k: 0.02 dk: 0.00221
TNNC2 − IDFDEFLK_2
AA R2: 0.98 k: 0.0526 dk: 0.0047
HW R2: 0.93 k: 0.0469 dk: 0.0101
TITIN − YEITAANSSGTTK_2
AA R2: 0.99 k: 0.0223 dk: 0.00097
HW R2: 0.96 k: 0.0166 dk: 0.0015
TITIN − YGVSGSDQTLTIK_2
AA R2: 0.97 k: 0.0317 dk: 0.0030
HW R2: 0.93 k: 0.0353 dk: 0.0049
TNNI2 − MSADAMLK_2
AA R2: 0.98 k: 0.05 dk: 0.00458
HW R2: 0.94 k: 0.0266 dk: 0.0041
TITIN − YFLTLENTAGVK_2
AA R2: 0.97 k: 0.0259 dk: 0.00207
HW R2: 0.93 k: 0.0171 dk: 0.0023
TITIN − YVITATNSCGSK_2
AA R2: 0.98 k: 0.0242 dk: 0.0014
HW R2: 0.93 k: 0.0132 dk: 0.0017
TNNI2 − SVMLQIAATELEKEESR_2
AA R2: 0.96 k: 0.0305 dk: 0.0033
HW R2: 0.94 k: 0.0405 dk: 0.0057

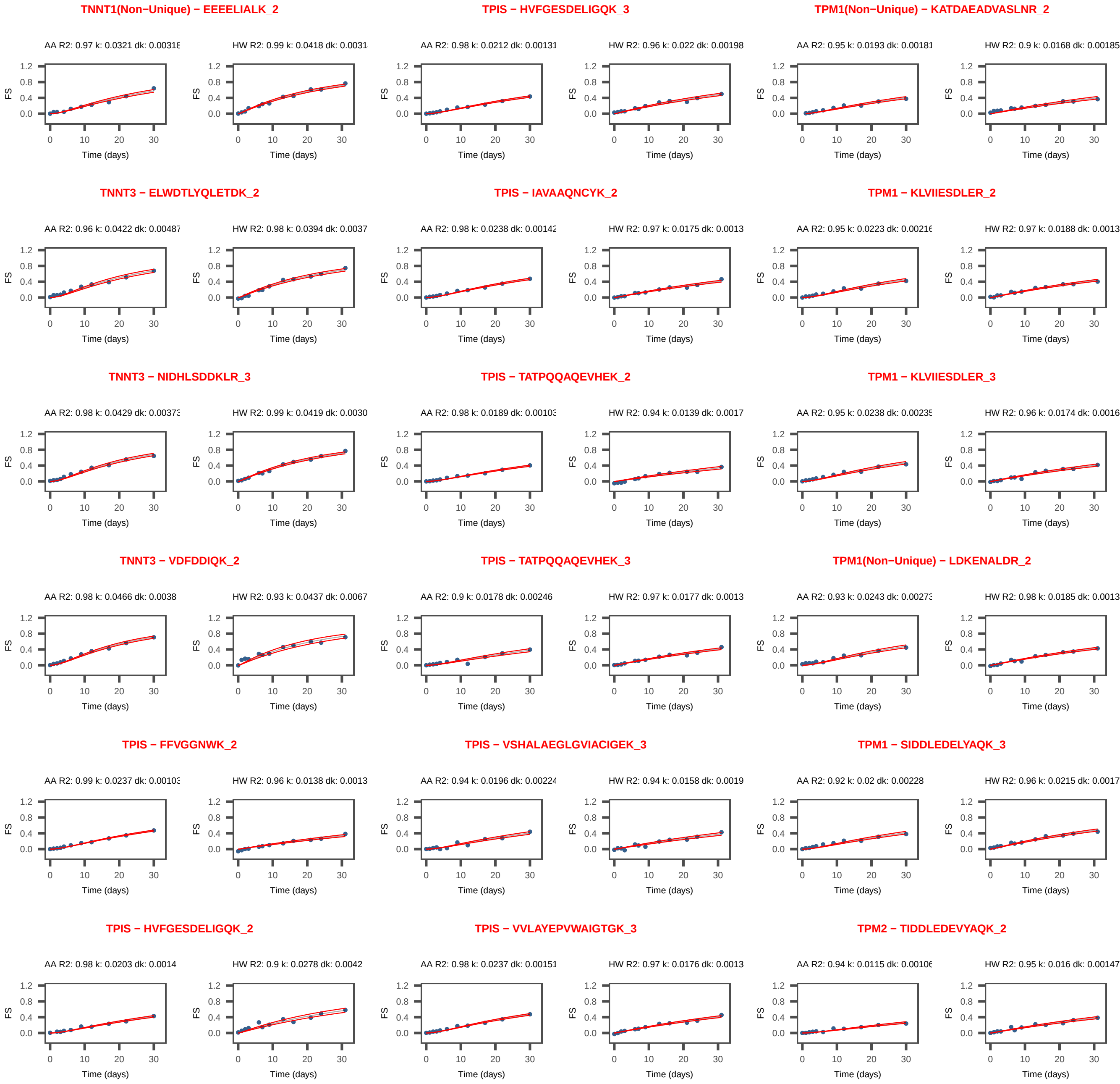
TNNT1(Non−Unique) − EEEELIALK_2
AA R2: 0.97 k: 0.0321 dk: 0.0031
HW R2: 0.99 k: 0.0418 dk: 0.0031
TPIS − HVFGESDELIGQK_3
AA R2: 0.98 k: 0.0212 dk: 0.00131
HW R2: 0.96 k: 0.022 dk: 0.00198
TPM1(Non−Unique) − KATDAEADVASLNR_2
AA R2: 0.95 k: 0.0193 dk: 0.00181
HW R2: 0.9 k: 0.0168 dk: 0.00185
TNNT3 − ELWDTLYQLETDK_2
AA R2: 0.96 k: 0.0422 dk: 0.0048
HW R2: 0.98 k: 0.0394 dk: 0.0037
TPIS − IAVAAQNCYK_2
AA R2: 0.98 k: 0.0238 dk: 0.00142
HW R2: 0.97 k: 0.0175 dk: 0.0013
TPM1 − KLVIIESDLER_2
AA R2: 0.95 k: 0.0223 dk: 0.0021
HW R2: 0.97 k: 0.0188 dk: 0.0013
TNNT3 − NIDHLSDDKLR_3
AA R2: 0.98 k: 0.0429 dk: 0.0037
HW R2: 0.99 k: 0.0419 dk: 0.0030
TPIS − TATPQQAQEVHEK_2
AA R2: 0.98 k: 0.0189 dk: 0.00103
HW R2: 0.94 k: 0.0139 dk: 0.0017
TPM1 − KLVIIESDLER_3
AA R2: 0.95 k: 0.0238 dk: 0.0023
HW R2: 0.96 k: 0.0174 dk: 0.0016
TNNT3 − VDFDDIQK_2
AA R2: 0.98 k: 0.0466 dk: 0.0038
HW R2: 0.93 k: 0.0437 dk: 0.0067
TPIS − TATPQQAQEVHEK_3
AA R2: 0.9 k: 0.0178 dk: 0.00246
HW R2: 0.97 k: 0.0177 dk: 0.0013
TPM1(Non−Unique) − LDKENALDR_2
AA R2: 0.93 k: 0.0243 dk: 0.0027
HW R2: 0.98 k: 0.0185 dk: 0.0013
TPIS − FFVGGNWK_2
AA R2: 0.99 k: 0.0237 dk: 0.0010
HW R2: 0.96 k: 0.0138 dk: 0.0013
TPIS − VSHALAEGLGVIACIGEK_3
AA R2: 0.94 k: 0.0196 dk: 0.0022
HW R2: 0.94 k: 0.0158 dk: 0.0019
TPM1 − SIDDLEDELYAQK_3
AA R2: 0.92 k: 0.02 dk: 0.00228
HW R2: 0.96 k: 0.0215 dk: 0.0017
TPIS − HVFGESDELIGQK_2
AA R2: 0.98 k: 0.0203 dk: 0.0014
HW R2: 0.9 k: 0.0278 dk: 0.0042
TPIS − VVLAYEPVWAIGTGK_3
AA R2: 0.98 k: 0.0237 dk: 0.00151
HW R2: 0.97 k: 0.0176 dk: 0.0013
TPM2 − TIDDLEDEVYAQK_2
AA R2: 0.94 k: 0.0115 dk: 0.0010
HW R2: 0.95 k: 0.016 dk: 0.00147
FS
Time (days)

TPM3(Non−Unique) − LATALQK_2

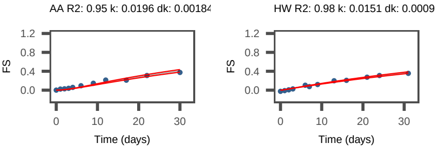

TRFE − HQTVLDNTEGK_2

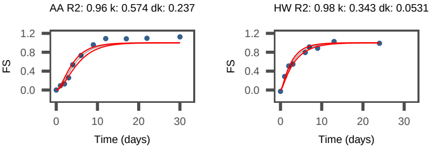

UBB(Non−Unique) − TITLEVEPSDTIENVK_2

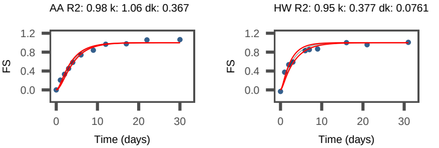

UBB(Non−Unique) − TLSDYNIQK_2

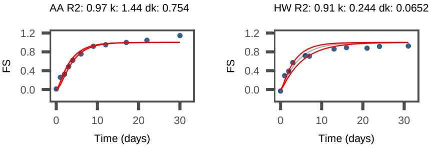

UCRI − NVVSQFVSSMSASADVLAMSK_3

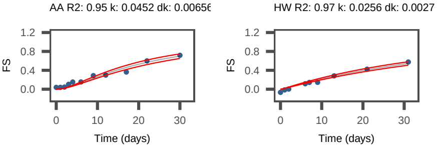

UGPA − TLDGGLNVIQLETAVGAAIK_2

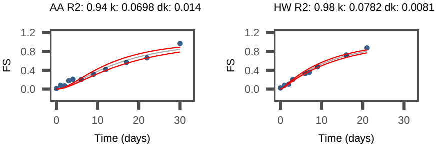

UGPA − TLDGGLNVIQLETAVGAAIK_3

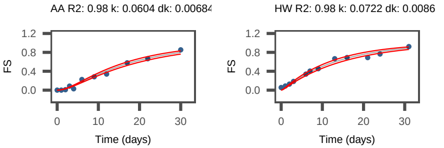

VDAC1 − GYGFGLIK_2

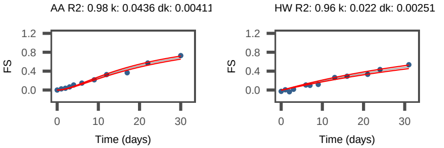

VDAC1 − LTFDSSFSPNTGK_2

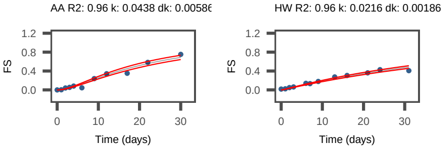

VDAC1 − VTQSNFAVGYK_2

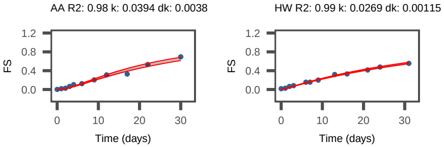

VDAC1 − YQVDPDACFSAK_2

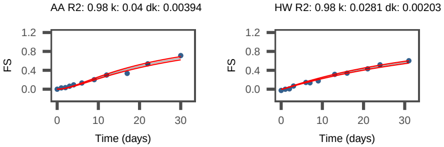

VDAC2 − VNNSSLIGVGYTQTLRPGVK_3

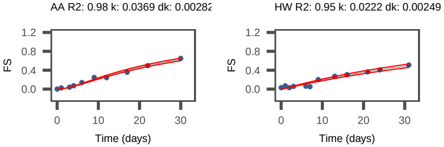

VDAC3 − AADFQLHTHVNDGTEFGGSIYQK_4

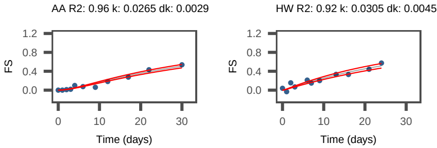

VDAC3(Non−Unique) − LTLSALIDGK_2

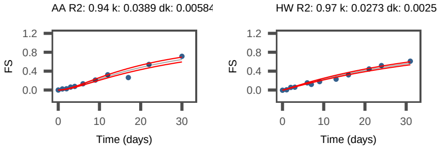

Supplement: Supplemental Data S4 [file mmc5.pdf]
