# Supplementary material for: Harmonizing Labeling and Analytical Strategies to Obtain Protein Turnover Rates in Intact Adult Animals
Source: Mol Cell Proteomics. 2022 May 28;21(7):100252. doi: 10.1016/j.mcpro.2022.100252 (PMC9249856; doi:10.1016/j.mcpro.2022.100252)

1433E

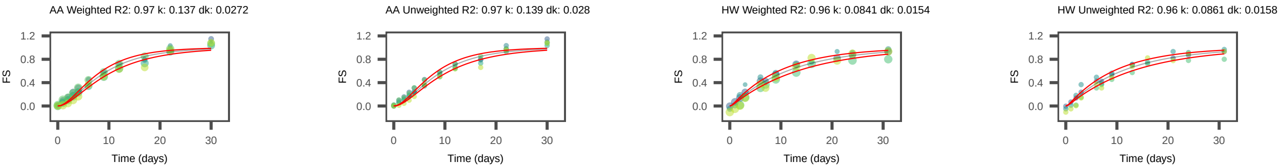

1433G

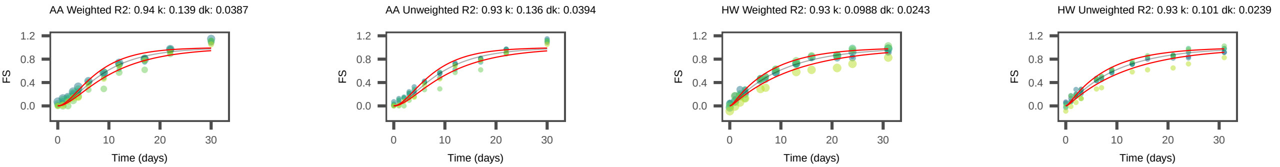

1433Z

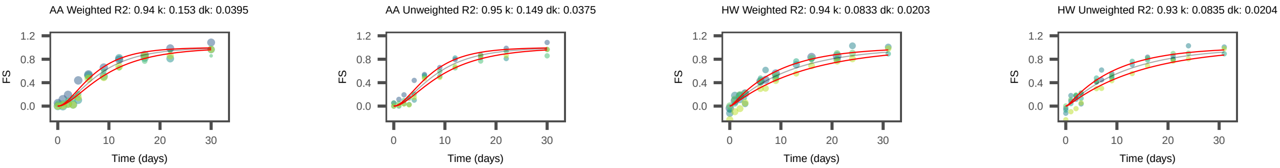

2AAA

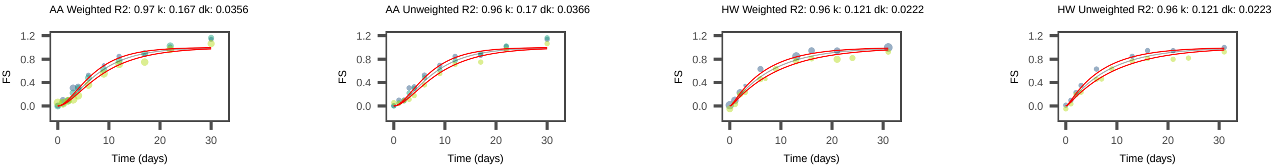

3HIDH

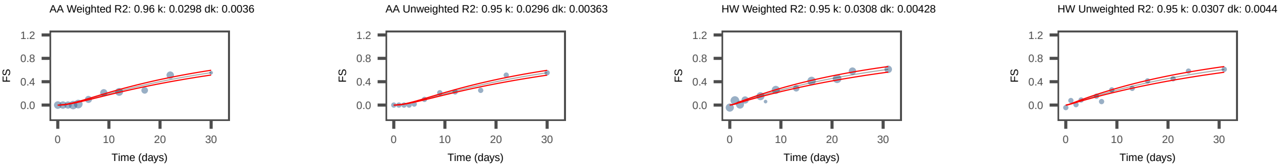

5NT3A

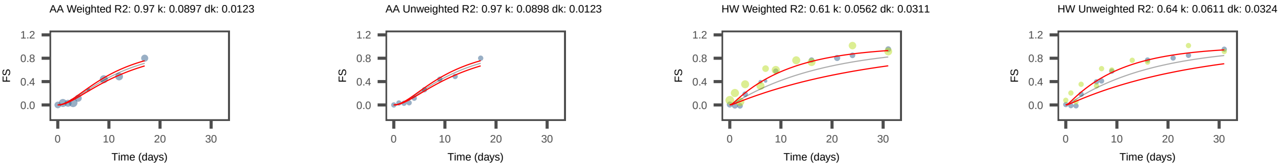

6PGL

AA Weighted R2: 0.91 k: 0.0548 dk: 0.0141

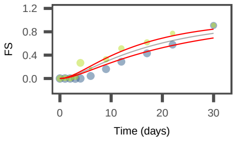

AA Unweighted R2: 0.91 k: 0.0578 dk: 0.0148

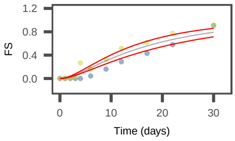

HW Weighted R2: 0.93 k: 0.0404 dk: 0.00832

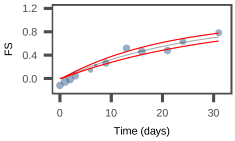

HW Unweighted R2: 0.93 k: 0.0404 dk: 0.00825

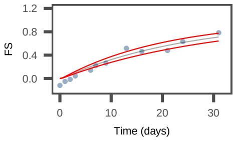

A1AT2

AA Weighted R2: 0.94 k: 9.36 dk: 32.8

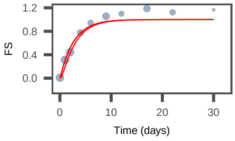

AA Unweighted R2: 0.93 k: 9.78 dk: 37.8

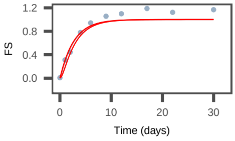

HW Weighted R2: 0.97 k: 0.592 dk: 0.0959

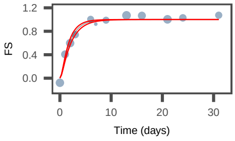

HW Unweighted R2: 0.97 k: 0.59 dk: 0.0951

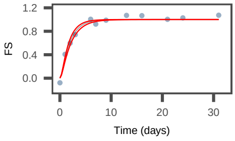

AATC

AA Weighted R2: 0.96 k: 0.0899 dk: 0.0157

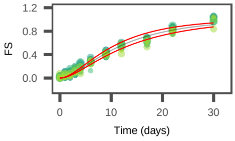

AA Unweighted R2: 0.96 k: 0.0879 dk: 0.0161

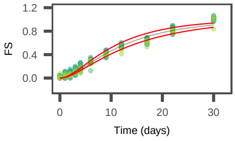

HW Weighted R2: 0.92 k: 0.0609 dk: 0.0137

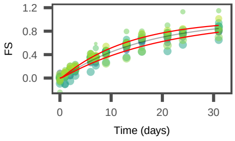

HW Unweighted R2: 0.91 k: 0.0631 dk: 0.0154

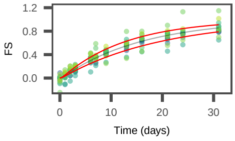

AATM

AA Weighted R2: 0.98 k: 0.0366 dk: 0.00314

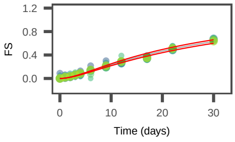

AA Unweighted R2: 0.97 k: 0.0371 dk: 0.00391

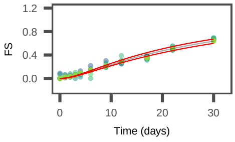

HW Weighted R2: 0.88 k: 0.0246 dk: 0.00449

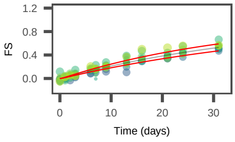

HW Unweighted R2: 0.9 k: 0.0246 dk: 0.00408

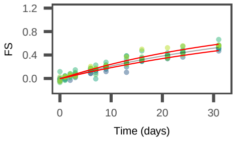

ABCB7

AA Weighted R2: 0.97 k: 0.0519 dk: 0.00783

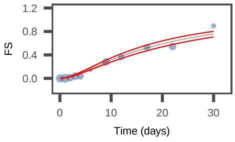

AA Unweighted R2: 0.96 k: 0.0524 dk: 0.00817

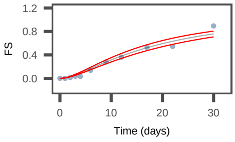

HW Weighted R2: 0.95 k: 0.0427 dk: 0.00629

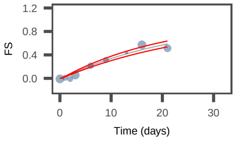

HW Unweighted R2: 0.95 k: 0.0427 dk: 0.00622

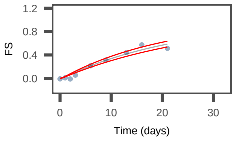

ABEC2

AA Weighted R2: 0.98 k: 0.156 dk: 0.0254

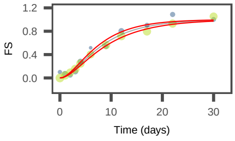

AA Unweighted R2: 0.98 k: 0.16 dk: 0.0285

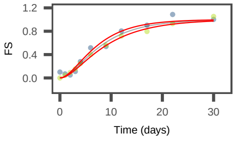

HW Weighted R2: 0.95 k: 0.125 dk: 0.0301

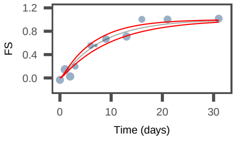

HW Unweighted R2: 0.95 k: 0.126 dk: 0.03

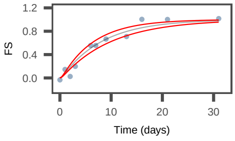

ABLM1

AA Weighted R2: 0.94 k: 0.137 dk: 0.038 | AA Unweighted R2: 0.94 k: 0.132 dk: 0.0367 | HW Weighted R2: 0.92 k: 0.0851 dk: 0.0218 | HW Unweighted R2: 0.92 k: 0.0848 dk: 0.0222

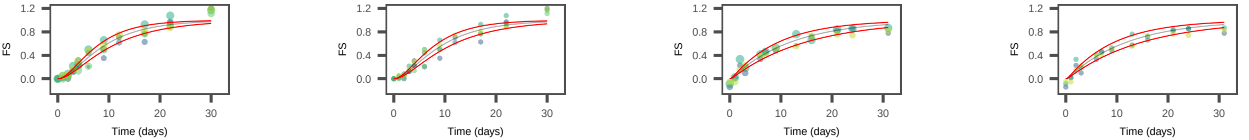

ACADL

AA Weighted R2: 0.92 k: 0.0394 dk: 0.00691 | AA Unweighted R2: 0.91 k: 0.0387 dk: 0.00724 | HW Weighted R2: 0.94 k: 0.0264 dk: 0.0034 | HW Unweighted R2: 0.94 k: 0.0267 dk: 0.00343

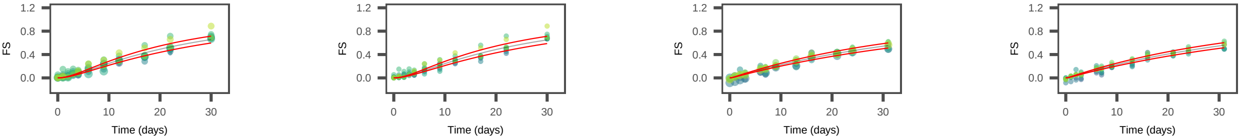

ACADM

AA Weighted R2: 0.96 k: 0.0546 dk: 0.00803 | AA Unweighted R2: 0.94 k: 0.0517 dk: 0.00918 | HW Weighted R2: 0.97 k: 0.0418 dk: 0.005 | HW Unweighted R2: 0.95 k: 0.041 dk: 0.0061

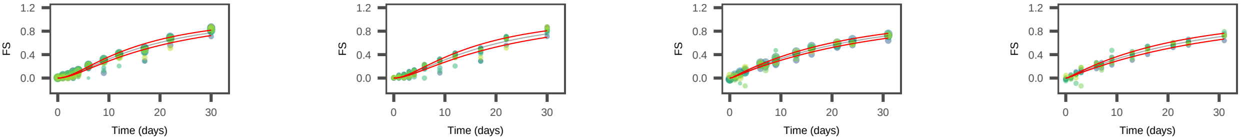

ACADS

AA Weighted R2: 0.93 k: 0.0388 dk: 0.00609 | AA Unweighted R2: 0.93 k: 0.0382 dk: 0.00605 | HW Weighted R2: 0.86 k: 0.028 dk: 0.00599 | HW Unweighted R2: 0.86 k: 0.0288 dk: 0.00624

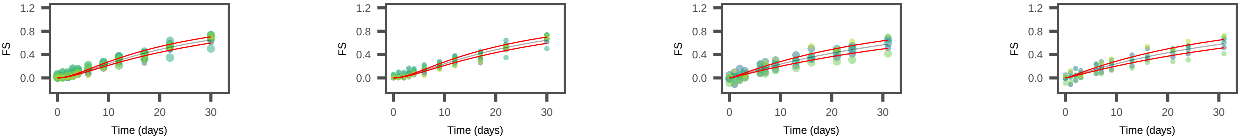

ACADV

AA Weighted R2: 0.96 k: 0.0699 dk: 0.0115 | AA Unweighted R2: 0.95 k: 0.0679 dk: 0.0119 | HW Weighted R2: 0.95 k: 0.0491 dk: 0.0082 | HW Unweighted R2: 0.94 k: 0.0486 dk: 0.00847

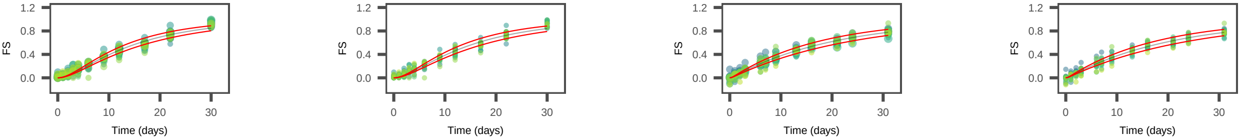

ACBP

AA Weighted R2: 0.98 k: 0.0759 dk: 0.00905 | AA Unweighted R2: 0.98 k: 0.0757 dk: 0.00929 | HW Weighted R2: 0.99 k: 0.0654 dk: 0.00597 | HW Unweighted R2: 0.99 k: 0.0654 dk: 0.00606

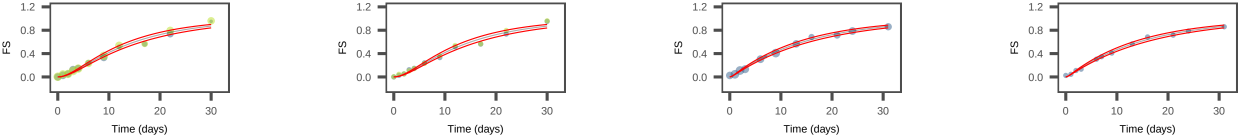

ACD10

AA Weighted R2: 0.7 k: 0.0466 dk: 0.0238 | AA Unweighted R2: 0.59 k: 0.0561 dk: 0.0333 | HW Weighted R2: 0.95 k: 0.0327 dk: 0.00374 | HW Unweighted R2: 0.95 k: 0.0327 dk: 0.00371

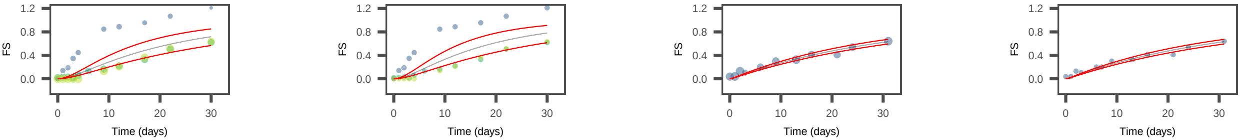

ACDSB

AA Weighted R2: 0.96 k: 0.0708 dk: 0.0141 | AA Unweighted R2: 0.96 k: 0.0723 dk: 0.0145 | HW Weighted R2: 0.91 k: 0.0741 dk: 0.0185 | HW Unweighted R2: 0.91 k: 0.0757 dk: 0.0188

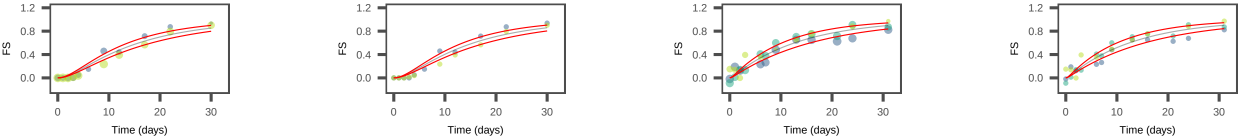

ACO13

AA Weighted R2: 0.91 k: 0.0443 dk: 0.00892 | AA Unweighted R2: 0.84 k: 0.0391 dk: 0.0101 | HW Weighted R2: 0.92 k: 0.0271 dk: 0.00404 | HW Unweighted R2: 0.92 k: 0.027 dk: 0.00398

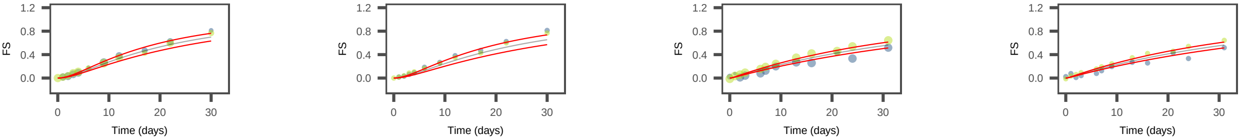

ACOC

AA Weighted R2: 0.93 k: 0.062 dk: 0.0166 | AA Unweighted R2: 0.92 k: 0.0622 dk: 0.0168 | HW Weighted R2: 0.96 k: 0.0688 dk: 0.0127 | HW Unweighted R2: 0.96 k: 0.0686 dk: 0.0127

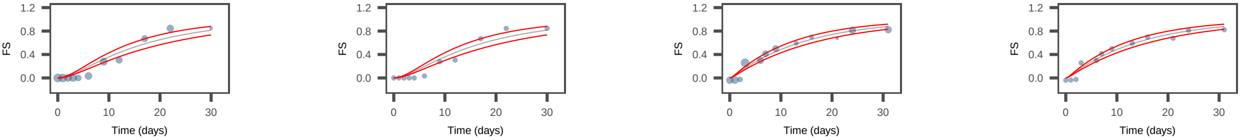

ACON

AA Weighted R2: 0.94 k: 0.0414 dk: 0.00657 | AA Unweighted R2: 0.91 k: 0.0408 dk: 0.00795 | HW Weighted R2: 0.94 k: 0.0299 dk: 0.00397 | HW Unweighted R2: 0.94 k: 0.0298 dk: 0.00391

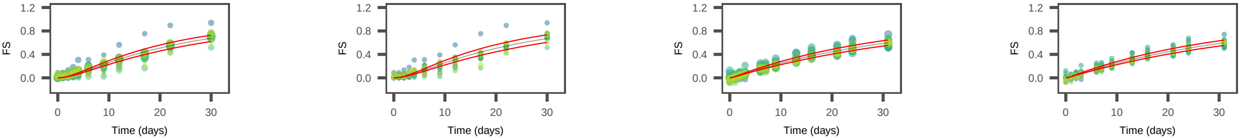

ACOT2

AA Weighted R2: 0.98 k: 0.0316 dk: 0.00213 | AA Unweighted R2: 0.98 k: 0.0319 dk: 0.00215 | HW Weighted R2: 0.99 k: 0.0289 dk: 0.00162 | HW Unweighted R2: 0.99 k: 0.029 dk: 0.00163

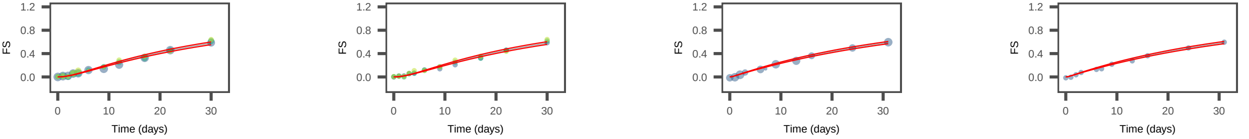

ACOX1

AA Weighted R2: 0.89 k: 0.354 dk: 0.177

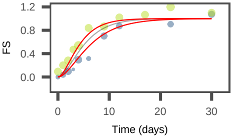

AA Unweighted R2: 0.89 k: 0.329 dk: 0.161

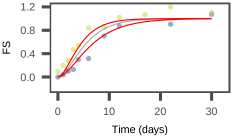

HW Weighted R2: 0.94 k: 0.434 dk: 0.117

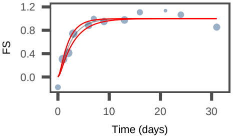

HW Unweighted R2: 0.94 k: 0.435 dk: 0.119

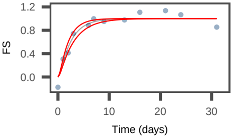

ACPM

AA Weighted R2: 0.98 k: 0.0524 dk: 0.00544

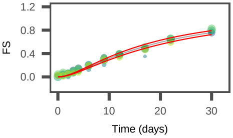

AA Unweighted R2: 0.97 k: 0.0515 dk: 0.0057

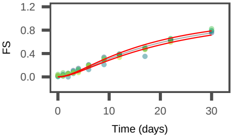

HW Weighted R2: 0.97 k: 0.0396 dk: 0.00412

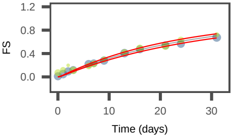

HW Unweighted R2: 0.96 k: 0.0399 dk: 0.00457

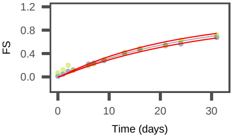

ACS2L

AA Weighted R2: 0.95 k: 0.0647 dk: 0.0119

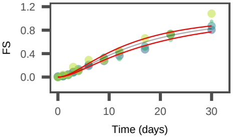

AA Unweighted R2: 0.94 k: 0.0639 dk: 0.0133

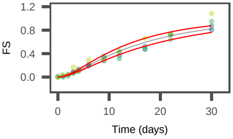

HW Weighted R2: 0.89 k: 0.0587 dk: 0.0161

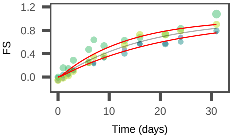

HW Unweighted R2: 0.89 k: 0.0564 dk: 0.0152

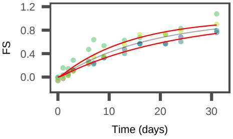

ACSF2

AA Weighted R2: 0.91 k: 0.064 dk: 0.0162

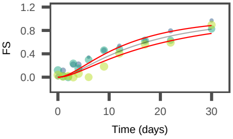

AA Unweighted R2: 0.9 k: 0.0696 dk: 0.0183

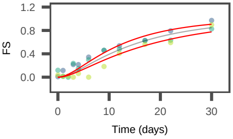

HW Weighted R2: 0.96 k: 0.0735 dk: 0.0143

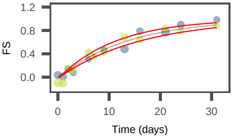

HW Unweighted R2: 0.96 k: 0.0736 dk: 0.0141

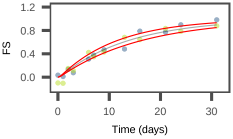

ACSL1

AA Weighted R2: 0.94 k: 0.0553 dk: 0.0107

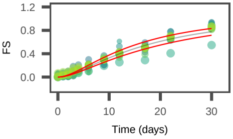

AA Unweighted R2: 0.94 k: 0.0568 dk: 0.0109

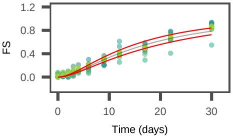

HW Weighted R2: 0.93 k: 0.0423 dk: 0.00713

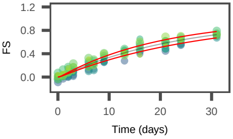

HW Unweighted R2: 0.94 k: 0.0418 dk: 0.00693

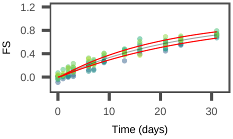

ACTN2

AA Weighted R2: 0.97 k: 0.0808 dk: 0.0121

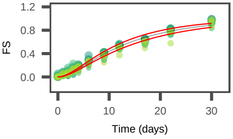

AA Unweighted R2: 0.97 k: 0.0787 dk: 0.012

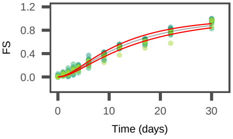

HW Weighted R2: 0.96 k: 0.0583 dk: 0.00851

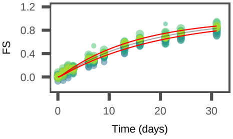

HW Unweighted R2: 0.96 k: 0.0582 dk: 0.009

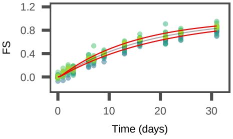

ACYP1

AA Weighted R2: 0.98 k: 0.0453 dk: 0.00346 | AA Unweighted R2: 0.98 k: 0.0452 dk: 0.00351 | HW Weighted R2: 0.95 k: 0.0583 dk: 0.00968 | HW Unweighted R2: 0.96 k: 0.0586 dk: 0.00949

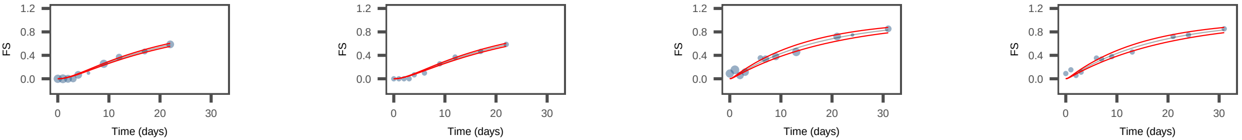

ADIPO

AA Weighted R2: 0.92 k: 0.187 dk: 0.0783 | AA Unweighted R2: 0.92 k: 0.187 dk: 0.0777 | HW Weighted R2: 0.91 k: 0.0454 dk: 0.00892 | HW Unweighted R2: 0.91 k: 0.0453 dk: 0.00903

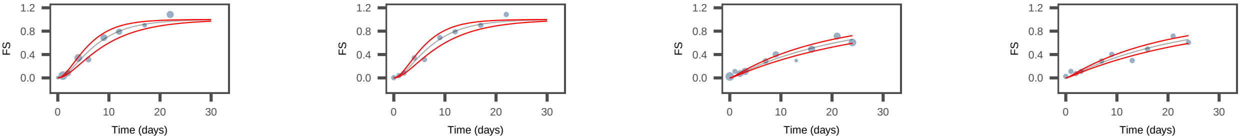

ADK

AA Weighted R2: 0.94 k: 0.102 dk: 0.025 | AA Unweighted R2: 0.94 k: 0.111 dk: 0.027 | HW Weighted R2: 0.92 k: 0.0941 dk: 0.0223 | HW Unweighted R2: 0.92 k: 0.0947 dk: 0.0225

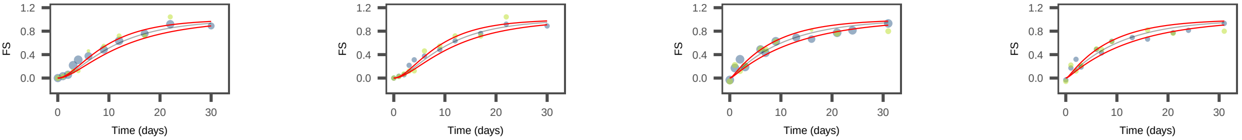

ADT1

AA Weighted R2: 0.99 k: 0.0386 dk: 0.00222 | AA Unweighted R2: 0.99 k: 0.0384 dk: 0.00228 | HW Weighted R2: 0.94 k: 0.0303 dk: 0.0042 | HW Unweighted R2: 0.92 k: 0.0295 dk: 0.00473

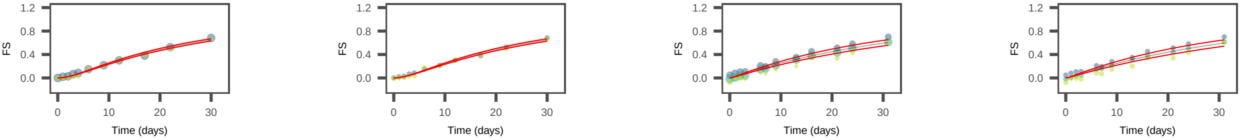

ADT2

AA Weighted R2: 0.95 k: 0.0552 dk: 0.00797 | AA Unweighted R2: 0.95 k: 0.0538 dk: 0.00806 | HW Weighted R2: 0.99 k: 0.0308 dk: 0.00202 | HW Unweighted R2: 0.99 k: 0.0307 dk: 0.00202

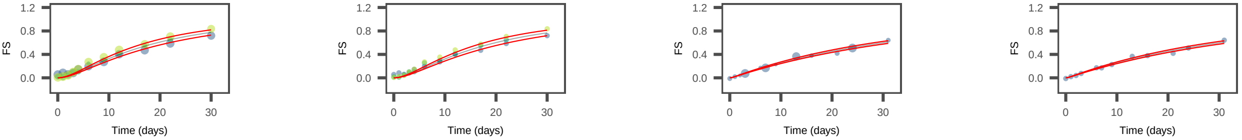

AIFM1

AA Weighted R2: 0.87 k: 0.0293 dk: 0.00556 | AA Unweighted R2: 0.88 k: 0.0299 dk: 0.00554 | HW Weighted R2: 0.95 k: 0.0276 dk: 0.00296 | HW Unweighted R2: 0.95 k: 0.0275 dk: 0.00296

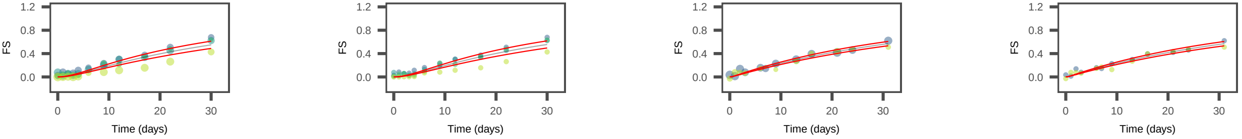

AL4A1

AA Weighted R2: 0.91 k: 0.0321 dk: 0.00567

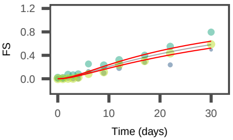

AA Unweighted R2: 0.89 k: 0.0314 dk: 0.00597

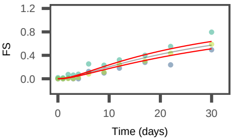

HW Weighted R2: 0.88 k: 0.0251 dk: 0.00478

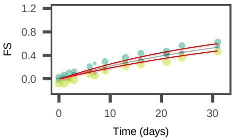

HW Unweighted R2: 0.88 k: 0.0256 dk: 0.00475

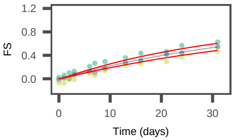

ALBU

AA Weighted R2: 0.94 k: 0.385 dk: 0.142

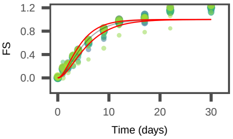

AA Unweighted R2: 0.94 k: 0.355 dk: 0.135

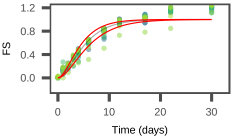

HW Weighted R2: 0.96 k: 0.194 dk: 0.0382

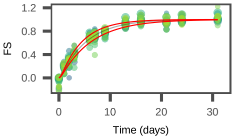

HW Unweighted R2: 0.95 k: 0.192 dk: 0.0421

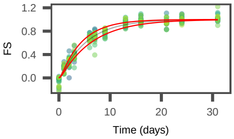

ALDH2

AA Weighted R2: 0.9 k: 0.118 dk: 0.0344

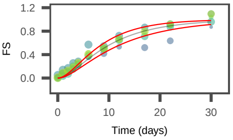

AA Unweighted R2: 0.81 k: 0.0949 dk: 0.0392

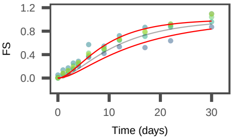

HW Weighted R2: 0.96 k: 0.0751 dk: 0.0121

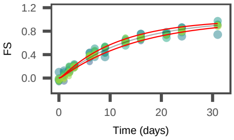

HW Unweighted R2: 0.96 k: 0.0756 dk: 0.012

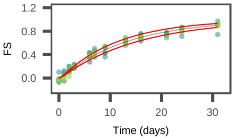

ALDOA

AA Weighted R2: 0.98 k: 0.158 dk: 0.0254

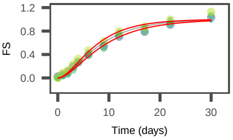

AA Unweighted R2: 0.98 k: 0.156 dk: 0.024

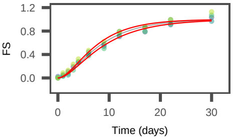

HW Weighted R2: 0.97 k: 0.12 dk: 0.0208

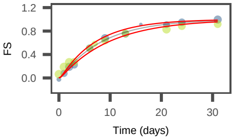

HW Unweighted R2: 0.96 k: 0.12 dk: 0.025

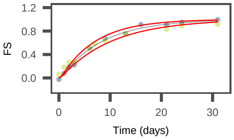

ALDR

AA Weighted R2: 0.96 k: 0.0661 dk: 0.0111

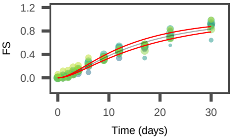

AA Unweighted R2: 0.95 k: 0.065 dk: 0.0122

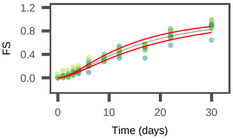

HW Weighted R2: 0.93 k: 0.0462 dk: 0.00971

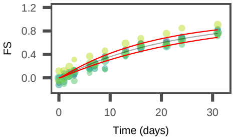

HW Unweighted R2: 0.92 k: 0.0459 dk: 0.00983

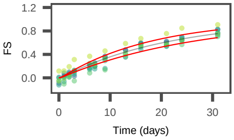

ANXA2

AA Weighted R2: 0.92 k: 0.0438 dk: 0.00816

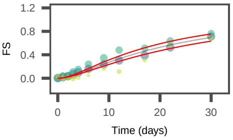

AA Unweighted R2: 0.91 k: 0.0425 dk: 0.00852

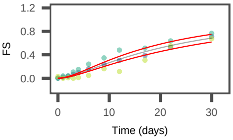

HW Weighted R2: 0.98 k: 0.0447 dk: 0.00406

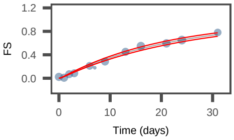

HW Unweighted R2: 0.98 k: 0.0446 dk: 0.00419

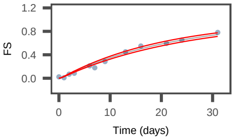

ANXA6

AA Weighted R2: 0.79 k: 0.08 dk: 0.0348 | AA Unweighted R2: 0.79 k: 0.0808 dk: 0.035 | HW Weighted R2: 0.89 k: 0.0919 dk: 0.0258 | HW Unweighted R2: 0.9 k: 0.0836 dk: 0.0225

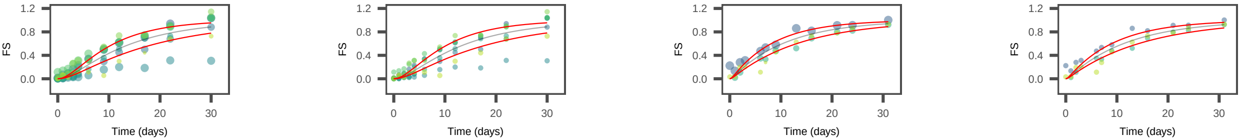

AOFB

AA Weighted R2: 0.93 k: 0.041 dk: 0.00685 | AA Unweighted R2: 0.93 k: 0.0407 dk: 0.00699 | HW Weighted R2: 0.9 k: 0.0262 dk: 0.00482 | HW Unweighted R2: 0.92 k: 0.0264 dk: 0.00449

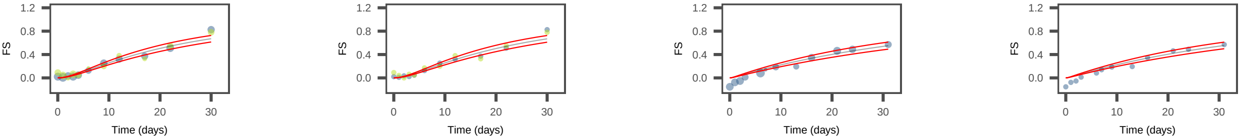

APOA1

AA Weighted R2: 0.94 k: 67.7 dk: 1630 | AA Unweighted R2: 0.94 k: 66.8 dk: 1680 | HW Weighted R2: 0.83 k: 3.59 dk: 2.6 | HW Unweighted R2: 0.83 k: 3.66 dk: 2.72

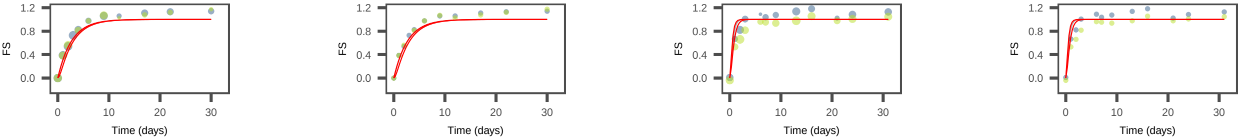

ARHL1

AA Weighted R2: 0.93 k: 0.0545 dk: 0.0125 | AA Unweighted R2: 0.93 k: 0.0542 dk: 0.0126 | HW Weighted R2: 0.93 k: 0.127 dk: 0.037 | HW Unweighted R2: 0.93 k: 0.127 dk: 0.0374

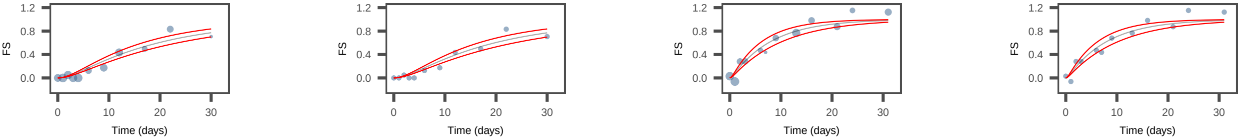

AT1A1

AA Weighted R2: 0.95 k: 0.0648 dk: 0.0114 | AA Unweighted R2: 0.93 k: 0.0629 dk: 0.0134 | HW Weighted R2: 0.92 k: 0.0436 dk: 0.00931 | HW Unweighted R2: 0.92 k: 0.0451 dk: 0.00954

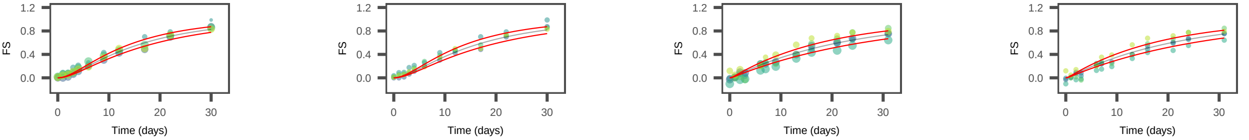

AT1B1

AA Weighted R2: 0.98 k: 0.0722 dk: 0.00899 | AA Unweighted R2: 0.98 k: 0.072 dk: 0.0091 | HW Weighted R2: 0.97 k: 0.0537 dk: 0.00622 | HW Unweighted R2: 0.97 k: 0.0549 dk: 0.00675

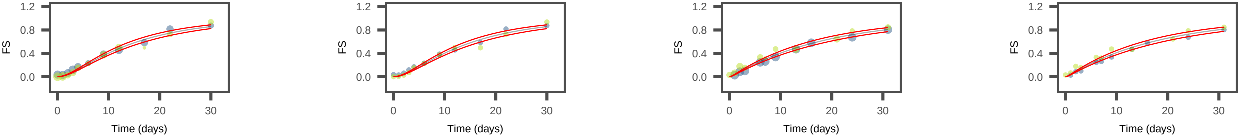

AT2A2

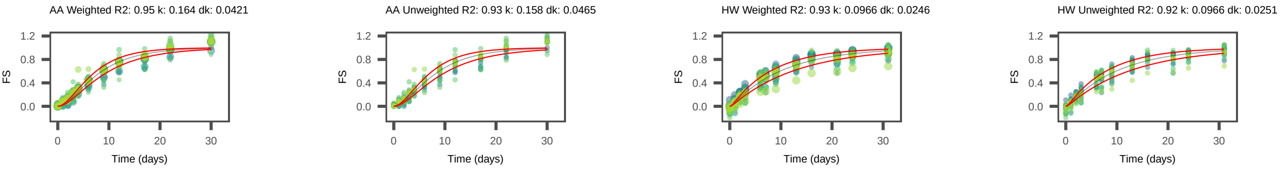

AT5F1

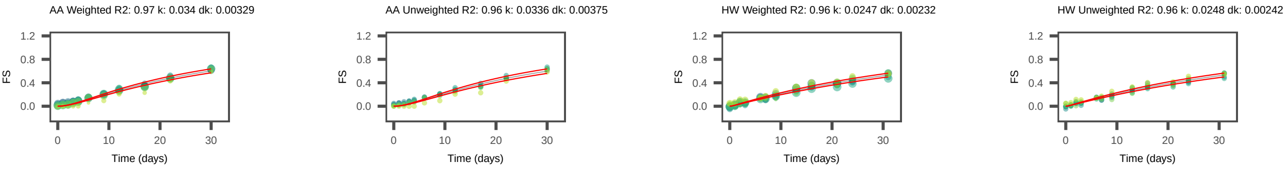

ATP5H

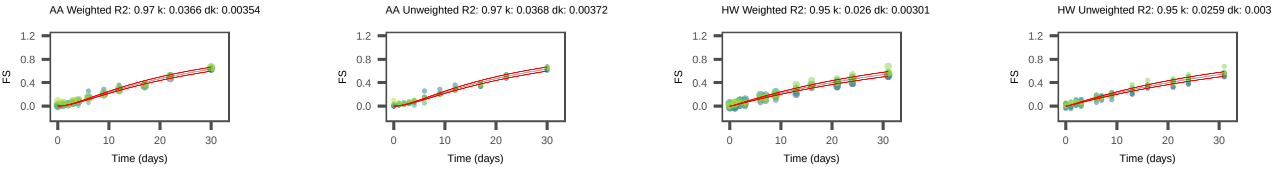

ATP5I

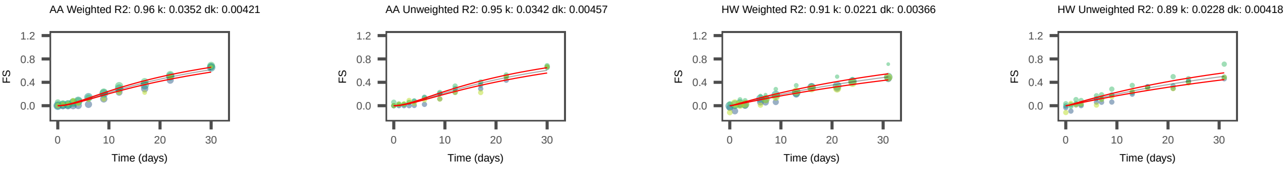

ATP5J

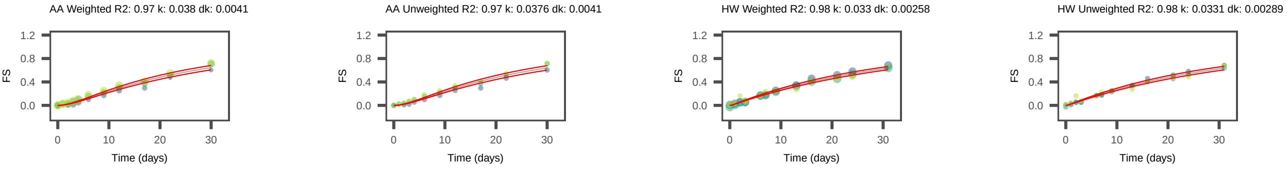

ATP5L

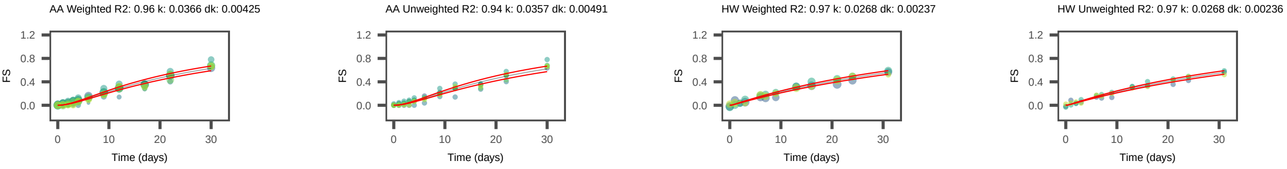

ATPA

AA Weighted R2: 0.96 k: 0.0414 dk: 0.00522 | AA Unweighted R2: 0.95 k: 0.0403 dk: 0.00551 | HW Weighted R2: 0.95 k: 0.0341 dk: 0.00444 | HW Unweighted R2: 0.95 k: 0.0338 dk: 0.00451

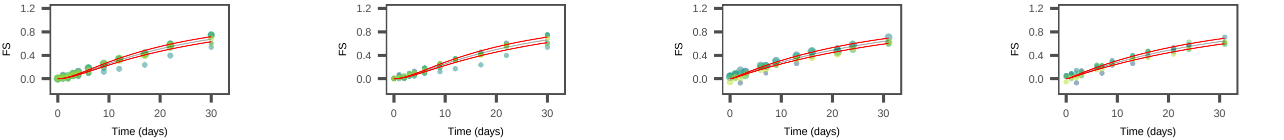

ATPB

AA Weighted R2: 0.94 k: 0.0358 dk: 0.00501 | AA Unweighted R2: 0.92 k: 0.0356 dk: 0.00573 | HW Weighted R2: 0.96 k: 0.0264 dk: 0.00288 | HW Unweighted R2: 0.96 k: 0.0264 dk: 0.00286

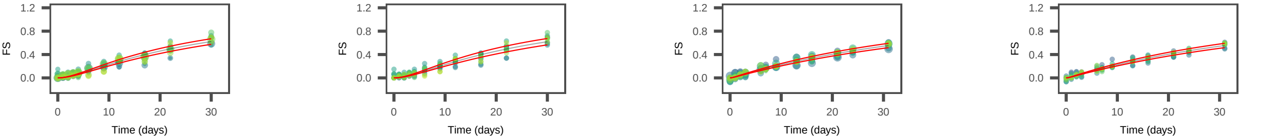

ATPD

AA Weighted R2: 0.96 k: 0.0302 dk: 0.00327 | AA Unweighted R2: 0.95 k: 0.0293 dk: 0.00362 | HW Weighted R2: 0.92 k: 0.0245 dk: 0.00357 | HW Unweighted R2: 0.92 k: 0.0241 dk: 0.00353

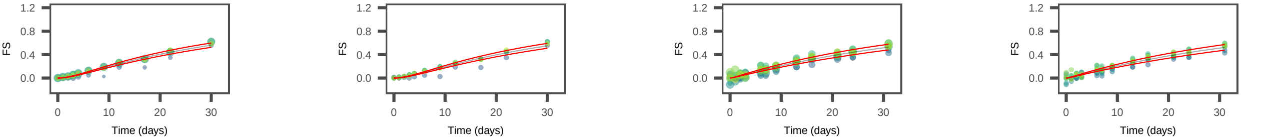

ATPG

AA Weighted R2: 0.98 k: 0.035 dk: 0.003 | AA Unweighted R2: 0.98 k: 0.035 dk: 0.00301 | HW Weighted R2: 0.98 k: 0.0211 dk: 0.00145 | HW Unweighted R2: 0.98 k: 0.021 dk: 0.00144

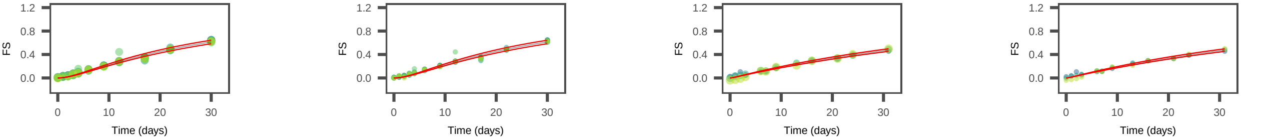

ATPO

AA Weighted R2: 0.97 k: 0.0364 dk: 0.00323 | AA Unweighted R2: 0.97 k: 0.0366 dk: 0.00336 | HW Weighted R2: 0.98 k: 0.023 dk: 0.00162 | HW Unweighted R2: 0.98 k: 0.0232 dk: 0.00164

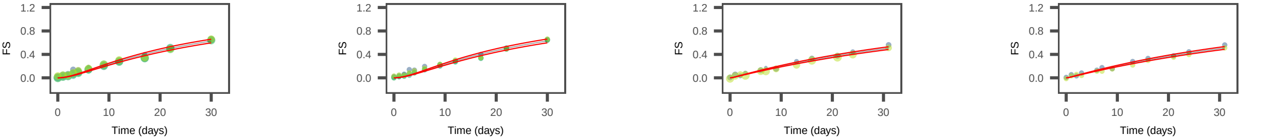

BAG3

AA Weighted R2: 0.69 k: 0.25 dk: 0.185 | AA Unweighted R2: 0.68 k: 0.234 dk: 0.177 | HW Weighted R2: 0.9 k: 0.197 dk: 0.0533 | HW Unweighted R2: 0.9 k: 0.194 dk: 0.0539

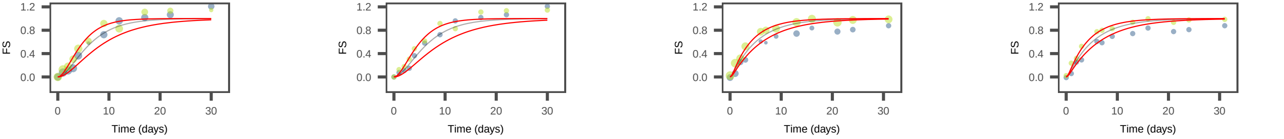

BASI

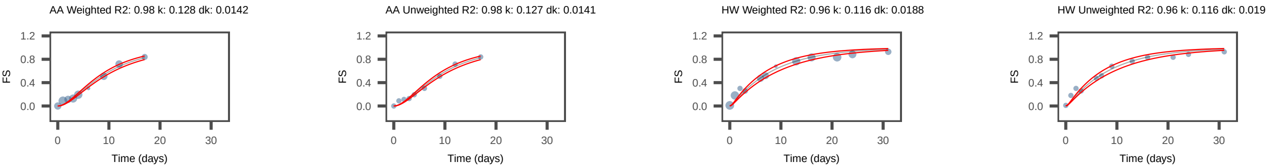

BCAT2

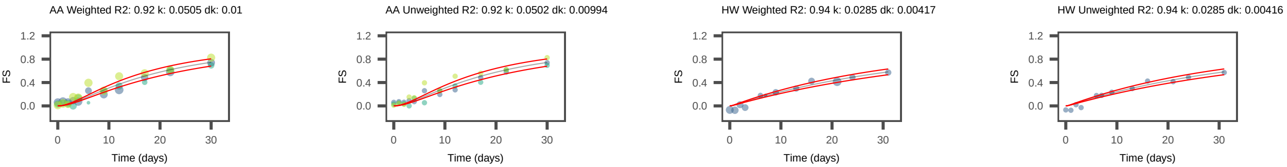

BDH

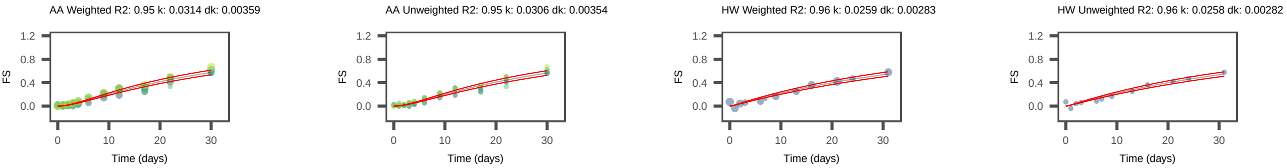

BIP

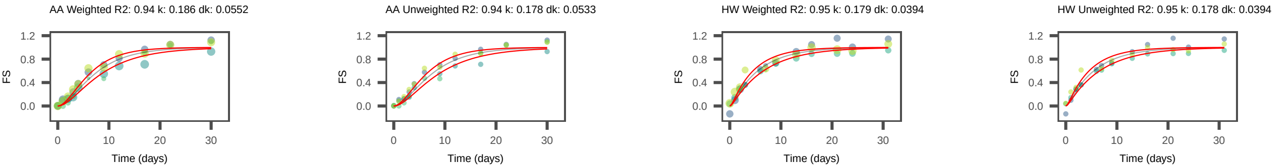

BOLA3

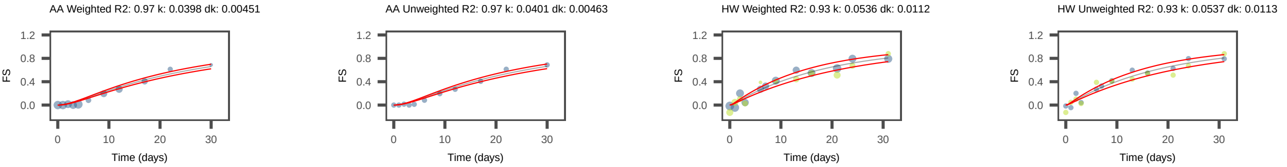

C560

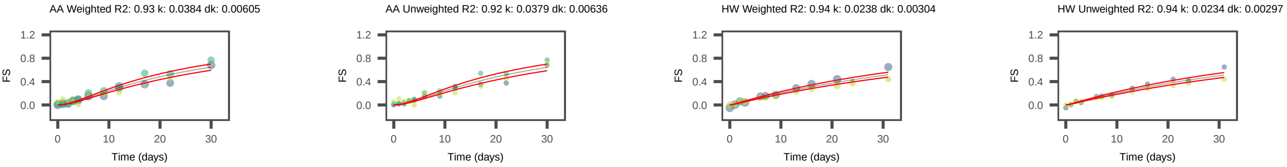

CACP

AA Weighted R2: 0.94 k: 0.0403 dk: 0.00602 | AA Unweighted R2: 0.93 k: 0.0397 dk: 0.00629 | HW Weighted R2: 0.94 k: 0.0272 dk: 0.00368 | HW Unweighted R2: 0.94 k: 0.0271 dk: 0.0037

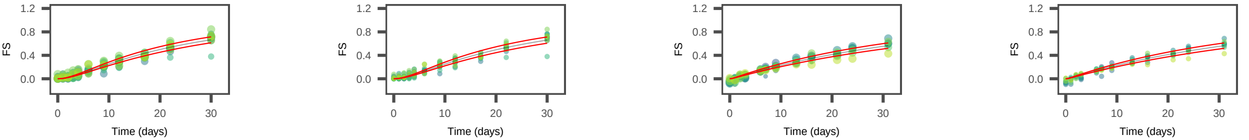

CAD13

AA Weighted R2: 0.97 k: 0.0522 dk: 0.00697 | AA Unweighted R2: 0.97 k: 0.0522 dk: 0.007 | HW Weighted R2: 0.92 k: 0.0431 dk: 0.00769 | HW Unweighted R2: 0.92 k: 0.0431 dk: 0.00769

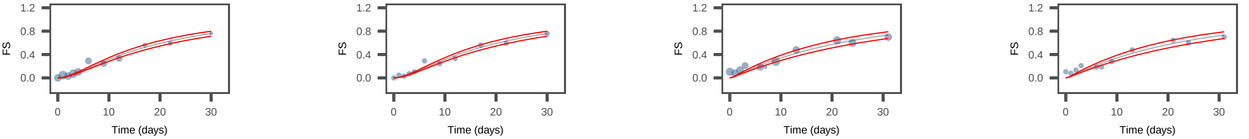

CADH2

AA Weighted R2: 0.91 k: 0.0688 dk: 0.0188 | AA Unweighted R2: 0.9 k: 0.0693 dk: 0.0194 | HW Weighted R2: 0.88 k: 0.0617 dk: 0.0172 | HW Unweighted R2: 0.89 k: 0.0613 dk: 0.017

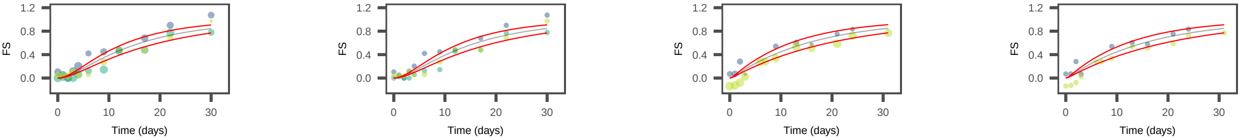

CALR

AA Weighted R2: 0.97 k: 0.248 dk: 0.0656 | AA Unweighted R2: 0.96 k: 0.255 dk: 0.0713 | HW Weighted R2: 0.87 k: 0.12 dk: 0.0487 | HW Unweighted R2: 0.87 k: 0.13 dk: 0.0519

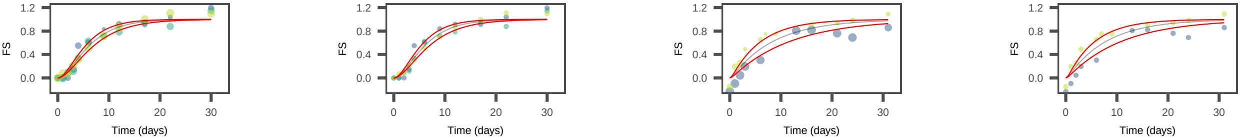

CAND2

AA Weighted R2: 0.96 k: 0.158 dk: 0.0317 | AA Unweighted R2: 0.96 k: 0.157 dk: 0.0318 | HW Weighted R2: 0.94 k: 0.0982 dk: 0.0253 | HW Unweighted R2: 0.94 k: 0.0984 dk: 0.0253

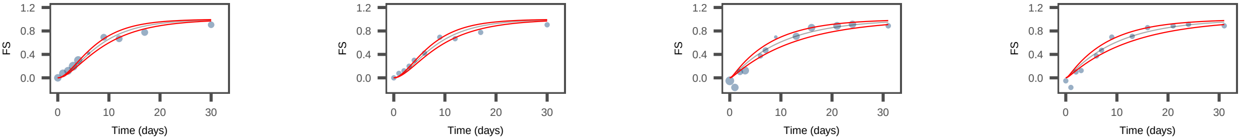

CASQ2

AA Weighted R2: 0.97 k: 0.0593 dk: 0.00766 | AA Unweighted R2: 0.97 k: 0.058 dk: 0.00795 | HW Weighted R2: 0.94 k: 0.0487 dk: 0.00848 | HW Unweighted R2: 0.94 k: 0.0482 dk: 0.00832

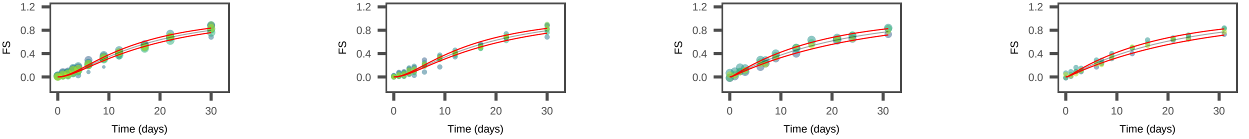

CATD

AA Weighted R2: 0.88 k: 0.185 dk: 0.066

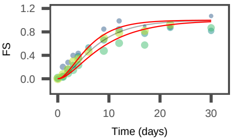

AA Unweighted R2: 0.87 k: 0.194 dk: 0.0728

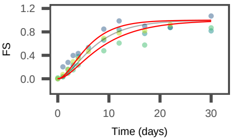

HW Weighted R2: 0.82 k: 0.122 dk: 0.0454

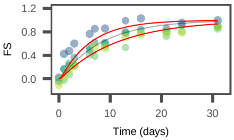

HW Unweighted R2: 0.82 k: 0.121 dk: 0.0443

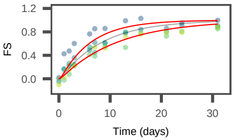

CAV1

AA Weighted R2: 0.93 k: 0.0745 dk: 0.0152

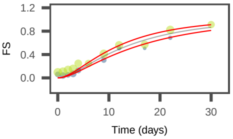

AA Unweighted R2: 0.93 k: 0.0732 dk: 0.0148

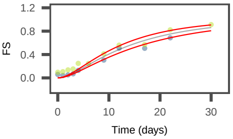

HW Weighted R2: 0.83 k: 0.0629 dk: 0.0206

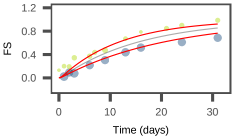

HW Unweighted R2: 0.82 k: 0.0663 dk: 0.0219

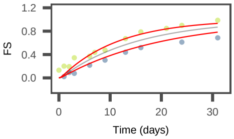

CAVN1

AA Weighted R2: 0.92 k: 0.0699 dk: 0.0158

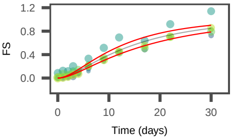

AA Unweighted R2: 0.93 k: 0.0677 dk: 0.0146

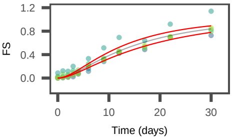

HW Weighted R2: 0.87 k: 0.048 dk: 0.0131

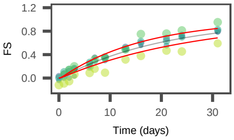

HW Unweighted R2: 0.89 k: 0.0484 dk: 0.0124

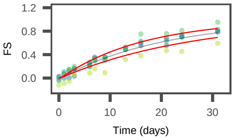

CAVN2

AA Weighted R2: 0.96 k: 0.0952 dk: 0.0189

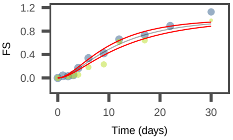

AA Unweighted R2: 0.95 k: 0.0939 dk: 0.0201

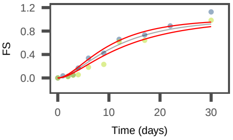

HW Weighted R2: 0.96 k: 0.0824 dk: 0.0166

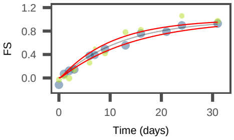

HW Unweighted R2: 0.95 k: 0.0845 dk: 0.019

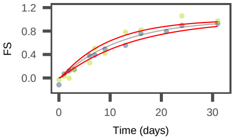

CAVN4

AA Weighted R2: 0.96 k: 0.0783 dk: 0.0162

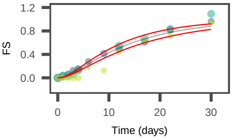

AA Unweighted R2: 0.95 k: 0.0765 dk: 0.0165

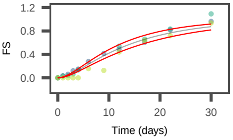

HW Weighted R2: 0.96 k: 0.0709 dk: 0.0125

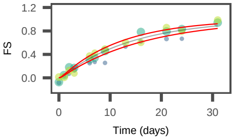

HW Unweighted R2: 0.95 k: 0.0668 dk: 0.0129

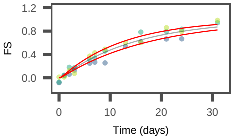

CAZA2

AA Weighted R2: 0.97 k: 0.0874 dk: 0.0157

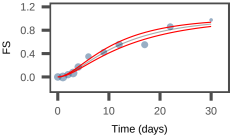

AA Unweighted R2: 0.97 k: 0.0877 dk: 0.016

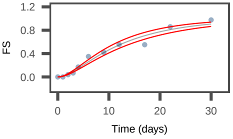

HW Weighted R2: 0.96 k: 0.0639 dk: 0.0122

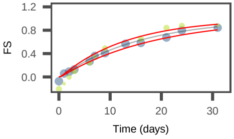

HW Unweighted R2: 0.95 k: 0.0646 dk: 0.0134

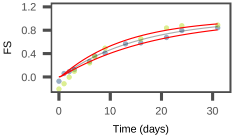

CD36

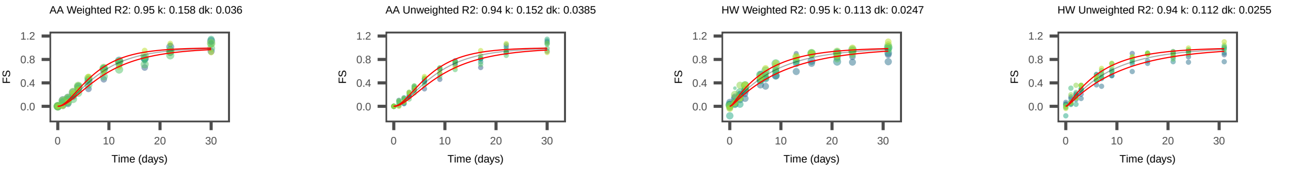

CH10

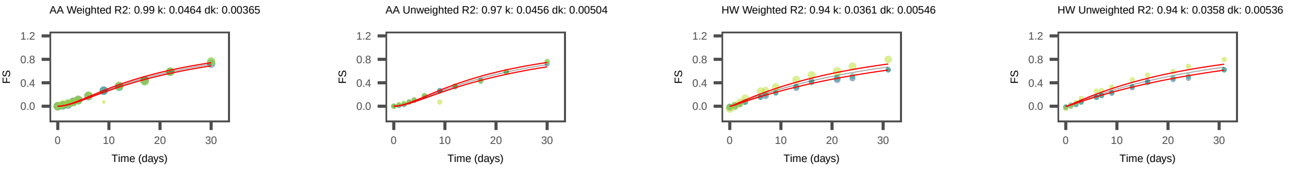

CH60

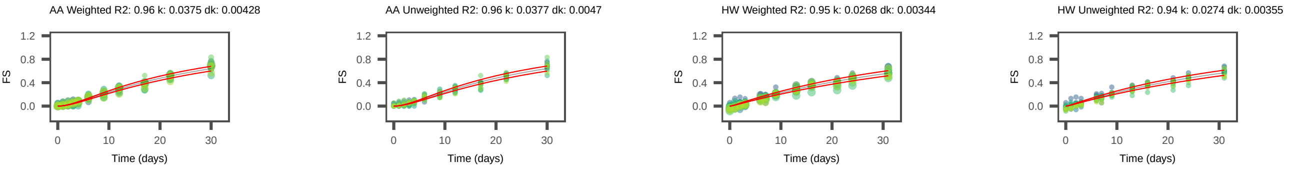

CISD1

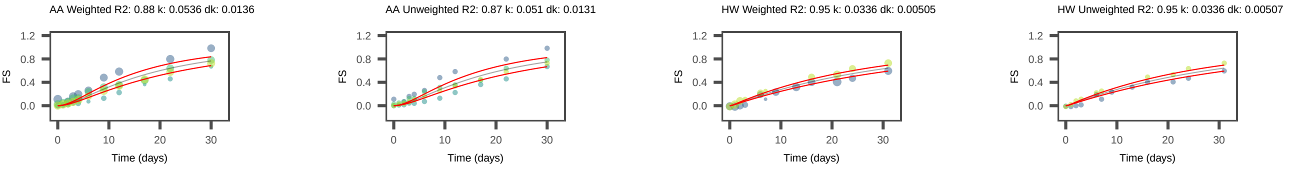

CISY

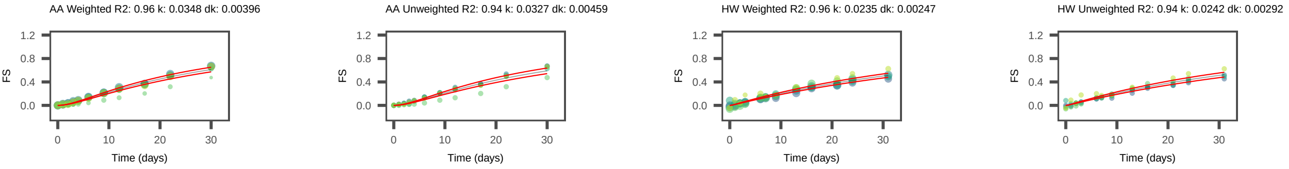

CLH1

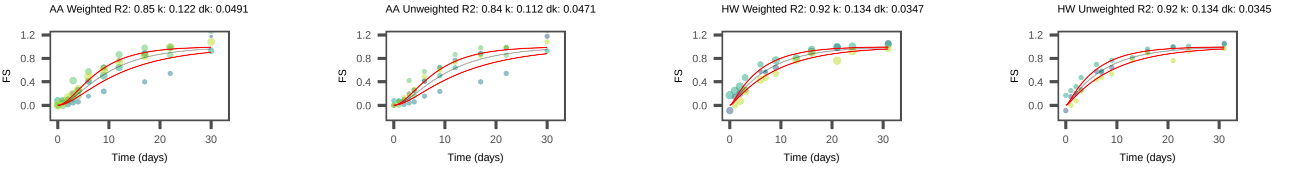

CLYBL

AA Weighted R2: 0.98 k: 0.0388 dk: 0.00375

AA Unweighted R2: 0.97 k: 0.039 dk: 0.00388

HW Weighted R2: 0.98 k: 0.0314 dk: 0.0024

HW Unweighted R2: 0.98 k: 0.0313 dk: 0.0024

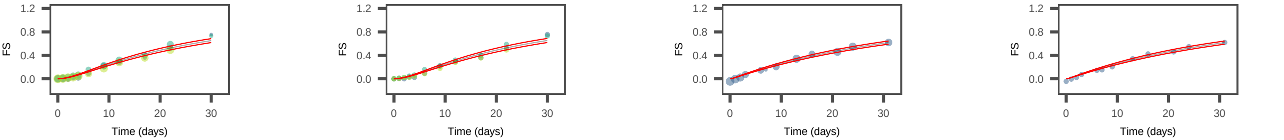

CMC1

AA Weighted R2: 0.93 k: 0.0353 dk: 0.00552

AA Unweighted R2: 0.93 k: 0.0352 dk: 0.00547

HW Weighted R2: 0.92 k: 0.0253 dk: 0.00385

HW Unweighted R2: 0.92 k: 0.0255 dk: 0.00396

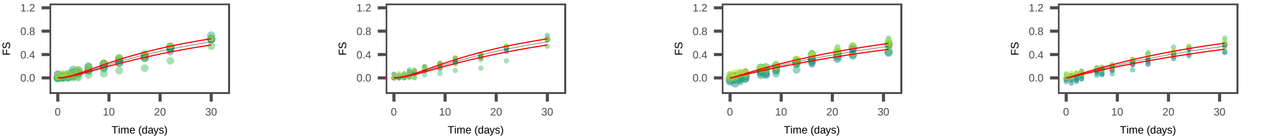

CMC2

AA Weighted R2: 0.95 k: 0.0375 dk: 0.00541

AA Unweighted R2: 0.95 k: 0.0374 dk: 0.00534

HW Weighted R2: 0.97 k: 0.0278 dk: 0.00274

HW Unweighted R2: 0.96 k: 0.0273 dk: 0.00309

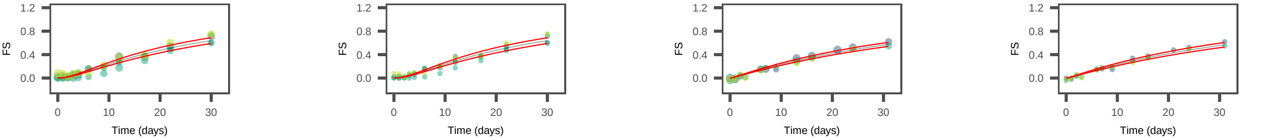

COF1

AA Weighted R2: 0.98 k: 0.189 dk: 0.0365

AA Unweighted R2: 0.97 k: 0.18 dk: 0.0376

HW Weighted R2: 0.94 k: 0.113 dk: 0.0294

HW Unweighted R2: 0.92 k: 0.115 dk: 0.0335

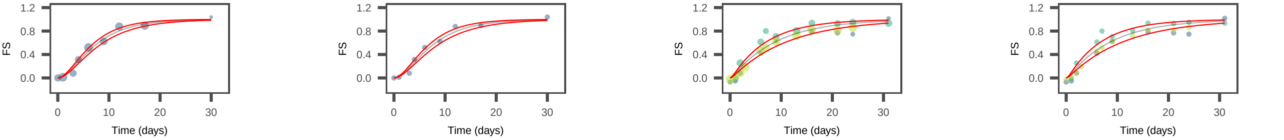

COF2

AA Weighted R2: 0.95 k: 0.101 dk: 0.0238

AA Unweighted R2: 0.95 k: 0.102 dk: 0.0236

HW Weighted R2: 0.97 k: 0.0884 dk: 0.0143

HW Unweighted R2: 0.96 k: 0.0884 dk: 0.0158

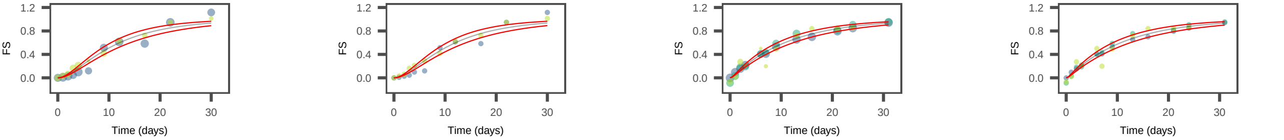

COMT

AA Weighted R2: 0.96 k: 0.189 dk: 0.0432

AA Unweighted R2: 0.96 k: 0.191 dk: 0.0441

HW Weighted R2: 0.81 k: 0.265 dk: 0.14

HW Unweighted R2: 0.81 k: 0.251 dk: 0.134

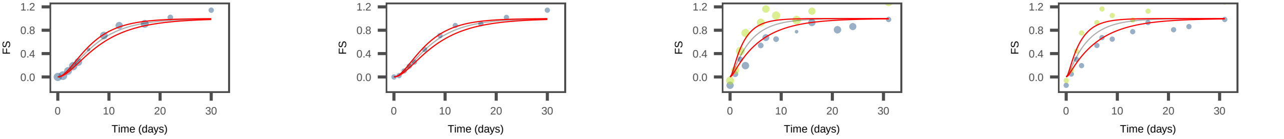

COQ5

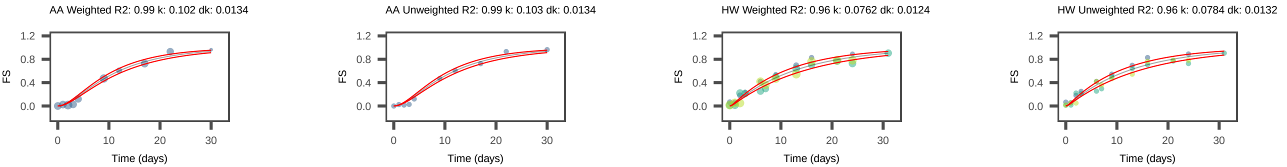

COQ6

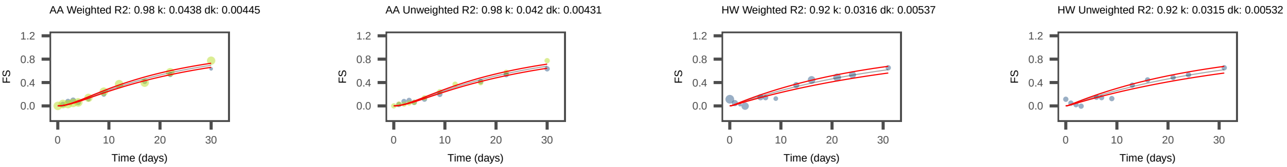

COQ8A

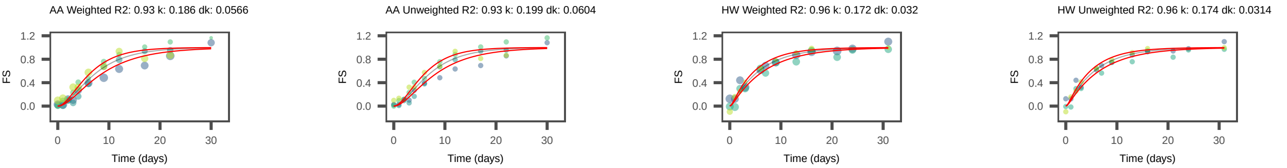

COQ9

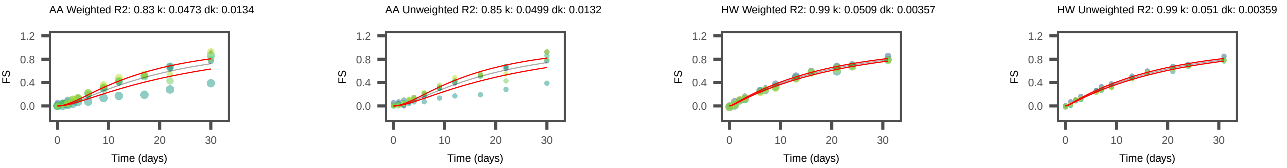

COX1

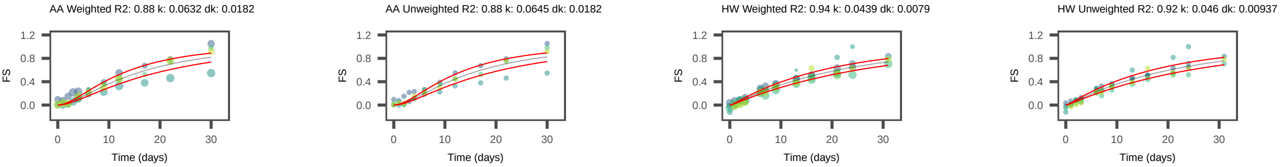

COX17

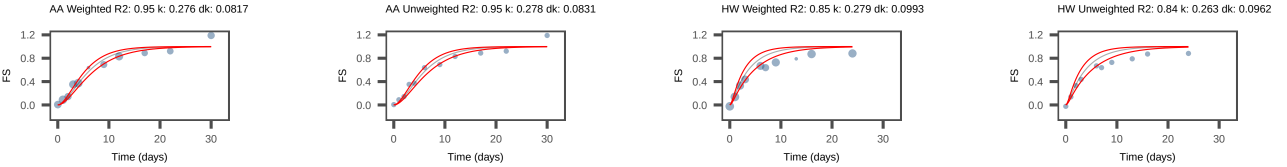

COX2

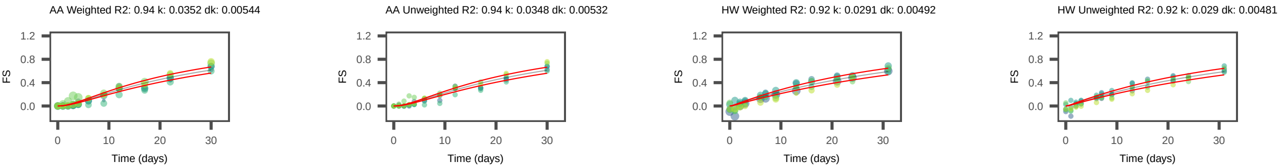

COX3

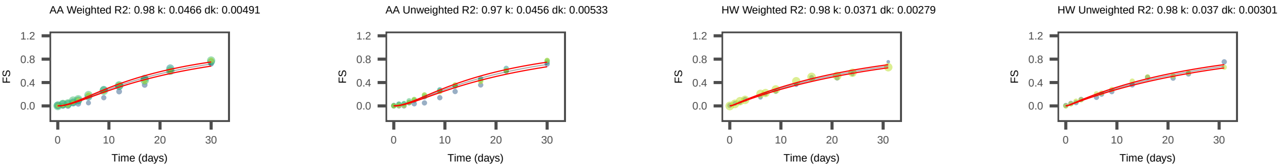

COX41

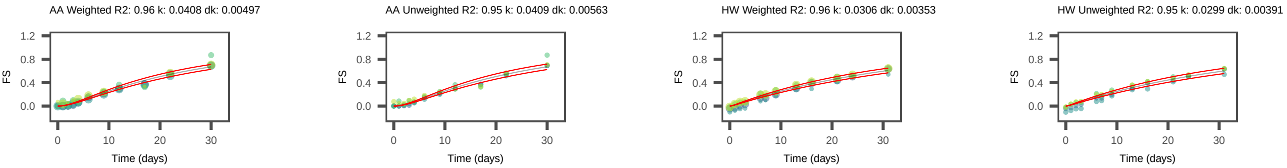

COX5A

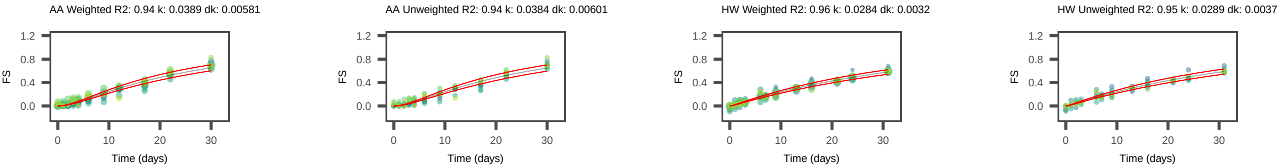

COX5B

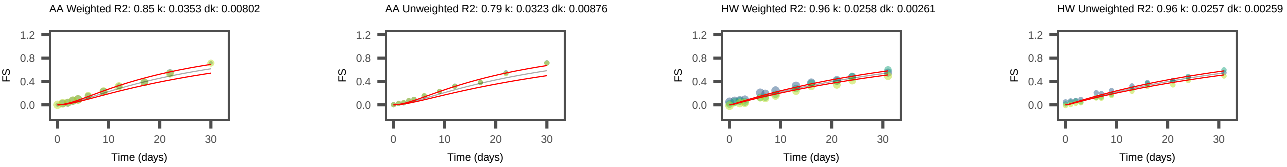

COX6C

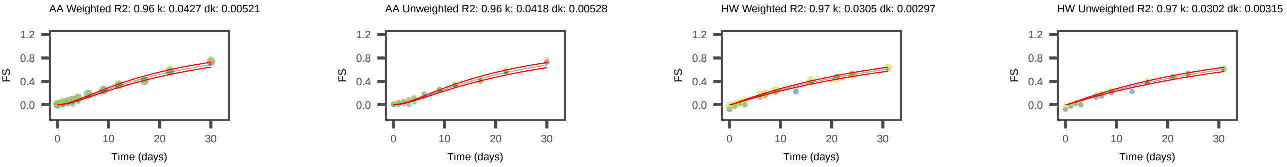

CPT1B

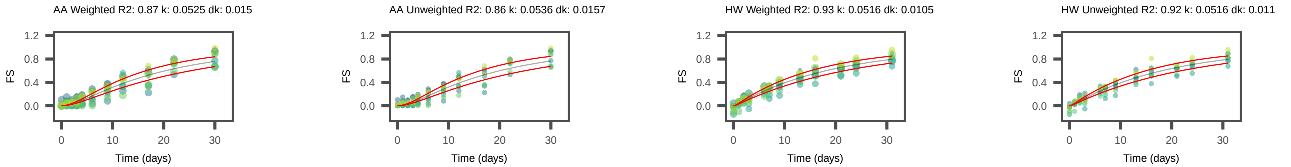

CPT2

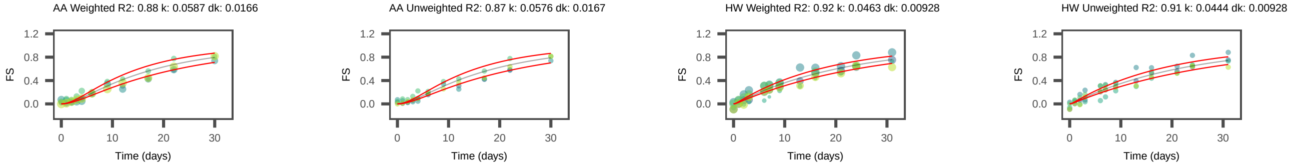

CRIP2

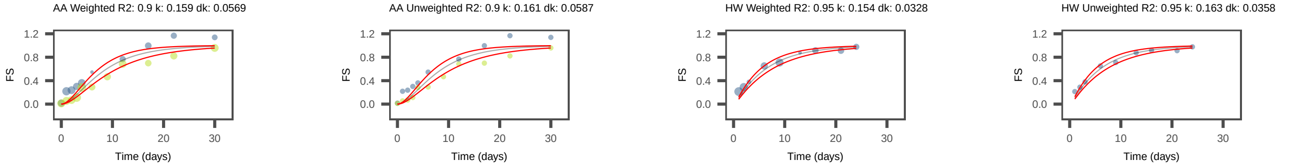

CRYAB

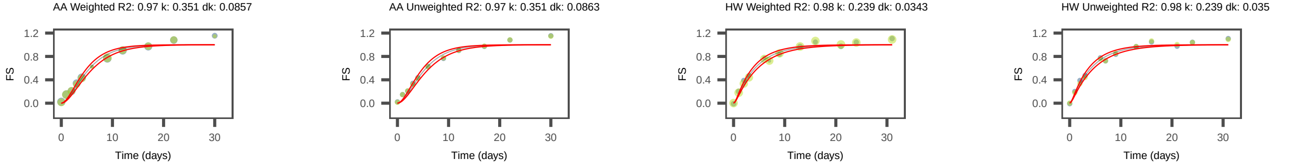

CSRP1

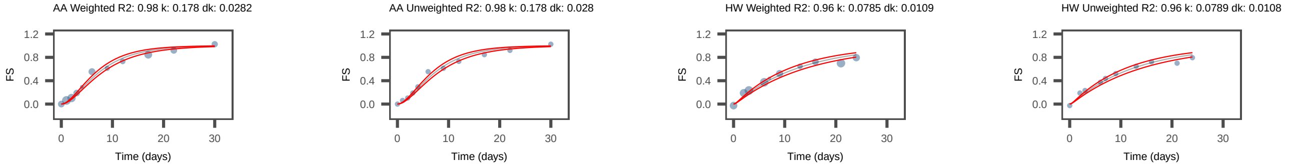

CSRP3

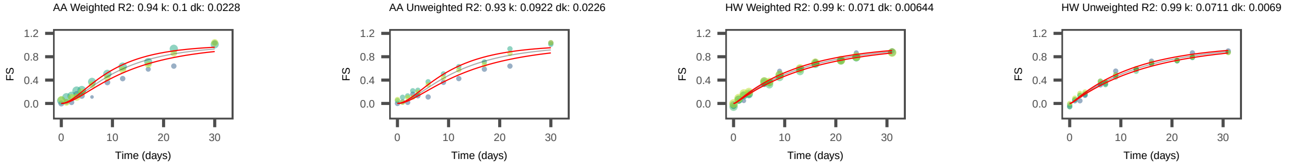

CTNA1

AA Weighted R2: 0.9 k: 0.424 dk: 0.226

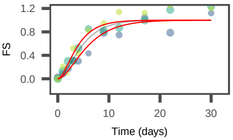

AA Unweighted R2: 0.89 k: 0.424 dk: 0.233

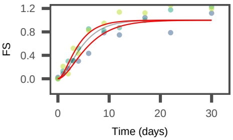

HW Weighted R2: 0.93 k: 0.33 dk: 0.0955

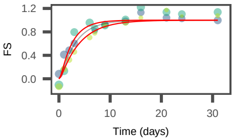

HW Unweighted R2: 0.93 k: 0.321 dk: 0.0915

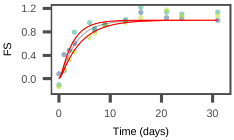

CX6B1

AA Weighted R2: 0.92 k: 0.0543 dk: 0.012

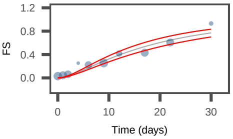

AA Unweighted R2: 0.9 k: 0.0568 dk: 0.0138

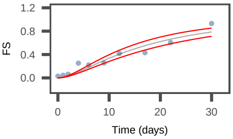

HW Weighted R2: 0.98 k: 0.0253 dk: 0.00189

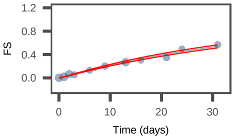

HW Unweighted R2: 0.98 k: 0.0253 dk: 0.0019

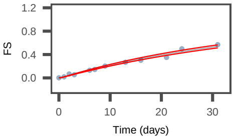

CX7A2

AA Weighted R2: 0.92 k: 0.0451 dk: 0.0108

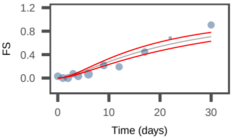

AA Unweighted R2: 0.91 k: 0.0484 dk: 0.0121

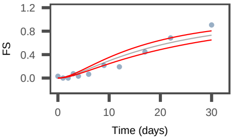

HW Weighted R2: 0.97 k: 0.0649 dk: 0.00933

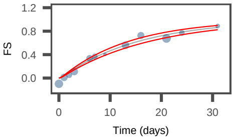

HW Unweighted R2: 0.98 k: 0.065 dk: 0.00918

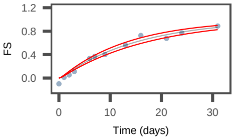

CY1

AA Weighted R2: 0.93 k: 0.0308 dk: 0.00421

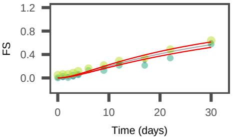

AA Unweighted R2: 0.93 k: 0.0301 dk: 0.00406

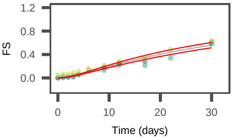

HW Weighted R2: 0.92 k: 0.024 dk: 0.00371

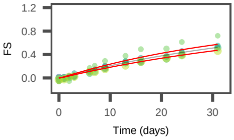

HW Unweighted R2: 0.91 k: 0.0246 dk: 0.00388

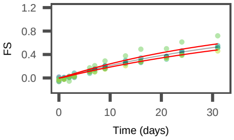

D39U1

AA Weighted R2: 0.94 k: 0.0352 dk: 0.00497

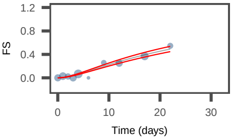

AA Unweighted R2: 0.93 k: 0.0354 dk: 0.00531

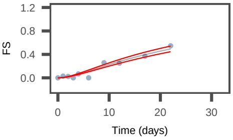

HW Weighted R2: 0.9 k: 0.0356 dk: 0.00806

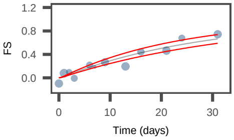

HW Unweighted R2: 0.9 k: 0.0357 dk: 0.00804

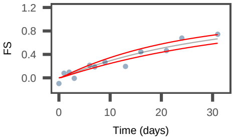

DECR

AA Weighted R2: 0.96 k: 0.0328 dk: 0.00364

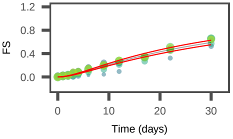

AA Unweighted R2: 0.95 k: 0.032 dk: 0.00401

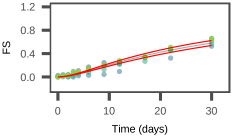

HW Weighted R2: 0.93 k: 0.0267 dk: 0.00369

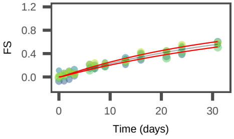

HW Unweighted R2: 0.93 k: 0.027 dk: 0.00375

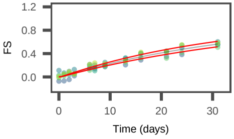

DESM

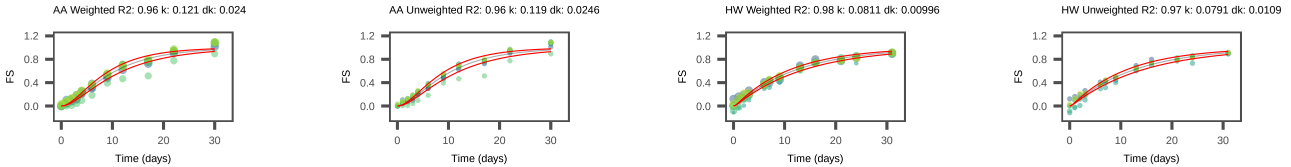

DESP

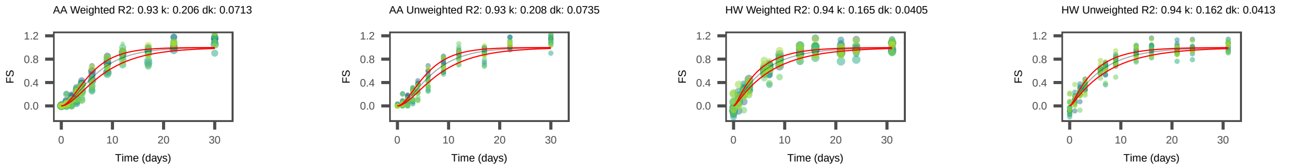

DEST

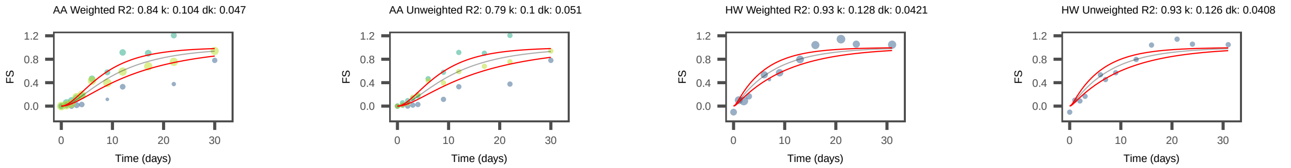

DHB4

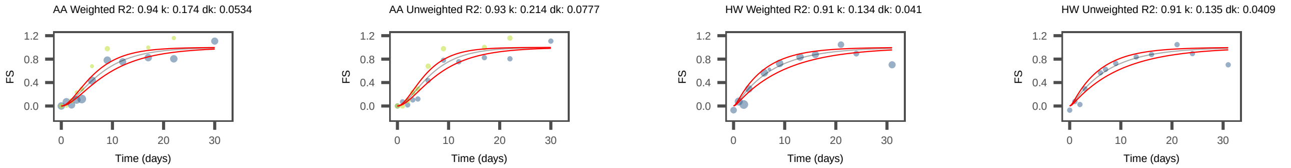

DHPR

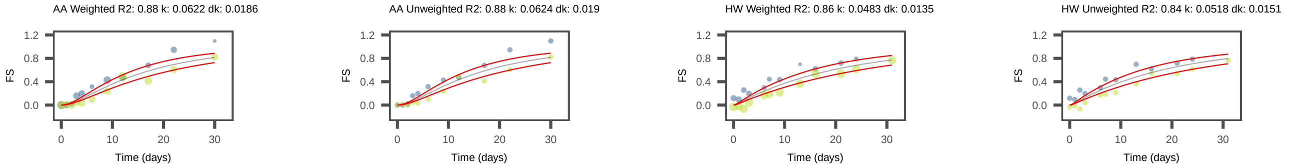

DHRS4

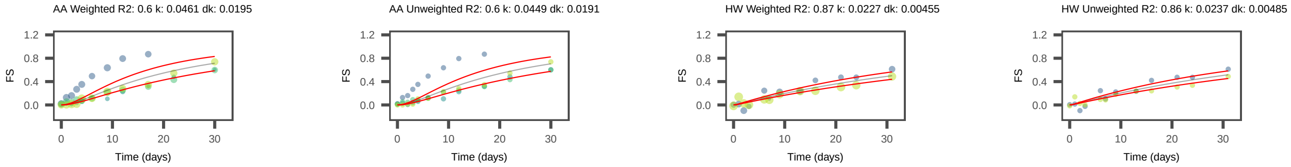

DLDH

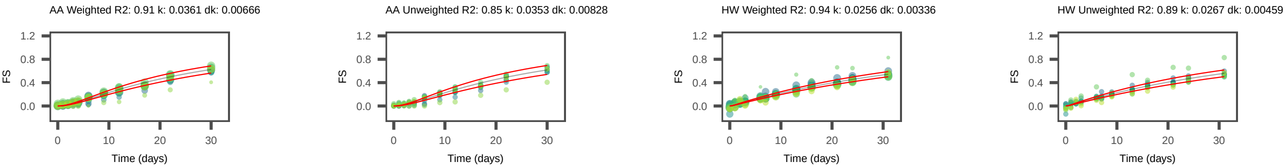

DMD

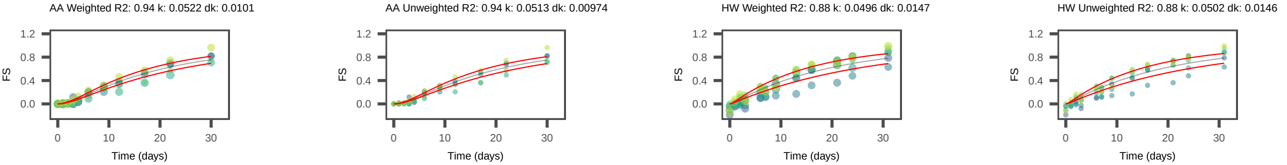

DNJA2

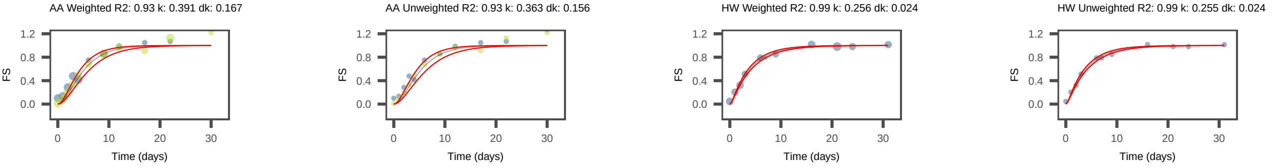

DNM1L

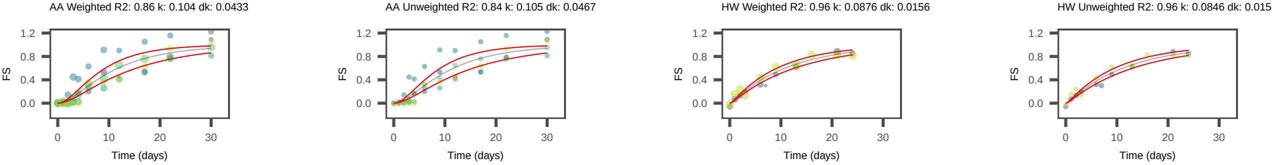

DRS7C

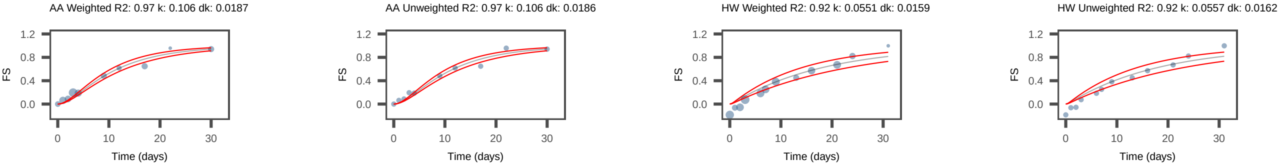

DYHC1

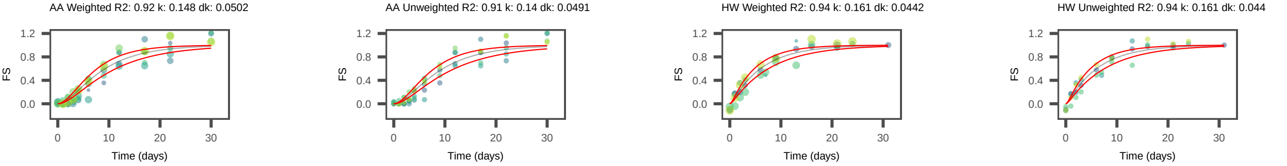

ECH1

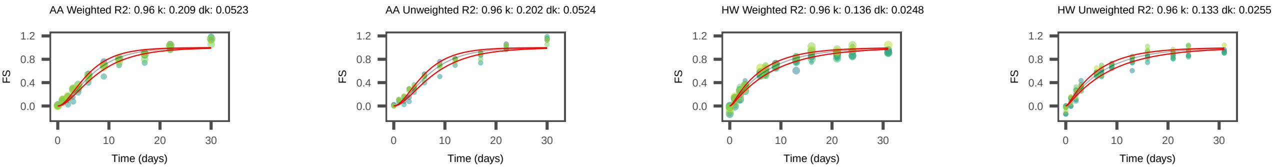

ECHA

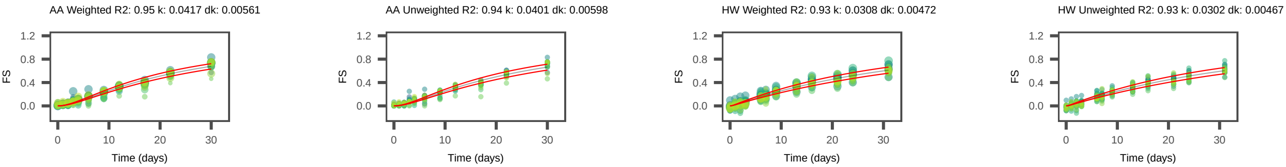

ECHB

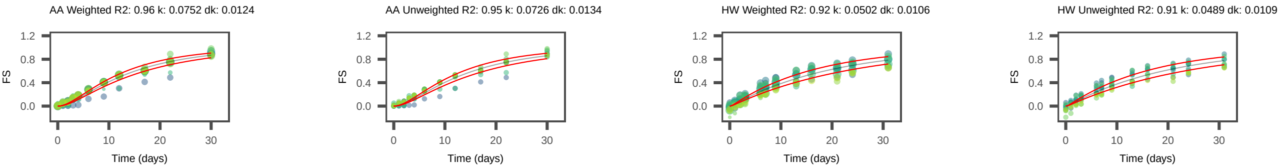

ECHM

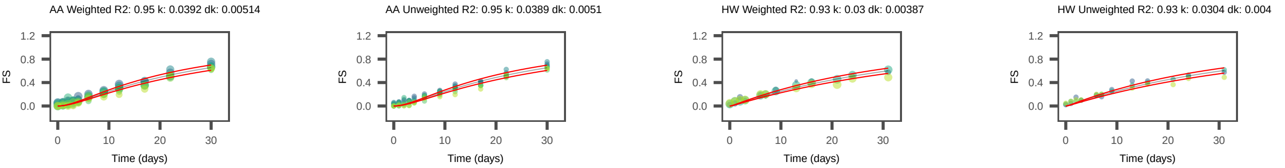

ECI1

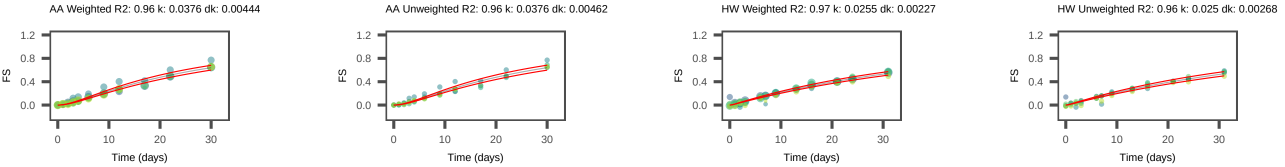

ECI2

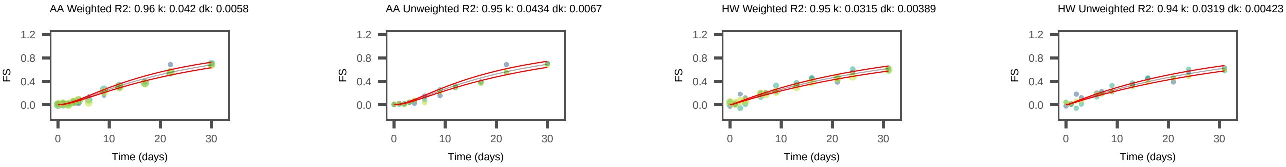

EF1A1

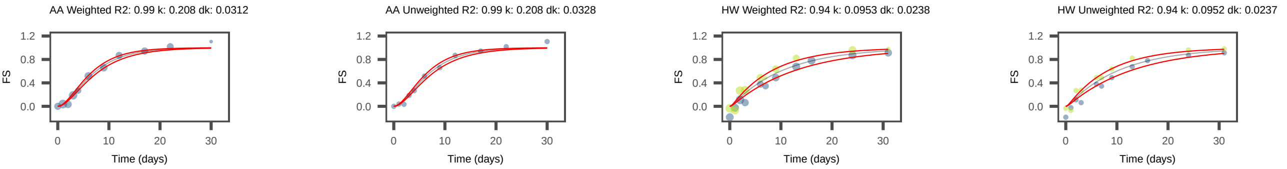

EF1A2

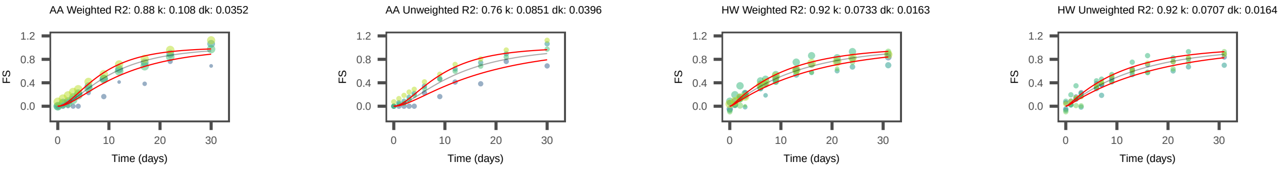

EF1G

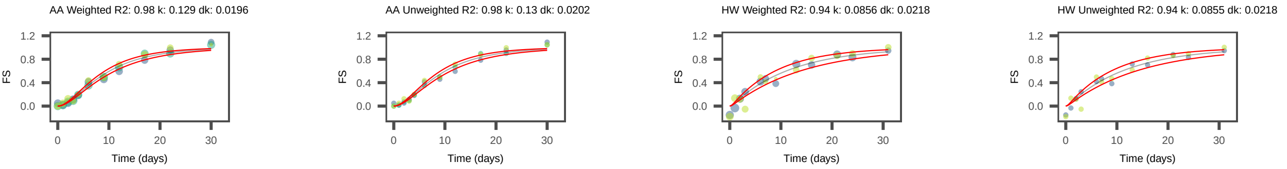

EF2

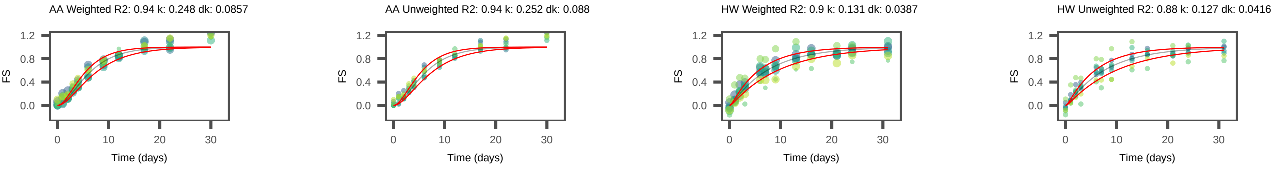

EFTU

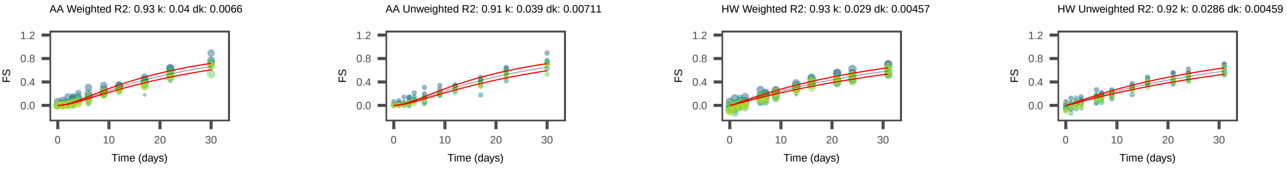

EHD2

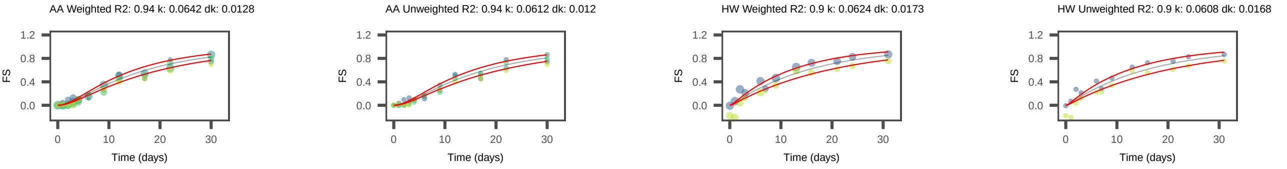

EHD4

AA Weighted R2: 0.96 k: 0.236 dk: 0.0598 | AA Unweighted R2: 0.96 k: 0.234 dk: 0.0634 | HW Weighted R2: 0.94 k: 0.216 dk: 0.0558 | HW Unweighted R2: 0.94 k: 0.216 dk: 0.0558

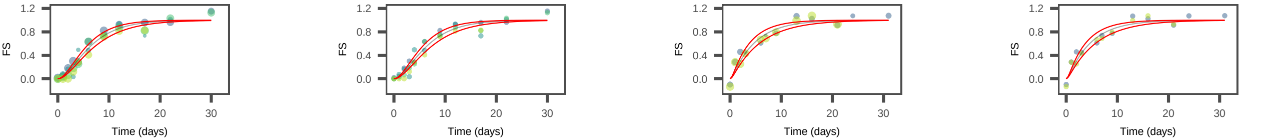

ENOA

AA Weighted R2: 0.84 k: 0.0719 dk: 0.0254 | AA Unweighted R2: 0.81 k: 0.0822 dk: 0.0324 | HW Weighted R2: 0.93 k: 0.0486 dk: 0.0089 | HW Unweighted R2: 0.93 k: 0.0471 dk: 0.00904

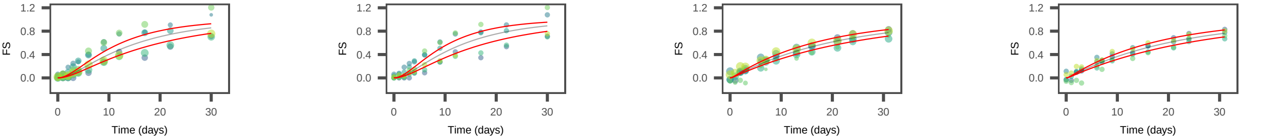

ENOB

AA Weighted R2: 0.97 k: 0.0822 dk: 0.0129 | AA Unweighted R2: 0.97 k: 0.0803 dk: 0.0131 | HW Weighted R2: 0.97 k: 0.0598 dk: 0.00758 | HW Unweighted R2: 0.97 k: 0.06 dk: 0.00767

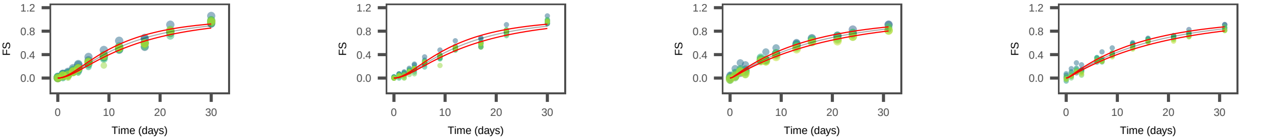

ENPL

AA Weighted R2: 0.9 k: 0.277 dk: 0.126 | AA Unweighted R2: 0.9 k: 0.28 dk: 0.129 | HW Weighted R2: 0.84 k: 0.113 dk: 0.0392 | HW Unweighted R2: 0.84 k: 0.114 dk: 0.04

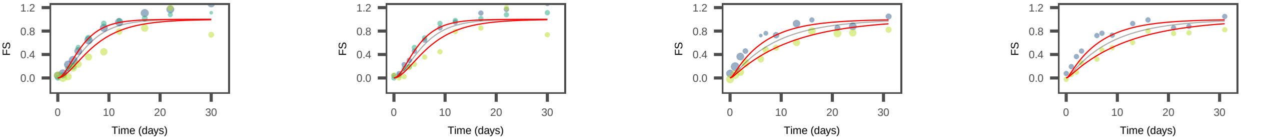

EST1D

AA Weighted R2: 0.9 k: 0.175 dk: 0.0642 | AA Unweighted R2: 0.9 k: 0.173 dk: 0.0627 | HW Weighted R2: 0.92 k: 0.127 dk: 0.0362 | HW Unweighted R2: 0.91 k: 0.127 dk: 0.0368

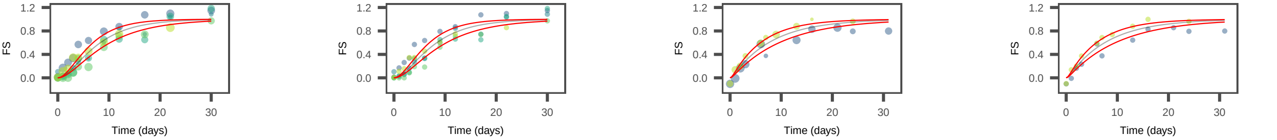

ETFA

AA Weighted R2: 0.95 k: 0.0368 dk: 0.00452 | AA Unweighted R2: 0.94 k: 0.0353 dk: 0.00493 | HW Weighted R2: 0.96 k: 0.0269 dk: 0.00279 | HW Unweighted R2: 0.95 k: 0.0266 dk: 0.00295

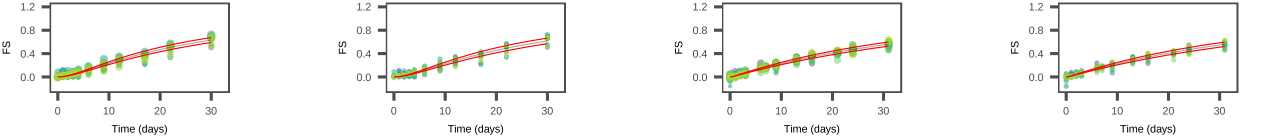

ETFB

AA Weighted R2: 0.93 k: 0.0378 dk: 0.00547

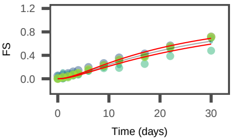

AA Unweighted R2: 0.93 k: 0.0372 dk: 0.00543

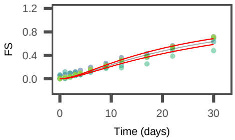

HW Weighted R2: 0.93 k: 0.0297 dk: 0.00428

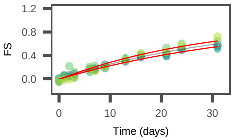

HW Unweighted R2: 0.92 k: 0.0288 dk: 0.00436

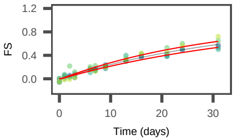

ETFD

AA Weighted R2: 0.96 k: 0.0354 dk: 0.00408

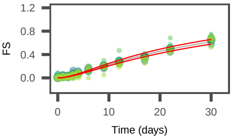

AA Unweighted R2: 0.95 k: 0.0354 dk: 0.00454

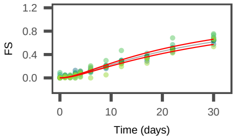

HW Weighted R2: 0.94 k: 0.0276 dk: 0.00339

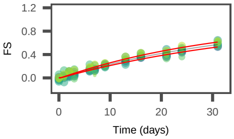

HW Unweighted R2: 0.94 k: 0.0276 dk: 0.00348

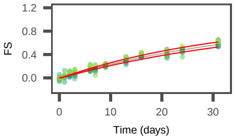

FABP4

AA Weighted R2: 0.98 k: 0.092 dk: 0.0105

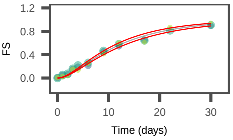

AA Unweighted R2: 0.98 k: 0.091 dk: 0.0105

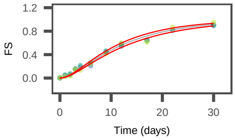

HW Weighted R2: 0.94 k: 0.0649 dk: 0.0139

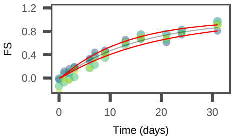

HW Unweighted R2: 0.94 k: 0.0644 dk: 0.0144

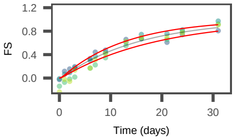

FERM2

AA Weighted R2: 0.94 k: 0.161 dk: 0.039

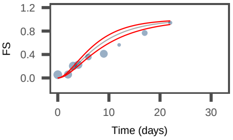

AA Unweighted R2: 0.93 k: 0.129 dk: 0.0316

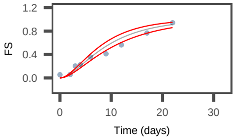

HW Weighted R2: 0.98 k: 0.101 dk: 0.0145

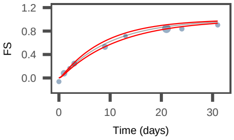

HW Unweighted R2: 0.98 k: 0.103 dk: 0.0153

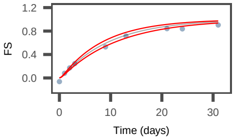

FHL1

AA Weighted R2: 0.92 k: 0.152 dk: 0.0482

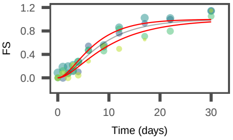

AA Unweighted R2: 0.92 k: 0.145 dk: 0.0475

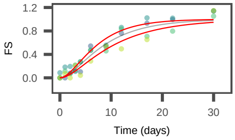

HW Weighted R2: 0.94 k: 0.142 dk: 0.0347

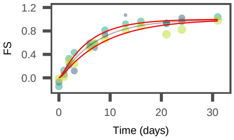

HW Unweighted R2: 0.94 k: 0.143 dk: 0.0357

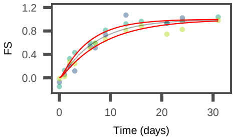

FHL2

AA Weighted R2: 0.95 k: 0.18 dk: 0.0437

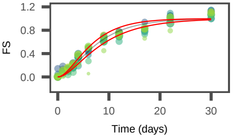

AA Unweighted R2: 0.94 k: 0.17 dk: 0.0458

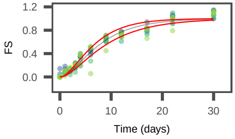

HW Weighted R2: 0.95 k: 0.131 dk: 0.0294

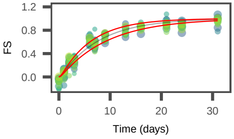

HW Unweighted R2: 0.94 k: 0.13 dk: 0.0319

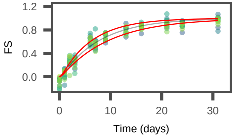

FIBA

AA Weighted R2: 0.9 k: 8.64 dk: 34.3 | AA Unweighted R2: 0.89 k: 7.76 dk: 28.6 | HW Weighted R2: 0.72 k: 5.28 dk: 9.01 | HW Unweighted R2: 0.76 k: 4.21 dk: 4.63

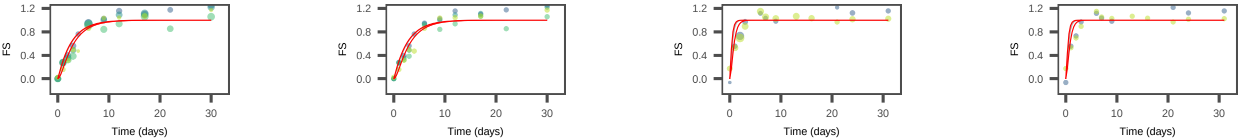

FIBB

AA Weighted R2: 0.91 k: 5.53 dk: 12.7 | AA Unweighted R2: 0.9 k: 4.5 dk: 8.84 | HW Weighted R2: 0.92 k: 0.484 dk: 0.142 | HW Unweighted R2: 0.92 k: 0.487 dk: 0.145

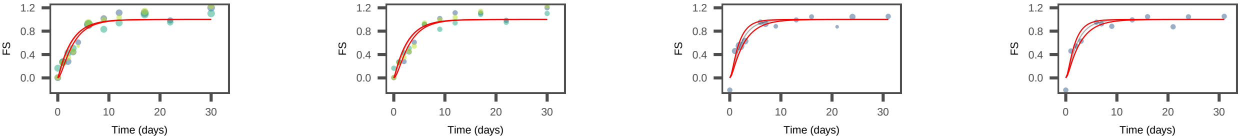

FIS1

AA Weighted R2: 0.97 k: 0.0702 dk: 0.0113 | AA Unweighted R2: 0.96 k: 0.0691 dk: 0.0119 | HW Weighted R2: 0.99 k: 0.0718 dk: 0.0053 | HW Unweighted R2: 0.99 k: 0.0719 dk: 0.00535

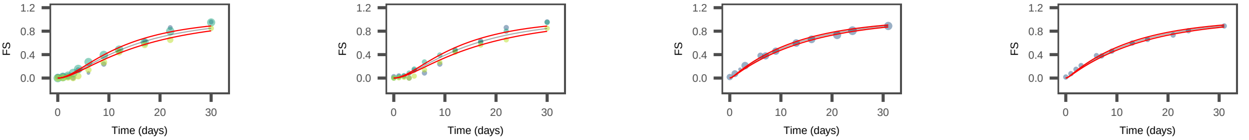

FKBP3

AA Weighted R2: 0.94 k: 0.417 dk: 0.19 | AA Unweighted R2: 0.94 k: 0.406 dk: 0.181 | HW Weighted R2: 0.97 k: 0.197 dk: 0.0352 | HW Unweighted R2: 0.97 k: 0.196 dk: 0.0347

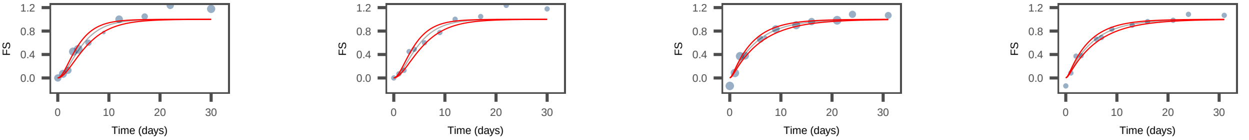

FLNC

AA Weighted R2: 0.84 k: 0.125 dk: 0.0439 | AA Unweighted R2: 0.85 k: 0.119 dk: 0.0406 | HW Weighted R2: 0.9 k: 0.097 dk: 0.0285 | HW Unweighted R2: 0.9 k: 0.0996 dk: 0.0299

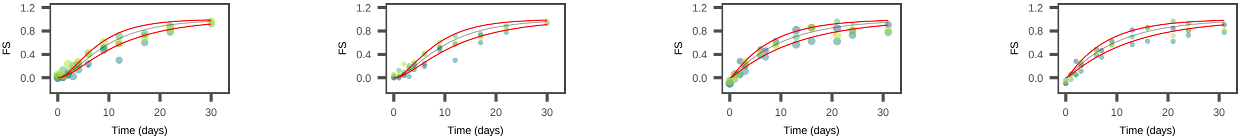

FMO1

AA Weighted R2: 0.98 k: 0.118 dk: 0.0191 | AA Unweighted R2: 0.98 k: 0.118 dk: 0.0187 | HW Weighted R2: 0.9 k: 0.0628 dk: 0.0217 | HW Unweighted R2: 0.9 k: 0.0624 dk: 0.0212

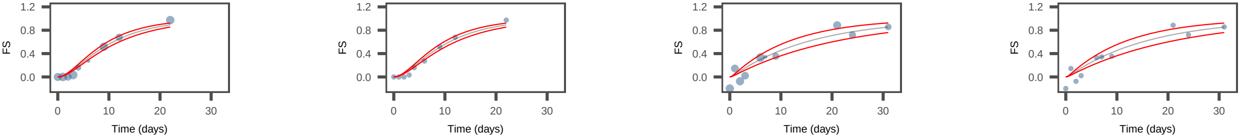

FRIH

AA Weighted R2: 0.97 k: 0.147 dk: 0.0314 | AA Unweighted R2: 0.96 k: 0.143 dk: 0.0334 | HW Weighted R2: 0.81 k: 0.0986 dk: 0.0439 | HW Unweighted R2: 0.81 k: 0.096 dk: 0.0426

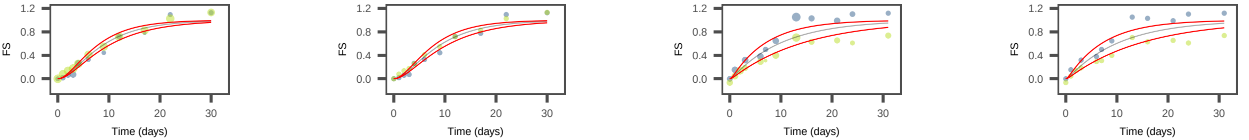

FUMH

AA Weighted R2: 0.96 k: 0.0404 dk: 0.00518 | AA Unweighted R2: 0.94 k: 0.0409 dk: 0.00606 | HW Weighted R2: 0.94 k: 0.027 dk: 0.00337 | HW Unweighted R2: 0.94 k: 0.0271 dk: 0.00342

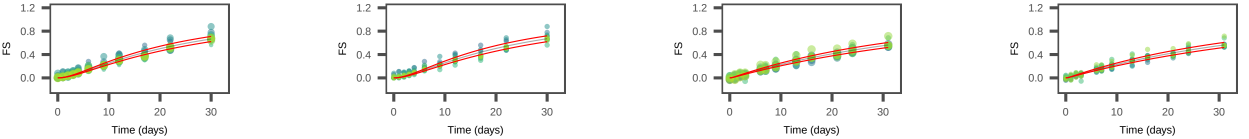

G3P

AA Weighted R2: 0.95 k: 0.113 dk: 0.0258 | AA Unweighted R2: 0.94 k: 0.105 dk: 0.0268 | HW Weighted R2: 0.96 k: 0.0721 dk: 0.0124 | HW Unweighted R2: 0.96 k: 0.074 dk: 0.0132

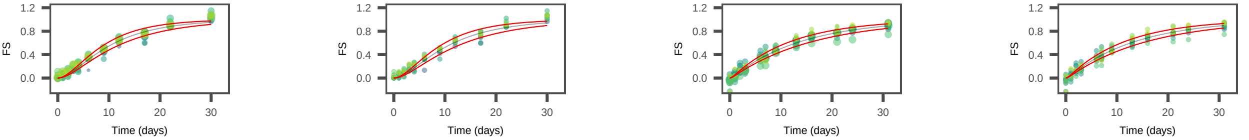

G6PI

AA Weighted R2: 0.97 k: 0.0913 dk: 0.0148 | AA Unweighted R2: 0.96 k: 0.0921 dk: 0.0167 | HW Weighted R2: 0.87 k: 0.0699 dk: 0.0216 | HW Unweighted R2: 0.89 k: 0.0693 dk: 0.0199

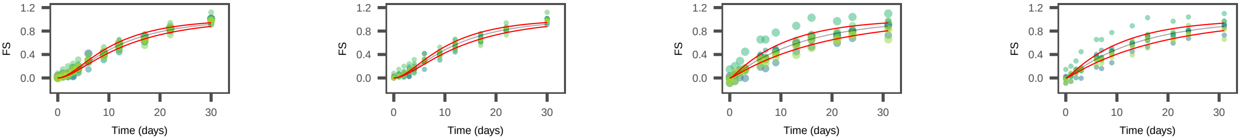

GAL3A

AA Weighted R2: 0.95 k: 0.0388 dk: 0.00513 | AA Unweighted R2: 0.93 k: 0.0365 dk: 0.00572 | HW Weighted R2: 0.94 k: 0.0294 dk: 0.00395 | HW Unweighted R2: 0.93 k: 0.029 dk: 0.00416

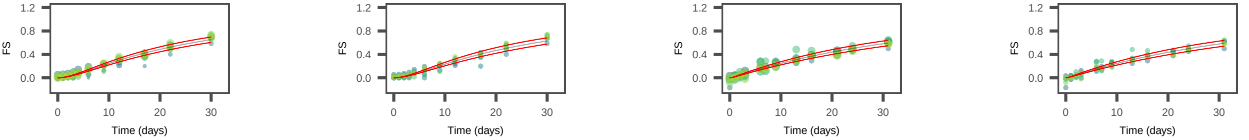

GBB1

AA Weighted R2: 0.92 k: 0.0449 dk: 0.00895 | AA Unweighted R2: 0.92 k: 0.0444 dk: 0.00893 | HW Weighted R2: 0.97 k: 0.126 dk: 0.0215 | HW Unweighted R2: 0.97 k: 0.126 dk: 0.0216

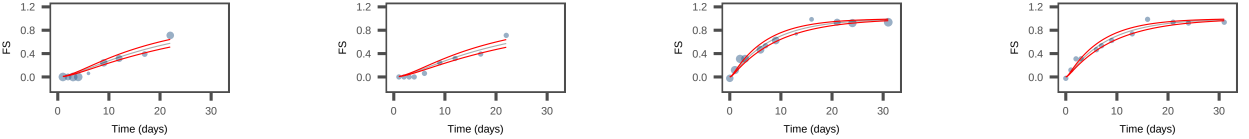

GBB2

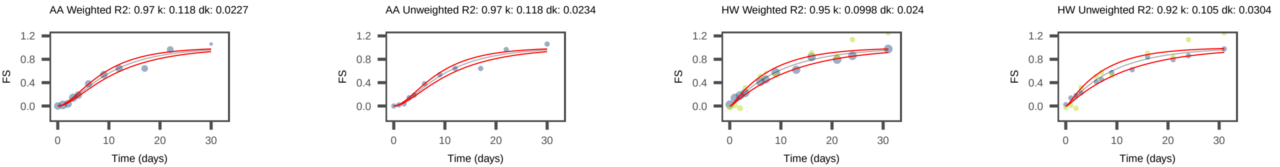

GCDH

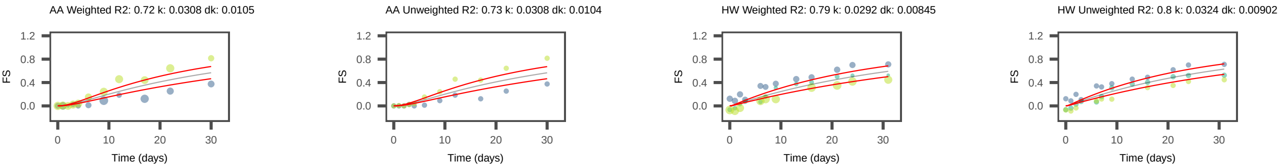

GDIA

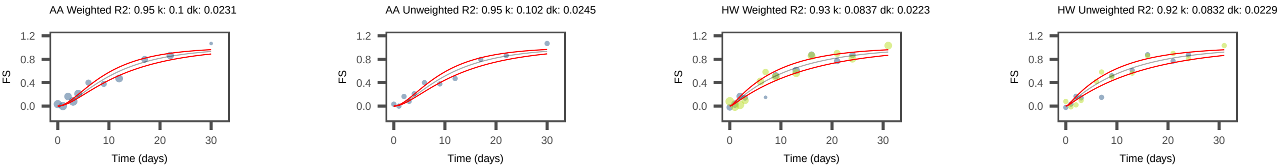

GDIB

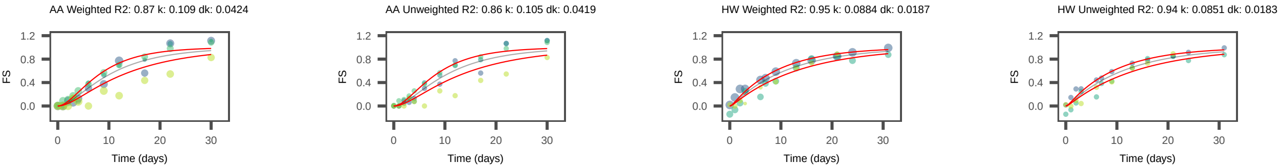

GLOD4

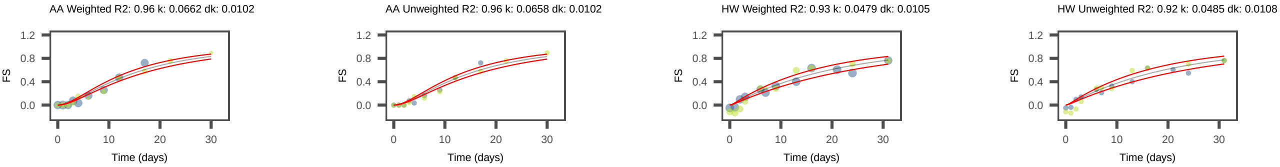

GLRX5

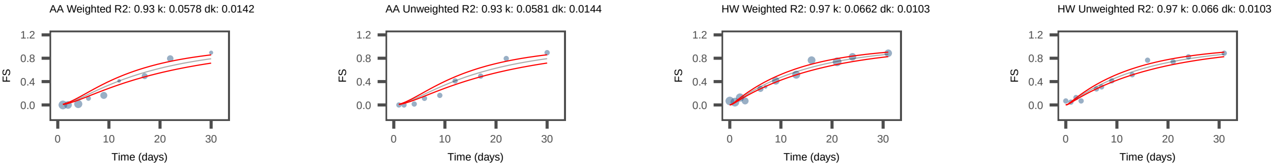

GLYG

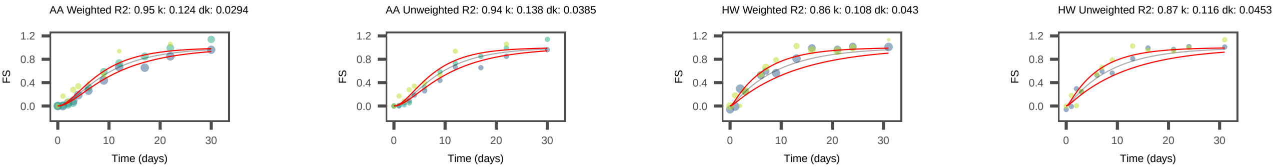

GRP75

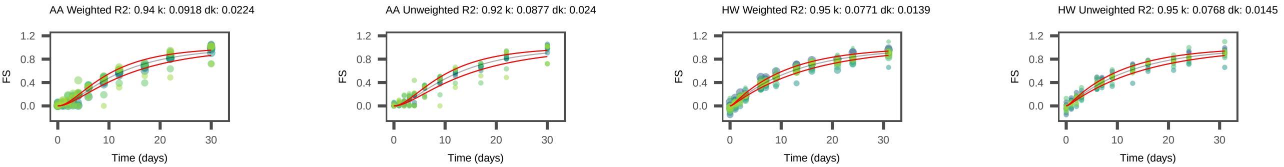

GRPE1

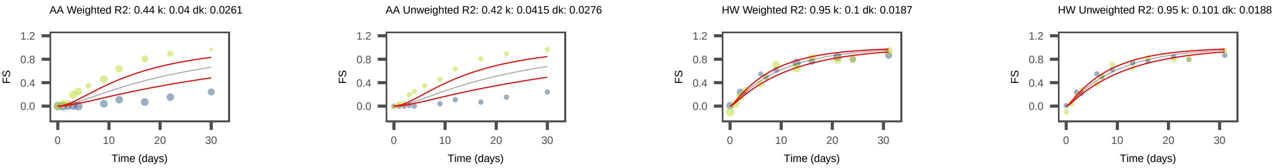

GSTA4

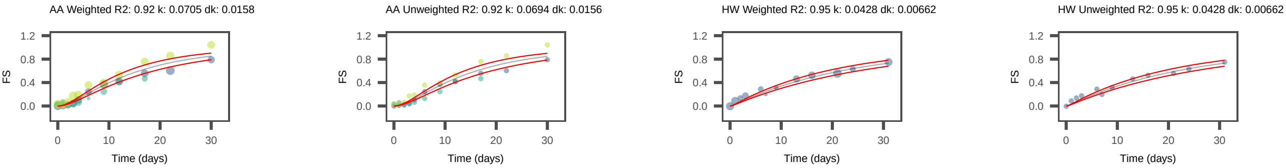

GSTK1

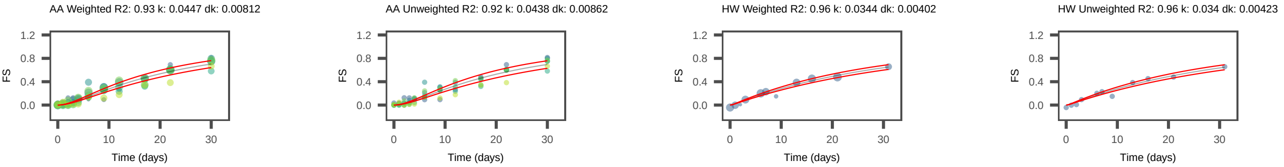

GSTM1

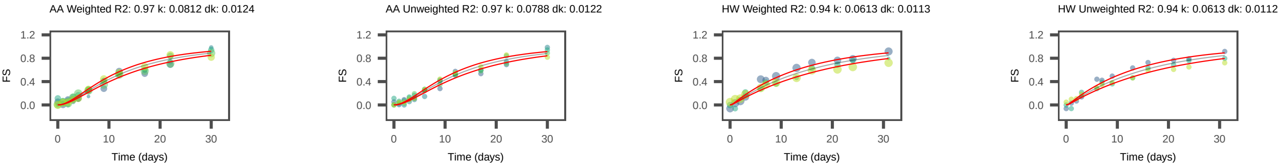

GSTM2

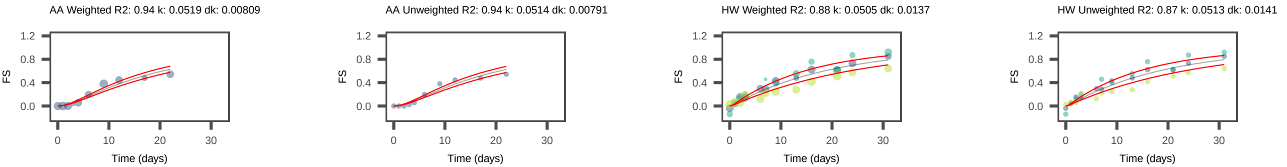

GSTO1

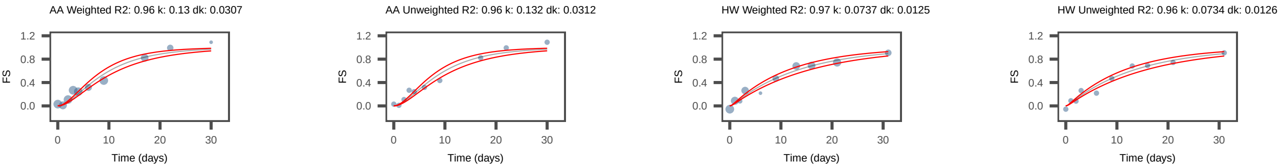

HBA

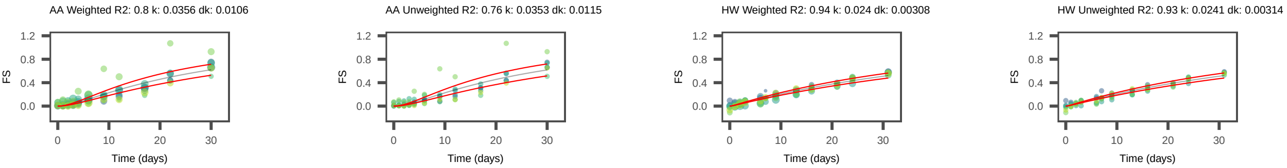

HBB1

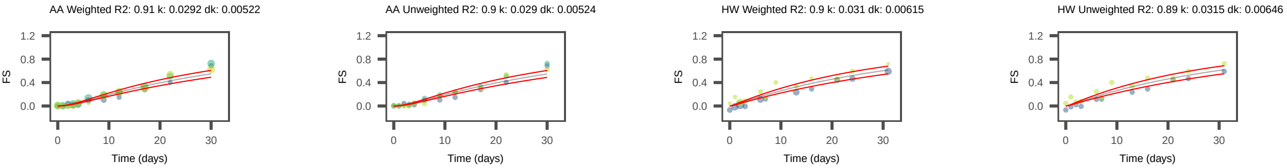

HCD2

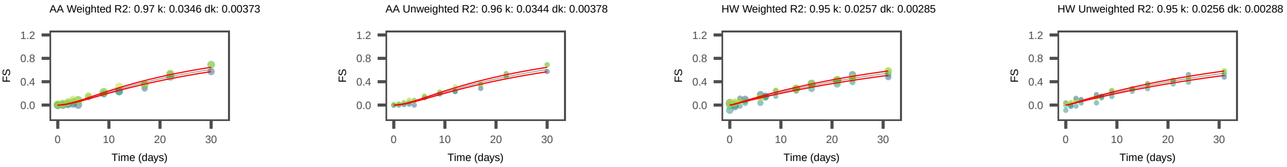

HCDH

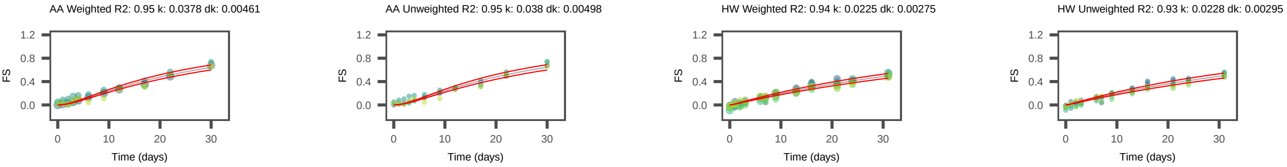

HDHD2

AA Weighted R2: 0.85 k: 0.0454 dk: 0.0127

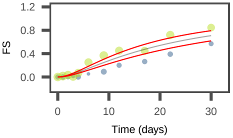

AA Unweighted R2: 0.84 k: 0.0403 dk: 0.0114

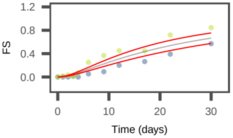

HW Weighted R2: 0.97 k: 0.0465 dk: 0.00651

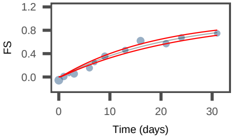

HW Unweighted R2: 0.97 k: 0.0465 dk: 0.00645

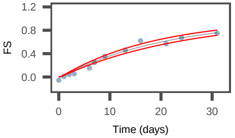

HEM2

AA Weighted R2: 0.95 k: 0.106 dk: 0.0266

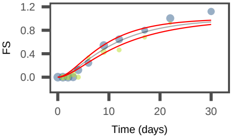

AA Unweighted R2: 0.95 k: 0.103 dk: 0.0265

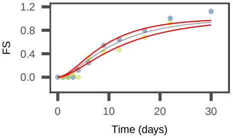

HW Weighted R2: 0.95 k: 0.114 dk: 0.0257

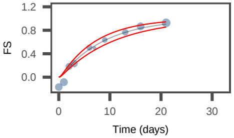

HW Unweighted R2: 0.96 k: 0.114 dk: 0.025

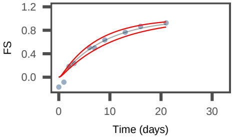

HEMO

AA Weighted R2: 0.91 k: 1.48 dk: 1.37

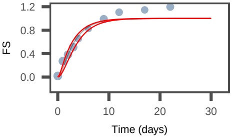

AA Unweighted R2: 0.9 k: 1.46 dk: 1.41

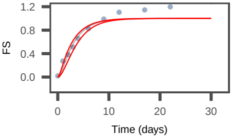

HW Weighted R2: 0.9 k: 0.708 dk: 0.172

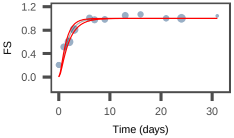

HW Unweighted R2: 0.9 k: 0.715 dk: 0.176

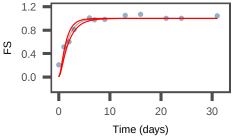

HHATL

AA Weighted R2: 0.97 k: 0.0434 dk: 0.00456

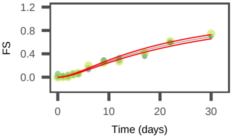

AA Unweighted R2: 0.97 k: 0.0429 dk: 0.00457

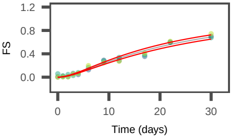

HW Weighted R2: 0.93 k: 0.0276 dk: 0.00466

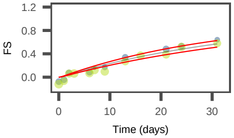

HW Unweighted R2: 0.93 k: 0.0279 dk: 0.0046

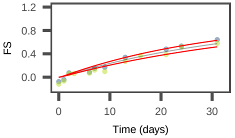

HIBCH

AA Weighted R2: 0.93 k: 0.0336 dk: 0.00497

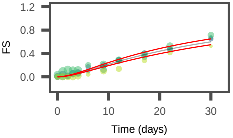

AA Unweighted R2: 0.93 k: 0.0335 dk: 0.00496

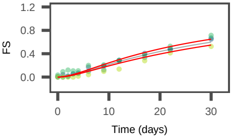

HW Weighted R2: 0.86 k: 0.0264 dk: 0.00552

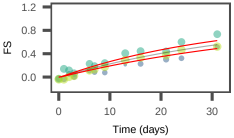

HW Unweighted R2: 0.86 k: 0.0258 dk: 0.00553

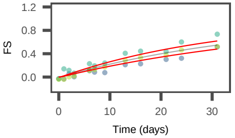

HINT1

AA Weighted R2: 0.97 k: 0.0756 dk: 0.0105

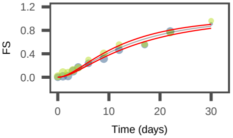

AA Unweighted R2: 0.97 k: 0.0758 dk: 0.0106

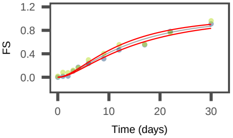

HW Weighted R2: 0.89 k: 0.0512 dk: 0.0136

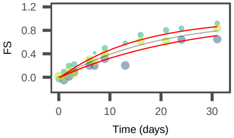

HW Unweighted R2: 0.89 k: 0.0552 dk: 0.015

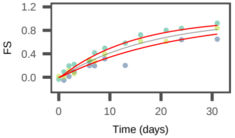

HINT2

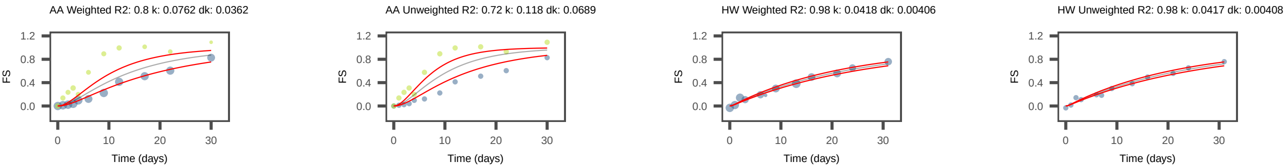

HNRPK

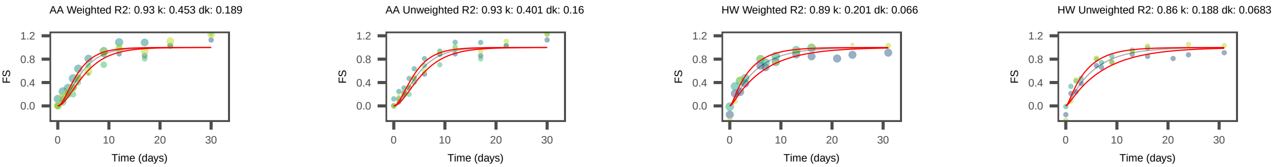

HNRPU

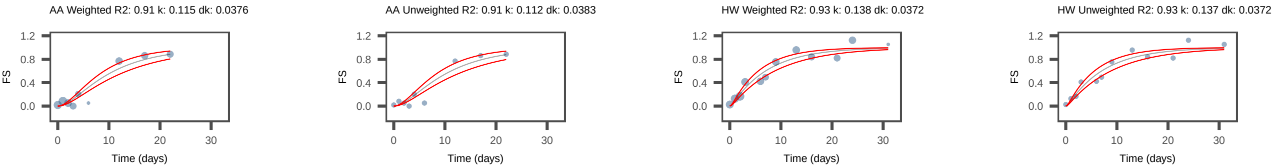

HS90A

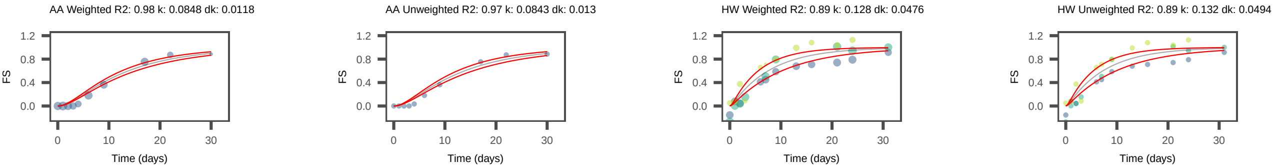

HS90B

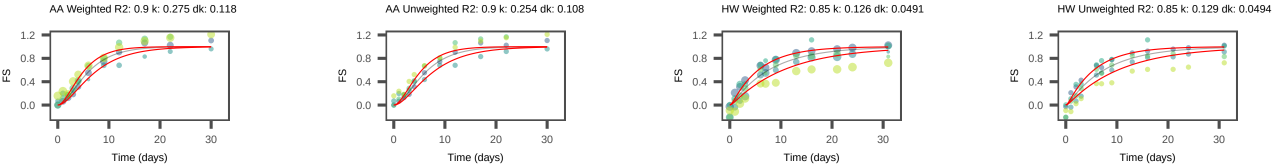

HSDL2

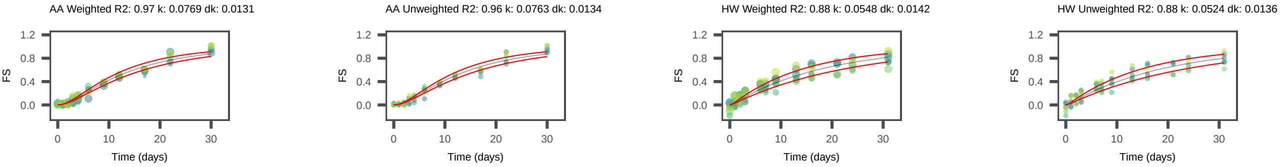

HSP74

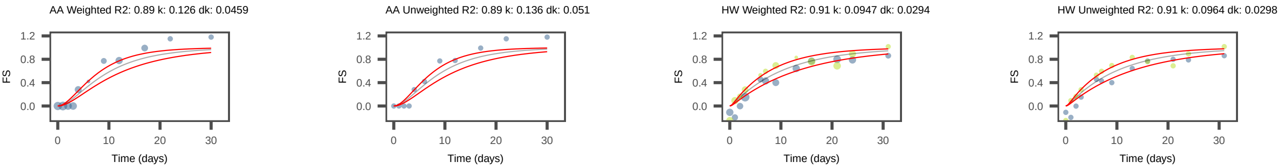

HSP7C

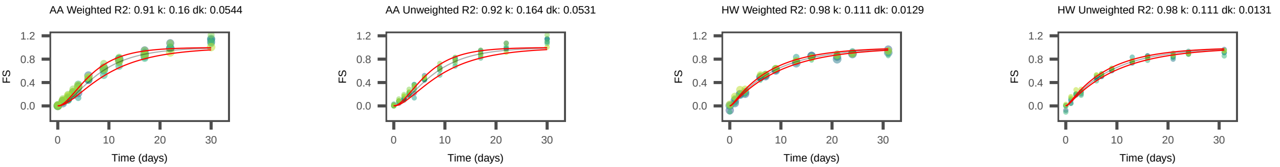

HSPB1

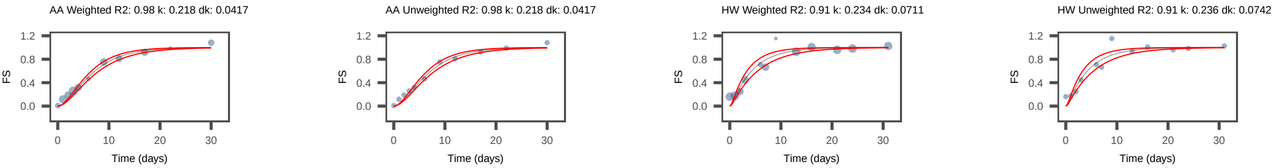

HSPB6

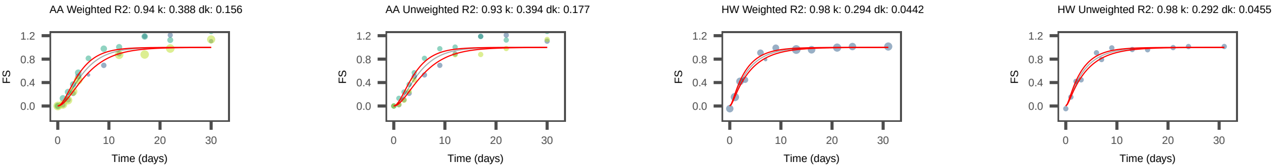

HYES

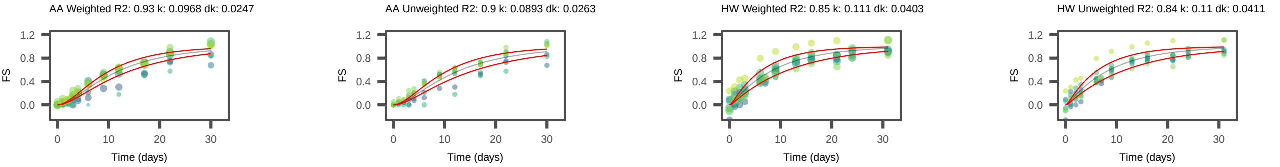

IDH3A

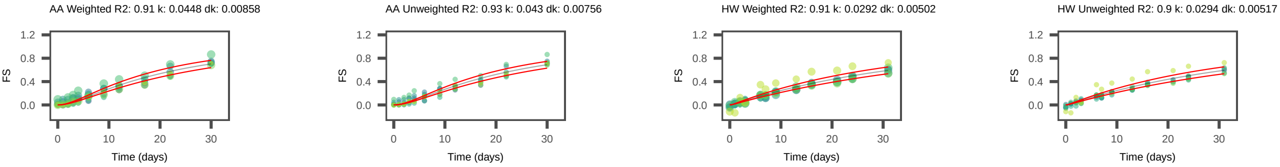

IDHG1

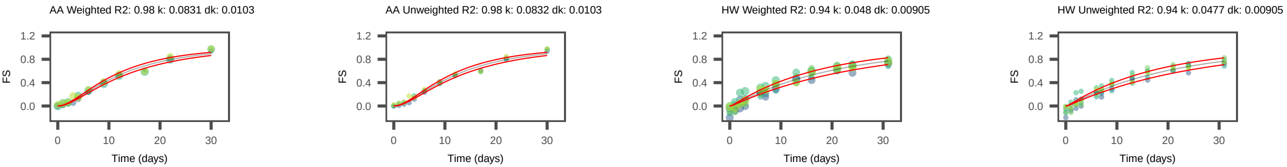

IDHP

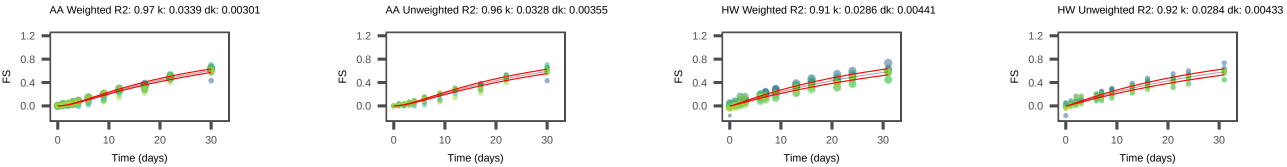

IGHM

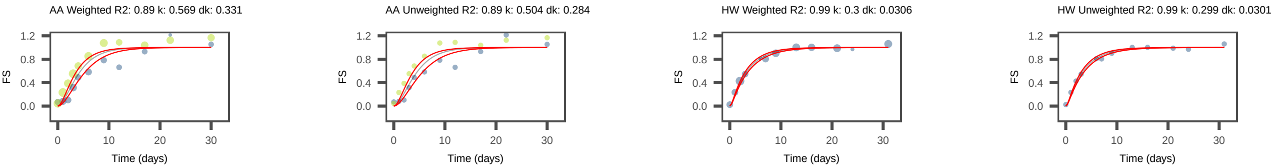

IGKC

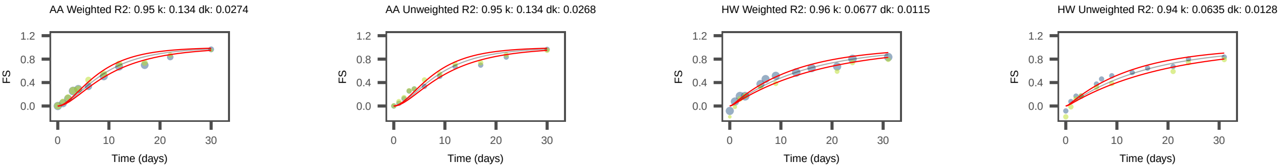

IPYR

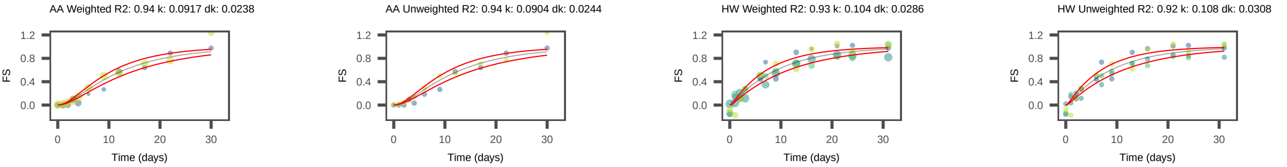

IPYR2

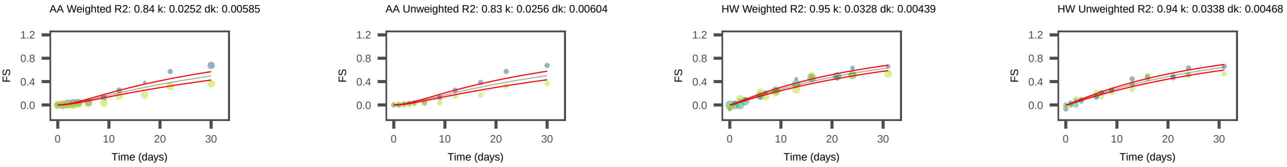

ISC2A

AA Weighted R2: 0.98 k: 0.0105 dk: 0.00055 | AA Unweighted R2: 0.98 k: 0.0104 dk: 0.000534 | HW Weighted R2: 0.94 k: 0.0377 dk: 0.00663 | HW Unweighted R2: 0.93 k: 0.0388 dk: 0.00756

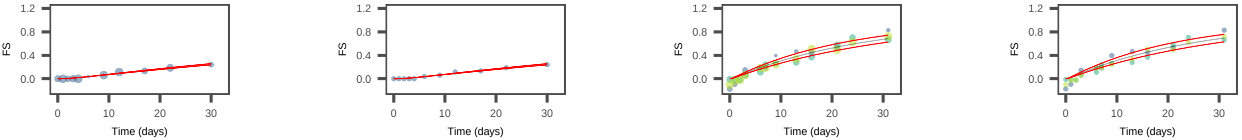

ISOC1

AA Weighted R2: 0.97 k: 0.0742 dk: 0.0116 | AA Unweighted R2: 0.97 k: 0.0744 dk: 0.0114 | HW Weighted R2: 0.94 k: 0.105 dk: 0.028 | HW Unweighted R2: 0.94 k: 0.105 dk: 0.0282

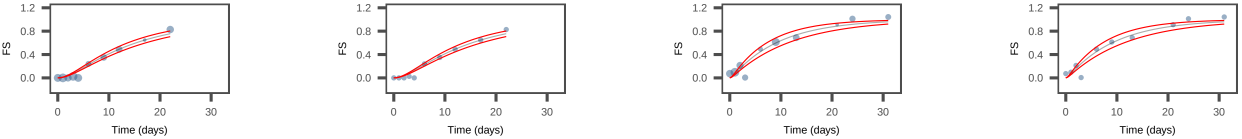

IVD

AA Weighted R2: 0.96 k: 0.0398 dk: 0.00492 | AA Unweighted R2: 0.96 k: 0.0398 dk: 0.0049 | HW Weighted R2: 0.91 k: 0.034 dk: 0.006 | HW Unweighted R2: 0.91 k: 0.0338 dk: 0.00616

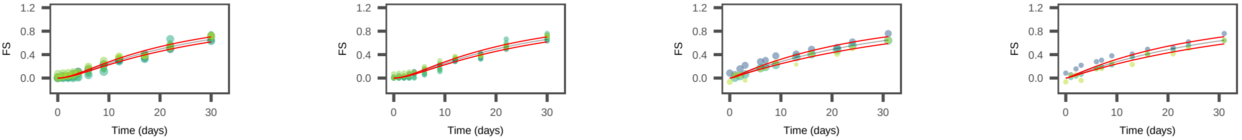

KAD1

AA Weighted R2: 0.94 k: 0.0698 dk: 0.0139 | AA Unweighted R2: 0.92 k: 0.0692 dk: 0.0149 | HW Weighted R2: 0.91 k: 0.0534 dk: 0.0133 | HW Unweighted R2: 0.9 k: 0.0593 dk: 0.0156

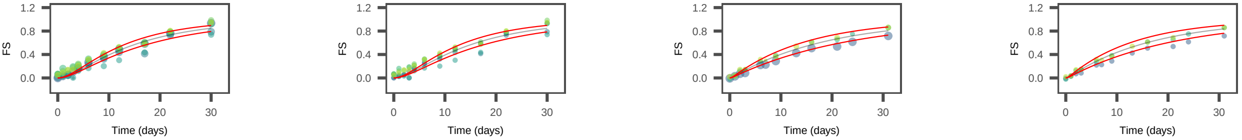

KAD2

AA Weighted R2: 0.97 k: 0.0267 dk: 0.00194 | AA Unweighted R2: 0.97 k: 0.0266 dk: 0.00195 | HW Weighted R2: 0.93 k: 0.0193 dk: 0.0024 | HW Unweighted R2: 0.93 k: 0.0192 dk: 0.00246

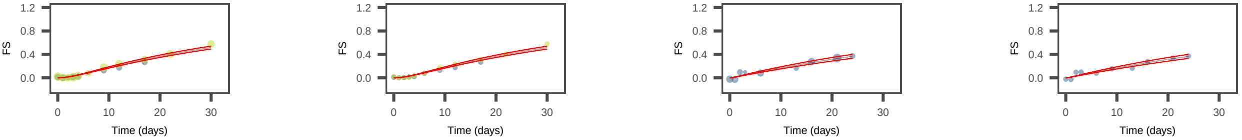

KAP2

AA Weighted R2: 0.94 k: 0.0384 dk: 0.00695 | AA Unweighted R2: 0.92 k: 0.0409 dk: 0.00849 | HW Weighted R2: 0.95 k: 0.069 dk: 0.0134 | HW Unweighted R2: 0.95 k: 0.0697 dk: 0.0139

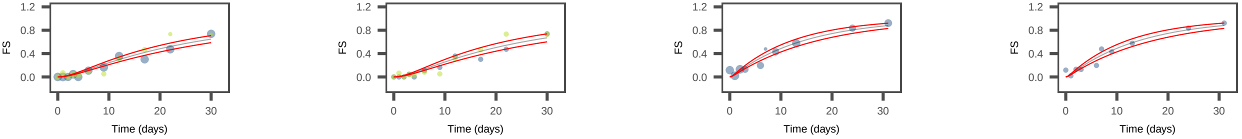

KCRB

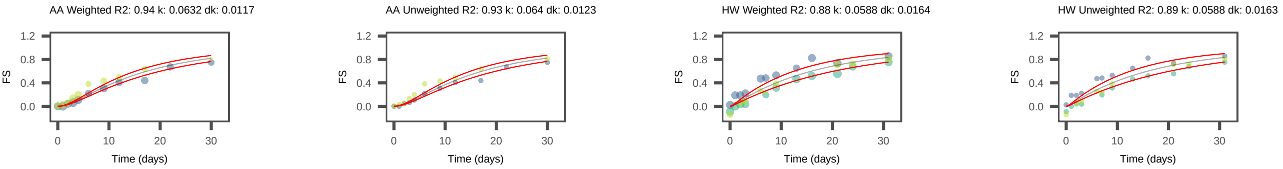

KCRM

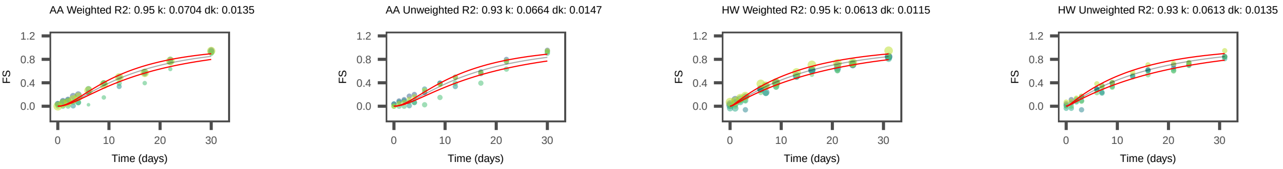

KCRS

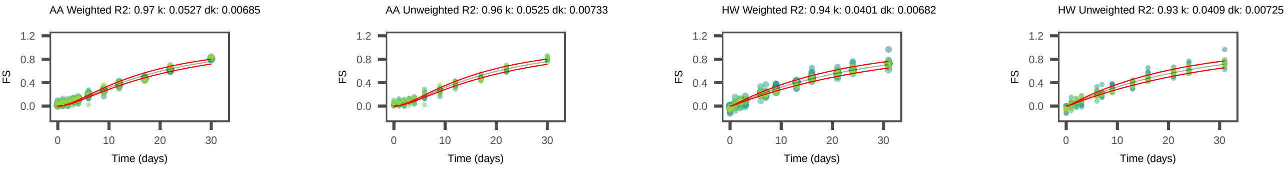

KPYM

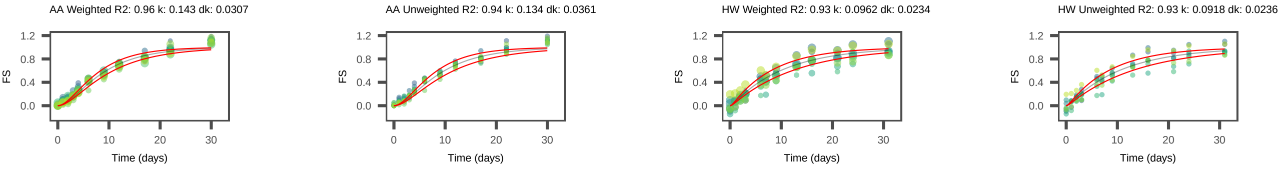

LAMB1

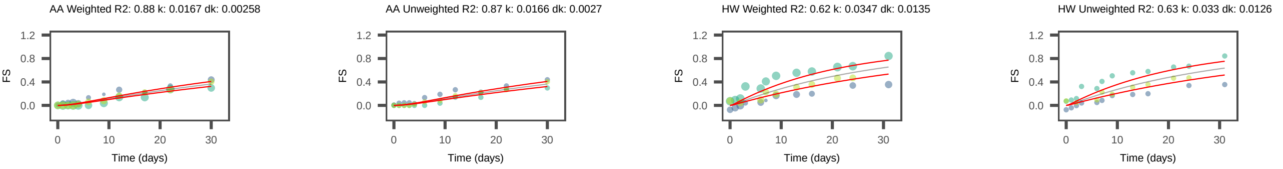

LAMC1

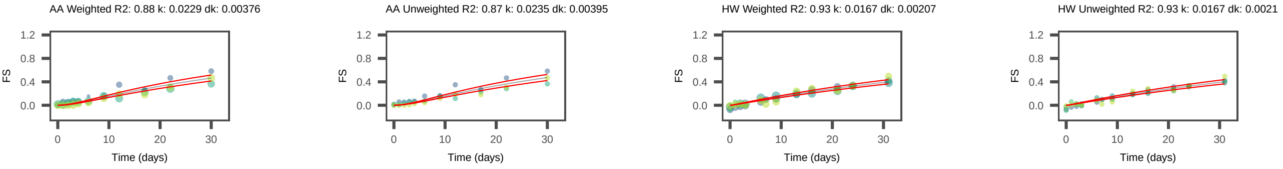

LDB3

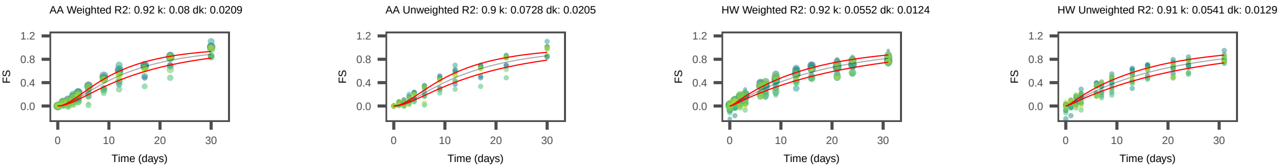

LDHA

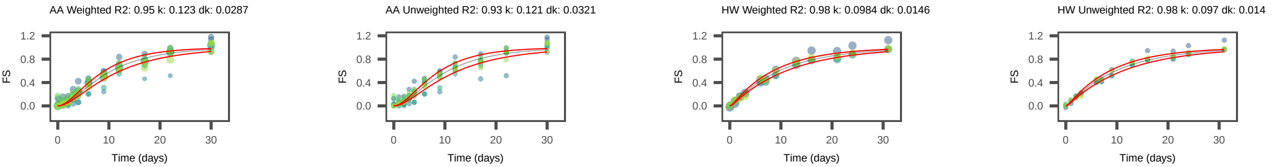

LDHB

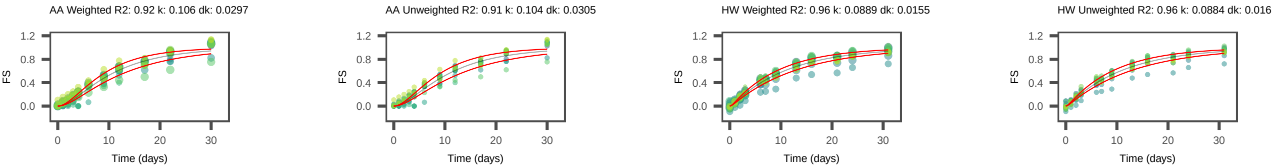

LEG1

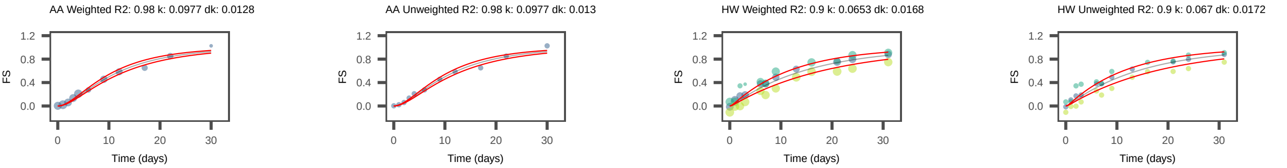

LGUL

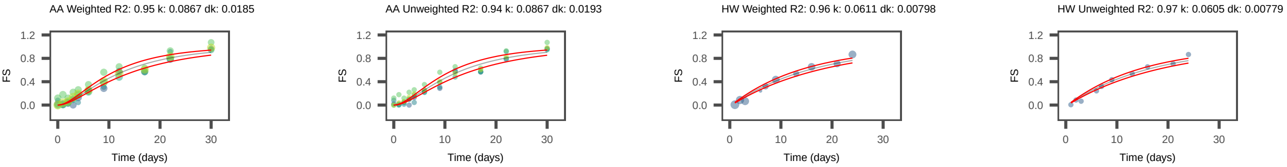

LKHA4

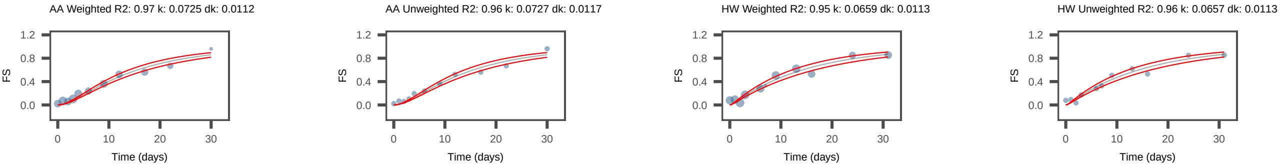

LONM

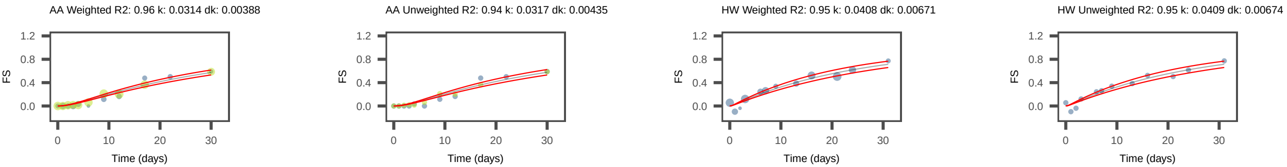

LPPRC

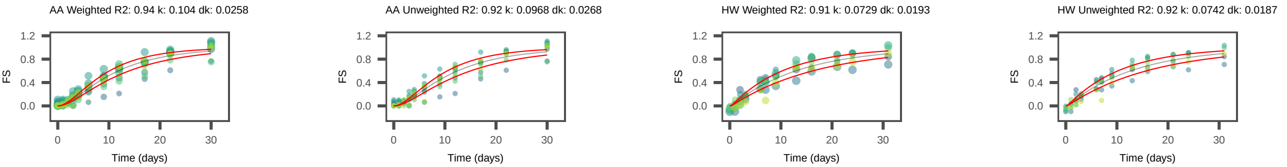

LUM

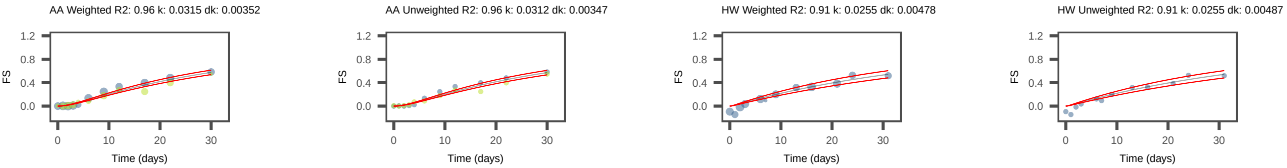

M2OM

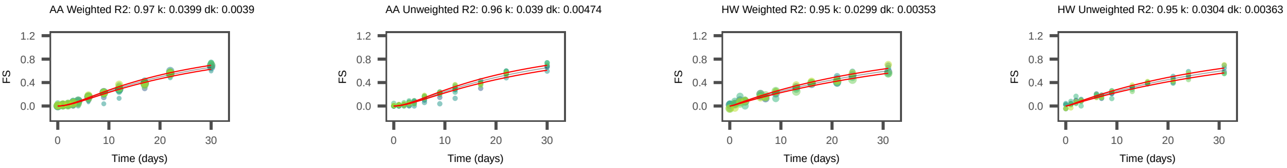

MAAI

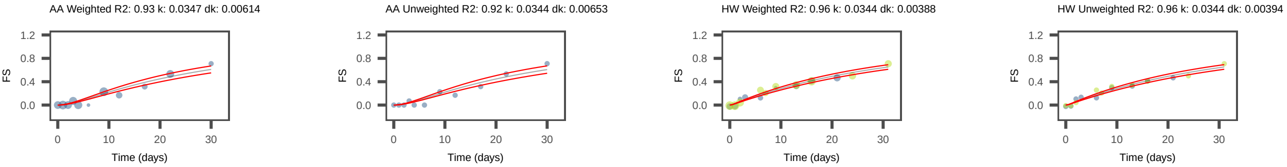

MACD1

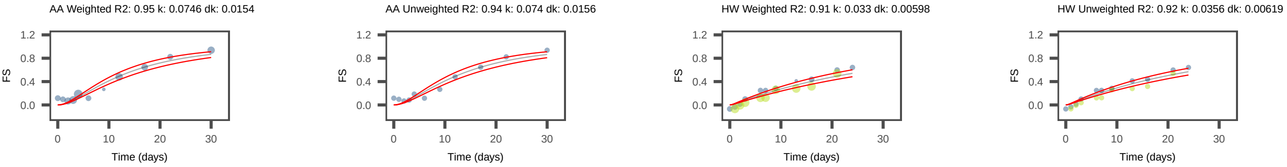

MCAT

AA Weighted R2: 0.96 k: 0.0481 dk: 0.00656

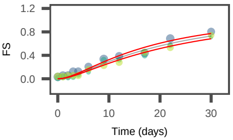

AA Unweighted R2: 0.96 k: 0.0469 dk: 0.00613

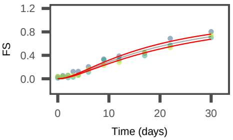

HW Weighted R2: 0.98 k: 0.0298 dk: 0.00278

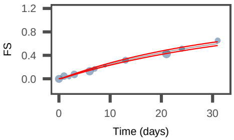

HW Unweighted R2: 0.97 k: 0.03 dk: 0.00304

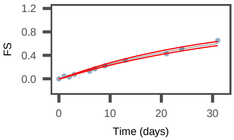

MCCA

AA Weighted R2: 0.82 k: 0.0308 dk: 0.00852

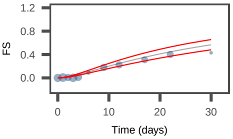

AA Unweighted R2: 0.71 k: 0.0389 dk: 0.0136

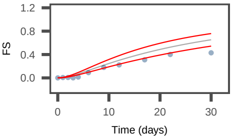

HW Weighted R2: 0.93 k: 0.0303 dk: 0.00464

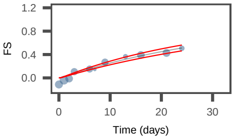

HW Unweighted R2: 0.94 k: 0.0303 dk: 0.00456

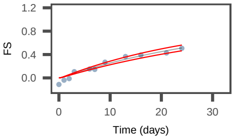

MDHC

AA Weighted R2: 0.97 k: 0.0782 dk: 0.0123

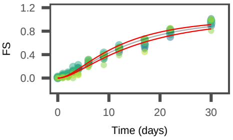

AA Unweighted R2: 0.96 k: 0.076 dk: 0.0129

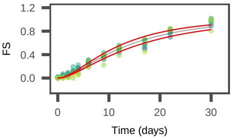

HW Weighted R2: 0.93 k: 0.0546 dk: 0.0105

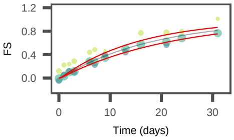

HW Unweighted R2: 0.88 k: 0.0604 dk: 0.0156

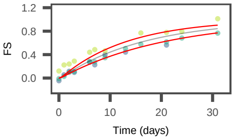

MDHM

AA Weighted R2: 0.97 k: 0.0353 dk: 0.0034

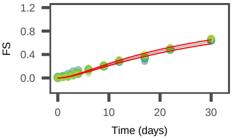

AA Unweighted R2: 0.94 k: 0.0334 dk: 0.00467

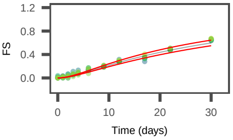

HW Weighted R2: 0.97 k: 0.0249 dk: 0.0021

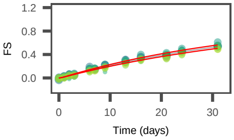

HW Unweighted R2: 0.97 k: 0.0247 dk: 0.00222

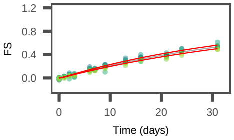

MGDP1

AA Weighted R2: 0.98 k: 0.0707 dk: 0.0089

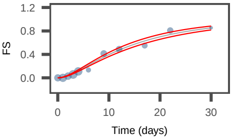

AA Unweighted R2: 0.98 k: 0.0705 dk: 0.00924

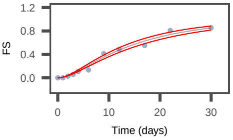

HW Weighted R2: 0.86 k: 0.0913 dk: 0.0358

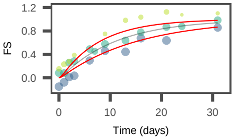

HW Unweighted R2: 0.84 k: 0.102 dk: 0.0416

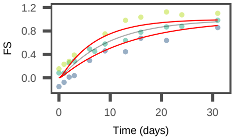

MIC13

AA Weighted R2: 0.96 k: 0.0434 dk: 0.00496

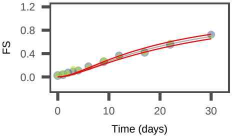

AA Unweighted R2: 0.96 k: 0.044 dk: 0.00507

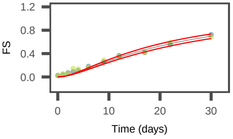

HW Weighted R2: 0.97 k: 0.0283 dk: 0.0022

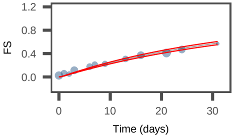

HW Unweighted R2: 0.97 k: 0.0283 dk: 0.00219

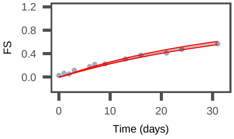

MIC19

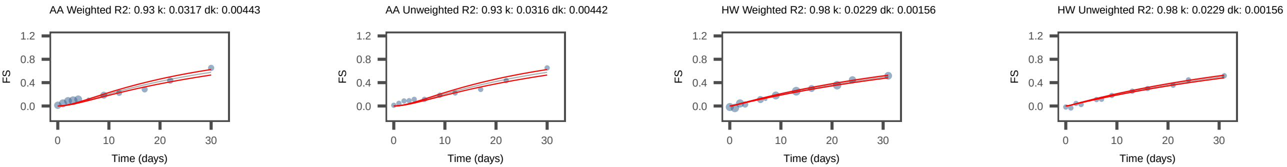

MIC26

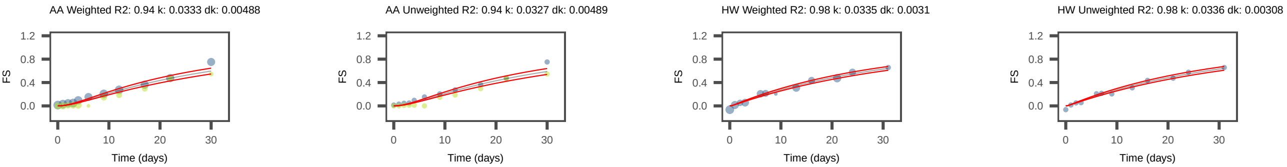

MIC27

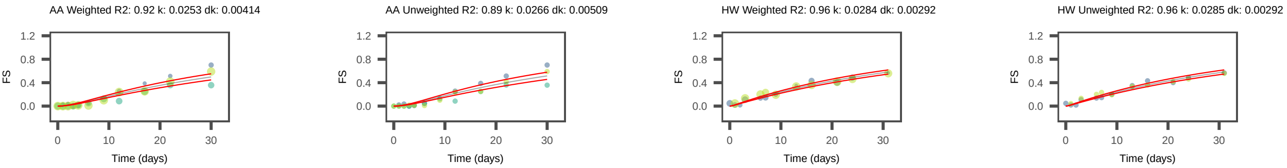

MIC60

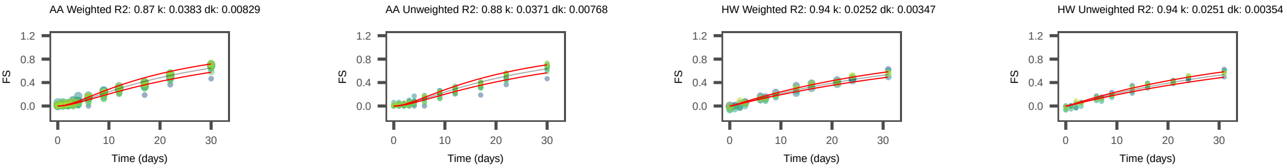

MLRV

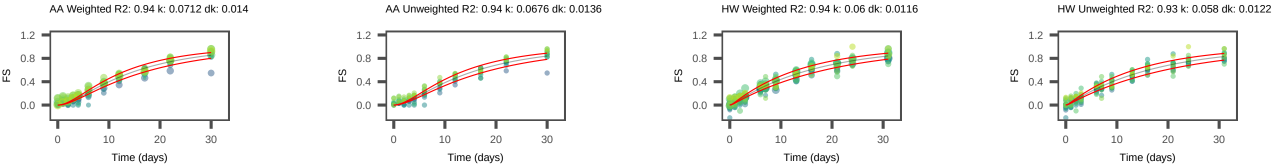

MMSA

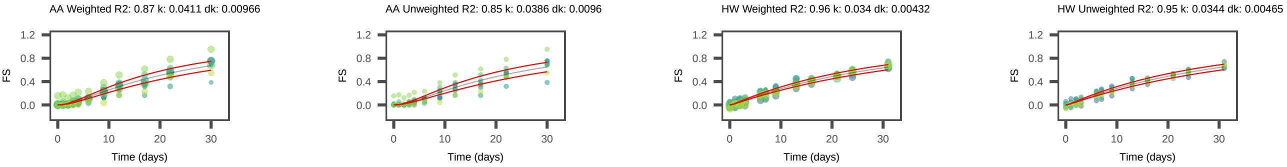

MOES

AA Weighted R2: 0.86 k: 0.0862 dk: 0.0308

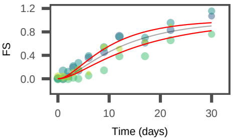

AA Unweighted R2: 0.86 k: 0.0859 dk: 0.0308

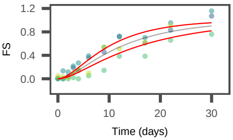

HW Weighted R2: 0.86 k: 0.106 dk: 0.0384

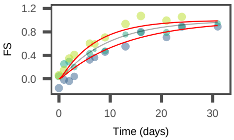

HW Unweighted R2: 0.86 k: 0.105 dk: 0.0373

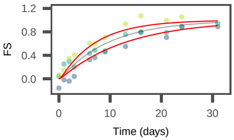

MOT1

AA Weighted R2: 0.99 k: 0.134 dk: 0.0144

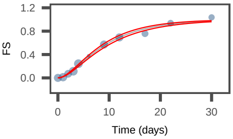

AA Unweighted R2: 0.99 k: 0.134 dk: 0.0147

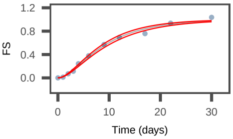

HW Weighted R2: 0.96 k: 0.0827 dk: 0.014

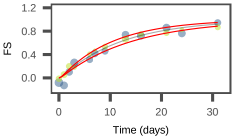

HW Unweighted R2: 0.97 k: 0.0827 dk: 0.0139

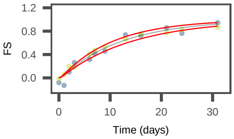

MPC1

AA Weighted R2: 0.98 k: 0.0838 dk: 0.0114

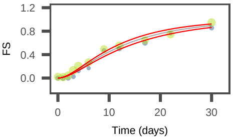

AA Unweighted R2: 0.98 k: 0.0805 dk: 0.0111

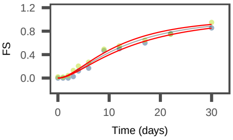

HW Weighted R2: 0.94 k: 0.0483 dk: 0.00932

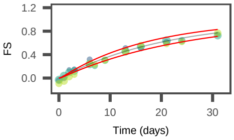

HW Unweighted R2: 0.89 k: 0.0516 dk: 0.0131

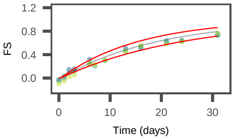

MPCP

AA Weighted R2: 0.97 k: 0.0448 dk: 0.00463

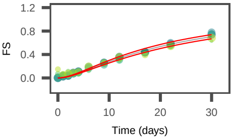

AA Unweighted R2: 0.97 k: 0.0442 dk: 0.00509

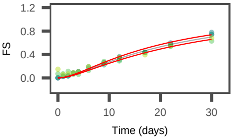

HW Weighted R2: 0.85 k: 0.0473 dk: 0.0114

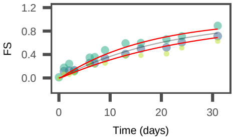

HW Unweighted R2: 0.85 k: 0.0443 dk: 0.0109

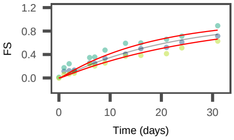

MPI

AA Weighted R2: 0.94 k: 0.0876 dk: 0.0214

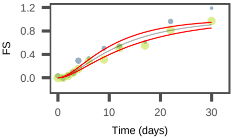

AA Unweighted R2: 0.93 k: 0.092 dk: 0.025

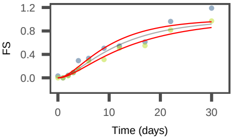

HW Weighted R2: 0.94 k: 0.0708 dk: 0.0149

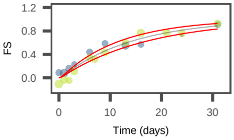

HW Unweighted R2: 0.94 k: 0.0706 dk: 0.015

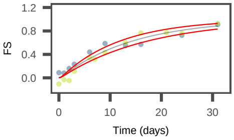

MSRA

AA Weighted R2: 0.81 k: 0.0434 dk: 0.0151

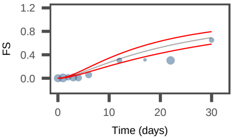

AA Unweighted R2: 0.81 k: 0.0444 dk: 0.0155

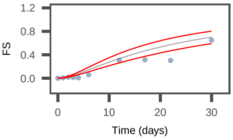

HW Weighted R2: 0.96 k: 0.0615 dk: 0.00948

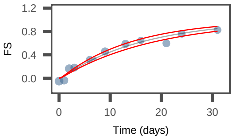

HW Unweighted R2: 0.96 k: 0.0615 dk: 0.0094

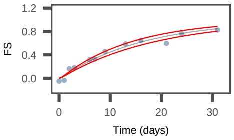

MSRB2

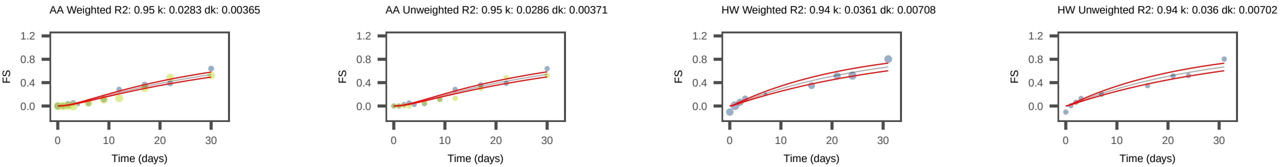

MTX2

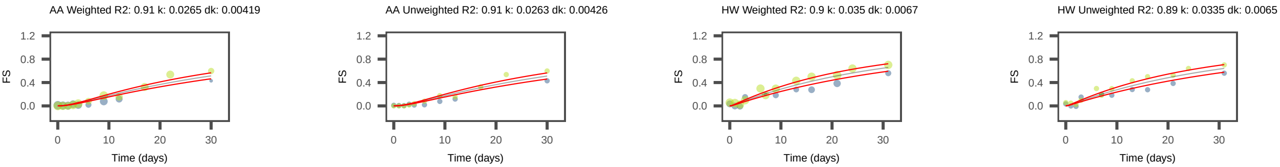

MUTA

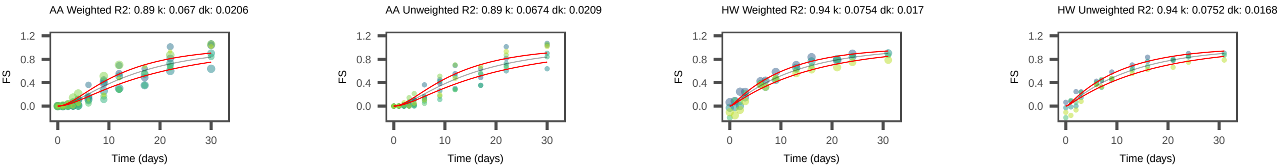

MYG

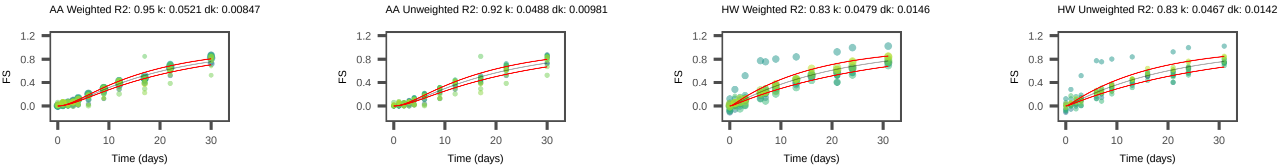

MYH11

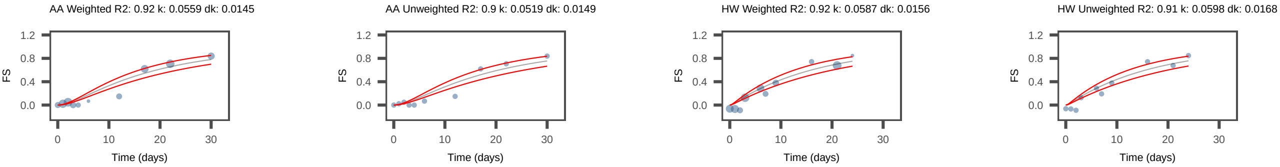

MYH6

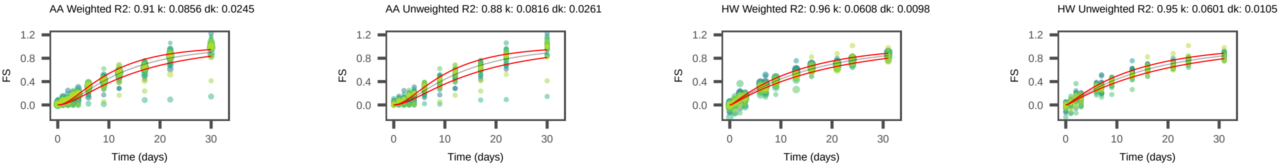

MYH7

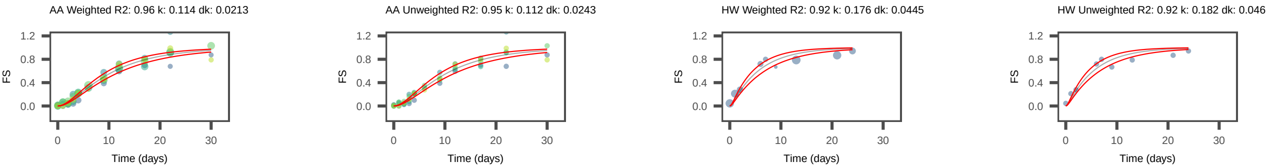

MYH9

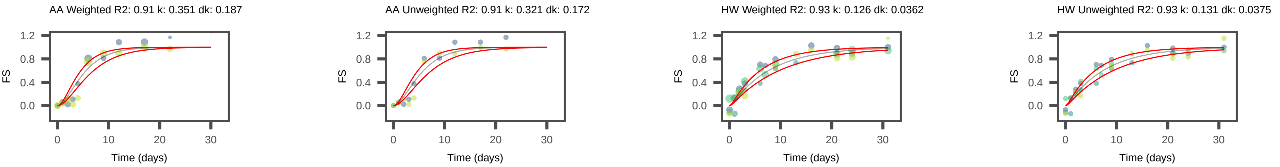

MYL3

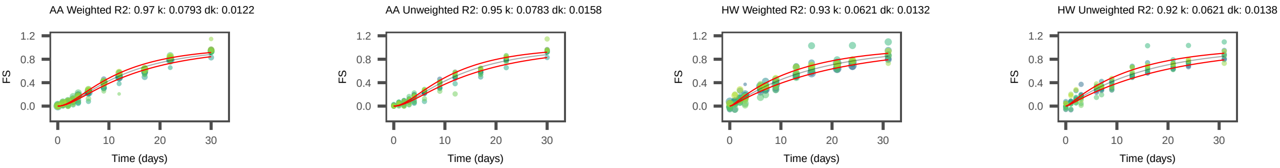

MYL6

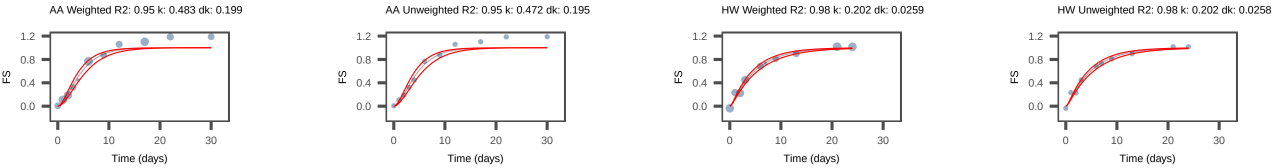

MYOM1

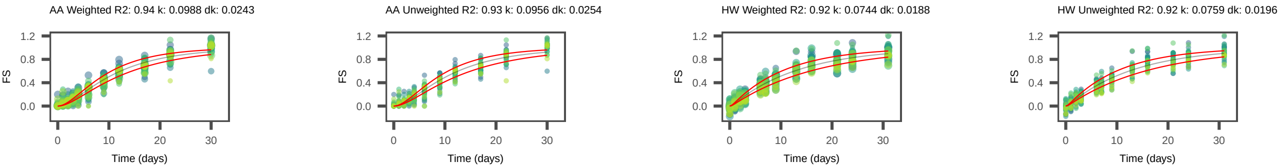

MYOTI

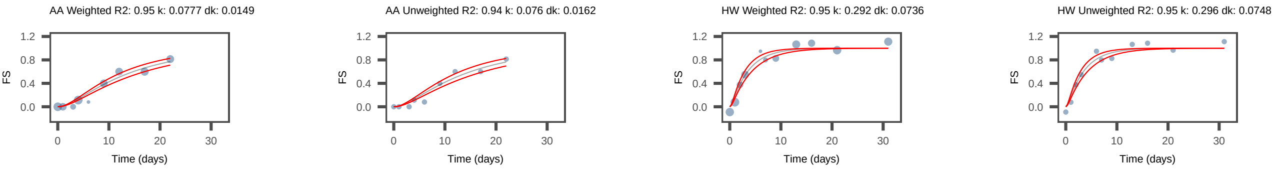

MYOZ2

AA Weighted R2: 0.97 k: 0.0572 dk: 0.00803

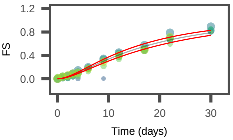

AA Unweighted R2: 0.96 k: 0.0558 dk: 0.00854

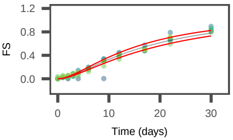

HW Weighted R2: 0.98 k: 0.0392 dk: 0.0037

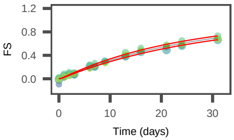

HW Unweighted R2: 0.98 k: 0.0395 dk: 0.00394

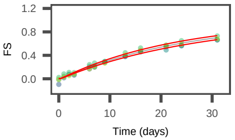

MYPC3

AA Weighted R2: 0.91 k: 0.0826 dk: 0.0218

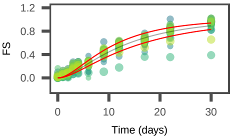

AA Unweighted R2: 0.91 k: 0.0819 dk: 0.0211

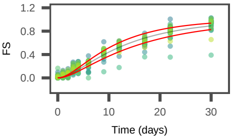

HW Weighted R2: 0.93 k: 0.0649 dk: 0.0145

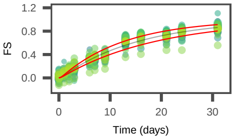

HW Unweighted R2: 0.92 k: 0.0651 dk: 0.0152

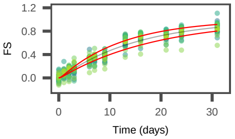

NAC1

AA Weighted R2: 0.93 k: 0.16 dk: 0.0501

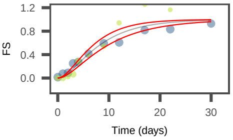

AA Unweighted R2: 0.91 k: 0.173 dk: 0.063

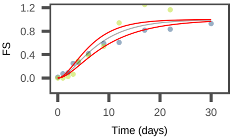

HW Weighted R2: 0.97 k: 0.157 dk: 0.0246

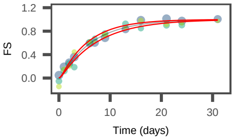

HW Unweighted R2: 0.97 k: 0.157 dk: 0.025

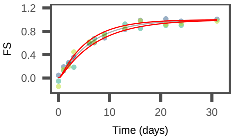

NACAM

AA Weighted R2: 0.82 k: 0.0826 dk: 0.0387

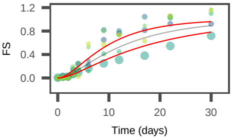

AA Unweighted R2: 0.82 k: 0.105 dk: 0.05

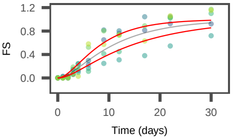

HW Weighted R2: 0.85 k: 0.177 dk: 0.0692

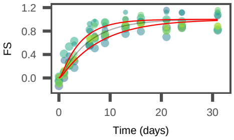

HW Unweighted R2: 0.86 k: 0.187 dk: 0.0727

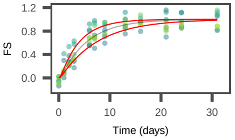

NAMPT

AA Weighted R2: 0.94 k: 0.272 dk: 0.0922

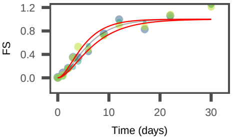

AA Unweighted R2: 0.94 k: 0.266 dk: 0.0901

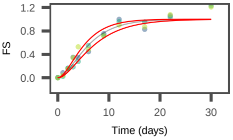

HW Weighted R2: 0.88 k: 0.163 dk: 0.0589

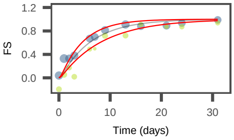

HW Unweighted R2: 0.86 k: 0.171 dk: 0.0654

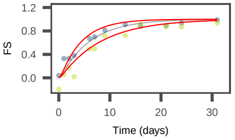

NAR3

AA Weighted R2: 0.94 k: 0.056 dk: 0.0125

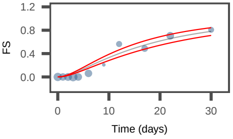

AA Unweighted R2: 0.93 k: 0.0562 dk: 0.0129

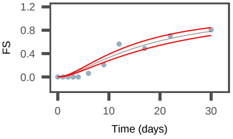

HW Weighted R2: 0.94 k: 0.107 dk: 0.0256

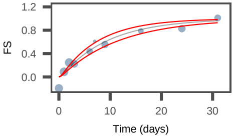

HW Unweighted R2: 0.94 k: 0.107 dk: 0.0256

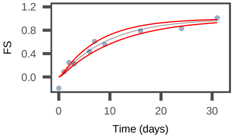

NDKB

AA Weighted R2: 0.99 k: 0.1 dk: 0.0109
AA Unweighted R2: 0.99 k: 0.1 dk: 0.0109
HW Weighted R2: 0.99 k: 0.0522 dk: 0.00468
HW Unweighted R2: 0.99 k: 0.0522 dk: 0.0047

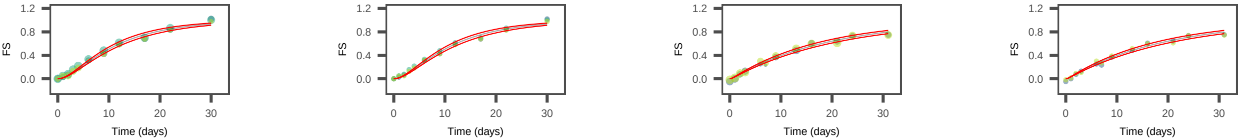

NDRG2

AA Weighted R2: 0.95 k: 0.306 dk: 0.0969
AA Unweighted R2: 0.95 k: 0.304 dk: 0.0989
HW Weighted R2: 0.88 k: 0.143 dk: 0.0476
HW Unweighted R2: 0.86 k: 0.142 dk: 0.0506

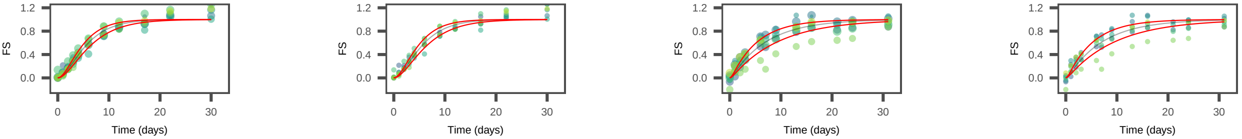

NDUA2

AA Weighted R2: 0.96 k: 0.046 dk: 0.0057
AA Unweighted R2: 0.96 k: 0.0459 dk: 0.0057
HW Weighted R2: 0.91 k: 0.0199 dk: 0.00357
HW Unweighted R2: 0.91 k: 0.0199 dk: 0.00355

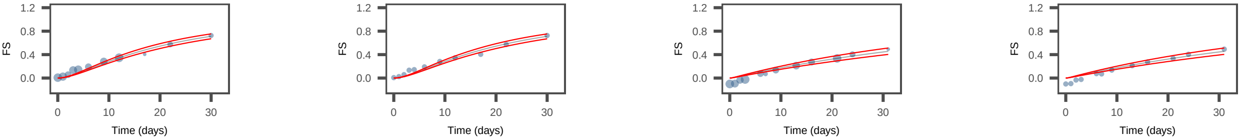

NDUA3

AA Weighted R2: 0.97 k: 0.0483 dk: 0.0054
AA Unweighted R2: 0.97 k: 0.0483 dk: 0.00541
HW Weighted R2: 0.93 k: 0.0159 dk: 0.00202
HW Unweighted R2: 0.93 k: 0.0159 dk: 0.00202

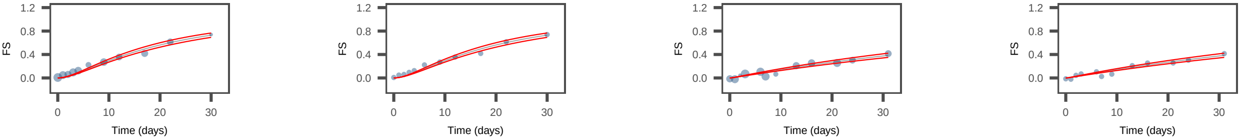

NDUA6

AA Weighted R2: 0.95 k: 0.0596 dk: 0.0106
AA Unweighted R2: 0.95 k: 0.0576 dk: 0.0105
HW Weighted R2: 0.82 k: 0.0486 dk: 0.0161
HW Unweighted R2: 0.83 k: 0.0455 dk: 0.0149

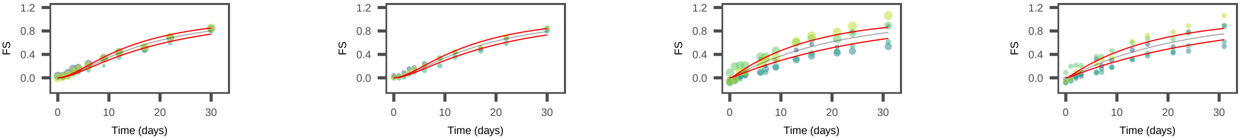

NDUA7

AA Weighted R2: 0.89 k: 0.0895 dk: 0.0266
AA Unweighted R2: 0.89 k: 0.0886 dk: 0.0262
HW Weighted R2: 0.89 k: 0.0359 dk: 0.00812
HW Unweighted R2: 0.9 k: 0.0358 dk: 0.00769

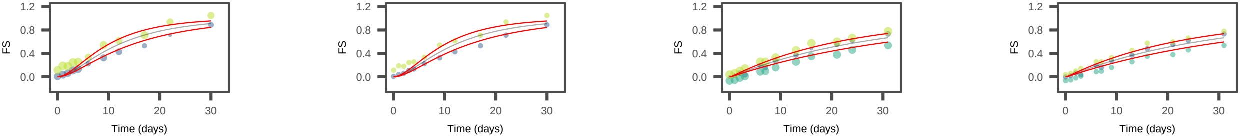

NDUA8

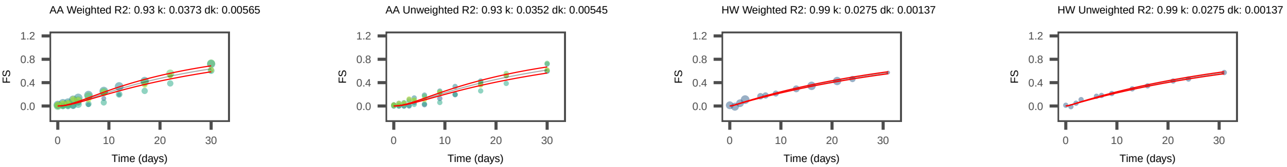

NDUA9

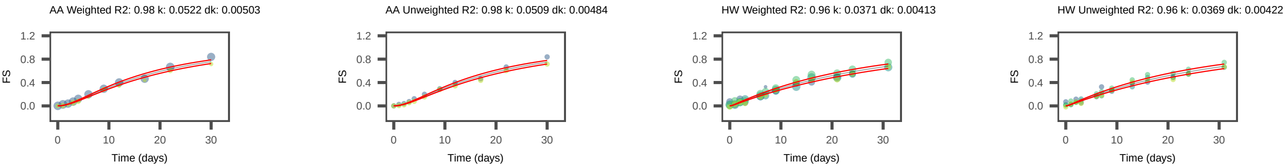

NDUAA

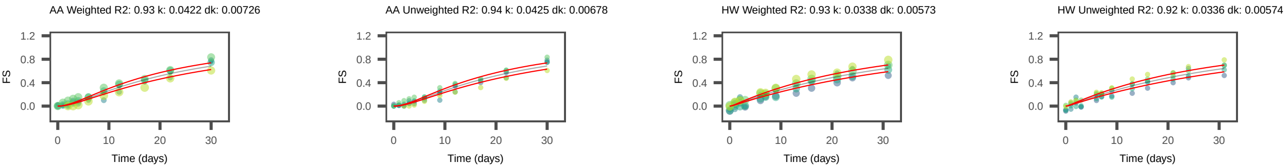

NDUAB

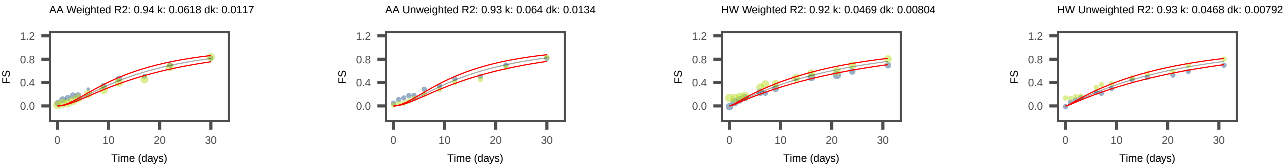

NDUAC

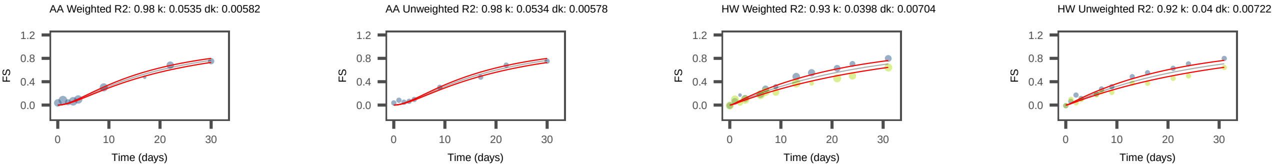

NDUAD

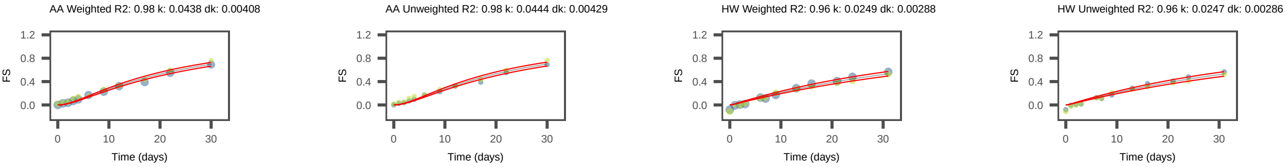

NDUB1

AA Weighted R2: 0.97 k: 0.0347 dk: 0.00321 | AA Unweighted R2: 0.97 k: 0.0333 dk: 0.00334 | HW Weighted R2: 0.99 k: 0.0273 dk: 0.00154 | HW Unweighted R2: 0.99 k: 0.0273 dk: 0.00153

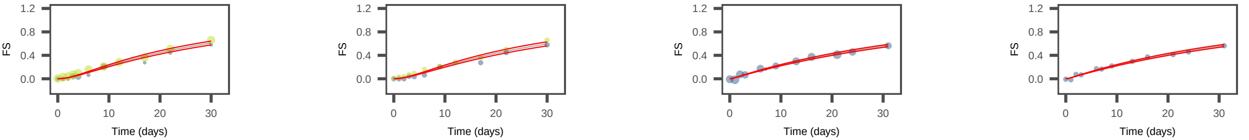

NDUB3

AA Weighted R2: 0.99 k: 0.0377 dk: 0.00232 | AA Unweighted R2: 0.99 k: 0.0375 dk: 0.00232 | HW Weighted R2: 0.98 k: 0.0283 dk: 0.00221 | HW Unweighted R2: 0.98 k: 0.0281 dk: 0.0023

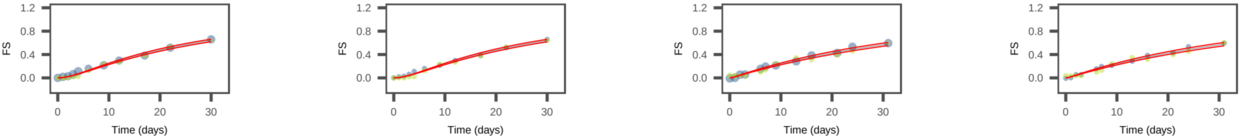

NDUB4

AA Weighted R2: 0.82 k: 0.0489 dk: 0.0138 | AA Unweighted R2: 0.82 k: 0.0462 dk: 0.0134 | HW Weighted R2: 0.92 k: 0.0301 dk: 0.00419 | HW Unweighted R2: 0.92 k: 0.03 dk: 0.00419

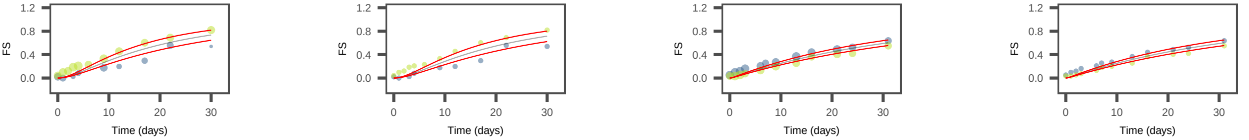

NDUB5

AA Weighted R2: 0.97 k: 0.0387 dk: 0.00415 | AA Unweighted R2: 0.97 k: 0.0379 dk: 0.00421 | HW Weighted R2: 0.92 k: 0.0254 dk: 0.00366 | HW Unweighted R2: 0.92 k: 0.0255 dk: 0.00365

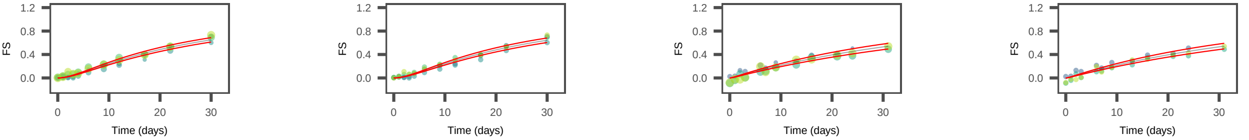

NDUB7

AA Weighted R2: 0.95 k: 0.0356 dk: 0.00536 | AA Unweighted R2: 0.94 k: 0.0359 dk: 0.00579 | HW Weighted R2: 0.95 k: 0.0323 dk: 0.00411 | HW Unweighted R2: 0.94 k: 0.0322 dk: 0.0042

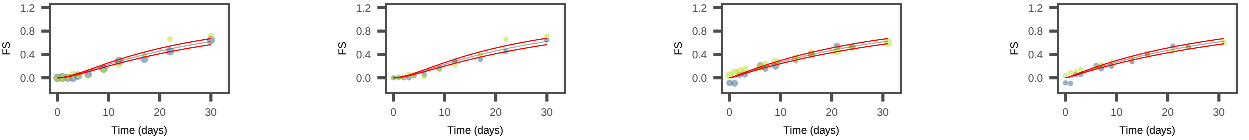

NDUB9

AA Weighted R2: 0.99 k: 0.043 dk: 0.0033 | AA Unweighted R2: 0.99 k: 0.0428 dk: 0.00332 | HW Weighted R2: 0.98 k: 0.0283 dk: 0.00198 | HW Unweighted R2: 0.98 k: 0.0283 dk: 0.00198

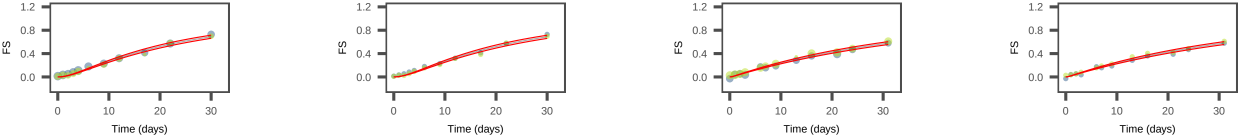

NDUBA

AA Weighted R2: 0.95 k: 0.0402 dk: 0.00552

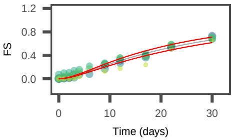

AA Unweighted R2: 0.94 k: 0.0395 dk: 0.00595

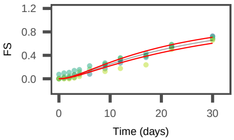

HW Weighted R2: 0.76 k: 0.034 dk: 0.0104

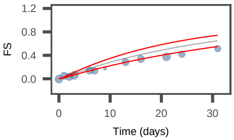

HW Unweighted R2: 0.74 k: 0.0353 dk: 0.0111

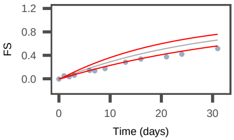

NDUBB

AA Weighted R2: 0.95 k: 0.0358 dk: 0.00431

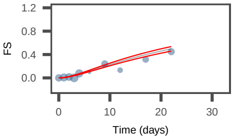

AA Unweighted R2: 0.94 k: 0.034 dk: 0.00456

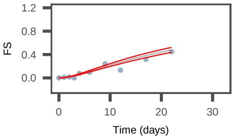

HW Weighted R2: 0.95 k: 0.0328 dk: 0.00378

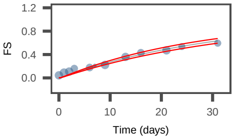

HW Unweighted R2: 0.95 k: 0.0328 dk: 0.00376

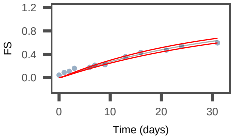

NDUC2

AA Weighted R2: 0.86 k: 0.0494 dk: 0.0128

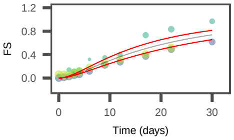

AA Unweighted R2: 0.85 k: 0.0512 dk: 0.0139

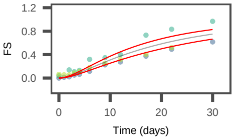

HW Weighted R2: 0.95 k: 0.0311 dk: 0.00415

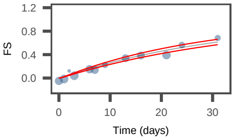

HW Unweighted R2: 0.95 k: 0.0312 dk: 0.00418

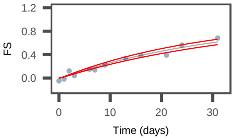

NDUF4

AA Weighted R2: 0.95 k: 0.0332 dk: 0.00473

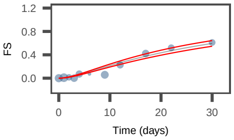

AA Unweighted R2: 0.95 k: 0.0333 dk: 0.0047

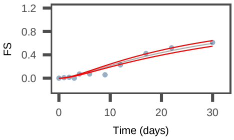

HW Weighted R2: 0.93 k: 0.0316 dk: 0.0055

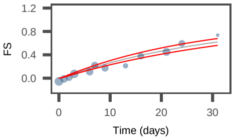

HW Unweighted R2: 0.93 k: 0.0317 dk: 0.00563

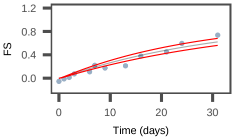

NDUS1

AA Weighted R2: 0.96 k: 0.0518 dk: 0.00778

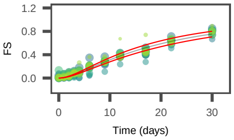

AA Unweighted R2: 0.93 k: 0.052 dk: 0.00953

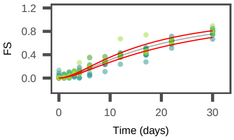

HW Weighted R2: 0.95 k: 0.0358 dk: 0.00516

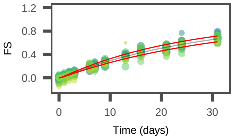

HW Unweighted R2: 0.94 k: 0.0358 dk: 0.00561

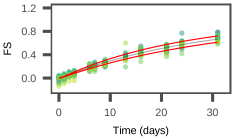

NDUS2

AA Weighted R2: 0.95 k: 0.0564 dk: 0.00869

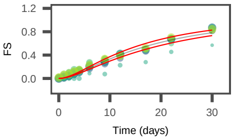

AA Unweighted R2: 0.93 k: 0.0531 dk: 0.0101

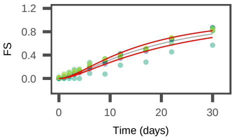

HW Weighted R2: 0.98 k: 0.0369 dk: 0.00366

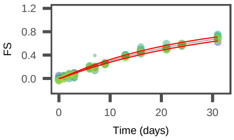

HW Unweighted R2: 0.97 k: 0.0371 dk: 0.00401

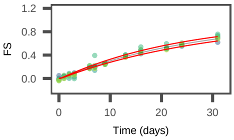

NDUS3

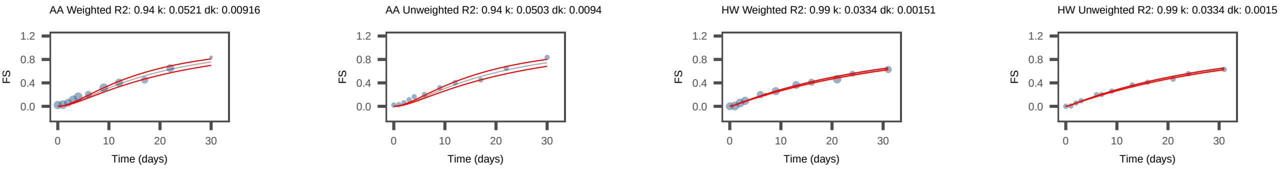

NDUS4

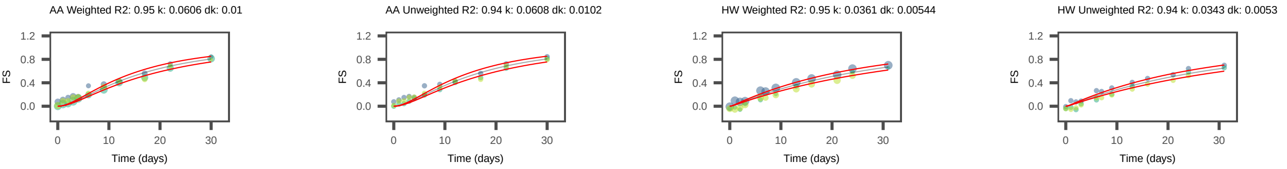

NDUS6

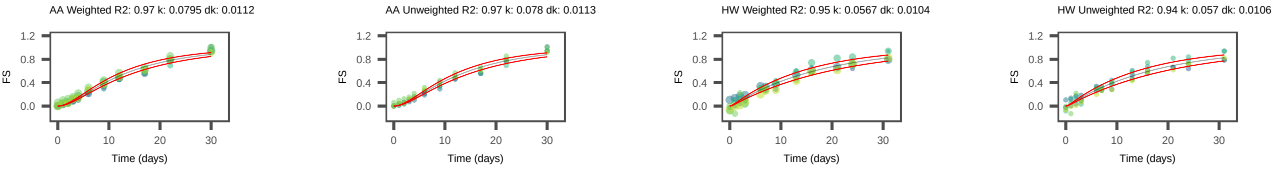

NDUS7

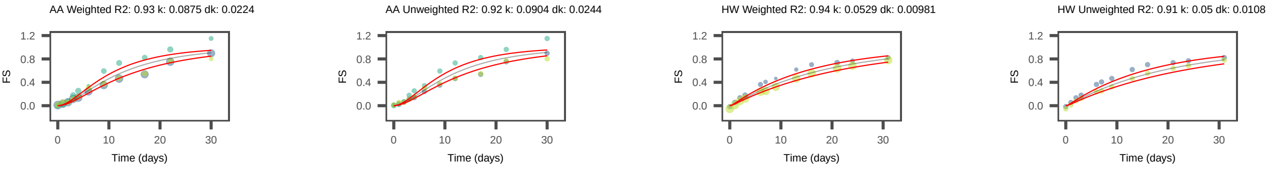

NDUS8

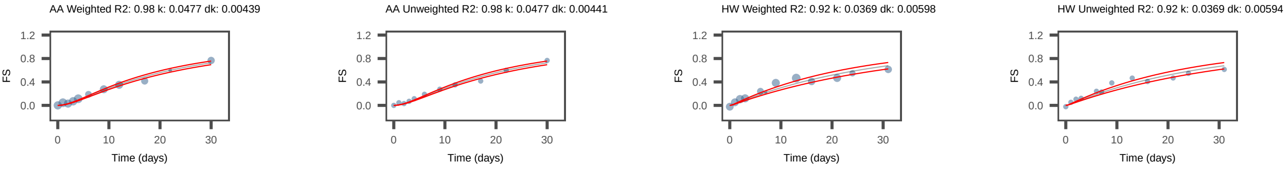

NDUV1

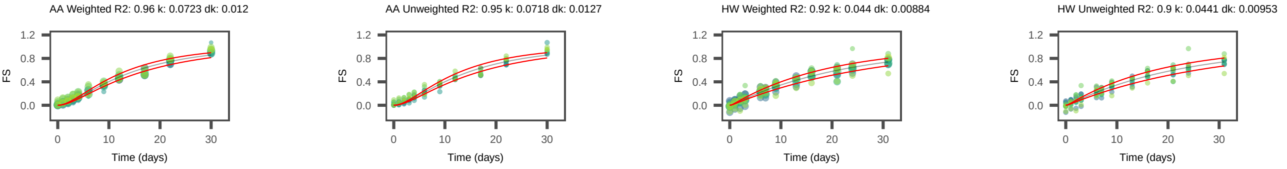

NDUV2

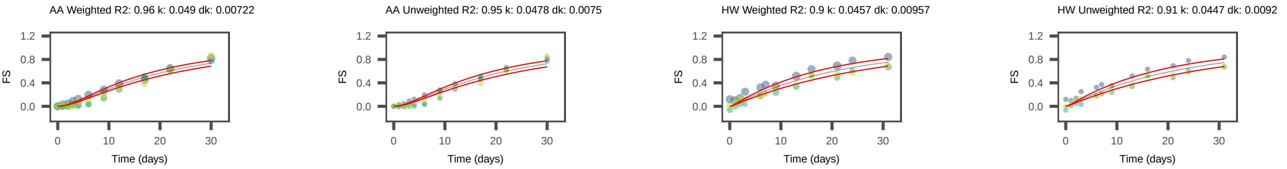

NDUV3

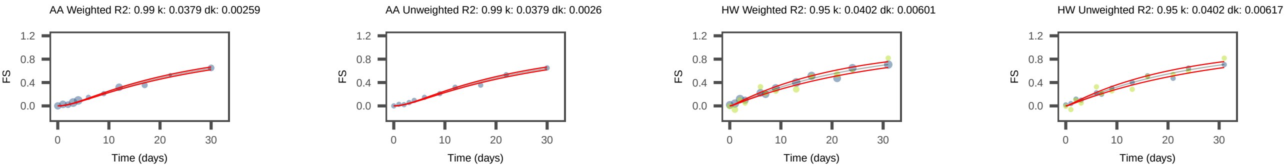

NEBL

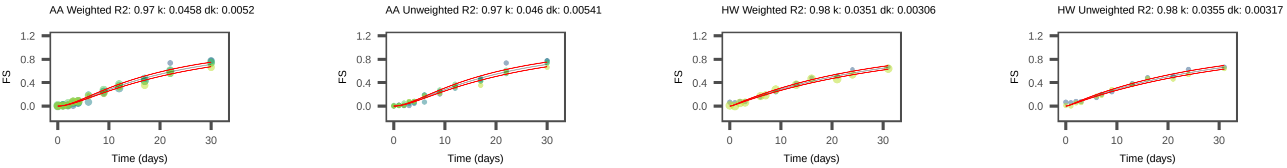

NID1

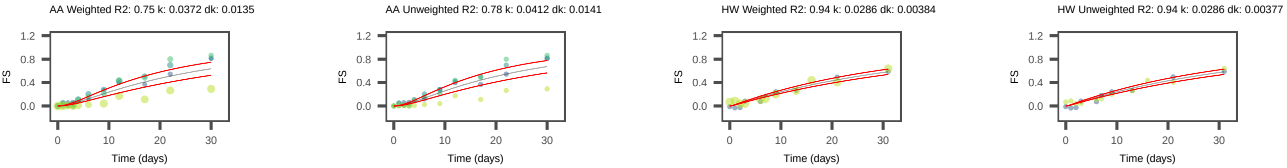

NIPS2

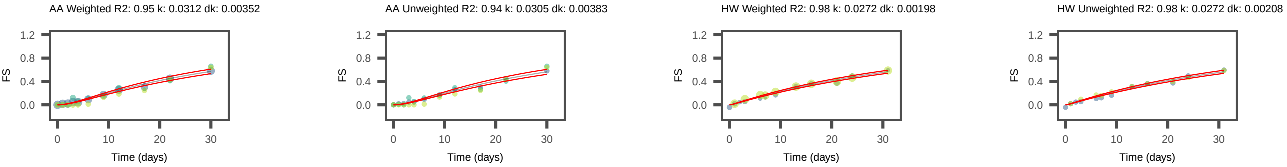

NIT2

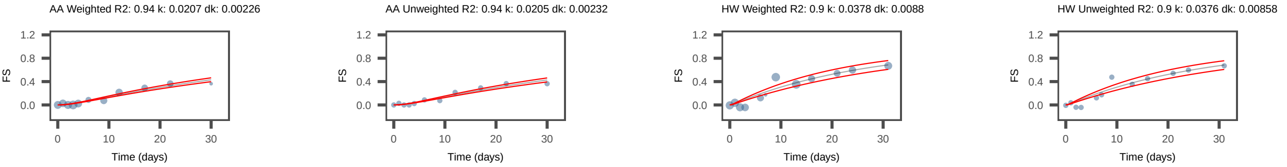

NNTM

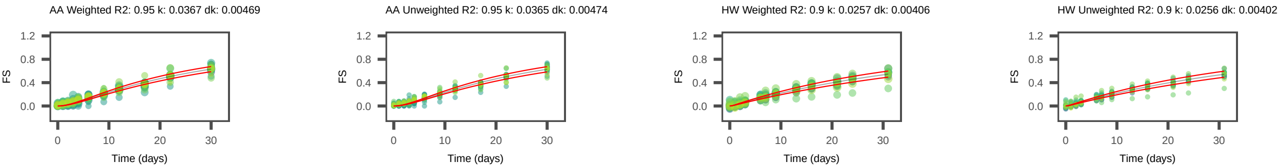

NTF2

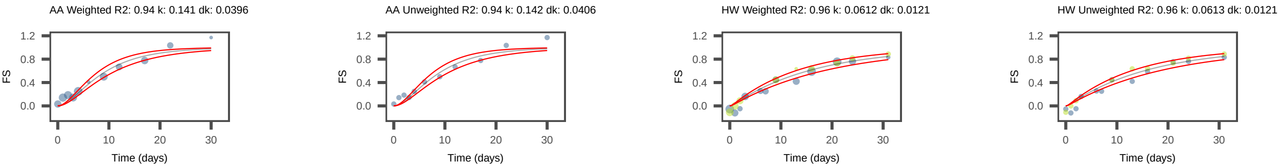

NU1M

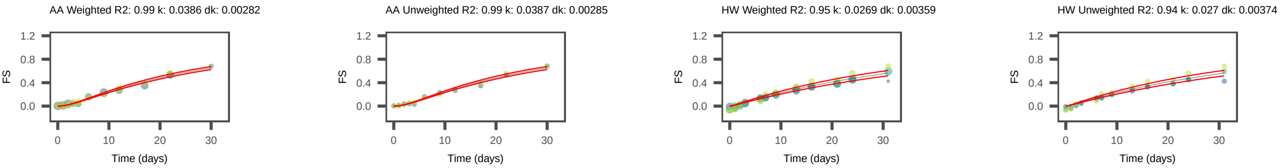

NU2M

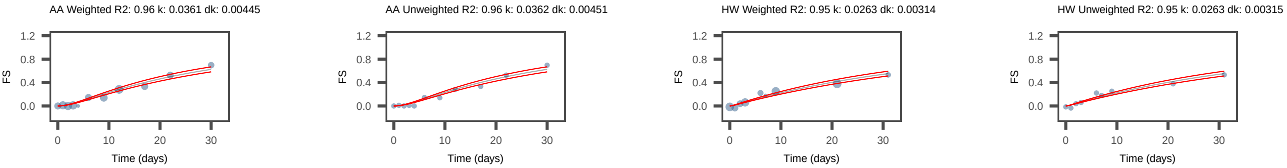

NU4M

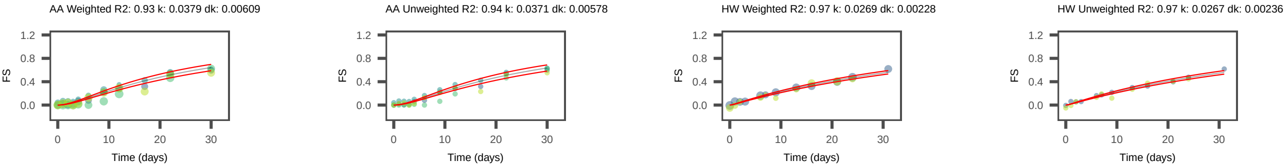

NU5M

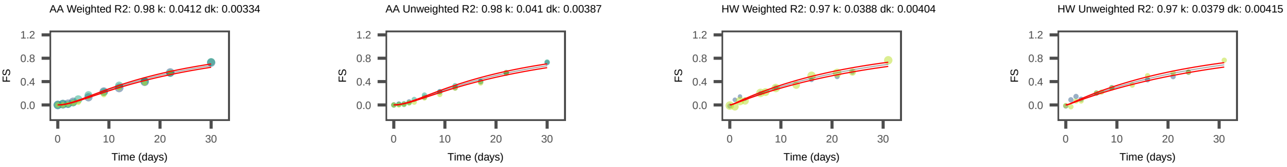

NUDT8

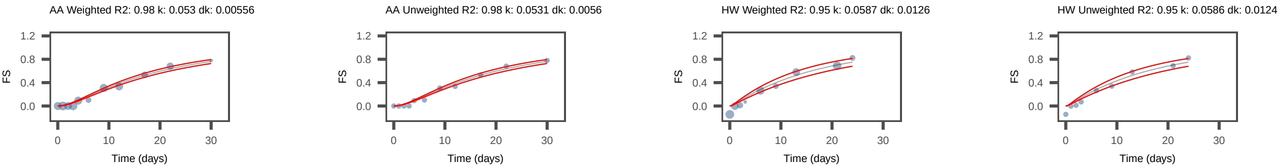

OAT

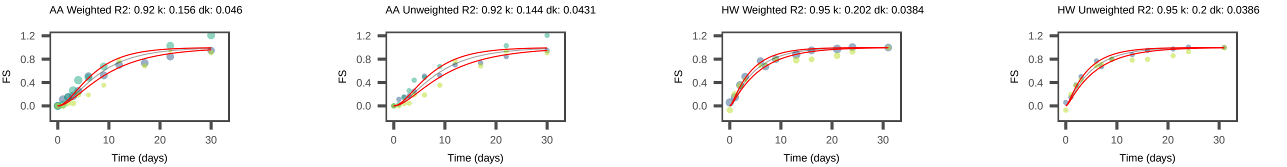

OBSCN

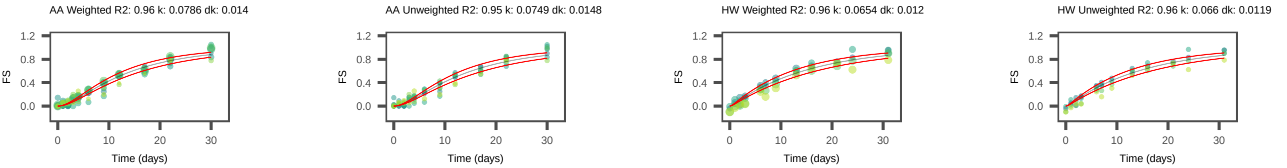

ODB2

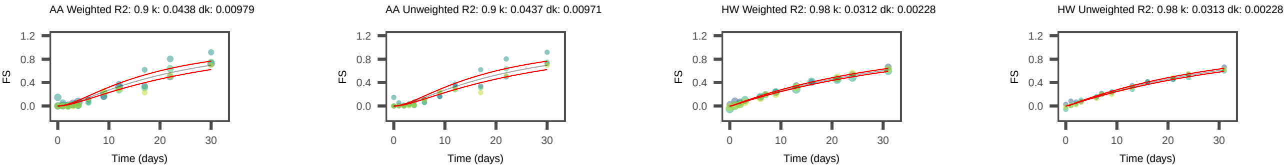

ODBB

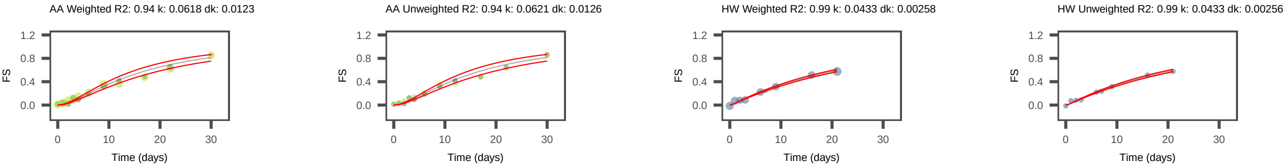

ODO1

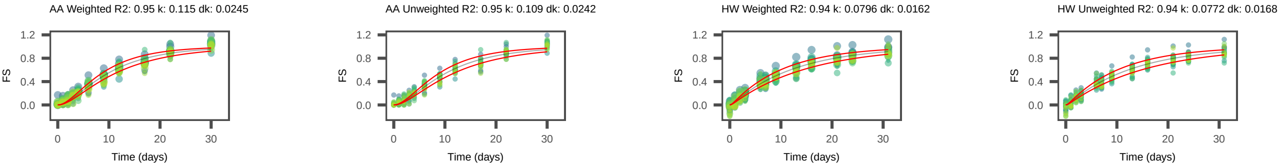

ODO2

AA Weighted R2: 0.95 k: 0.0359 dk: 0.00461 | AA Unweighted R2: 0.94 k: 0.0346 dk: 0.005 | HW Weighted R2: 0.94 k: 0.021 dk: 0.00264 | HW Unweighted R2: 0.94 k: 0.0211 dk: 0.00255

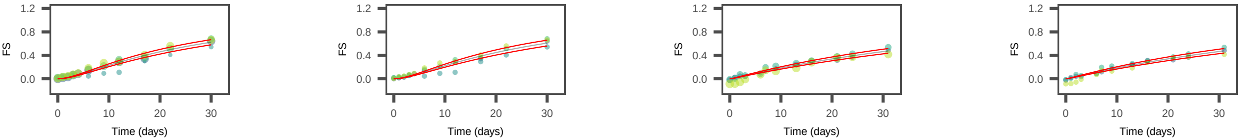

ODP2

AA Weighted R2: 0.93 k: 0.0377 dk: 0.00608 | AA Unweighted R2: 0.92 k: 0.0375 dk: 0.00616 | HW Weighted R2: 0.88 k: 0.0267 dk: 0.00442 | HW Unweighted R2: 0.89 k: 0.0266 dk: 0.00417

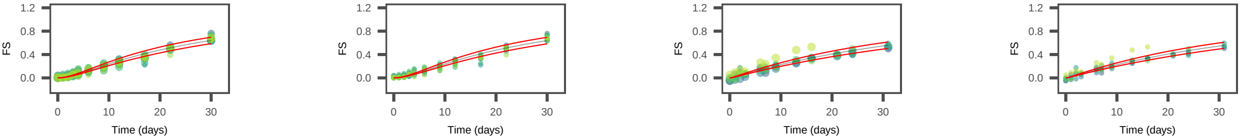

ODPA

AA Weighted R2: 0.93 k: 0.047 dk: 0.00817 | AA Unweighted R2: 0.85 k: 0.05 dk: 0.0129 | HW Weighted R2: 0.93 k: 0.0179 dk: 0.00248 | HW Unweighted R2: 0.93 k: 0.0178 dk: 0.0025

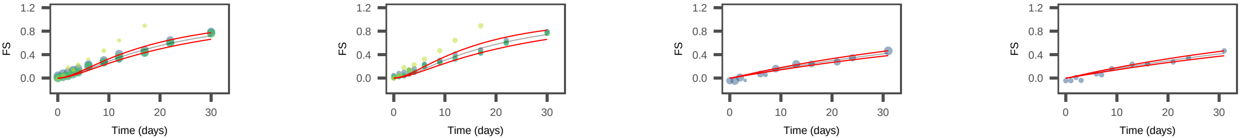

ODPB

AA Weighted R2: 0.95 k: 0.041 dk: 0.00575 | AA Unweighted R2: 0.93 k: 0.0412 dk: 0.00712 | HW Weighted R2: 0.93 k: 0.0302 dk: 0.00463 | HW Unweighted R2: 0.93 k: 0.0294 dk: 0.00462

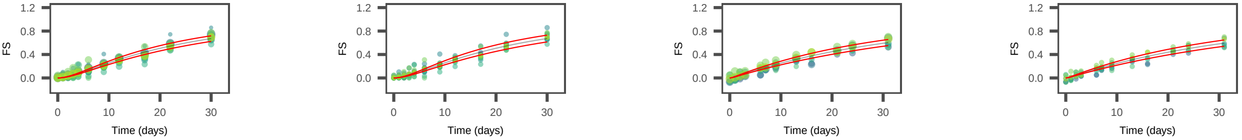

ODPX

AA Weighted R2: 0.95 k: 0.0338 dk: 0.00427 | AA Unweighted R2: 0.95 k: 0.0344 dk: 0.00475 | HW Weighted R2: 0.91 k: 0.0279 dk: 0.00451 | HW Unweighted R2: 0.89 k: 0.0292 dk: 0.00509

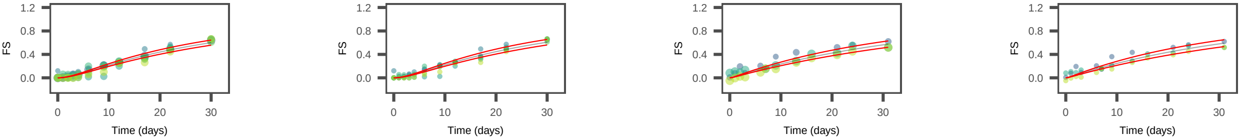

OLA1

AA Weighted R2: 0.97 k: 0.0802 dk: 0.0136 | AA Unweighted R2: 0.96 k: 0.0805 dk: 0.0157 | HW Weighted R2: 0.94 k: 0.0634 dk: 0.014 | HW Unweighted R2: 0.94 k: 0.063 dk: 0.0139

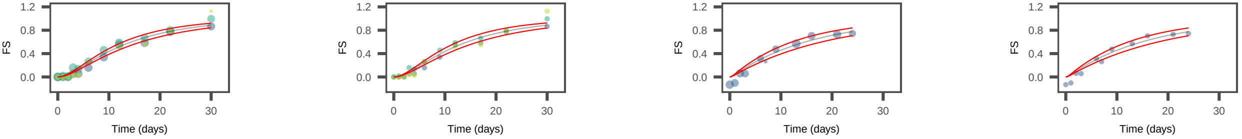

OPA1

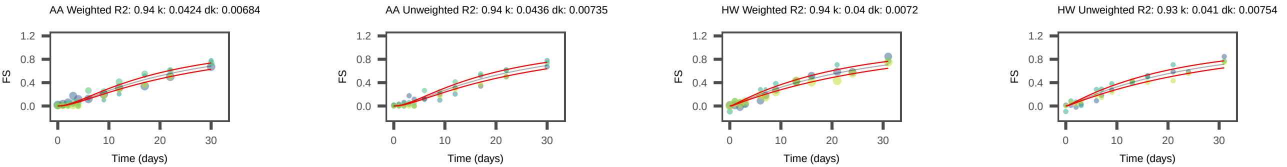

OTUB1

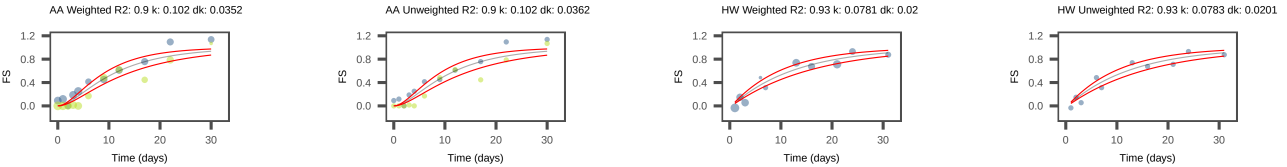

PARK7

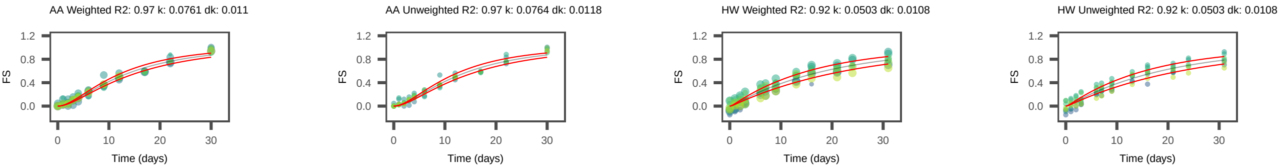

PCBP1

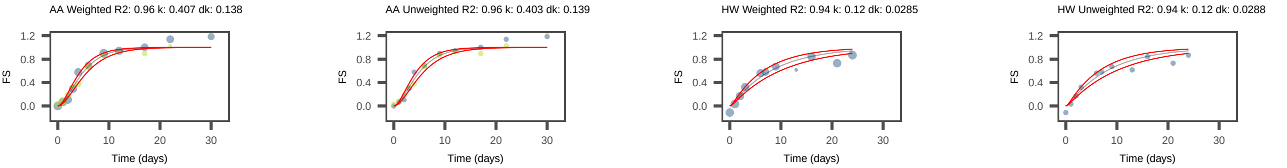

PCBP2

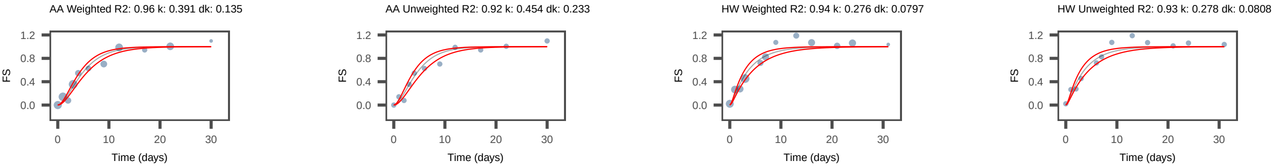

PCCA

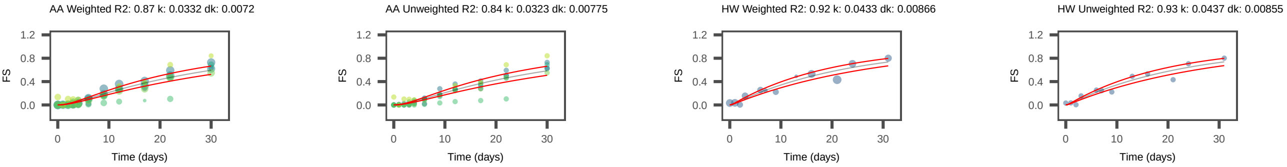

PCCB

AA Weighted R2: 0.95 k: 0.0288 dk: 0.00325
AA Unweighted R2: 0.94 k: 0.0284 dk: 0.00353
HW Weighted R2: 0.94 k: 0.0284 dk: 0.00389
HW Unweighted R2: 0.94 k: 0.0284 dk: 0.00384

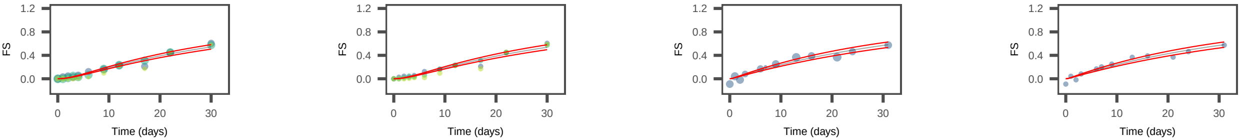

PDIA1

AA Weighted R2: 0.96 k: 0.226 dk: 0.0629
AA Unweighted R2: 0.95 k: 0.223 dk: 0.0698
HW Weighted R2: 0.92 k: 0.2 dk: 0.0577
HW Unweighted R2: 0.91 k: 0.192 dk: 0.0571

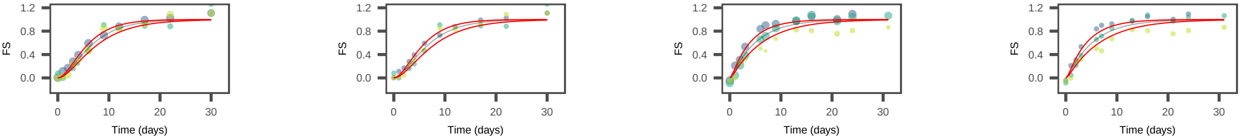

PDIA3

AA Weighted R2: 0.91 k: 0.293 dk: 0.124
AA Unweighted R2: 0.91 k: 0.288 dk: 0.122
HW Weighted R2: 0.7 k: 0.168 dk: 0.087
HW Unweighted R2: 0.72 k: 0.174 dk: 0.0867

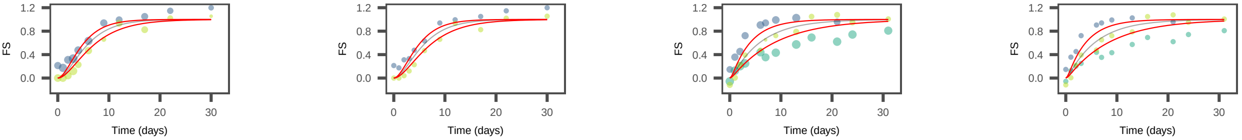

PDK2

AA Weighted R2: 0.93 k: 0.0635 dk: 0.00952
AA Unweighted R2: 0.92 k: 0.0628 dk: 0.00969
HW Weighted R2: 0.87 k: 0.246 dk: 0.0958
HW Unweighted R2: 0.87 k: 0.243 dk: 0.0957

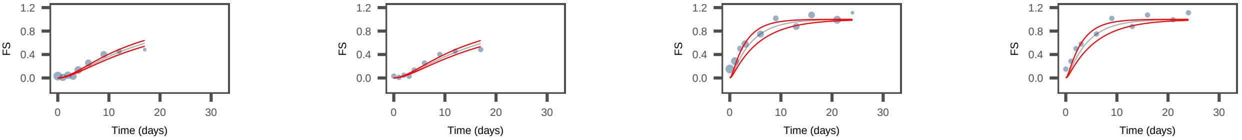

PDLI5

AA Weighted R2: 0.92 k: 0.112 dk: 0.0319
AA Unweighted R2: 0.9 k: 0.0983 dk: 0.031
HW Weighted R2: 0.93 k: 0.0922 dk: 0.0202
HW Unweighted R2: 0.93 k: 0.0952 dk: 0.0209

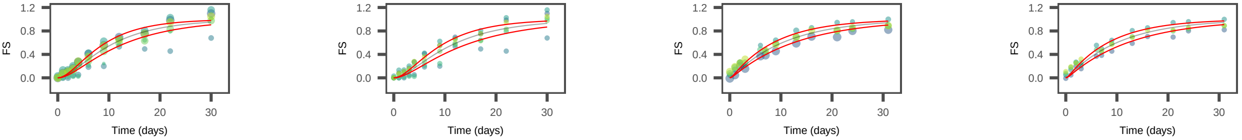

PEBP1

AA Weighted R2: 0.95 k: 0.0735 dk: 0.013
AA Unweighted R2: 0.95 k: 0.0732 dk: 0.0134
HW Weighted R2: 0.96 k: 0.0453 dk: 0.00652
HW Unweighted R2: 0.96 k: 0.0458 dk: 0.00672

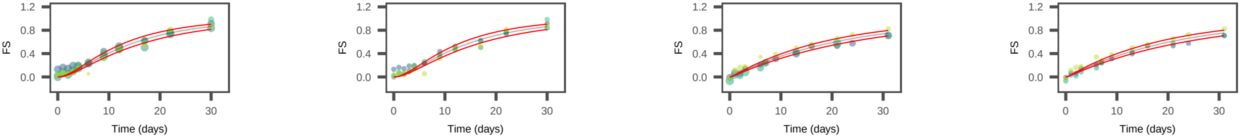

PERM1

AA Weighted R2: 0.92 k: 0.243 dk: 0.088

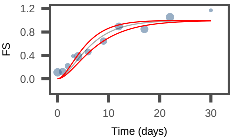

AA Unweighted R2: 0.92 k: 0.244 dk: 0.0896

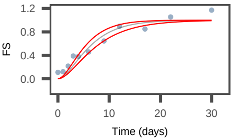

HW Weighted R2: 0.95 k: 0.181 dk: 0.037

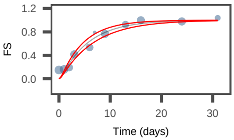

HW Unweighted R2: 0.96 k: 0.182 dk: 0.037

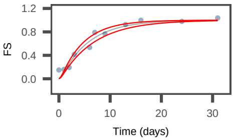

PFKAM

AA Weighted R2: 0.88 k: 0.195 dk: 0.0813

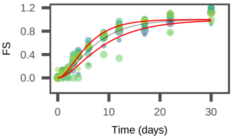

AA Unweighted R2: 0.87 k: 0.184 dk: 0.0809

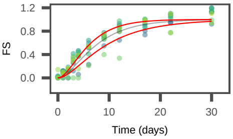

HW Weighted R2: 0.94 k: 0.16 dk: 0.0376

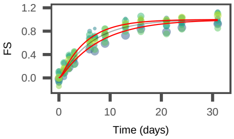

HW Unweighted R2: 0.94 k: 0.165 dk: 0.039

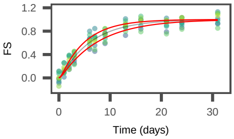

PGAM1

AA Weighted R2: 0.93 k: 0.0703 dk: 0.018

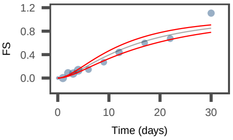

AA Unweighted R2: 0.93 k: 0.0699 dk: 0.0181

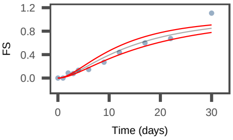

HW Weighted R2: 0.97 k: 0.0622 dk: 0.0094

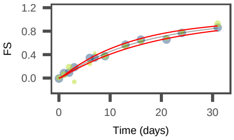

HW Unweighted R2: 0.96 k: 0.062 dk: 0.0104

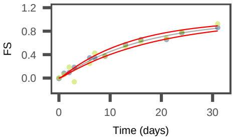

PGAM2

AA Weighted R2: 0.98 k: 0.0744 dk: 0.00841

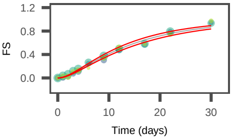

AA Unweighted R2: 0.98 k: 0.0742 dk: 0.00861

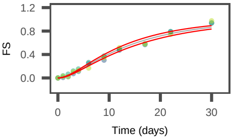

HW Weighted R2: 0.96 k: 0.0493 dk: 0.00764

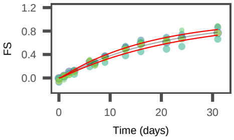

HW Unweighted R2: 0.96 k: 0.0496 dk: 0.00779

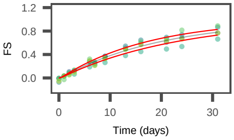

PGBM

AA Weighted R2: 0.87 k: 0.0532 dk: 0.014

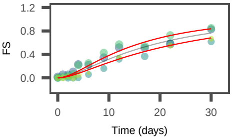

AA Unweighted R2: 0.87 k: 0.0495 dk: 0.0132

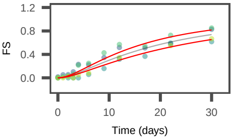

HW Weighted R2: 0.89 k: 0.0414 dk: 0.0101

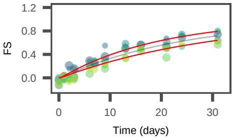

HW Unweighted R2: 0.89 k: 0.0422 dk: 0.0103

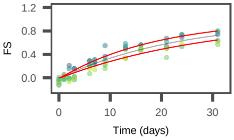

PGK1

AA Weighted R2: 0.95 k: 0.0662 dk: 0.0116

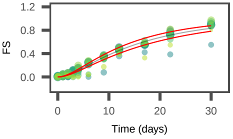

AA Unweighted R2: 0.93 k: 0.062 dk: 0.0134

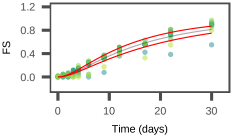

HW Weighted R2: 0.93 k: 0.0454 dk: 0.00877

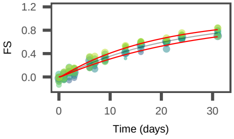

HW Unweighted R2: 0.93 k: 0.0455 dk: 0.00897

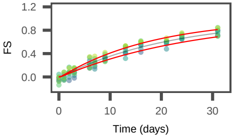

PGM1

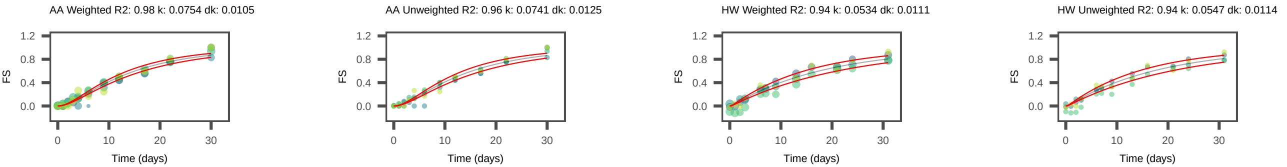

PHB

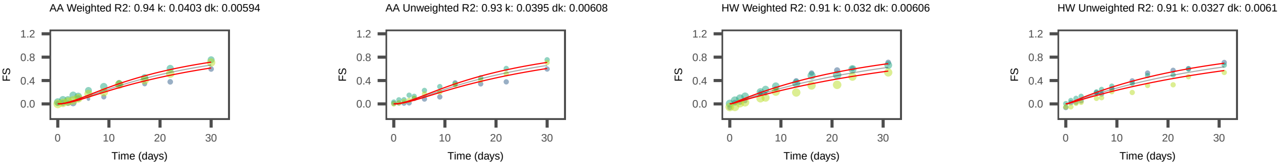

PHB2

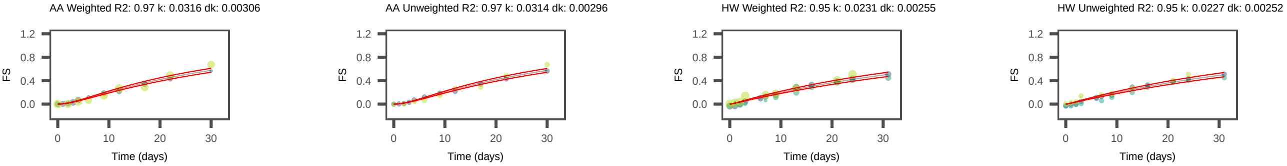

PHP14

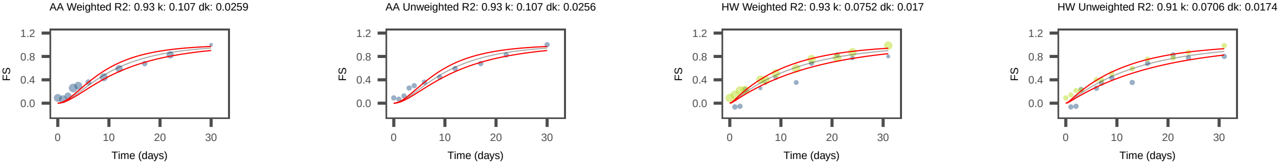

PIMT

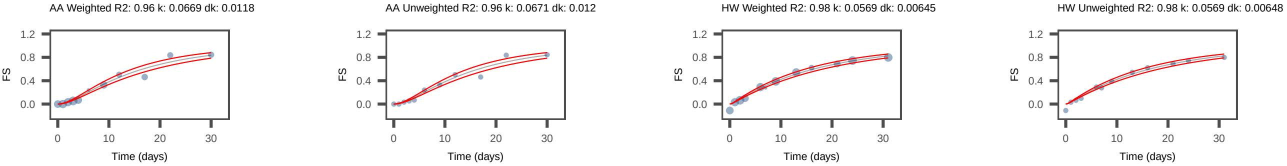

PLAK

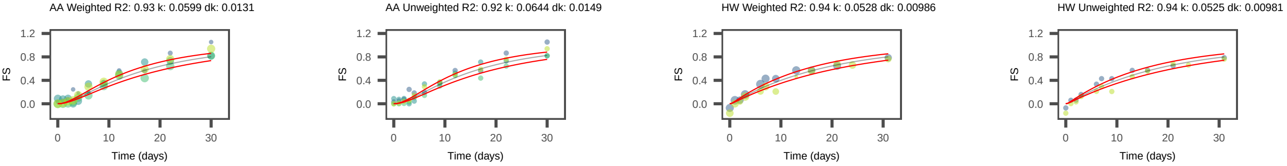

PLEC

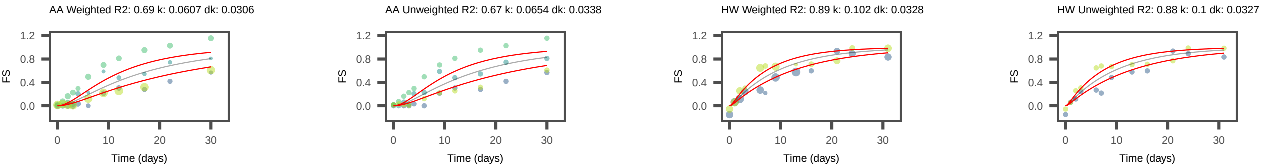

PLIN3

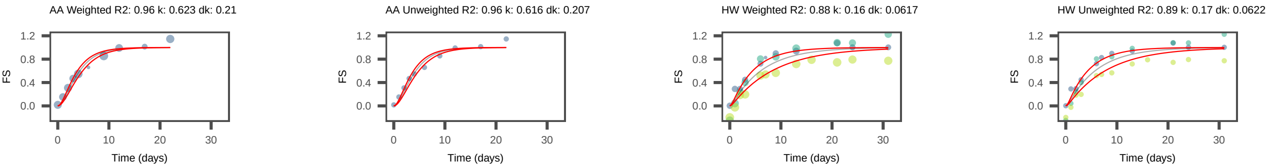

PLIN4

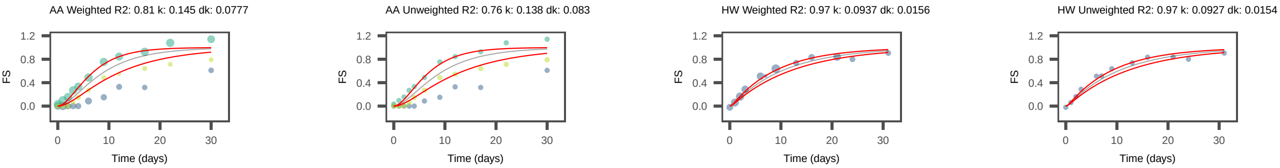

PLST

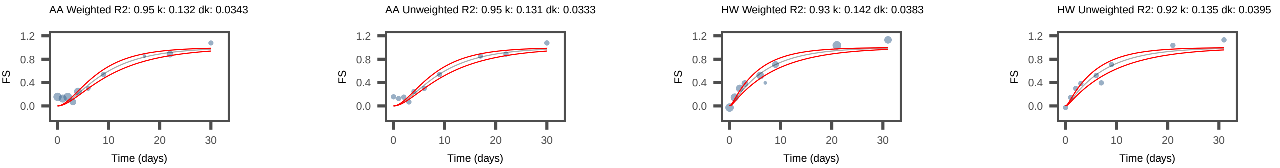

PRDX2

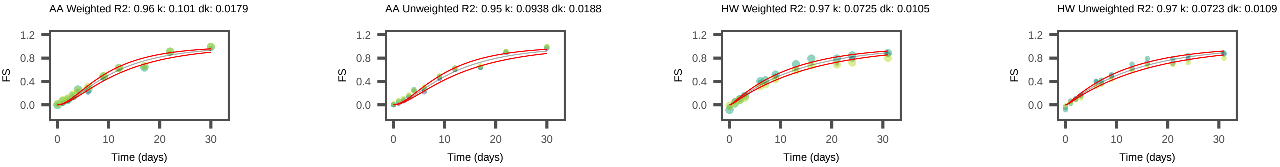

PRDX3

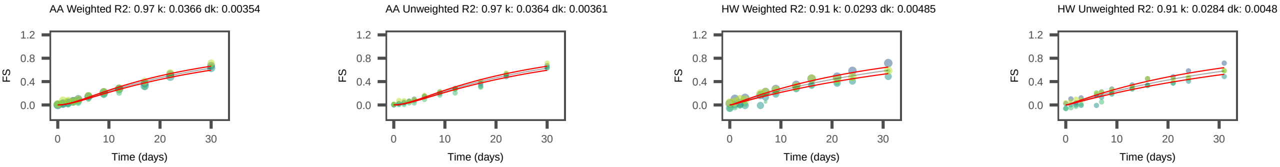

PRDX5

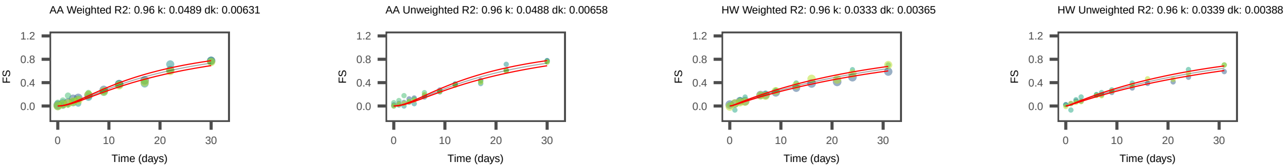

PRDX6

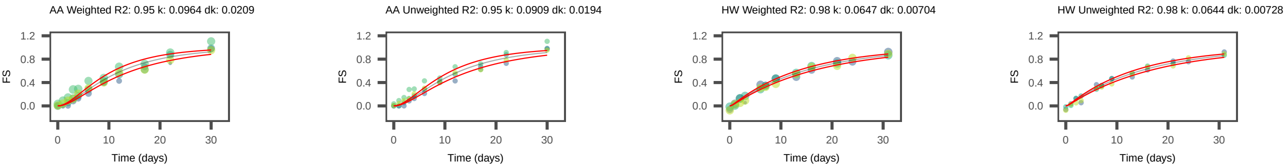

PROF1

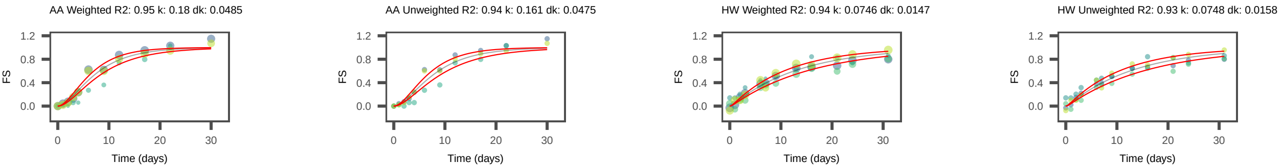

PSA

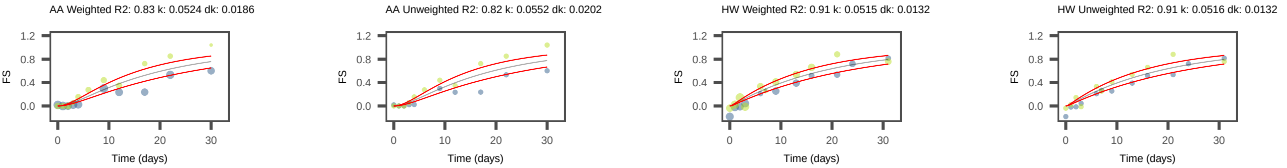

PSA2

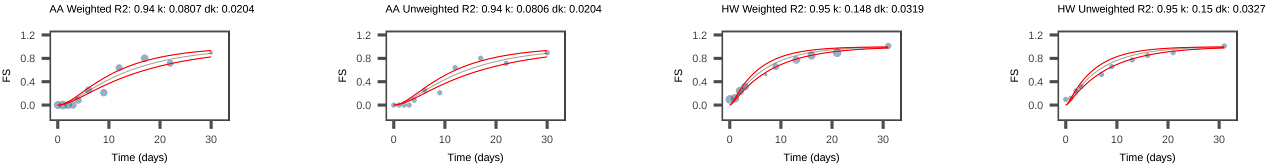

PSA3

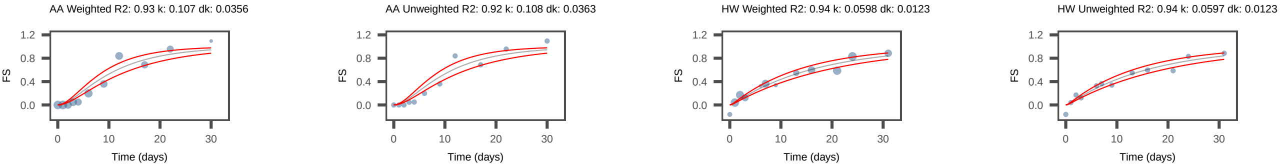

PSA4

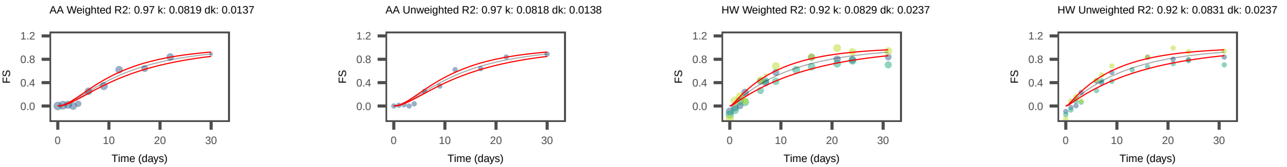

PSB3

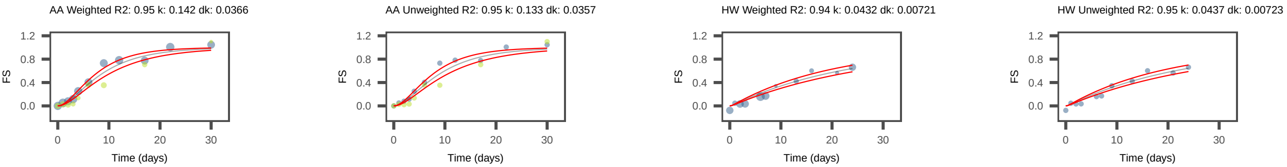

PSD11

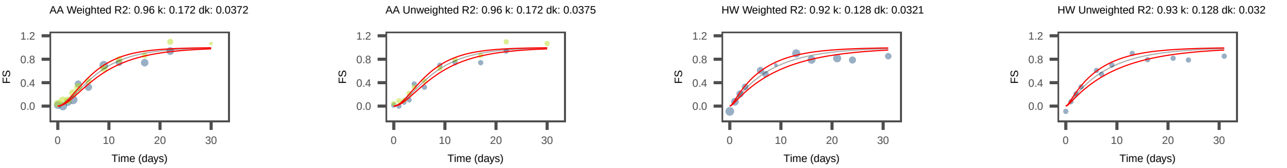

PSD12

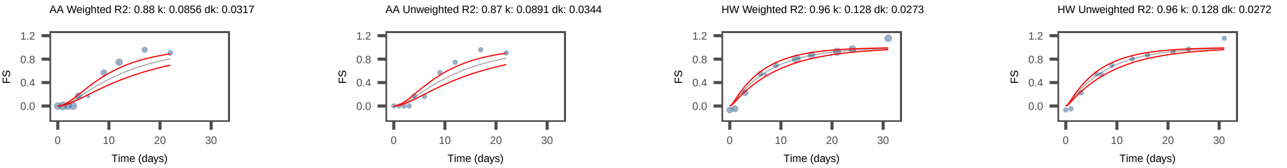

PSMD2

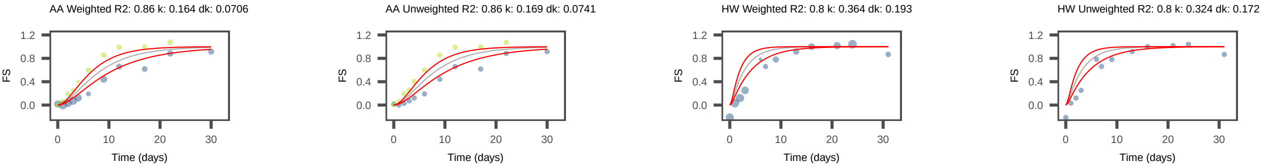

PSMD8

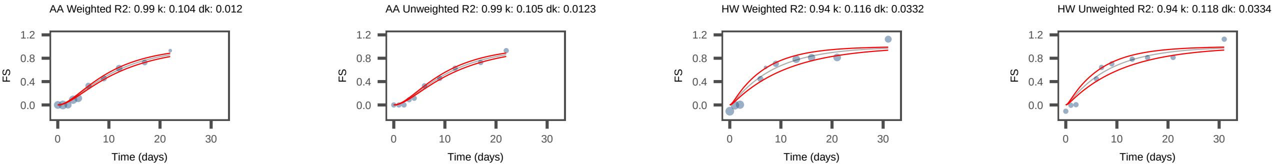

PTGR2

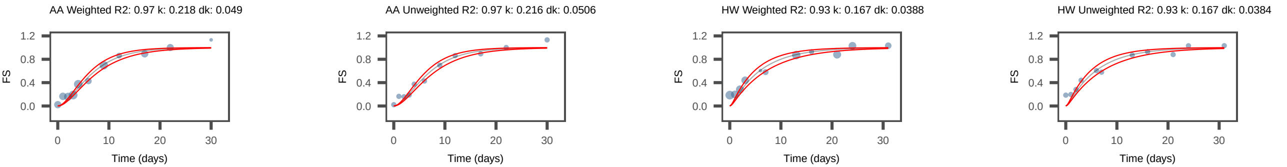

PTGR3

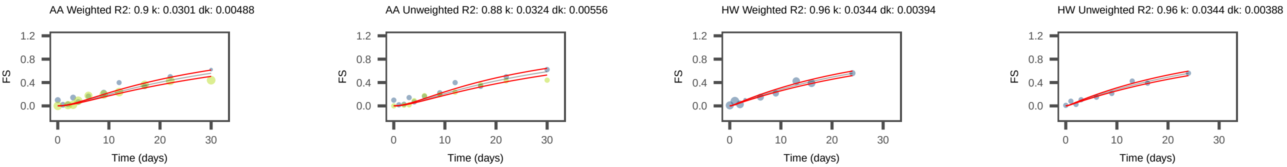

PURA1

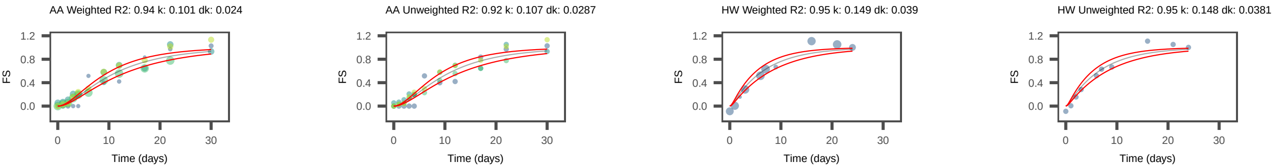

PXMP2

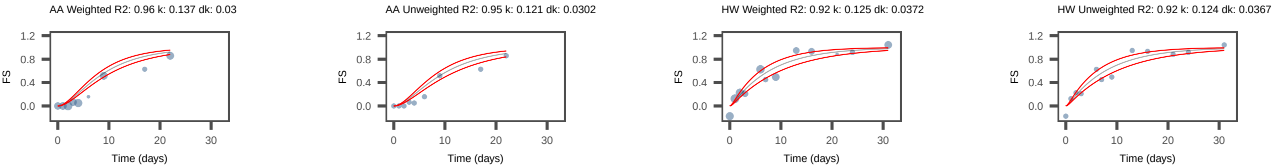

PYGB

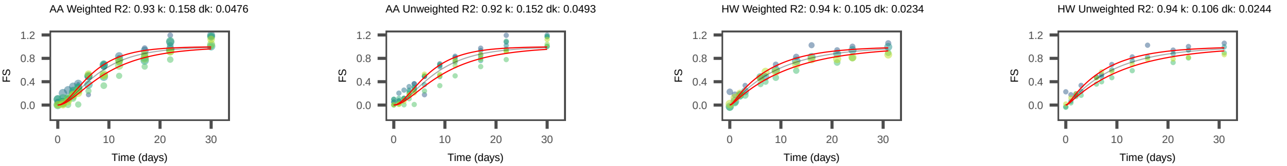

PYGM

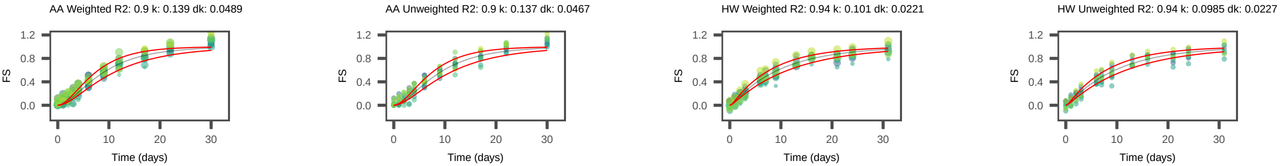

PZP

AA Weighted R2: 0.88 k: 0.306 dk: 0.143

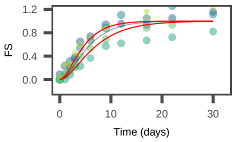

AA Unweighted R2: 0.87 k: 0.31 dk: 0.153

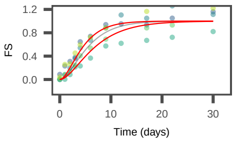

HW Weighted R2: 0.9 k: 0.27 dk: 0.088

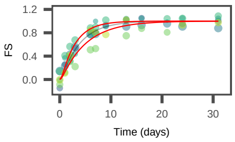

HW Unweighted R2: 0.9 k: 0.27 dk: 0.0876

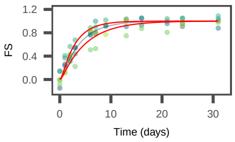

QCR1

AA Weighted R2: 0.86 k: 0.0269 dk: 0.00503

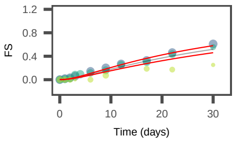

AA Unweighted R2: 0.8 k: 0.0239 dk: 0.00549

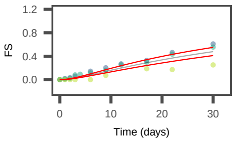

HW Weighted R2: 0.97 k: 0.0258 dk: 0.00214

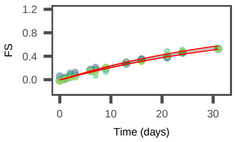

HW Unweighted R2: 0.96 k: 0.026 dk: 0.00246

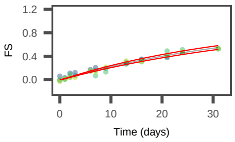

QCR10

AA Weighted R2: 0.99 k: 0.0684 dk: 0.00748

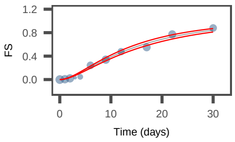

AA Unweighted R2: 0.98 k: 0.0682 dk: 0.00768

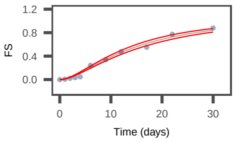

HW Weighted R2: 0.99 k: 0.0486 dk: 0.00415

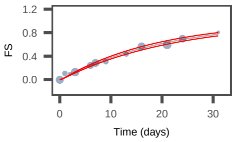

HW Unweighted R2: 0.98 k: 0.0487 dk: 0.00427

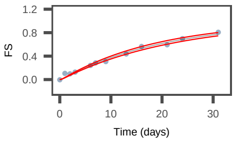

QCR2

AA Weighted R2: 0.95 k: 0.0351 dk: 0.00421

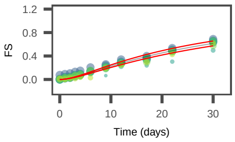

AA Unweighted R2: 0.94 k: 0.0334 dk: 0.00449

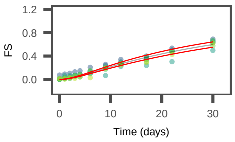

HW Weighted R2: 0.9 k: 0.0231 dk: 0.00348

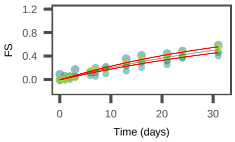

HW Unweighted R2: 0.91 k: 0.0224 dk: 0.00332

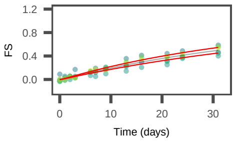

QCR7

AA Weighted R2: 0.95 k: 0.037 dk: 0.00468

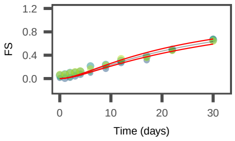

AA Unweighted R2: 0.95 k: 0.0366 dk: 0.0045

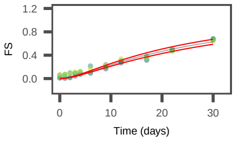

HW Weighted R2: 0.88 k: 0.0301 dk: 0.00589

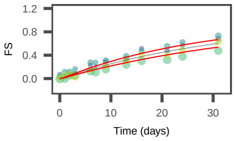

HW Unweighted R2: 0.88 k: 0.0315 dk: 0.00602

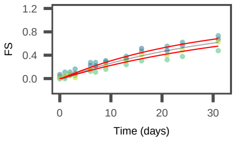

RAB1B

AA Weighted R2: 0.95 k: 0.0729 dk: 0.0123

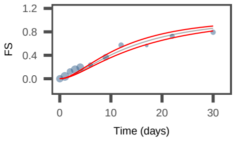

AA Unweighted R2: 0.95 k: 0.0724 dk: 0.0122

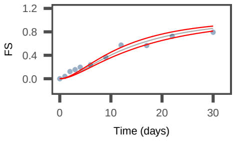

HW Weighted R2: 0.93 k: 0.108 dk: 0.0244

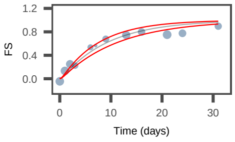

HW Unweighted R2: 0.93 k: 0.109 dk: 0.0244

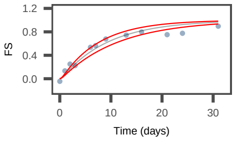

RACK1

AA Weighted R2: 0.95 k: 0.251 dk: 0.0786

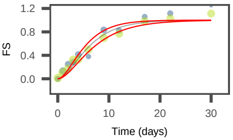

AA Unweighted R2: 0.94 k: 0.251 dk: 0.0824

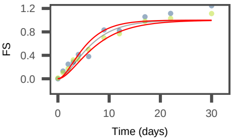

HW Weighted R2: 0.91 k: 0.132 dk: 0.0432

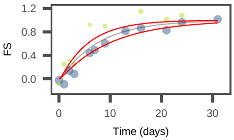

HW Unweighted R2: 0.89 k: 0.146 dk: 0.0527

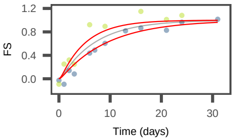

REEP5

AA Weighted R2: 0.95 k: 0.087 dk: 0.0173

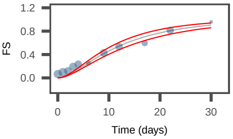

AA Unweighted R2: 0.95 k: 0.0868 dk: 0.0172

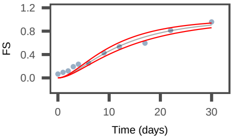

HW Weighted R2: 1 k: 0.0577 dk: 0.00311

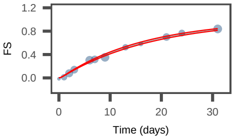

HW Unweighted R2: 1 k: 0.0577 dk: 0.00308

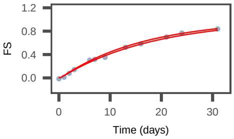

RL15

AA Weighted R2: 0.98 k: 0.143 dk: 0.02

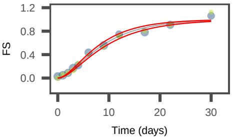

AA Unweighted R2: 0.98 k: 0.143 dk: 0.0208

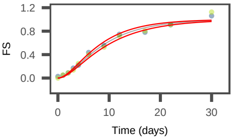

HW Weighted R2: 0.97 k: 0.0858 dk: 0.0145

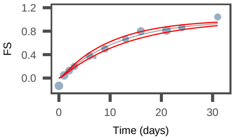

HW Unweighted R2: 0.97 k: 0.0855 dk: 0.0145

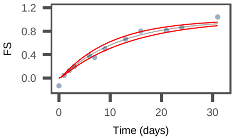

RL23

AA Weighted R2: 0.92 k: 0.113 dk: 0.0418

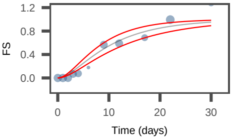

AA Unweighted R2: 0.91 k: 0.111 dk: 0.0425

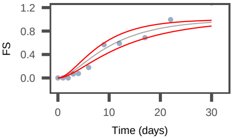

HW Weighted R2: 0.94 k: 0.096 dk: 0.025

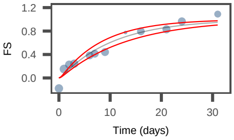

HW Unweighted R2: 0.94 k: 0.0966 dk: 0.0251

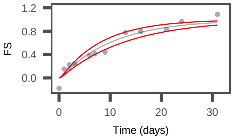

RL4

AA Weighted R2: 0.96 k: 0.0957 dk: 0.019

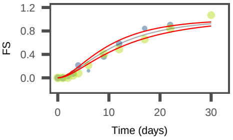

AA Unweighted R2: 0.96 k: 0.0961 dk: 0.0198

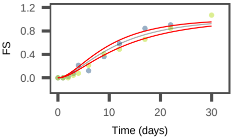

HW Weighted R2: 0.94 k: 0.0501 dk: 0.00887

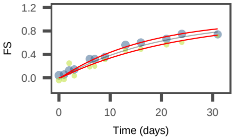

HW Unweighted R2: 0.93 k: 0.0484 dk: 0.0089

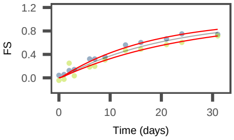

RL5

AA Weighted R2: 0.98 k: 0.0765 dk: 0.00961

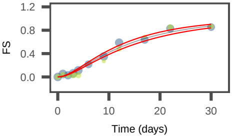

AA Unweighted R2: 0.98 k: 0.0759 dk: 0.00992

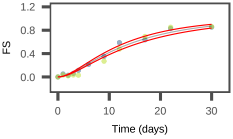

HW Weighted R2: 0.93 k: 0.0522 dk: 0.0127

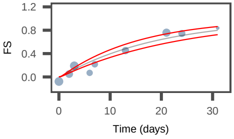

HW Unweighted R2: 0.93 k: 0.0519 dk: 0.0127

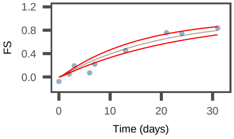

RL7A

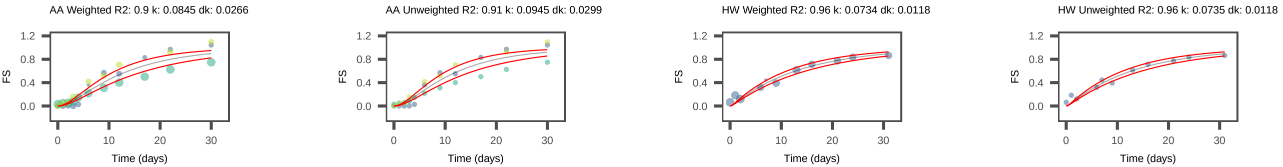

RL8

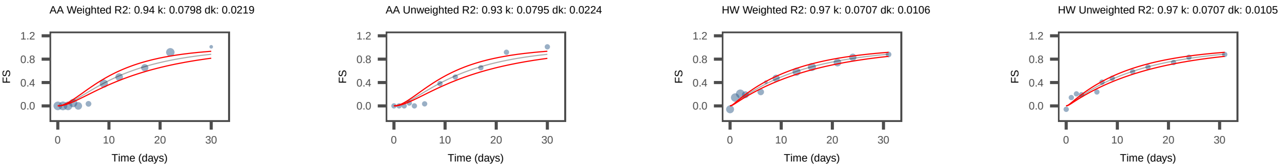

RL9

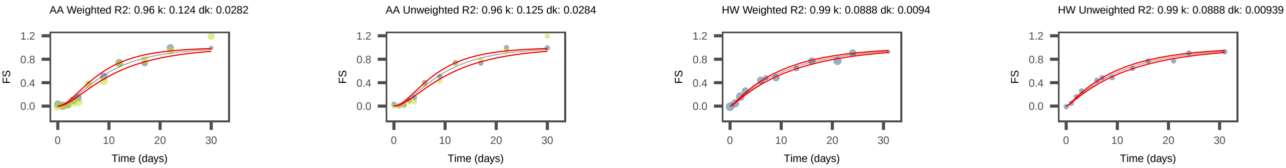

RLA0

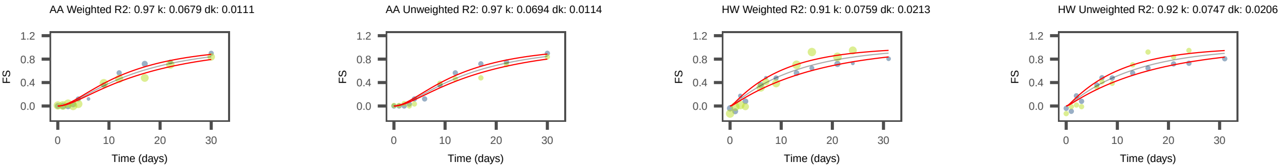

RLA2

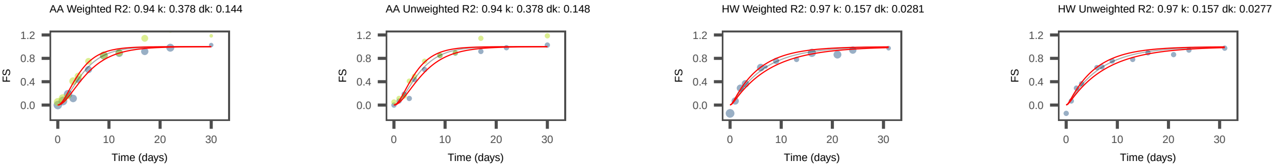

ROA2

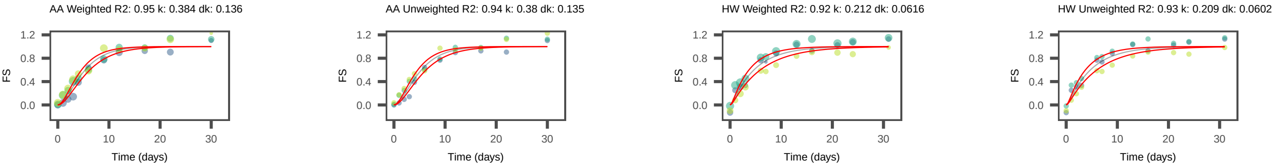

ROA3

AA Weighted R2: 0.94 k: 0.136 dk: 0.0402

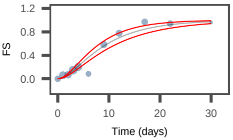

AA Unweighted R2: 0.94 k: 0.135 dk: 0.041

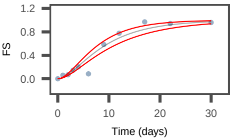

HW Weighted R2: 0.97 k: 0.169 dk: 0.0307

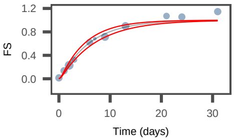

HW Unweighted R2: 0.97 k: 0.169 dk: 0.0307

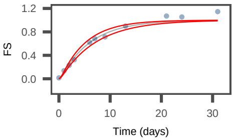

RS10

AA Weighted R2: 0.99 k: 0.147 dk: 0.0199

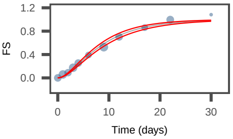

AA Unweighted R2: 0.99 k: 0.148 dk: 0.0202

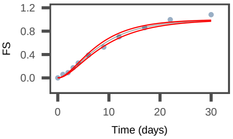

HW Weighted R2: 0.98 k: 0.0954 dk: 0.0144

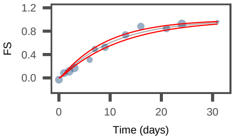

HW Unweighted R2: 0.98 k: 0.0953 dk: 0.0144

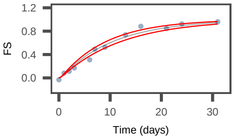

RS11

AA Weighted R2: 0.97 k: 0.122 dk: 0.0227

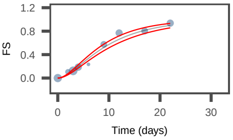

AA Unweighted R2: 0.97 k: 0.121 dk: 0.023

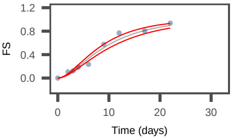

HW Weighted R2: 0.96 k: 0.0658 dk: 0.0121

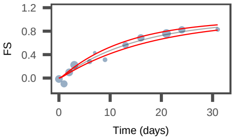

HW Unweighted R2: 0.96 k: 0.0657 dk: 0.0122

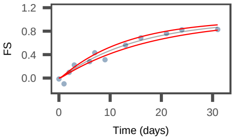

RS13

AA Weighted R2: 0.99 k: 0.122 dk: 0.0146

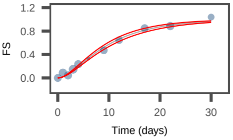

AA Unweighted R2: 0.99 k: 0.122 dk: 0.0147

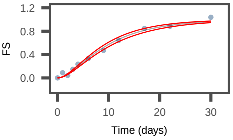

HW Weighted R2: 0.99 k: 0.104 dk: 0.0117

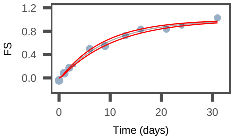

HW Unweighted R2: 0.99 k: 0.104 dk: 0.0117

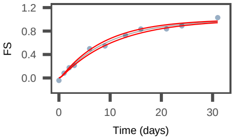

RS14

AA Weighted R2: 0.97 k: 0.124 dk: 0.0267

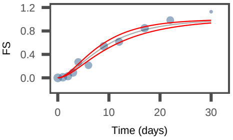

AA Unweighted R2: 0.96 k: 0.125 dk: 0.0286

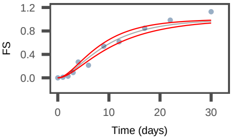

HW Weighted R2: 0.97 k: 0.0804 dk: 0.0123

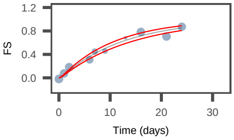

HW Unweighted R2: 0.97 k: 0.0804 dk: 0.0123

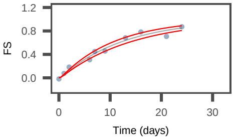

RS15

AA Weighted R2: 0.91 k: 0.0962 dk: 0.0317

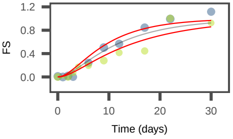

AA Unweighted R2: 0.91 k: 0.0908 dk: 0.0301

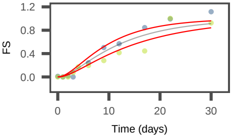

HW Weighted R2: 0.97 k: 0.072 dk: 0.012

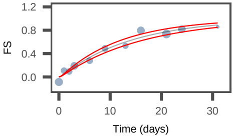

HW Unweighted R2: 0.97 k: 0.0716 dk: 0.0118

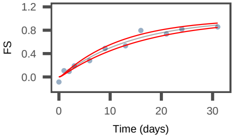

RS16

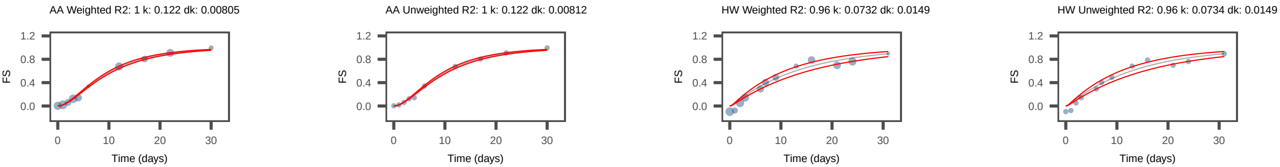

RS17

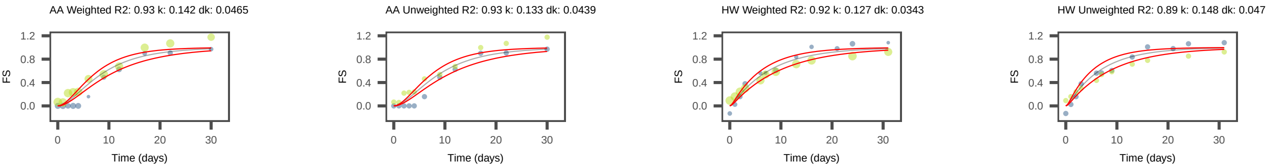

RS2

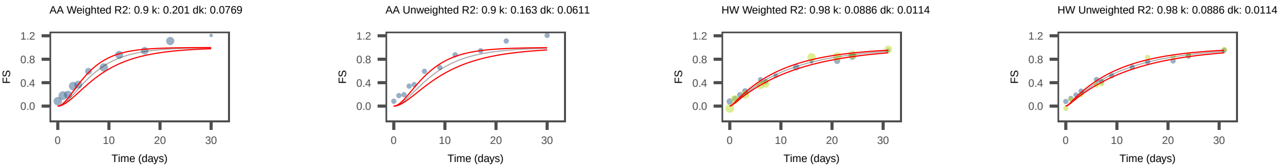

RS23

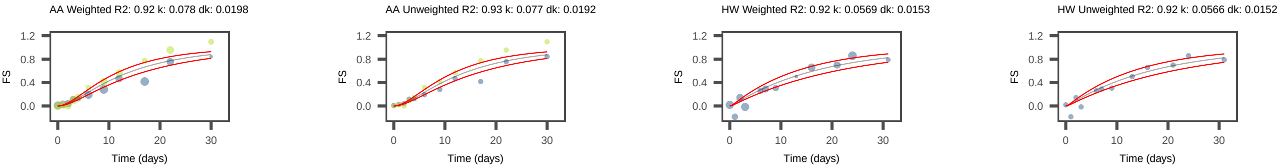

RS24

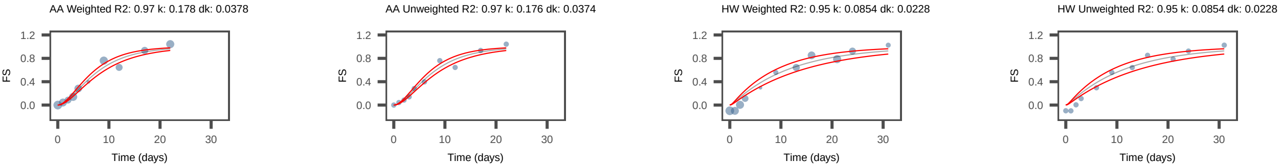

RS4X

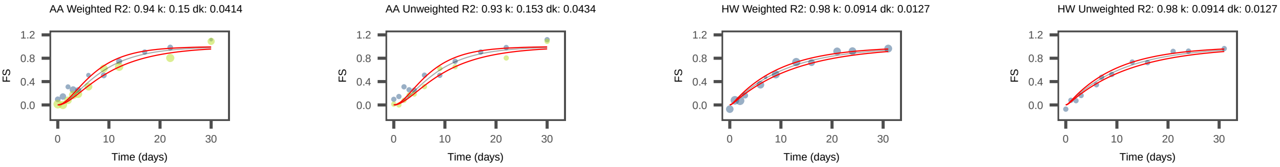

RS7

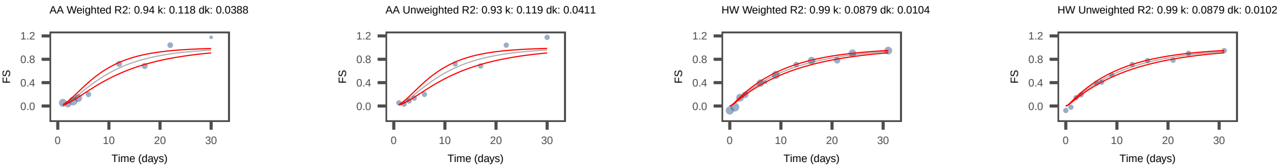

RT4I1

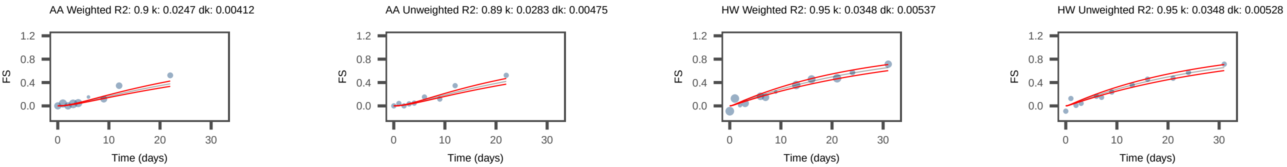

RTN2

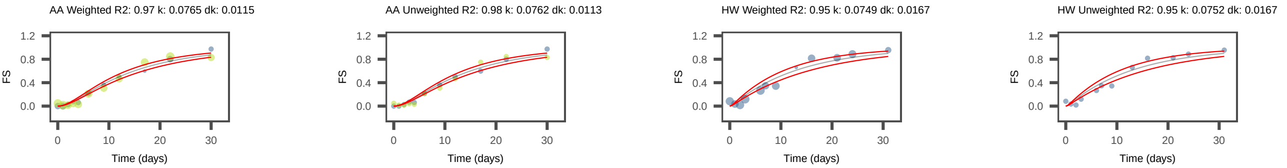

RYR2

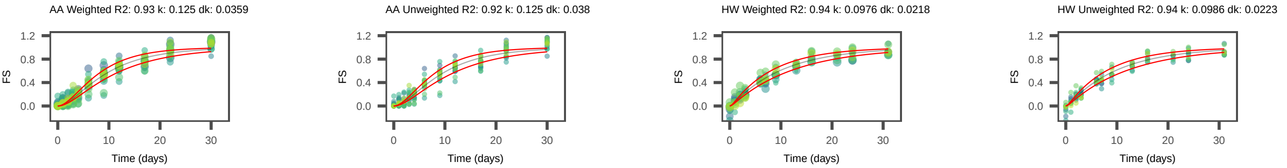

S10A1

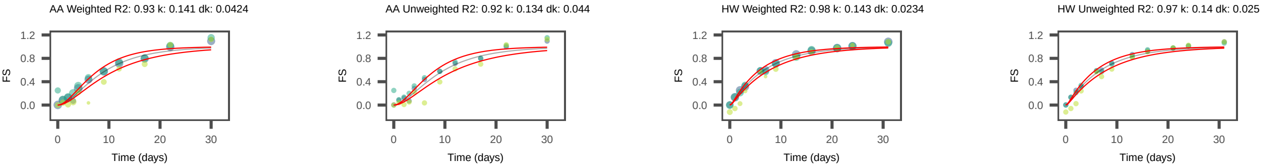

S27A1

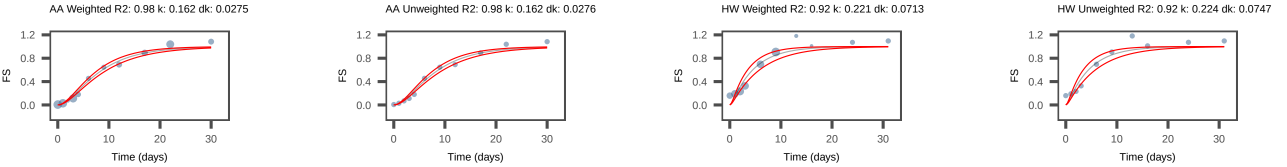

SAM50

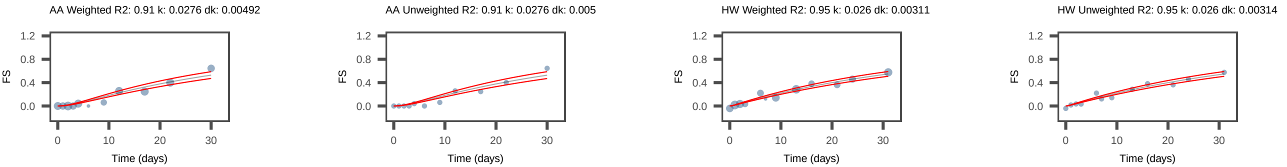

SAP

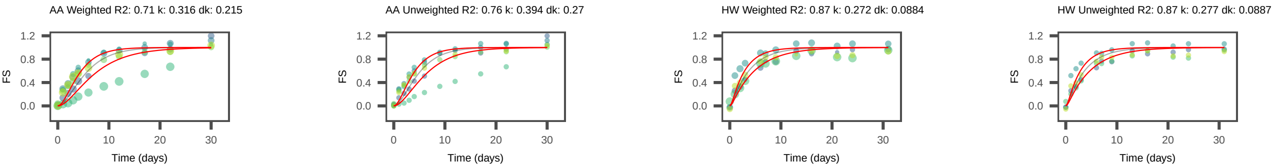

SBP1

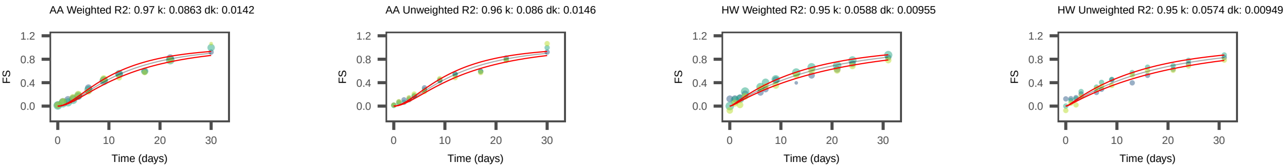

SCOT1

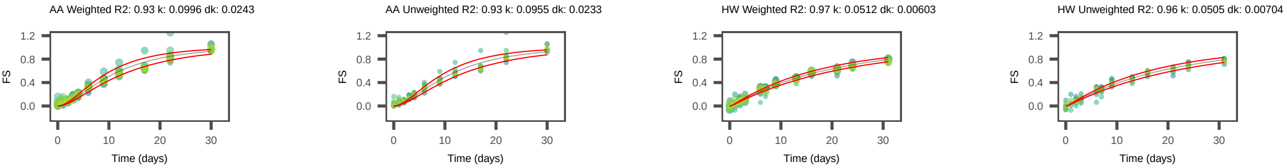

SCP2

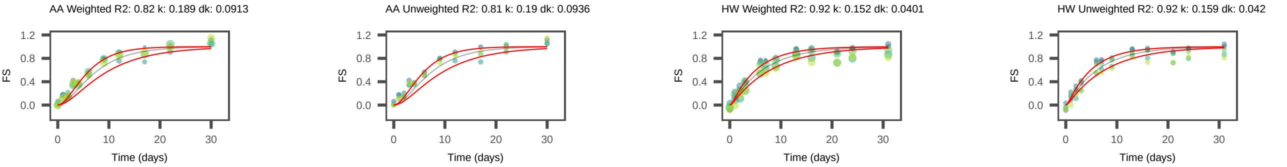

SDHA

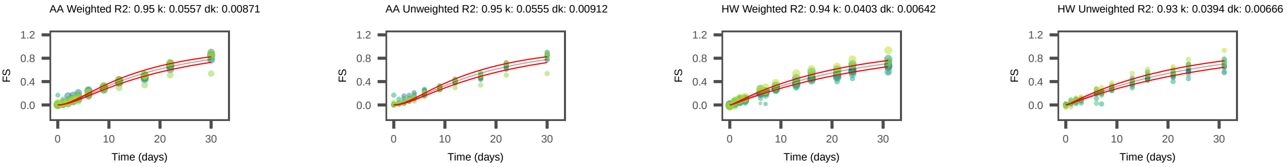

SDHB

AA Weighted R2: 0.98 k: 0.0688 dk: 0.00863 | AA Unweighted R2: 0.97 k: 0.0677 dk: 0.0097 | HW Weighted R2: 0.89 k: 0.049 dk: 0.012 | HW Unweighted R2: 0.88 k: 0.0479 dk: 0.0123

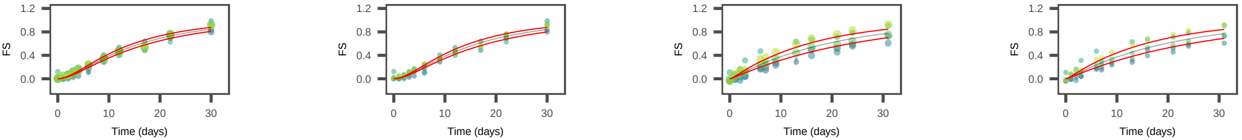

SERPH

AA Weighted R2: 0.95 k: 0.388 dk: 0.14 | AA Unweighted R2: 0.95 k: 0.381 dk: 0.139 | HW Weighted R2: 0.92 k: 0.302 dk: 0.0961 | HW Unweighted R2: 0.93 k: 0.298 dk: 0.0943

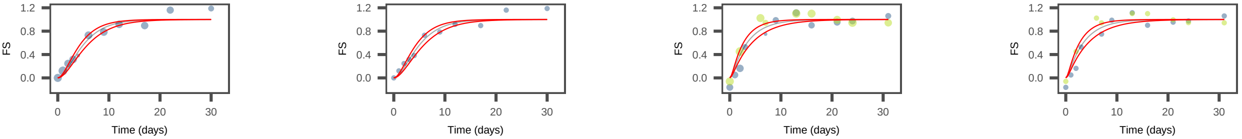

SGCD

AA Weighted R2: 0.94 k: 0.0533 dk: 0.012 | AA Unweighted R2: 0.93 k: 0.0536 dk: 0.0124 | HW Weighted R2: 0.95 k: 0.0456 dk: 0.00671 | HW Unweighted R2: 0.95 k: 0.0457 dk: 0.00662

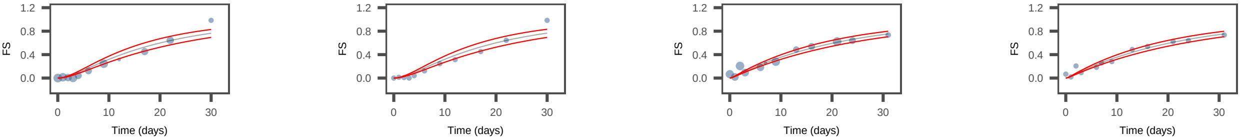

SH3BG

AA Weighted R2: 0.92 k: 0.459 dk: 0.226 | AA Unweighted R2: 0.91 k: 0.454 dk: 0.228 | HW Weighted R2: 0.94 k: 0.0993 dk: 0.0222 | HW Unweighted R2: 0.94 k: 0.0995 dk: 0.022

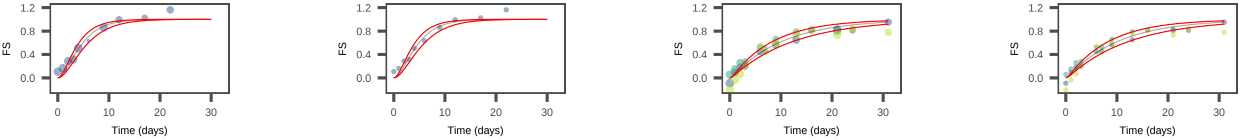

SMYD1

AA Weighted R2: 0.96 k: 0.0551 dk: 0.00838 | AA Unweighted R2: 0.95 k: 0.0518 dk: 0.00833 | HW Weighted R2: 0.92 k: 0.0484 dk: 0.0108 | HW Unweighted R2: 0.92 k: 0.0468 dk: 0.0111

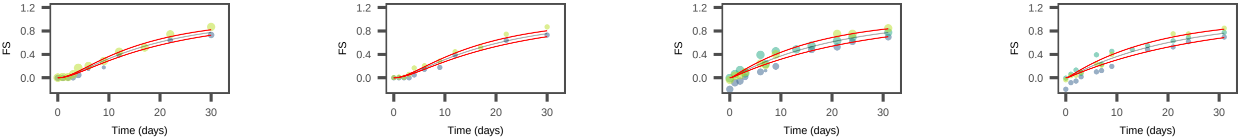

SODC

AA Weighted R2: 0.93 k: 0.0774 dk: 0.0177 | AA Unweighted R2: 0.93 k: 0.0775 dk: 0.0176 | HW Weighted R2: 0.95 k: 0.0649 dk: 0.0102 | HW Unweighted R2: 0.95 k: 0.0649 dk: 0.0102

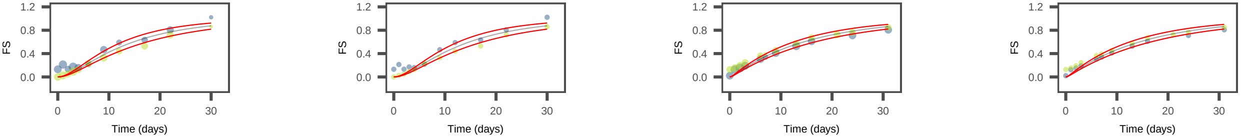

SODM

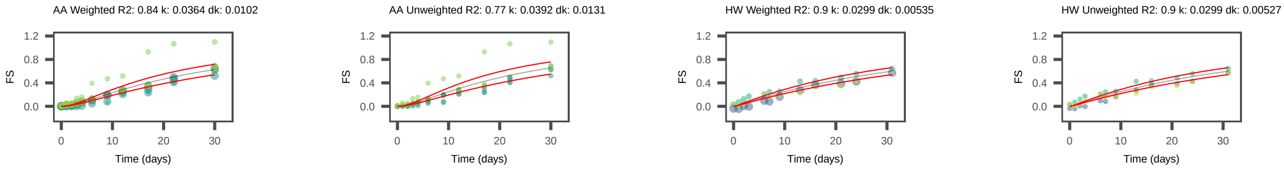

SPA3K

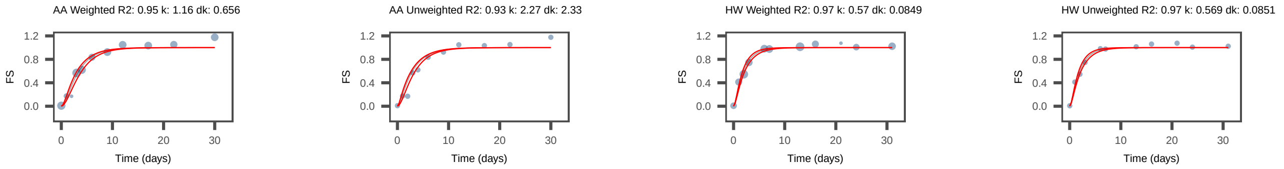

SPB6

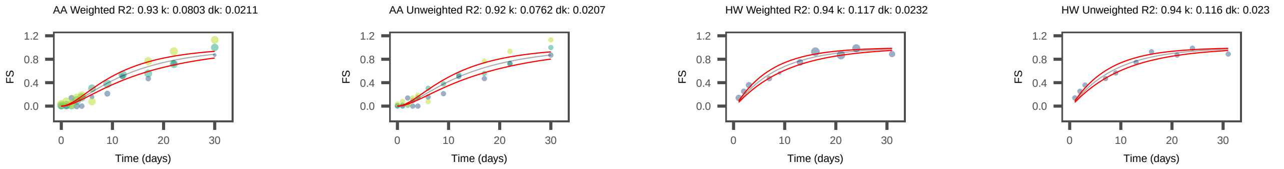

SPEG

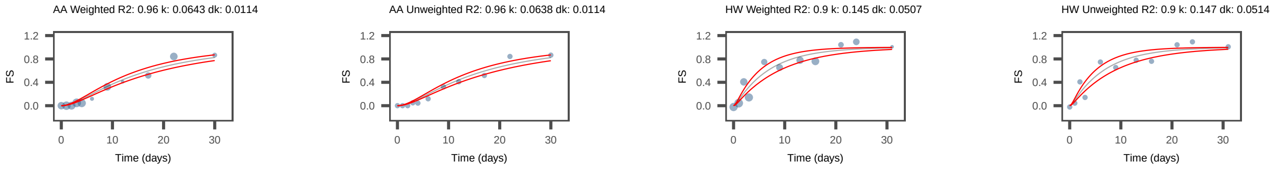

SPTB2

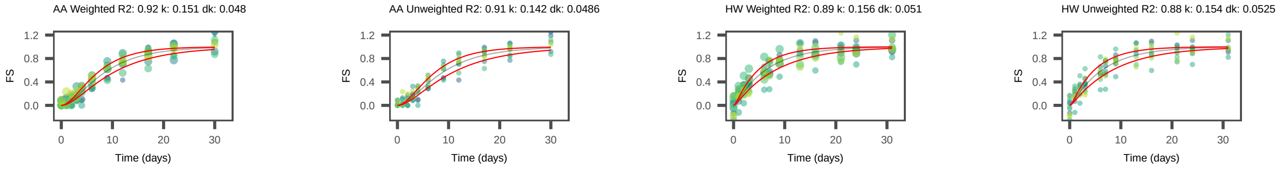

SPTN1

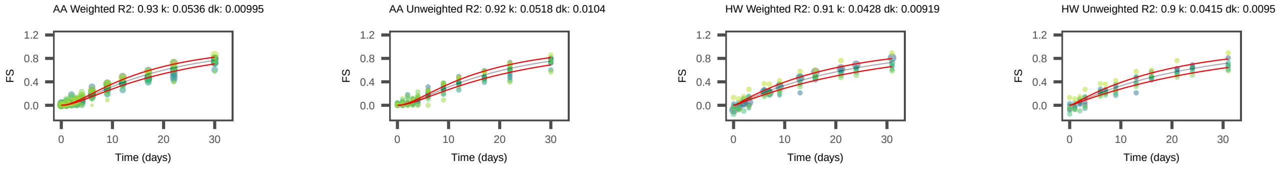

SRBS1

AA Weighted R2: 0.99 k: 0.12 dk: 0.016

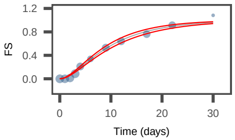

AA Unweighted R2: 0.99 k: 0.12 dk: 0.0167

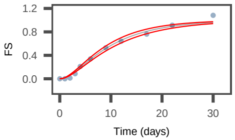

HW Weighted R2: 0.98 k: 0.103 dk: 0.0143

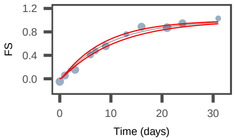

HW Unweighted R2: 0.98 k: 0.104 dk: 0.0143

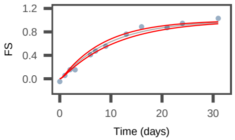

SRBS2

AA Weighted R2: 0.94 k: 0.172 dk: 0.0504

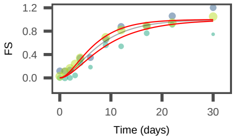

AA Unweighted R2: 0.92 k: 0.156 dk: 0.0508

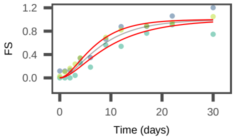

HW Weighted R2: 0.87 k: 0.114 dk: 0.0408

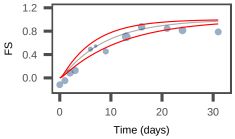

HW Unweighted R2: 0.87 k: 0.116 dk: 0.0414

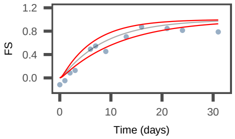

SRCA

AA Weighted R2: 0.94 k: 0.0348 dk: 0.0046

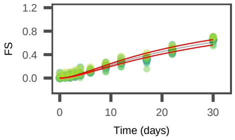

AA Unweighted R2: 0.94 k: 0.0344 dk: 0.00481

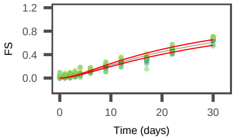

HW Weighted R2: 0.92 k: 0.0239 dk: 0.00356

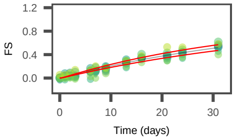

HW Unweighted R2: 0.91 k: 0.0235 dk: 0.00366

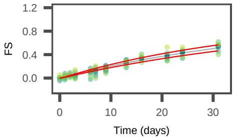

SUCA

AA Weighted R2: 0.96 k: 0.0359 dk: 0.00436

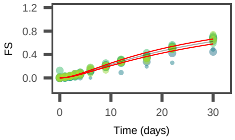

AA Unweighted R2: 0.94 k: 0.0351 dk: 0.00528

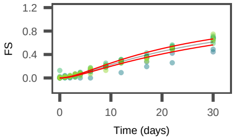

HW Weighted R2: 0.92 k: 0.0236 dk: 0.00356

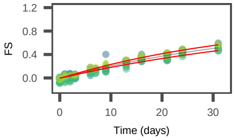

HW Unweighted R2: 0.92 k: 0.0238 dk: 0.00356

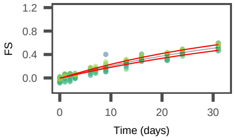

SUCB1

AA Weighted R2: 0.94 k: 0.0451 dk: 0.00732

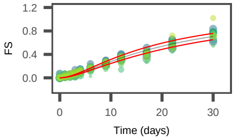

AA Unweighted R2: 0.93 k: 0.0455 dk: 0.00845

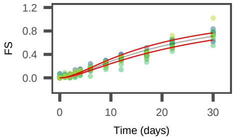

HW Weighted R2: 0.93 k: 0.0328 dk: 0.005

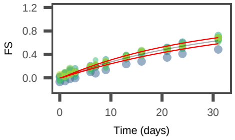

HW Unweighted R2: 0.93 k: 0.0332 dk: 0.00485

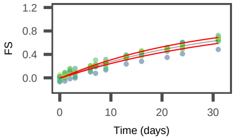

SUCB2

AA Weighted R2: 0.95 k: 0.0825 dk: 0.0175

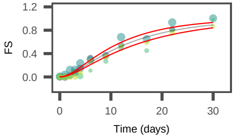

AA Unweighted R2: 0.95 k: 0.0798 dk: 0.017

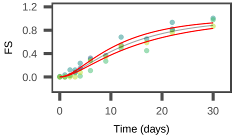

HW Weighted R2: 0.96 k: 0.0716 dk: 0.0121

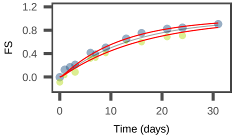

HW Unweighted R2: 0.96 k: 0.071 dk: 0.012

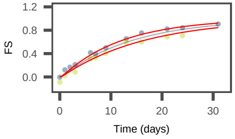

SYNP2

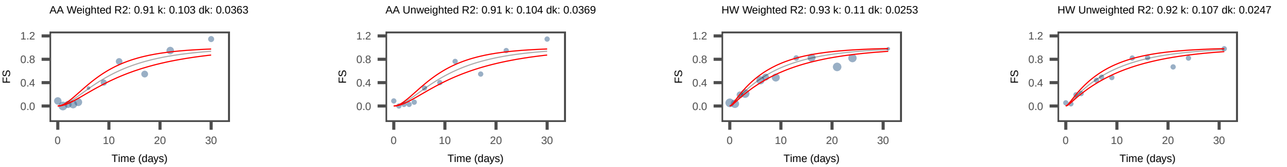

TADBP

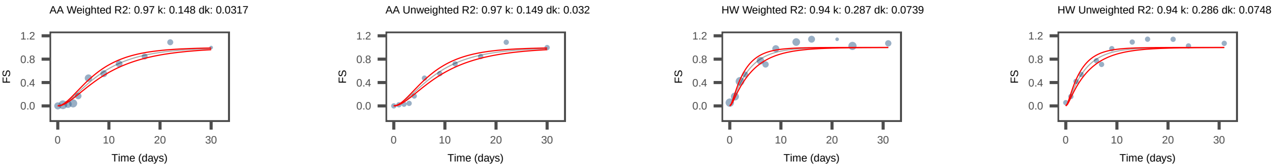

TALDO

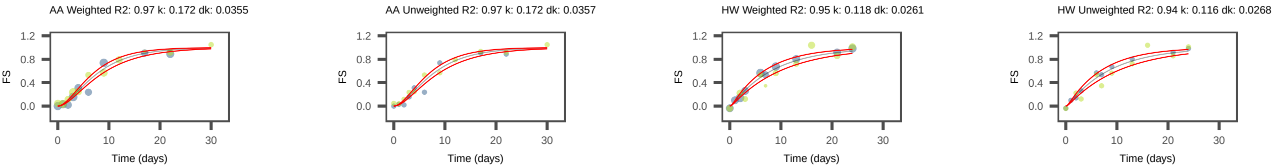

TBA4A

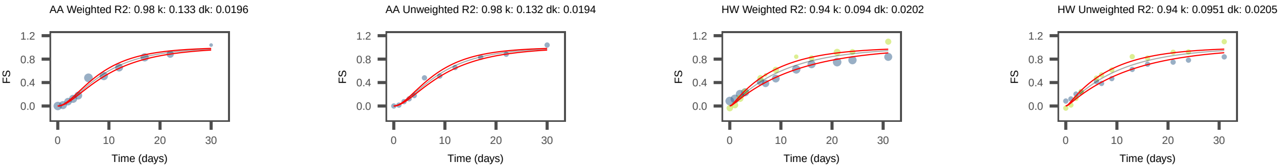

TBB2A

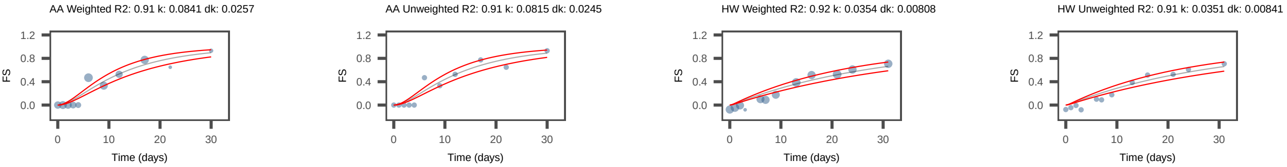

TBB5

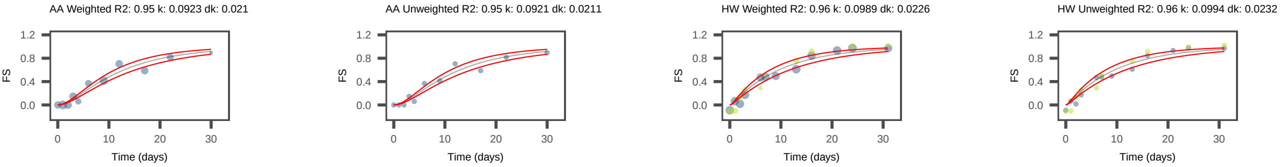

TCPA

AA Weighted R2: 0.91 k: 9.83 dk: 39.9 | AA Unweighted R2: 0.91 k: 9.51 dk: 37.7 | HW Weighted R2: 0.95 k: 0.208 dk: 0.0508 | HW Unweighted R2: 0.95 k: 0.2 dk: 0.0495

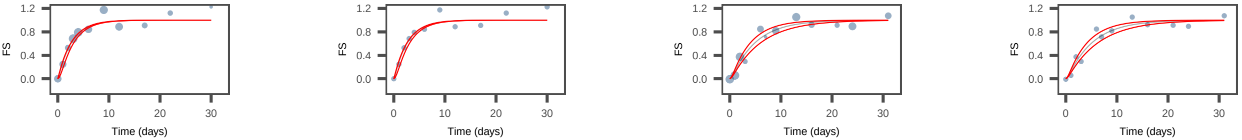

TCPB

AA Weighted R2: 0.96 k: 0.164 dk: 0.0434 | AA Unweighted R2: 0.96 k: 0.164 dk: 0.0436 | HW Weighted R2: 0.96 k: 0.147 dk: 0.0298 | HW Unweighted R2: 0.96 k: 0.148 dk: 0.0298

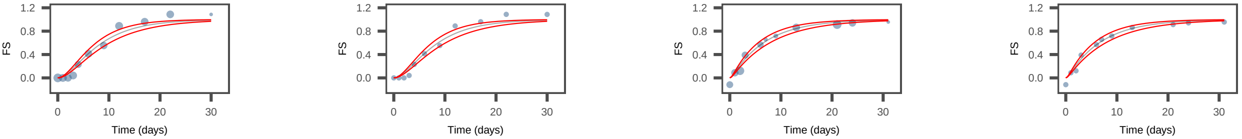

TCPD

AA Weighted R2: 0.97 k: 0.211 dk: 0.0552 | AA Unweighted R2: 0.97 k: 0.212 dk: 0.0568 | HW Weighted R2: 0.92 k: 0.197 dk: 0.0668 | HW Unweighted R2: 0.92 k: 0.201 dk: 0.0668

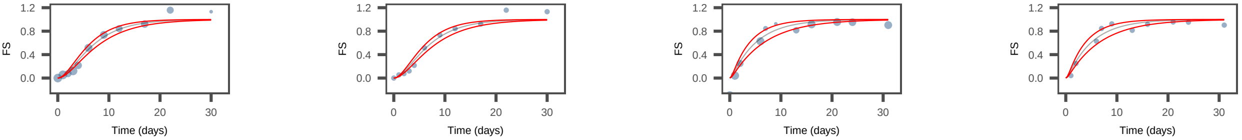

TCPQ

AA Weighted R2: 0.98 k: 0.192 dk: 0.0412 | AA Unweighted R2: 0.98 k: 0.192 dk: 0.0414 | HW Weighted R2: 0.94 k: 0.136 dk: 0.036 | HW Unweighted R2: 0.94 k: 0.137 dk: 0.036

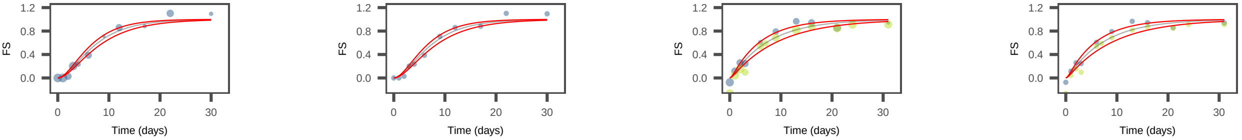

TCTP

AA Weighted R2: 0.95 k: 0.26 dk: 0.0873 | AA Unweighted R2: 0.95 k: 0.254 dk: 0.0881 | HW Weighted R2: 0.97 k: 0.283 dk: 0.0513 | HW Unweighted R2: 0.97 k: 0.282 dk: 0.051

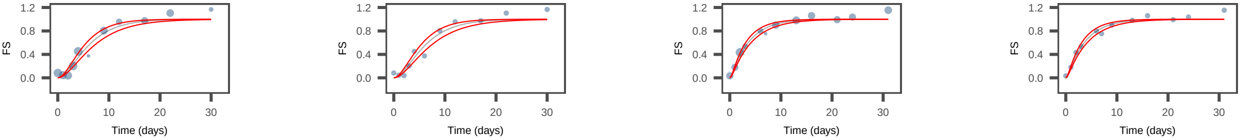

TELT

AA Weighted R2: 0.92 k: 64.2 dk: 1440 | AA Unweighted R2: 0.92 k: 63.1 dk: 1390 | HW Weighted R2: 0.94 k: 1.66 dk: 0.449 | HW Unweighted R2: 0.94 k: 1.65 dk: 0.446

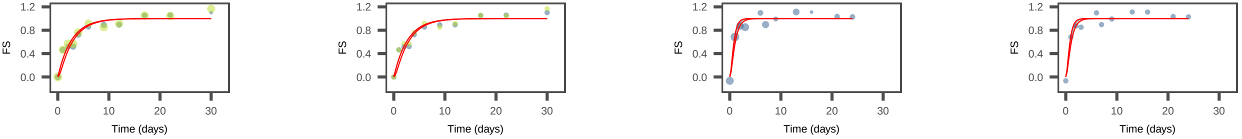

TERA

AA Weighted R2: 0.92 k: 0.223 dk: 0.0822 | AA Unweighted R2: 0.91 k: 0.218 dk: 0.0859 | HW Weighted R2: 0.95 k: 0.2 dk: 0.0483 | HW Unweighted R2: 0.95 k: 0.198 dk: 0.0486

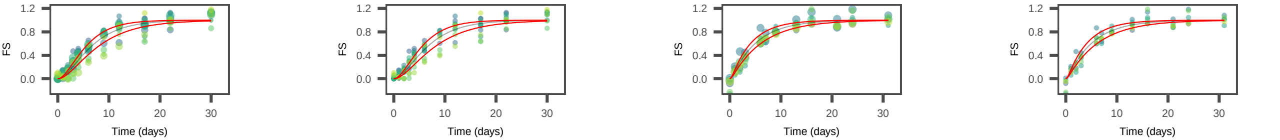

TGM2

AA Weighted R2: 0.95 k: 0.0838 dk: 0.0184 | AA Unweighted R2: 0.94 k: 0.079 dk: 0.0181 | HW Weighted R2: 0.94 k: 0.061 dk: 0.0122 | HW Unweighted R2: 0.94 k: 0.0599 dk: 0.0119

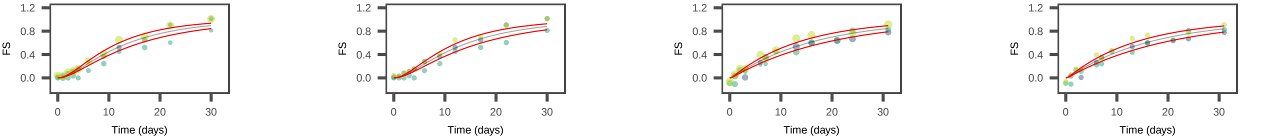

THIL

AA Weighted R2: 0.96 k: 0.0464 dk: 0.00619 | AA Unweighted R2: 0.93 k: 0.043 dk: 0.00776 | HW Weighted R2: 0.95 k: 0.0298 dk: 0.00387 | HW Unweighted R2: 0.95 k: 0.0297 dk: 0.00382

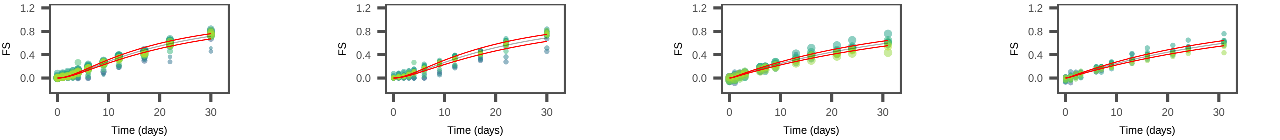

THIM

AA Weighted R2: 0.96 k: 0.0379 dk: 0.00454 | AA Unweighted R2: 0.95 k: 0.0368 dk: 0.00492 | HW Weighted R2: 0.97 k: 0.0262 dk: 0.00254 | HW Unweighted R2: 0.96 k: 0.0263 dk: 0.00271

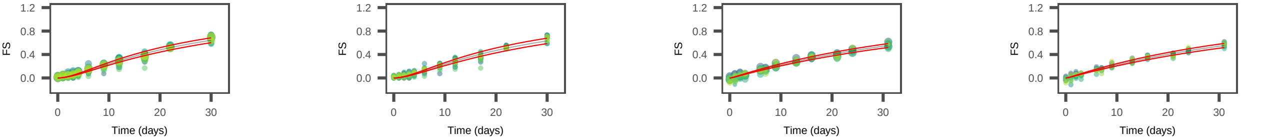

THIO

AA Weighted R2: 0.93 k: 0.0785 dk: 0.0159 | AA Unweighted R2: 0.93 k: 0.0783 dk: 0.0157 | HW Weighted R2: 0.91 k: 0.0462 dk: 0.00994 | HW Unweighted R2: 0.91 k: 0.0459 dk: 0.00991

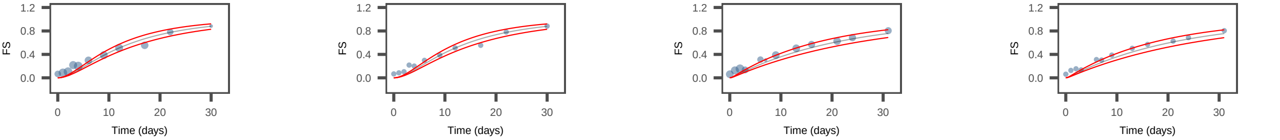

TIM44

AA Weighted R2: 0.98 k: 0.077 dk: 0.00939 | AA Unweighted R2: 0.98 k: 0.0754 dk: 0.0091 | HW Weighted R2: 0.95 k: 0.0633 dk: 0.0125 | HW Unweighted R2: 0.95 k: 0.0635 dk: 0.0123

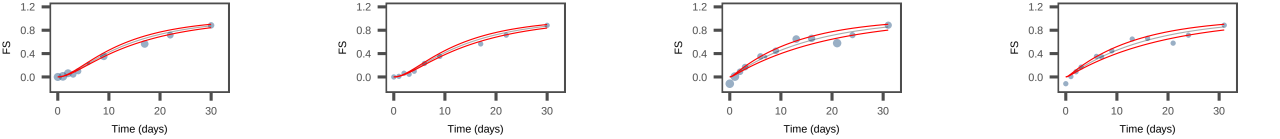

TIM50

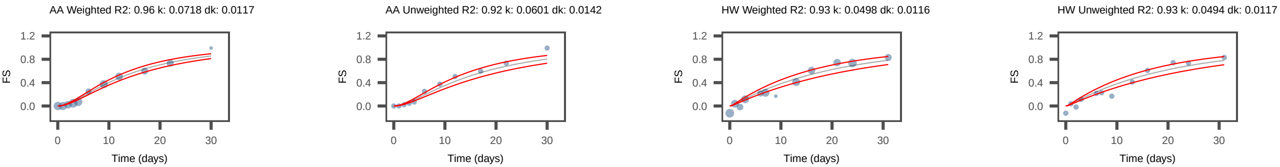

TITIN

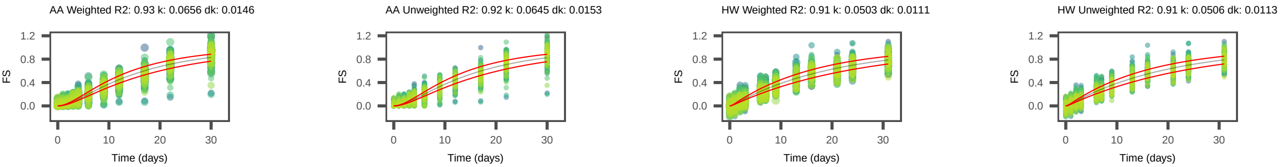

TLN1

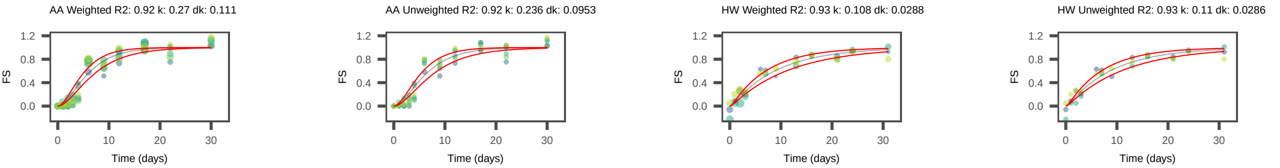

TMOD1

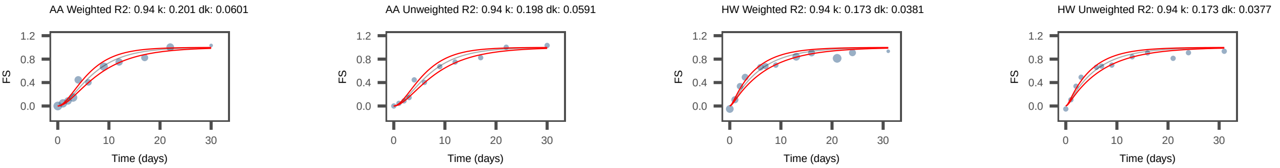

TNNC1

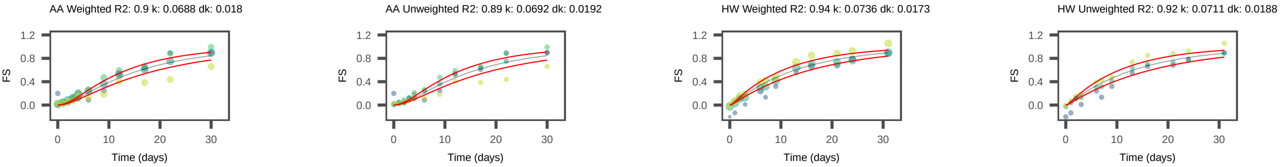

TNNI3

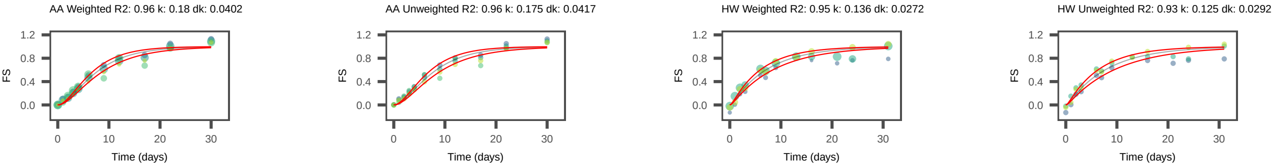

TNNT2

AA Weighted R2: 0.96 k: 0.164 dk: 0.0372
AA Unweighted R2: 0.96 k: 0.158 dk: 0.0376
HW Weighted R2: 0.9 k: 0.0931 dk: 0.0272
HW Unweighted R2: 0.9 k: 0.0973 dk: 0.028

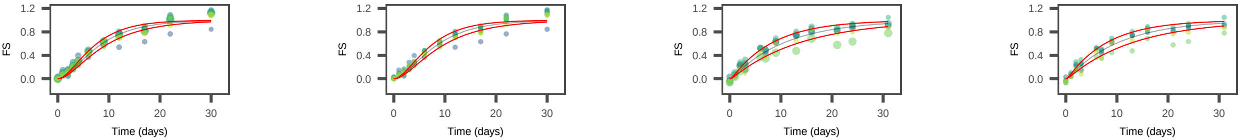

TOM40

AA Weighted R2: 0.78 k: 0.0303 dk: 0.00851
AA Unweighted R2: 0.76 k: 0.0284 dk: 0.00837
HW Weighted R2: 0.91 k: 0.0357 dk: 0.00776
HW Unweighted R2: 0.91 k: 0.0357 dk: 0.00784

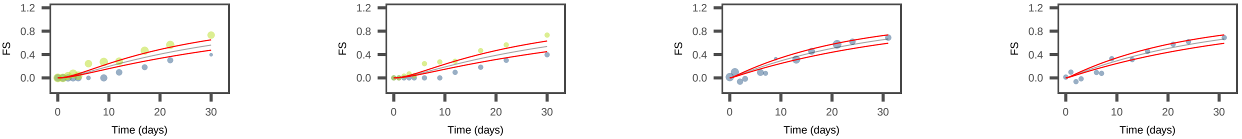

TPIS

AA Weighted R2: 0.97 k: 0.0736 dk: 0.0106
AA Unweighted R2: 0.97 k: 0.0722 dk: 0.0109
HW Weighted R2: 0.96 k: 0.0555 dk: 0.00819
HW Unweighted R2: 0.96 k: 0.0555 dk: 0.00799

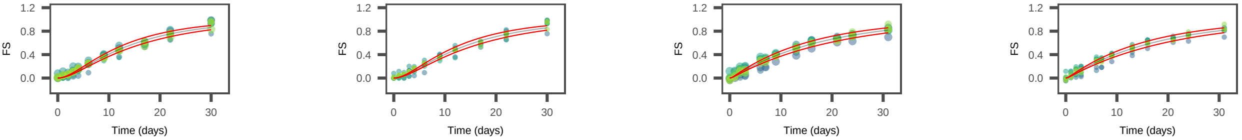

TPM1

AA Weighted R2: 0.98 k: 0.119 dk: 0.0166
AA Unweighted R2: 0.98 k: 0.119 dk: 0.0168
HW Weighted R2: 0.96 k: 0.0954 dk: 0.0178
HW Unweighted R2: 0.96 k: 0.0934 dk: 0.0171

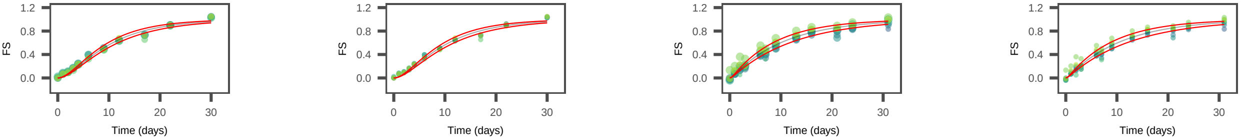

TRFE

AA Weighted R2: 0.89 k: 18.7 dk: 176
AA Unweighted R2: 0.88 k: 18.9 dk: 182
HW Weighted R2: 0.94 k: 0.364 dk: 0.0837
HW Unweighted R2: 0.94 k: 0.365 dk: 0.0835

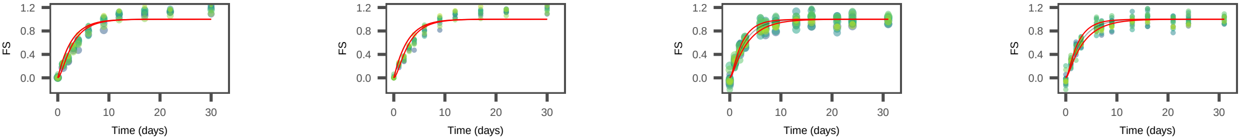

TRI72

AA Weighted R2: 0.94 k: 0.212 dk: 0.0638
AA Unweighted R2: 0.94 k: 0.19 dk: 0.0559
HW Weighted R2: 0.99 k: 0.109 dk: 0.0112
HW Unweighted R2: 0.99 k: 0.109 dk: 0.0112

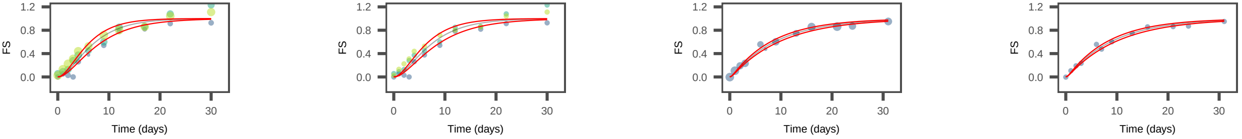

TTHY

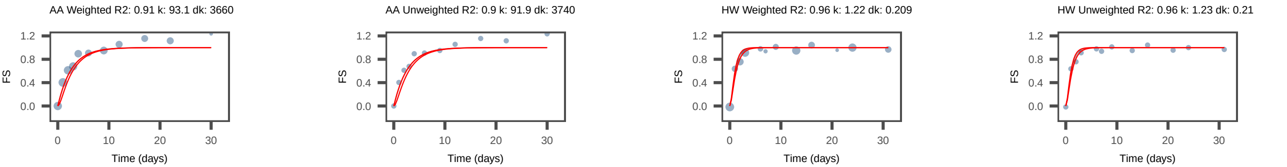

UBA1

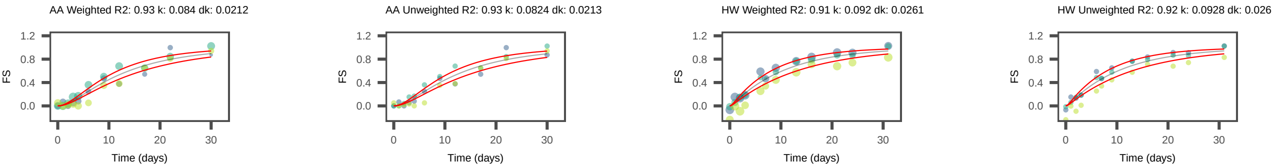

UBE2N

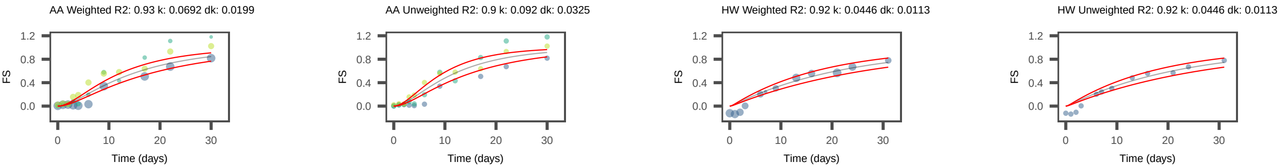

UCRI

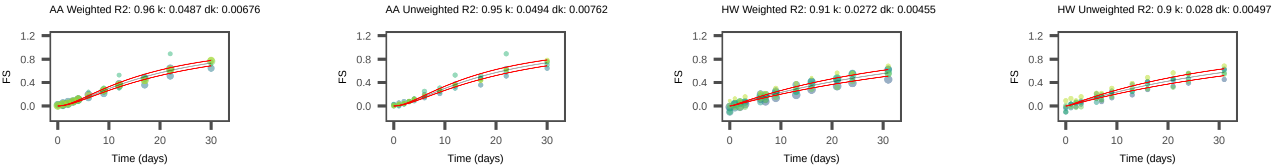

UGPA

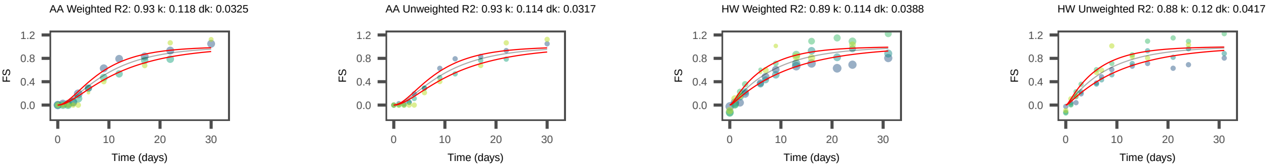

VDAC1

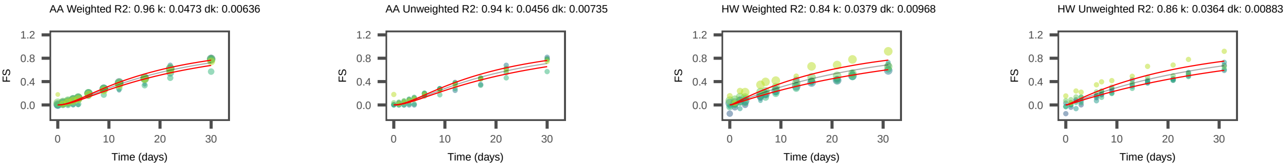

VDAC2

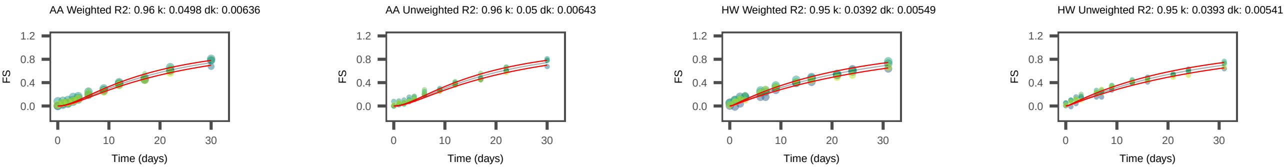

VDAC3

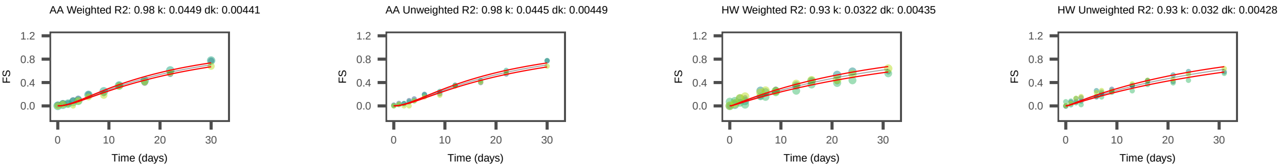

VIME

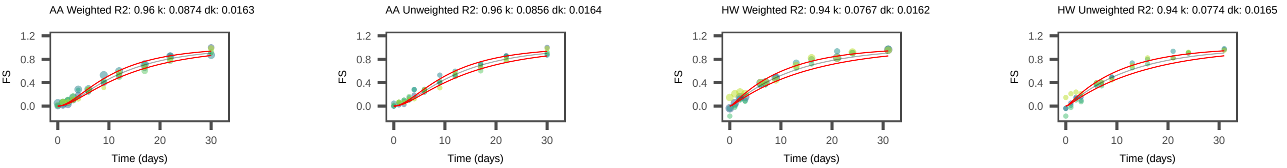

VINC

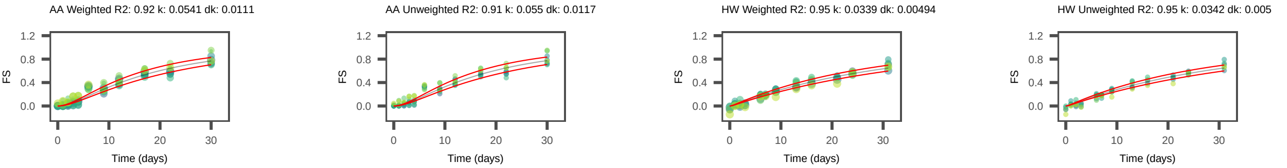

VWA8

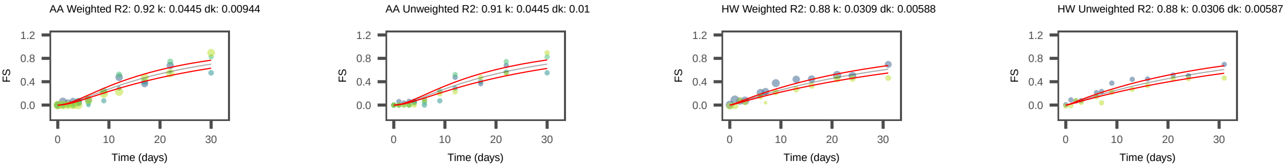

WDR1

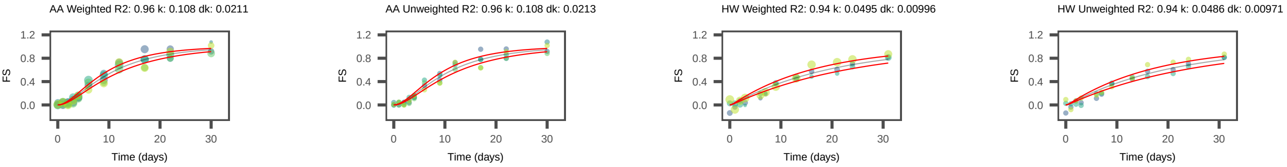

Supplement: Supplemental Data S6 [file mmc7.pdf]
