# Supplementary material for: Harmonizing Labeling and Analytical Strategies to Obtain Protein Turnover Rates in Intact Adult Animals
Source: Mol Cell Proteomics. 2022 May 28;21(7):100252. doi: 10.1016/j.mcpro.2022.100252 (PMC9249856; doi:10.1016/j.mcpro.2022.100252)

1433B

AA Weighted R2: 0.93 k: 0.369 dk: 0.137

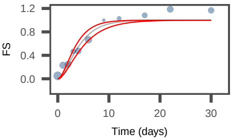

AA Unweighted R2: 0.93 k: 0.382 dk: 0.142

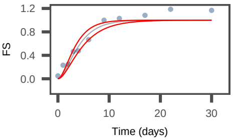

HW Weighted R2: 0.96 k: 0.257 dk: 0.0511

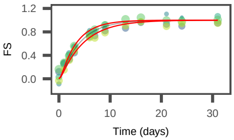

HW Unweighted R2: 0.96 k: 0.259 dk: 0.0509

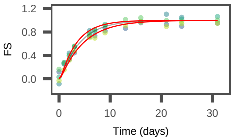

1433E

AA Weighted R2: 0.96 k: 0.242 dk: 0.0547

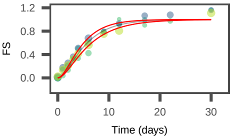

AA Unweighted R2: 0.96 k: 0.244 dk: 0.0564

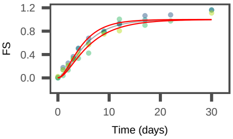

HW Weighted R2: 0.95 k: 0.202 dk: 0.0464

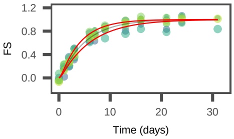

HW Unweighted R2: 0.95 k: 0.204 dk: 0.047

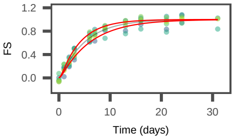

1433G

AA Weighted R2: 0.91 k: 0.197 dk: 0.0751

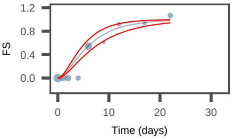

AA Unweighted R2: 0.9 k: 0.181 dk: 0.0719

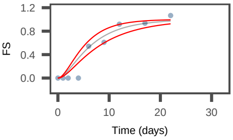

HW Weighted R2: 0.98 k: 0.208 dk: 0.0293

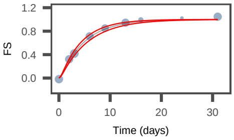

HW Unweighted R2: 0.98 k: 0.207 dk: 0.032

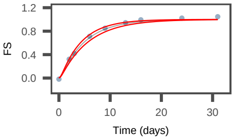

1433T

AA Weighted R2: 0.93 k: 0.231 dk: 0.0702

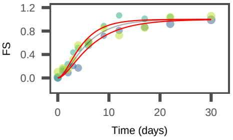

AA Unweighted R2: 0.92 k: 0.235 dk: 0.0788

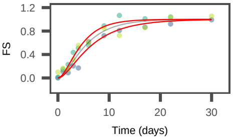

HW Weighted R2: 0.94 k: 0.289 dk: 0.0754

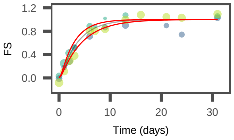

HW Unweighted R2: 0.93 k: 0.295 dk: 0.0782

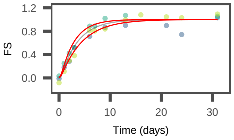

1433Z

AA Weighted R2: 0.95 k: 0.259 dk: 0.0654

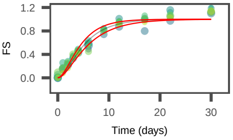

AA Unweighted R2: 0.95 k: 0.259 dk: 0.0661

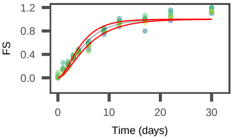

HW Weighted R2: 0.98 k: 0.222 dk: 0.0276

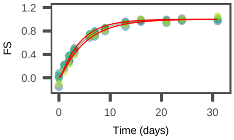

HW Unweighted R2: 0.98 k: 0.222 dk: 0.0277

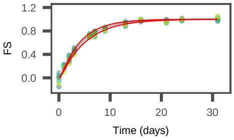

2AAA

AA Weighted R2: 0.91 k: 0.685 dk: 0.325

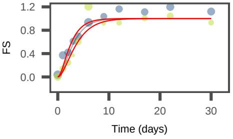

AA Unweighted R2: 0.92 k: 0.625 dk: 0.274

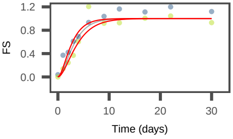

HW Weighted R2: 0.98 k: 0.303 dk: 0.0412

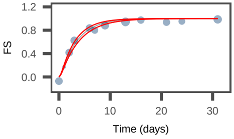

HW Unweighted R2: 0.98 k: 0.303 dk: 0.0408

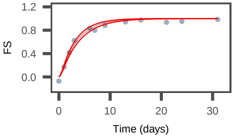

3BHS4

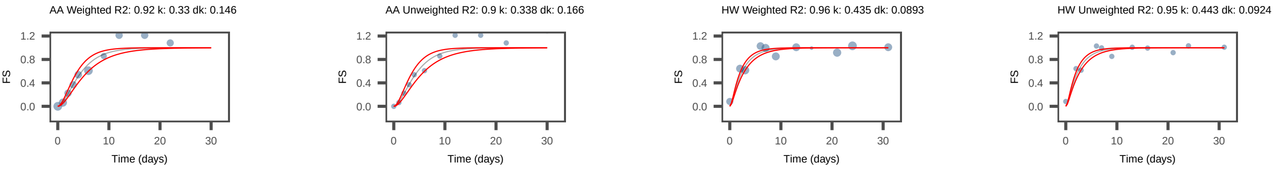

3HIDH

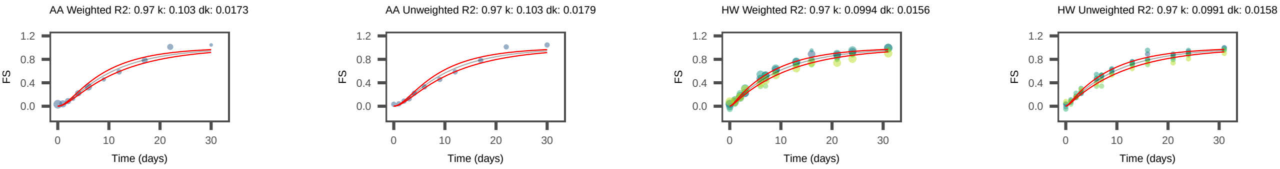

4F2

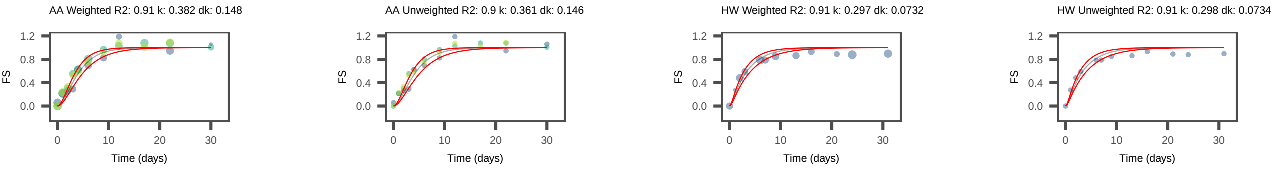

6PGD

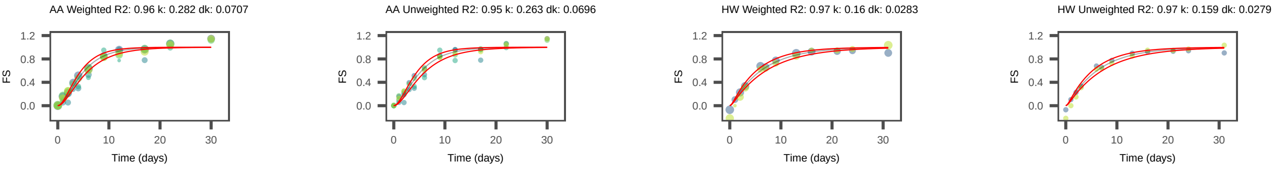

6PGL

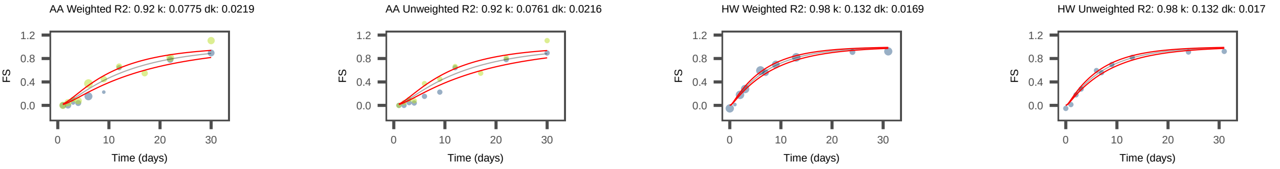

AADAT

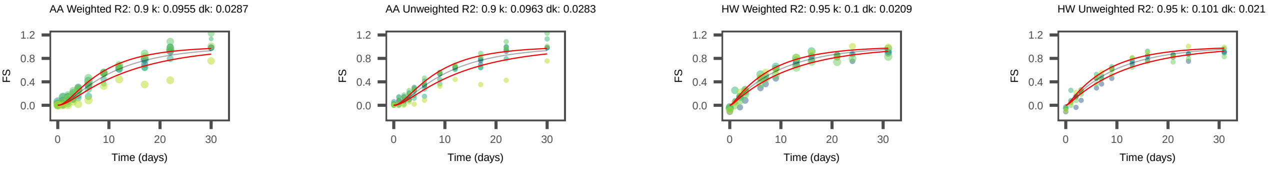

AASS

AA Weighted R2: 0.92 k: 0.221 dk: 0.0716 | AA Unweighted R2: 0.91 k: 0.22 dk: 0.0745 | HW Weighted R2: 0.92 k: 0.144 dk: 0.0384 | HW Unweighted R2: 0.92 k: 0.139 dk: 0.0365

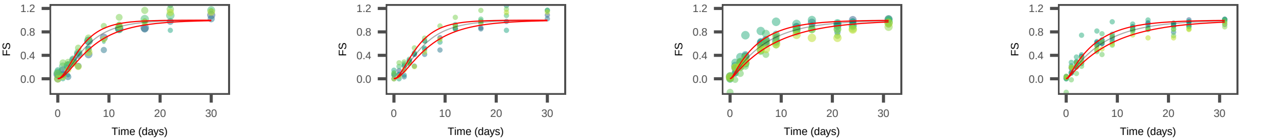

AATC

AA Weighted R2: 0.94 k: 0.139 dk: 0.0387 | AA Unweighted R2: 0.94 k: 0.14 dk: 0.0395 | HW Weighted R2: 0.94 k: 0.127 dk: 0.0305 | HW Unweighted R2: 0.93 k: 0.127 dk: 0.0328

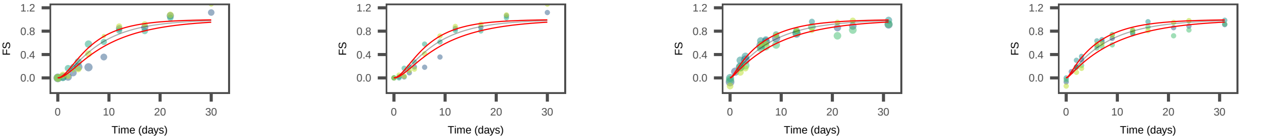

AATM

AA Weighted R2: 0.93 k: 0.114 dk: 0.0288 | AA Unweighted R2: 0.92 k: 0.103 dk: 0.0283 | HW Weighted R2: 0.94 k: 0.095 dk: 0.0206 | HW Unweighted R2: 0.94 k: 0.0935 dk: 0.0199

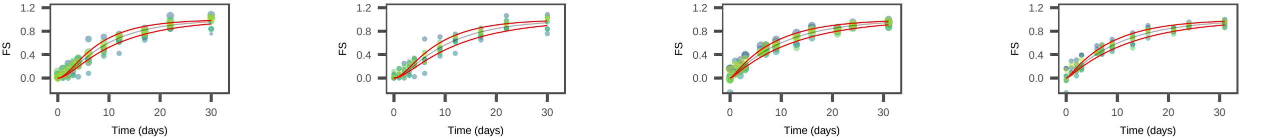

ABCB7

AA Weighted R2: 0.95 k: 0.151 dk: 0.0364 | AA Unweighted R2: 0.95 k: 0.151 dk: 0.036 | HW Weighted R2: 0.86 k: 0.0911 dk: 0.0329 | HW Unweighted R2: 0.85 k: 0.0934 dk: 0.0343

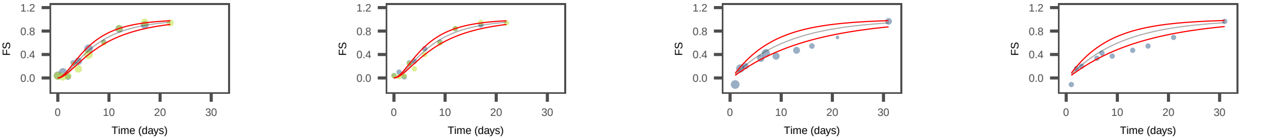

ABCD3

AA Weighted R2: 0.91 k: 0.171 dk: 0.056 | AA Unweighted R2: 0.91 k: 0.195 dk: 0.0665 | HW Weighted R2: 0.96 k: 0.212 dk: 0.0443 | HW Unweighted R2: 0.96 k: 0.221 dk: 0.0475

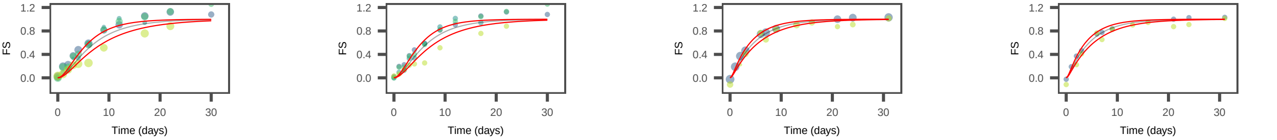

ABCG2

AA Weighted R2: 0.97 k: 0.388 dk: 0.0886 | AA Unweighted R2: 0.97 k: 0.378 dk: 0.0875 | HW Weighted R2: 0.99 k: 0.266 dk: 0.025 | HW Unweighted R2: 0.99 k: 0.266 dk: 0.025

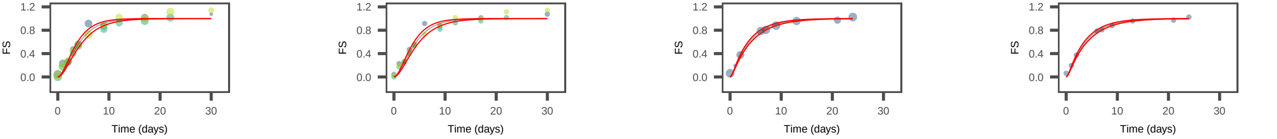

ABHDB

AA Weighted R2: 0.98 k: 0.0969 dk: 0.0129 | AA Unweighted R2: 0.98 k: 0.0967 dk: 0.0128 | HW Weighted R2: 0.96 k: 0.101 dk: 0.0185 | HW Unweighted R2: 0.96 k: 0.101 dk: 0.0189

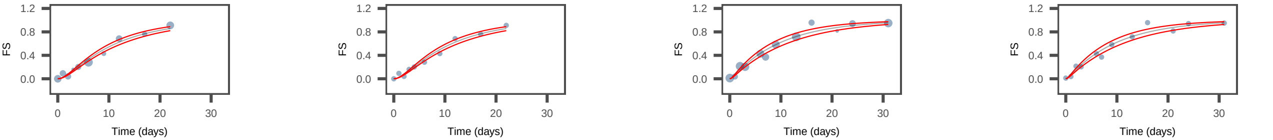

ABHEB

AA Weighted R2: 0.95 k: 0.26 dk: 0.0735 | AA Unweighted R2: 0.95 k: 0.264 dk: 0.0727 | HW Weighted R2: 0.98 k: 0.196 dk: 0.0261 | HW Unweighted R2: 0.98 k: 0.197 dk: 0.0265

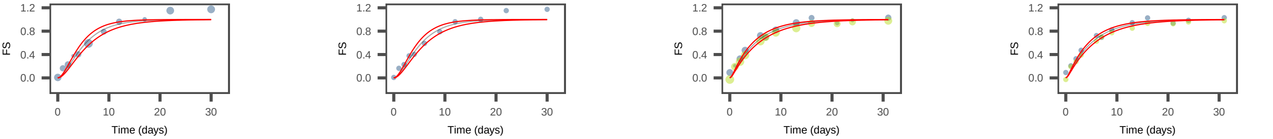

ACADL

AA Weighted R2: 0.95 k: 0.118 dk: 0.0275 | AA Unweighted R2: 0.94 k: 0.118 dk: 0.0303 | HW Weighted R2: 0.97 k: 0.104 dk: 0.0157 | HW Unweighted R2: 0.97 k: 0.103 dk: 0.0154

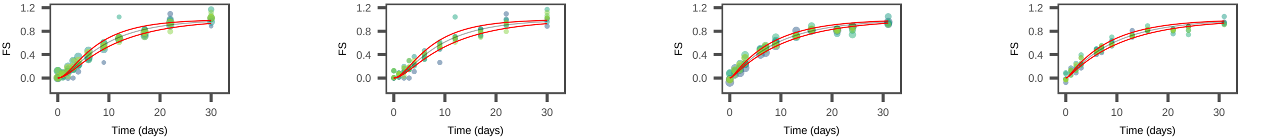

ACADM

AA Weighted R2: 0.96 k: 0.161 dk: 0.0358 | AA Unweighted R2: 0.94 k: 0.152 dk: 0.0388 | HW Weighted R2: 0.97 k: 0.152 dk: 0.0255 | HW Unweighted R2: 0.96 k: 0.152 dk: 0.0258

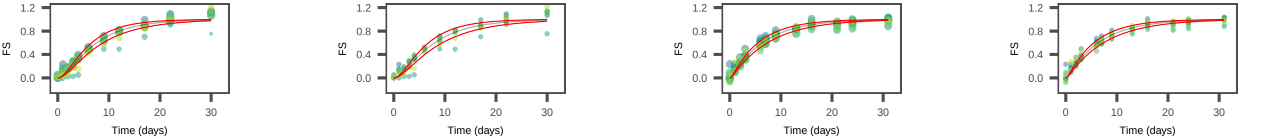

ACADS

AA Weighted R2: 0.95 k: 0.133 dk: 0.0318 | AA Unweighted R2: 0.95 k: 0.126 dk: 0.0315 | HW Weighted R2: 0.95 k: 0.118 dk: 0.0244 | HW Unweighted R2: 0.93 k: 0.122 dk: 0.0284

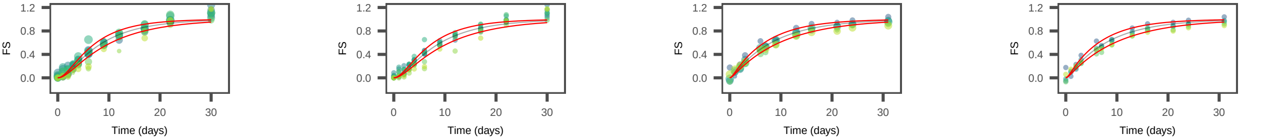

ACADV

AA Weighted R2: 0.95 k: 0.215 dk: 0.0531 | AA Unweighted R2: 0.94 k: 0.209 dk: 0.0573 | HW Weighted R2: 0.9 k: 0.168 dk: 0.0514 | HW Unweighted R2: 0.9 k: 0.178 dk: 0.0532

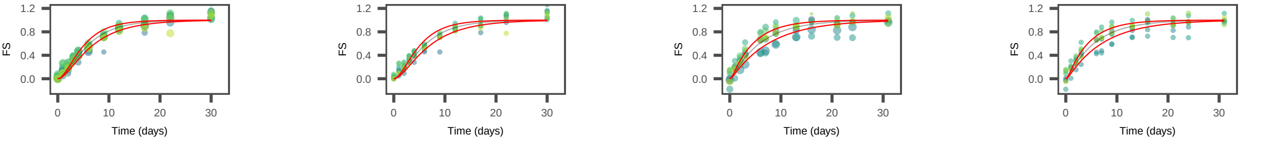

ACBP

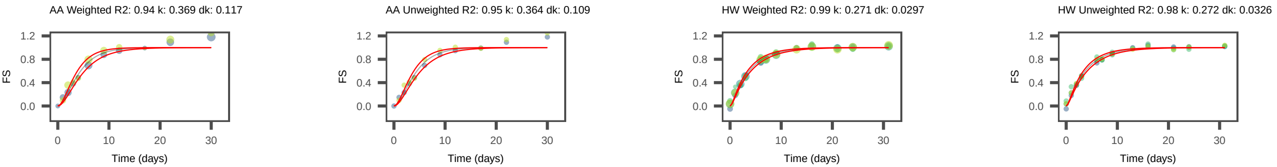

ACD10

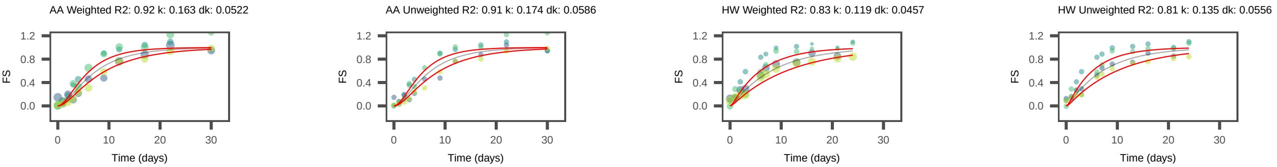

ACD11

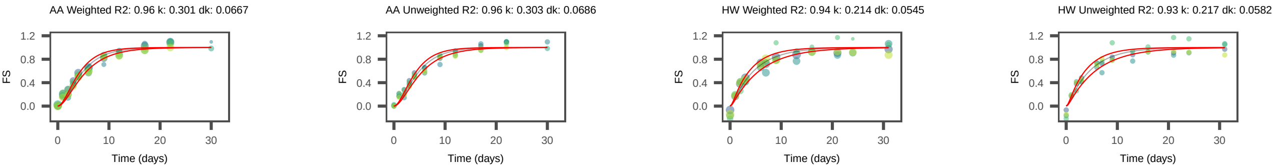

ACDSB

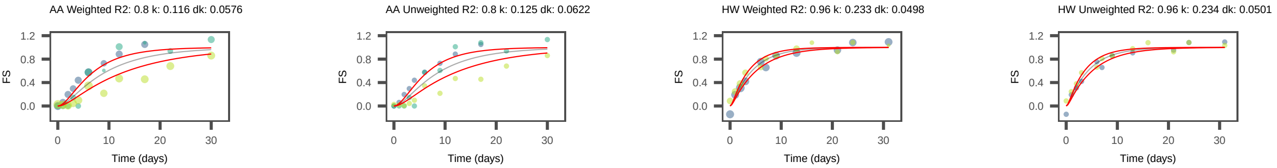

ACLY

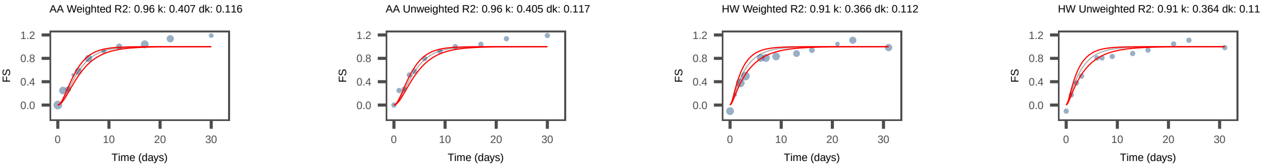

ACO12

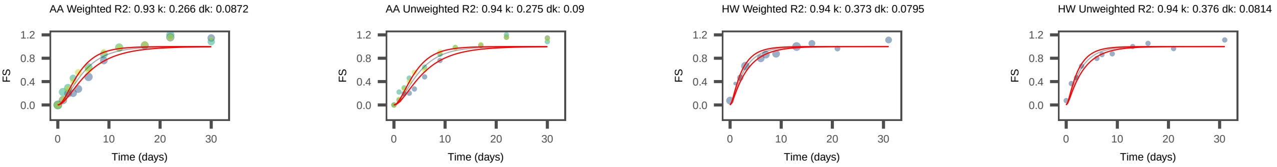

ACO13

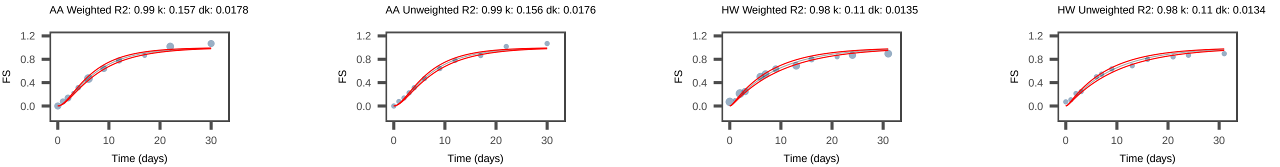

ACOC

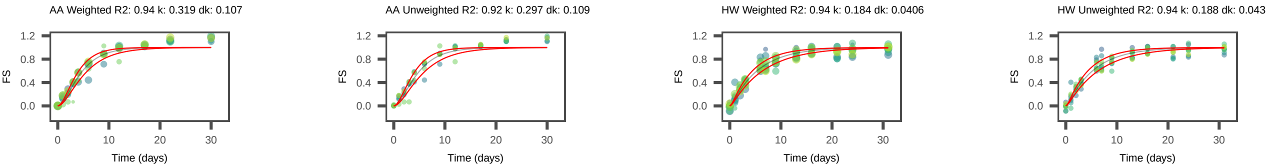

ACON

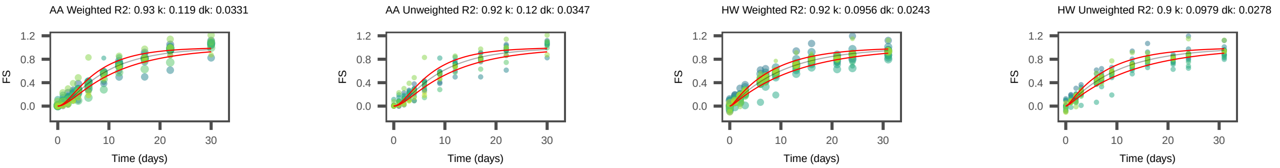

ACOT9

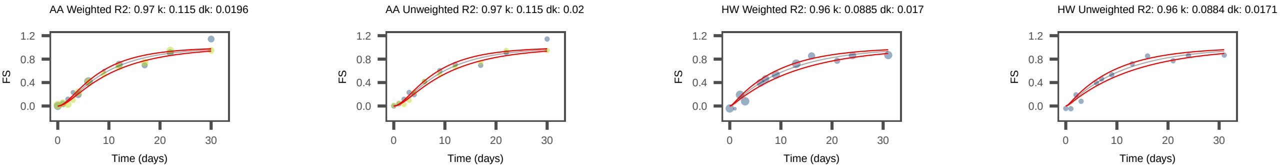

ACOX1

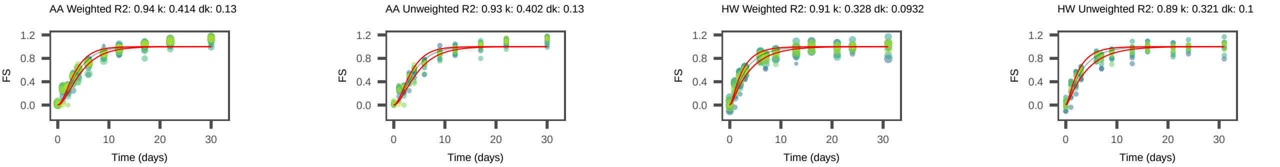

ACOX3

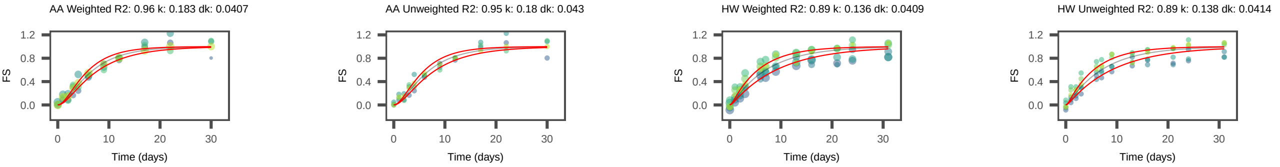

ACPM

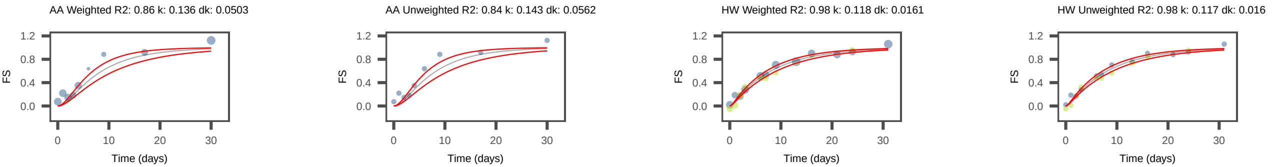

ACSA

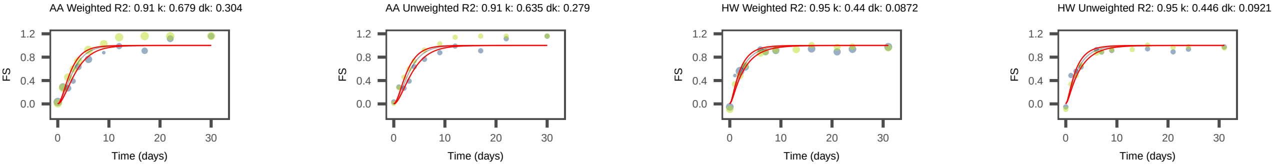

ACSM1

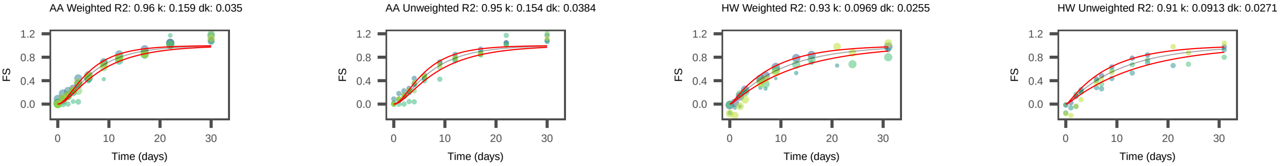

ACSM2

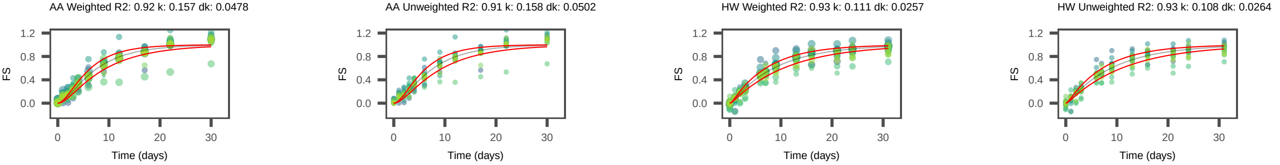

ACSM3

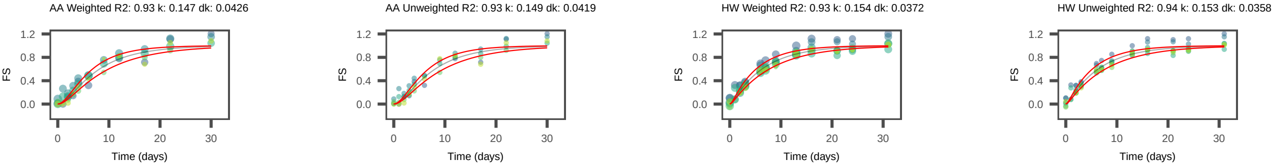

ACTN1

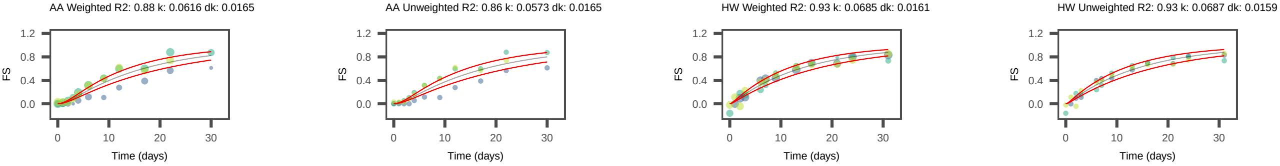

ACTN4

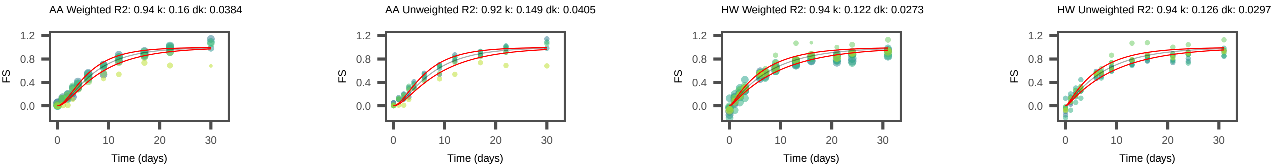

ACY2

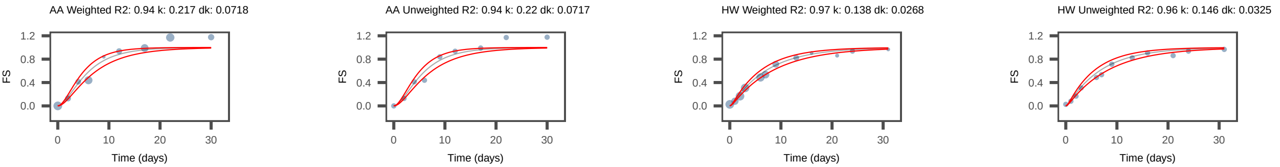

ACY3

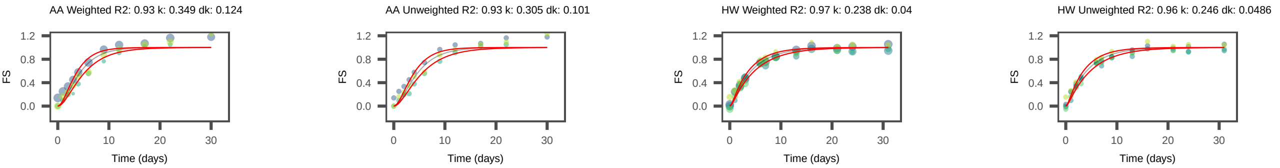

ADH1

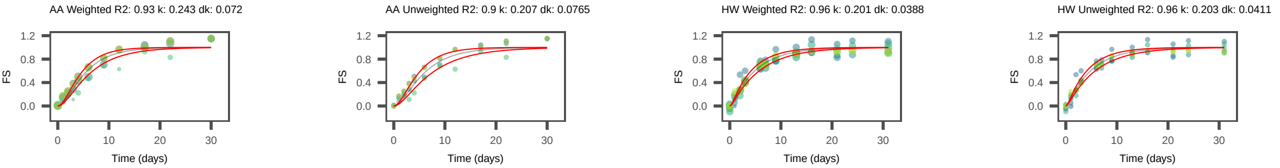

ADK

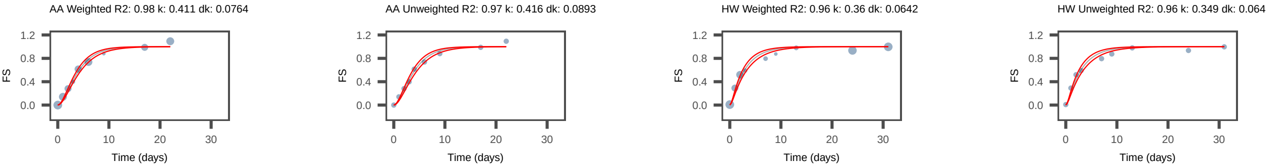

ADT1

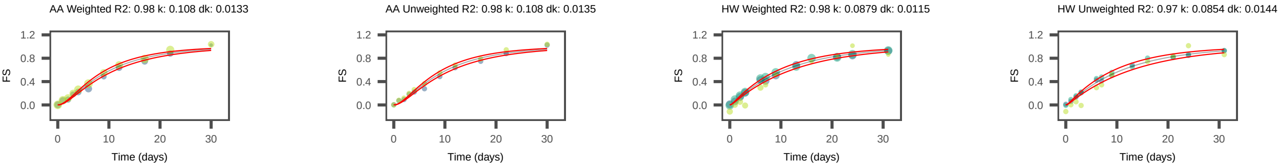

ADT2

AA Weighted R2: 0.97 k: 0.115 dk: 0.0191

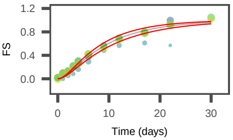

AA Unweighted R2: 0.94 k: 0.106 dk: 0.0231

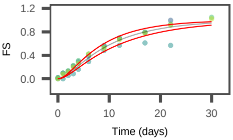

HW Weighted R2: 0.98 k: 0.0916 dk: 0.012

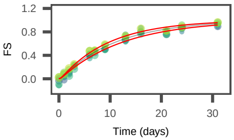

HW Unweighted R2: 0.98 k: 0.0904 dk: 0.0121

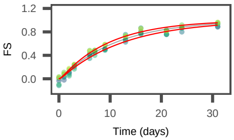

AFG1L

AA Weighted R2: 0.99 k: 0.147 dk: 0.016

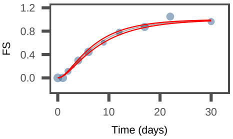

AA Unweighted R2: 0.99 k: 0.146 dk: 0.0158

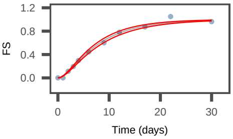

HW Weighted R2: 0.95 k: 0.283 dk: 0.0808

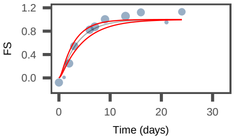

HW Unweighted R2: 0.95 k: 0.28 dk: 0.0813

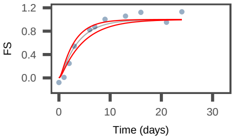

AGT2

AA Weighted R2: 0.88 k: 0.187 dk: 0.0839

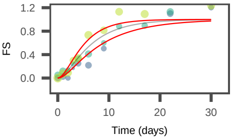

AA Unweighted R2: 0.89 k: 0.171 dk: 0.0694

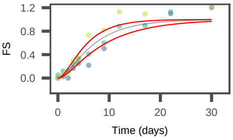

HW Weighted R2: 0.97 k: 0.108 dk: 0.0151

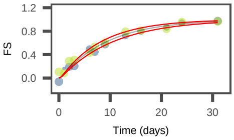

HW Unweighted R2: 0.98 k: 0.108 dk: 0.0151

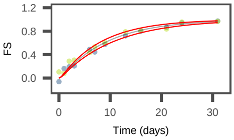

AIFM1

AA Weighted R2: 0.94 k: 0.0988 dk: 0.0235

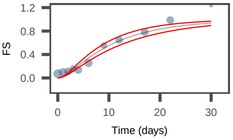

AA Unweighted R2: 0.93 k: 0.1 dk: 0.0253

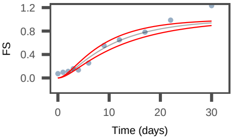

HW Weighted R2: 0.91 k: 0.103 dk: 0.0278

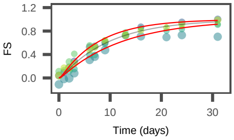

HW Unweighted R2: 0.91 k: 0.107 dk: 0.0289

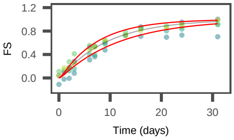

AK1A1

AA Weighted R2: 0.96 k: 0.253 dk: 0.06

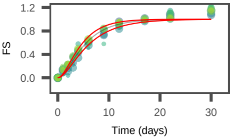

AA Unweighted R2: 0.96 k: 0.252 dk: 0.0657

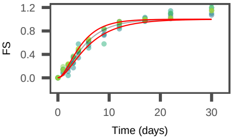

HW Weighted R2: 0.95 k: 0.192 dk: 0.04

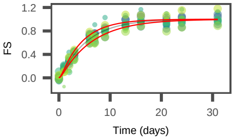

HW Unweighted R2: 0.95 k: 0.193 dk: 0.0437

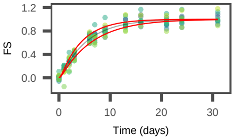

AK1CL

AA Weighted R2: 0.95 k: 0.518 dk: 0.169

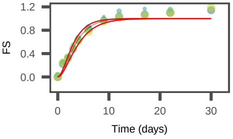

AA Unweighted R2: 0.94 k: 0.466 dk: 0.166

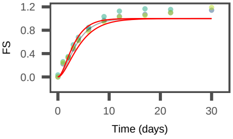

HW Weighted R2: 0.97 k: 0.3 dk: 0.0492

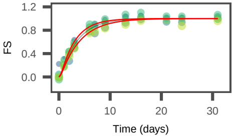

HW Unweighted R2: 0.97 k: 0.307 dk: 0.0514

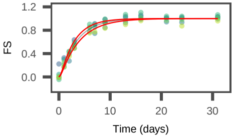

AL1L1

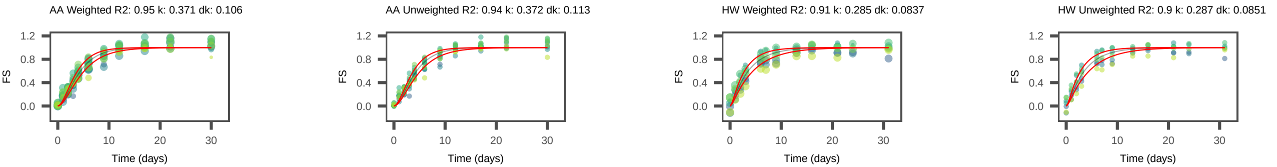

AL3A2

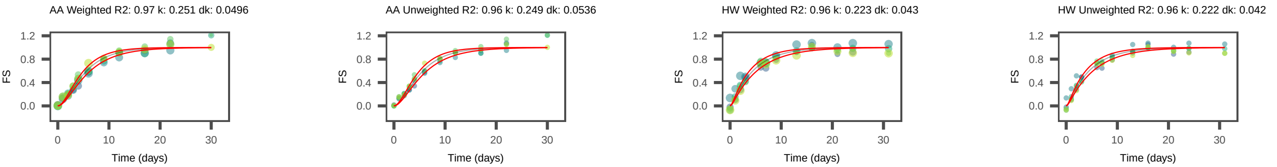

AL4A1

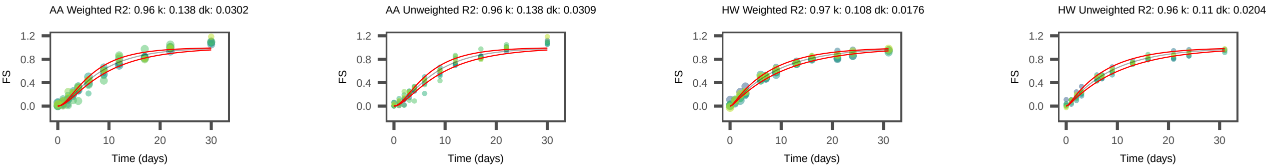

AL7A1

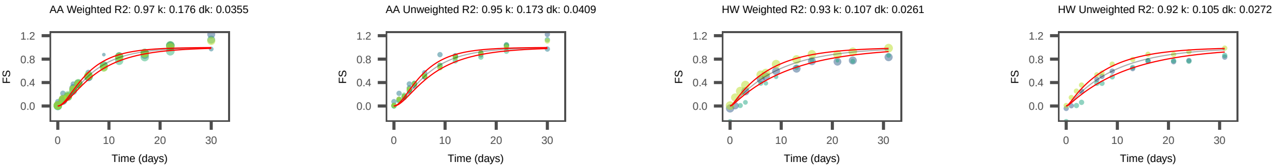

AL8A1

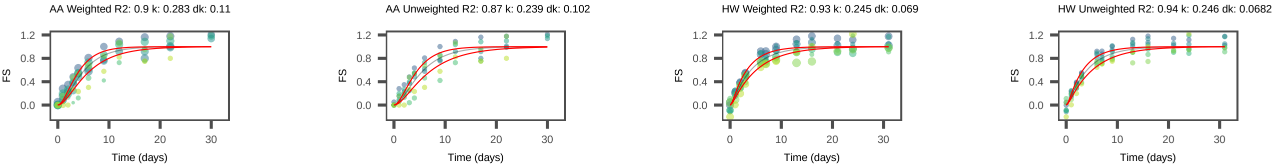

AL9A1

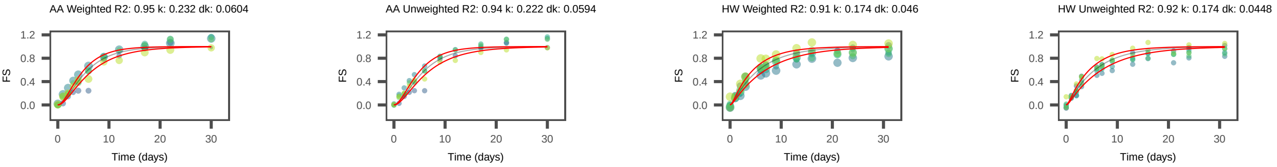

ALBU

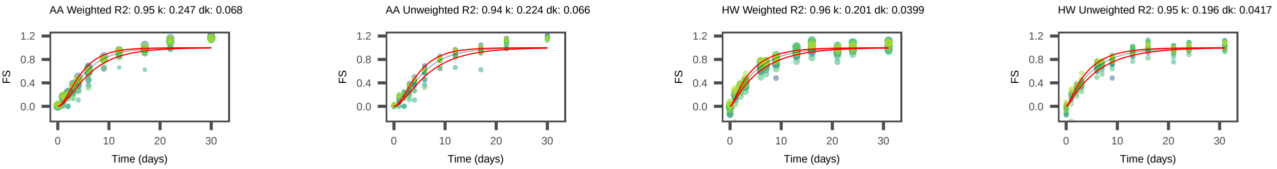

ALDH2

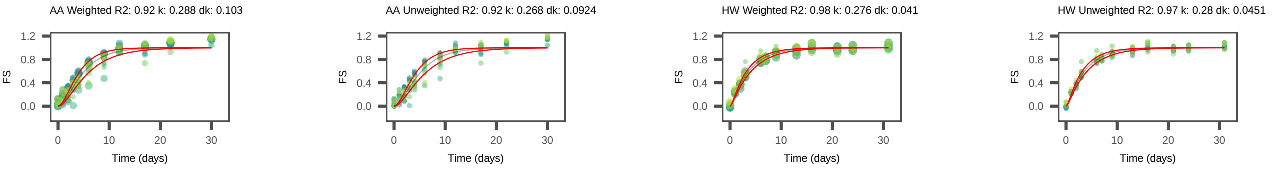

ALDOA

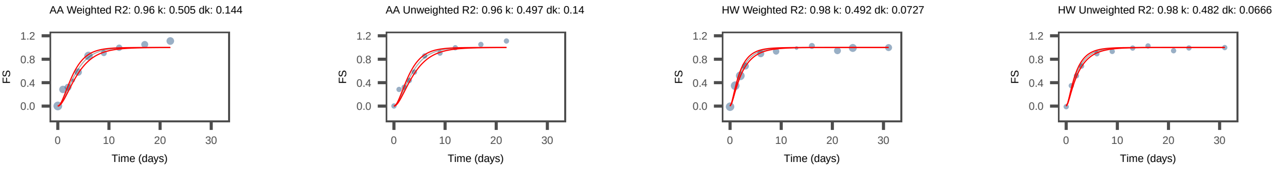

ALDOB

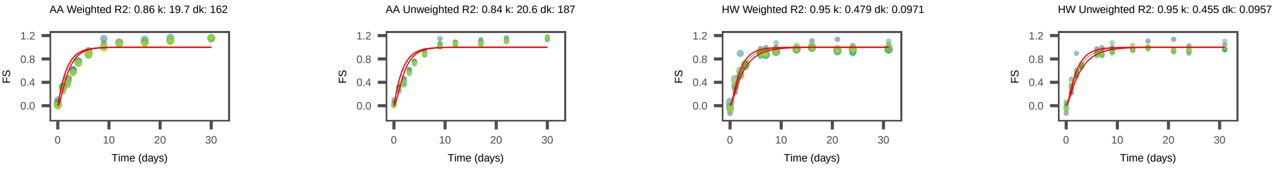

AMACR

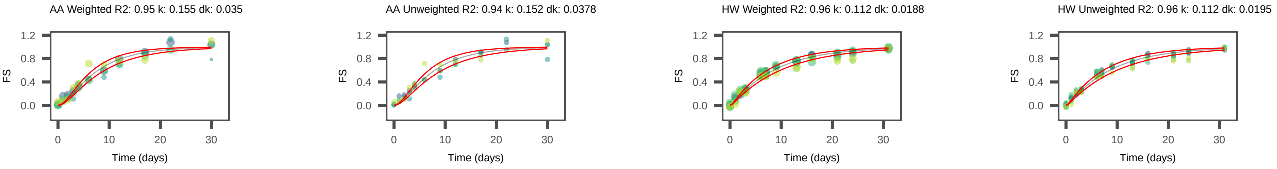

AMPE

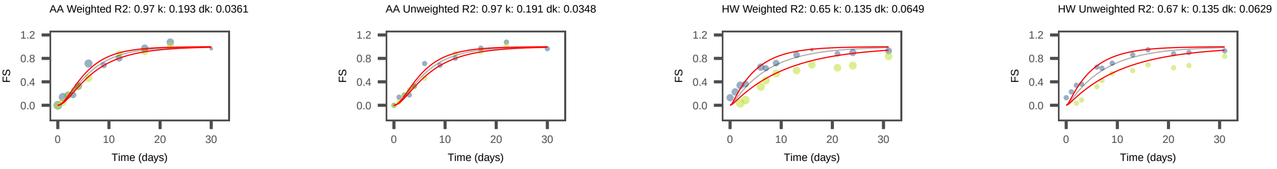

AMPL

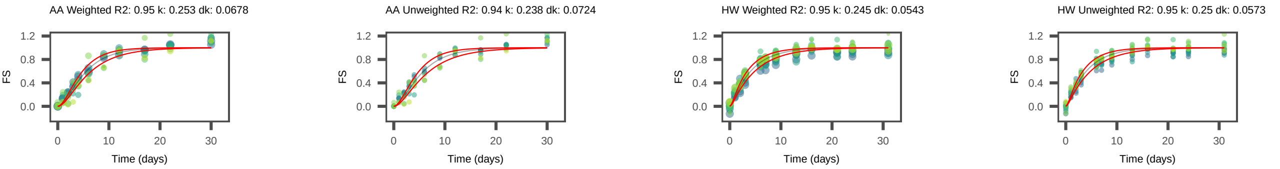

AMPN

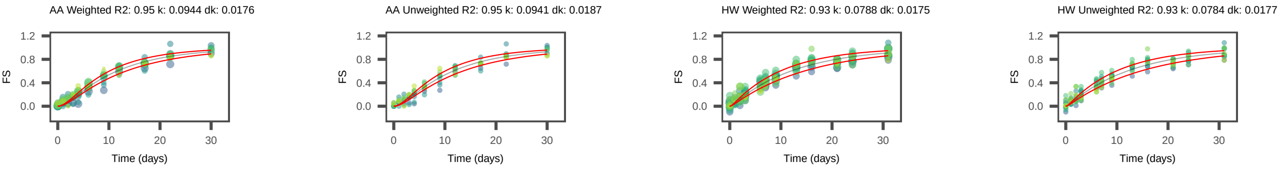

ANXA1

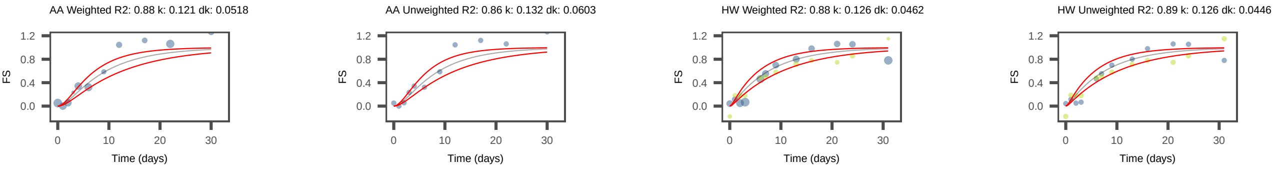

ANXA2

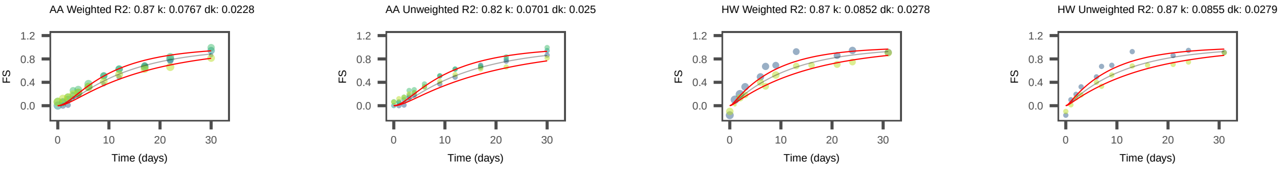

ANXA5

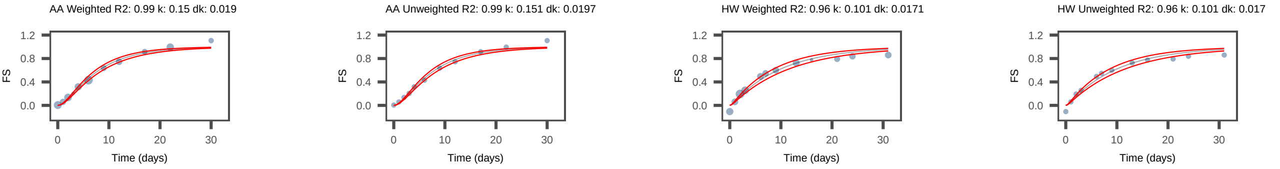

AP1B1

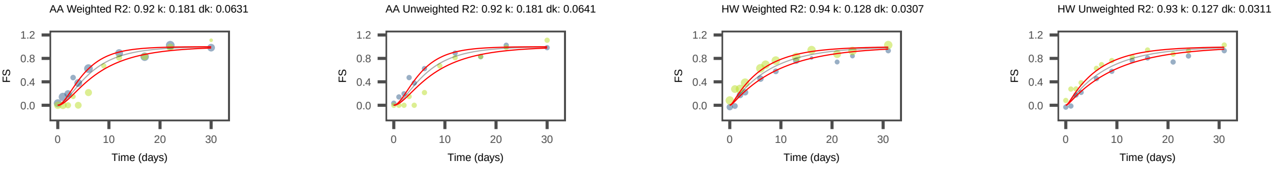

AP1G1

AA Weighted R2: 0.96 k: 0.287 dk: 0.0645

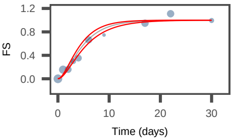

AA Unweighted R2: 0.96 k: 0.274 dk: 0.0638

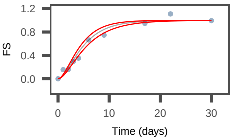

HW Weighted R2: 0.9 k: 0.198 dk: 0.0634

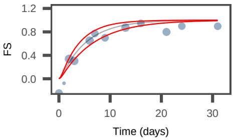

HW Unweighted R2: 0.9 k: 0.198 dk: 0.0636

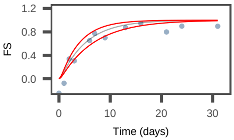

AP2A2

AA Weighted R2: 0.93 k: 0.349 dk: 0.114

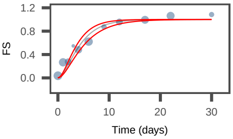

AA Unweighted R2: 0.93 k: 0.364 dk: 0.118

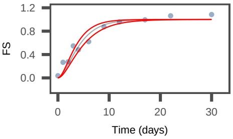

HW Weighted R2: 0.92 k: 0.214 dk: 0.0642

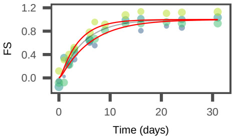

HW Unweighted R2: 0.92 k: 0.21 dk: 0.0646

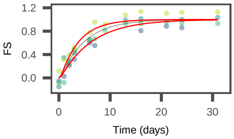

AP2B1

AA Weighted R2: 0.94 k: 0.161 dk: 0.0382

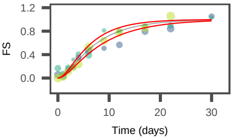

AA Unweighted R2: 0.93 k: 0.156 dk: 0.0384

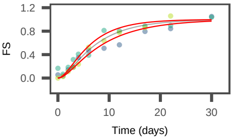

HW Weighted R2: 0.94 k: 0.109 dk: 0.0275

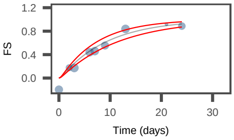

HW Unweighted R2: 0.94 k: 0.111 dk: 0.0285

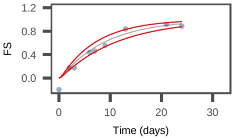

AP2M1

AA Weighted R2: 0.97 k: 0.288 dk: 0.0625

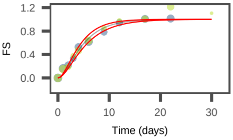

AA Unweighted R2: 0.97 k: 0.29 dk: 0.0624

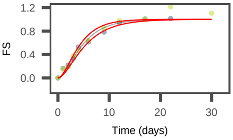

HW Weighted R2: 0.92 k: 0.12 dk: 0.0399

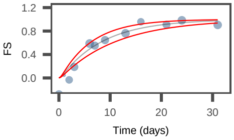

HW Unweighted R2: 0.92 k: 0.12 dk: 0.0396

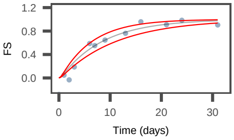

APOA1

AA Weighted R2: 0.96 k: 2.09 dk: 1.18

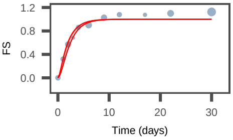

AA Unweighted R2: 0.96 k: 2.06 dk: 1.07

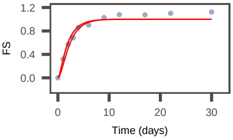

HW Weighted R2: 0.89 k: 0.538 dk: 0.162

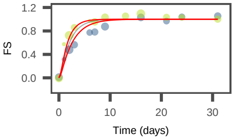

HW Unweighted R2: 0.89 k: 0.543 dk: 0.165

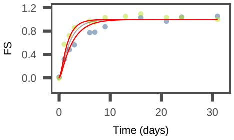

ARC1A

AA Weighted R2: 0.98 k: 0.195 dk: 0.036

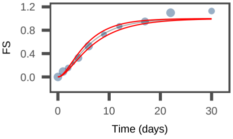

AA Unweighted R2: 0.98 k: 0.195 dk: 0.0353

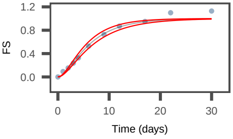

HW Weighted R2: 0.94 k: 0.141 dk: 0.0331

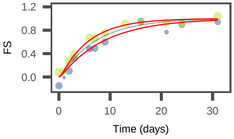

HW Unweighted R2: 0.94 k: 0.139 dk: 0.033

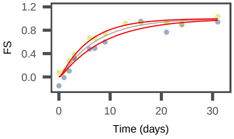

ARLY

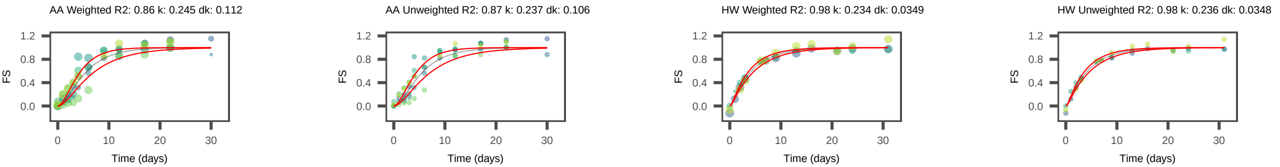

ARP2

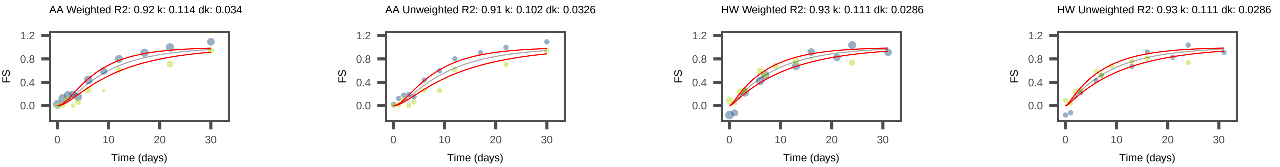

ARPC4

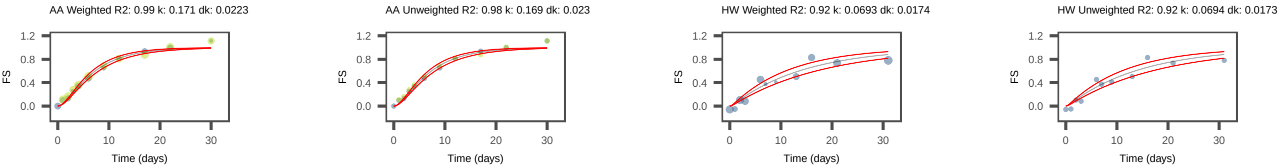

ASSY

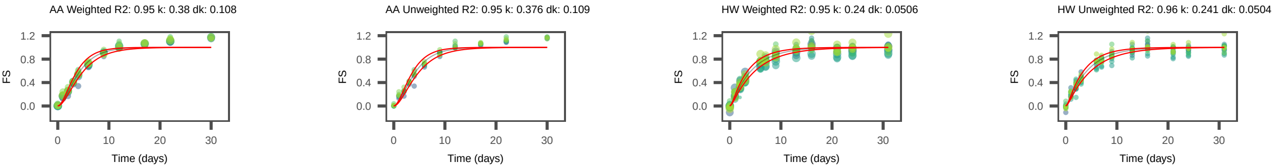

AT1A1

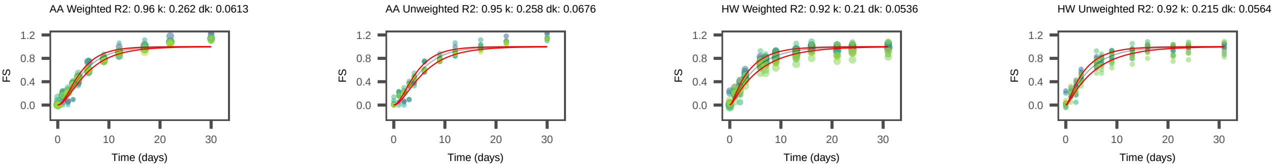

AT1B1

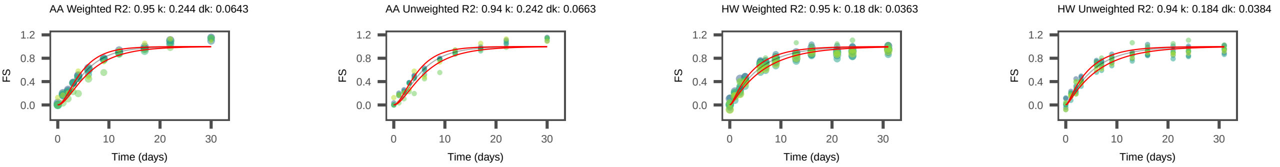

AT2A2

AA Weighted R2: 0.89 k: 0.405 dk: 0.18

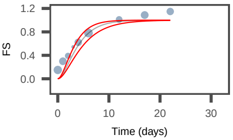

AA Unweighted R2: 0.89 k: 0.394 dk: 0.176

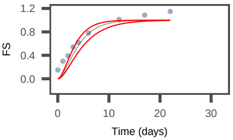

HW Weighted R2: 0.93 k: 0.47 dk: 0.143

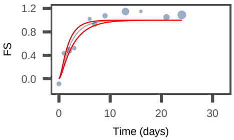

HW Unweighted R2: 0.93 k: 0.471 dk: 0.144

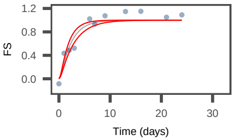

AT5F1

AA Weighted R2: 0.9 k: 0.0828 dk: 0.0238

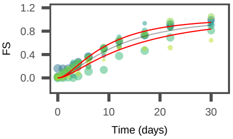

AA Unweighted R2: 0.9 k: 0.0829 dk: 0.0234

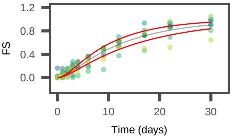

HW Weighted R2: 0.98 k: 0.0826 dk: 0.0107

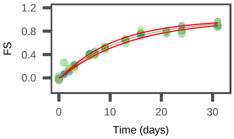

HW Unweighted R2: 0.98 k: 0.0831 dk: 0.0102

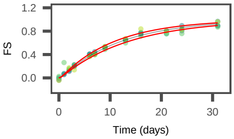

ATNG

AA Weighted R2: 0.93 k: 0.856 dk: 0.372

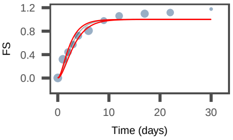

AA Unweighted R2: 0.93 k: 0.85 dk: 0.373

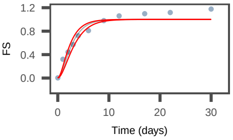

HW Weighted R2: 0.97 k: 0.509 dk: 0.074

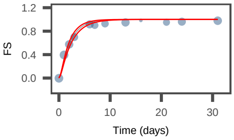

HW Unweighted R2: 0.97 k: 0.507 dk: 0.0735

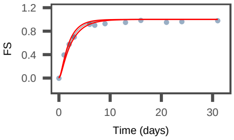

ATP5H

AA Weighted R2: 0.96 k: 0.105 dk: 0.0191

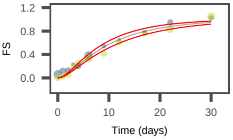

AA Unweighted R2: 0.96 k: 0.105 dk: 0.0189

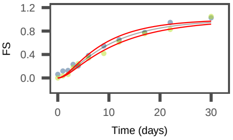

HW Weighted R2: 0.96 k: 0.0739 dk: 0.0122

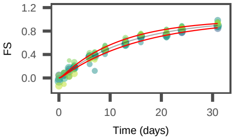

HW Unweighted R2: 0.96 k: 0.0744 dk: 0.0133

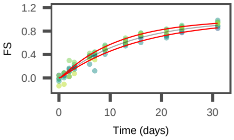

ATP5I

AA Weighted R2: 0.98 k: 0.0934 dk: 0.0137

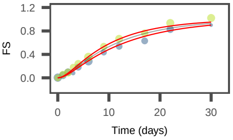

AA Unweighted R2: 0.98 k: 0.0924 dk: 0.0136

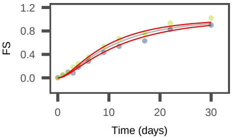

HW Weighted R2: 0.96 k: 0.0694 dk: 0.0108

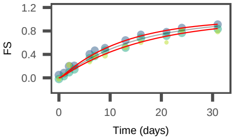

HW Unweighted R2: 0.96 k: 0.0681 dk: 0.011

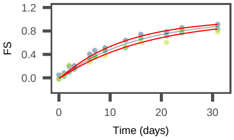

ATP5L

AA Weighted R2: 0.96 k: 0.104 dk: 0.0188

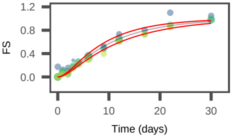

AA Unweighted R2: 0.96 k: 0.105 dk: 0.0203

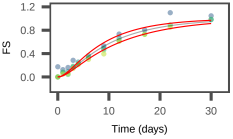

HW Weighted R2: 0.98 k: 0.0786 dk: 0.00985

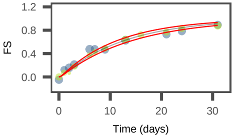

HW Unweighted R2: 0.98 k: 0.0784 dk: 0.00967

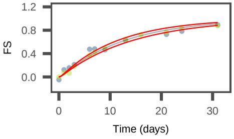

ATPA

AA Weighted R2: 0.98 k: 0.125 dk: 0.0175
AA Unweighted R2: 0.98 k: 0.12 dk: 0.0177
HW Weighted R2: 0.96 k: 0.104 dk: 0.0194
HW Unweighted R2: 0.95 k: 0.104 dk: 0.0207

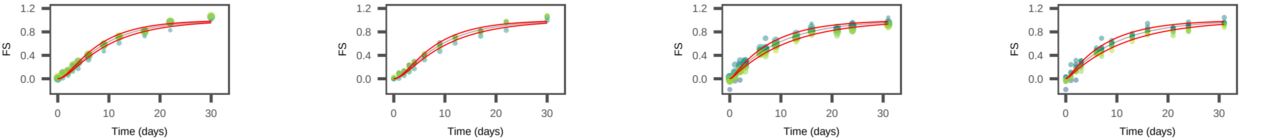

ATPB

AA Weighted R2: 0.97 k: 0.117 dk: 0.0196
AA Unweighted R2: 0.96 k: 0.112 dk: 0.0234
HW Weighted R2: 0.94 k: 0.101 dk: 0.0225
HW Unweighted R2: 0.92 k: 0.106 dk: 0.027

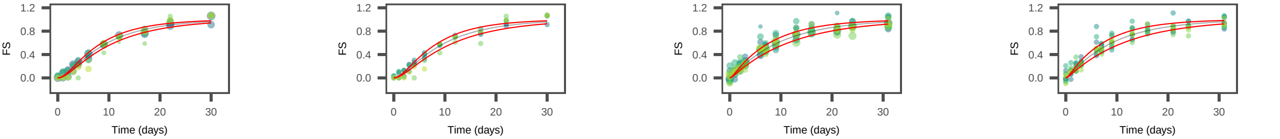

ATPD

AA Weighted R2: 0.96 k: 0.0967 dk: 0.018
AA Unweighted R2: 0.96 k: 0.0958 dk: 0.0182
HW Weighted R2: 0.97 k: 0.0874 dk: 0.0133
HW Unweighted R2: 0.96 k: 0.0884 dk: 0.0139

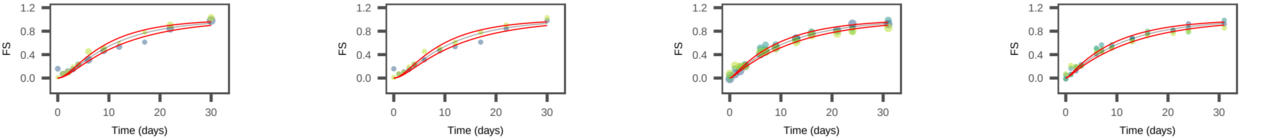

ATPG

AA Weighted R2: 0.98 k: 0.107 dk: 0.0153
AA Unweighted R2: 0.98 k: 0.108 dk: 0.016
HW Weighted R2: 0.96 k: 0.089 dk: 0.0143
HW Unweighted R2: 0.96 k: 0.0876 dk: 0.0138

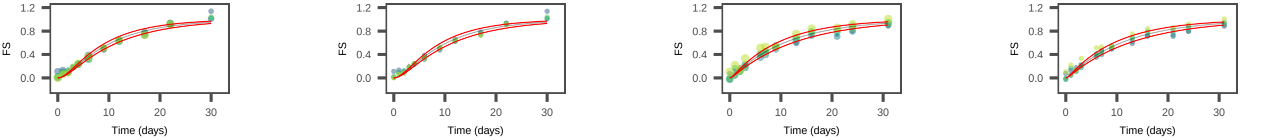

ATPO

AA Weighted R2: 0.98 k: 0.106 dk: 0.0145
AA Unweighted R2: 0.97 k: 0.105 dk: 0.0156
HW Weighted R2: 0.98 k: 0.0756 dk: 0.00942
HW Unweighted R2: 0.97 k: 0.0776 dk: 0.011

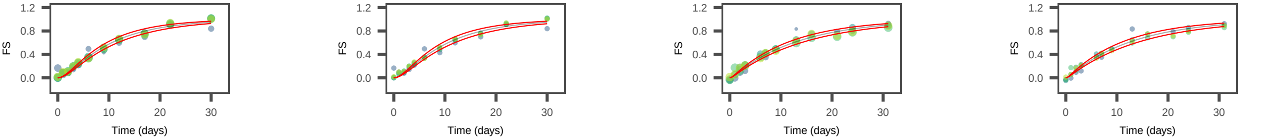

AUHM

AA Weighted R2: 0.99 k: 0.1 dk: 0.00919
AA Unweighted R2: 0.99 k: 0.0999 dk: 0.00917
HW Weighted R2: 0.97 k: 0.0883 dk: 0.0153
HW Unweighted R2: 0.97 k: 0.0883 dk: 0.0155

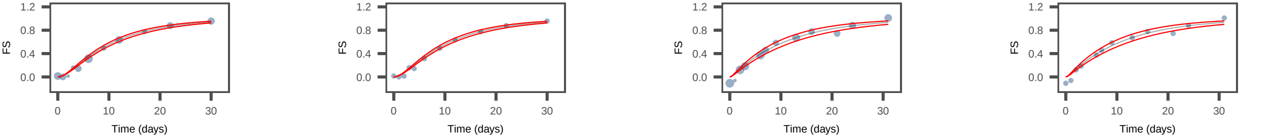

B3AT

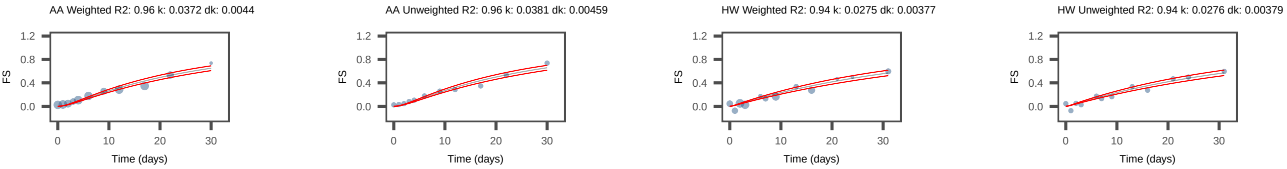

BAP31

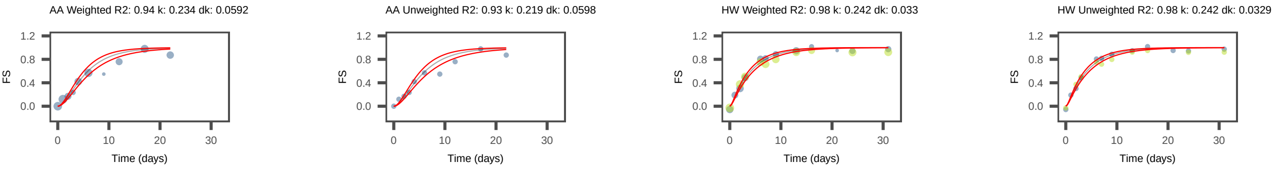

BASI

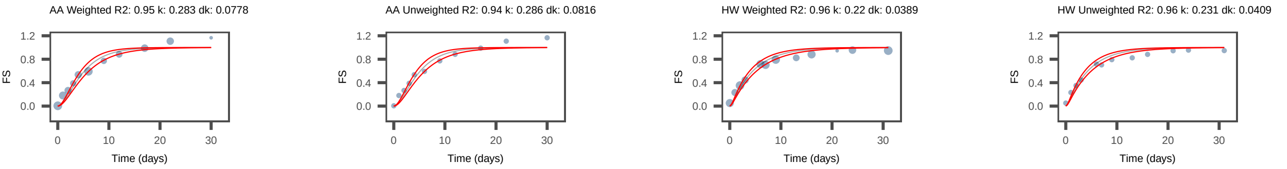

BCAT2

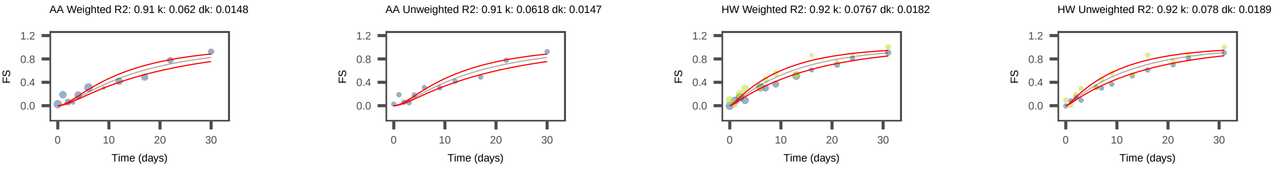

BDH

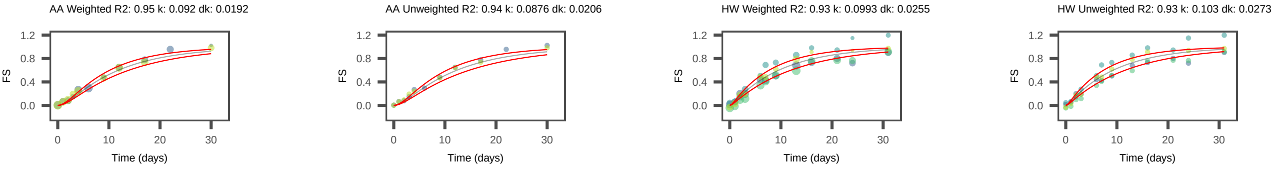

BDH2

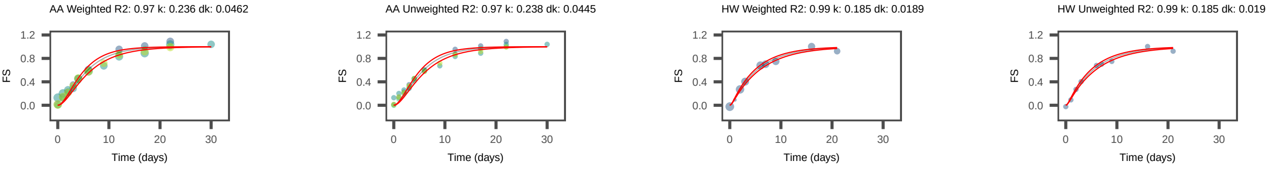

BIEA

AA Weighted R2: 0.91 k: 0.291 dk: 0.11

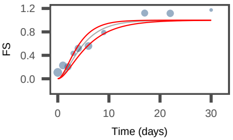

AA Unweighted R2: 0.91 k: 0.294 dk: 0.112

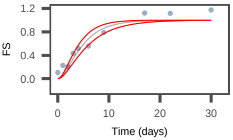

HW Weighted R2: 0.91 k: 0.498 dk: 0.138

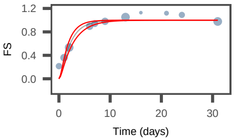

HW Unweighted R2: 0.91 k: 0.499 dk: 0.14

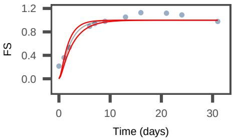

BIP

AA Weighted R2: 0.94 k: 0.439 dk: 0.148

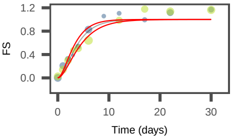

AA Unweighted R2: 0.94 k: 0.445 dk: 0.15

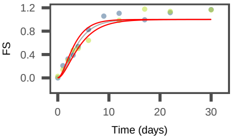

HW Weighted R2: 0.88 k: 0.388 dk: 0.12

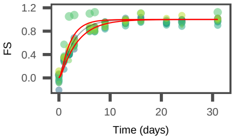

HW Unweighted R2: 0.9 k: 0.362 dk: 0.103

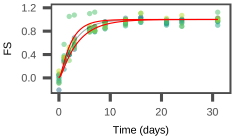

BPHL

AA Weighted R2: 0.97 k: 0.146 dk: 0.0252

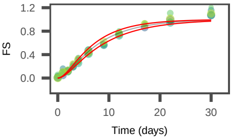

AA Unweighted R2: 0.97 k: 0.147 dk: 0.0272

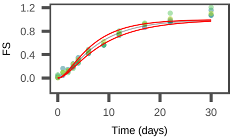

HW Weighted R2: 0.94 k: 0.12 dk: 0.028

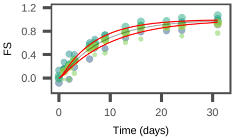

HW Unweighted R2: 0.92 k: 0.116 dk: 0.0306

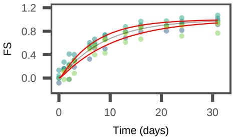

BPNT1

AA Weighted R2: 0.96 k: 0.228 dk: 0.0513

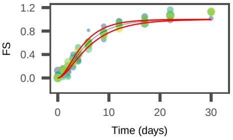

AA Unweighted R2: 0.96 k: 0.23 dk: 0.0556

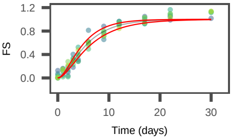

HW Weighted R2: 0.92 k: 0.167 dk: 0.0417

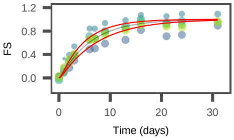

HW Unweighted R2: 0.91 k: 0.176 dk: 0.0466

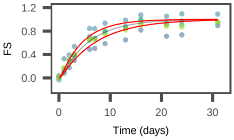

C1TC

AA Weighted R2: 0.95 k: 0.319 dk: 0.0872

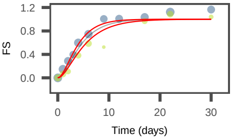

AA Unweighted R2: 0.94 k: 0.292 dk: 0.089

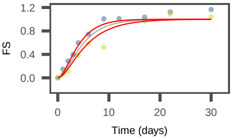

HW Weighted R2: 0.89 k: 0.18 dk: 0.0595

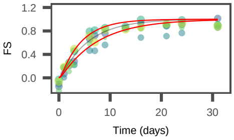

HW Unweighted R2: 0.87 k: 0.176 dk: 0.0621

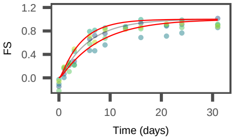

C560

AA Weighted R2: 0.97 k: 0.121 dk: 0.0239

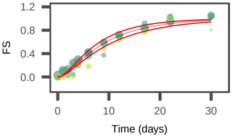

AA Unweighted R2: 0.95 k: 0.112 dk: 0.0256

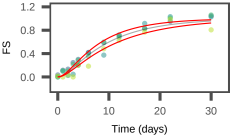

HW Weighted R2: 0.88 k: 0.0732 dk: 0.0238

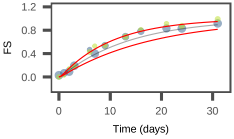

HW Unweighted R2: 0.89 k: 0.0766 dk: 0.0242

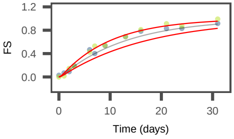

CACP

AA Weighted R2: 0.93 k: 0.105 dk: 0.0268 | AA Unweighted R2: 0.94 k: 0.104 dk: 0.0245 | HW Weighted R2: 0.93 k: 0.0787 dk: 0.0212 | HW Unweighted R2: 0.93 k: 0.0794 dk: 0.0218

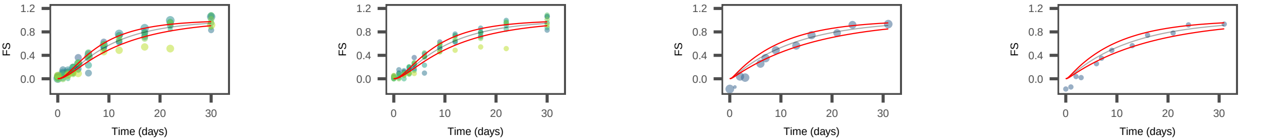

CAD16

AA Weighted R2: 0.93 k: 0.0894 dk: 0.0211 | AA Unweighted R2: 0.93 k: 0.0857 dk: 0.0204 | HW Weighted R2: 0.89 k: 0.0777 dk: 0.0221 | HW Unweighted R2: 0.9 k: 0.0786 dk: 0.0213

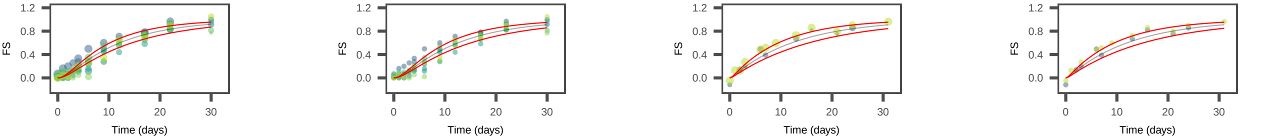

CAH2

AA Weighted R2: 0.93 k: 0.105 dk: 0.0255 | AA Unweighted R2: 0.92 k: 0.101 dk: 0.0263 | HW Weighted R2: 0.76 k: 0.0773 dk: 0.027 | HW Unweighted R2: 0.79 k: 0.0721 dk: 0.0237

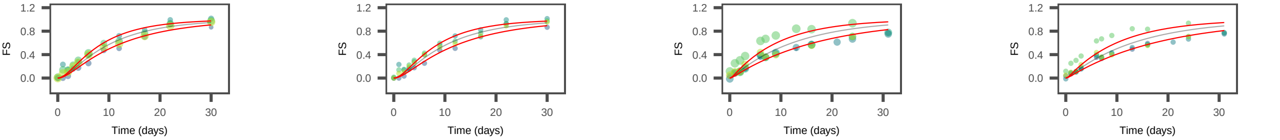

CALB1

AA Weighted R2: 0.95 k: 0.147 dk: 0.0388 | AA Unweighted R2: 0.96 k: 0.145 dk: 0.0379 | HW Weighted R2: 0.91 k: 0.132 dk: 0.0362 | HW Unweighted R2: 0.9 k: 0.139 dk: 0.0417

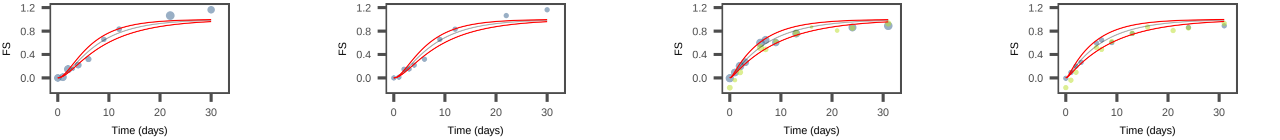

CALR

AA Weighted R2: 0.94 k: 0.436 dk: 0.143 | AA Unweighted R2: 0.93 k: 0.39 dk: 0.135 | HW Weighted R2: 0.98 k: 0.326 dk: 0.045 | HW Unweighted R2: 0.98 k: 0.328 dk: 0.049

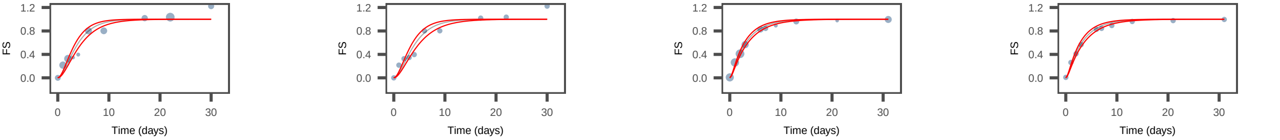

CALX

AA Weighted R2: 0.94 k: 0.399 dk: 0.124 | AA Unweighted R2: 0.92 k: 0.358 dk: 0.128 | HW Weighted R2: 0.98 k: 0.316 dk: 0.0445 | HW Unweighted R2: 0.98 k: 0.314 dk: 0.0442

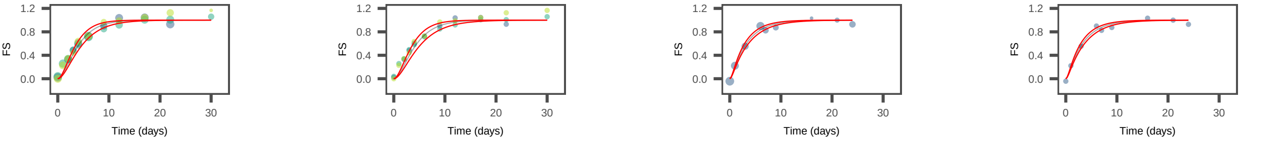

CAND1

AA Weighted R2: 0.95 k: 0.25 dk: 0.0709

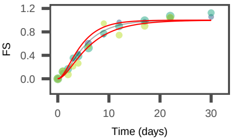

AA Unweighted R2: 0.95 k: 0.25 dk: 0.071

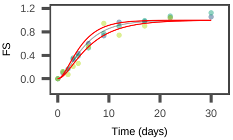

HW Weighted R2: 0.93 k: 0.179 dk: 0.0468

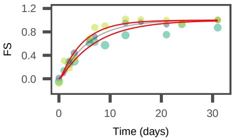

HW Unweighted R2: 0.93 k: 0.182 dk: 0.0467

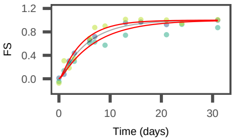

CAP1

AA Weighted R2: 0.93 k: 0.126 dk: 0.0427

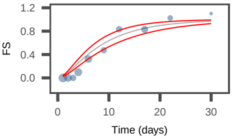

AA Unweighted R2: 0.94 k: 0.124 dk: 0.0412

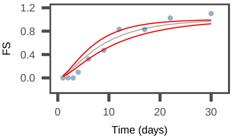

HW Weighted R2: 0.94 k: 0.265 dk: 0.0723

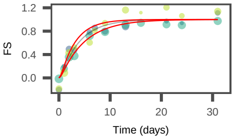

HW Unweighted R2: 0.93 k: 0.268 dk: 0.075

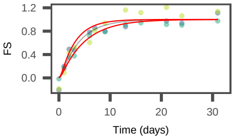

CATA

AA Weighted R2: 0.94 k: 0.28 dk: 0.0767

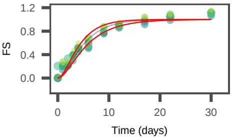

AA Unweighted R2: 0.93 k: 0.291 dk: 0.0827

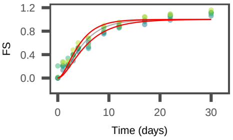

HW Weighted R2: 0.96 k: 0.202 dk: 0.0359

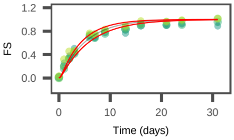

HW Unweighted R2: 0.96 k: 0.201 dk: 0.0361

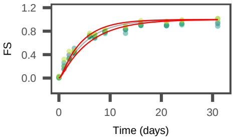

CATH

AA Weighted R2: 0.91 k: 0.397 dk: 0.136

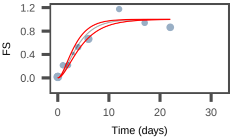

AA Unweighted R2: 0.92 k: 0.414 dk: 0.141

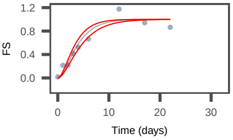

HW Weighted R2: 0.93 k: 0.222 dk: 0.0634

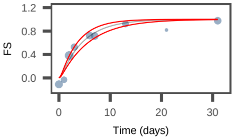

HW Unweighted R2: 0.93 k: 0.22 dk: 0.0639

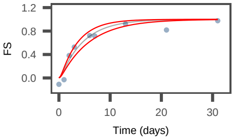

CBR1

AA Weighted R2: 0.93 k: 0.156 dk: 0.035

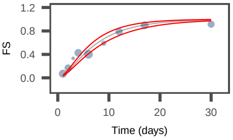

AA Unweighted R2: 0.93 k: 0.157 dk: 0.0361

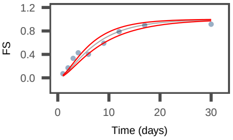

HW Weighted R2: 0.74 k: 0.116 dk: 0.0502

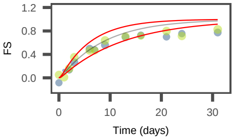

HW Unweighted R2: 0.72 k: 0.121 dk: 0.0543

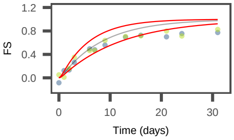

CBR4

AA Weighted R2: 0.89 k: 0.0752 dk: 0.0229

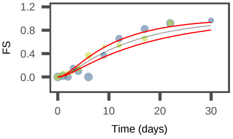

AA Unweighted R2: 0.9 k: 0.0777 dk: 0.0227

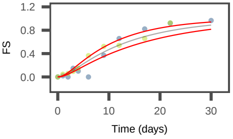

HW Weighted R2: 0.9 k: 0.0799 dk: 0.0238

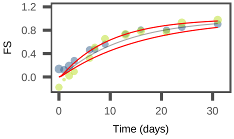

HW Unweighted R2: 0.9 k: 0.0801 dk: 0.0238

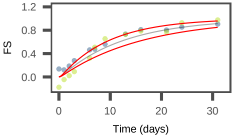

CDC42

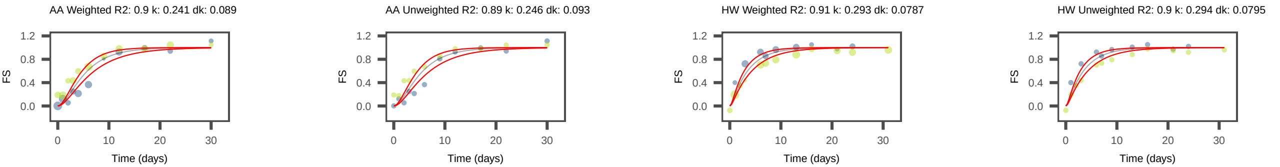

CENPV

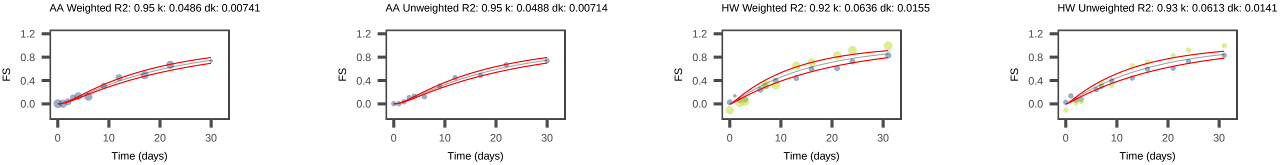

CGL

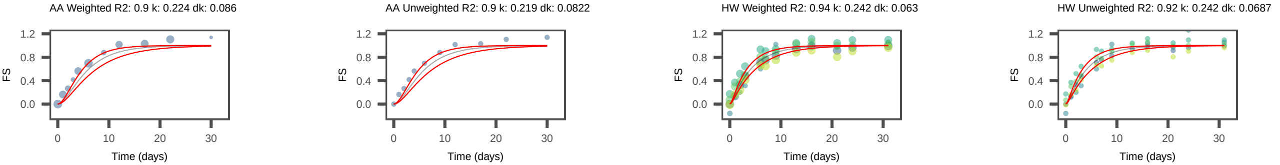

CH10

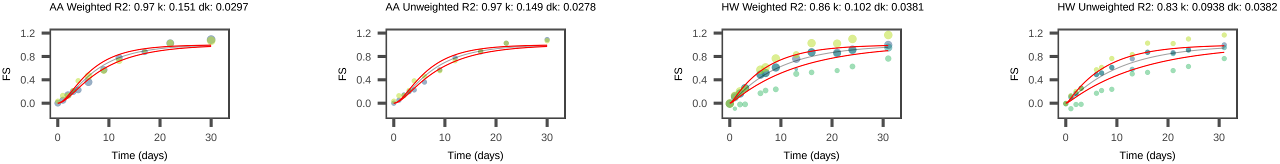

CH60

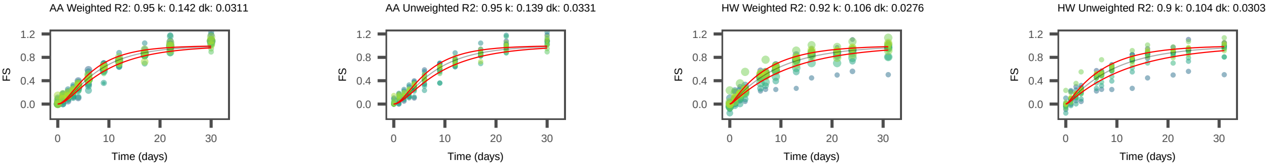

CHDH

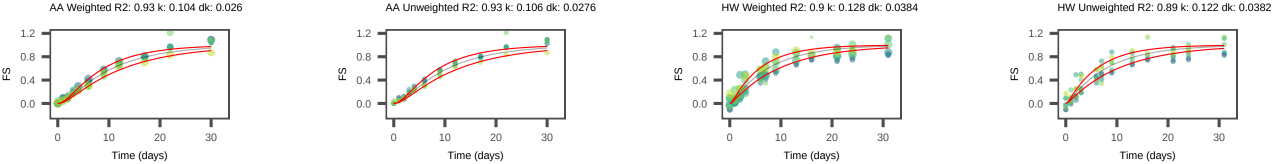

CISD1

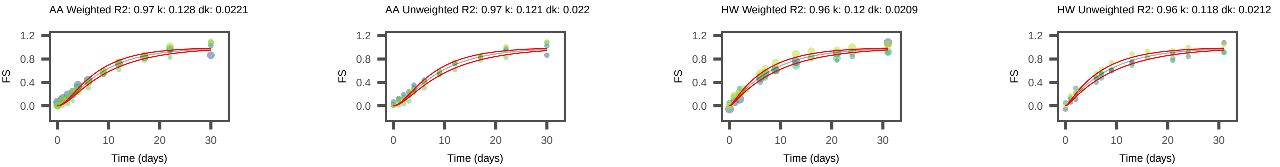

CISY

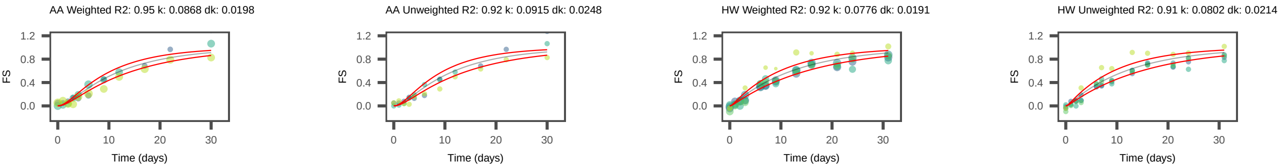

CLH1

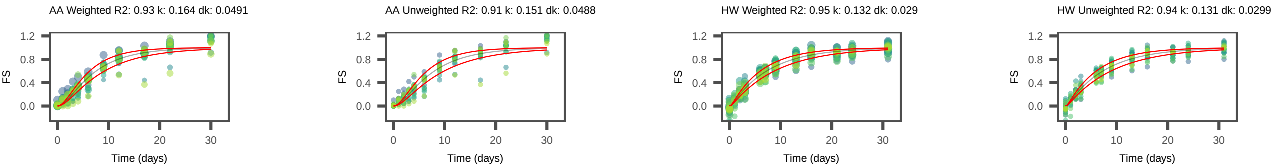

CLIC1

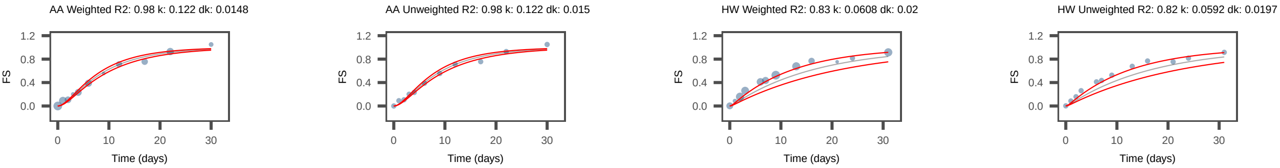

CLIC4

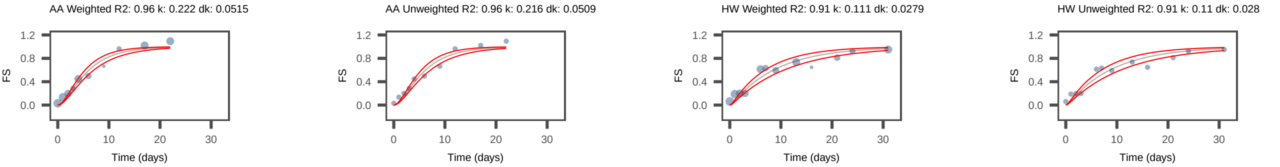

CLTRN

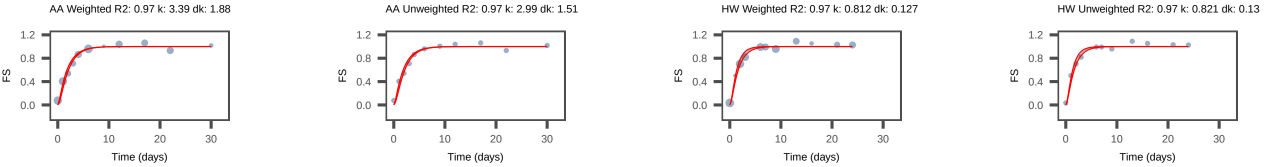

CLYBL

AA Weighted R2: 0.91 k: 0.0915 dk: 0.0275 | AA Unweighted R2: 0.91 k: 0.0917 dk: 0.0276 | HW Weighted R2: 0.96 k: 0.0939 dk: 0.019 | HW Unweighted R2: 0.94 k: 0.092 dk: 0.0207

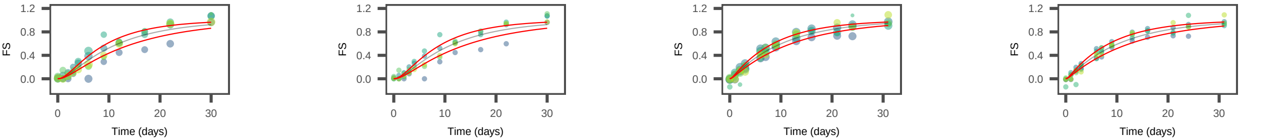

CMBL

AA Weighted R2: 0.96 k: 0.313 dk: 0.0782 | AA Unweighted R2: 0.96 k: 0.318 dk: 0.0776 | HW Weighted R2: 0.83 k: 0.147 dk: 0.061 | HW Unweighted R2: 0.87 k: 0.168 dk: 0.0612

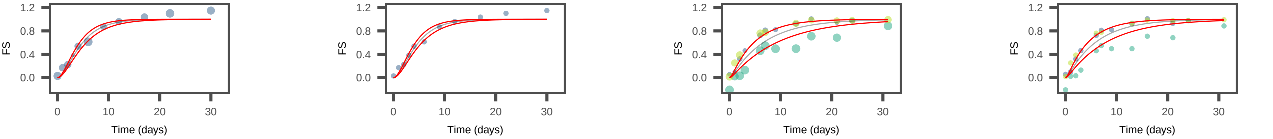

CMC1

AA Weighted R2: 0.98 k: 0.0981 dk: 0.0131 | AA Unweighted R2: 0.98 k: 0.0978 dk: 0.0141 | HW Weighted R2: 0.97 k: 0.0909 dk: 0.0138 | HW Unweighted R2: 0.97 k: 0.0911 dk: 0.0137

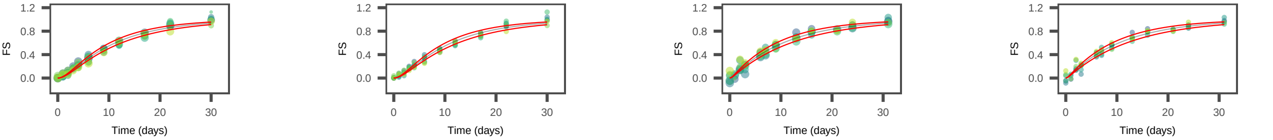

CMC2

AA Weighted R2: 0.96 k: 0.128 dk: 0.0267 | AA Unweighted R2: 0.95 k: 0.127 dk: 0.0292 | HW Weighted R2: 0.93 k: 0.11 dk: 0.0274 | HW Unweighted R2: 0.93 k: 0.104 dk: 0.0275

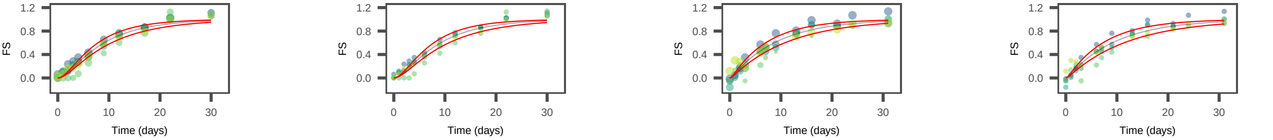

CNDP2

AA Weighted R2: 0.93 k: 0.229 dk: 0.0726 | AA Unweighted R2: 0.92 k: 0.236 dk: 0.0772 | HW Weighted R2: 0.93 k: 0.24 dk: 0.0653 | HW Unweighted R2: 0.92 k: 0.24 dk: 0.0667

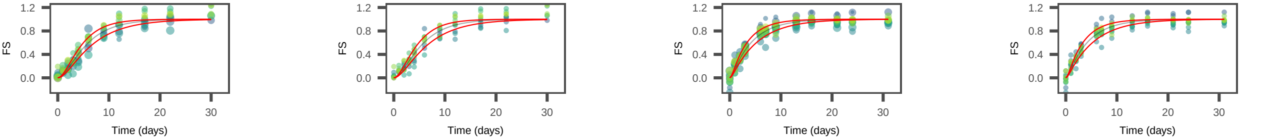

COASY

AA Weighted R2: 0.93 k: 0.234 dk: 0.0763 | AA Unweighted R2: 0.93 k: 0.235 dk: 0.0778 | HW Weighted R2: 0.93 k: 0.184 dk: 0.042 | HW Unweighted R2: 0.93 k: 0.18 dk: 0.0422

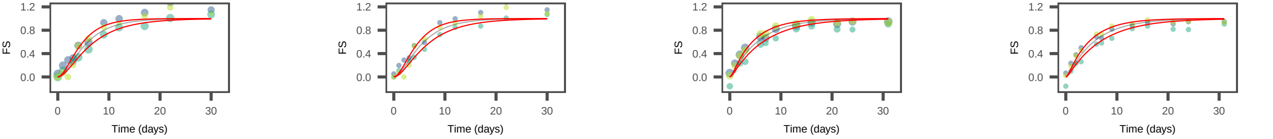

COBL1

AA Weighted R2: 0.94 k: 0.228 dk: 0.0674

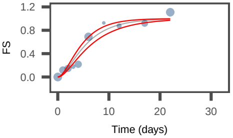

AA Unweighted R2: 0.94 k: 0.226 dk: 0.068

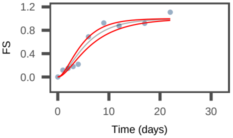

HW Weighted R2: 0.92 k: 0.222 dk: 0.0649

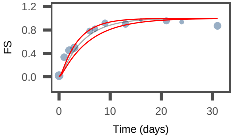

HW Unweighted R2: 0.92 k: 0.218 dk: 0.064

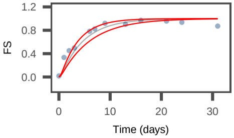

COEA1

AA Weighted R2: 0.89 k: 0.0748 dk: 0.0226

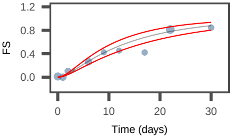

AA Unweighted R2: 0.87 k: 0.0803 dk: 0.0268

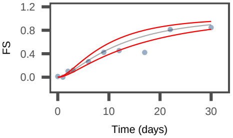

HW Weighted R2: 0.97 k: 0.0672 dk: 0.00876

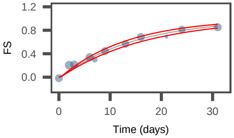

HW Unweighted R2: 0.97 k: 0.0669 dk: 0.00871

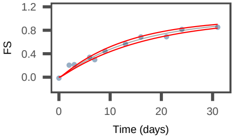

COF1

AA Weighted R2: 0.93 k: 0.201 dk: 0.0556

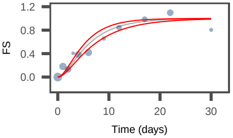

AA Unweighted R2: 0.92 k: 0.199 dk: 0.0581

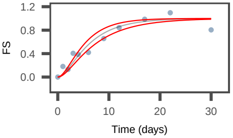

HW Weighted R2: 0.91 k: 0.23 dk: 0.0685

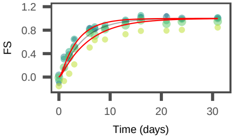

HW Unweighted R2: 0.9 k: 0.226 dk: 0.0731

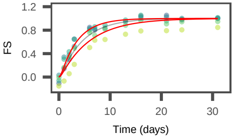

COMT

AA Weighted R2: 0.96 k: 0.433 dk: 0.12

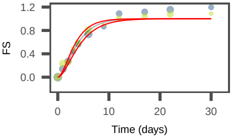

AA Unweighted R2: 0.96 k: 0.434 dk: 0.122

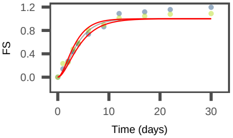

HW Weighted R2: 0.97 k: 0.29 dk: 0.0538

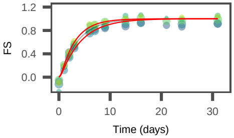

HW Unweighted R2: 0.96 k: 0.295 dk: 0.0562

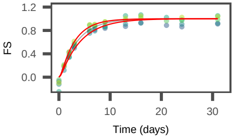

COPA

AA Weighted R2: 0.92 k: 0.318 dk: 0.12

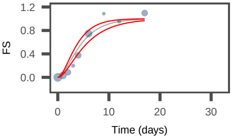

AA Unweighted R2: 0.9 k: 0.31 dk: 0.13

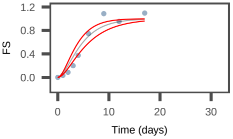

HW Weighted R2: 0.94 k: 0.312 dk: 0.0657

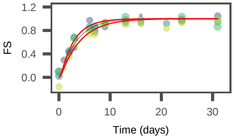

HW Unweighted R2: 0.94 k: 0.31 dk: 0.0657

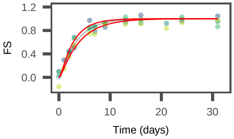

COPB

AA Weighted R2: 0.91 k: 0.285 dk: 0.109

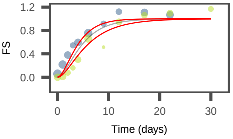

AA Unweighted R2: 0.9 k: 0.278 dk: 0.111

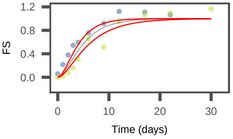

HW Weighted R2: 0.91 k: 0.233 dk: 0.0692

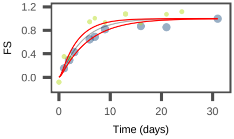

HW Unweighted R2: 0.9 k: 0.262 dk: 0.0858

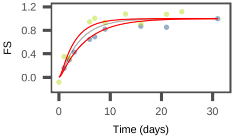

COPB2

AA Weighted R2: 0.85 k: 0.254 dk: 0.117 | AA Unweighted R2: 0.85 k: 0.262 dk: 0.122 | HW Weighted R2: 0.93 k: 0.43 dk: 0.112 | HW Unweighted R2: 0.93 k: 0.419 dk: 0.111

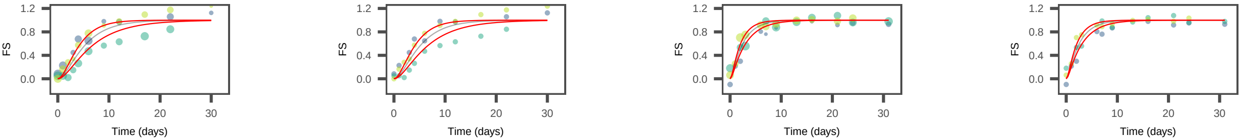

COPG1

AA Weighted R2: 0.92 k: 1.02 dk: 0.469 | AA Unweighted R2: 0.92 k: 0.991 dk: 0.444 | HW Weighted R2: 0.92 k: 0.279 dk: 0.071 | HW Unweighted R2: 0.93 k: 0.276 dk: 0.0682

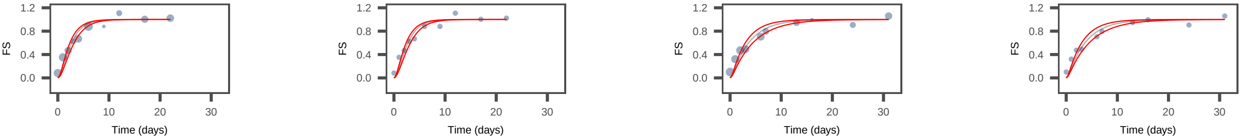

COQ9

AA Weighted R2: 0.97 k: 0.209 dk: 0.0472 | AA Unweighted R2: 0.97 k: 0.204 dk: 0.0455 | HW Weighted R2: 0.96 k: 0.184 dk: 0.0367 | HW Unweighted R2: 0.95 k: 0.181 dk: 0.0384

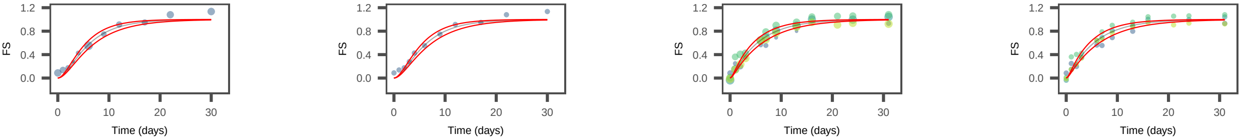

COX1

AA Weighted R2: 0.94 k: 0.114 dk: 0.0258 | AA Unweighted R2: 0.93 k: 0.111 dk: 0.027 | HW Weighted R2: 0.99 k: 0.0956 dk: 0.00875 | HW Unweighted R2: 0.99 k: 0.0959 dk: 0.00884

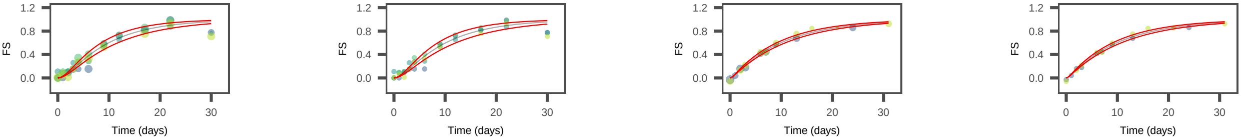

COX2

AA Weighted R2: 0.94 k: 0.113 dk: 0.0268 | AA Unweighted R2: 0.94 k: 0.109 dk: 0.0264 | HW Weighted R2: 0.96 k: 0.0853 dk: 0.0153 | HW Unweighted R2: 0.96 k: 0.0814 dk: 0.015

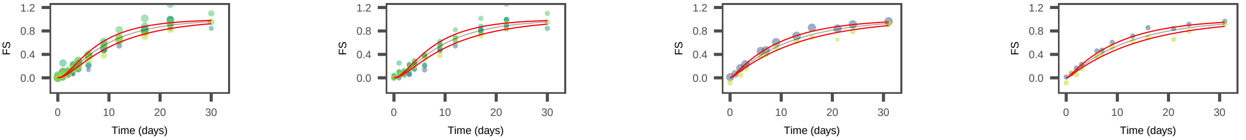

COX3

AA Weighted R2: 0.98 k: 0.172 dk: 0.0274 | AA Unweighted R2: 0.98 k: 0.168 dk: 0.0262 | HW Weighted R2: 0.98 k: 0.111 dk: 0.0129 | HW Unweighted R2: 0.98 k: 0.111 dk: 0.013

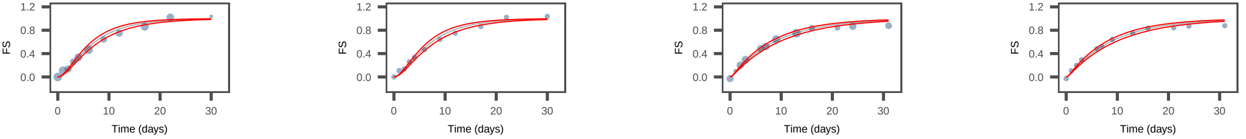

COX41

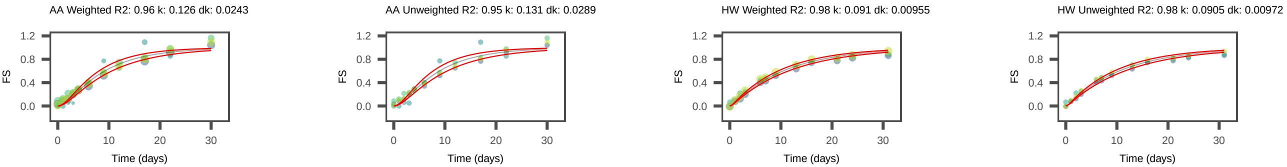

COX5A

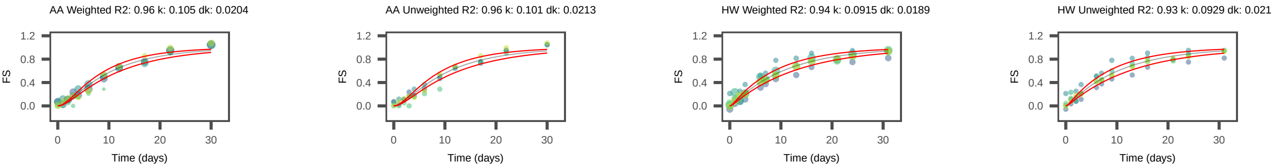

COX5B

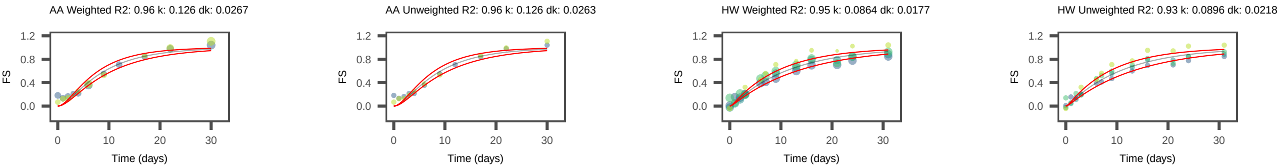

COX6C

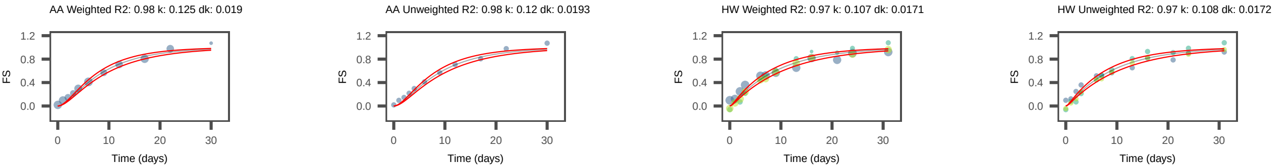

CP2J5

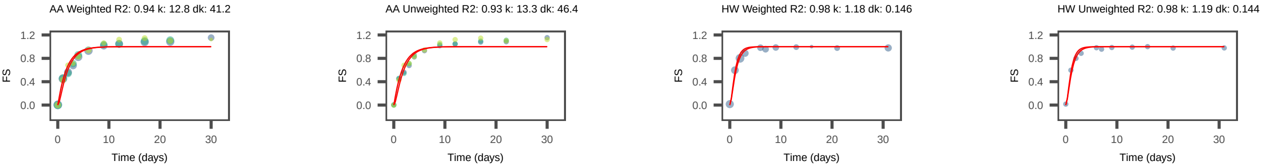

CP4B1

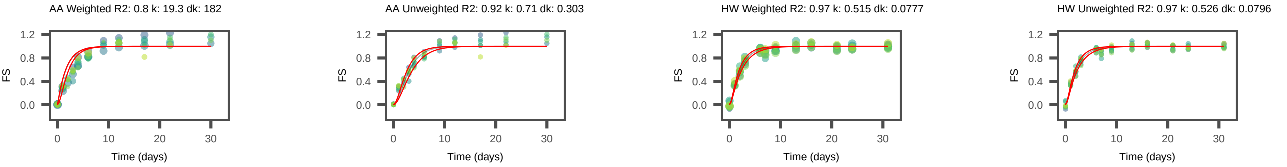

CP51A

AA Weighted R2: 0.9 k: 0.959 dk: 0.521

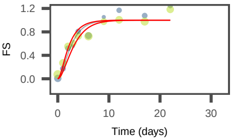

AA Unweighted R2: 0.9 k: 0.953 dk: 0.521

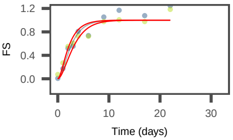

HW Weighted R2: 0.91 k: 1.33 dk: 0.471

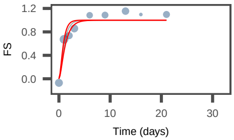

HW Unweighted R2: 0.91 k: 1.23 dk: 0.434

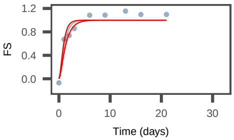

CPT1A

AA Weighted R2: 0.96 k: 0.203 dk: 0.0454

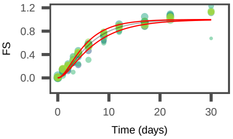

AA Unweighted R2: 0.95 k: 0.197 dk: 0.0495

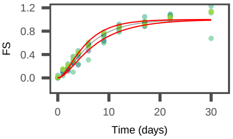

HW Weighted R2: 0.95 k: 0.224 dk: 0.0529

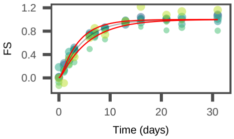

HW Unweighted R2: 0.94 k: 0.217 dk: 0.0523

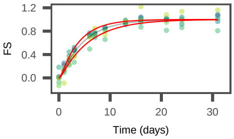

CPT2

AA Weighted R2: 0.96 k: 0.151 dk: 0.0317

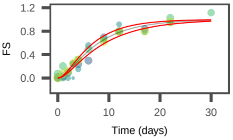

AA Unweighted R2: 0.95 k: 0.152 dk: 0.0362

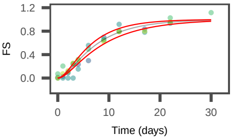

HW Weighted R2: 0.89 k: 0.131 dk: 0.0438

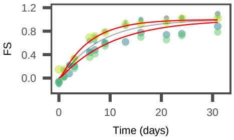

HW Unweighted R2: 0.89 k: 0.137 dk: 0.0465

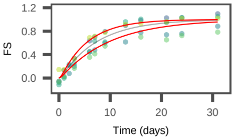

CRYL1

AA Weighted R2: 0.94 k: 0.254 dk: 0.0745

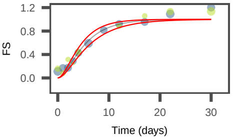

AA Unweighted R2: 0.95 k: 0.256 dk: 0.0742

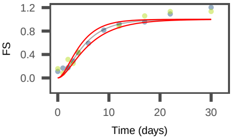

HW Weighted R2: 0.98 k: 0.147 dk: 0.0186

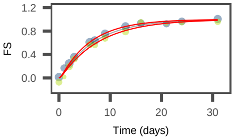

HW Unweighted R2: 0.98 k: 0.147 dk: 0.0194

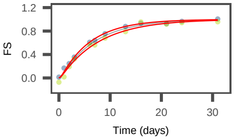

CSAD

AA Weighted R2: 0.98 k: 0.342 dk: 0.064

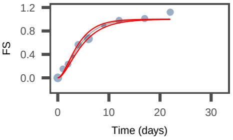

AA Unweighted R2: 0.98 k: 0.344 dk: 0.0639

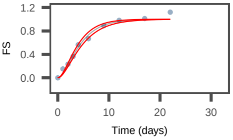

HW Weighted R2: 0.98 k: 0.172 dk: 0.0235

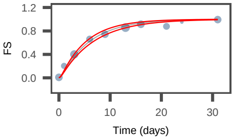

HW Unweighted R2: 0.98 k: 0.172 dk: 0.0239

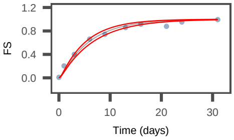

CSRP1

AA Weighted R2: 0.99 k: 0.0982 dk: 0.00973

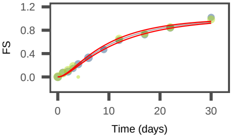

AA Unweighted R2: 0.97 k: 0.0944 dk: 0.0153

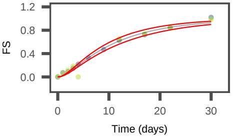

HW Weighted R2: 0.93 k: 0.0882 dk: 0.0198

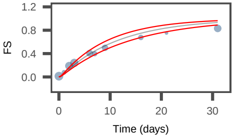

HW Unweighted R2: 0.92 k: 0.0918 dk: 0.0219

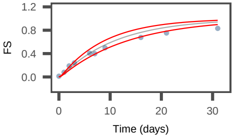

CTND1

AA Weighted R2: 0.94 k: 0.58 dk: 0.212

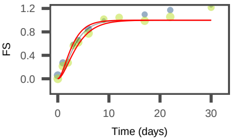

AA Unweighted R2: 0.93 k: 0.605 dk: 0.243

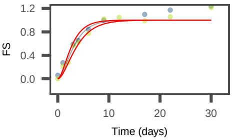

HW Weighted R2: 0.93 k: 0.349 dk: 0.0943

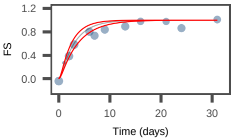

HW Unweighted R2: 0.93 k: 0.369 dk: 0.1

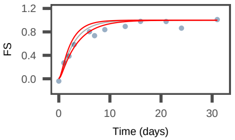

CUBN

AA Weighted R2: 0.91 k: 0.743 dk: 0.334

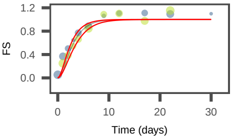

AA Unweighted R2: 0.91 k: 0.742 dk: 0.336

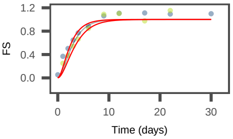

HW Weighted R2: 0.92 k: 0.336 dk: 0.101

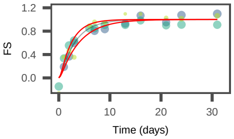

HW Unweighted R2: 0.92 k: 0.334 dk: 0.104

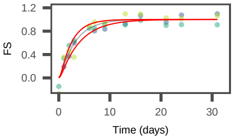

CX6B1

AA Weighted R2: 0.98 k: 0.116 dk: 0.0166

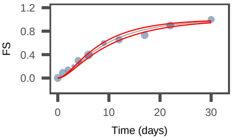

AA Unweighted R2: 0.98 k: 0.117 dk: 0.0167

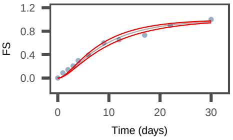

HW Weighted R2: 0.95 k: 0.119 dk: 0.0211

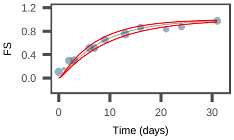

HW Unweighted R2: 0.95 k: 0.119 dk: 0.0212

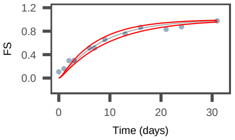

CY1

AA Weighted R2: 0.93 k: 0.108 dk: 0.0298

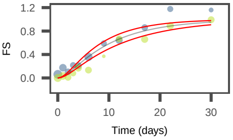

AA Unweighted R2: 0.93 k: 0.108 dk: 0.0316

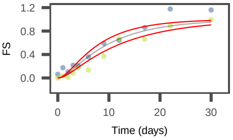

HW Weighted R2: 0.96 k: 0.0722 dk: 0.0112

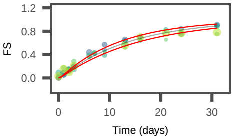

HW Unweighted R2: 0.96 k: 0.0725 dk: 0.0114

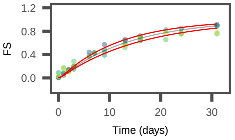

CYC

AA Weighted R2: 0.99 k: 0.116 dk: 0.0135

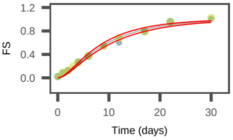

AA Unweighted R2: 0.99 k: 0.115 dk: 0.0136

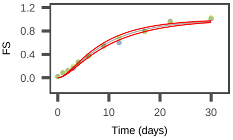

HW Weighted R2: 0.99 k: 0.097 dk: 0.00895

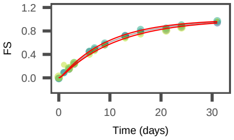

HW Unweighted R2: 0.99 k: 0.097 dk: 0.00984

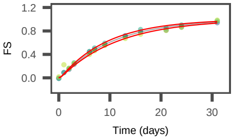

CYTB

AA Weighted R2: 0.97 k: 0.199 dk: 0.0413

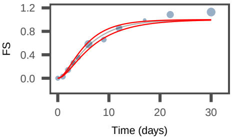

AA Unweighted R2: 0.98 k: 0.197 dk: 0.0372

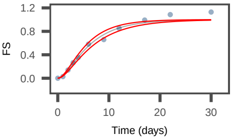

HW Weighted R2: 0.99 k: 0.167 dk: 0.0182

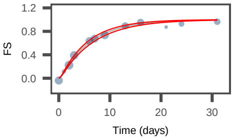

HW Unweighted R2: 0.99 k: 0.167 dk: 0.0184

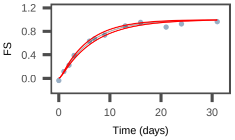

D39U1

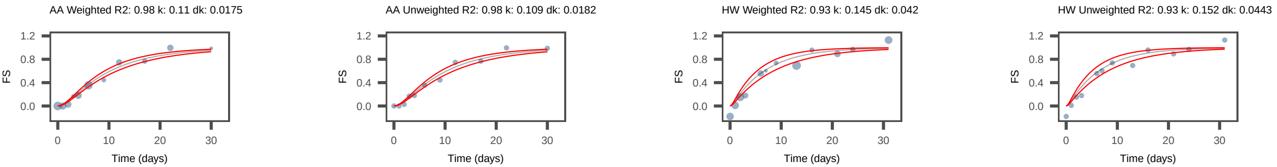

DAB2

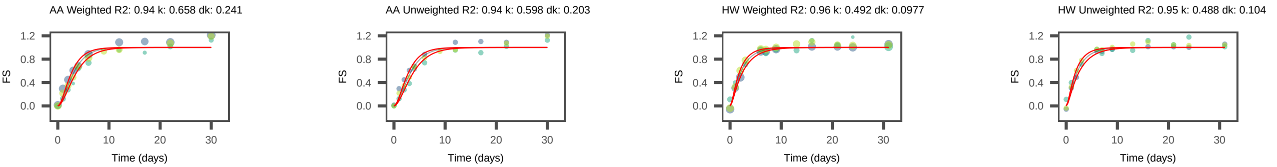

DCXR

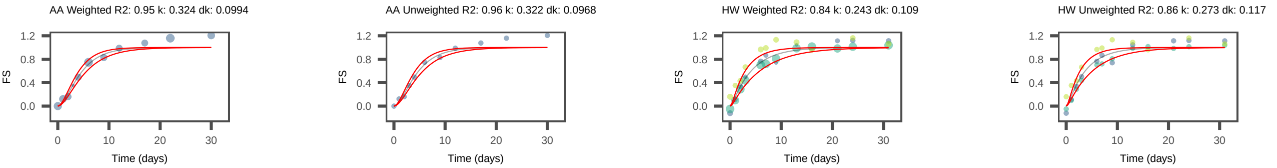

DDAH1

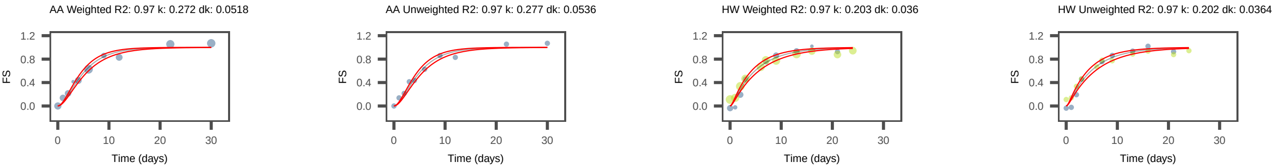

DDX1

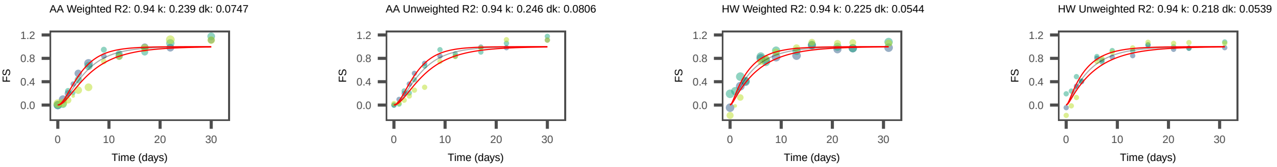

DECR

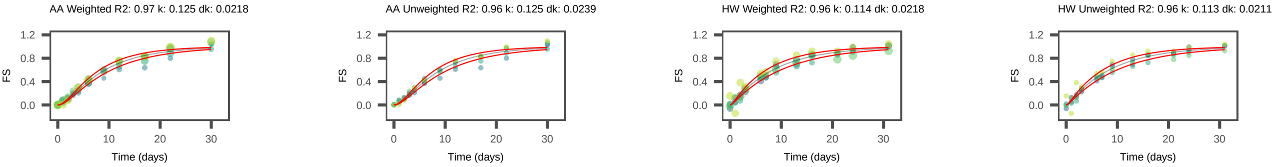

DHB4

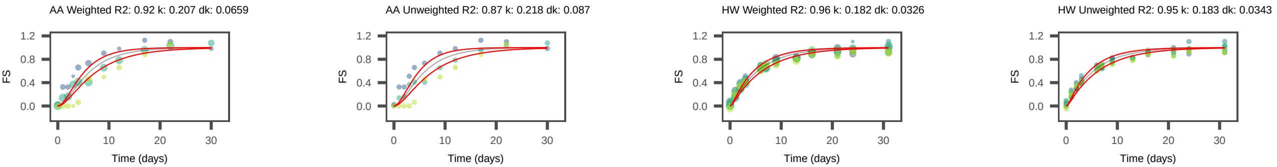

DHE3

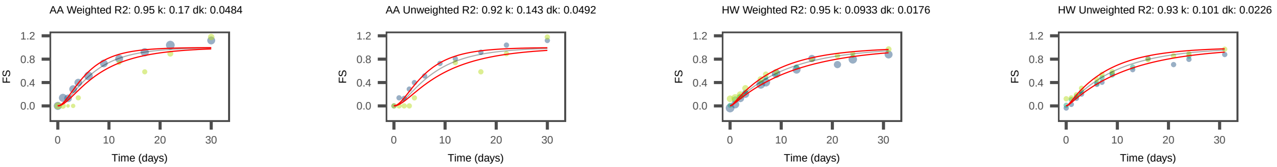

DHPR

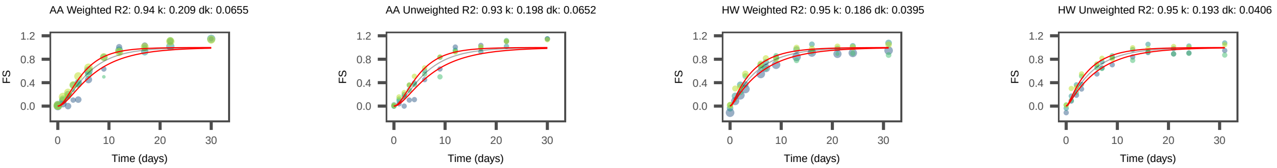

DHRS1

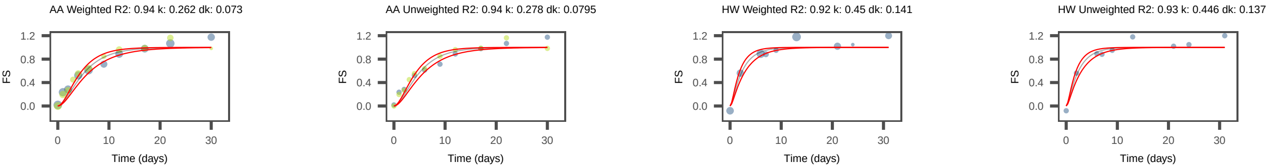

DHRS4

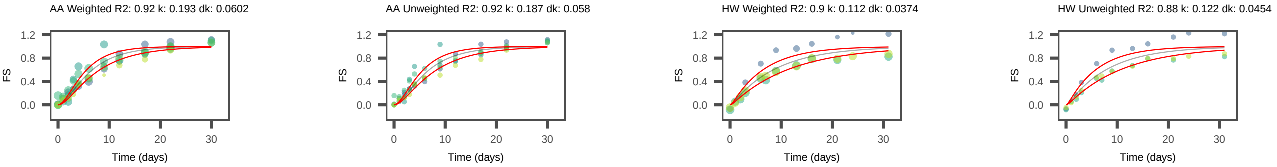

DHSO

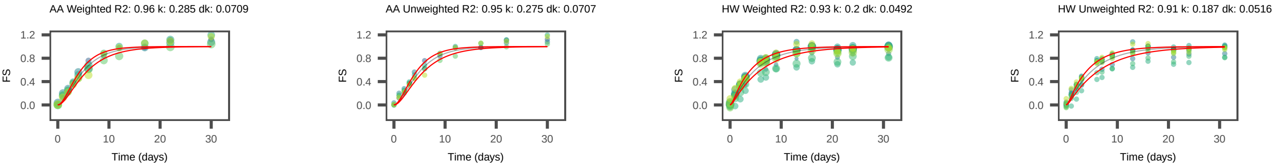

DHTK1

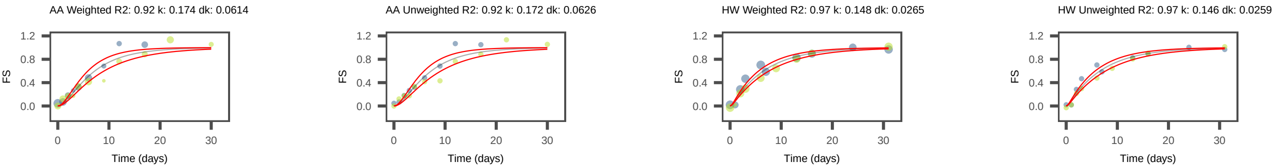

DHX9

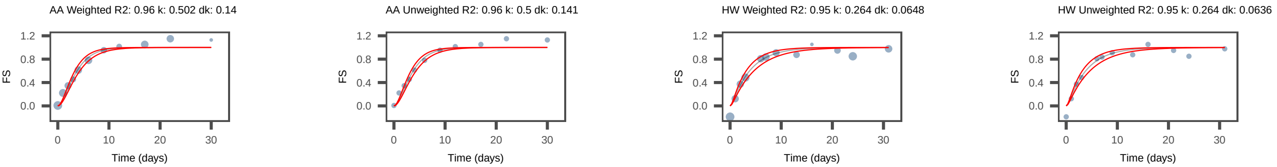

DIC

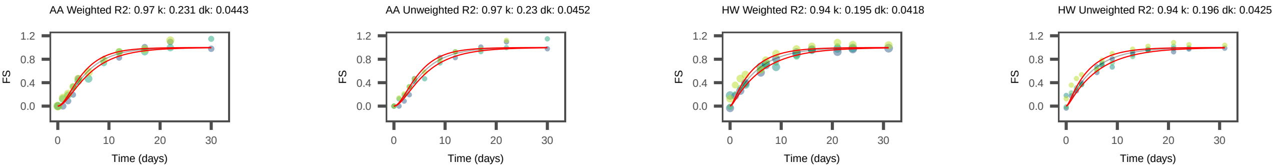

DLDH

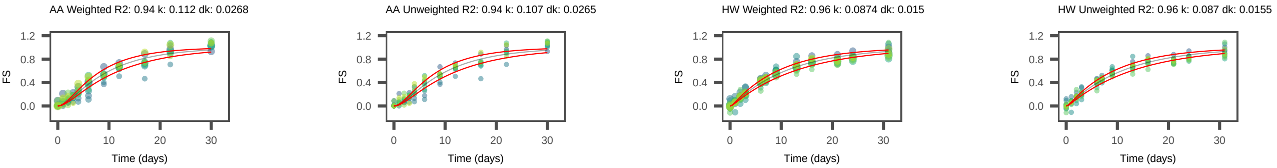

DNJA3

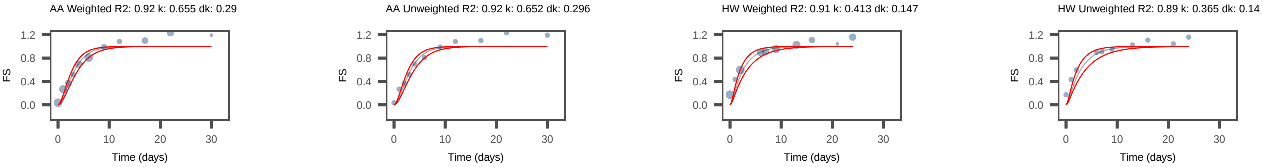

DPEP1

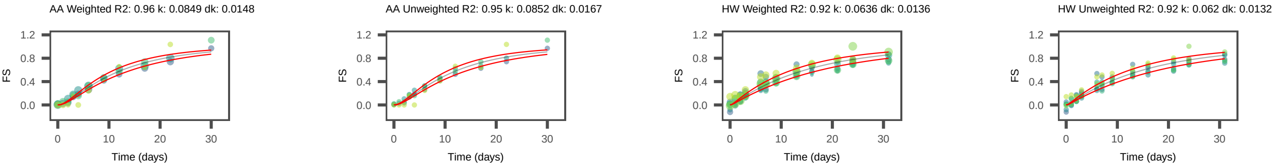

DYHC1

AA Weighted R2: 0.9 k: 0.279 dk: 0.107 | AA Unweighted R2: 0.88 k: 0.258 dk: 0.105 | HW Weighted R2: 0.81 k: 0.236 dk: 0.108 | HW Unweighted R2: 0.81 k: 0.227 dk: 0.102

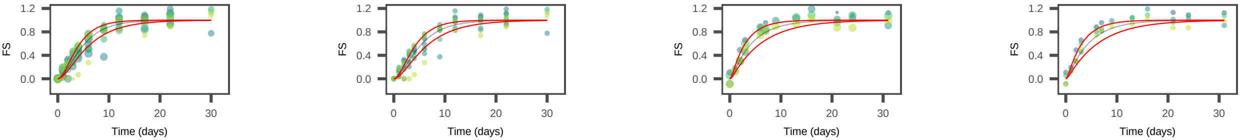

E41L3

AA Weighted R2: 0.91 k: 0.424 dk: 0.164 | AA Unweighted R2: 0.91 k: 0.422 dk: 0.162 | HW Weighted R2: 0.94 k: 0.142 dk: 0.0333 | HW Unweighted R2: 0.93 k: 0.144 dk: 0.0353

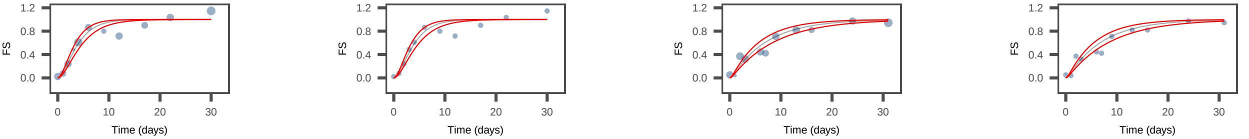

ECHA

AA Weighted R2: 0.93 k: 0.0979 dk: 0.0233 | AA Unweighted R2: 0.93 k: 0.0977 dk: 0.0238 | HW Weighted R2: 0.91 k: 0.0991 dk: 0.026 | HW Unweighted R2: 0.91 k: 0.0988 dk: 0.0263

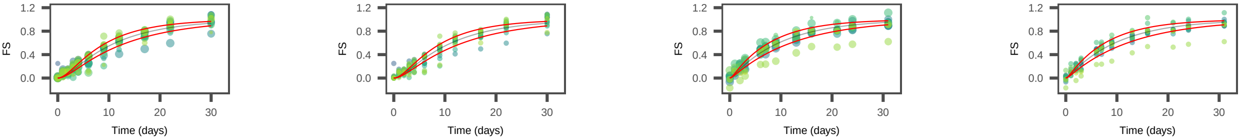

ECHB

AA Weighted R2: 0.95 k: 0.175 dk: 0.0404 | AA Unweighted R2: 0.94 k: 0.168 dk: 0.0414 | HW Weighted R2: 0.86 k: 0.149 dk: 0.0496 | HW Unweighted R2: 0.85 k: 0.154 dk: 0.0536

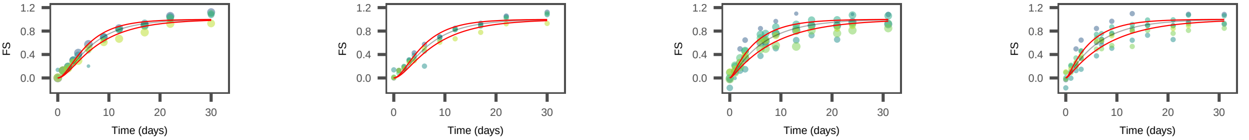

ECHM

AA Weighted R2: 0.98 k: 0.114 dk: 0.0177 | AA Unweighted R2: 0.97 k: 0.111 dk: 0.0189 | HW Weighted R2: 0.88 k: 0.0774 dk: 0.0226 | HW Unweighted R2: 0.88 k: 0.0759 dk: 0.022

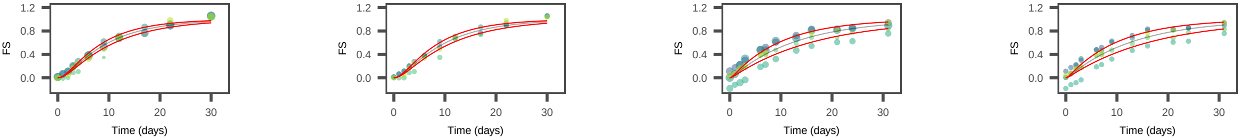

ECHP

AA Weighted R2: 0.95 k: 0.147 dk: 0.0312 | AA Unweighted R2: 0.94 k: 0.147 dk: 0.0331 | HW Weighted R2: 0.92 k: 0.125 dk: 0.0316 | HW Unweighted R2: 0.92 k: 0.124 dk: 0.0316

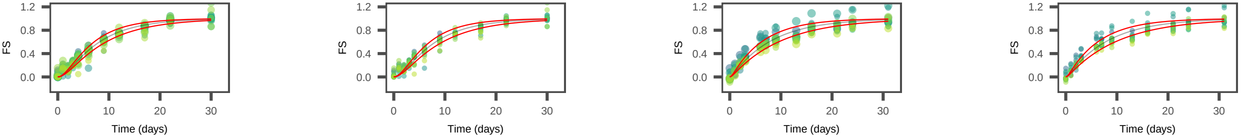

ECI1

AA Weighted R2: 0.96 k: 0.106 dk: 0.0213 | AA Unweighted R2: 0.96 k: 0.104 dk: 0.0214 | HW Weighted R2: 0.94 k: 0.0904 dk: 0.0202 | HW Unweighted R2: 0.93 k: 0.0936 dk: 0.0226

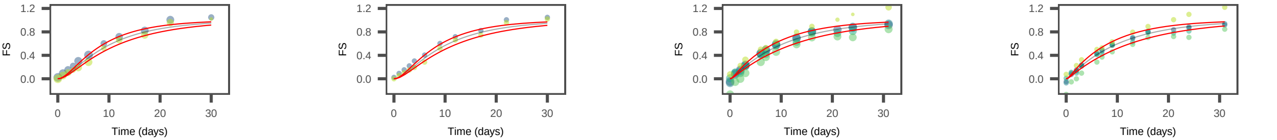

ECI2

AA Weighted R2: 0.93 k: 0.154 dk: 0.0458 | AA Unweighted R2: 0.93 k: 0.153 dk: 0.0463 | HW Weighted R2: 0.94 k: 0.105 dk: 0.0242 | HW Unweighted R2: 0.94 k: 0.101 dk: 0.0238

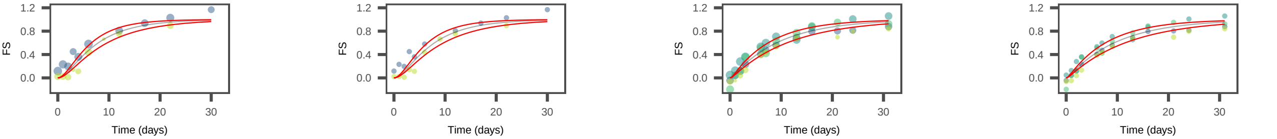

ECI3

AA Weighted R2: 0.95 k: 0.168 dk: 0.0411 | AA Unweighted R2: 0.95 k: 0.168 dk: 0.0423 | HW Weighted R2: 0.91 k: 0.122 dk: 0.0364 | HW Unweighted R2: 0.91 k: 0.129 dk: 0.0394

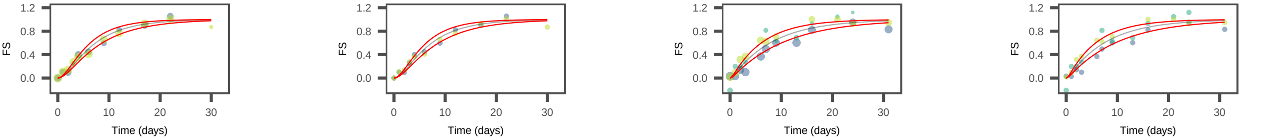

ECSIT

AA Weighted R2: 0.96 k: 0.721 dk: 0.222 | AA Unweighted R2: 0.96 k: 0.719 dk: 0.225 | HW Weighted R2: 0.96 k: 0.418 dk: 0.0887 | HW Unweighted R2: 0.96 k: 0.418 dk: 0.09

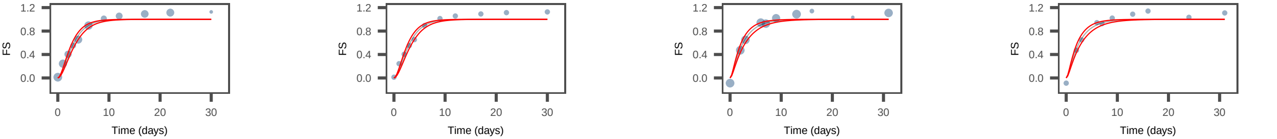

EF1A1

AA Weighted R2: 0.94 k: 0.312 dk: 0.0928 | AA Unweighted R2: 0.93 k: 0.295 dk: 0.093 | HW Weighted R2: 0.95 k: 0.216 dk: 0.0477 | HW Unweighted R2: 0.94 k: 0.216 dk: 0.0499

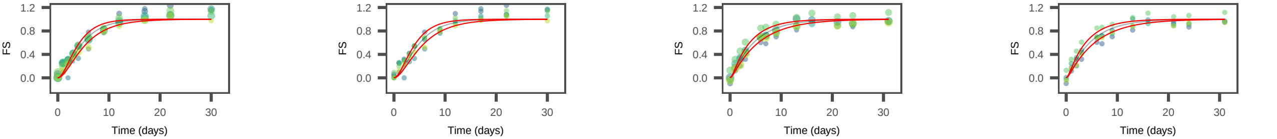

EF1G

AA Weighted R2: 0.94 k: 0.183 dk: 0.0517 | AA Unweighted R2: 0.94 k: 0.178 dk: 0.0511 | HW Weighted R2: 0.97 k: 0.158 dk: 0.0306 | HW Unweighted R2: 0.97 k: 0.158 dk: 0.0301

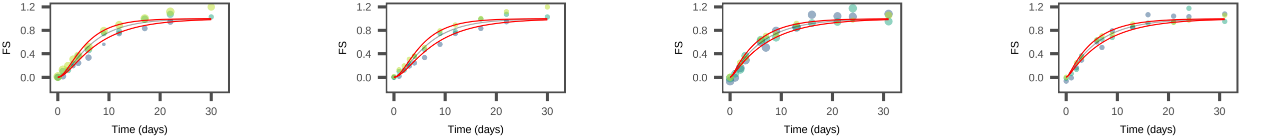

EF2

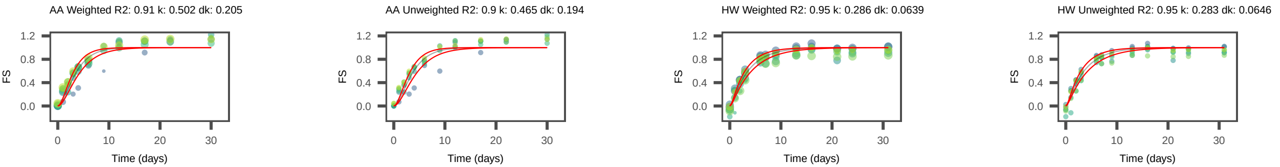

EFTU

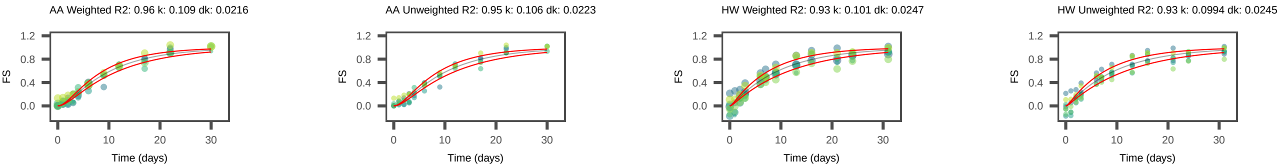

EHD4

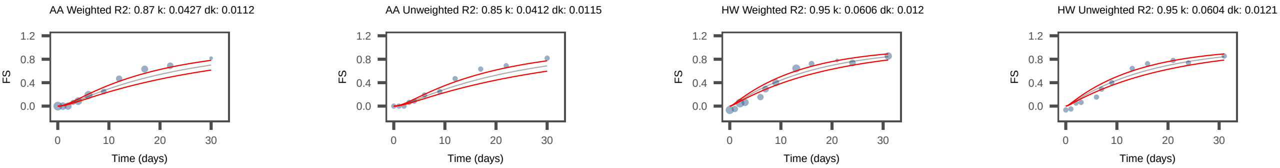

EIF3B

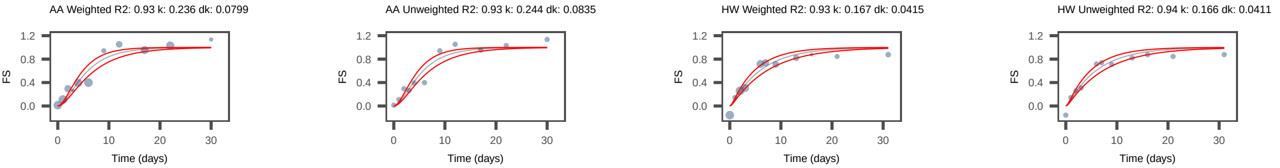

EIF3H

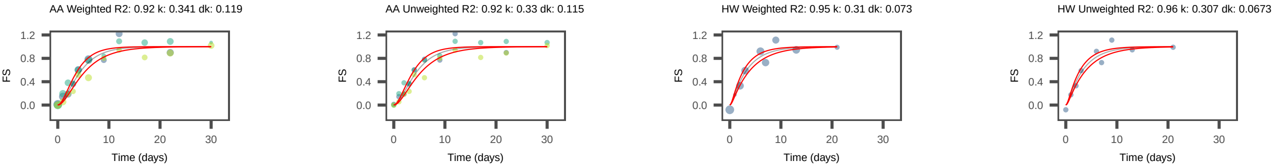

EM55

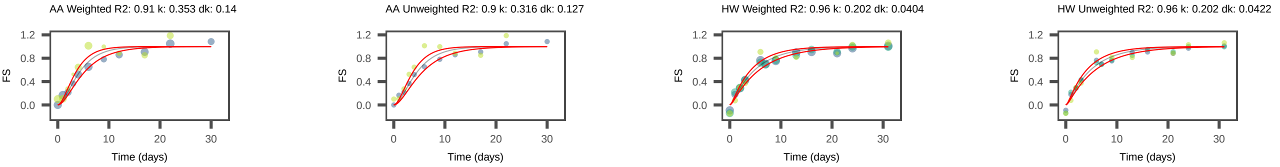

ENOA

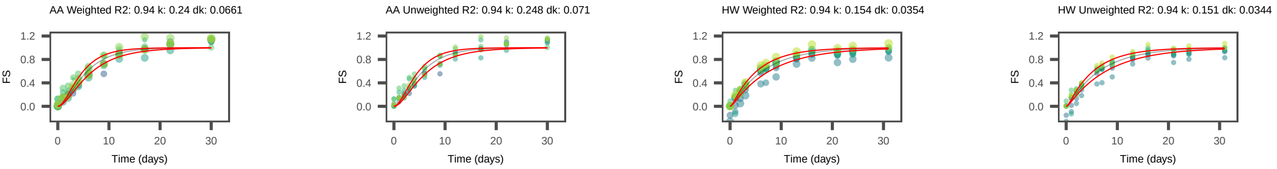

ENPL

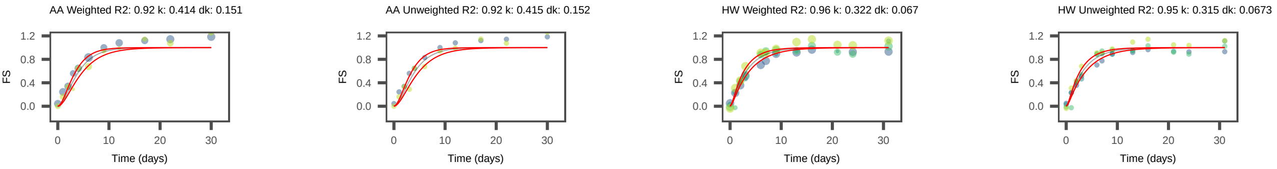

ENPP3

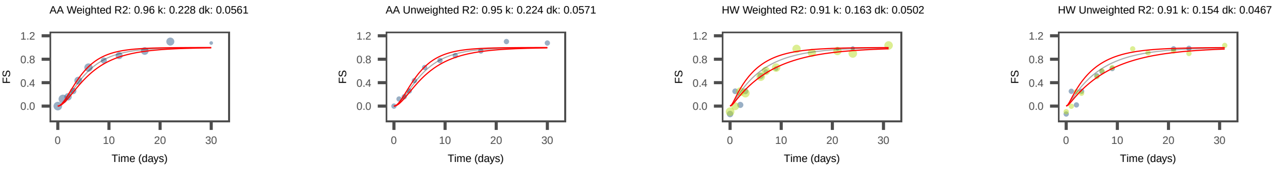

ENTP5

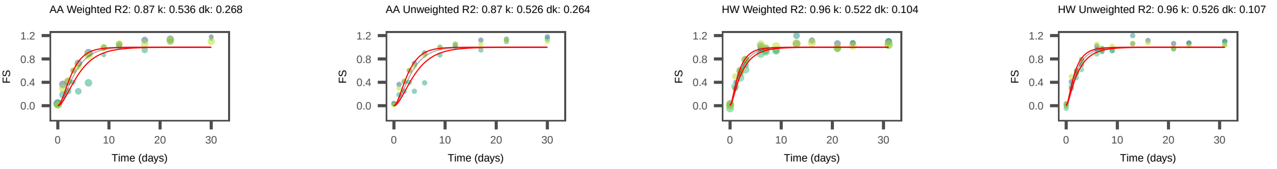

ERLN2

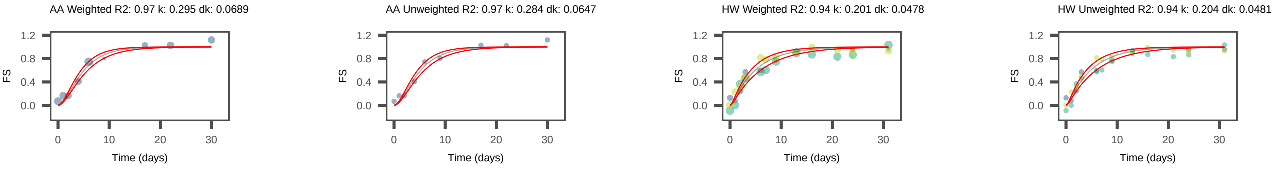

ES8L2

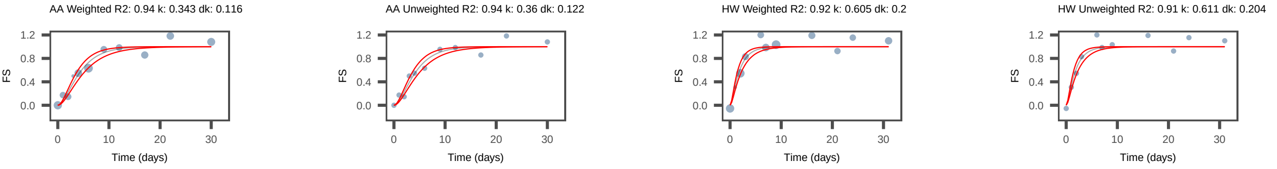

EST1C

AA Weighted R2: 0.96 k: 0.786 dk: 0.271

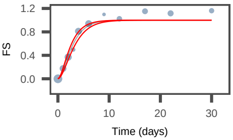

AA Unweighted R2: 0.95 k: 0.776 dk: 0.28

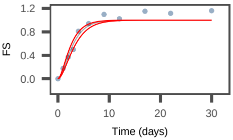

HW Weighted R2: 0.9 k: 0.322 dk: 0.124

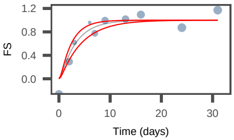

HW Unweighted R2: 0.9 k: 0.325 dk: 0.124

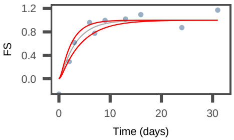

EST1D

AA Weighted R2: 0.94 k: 0.47 dk: 0.15

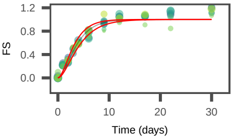

AA Unweighted R2: 0.94 k: 0.463 dk: 0.146

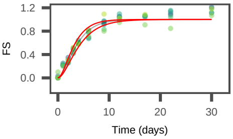

HW Weighted R2: 0.93 k: 0.305 dk: 0.0812

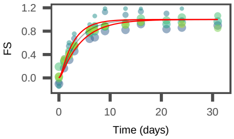

HW Unweighted R2: 0.91 k: 0.341 dk: 0.0975

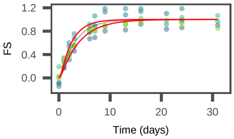

EST1F

AA Weighted R2: 0.92 k: 0.368 dk: 0.133

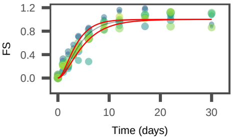

AA Unweighted R2: 0.9 k: 0.392 dk: 0.153

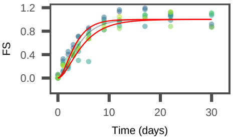

HW Weighted R2: 0.95 k: 0.303 dk: 0.0658

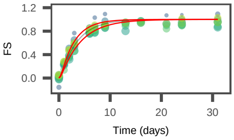

HW Unweighted R2: 0.93 k: 0.33 dk: 0.0843

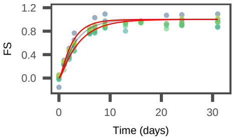

EST2C

AA Weighted R2: 0.93 k: 0.364 dk: 0.128

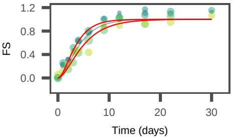

AA Unweighted R2: 0.91 k: 0.372 dk: 0.144

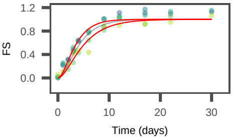

HW Weighted R2: 0.99 k: 0.317 dk: 0.0373

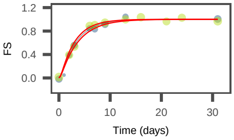

HW Unweighted R2: 0.98 k: 0.31 dk: 0.042

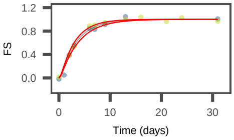

ESTD

AA Weighted R2: 0.95 k: 0.303 dk: 0.0885

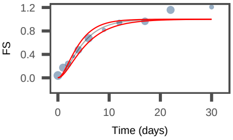

AA Unweighted R2: 0.95 k: 0.302 dk: 0.0896

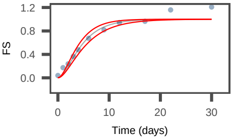

HW Weighted R2: 0.95 k: 0.17 dk: 0.0349

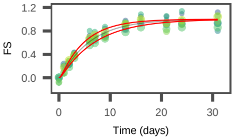

HW Unweighted R2: 0.95 k: 0.174 dk: 0.0363

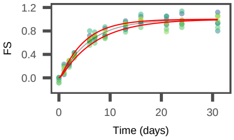

ETFA

AA Weighted R2: 0.98 k: 0.127 dk: 0.0159

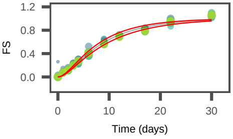

AA Unweighted R2: 0.98 k: 0.127 dk: 0.018

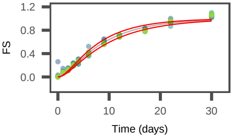

HW Weighted R2: 0.96 k: 0.108 dk: 0.0175

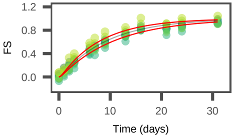

HW Unweighted R2: 0.97 k: 0.106 dk: 0.0163

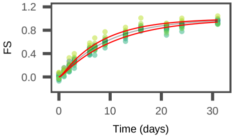

ETFB

AA Weighted R2: 0.98 k: 0.136 dk: 0.0174

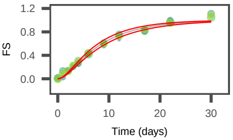

AA Unweighted R2: 0.98 k: 0.135 dk: 0.0174

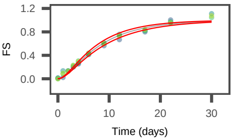

HW Weighted R2: 0.95 k: 0.125 dk: 0.0273

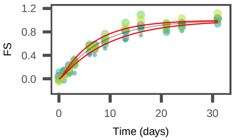

HW Unweighted R2: 0.94 k: 0.119 dk: 0.0278

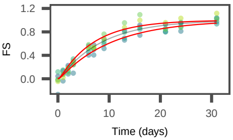

ETFD

AA Weighted R2: 0.9 k: 0.0891 dk: 0.0258

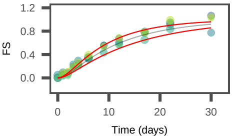

AA Unweighted R2: 0.91 k: 0.0926 dk: 0.0249

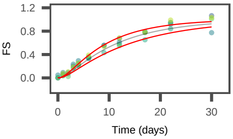

HW Weighted R2: 0.92 k: 0.107 dk: 0.0262

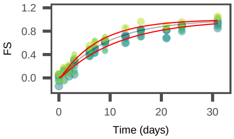

HW Unweighted R2: 0.92 k: 0.106 dk: 0.0262

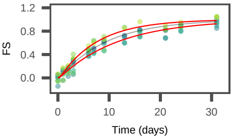

EZRI

AA Weighted R2: 0.92 k: 0.238 dk: 0.0808

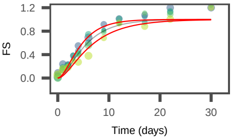

AA Unweighted R2: 0.91 k: 0.229 dk: 0.0793

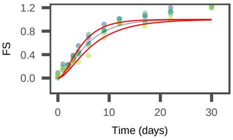

HW Weighted R2: 0.9 k: 0.213 dk: 0.0677

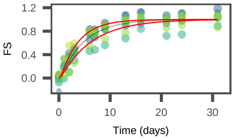

HW Unweighted R2: 0.91 k: 0.216 dk: 0.0662

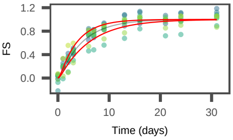

F16P1

AA Weighted R2: 0.96 k: 0.413 dk: 0.107

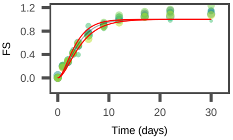

AA Unweighted R2: 0.96 k: 0.411 dk: 0.109

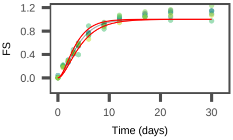

HW Weighted R2: 0.95 k: 0.243 dk: 0.0538

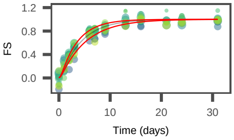

HW Unweighted R2: 0.95 k: 0.239 dk: 0.0563

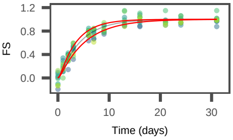

FAAA

AA Weighted R2: 0.96 k: 0.258 dk: 0.0663

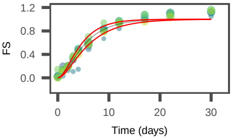

AA Unweighted R2: 0.95 k: 0.247 dk: 0.0687

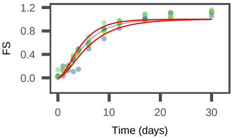

HW Weighted R2: 0.95 k: 0.183 dk: 0.0381

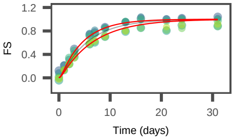

HW Unweighted R2: 0.95 k: 0.182 dk: 0.0376

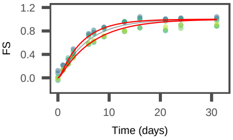

FABP4

AA Weighted R2: 0.97 k: 0.119 dk: 0.0195

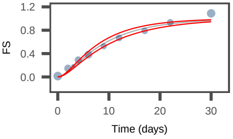

AA Unweighted R2: 0.98 k: 0.118 dk: 0.0185

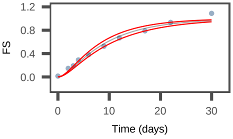

HW Weighted R2: 0.94 k: 0.121 dk: 0.0272

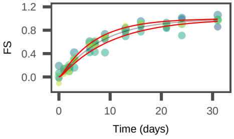

HW Unweighted R2: 0.94 k: 0.121 dk: 0.0268

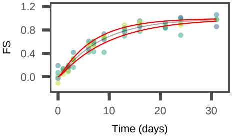

FAHD1

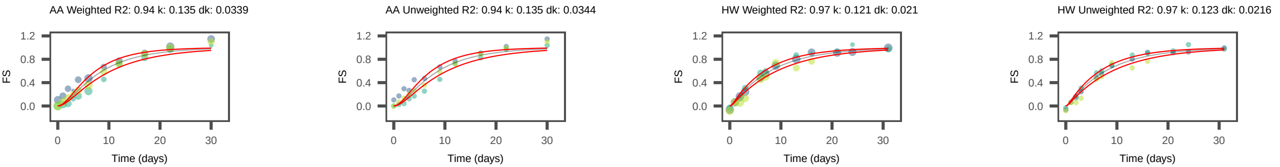

FAHD2

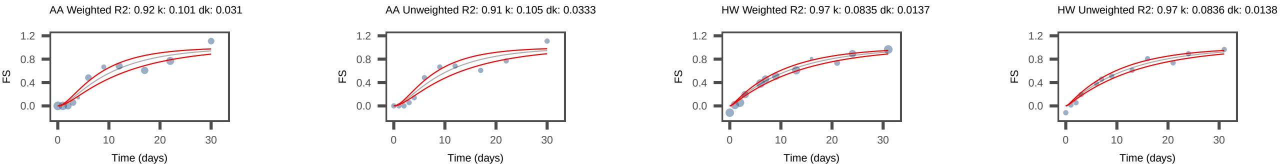

FAS

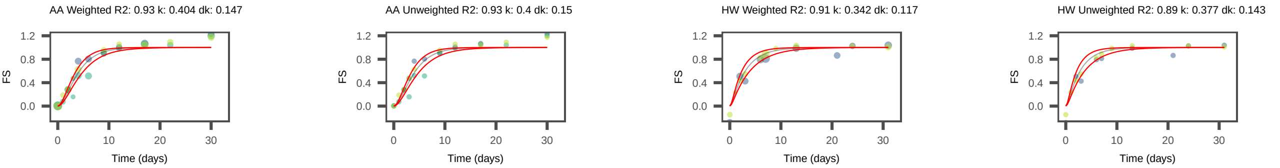

FERM2

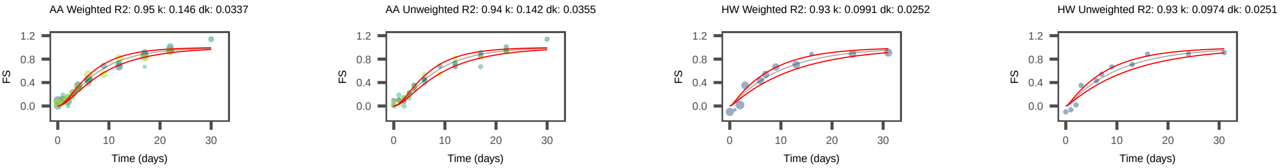

FHL1

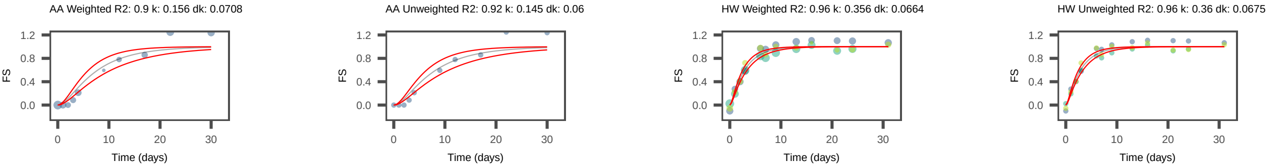

FIBA

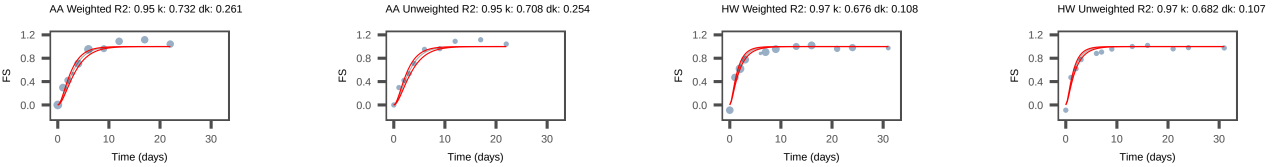

FIBG

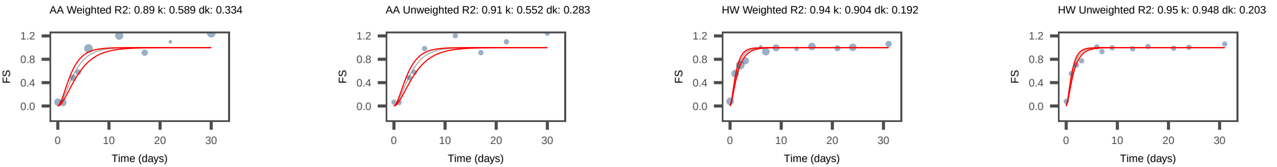

FIS1

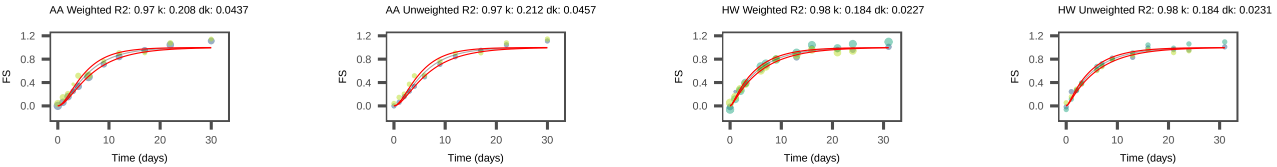

FLNA

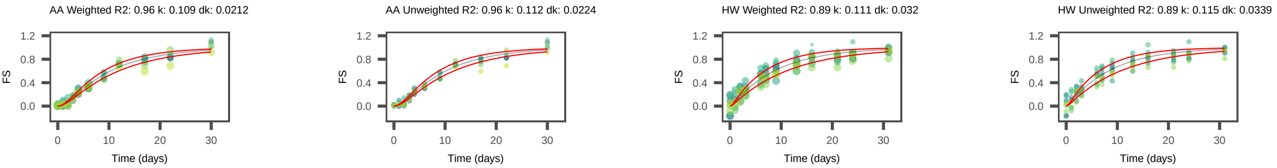

FLNB

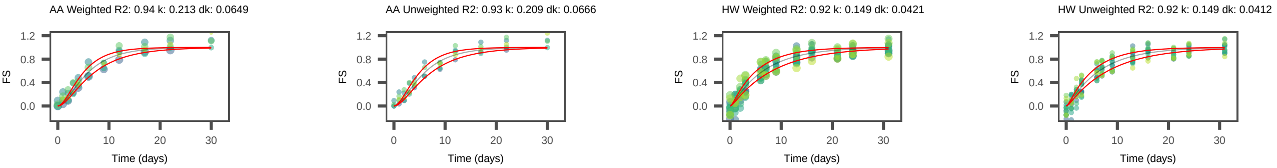

FMO1

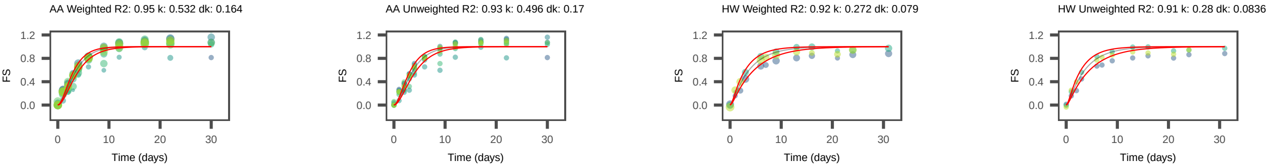

FMO2

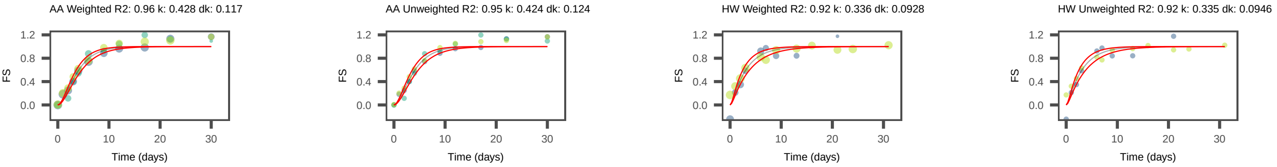

FMO4

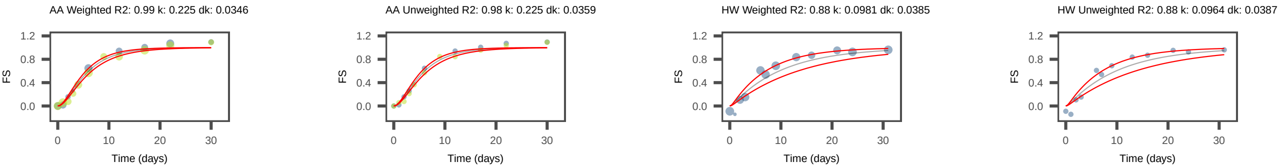

FMO5

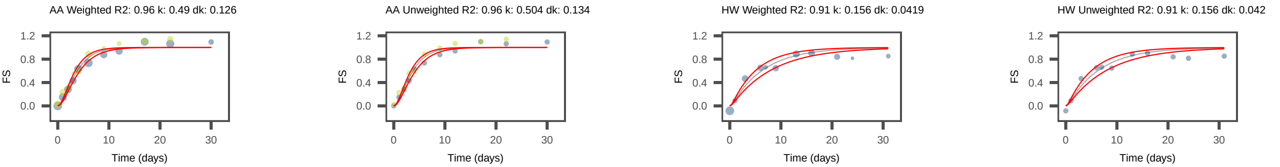

FOLH1

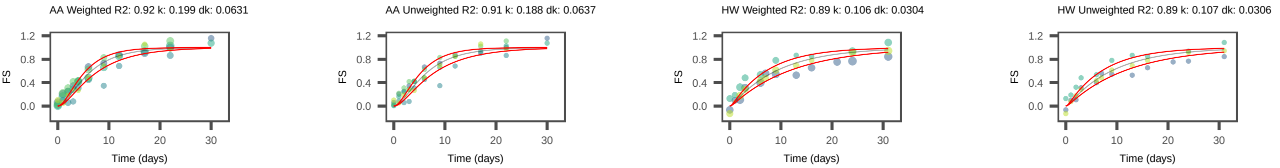

FUMH

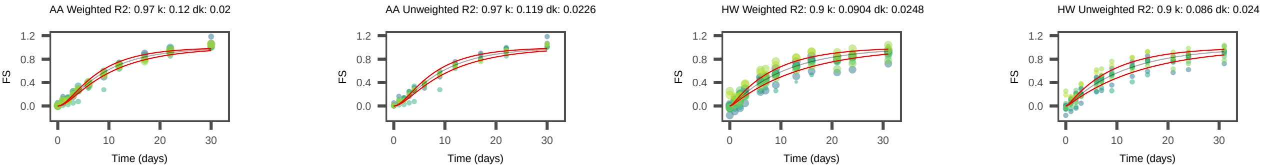

G3P

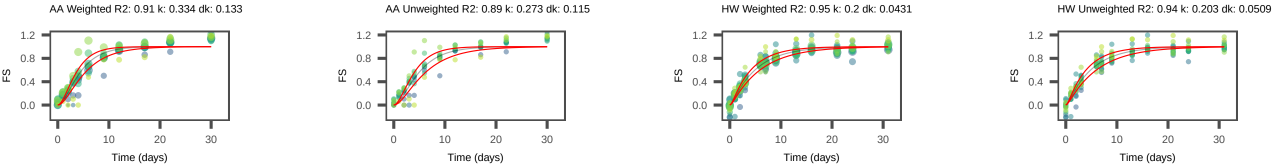

G6PI

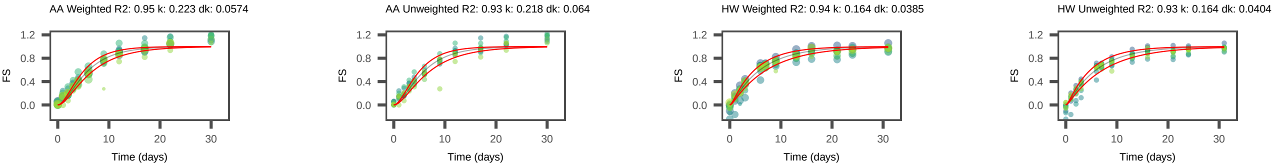

GABT

AA Weighted R2: 0.92 k: 0.145 dk: 0.0443 | AA Unweighted R2: 0.92 k: 0.14 dk: 0.0433 | HW Weighted R2: 0.85 k: 0.115 dk: 0.0478 | HW Unweighted R2: 0.83 k: 0.125 dk: 0.0561

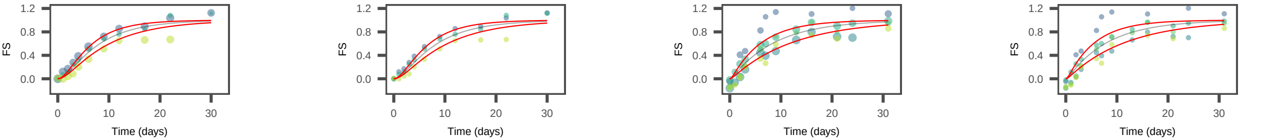

GAL3A

AA Weighted R2: 0.97 k: 0.104 dk: 0.017 | AA Unweighted R2: 0.97 k: 0.102 dk: 0.0171 | HW Weighted R2: 0.92 k: 0.117 dk: 0.03 | HW Unweighted R2: 0.92 k: 0.121 dk: 0.0316

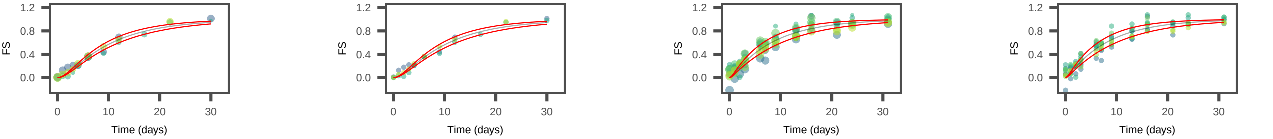

GALM

AA Weighted R2: 0.92 k: 0.109 dk: 0.0345 | AA Unweighted R2: 0.91 k: 0.114 dk: 0.0381 | HW Weighted R2: 0.96 k: 0.247 dk: 0.0518 | HW Unweighted R2: 0.96 k: 0.247 dk: 0.0509

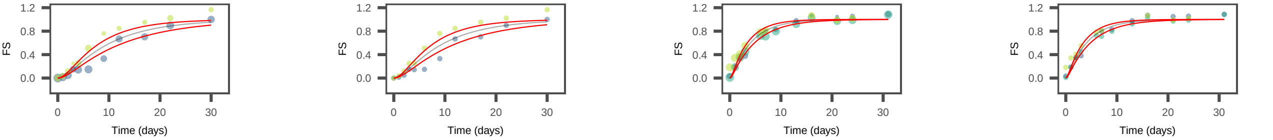

GALNS

AA Weighted R2: 0.95 k: 0.33 dk: 0.0973 | AA Unweighted R2: 0.95 k: 0.324 dk: 0.093 | HW Weighted R2: 0.91 k: 0.216 dk: 0.0634 | HW Unweighted R2: 0.91 k: 0.216 dk: 0.0637

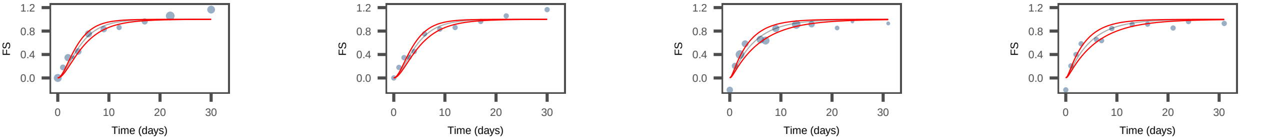

GANAB

AA Weighted R2: 0.96 k: 0.376 dk: 0.0899 | AA Unweighted R2: 0.96 k: 0.366 dk: 0.094 | HW Weighted R2: 0.96 k: 0.328 dk: 0.0715 | HW Unweighted R2: 0.96 k: 0.322 dk: 0.074

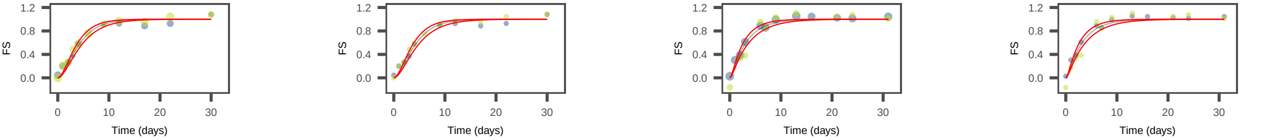

GAS2

AA Weighted R2: 0.96 k: 0.686 dk: 0.217 | AA Unweighted R2: 0.96 k: 0.678 dk: 0.209 | HW Weighted R2: 0.98 k: 0.506 dk: 0.0713 | HW Unweighted R2: 0.98 k: 0.512 dk: 0.0757

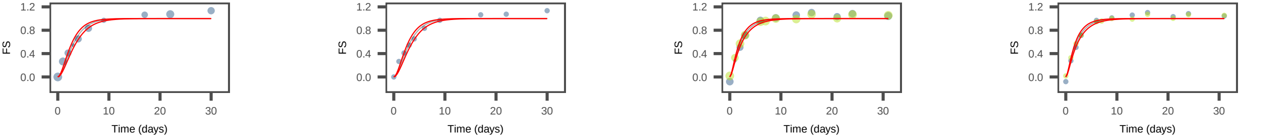

GATM

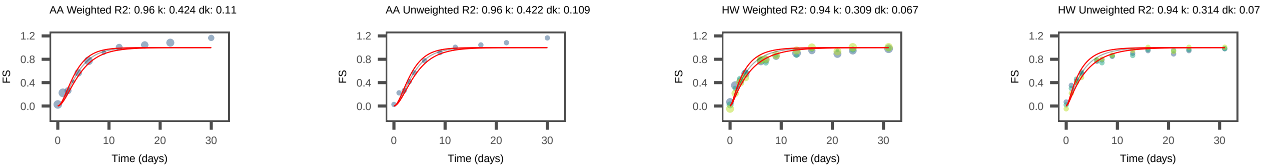

GCDH

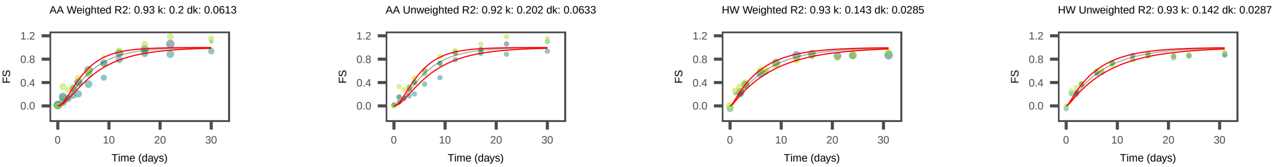

GCST

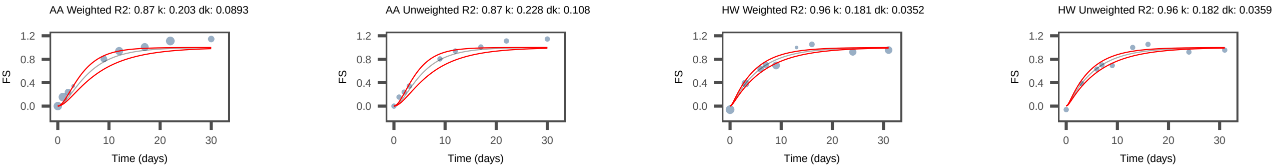

GDIA

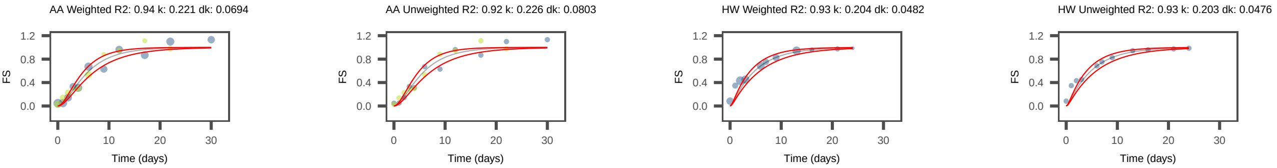

GDIB

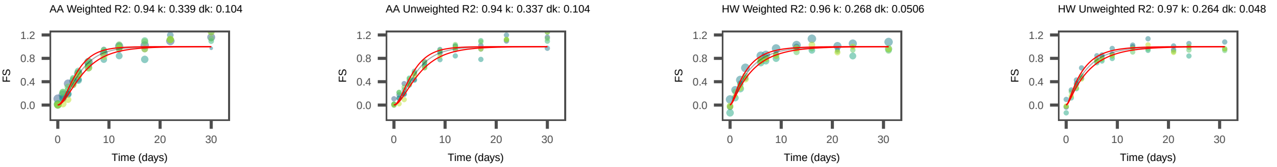

GDIR1

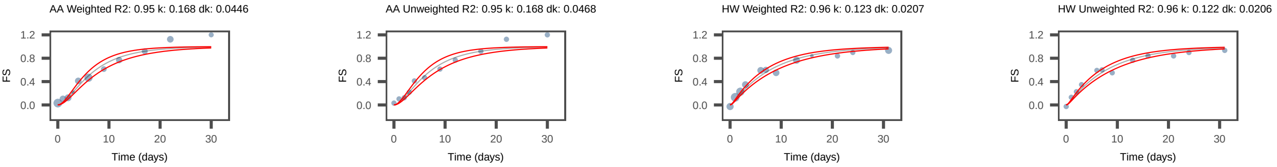

GGT1

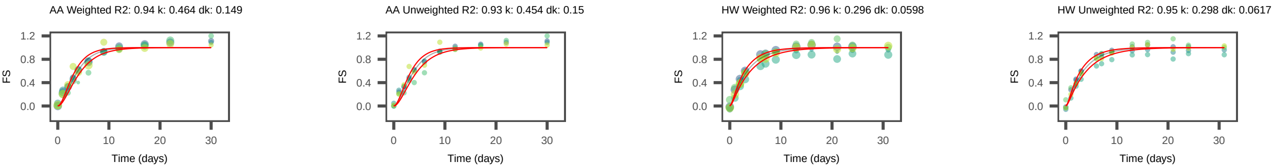

GLCTK

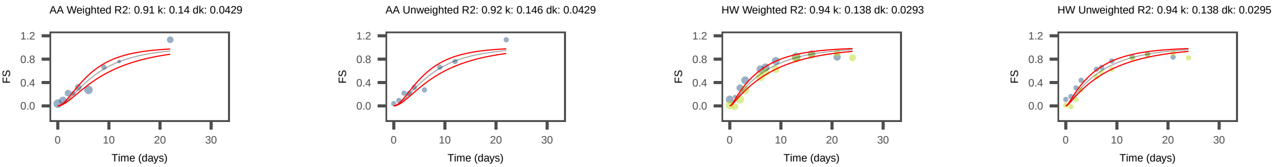

GLO2

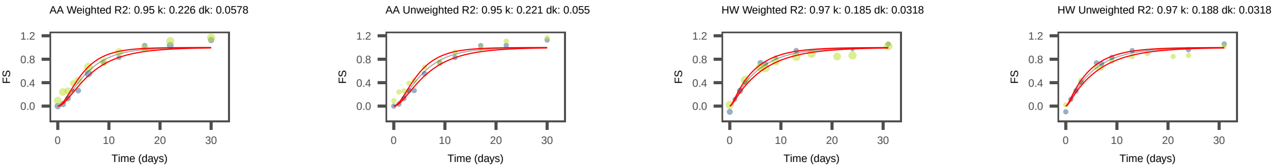

GLPK

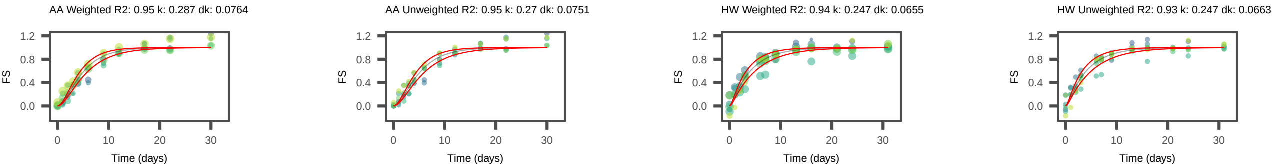

GLUCM

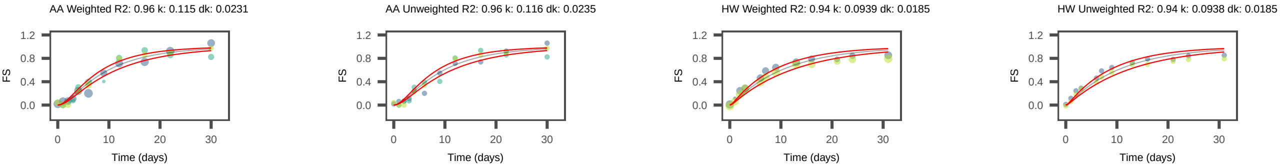

GLYAT

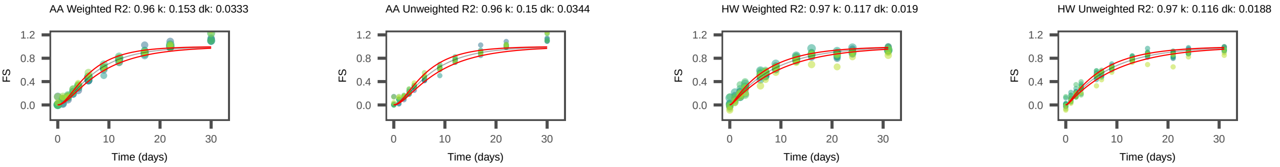

GLYM

AA Weighted R2: 0.92 k: 0.28 dk: 0.0952

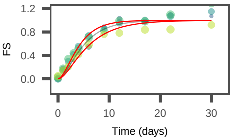

AA Unweighted R2: 0.91 k: 0.271 dk: 0.0937

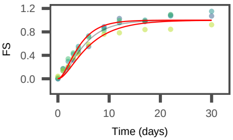

HW Weighted R2: 0.88 k: 0.209 dk: 0.0725

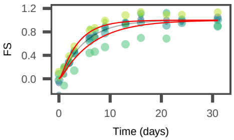

HW Unweighted R2: 0.89 k: 0.214 dk: 0.072

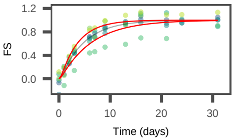

GPDA

AA Weighted R2: 0.96 k: 0.302 dk: 0.0803

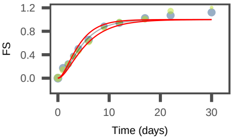

AA Unweighted R2: 0.96 k: 0.303 dk: 0.0823

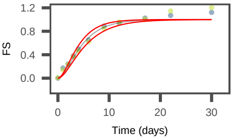

HW Weighted R2: 0.93 k: 0.201 dk: 0.0519

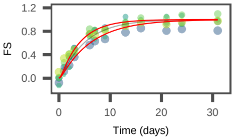

HW Unweighted R2: 0.93 k: 0.205 dk: 0.0509

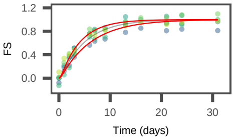

GPDM

AA Weighted R2: 0.97 k: 0.135 dk: 0.024

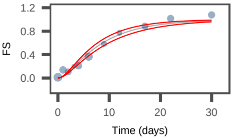

AA Unweighted R2: 0.97 k: 0.136 dk: 0.0238

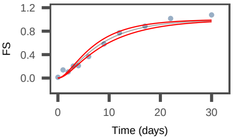

HW Weighted R2: 0.88 k: 0.138 dk: 0.0464

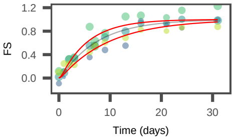

HW Unweighted R2: 0.88 k: 0.135 dk: 0.0454

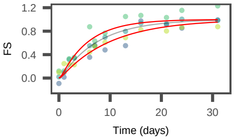

GPX1

AA Weighted R2: 0.96 k: 0.187 dk: 0.0413

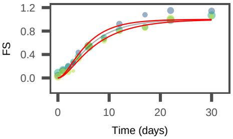

AA Unweighted R2: 0.96 k: 0.186 dk: 0.0416

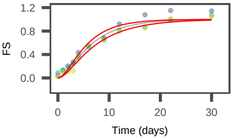

HW Weighted R2: 0.93 k: 0.114 dk: 0.0243

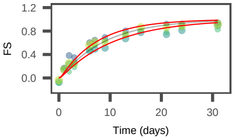

HW Unweighted R2: 0.93 k: 0.113 dk: 0.0245

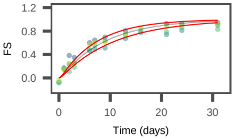

GPX3

AA Weighted R2: 0.93 k: 5.06 dk: 6.17

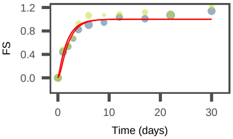

AA Unweighted R2: 0.92 k: 4.34 dk: 4.74

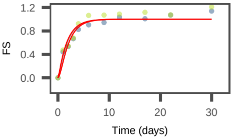

HW Weighted R2: 0.92 k: 0.836 dk: 0.19

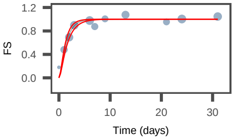

HW Unweighted R2: 0.92 k: 0.84 dk: 0.199

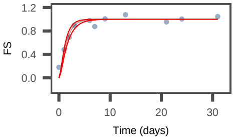

GRHPR

AA Weighted R2: 0.93 k: 0.163 dk: 0.0451

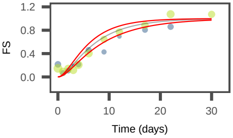

AA Unweighted R2: 0.93 k: 0.153 dk: 0.0416

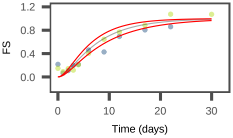

HW Weighted R2: 0.99 k: 0.142 dk: 0.0132

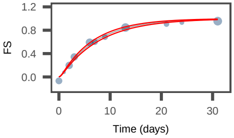

HW Unweighted R2: 0.99 k: 0.141 dk: 0.0132

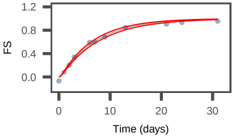

GRP75

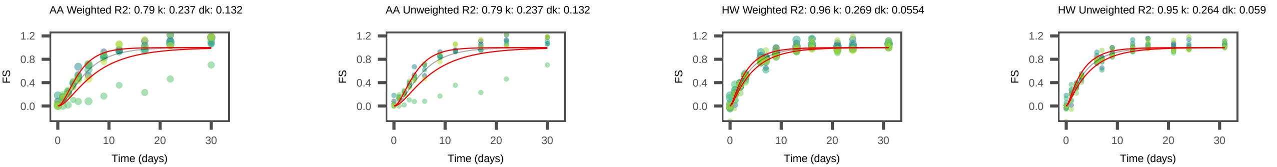

GSTA3

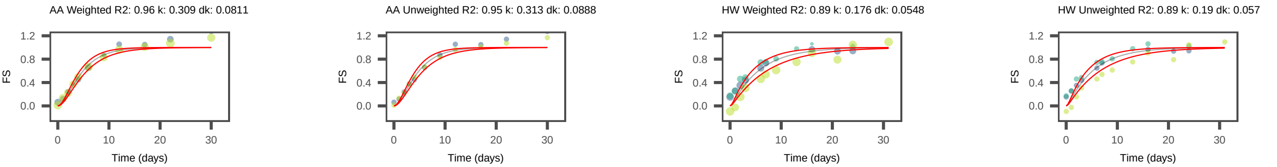

GSTK1

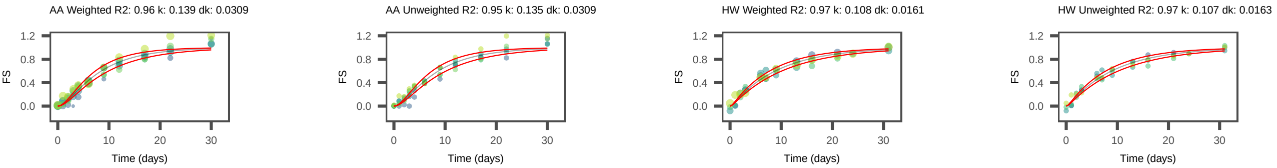

GSTM1

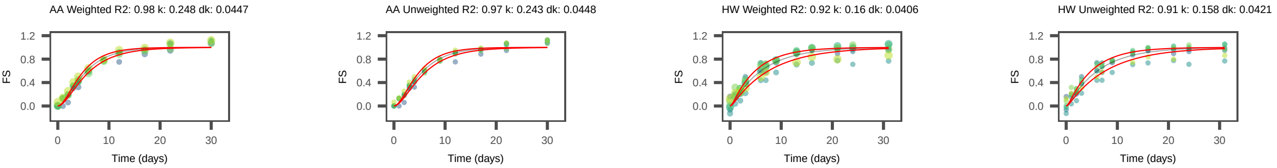

GSTM2

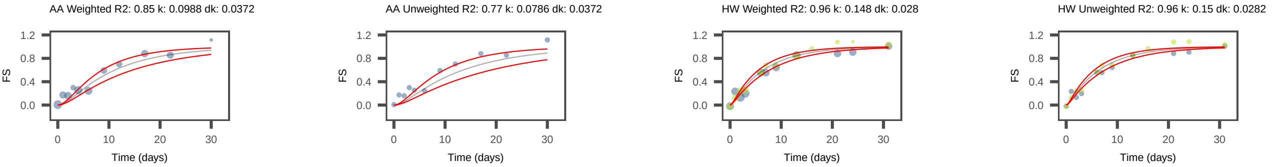

GSTT2

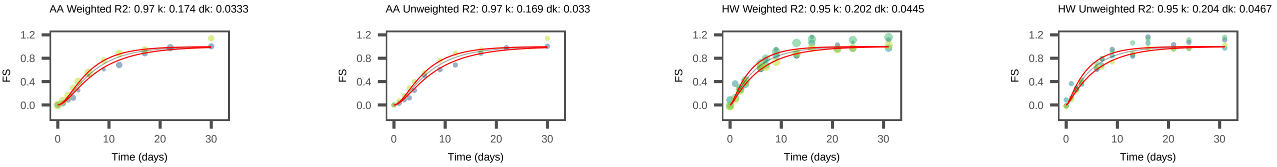

H10

AA Weighted R2: 0.98 k: 0.0348 dk: 0.00279

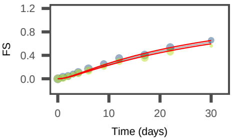

AA Unweighted R2: 0.98 k: 0.0345 dk: 0.00283

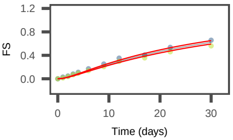

HW Weighted R2: 0.94 k: 0.0266 dk: 0.00335

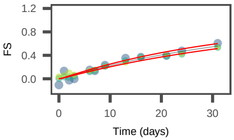

HW Unweighted R2: 0.95 k: 0.0264 dk: 0.00308

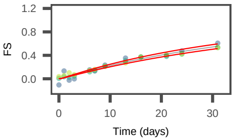

H14

AA Weighted R2: 0.98 k: 0.0344 dk: 0.00284

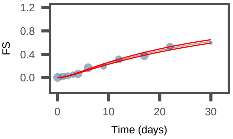

AA Unweighted R2: 0.96 k: 0.0349 dk: 0.00386

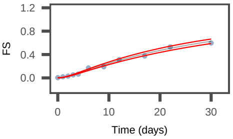

HW Weighted R2: 0.98 k: 0.0239 dk: 0.00149

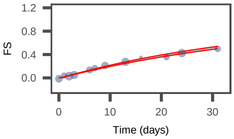

HW Unweighted R2: 0.98 k: 0.0239 dk: 0.0015

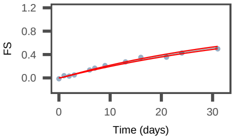

H2AY

AA Weighted R2: 0.88 k: 0.0575 dk: 0.0145

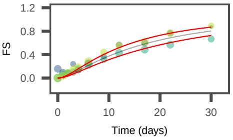

AA Unweighted R2: 0.85 k: 0.0555 dk: 0.0158

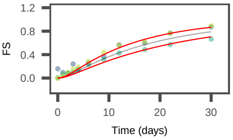

HW Weighted R2: 0.89 k: 0.0482 dk: 0.0121

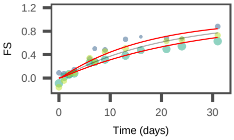

HW Unweighted R2: 0.88 k: 0.051 dk: 0.0132

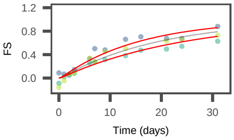

H4

AA Weighted R2: 0.93 k: 0.0158 dk: 0.00164

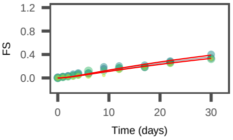

AA Unweighted R2: 0.93 k: 0.0155 dk: 0.00164

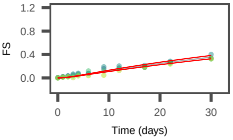

HW Weighted R2: 0.94 k: 0.0141 dk: 0.00131

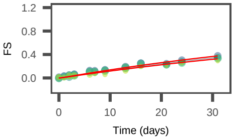

HW Unweighted R2: 0.94 k: 0.0139 dk: 0.00135

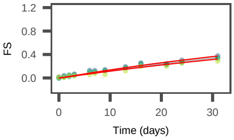

HACL2

AA Weighted R2: 0.94 k: 0.318 dk: 0.11

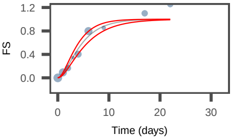

AA Unweighted R2: 0.94 k: 0.312 dk: 0.11

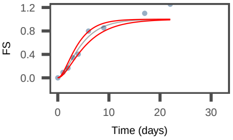

HW Weighted R2: 0.97 k: 0.364 dk: 0.0634

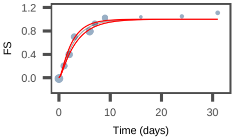

HW Unweighted R2: 0.97 k: 0.366 dk: 0.0643

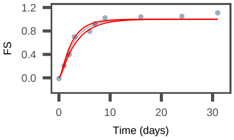

HAOX2

AA Weighted R2: 0.94 k: 0.102 dk: 0.0221

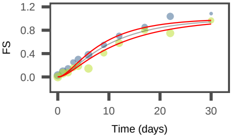

AA Unweighted R2: 0.95 k: 0.102 dk: 0.0218

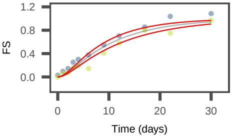

HW Weighted R2: 0.92 k: 0.172 dk: 0.0458

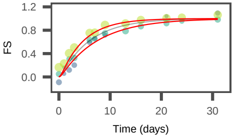

HW Unweighted R2: 0.93 k: 0.163 dk: 0.0424

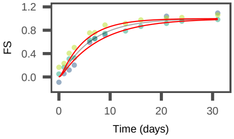

HBA

AA Weighted R2: 0.94 k: 0.0314 dk: 0.00426 | AA Unweighted R2: 0.94 k: 0.0316 dk: 0.00438 | HW Weighted R2: 0.93 k: 0.0259 dk: 0.00357 | HW Unweighted R2: 0.92 k: 0.0259 dk: 0.00389

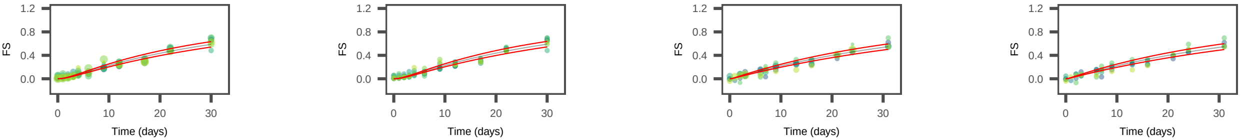

HBB1

AA Weighted R2: 0.95 k: 0.032 dk: 0.00422 | AA Unweighted R2: 0.95 k: 0.0314 dk: 0.00414 | HW Weighted R2: 0.96 k: 0.0237 dk: 0.00254 | HW Unweighted R2: 0.96 k: 0.0235 dk: 0.00264

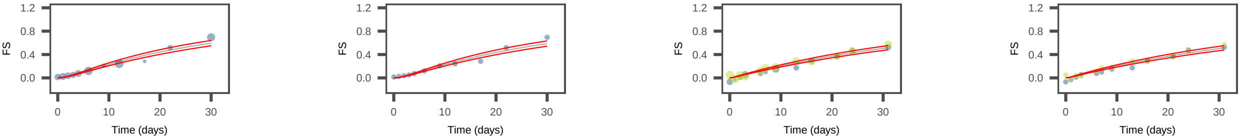

HCD2

AA Weighted R2: 0.87 k: 0.123 dk: 0.0461 | AA Unweighted R2: 0.89 k: 0.108 dk: 0.0359 | HW Weighted R2: 0.95 k: 0.1 dk: 0.019 | HW Unweighted R2: 0.95 k: 0.0983 dk: 0.0179

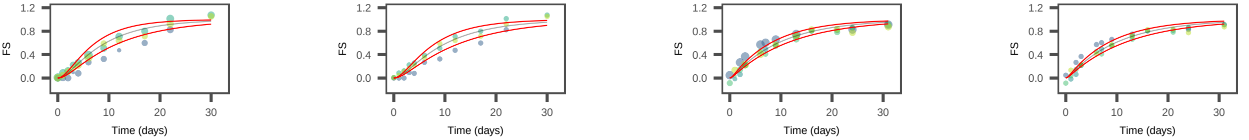

HCDH

AA Weighted R2: 0.92 k: 0.134 dk: 0.037 | AA Unweighted R2: 0.91 k: 0.134 dk: 0.0403 | HW Weighted R2: 0.95 k: 0.103 dk: 0.0207 | HW Unweighted R2: 0.95 k: 0.105 dk: 0.0224

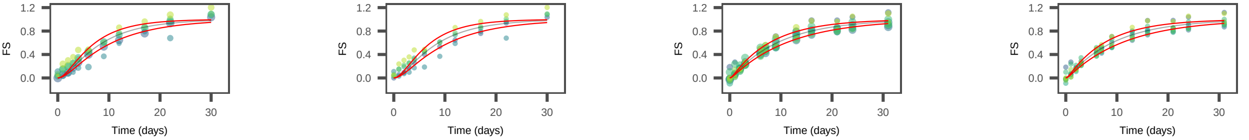

HEM2

AA Weighted R2: 0.92 k: 0.415 dk: 0.17 | AA Unweighted R2: 0.91 k: 0.428 dk: 0.189 | HW Weighted R2: 0.96 k: 0.224 dk: 0.0459 | HW Unweighted R2: 0.96 k: 0.23 dk: 0.0465

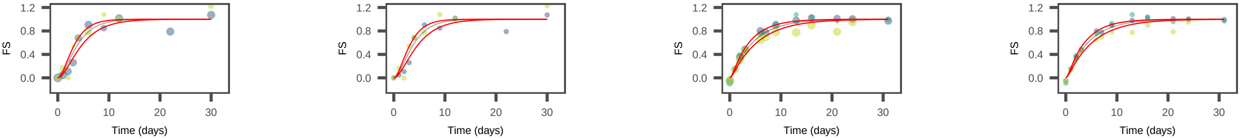

HEMH

AA Weighted R2: 0.92 k: 0.117 dk: 0.0339 | AA Unweighted R2: 0.92 k: 0.122 dk: 0.035 | HW Weighted R2: 0.99 k: 0.148 dk: 0.0162 | HW Unweighted R2: 0.99 k: 0.148 dk: 0.0161

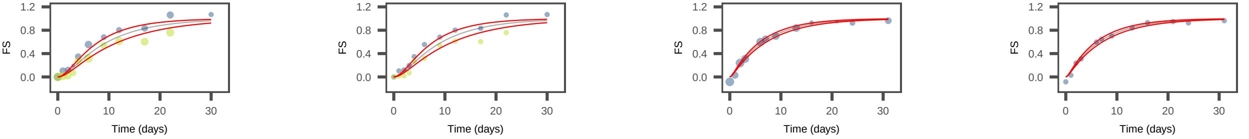

HGD

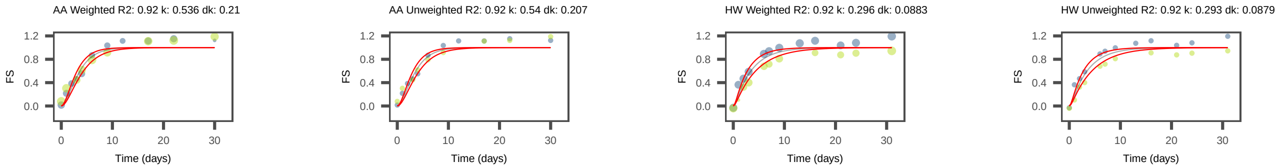

HIBCH

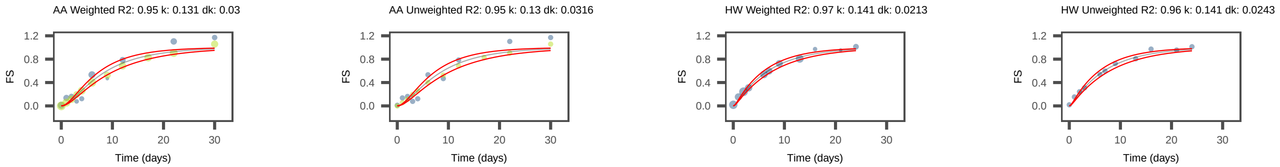

HINT1

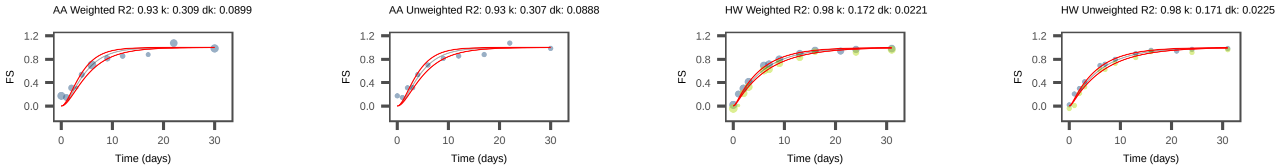

HINT2

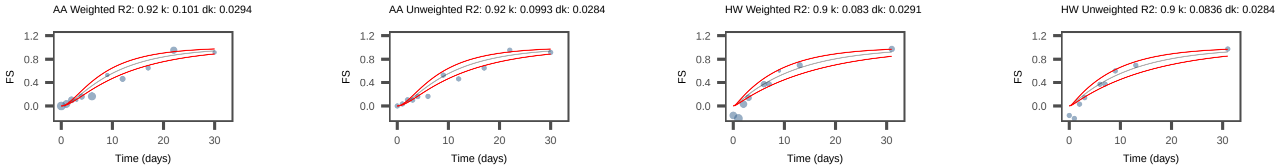

HMGCL

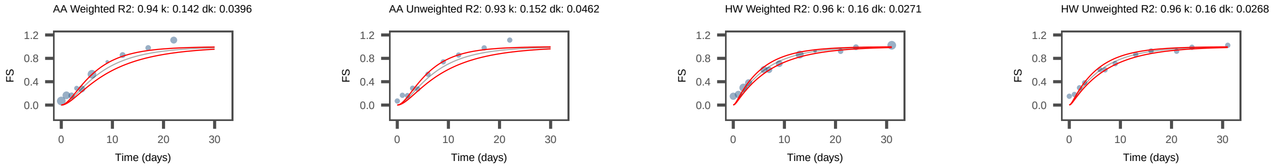

HNRPK

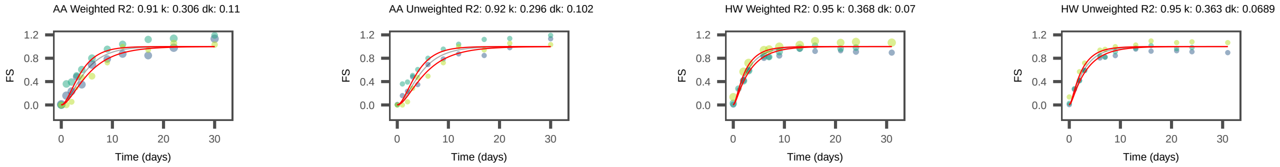

HNRPL

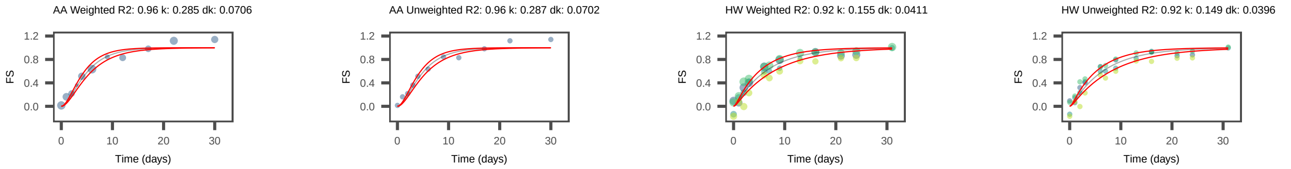

HNRPU

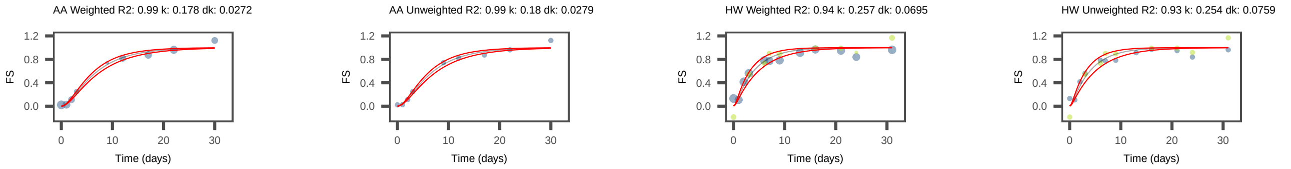

HOGA1

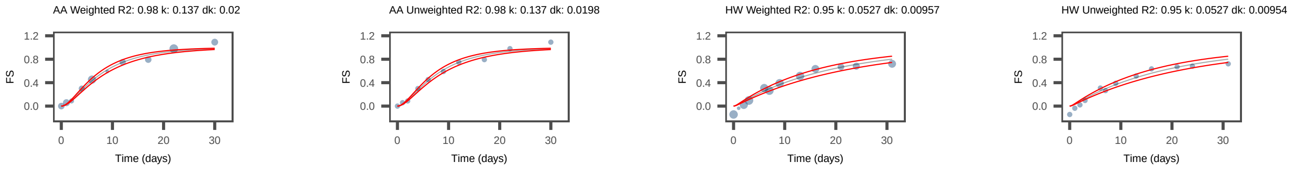

HPRT

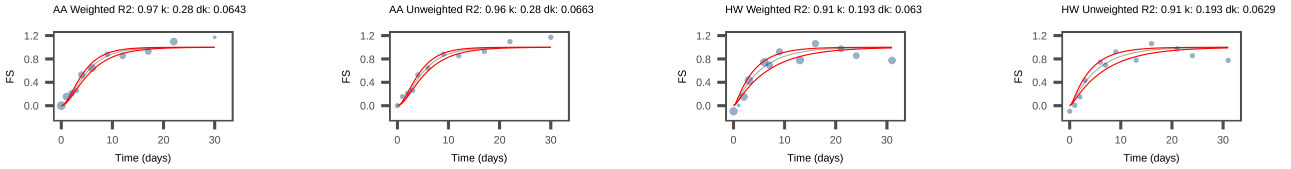

HS90A

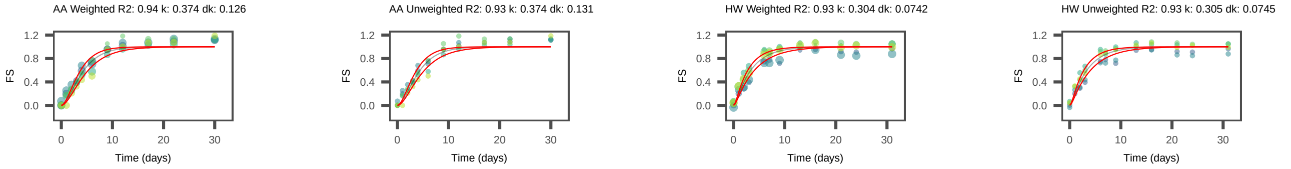

HS90B

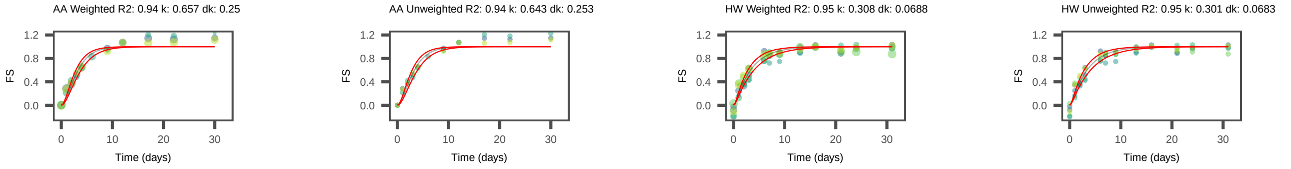

HSP74

AA Weighted R2: 0.95 k: 0.221 dk: 0.0647

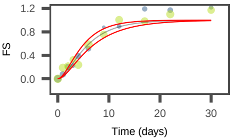

AA Unweighted R2: 0.94 k: 0.227 dk: 0.0717

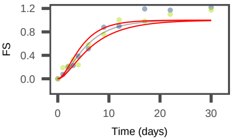

HW Weighted R2: 0.96 k: 0.31 dk: 0.0494

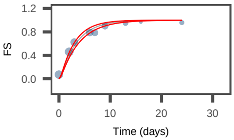

HW Unweighted R2: 0.96 k: 0.308 dk: 0.0488

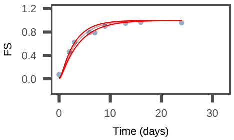

HSP7C

AA Weighted R2: 0.92 k: 0.849 dk: 0.397

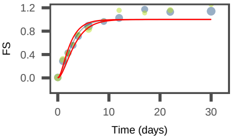

AA Unweighted R2: 0.92 k: 0.842 dk: 0.402

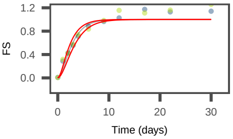

HW Weighted R2: 0.95 k: 0.494 dk: 0.0951

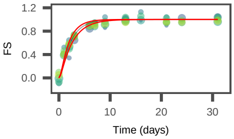

HW Unweighted R2: 0.94 k: 0.494 dk: 0.105

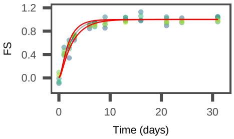

HYEP

AA Weighted R2: 0.97 k: 0.319 dk: 0.0641

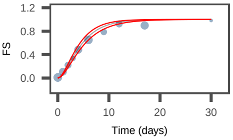

AA Unweighted R2: 0.97 k: 0.325 dk: 0.0673

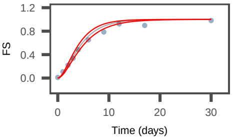

HW Weighted R2: 0.98 k: 0.175 dk: 0.0222

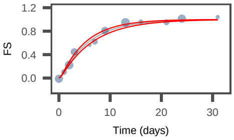

HW Unweighted R2: 0.98 k: 0.174 dk: 0.0221

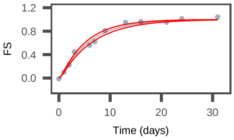

HYES

AA Weighted R2: 0.94 k: 0.223 dk: 0.0591

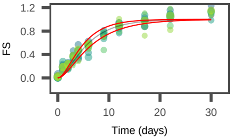

AA Unweighted R2: 0.93 k: 0.218 dk: 0.0624

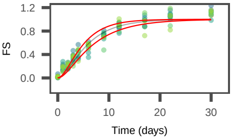

HW Weighted R2: 0.96 k: 0.168 dk: 0.0305

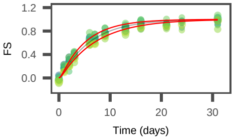

HW Unweighted R2: 0.96 k: 0.17 dk: 0.0311

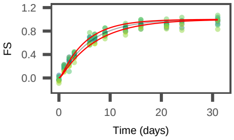

HYKK

AA Weighted R2: 0.94 k: 0.336 dk: 0.116

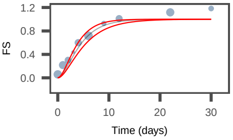

AA Unweighted R2: 0.94 k: 0.32 dk: 0.108

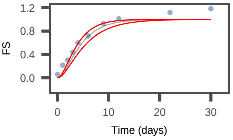

HW Weighted R2: 0.95 k: 0.26 dk: 0.0577

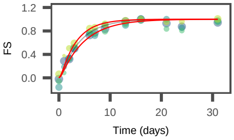

HW Unweighted R2: 0.94 k: 0.261 dk: 0.0605

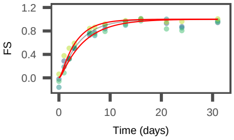

IAH1

AA Weighted R2: 0.9 k: 0.109 dk: 0.0433

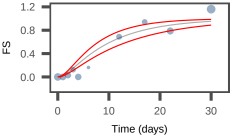

AA Unweighted R2: 0.91 k: 0.0994 dk: 0.0371

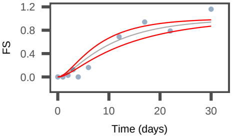

HW Weighted R2: 0.96 k: 0.245 dk: 0.0542

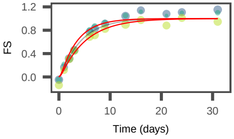

HW Unweighted R2: 0.96 k: 0.249 dk: 0.0559

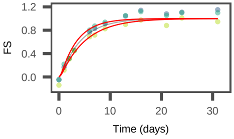

IDHC

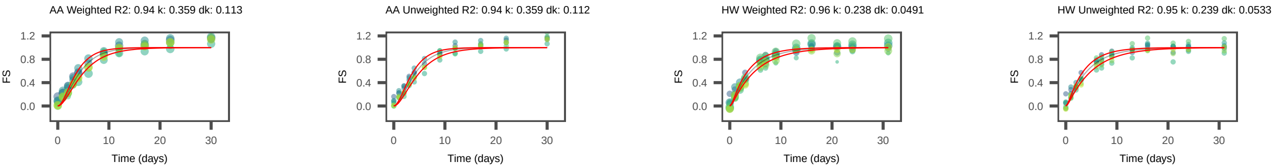

IDHG1

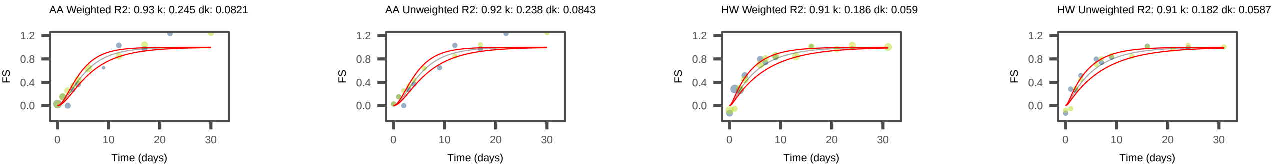

IDHP

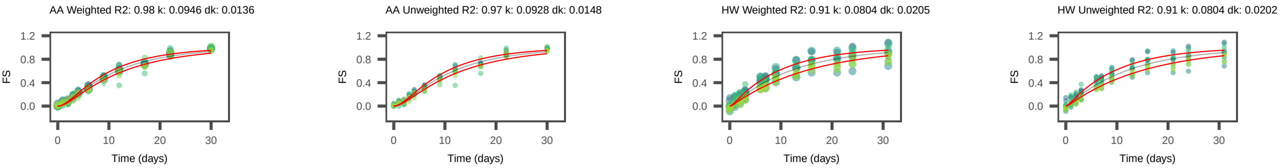

IF4G1

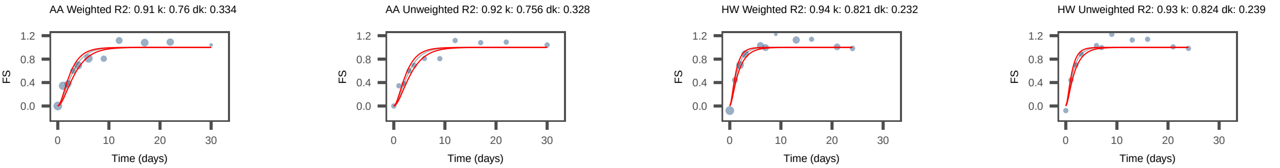

ILK

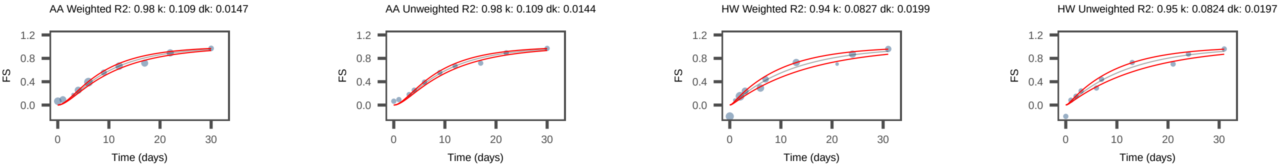

INMT

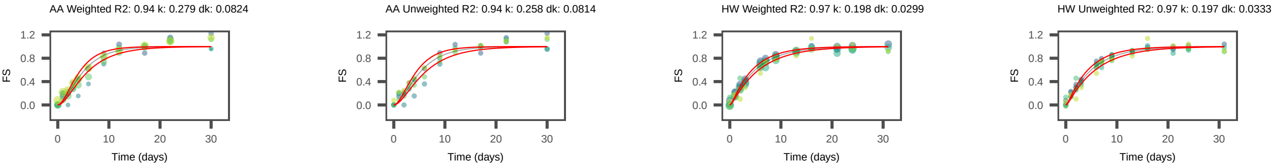

IPYR2

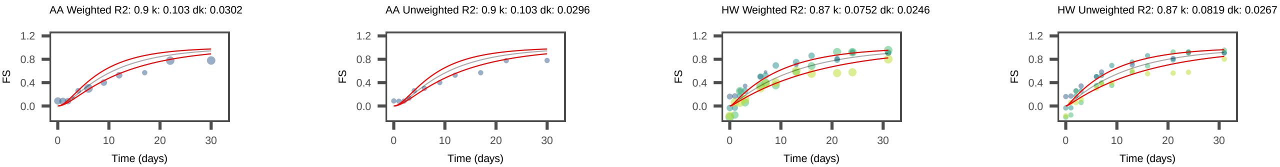

IQGA1

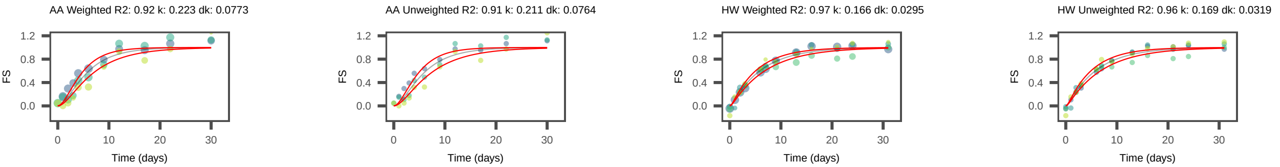

IRGM1

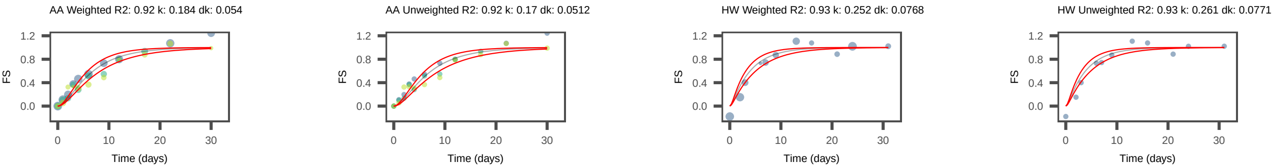

ISC2A

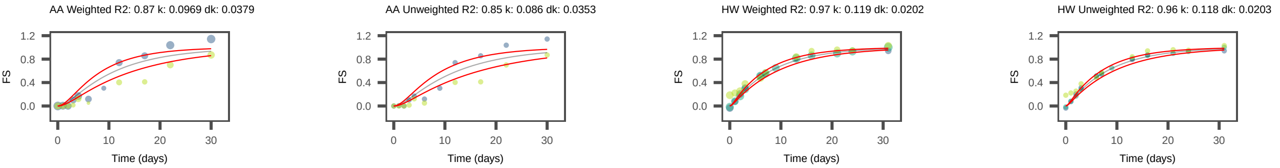

ITB1

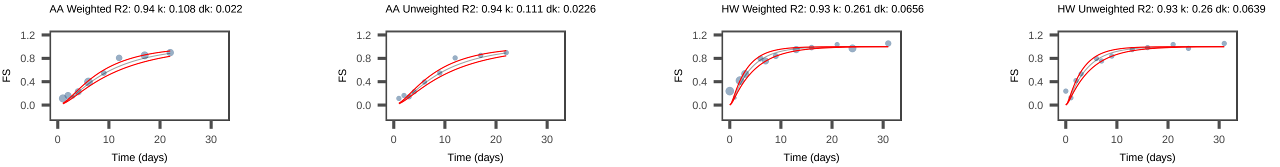

IVD

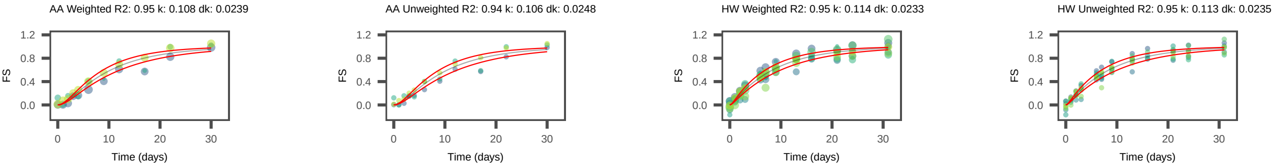

KAD2

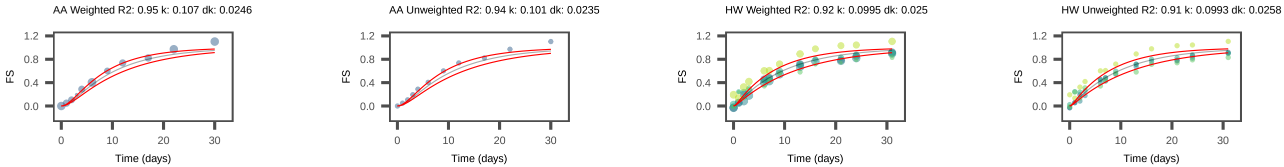

KAD3

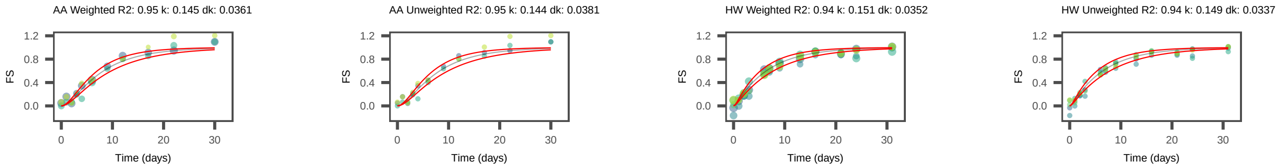

KAD4

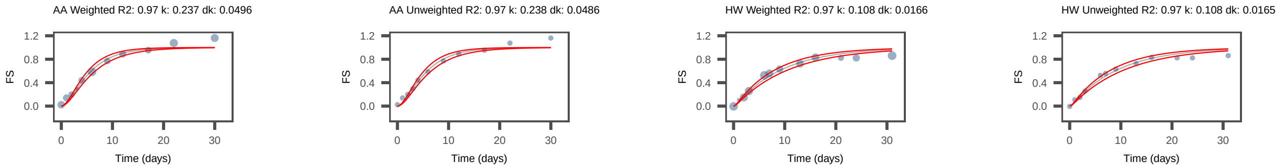

KAT1

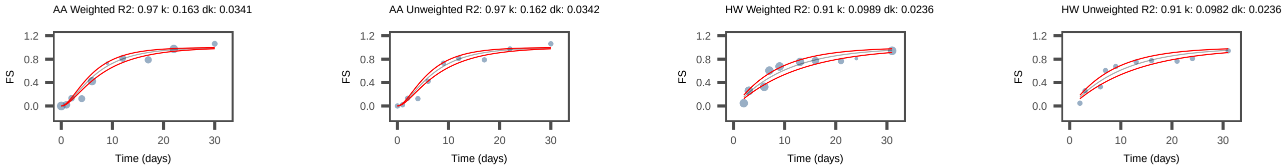

KAT3

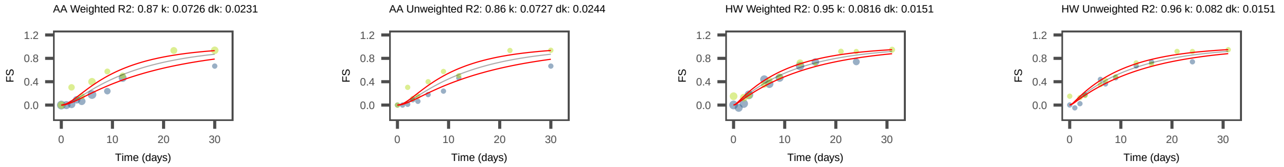

KCRU

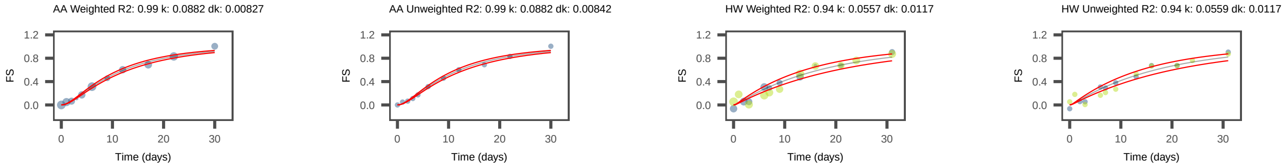

KEG1

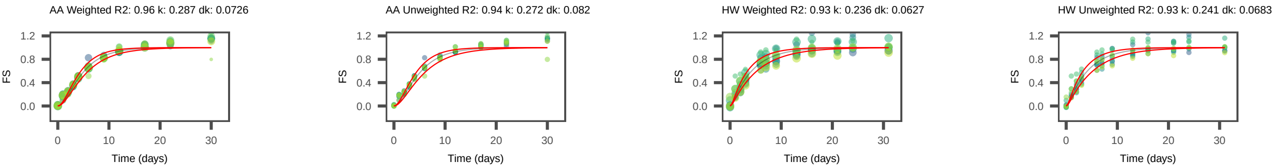

KHK

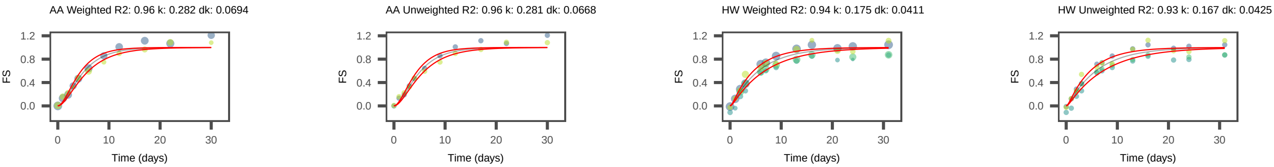

KMO

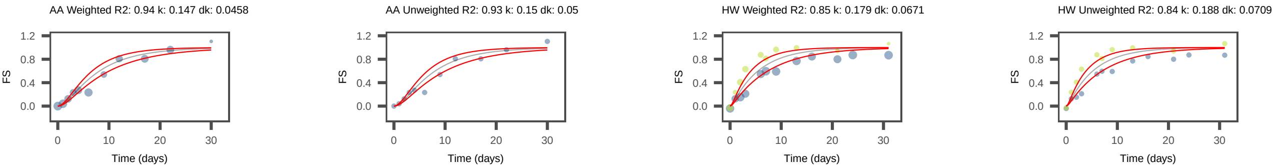

KPYM

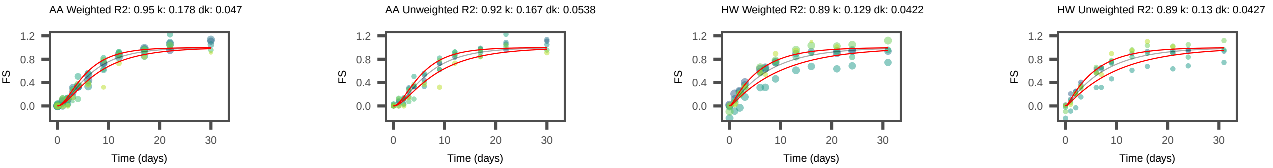

LA

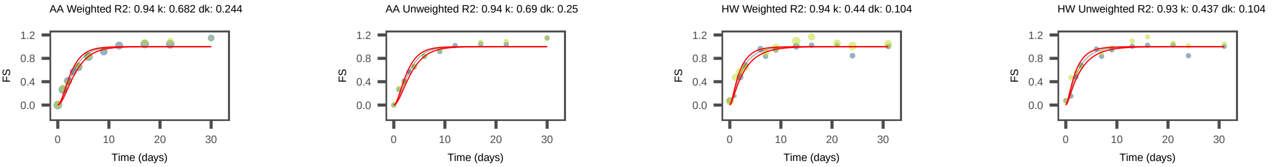

LACB2

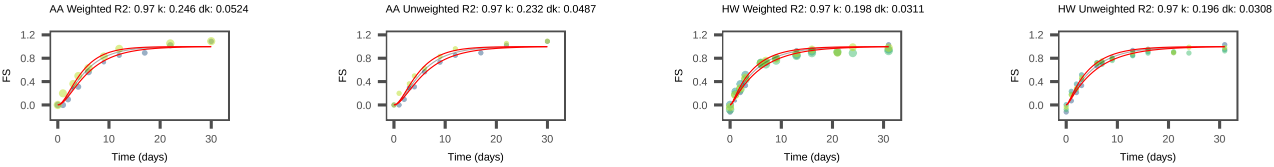

LACTB

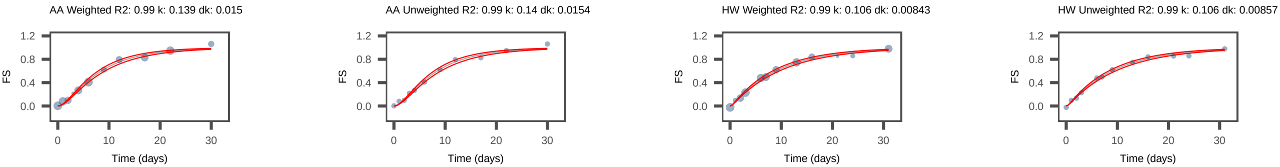

LAD1

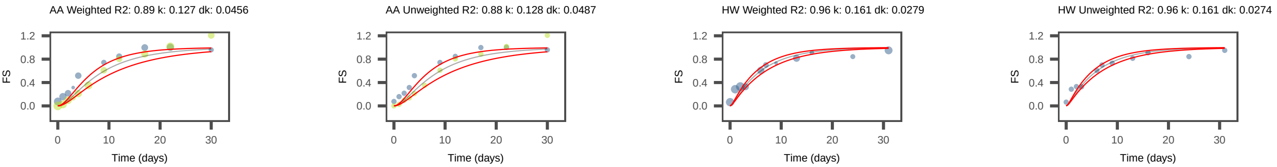

LAT4

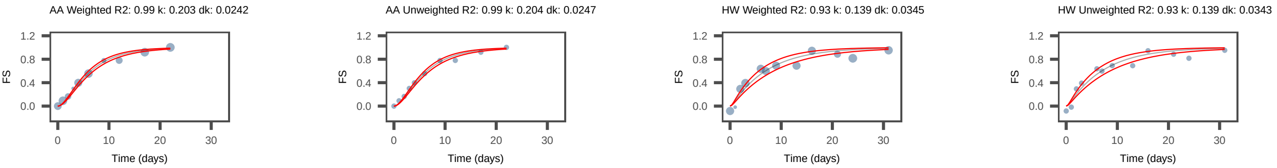

LDHA

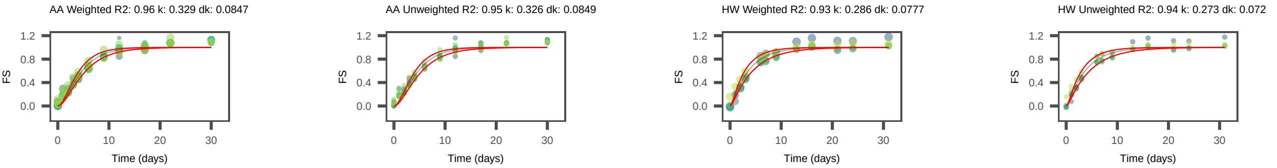

LDHB

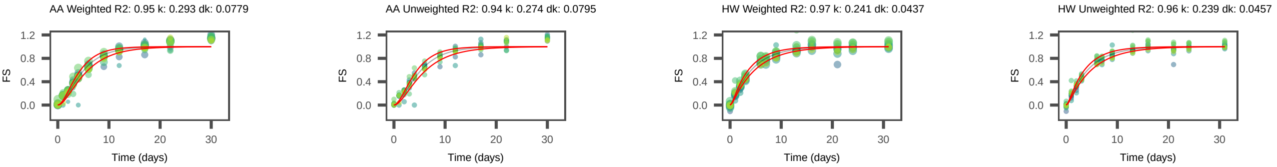

LDHD

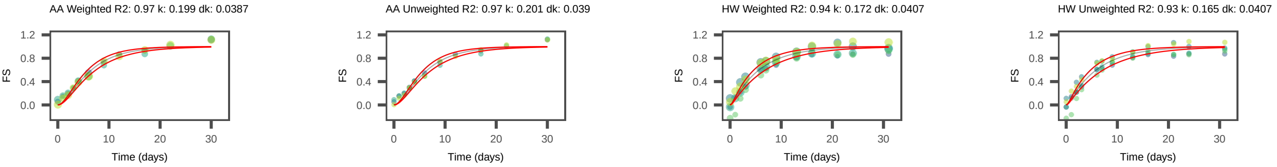

LETM1

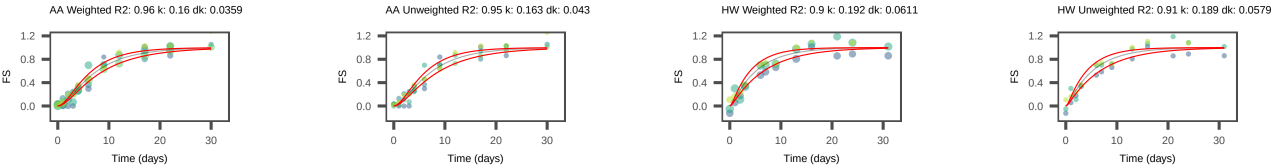

LKHA4

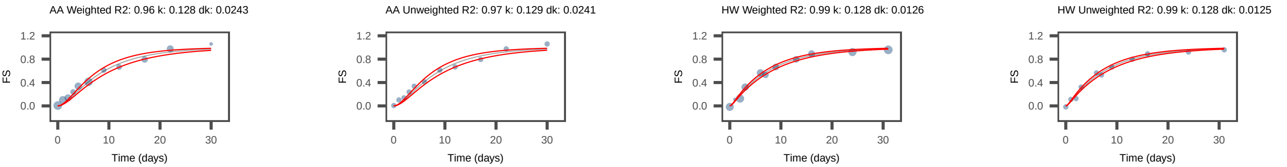

LMNA

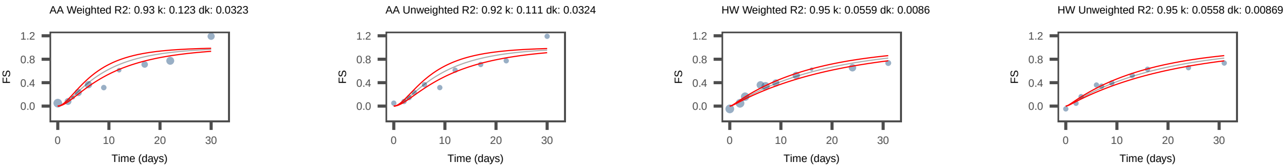

LPPRC

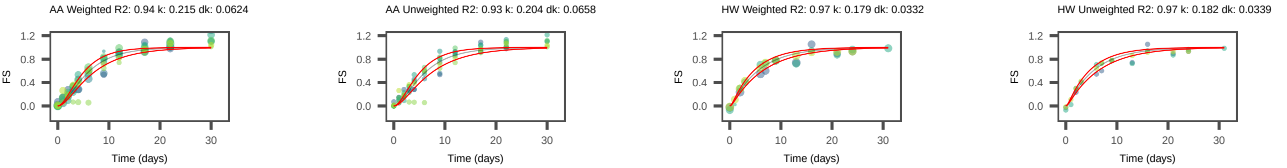

LRC59

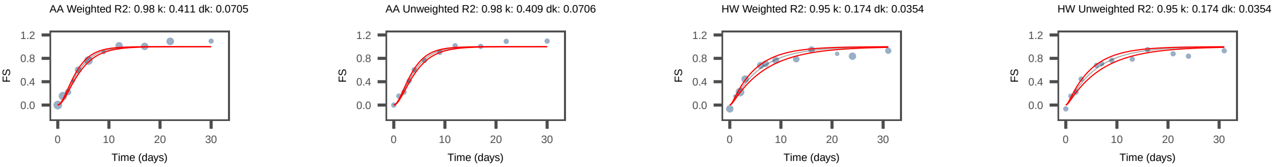

LRP2

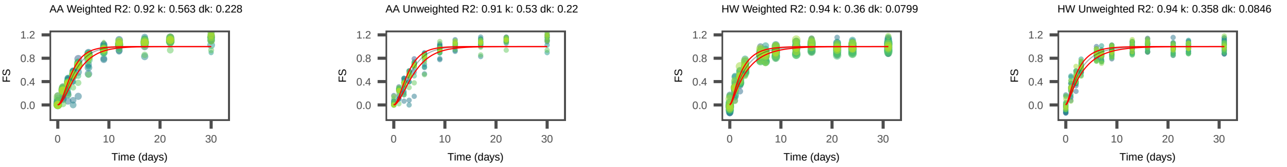

M2GD

AA Weighted R2: 0.94 k: 0.112 dk: 0.0258 | AA Unweighted R2: 0.92 k: 0.101 dk: 0.0269 | HW Weighted R2: 0.92 k: 0.125 dk: 0.0358 | HW Unweighted R2: 0.91 k: 0.131 dk: 0.0405

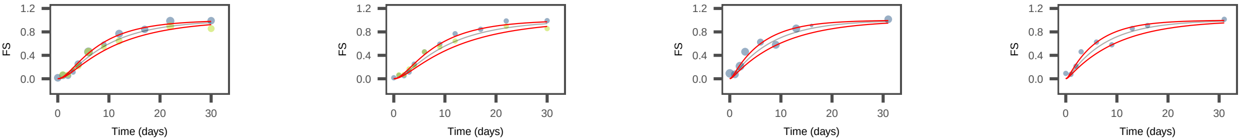

M2OM

AA Weighted R2: 0.94 k: 0.107 dk: 0.0242 | AA Unweighted R2: 0.89 k: 0.095 dk: 0.029 | HW Weighted R2: 0.93 k: 0.0707 dk: 0.0165 | HW Unweighted R2: 0.92 k: 0.0729 dk: 0.0176

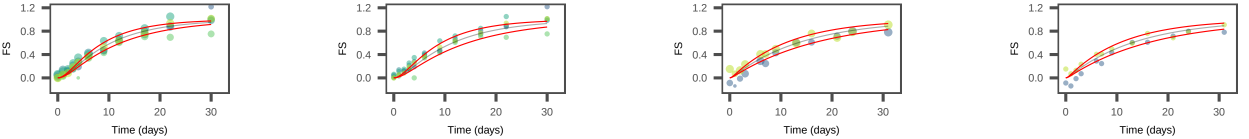

MAAI

AA Weighted R2: 0.95 k: 0.23 dk: 0.059 | AA Unweighted R2: 0.95 k: 0.226 dk: 0.0613 | HW Weighted R2: 0.98 k: 0.181 dk: 0.0217 | HW Unweighted R2: 0.98 k: 0.182 dk: 0.022

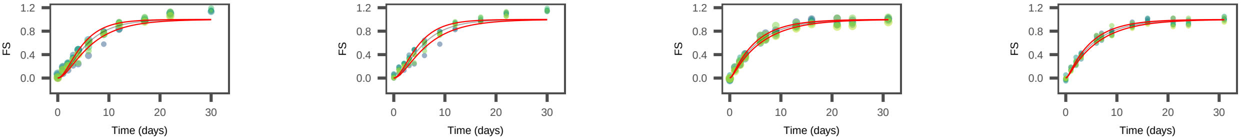

MAOX

AA Weighted R2: 0.96 k: 0.302 dk: 0.0758 | AA Unweighted R2: 0.96 k: 0.302 dk: 0.0776 | HW Weighted R2: 0.96 k: 0.271 dk: 0.0548 | HW Unweighted R2: 0.96 k: 0.268 dk: 0.0532

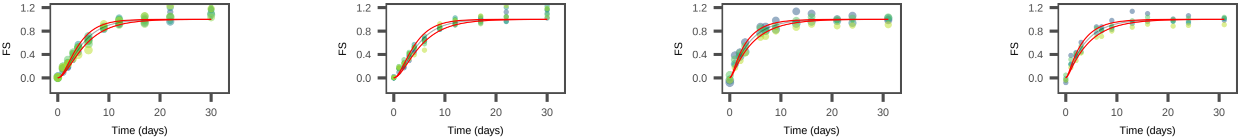

MARC2

AA Weighted R2: 0.97 k: 0.216 dk: 0.0403 | AA Unweighted R2: 0.97 k: 0.216 dk: 0.0427 | HW Weighted R2: 0.97 k: 0.185 dk: 0.0268 | HW Unweighted R2: 0.98 k: 0.183 dk: 0.026

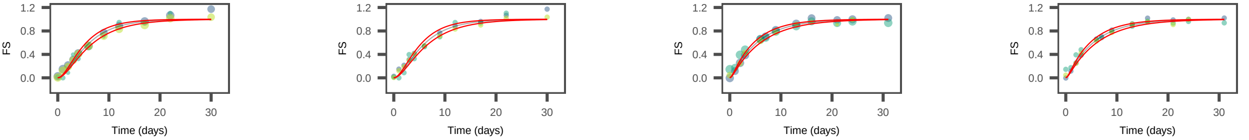

MCCA

AA Weighted R2: 0.98 k: 0.156 dk: 0.0226 | AA Unweighted R2: 0.98 k: 0.156 dk: 0.024 | HW Weighted R2: 0.93 k: 0.143 dk: 0.0399 | HW Unweighted R2: 0.92 k: 0.144 dk: 0.043

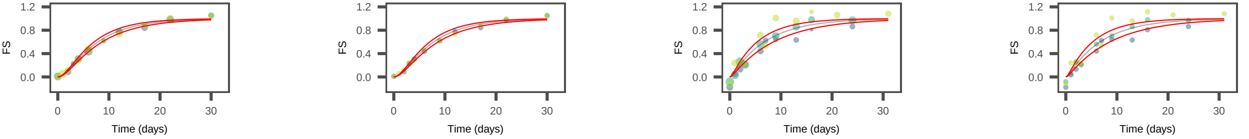

MCCB

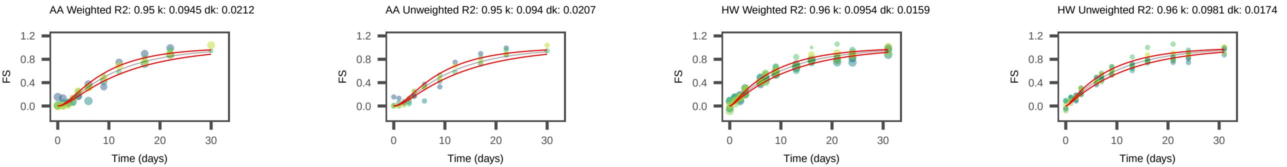

MDHC

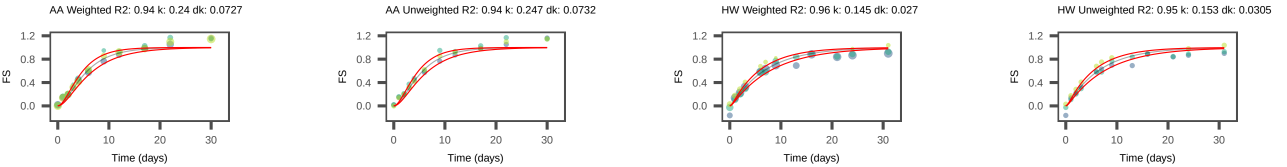

MDHM

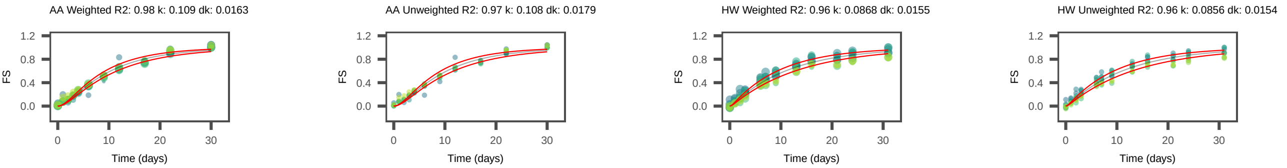

MECP2

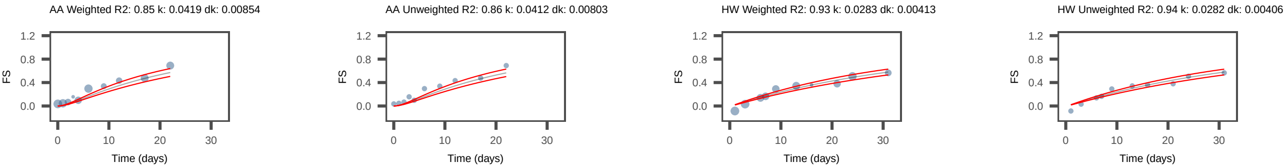

MECR

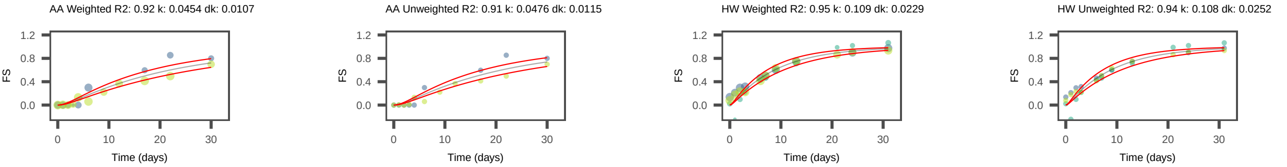

MEP1A

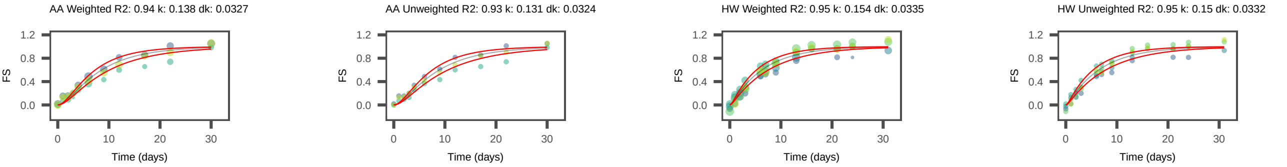

MEP1B

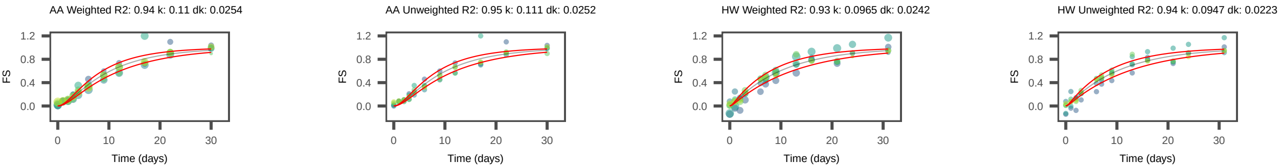

MIC13

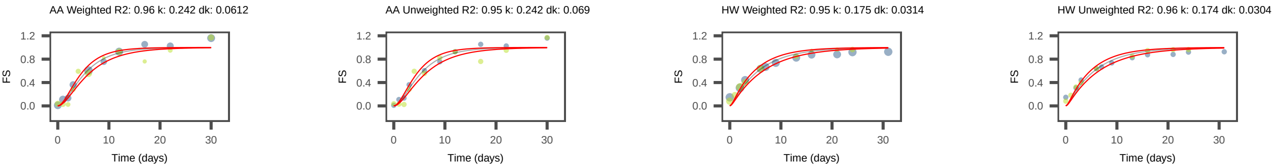

MIC19

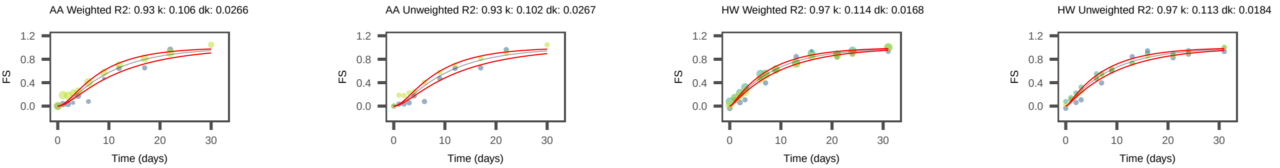

MIC26

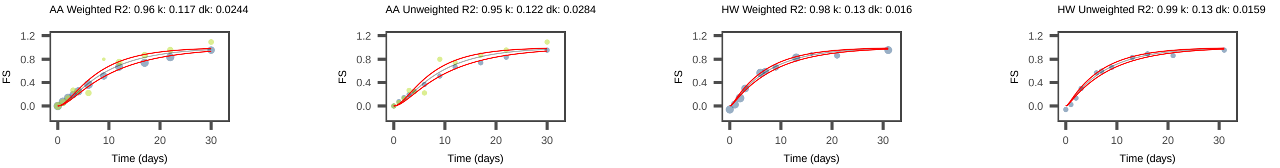

MIC27

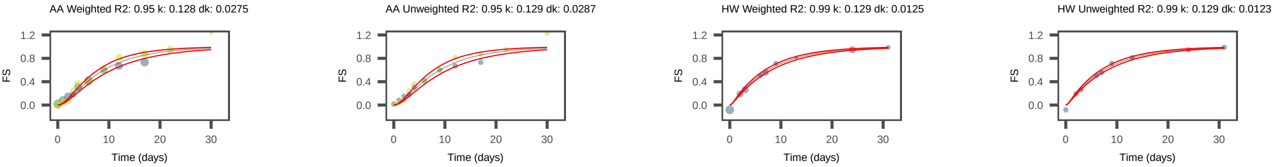

MIC60

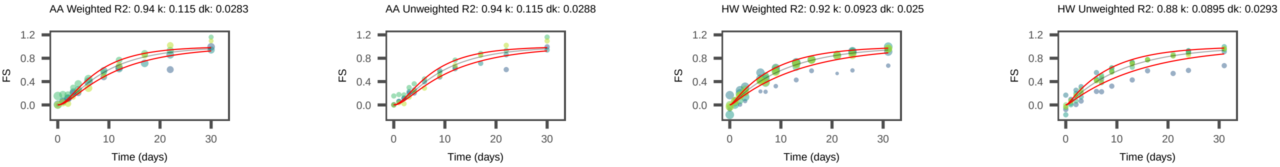

MIF

AA Weighted R2: 0.94 k: 0.215 dk: 0.0628 | AA Unweighted R2: 0.94 k: 0.214 dk: 0.0614 | HW Weighted R2: 0.99 k: 0.149 dk: 0.00978 | HW Unweighted R2: 0.99 k: 0.149 dk: 0.00998

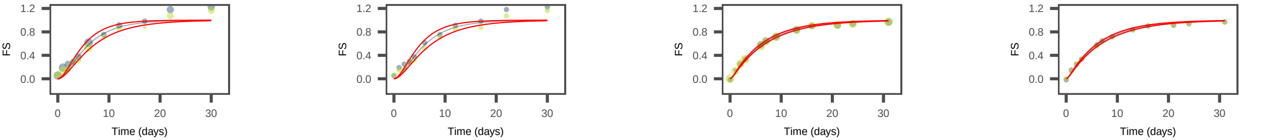

MIOX

AA Weighted R2: 0.92 k: 19.1 dk: 107 | AA Unweighted R2: 0.91 k: 11 dk: 36 | HW Weighted R2: 0.91 k: 9.86 dk: 34.5 | HW Unweighted R2: 0.89 k: 9.94 dk: 37.4

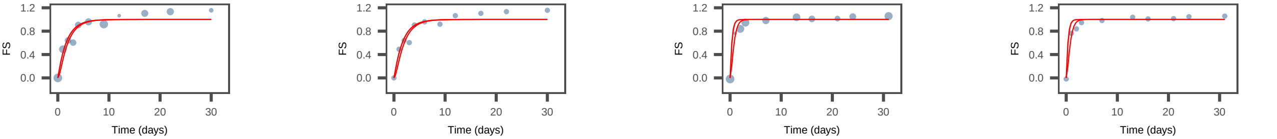

MLEC

AA Weighted R2: 0.98 k: 0.274 dk: 0.045 | AA Unweighted R2: 0.98 k: 0.273 dk: 0.0445 | HW Weighted R2: 0.93 k: 0.211 dk: 0.0568 | HW Unweighted R2: 0.92 k: 0.216 dk: 0.0621

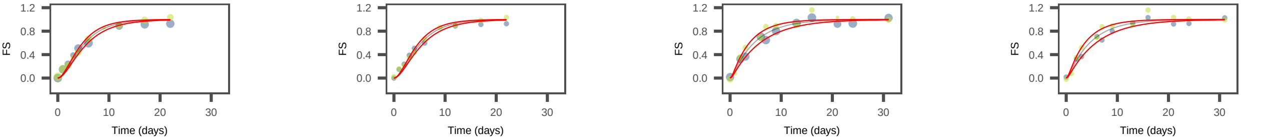

MMSA

AA Weighted R2: 0.96 k: 0.13 dk: 0.027 | AA Unweighted R2: 0.96 k: 0.129 dk: 0.0283 | HW Weighted R2: 0.95 k: 0.1 dk: 0.0193 | HW Unweighted R2: 0.95 k: 0.0992 dk: 0.0205

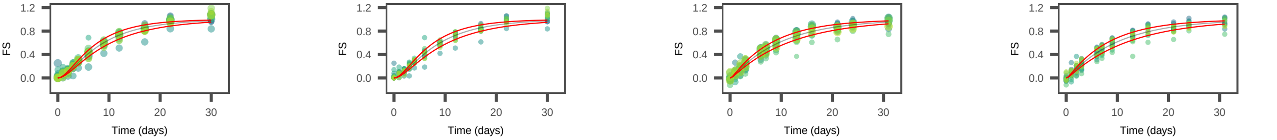

MOES

AA Weighted R2: 0.95 k: 0.139 dk: 0.0323 | AA Unweighted R2: 0.95 k: 0.135 dk: 0.0311 | HW Weighted R2: 0.96 k: 0.142 dk: 0.0289 | HW Unweighted R2: 0.96 k: 0.14 dk: 0.028

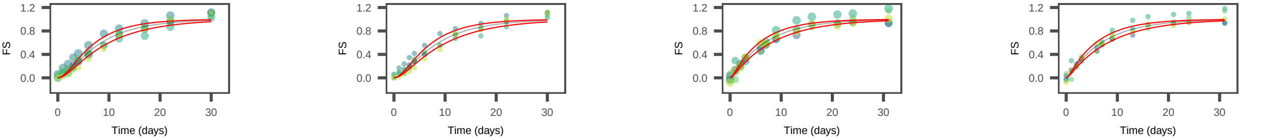

MPC1

AA Weighted R2: 0.98 k: 0.169 dk: 0.0247 | AA Unweighted R2: 0.98 k: 0.177 dk: 0.0305 | HW Weighted R2: 0.88 k: 0.112 dk: 0.0353 | HW Unweighted R2: 0.85 k: 0.106 dk: 0.037

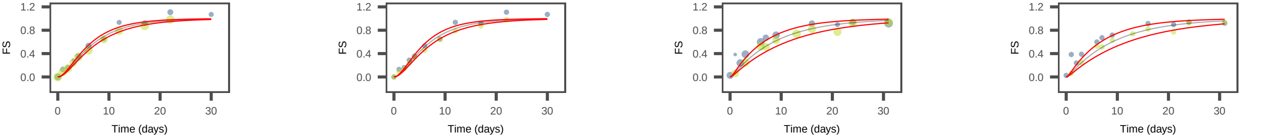

MPCP

AA Weighted R2: 0.98 k: 0.138 dk: 0.0209

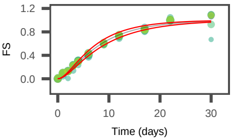

AA Unweighted R2: 0.97 k: 0.137 dk: 0.0236

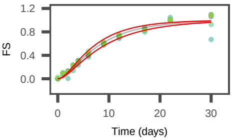

HW Weighted R2: 0.86 k: 0.134 dk: 0.0475

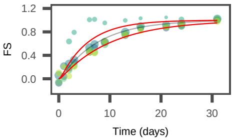

HW Unweighted R2: 0.83 k: 0.138 dk: 0.0532

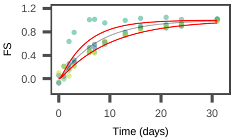

MPU1

AA Weighted R2: 0.97 k: 0.351 dk: 0.0796

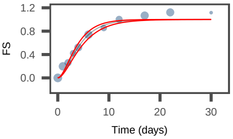

AA Unweighted R2: 0.97 k: 0.342 dk: 0.0766

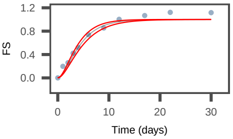

HW Weighted R2: 0.96 k: 0.208 dk: 0.0421

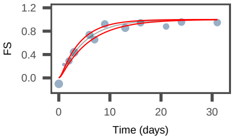

HW Unweighted R2: 0.96 k: 0.209 dk: 0.0424

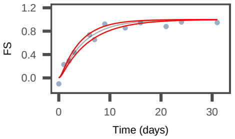

MSRA

AA Weighted R2: 0.88 k: 0.334 dk: 0.159

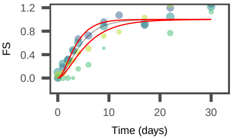

AA Unweighted R2: 0.85 k: 0.244 dk: 0.121

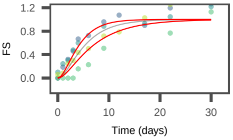

HW Weighted R2: 0.94 k: 0.201 dk: 0.042

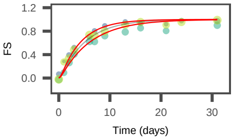

HW Unweighted R2: 0.94 k: 0.203 dk: 0.0441

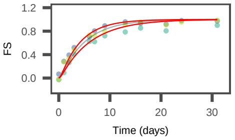

MTL26

AA Weighted R2: 0.95 k: 0.221 dk: 0.0706

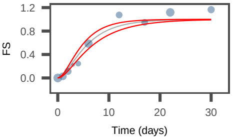

AA Unweighted R2: 0.95 k: 0.217 dk: 0.0706

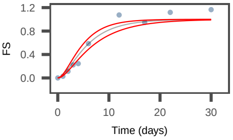

HW Weighted R2: 0.93 k: 0.2 dk: 0.0491

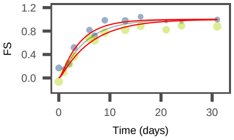

HW Unweighted R2: 0.93 k: 0.204 dk: 0.0503

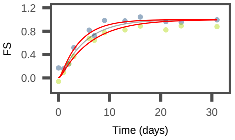

MTX2

AA Weighted R2: 0.9 k: 0.0861 dk: 0.0296

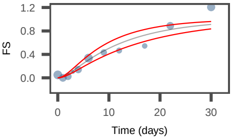

AA Unweighted R2: 0.9 k: 0.0817 dk: 0.0277

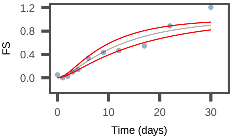

HW Weighted R2: 0.95 k: 0.0911 dk: 0.0201

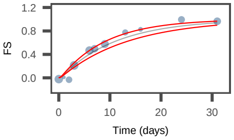

HW Unweighted R2: 0.94 k: 0.0879 dk: 0.0199

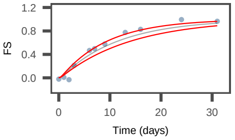

MUTA

AA Weighted R2: 0.91 k: 0.369 dk: 0.139

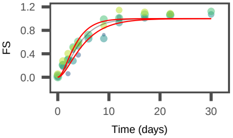

AA Unweighted R2: 0.89 k: 0.33 dk: 0.14

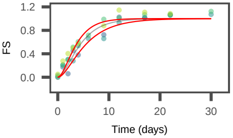

HW Weighted R2: 0.93 k: 0.568 dk: 0.129

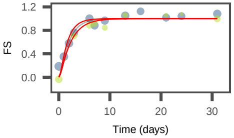

HW Unweighted R2: 0.94 k: 0.551 dk: 0.124

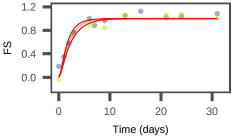

MYH9

AA Weighted R2: 0.94 k: 0.172 dk: 0.0439 | AA Unweighted R2: 0.93 k: 0.17 dk: 0.0496 | HW Weighted R2: 0.93 k: 0.159 dk: 0.0395 | HW Unweighted R2: 0.93 k: 0.158 dk: 0.0403

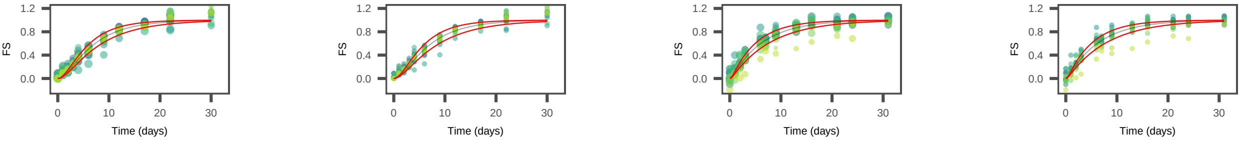

MYL6

AA Weighted R2: 0.95 k: 0.237 dk: 0.0683 | AA Unweighted R2: 0.95 k: 0.226 dk: 0.0613 | HW Weighted R2: 0.97 k: 0.278 dk: 0.0489 | HW Unweighted R2: 0.96 k: 0.29 dk: 0.0516

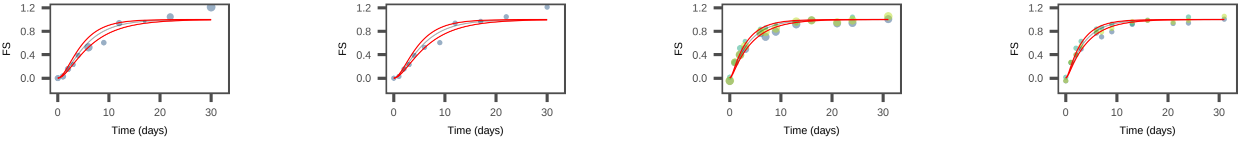

MYLK

AA Weighted R2: 0.94 k: 0.109 dk: 0.0252 | AA Unweighted R2: 0.93 k: 0.116 dk: 0.0277 | HW Weighted R2: 0.94 k: 0.385 dk: 0.102 | HW Unweighted R2: 0.93 k: 0.391 dk: 0.115

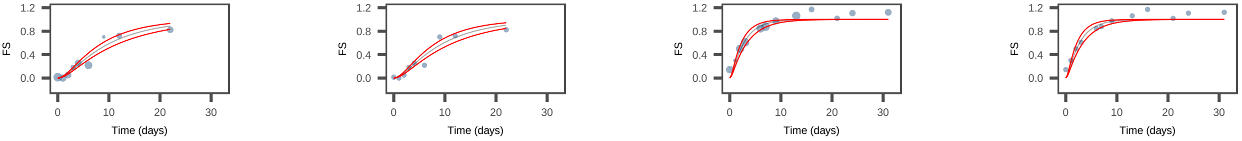

MYO1C

AA Weighted R2: 0.95 k: 0.104 dk: 0.0236 | AA Unweighted R2: 0.94 k: 0.111 dk: 0.0279 | HW Weighted R2: 0.93 k: 0.109 dk: 0.0254 | HW Unweighted R2: 0.93 k: 0.108 dk: 0.0252

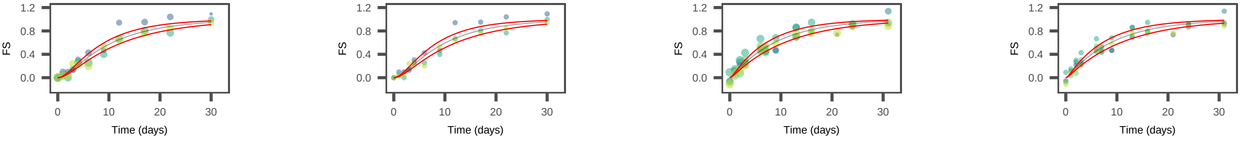

MYO6

AA Weighted R2: 0.94 k: 0.404 dk: 0.126 | AA Unweighted R2: 0.93 k: 0.404 dk: 0.13 | HW Weighted R2: 0.97 k: 0.33 dk: 0.054 | HW Unweighted R2: 0.97 k: 0.335 dk: 0.055

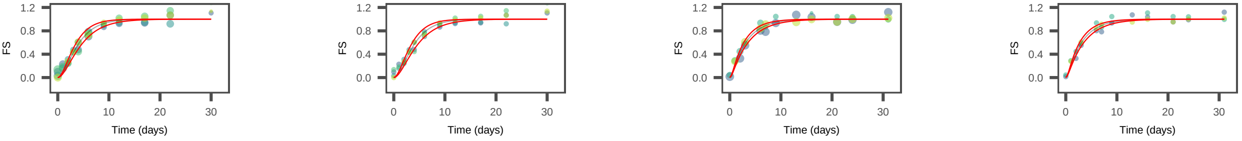

MYO7B

AA Weighted R2: 0.89 k: 0.184 dk: 0.069 | AA Unweighted R2: 0.9 k: 0.203 dk: 0.0754 | HW Weighted R2: 0.9 k: 0.0792 dk: 0.018 | HW Unweighted R2: 0.9 k: 0.079 dk: 0.0179

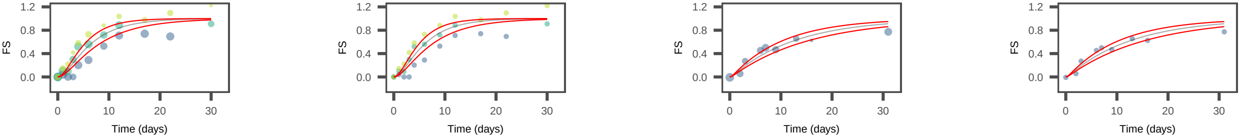

NB5R3

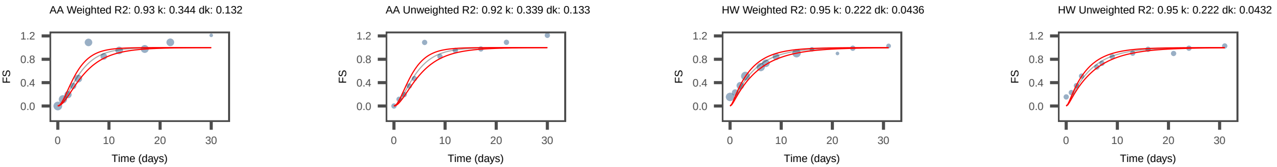

NCEH1

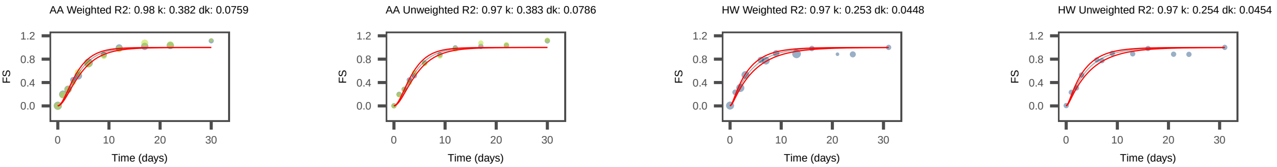

NCPR

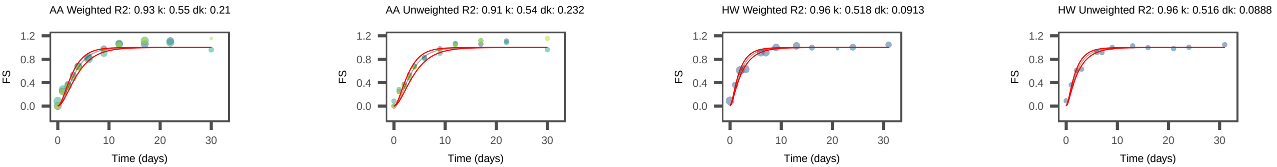

NDKA

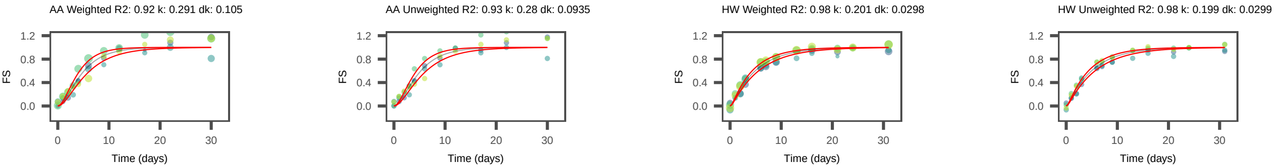

NDKB

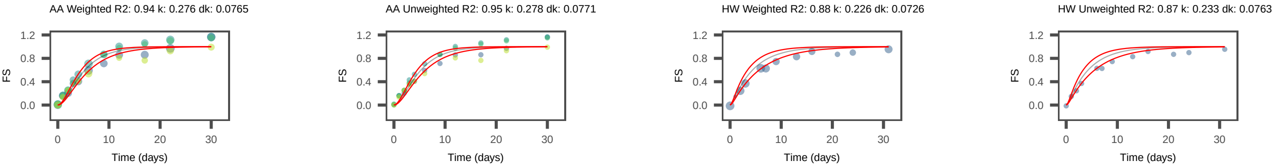

NDRG1

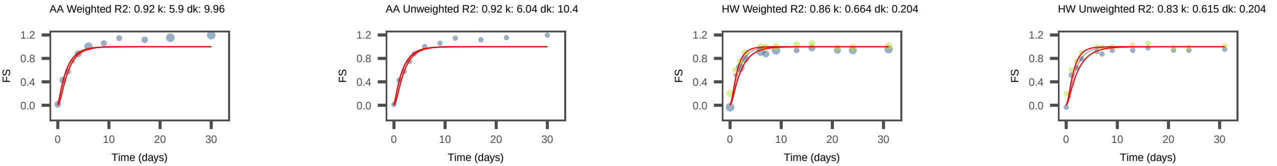

NDUA2

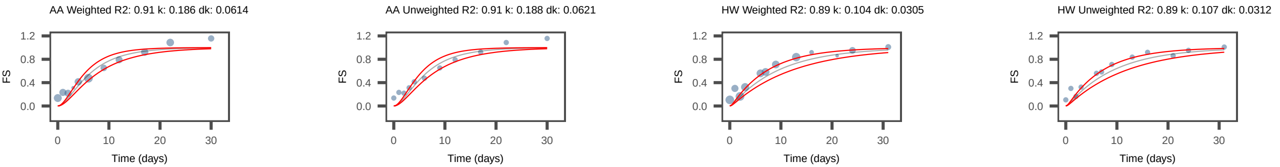

NDUA4

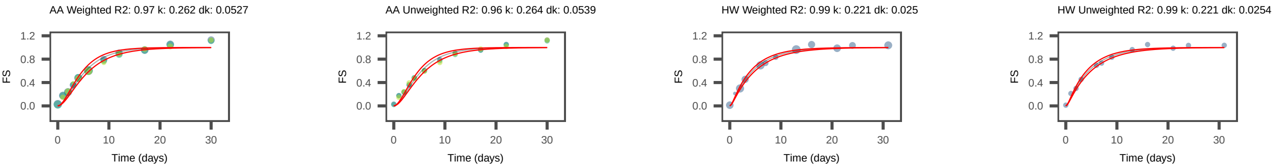

NDUA6

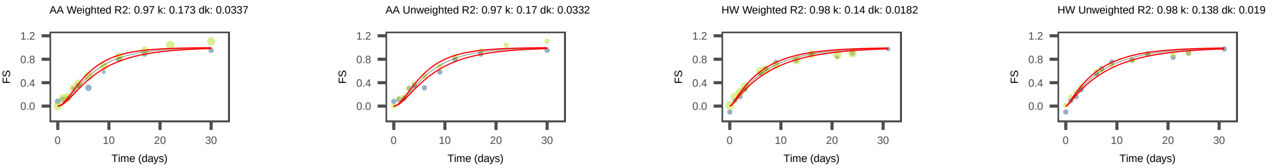

NDUA7

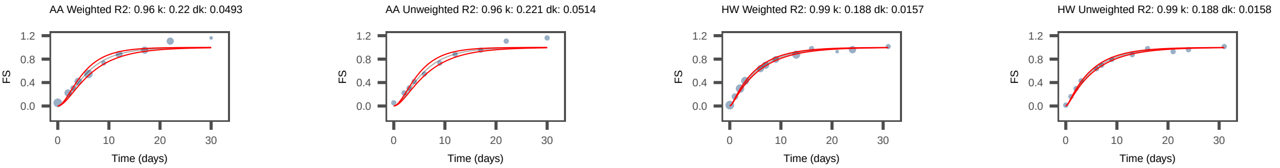

NDUA8

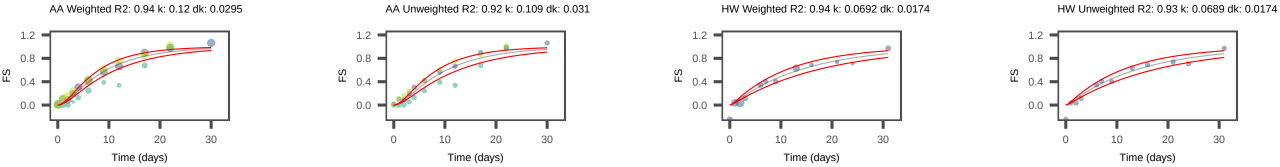

NDUA9

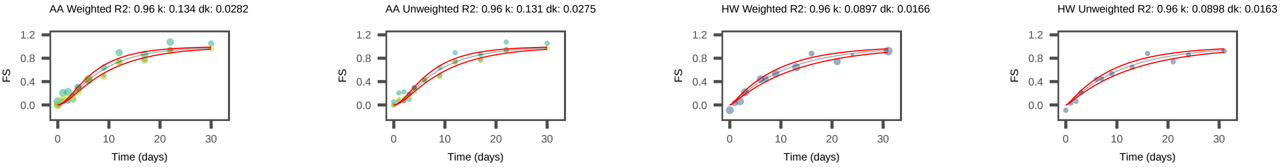

NDUAA

AA Weighted R2: 0.96 k: 0.144 dk: 0.0313 | AA Unweighted R2: 0.95 k: 0.147 dk: 0.0349 | HW Weighted R2: 0.96 k: 0.124 dk: 0.0239 | HW Unweighted R2: 0.95 k: 0.12 dk: 0.025

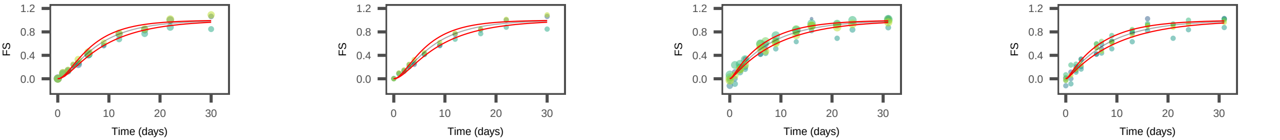

NDUAB

AA Weighted R2: 0.98 k: 0.123 dk: 0.0206 | AA Unweighted R2: 0.98 k: 0.124 dk: 0.0203 | HW Weighted R2: 0.86 k: 0.117 dk: 0.0437 | HW Unweighted R2: 0.86 k: 0.11 dk: 0.0425

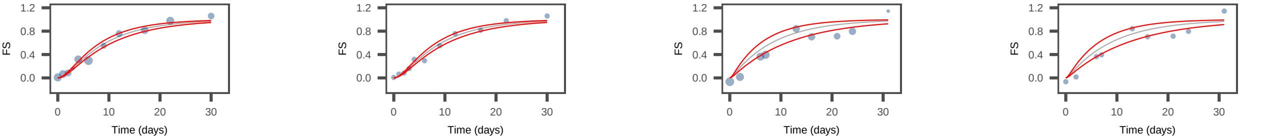

NDUAD

AA Weighted R2: 0.97 k: 0.141 dk: 0.0275 | AA Unweighted R2: 0.97 k: 0.141 dk: 0.0292 | HW Weighted R2: 0.98 k: 0.116 dk: 0.0136 | HW Unweighted R2: 0.98 k: 0.116 dk: 0.0138

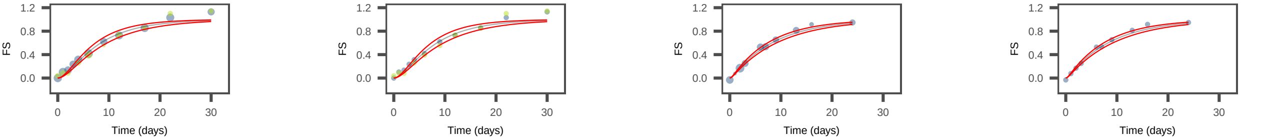

NDUB3

AA Weighted R2: 0.94 k: 0.104 dk: 0.0231 | AA Unweighted R2: 0.94 k: 0.0963 dk: 0.0219 | HW Weighted R2: 0.97 k: 0.125 dk: 0.0234 | HW Unweighted R2: 0.97 k: 0.125 dk: 0.0238

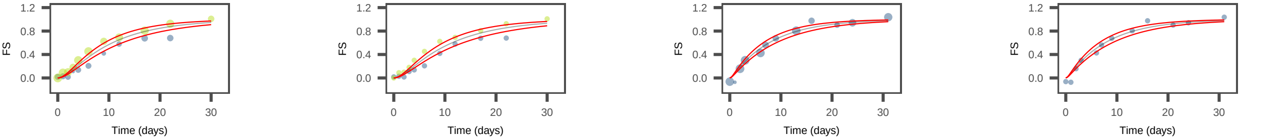

NDUB5

AA Weighted R2: 0.96 k: 0.126 dk: 0.0228 | AA Unweighted R2: 0.96 k: 0.125 dk: 0.0231 | HW Weighted R2: 0.97 k: 0.0856 dk: 0.0138 | HW Unweighted R2: 0.97 k: 0.0865 dk: 0.0138

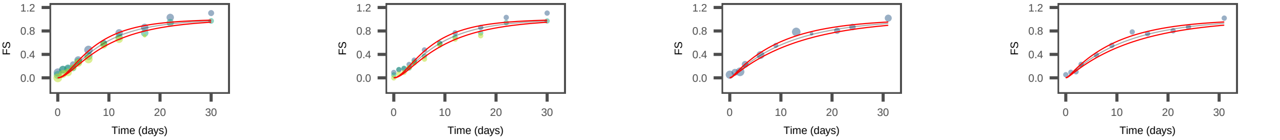

NDUB9

AA Weighted R2: 0.98 k: 0.139 dk: 0.0232 | AA Unweighted R2: 0.97 k: 0.14 dk: 0.0236 | HW Weighted R2: 0.98 k: 0.0963 dk: 0.0132 | HW Unweighted R2: 0.98 k: 0.0963 dk: 0.0132

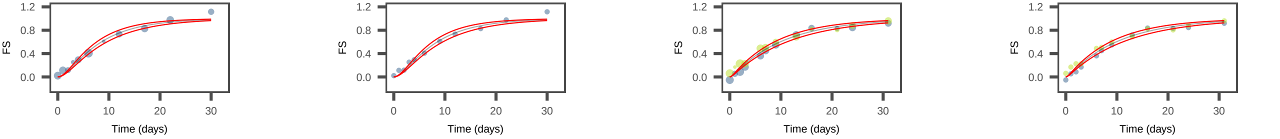

NDUBB

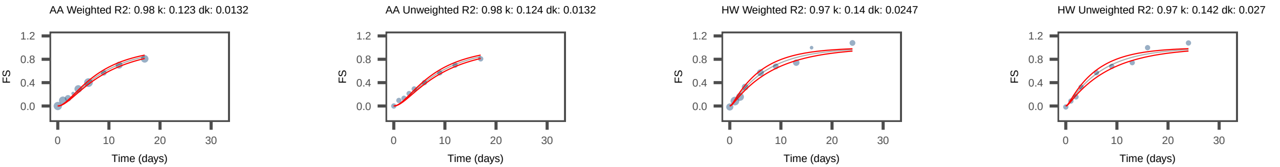

NDUC2

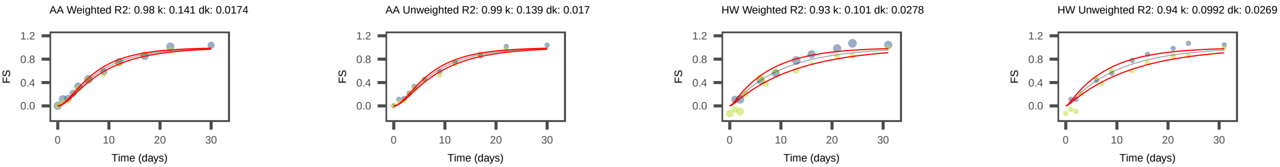

NDUS1

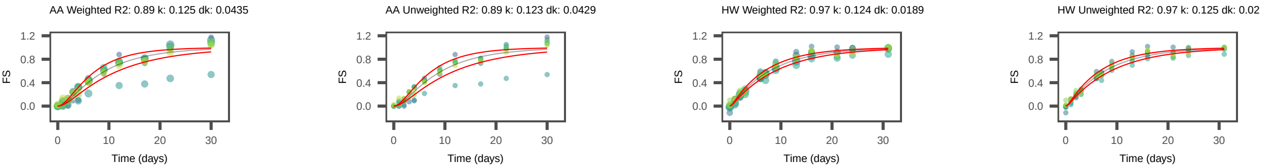

NDUS2

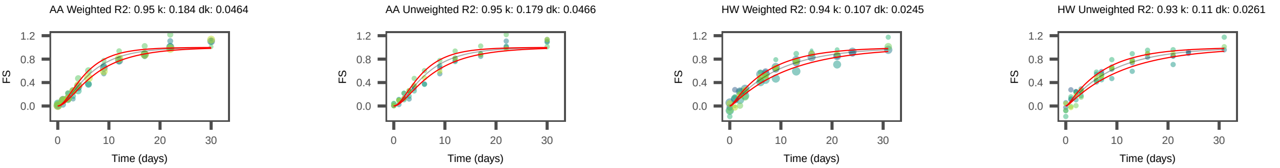

NDUS3

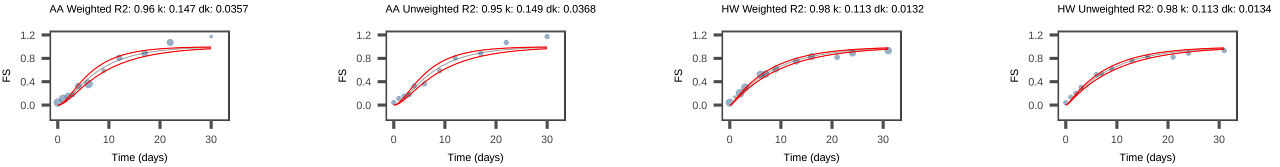

NDUS4

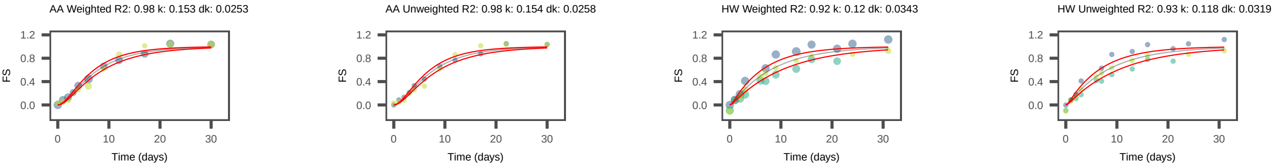

NDUS6

AA Weighted R2: 0.9 k: 0.107 dk: 0.0288

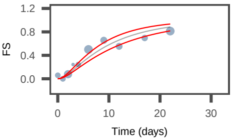

AA Unweighted R2: 0.91 k: 0.107 dk: 0.0273

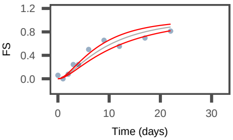

HW Weighted R2: 0.94 k: 0.166 dk: 0.0414

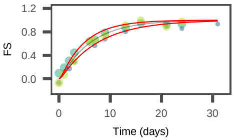

HW Unweighted R2: 0.93 k: 0.159 dk: 0.041

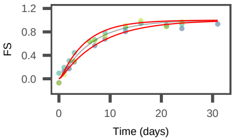

NDUV1

AA Weighted R2: 0.93 k: 0.139 dk: 0.0376

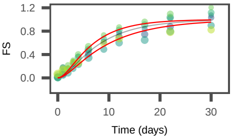

AA Unweighted R2: 0.93 k: 0.144 dk: 0.0391

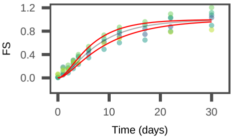

HW Weighted R2: 0.93 k: 0.134 dk: 0.0334

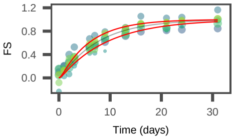

HW Unweighted R2: 0.92 k: 0.131 dk: 0.0339

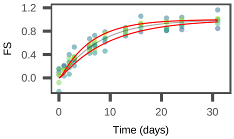

NDUV2

AA Weighted R2: 0.97 k: 0.125 dk: 0.0219

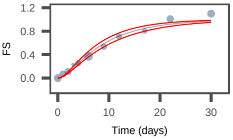

AA Unweighted R2: 0.98 k: 0.125 dk: 0.0214

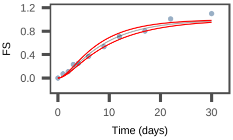

HW Weighted R2: 0.94 k: 0.136 dk: 0.036

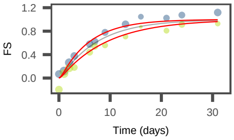

HW Unweighted R2: 0.93 k: 0.135 dk: 0.0361

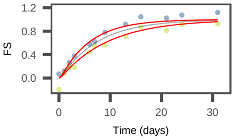

NEP

AA Weighted R2: 0.93 k: 0.328 dk: 0.112

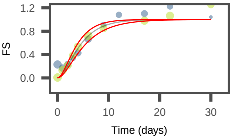

AA Unweighted R2: 0.93 k: 0.328 dk: 0.115

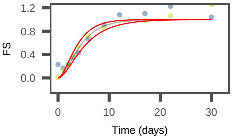

HW Weighted R2: 0.93 k: 0.376 dk: 0.107

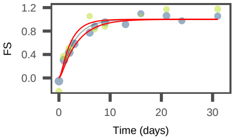

HW Unweighted R2: 0.93 k: 0.381 dk: 0.113

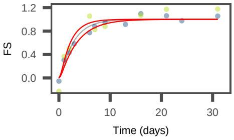

NFS1

AA Weighted R2: 0.94 k: 0.196 dk: 0.0625

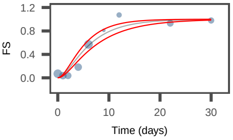

AA Unweighted R2: 0.94 k: 0.206 dk: 0.0688

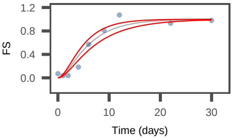

HW Weighted R2: 0.87 k: 0.2 dk: 0.0711

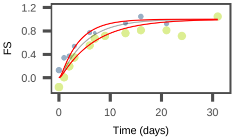

HW Unweighted R2: 0.87 k: 0.207 dk: 0.0724

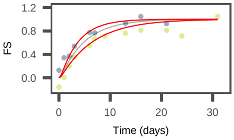

NHRF3

AA Weighted R2: 0.93 k: 0.561 dk: 0.207

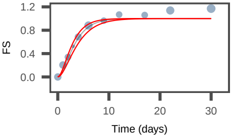

AA Unweighted R2: 0.94 k: 0.528 dk: 0.186

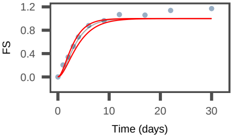

HW Weighted R2: 0.92 k: 0.392 dk: 0.109

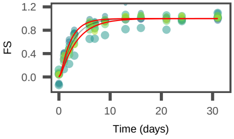

HW Unweighted R2: 0.91 k: 0.411 dk: 0.116

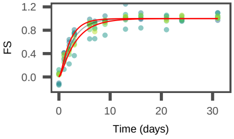

NIPS1

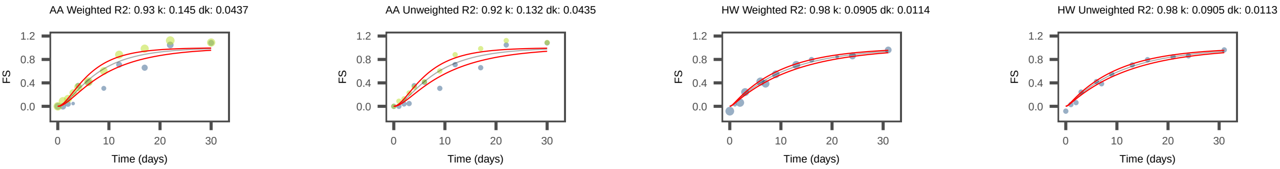

NIT2

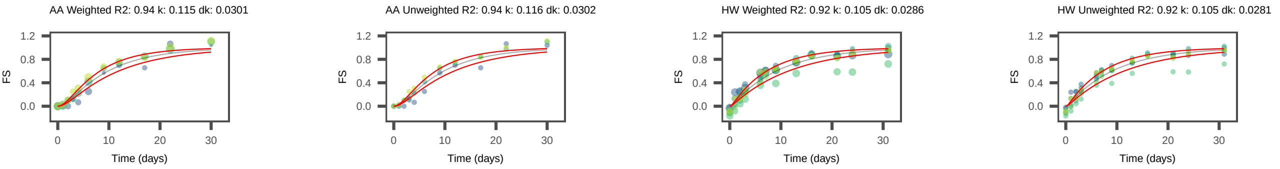

NMT1

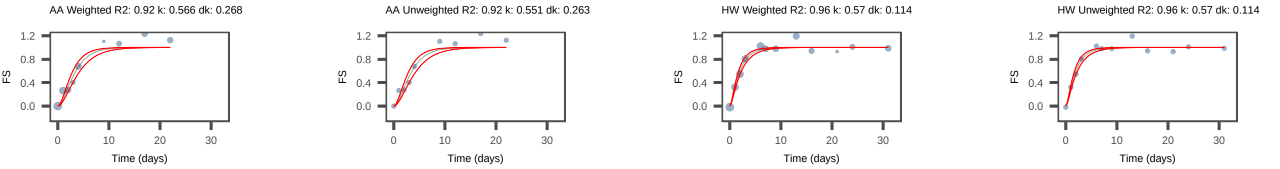

NNRD

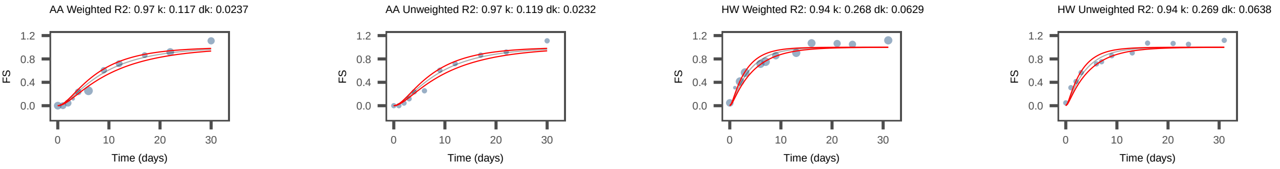

NNRE

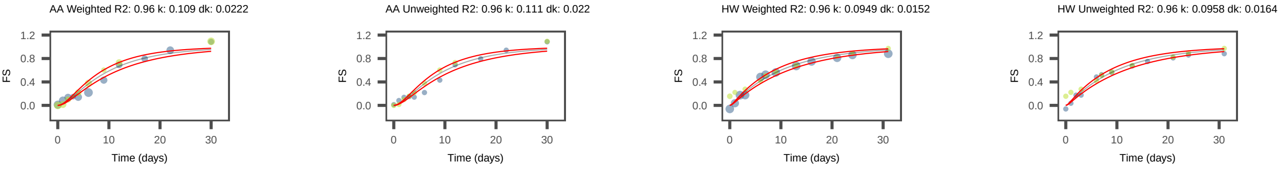

NNTM

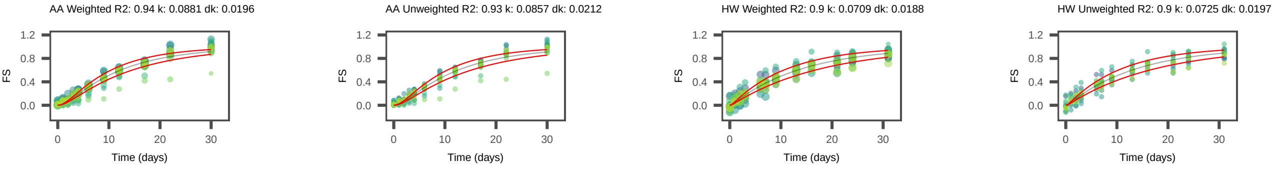

NQO1

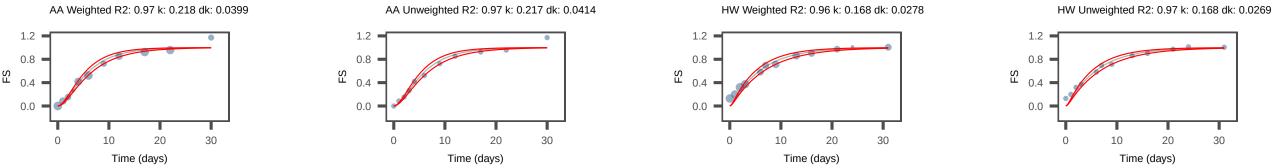

NU4M

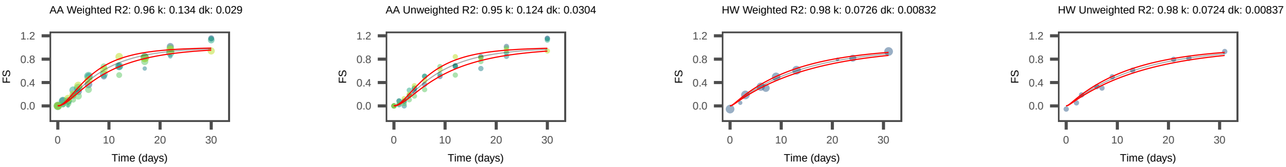

NU5M

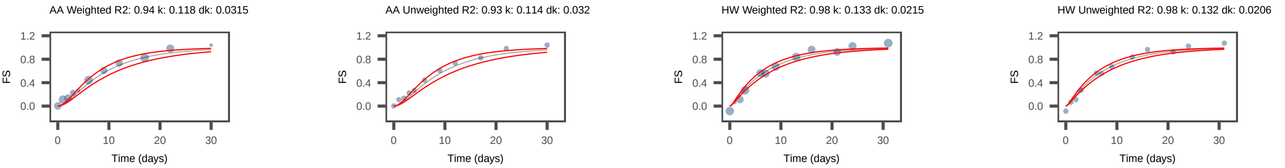

NUD19

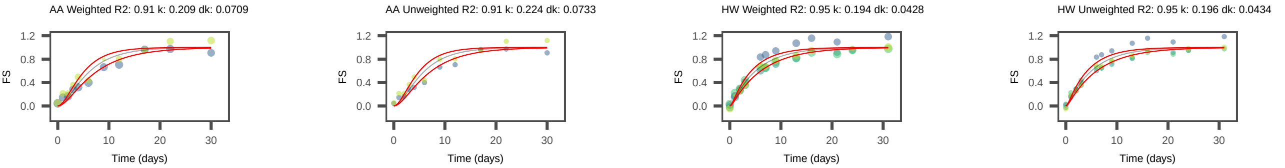

OCTC

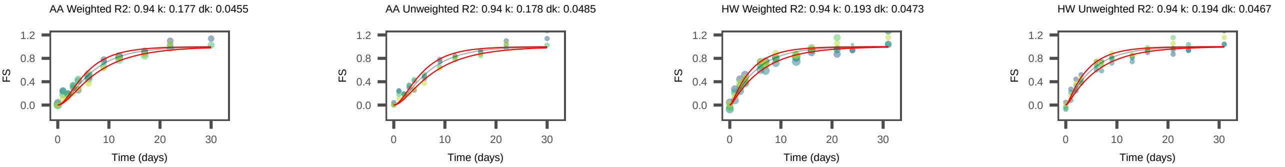

ODB2

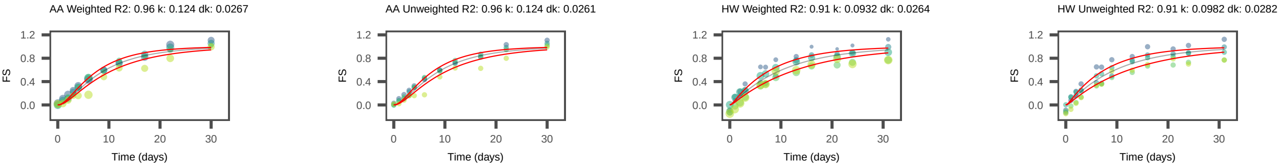

ODBA

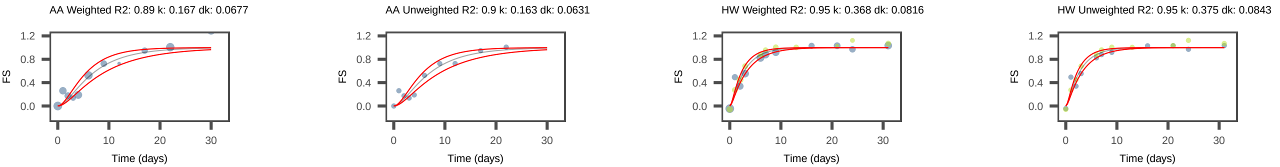

ODBB

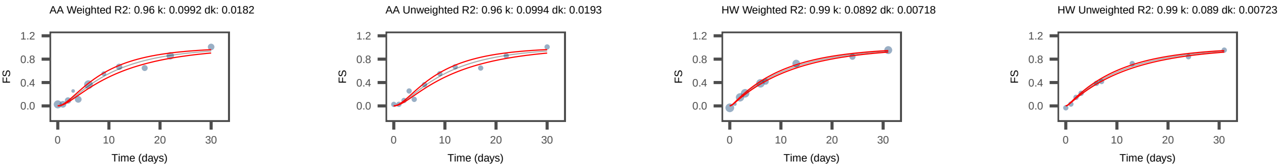

ODC

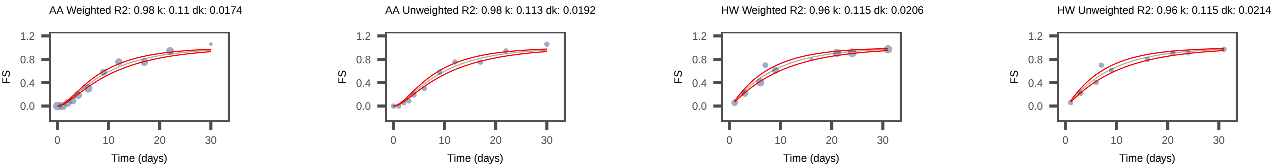

ODO1

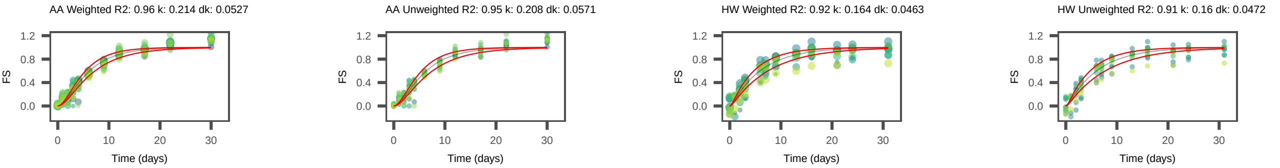

ODO2

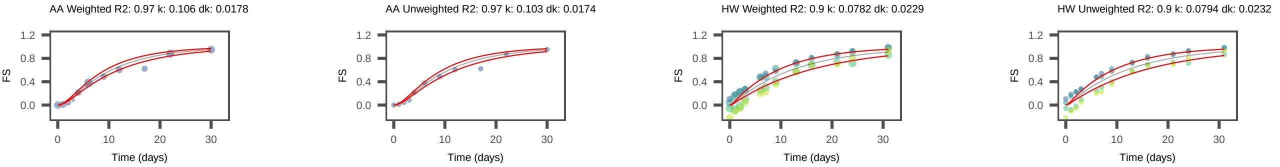

ODP2

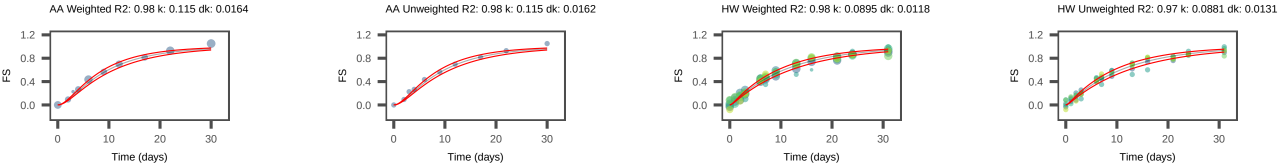

ODPA

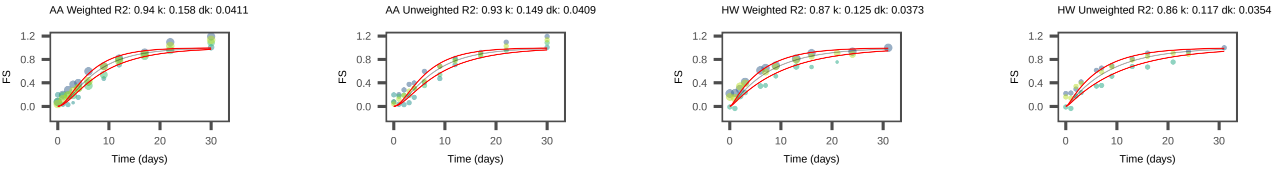

ODPB

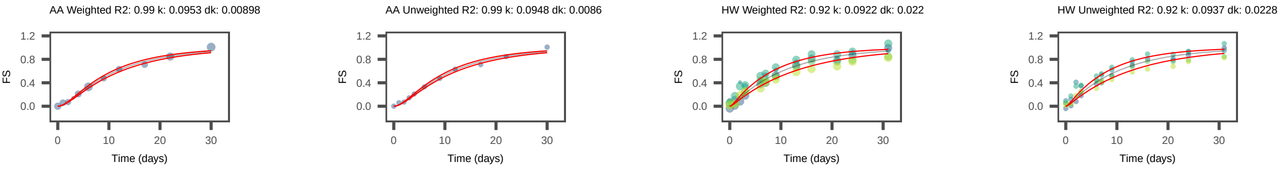

ODPX

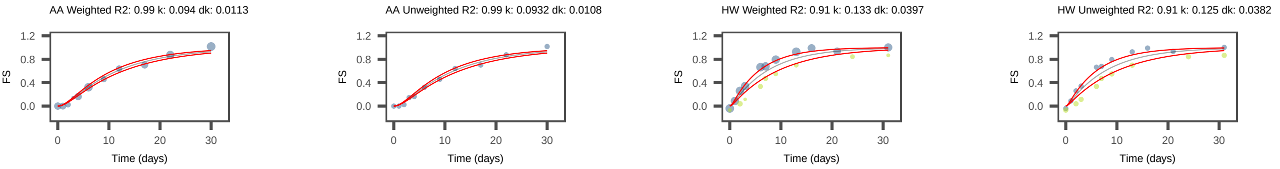

OPA1

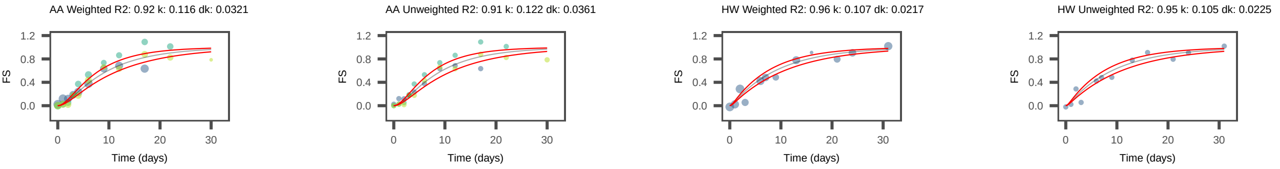

ORNT1

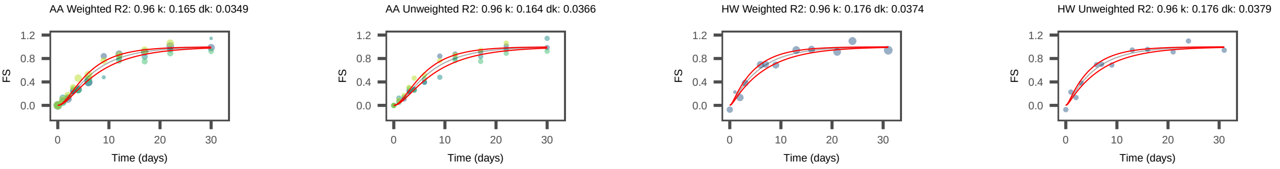

OXDA

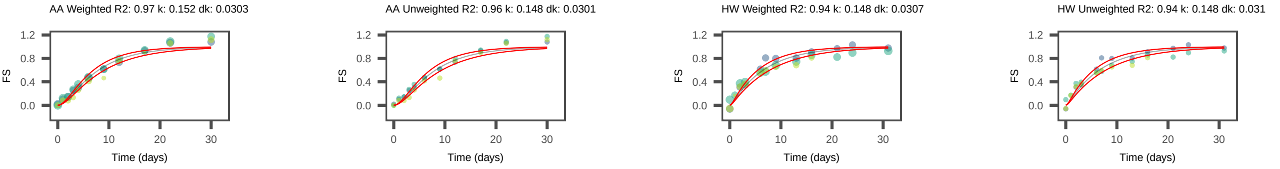

PA2G4

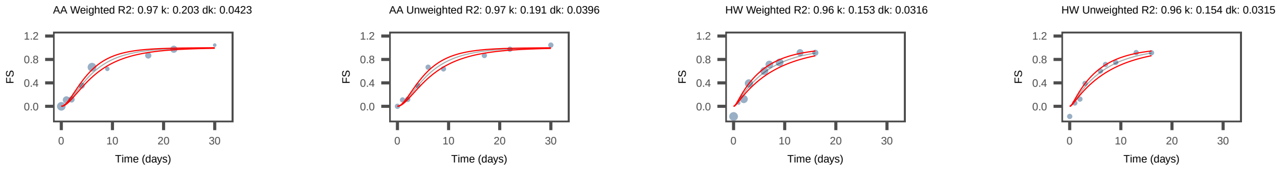

PARK7

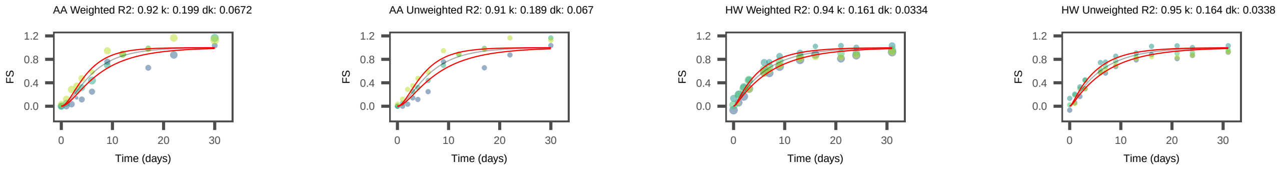

PCBP2

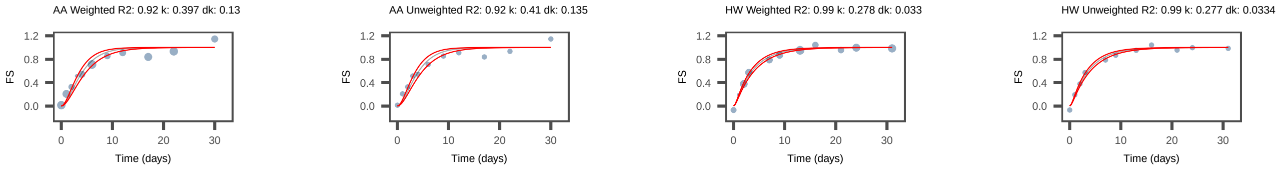

PCCA

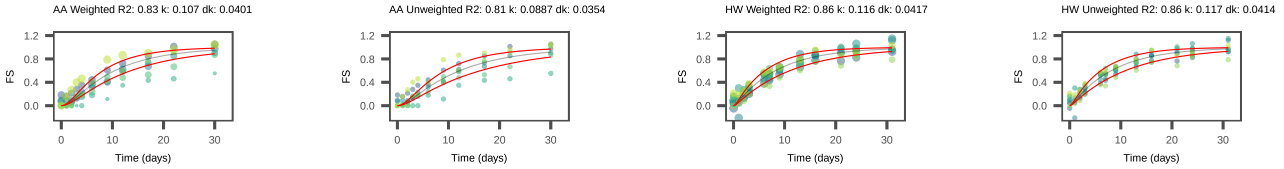

PCCB

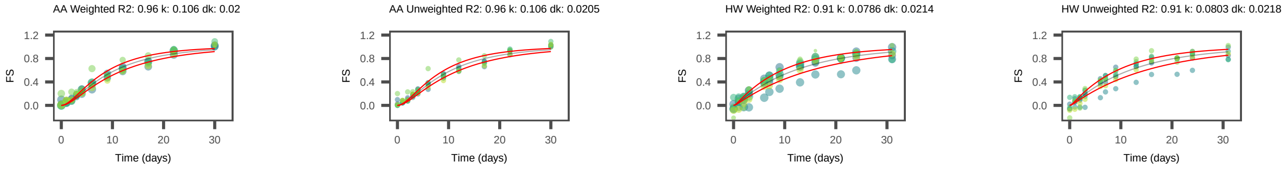

PCKGC

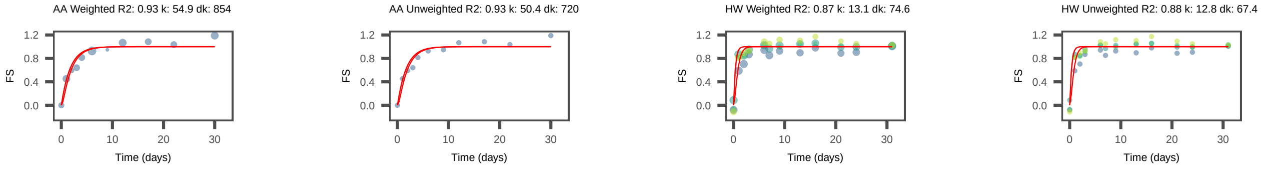

PDC6I

AA Weighted R2: 0.93 k: 0.324 dk: 0.113 | AA Unweighted R2: 0.92 k: 0.341 dk: 0.13 | HW Weighted R2: 0.92 k: 0.338 dk: 0.0853 | HW Unweighted R2: 0.91 k: 0.34 dk: 0.089

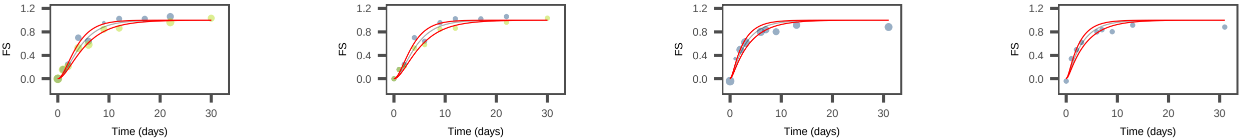

PDIA1

AA Weighted R2: 0.95 k: 0.407 dk: 0.115 | AA Unweighted R2: 0.95 k: 0.431 dk: 0.123 | HW Weighted R2: 0.9 k: 0.326 dk: 0.102 | HW Unweighted R2: 0.89 k: 0.329 dk: 0.105

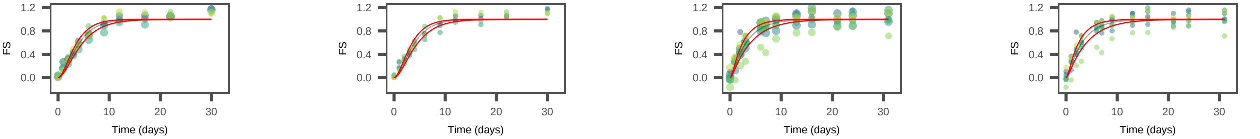

PDIA3

AA Weighted R2: 0.88 k: 0.316 dk: 0.145 | AA Unweighted R2: 0.88 k: 0.308 dk: 0.144 | HW Weighted R2: 0.94 k: 0.355 dk: 0.081 | HW Unweighted R2: 0.94 k: 0.348 dk: 0.0796

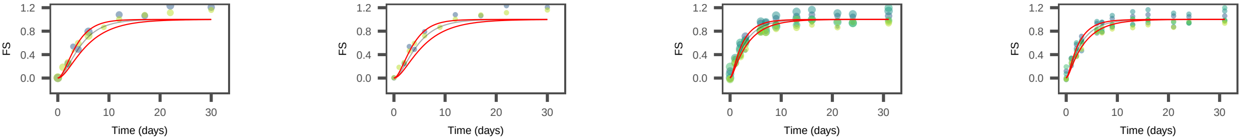

PEBP1

AA Weighted R2: 0.94 k: 0.245 dk: 0.0707 | AA Unweighted R2: 0.94 k: 0.249 dk: 0.0702 | HW Weighted R2: 0.96 k: 0.204 dk: 0.0383 | HW Unweighted R2: 0.97 k: 0.202 dk: 0.0368

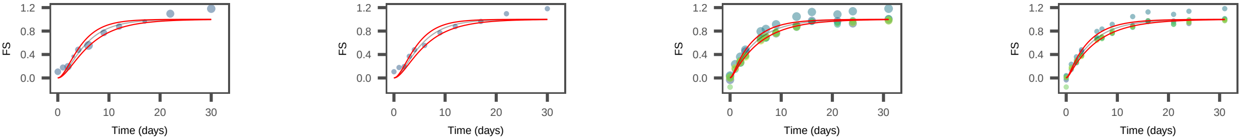

PECR

AA Weighted R2: 0.92 k: 0.147 dk: 0.04 | AA Unweighted R2: 0.91 k: 0.136 dk: 0.0411 | HW Weighted R2: 0.96 k: 0.109 dk: 0.021 | HW Unweighted R2: 0.96 k: 0.108 dk: 0.0215

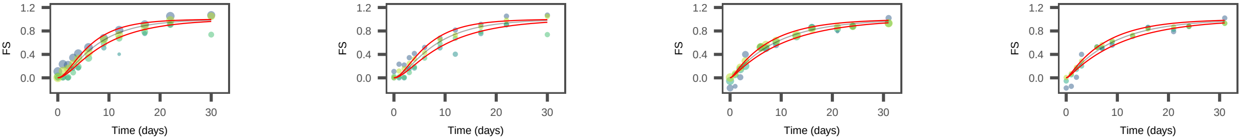

PGAM1

AA Weighted R2: 0.91 k: 0.182 dk: 0.0636 | AA Unweighted R2: 0.9 k: 0.185 dk: 0.0685 | HW Weighted R2: 0.97 k: 0.195 dk: 0.034 | HW Unweighted R2: 0.97 k: 0.195 dk: 0.0333

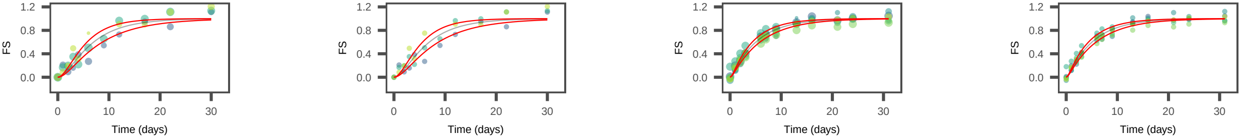

PGAM2

AA Weighted R2: 0.91 k: 0.075 dk: 0.0223
AA Unweighted R2: 0.9 k: 0.0724 dk: 0.0219
HW Weighted R2: 0.88 k: 0.137 dk: 0.0488
HW Unweighted R2: 0.89 k: 0.125 dk: 0.0429

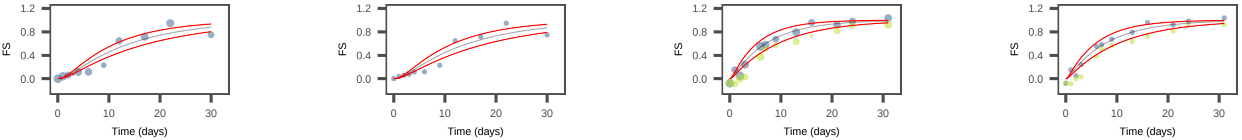

PGBM

AA Weighted R2: 0.93 k: 0.0295 dk: 0.00429
AA Unweighted R2: 0.93 k: 0.0295 dk: 0.00435
HW Weighted R2: 0.96 k: 0.025 dk: 0.00278
HW Unweighted R2: 0.96 k: 0.0251 dk: 0.00285

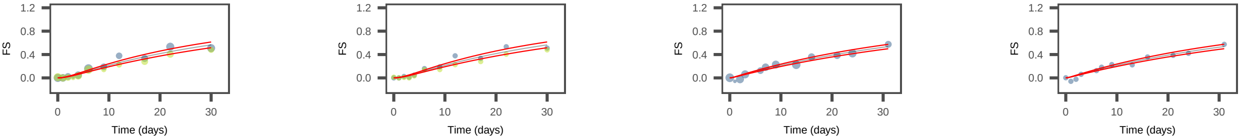

PGK1

AA Weighted R2: 0.95 k: 0.233 dk: 0.0589
AA Unweighted R2: 0.95 k: 0.231 dk: 0.0582
HW Weighted R2: 0.95 k: 0.135 dk: 0.0283
HW Unweighted R2: 0.94 k: 0.137 dk: 0.0298

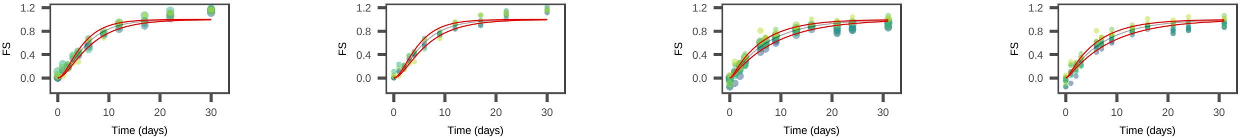

PGM1

AA Weighted R2: 0.96 k: 0.214 dk: 0.0543
AA Unweighted R2: 0.95 k: 0.205 dk: 0.0562
HW Weighted R2: 0.97 k: 0.184 dk: 0.0337
HW Unweighted R2: 0.97 k: 0.186 dk: 0.0342

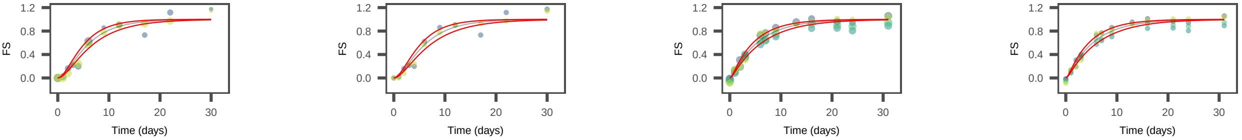

PH4H

AA Weighted R2: 0.95 k: 6.62 dk: 9.34
AA Unweighted R2: 0.94 k: 5.4 dk: 6.43
HW Weighted R2: 0.92 k: 8.8 dk: 21.1
HW Unweighted R2: 0.92 k: 9.43 dk: 25.6

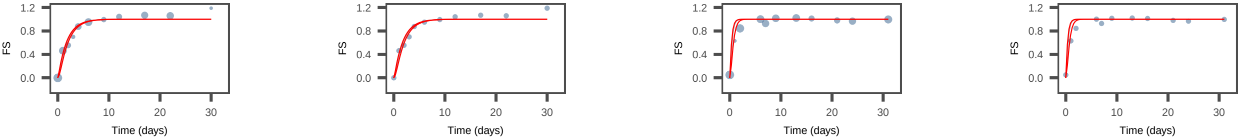

PHB

AA Weighted R2: 0.94 k: 0.124 dk: 0.0307
AA Unweighted R2: 0.89 k: 0.109 dk: 0.0374
HW Weighted R2: 0.92 k: 0.114 dk: 0.0306
HW Unweighted R2: 0.92 k: 0.113 dk: 0.0308

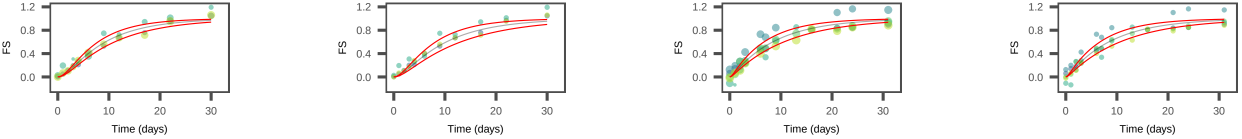

PHB2

AA Weighted R2: 0.97 k: 0.0976 dk: 0.015

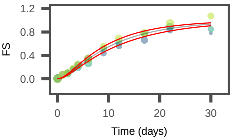

AA Unweighted R2: 0.97 k: 0.0966 dk: 0.0156

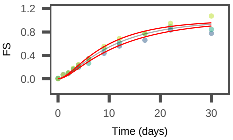

HW Weighted R2: 0.99 k: 0.0855 dk: 0.00626

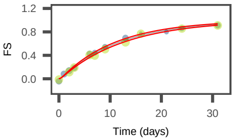

HW Unweighted R2: 0.99 k: 0.086 dk: 0.00636

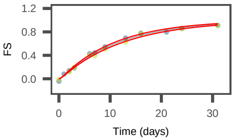

PICAL

AA Weighted R2: 0.93 k: 0.91 dk: 0.372

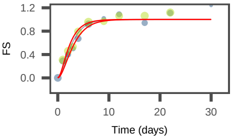

AA Unweighted R2: 0.92 k: 0.837 dk: 0.35

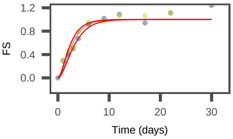

HW Weighted R2: 0.94 k: 0.448 dk: 0.109

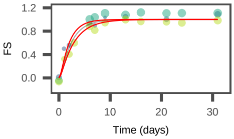

HW Unweighted R2: 0.94 k: 0.46 dk: 0.113

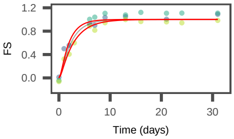

PLSI

AA Weighted R2: 0.89 k: 0.164 dk: 0.058

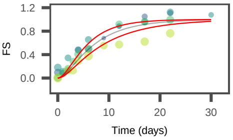

AA Unweighted R2: 0.89 k: 0.177 dk: 0.0648

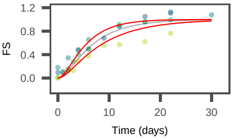

HW Weighted R2: 0.92 k: 0.159 dk: 0.0508

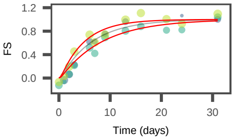

HW Unweighted R2: 0.92 k: 0.157 dk: 0.0505

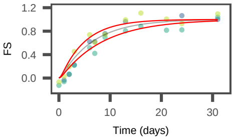

PLSL

AA Weighted R2: 0.96 k: 0.152 dk: 0.0344

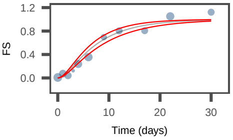

AA Unweighted R2: 0.96 k: 0.158 dk: 0.0358

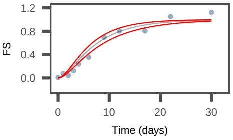

HW Weighted R2: 0.92 k: 0.11 dk: 0.033

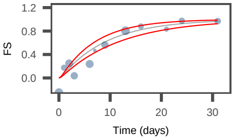

HW Unweighted R2: 0.92 k: 0.11 dk: 0.0325

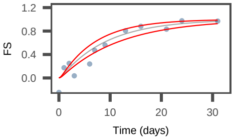

PLST

AA Weighted R2: 0.92 k: 0.13 dk: 0.0428

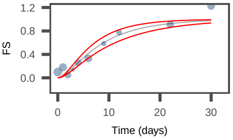

AA Unweighted R2: 0.92 k: 0.13 dk: 0.0405

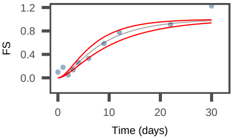

HW Weighted R2: 0.95 k: 0.12 dk: 0.0236

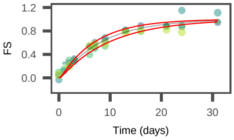

HW Unweighted R2: 0.95 k: 0.12 dk: 0.024

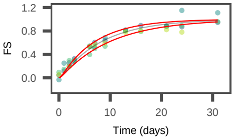

PPID

AA Weighted R2: 0.91 k: 0.125 dk: 0.0368

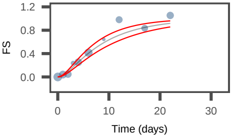

AA Unweighted R2: 0.92 k: 0.135 dk: 0.0382

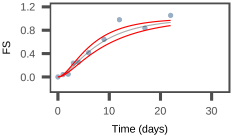

HW Weighted R2: 0.94 k: 0.253 dk: 0.0755

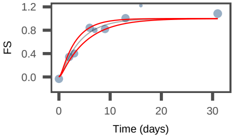

HW Unweighted R2: 0.93 k: 0.254 dk: 0.0778

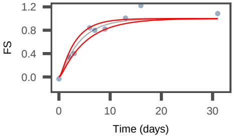

PRD16

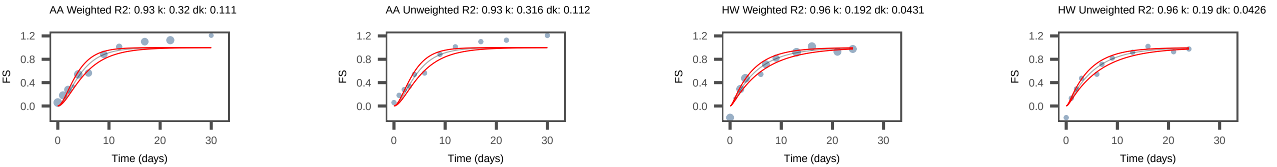

PRDX1

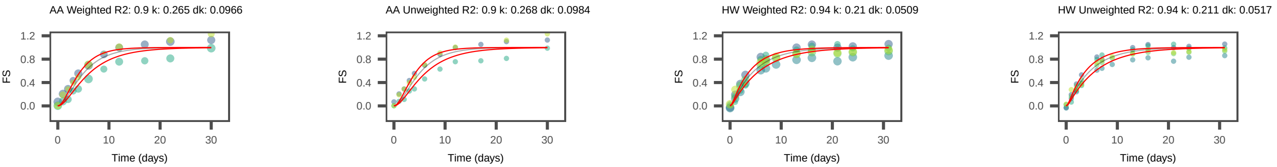

PRDX2

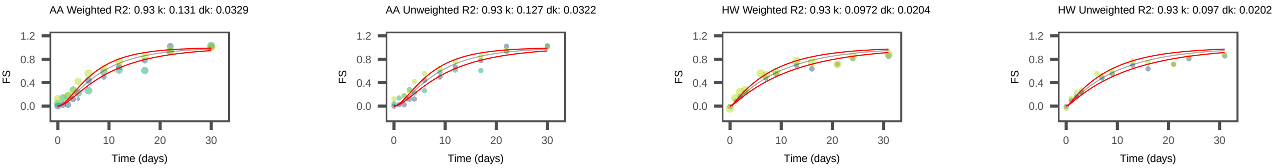

PRDX5

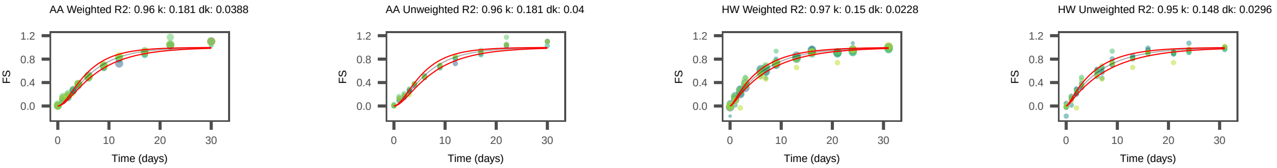

PRDX6

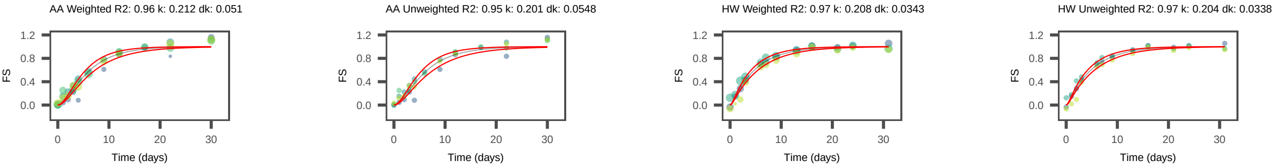

PROF1

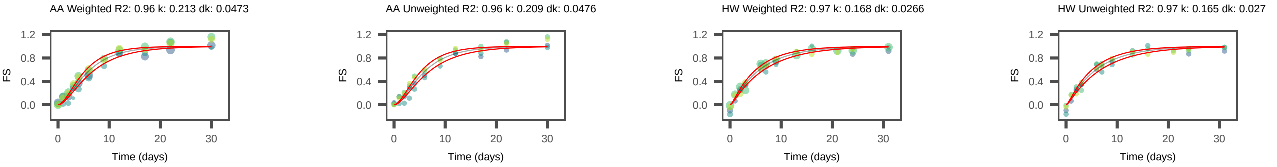

PSA2

AA Weighted R2: 0.93 k: 0.0844 dk: 0.0279

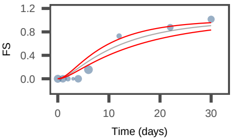

AA Unweighted R2: 0.93 k: 0.0875 dk: 0.0287

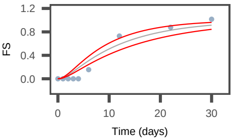

HW Weighted R2: 0.93 k: 0.222 dk: 0.0596

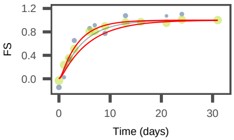

HW Unweighted R2: 0.93 k: 0.234 dk: 0.0665

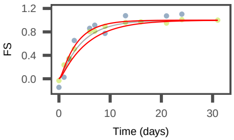

PSB3

AA Weighted R2: 0.97 k: 0.4 dk: 0.0908

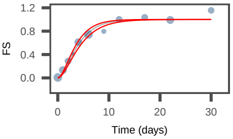

AA Unweighted R2: 0.97 k: 0.397 dk: 0.0938

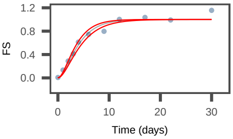

HW Weighted R2: 0.98 k: 0.26 dk: 0.0364

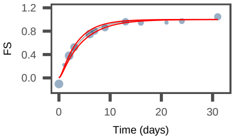

HW Unweighted R2: 0.98 k: 0.26 dk: 0.036

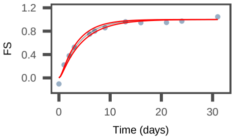

PSB4

AA Weighted R2: 0.91 k: 0.406 dk: 0.171

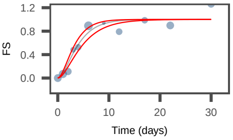

AA Unweighted R2: 0.92 k: 0.398 dk: 0.165

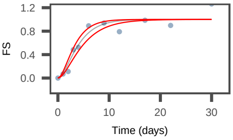

HW Weighted R2: 0.99 k: 0.281 dk: 0.0331

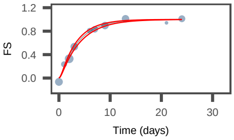

HW Unweighted R2: 0.99 k: 0.281 dk: 0.0332

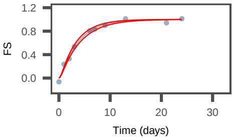

PSB5

AA Weighted R2: 0.97 k: 0.269 dk: 0.0579

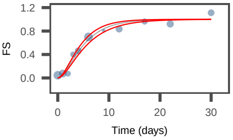

AA Unweighted R2: 0.97 k: 0.265 dk: 0.0578

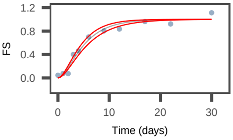

HW Weighted R2: 0.92 k: 0.4 dk: 0.0922

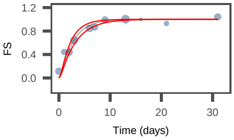

HW Unweighted R2: 0.92 k: 0.404 dk: 0.0944

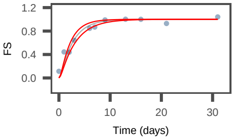

PSMD1

AA Weighted R2: 0.93 k: 0.288 dk: 0.0921

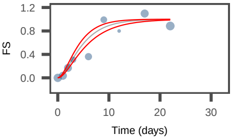

AA Unweighted R2: 0.92 k: 0.269 dk: 0.0906

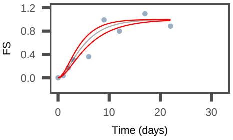

HW Weighted R2: 0.95 k: 0.235 dk: 0.0396

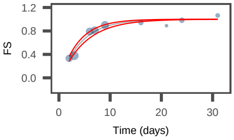

HW Unweighted R2: 0.95 k: 0.236 dk: 0.0398

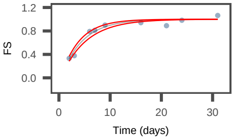

PTER

AA Weighted R2: 0.95 k: 0.343 dk: 0.0981

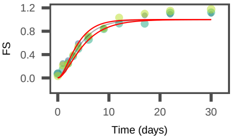

AA Unweighted R2: 0.95 k: 0.352 dk: 0.0992

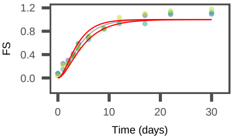

HW Weighted R2: 0.93 k: 0.15 dk: 0.0385

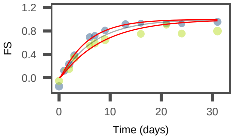

HW Unweighted R2: 0.93 k: 0.151 dk: 0.0383

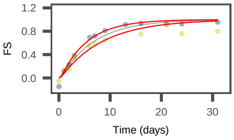

PUR8

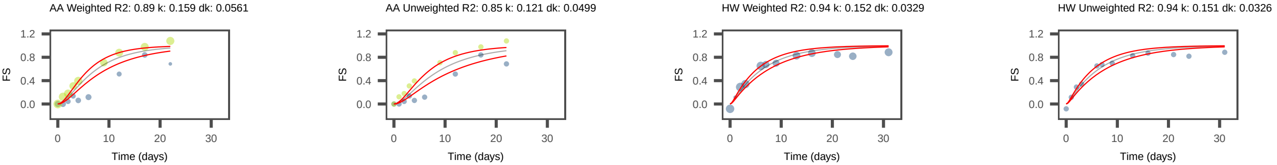

PXL2A

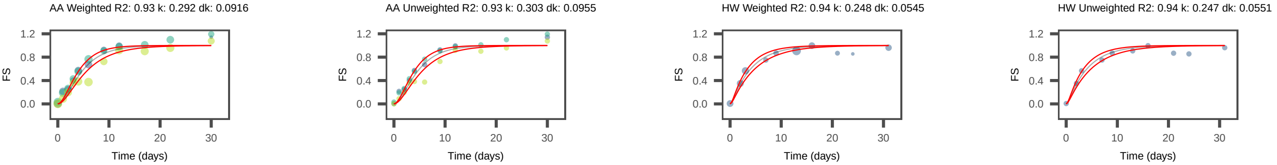

PXMP2

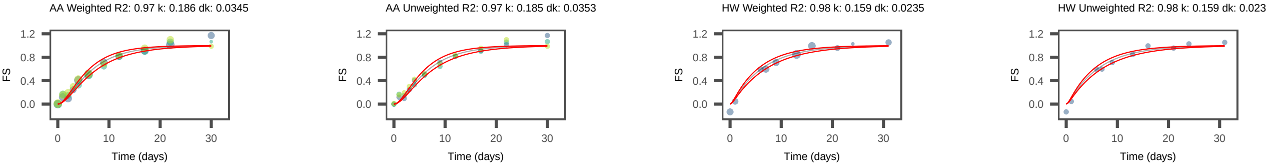

PYC

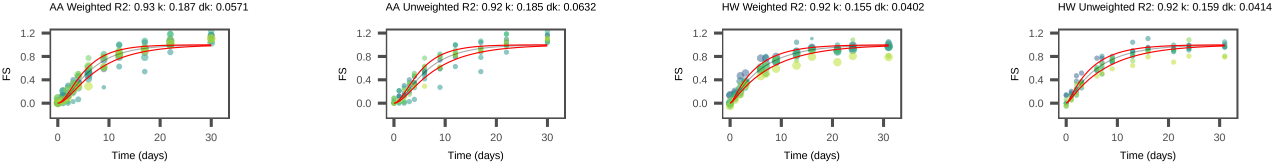

PZP

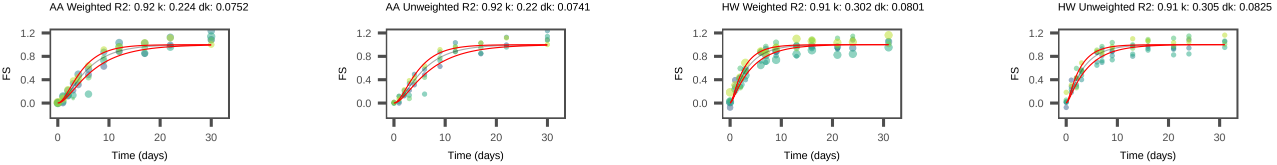

QCR1

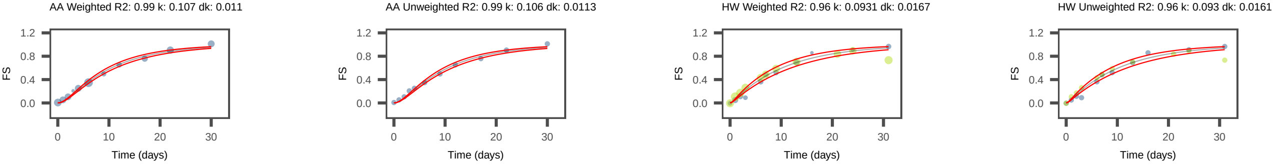

QCR2

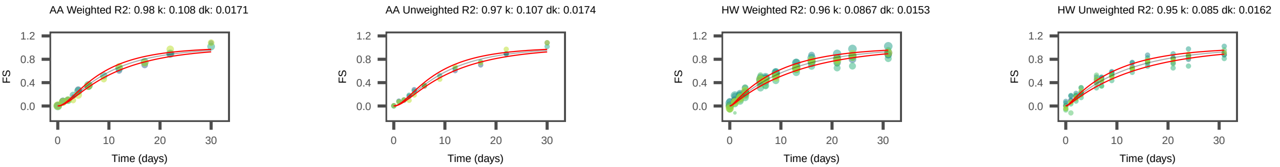

QCR7

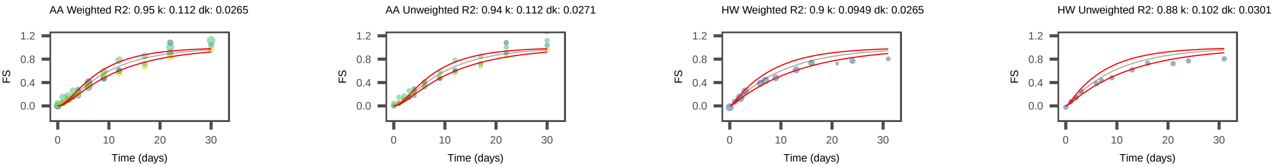

QCR8

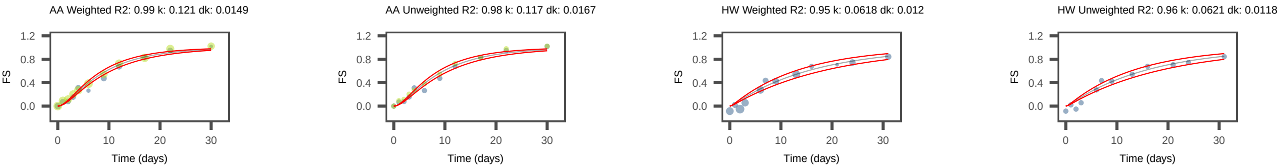

QOR

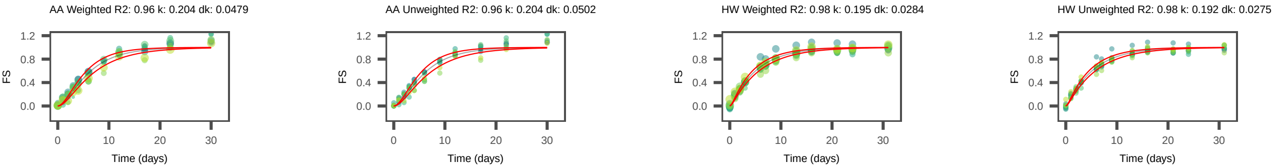

QORL2

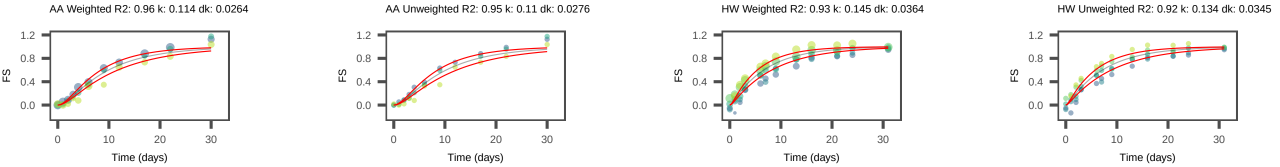

RAB18

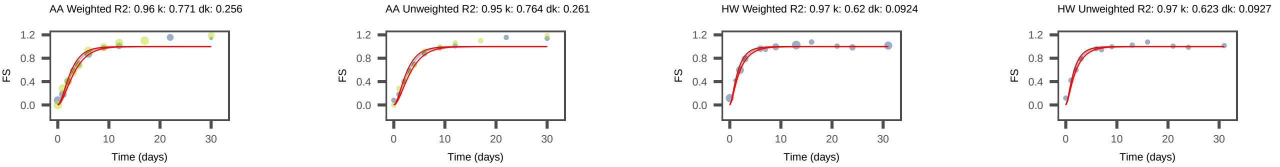

RAB1A

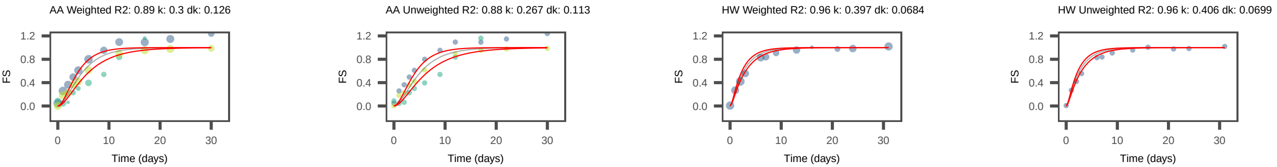

RAB1B

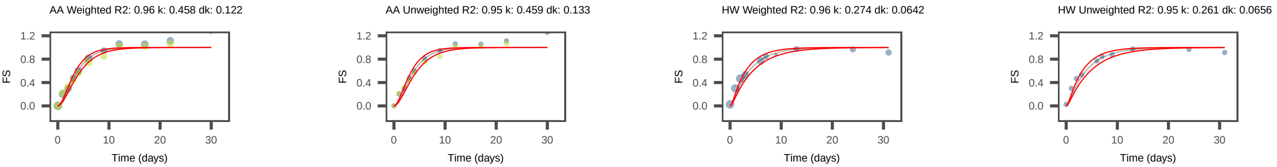

RAB5C

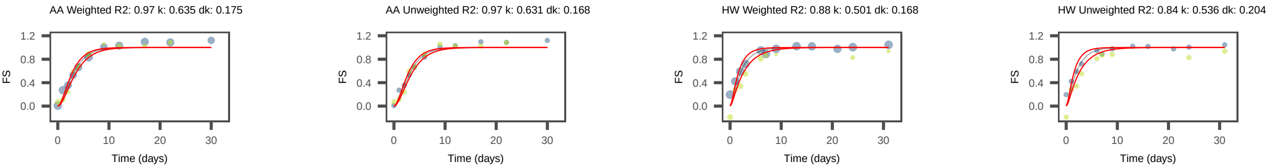

RACK1

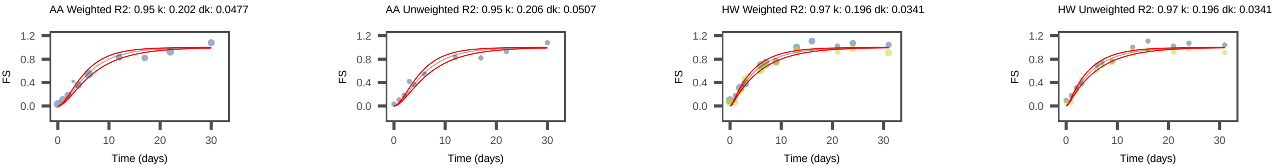

RADI

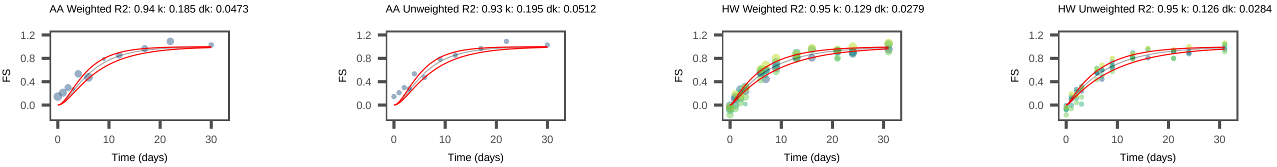

RALA

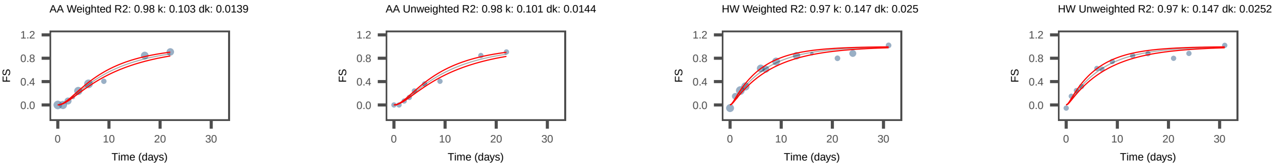

REEP5

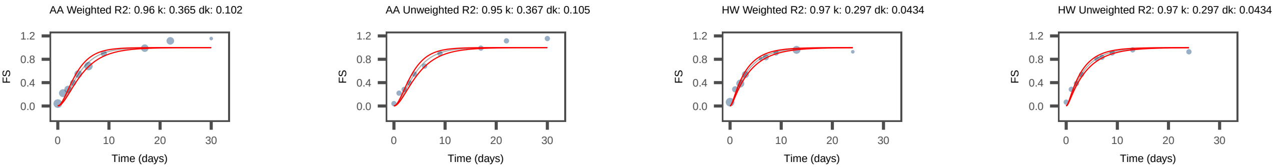

RENT1

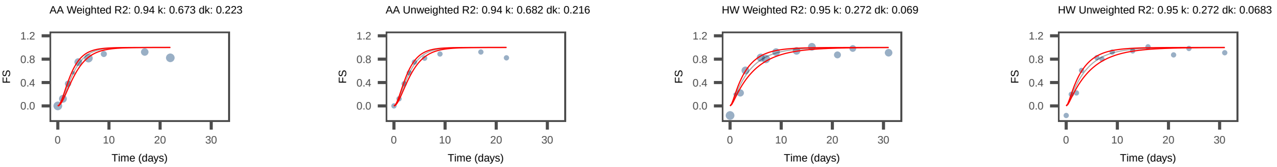

RETST

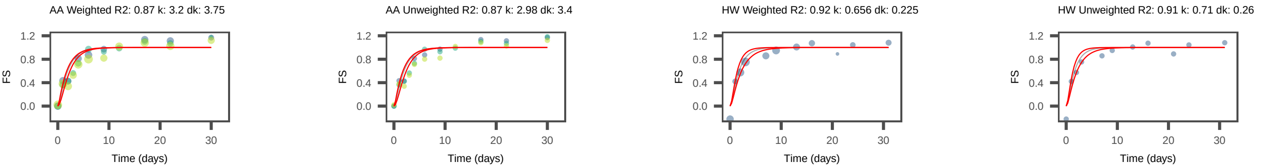

RHOA

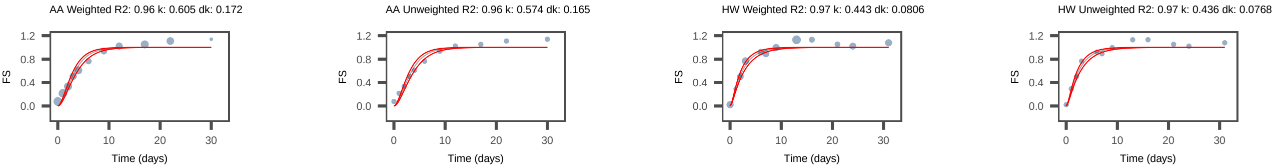

RIDA

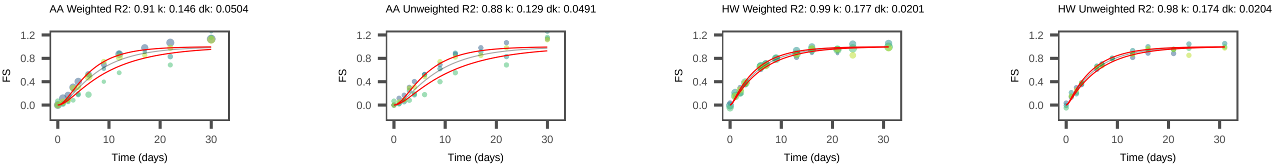

RL11

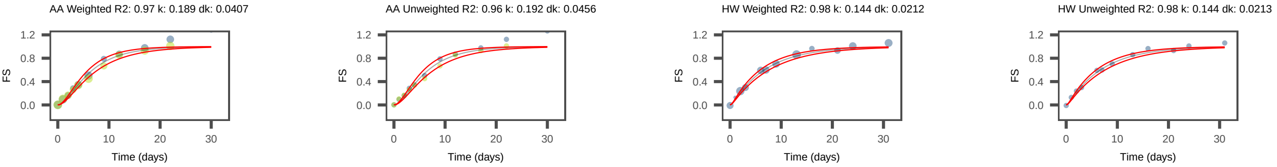

RL12

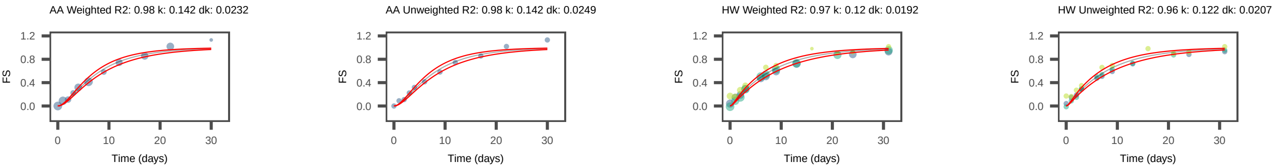

RL13A

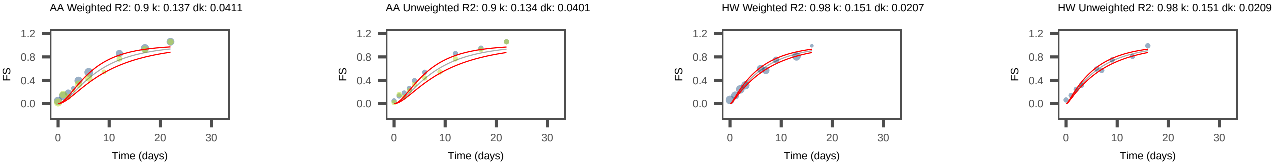

RL15

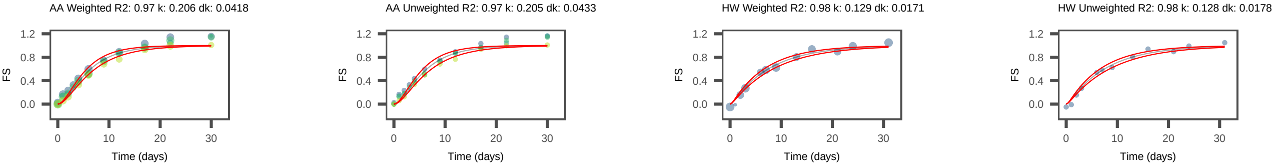

RL17

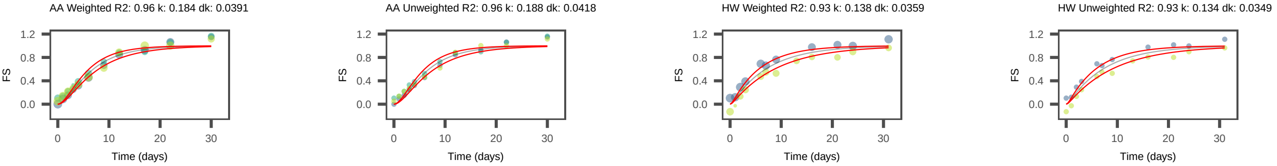

RL18

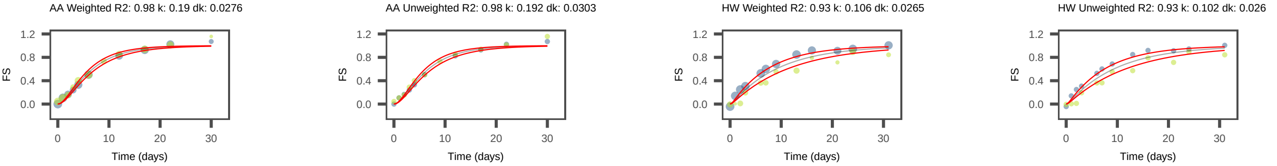

RL23

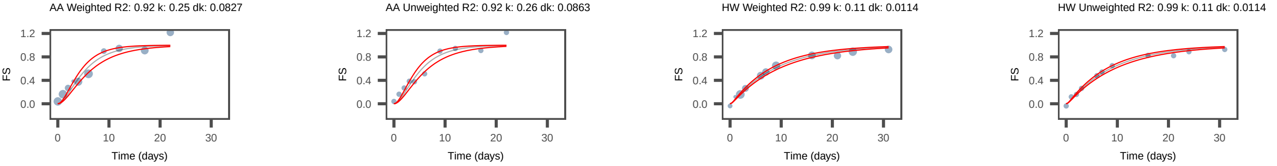

RL26

AA Weighted R2: 0.97 k: 0.19 dk: 0.0394

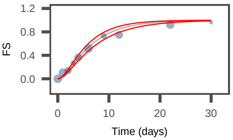

AA Unweighted R2: 0.96 k: 0.196 dk: 0.0481

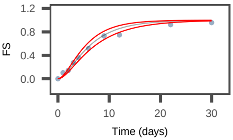

HW Weighted R2: 0.94 k: 0.199 dk: 0.045

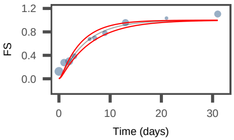

HW Unweighted R2: 0.95 k: 0.198 dk: 0.044

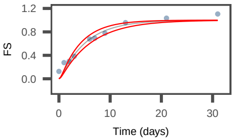

RL27A

AA Weighted R2: 0.98 k: 0.204 dk: 0.0389

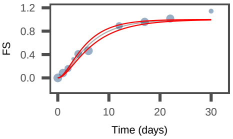

AA Unweighted R2: 0.97 k: 0.209 dk: 0.0417

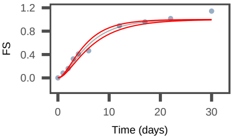

HW Weighted R2: 0.93 k: 0.139 dk: 0.0286

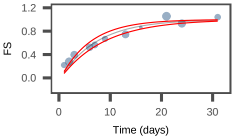

HW Unweighted R2: 0.93 k: 0.139 dk: 0.0284

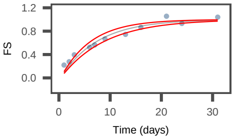

RL28

AA Weighted R2: 0.97 k: 0.234 dk: 0.0576

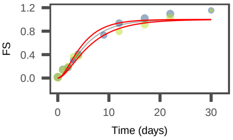

AA Unweighted R2: 0.97 k: 0.235 dk: 0.0613

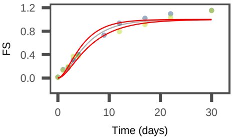

HW Weighted R2: 0.9 k: 0.0912 dk: 0.026

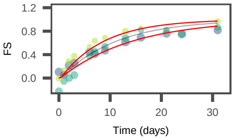

HW Unweighted R2: 0.9 k: 0.093 dk: 0.0266

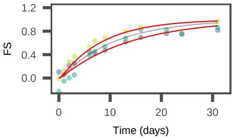

RL3

AA Weighted R2: 0.94 k: 0.157 dk: 0.0403

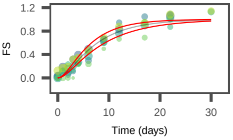

AA Unweighted R2: 0.93 k: 0.147 dk: 0.0408

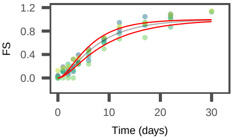

HW Weighted R2: 0.96 k: 0.101 dk: 0.0186

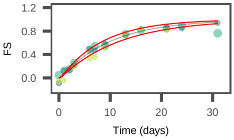

HW Unweighted R2: 0.96 k: 0.101 dk: 0.0183

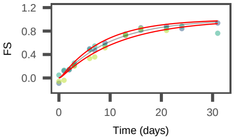

RL31

AA Weighted R2: 0.96 k: 0.202 dk: 0.0424

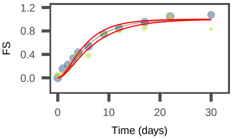

AA Unweighted R2: 0.96 k: 0.191 dk: 0.0432

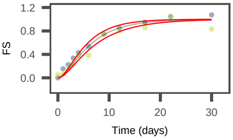

HW Weighted R2: 0.95 k: 0.0968 dk: 0.0216

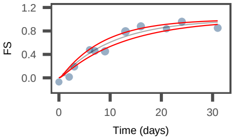

HW Unweighted R2: 0.95 k: 0.0966 dk: 0.0215

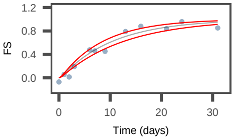

RL38

AA Weighted R2: 0.93 k: 0.214 dk: 0.0645

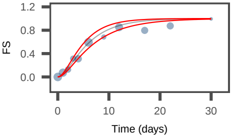

AA Unweighted R2: 0.94 k: 0.202 dk: 0.0562

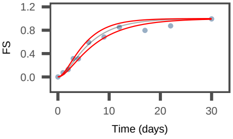

HW Weighted R2: 0.99 k: 0.188 dk: 0.0189

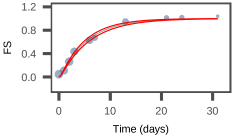

HW Unweighted R2: 0.99 k: 0.188 dk: 0.019

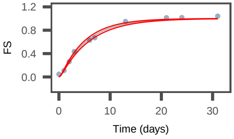

RL4

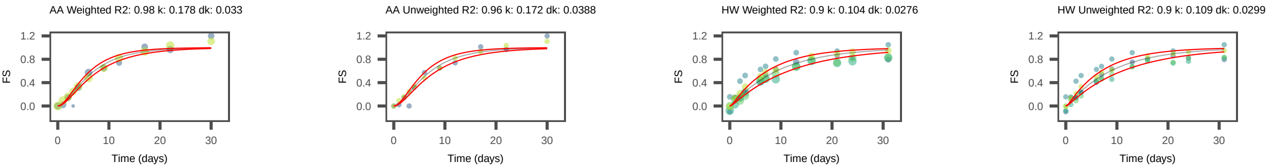

RL5

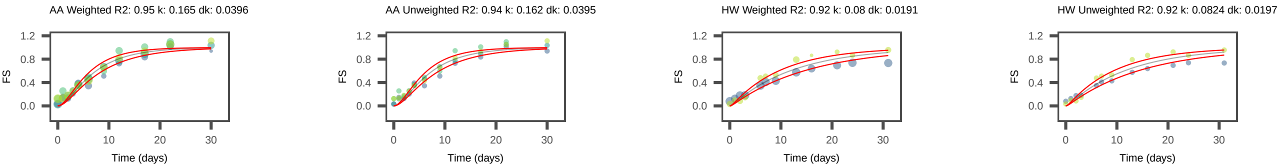

RL6

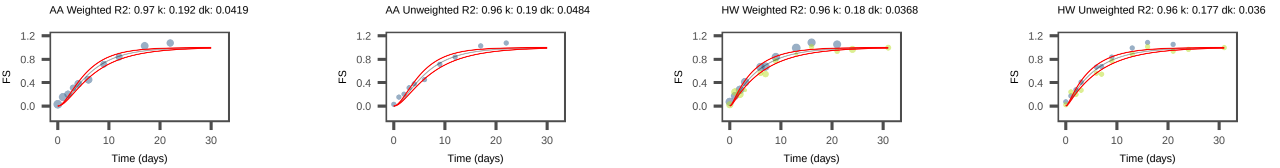

RL7

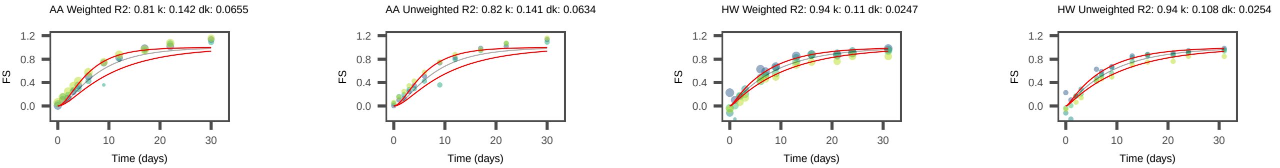

RL7A

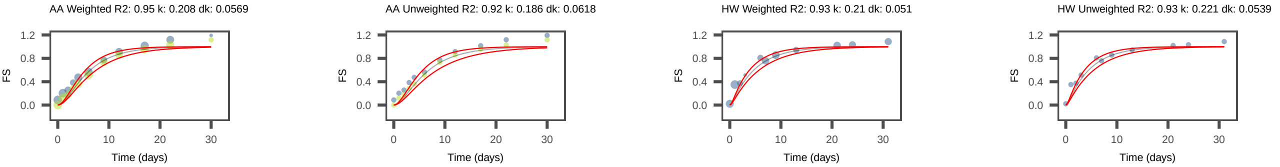

RL9

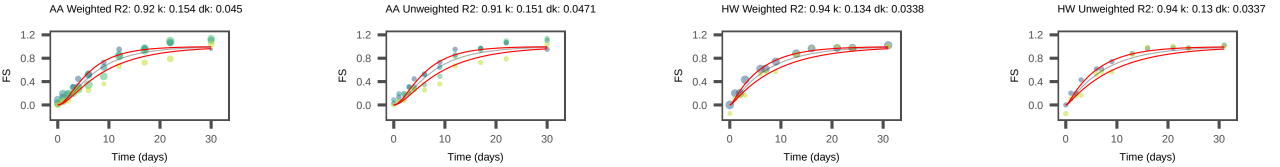

RLA0

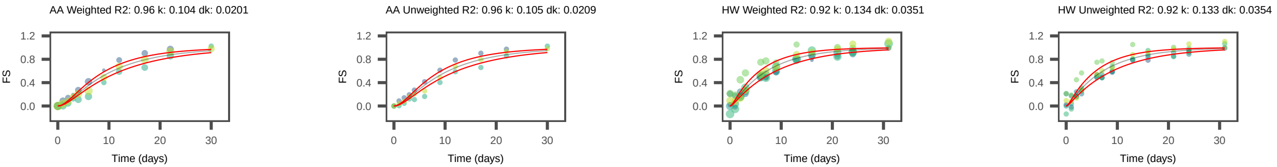

RLA1

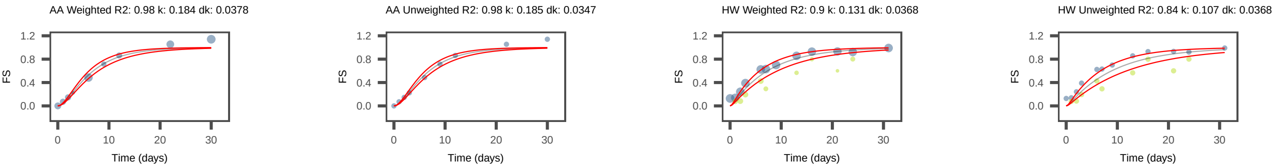

ROA1

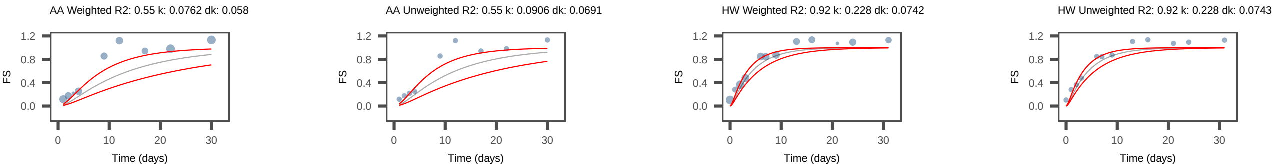

ROA2

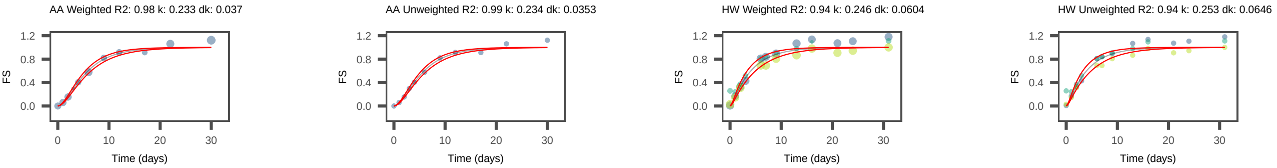

ROA3

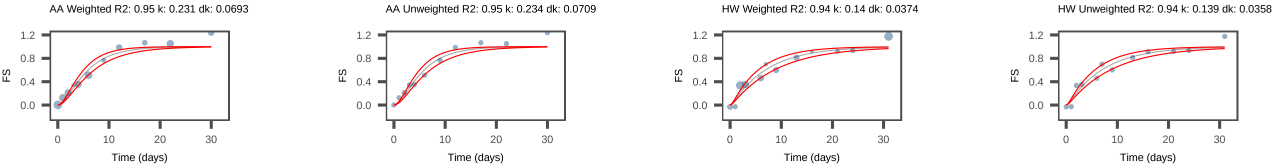

RPN1

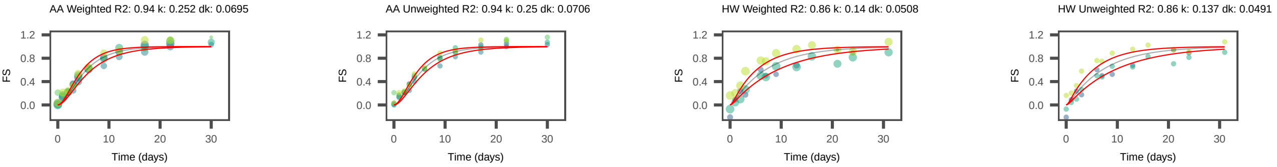

RPN2

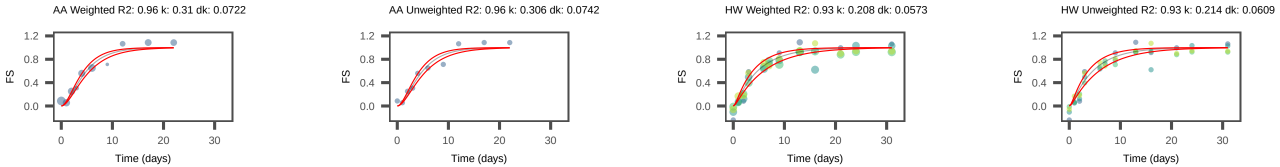

RS10

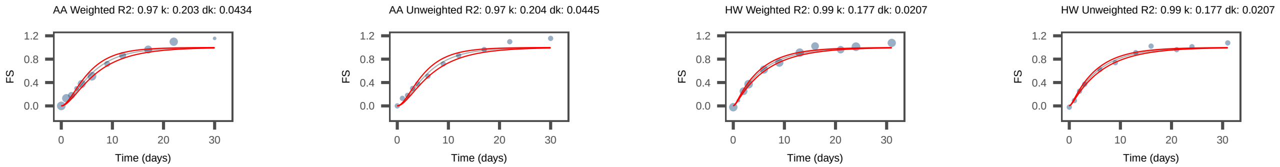

RS11

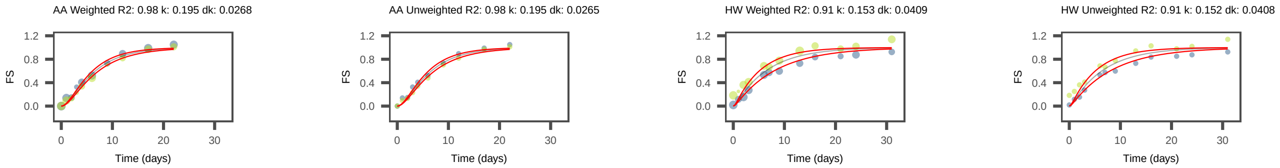

RS15A

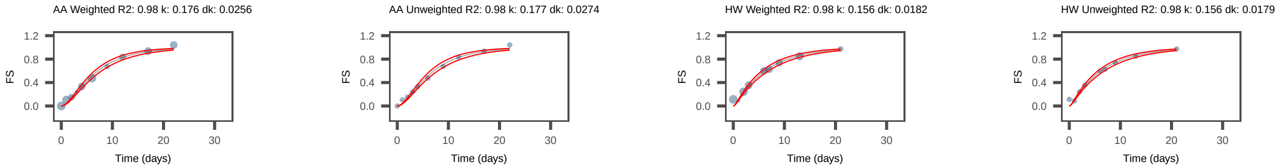

RS16

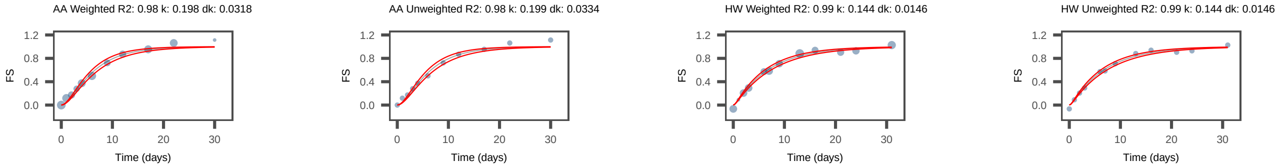

RS17

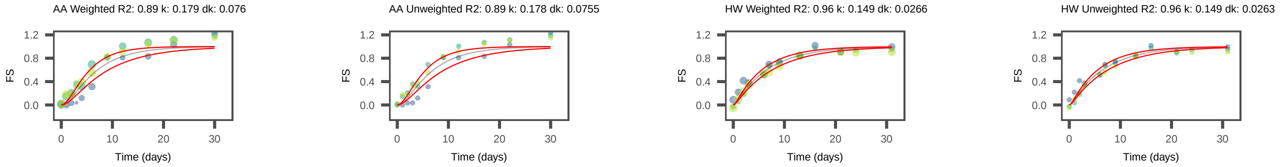

RS19

AA Weighted R2: 0.97 k: 0.191 dk: 0.0361 | AA Unweighted R2: 0.97 k: 0.192 dk: 0.0358 | HW Weighted R2: 0.95 k: 0.115 dk: 0.0248 | HW Unweighted R2: 0.95 k: 0.114 dk: 0.0247

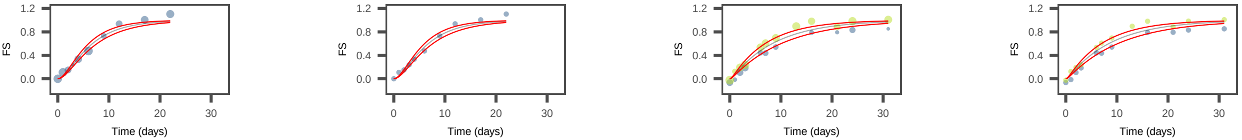

RS2

AA Weighted R2: 0.98 k: 0.176 dk: 0.0275 | AA Unweighted R2: 0.98 k: 0.175 dk: 0.0282 | HW Weighted R2: 0.95 k: 0.107 dk: 0.0218 | HW Unweighted R2: 0.95 k: 0.104 dk: 0.0222

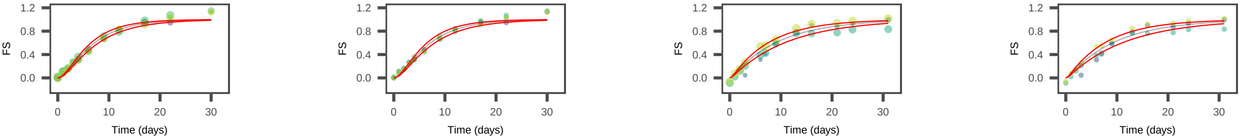

RS23

AA Weighted R2: 0.96 k: 0.172 dk: 0.0352 | AA Unweighted R2: 0.96 k: 0.172 dk: 0.0356 | HW Weighted R2: 0.99 k: 0.113 dk: 0.0114 | HW Unweighted R2: 0.99 k: 0.113 dk: 0.0114

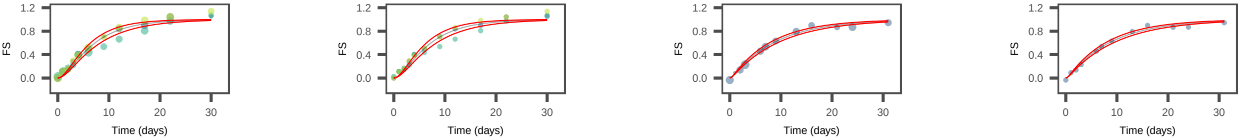

RS24

AA Weighted R2: 0.97 k: 0.176 dk: 0.0364 | AA Unweighted R2: 0.96 k: 0.178 dk: 0.0378 | HW Weighted R2: 0.99 k: 0.153 dk: 0.0154 | HW Unweighted R2: 0.99 k: 0.153 dk: 0.0155

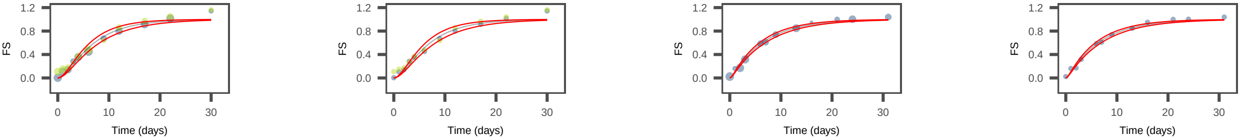

RS25

AA Weighted R2: 0.97 k: 0.215 dk: 0.0469 | AA Unweighted R2: 0.97 k: 0.216 dk: 0.0487 | HW Weighted R2: 0.99 k: 0.146 dk: 0.0122 | HW Unweighted R2: 0.99 k: 0.146 dk: 0.0122

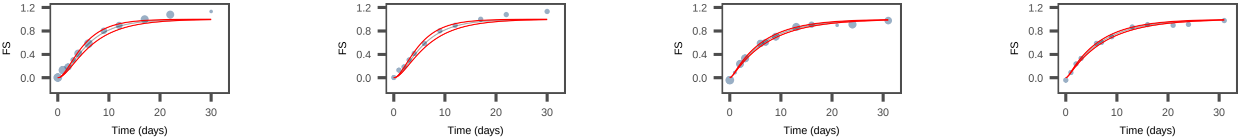

RS3

AA Weighted R2: 0.98 k: 0.179 dk: 0.0237 | AA Unweighted R2: 0.98 k: 0.181 dk: 0.0244 | HW Weighted R2: 0.93 k: 0.131 dk: 0.0341 | HW Unweighted R2: 0.93 k: 0.131 dk: 0.0342

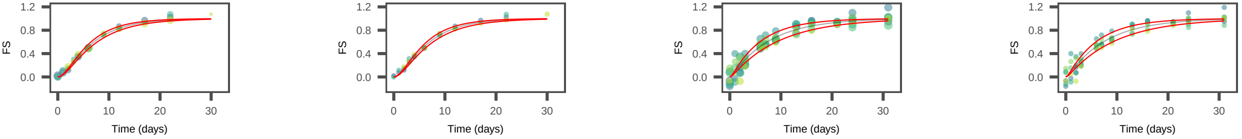

RS4X

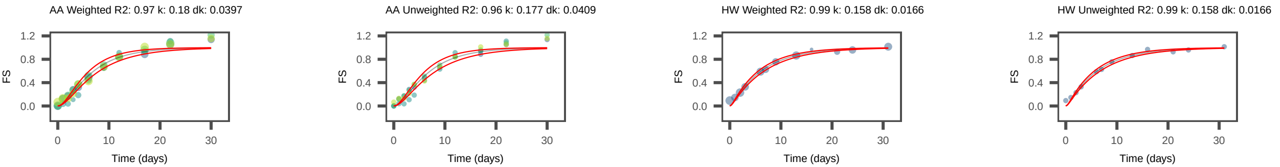

RS7

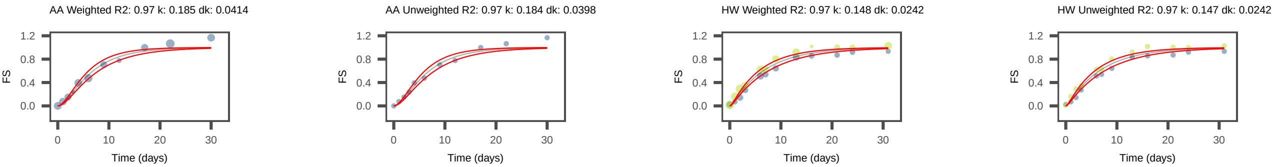

RSSA

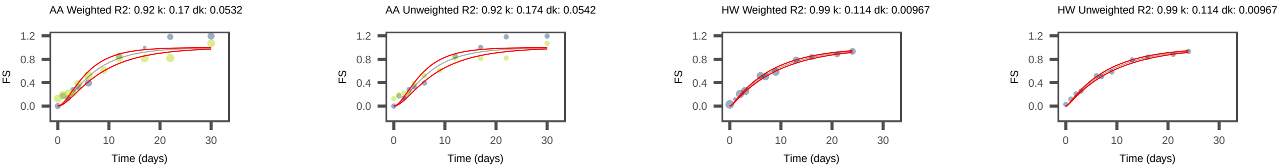

RSU1

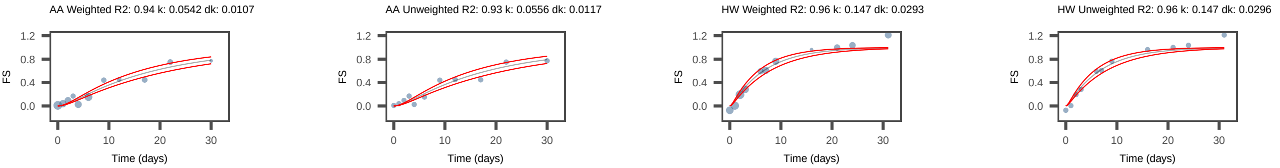

RT36

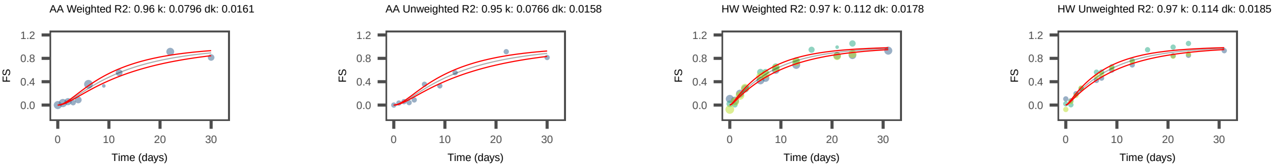

RT4I1

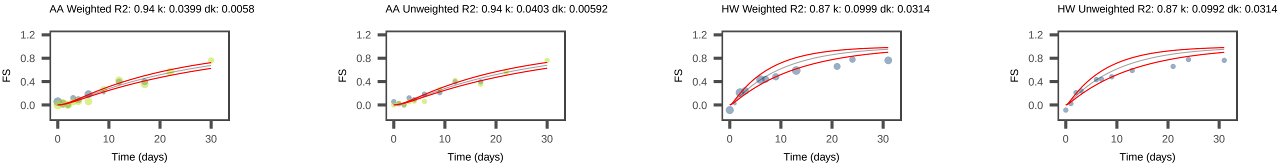

S100G

AA Weighted R2: 0.97 k: 0.354 dk: 0.09 | AA Unweighted R2: 0.97 k: 0.355 dk: 0.0789 | HW Weighted R2: 0.99 k: 0.226 dk: 0.0249 | HW Unweighted R2: 0.98 k: 0.224 dk: 0.0258

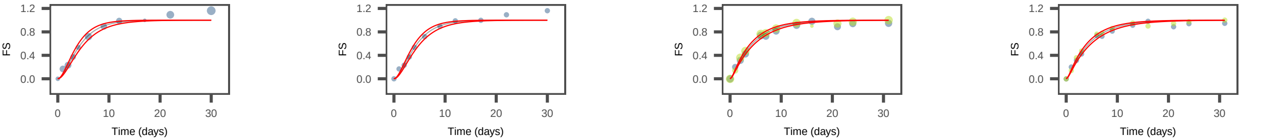

S10A1

AA Weighted R2: 0.96 k: 0.318 dk: 0.0883 | AA Unweighted R2: 0.95 k: 0.325 dk: 0.097 | HW Weighted R2: 0.93 k: 0.503 dk: 0.131 | HW Unweighted R2: 0.93 k: 0.502 dk: 0.133

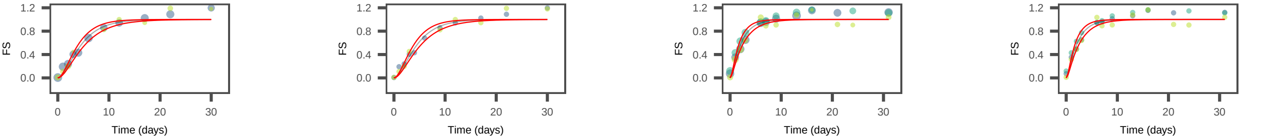

S22A6

AA Weighted R2: 0.95 k: 0.61 dk: 0.2 | AA Unweighted R2: 0.94 k: 0.574 dk: 0.202 | HW Weighted R2: 0.94 k: 0.447 dk: 0.105 | HW Unweighted R2: 0.92 k: 0.448 dk: 0.121

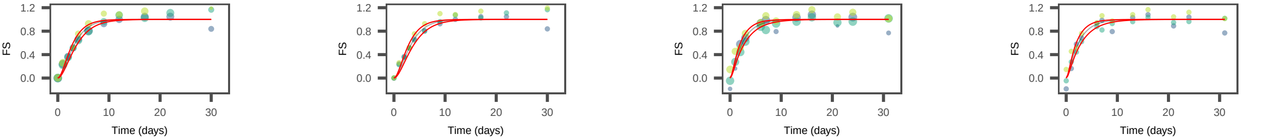

S22AC

AA Weighted R2: 0.94 k: 0.323 dk: 0.105 | AA Unweighted R2: 0.93 k: 0.323 dk: 0.115 | HW Weighted R2: 0.95 k: 0.166 dk: 0.0369 | HW Unweighted R2: 0.95 k: 0.162 dk: 0.0353

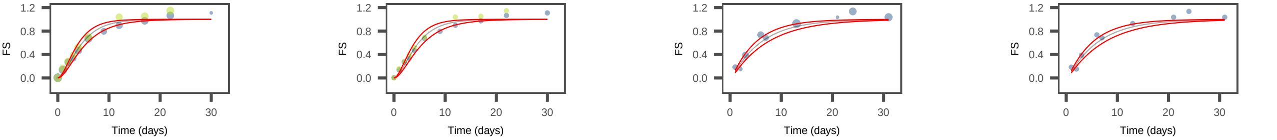

S22AI

AA Weighted R2: 0.95 k: 1.56 dk: 0.768 | AA Unweighted R2: 0.95 k: 1.42 dk: 0.64 | HW Weighted R2: 0.97 k: 0.309 dk: 0.0577 | HW Unweighted R2: 0.97 k: 0.308 dk: 0.0573

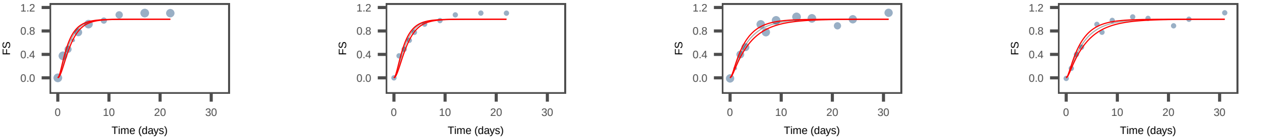

S23A1

AA Weighted R2: 0.82 k: 0.1 dk: 0.0426 | AA Unweighted R2: 0.81 k: 0.11 dk: 0.0484 | HW Weighted R2: 0.92 k: 0.0795 dk: 0.0202 | HW Unweighted R2: 0.93 k: 0.079 dk: 0.019

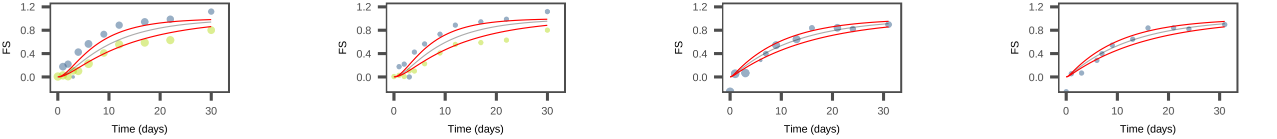

S27A2

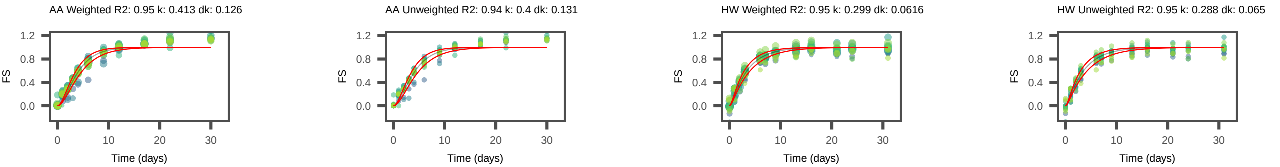

S4A4

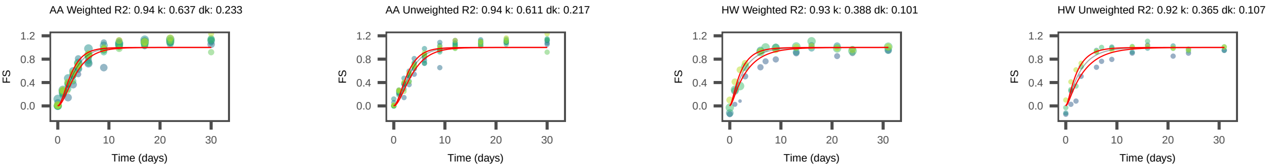

SAHH

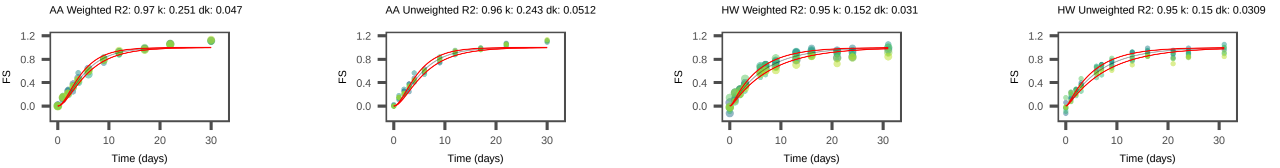

SARDH

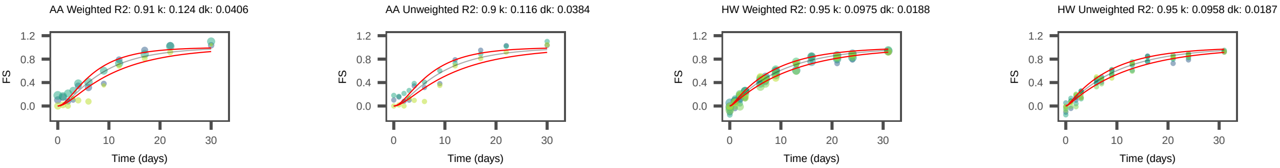

SBP1

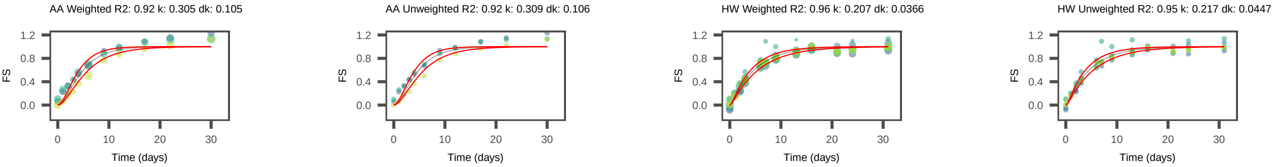

SC31A

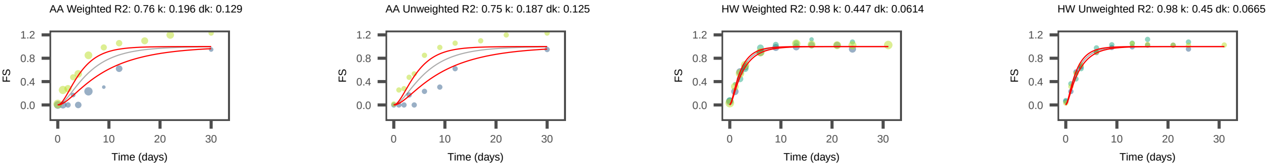

SC5AA

AA Weighted R2: 0.99 k: 0.185 dk: 0.0237

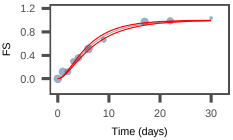

AA Unweighted R2: 0.99 k: 0.184 dk: 0.0238

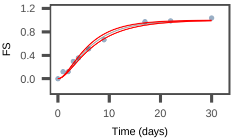

HW Weighted R2: 0.89 k: 0.0918 dk: 0.0301

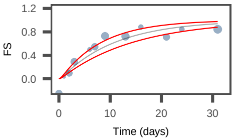

HW Unweighted R2: 0.89 k: 0.0942 dk: 0.031

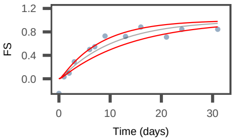

SC5AC

AA Weighted R2: 0.91 k: 0.242 dk: 0.1

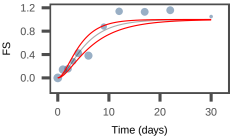

AA Unweighted R2: 0.91 k: 0.248 dk: 0.1

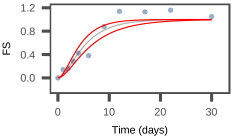

HW Weighted R2: 0.7 k: 0.54 dk: 0.212

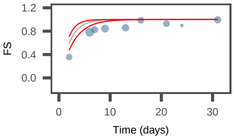

HW Unweighted R2: 0.71 k: 0.608 dk: 0.231

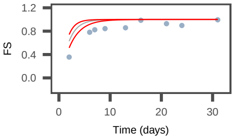

SCOT1

AA Weighted R2: 0.95 k: 0.197 dk: 0.0509

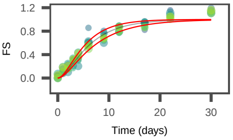

AA Unweighted R2: 0.94 k: 0.192 dk: 0.054

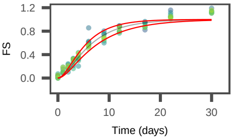

HW Weighted R2: 0.94 k: 0.157 dk: 0.0335

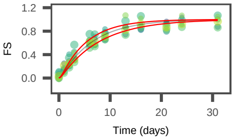

HW Unweighted R2: 0.94 k: 0.157 dk: 0.0336

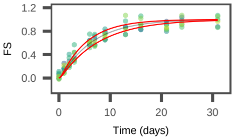

SCP2

AA Weighted R2: 0.92 k: 0.244 dk: 0.0759

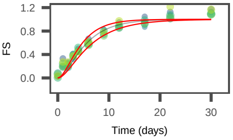

AA Unweighted R2: 0.91 k: 0.238 dk: 0.0791

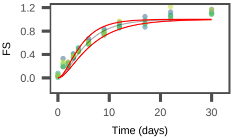

HW Weighted R2: 0.94 k: 0.202 dk: 0.0477

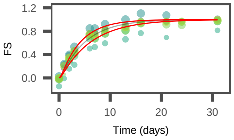

HW Unweighted R2: 0.93 k: 0.195 dk: 0.0494

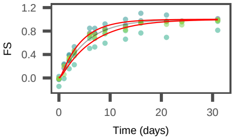

SDHA

AA Weighted R2: 0.92 k: 0.152 dk: 0.0449

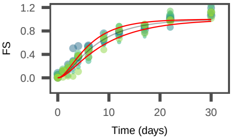

AA Unweighted R2: 0.92 k: 0.148 dk: 0.0443

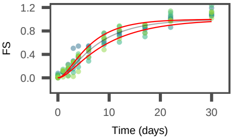

HW Weighted R2: 0.95 k: 0.126 dk: 0.026

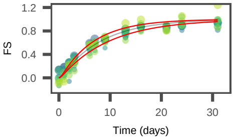

HW Unweighted R2: 0.95 k: 0.121 dk: 0.026

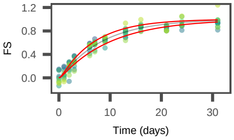

SDHB

AA Weighted R2: 0.97 k: 0.171 dk: 0.0319

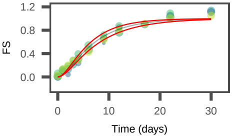

AA Unweighted R2: 0.97 k: 0.169 dk: 0.0322

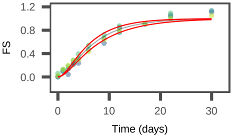

HW Weighted R2: 0.92 k: 0.118 dk: 0.0313

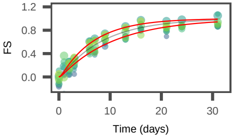

HW Unweighted R2: 0.91 k: 0.112 dk: 0.0309

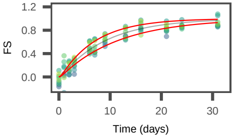

SFXN1

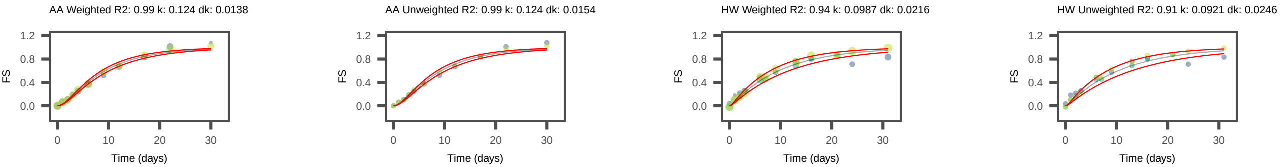

SLC31

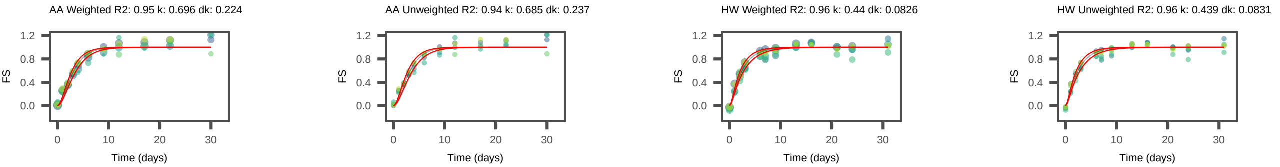

SMD3

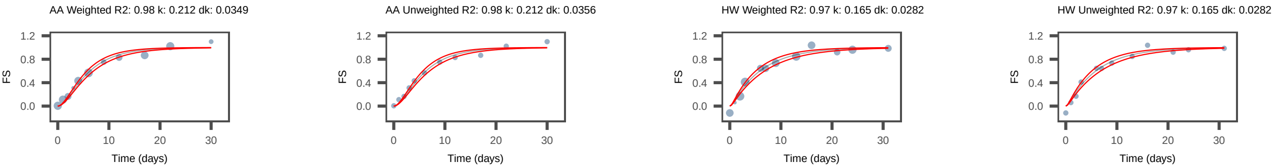

SND1

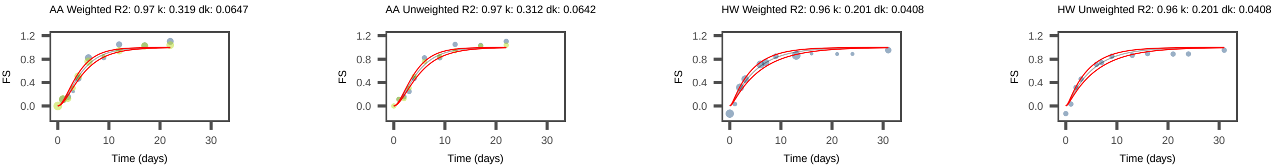

SNX2

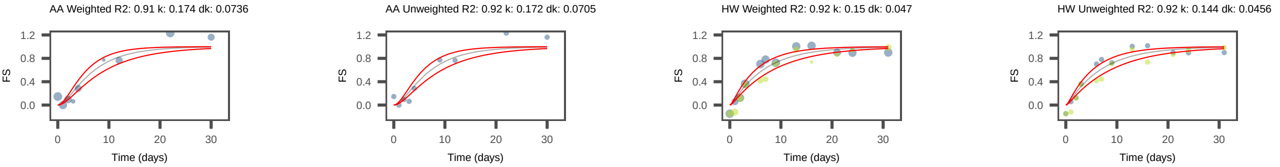

SODC

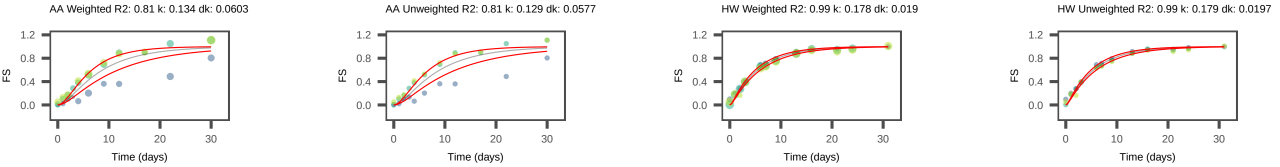

SODM

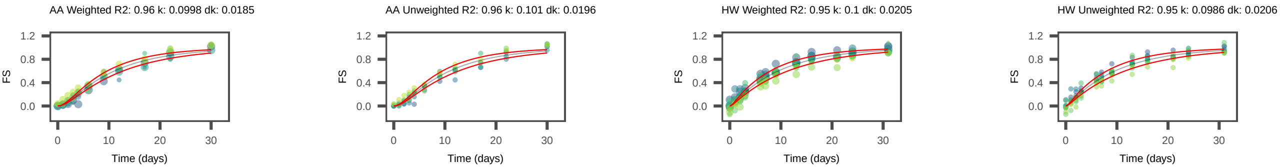

SORCN

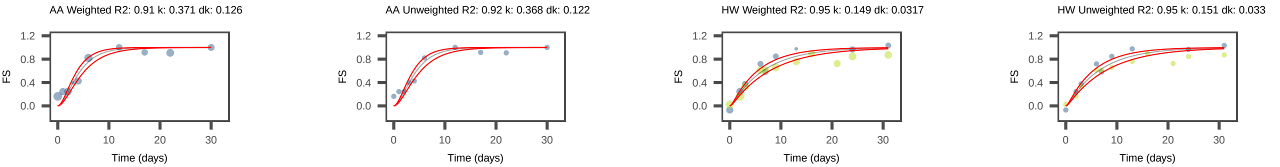

SOX

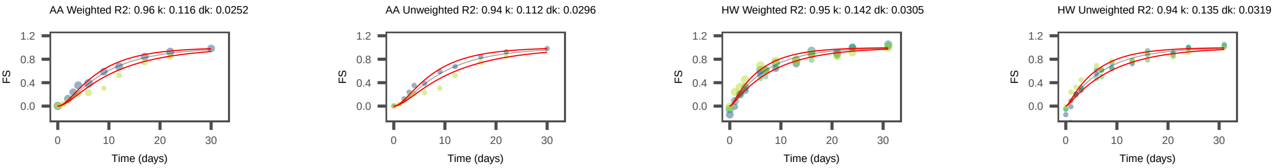

SPTB2

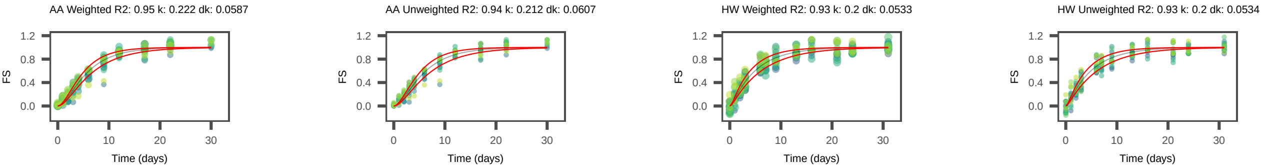

SPTN1

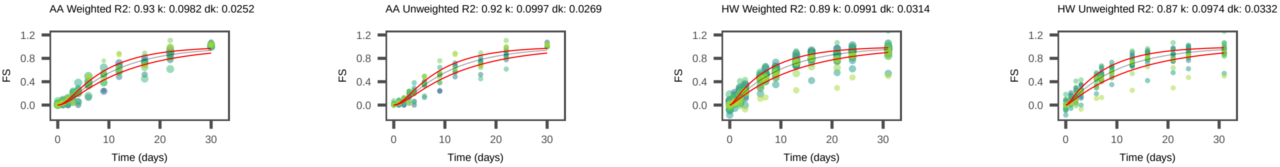

SQOR

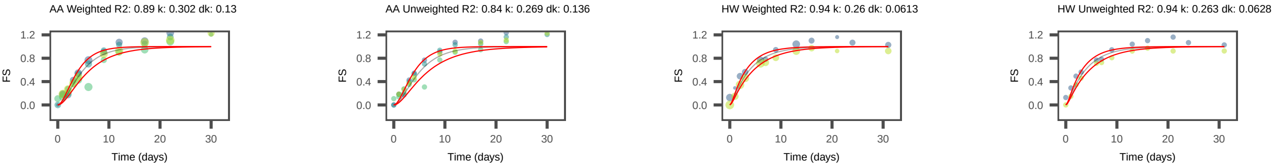

SSDH

AA Weighted R2: 0.92 k: 0.177 dk: 0.0569 | AA Unweighted R2: 0.93 k: 0.16 dk: 0.0468 | HW Weighted R2: 0.94 k: 0.11 dk: 0.024 | HW Unweighted R2: 0.94 k: 0.109 dk: 0.024

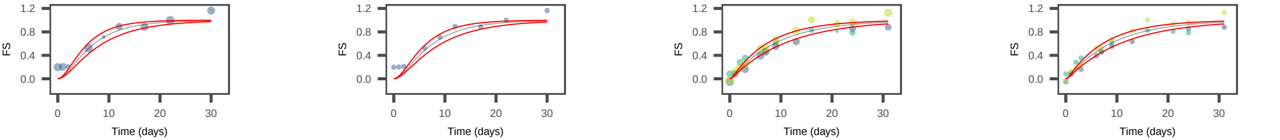

ST1C2

AA Weighted R2: 0.96 k: 0.271 dk: 0.0709 | AA Unweighted R2: 0.96 k: 0.272 dk: 0.068 | HW Weighted R2: 0.95 k: 0.187 dk: 0.0418 | HW Unweighted R2: 0.94 k: 0.185 dk: 0.044

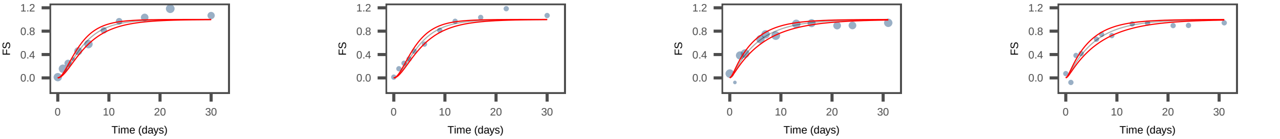

ST1D1

AA Weighted R2: 0.86 k: 0.346 dk: 0.17 | AA Unweighted R2: 0.8 k: 0.31 dk: 0.177 | HW Weighted R2: 0.93 k: 0.291 dk: 0.0735 | HW Unweighted R2: 0.93 k: 0.291 dk: 0.0763

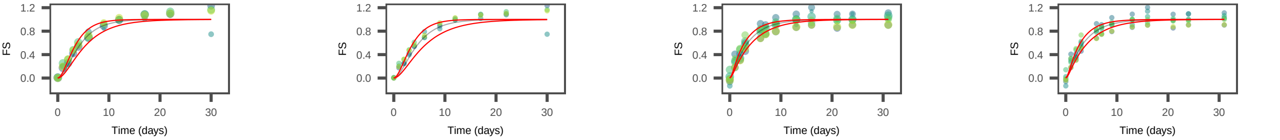

STIP1

AA Weighted R2: 0.92 k: 0.27 dk: 0.114 | AA Unweighted R2: 0.91 k: 0.277 dk: 0.122 | HW Weighted R2: 0.96 k: 0.277 dk: 0.0564 | HW Unweighted R2: 0.96 k: 0.276 dk: 0.0555

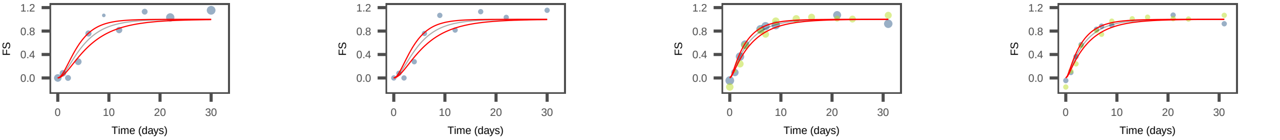

SUCA

AA Weighted R2: 0.97 k: 0.128 dk: 0.0225 | AA Unweighted R2: 0.96 k: 0.127 dk: 0.0247 | HW Weighted R2: 0.94 k: 0.0844 dk: 0.0183 | HW Unweighted R2: 0.93 k: 0.0838 dk: 0.0188

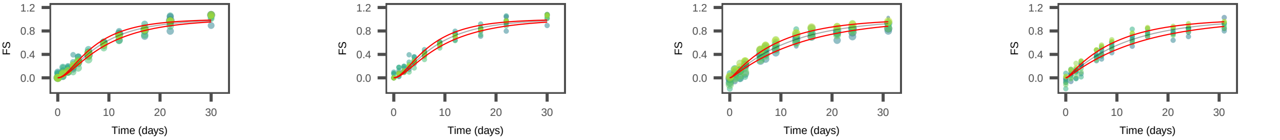

SUCB1

AA Weighted R2: 0.95 k: 0.127 dk: 0.0287 | AA Unweighted R2: 0.94 k: 0.127 dk: 0.0312 | HW Weighted R2: 0.91 k: 0.128 dk: 0.0362 | HW Unweighted R2: 0.9 k: 0.131 dk: 0.0379

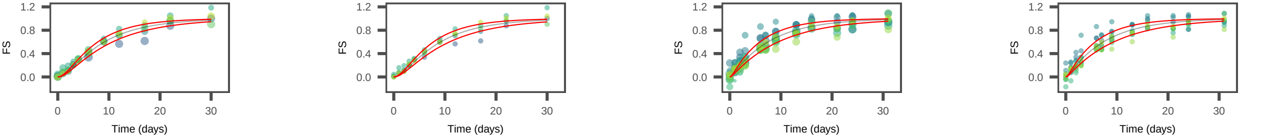

SUCB2

AA Weighted R2: 0.97 k: 0.145 dk: 0.0304

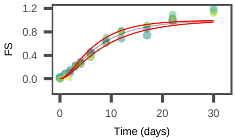

AA Unweighted R2: 0.96 k: 0.141 dk: 0.0343

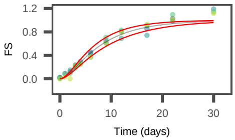

HW Weighted R2: 0.9 k: 0.104 dk: 0.0303

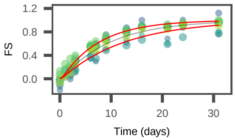

HW Unweighted R2: 0.9 k: 0.103 dk: 0.0301

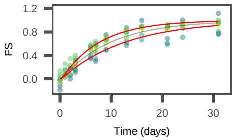

SUCHY

AA Weighted R2: 0.91 k: 0.258 dk: 0.0789

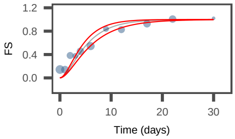

AA Unweighted R2: 0.91 k: 0.264 dk: 0.0789

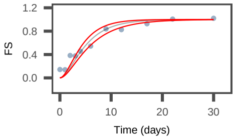

HW Weighted R2: 0.9 k: 0.192 dk: 0.0592

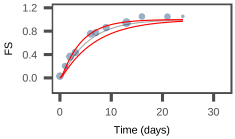

HW Unweighted R2: 0.91 k: 0.2 dk: 0.0596

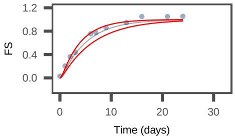

SUSD2

AA Weighted R2: 0.96 k: 0.356 dk: 0.0953

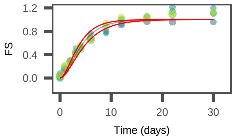

AA Unweighted R2: 0.95 k: 0.361 dk: 0.0999

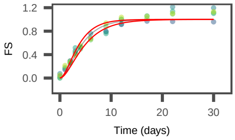

HW Weighted R2: 0.96 k: 0.334 dk: 0.0683

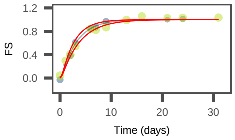

HW Unweighted R2: 0.95 k: 0.345 dk: 0.0818

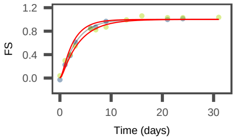

SYDC

AA Weighted R2: 0.91 k: 0.155 dk: 0.04

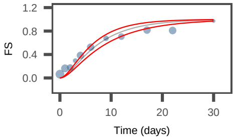

AA Unweighted R2: 0.92 k: 0.156 dk: 0.0394

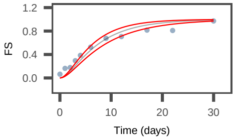

HW Weighted R2: 0.92 k: 0.28 dk: 0.0762

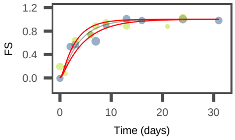

HW Unweighted R2: 0.92 k: 0.281 dk: 0.0754

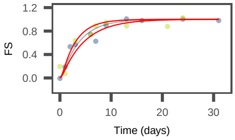

SYEP

AA Weighted R2: 0.94 k: 0.277 dk: 0.093

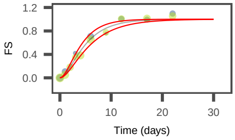

AA Unweighted R2: 0.92 k: 0.281 dk: 0.106

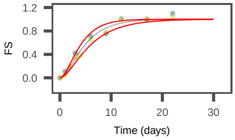

HW Weighted R2: 0.95 k: 0.153 dk: 0.0342

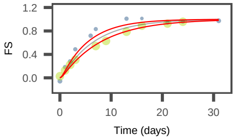

HW Unweighted R2: 0.94 k: 0.196 dk: 0.0495

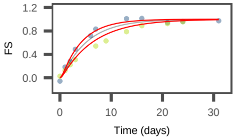

SYFA

AA Weighted R2: 0.66 k: 0.0428 dk: 0.0225

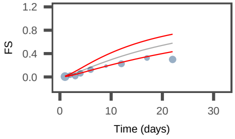

AA Unweighted R2: 0.56 k: 0.0592 dk: 0.0352

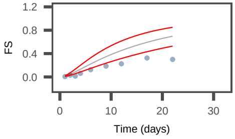

HW Weighted R2: 0.85 k: 0.0647 dk: 0.0228

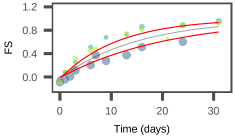

HW Unweighted R2: 0.86 k: 0.0765 dk: 0.0261

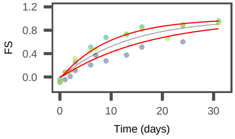

SYIM

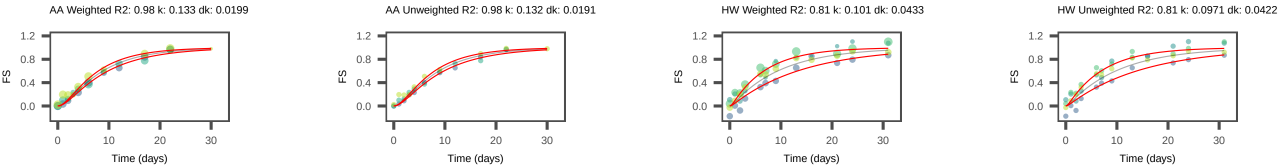

TADBP

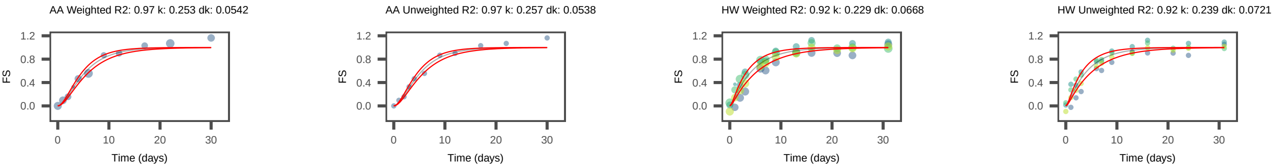

TAGL2

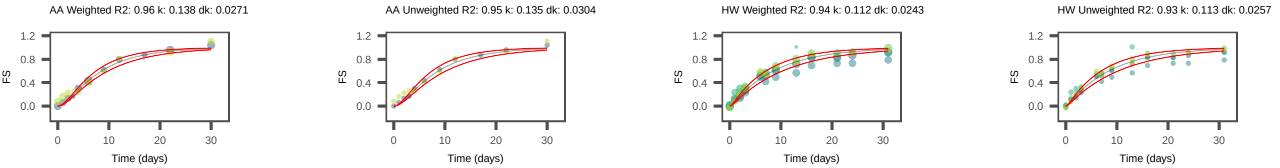

TALDO

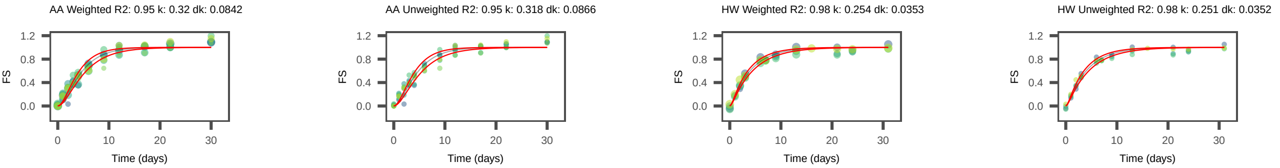

TCPA

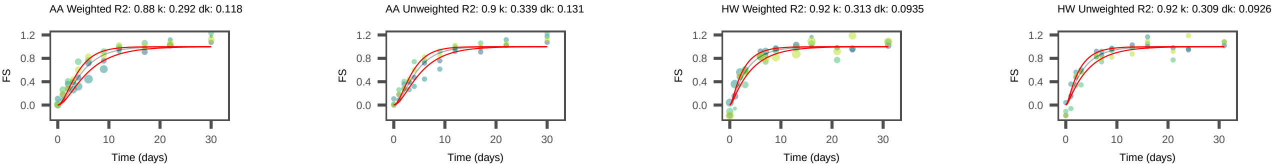

TCPD

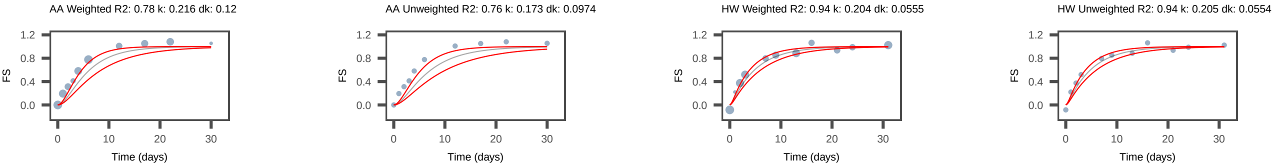

TCPH

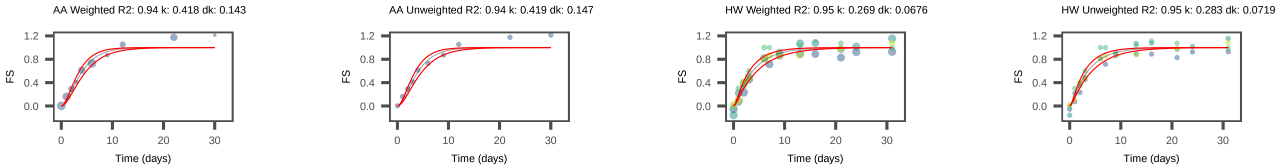

TCPQ

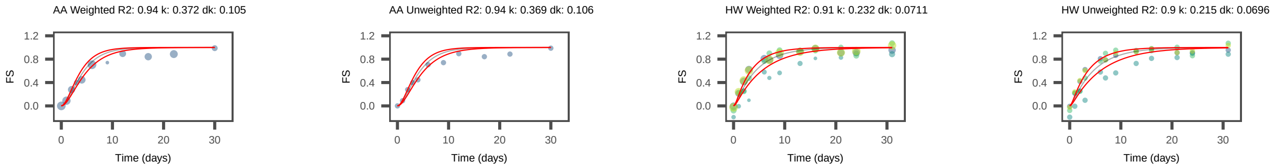

TERA

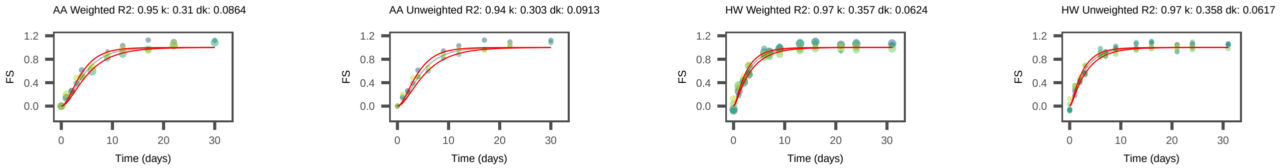

TGM2

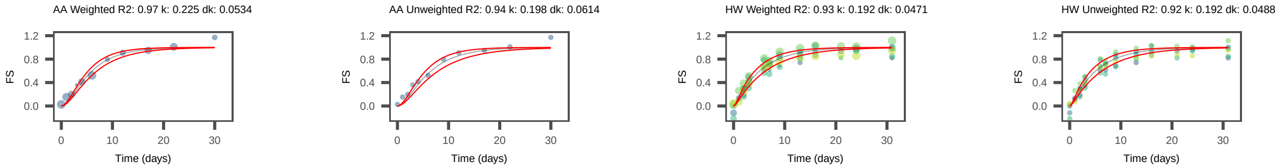

THIKA

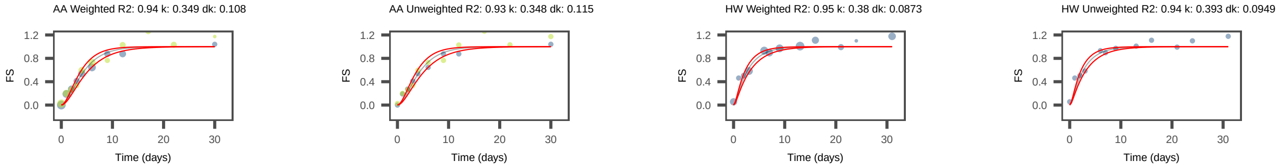

THIL

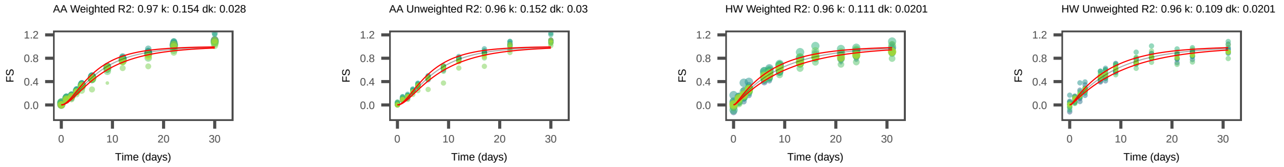

THIM

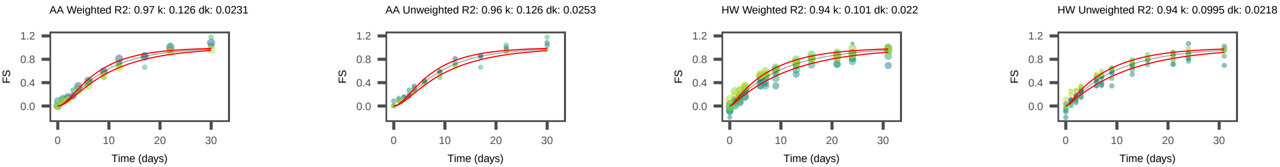

THNS2

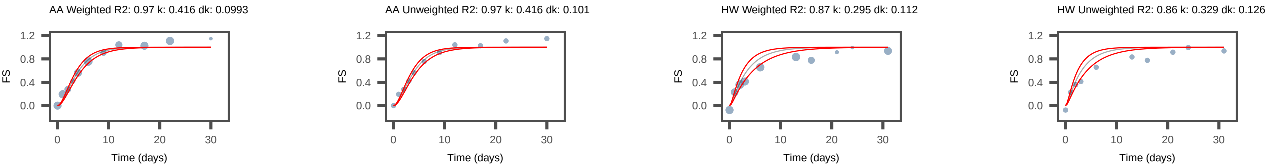

THTR

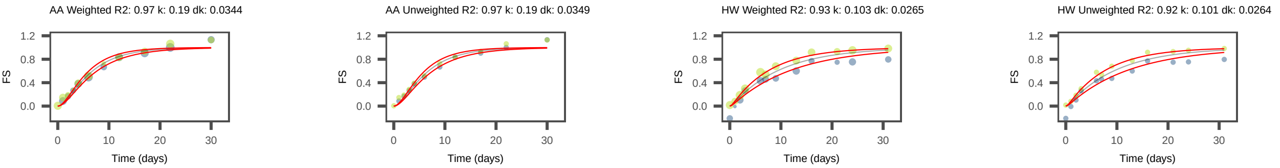

TIM50

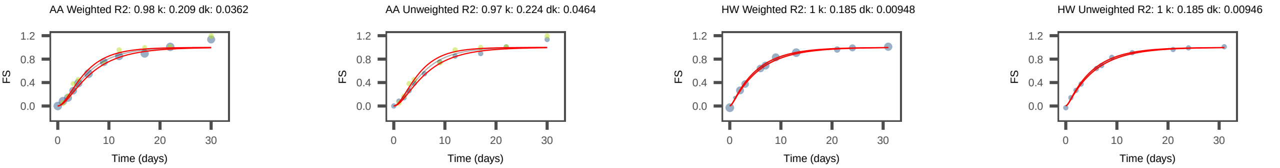

TKFC

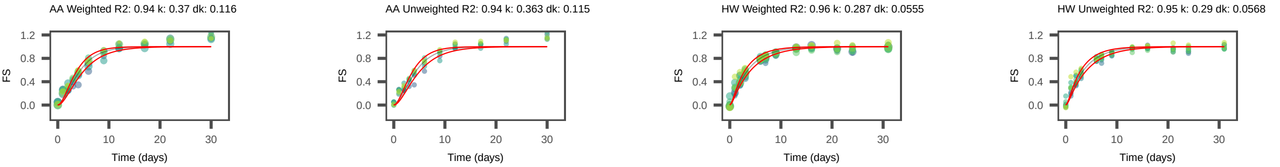

TKT

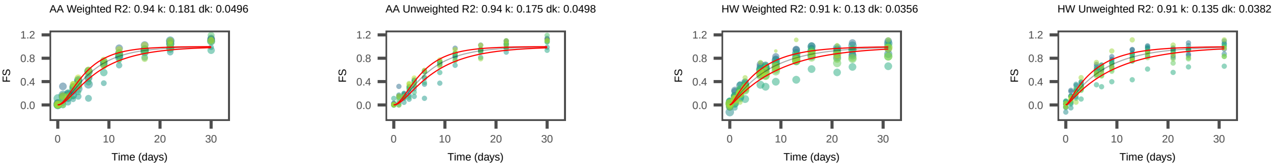

TLN1

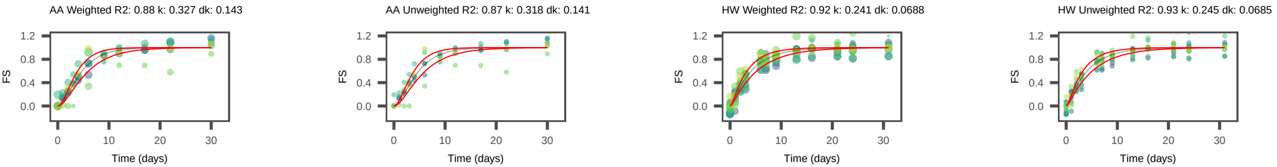

TMEDA

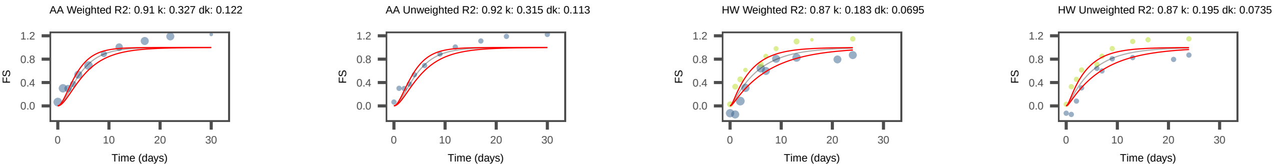

TMM19

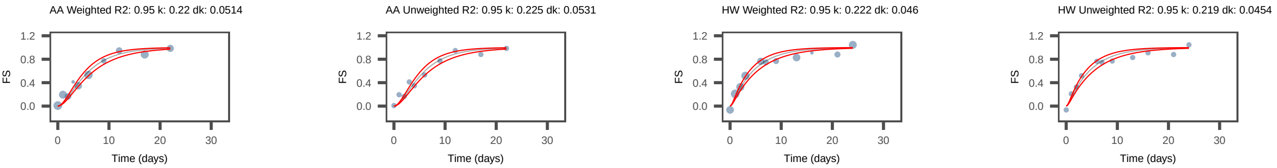

TOM40

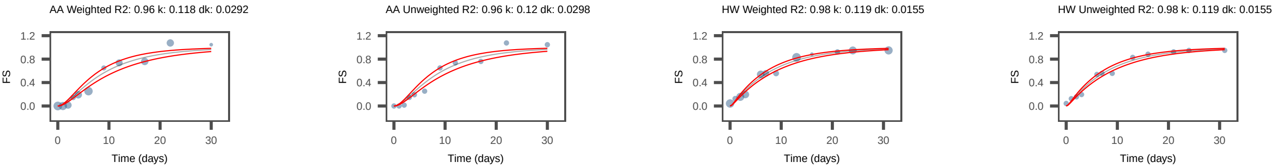

TOM70

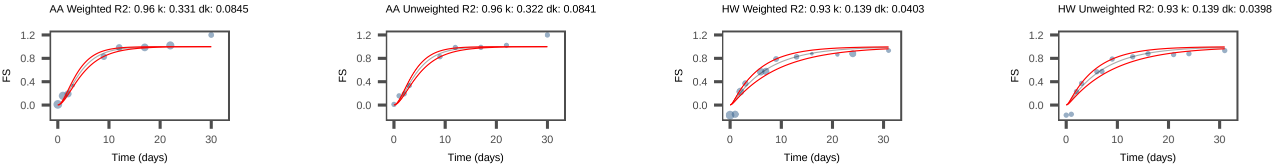

TPIS

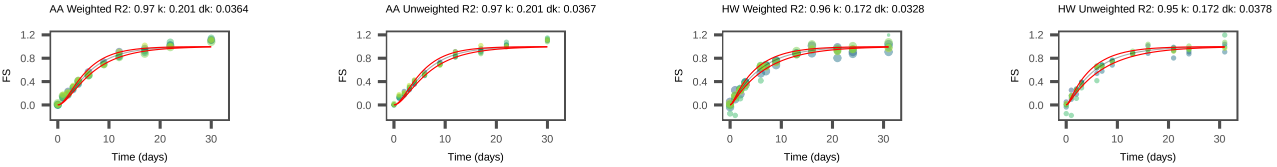

TPMT

AA Weighted R2: 0.96 k: 0.275 dk: 0.0656 | AA Unweighted R2: 0.96 k: 0.265 dk: 0.0662 | HW Weighted R2: 0.94 k: 0.256 dk: 0.0663 | HW Unweighted R2: 0.95 k: 0.252 dk: 0.0614

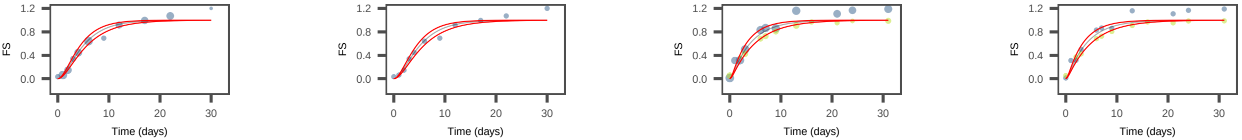

TRAP1

AA Weighted R2: 0.91 k: 0.349 dk: 0.142 | AA Unweighted R2: 0.9 k: 0.348 dk: 0.143 | HW Weighted R2: 0.9 k: 0.294 dk: 0.0805 | HW Unweighted R2: 0.9 k: 0.296 dk: 0.0804

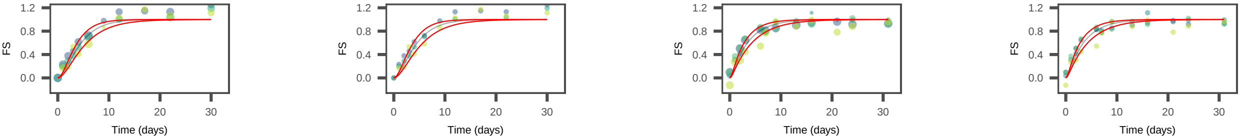

TRFE

AA Weighted R2: 0.92 k: 0.472 dk: 0.187 | AA Unweighted R2: 0.92 k: 0.443 dk: 0.176 | HW Weighted R2: 0.95 k: 0.422 dk: 0.0854 | HW Unweighted R2: 0.95 k: 0.424 dk: 0.0904

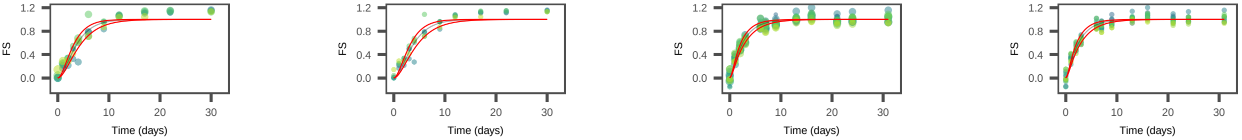

TXTP

AA Weighted R2: 0.97 k: 0.134 dk: 0.0257 | AA Unweighted R2: 0.95 k: 0.138 dk: 0.0326 | HW Weighted R2: 0.89 k: 0.114 dk: 0.033 | HW Unweighted R2: 0.89 k: 0.111 dk: 0.0312

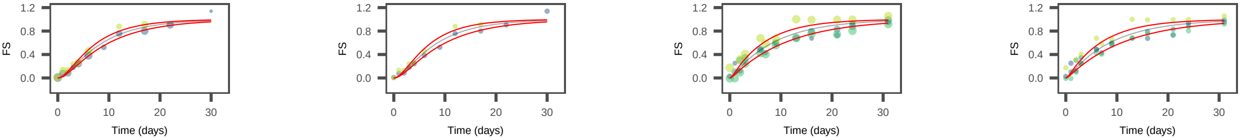

UBA1

AA Weighted R2: 0.95 k: 0.235 dk: 0.0604 | AA Unweighted R2: 0.95 k: 0.228 dk: 0.0591 | HW Weighted R2: 0.95 k: 0.229 dk: 0.0509 | HW Unweighted R2: 0.95 k: 0.232 dk: 0.0532

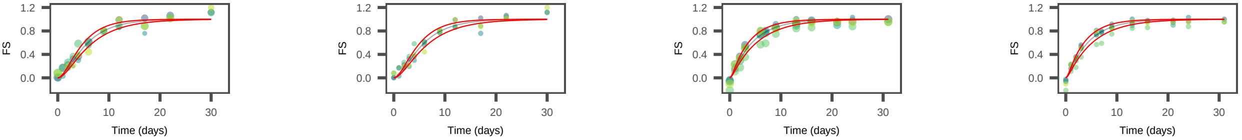

UCRI

AA Weighted R2: 0.93 k: 0.164 dk: 0.0468 | AA Unweighted R2: 0.93 k: 0.156 dk: 0.0447 | HW Weighted R2: 0.93 k: 0.113 dk: 0.0265 | HW Unweighted R2: 0.92 k: 0.111 dk: 0.0277

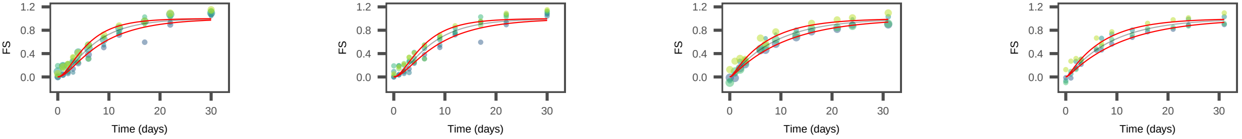

UD17

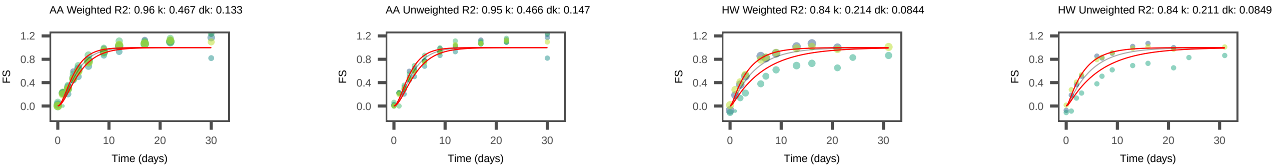

UGDH

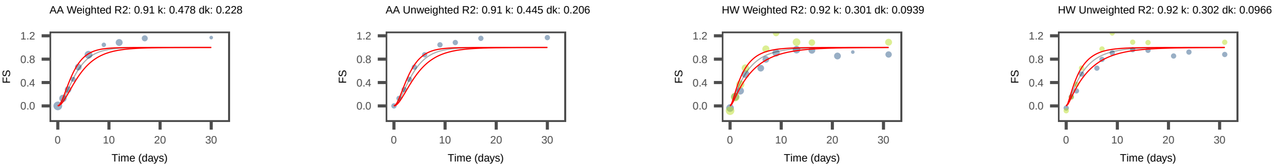

UGPA

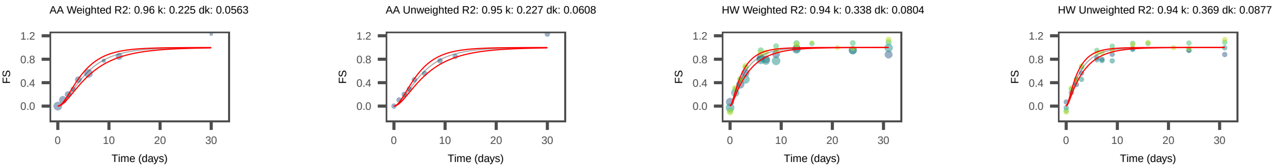

VA0D1

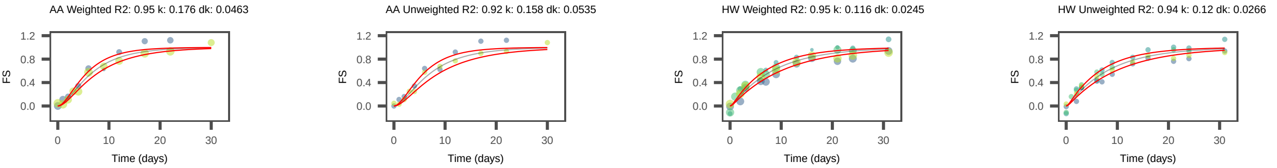

VATA

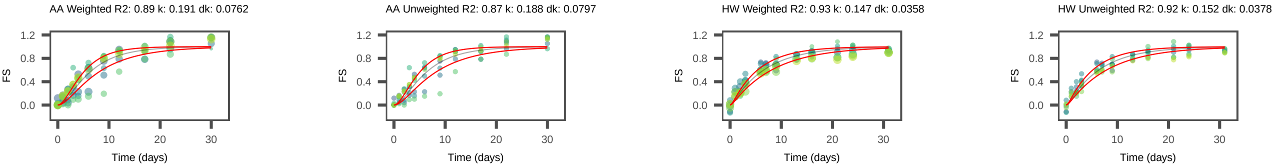

VATB2

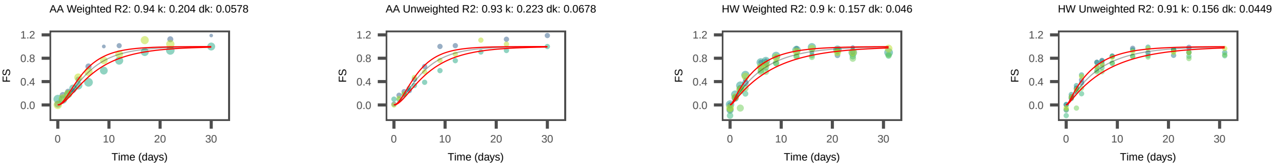

VATC1

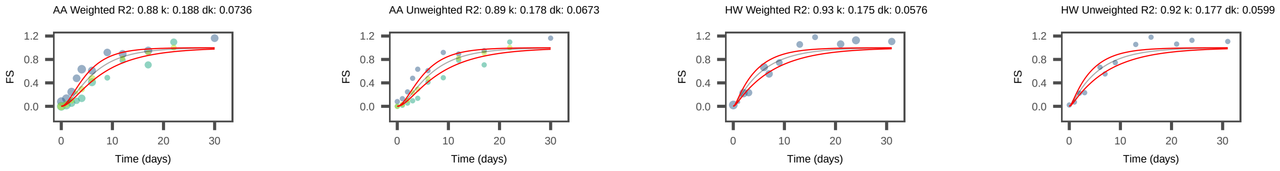

VATD

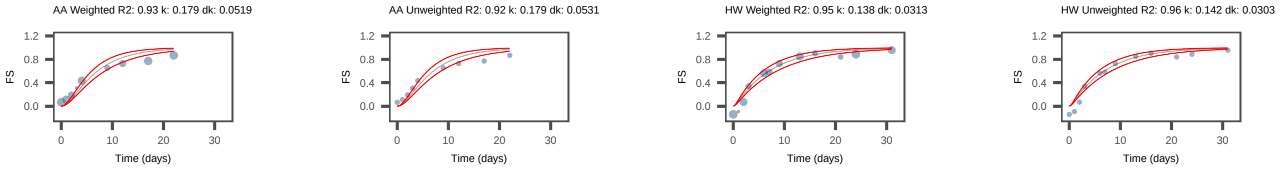

VATE1

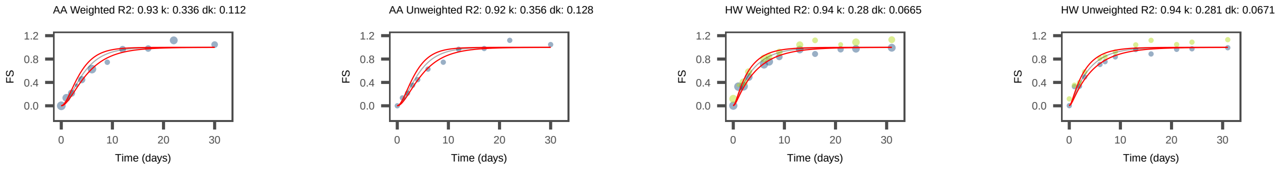

VATH

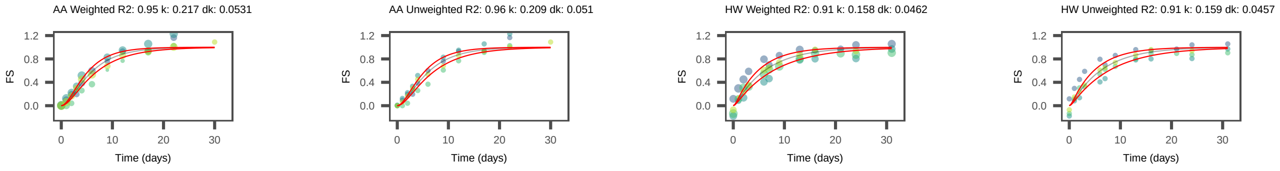

VDAC1

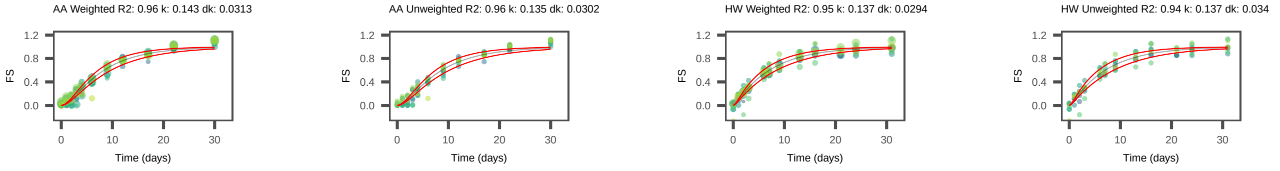

VDAC2

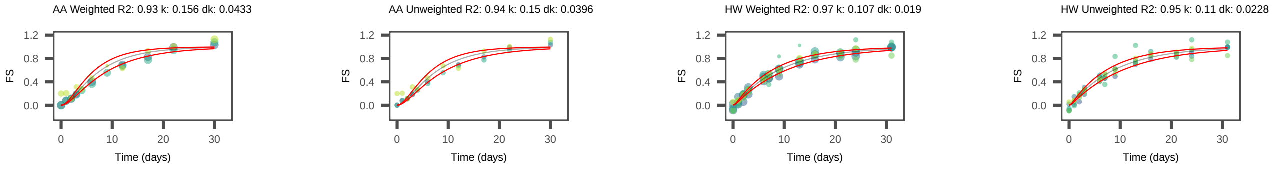

VDAC3

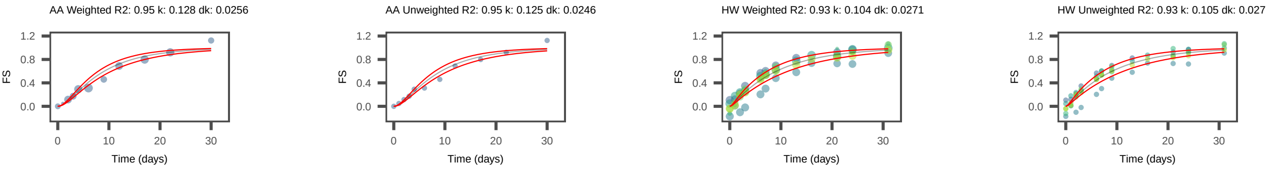

VILI

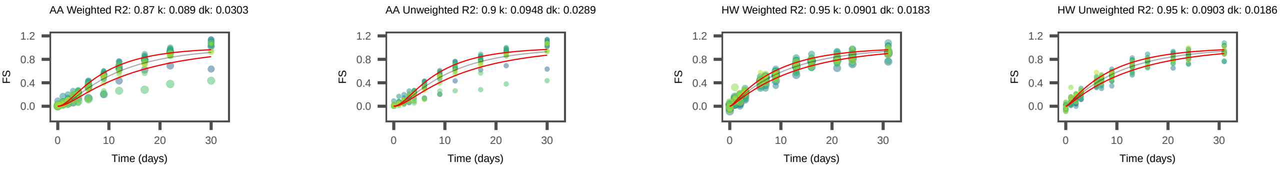

VINC

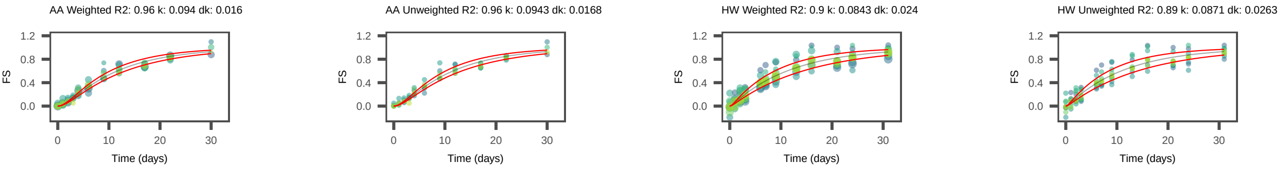

VPP4

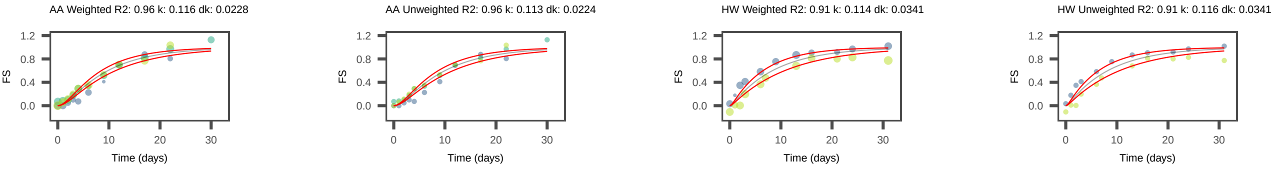

VWA8

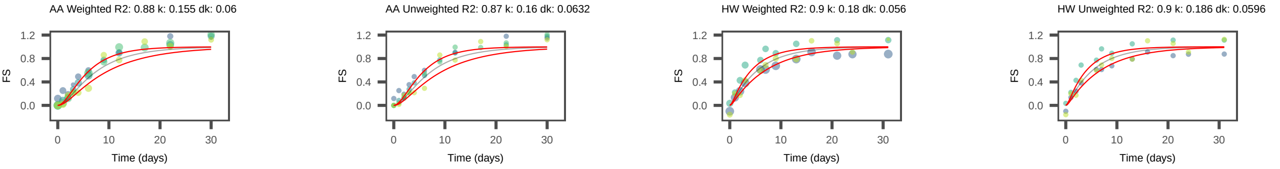

XPO1

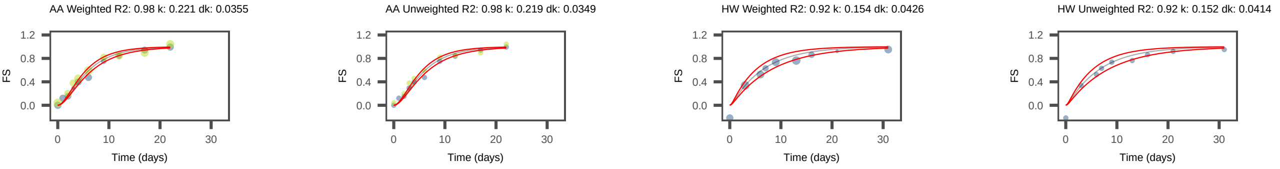

XYLB

AA Weighted R2: 0.96 k: 0.274 dk: 0.0683

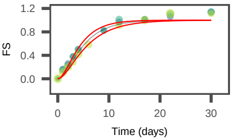

AA Unweighted R2: 0.96 k: 0.263 dk: 0.0683

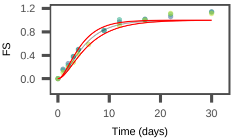

HW Weighted R2: 0.97 k: 0.218 dk: 0.0376

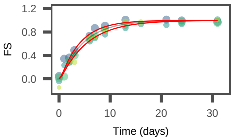

HW Unweighted R2: 0.97 k: 0.212 dk: 0.0374

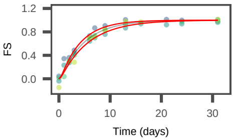

YBOX1

AA Weighted R2: 0.9 k: 0.732 dk: 0.376

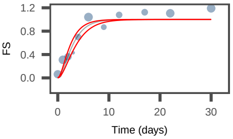

AA Unweighted R2: 0.9 k: 0.688 dk: 0.34

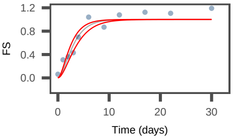

HW Weighted R2: 0.93 k: 0.442 dk: 0.0938

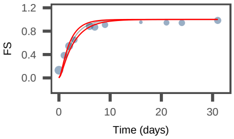

HW Unweighted R2: 0.93 k: 0.442 dk: 0.0931

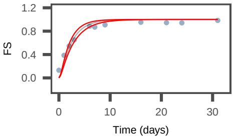

Supplement: Supplemental Data S7 [file mmc8.pdf]
