# Supplementary material for: Harmonizing Labeling and Analytical Strategies to Obtain Protein Turnover Rates in Intact Adult Animals
Source: Mol Cell Proteomics. 2022 May 28;21(7):100252. doi: 10.1016/j.mcpro.2022.100252 (PMC9249856; doi:10.1016/j.mcpro.2022.100252)

1433B

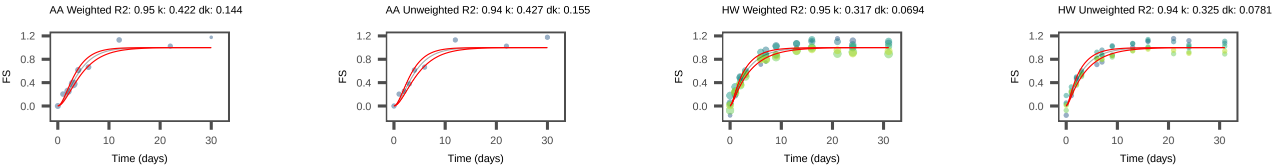

1433E

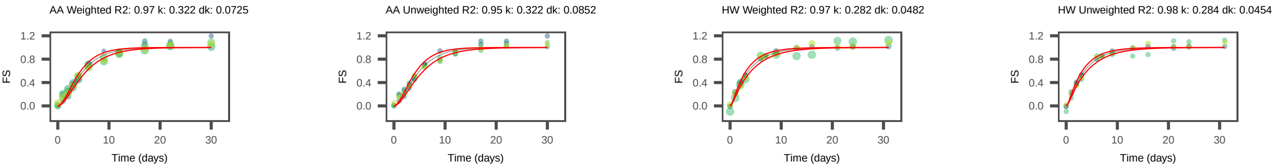

1433G

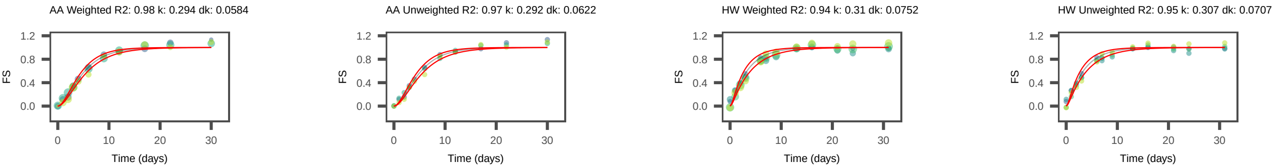

1433T

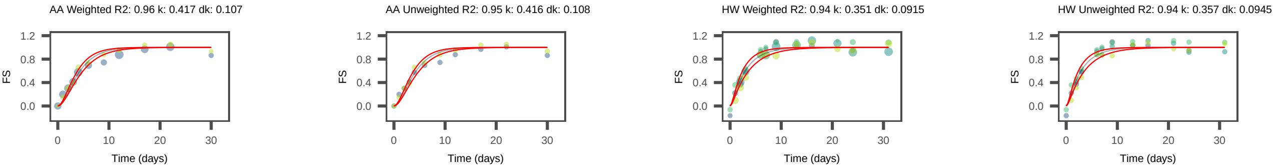

1433Z

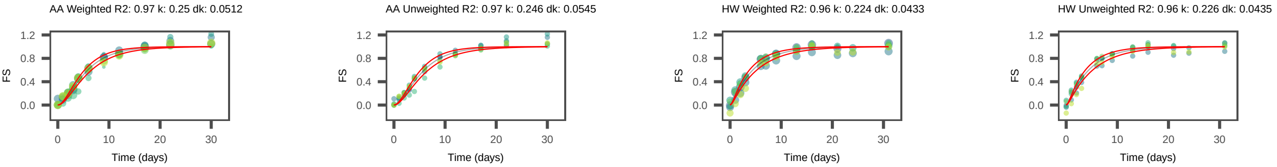

2AAA

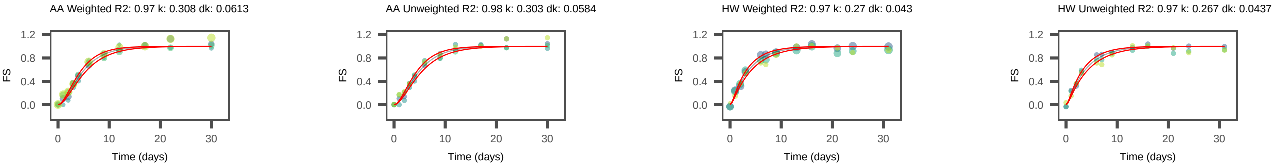

3HAO

AA Weighted R2: 0.97 k: 0.246 dk: 0.0499

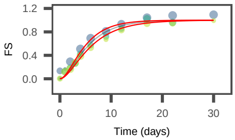

AA Unweighted R2: 0.97 k: 0.246 dk: 0.0486

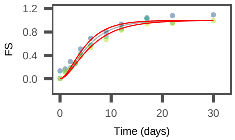

HW Weighted R2: 0.93 k: 0.274 dk: 0.0765

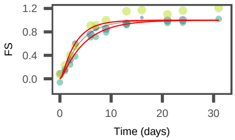

HW Unweighted R2: 0.94 k: 0.262 dk: 0.0704

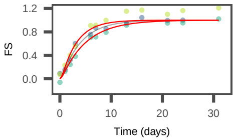

3HIDH

AA Weighted R2: 0.95 k: 0.141 dk: 0.036

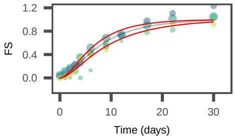

AA Unweighted R2: 0.93 k: 0.13 dk: 0.0368

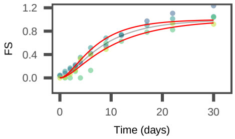

HW Weighted R2: 0.92 k: 0.102 dk: 0.0265

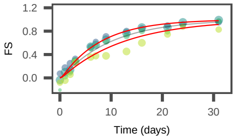

HW Unweighted R2: 0.92 k: 0.102 dk: 0.0268

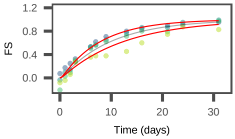

6PGD

AA Weighted R2: 0.94 k: 0.273 dk: 0.0701

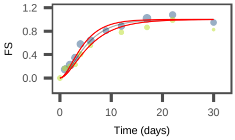

AA Unweighted R2: 0.94 k: 0.263 dk: 0.0688

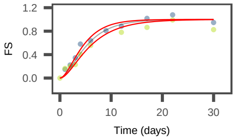

HW Weighted R2: 0.9 k: 0.186 dk: 0.0616

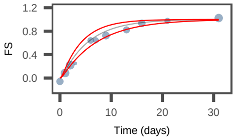

HW Unweighted R2: 0.88 k: 0.2 dk: 0.0728

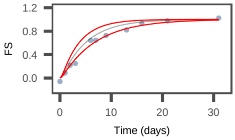

6PGL

AA Weighted R2: 0.96 k: 0.17 dk: 0.0366

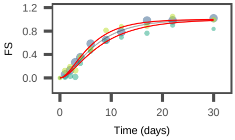

AA Unweighted R2: 0.96 k: 0.165 dk: 0.0374

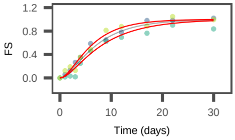

HW Weighted R2: 0.97 k: 0.181 dk: 0.0276

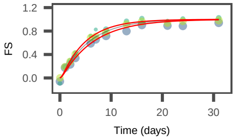

HW Unweighted R2: 0.97 k: 0.188 dk: 0.0291

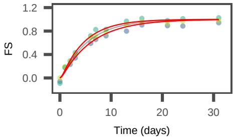

AAAD

AA Weighted R2: 0.97 k: 0.367 dk: 0.0765

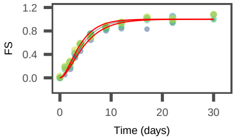

AA Unweighted R2: 0.97 k: 0.362 dk: 0.0761

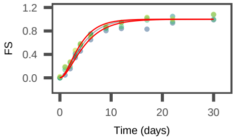

HW Weighted R2: 0.93 k: 0.366 dk: 0.0993

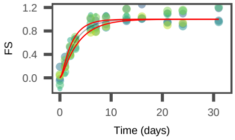

HW Unweighted R2: 0.93 k: 0.374 dk: 0.105

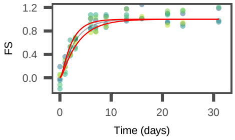

AADAT

AA Weighted R2: 0.97 k: 0.131 dk: 0.0236

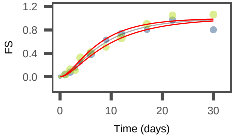

AA Unweighted R2: 0.97 k: 0.13 dk: 0.0235

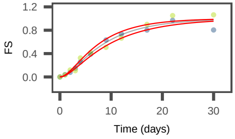

HW Weighted R2: 0.89 k: 0.0944 dk: 0.0262

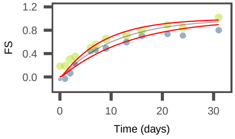

HW Unweighted R2: 0.89 k: 0.0924 dk: 0.0254

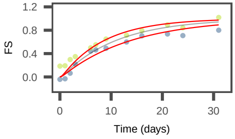

AAKG1

AA Weighted R2: 0.95 k: 0.149 dk: 0.0393 | AA Unweighted R2: 0.95 k: 0.148 dk: 0.0388 | HW Weighted R2: 0.97 k: 0.266 dk: 0.0496 | HW Unweighted R2: 0.97 k: 0.268 dk: 0.0494

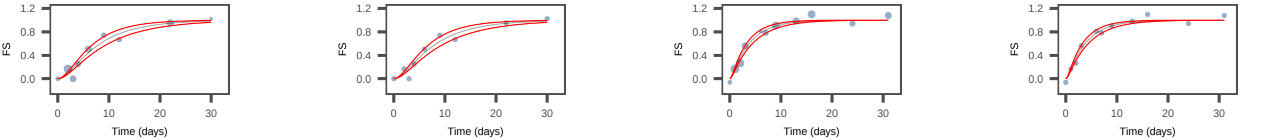

AASS

AA Weighted R2: 0.94 k: 0.202 dk: 0.0586 | AA Unweighted R2: 0.93 k: 0.195 dk: 0.0612 | HW Weighted R2: 0.92 k: 0.129 dk: 0.0323 | HW Unweighted R2: 0.92 k: 0.123 dk: 0.0323

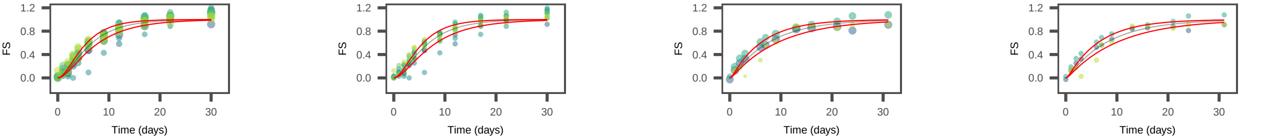

AATC

AA Weighted R2: 0.92 k: 0.168 dk: 0.0556 | AA Unweighted R2: 0.89 k: 0.155 dk: 0.0575 | HW Weighted R2: 0.93 k: 0.166 dk: 0.0443 | HW Unweighted R2: 0.93 k: 0.158 dk: 0.0439

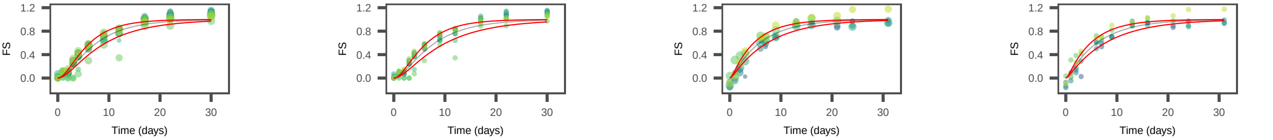

AATM

AA Weighted R2: 0.89 k: 0.13 dk: 0.048 | AA Unweighted R2: 0.89 k: 0.128 dk: 0.0461 | HW Weighted R2: 0.95 k: 0.119 dk: 0.024 | HW Unweighted R2: 0.91 k: 0.12 dk: 0.0335

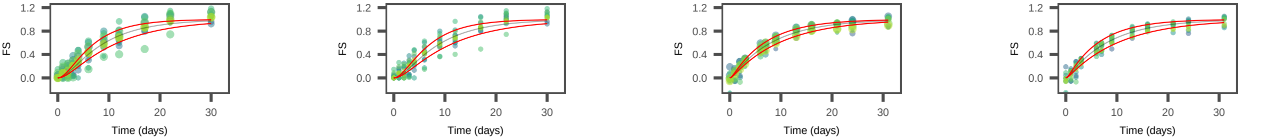

ABCBB

AA Weighted R2: 0.92 k: 0.171 dk: 0.0645 | AA Unweighted R2: 0.91 k: 0.164 dk: 0.0686 | HW Weighted R2: 0.95 k: 0.348 dk: 0.0931 | HW Unweighted R2: 0.95 k: 0.344 dk: 0.093

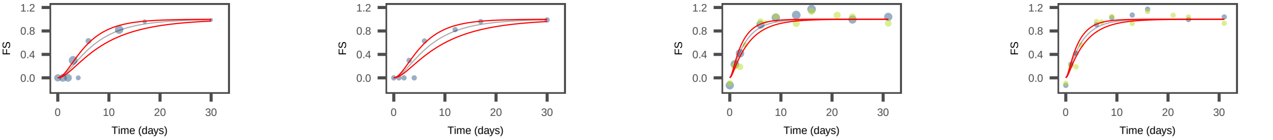

ABCD3

AA Weighted R2: 0.9 k: 0.312 dk: 0.123 | AA Unweighted R2: 0.92 k: 0.335 dk: 0.126 | HW Weighted R2: 0.96 k: 0.302 dk: 0.06 | HW Unweighted R2: 0.96 k: 0.307 dk: 0.0625

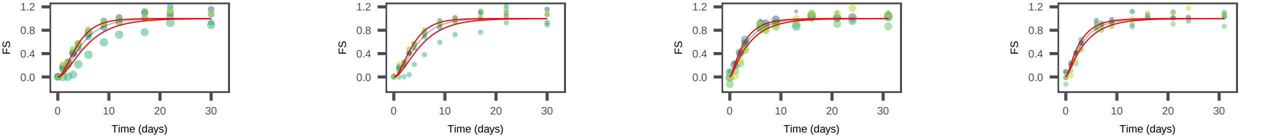

ABHEB

AA Weighted R2: 0.96 k: 0.23 dk: 0.0526

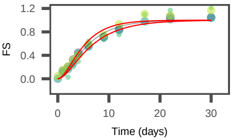

AA Unweighted R2: 0.95 k: 0.224 dk: 0.0574

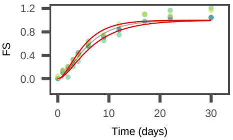

HW Weighted R2: 0.99 k: 0.187 dk: 0.0152

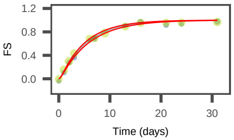

HW Unweighted R2: 0.99 k: 0.186 dk: 0.0152

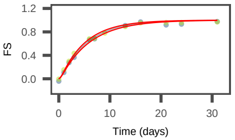

ACACA

AA Weighted R2: 0.89 k: 0.29 dk: 0.109

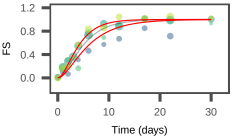

AA Unweighted R2: 0.88 k: 0.272 dk: 0.104

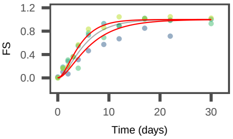

HW Weighted R2: 0.88 k: 0.261 dk: 0.105

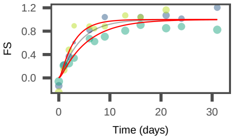

HW Unweighted R2: 0.87 k: 0.28 dk: 0.112

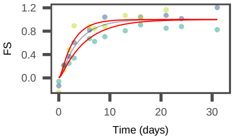

ACAD8

AA Weighted R2: 0.95 k: 0.117 dk: 0.0276

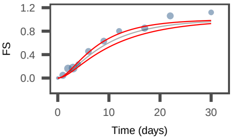

AA Unweighted R2: 0.95 k: 0.124 dk: 0.0297

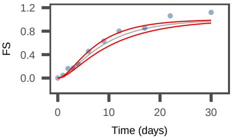

HW Weighted R2: 0.81 k: 0.171 dk: 0.0704

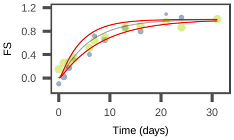

HW Unweighted R2: 0.76 k: 0.188 dk: 0.0857

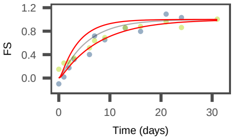

ACADL

AA Weighted R2: 0.92 k: 0.16 dk: 0.0479

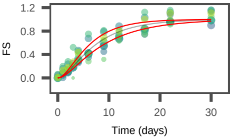

AA Unweighted R2: 0.91 k: 0.158 dk: 0.0516

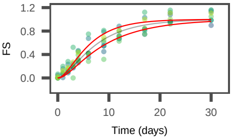

HW Weighted R2: 0.92 k: 0.143 dk: 0.0363

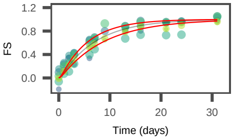

HW Unweighted R2: 0.93 k: 0.14 dk: 0.0341

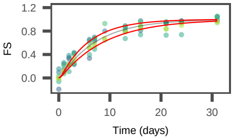

ACADM

AA Weighted R2: 0.94 k: 0.242 dk: 0.0672

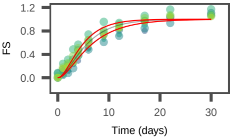

AA Unweighted R2: 0.94 k: 0.234 dk: 0.0639

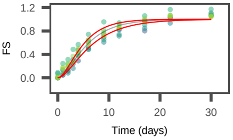

HW Weighted R2: 0.96 k: 0.184 dk: 0.0324

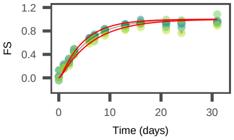

HW Unweighted R2: 0.96 k: 0.183 dk: 0.0327

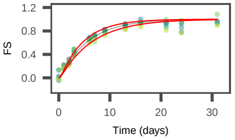

ACADS

AA Weighted R2: 0.97 k: 0.144 dk: 0.0244

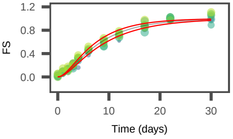

AA Unweighted R2: 0.97 k: 0.14 dk: 0.0255

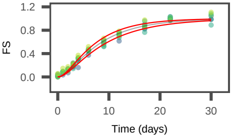

HW Weighted R2: 0.97 k: 0.132 dk: 0.0211

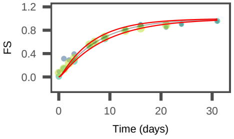

HW Unweighted R2: 0.96 k: 0.132 dk: 0.0218

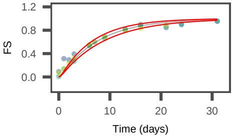

ACADV

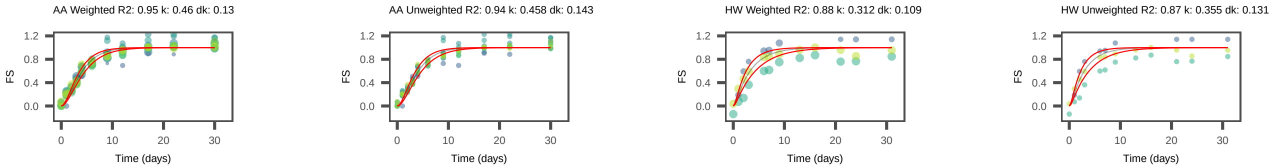

ACBP

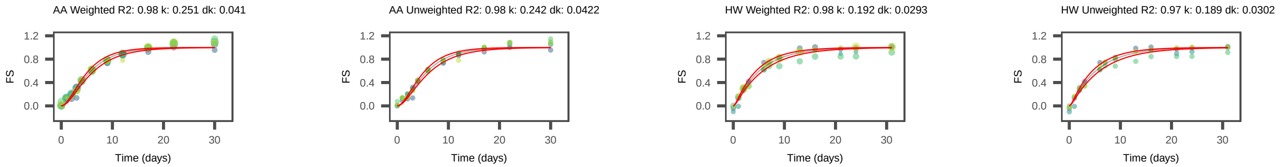

ACD10

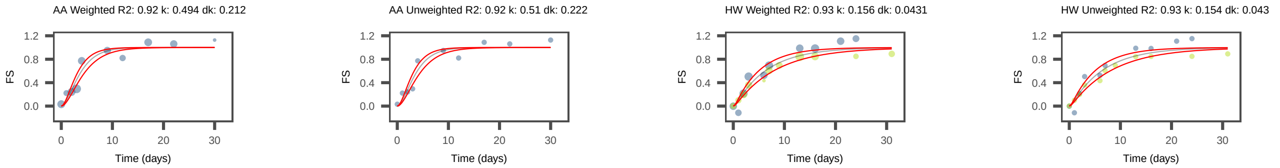

ACD11

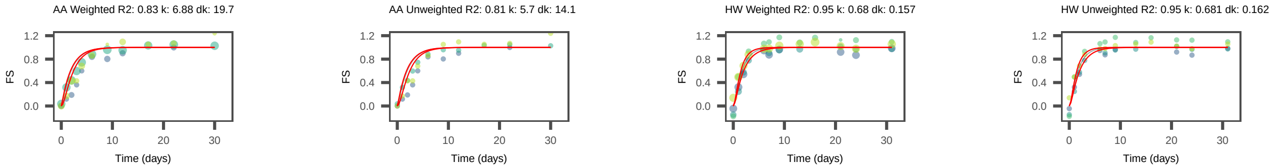

ACDSB

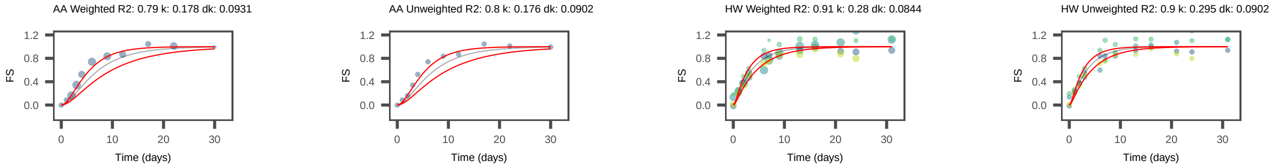

ACLY

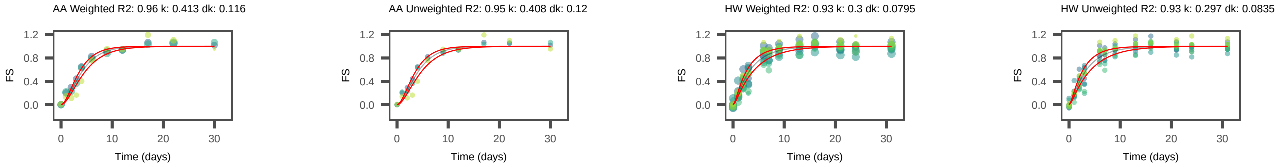

ACO12

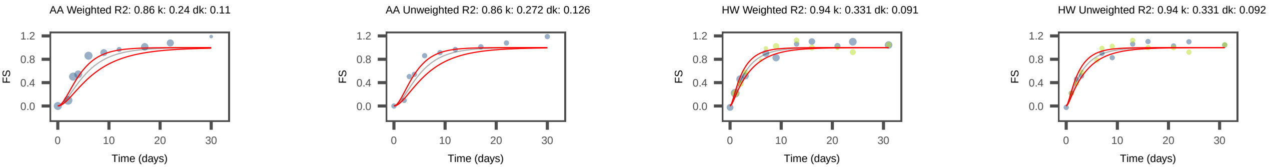

ACO13

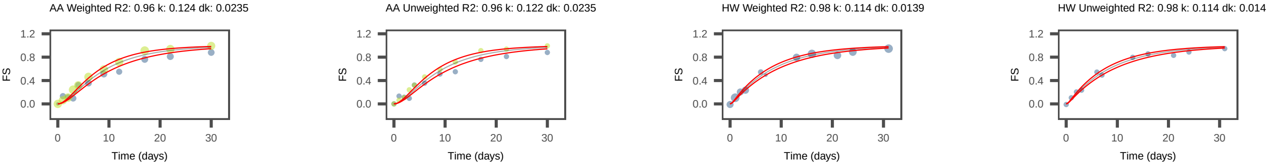

ACOC

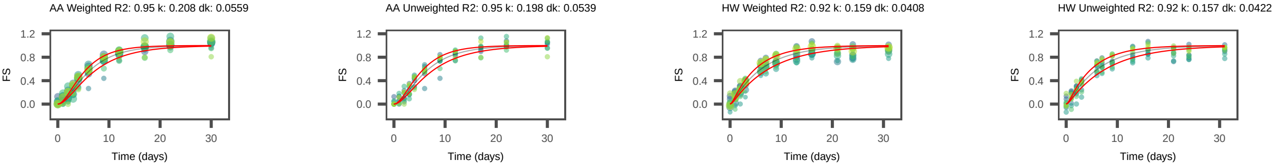

ACON

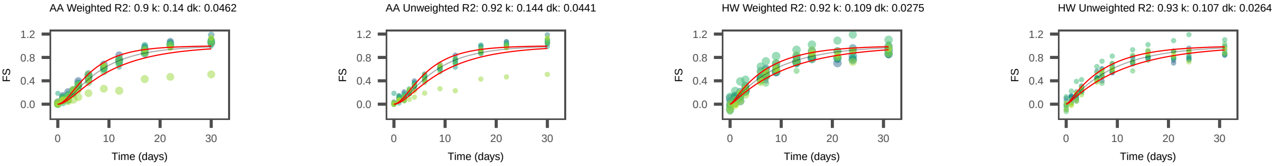

ACOX1

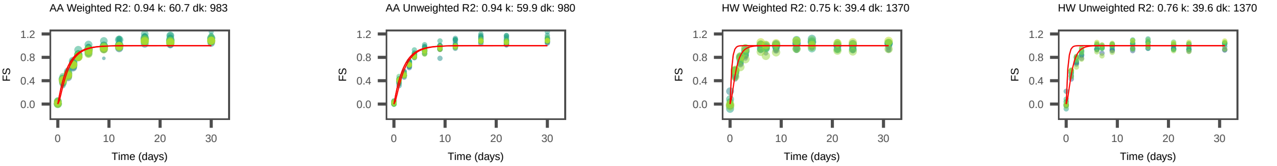

ACOX2

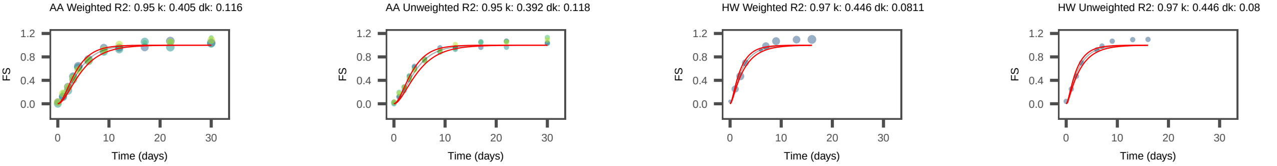

ACPM

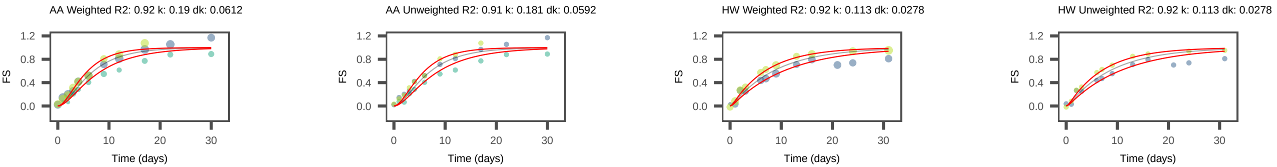

ACSA

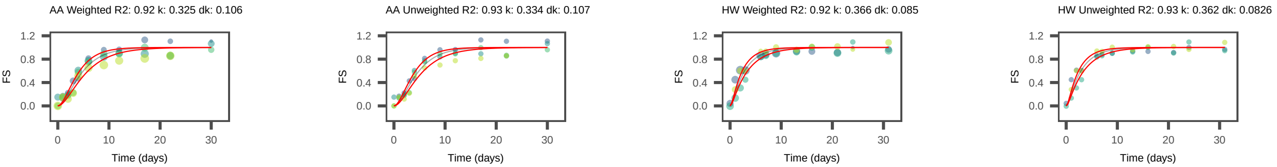

ACSF2

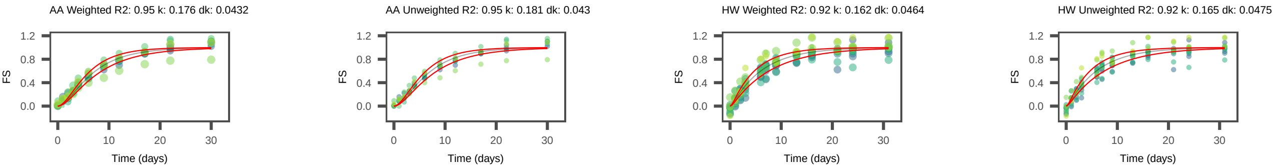

ACSL1

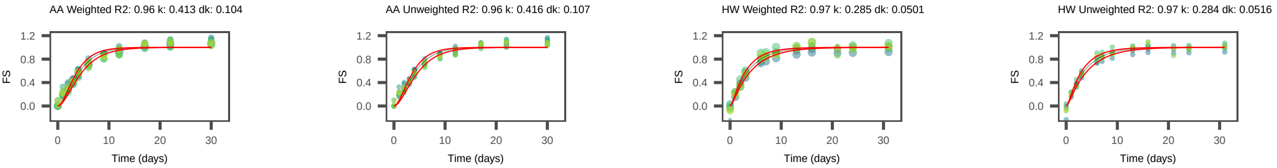

ACSL5

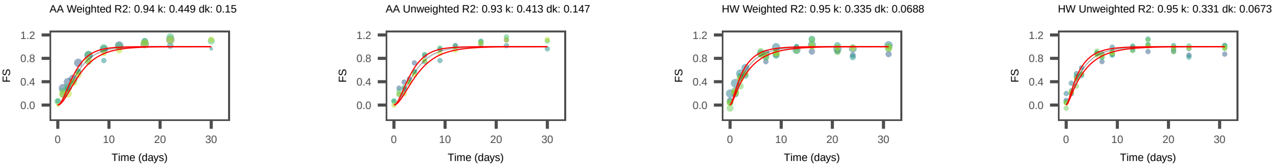

ACSM1

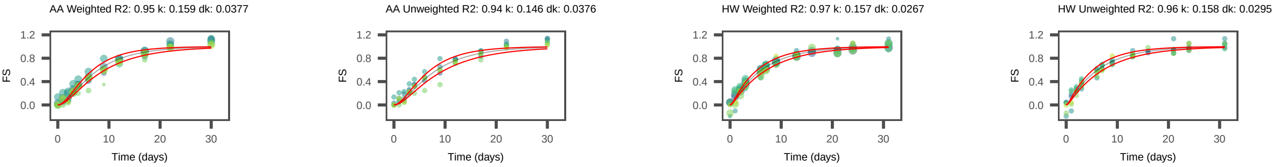

ACSM3

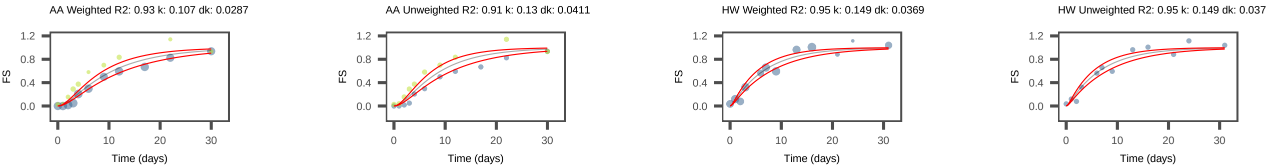

ACSM5

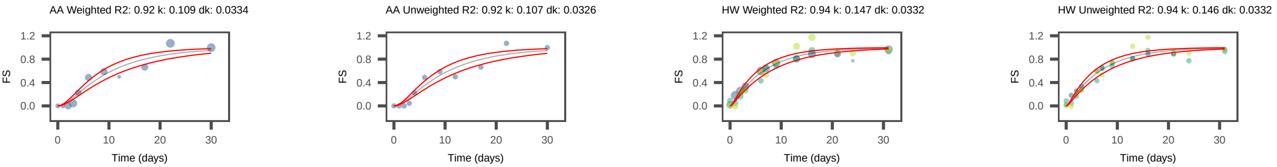

ACSS3

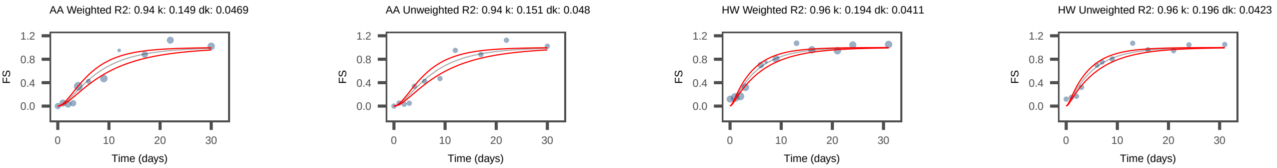

ACTN1

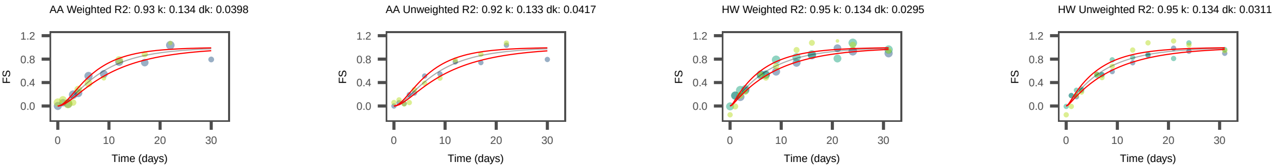

ACTN4

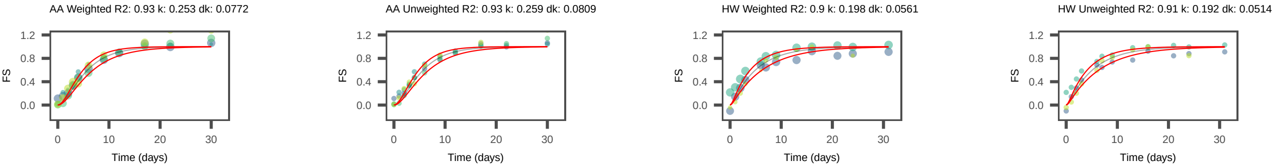

ACY1

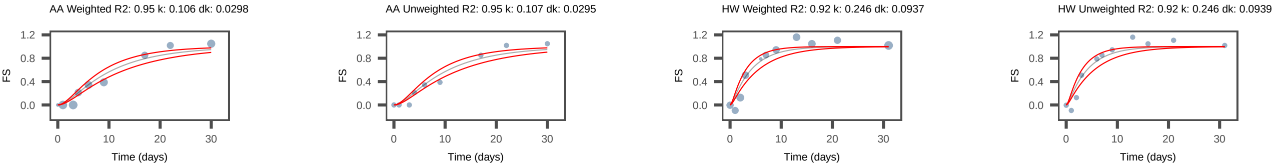

ACY3

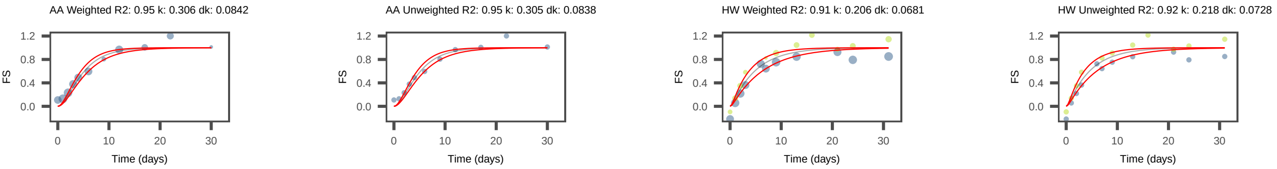

ADH1

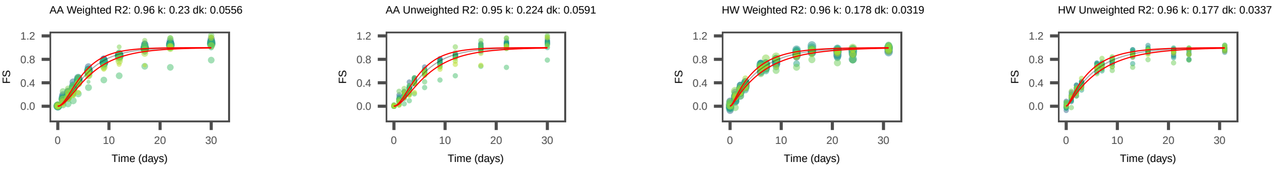

ADHX

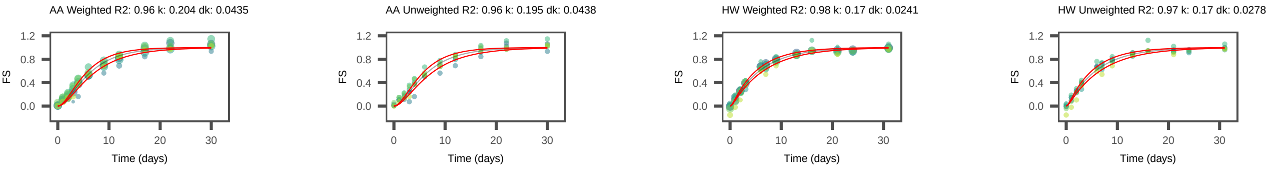

ADK

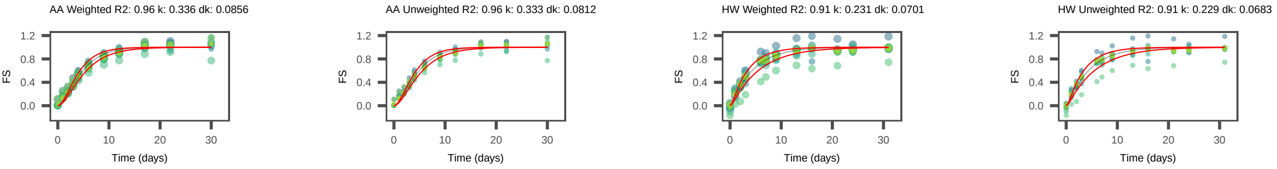

ADT2

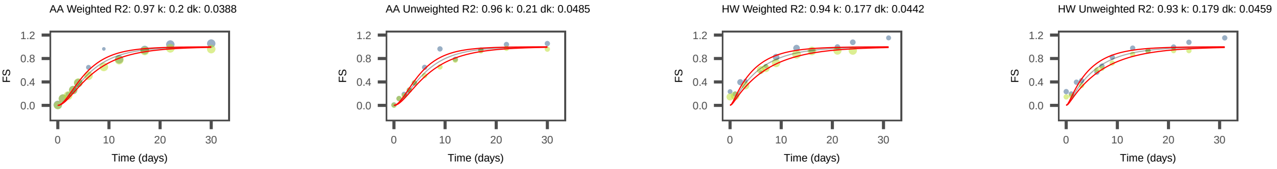

AGT2

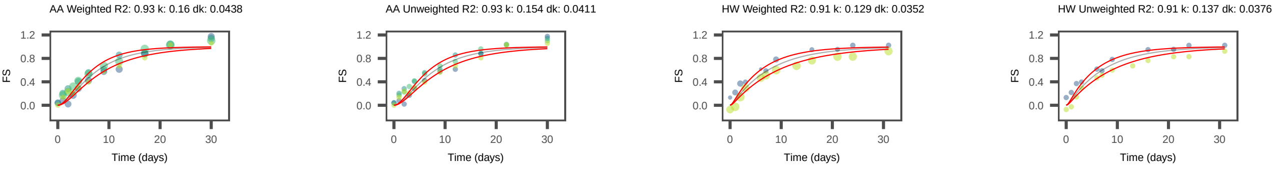

AIFM1

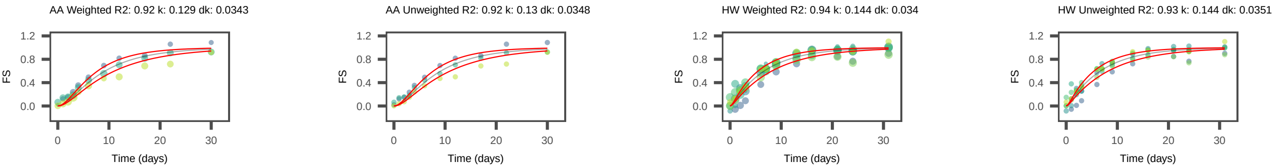

AIMP2

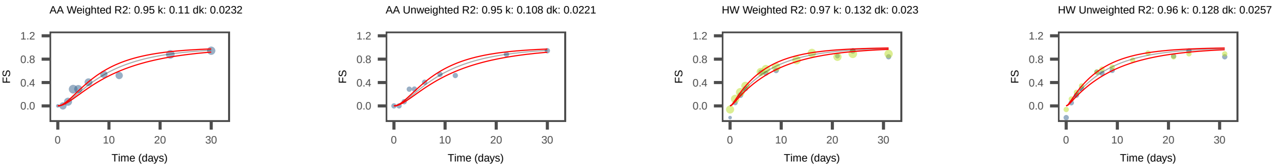

AK1A1

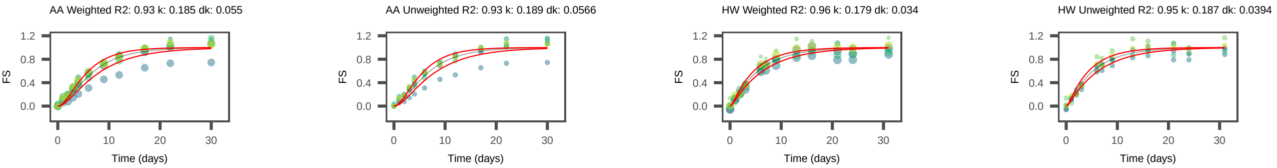

AK1CD

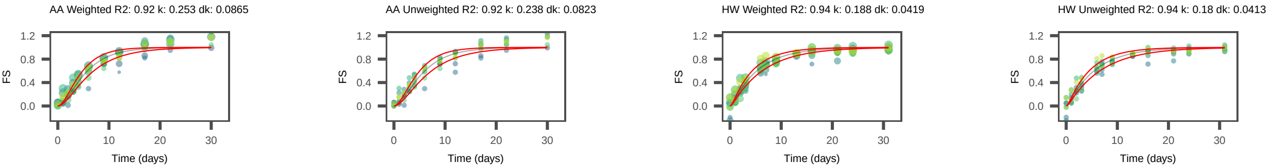

AK1D1

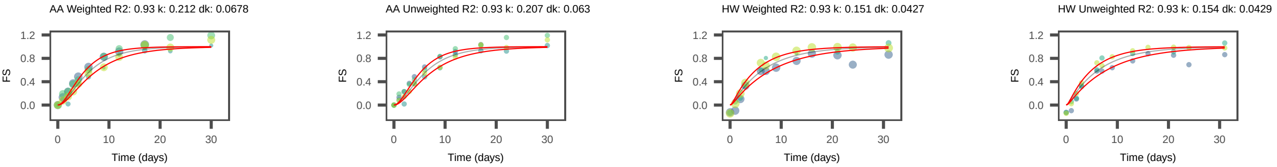

AL1A1

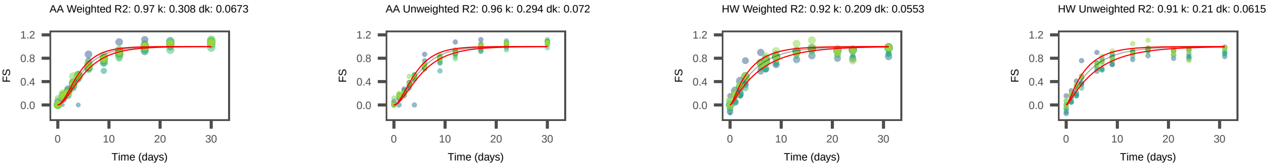

AL1A7

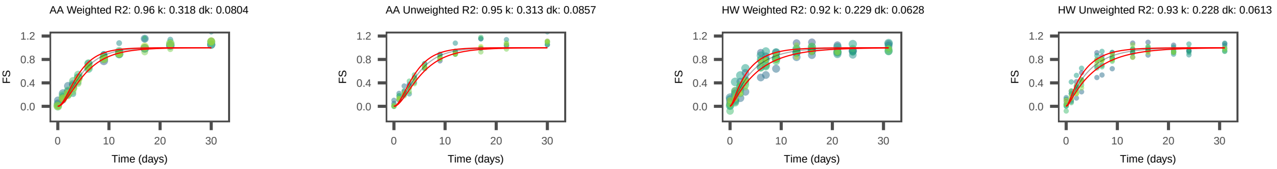

AL1B1

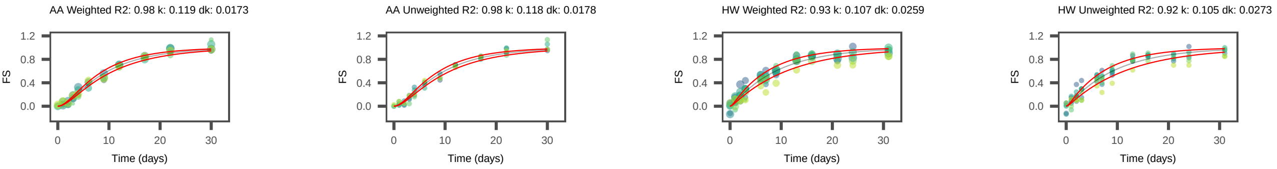

AL1L1

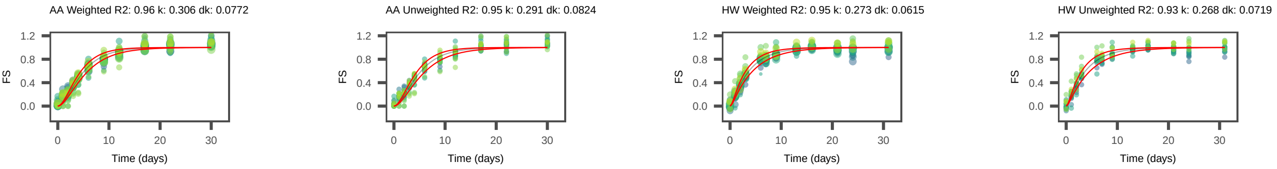

AL3A2

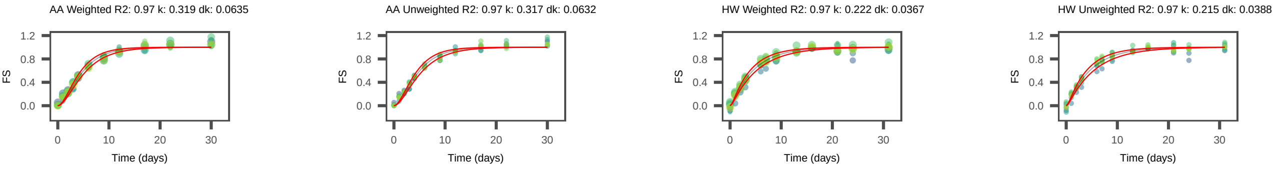

AL4A1

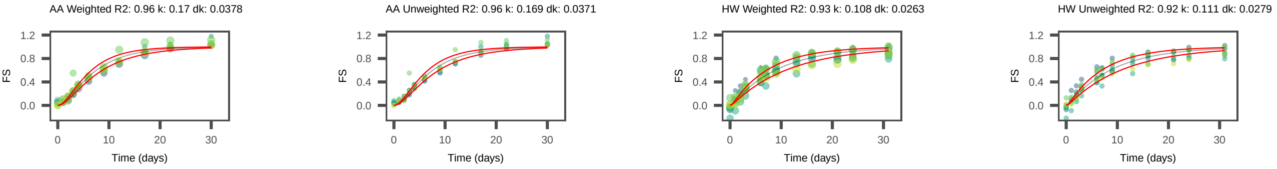

AL7A1

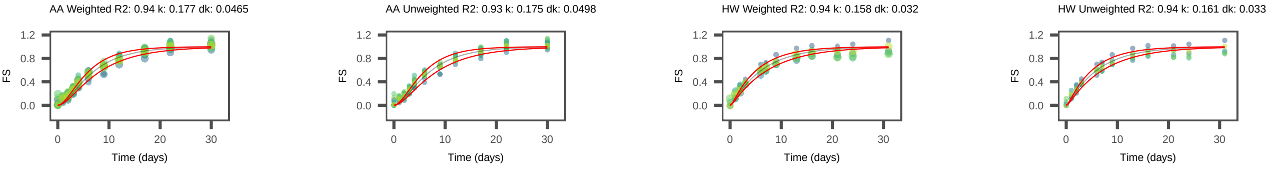

AL8A1

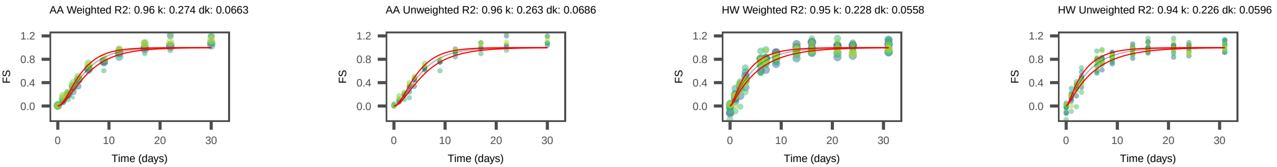

AL9A1

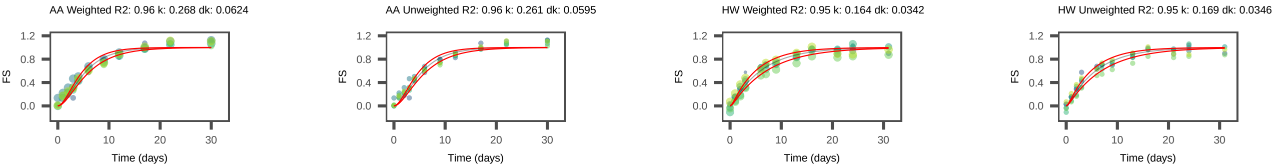

ALAT1

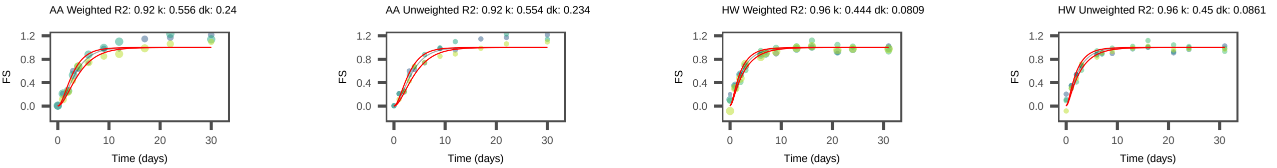

ALAT2

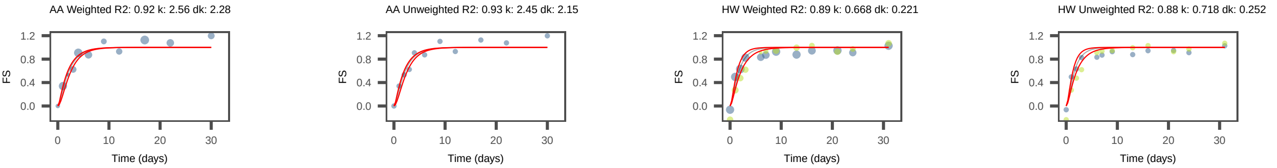

ALBU

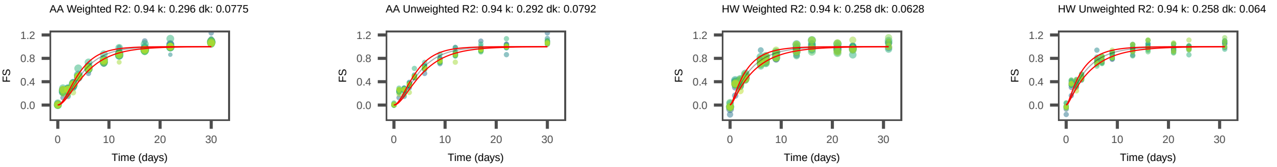

ALDH2

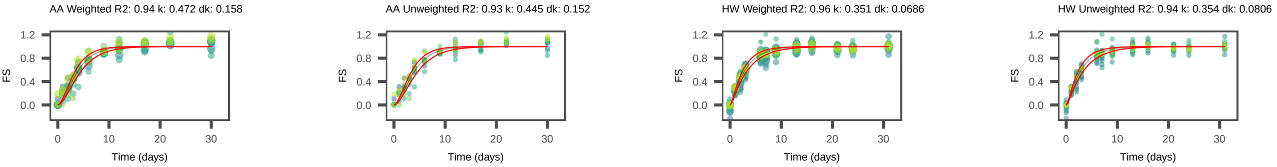

ALDOB

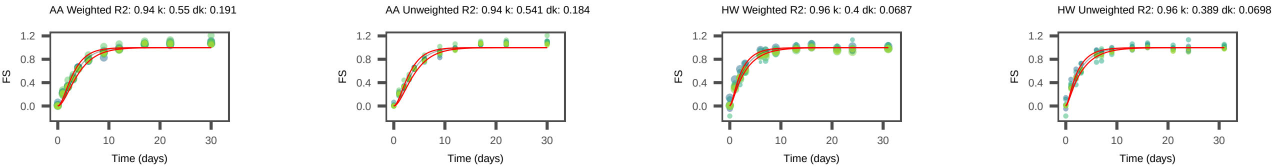

AMPL

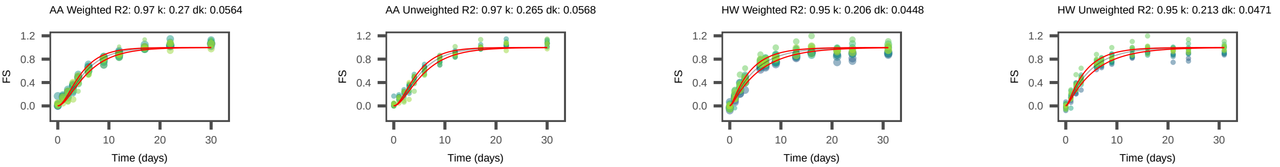

AMPN

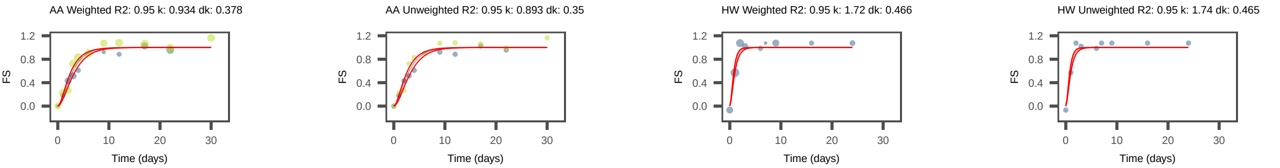

ANT3

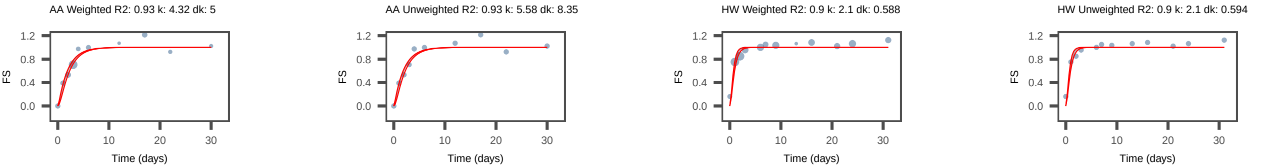

ANXA2

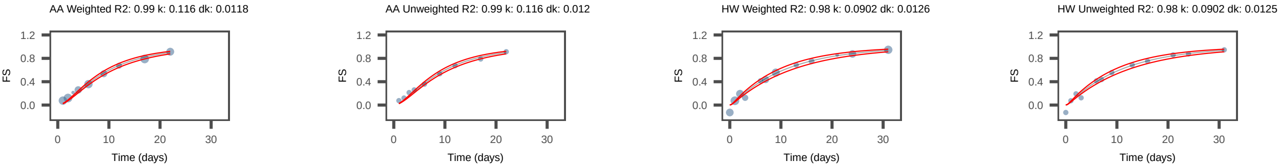

ANXA6

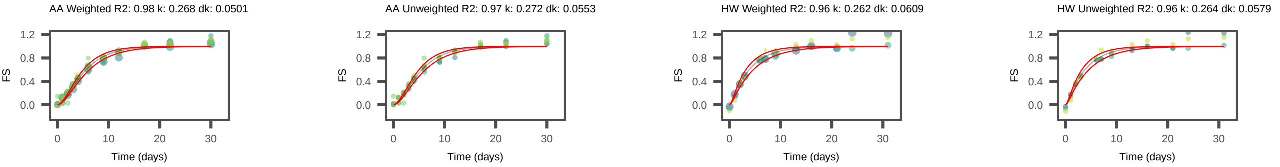

AOFB

AA Weighted R2: 0.96 k: 0.227 dk: 0.0513 | AA Unweighted R2: 0.96 k: 0.227 dk: 0.0519 | HW Weighted R2: 0.95 k: 0.168 dk: 0.0346 | HW Unweighted R2: 0.95 k: 0.171 dk: 0.0371

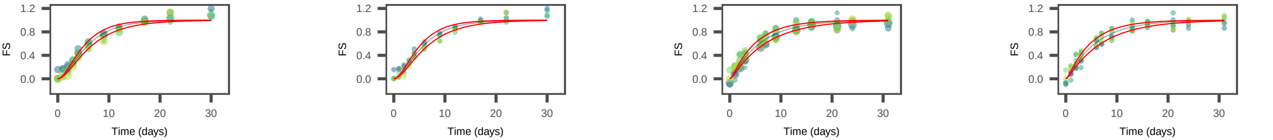

AOXC

AA Weighted R2: 0.95 k: 0.441 dk: 0.126 | AA Unweighted R2: 0.94 k: 0.424 dk: 0.132 | HW Weighted R2: 0.95 k: 0.371 dk: 0.0882 | HW Unweighted R2: 0.94 k: 0.374 dk: 0.0939

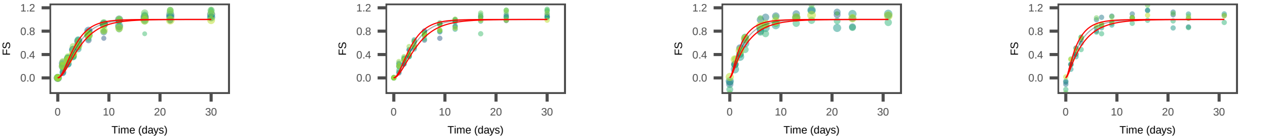

AP1B1

AA Weighted R2: 0.91 k: 0.133 dk: 0.0414 | AA Unweighted R2: 0.91 k: 0.124 dk: 0.0389 | HW Weighted R2: 0.99 k: 0.15 dk: 0.0164 | HW Unweighted R2: 0.98 k: 0.155 dk: 0.0197

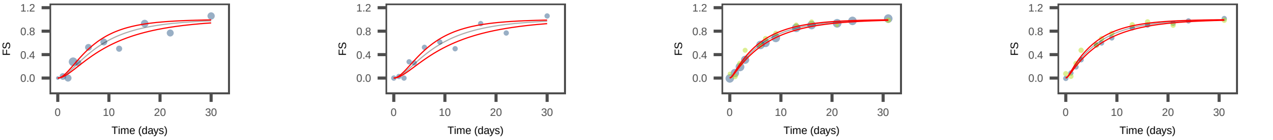

AP2B1

AA Weighted R2: 0.81 k: 0.128 dk: 0.0557 | AA Unweighted R2: 0.81 k: 0.12 dk: 0.0518 | HW Weighted R2: 0.94 k: 0.144 dk: 0.0318 | HW Unweighted R2: 0.93 k: 0.133 dk: 0.0334

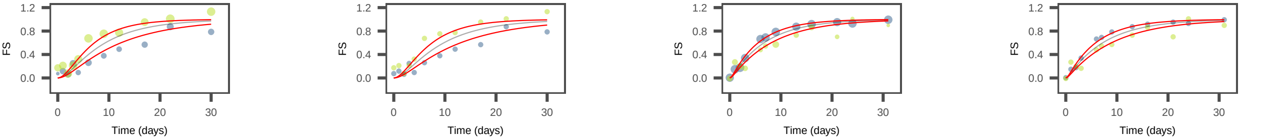

APEH

AA Weighted R2: 0.97 k: 0.25 dk: 0.0455 | AA Unweighted R2: 0.97 k: 0.252 dk: 0.0482 | HW Weighted R2: 0.86 k: 0.78 dk: 0.279 | HW Unweighted R2: 0.85 k: 0.734 dk: 0.261

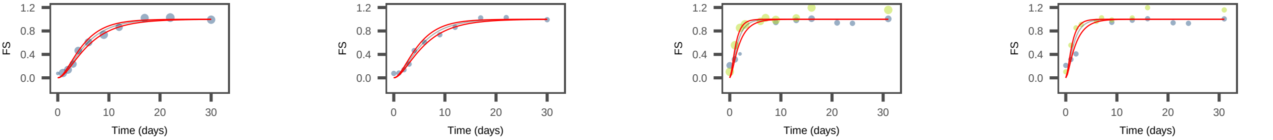

APOA1

AA Weighted R2: 0.9 k: 5.87 dk: 9 | AA Unweighted R2: 0.91 k: 5.91 dk: 8.8 | HW Weighted R2: 0.93 k: 1.66 dk: 0.406 | HW Unweighted R2: 0.93 k: 1.76 dk: 0.432

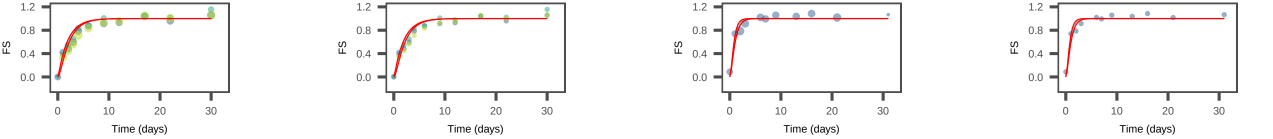

APOB

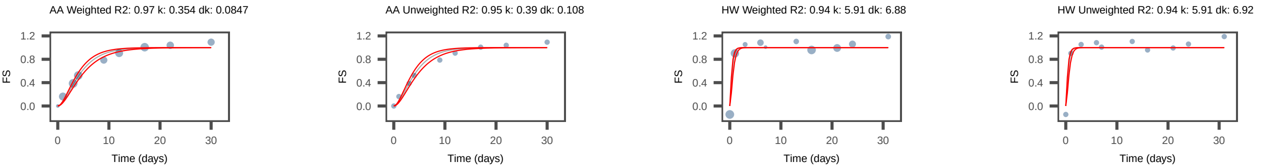

ARF6

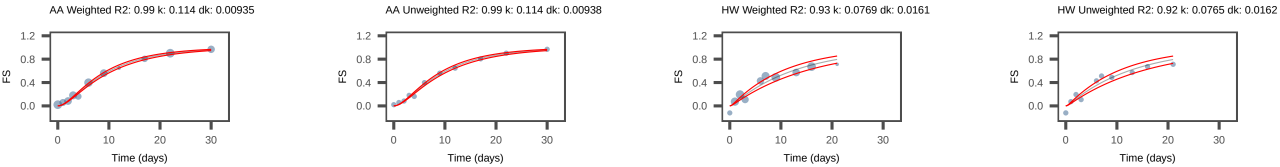

ARGI1

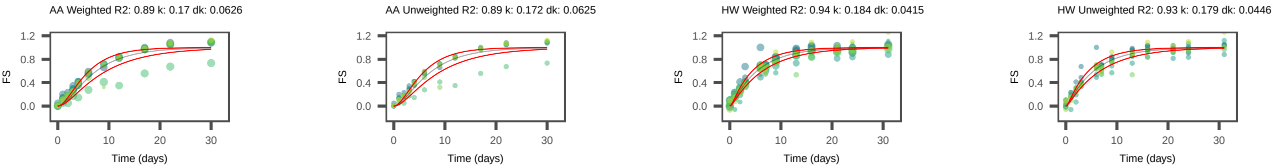

ARK72

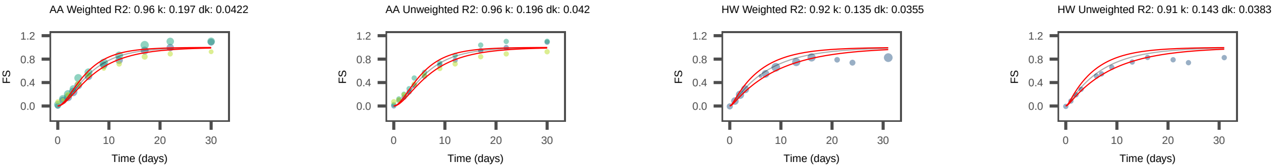

ARLY

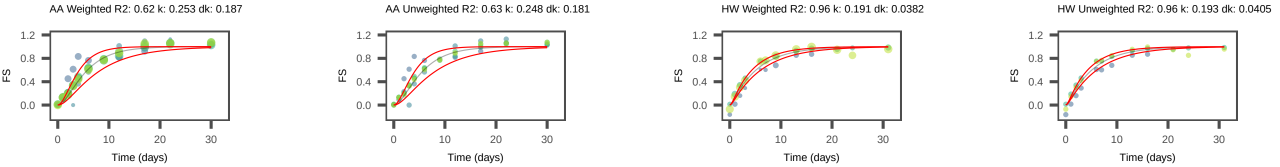

ARP3

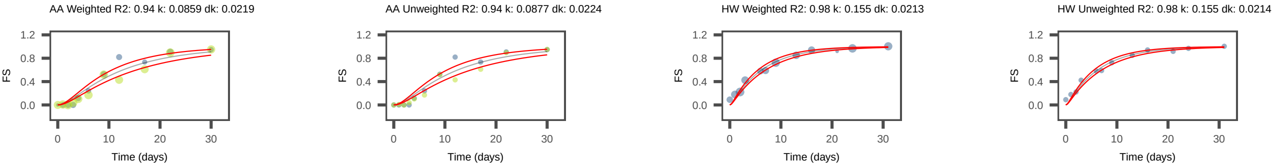

ARPC4

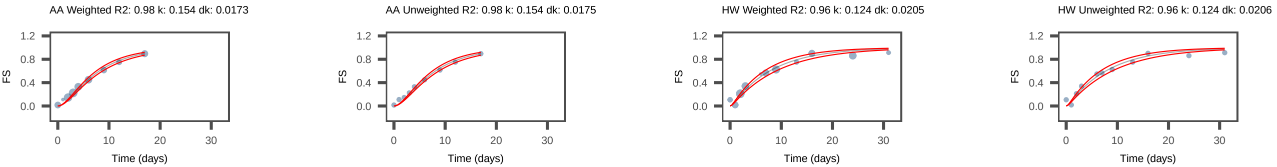

ARPC5

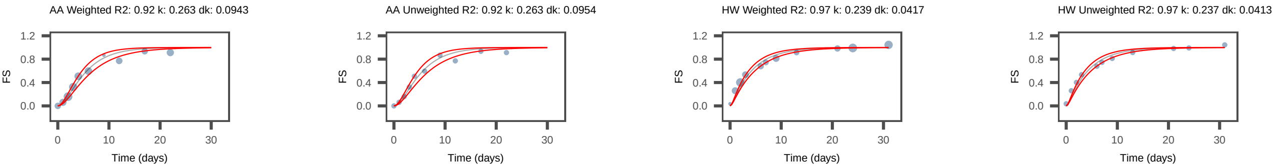

ASPD

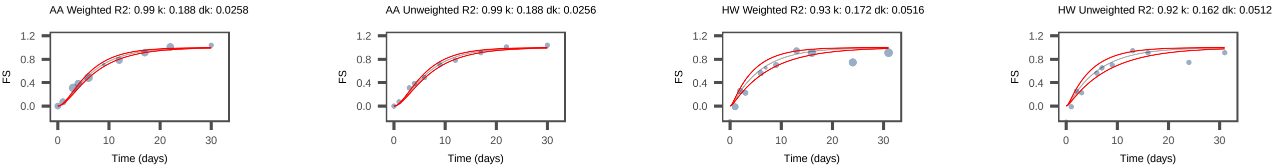

ASSY

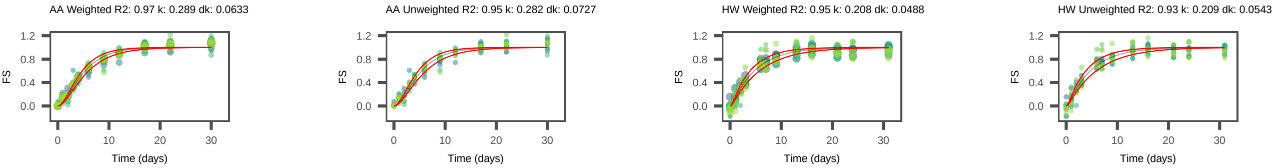

AT11C

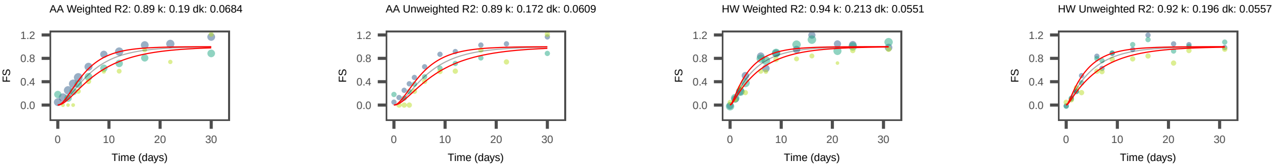

AT1A1

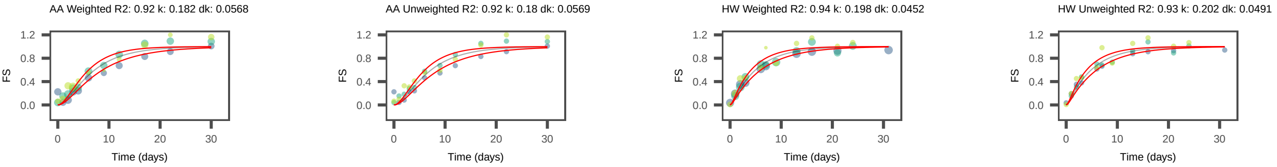

AT1B1

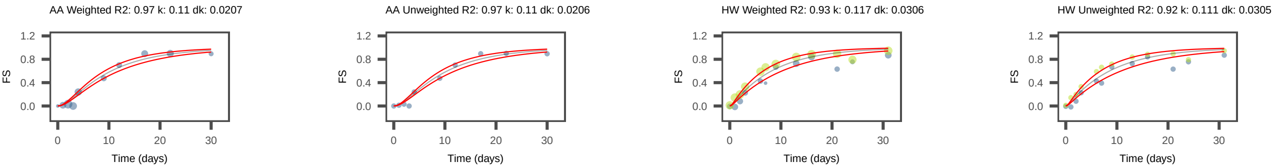

AT5F1

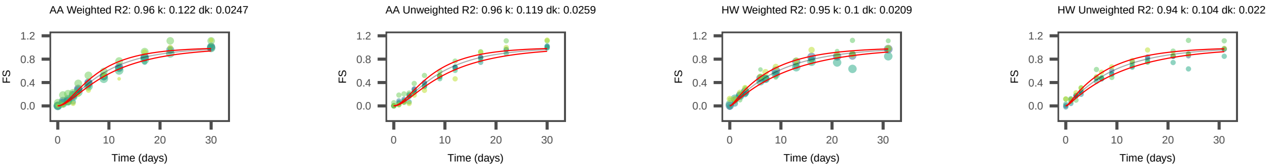

ATLA2

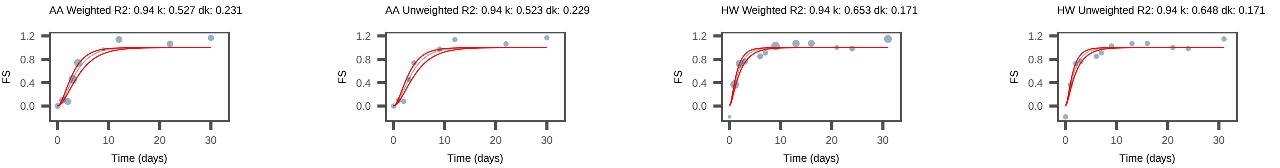

ATP5H

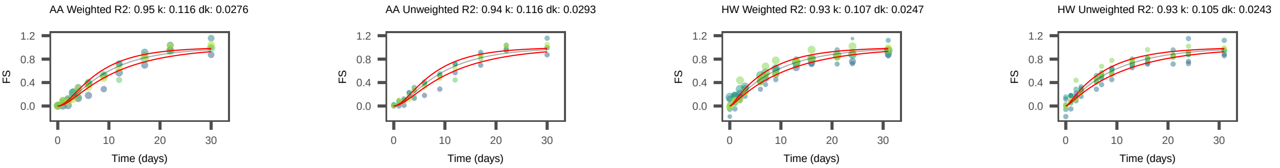

ATP5I

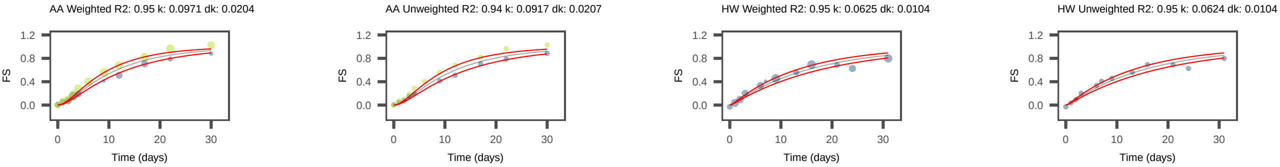

ATP5J

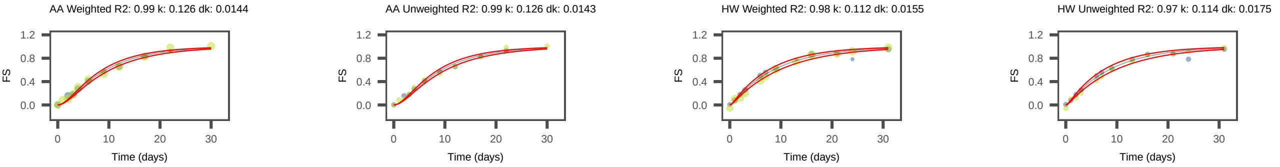

ATP5L

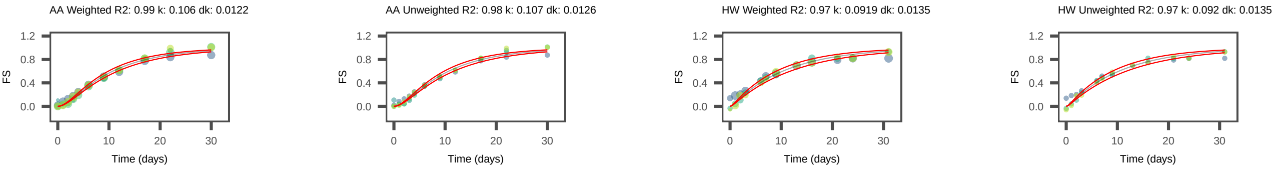

ATPA

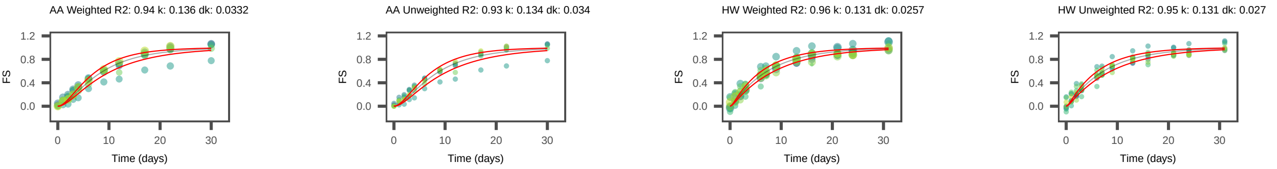

ATPB

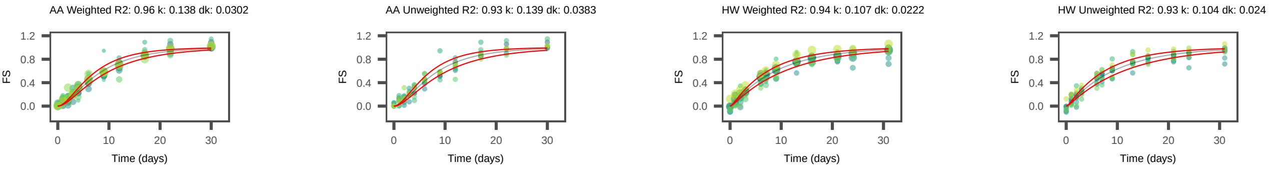

ATPD

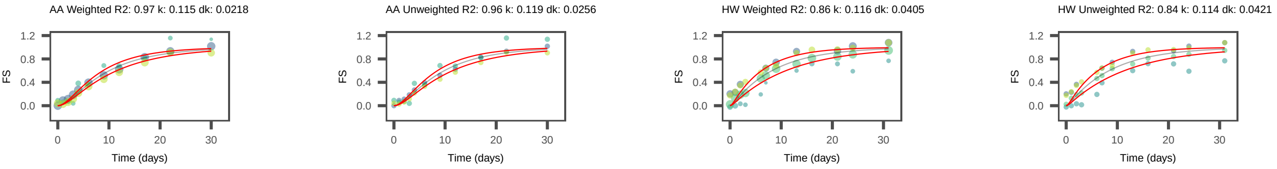

ATPG

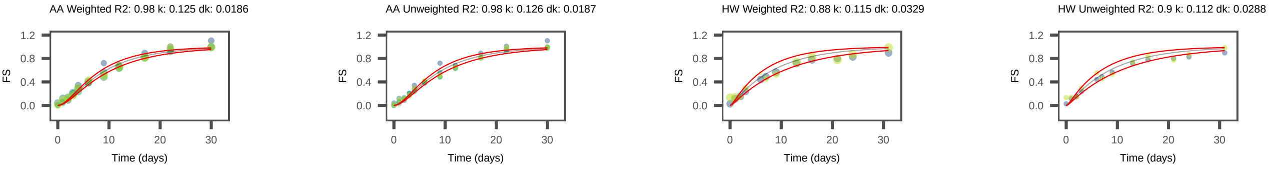

ATPO

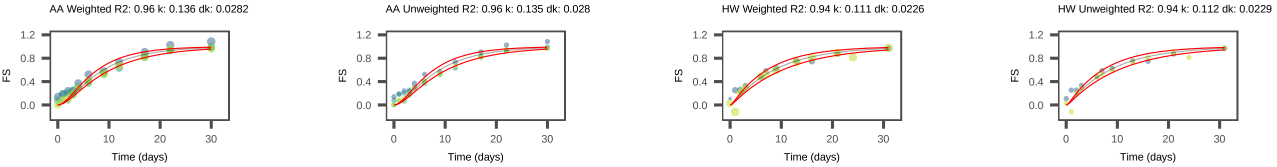

B2MG

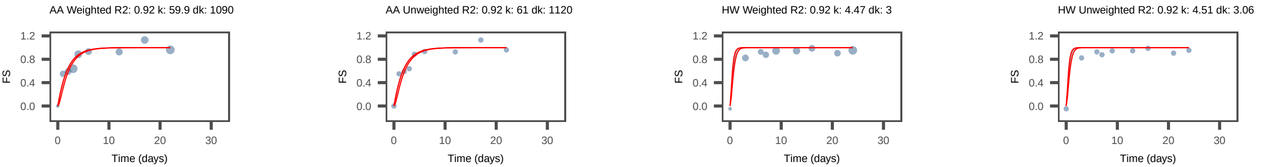

BAAT

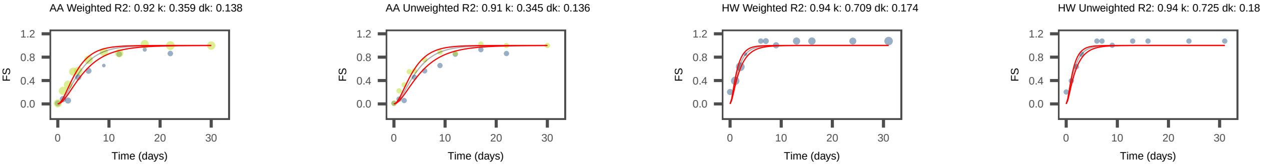

BAP31

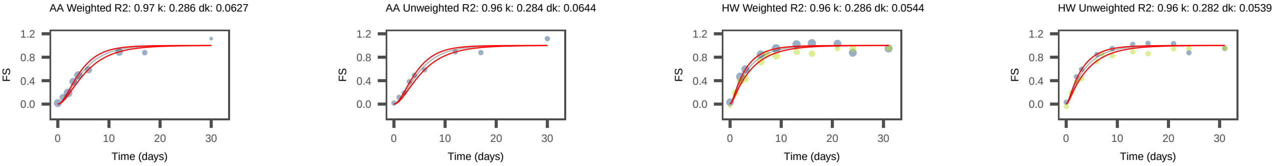

BDH

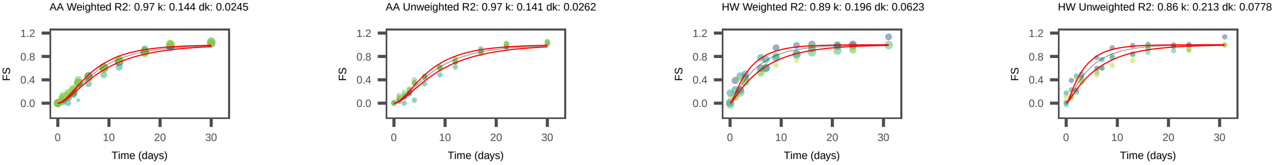

BGLR

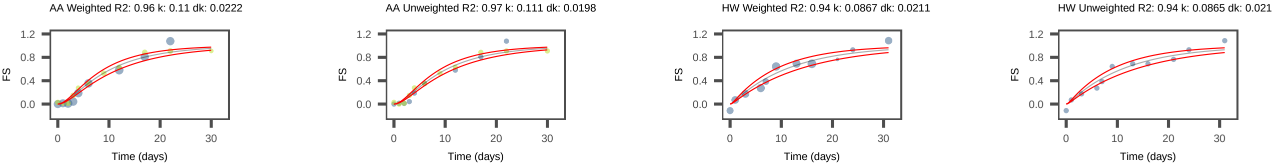

BHMT1

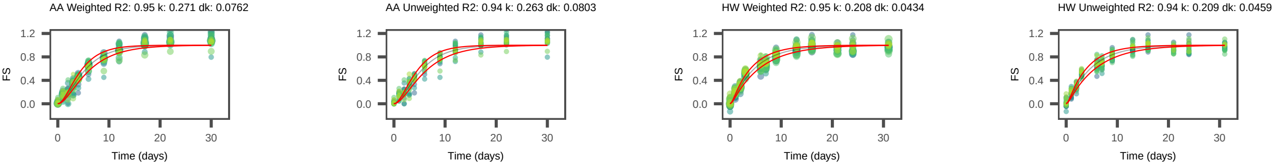

BIP

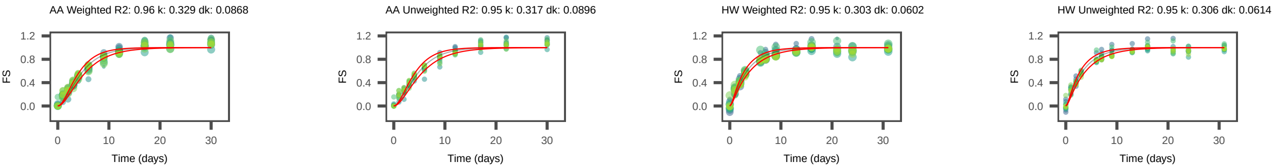

BPHL

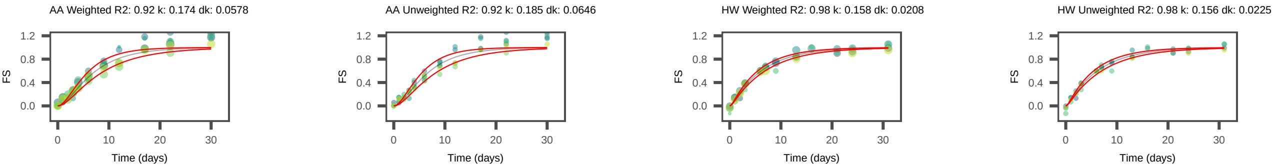

BPNT1

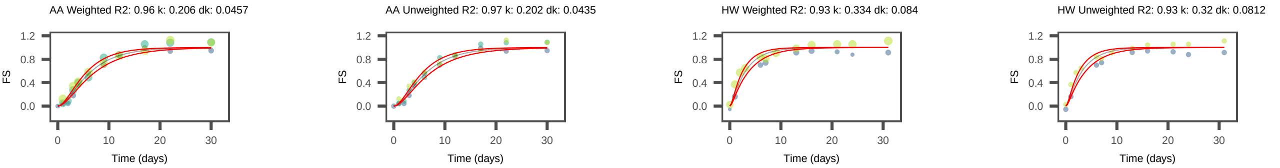

C1TC

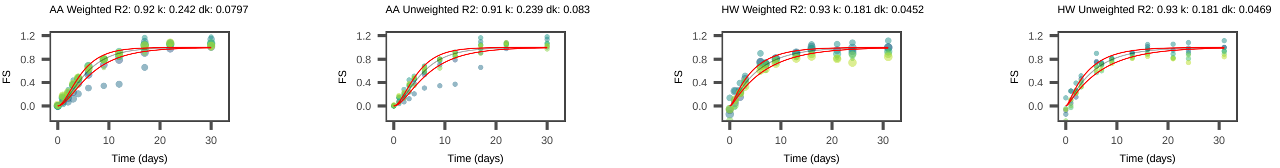

C560

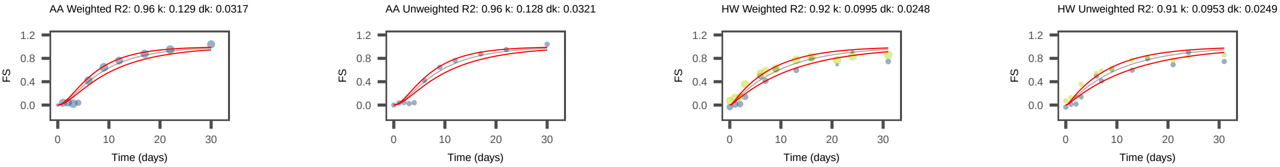

CAH3

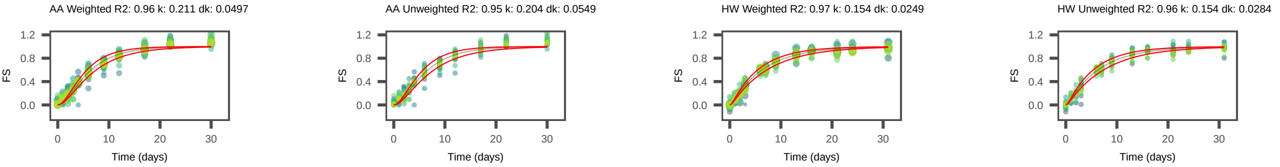

CALR

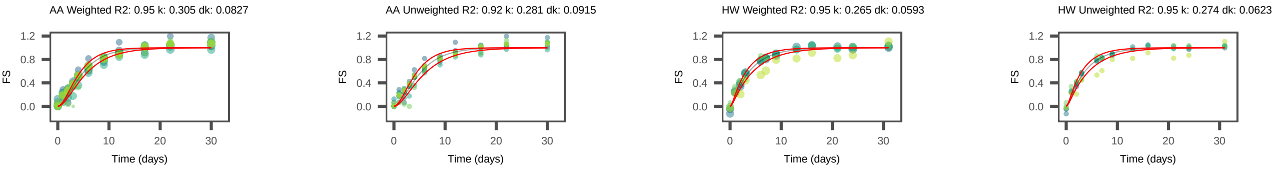

CALU

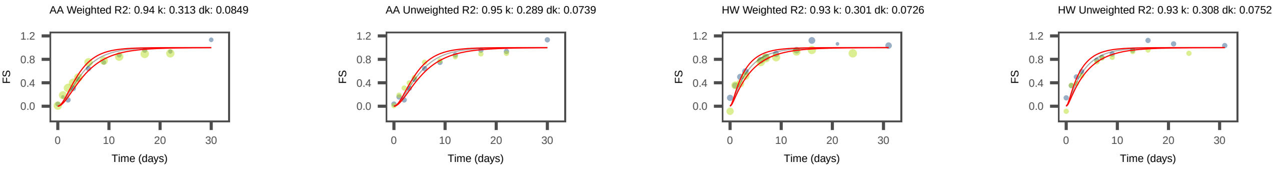

CALX

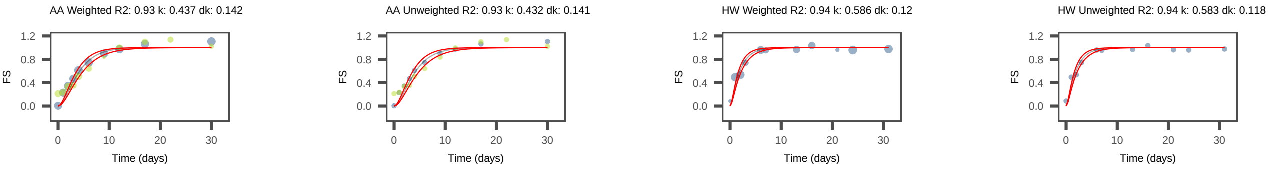

CAND1

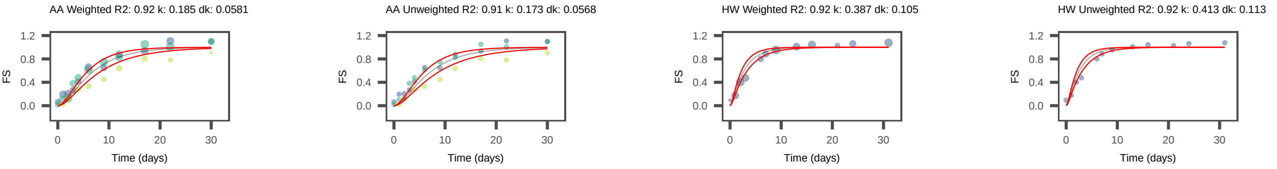

CAP1

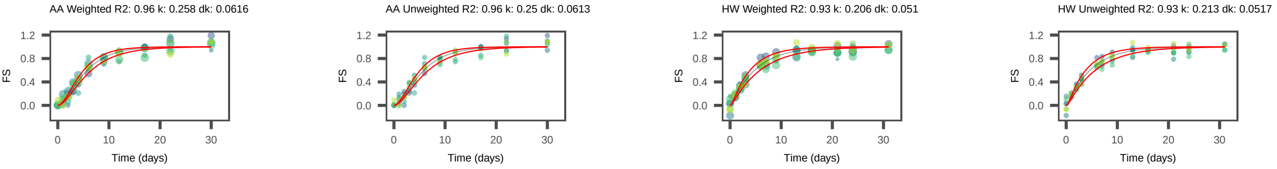

CAPZB

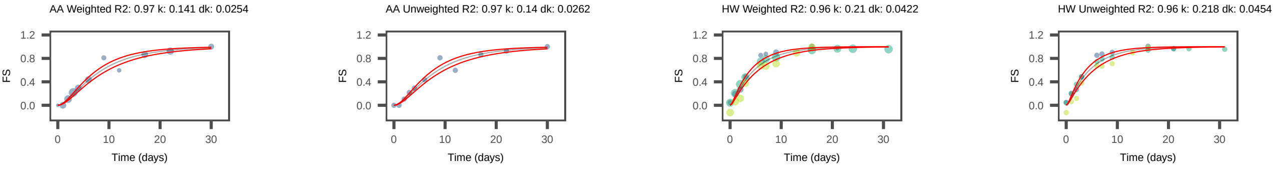

CATA

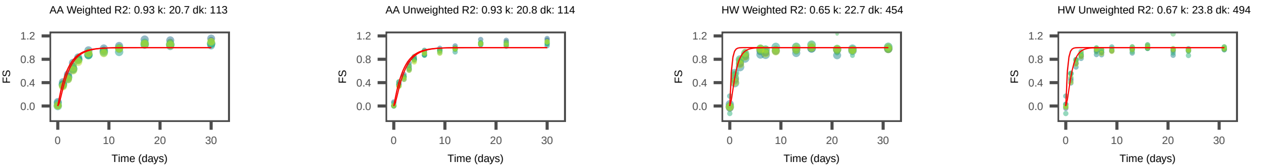

CATB

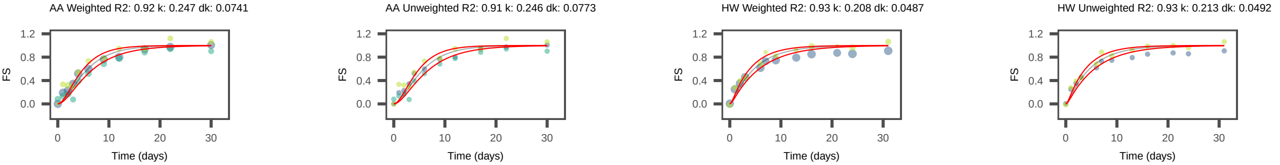

CATD

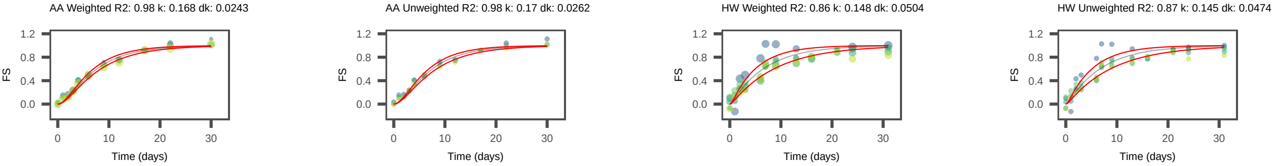

CATF

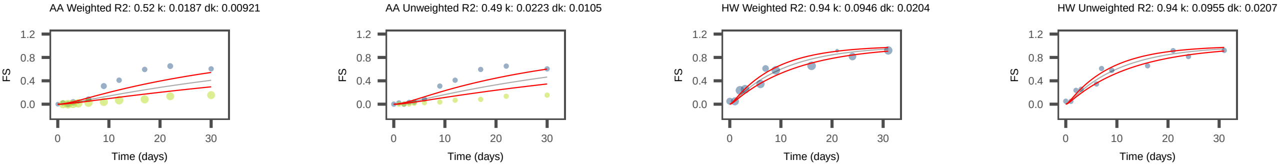

CATZ

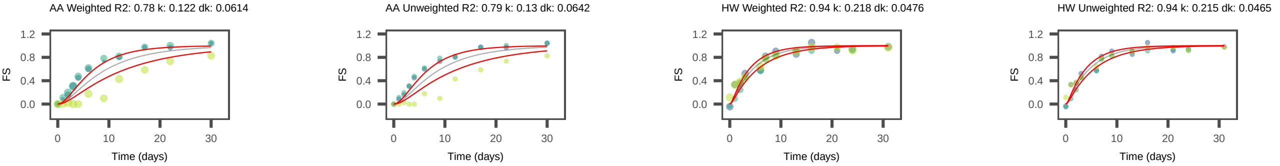

CBPQ

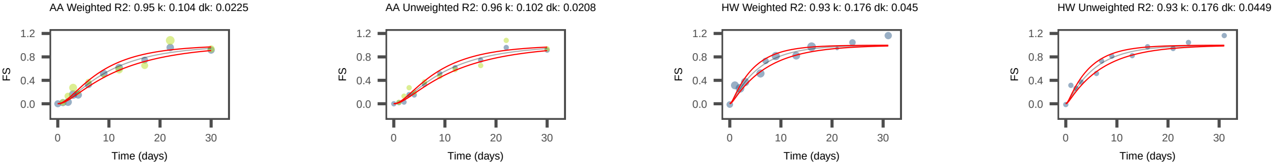

CBR1

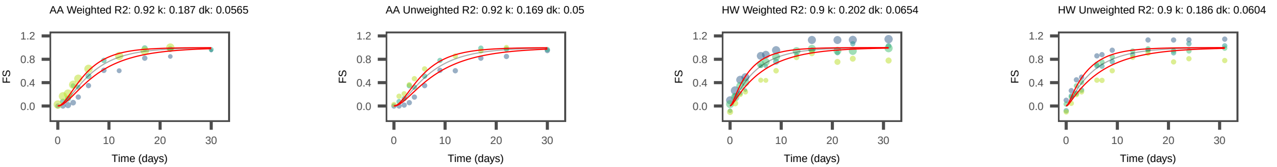

CBR4

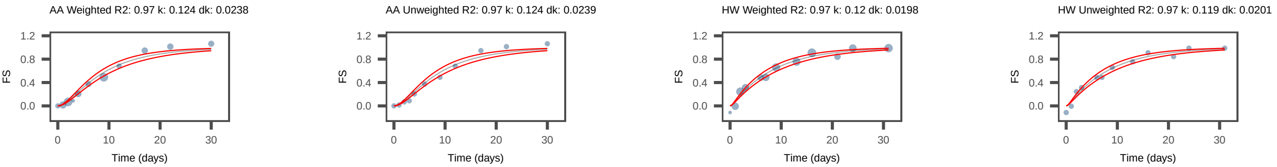

CBS

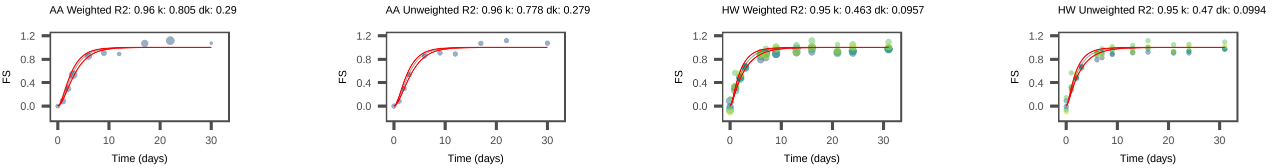

CDC42

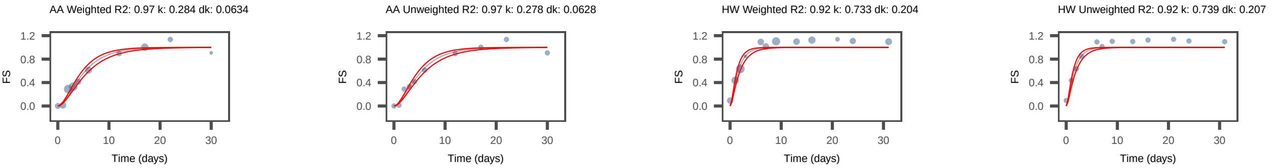

CGL

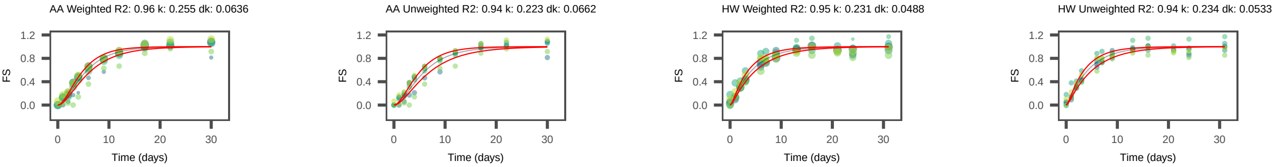

CH10

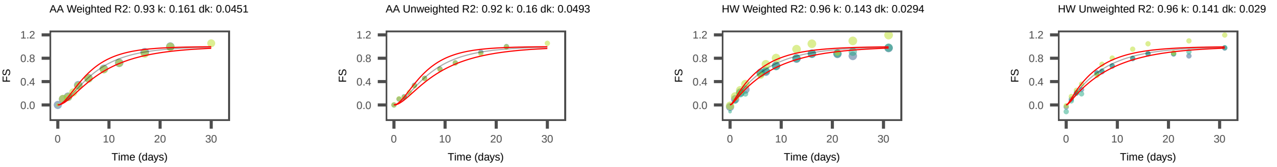

CH60

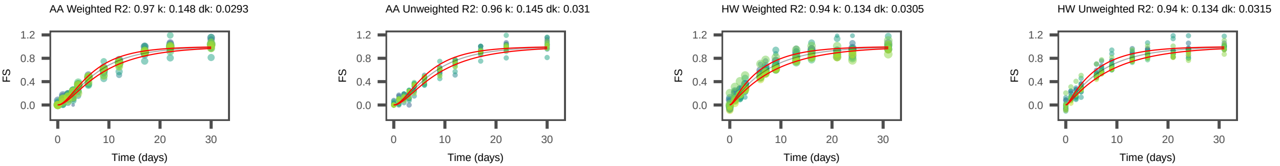

CHDH

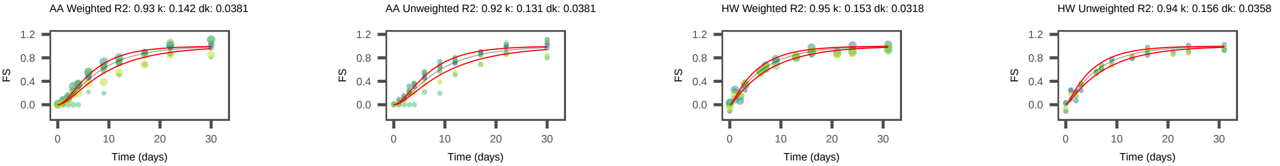

CISD1

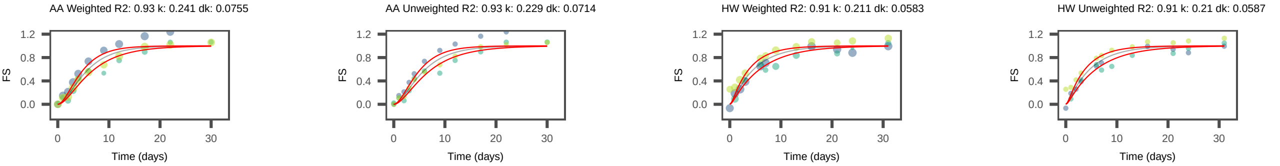

CISY

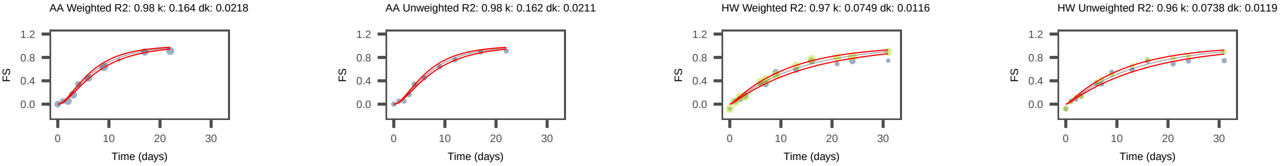

CK054

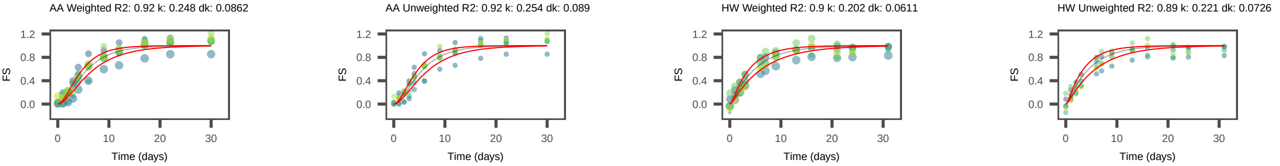

CLH1

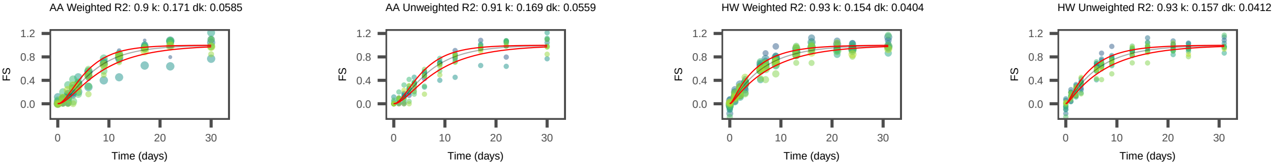

CLPP

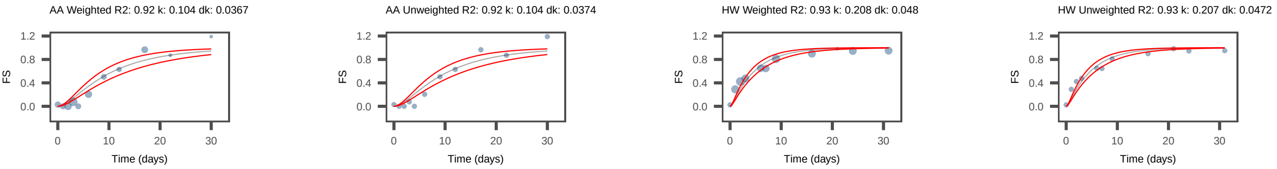

CLYBL

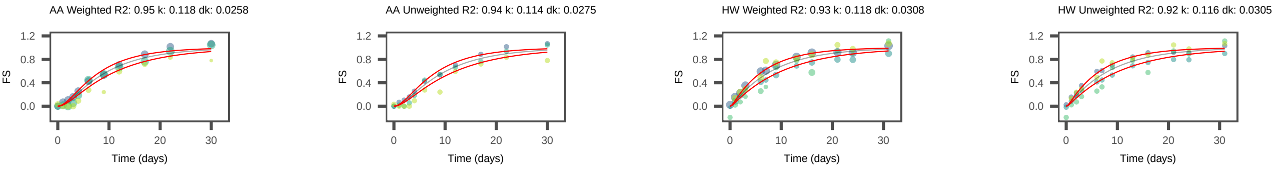

CMBL

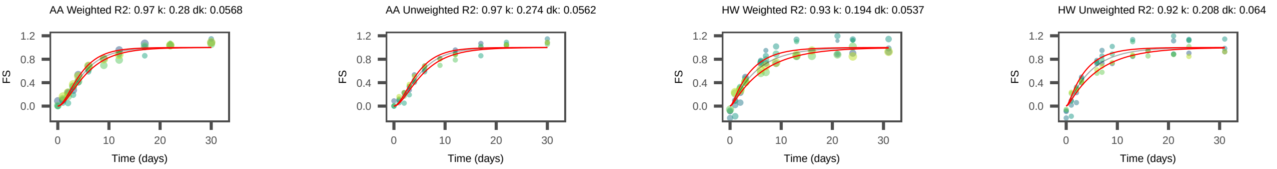

CMC2

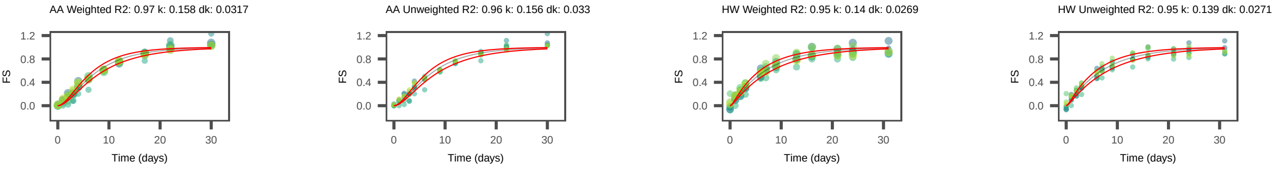

CNBP

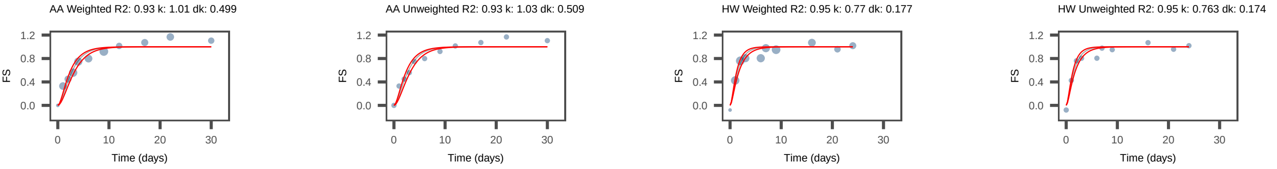

CNDP2

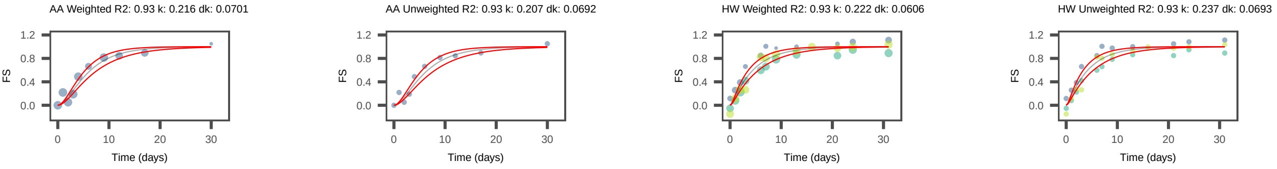

COASY

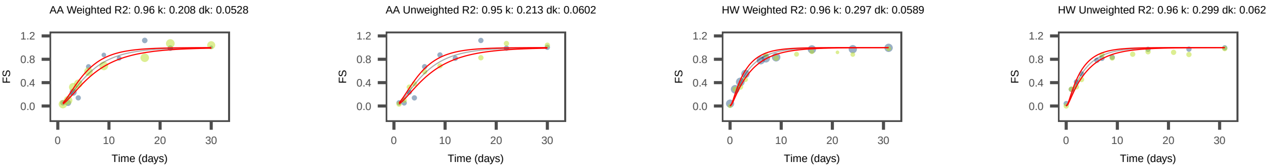

COF1

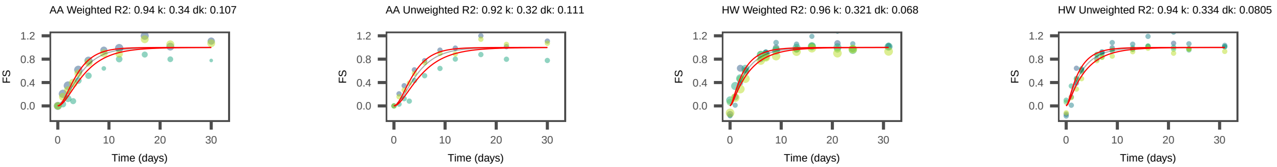

COMT

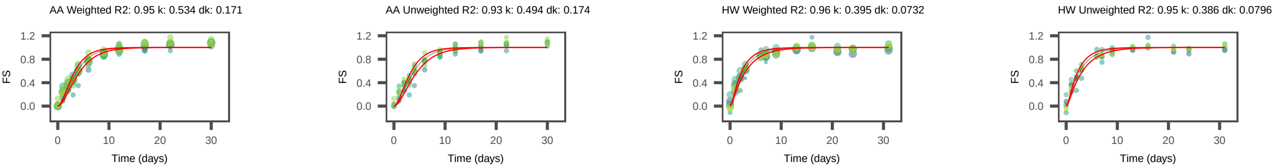

COPB

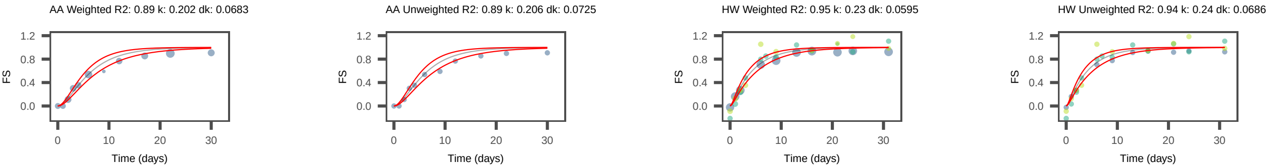

COPB2

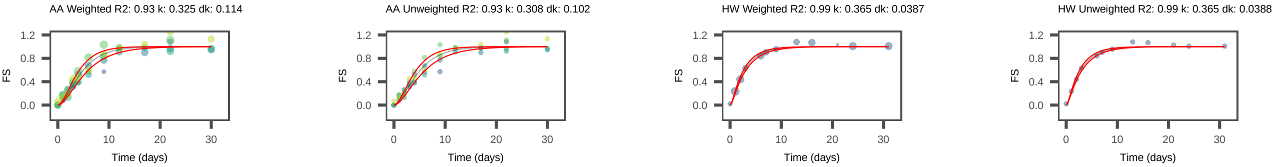

COPD

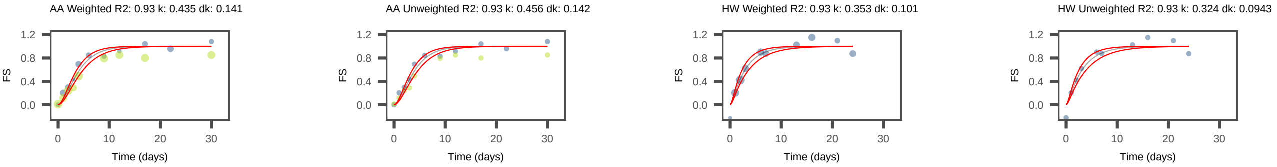

COPG1

AA Weighted R2: 0.94 k: 0.309 dk: 0.0911

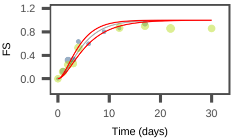

AA Unweighted R2: 0.94 k: 0.303 dk: 0.0892

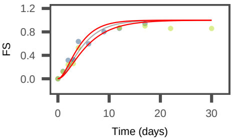

HW Weighted R2: 0.94 k: 0.295 dk: 0.0729

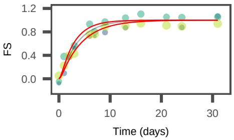

HW Unweighted R2: 0.94 k: 0.288 dk: 0.072

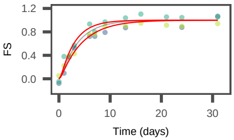

COQ6

AA Weighted R2: 0.97 k: 0.0961 dk: 0.0156

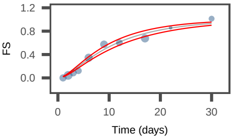

AA Unweighted R2: 0.97 k: 0.0962 dk: 0.0156

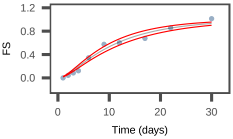

HW Weighted R2: 0.94 k: 0.118 dk: 0.0258

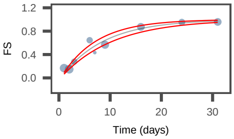

HW Unweighted R2: 0.94 k: 0.118 dk: 0.0262

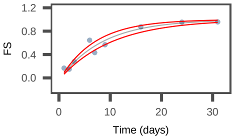

COX1

AA Weighted R2: 0.95 k: 0.152 dk: 0.0441

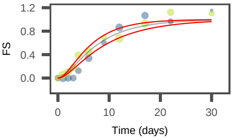

AA Unweighted R2: 0.94 k: 0.152 dk: 0.0447

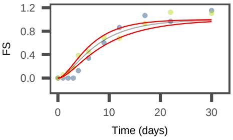

HW Weighted R2: 0.87 k: 0.2 dk: 0.0705

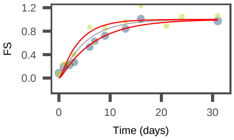

HW Unweighted R2: 0.84 k: 0.221 dk: 0.0889

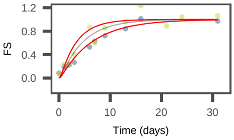

COX2

AA Weighted R2: 0.96 k: 0.166 dk: 0.0346

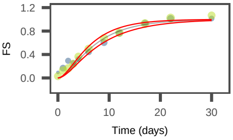

AA Unweighted R2: 0.95 k: 0.155 dk: 0.0362

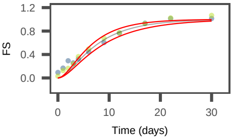

HW Weighted R2: 0.94 k: 0.127 dk: 0.032

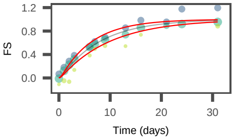

HW Unweighted R2: 0.91 k: 0.118 dk: 0.0352

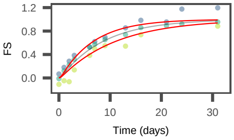

COX3

AA Weighted R2: 0.99 k: 0.166 dk: 0.0195

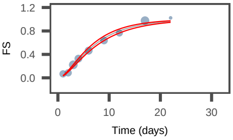

AA Unweighted R2: 0.99 k: 0.165 dk: 0.0196

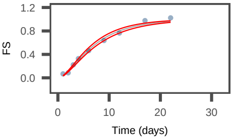

HW Weighted R2: 1 k: 0.145 dk: 0.00914

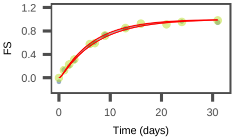

HW Unweighted R2: 0.99 k: 0.144 dk: 0.00987

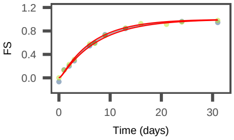

COX41

AA Weighted R2: 0.95 k: 0.202 dk: 0.0518

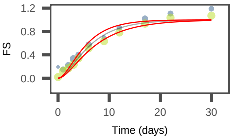

AA Unweighted R2: 0.94 k: 0.204 dk: 0.0554

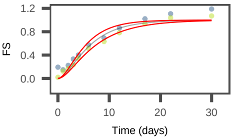

HW Weighted R2: 0.88 k: 0.104 dk: 0.0299

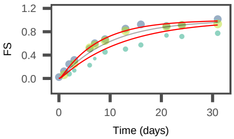

HW Unweighted R2: 0.86 k: 0.0964 dk: 0.0304

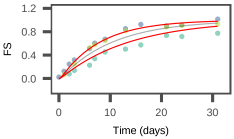

COX5A

AA Weighted R2: 0.86 k: 0.148 dk: 0.0637

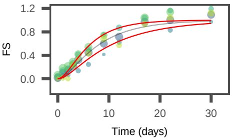

AA Unweighted R2: 0.84 k: 0.136 dk: 0.0611

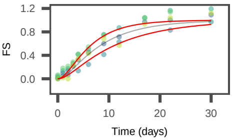

HW Weighted R2: 0.95 k: 0.147 dk: 0.0338

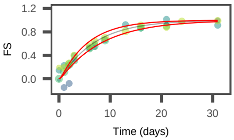

HW Unweighted R2: 0.94 k: 0.149 dk: 0.0361

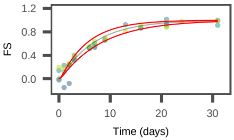

COX5B

AA Weighted R2: 0.95 k: 0.186 dk: 0.0487

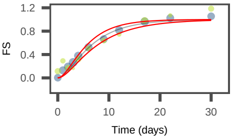

AA Unweighted R2: 0.94 k: 0.184 dk: 0.0497

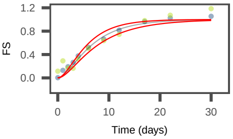

HW Weighted R2: 0.97 k: 0.107 dk: 0.016

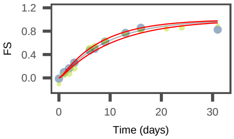

HW Unweighted R2: 0.97 k: 0.107 dk: 0.0162

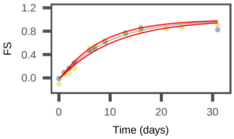

COX6C

AA Weighted R2: 0.98 k: 0.136 dk: 0.0216

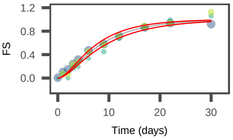

AA Unweighted R2: 0.97 k: 0.131 dk: 0.0223

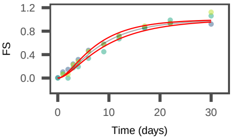

HW Weighted R2: 0.97 k: 0.14 dk: 0.0278

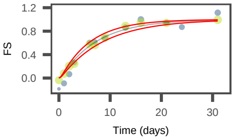

HW Unweighted R2: 0.96 k: 0.14 dk: 0.0312

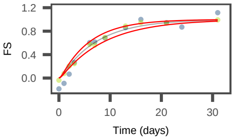

CP1A2

AA Weighted R2: 0.94 k: 8.65 dk: 16.8

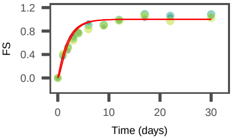

AA Unweighted R2: 0.93 k: 9.39 dk: 21

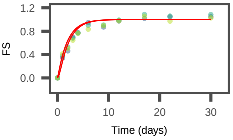

HW Weighted R2: 0.92 k: 0.869 dk: 0.231

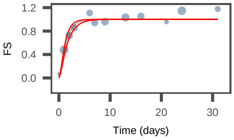

HW Unweighted R2: 0.92 k: 0.865 dk: 0.23

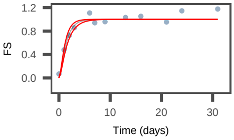

CP254

AA Weighted R2: 0.97 k: 0.496 dk: 0.11

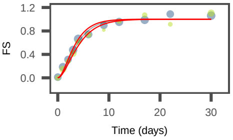

AA Unweighted R2: 0.97 k: 0.479 dk: 0.108

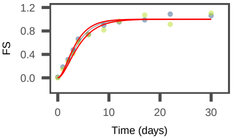

HW Weighted R2: 0.93 k: 0.465 dk: 0.15

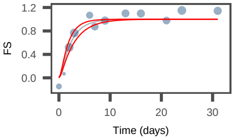

HW Unweighted R2: 0.93 k: 0.454 dk: 0.15

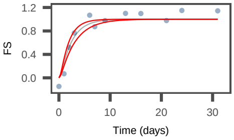

CP27A

AA Weighted R2: 0.94 k: 0.422 dk: 0.14

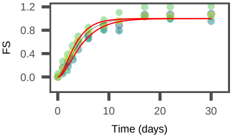

AA Unweighted R2: 0.93 k: 0.403 dk: 0.137

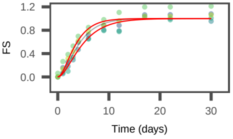

HW Weighted R2: 0.85 k: 0.249 dk: 0.0959

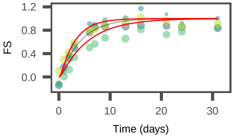

HW Unweighted R2: 0.85 k: 0.246 dk: 0.094

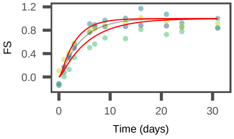

CP2AC

AA Weighted R2: 0.88 k: 12.6 dk: 56

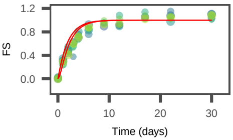

AA Unweighted R2: 0.87 k: 12.4 dk: 55.4

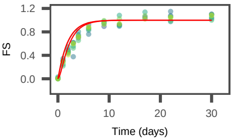

HW Weighted R2: 0.94 k: 0.579 dk: 0.129

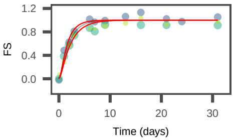

HW Unweighted R2: 0.94 k: 0.591 dk: 0.134

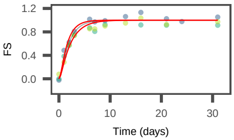

CP2CN

AA Weighted R2: 0.92 k: 6.86 dk: 11.2

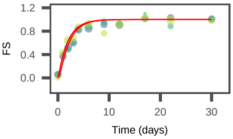

AA Unweighted R2: 0.92 k: 6.72 dk: 10.9

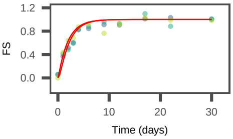

HW Weighted R2: 0.86 k: 6.59 dk: 13.3

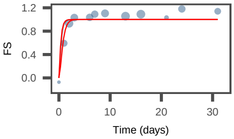

HW Unweighted R2: 0.86 k: 6 dk: 10.3

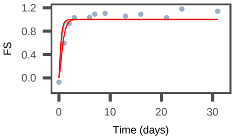

CP2CT

AA Weighted R2: 0.89 k: 17.1 dk: 99.4

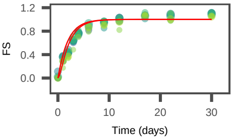

AA Unweighted R2: 0.89 k: 16.4 dk: 94.8

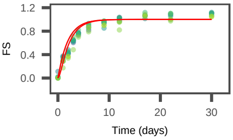

HW Weighted R2: 0.96 k: 0.61 dk: 0.108

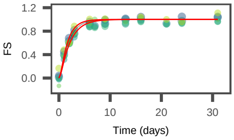

HW Unweighted R2: 0.96 k: 0.606 dk: 0.109

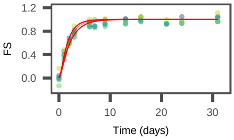

CP2D9

AA Weighted R2: 0.91 k: 0.894 dk: 0.396

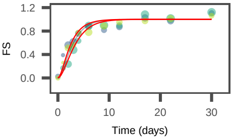

AA Unweighted R2: 0.91 k: 0.914 dk: 0.413

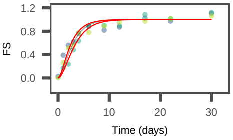

HW Weighted R2: 0.95 k: 0.935 dk: 0.185

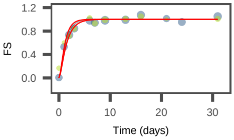

HW Unweighted R2: 0.95 k: 0.95 dk: 0.197

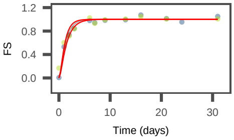

CP2DQ

AA Weighted R2: 0.86 k: 18 dk: 132

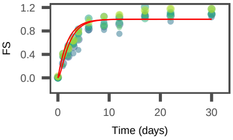

AA Unweighted R2: 0.86 k: 17.4 dk: 124

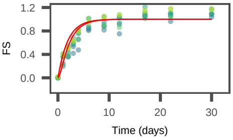

HW Weighted R2: 0.96 k: 0.833 dk: 0.157

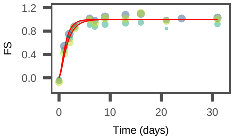

HW Unweighted R2: 0.96 k: 0.83 dk: 0.156

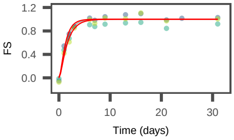

CP2E1

AA Weighted R2: 0.93 k: 50.4 dk: 683

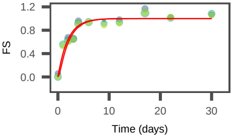

AA Unweighted R2: 0.93 k: 47.3 dk: 602

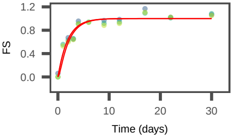

HW Weighted R2: 0.91 k: 7.12 dk: 12.7

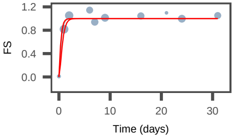

HW Unweighted R2: 0.92 k: 6.67 dk: 9.82

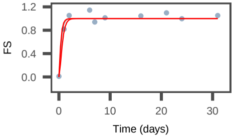

CP2F2

AA Weighted R2: 0.94 k: 13.6 dk: 46.6

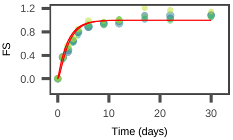

AA Unweighted R2: 0.94 k: 13.4 dk: 45.7

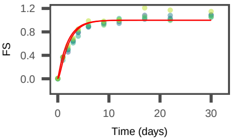

HW Weighted R2: 0.91 k: 0.552 dk: 0.159

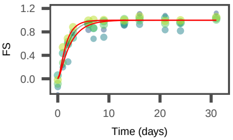

HW Unweighted R2: 0.9 k: 0.54 dk: 0.165

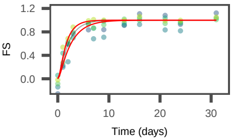

CP2J5

AA Weighted R2: 0.54 k: 0.206 dk: 0.171

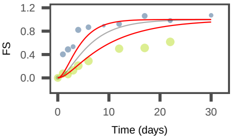

AA Unweighted R2: 0.6 k: 0.414 dk: 0.366

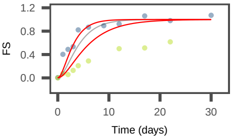

HW Weighted R2: 0.89 k: 7.72 dk: 15.5

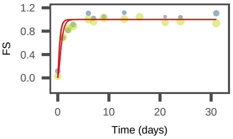

HW Unweighted R2: 0.89 k: 7.49 dk: 14.1

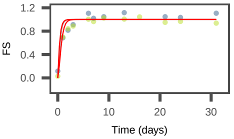

CP3AB

AA Weighted R2: 0.91 k: 98.4 dk: 3000

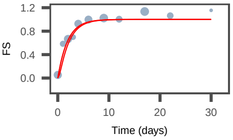

AA Unweighted R2: 0.91 k: 98.1 dk: 3020

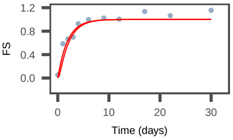

HW Weighted R2: 0.98 k: 2.45 dk: 0.39

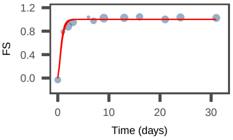

HW Unweighted R2: 0.98 k: 2.56 dk: 0.414

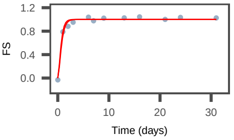

CP3AD

AA Weighted R2: 0.88 k: 0.912 dk: 0.56

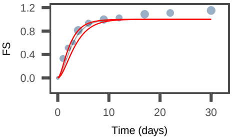

AA Unweighted R2: 0.9 k: 1.03 dk: 0.618

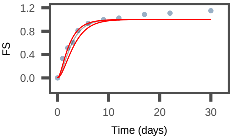

HW Weighted R2: 0.93 k: 0.882 dk: 0.223

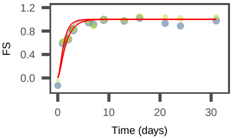

HW Unweighted R2: 0.93 k: 0.889 dk: 0.224

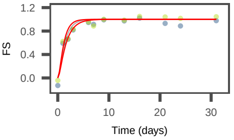

CP7B1

AA Weighted R2: 0.92 k: 2.79 dk: 2.5

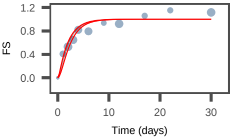

AA Unweighted R2: 0.92 k: 3.14 dk: 2.99

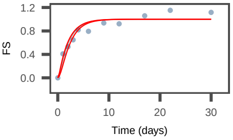

HW Weighted R2: 0.96 k: 0.787 dk: 0.16

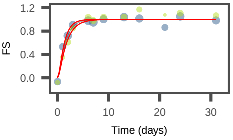

HW Unweighted R2: 0.95 k: 0.772 dk: 0.165

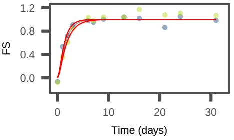

CPSM

AA Weighted R2: 0.94 k: 0.14 dk: 0.0344

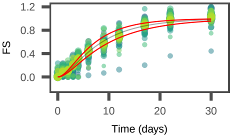

AA Unweighted R2: 0.92 k: 0.135 dk: 0.0404

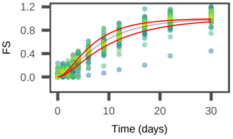

HW Weighted R2: 0.95 k: 0.117 dk: 0.0241

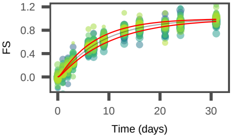

HW Unweighted R2: 0.94 k: 0.118 dk: 0.0279

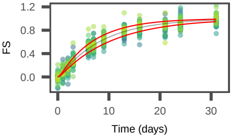

CPT1A

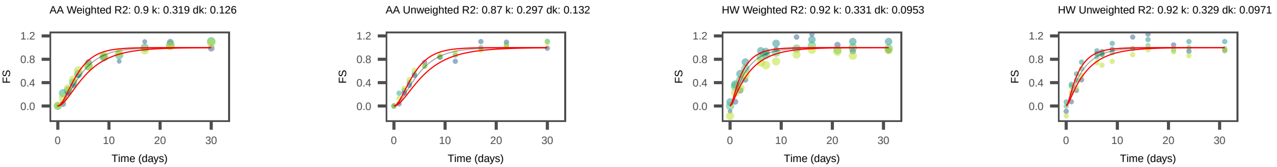

CPT2

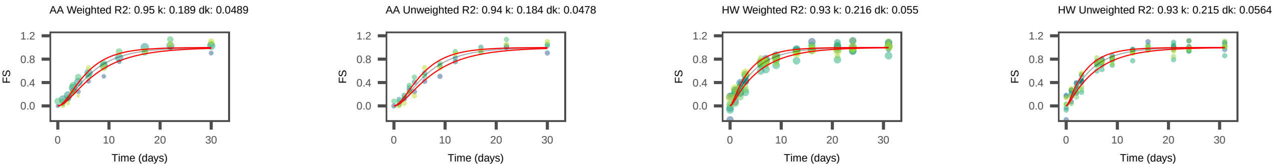

CSAD

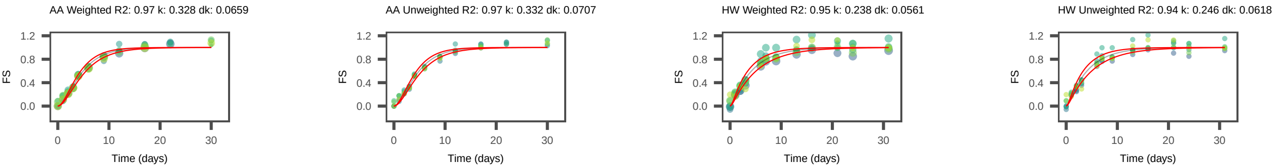

CSN2

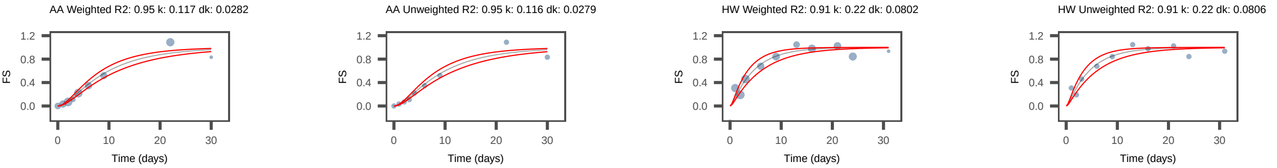

CSN4

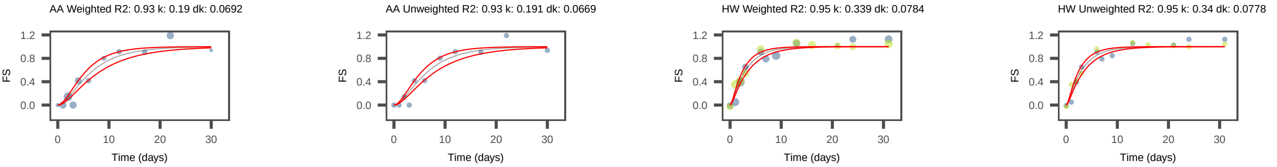

CSRP1

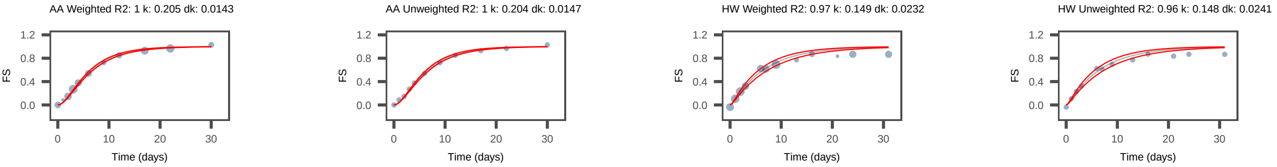

CTNA1

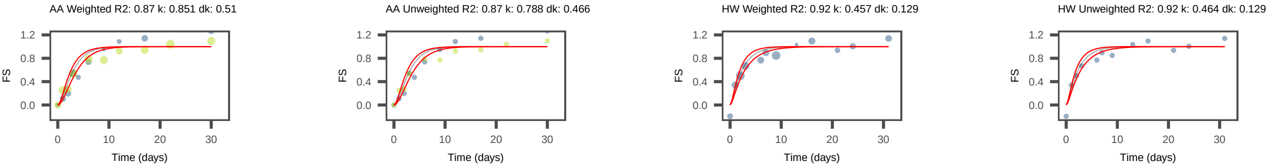

CTND1

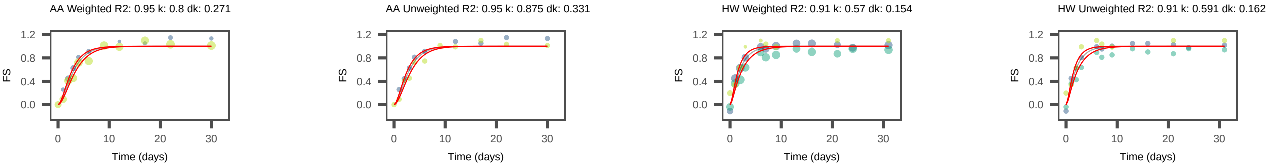

CX6A1

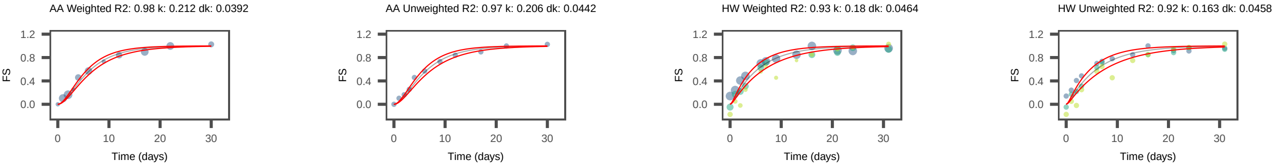

CY1

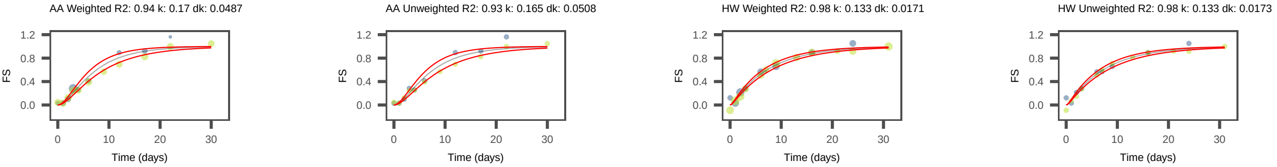

CYB5

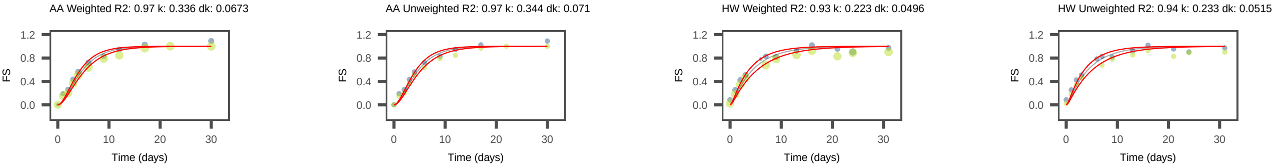

CYC

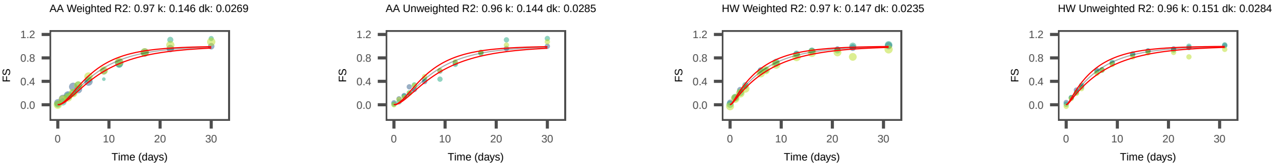

DCXR

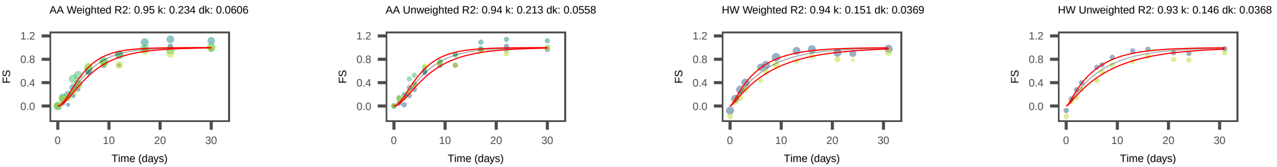

DDAH1

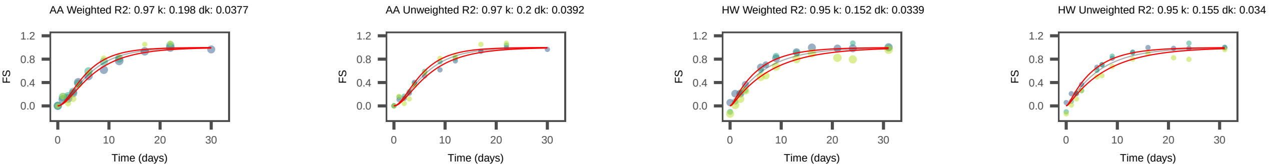

DDB1

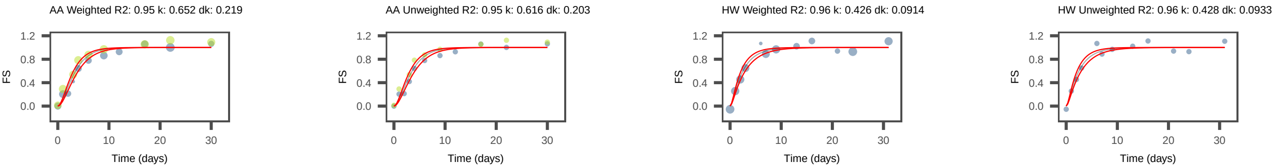

DDC

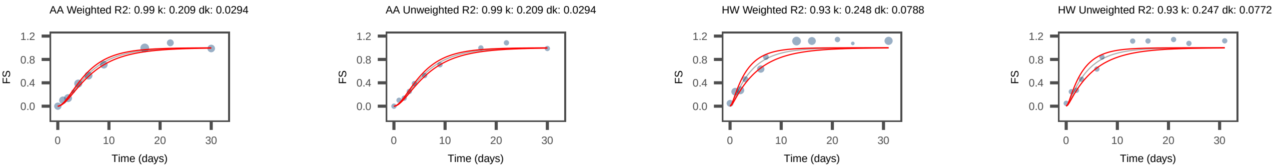

DDX1

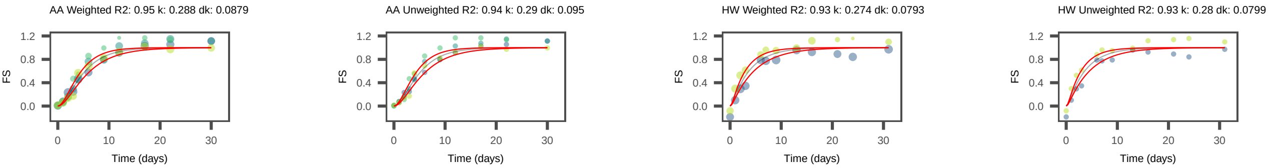

DECR

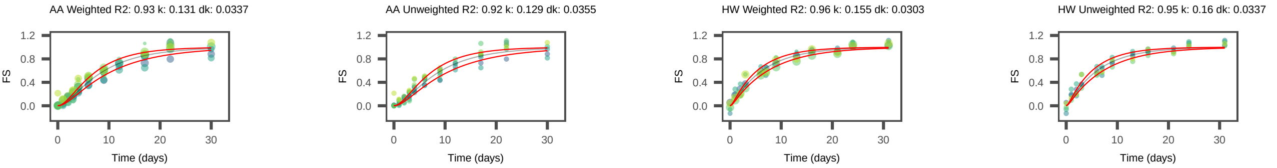

DECR2

AA Weighted R2: 0.98 k: 0.283 dk: 0.042

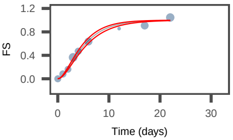

AA Unweighted R2: 0.98 k: 0.28 dk: 0.0416

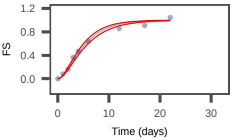

HW Weighted R2: 0.99 k: 0.405 dk: 0.0278

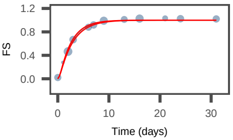

HW Unweighted R2: 0.99 k: 0.407 dk: 0.0283

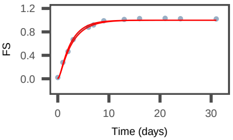

DEST

AA Weighted R2: 0.92 k: 0.411 dk: 0.144

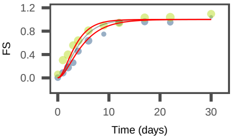

AA Unweighted R2: 0.92 k: 0.395 dk: 0.137

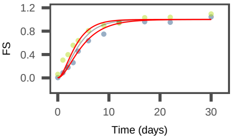

HW Weighted R2: 0.95 k: 0.205 dk: 0.0421

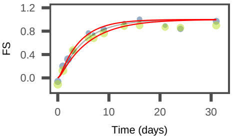

HW Unweighted R2: 0.95 k: 0.205 dk: 0.0423

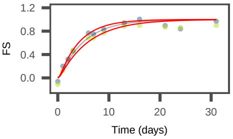

DHB12

AA Weighted R2: 0.96 k: 0.286 dk: 0.0697

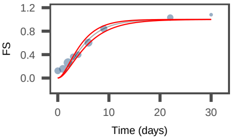

AA Unweighted R2: 0.96 k: 0.286 dk: 0.0692

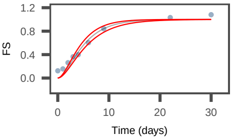

HW Weighted R2: 0.87 k: 0.278 dk: 0.0934

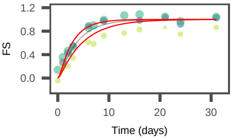

HW Unweighted R2: 0.85 k: 0.257 dk: 0.0927

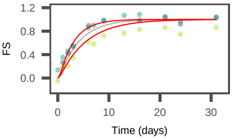

DHB13

AA Weighted R2: 0.97 k: 0.712 dk: 0.185

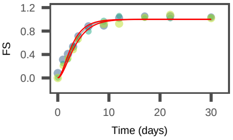

AA Unweighted R2: 0.97 k: 0.702 dk: 0.18

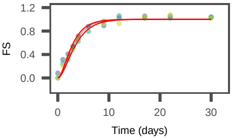

HW Weighted R2: 0.88 k: 0.325 dk: 0.127

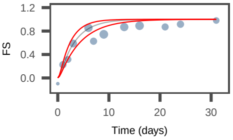

HW Unweighted R2: 0.87 k: 0.362 dk: 0.145

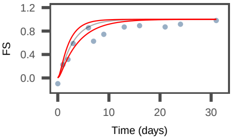

DHB4

AA Weighted R2: 0.95 k: 0.53 dk: 0.167

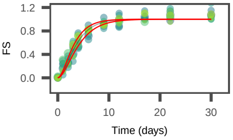

AA Unweighted R2: 0.95 k: 0.521 dk: 0.163

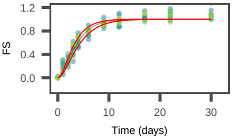

HW Weighted R2: 0.96 k: 0.508 dk: 0.103

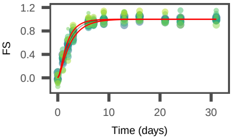

HW Unweighted R2: 0.95 k: 0.516 dk: 0.116

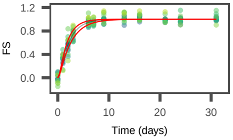

DHB5

AA Weighted R2: 0.93 k: 0.283 dk: 0.0917

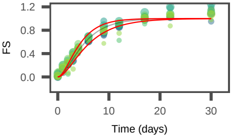

AA Unweighted R2: 0.9 k: 0.247 dk: 0.0914

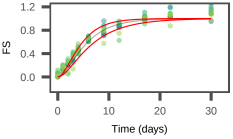

HW Weighted R2: 0.98 k: 0.244 dk: 0.0403

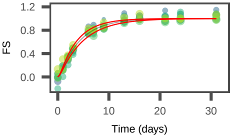

HW Unweighted R2: 0.97 k: 0.242 dk: 0.0438

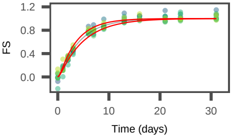

DHCR7

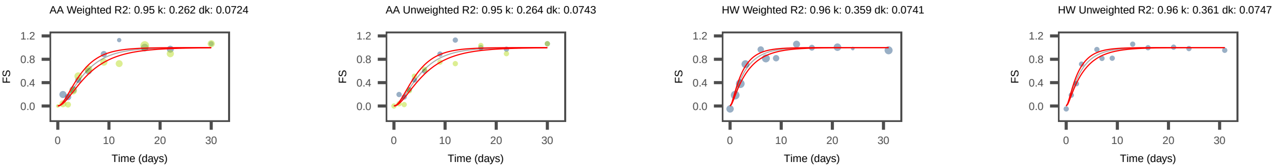

DHDH

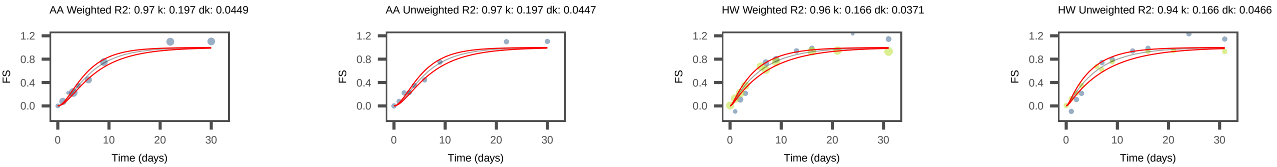

DHE3

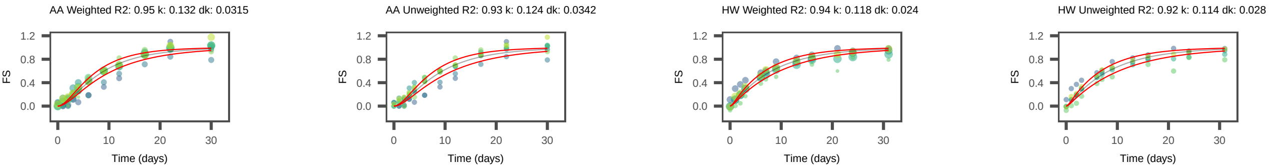

DHI1

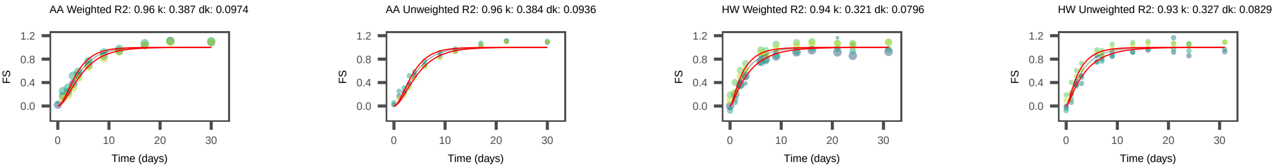

DHPR

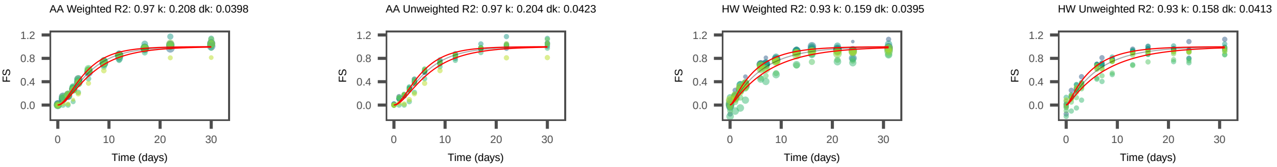

DHRS1

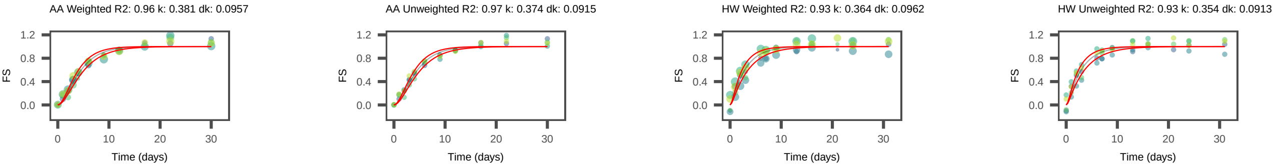

DHRS4

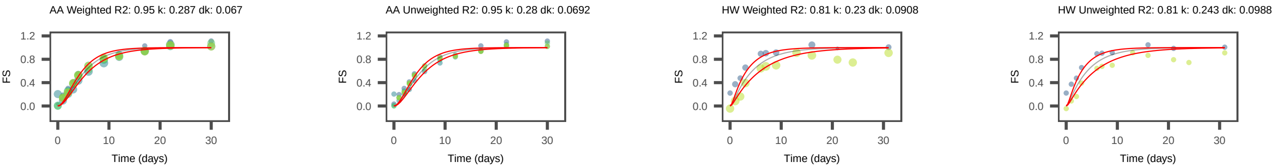

DHSO

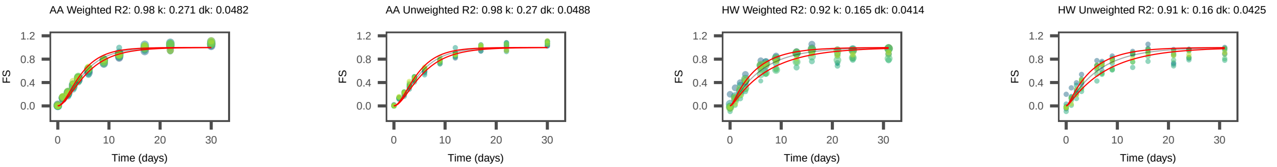

DHTK1

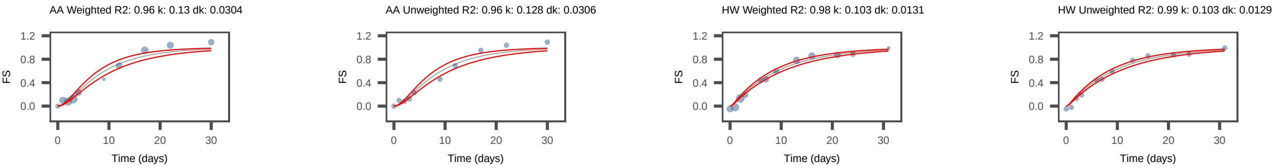

DHX9

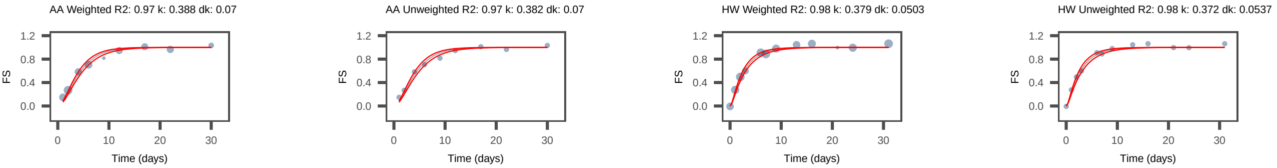

DIC

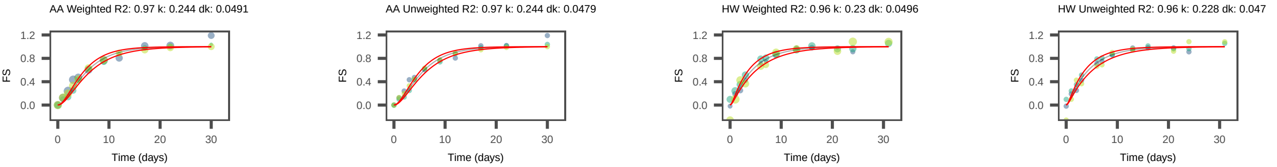

DLDH

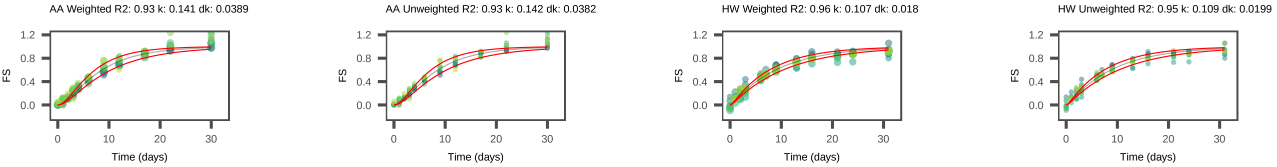

DNJA3

AA Weighted R2: 0.94 k: 0.776 dk: 0.26

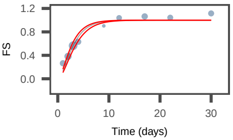

AA Unweighted R2: 0.94 k: 0.769 dk: 0.262

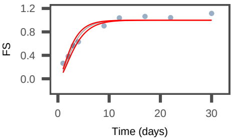

HW Weighted R2: 0.95 k: 0.515 dk: 0.103

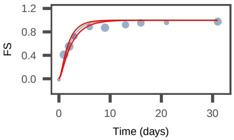

HW Unweighted R2: 0.95 k: 0.512 dk: 0.1

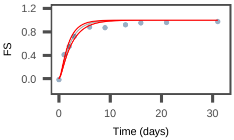

DOPD

AA Weighted R2: 0.98 k: 0.206 dk: 0.0362

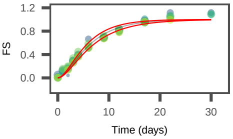

AA Unweighted R2: 0.97 k: 0.206 dk: 0.0374

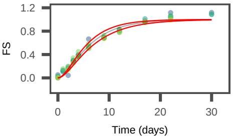

HW Weighted R2: 0.94 k: 0.148 dk: 0.0369

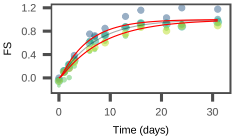

HW Unweighted R2: 0.93 k: 0.144 dk: 0.0383

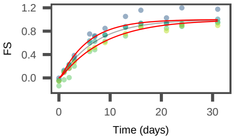

DPP4

AA Weighted R2: 0.92 k: 0.105 dk: 0.0288

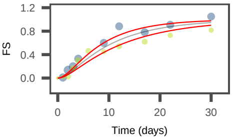

AA Unweighted R2: 0.92 k: 0.1 dk: 0.0274

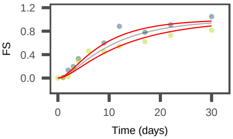

HW Weighted R2: 0.89 k: 0.155 dk: 0.0567

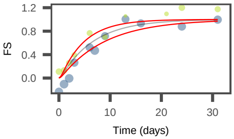

HW Unweighted R2: 0.9 k: 0.16 dk: 0.0557

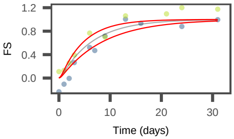

DPYD

AA Weighted R2: 0.94 k: 0.696 dk: 0.253

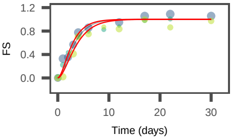

AA Unweighted R2: 0.94 k: 0.675 dk: 0.241

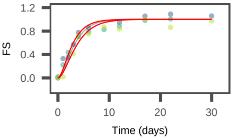

HW Weighted R2: 0.93 k: 0.726 dk: 0.18

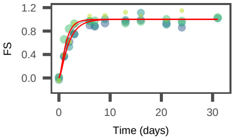

HW Unweighted R2: 0.93 k: 0.721 dk: 0.176

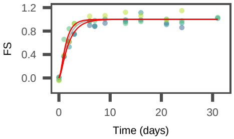

DPYS

AA Weighted R2: 0.96 k: 0.273 dk: 0.0661

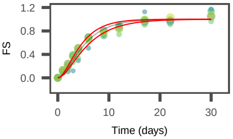

AA Unweighted R2: 0.95 k: 0.257 dk: 0.0665

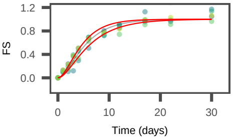

HW Weighted R2: 0.97 k: 0.305 dk: 0.0547

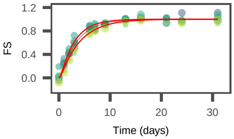

HW Unweighted R2: 0.96 k: 0.304 dk: 0.0557

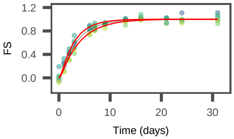

DYHC1

AA Weighted R2: 0.99 k: 0.106 dk: 0.0081

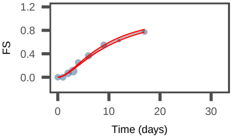

AA Unweighted R2: 0.99 k: 0.106 dk: 0.00806

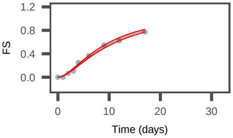

HW Weighted R2: 0.86 k: 0.196 dk: 0.0753

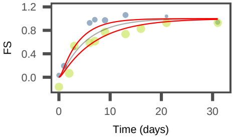

HW Unweighted R2: 0.87 k: 0.226 dk: 0.0876

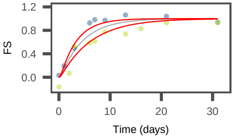

EBP

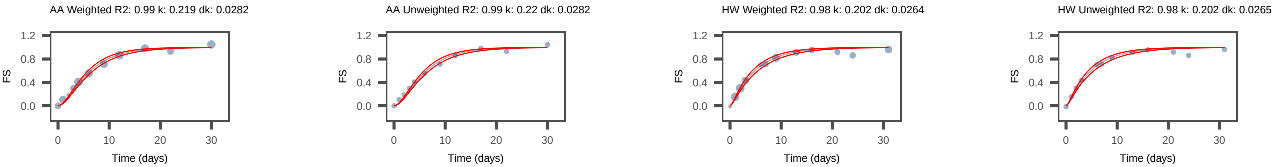

ECH1

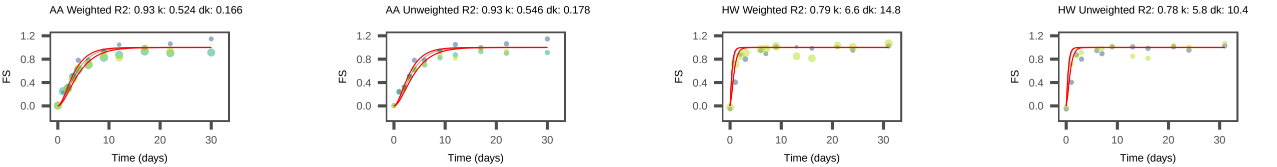

ECHA

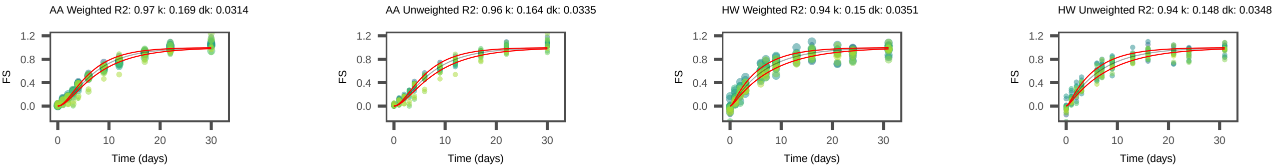

ECHB

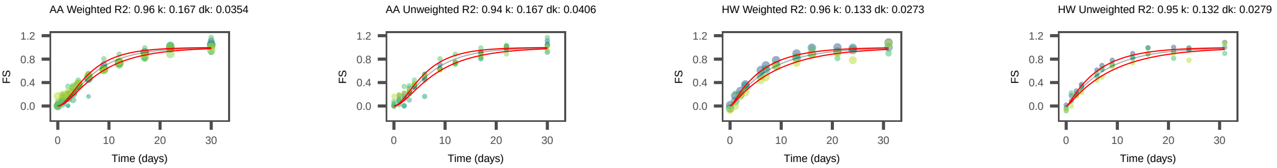

ECHD3

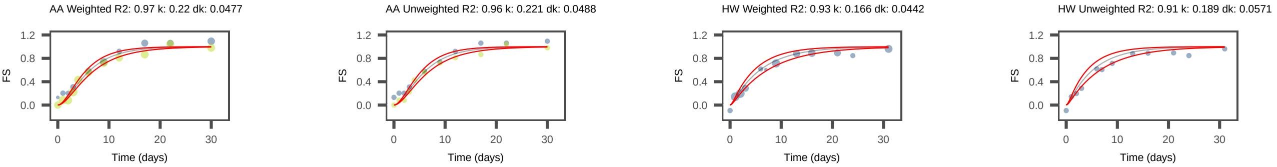

ECHM

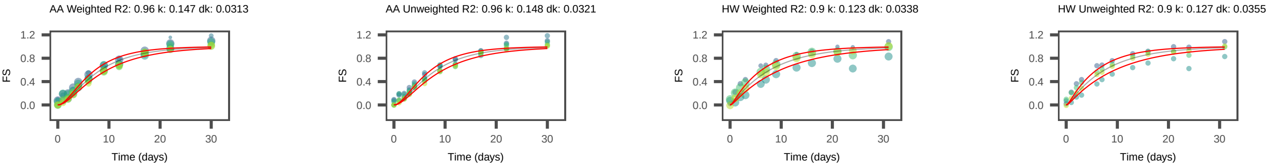

ECHP

AA Weighted R2: 0.96 k: 0.384 dk: 0.101

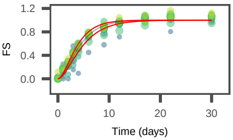

AA Unweighted R2: 0.95 k: 0.372 dk: 0.11

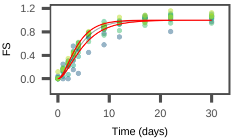

HW Weighted R2: 0.94 k: 0.355 dk: 0.0822

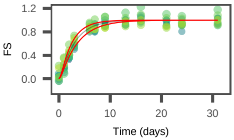

HW Unweighted R2: 0.94 k: 0.355 dk: 0.0824

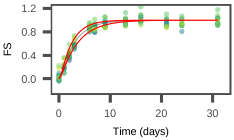

ECI1

AA Weighted R2: 0.96 k: 0.139 dk: 0.0269

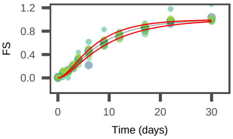

AA Unweighted R2: 0.95 k: 0.144 dk: 0.0316

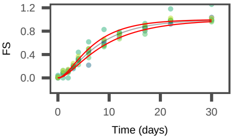

HW Weighted R2: 0.95 k: 0.11 dk: 0.022

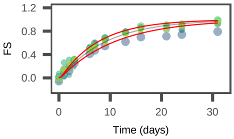

HW Unweighted R2: 0.95 k: 0.113 dk: 0.0213

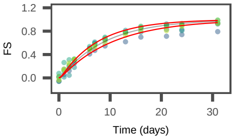

ECI2

AA Weighted R2: 0.94 k: 0.272 dk: 0.0771

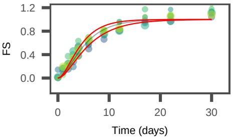

AA Unweighted R2: 0.94 k: 0.271 dk: 0.079

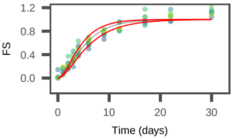

HW Weighted R2: 0.91 k: 0.188 dk: 0.0556

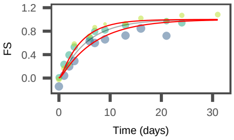

HW Unweighted R2: 0.91 k: 0.199 dk: 0.057

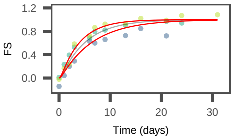

EF1A1

AA Weighted R2: 0.95 k: 0.305 dk: 0.0874

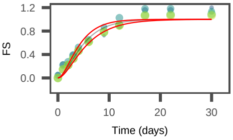

AA Unweighted R2: 0.93 k: 0.287 dk: 0.0928

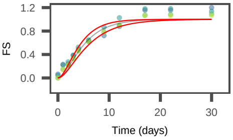

HW Weighted R2: 0.95 k: 0.174 dk: 0.0373

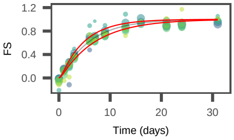

HW Unweighted R2: 0.93 k: 0.18 dk: 0.047

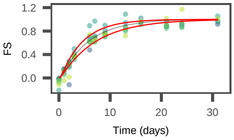

EF1B

AA Weighted R2: 0.96 k: 0.143 dk: 0.0313

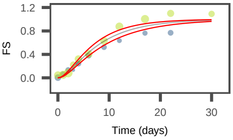

AA Unweighted R2: 0.96 k: 0.141 dk: 0.0304

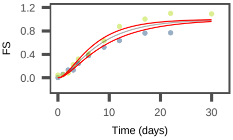

HW Weighted R2: 0.9 k: 0.121 dk: 0.0317

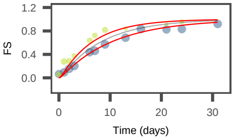

HW Unweighted R2: 0.9 k: 0.128 dk: 0.0343

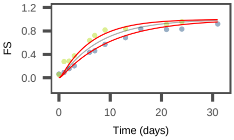

EF1D

AA Weighted R2: 0.9 k: 0.233 dk: 0.0868

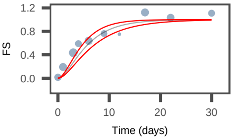

AA Unweighted R2: 0.9 k: 0.233 dk: 0.0872

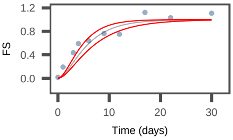

HW Weighted R2: 0.79 k: 0.118 dk: 0.0579

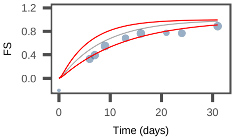

HW Unweighted R2: 0.77 k: 0.14 dk: 0.073

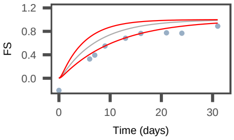

EF1G

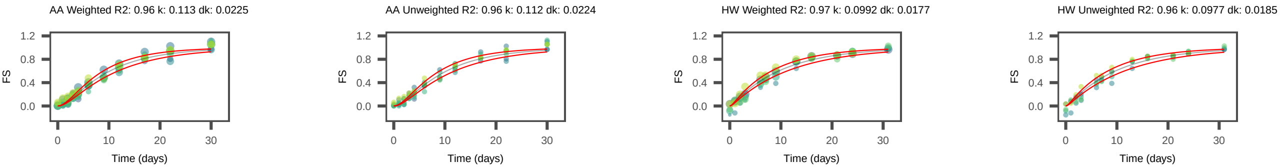

EF2

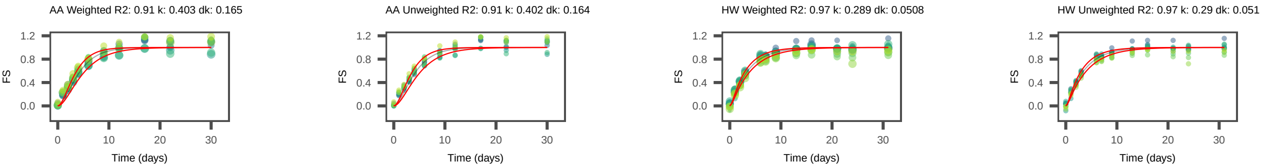

EFTU

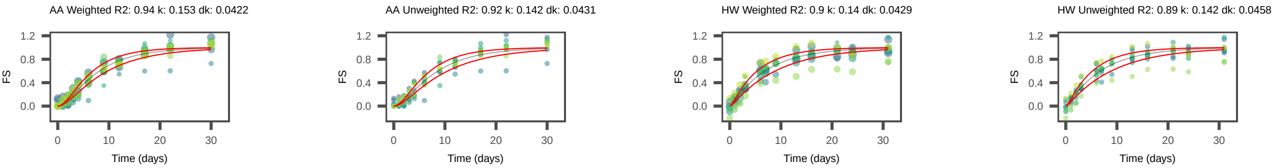

EGFR

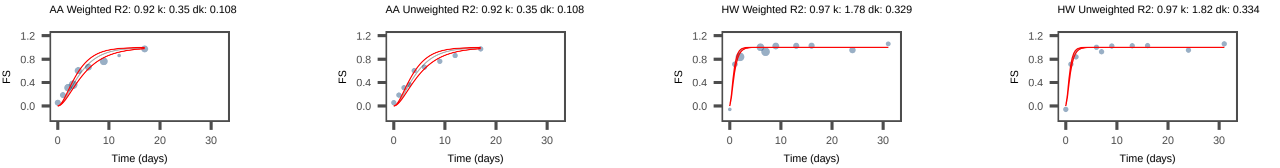

EIF3E

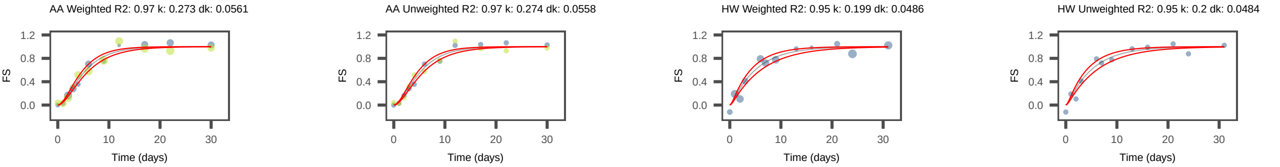

EIF3F

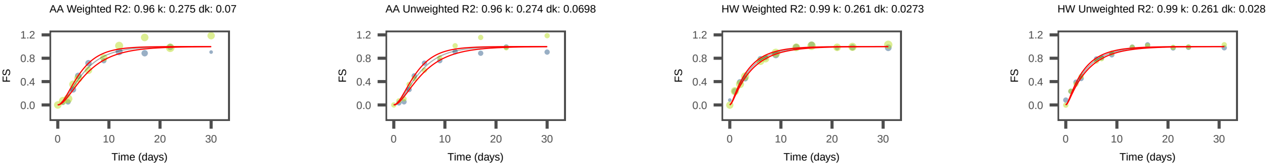

EIF3H

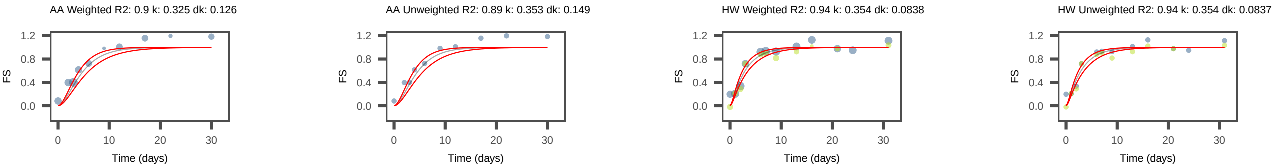

EIF3L

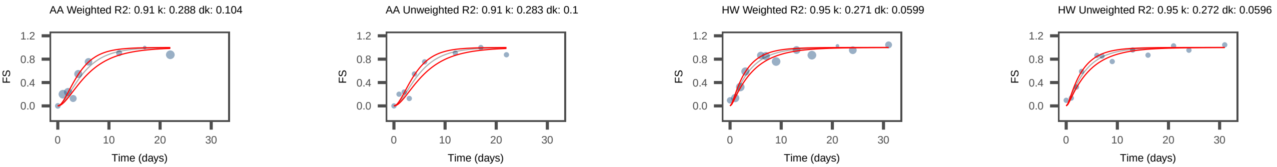

ENOA

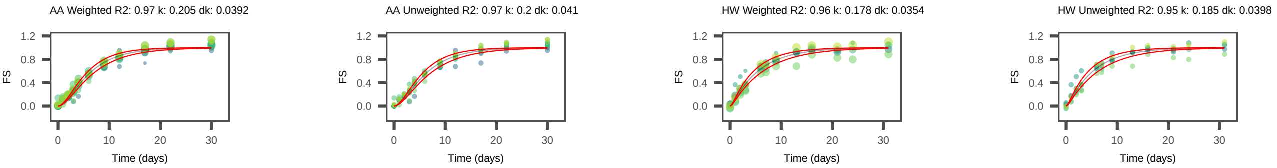

ENPL

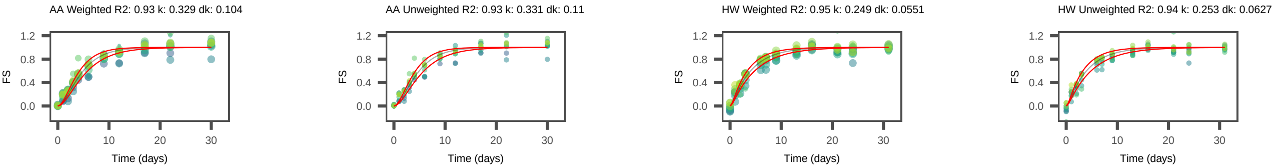

ENTP5

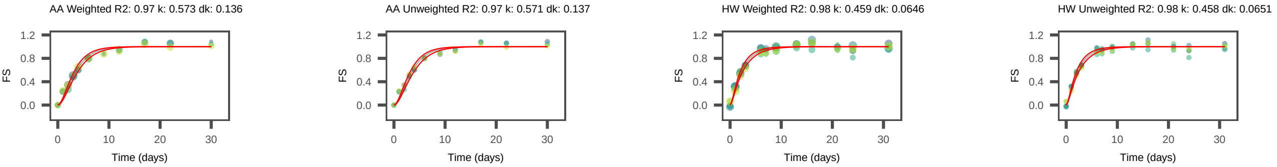

ERAP1

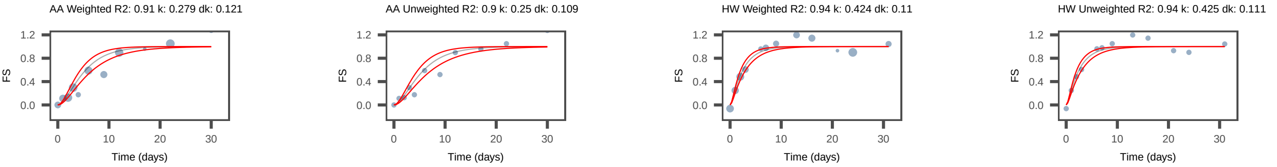

EST1

AA Weighted R2: 0.96 k: 0.305 dk: 0.0758

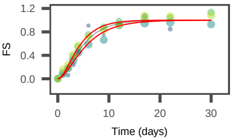

AA Unweighted R2: 0.95 k: 0.295 dk: 0.0788

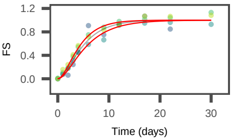

HW Weighted R2: 0.96 k: 0.188 dk: 0.0375

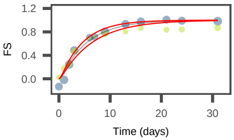

HW Unweighted R2: 0.96 k: 0.188 dk: 0.0374

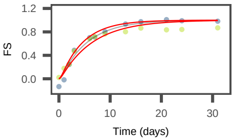

EST1D

AA Weighted R2: 0.95 k: 0.448 dk: 0.134

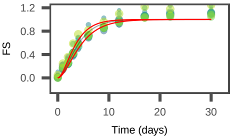

AA Unweighted R2: 0.95 k: 0.448 dk: 0.138

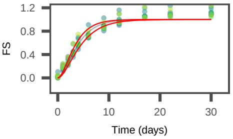

HW Weighted R2: 0.95 k: 0.241 dk: 0.0538

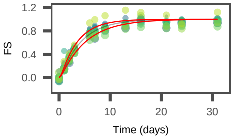

HW Unweighted R2: 0.95 k: 0.253 dk: 0.0583

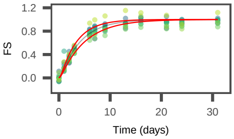

EST1F

AA Weighted R2: 0.93 k: 0.314 dk: 0.104

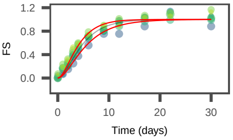

AA Unweighted R2: 0.91 k: 0.301 dk: 0.108

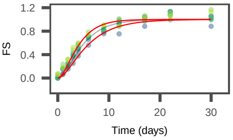

HW Weighted R2: 0.95 k: 0.257 dk: 0.0588

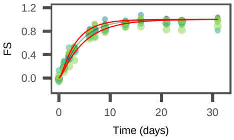

HW Unweighted R2: 0.95 k: 0.269 dk: 0.063

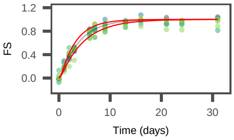

EST2A

AA Weighted R2: 0.94 k: 0.387 dk: 0.118

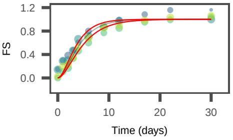

AA Unweighted R2: 0.94 k: 0.388 dk: 0.122

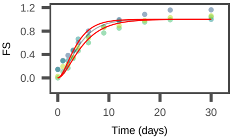

HW Weighted R2: 0.95 k: 0.279 dk: 0.0651

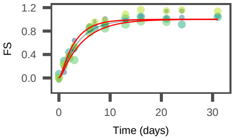

HW Unweighted R2: 0.95 k: 0.286 dk: 0.0687

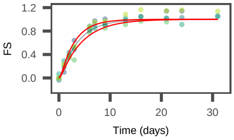

EST2C

AA Weighted R2: 0.91 k: 0.325 dk: 0.126

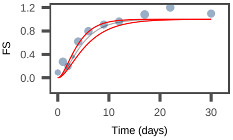

AA Unweighted R2: 0.89 k: 0.301 dk: 0.127

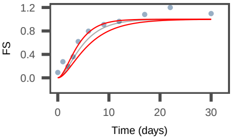

HW Weighted R2: 0.94 k: 0.212 dk: 0.0482

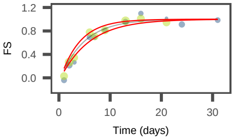

HW Unweighted R2: 0.94 k: 0.207 dk: 0.0492

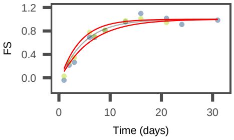

EST2E

AA Weighted R2: 0.95 k: 0.315 dk: 0.0779

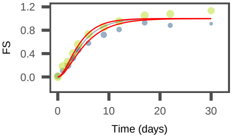

AA Unweighted R2: 0.95 k: 0.308 dk: 0.0759

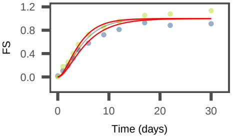

HW Weighted R2: 0.97 k: 0.31 dk: 0.0549

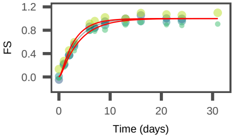

HW Unweighted R2: 0.96 k: 0.3 dk: 0.0552

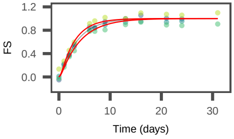

EST3A

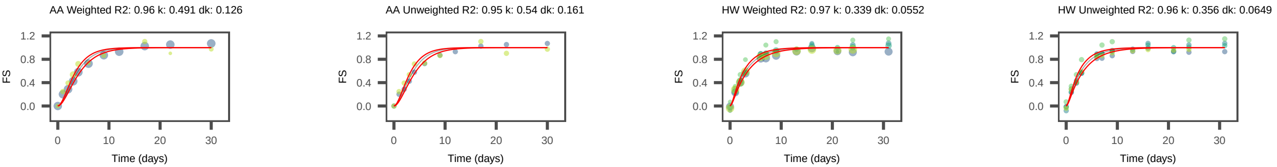

EST3B

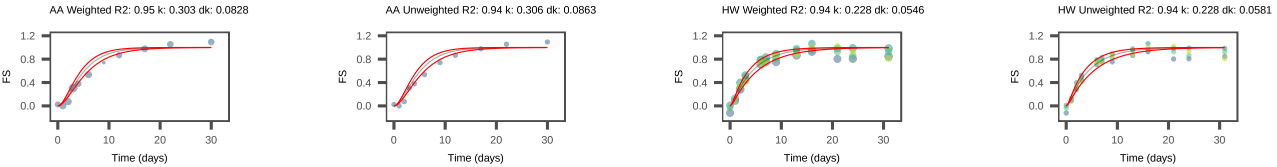

ESTD

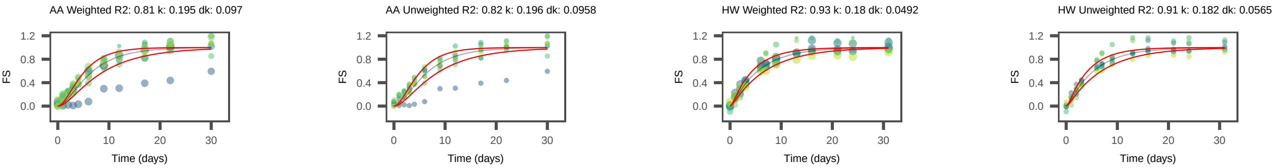

ETFA

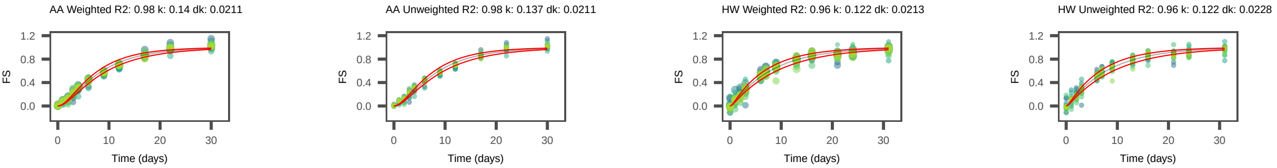

ETFB

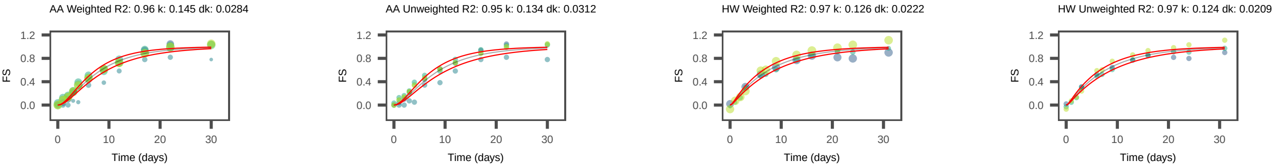

ETFD

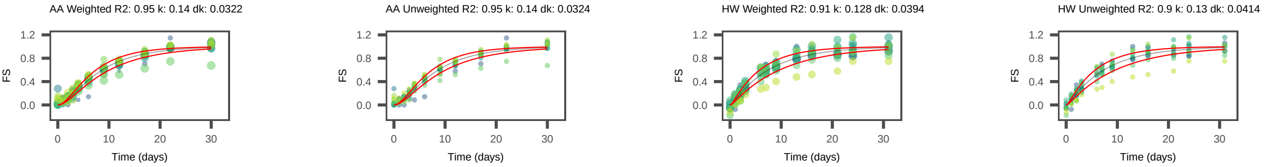

ETHE1

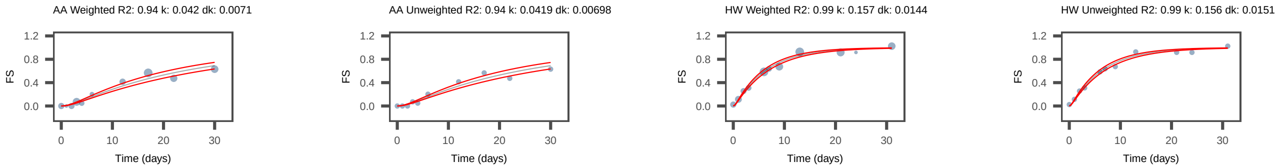

F16P1

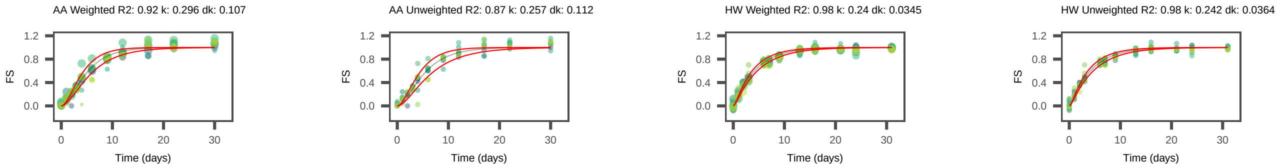

FAAA

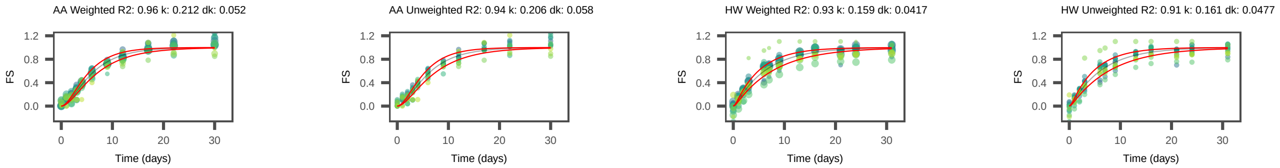

FAAH1

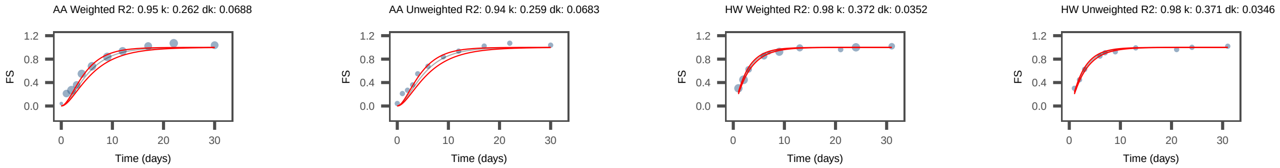

FABPI

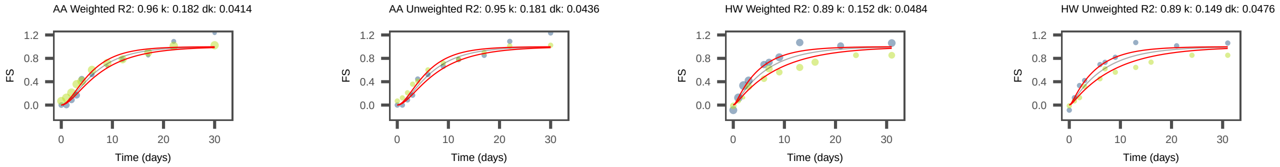

FABPL

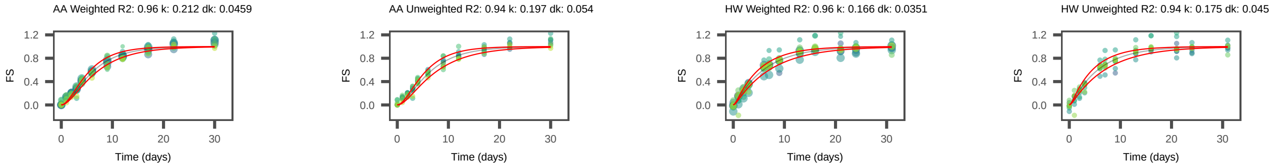

FAHD1

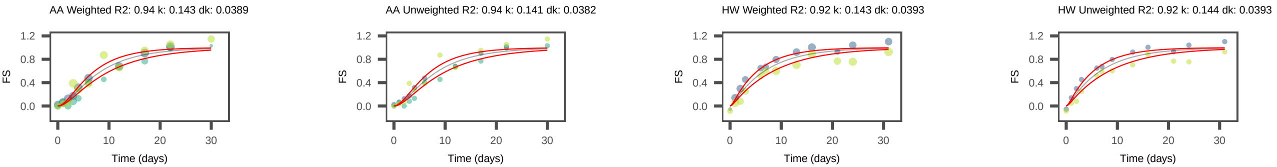

FAS

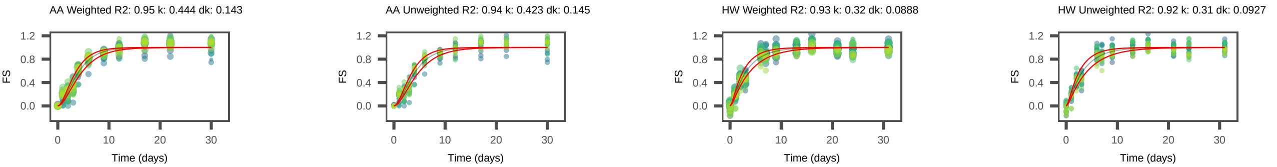

FGGY

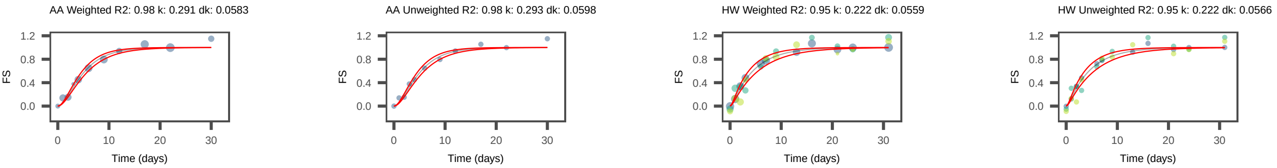

FIBB

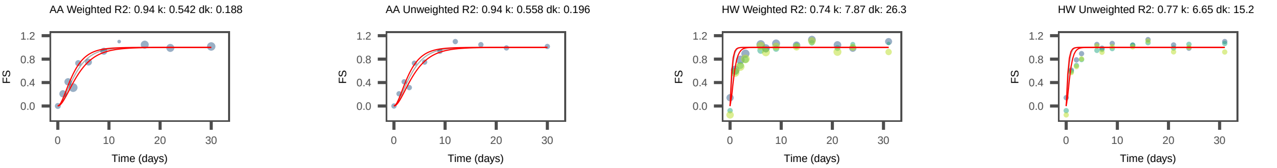

FIS1

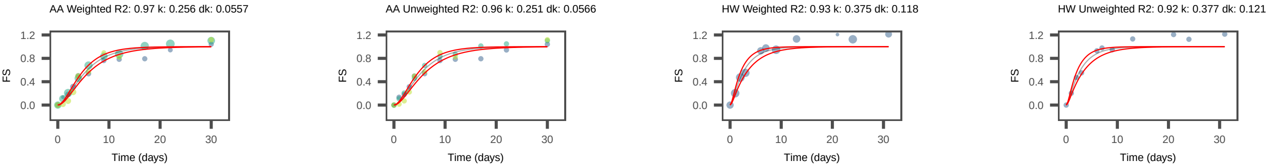

FKBP2

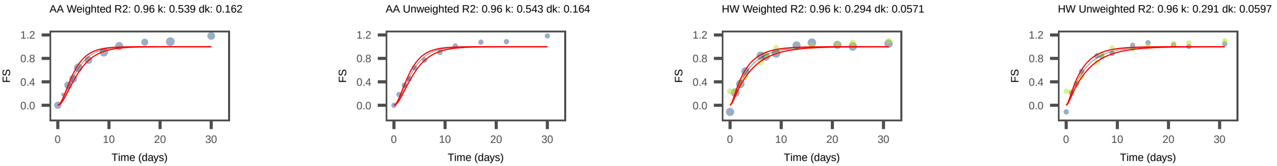

FLNB

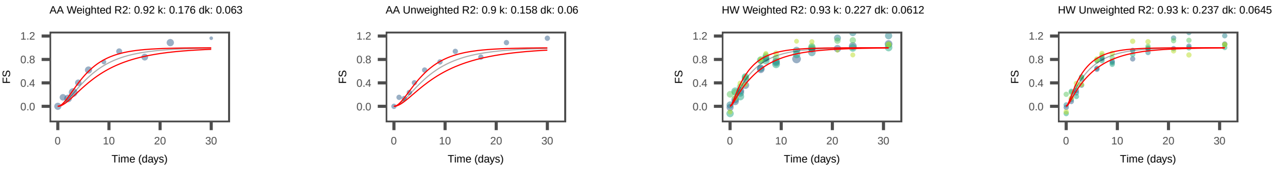

FMO1

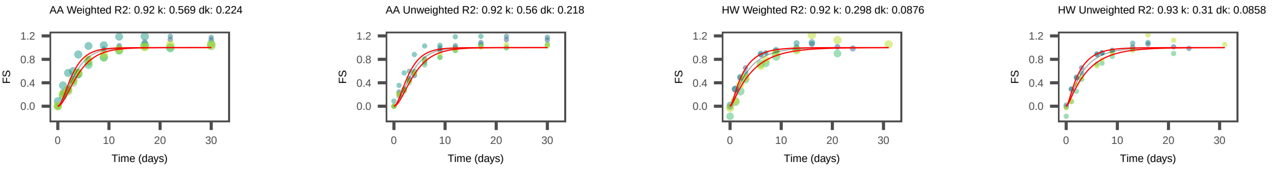

FMO5

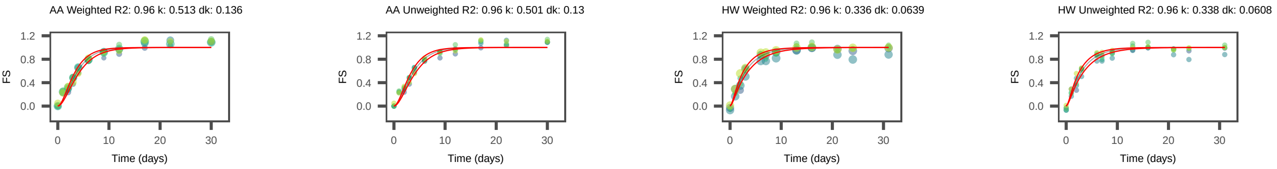

FPPS

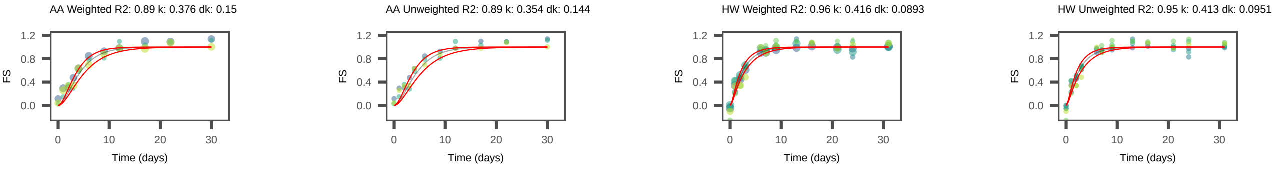

FTCD

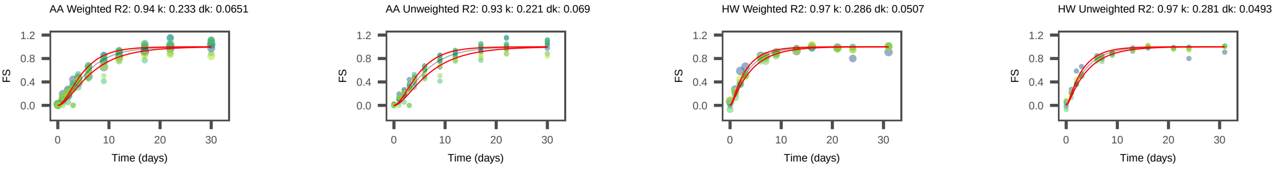

FUMH

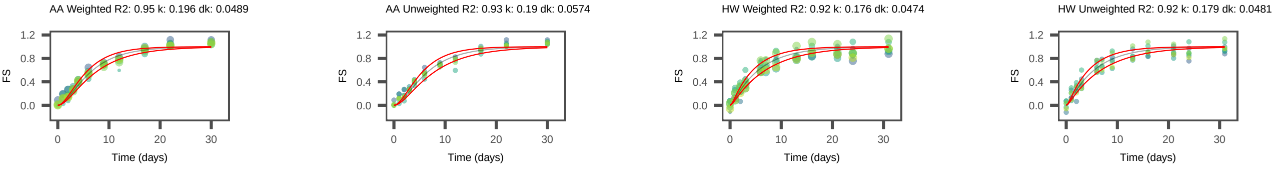

FUS

AA Weighted R2: 0.93 k: 0.301 dk: 0.102

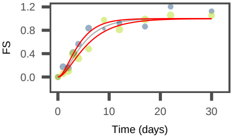

AA Unweighted R2: 0.93 k: 0.303 dk: 0.104

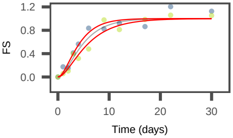

HW Weighted R2: 0.96 k: 0.351 dk: 0.0749

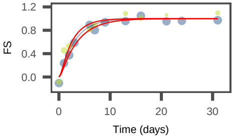

HW Unweighted R2: 0.95 k: 0.365 dk: 0.083

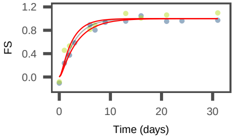

G3P

AA Weighted R2: 0.96 k: 0.247 dk: 0.0576

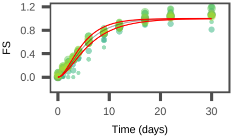

AA Unweighted R2: 0.95 k: 0.229 dk: 0.0618

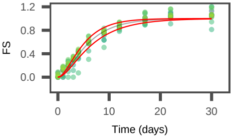

HW Weighted R2: 0.93 k: 0.177 dk: 0.0466

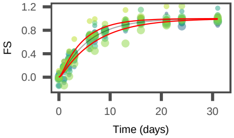

HW Unweighted R2: 0.92 k: 0.185 dk: 0.0527

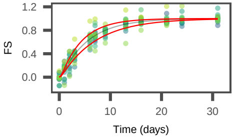

G6PC1

AA Weighted R2: 0.93 k: 0.427 dk: 0.155

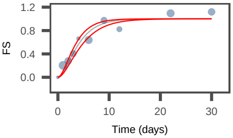

AA Unweighted R2: 0.93 k: 0.438 dk: 0.157

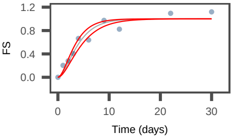

HW Weighted R2: 0.93 k: 0.379 dk: 0.0915

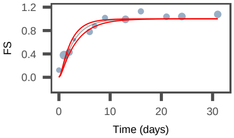

HW Unweighted R2: 0.93 k: 0.374 dk: 0.0891

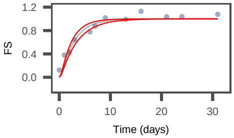

G6PE

AA Weighted R2: 0.94 k: 0.383 dk: 0.134

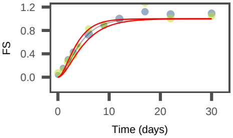

AA Unweighted R2: 0.93 k: 0.369 dk: 0.133

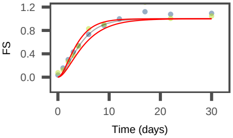

HW Weighted R2: 0.94 k: 0.329 dk: 0.0668

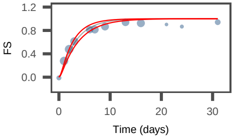

HW Unweighted R2: 0.94 k: 0.327 dk: 0.0671

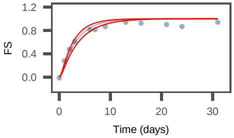

G6PI

AA Weighted R2: 0.96 k: 0.208 dk: 0.05

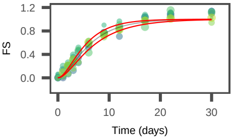

AA Unweighted R2: 0.95 k: 0.21 dk: 0.0536

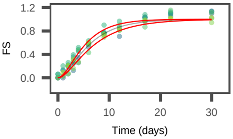

HW Weighted R2: 0.95 k: 0.173 dk: 0.0367

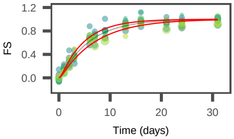

HW Unweighted R2: 0.95 k: 0.181 dk: 0.0378

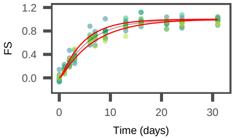

GABT

AA Weighted R2: 0.92 k: 0.229 dk: 0.0755

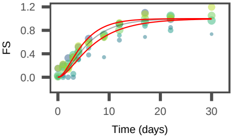

AA Unweighted R2: 0.89 k: 0.201 dk: 0.0773

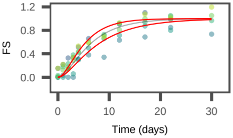

HW Weighted R2: 0.95 k: 0.152 dk: 0.0321

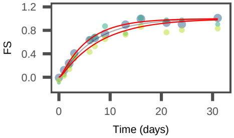

HW Unweighted R2: 0.94 k: 0.146 dk: 0.034

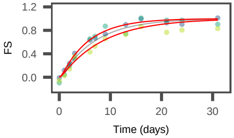

GAL3A

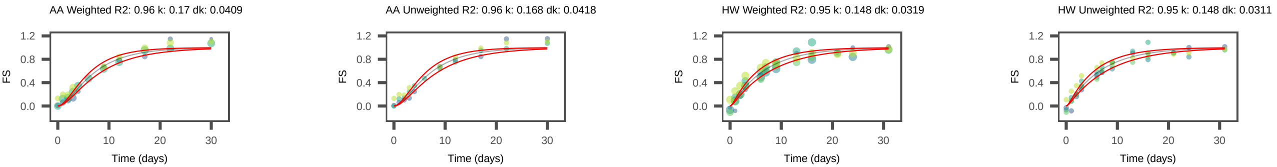

GALK1

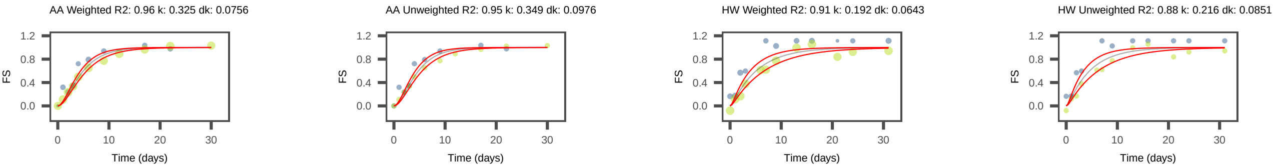

GALM

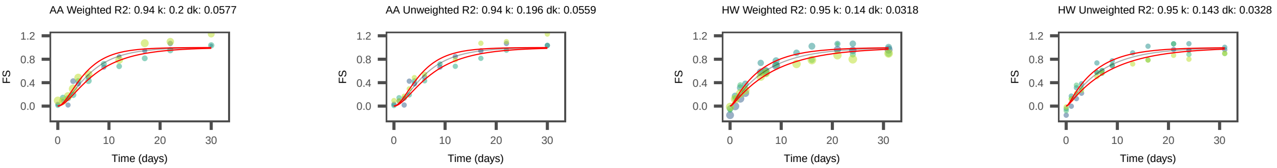

GAMT

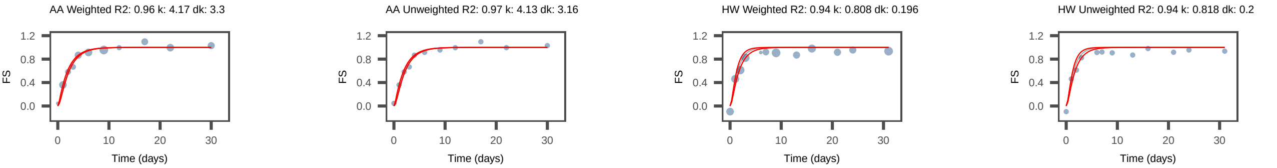

GANAB

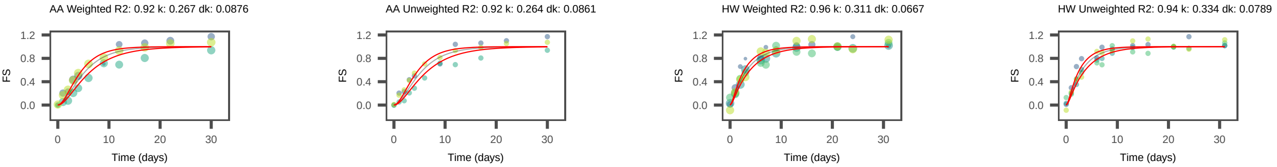

GBB2

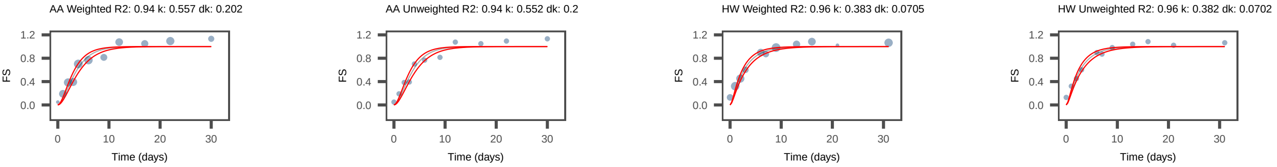

GCDH

AA Weighted R2: 0.93 k: 0.232 dk: 0.0699

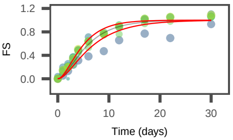

AA Unweighted R2: 0.93 k: 0.232 dk: 0.0687

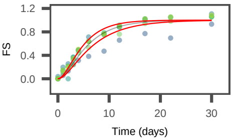

HW Weighted R2: 0.94 k: 0.152 dk: 0.0334

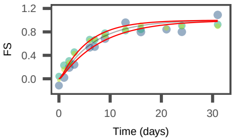

HW Unweighted R2: 0.94 k: 0.154 dk: 0.0331

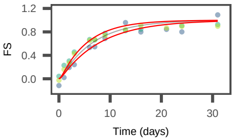

GCKR

AA Weighted R2: 0.96 k: 0.187 dk: 0.0407

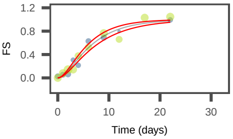

AA Unweighted R2: 0.96 k: 0.185 dk: 0.0408

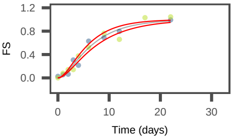

HW Weighted R2: 0.93 k: 0.182 dk: 0.0483

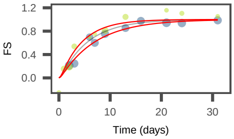

HW Unweighted R2: 0.93 k: 0.188 dk: 0.0529

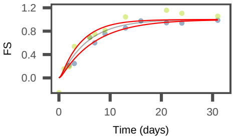

GCSH

AA Weighted R2: 0.98 k: 0.156 dk: 0.0232

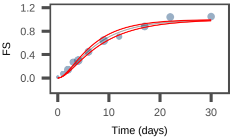

AA Unweighted R2: 0.98 k: 0.156 dk: 0.0232

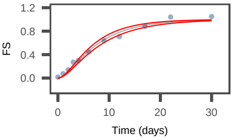

HW Weighted R2: 0.95 k: 0.18 dk: 0.0428

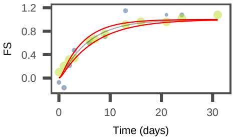

HW Unweighted R2: 0.93 k: 0.182 dk: 0.0519

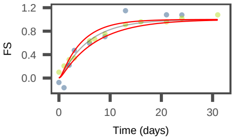

GCSP

AA Weighted R2: 0.96 k: 0.189 dk: 0.0456

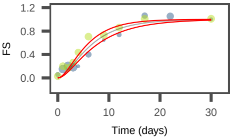

AA Unweighted R2: 0.95 k: 0.18 dk: 0.0443

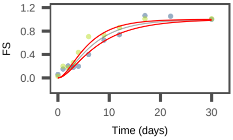

HW Weighted R2: 0.96 k: 0.196 dk: 0.0402

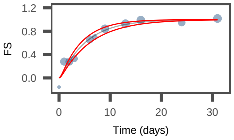

HW Unweighted R2: 0.96 k: 0.194 dk: 0.0411

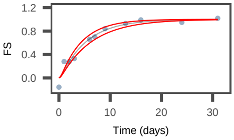

GDIA

AA Weighted R2: 0.98 k: 0.254 dk: 0.0462

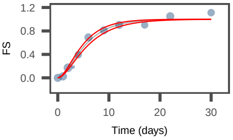

AA Unweighted R2: 0.98 k: 0.248 dk: 0.0472

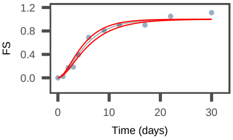

HW Weighted R2: 0.96 k: 0.266 dk: 0.0582

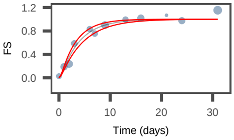

HW Unweighted R2: 0.96 k: 0.267 dk: 0.0576

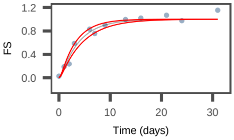

GDIB

AA Weighted R2: 0.82 k: 0.286 dk: 0.145

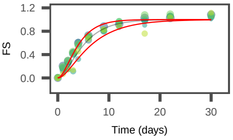

AA Unweighted R2: 0.8 k: 0.265 dk: 0.139

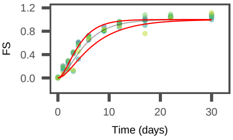

HW Weighted R2: 0.94 k: 0.324 dk: 0.0755

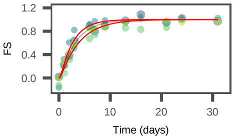

HW Unweighted R2: 0.94 k: 0.327 dk: 0.0772

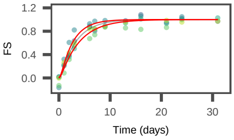

GFRP

AA Weighted R2: 0.94 k: 0.208 dk: 0.0603

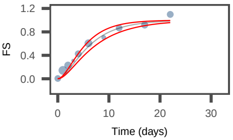

AA Unweighted R2: 0.93 k: 0.192 dk: 0.0598

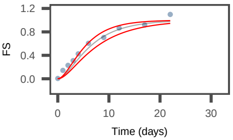

HW Weighted R2: 0.95 k: 0.237 dk: 0.0472

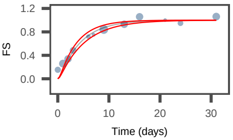

HW Unweighted R2: 0.95 k: 0.237 dk: 0.047

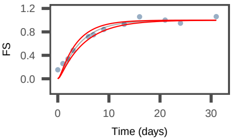

GGLO

AA Weighted R2: 0.95 k: 0.343 dk: 0.102

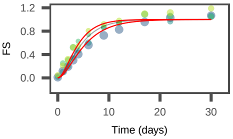

AA Unweighted R2: 0.95 k: 0.366 dk: 0.108

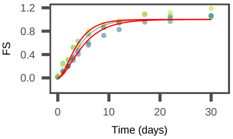

HW Weighted R2: 0.96 k: 0.262 dk: 0.0497

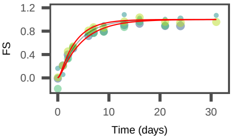

HW Unweighted R2: 0.96 k: 0.262 dk: 0.0506

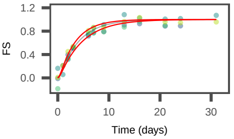

GLCTK

AA Weighted R2: 0.97 k: 0.216 dk: 0.045

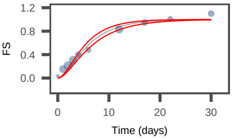

AA Unweighted R2: 0.97 k: 0.212 dk: 0.0438

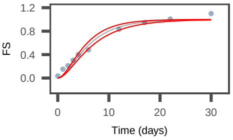

HW Weighted R2: 0.98 k: 0.157 dk: 0.0192

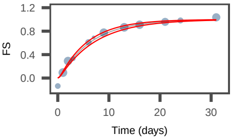

HW Unweighted R2: 0.98 k: 0.159 dk: 0.0197

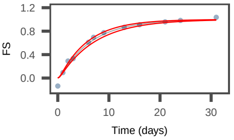

GLGB

AA Weighted R2: 0.93 k: 0.141 dk: 0.039

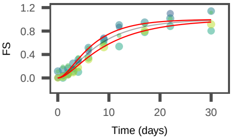

AA Unweighted R2: 0.93 k: 0.144 dk: 0.0402

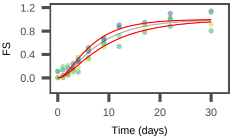

HW Weighted R2: 0.93 k: 0.12 dk: 0.0303

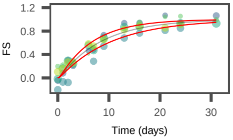

HW Unweighted R2: 0.93 k: 0.121 dk: 0.0315

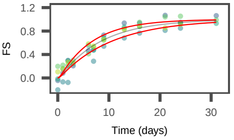

GLNA

AA Weighted R2: 0.96 k: 0.301 dk: 0.0757

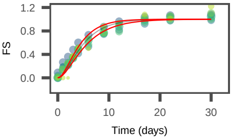

AA Unweighted R2: 0.95 k: 0.296 dk: 0.0772

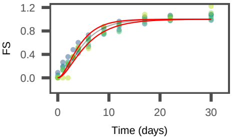

HW Weighted R2: 0.93 k: 0.246 dk: 0.0699

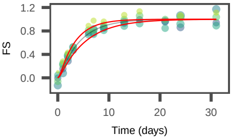

HW Unweighted R2: 0.89 k: 0.232 dk: 0.0788

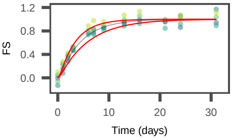

GLO2

AA Weighted R2: 0.9 k: 0.284 dk: 0.106

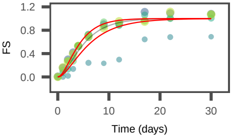

AA Unweighted R2: 0.84 k: 0.245 dk: 0.114

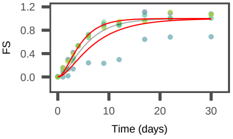

HW Weighted R2: 0.94 k: 0.32 dk: 0.0763

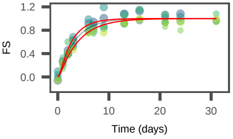

HW Unweighted R2: 0.94 k: 0.312 dk: 0.0746

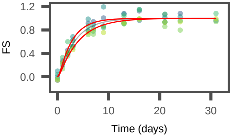

GLOD4

AA Weighted R2: 0.97 k: 0.169 dk: 0.0293

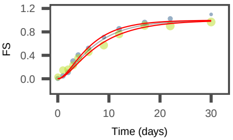

AA Unweighted R2: 0.97 k: 0.178 dk: 0.0313

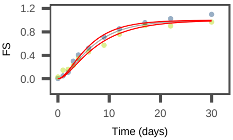

HW Weighted R2: 0.94 k: 0.27 dk: 0.0715

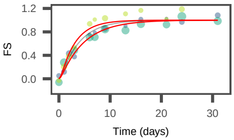

HW Unweighted R2: 0.94 k: 0.275 dk: 0.0729

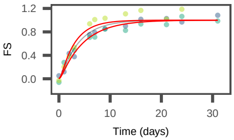

GLPK

AA Weighted R2: 0.93 k: 0.232 dk: 0.076

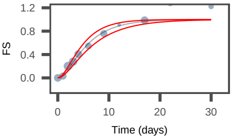

AA Unweighted R2: 0.92 k: 0.229 dk: 0.0788

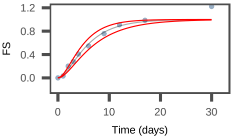

HW Weighted R2: 0.94 k: 0.194 dk: 0.0503

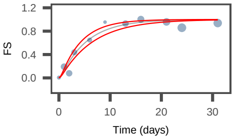

HW Unweighted R2: 0.94 k: 0.199 dk: 0.0514

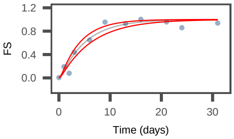

GLSL

AA Weighted R2: 0.93 k: 0.281 dk: 0.0936

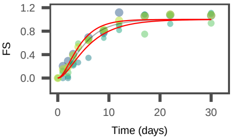

AA Unweighted R2: 0.93 k: 0.268 dk: 0.0898

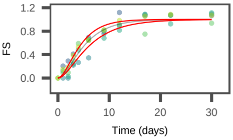

HW Weighted R2: 0.97 k: 0.234 dk: 0.0381

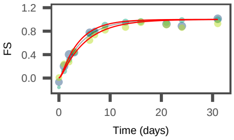

HW Unweighted R2: 0.97 k: 0.234 dk: 0.0382

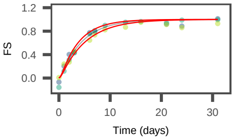

GLU2B

AA Weighted R2: 0.96 k: 0.298 dk: 0.0703

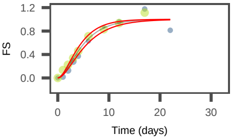

AA Unweighted R2: 0.96 k: 0.293 dk: 0.0735

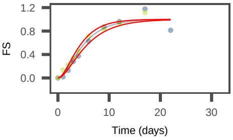

HW Weighted R2: 0.95 k: 0.287 dk: 0.0593

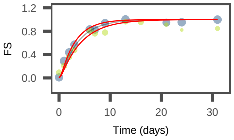

HW Unweighted R2: 0.94 k: 0.281 dk: 0.0613

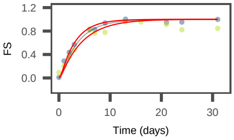

GLUCM

AA Weighted R2: 0.93 k: 0.0659 dk: 0.0116

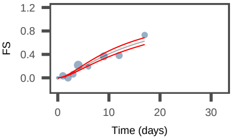

AA Unweighted R2: 0.93 k: 0.0659 dk: 0.0116

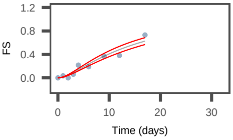

HW Weighted R2: 0.92 k: 0.169 dk: 0.0522

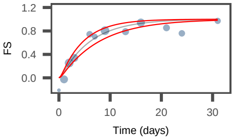

HW Unweighted R2: 0.91 k: 0.169 dk: 0.0546

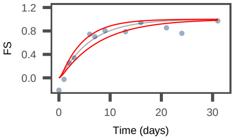

GLYAL

AA Weighted R2: 0.88 k: 0.131 dk: 0.0478

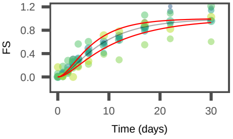

AA Unweighted R2: 0.87 k: 0.131 dk: 0.0491

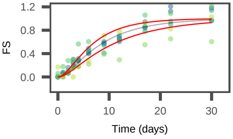

HW Weighted R2: 0.96 k: 0.142 dk: 0.0252

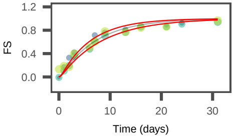

HW Unweighted R2: 0.96 k: 0.142 dk: 0.0254

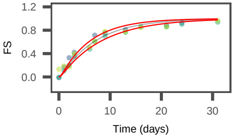

GLYAT

AA Weighted R2: 0.93 k: 0.162 dk: 0.0451 | AA Unweighted R2: 0.92 k: 0.152 dk: 0.0456 | HW Weighted R2: 0.91 k: 0.119 dk: 0.0324 | HW Unweighted R2: 0.9 k: 0.12 dk: 0.0345

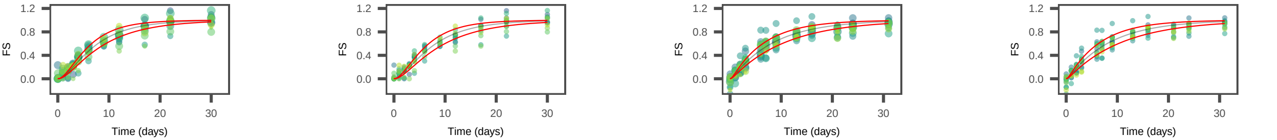

GLYC

AA Weighted R2: 0.96 k: 0.249 dk: 0.0596 | AA Unweighted R2: 0.95 k: 0.248 dk: 0.0614 | HW Weighted R2: 0.95 k: 0.254 dk: 0.0576 | HW Unweighted R2: 0.95 k: 0.25 dk: 0.0575

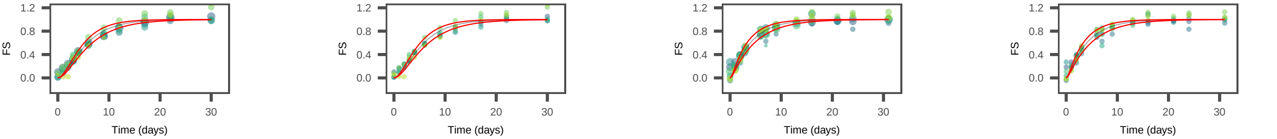

GLYM

AA Weighted R2: 0.87 k: 0.332 dk: 0.147 | AA Unweighted R2: 0.86 k: 0.337 dk: 0.156 | HW Weighted R2: 0.95 k: 0.358 dk: 0.0788 | HW Unweighted R2: 0.94 k: 0.366 dk: 0.0844

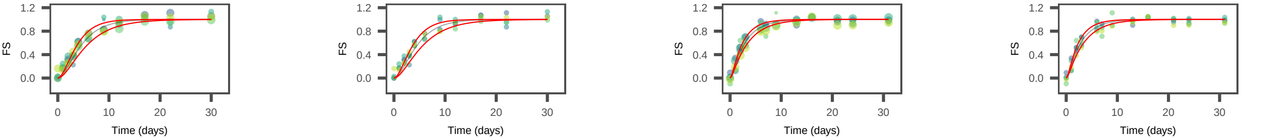

GNMT

AA Weighted R2: 0.94 k: 0.347 dk: 0.11 | AA Unweighted R2: 0.94 k: 0.337 dk: 0.108 | HW Weighted R2: 0.94 k: 0.189 dk: 0.0436 | HW Unweighted R2: 0.92 k: 0.195 dk: 0.0539

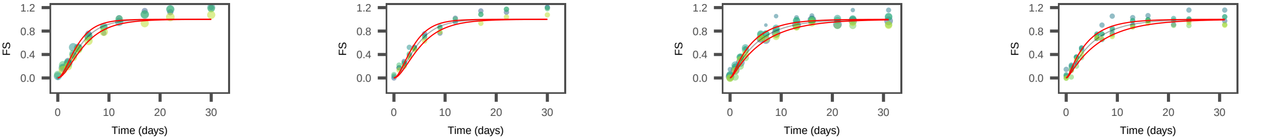

GPDA

AA Weighted R2: 0.97 k: 0.278 dk: 0.0553 | AA Unweighted R2: 0.97 k: 0.28 dk: 0.0581 | HW Weighted R2: 0.95 k: 0.175 dk: 0.0358 | HW Unweighted R2: 0.95 k: 0.179 dk: 0.0365

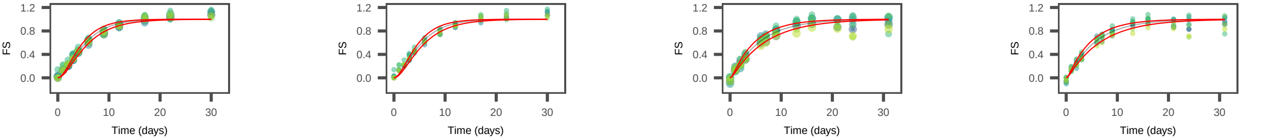

GPDM

AA Weighted R2: 0.96 k: 0.128 dk: 0.0249 | AA Unweighted R2: 0.96 k: 0.123 dk: 0.0237 | HW Weighted R2: 0.95 k: 0.138 dk: 0.0312 | HW Unweighted R2: 0.95 k: 0.142 dk: 0.0329

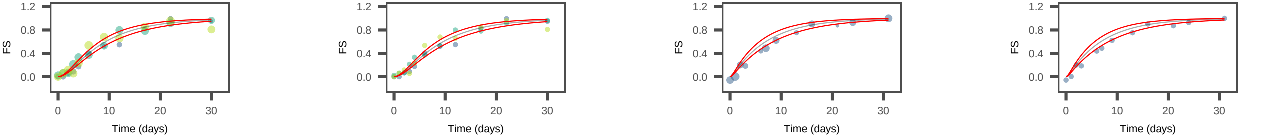

GPX1

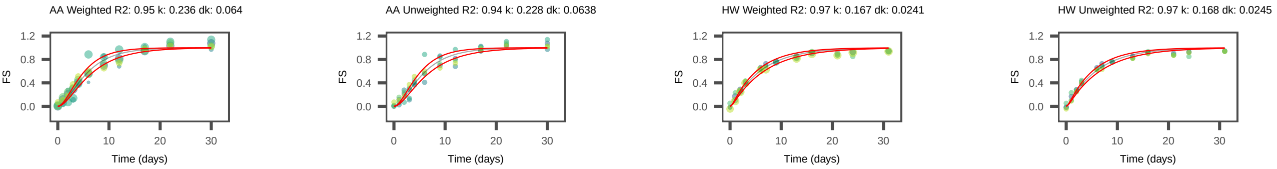

GRHPR

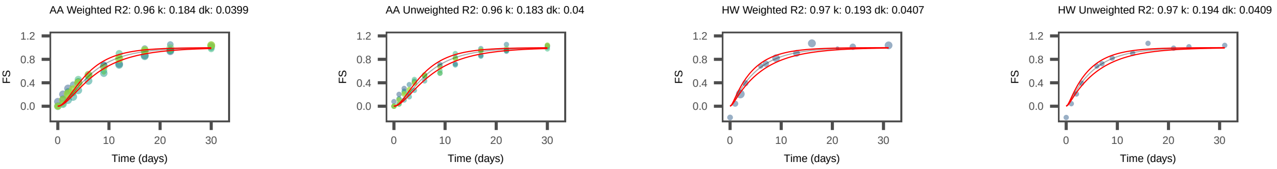

GRP75

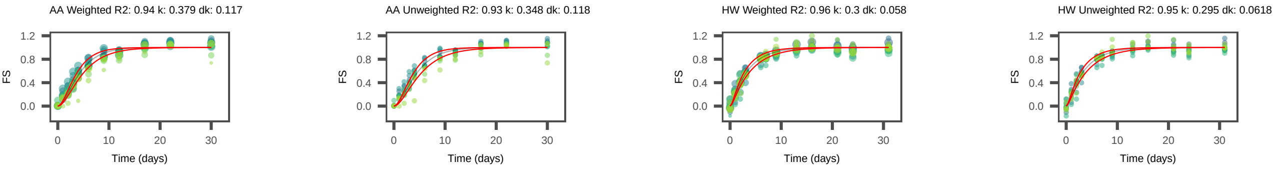

GRPE1

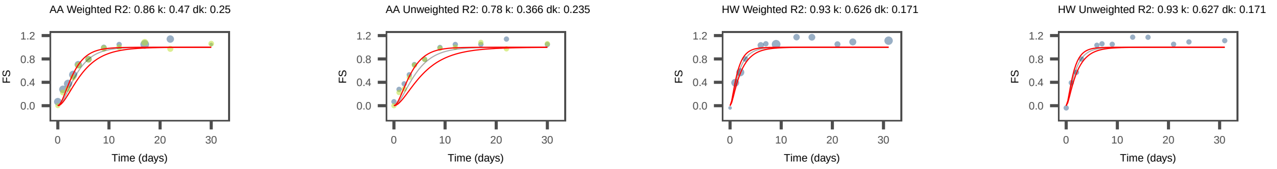

GSH0

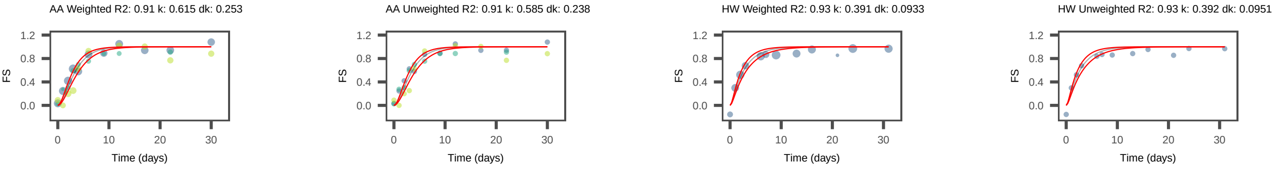

GSH1

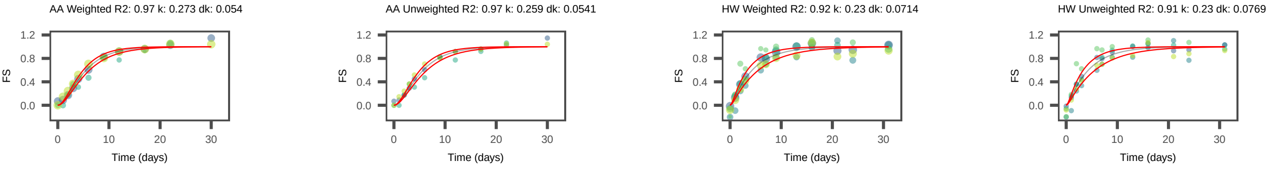

GSHR

AA Weighted R2: 0.95 k: 0.237 dk: 0.0579

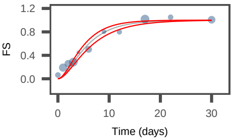

AA Unweighted R2: 0.95 k: 0.236 dk: 0.0574

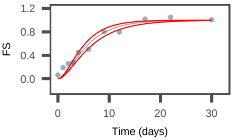

HW Weighted R2: 0.97 k: 0.185 dk: 0.0355

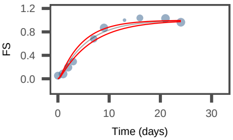

HW Unweighted R2: 0.97 k: 0.186 dk: 0.0368

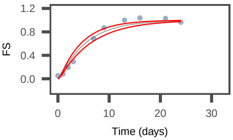

GSTA3

AA Weighted R2: 0.99 k: 0.22 dk: 0.0309

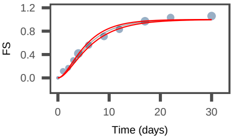

AA Unweighted R2: 0.99 k: 0.22 dk: 0.0309

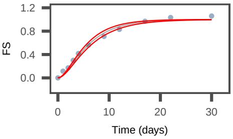

HW Weighted R2: 0.96 k: 0.162 dk: 0.0346

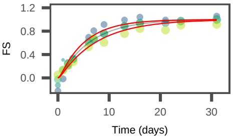

HW Unweighted R2: 0.94 k: 0.178 dk: 0.0455

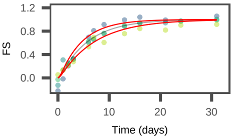

GSTA4

AA Weighted R2: 0.95 k: 0.207 dk: 0.0515

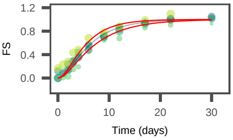

AA Unweighted R2: 0.95 k: 0.199 dk: 0.0499

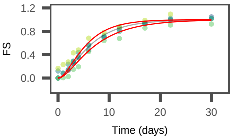

HW Weighted R2: 0.85 k: 0.119 dk: 0.0426

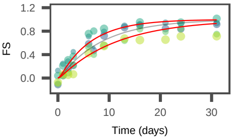

HW Unweighted R2: 0.85 k: 0.122 dk: 0.0428

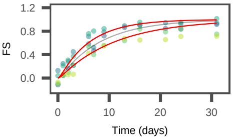

GSTK1

AA Weighted R2: 0.92 k: 0.243 dk: 0.0735

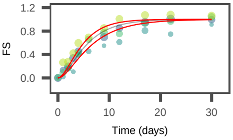

AA Unweighted R2: 0.91 k: 0.226 dk: 0.0719

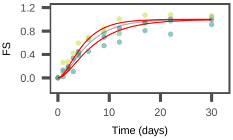

HW Weighted R2: 0.96 k: 0.192 dk: 0.0395

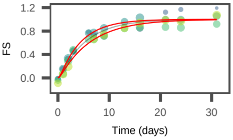

HW Unweighted R2: 0.96 k: 0.195 dk: 0.0418

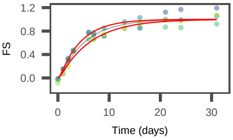

GSTM1

AA Weighted R2: 0.97 k: 0.227 dk: 0.0488

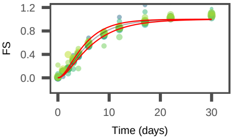

AA Unweighted R2: 0.95 k: 0.229 dk: 0.06

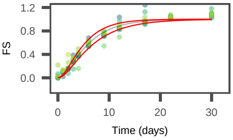

HW Weighted R2: 0.95 k: 0.173 dk: 0.0427

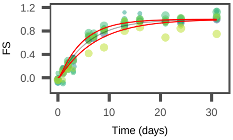

HW Unweighted R2: 0.94 k: 0.174 dk: 0.0433

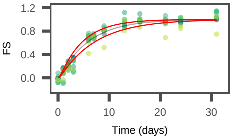

GSTM2

AA Weighted R2: 0.91 k: 0.194 dk: 0.0592

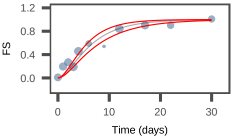

AA Unweighted R2: 0.91 k: 0.188 dk: 0.0577

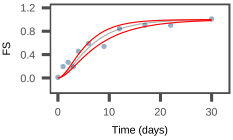

HW Weighted R2: 0.95 k: 0.15 dk: 0.0367

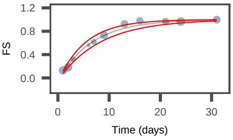

HW Unweighted R2: 0.94 k: 0.145 dk: 0.0379

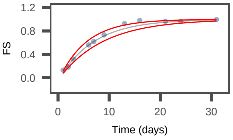

GSTM3

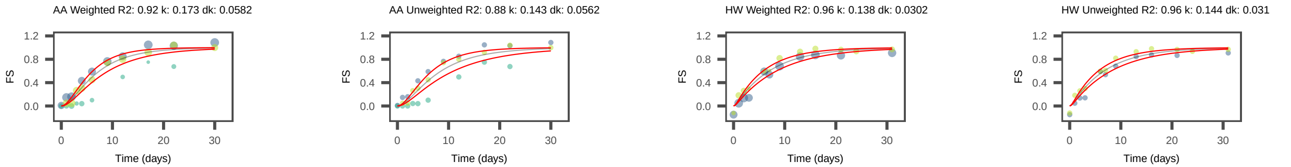

GSTM6

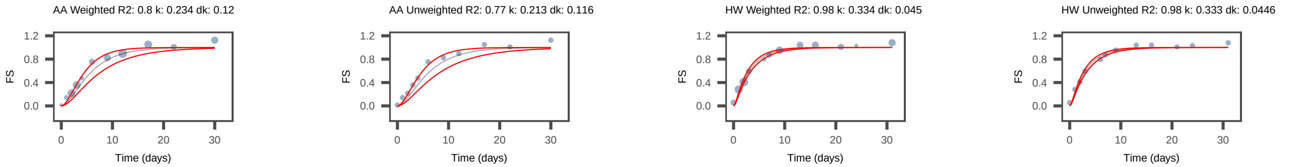

GSTM7

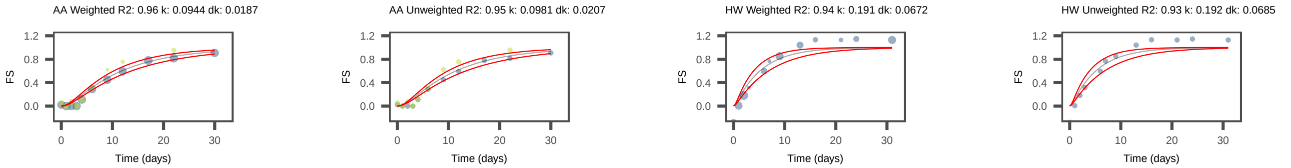

GSTT2

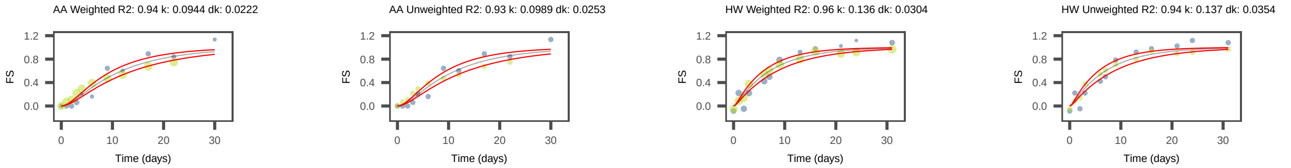

GYS2

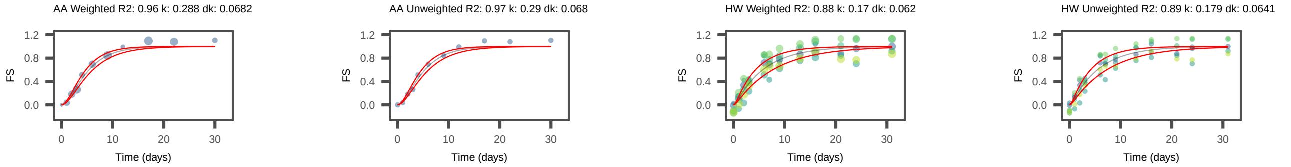

H14

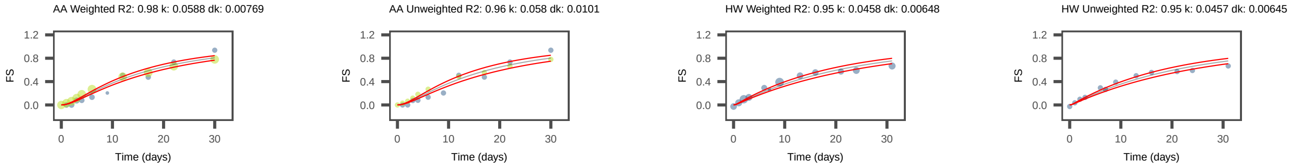

H2AY

AA Weighted R2: 0.97 k: 0.0997 dk: 0.0134

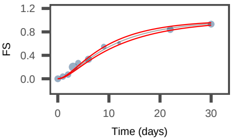

AA Unweighted R2: 0.97 k: 0.0975 dk: 0.0136

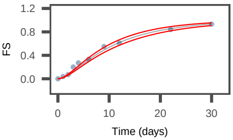

HW Weighted R2: 0.94 k: 0.1 dk: 0.0231

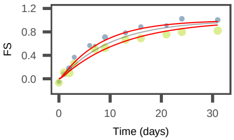

HW Unweighted R2: 0.94 k: 0.109 dk: 0.0247

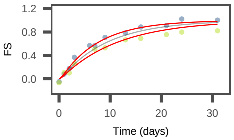

H4

AA Weighted R2: 0.9 k: 0.0146 dk: 0.0017

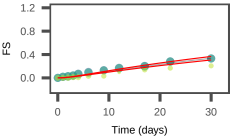

AA Unweighted R2: 0.87 k: 0.0134 dk: 0.00189

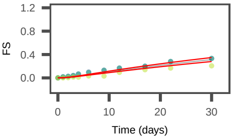

HW Weighted R2: 0.87 k: 0.0218 dk: 0.00391

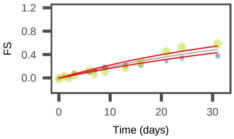

HW Unweighted R2: 0.88 k: 0.0205 dk: 0.00359

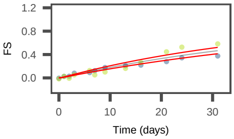

HACL1

AA Weighted R2: 0.94 k: 0.459 dk: 0.15

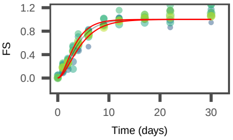

AA Unweighted R2: 0.94 k: 0.451 dk: 0.152

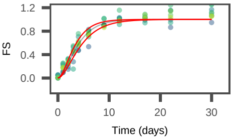

HW Weighted R2: 0.97 k: 0.295 dk: 0.0505

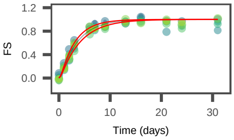

HW Unweighted R2: 0.97 k: 0.298 dk: 0.0504

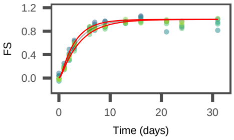

HBA

AA Weighted R2: 0.95 k: 0.0277 dk: 0.00305

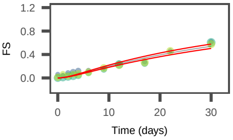

AA Unweighted R2: 0.95 k: 0.0274 dk: 0.0032

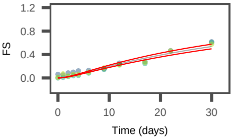

HW Weighted R2: 0.92 k: 0.0214 dk: 0.00294

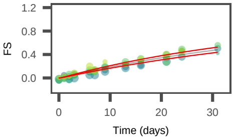

HW Unweighted R2: 0.92 k: 0.0217 dk: 0.00299

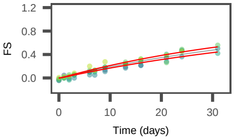

HBB1

AA Weighted R2: 0.98 k: 0.0269 dk: 0.00203

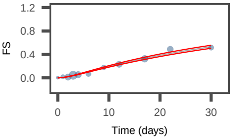

AA Unweighted R2: 0.98 k: 0.0268 dk: 0.00204

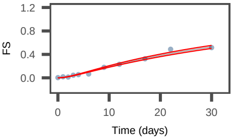

HW Weighted R2: 0.91 k: 0.0242 dk: 0.00339

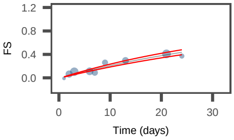

HW Unweighted R2: 0.9 k: 0.0239 dk: 0.00346

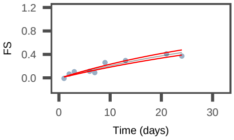

HCD2

AA Weighted R2: 0.97 k: 0.145 dk: 0.0262

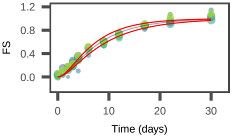

AA Unweighted R2: 0.97 k: 0.141 dk: 0.027

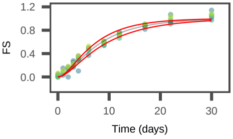

HW Weighted R2: 0.97 k: 0.11 dk: 0.0152

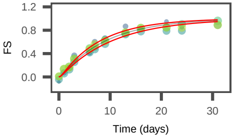

HW Unweighted R2: 0.97 k: 0.11 dk: 0.0159

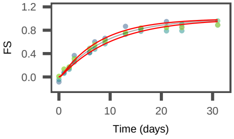

HCDH

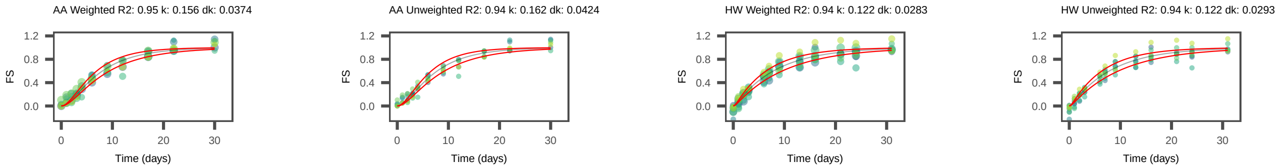

HDHD2

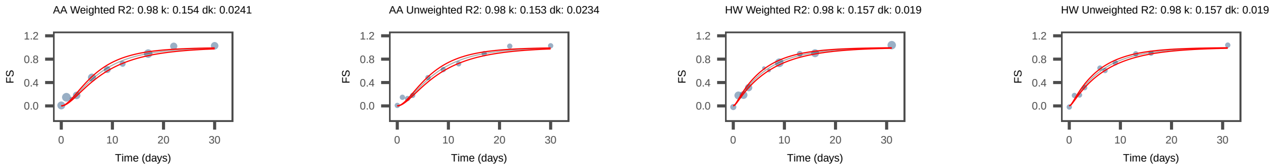

HEBP1

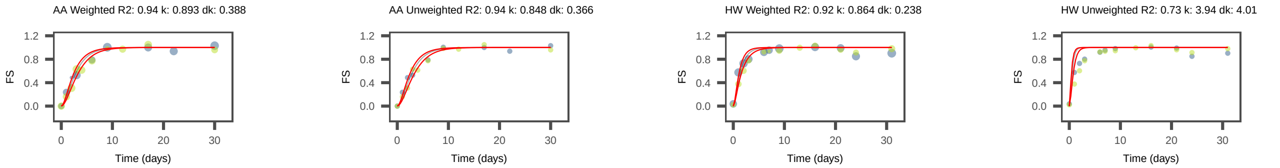

HEM2

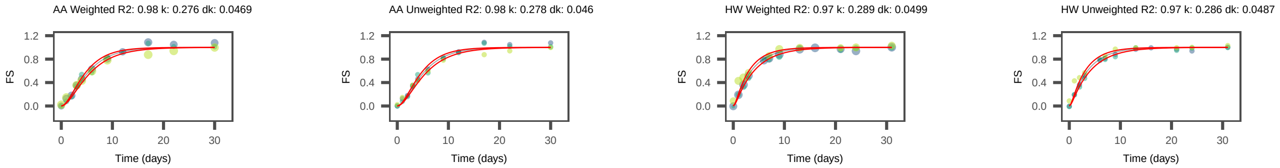

HEM6

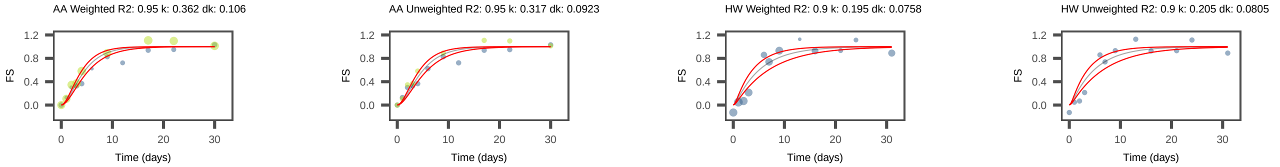

HEMH

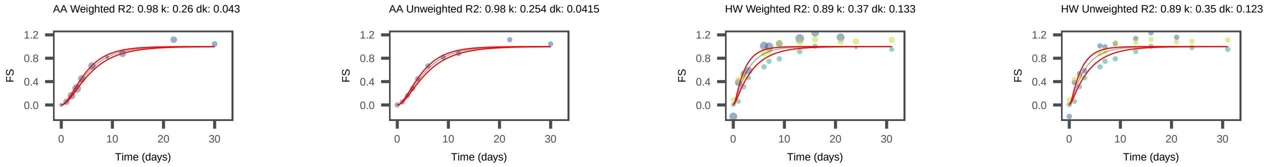

HGD

AA Weighted R2: 0.96 k: 0.359 dk: 0.0951 | AA Unweighted R2: 0.95 k: 0.349 dk: 0.0934 | HW Weighted R2: 0.92 k: 0.228 dk: 0.0681 | HW Unweighted R2: 0.9 k: 0.232 dk: 0.0776

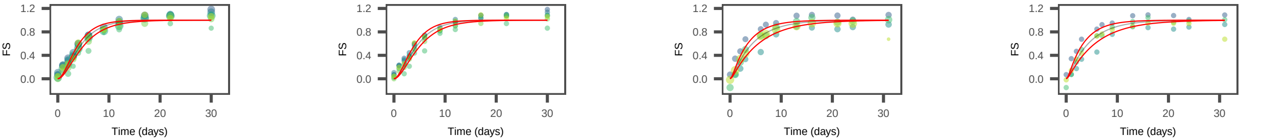

HIBCH

AA Weighted R2: 0.92 k: 0.152 dk: 0.044 | AA Unweighted R2: 0.91 k: 0.149 dk: 0.0459 | HW Weighted R2: 0.85 k: 0.106 dk: 0.0347 | HW Unweighted R2: 0.85 k: 0.103 dk: 0.0337

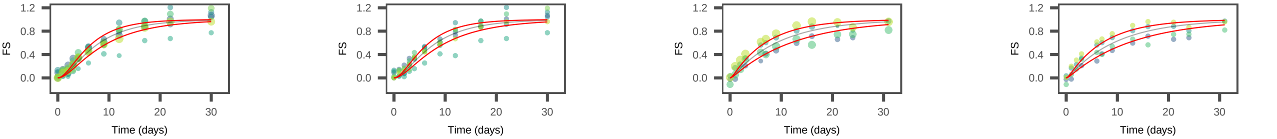

HINT1

AA Weighted R2: 0.95 k: 0.205 dk: 0.0534 | AA Unweighted R2: 0.95 k: 0.209 dk: 0.0546 | HW Weighted R2: 0.93 k: 0.173 dk: 0.0428 | HW Unweighted R2: 0.92 k: 0.173 dk: 0.0465

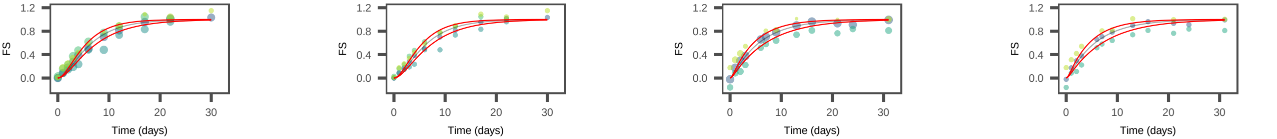

HIUH

AA Weighted R2: 0.94 k: 0.289 dk: 0.0927 | AA Unweighted R2: 0.95 k: 0.274 dk: 0.0841 | HW Weighted R2: 0.98 k: 0.302 dk: 0.0381 | HW Unweighted R2: 0.98 k: 0.303 dk: 0.0383

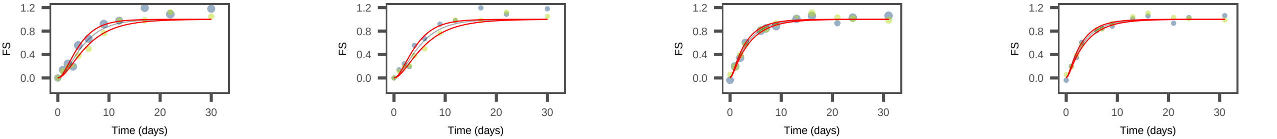

HMCS2

AA Weighted R2: 0.95 k: 0.319 dk: 0.0831 | AA Unweighted R2: 0.95 k: 0.315 dk: 0.0851 | HW Weighted R2: 0.95 k: 0.229 dk: 0.0473 | HW Unweighted R2: 0.95 k: 0.233 dk: 0.0474

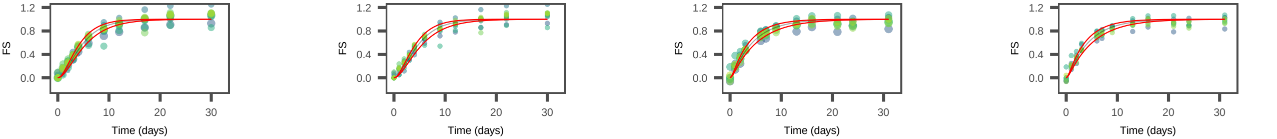

HMGCL

AA Weighted R2: 0.86 k: 0.16 dk: 0.0631 | AA Unweighted R2: 0.84 k: 0.154 dk: 0.0637 | HW Weighted R2: 0.96 k: 0.132 dk: 0.0242 | HW Unweighted R2: 0.96 k: 0.13 dk: 0.0254

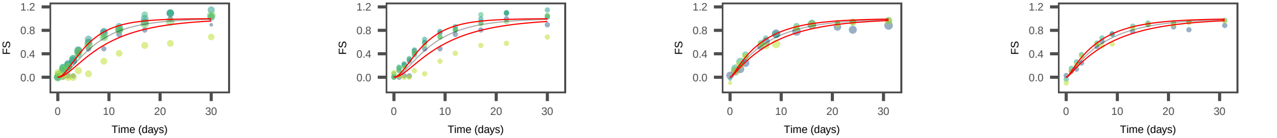

HNRPK

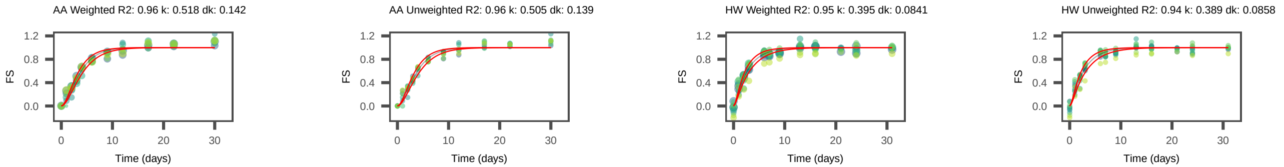

HNRPL

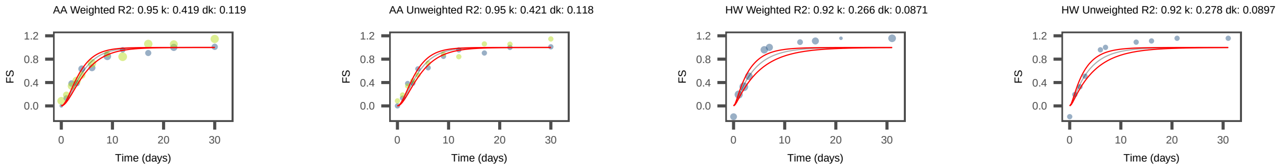

HNRPM

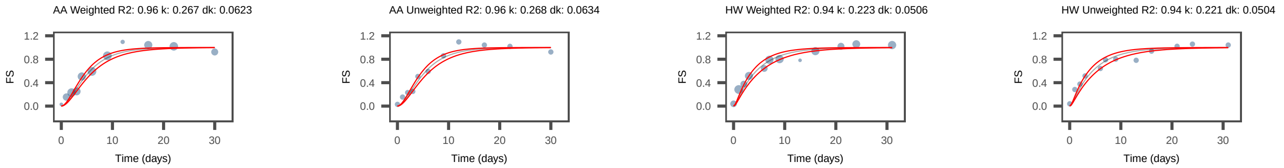

HNRPQ

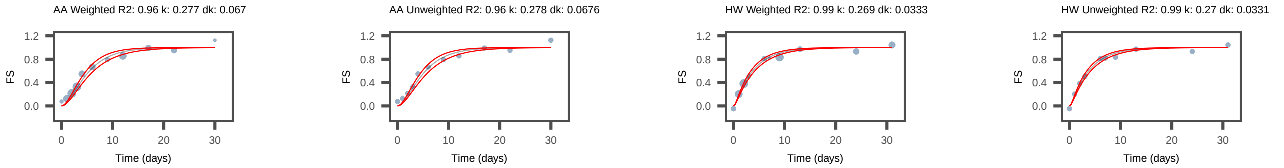

HNRPU

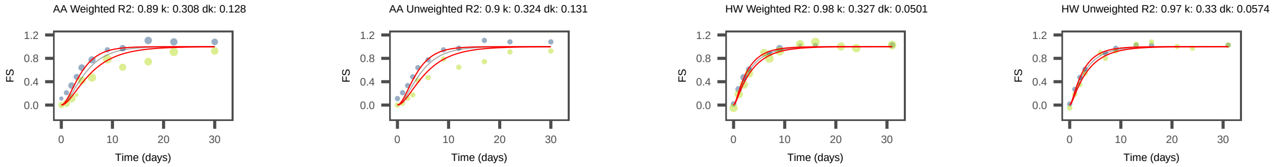

HOGA1

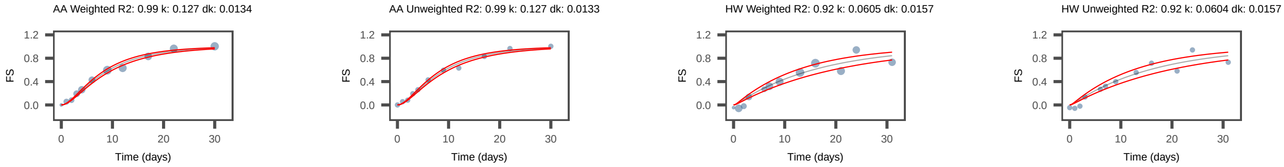

HPPD

AA Weighted R2: 0.83 k: 11.2 dk: 53.9

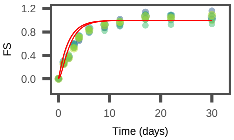

AA Unweighted R2: 0.93 k: 0.715 dk: 0.269

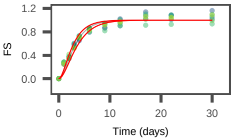

HW Weighted R2: 0.95 k: 0.506 dk: 0.0969

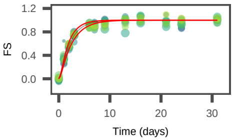

HW Unweighted R2: 0.95 k: 0.503 dk: 0.103

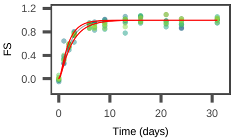

HPRT

AA Weighted R2: 0.98 k: 0.191 dk: 0.0308

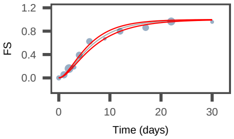

AA Unweighted R2: 0.98 k: 0.189 dk: 0.0306

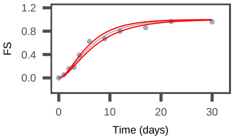

HW Weighted R2: 0.91 k: 0.286 dk: 0.0906

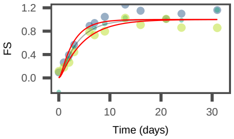

HW Unweighted R2: 0.91 k: 0.29 dk: 0.0907

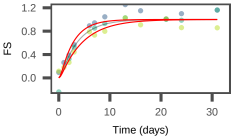

HS90A

AA Weighted R2: 0.94 k: 0.359 dk: 0.118

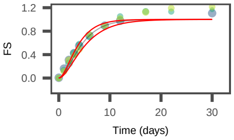

AA Unweighted R2: 0.94 k: 0.346 dk: 0.116

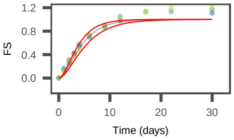

HW Weighted R2: 0.92 k: 0.243 dk: 0.0672

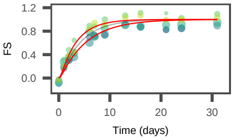

HW Unweighted R2: 0.92 k: 0.25 dk: 0.0703

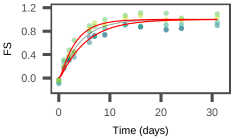

HS90B

AA Weighted R2: 0.95 k: 0.632 dk: 0.221

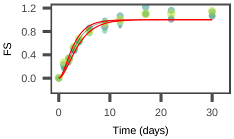

AA Unweighted R2: 0.95 k: 0.633 dk: 0.227

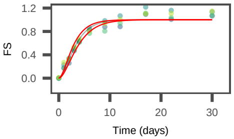

HW Weighted R2: 0.9 k: 0.382 dk: 0.11

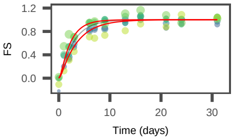

HW Unweighted R2: 0.9 k: 0.374 dk: 0.104

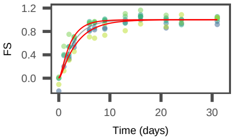

HSDL2

AA Weighted R2: 0.96 k: 0.231 dk: 0.0545

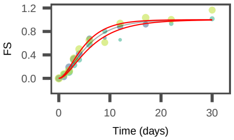

AA Unweighted R2: 0.95 k: 0.224 dk: 0.0544

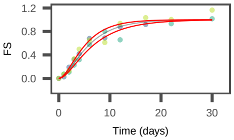

HW Weighted R2: 0.91 k: 0.301 dk: 0.0797

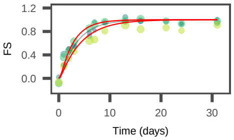

HW Unweighted R2: 0.91 k: 0.302 dk: 0.078

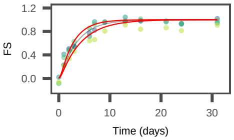

HSP74

AA Weighted R2: 0.93 k: 0.273 dk: 0.0871

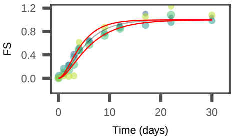

AA Unweighted R2: 0.92 k: 0.26 dk: 0.0877

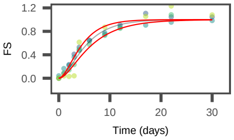

HW Weighted R2: 0.92 k: 0.182 dk: 0.0565

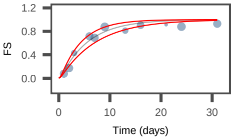

HW Unweighted R2: 0.92 k: 0.182 dk: 0.0572

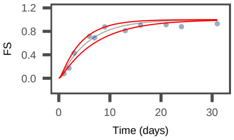

HSP7C

AA Weighted R2: 0.94 k: 0.632 dk: 0.233

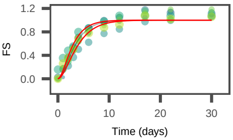

AA Unweighted R2: 0.94 k: 0.616 dk: 0.222

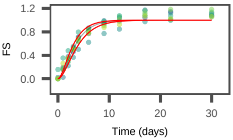

HW Weighted R2: 0.9 k: 0.393 dk: 0.11

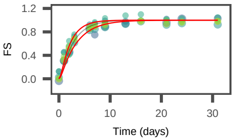

HW Unweighted R2: 0.91 k: 0.404 dk: 0.11

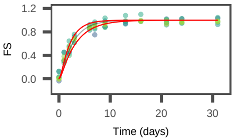

HUTH

AA Weighted R2: 0.9 k: 0.222 dk: 0.0783

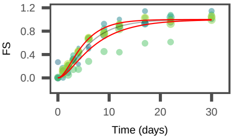

AA Unweighted R2: 0.9 k: 0.222 dk: 0.079

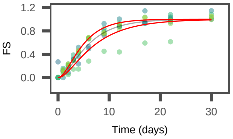

HW Weighted R2: 0.95 k: 0.3 dk: 0.0694

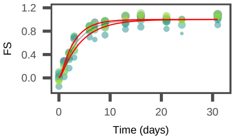

HW Unweighted R2: 0.94 k: 0.292 dk: 0.0712

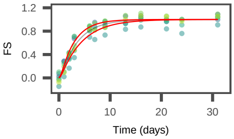

HUTI

AA Weighted R2: 0.93 k: 0.217 dk: 0.0662

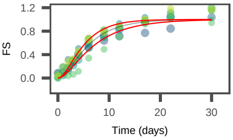

AA Unweighted R2: 0.93 k: 0.218 dk: 0.0676

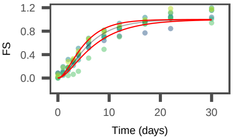

HW Weighted R2: 0.96 k: 0.195 dk: 0.0388

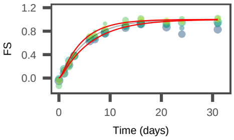

HW Unweighted R2: 0.96 k: 0.195 dk: 0.0382

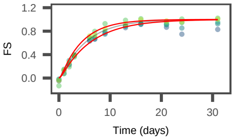

HUTU

AA Weighted R2: 0.95 k: 0.24 dk: 0.0639

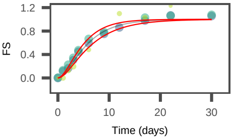

AA Unweighted R2: 0.93 k: 0.262 dk: 0.0845

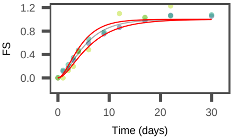

HW Weighted R2: 0.93 k: 0.194 dk: 0.05

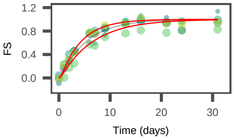

HW Unweighted R2: 0.93 k: 0.192 dk: 0.0498

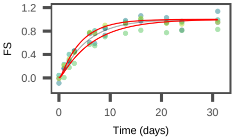

HYEP

AA Weighted R2: 0.96 k: 0.456 dk: 0.114

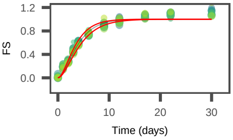

AA Unweighted R2: 0.96 k: 0.447 dk: 0.122

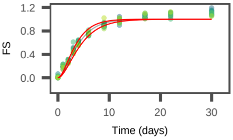

HW Weighted R2: 0.95 k: 0.292 dk: 0.0603

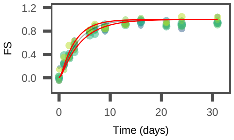

HW Unweighted R2: 0.95 k: 0.295 dk: 0.0612

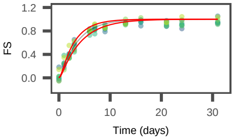

HYES

AA Weighted R2: 0.96 k: 0.408 dk: 0.112

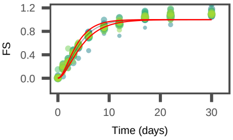

AA Unweighted R2: 0.95 k: 0.406 dk: 0.121

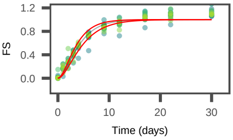

HW Weighted R2: 0.96 k: 0.286 dk: 0.0626

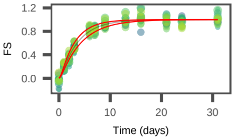

HW Unweighted R2: 0.95 k: 0.279 dk: 0.0657

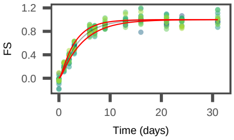

HYOU1

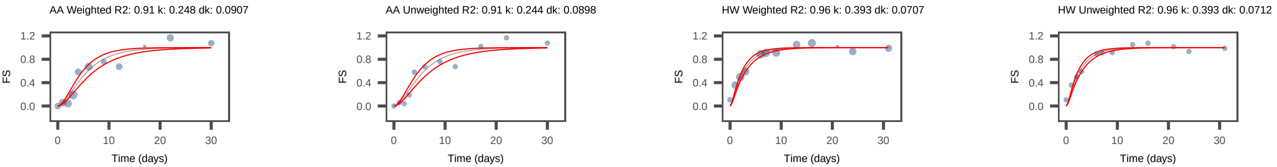

IAH1

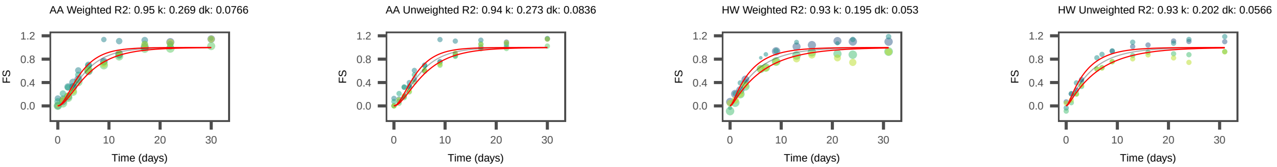

IDH3A

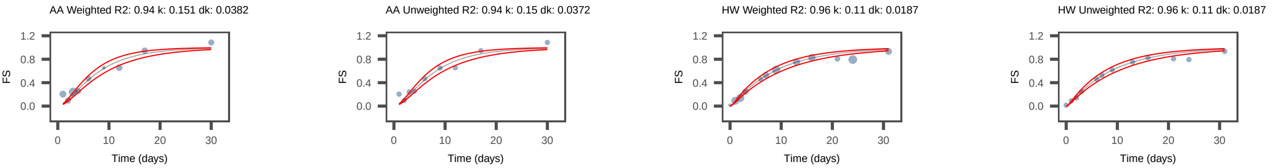

IDHC

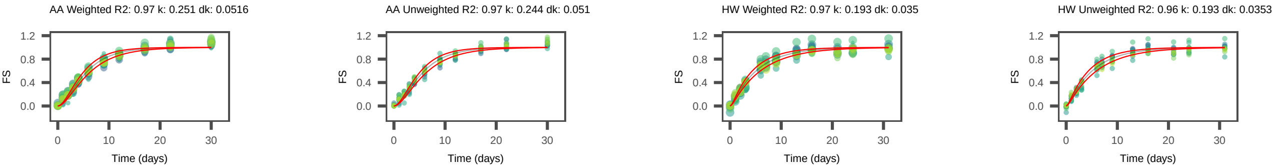

IDHP

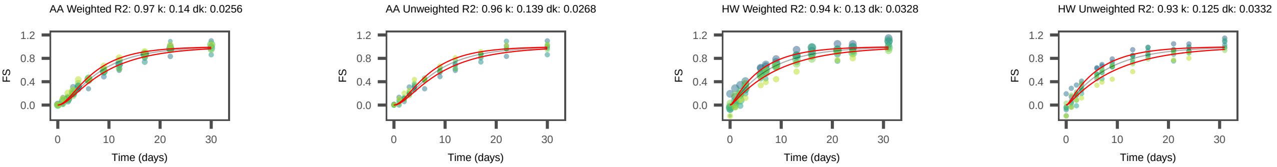

IF2A

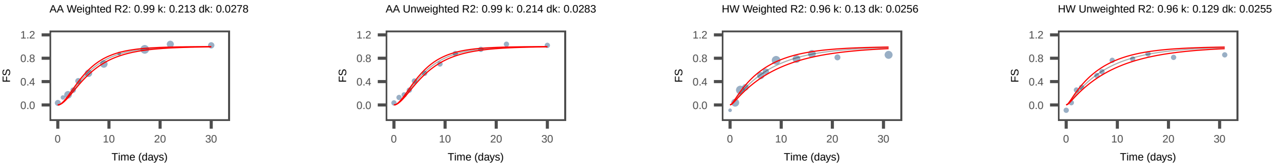

IF4A1

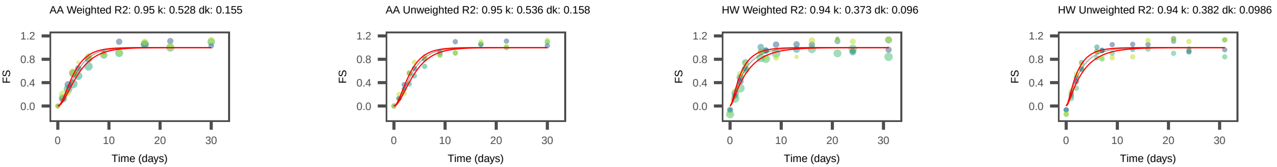

IF4G1

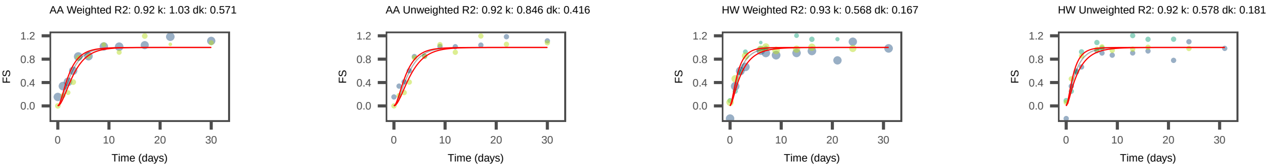

IF4G2

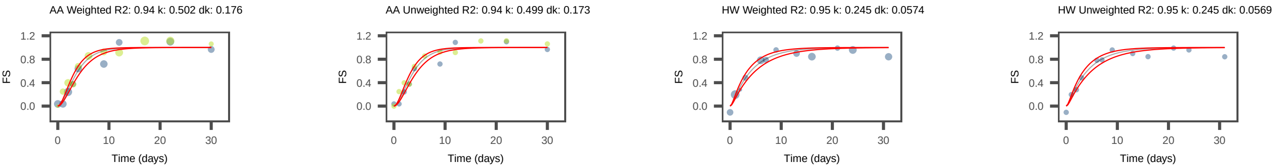

IMB1

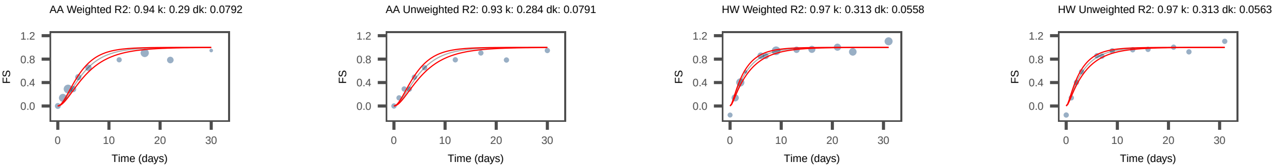

INMT

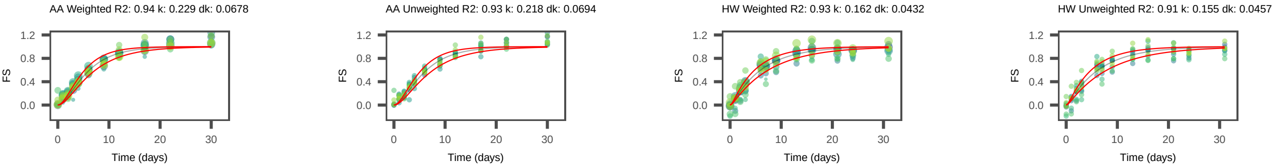

INPP

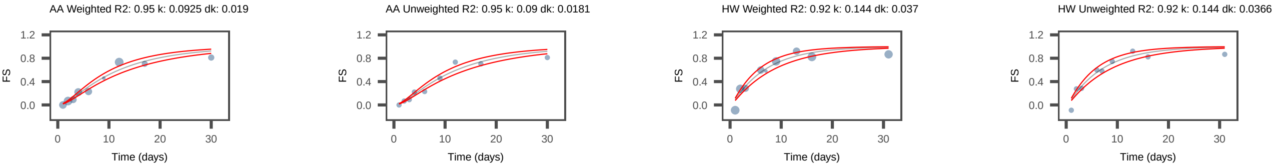

IPYR

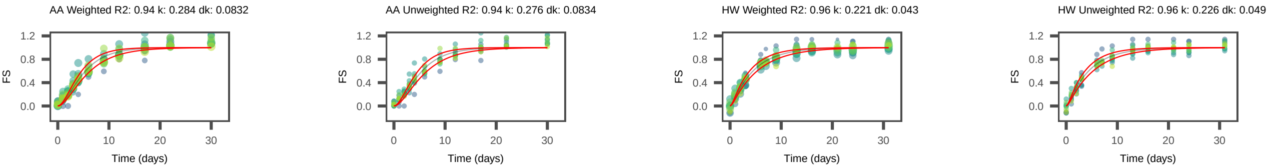

IPYR2

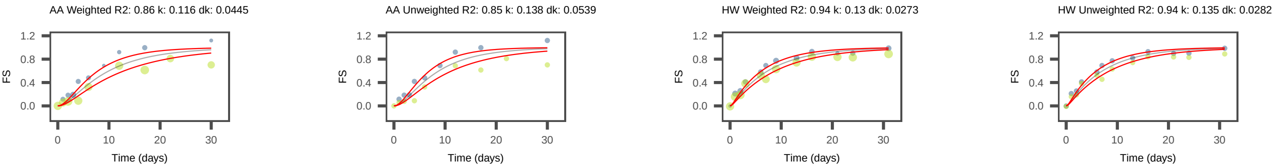

IQGA2

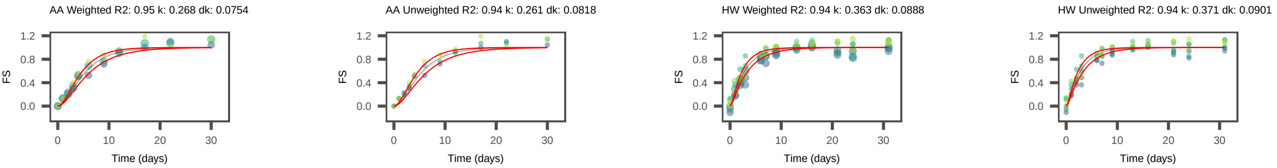

IRGM1

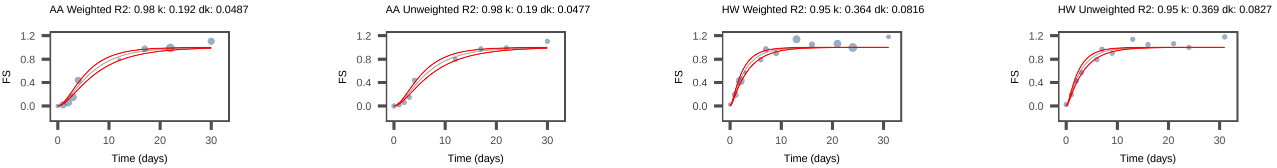

ISC2A

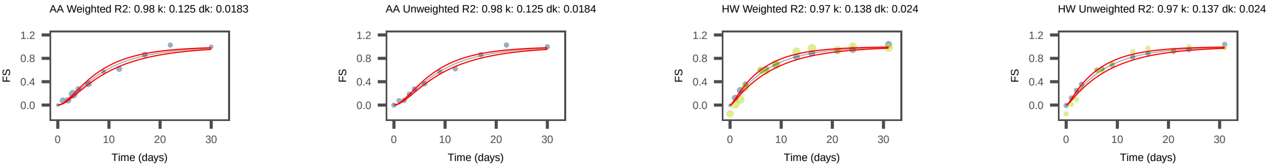

ISOC1

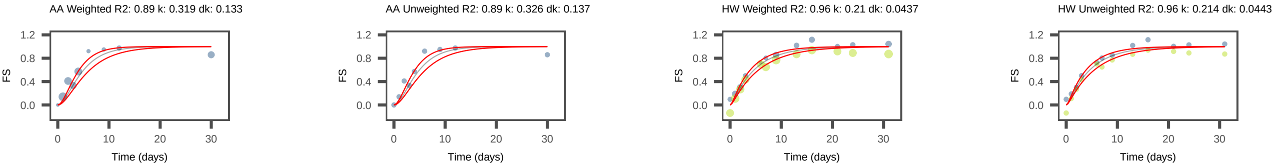

IVD

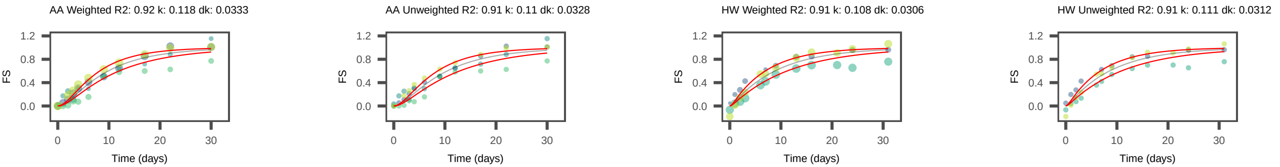

K1C18

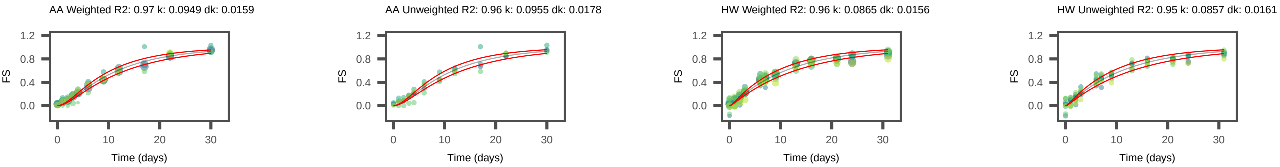

K2C8

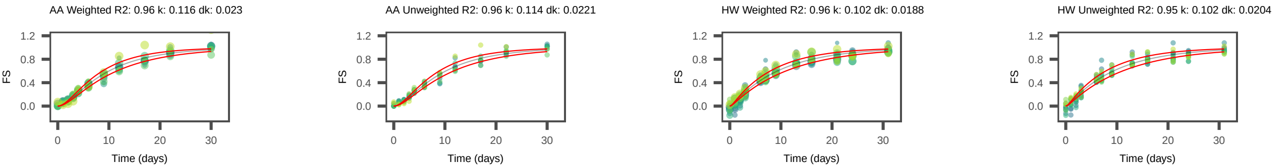

KAD2

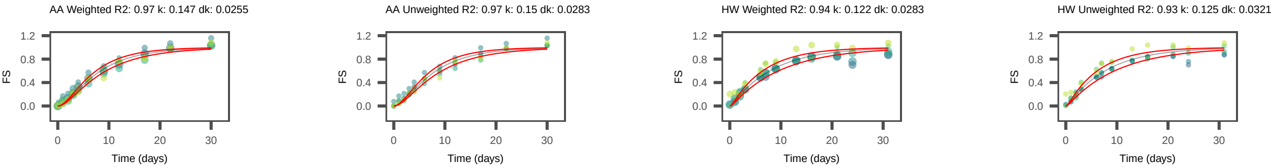

KAD3

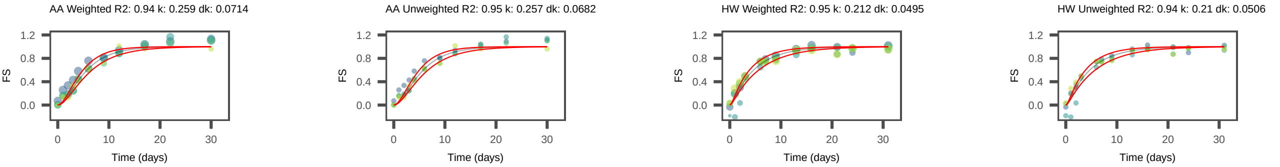

KAT1

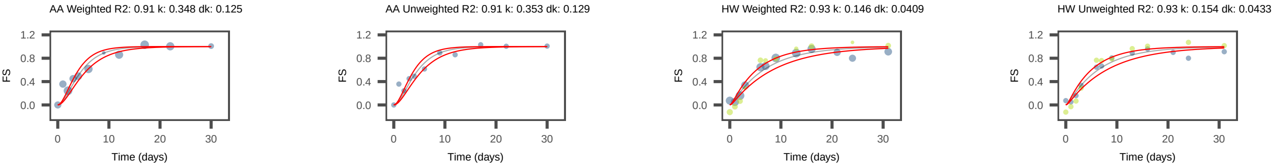

KAT3

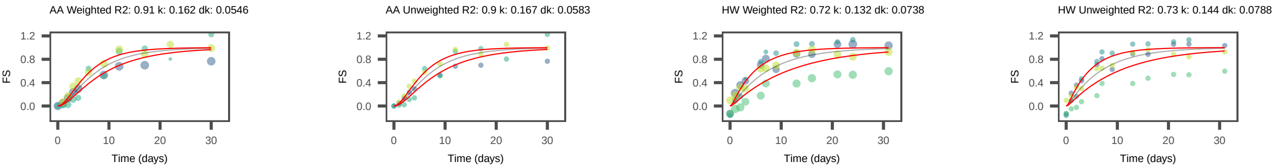

KBL

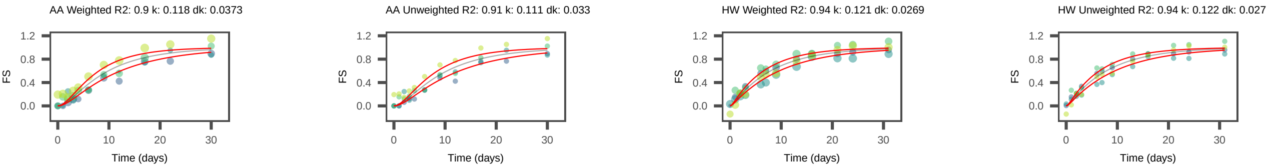

KCY

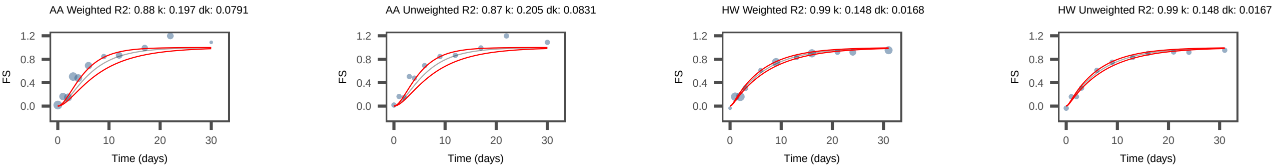

KEG1

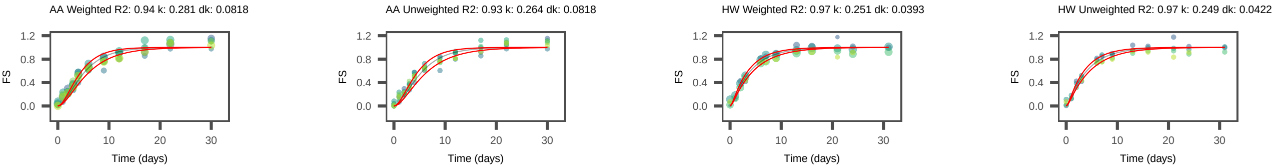

KHK

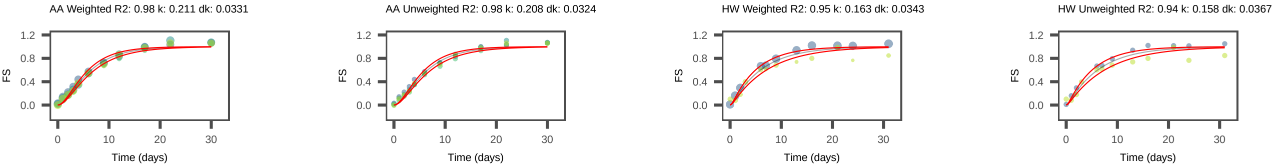

KMO

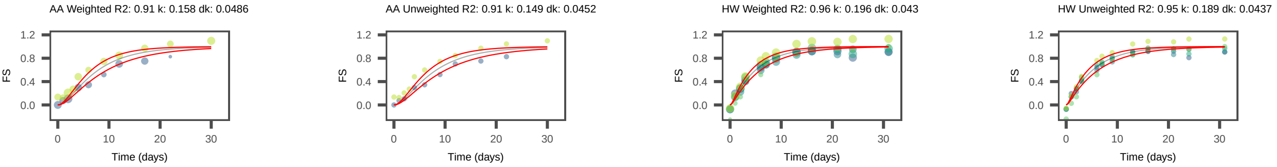

KPYR

AA Weighted R2: 0.92 k: 0.171 dk: 0.0544

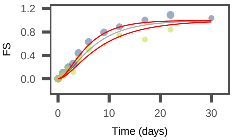

AA Unweighted R2: 0.9 k: 0.154 dk: 0.0531

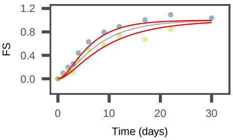

HW Weighted R2: 0.93 k: 0.198 dk: 0.0537

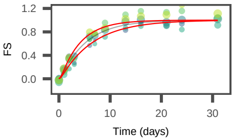

HW Unweighted R2: 0.91 k: 0.192 dk: 0.0601

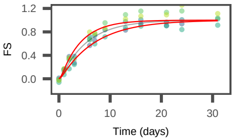

KYNU

AA Weighted R2: 0.84 k: 0.186 dk: 0.083

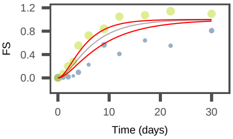

AA Unweighted R2: 0.82 k: 0.162 dk: 0.0762

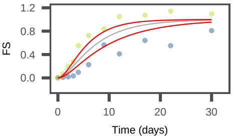

HW Weighted R2: 0.9 k: 0.329 dk: 0.117

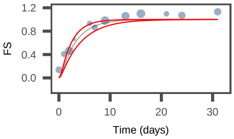

HW Unweighted R2: 0.91 k: 0.337 dk: 0.118

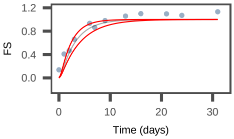

LACB2

AA Weighted R2: 0.9 k: 0.517 dk: 0.237

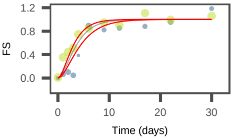

AA Unweighted R2: 0.88 k: 0.443 dk: 0.209

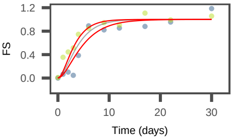

HW Weighted R2: 0.96 k: 0.435 dk: 0.0856

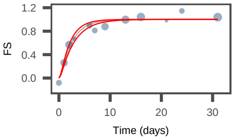

HW Unweighted R2: 0.96 k: 0.437 dk: 0.0863

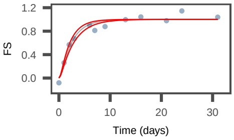

LAMP2

AA Weighted R2: 0.93 k: 5.51 dk: 6.74

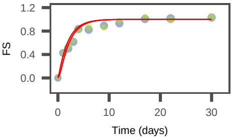

AA Unweighted R2: 0.93 k: 5.5 dk: 6.7

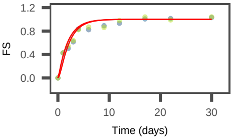

HW Weighted R2: 0.96 k: 0.691 dk: 0.126

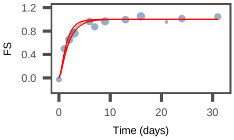

HW Unweighted R2: 0.96 k: 0.695 dk: 0.127

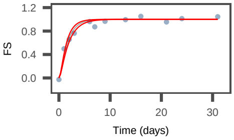

LDHA

AA Weighted R2: 0.94 k: 0.272 dk: 0.0743

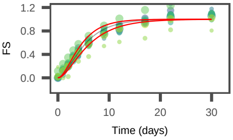

AA Unweighted R2: 0.93 k: 0.248 dk: 0.0751

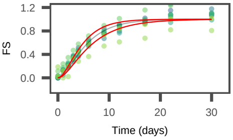

HW Weighted R2: 0.89 k: 0.195 dk: 0.0643

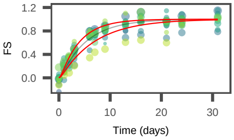

HW Unweighted R2: 0.89 k: 0.192 dk: 0.0625

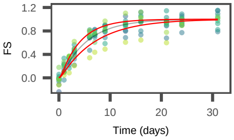

LEG1

AA Weighted R2: 0.99 k: 0.215 dk: 0.0285

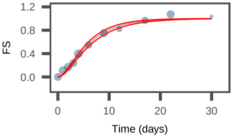

AA Unweighted R2: 0.99 k: 0.213 dk: 0.0281

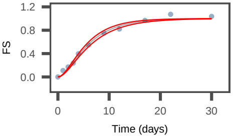

HW Weighted R2: 0.88 k: 0.106 dk: 0.0331

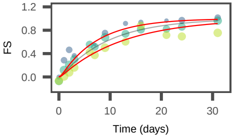

HW Unweighted R2: 0.88 k: 0.111 dk: 0.0353

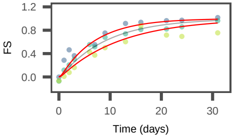

LEG9

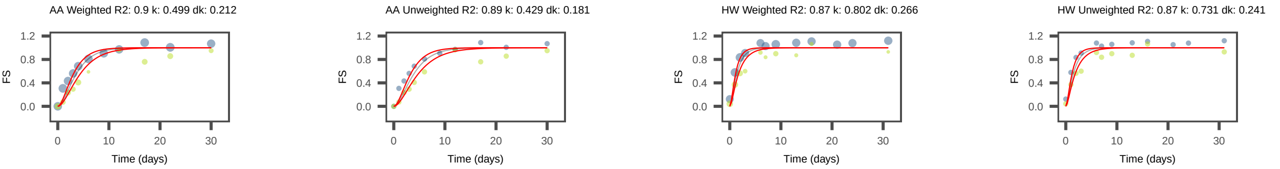

LGUL

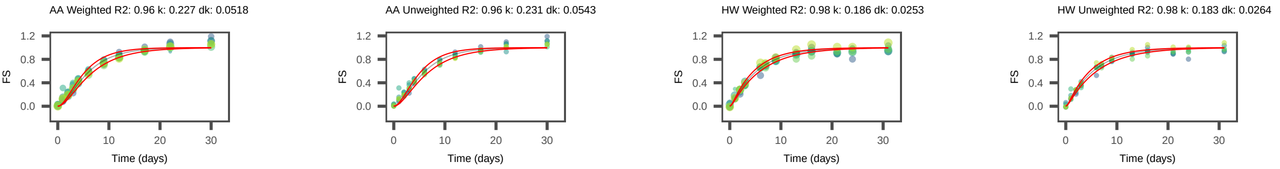

LHPP

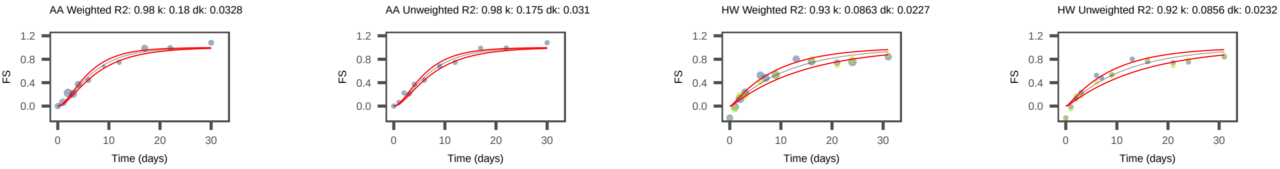

LICH

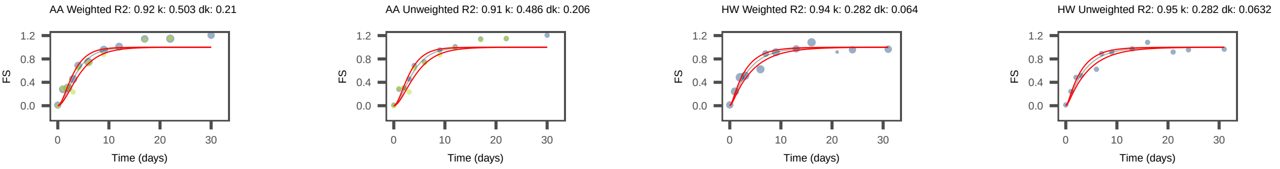

LKHA4

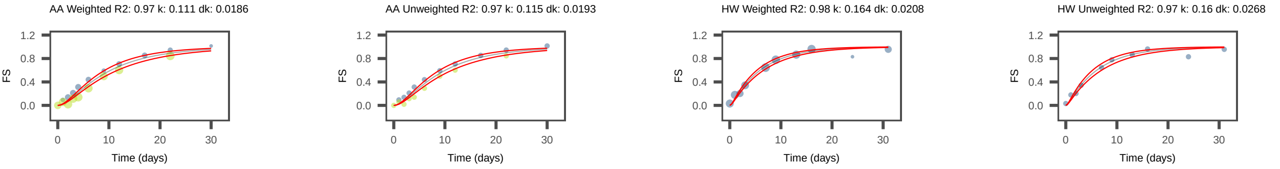

LMNA

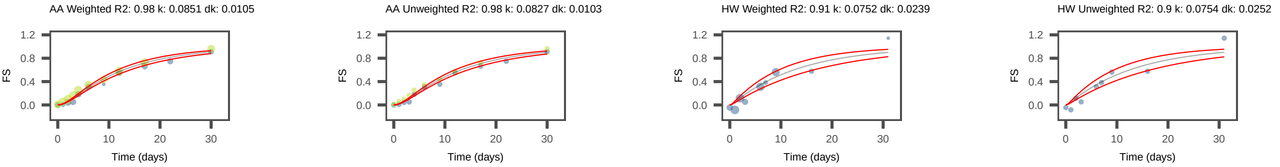

LONM

AA Weighted R2: 0.97 k: 0.205 dk: 0.0419 | AA Unweighted R2: 0.97 k: 0.203 dk: 0.0409 | HW Weighted R2: 0.98 k: 0.192 dk: 0.0304 | HW Unweighted R2: 0.98 k: 0.192 dk: 0.0305

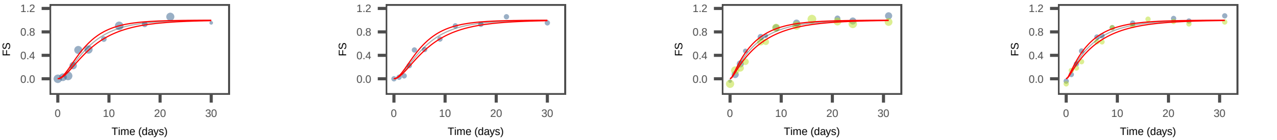

LPPRC

AA Weighted R2: 0.92 k: 0.384 dk: 0.135 | AA Unweighted R2: 0.92 k: 0.389 dk: 0.145 | HW Weighted R2: 0.94 k: 0.355 dk: 0.0814 | HW Unweighted R2: 0.94 k: 0.354 dk: 0.0817

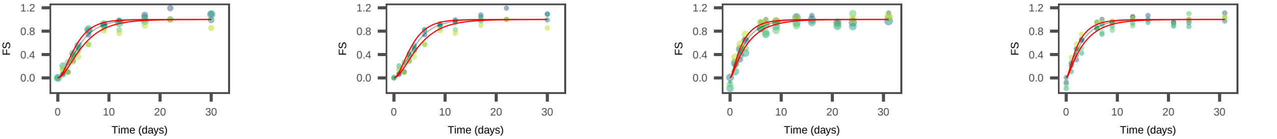

LRC59

AA Weighted R2: 0.88 k: 0.0841 dk: 0.026 | AA Unweighted R2: 0.88 k: 0.0819 dk: 0.0255 | HW Weighted R2: 0.91 k: 0.0771 dk: 0.0186 | HW Unweighted R2: 0.9 k: 0.0745 dk: 0.0182

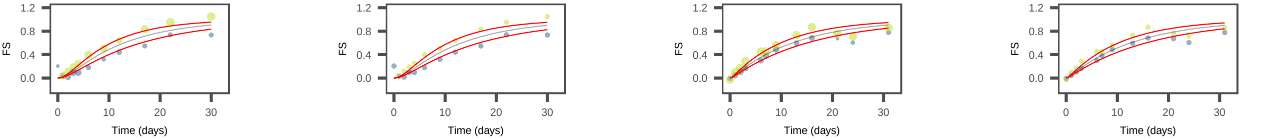

LYAG

AA Weighted R2: 0.98 k: 0.236 dk: 0.0407 | AA Unweighted R2: 0.98 k: 0.238 dk: 0.0412 | HW Weighted R2: 0.84 k: 0.134 dk: 0.055 | HW Unweighted R2: 0.85 k: 0.142 dk: 0.0559

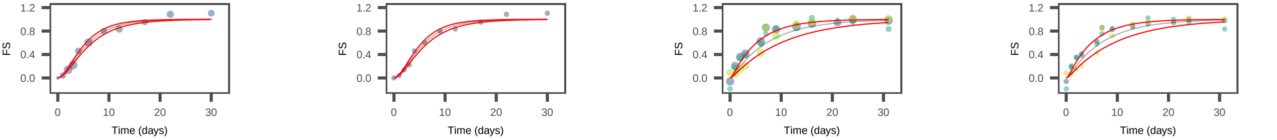

LYPA1

AA Weighted R2: 0.86 k: 0.226 dk: 0.0953 | AA Unweighted R2: 0.86 k: 0.237 dk: 0.1 | HW Weighted R2: 0.98 k: 0.208 dk: 0.033 | HW Unweighted R2: 0.97 k: 0.208 dk: 0.0338

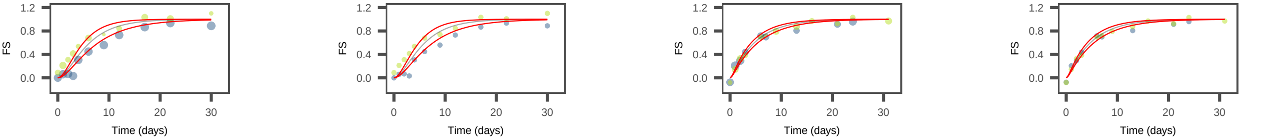

M2GD

AA Weighted R2: 0.94 k: 0.144 dk: 0.0388 | AA Unweighted R2: 0.92 k: 0.143 dk: 0.043 | HW Weighted R2: 0.94 k: 0.12 dk: 0.0268 | HW Unweighted R2: 0.94 k: 0.121 dk: 0.0274

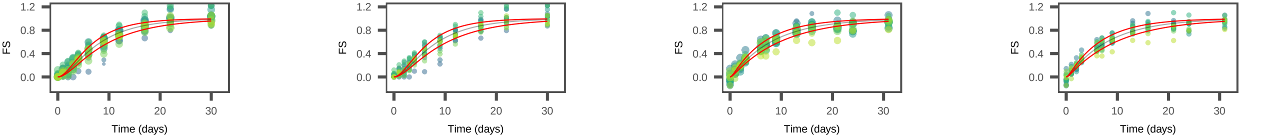

M2OM

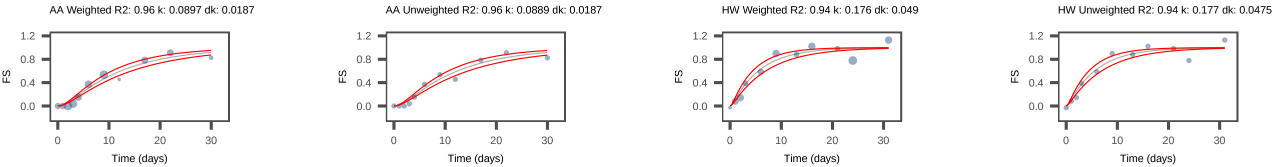

MAAI

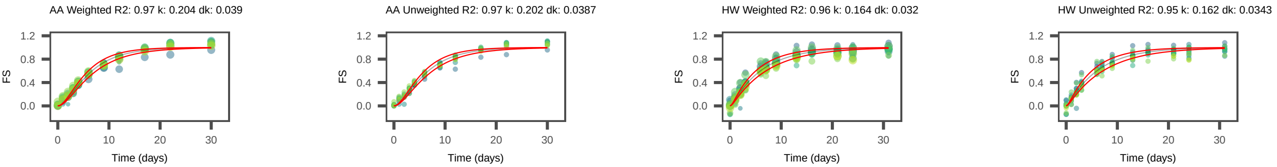

MAOX

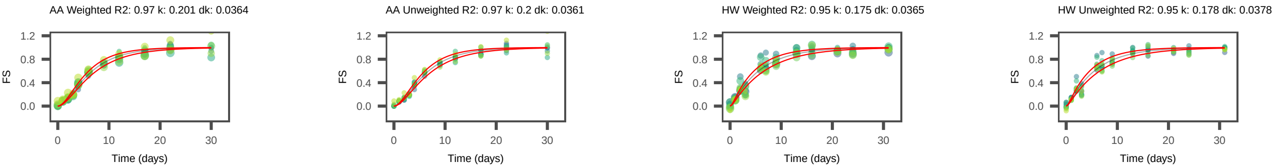

MARC1

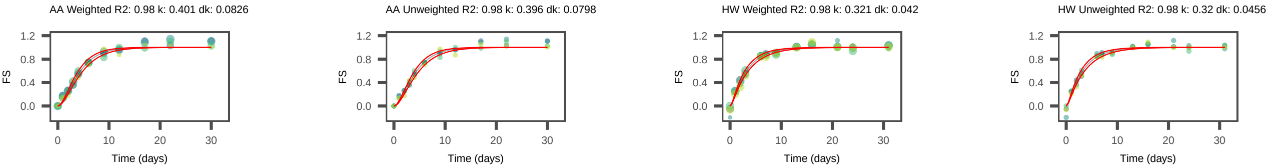

MARC2

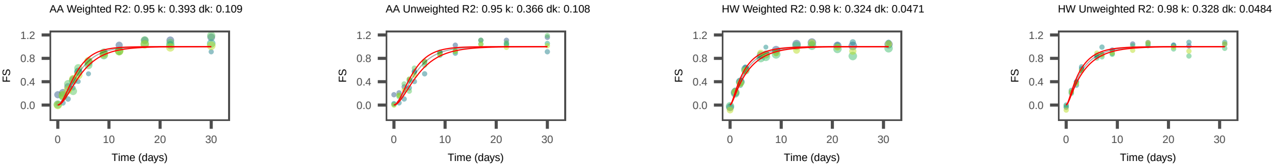

MATR3

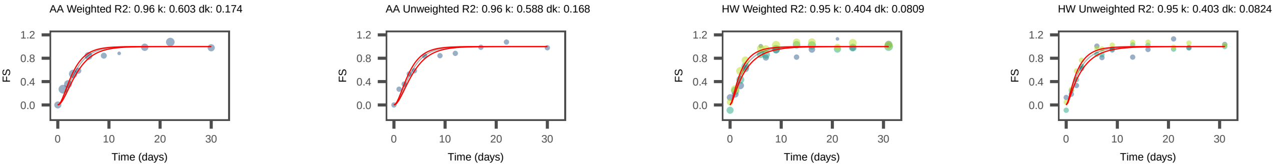

MCAT

AA Weighted R2: 0.98 k: 0.192 dk: 0.0277 | AA Unweighted R2: 0.98 k: 0.192 dk: 0.0279 | HW Weighted R2: 0.98 k: 0.152 dk: 0.0207 | HW Unweighted R2: 0.98 k: 0.153 dk: 0.0231

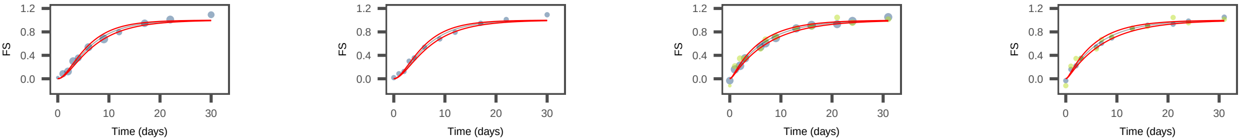

MCCA

AA Weighted R2: 0.92 k: 0.104 dk: 0.0308 | AA Unweighted R2: 0.9 k: 0.0987 dk: 0.0329 | HW Weighted R2: 0.96 k: 0.106 dk: 0.0219 | HW Unweighted R2: 0.95 k: 0.103 dk: 0.0234

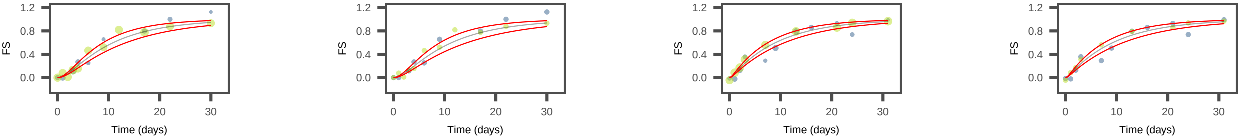

MCCB

AA Weighted R2: 0.95 k: 0.145 dk: 0.042 | AA Unweighted R2: 0.94 k: 0.145 dk: 0.0437 | HW Weighted R2: 0.93 k: 0.176 dk: 0.0405 | HW Unweighted R2: 0.92 k: 0.181 dk: 0.0442

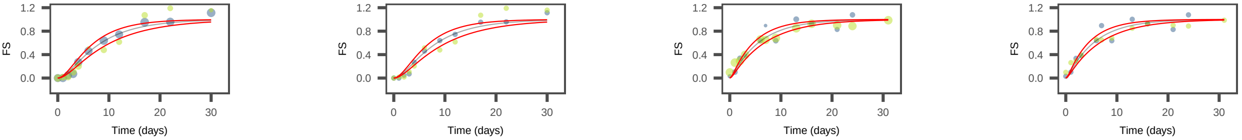

MCEE

AA Weighted R2: 0.97 k: 0.101 dk: 0.015 | AA Unweighted R2: 0.97 k: 0.101 dk: 0.0152 | HW Weighted R2: 0.94 k: 0.2 dk: 0.0594 | HW Unweighted R2: 0.94 k: 0.2 dk: 0.0581

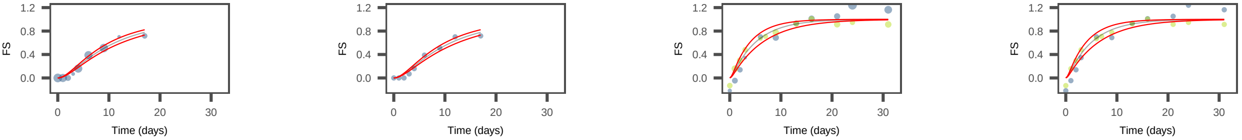

MCFD2

AA Weighted R2: 0.93 k: 3.93 dk: 4.07 | AA Unweighted R2: 0.92 k: 3.44 dk: 3.46 | HW Weighted R2: 0.98 k: 0.951 dk: 0.134 | HW Unweighted R2: 0.98 k: 0.949 dk: 0.129

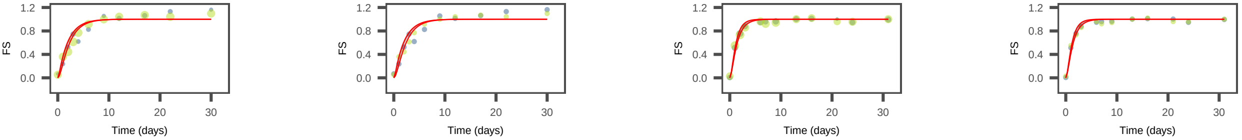

MDHC

AA Weighted R2: 0.93 k: 0.236 dk: 0.0724 | AA Unweighted R2: 0.93 k: 0.232 dk: 0.0689 | HW Weighted R2: 0.95 k: 0.179 dk: 0.0362 | HW Unweighted R2: 0.95 k: 0.184 dk: 0.0381

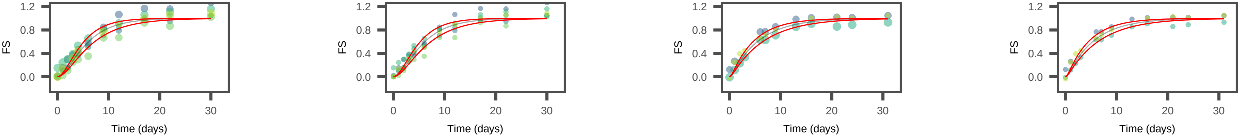

MDHM

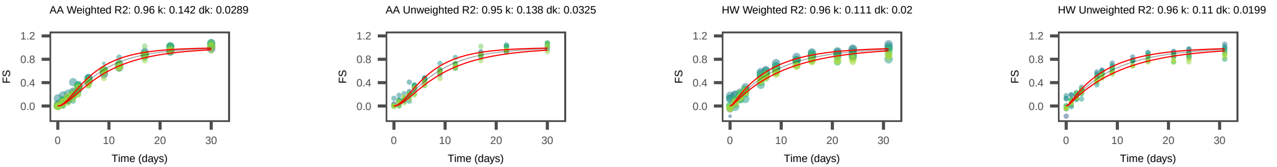

MET7B

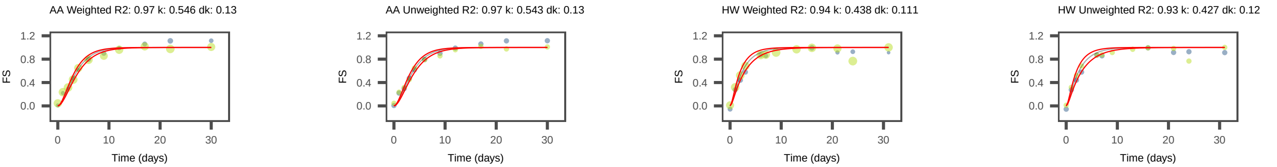

METK1

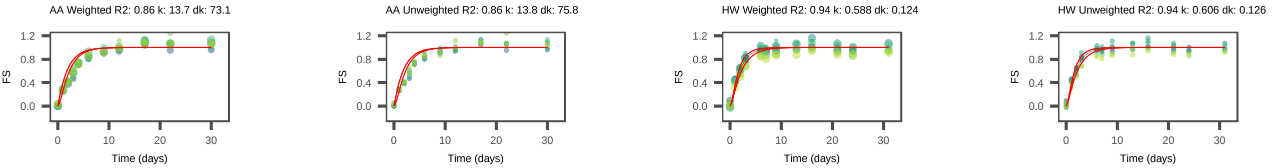

MGST1

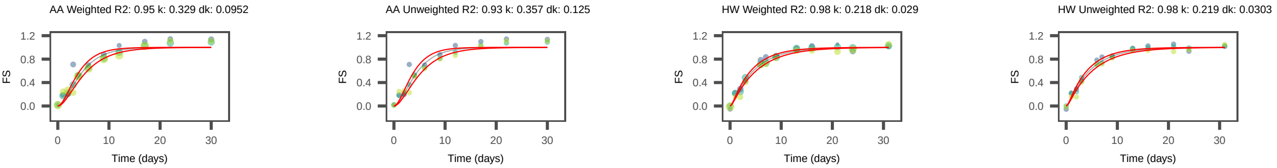

MIC13

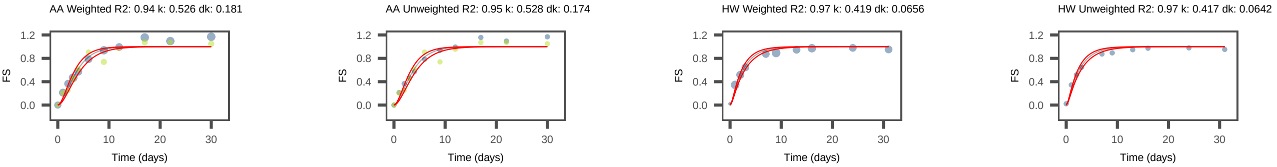

MIC26

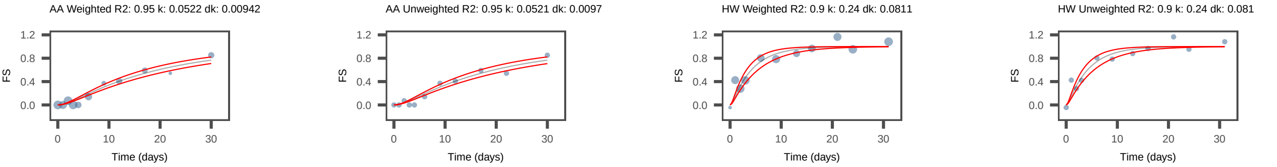

MIC27

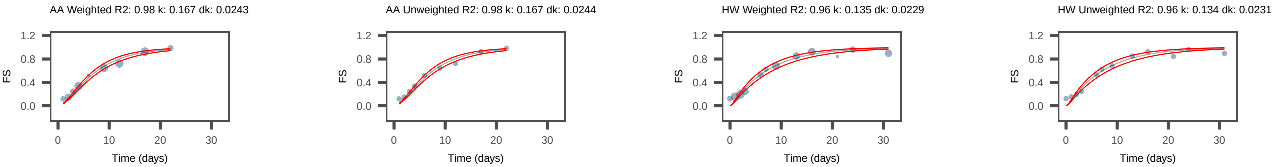

MIC60

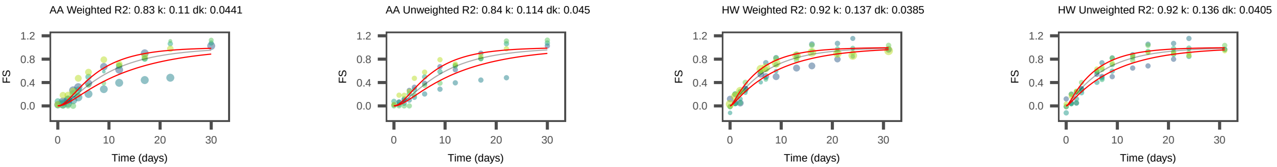

MIF

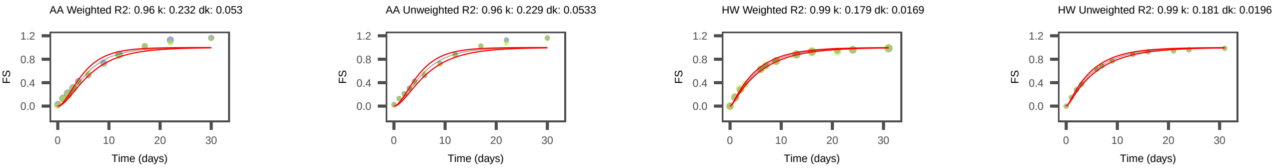

MMSA

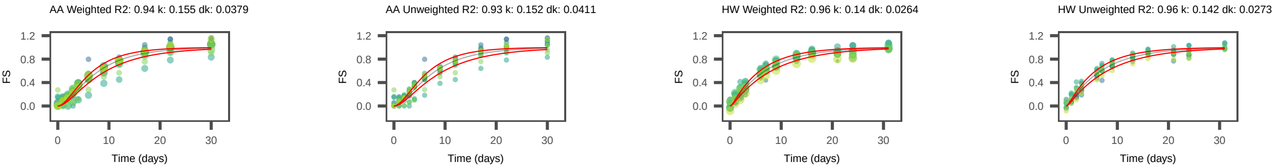

MPC1

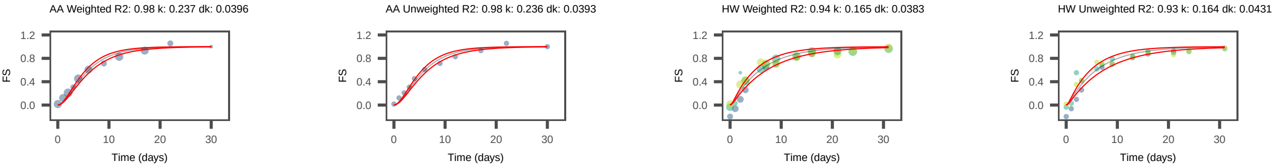

MPCP

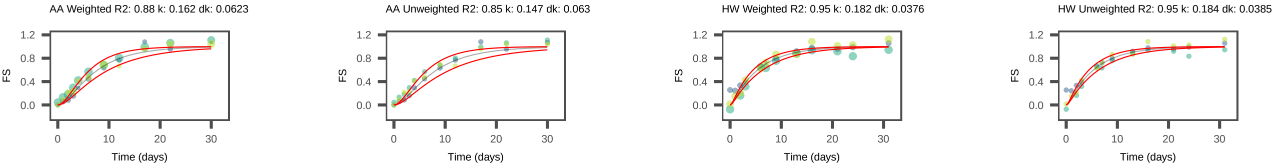

MPU1

AA Weighted R2: 0.97 k: 0.218 dk: 0.0492

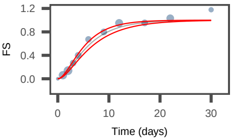

AA Unweighted R2: 0.96 k: 0.211 dk: 0.0489

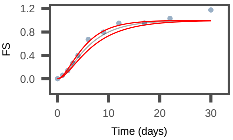

HW Weighted R2: 0.91 k: 0.137 dk: 0.0432

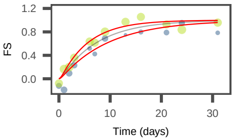

HW Unweighted R2: 0.9 k: 0.132 dk: 0.0425

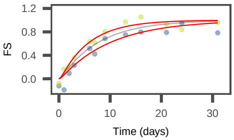

MRP6

AA Weighted R2: 0.95 k: 0.231 dk: 0.0595

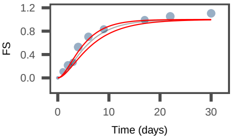

AA Unweighted R2: 0.96 k: 0.244 dk: 0.0615

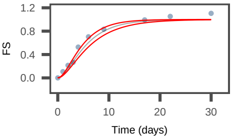

HW Weighted R2: 0.96 k: 0.31 dk: 0.0704

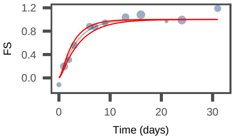

HW Unweighted R2: 0.96 k: 0.309 dk: 0.0707

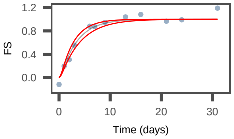

MSRA

AA Weighted R2: 0.95 k: 0.263 dk: 0.0785

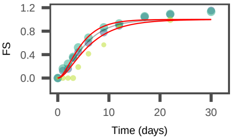

AA Unweighted R2: 0.93 k: 0.223 dk: 0.0752

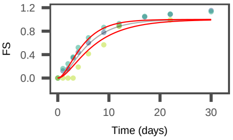

HW Weighted R2: 0.98 k: 0.255 dk: 0.039

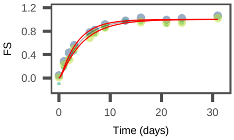

HW Unweighted R2: 0.98 k: 0.251 dk: 0.0382

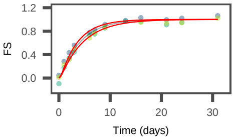

MTL26

AA Weighted R2: 0.98 k: 0.264 dk: 0.0509

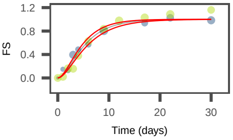

AA Unweighted R2: 0.98 k: 0.265 dk: 0.0498

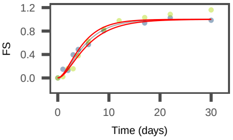

HW Weighted R2: 0.95 k: 0.169 dk: 0.0371

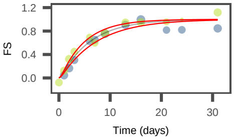

HW Unweighted R2: 0.94 k: 0.169 dk: 0.0371

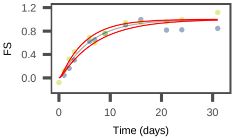

MTP

AA Weighted R2: 0.95 k: 0.279 dk: 0.0695

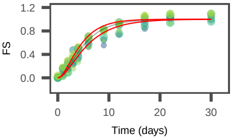

AA Unweighted R2: 0.95 k: 0.263 dk: 0.0693

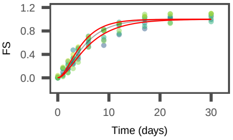

HW Weighted R2: 0.94 k: 0.253 dk: 0.0613

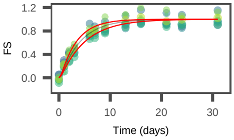

HW Unweighted R2: 0.94 k: 0.259 dk: 0.0629

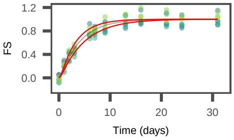

MTX2

AA Weighted R2: 0.98 k: 0.188 dk: 0.0351

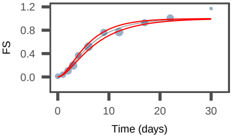

AA Unweighted R2: 0.98 k: 0.189 dk: 0.0369

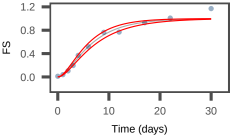

HW Weighted R2: 0.95 k: 0.227 dk: 0.0559

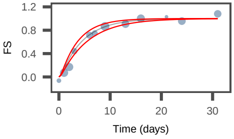

HW Unweighted R2: 0.94 k: 0.242 dk: 0.0652

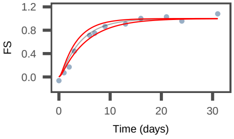

MUG1

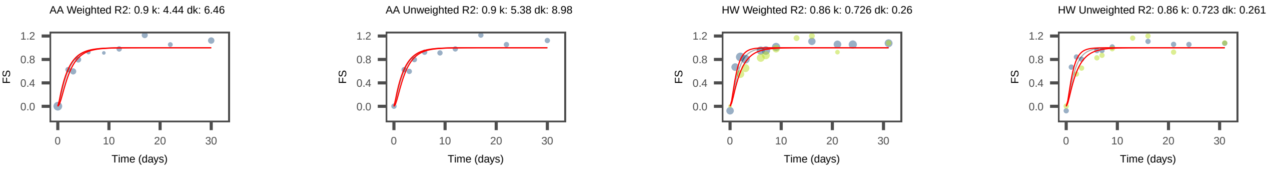

MUTA

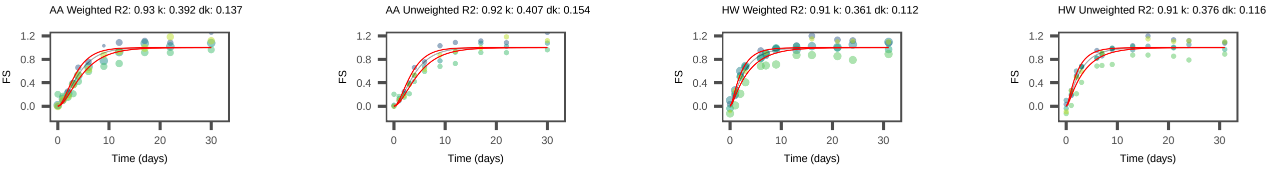

MVP

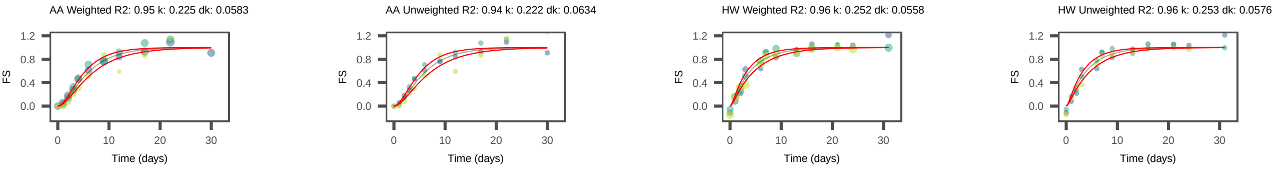

MYDGF

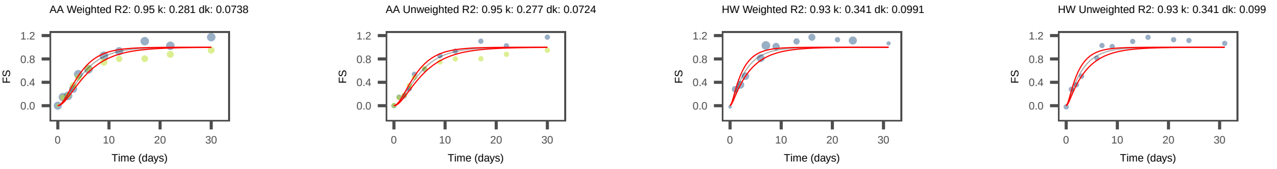

MYH9

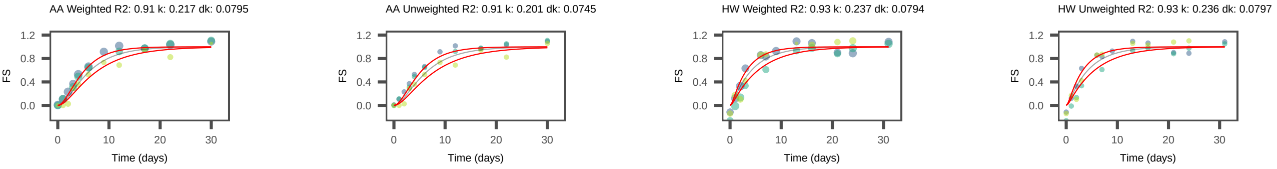

MYL6

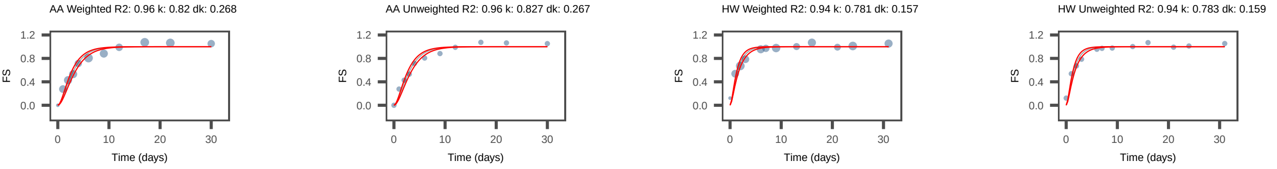

MYO1B

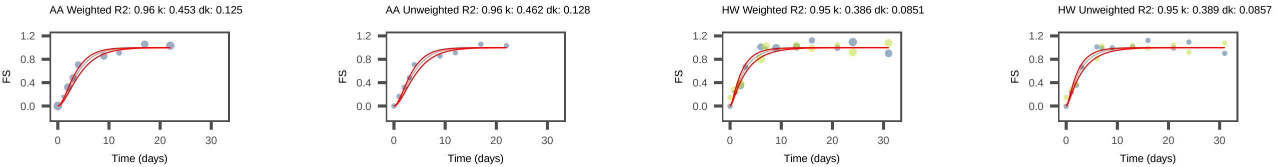

NB5R3

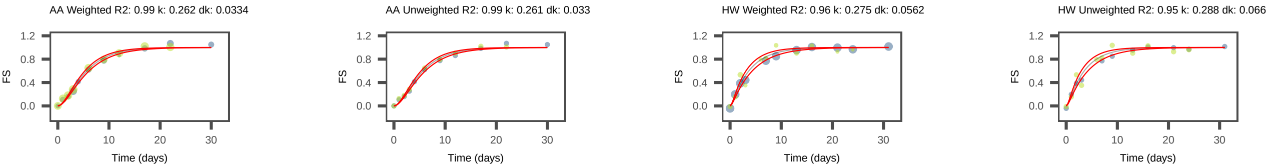

NCPR

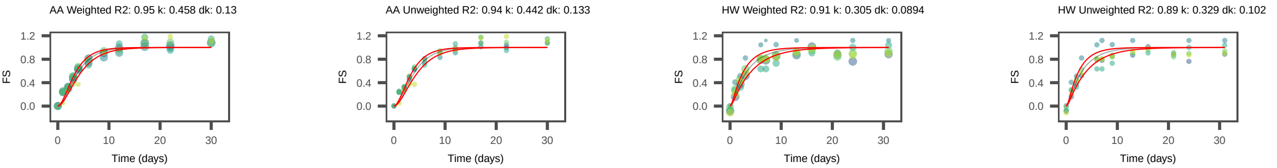

NDKA

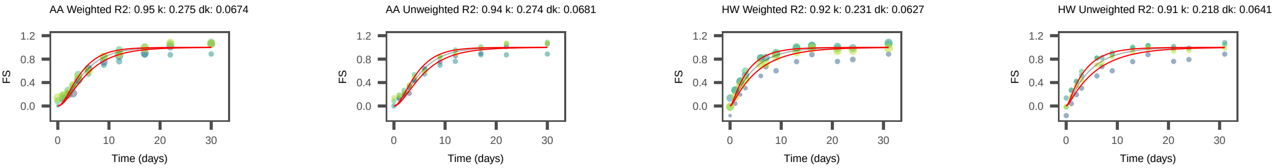

NDKB

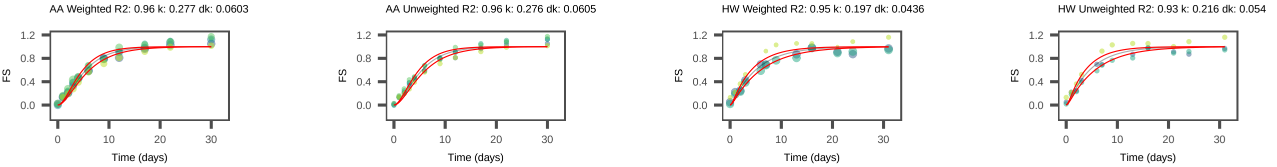

NDRG2

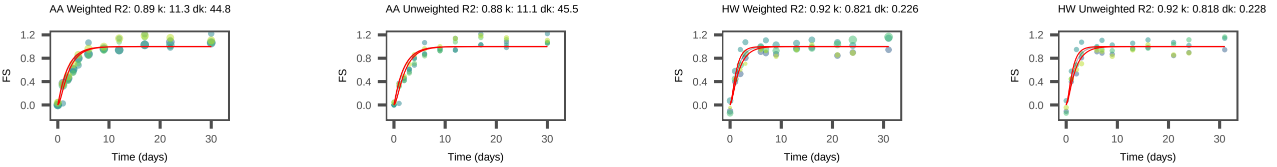

NDUA4

AA Weighted R2: 0.95 k: 0.502 dk: 0.142

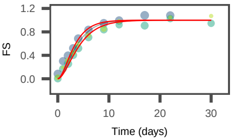

AA Unweighted R2: 0.95 k: 0.498 dk: 0.139

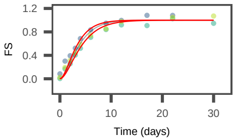

HW Weighted R2: 0.93 k: 0.277 dk: 0.0724

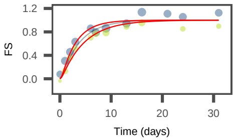

HW Unweighted R2: 0.93 k: 0.274 dk: 0.0713

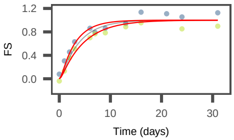

NDUA6

AA Weighted R2: 0.96 k: 0.315 dk: 0.0745

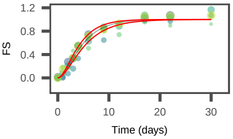

AA Unweighted R2: 0.95 k: 0.286 dk: 0.074

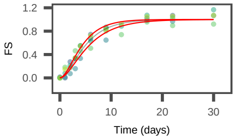

HW Weighted R2: 0.91 k: 0.297 dk: 0.0958

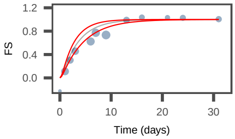

HW Unweighted R2: 0.91 k: 0.27 dk: 0.0873

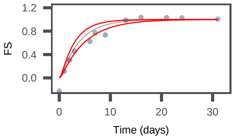

NDUA8

AA Weighted R2: 0.98 k: 0.0621 dk: 0.00596

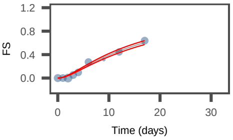

AA Unweighted R2: 0.98 k: 0.0617 dk: 0.0059

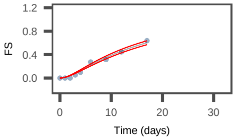

HW Weighted R2: 0.97 k: 0.124 dk: 0.0182

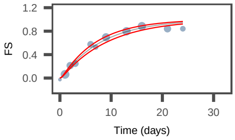

HW Unweighted R2: 0.97 k: 0.124 dk: 0.0183

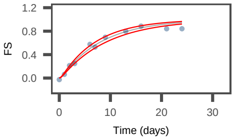

NDUAA

AA Weighted R2: 0.99 k: 0.227 dk: 0.0253

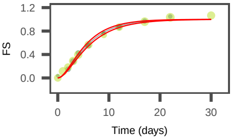

AA Unweighted R2: 0.99 k: 0.226 dk: 0.025

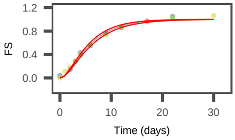

HW Weighted R2: 0.93 k: 0.227 dk: 0.0634

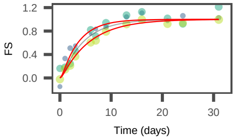

HW Unweighted R2: 0.93 k: 0.236 dk: 0.0674

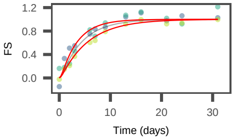

NDUAD

AA Weighted R2: 0.98 k: 0.183 dk: 0.0365

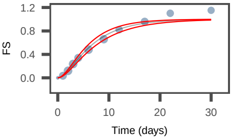

AA Unweighted R2: 0.97 k: 0.183 dk: 0.0368

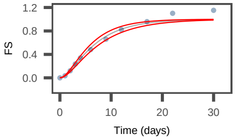

HW Weighted R2: 0.92 k: 0.153 dk: 0.0471

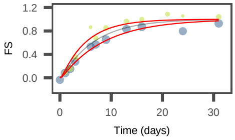

HW Unweighted R2: 0.91 k: 0.163 dk: 0.0539

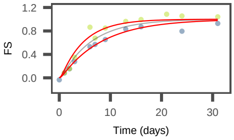

NDUB3

AA Weighted R2: 0.98 k: 0.139 dk: 0.0211

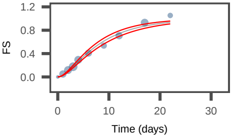

AA Unweighted R2: 0.98 k: 0.139 dk: 0.0215

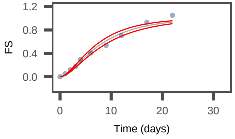

HW Weighted R2: 0.99 k: 0.151 dk: 0.0157

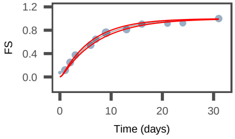

HW Unweighted R2: 0.99 k: 0.151 dk: 0.0159

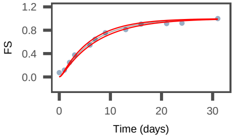

NDUS1

AA Weighted R2: 0.96 k: 0.153 dk: 0.0335

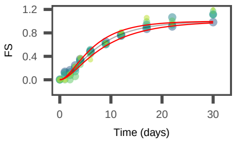

AA Unweighted R2: 0.95 k: 0.152 dk: 0.0358

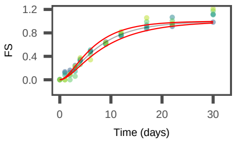

HW Weighted R2: 0.95 k: 0.138 dk: 0.0307

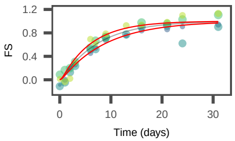

HW Unweighted R2: 0.95 k: 0.136 dk: 0.031

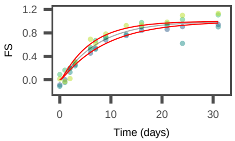

NDUS2

AA Weighted R2: 0.95 k: 0.202 dk: 0.0529

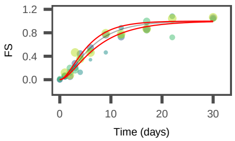

AA Unweighted R2: 0.94 k: 0.191 dk: 0.0521

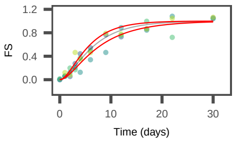

HW Weighted R2: 0.97 k: 0.157 dk: 0.0276

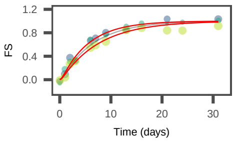

HW Unweighted R2: 0.97 k: 0.159 dk: 0.0276

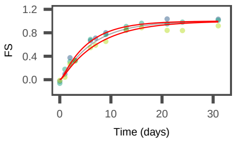

NDUS6

AA Weighted R2: 0.95 k: 0.383 dk: 0.107

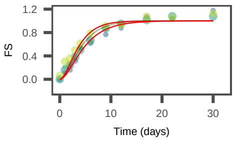

AA Unweighted R2: 0.95 k: 0.371 dk: 0.104

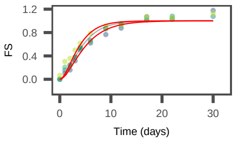

HW Weighted R2: 0.97 k: 0.298 dk: 0.0501

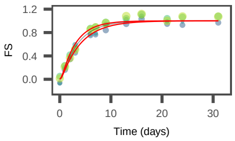

HW Unweighted R2: 0.97 k: 0.292 dk: 0.0486

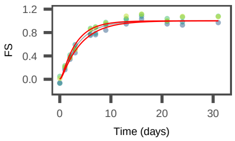

NDUV1

AA Weighted R2: 0.89 k: 0.183 dk: 0.0676

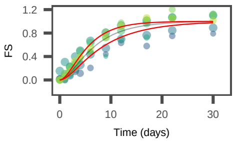

AA Unweighted R2: 0.88 k: 0.173 dk: 0.0655

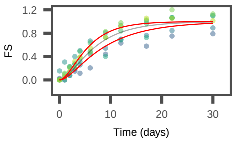

HW Weighted R2: 0.92 k: 0.166 dk: 0.0445

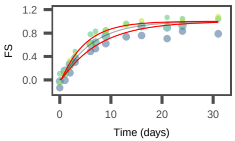

HW Unweighted R2: 0.92 k: 0.17 dk: 0.0454

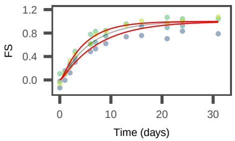

NDUV2

AA Weighted R2: 0.94 k: 0.166 dk: 0.0446

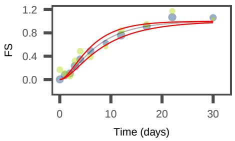

AA Unweighted R2: 0.94 k: 0.162 dk: 0.0462

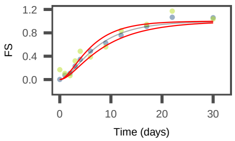

HW Weighted R2: 0.92 k: 0.206 dk: 0.0524

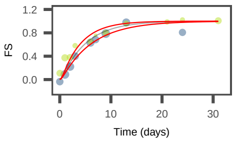

HW Unweighted R2: 0.92 k: 0.208 dk: 0.0534

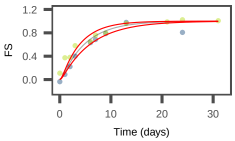

NDUV3

AA Weighted R2: 0.98 k: 0.19 dk: 0.0264

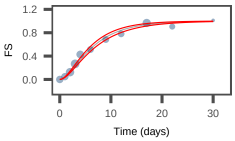

AA Unweighted R2: 0.98 k: 0.189 dk: 0.0262

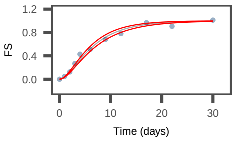

HW Weighted R2: 0.93 k: 0.277 dk: 0.0701

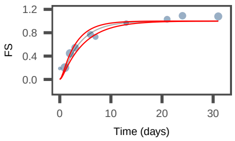

HW Unweighted R2: 0.93 k: 0.275 dk: 0.0707

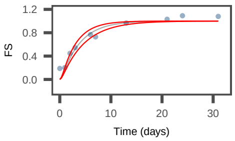

NFS1

AA Weighted R2: 0.98 k: 0.324 dk: 0.0584 | AA Unweighted R2: 0.98 k: 0.323 dk: 0.0578 | HW Weighted R2: 0.9 k: 0.0998 dk: 0.0286 | HW Unweighted R2: 0.9 k: 0.0993 dk: 0.0284

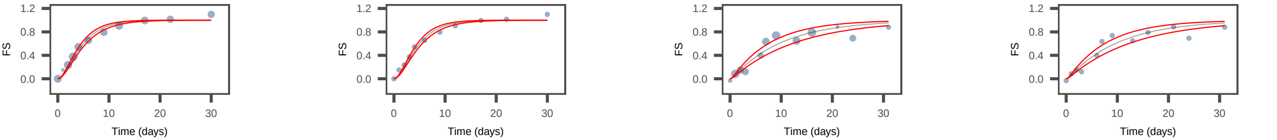

NIPS1

AA Weighted R2: 0.98 k: 0.149 dk: 0.0243 | AA Unweighted R2: 0.98 k: 0.147 dk: 0.0244 | HW Weighted R2: 0.98 k: 0.102 dk: 0.0143 | HW Unweighted R2: 0.97 k: 0.102 dk: 0.0156

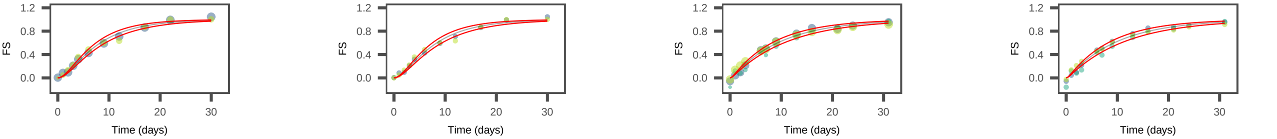

NIT2

AA Weighted R2: 0.92 k: 0.136 dk: 0.0399 | AA Unweighted R2: 0.92 k: 0.133 dk: 0.0397 | HW Weighted R2: 0.93 k: 0.135 dk: 0.036 | HW Unweighted R2: 0.92 k: 0.134 dk: 0.0366

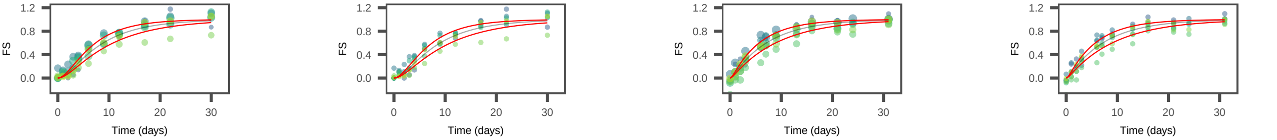

NNRD

AA Weighted R2: 0.98 k: 0.162 dk: 0.0255 | AA Unweighted R2: 0.98 k: 0.162 dk: 0.026 | HW Weighted R2: 0.98 k: 0.19 dk: 0.0253 | HW Unweighted R2: 0.98 k: 0.191 dk: 0.0254

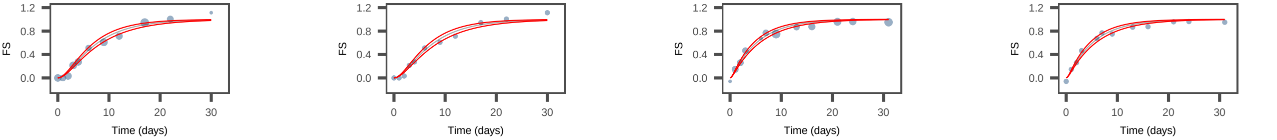

NNRE

AA Weighted R2: 0.92 k: 0.202 dk: 0.0617 | AA Unweighted R2: 0.93 k: 0.19 dk: 0.0562 | HW Weighted R2: 0.95 k: 0.102 dk: 0.0184 | HW Unweighted R2: 0.95 k: 0.101 dk: 0.0182

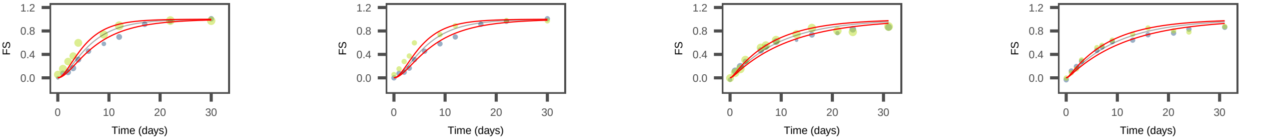

NNTM

AA Weighted R2: 0.95 k: 0.128 dk: 0.0298 | AA Unweighted R2: 0.95 k: 0.126 dk: 0.0303 | HW Weighted R2: 0.9 k: 0.133 dk: 0.042 | HW Unweighted R2: 0.89 k: 0.133 dk: 0.0432

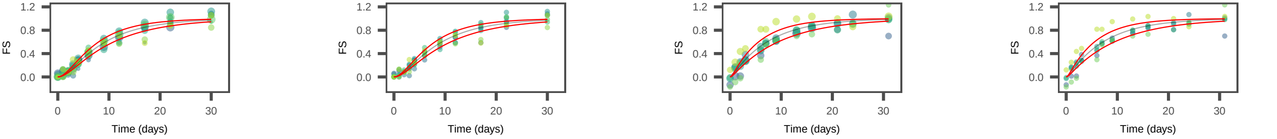

NPL4

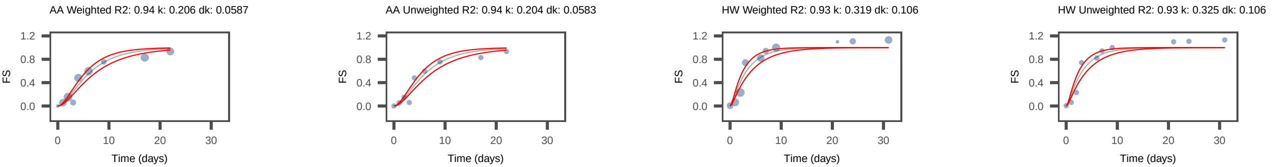

NPM

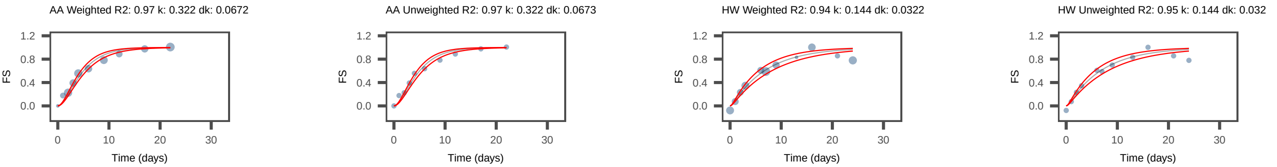

NPS3B

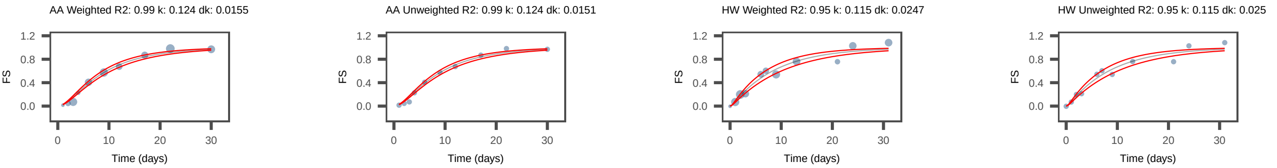

NQO2

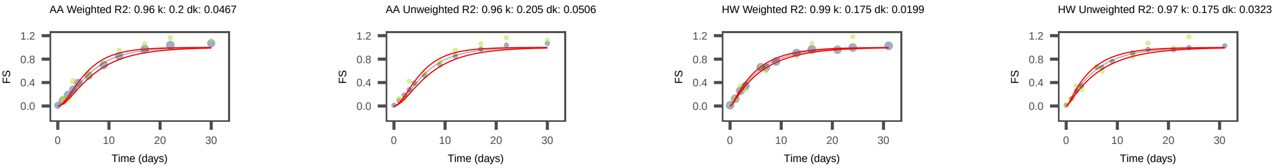

NT8F2

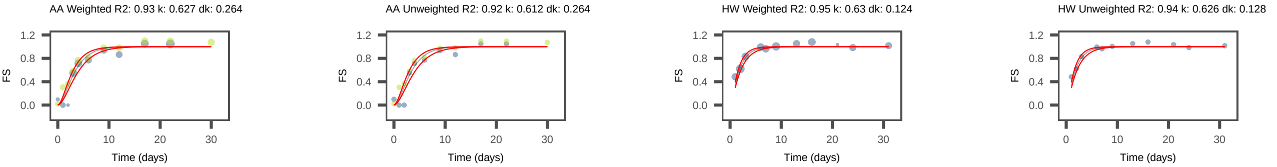

NU1M

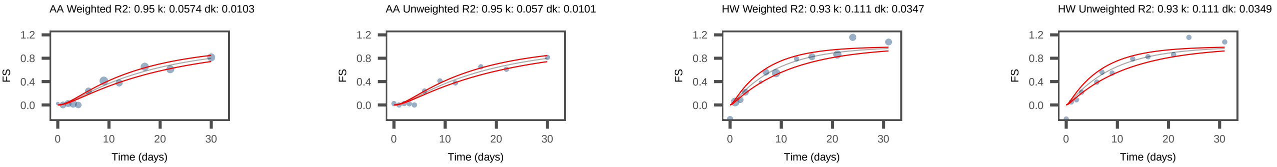

NU4M

AA Weighted R2: 0.94 k: 0.101 dk: 0.0269

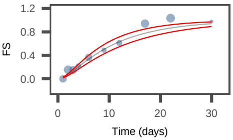

AA Unweighted R2: 0.94 k: 0.103 dk: 0.0264

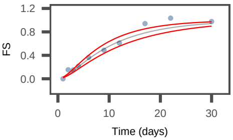

HW Weighted R2: 0.98 k: 0.132 dk: 0.0228

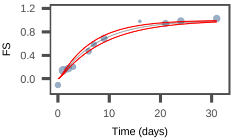

HW Unweighted R2: 0.97 k: 0.132 dk: 0.0233

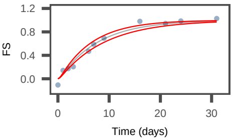

NU5M

AA Weighted R2: 0.99 k: 0.145 dk: 0.0148

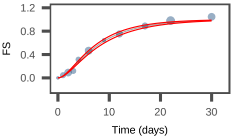

AA Unweighted R2: 0.99 k: 0.145 dk: 0.0147

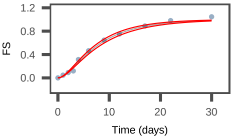

HW Weighted R2: 0.97 k: 0.165 dk: 0.0291

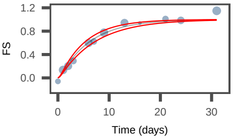

HW Unweighted R2: 0.97 k: 0.165 dk: 0.0295

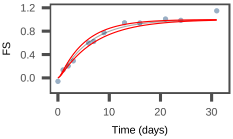

NUCL

AA Weighted R2: 0.94 k: 0.482 dk: 0.146

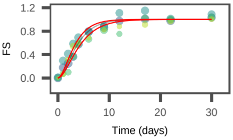

AA Unweighted R2: 0.94 k: 0.468 dk: 0.142

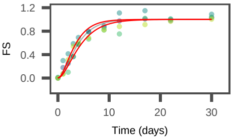

HW Weighted R2: 0.95 k: 0.375 dk: 0.0795

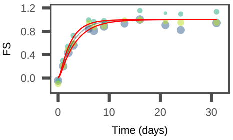

HW Unweighted R2: 0.95 k: 0.383 dk: 0.0816

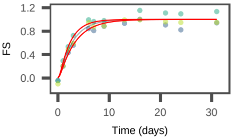

NUDT7

AA Weighted R2: 0.91 k: 0.521 dk: 0.205

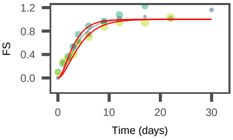

AA Unweighted R2: 0.9 k: 0.512 dk: 0.206

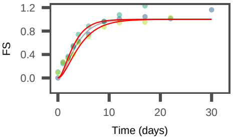

HW Weighted R2: 0.93 k: 0.442 dk: 0.116

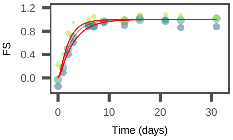

HW Unweighted R2: 0.91 k: 0.479 dk: 0.141

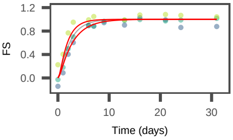

NUDT8

AA Weighted R2: 0.96 k: 0.0989 dk: 0.0222

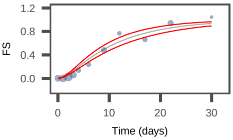

AA Unweighted R2: 0.96 k: 0.0997 dk: 0.0234

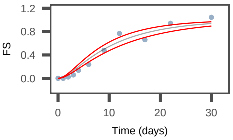

HW Weighted R2: 0.95 k: 0.114 dk: 0.0255

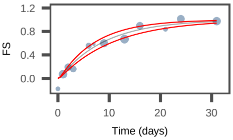

HW Unweighted R2: 0.95 k: 0.114 dk: 0.0261

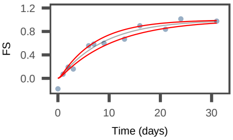

OAT

AA Weighted R2: 0.86 k: 8.31 dk: 24.3

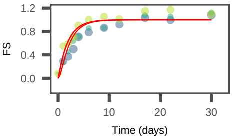

AA Unweighted R2: 0.87 k: 7.99 dk: 22

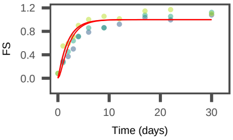

HW Weighted R2: 0.93 k: 0.959 dk: 0.269

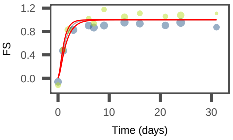

HW Unweighted R2: 0.93 k: 0.968 dk: 0.278

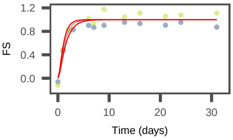

ODB2

AA Weighted R2: 0.96 k: 0.139 dk: 0.0298

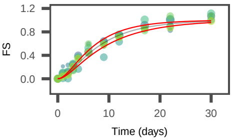

AA Unweighted R2: 0.95 k: 0.139 dk: 0.0308

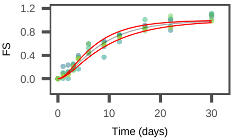

HW Weighted R2: 0.9 k: 0.144 dk: 0.0458

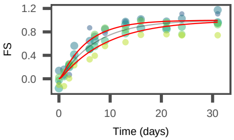

HW Unweighted R2: 0.89 k: 0.138 dk: 0.0477

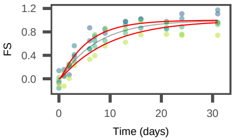

ODBA

AA Weighted R2: 0.93 k: 0.206 dk: 0.065

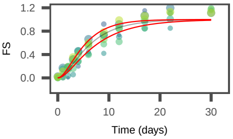

AA Unweighted R2: 0.92 k: 0.191 dk: 0.0625

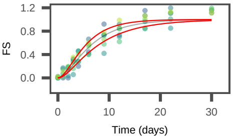

HW Weighted R2: 0.89 k: 0.195 dk: 0.0678

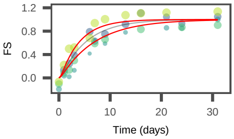

HW Unweighted R2: 0.88 k: 0.179 dk: 0.0637

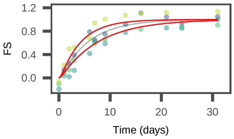

ODBB

AA Weighted R2: 0.63 k: 0.108 dk: 0.0634

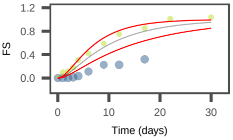

AA Unweighted R2: 0.69 k: 0.108 dk: 0.0589

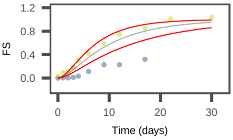

HW Weighted R2: 0.96 k: 0.147 dk: 0.0236

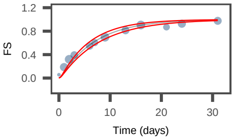

HW Unweighted R2: 0.96 k: 0.147 dk: 0.0235

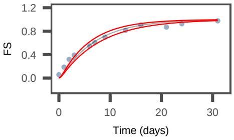

ODO1

AA Weighted R2: 0.92 k: 0.43 dk: 0.165

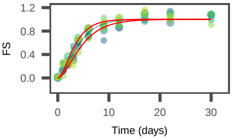

AA Unweighted R2: 0.92 k: 0.425 dk: 0.158

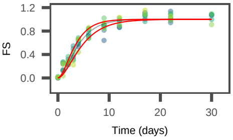

HW Weighted R2: 0.93 k: 0.353 dk: 0.0883

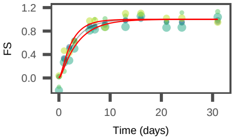

HW Unweighted R2: 0.94 k: 0.362 dk: 0.0854

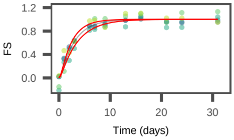

ODP2

AA Weighted R2: 0.93 k: 0.129 dk: 0.0348

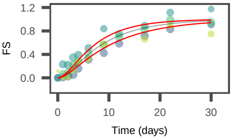

AA Unweighted R2: 0.92 k: 0.128 dk: 0.0356

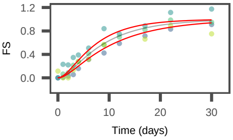

HW Weighted R2: 0.77 k: 0.0866 dk: 0.0381

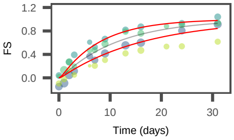

HW Unweighted R2: 0.77 k: 0.0857 dk: 0.0376

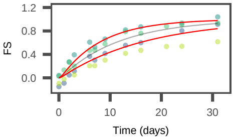

ODPA

AA Weighted R2: 0.99 k: 0.155 dk: 0.0118

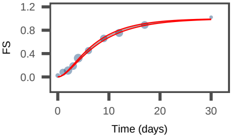

AA Unweighted R2: 0.99 k: 0.155 dk: 0.0118

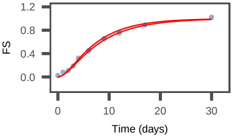

HW Weighted R2: 0.86 k: 0.0957 dk: 0.0319

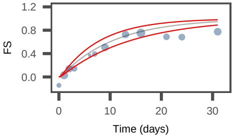

HW Unweighted R2: 0.86 k: 0.0996 dk: 0.0333

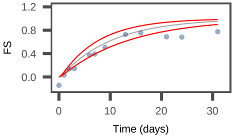

ODPB

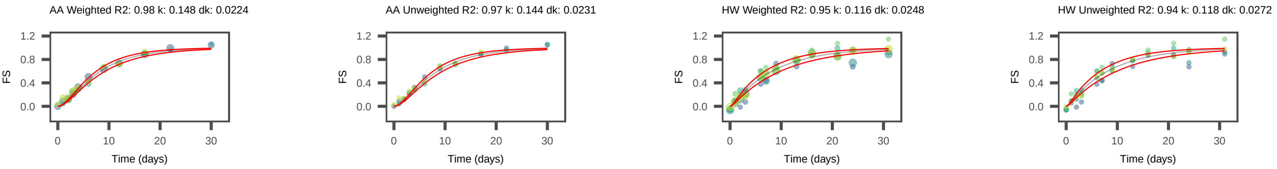

OLA1

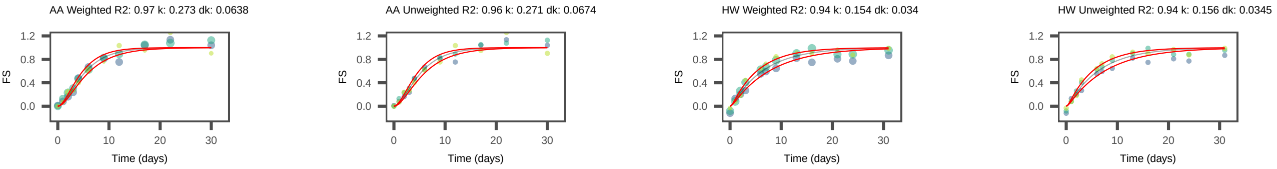

OPLA

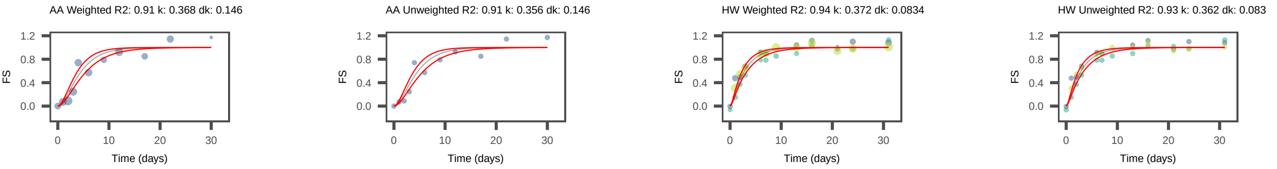

ORNT1

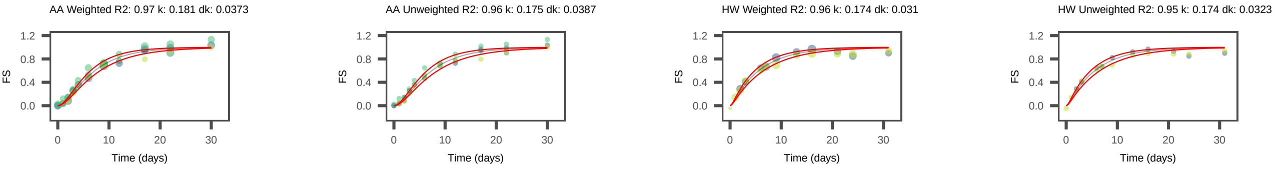

OST48

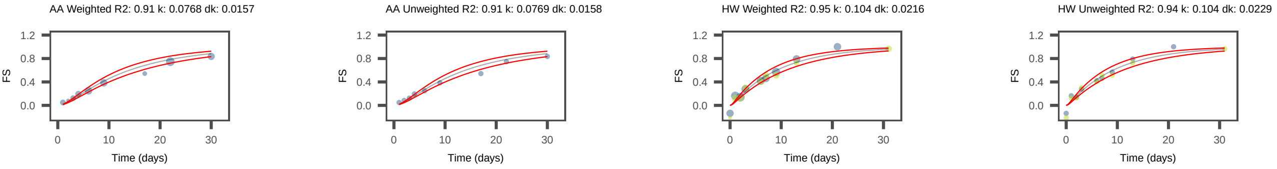

OSTF1

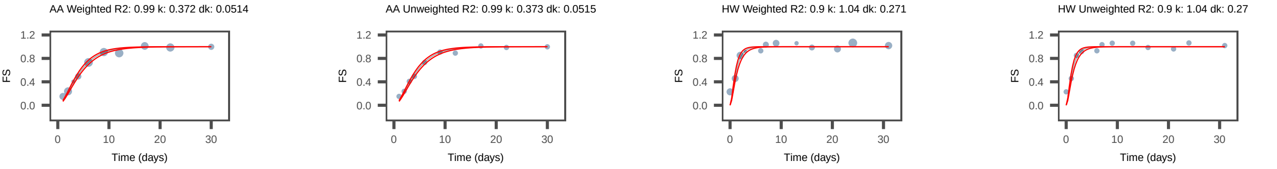

OTC

AA Weighted R2: 0.96 k: 0.136 dk: 0.0276

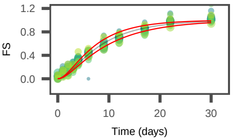

AA Unweighted R2: 0.95 k: 0.136 dk: 0.0319

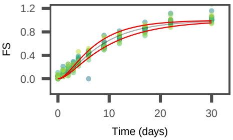

HW Weighted R2: 0.96 k: 0.114 dk: 0.0215

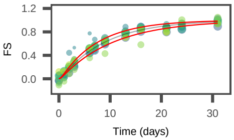

HW Unweighted R2: 0.95 k: 0.115 dk: 0.0242

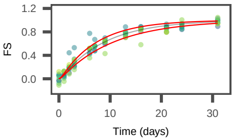

OTUB1

AA Weighted R2: 0.97 k: 0.31 dk: 0.0646

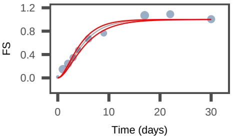

AA Unweighted R2: 0.97 k: 0.308 dk: 0.0624

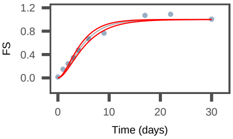

HW Weighted R2: 0.97 k: 0.494 dk: 0.0822

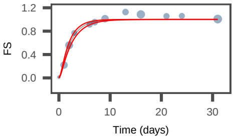

HW Unweighted R2: 0.97 k: 0.495 dk: 0.0823

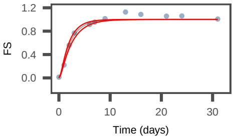

P20D1

AA Weighted R2: 0.96 k: 0.455 dk: 0.12

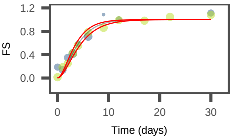

AA Unweighted R2: 0.96 k: 0.465 dk: 0.128

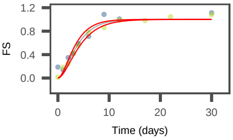

HW Weighted R2: 0.93 k: 0.393 dk: 0.103

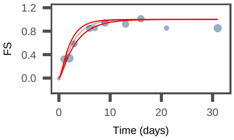

HW Unweighted R2: 0.93 k: 0.398 dk: 0.105

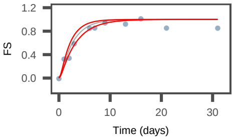

PA2G4

AA Weighted R2: 0.96 k: 0.205 dk: 0.0468

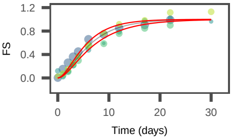

AA Unweighted R2: 0.96 k: 0.197 dk: 0.0442

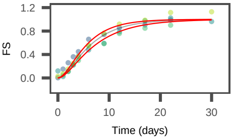

HW Weighted R2: 0.94 k: 0.153 dk: 0.0376

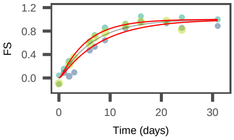

HW Unweighted R2: 0.94 k: 0.152 dk: 0.0381

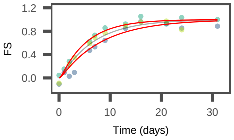

PABP1

AA Weighted R2: 0.97 k: 0.216 dk: 0.0401

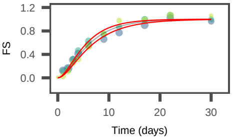

AA Unweighted R2: 0.97 k: 0.213 dk: 0.0409

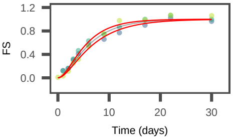

HW Weighted R2: 0.98 k: 0.171 dk: 0.018

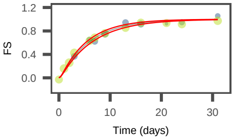

HW Unweighted R2: 0.98 k: 0.171 dk: 0.0182

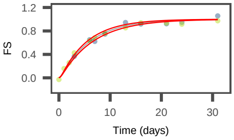

PAHX

AA Weighted R2: 0.92 k: 72.4 dk: 1470

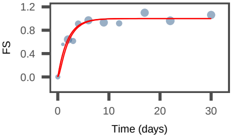

AA Unweighted R2: 0.92 k: 74.3 dk: 1580

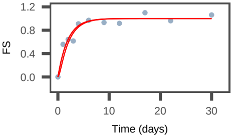

HW Weighted R2: 0.95 k: 2.01 dk: 0.42

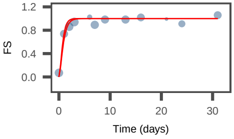

HW Unweighted R2: 0.95 k: 2.01 dk: 0.42

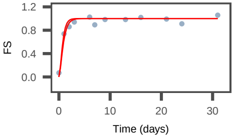

PAIRB

AA Weighted R2: 0.97 k: 0.483 dk: 0.115
AA Unweighted R2: 0.97 k: 0.468 dk: 0.114
HW Weighted R2: 0.92 k: 0.202 dk: 0.0478
HW Unweighted R2: 0.92 k: 0.201 dk: 0.0482

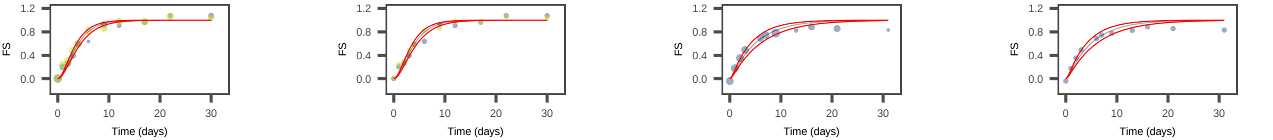

PAPS2

AA Weighted R2: 0.92 k: 0.575 dk: 0.276
AA Unweighted R2: 0.93 k: 0.566 dk: 0.264
HW Weighted R2: 0.93 k: 0.341 dk: 0.0863
HW Unweighted R2: 0.94 k: 0.346 dk: 0.086

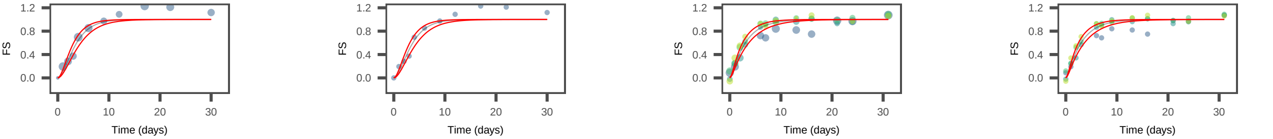

PARK7

AA Weighted R2: 0.95 k: 0.264 dk: 0.0648
AA Unweighted R2: 0.95 k: 0.255 dk: 0.0647
HW Weighted R2: 0.96 k: 0.176 dk: 0.0355
HW Unweighted R2: 0.96 k: 0.177 dk: 0.0365

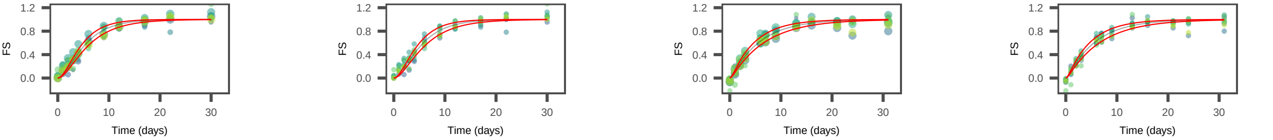

PCBP1

AA Weighted R2: 0.96 k: 0.732 dk: 0.208
AA Unweighted R2: 0.96 k: 0.73 dk: 0.206
HW Weighted R2: 0.95 k: 0.464 dk: 0.0885
HW Unweighted R2: 0.95 k: 0.464 dk: 0.0888

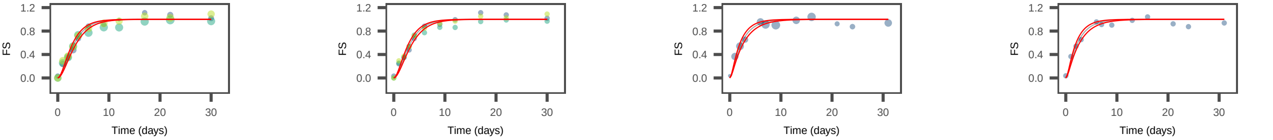

PCCA

AA Weighted R2: 0.91 k: 0.111 dk: 0.0324
AA Unweighted R2: 0.92 k: 0.102 dk: 0.0265
HW Weighted R2: 0.88 k: 0.1 dk: 0.0325
HW Unweighted R2: 0.89 k: 0.101 dk: 0.0308

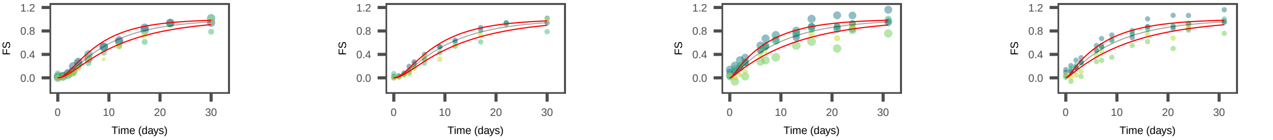

PCCB

AA Weighted R2: 0.95 k: 0.122 dk: 0.0278
AA Unweighted R2: 0.95 k: 0.125 dk: 0.0314
HW Weighted R2: 0.95 k: 0.147 dk: 0.026
HW Unweighted R2: 0.95 k: 0.149 dk: 0.0266

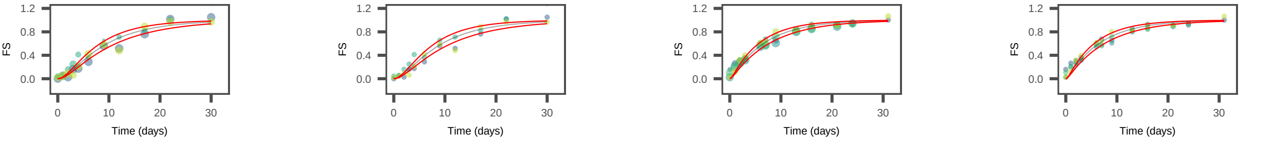

PCKGC

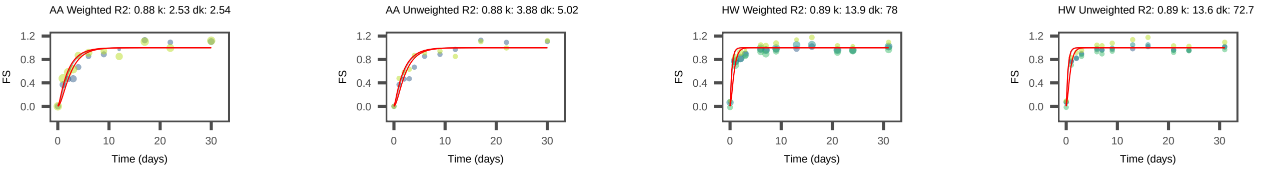

PCY2

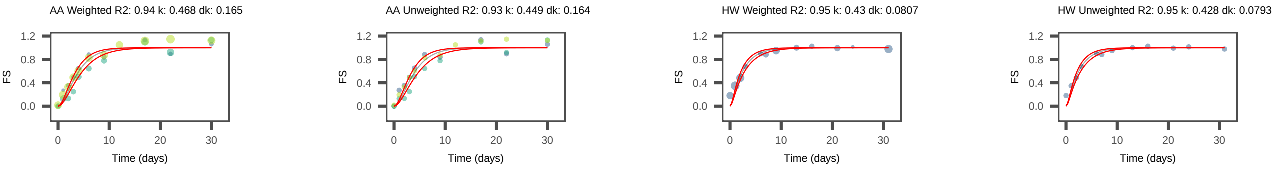

PCYOX

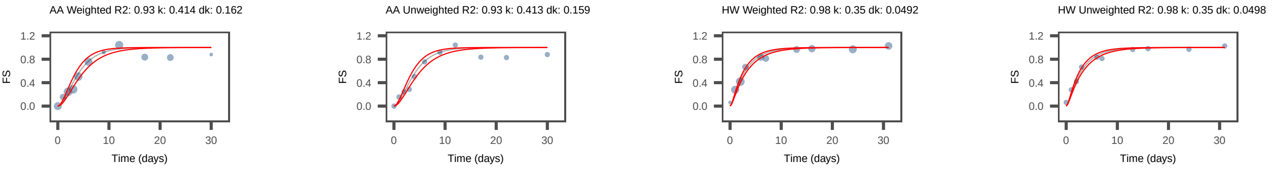

PDC6I

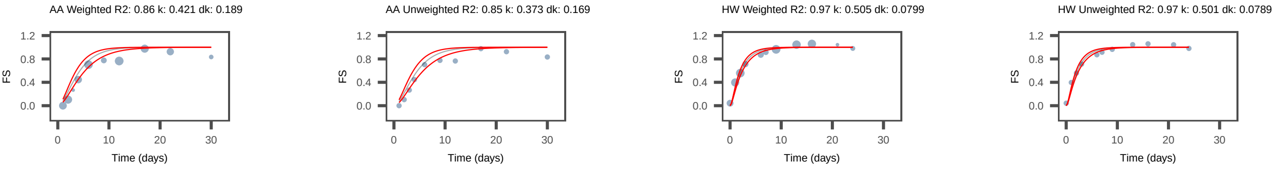

PDIA1

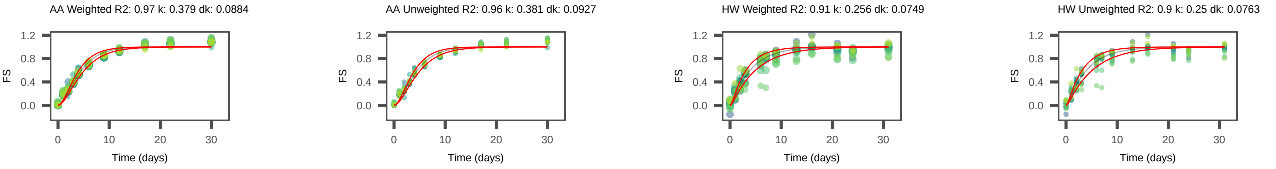

PDIA3

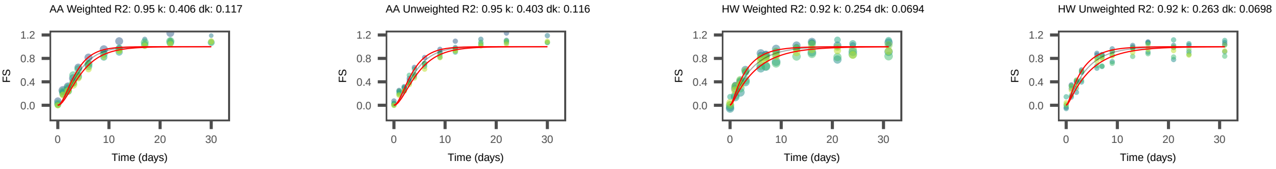

PDIA4

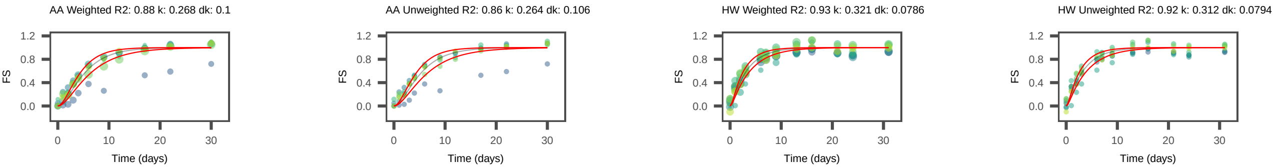

PDIA5

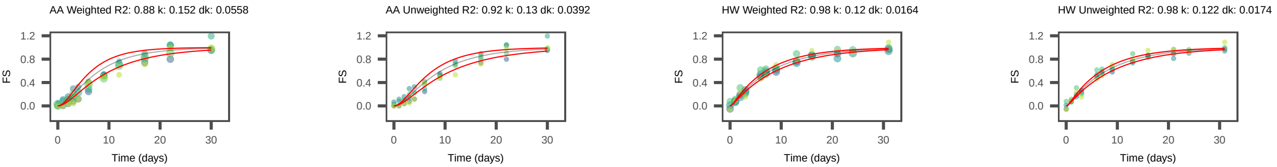

PDIA6

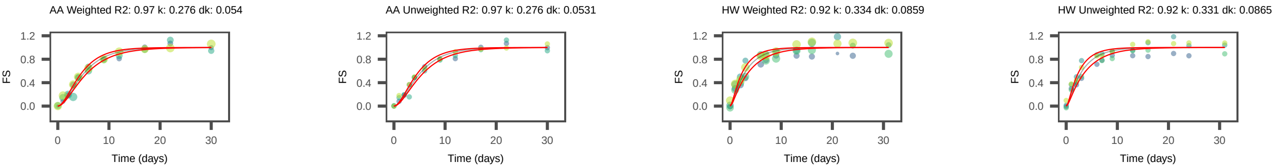

PDXK

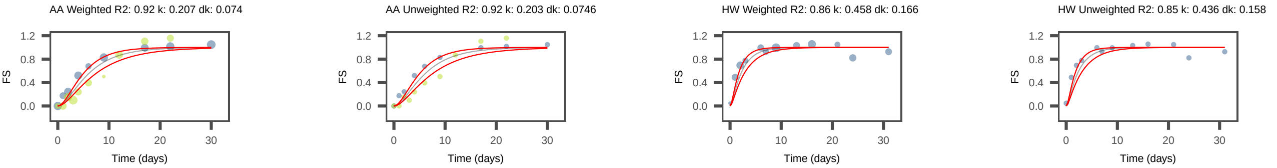

PEBP1

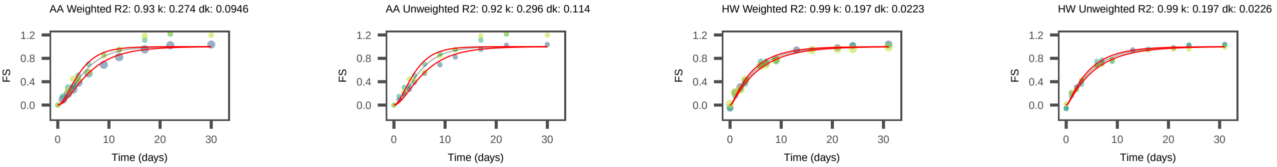

PECR

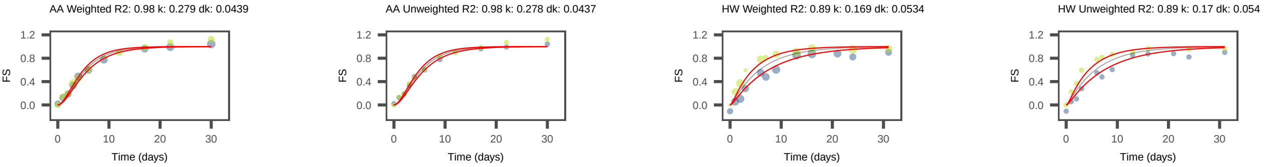

PGAM1

AA Weighted R2: 0.88 k: 0.211 dk: 0.0866

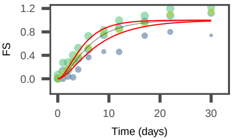

AA Unweighted R2: 0.87 k: 0.192 dk: 0.084

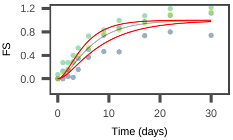

HW Weighted R2: 0.97 k: 0.189 dk: 0.0349

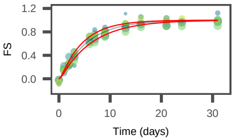

HW Unweighted R2: 0.96 k: 0.195 dk: 0.0402

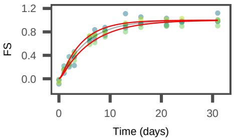

PGBM

AA Weighted R2: 0.98 k: 0.0808 dk: 0.0118

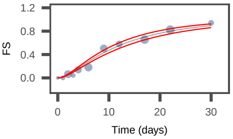

AA Unweighted R2: 0.98 k: 0.081 dk: 0.0117

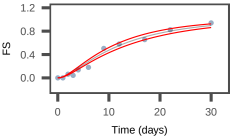

HW Weighted R2: 0.97 k: 0.0832 dk: 0.0113

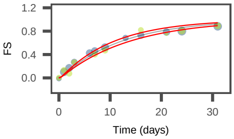

HW Unweighted R2: 0.97 k: 0.0835 dk: 0.0114

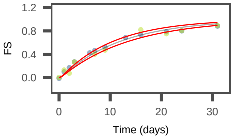

PGK1

AA Weighted R2: 0.97 k: 0.205 dk: 0.0382

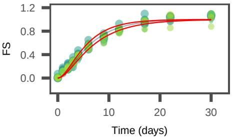

AA Unweighted R2: 0.97 k: 0.2 dk: 0.0381

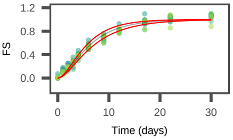

HW Weighted R2: 0.96 k: 0.158 dk: 0.0296

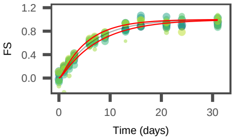

HW Unweighted R2: 0.95 k: 0.155 dk: 0.0313

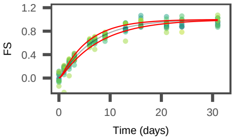

PGM1

AA Weighted R2: 0.95 k: 0.202 dk: 0.0495

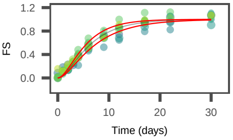

AA Unweighted R2: 0.95 k: 0.203 dk: 0.0504

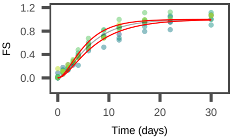

HW Weighted R2: 0.95 k: 0.178 dk: 0.0361

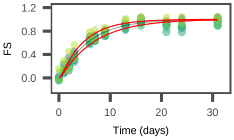

HW Unweighted R2: 0.95 k: 0.18 dk: 0.0368

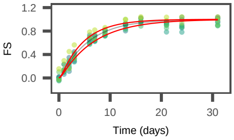

PHB

AA Weighted R2: 0.94 k: 0.129 dk: 0.0316

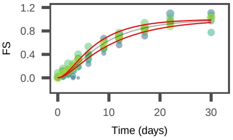

AA Unweighted R2: 0.94 k: 0.127 dk: 0.0338

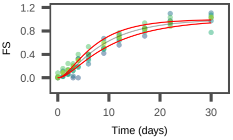

HW Weighted R2: 0.9 k: 0.0942 dk: 0.0273

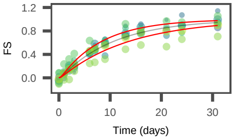

HW Unweighted R2: 0.9 k: 0.0958 dk: 0.0284

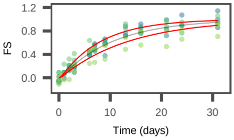

PHB2

AA Weighted R2: 0.96 k: 0.104 dk: 0.0196

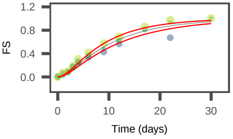

AA Unweighted R2: 0.96 k: 0.103 dk: 0.019

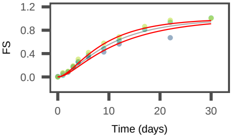

HW Weighted R2: 0.97 k: 0.103 dk: 0.0151

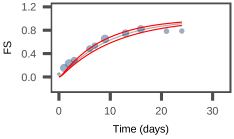

HW Unweighted R2: 0.97 k: 0.104 dk: 0.0163

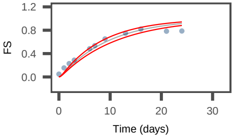

PHS

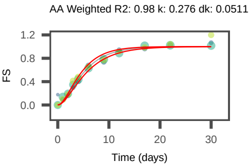

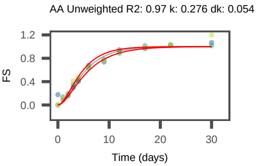

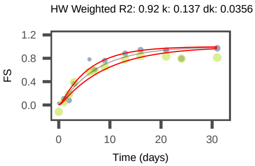

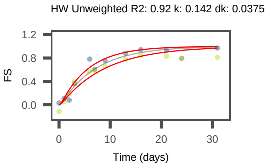

PICAL

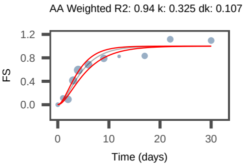

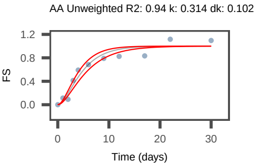

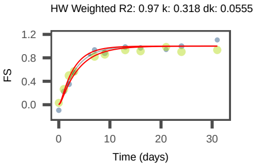

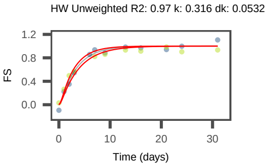

PLAP

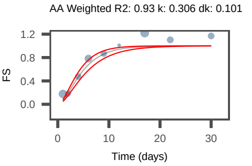

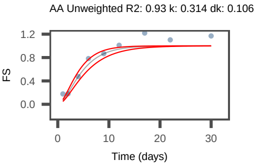

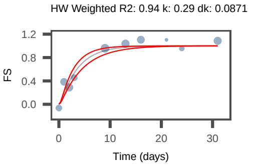

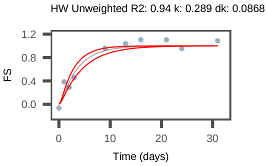

PLCB

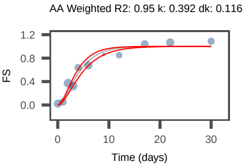

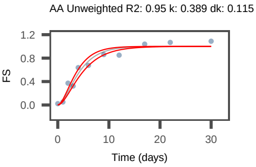

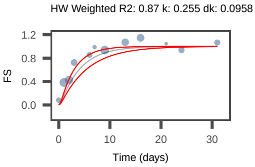

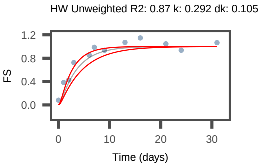

PLPHP

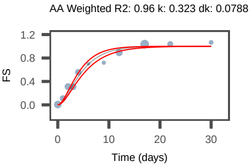

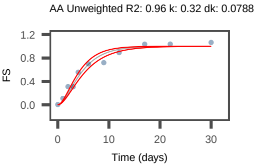

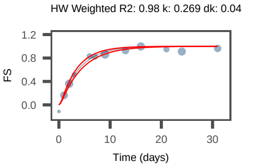

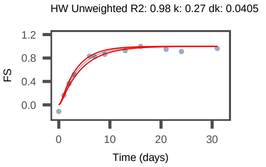

PLSL

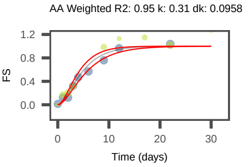

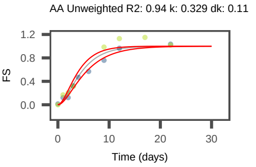

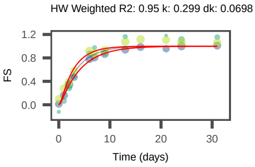

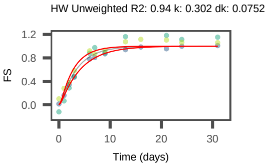

PLST

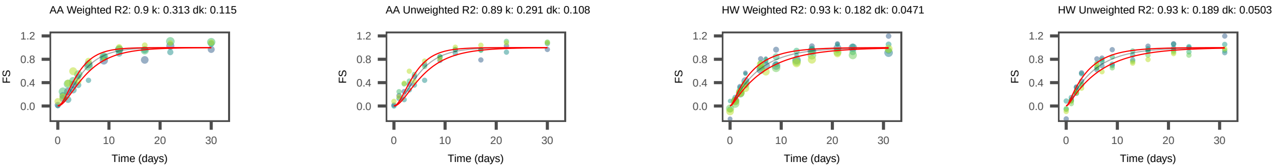

PNCB

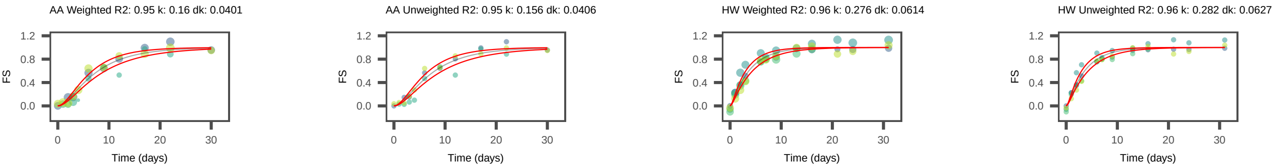

PON1

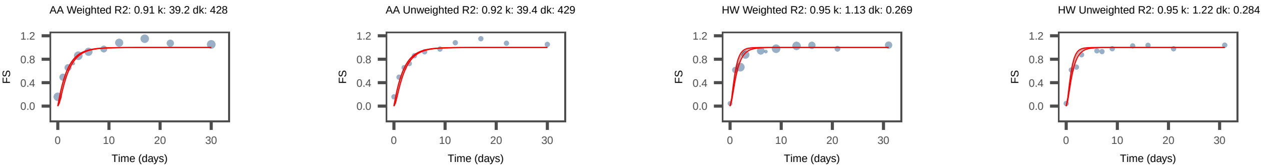

PON2

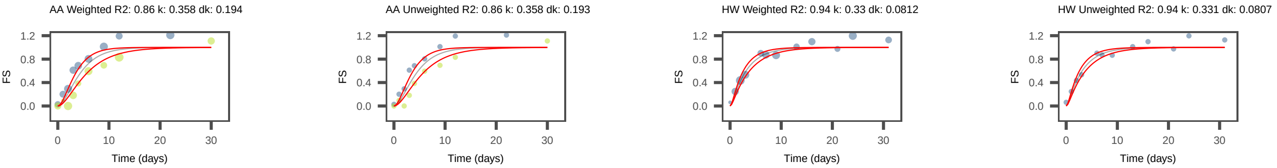

PPIB

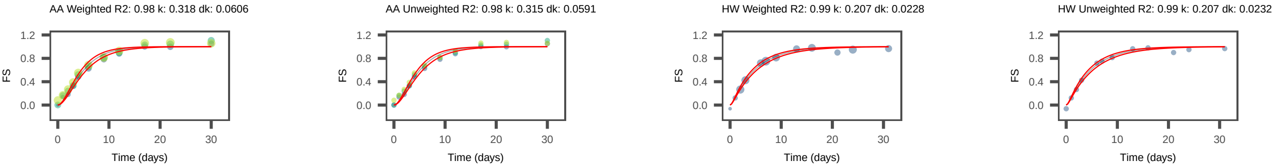

PPID

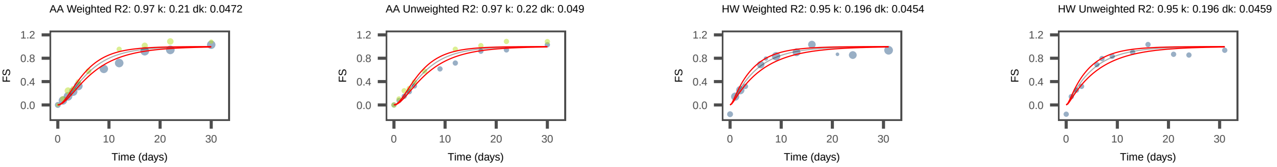

PRDX1

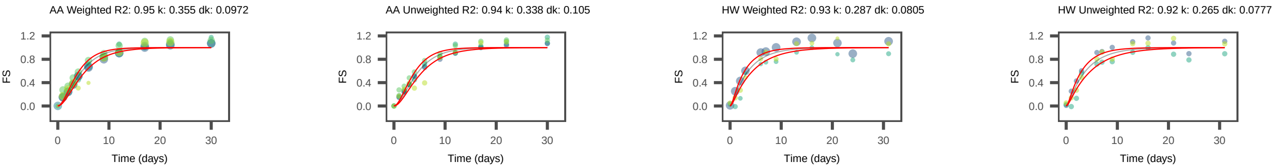

PRDX2

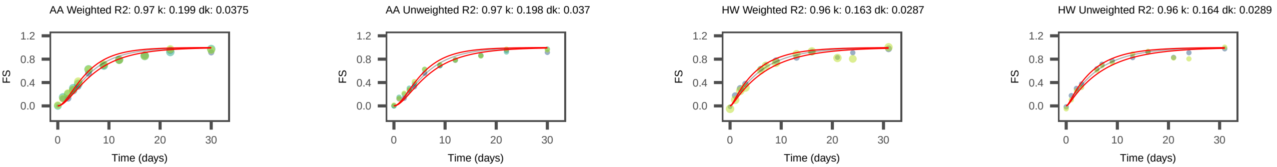

PRDX3

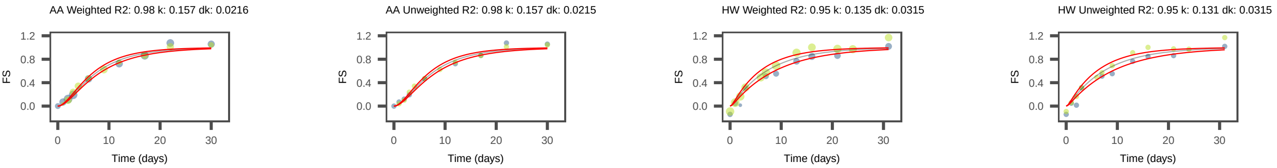

PRDX4

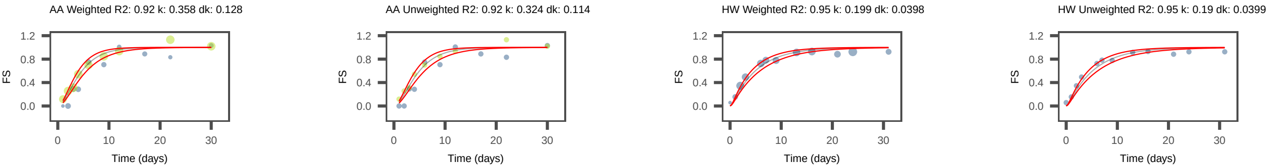

PRDX5

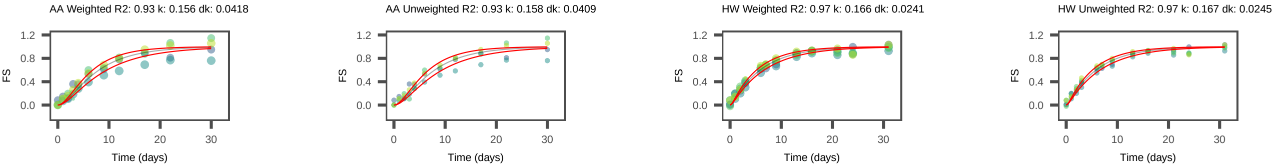

PRDX6

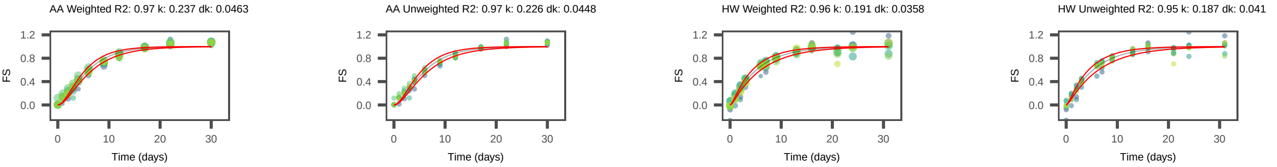

PROD

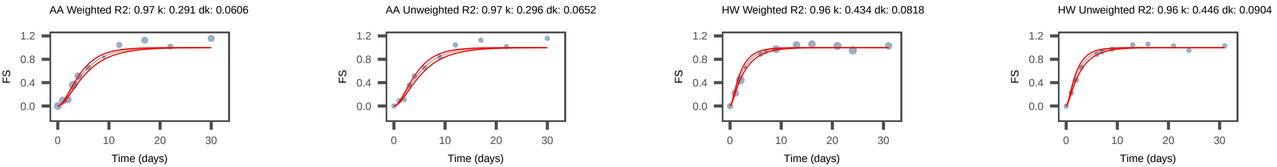

PROF1

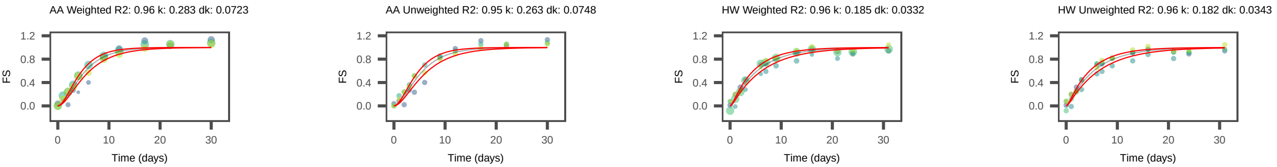

PRRC1

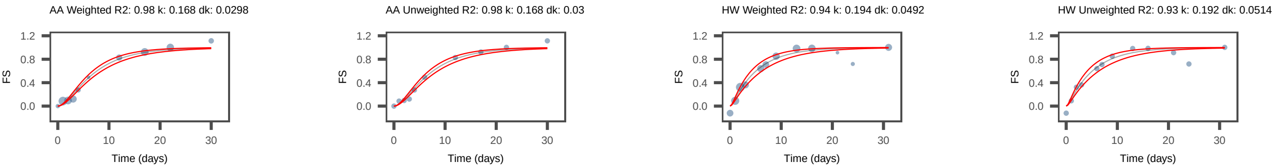

PSA1

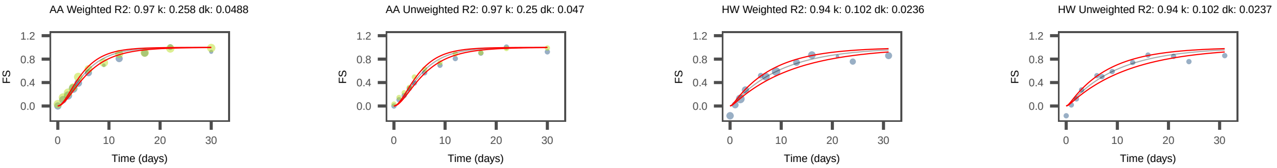

PSA2

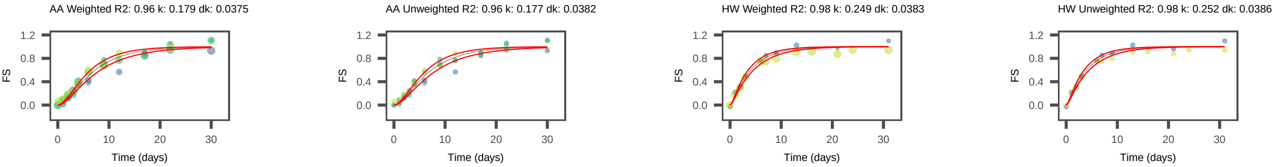

PSA4

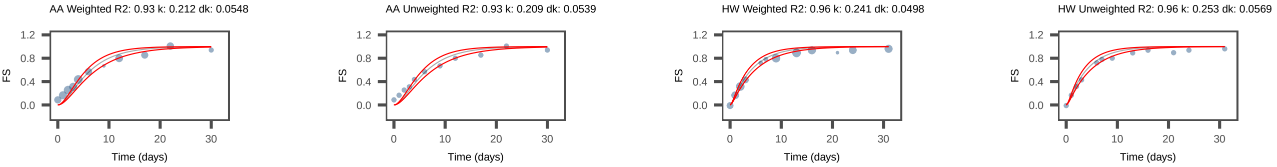

PSA6

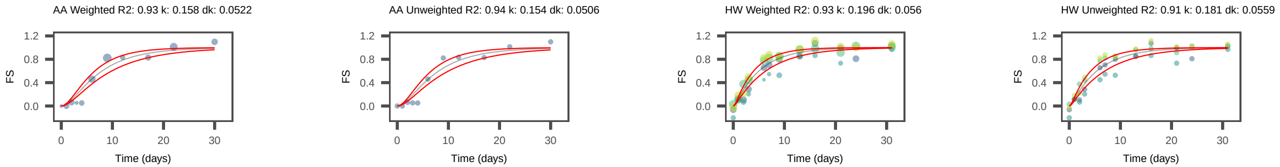

PSB4

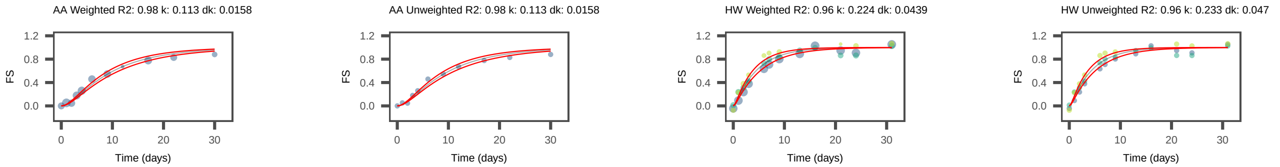

PSB5

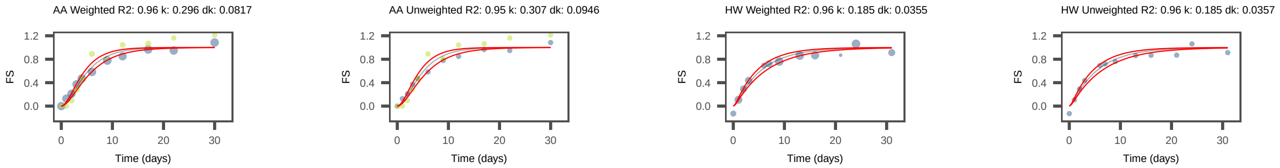

PSD11

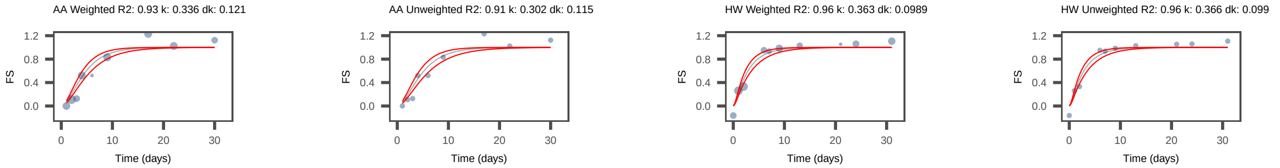

PSMD2

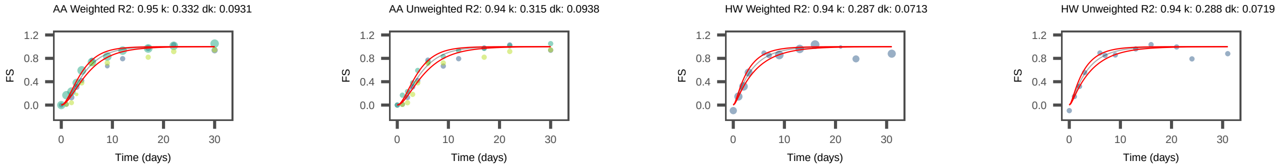

PSMD6

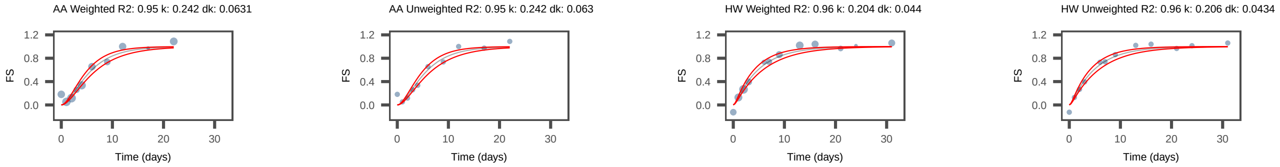

PSME1

AA Weighted R2: 0.9 k: 0.309 dk: 0.112 | AA Unweighted R2: 0.91 k: 0.295 dk: 0.104 | HW Weighted R2: 0.93 k: 0.342 dk: 0.0987 | HW Unweighted R2: 0.93 k: 0.349 dk: 0.105

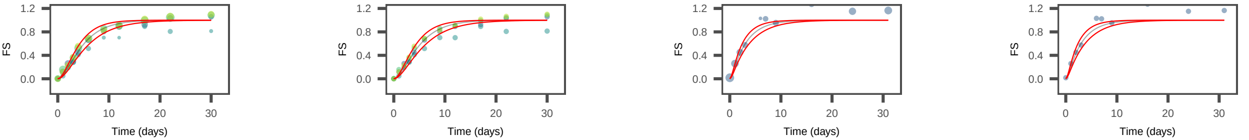

PTBP1

AA Weighted R2: 0.87 k: 0.301 dk: 0.142 | AA Unweighted R2: 0.88 k: 0.298 dk: 0.138 | HW Weighted R2: 0.94 k: 0.24 dk: 0.0556 | HW Unweighted R2: 0.94 k: 0.24 dk: 0.0558

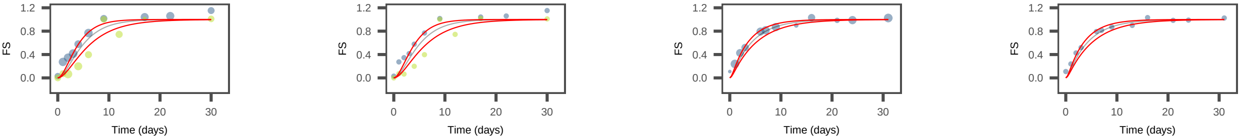

PTER

AA Weighted R2: 0.98 k: 0.165 dk: 0.0262 | AA Unweighted R2: 0.98 k: 0.164 dk: 0.0258 | HW Weighted R2: 0.99 k: 0.198 dk: 0.0181 | HW Unweighted R2: 0.99 k: 0.197 dk: 0.0178

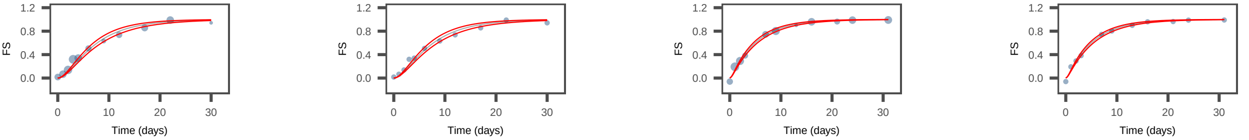

PTGR3

AA Weighted R2: 0.92 k: 0.183 dk: 0.0621 | AA Unweighted R2: 0.93 k: 0.18 dk: 0.0595 | HW Weighted R2: 0.95 k: 0.228 dk: 0.0496 | HW Unweighted R2: 0.94 k: 0.242 dk: 0.0594

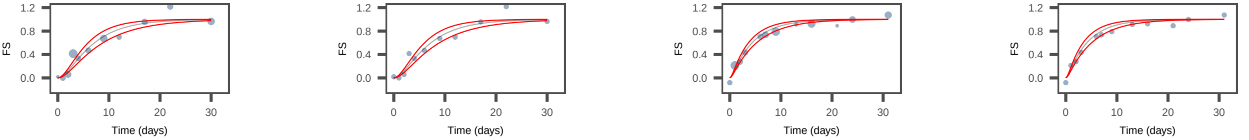

PTH2

AA Weighted R2: 0.96 k: 0.202 dk: 0.0489 | AA Unweighted R2: 0.94 k: 0.19 dk: 0.0544 | HW Weighted R2: 0.96 k: 0.232 dk: 0.0451 | HW Unweighted R2: 0.96 k: 0.225 dk: 0.0434

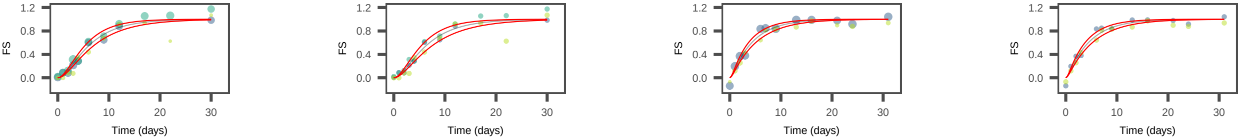

PURA

AA Weighted R2: 0.98 k: 0.108 dk: 0.0163 | AA Unweighted R2: 0.98 k: 0.108 dk: 0.0164 | HW Weighted R2: 0.95 k: 0.0716 dk: 0.0132 | HW Unweighted R2: 0.95 k: 0.0714 dk: 0.0132

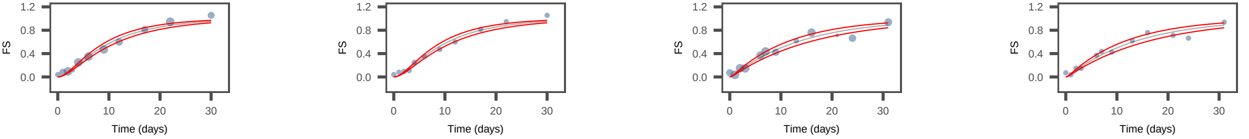

PXMP2

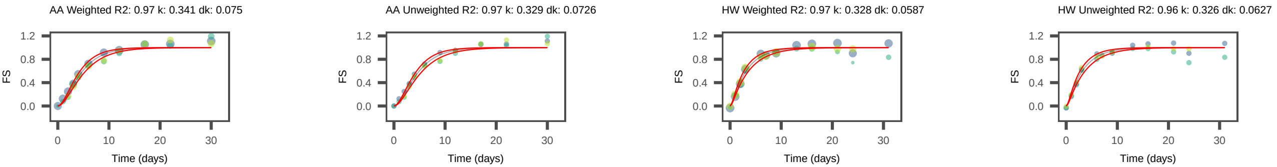

PXMP4

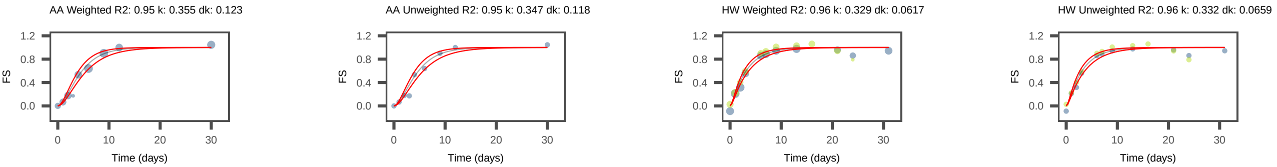

PYC

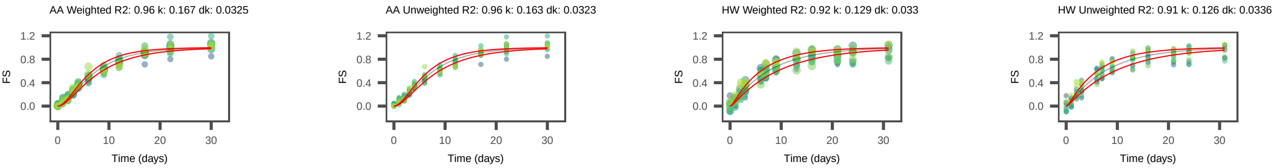

PYGL

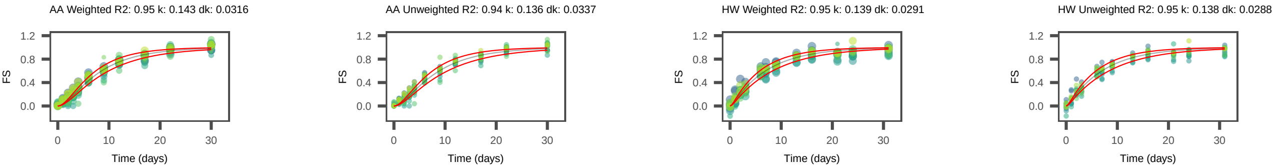

QCR1

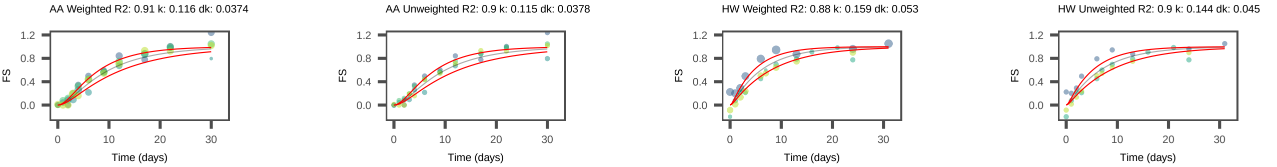

QCR10

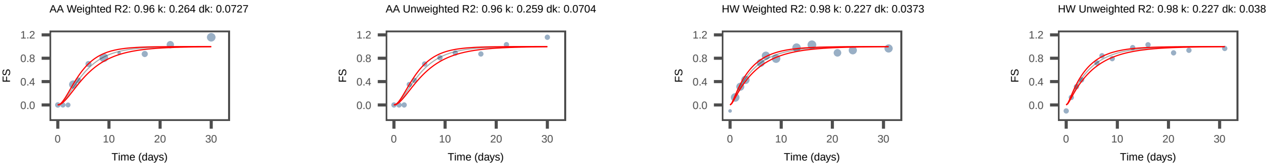

QCR2

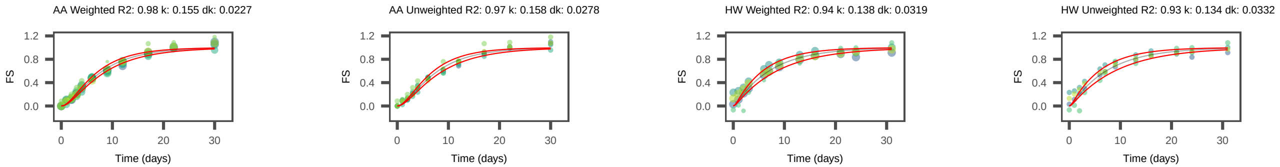

QOR

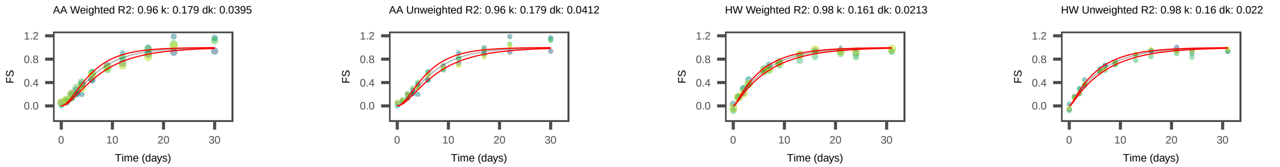

RAB18

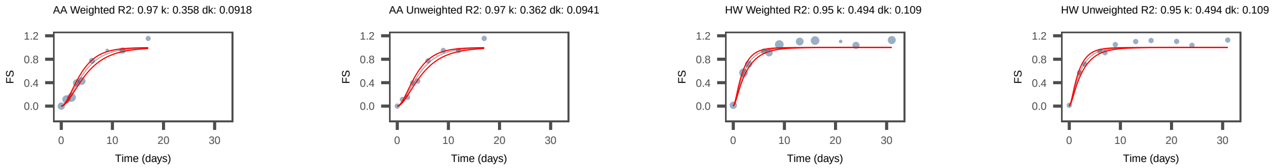

RACK1

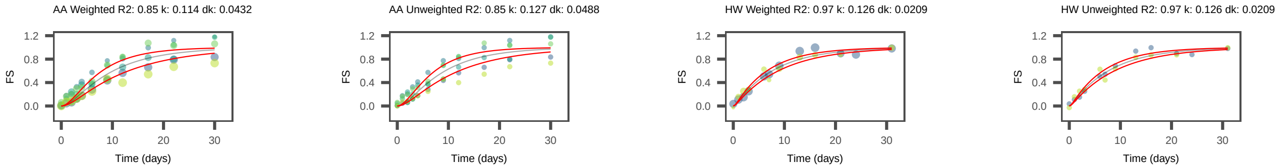

RADI

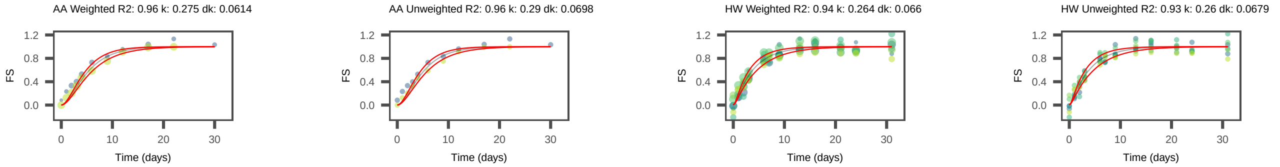

RDH7

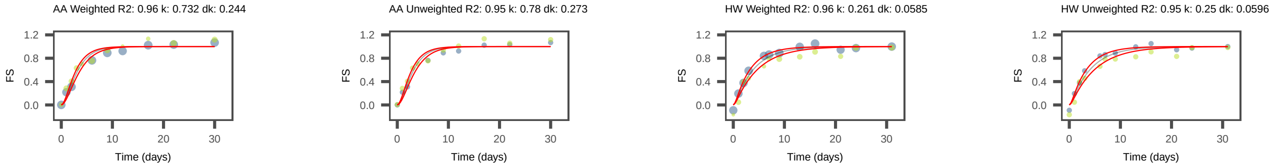

REEP5

AA Weighted R2: 0.98 k: 0.222 dk: 0.0374 | AA Unweighted R2: 0.98 k: 0.219 dk: 0.0357 | HW Weighted R2: 0.96 k: 0.236 dk: 0.0556 | HW Unweighted R2: 0.96 k: 0.237 dk: 0.0561

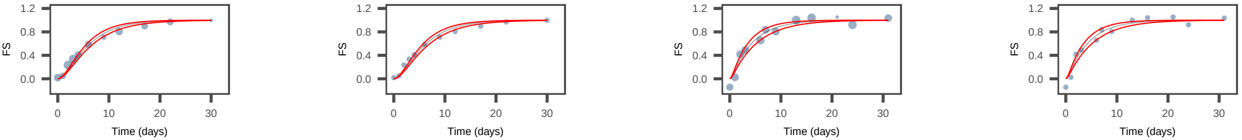

RENT1

AA Weighted R2: 0.79 k: 0.249 dk: 0.128 | AA Unweighted R2: 0.79 k: 0.268 dk: 0.14 | HW Weighted R2: 0.91 k: 1.24 dk: 0.333 | HW Unweighted R2: 0.91 k: 1.23 dk: 0.336

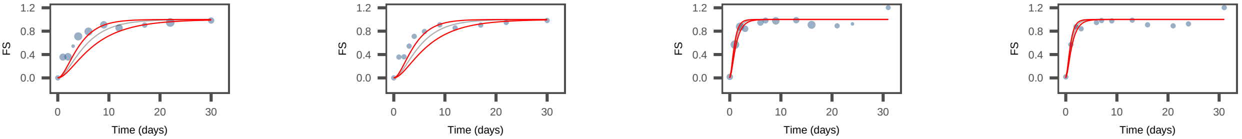

RGN

AA Weighted R2: 0.98 k: 0.202 dk: 0.03 | AA Unweighted R2: 0.98 k: 0.2 dk: 0.031 | HW Weighted R2: 0.98 k: 0.162 dk: 0.0201 | HW Unweighted R2: 0.98 k: 0.163 dk: 0.0225

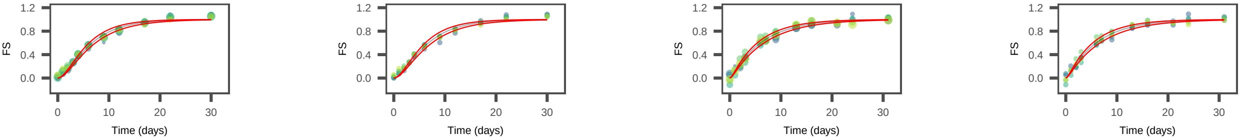

RHOA

AA Weighted R2: 0.95 k: 0.562 dk: 0.161 | AA Unweighted R2: 0.96 k: 0.561 dk: 0.153 | HW Weighted R2: 0.97 k: 0.432 dk: 0.0828 | HW Unweighted R2: 0.97 k: 0.428 dk: 0.0823

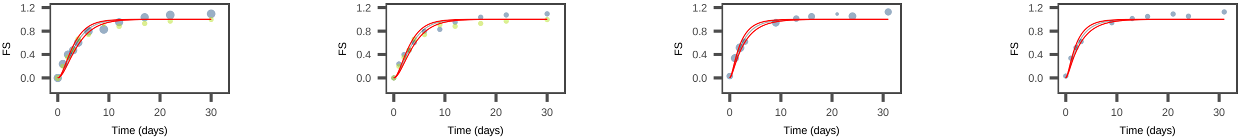

RIDA

AA Weighted R2: 0.97 k: 0.178 dk: 0.0345 | AA Unweighted R2: 0.96 k: 0.17 dk: 0.0371 | HW Weighted R2: 0.97 k: 0.157 dk: 0.0248 | HW Unweighted R2: 0.97 k: 0.156 dk: 0.0272

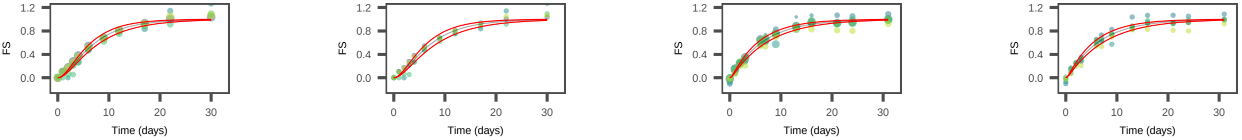

RINI

AA Weighted R2: 0.95 k: 0.332 dk: 0.0928 | AA Unweighted R2: 0.94 k: 0.37 dk: 0.112 | HW Weighted R2: 0.92 k: 0.453 dk: 0.135 | HW Unweighted R2: 0.9 k: 0.495 dk: 0.159

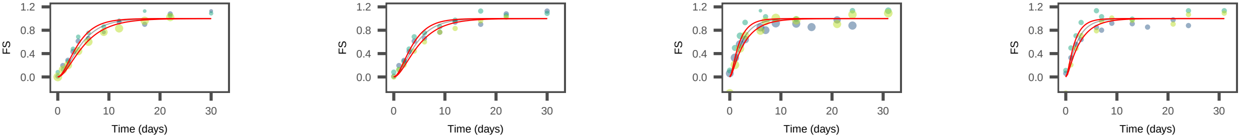

RISC

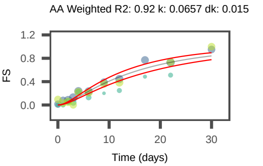

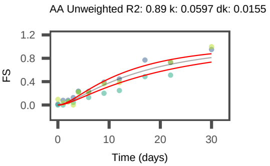

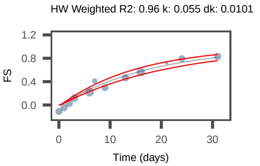

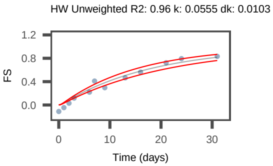

RL10

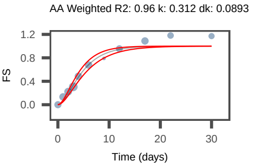

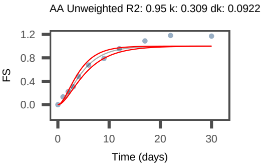

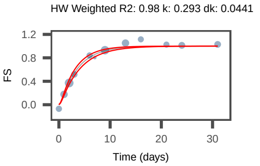

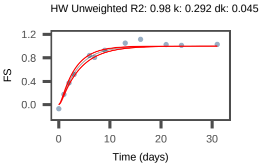

RL11

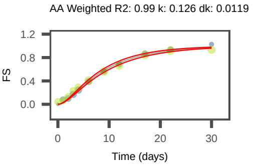

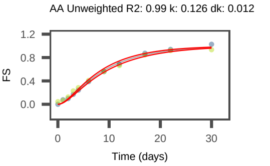

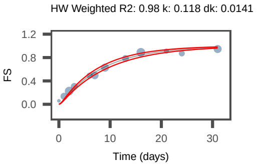

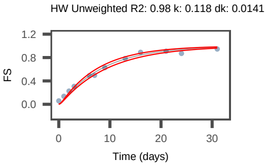

RL12

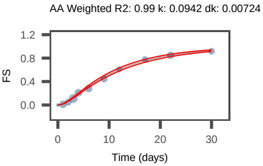

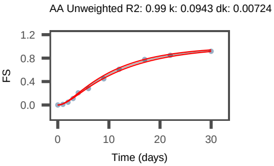

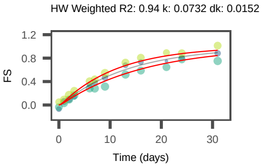

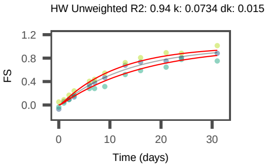

RL13A

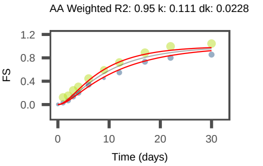

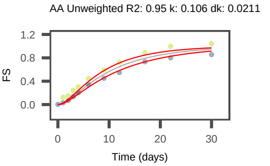

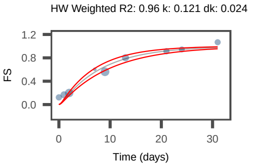

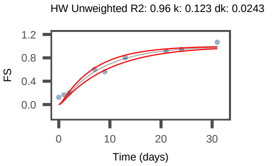

RL14

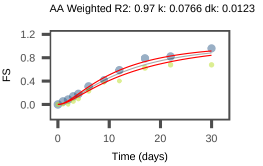

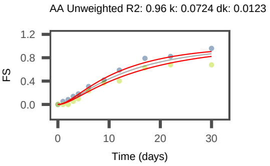

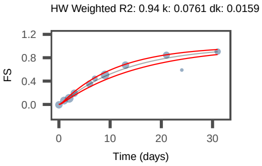

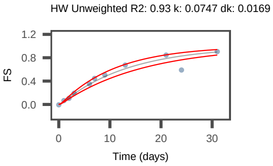

RL15

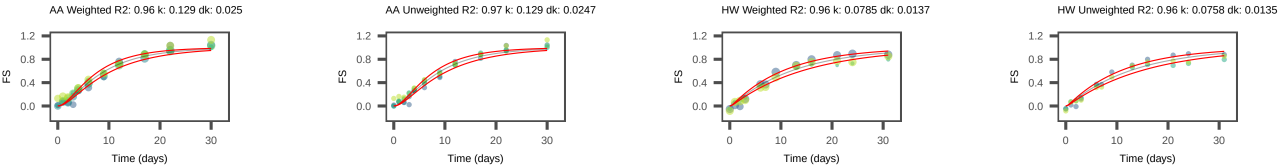

RL17

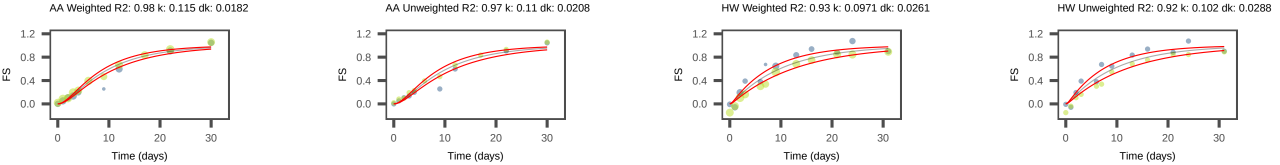

RL18

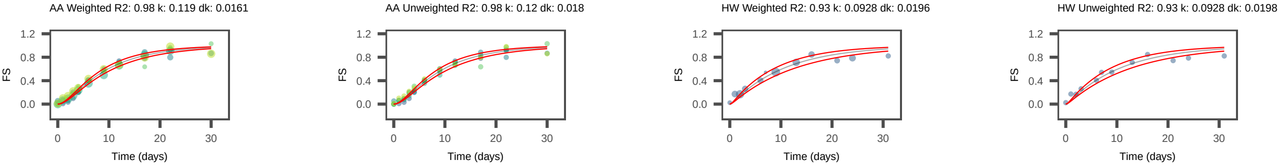

RL18A

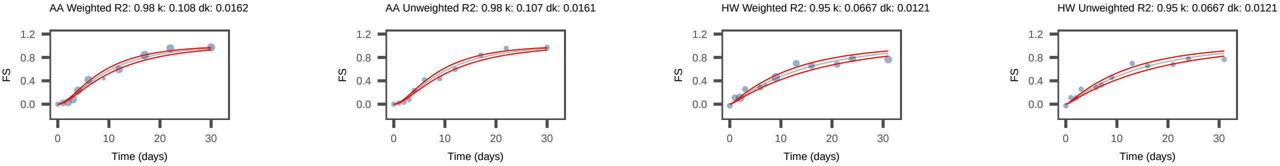

RL23

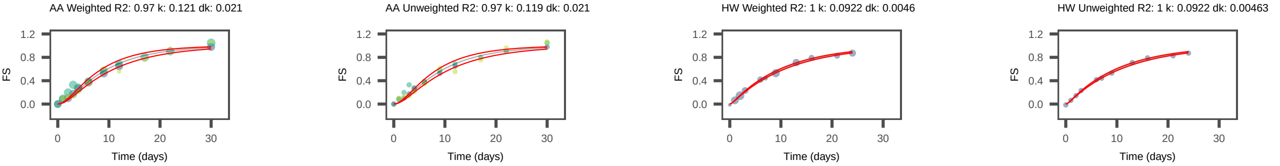

RL23A

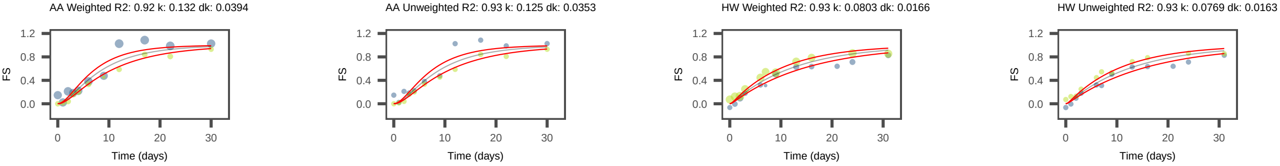

RL26

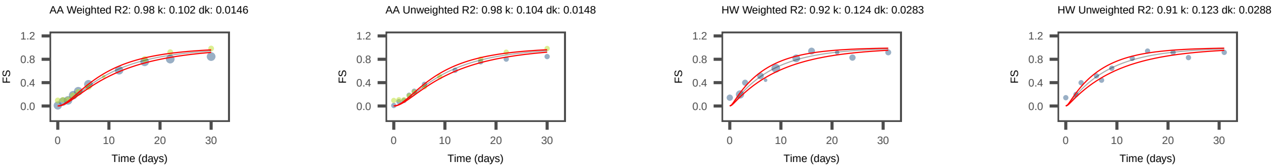

RL27A

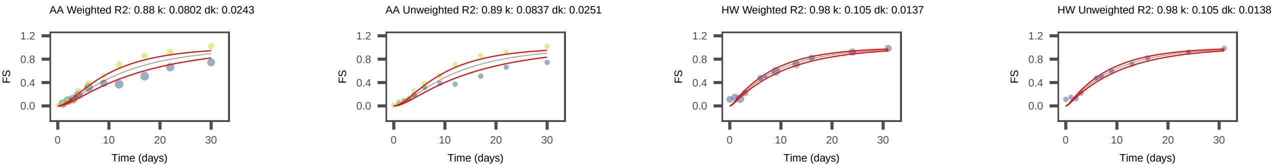

RL28

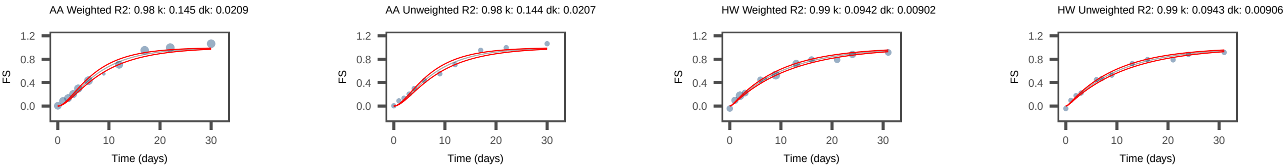

RL3

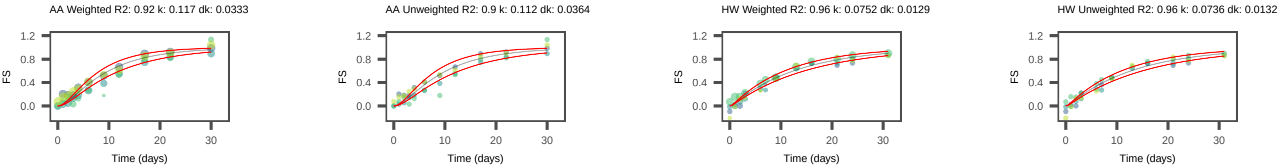

RL30

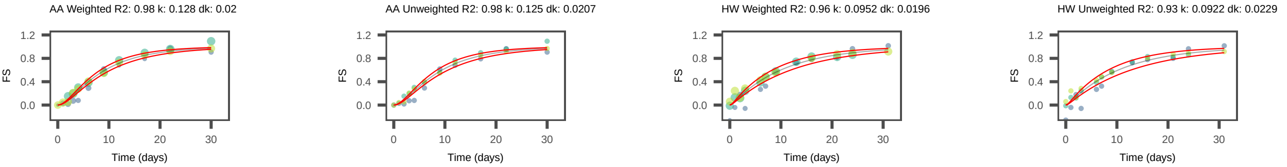

RL31

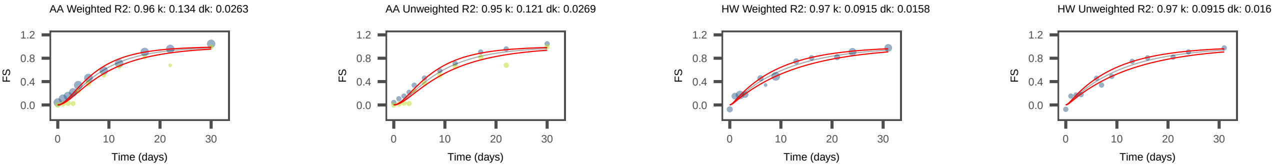

RL32

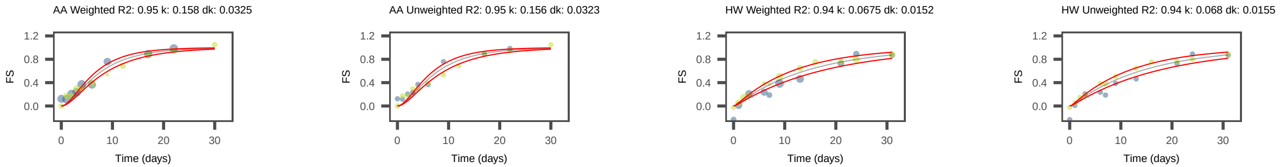

RL35

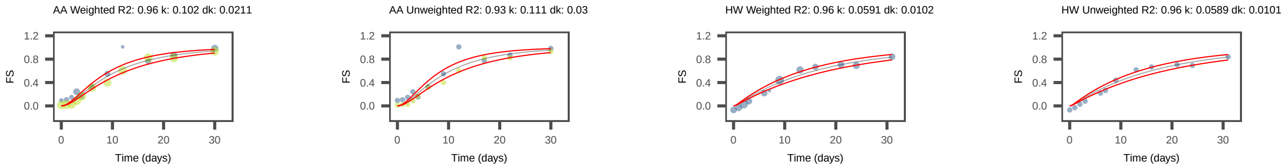

RL35A

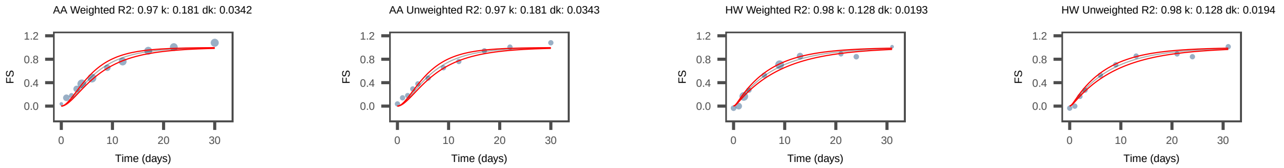

RL36A

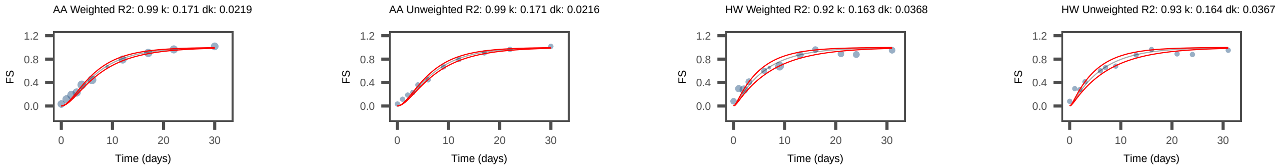

RL37A

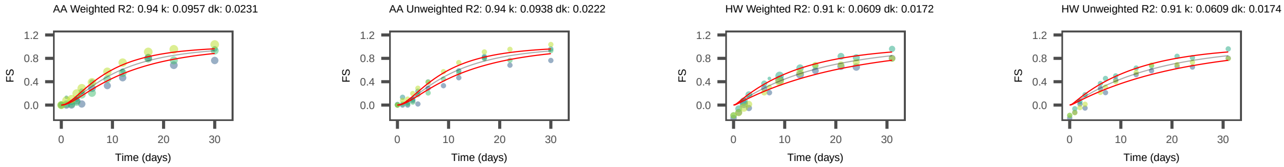

RL38

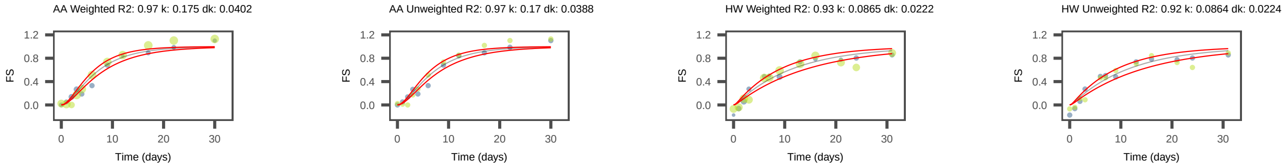

RL4

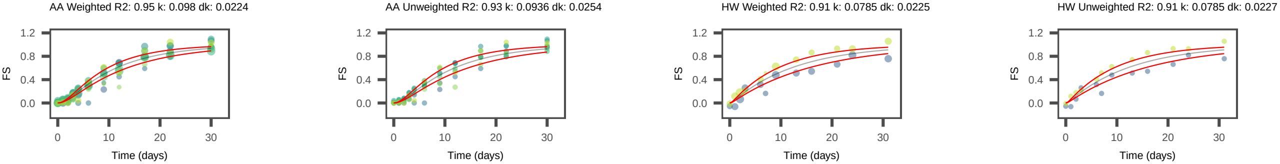

RL5

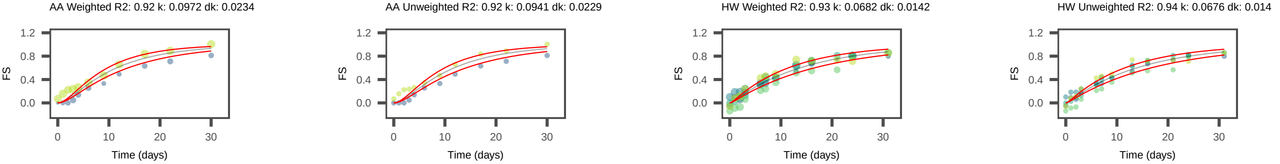

RL7

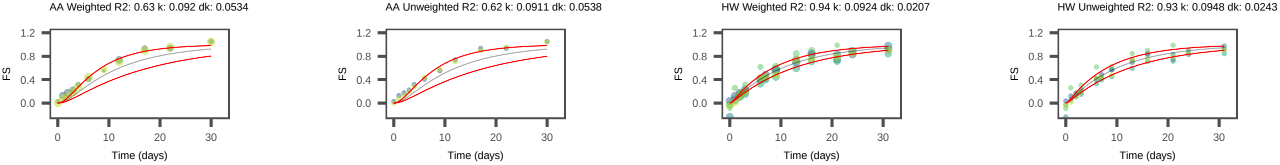

RL7A

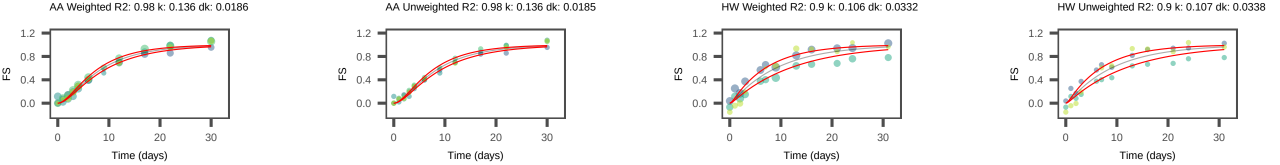

RL8

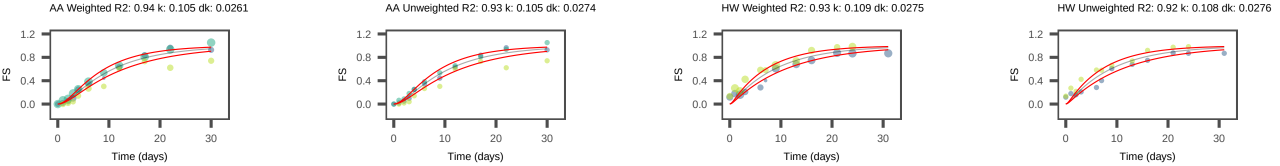

RL9

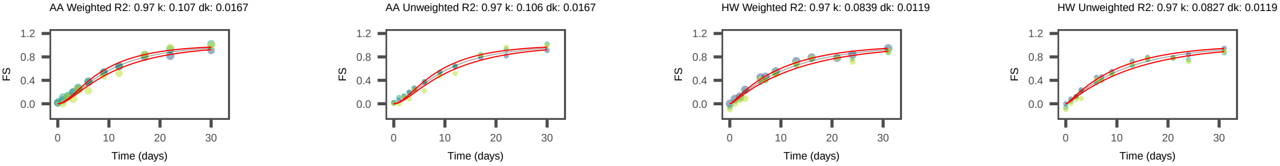

RLA0

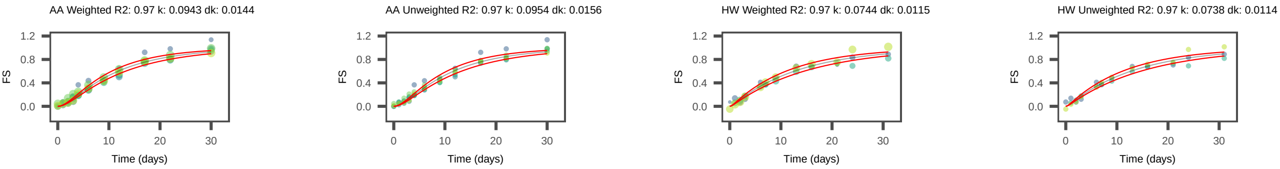

RLA1

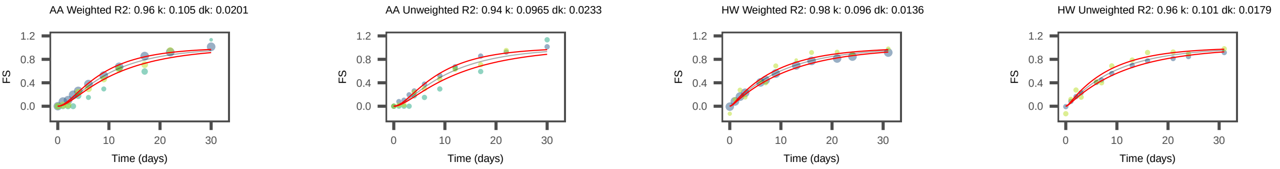

RLA2

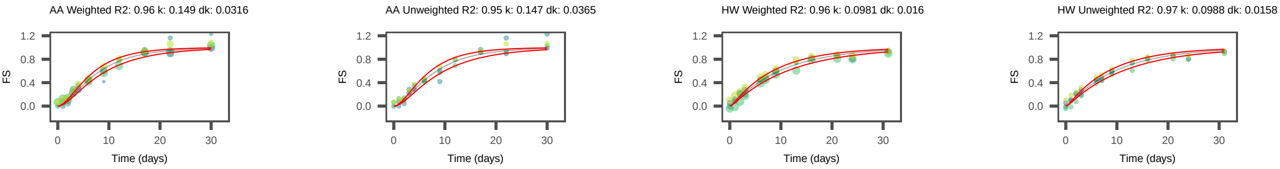

RMD1

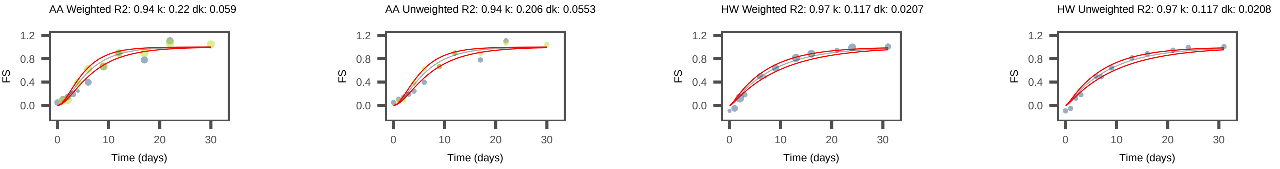

RMD2

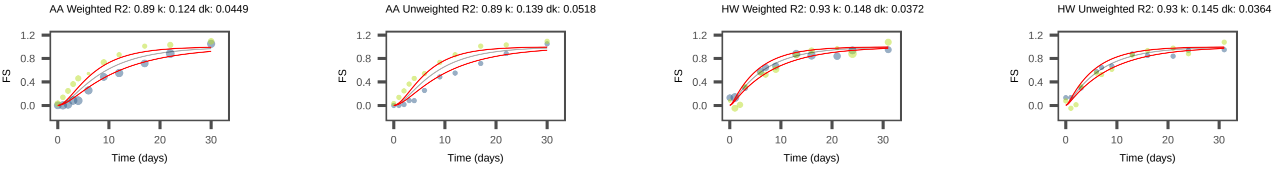

RMD3

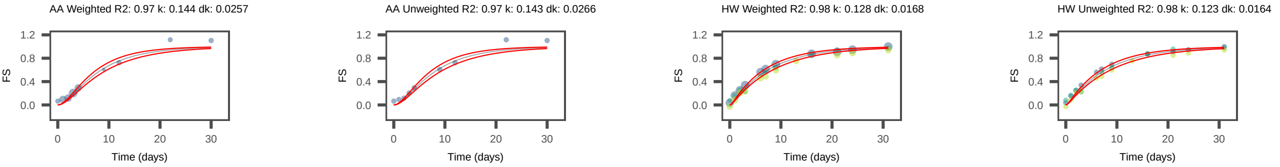

ROA2

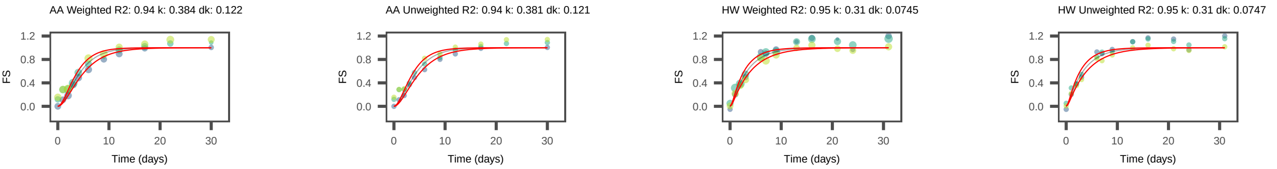

ROA3

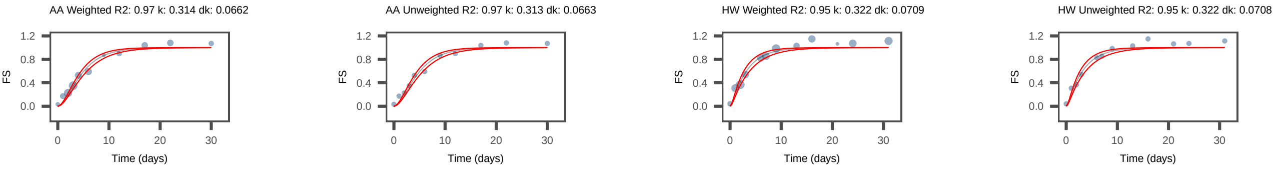

ROAA

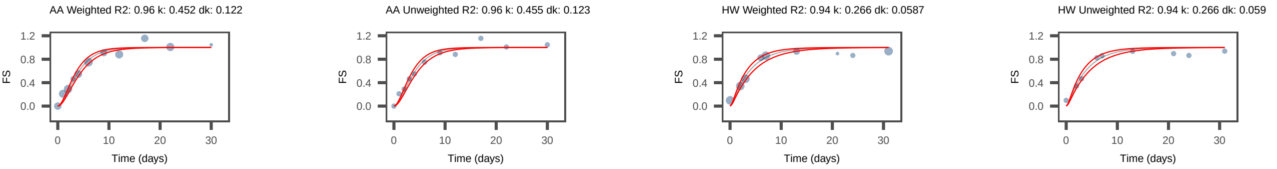

RPN1

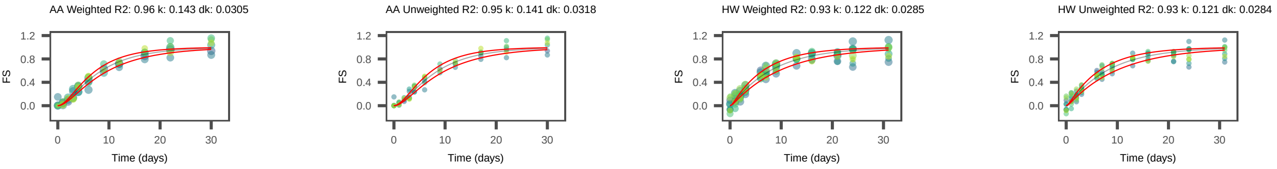

RPN2

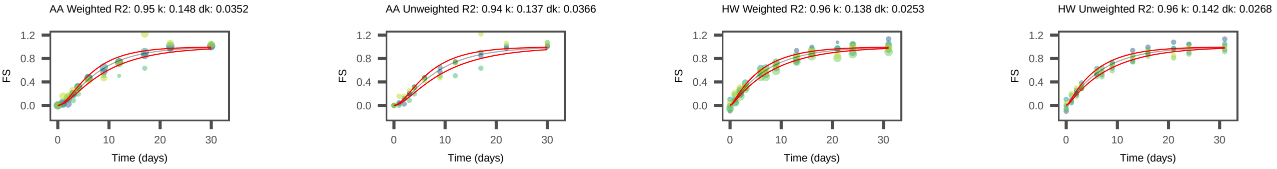

RRBP1

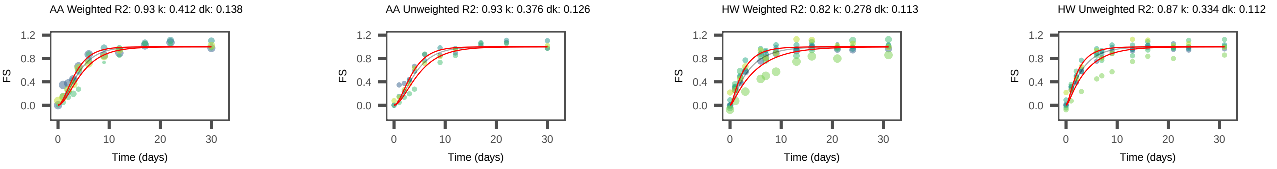

RS10

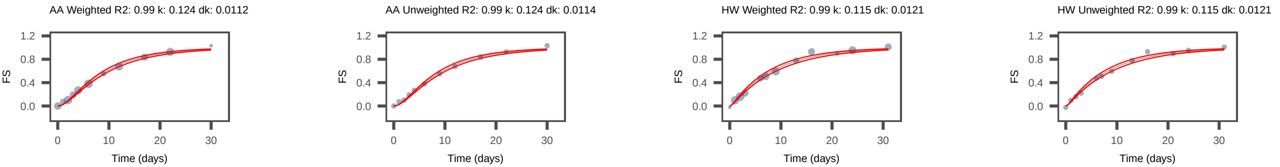

RS11

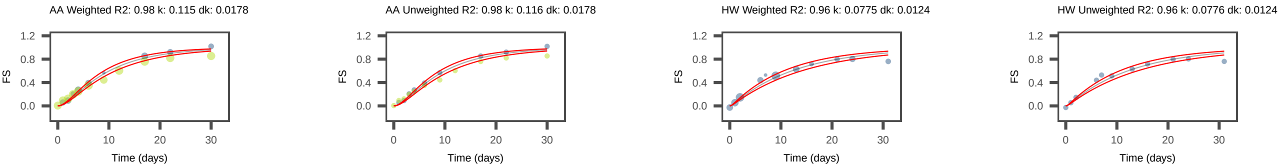

RS12

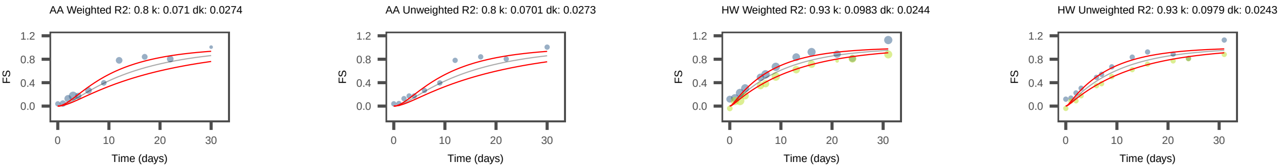

RS13

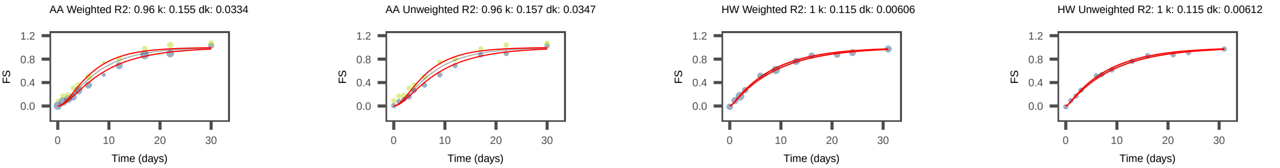

RS15

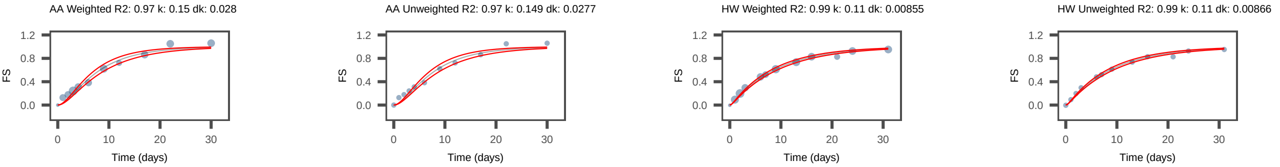

RS16

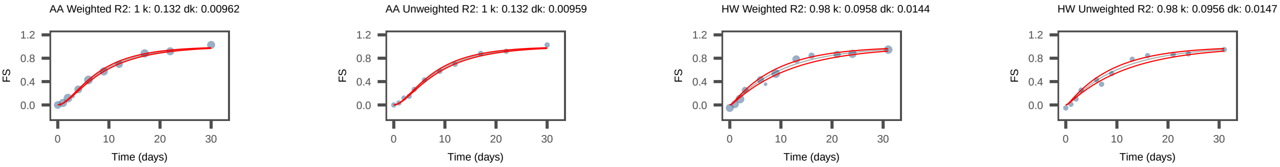

RS17

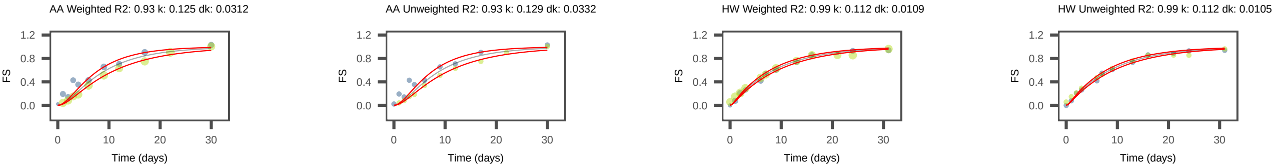

RS18

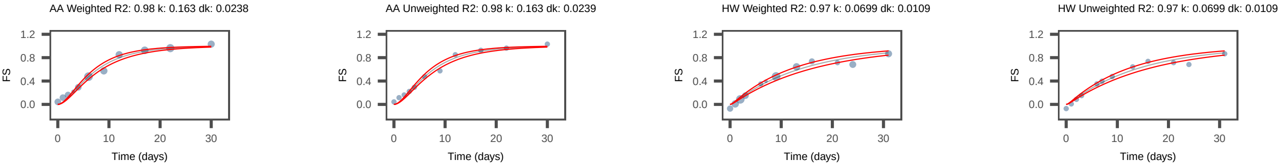

RS19

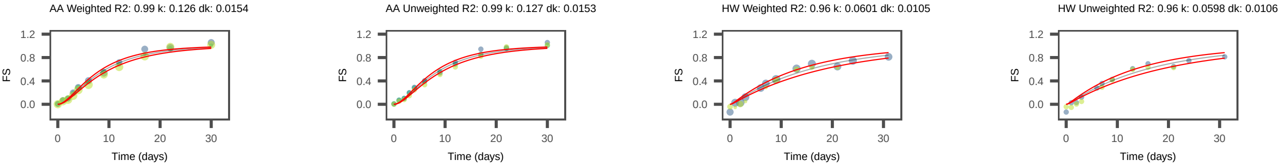

RS2

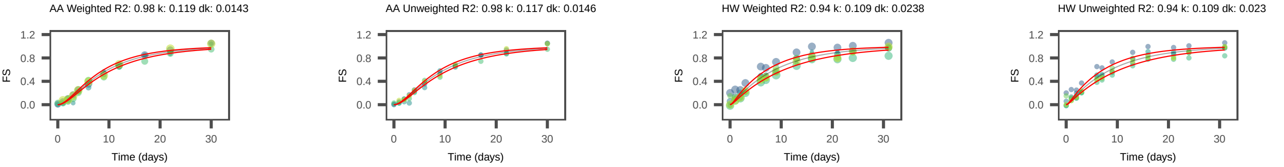

RS20

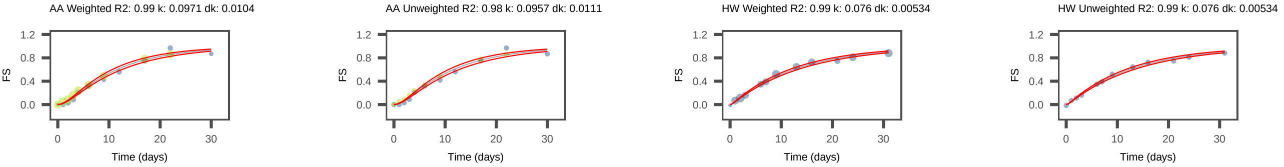

RS23

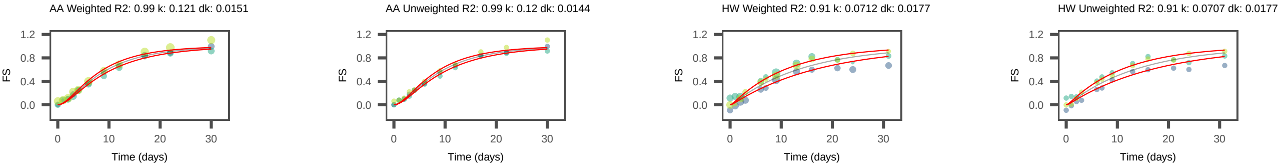

RS25

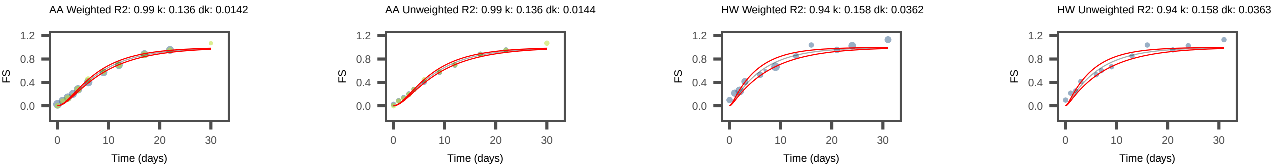

RS26

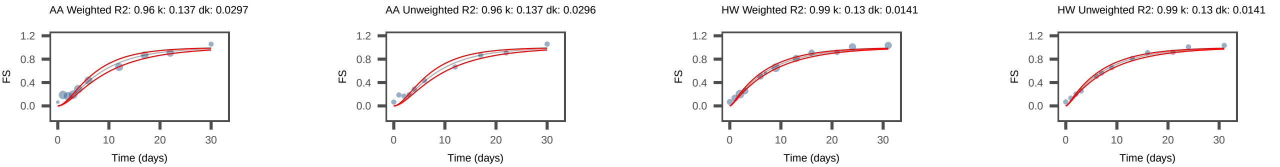

RS3

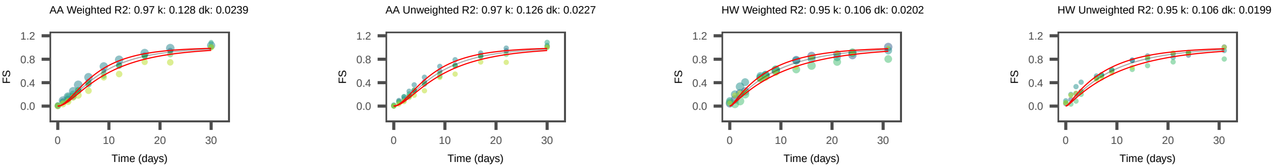

RS4X

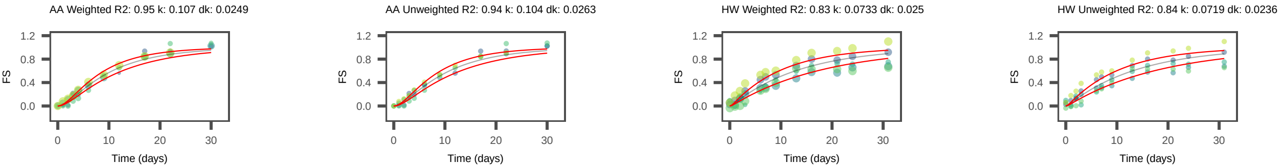

RS5

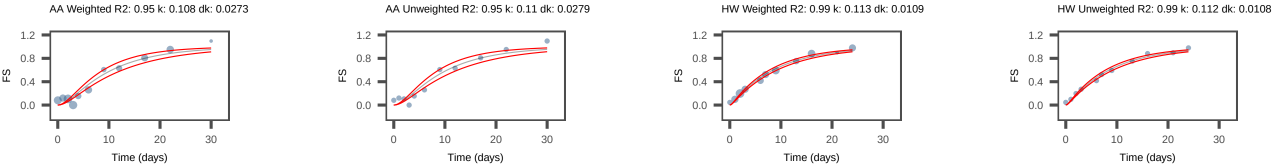

RS6

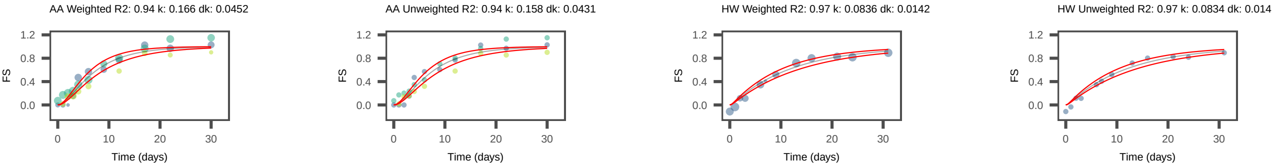

RS7

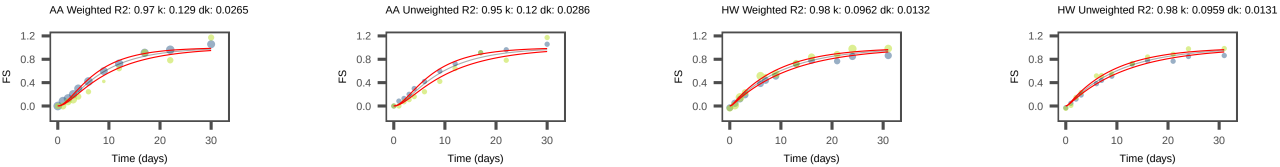

RS8

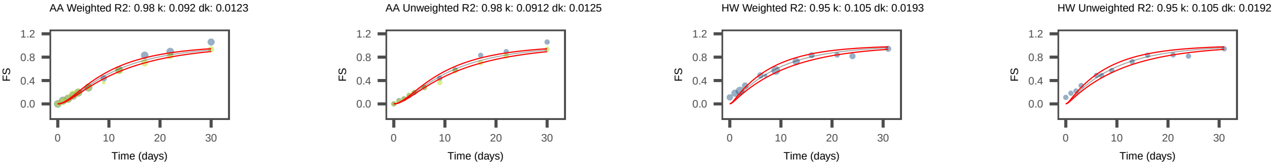

RSSA

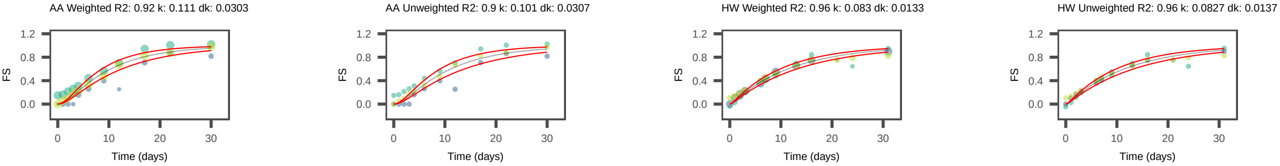

S10A1

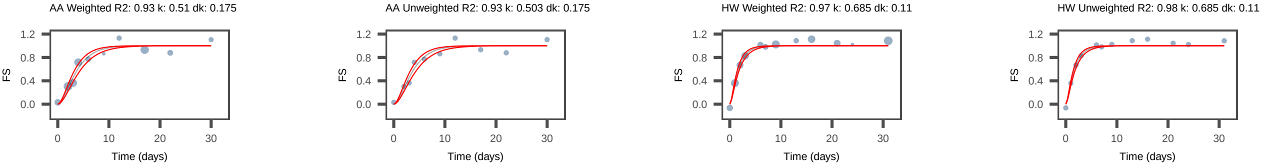

S14L2

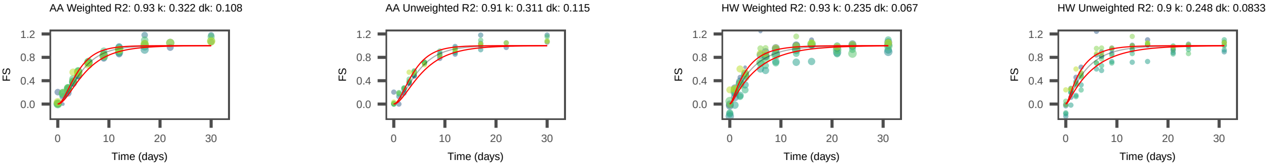

S14L4

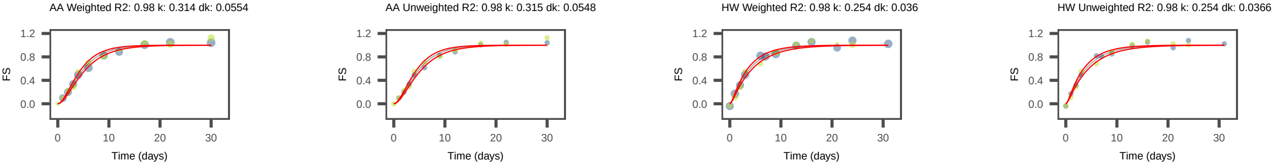

S27A2

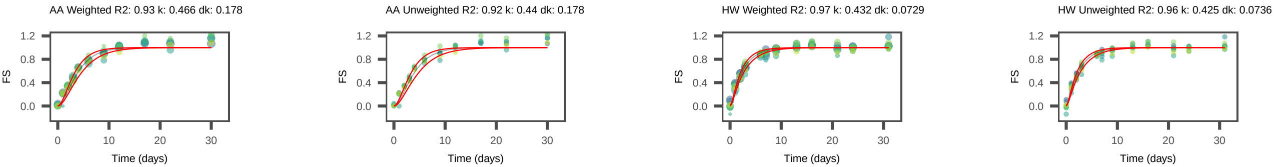

S27A5

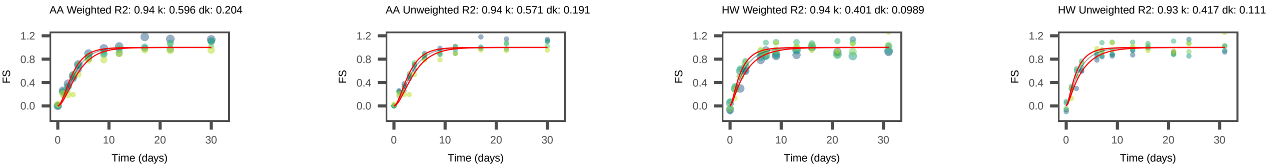

SAHH

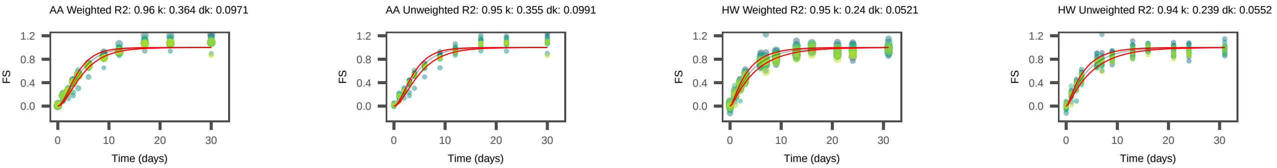

SAMH1

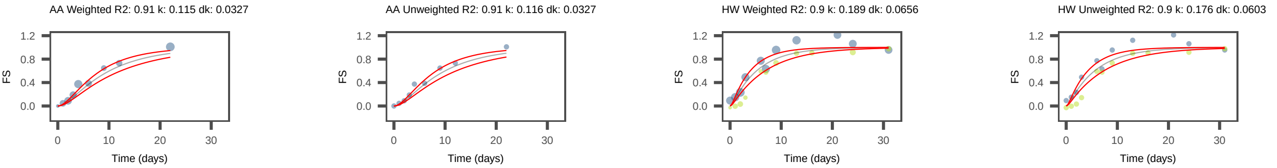

SAP

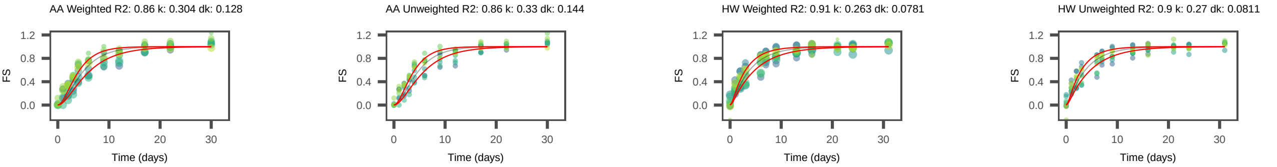

SARDH

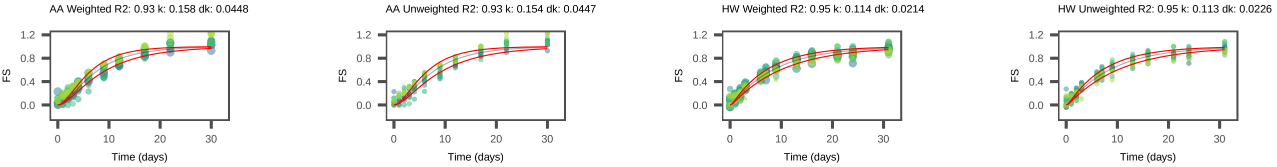

SBP1

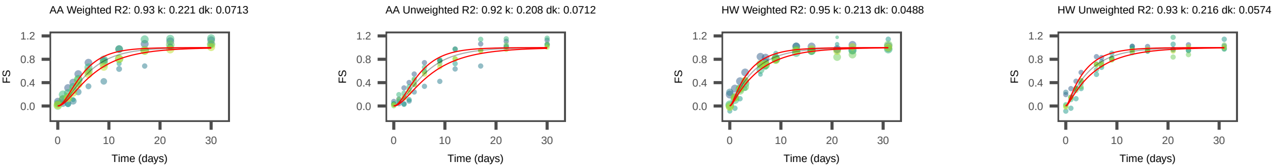

SC22B

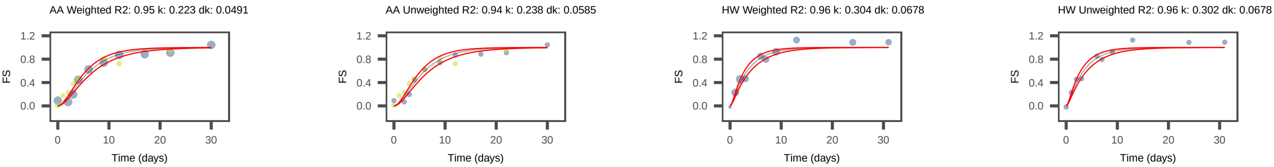

SC23A

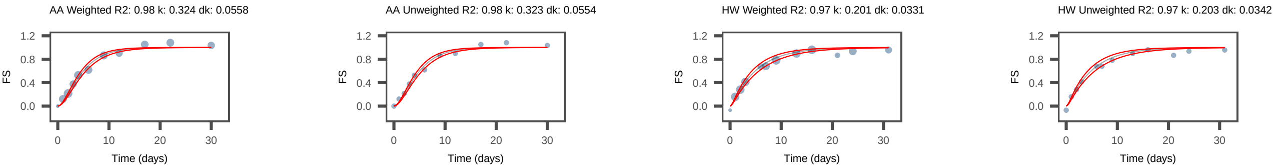

SC24A

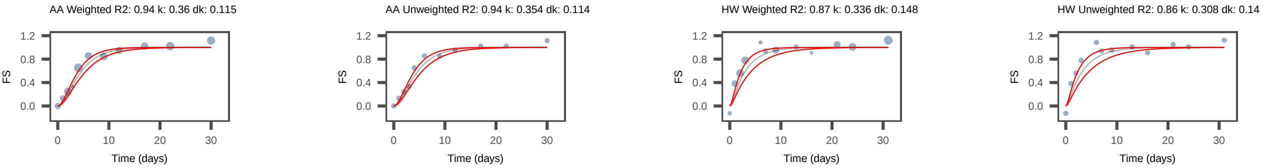

SC31A

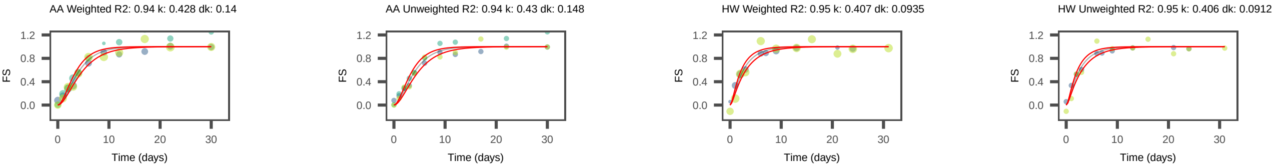

SCP2

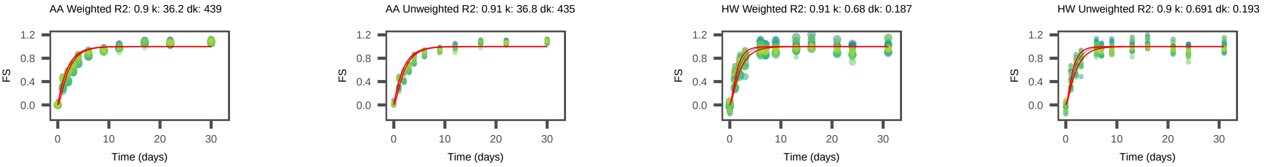

SDHA

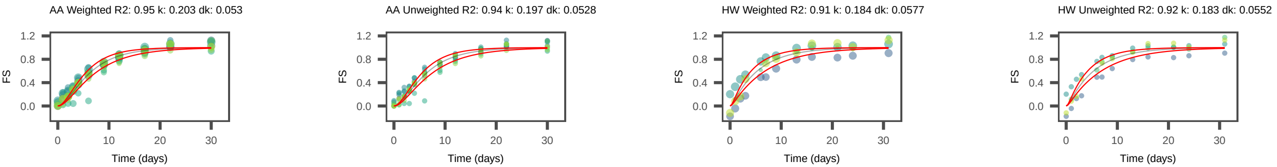

SDHB

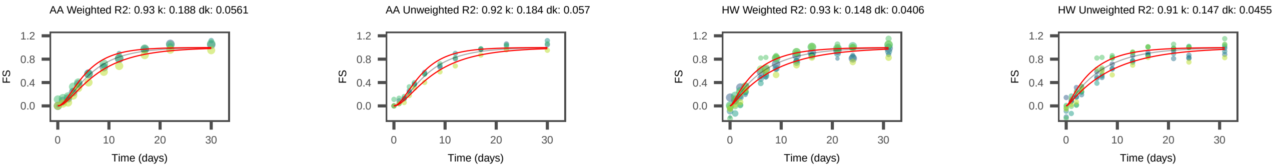

SDHL

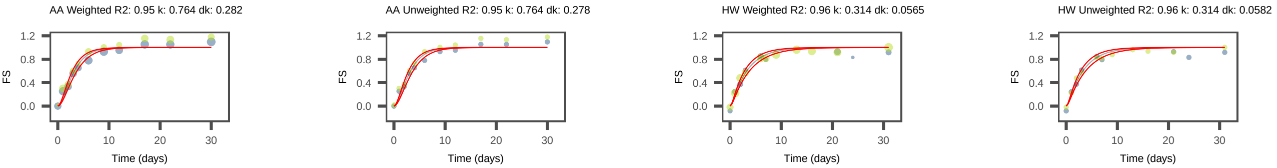

SF3B1

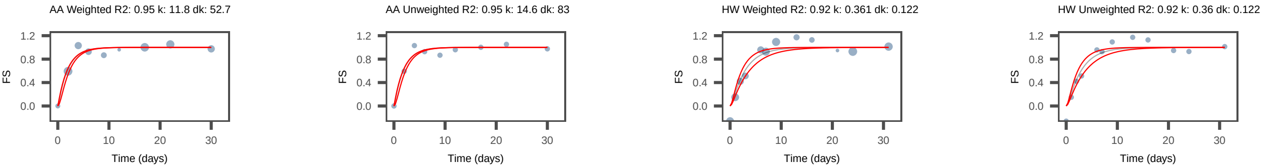

SFXN1

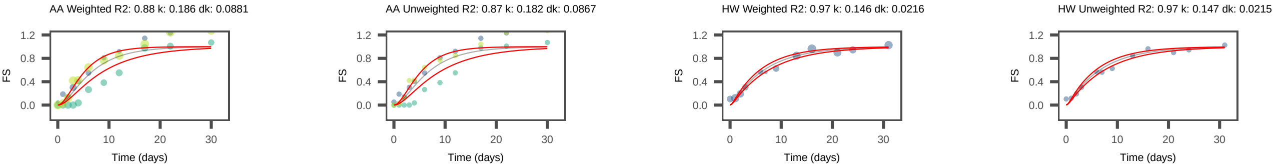

SGMR1

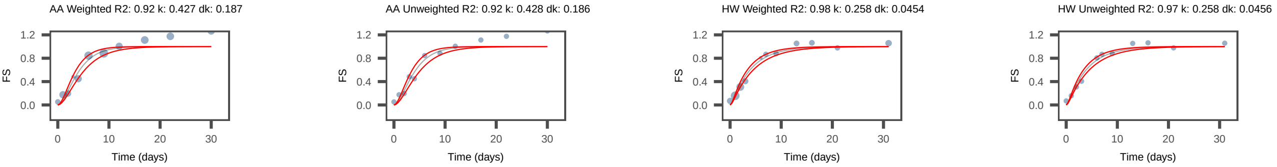

SMD1

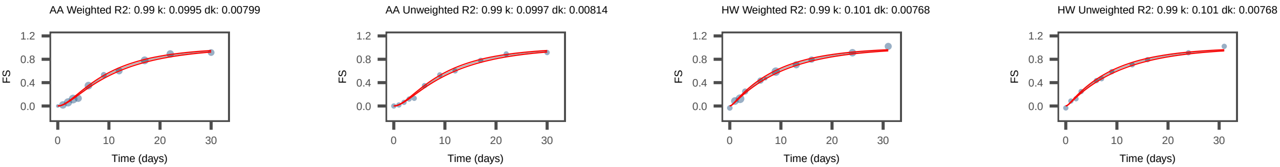

SMD3

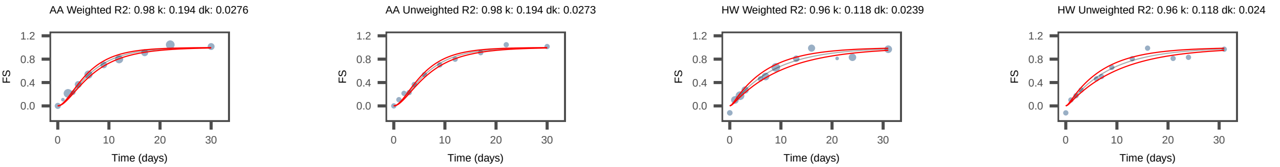

SODC

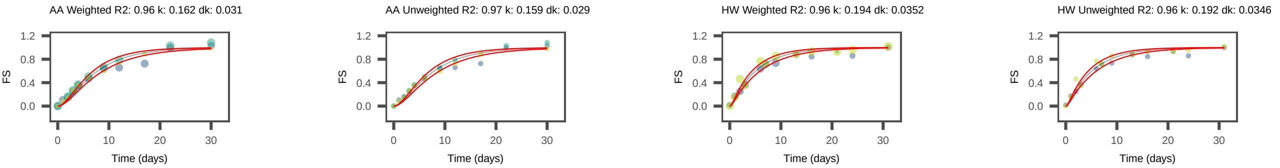

SODM

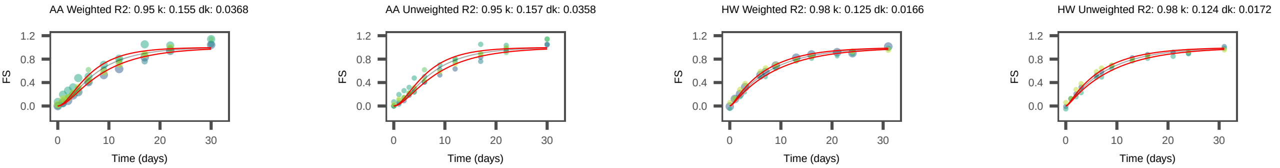

SOX

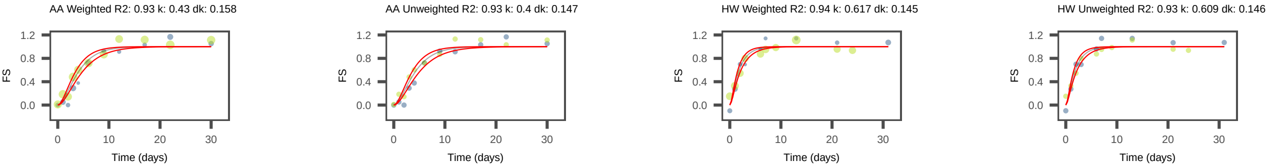

SPEB

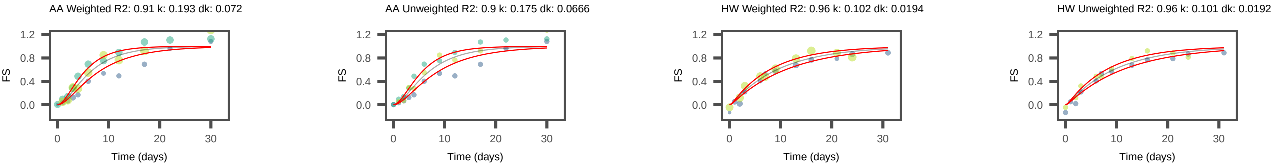

SPRE

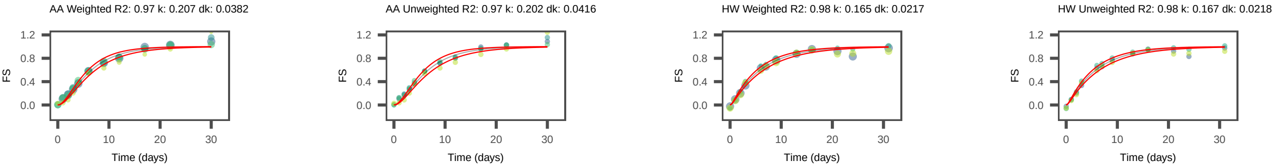

SPS1

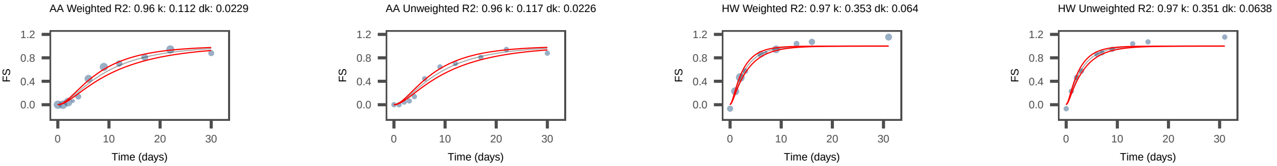

SPS2

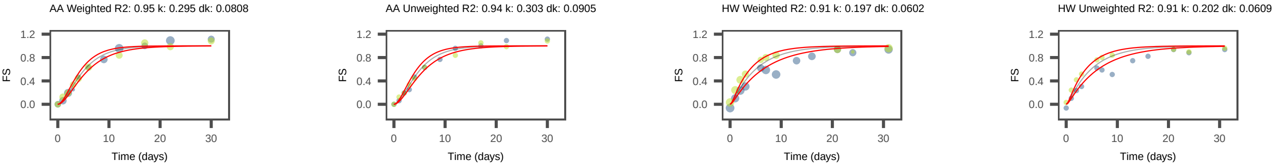

SPTN1

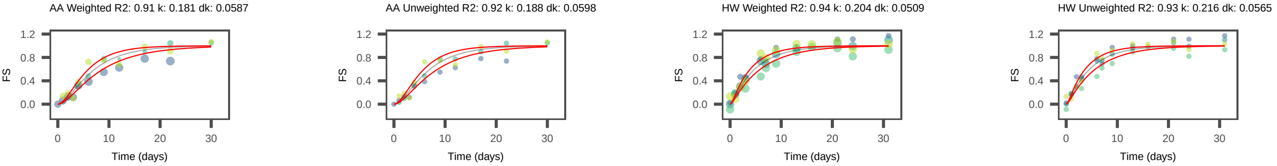

SRC8

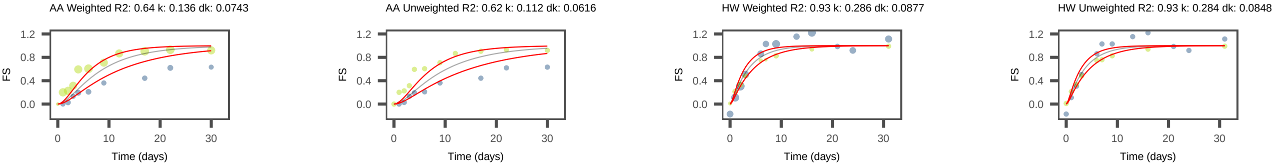

SSDH

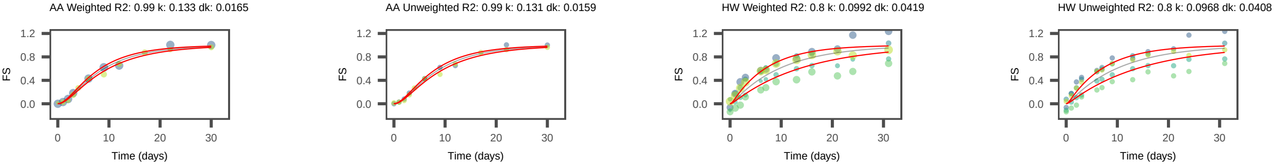

ST1A1

AA Weighted R2: 0.91 k: 0.476 dk: 0.194

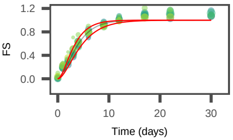

AA Unweighted R2: 0.9 k: 0.473 dk: 0.204

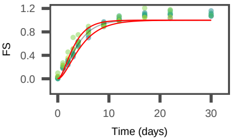

HW Weighted R2: 0.96 k: 0.312 dk: 0.0604

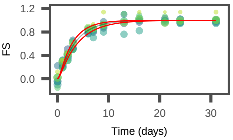

HW Unweighted R2: 0.95 k: 0.315 dk: 0.0673

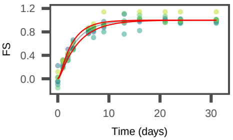

ST1D1

AA Weighted R2: 0.97 k: 0.292 dk: 0.0605

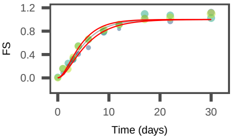

AA Unweighted R2: 0.97 k: 0.285 dk: 0.059

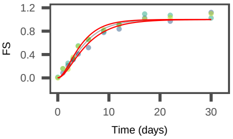

HW Weighted R2: 0.95 k: 0.252 dk: 0.0593

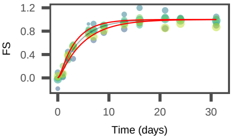

HW Unweighted R2: 0.95 k: 0.254 dk: 0.0606

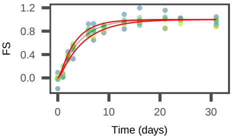

STA10

AA Weighted R2: 0.95 k: 0.198 dk: 0.0538

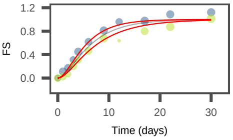

AA Unweighted R2: 0.95 k: 0.196 dk: 0.0538

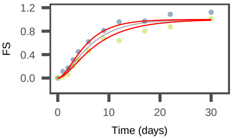

HW Weighted R2: 0.99 k: 0.188 dk: 0.0221

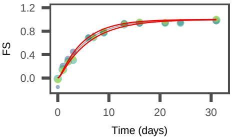

HW Unweighted R2: 0.98 k: 0.189 dk: 0.0229

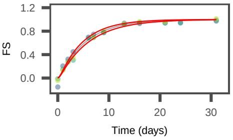

STIP1

AA Weighted R2: 0.93 k: 0.248 dk: 0.0818

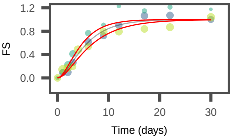

AA Unweighted R2: 0.92 k: 0.254 dk: 0.0878

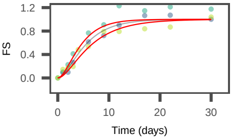

HW Weighted R2: 0.96 k: 0.295 dk: 0.057

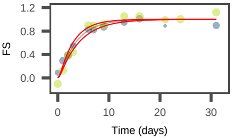

HW Unweighted R2: 0.96 k: 0.295 dk: 0.0571

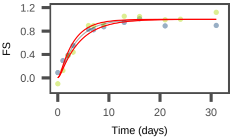

STML2

AA Weighted R2: 0.94 k: 0.126 dk: 0.0378

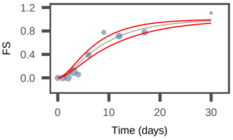

AA Unweighted R2: 0.93 k: 0.129 dk: 0.0402

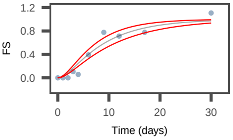

HW Weighted R2: 0.92 k: 0.159 dk: 0.0357

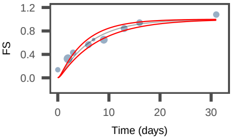

HW Unweighted R2: 0.93 k: 0.159 dk: 0.0356

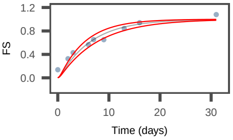

STT3A

AA Weighted R2: 0.94 k: 0.217 dk: 0.0656

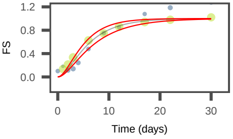

AA Unweighted R2: 0.93 k: 0.21 dk: 0.0698

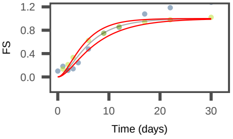

HW Weighted R2: 0.91 k: 0.098 dk: 0.0252

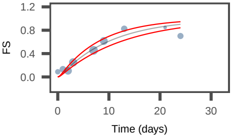

HW Unweighted R2: 0.91 k: 0.0979 dk: 0.0254

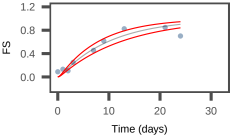

SUCA

AA Weighted R2: 0.97 k: 0.142 dk: 0.0258

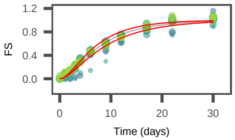

AA Unweighted R2: 0.96 k: 0.135 dk: 0.029

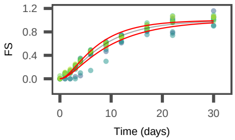

HW Weighted R2: 0.95 k: 0.108 dk: 0.0213

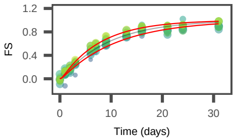

HW Unweighted R2: 0.94 k: 0.106 dk: 0.0231

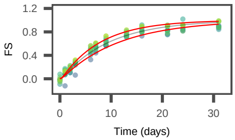

SUCB1

AA Weighted R2: 0.94 k: 0.18 dk: 0.0522

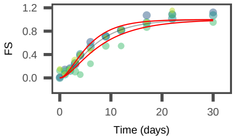

AA Unweighted R2: 0.93 k: 0.176 dk: 0.055

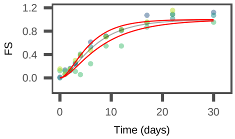

HW Weighted R2: 0.9 k: 0.13 dk: 0.0415

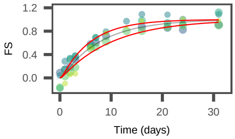

HW Unweighted R2: 0.9 k: 0.134 dk: 0.0437

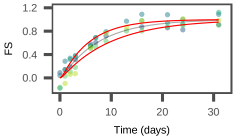

SUCB2

AA Weighted R2: 0.86 k: 0.179 dk: 0.0731

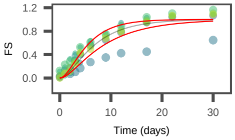

AA Unweighted R2: 0.88 k: 0.193 dk: 0.075

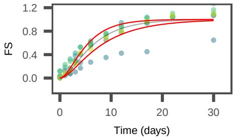

HW Weighted R2: 0.95 k: 0.182 dk: 0.0394

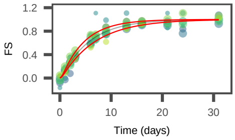

HW Unweighted R2: 0.94 k: 0.184 dk: 0.0456

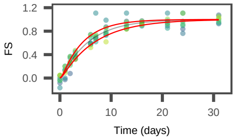

SUOX

AA Weighted R2: 0.96 k: 0.132 dk: 0.0251

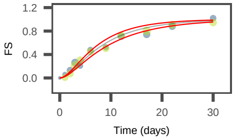

AA Unweighted R2: 0.96 k: 0.136 dk: 0.0285

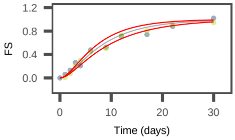

HW Weighted R2: 0.98 k: 0.149 dk: 0.0229

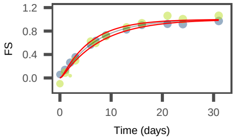

HW Unweighted R2: 0.97 k: 0.148 dk: 0.0238

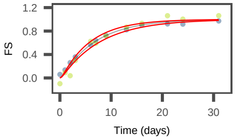

SYAC

AA Weighted R2: 0.94 k: 0.876 dk: 0.357

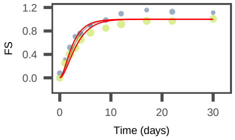

AA Unweighted R2: 0.93 k: 0.91 dk: 0.386

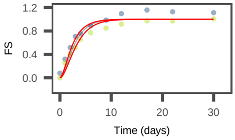

HW Weighted R2: 0.94 k: 0.735 dk: 0.186

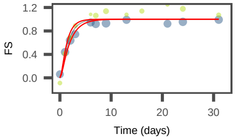

HW Unweighted R2: 0.93 k: 0.748 dk: 0.199

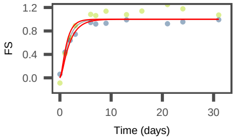

SYEP

AA Weighted R2: 0.93 k: 0.354 dk: 0.126

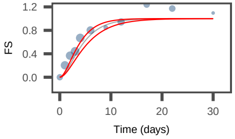

AA Unweighted R2: 0.93 k: 0.345 dk: 0.121

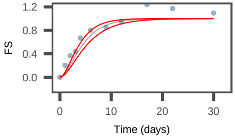

HW Weighted R2: 0.93 k: 0.232 dk: 0.0646

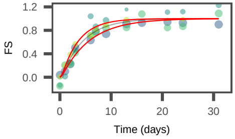

HW Unweighted R2: 0.93 k: 0.241 dk: 0.0708

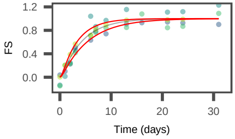

SYFB

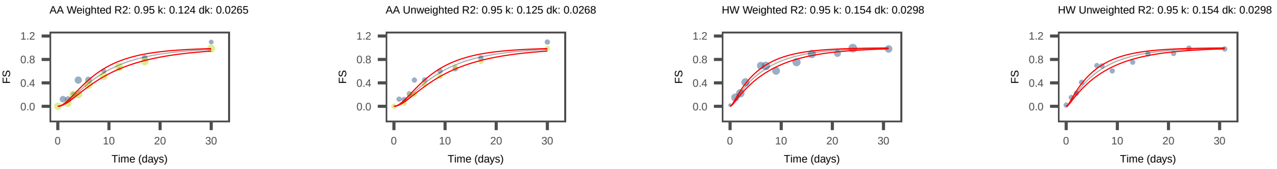

SYLC

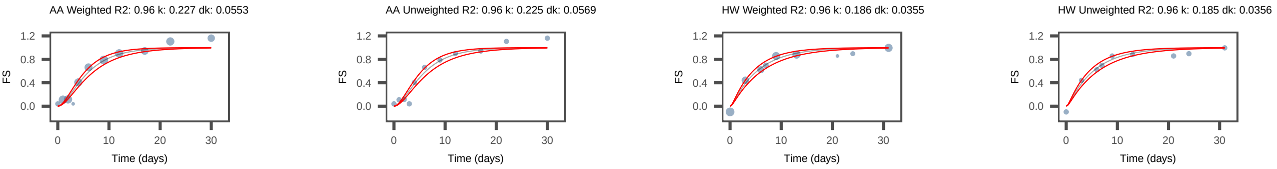

SYQ

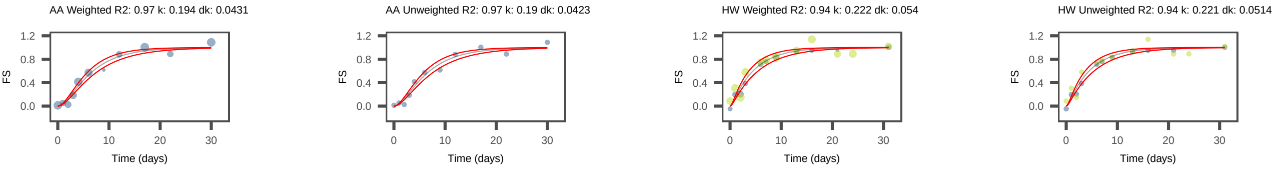

SYTC

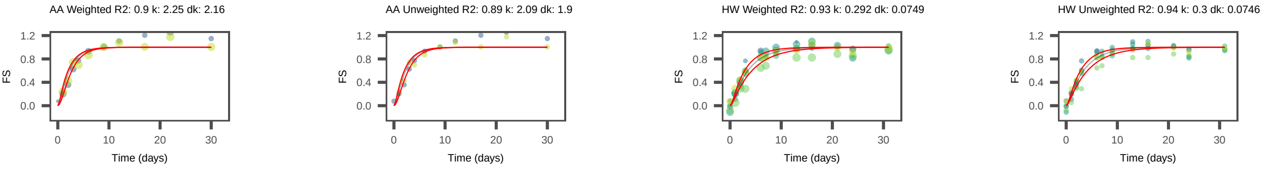

TADBP

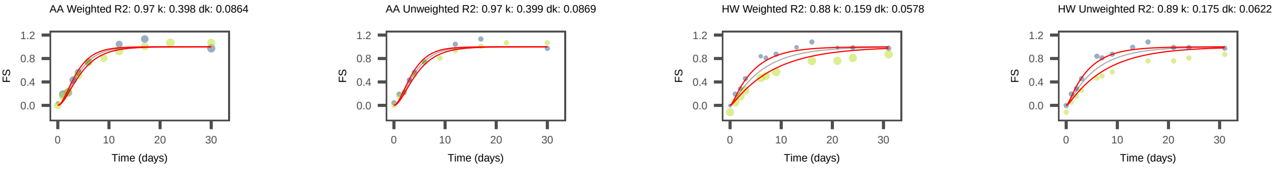

TALDO

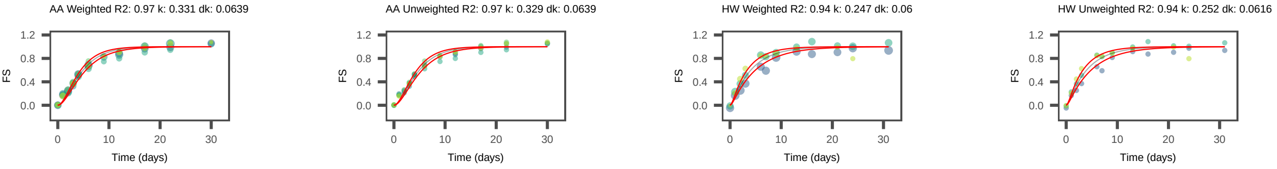

TBA4A

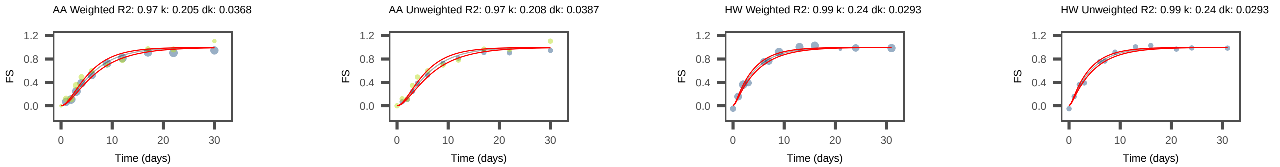

TBB4B

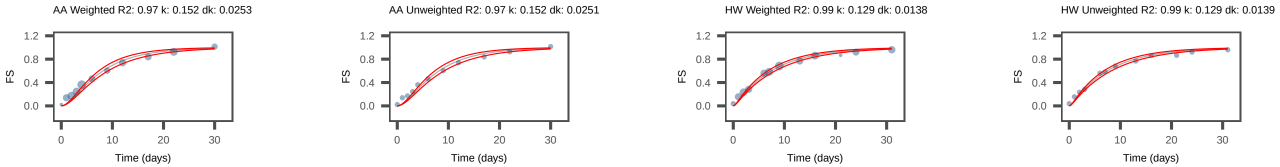

TBB5

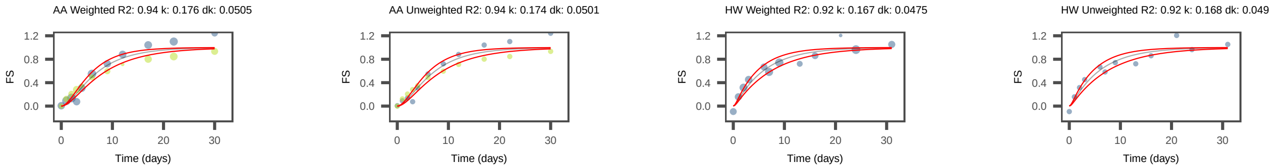

TCPA

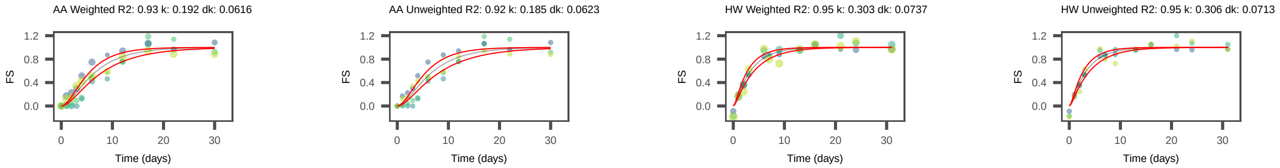

TCPB

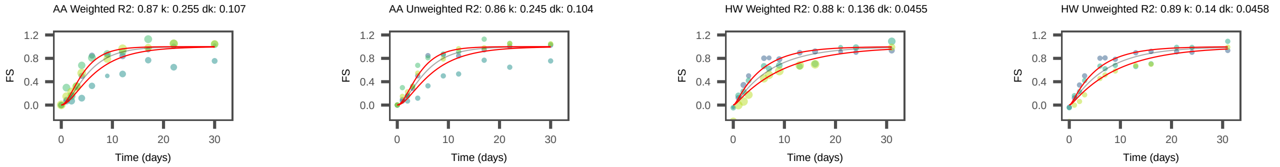

TCPD

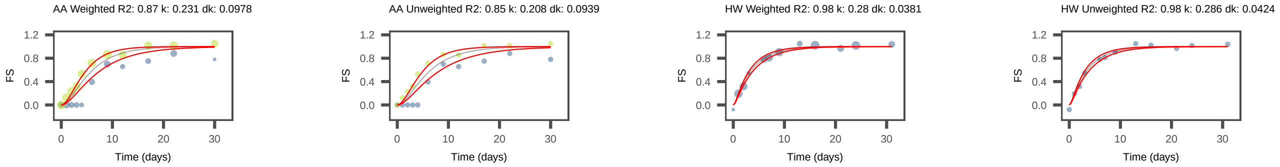

TCPE

AA Weighted R2: 0.96 k: 0.44 dk: 0.127

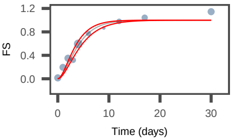

AA Unweighted R2: 0.96 k: 0.436 dk: 0.126

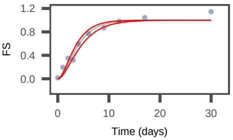

HW Weighted R2: 0.98 k: 0.234 dk: 0.0276

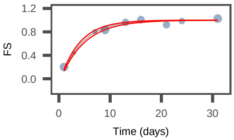

HW Unweighted R2: 0.98 k: 0.233 dk: 0.0275

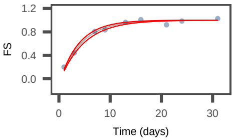

TCPG

AA Weighted R2: 0.95 k: 0.292 dk: 0.0761

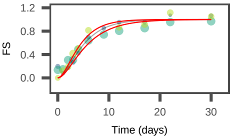

AA Unweighted R2: 0.96 k: 0.296 dk: 0.0737

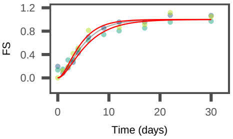

HW Weighted R2: 0.93 k: 0.188 dk: 0.0496

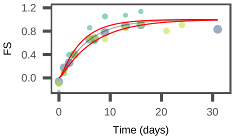

HW Unweighted R2: 0.91 k: 0.184 dk: 0.0526

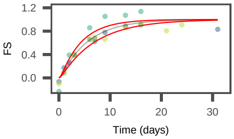

TCPH

AA Weighted R2: 0.9 k: 0.318 dk: 0.133

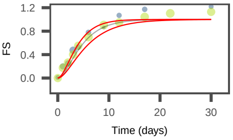

AA Unweighted R2: 0.85 k: 0.287 dk: 0.14

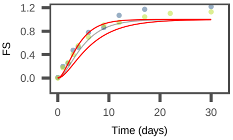

HW Weighted R2: 0.96 k: 0.34 dk: 0.0594

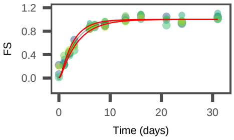

HW Unweighted R2: 0.96 k: 0.342 dk: 0.0595

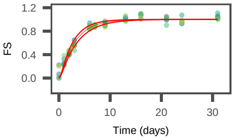

TCPQ

AA Weighted R2: 0.95 k: 0.233 dk: 0.0619

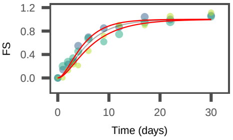

AA Unweighted R2: 0.95 k: 0.234 dk: 0.0628

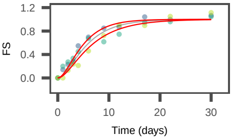

HW Weighted R2: 0.93 k: 0.268 dk: 0.0818

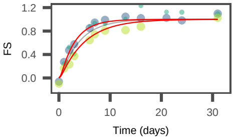

HW Unweighted R2: 0.92 k: 0.284 dk: 0.0921

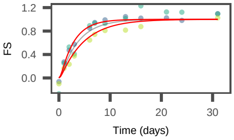

TCPZ

AA Weighted R2: 0.97 k: 0.37 dk: 0.08

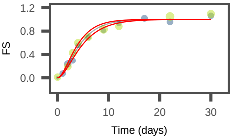

AA Unweighted R2: 0.97 k: 0.364 dk: 0.0771

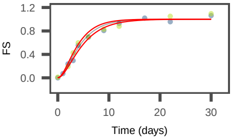

HW Weighted R2: 0.98 k: 0.349 dk: 0.0513

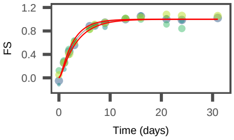

HW Unweighted R2: 0.97 k: 0.348 dk: 0.0527

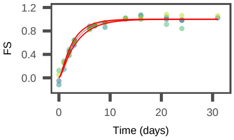

TERA

AA Weighted R2: 0.96 k: 0.426 dk: 0.107

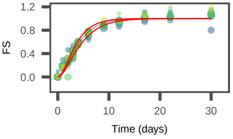

AA Unweighted R2: 0.96 k: 0.424 dk: 0.113

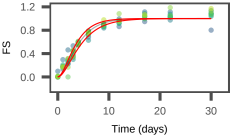

HW Weighted R2: 0.96 k: 0.336 dk: 0.0602

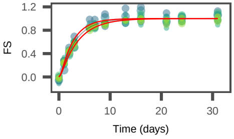

HW Unweighted R2: 0.96 k: 0.338 dk: 0.0597

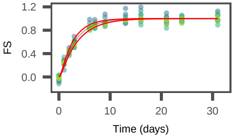

TGM2

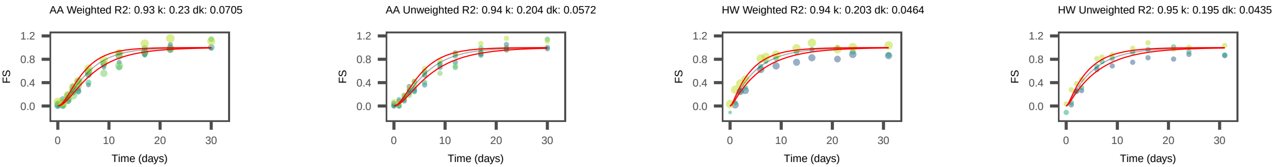

THIC

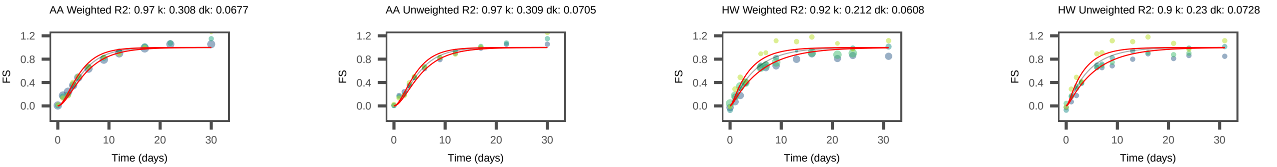

THIKB

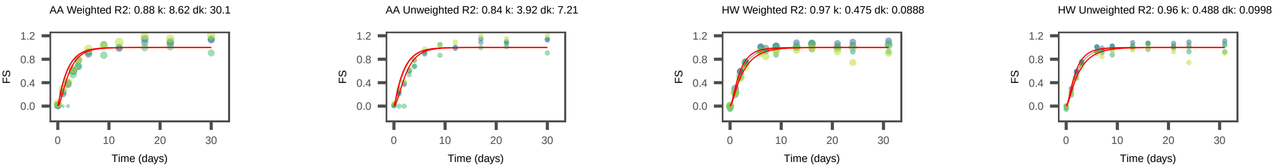

THIL

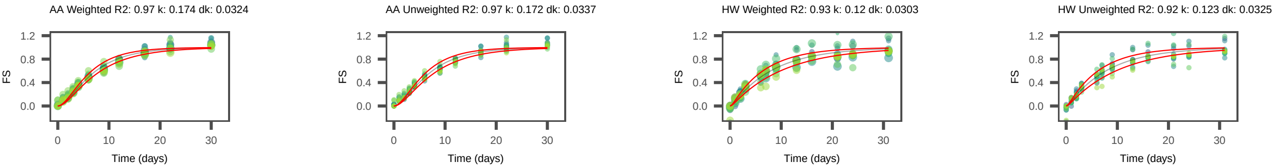

THIM

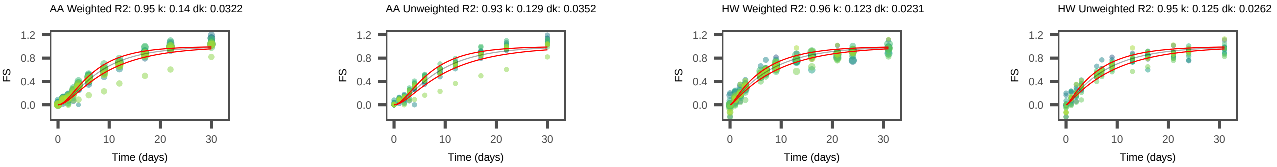

THIO

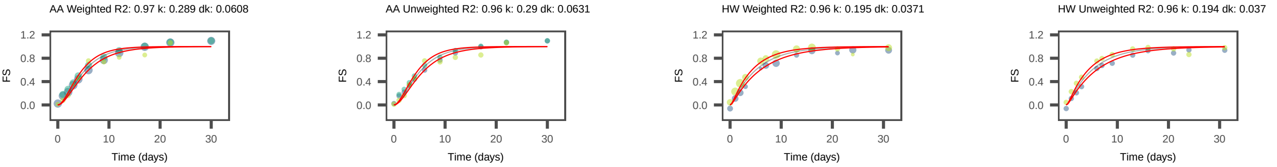

THTM

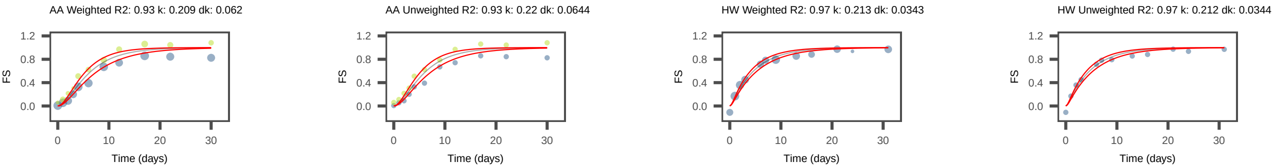

THTR

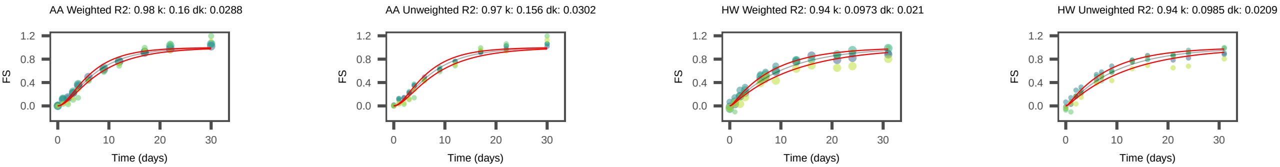

TIAR

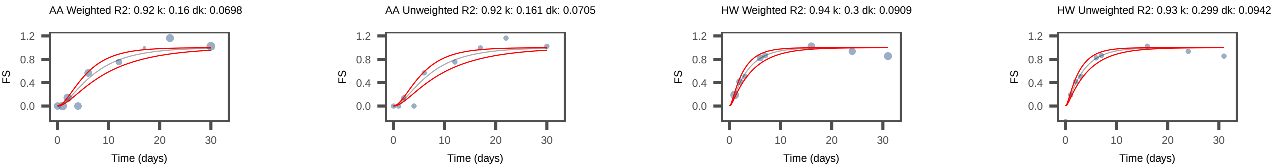

TIM44

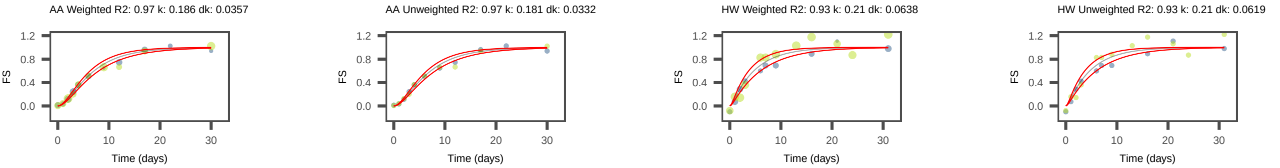

TIM50

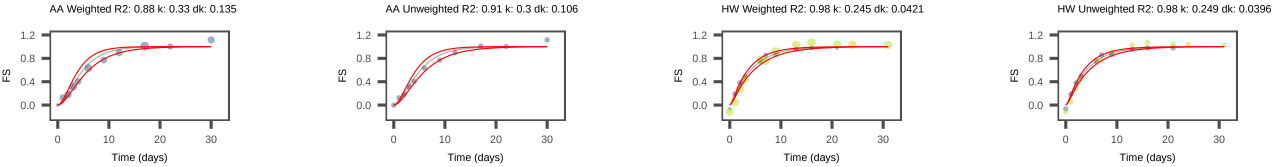

TKFC

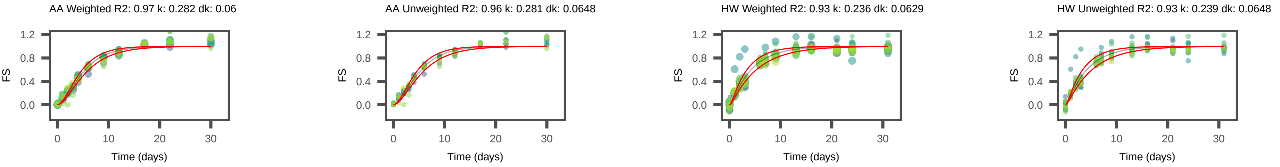

TKT

AA Weighted R2: 0.95 k: 0.132 dk: 0.03

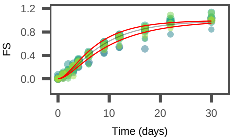

AA Unweighted R2: 0.94 k: 0.129 dk: 0.0315

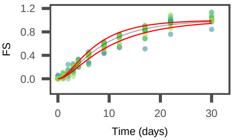

HW Weighted R2: 0.94 k: 0.0911 dk: 0.0206

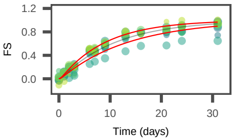

HW Unweighted R2: 0.93 k: 0.0907 dk: 0.0207

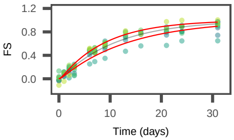

TLN1

AA Weighted R2: 0.88 k: 0.191 dk: 0.0749

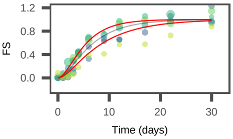

AA Unweighted R2: 0.88 k: 0.18 dk: 0.0707

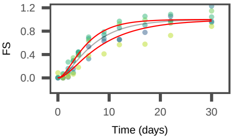

HW Weighted R2: 0.96 k: 0.27 dk: 0.058

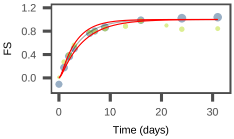

HW Unweighted R2: 0.95 k: 0.273 dk: 0.0614

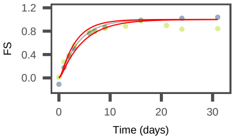

TPD54

AA Weighted R2: 0.93 k: 0.241 dk: 0.0682

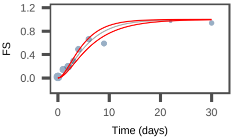

AA Unweighted R2: 0.92 k: 0.238 dk: 0.0686

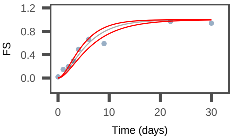

HW Weighted R2: 0.98 k: 0.339 dk: 0.0538

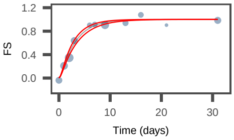

HW Unweighted R2: 0.98 k: 0.341 dk: 0.0542

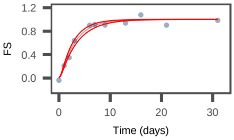

TPIS

AA Weighted R2: 0.95 k: 0.186 dk: 0.0481

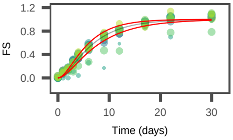

AA Unweighted R2: 0.92 k: 0.177 dk: 0.0546

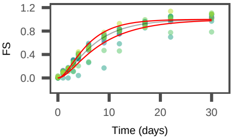

HW Weighted R2: 0.99 k: 0.166 dk: 0.0167

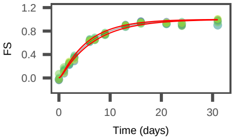

HW Unweighted R2: 0.99 k: 0.168 dk: 0.0164

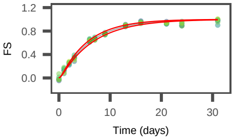

TPMT

AA Weighted R2: 0.79 k: 0.157 dk: 0.0815

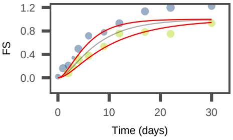

AA Unweighted R2: 0.76 k: 0.14 dk: 0.0765

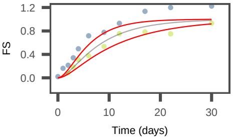

HW Weighted R2: 0.88 k: 0.204 dk: 0.0774

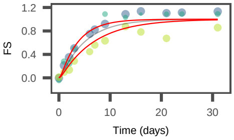

HW Unweighted R2: 0.88 k: 0.206 dk: 0.079

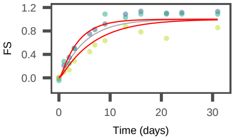

TRAP1

AA Weighted R2: 0.94 k: 0.518 dk: 0.177

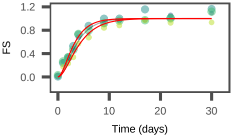

AA Unweighted R2: 0.93 k: 0.473 dk: 0.161

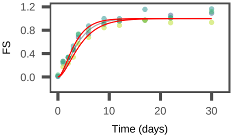

HW Weighted R2: 0.91 k: 0.747 dk: 0.21

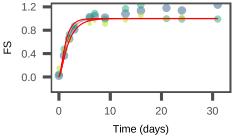

HW Unweighted R2: 0.92 k: 0.755 dk: 0.207

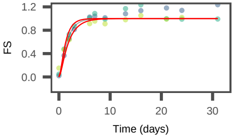

TRFE

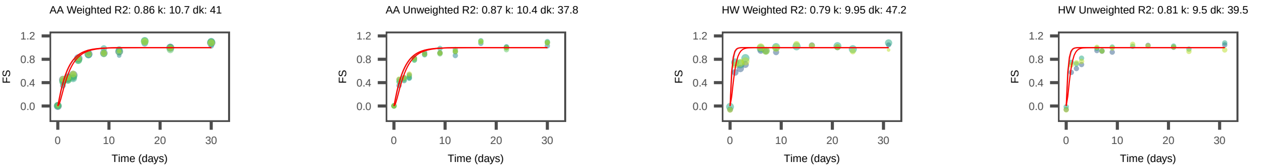

TTC38

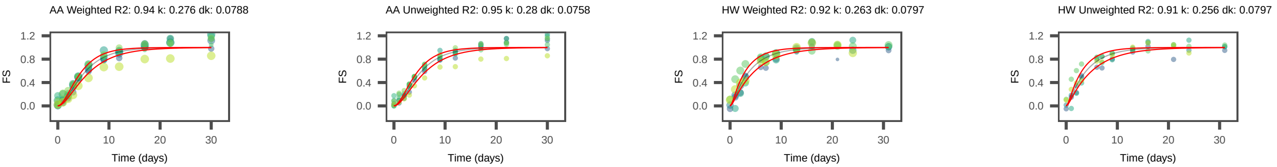

TTPA

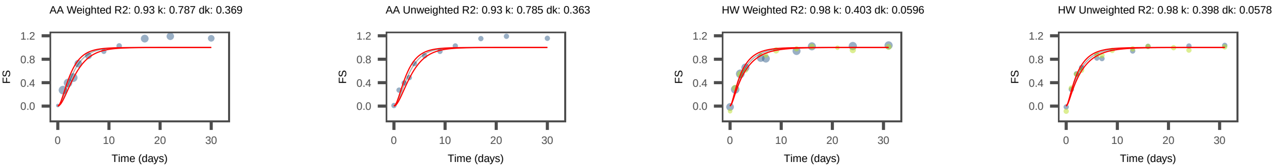

TXND5

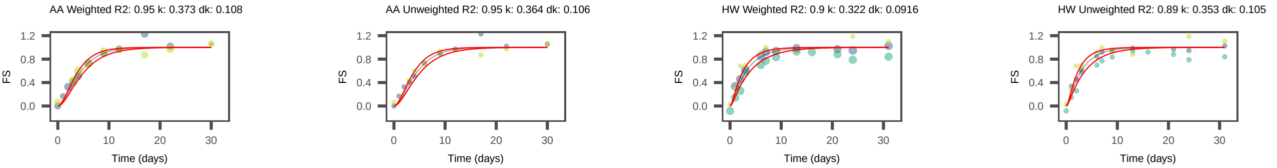

TXNL1

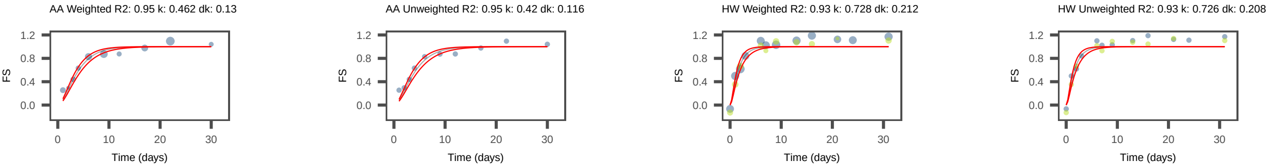

TXTP

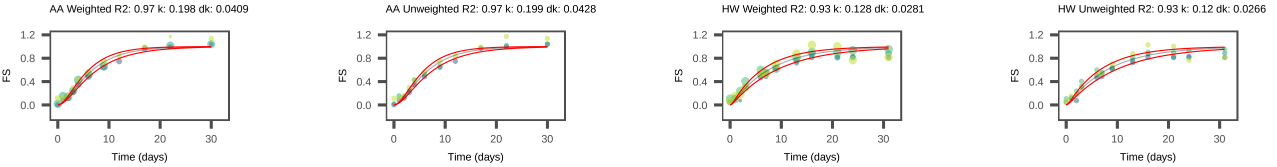

UBA1

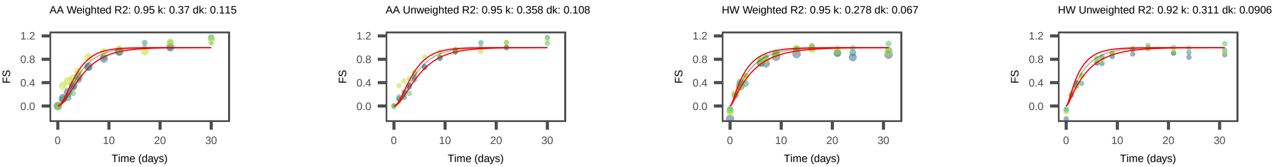

UBE2N

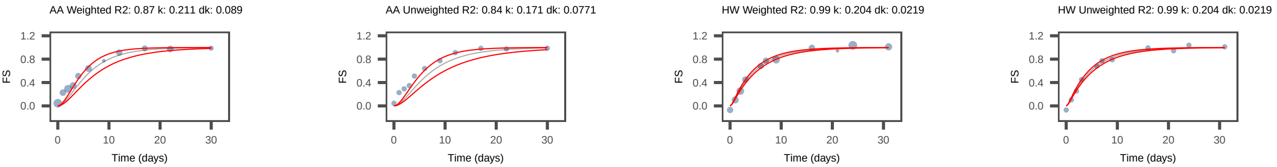

UCRI

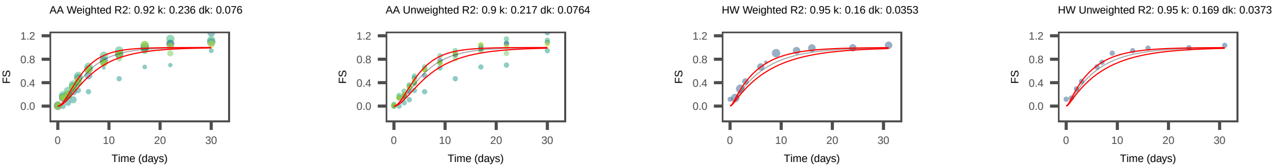

UD11

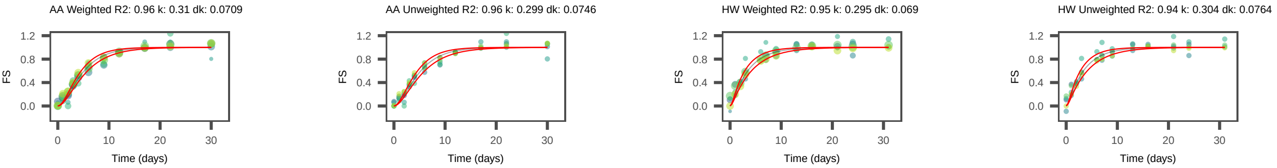

UD16

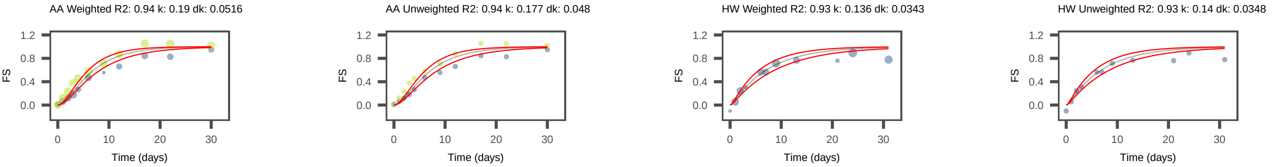

UD2A3

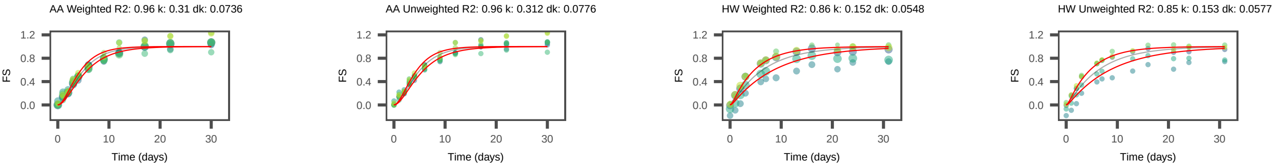

UD3A2

AA Weighted R2: 0.98 k: 0.424 dk: 0.0766 | AA Unweighted R2: 0.98 k: 0.424 dk: 0.0788 | HW Weighted R2: 0.91 k: 0.258 dk: 0.0884 | HW Unweighted R2: 0.91 k: 0.254 dk: 0.0897

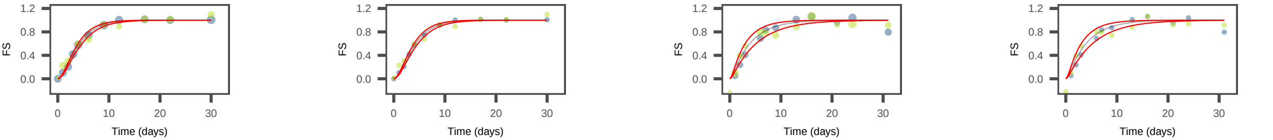

UDB17

AA Weighted R2: 0.94 k: 0.467 dk: 0.154 | AA Unweighted R2: 0.93 k: 0.427 dk: 0.151 | HW Weighted R2: 0.97 k: 0.282 dk: 0.05 | HW Unweighted R2: 0.95 k: 0.268 dk: 0.0583

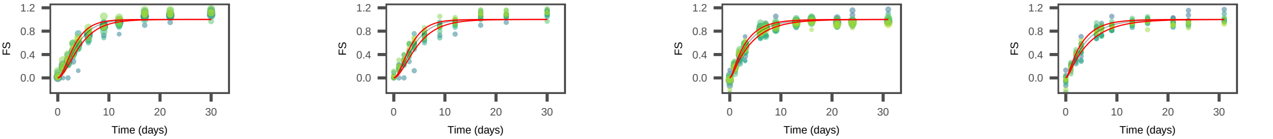

UGDH

AA Weighted R2: 0.96 k: 0.453 dk: 0.121 | AA Unweighted R2: 0.96 k: 0.445 dk: 0.12 | HW Weighted R2: 0.92 k: 0.324 dk: 0.0876 | HW Unweighted R2: 0.91 k: 0.312 dk: 0.0892

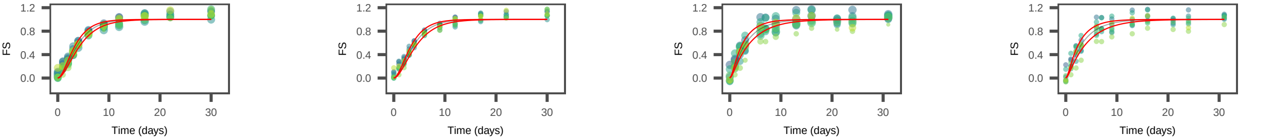

UGPA

AA Weighted R2: 0.95 k: 0.38 dk: 0.106 | AA Unweighted R2: 0.95 k: 0.371 dk: 0.108 | HW Weighted R2: 0.94 k: 0.287 dk: 0.0678 | HW Unweighted R2: 0.94 k: 0.291 dk: 0.0687

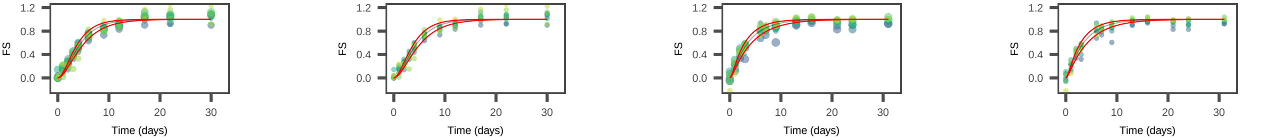

URIC

AA Weighted R2: 0.97 k: 0.483 dk: 0.109 | AA Unweighted R2: 0.97 k: 0.484 dk: 0.118 | HW Weighted R2: 0.96 k: 0.364 dk: 0.0738 | HW Unweighted R2: 0.95 k: 0.346 dk: 0.077

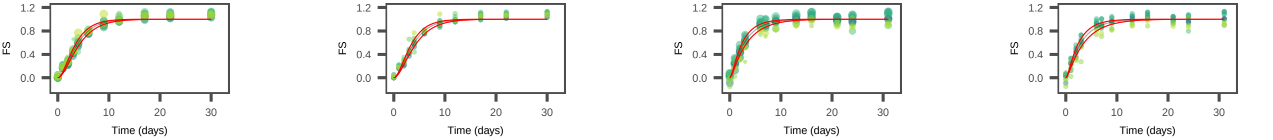

VAPA

AA Weighted R2: 0.9 k: 0.163 dk: 0.0428 | AA Unweighted R2: 0.9 k: 0.147 dk: 0.0399 | HW Weighted R2: 0.96 k: 0.113 dk: 0.02 | HW Unweighted R2: 0.96 k: 0.113 dk: 0.0202

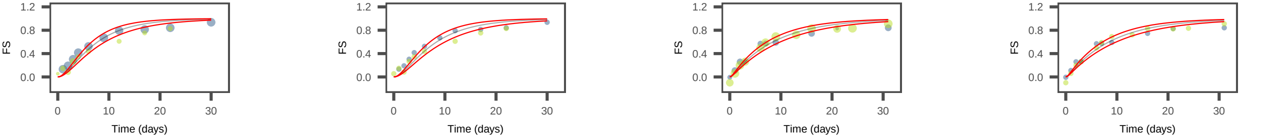

VDAC1

AA Weighted R2: 0.95 k: 0.185 dk: 0.0481

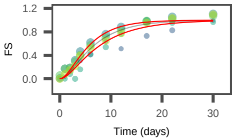

AA Unweighted R2: 0.94 k: 0.168 dk: 0.0449

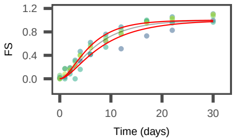

HW Weighted R2: 0.95 k: 0.175 dk: 0.0402

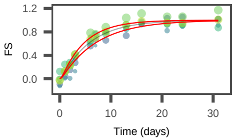

HW Unweighted R2: 0.95 k: 0.17 dk: 0.0398

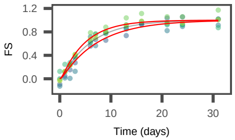

VDAC2

AA Weighted R2: 0.96 k: 0.196 dk: 0.0437

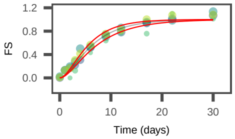

AA Unweighted R2: 0.96 k: 0.189 dk: 0.0435

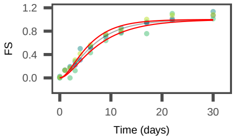

HW Weighted R2: 0.94 k: 0.158 dk: 0.0389

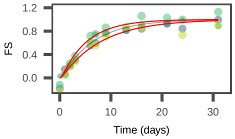

HW Unweighted R2: 0.95 k: 0.158 dk: 0.0377

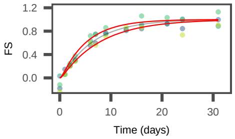

VDAC3

AA Weighted R2: 0.91 k: 0.154 dk: 0.044

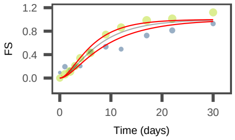

AA Unweighted R2: 0.91 k: 0.148 dk: 0.0431

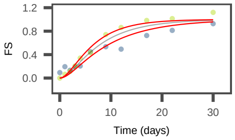

HW Weighted R2: 0.96 k: 0.198 dk: 0.0377

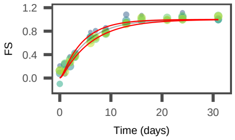

HW Unweighted R2: 0.96 k: 0.2 dk: 0.0399

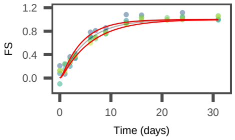

VIGLN

AA Weighted R2: 0.94 k: 0.623 dk: 0.237

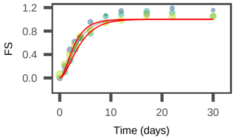

AA Unweighted R2: 0.93 k: 0.603 dk: 0.242

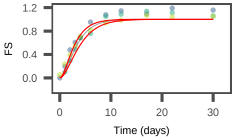

HW Weighted R2: 0.95 k: 0.609 dk: 0.13

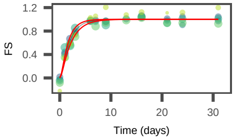

HW Unweighted R2: 0.95 k: 0.609 dk: 0.135

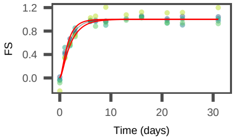

VINC

AA Weighted R2: 0.95 k: 0.155 dk: 0.0325

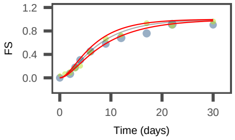

AA Unweighted R2: 0.95 k: 0.157 dk: 0.0332

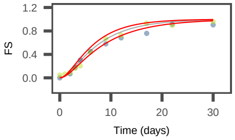

HW Weighted R2: 0.76 k: 0.158 dk: 0.0716

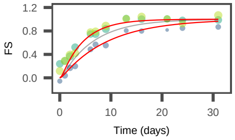

HW Unweighted R2: 0.75 k: 0.148 dk: 0.0688

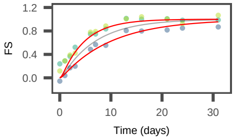

VPS35

AA Weighted R2: 0.91 k: 0.165 dk: 0.0464

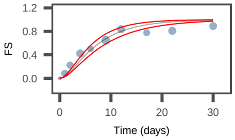

AA Unweighted R2: 0.91 k: 0.165 dk: 0.0462

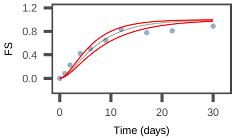

HW Weighted R2: 0.92 k: 0.164 dk: 0.0492

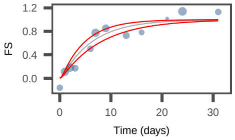

HW Unweighted R2: 0.92 k: 0.162 dk: 0.0492

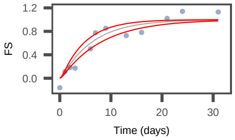

VWA8

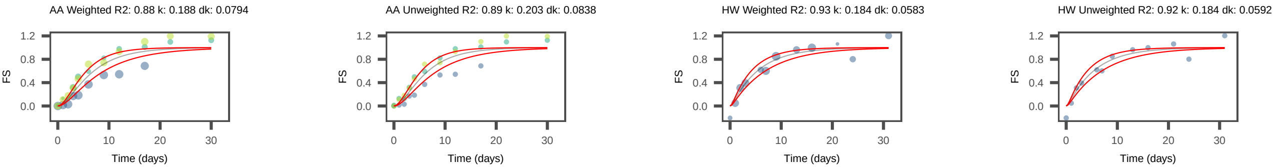

WDR1

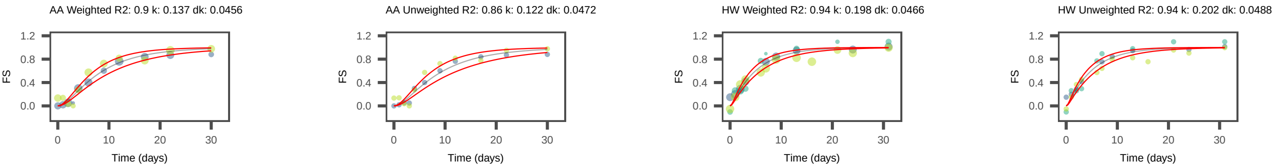

XDH

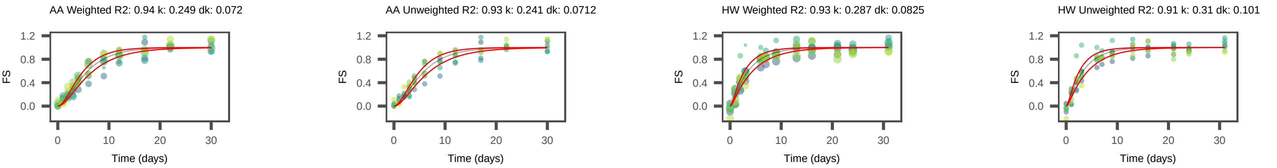

XYLB

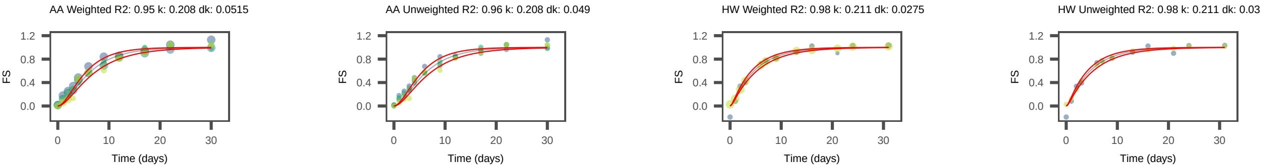

Supplement: Supplemental Data S8 [file mmc9.pdf]
