# Supplementary material for: Harmonizing Labeling and Analytical Strategies to Obtain Protein Turnover Rates in Intact Adult Animals
Source: Mol Cell Proteomics. 2022 May 28;21(7):100252. doi: 10.1016/j.mcpro.2022.100252 (PMC9249856; doi:10.1016/j.mcpro.2022.100252)

1433E

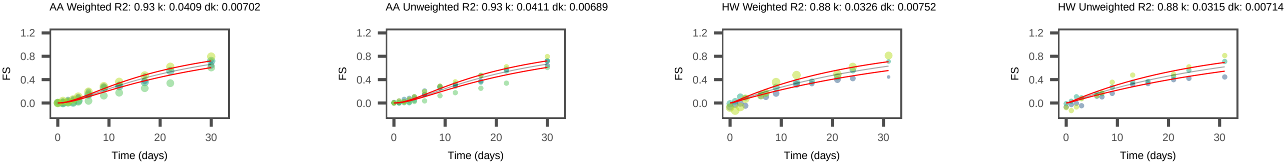

1433G

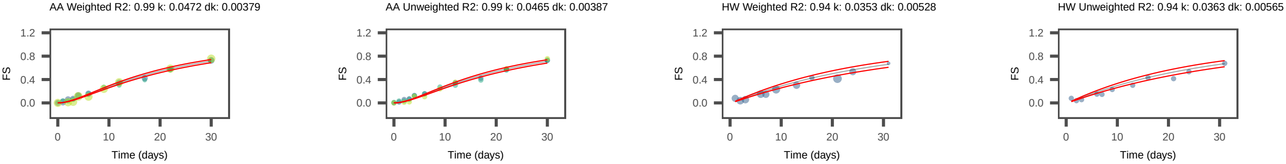

2AAA

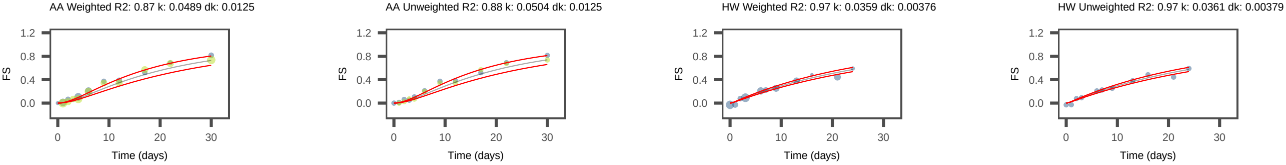

AATM

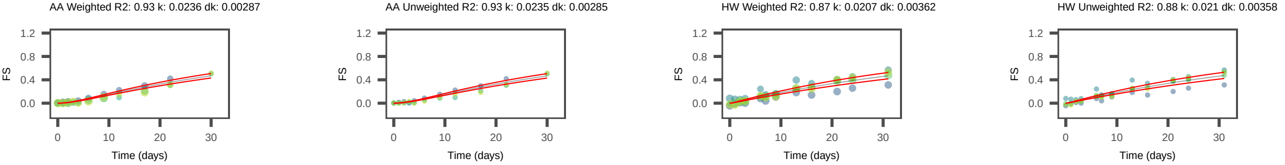

ABEC2

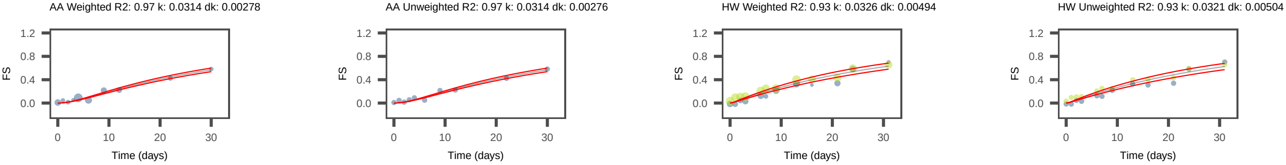

ACADL

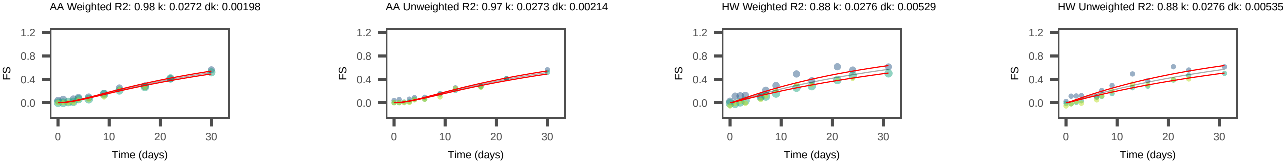

ACADM

AA Weighted R2: 0.93 k: 0.0798 dk: 0.0183
AA Unweighted R2: 0.92 k: 0.0818 dk: 0.0197
HW Weighted R2: 0.94 k: 0.0469 dk: 0.00796
HW Unweighted R2: 0.94 k: 0.0468 dk: 0.00793

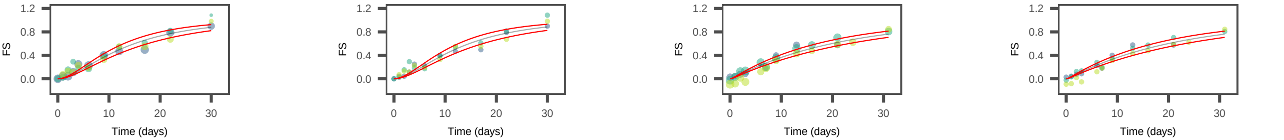

ACADV

AA Weighted R2: 0.95 k: 0.0531 dk: 0.00859
AA Unweighted R2: 0.95 k: 0.0532 dk: 0.00868
HW Weighted R2: 0.89 k: 0.0349 dk: 0.00786
HW Unweighted R2: 0.9 k: 0.036 dk: 0.00796

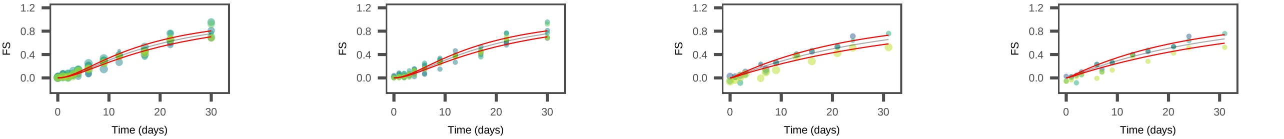

ACBP

AA Weighted R2: 0.94 k: 0.0244 dk: 0.00306
AA Unweighted R2: 0.93 k: 0.0242 dk: 0.00317
HW Weighted R2: 0.95 k: 0.0238 dk: 0.00252
HW Unweighted R2: 0.95 k: 0.0238 dk: 0.00257

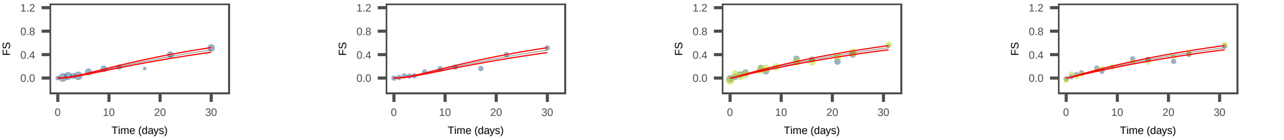

ACO13

AA Weighted R2: 0.98 k: 0.0392 dk: 0.00336
AA Unweighted R2: 0.98 k: 0.0397 dk: 0.00342
HW Weighted R2: 0.91 k: 0.022 dk: 0.00377
HW Unweighted R2: 0.92 k: 0.0221 dk: 0.00369

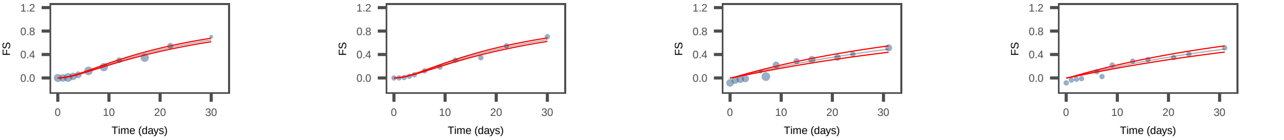

ACON

AA Weighted R2: 0.91 k: 0.037 dk: 0.00649
AA Unweighted R2: 0.89 k: 0.0355 dk: 0.00687
HW Weighted R2: 0.91 k: 0.0252 dk: 0.00401
HW Unweighted R2: 0.91 k: 0.0253 dk: 0.00411

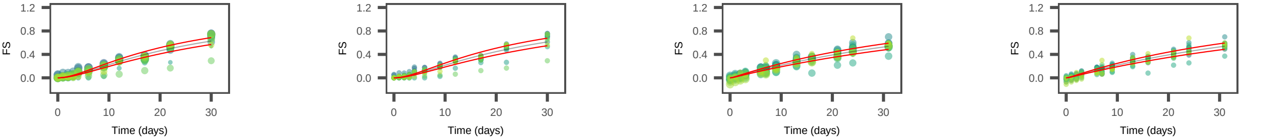

ACSL1

AA Weighted R2: 0.91 k: 0.0383 dk: 0.00709
AA Unweighted R2: 0.92 k: 0.0384 dk: 0.00702
HW Weighted R2: 0.95 k: 0.0492 dk: 0.00902
HW Unweighted R2: 0.95 k: 0.0494 dk: 0.00911

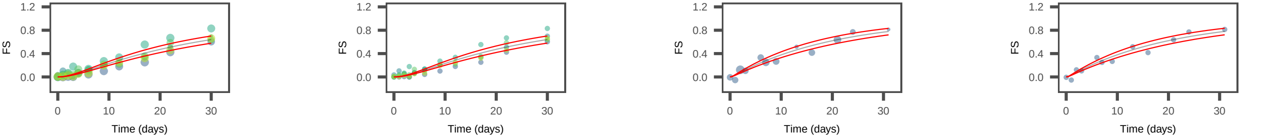

ACTN2

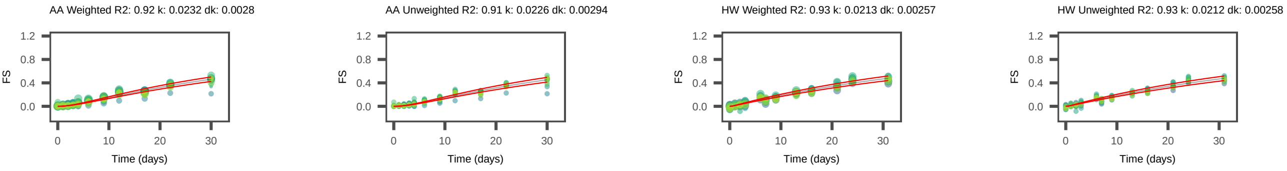

ACTN3

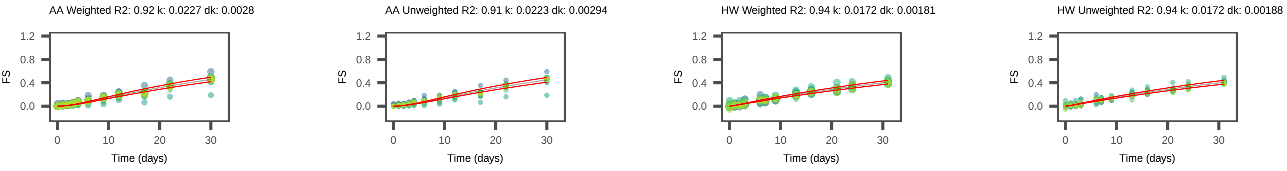

ADT1

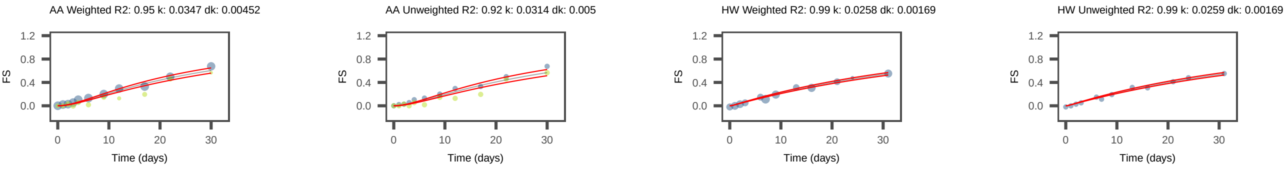

ALBU

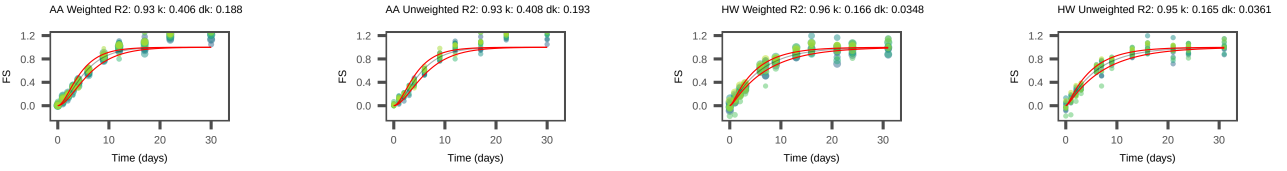

ALDH2

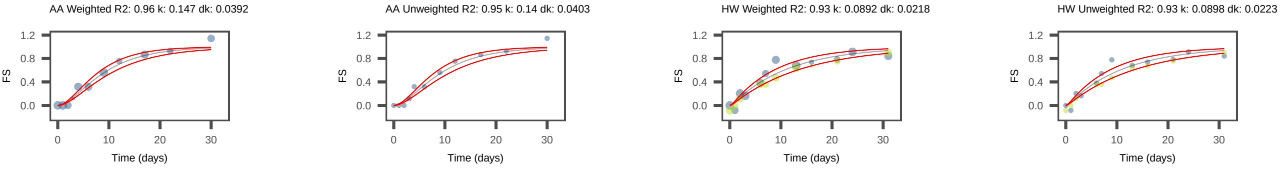

ALDOA

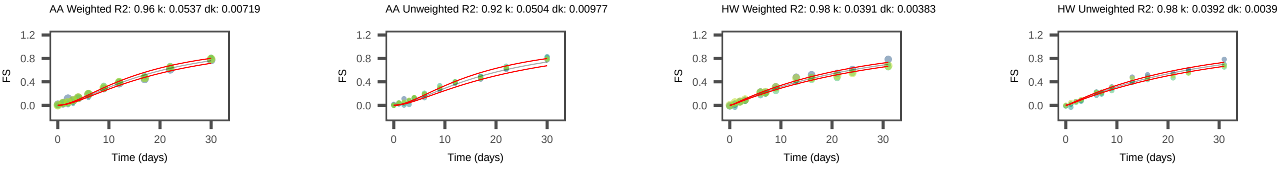

ALDR

AA Weighted R2: 0.93 k: 0.0184 dk: 0.00223

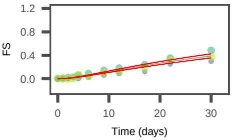

AA Unweighted R2: 0.92 k: 0.0181 dk: 0.00229

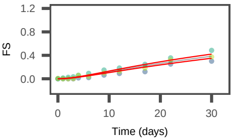

HW Weighted R2: 0.94 k: 0.0155 dk: 0.00178

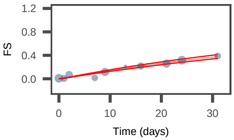

HW Unweighted R2: 0.94 k: 0.0155 dk: 0.0018

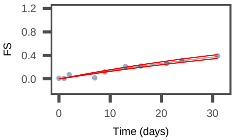

AMPD1

AA Weighted R2: 0.94 k: 0.0525 dk: 0.00882

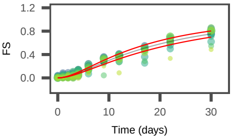

AA Unweighted R2: 0.93 k: 0.0505 dk: 0.00954

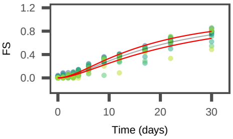

HW Weighted R2: 0.93 k: 0.0471 dk: 0.00881

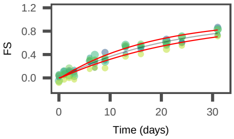

HW Unweighted R2: 0.93 k: 0.0464 dk: 0.00863

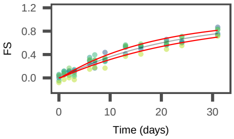

ANXA6

AA Weighted R2: 0.94 k: 0.0295 dk: 0.00354

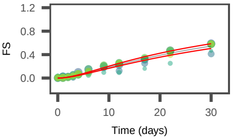

AA Unweighted R2: 0.93 k: 0.0286 dk: 0.00375

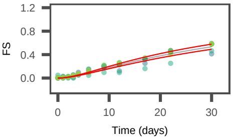

HW Weighted R2: 0.96 k: 0.024 dk: 0.0024

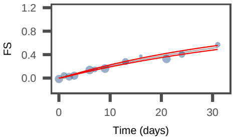

HW Unweighted R2: 0.96 k: 0.0242 dk: 0.00243

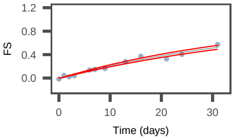

AT2A1

AA Weighted R2: 0.97 k: 0.0341 dk: 0.00334

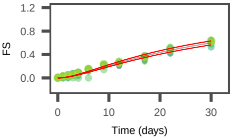

AA Unweighted R2: 0.96 k: 0.0336 dk: 0.00352

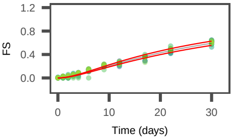

HW Weighted R2: 0.92 k: 0.0263 dk: 0.0042

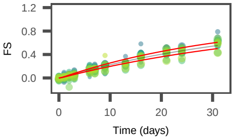

HW Unweighted R2: 0.91 k: 0.0266 dk: 0.00441

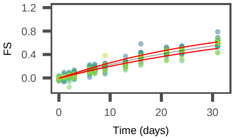

AT5F1

AA Weighted R2: 0.99 k: 0.0266 dk: 0.0016

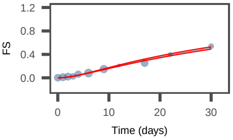

AA Unweighted R2: 0.99 k: 0.0266 dk: 0.0016

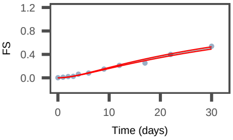

HW Weighted R2: 0.92 k: 0.0161 dk: 0.0021

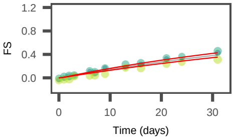

HW Unweighted R2: 0.93 k: 0.0162 dk: 0.00205

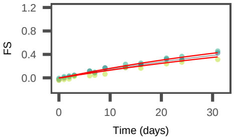

ATP5H

AA Weighted R2: 0.97 k: 0.0252 dk: 0.00196

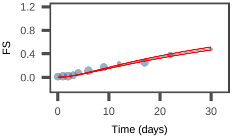

AA Unweighted R2: 0.97 k: 0.0252 dk: 0.00196

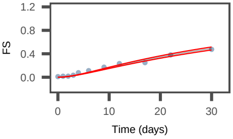

HW Weighted R2: 0.94 k: 0.0191 dk: 0.00222

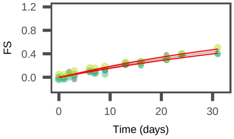

HW Unweighted R2: 0.94 k: 0.019 dk: 0.00224

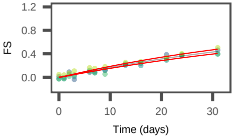

ATP5J

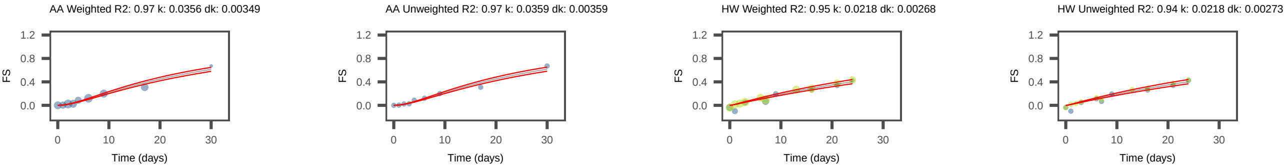

ATP5L

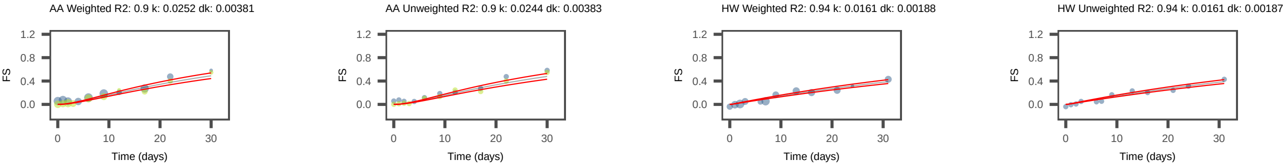

ATPA

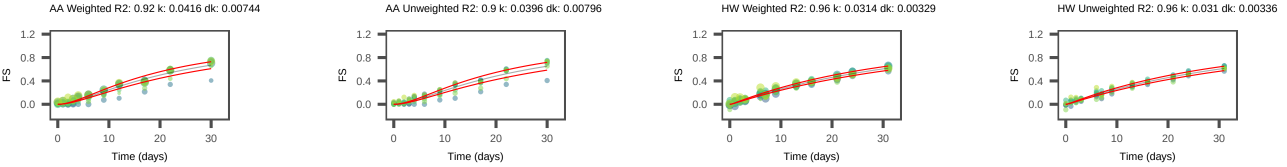

ATPB

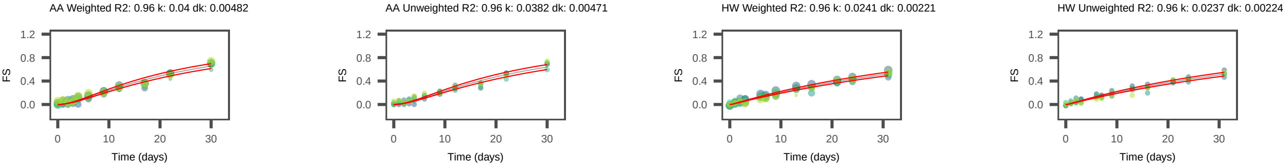

ATPD

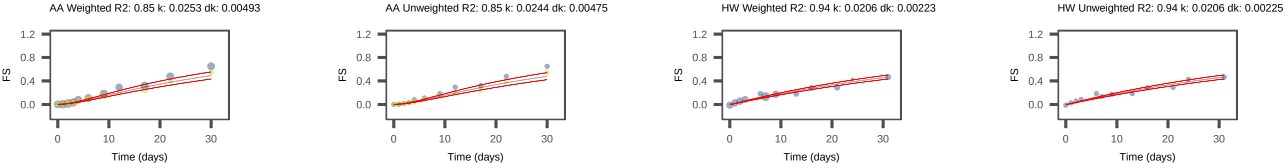

ATPG

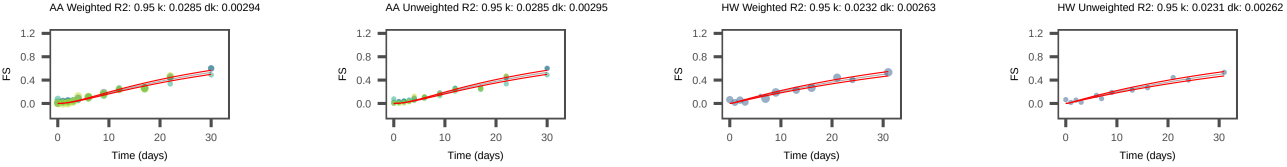

ATPO

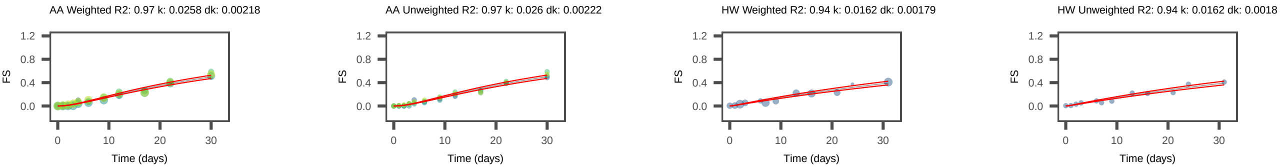

BIN1

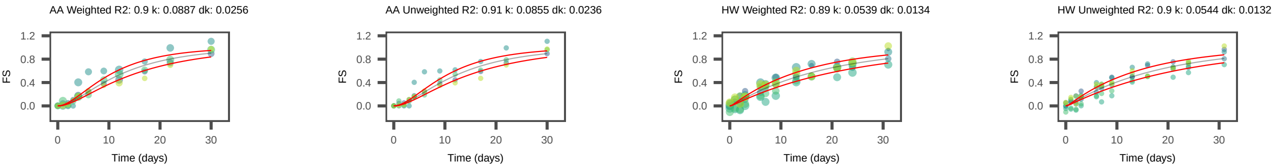

CAH3

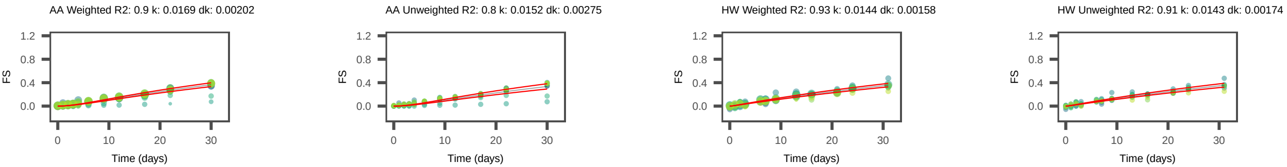

CH10

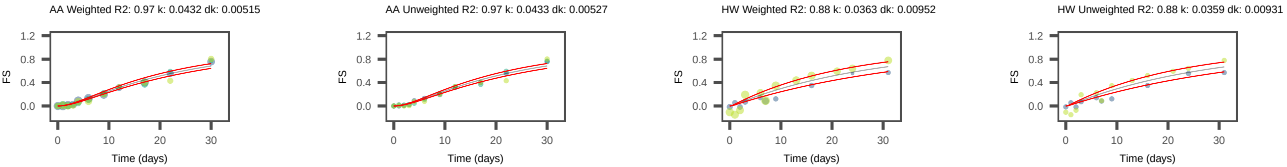

CH60

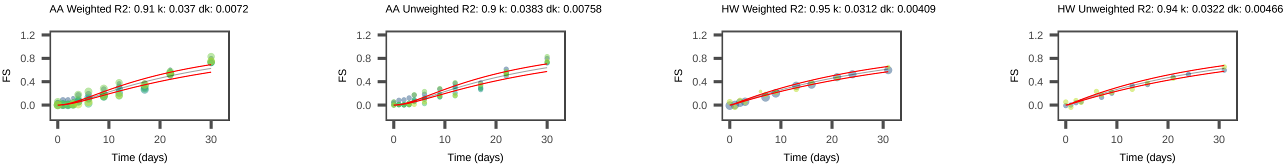

CISD1

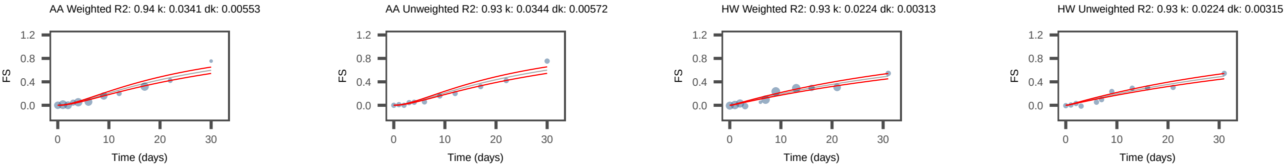

CLH1

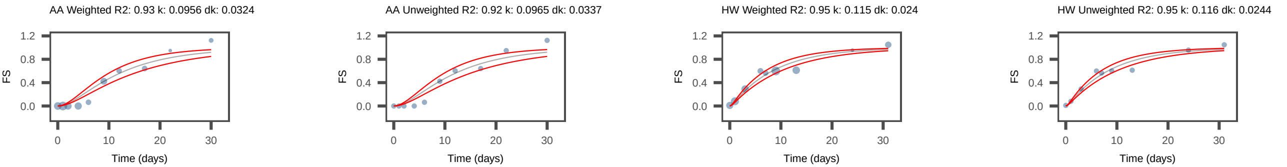

CMC1

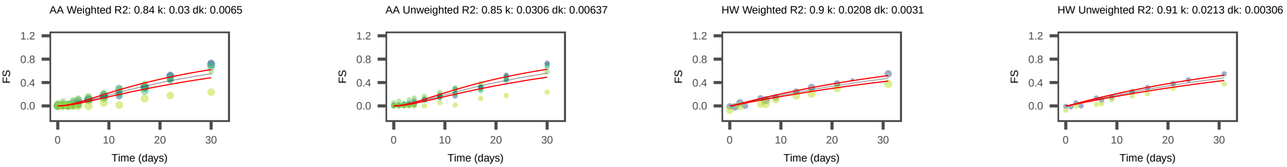

COF2

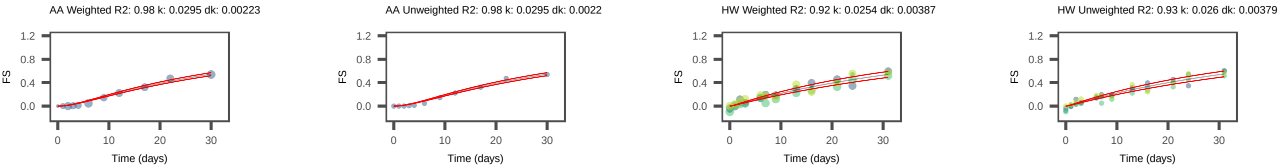

COQ9

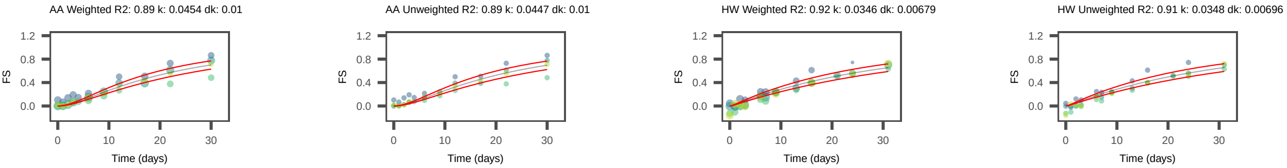

COX41

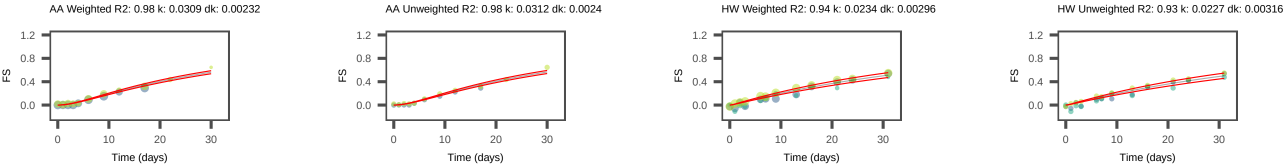

COX5A

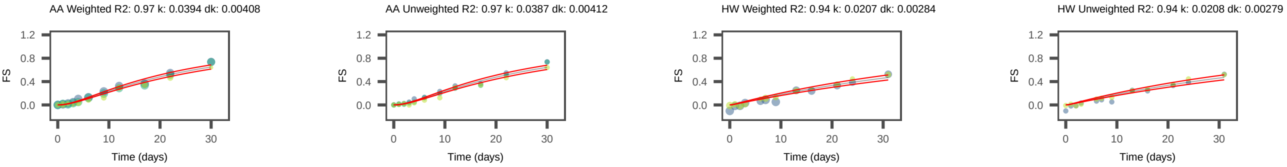

COX5B

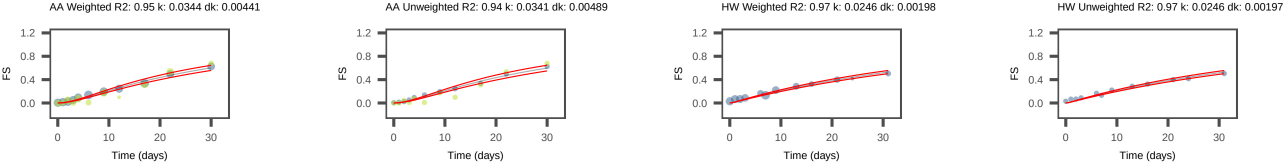

COX6C

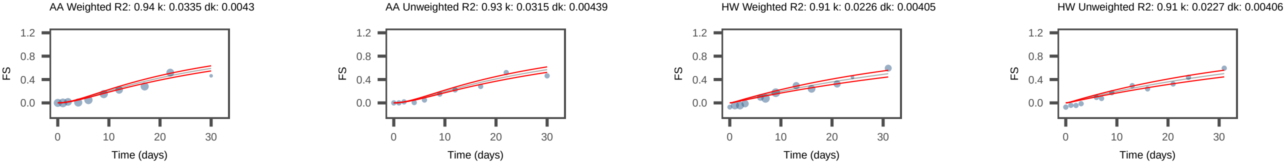

CPT1B

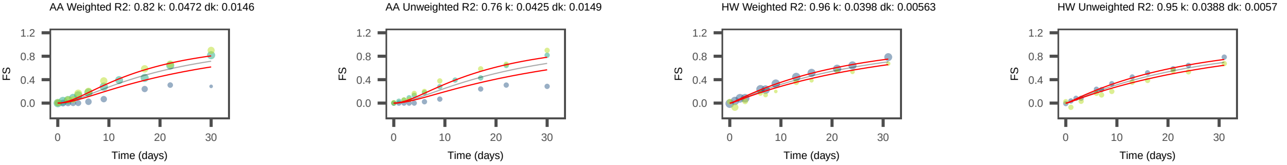

CRYAB

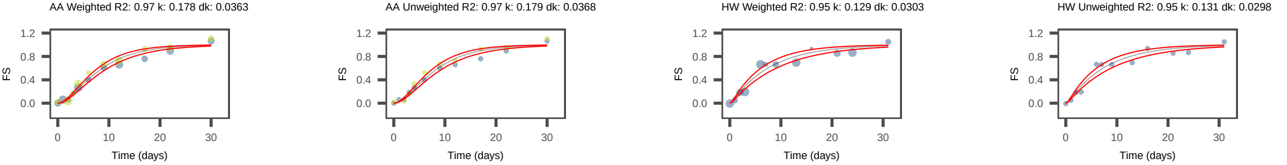

CYC

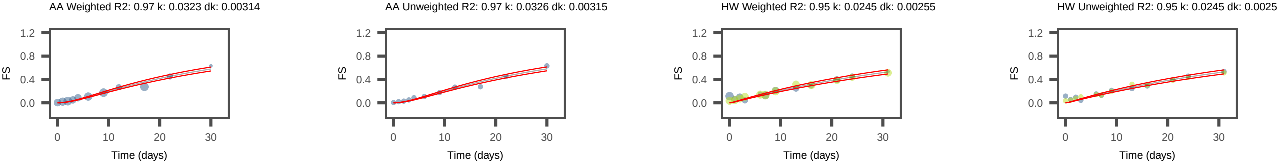

DESM

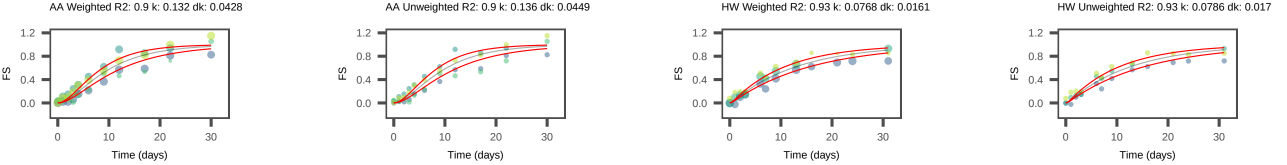

DLDH

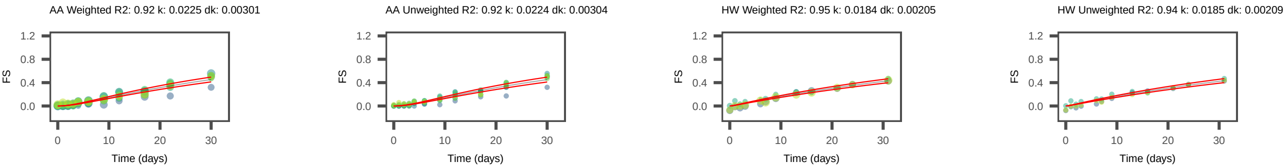

ECHA

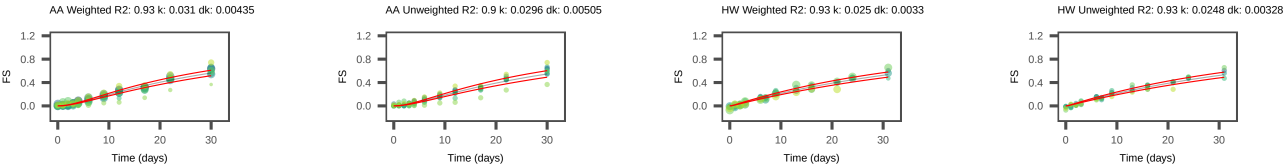

ECHB

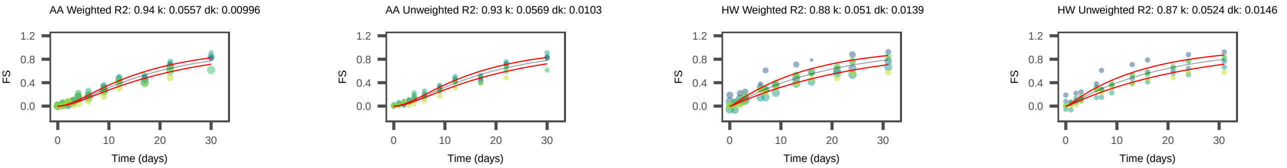

ECI1

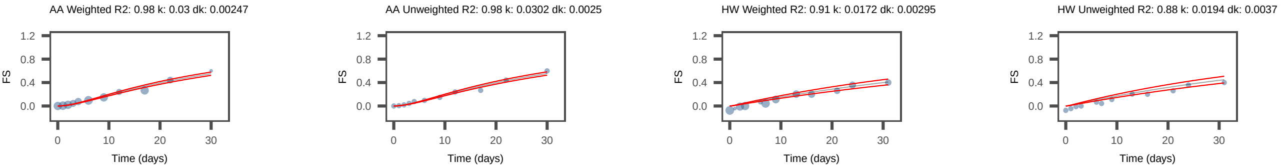

EF1A2

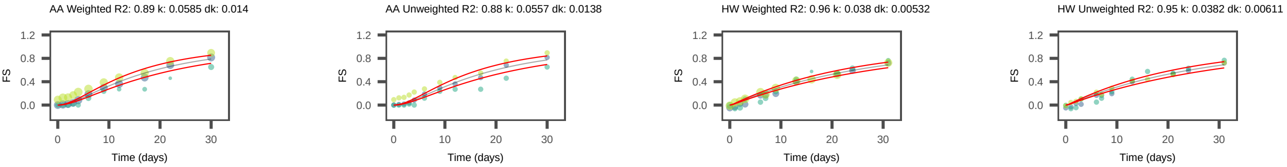

EF2

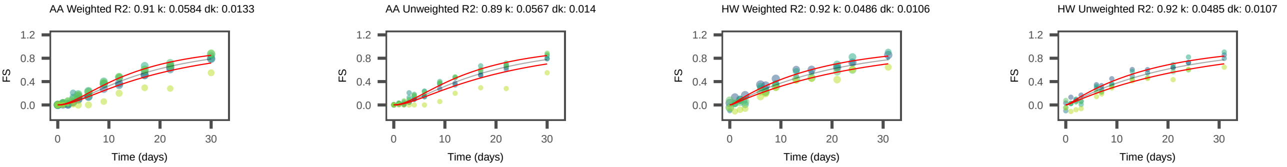

EFTU

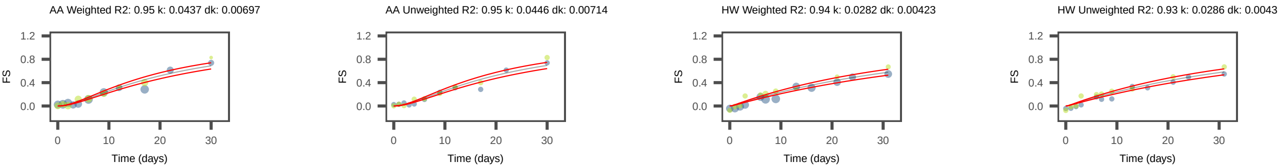

ENOB

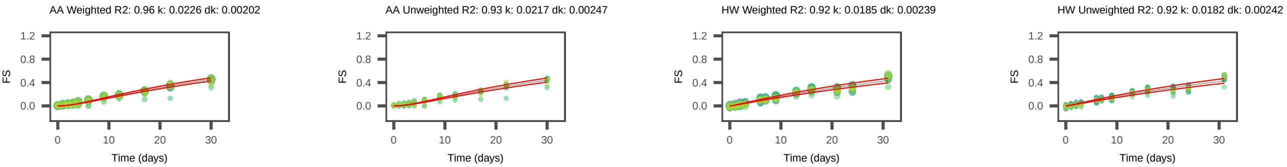

ETFA

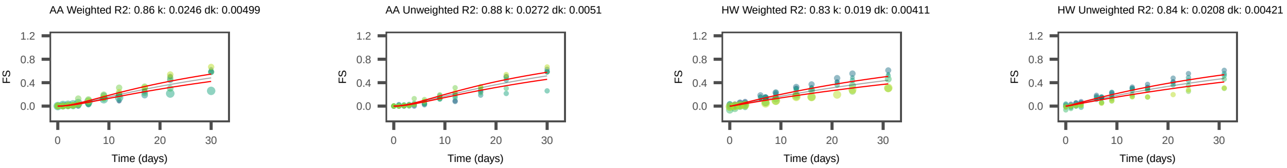

ETFD

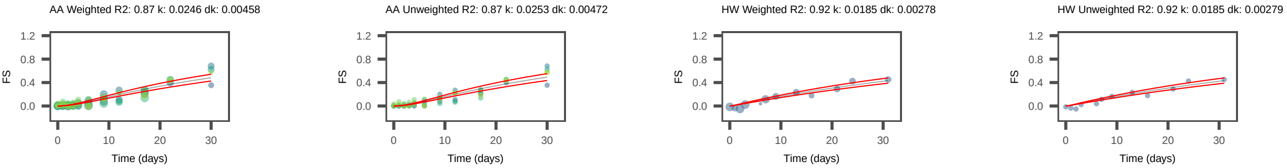

FABP4

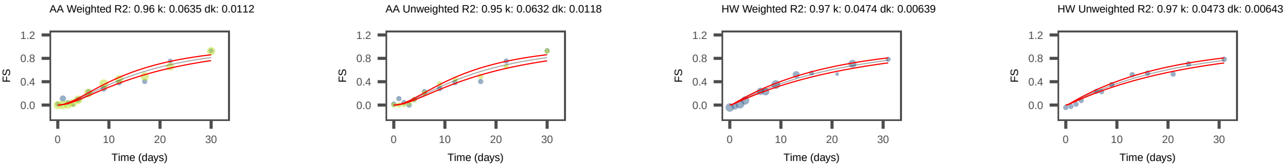

FHL1

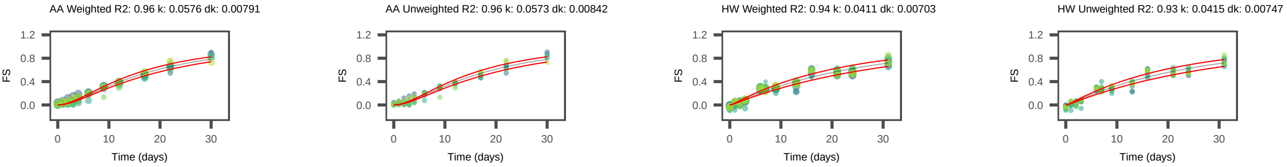

FHL3

AA Weighted R2: 0.9 k: 0.104 dk: 0.034 | AA Unweighted R2: 0.88 k: 0.0984 dk: 0.0346 | HW Weighted R2: 0.96 k: 0.08 dk: 0.0125 | HW Unweighted R2: 0.96 k: 0.0804 dk: 0.0132

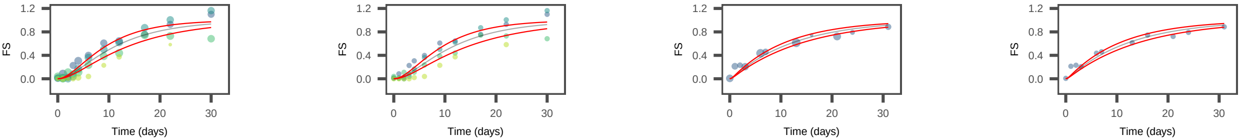

FKBP3

AA Weighted R2: 0.91 k: 0.0578 dk: 0.0144 | AA Unweighted R2: 0.9 k: 0.0587 dk: 0.0152 | HW Weighted R2: 0.96 k: 0.0404 dk: 0.00632 | HW Unweighted R2: 0.96 k: 0.0405 dk: 0.00635

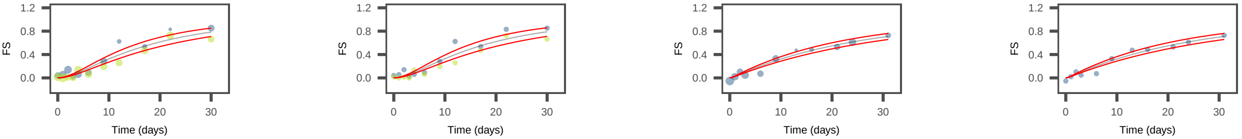

FLNC

AA Weighted R2: 0.91 k: 0.0648 dk: 0.0162 | AA Unweighted R2: 0.9 k: 0.0621 dk: 0.0162 | HW Weighted R2: 0.84 k: 0.0491 dk: 0.0147 | HW Unweighted R2: 0.84 k: 0.0488 dk: 0.0147

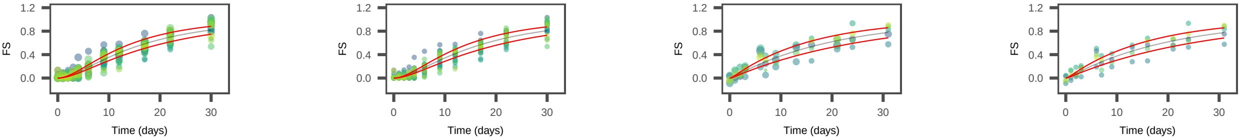

FUMH

AA Weighted R2: 0.9 k: 0.0301 dk: 0.00496 | AA Unweighted R2: 0.86 k: 0.0295 dk: 0.00583 | HW Weighted R2: 0.94 k: 0.0225 dk: 0.00295 | HW Unweighted R2: 0.94 k: 0.0226 dk: 0.00291

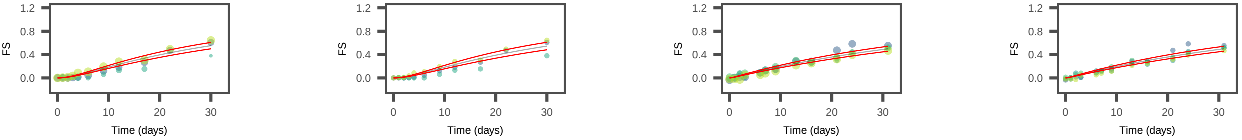

G3P

AA Weighted R2: 0.95 k: 0.0297 dk: 0.00334 | AA Unweighted R2: 0.93 k: 0.0284 dk: 0.00359 | HW Weighted R2: 0.93 k: 0.019 dk: 0.00248 | HW Unweighted R2: 0.93 k: 0.0188 dk: 0.00244

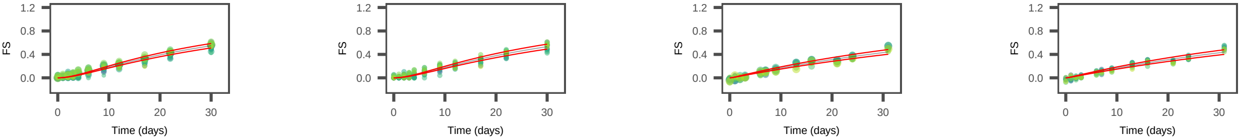

G6PI

AA Weighted R2: 0.91 k: 0.0207 dk: 0.00273 | AA Unweighted R2: 0.89 k: 0.0197 dk: 0.00291 | HW Weighted R2: 0.93 k: 0.0174 dk: 0.00214 | HW Unweighted R2: 0.93 k: 0.0174 dk: 0.00215

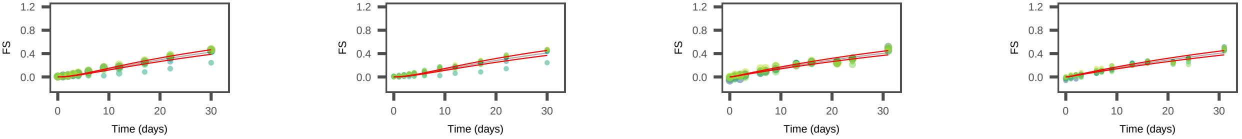

GAL3A

AA Weighted R2: 0.97 k: 0.029 dk: 0.00266 | AA Unweighted R2: 0.97 k: 0.0297 dk: 0.00276 | HW Weighted R2: 0.92 k: 0.0299 dk: 0.00402 | HW Unweighted R2: 0.92 k: 0.0299 dk: 0.004

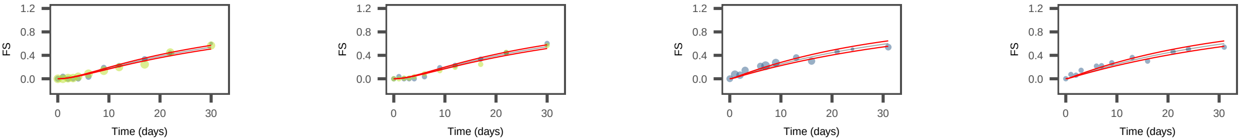

GLYG

AA Weighted R2: 0.94 k: 0.0568 dk: 0.0108 | AA Unweighted R2: 0.94 k: 0.0534 dk: 0.0103 | HW Weighted R2: 0.97 k: 0.0493 dk: 0.00643 | HW Unweighted R2: 0.97 k: 0.0495 dk: 0.00657

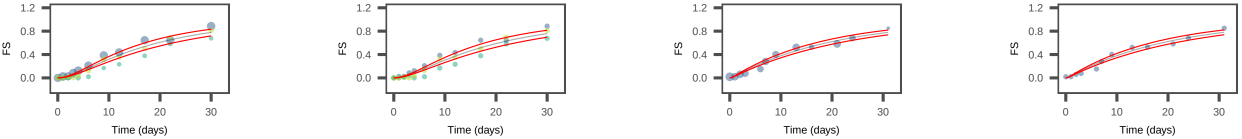

GPDA

AA Weighted R2: 0.93 k: 0.0313 dk: 0.00427 | AA Unweighted R2: 0.91 k: 0.0296 dk: 0.0046 | HW Weighted R2: 0.91 k: 0.0203 dk: 0.00299 | HW Unweighted R2: 0.91 k: 0.0205 dk: 0.00305

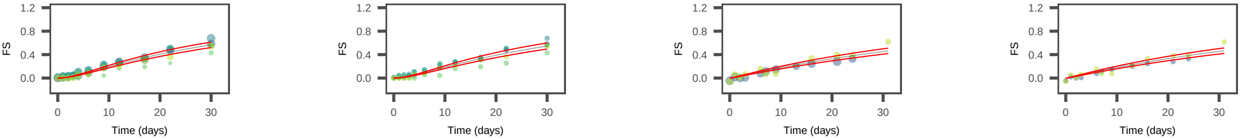

GPDM

AA Weighted R2: 0.93 k: 0.0409 dk: 0.00665 | AA Unweighted R2: 0.93 k: 0.0391 dk: 0.00628 | HW Weighted R2: 0.92 k: 0.0263 dk: 0.00499 | HW Unweighted R2: 0.92 k: 0.0264 dk: 0.005

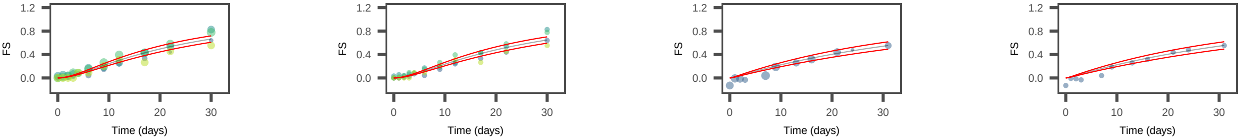

GRP75

AA Weighted R2: 0.95 k: 0.0746 dk: 0.0154 | AA Unweighted R2: 0.95 k: 0.0756 dk: 0.0159 | HW Weighted R2: 0.98 k: 0.0745 dk: 0.00891 | HW Unweighted R2: 0.98 k: 0.075 dk: 0.00893

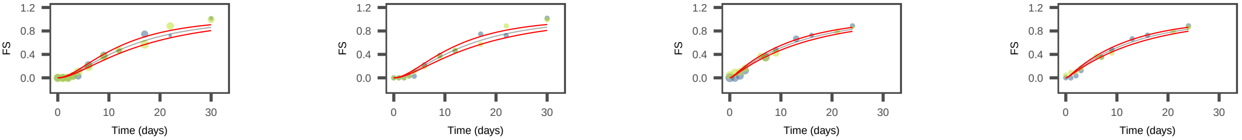

GSTM1

AA Weighted R2: 0.92 k: 0.0379 dk: 0.00592 | AA Unweighted R2: 0.92 k: 0.0356 dk: 0.0054 | HW Weighted R2: 0.93 k: 0.0165 dk: 0.00226 | HW Unweighted R2: 0.94 k: 0.0165 dk: 0.00225

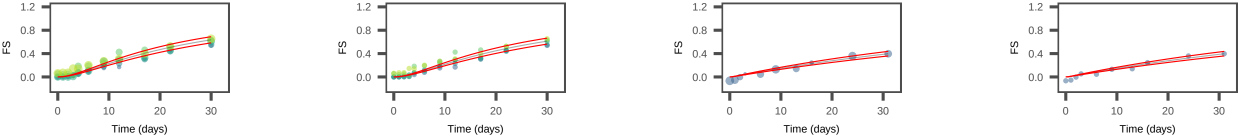

GYS1

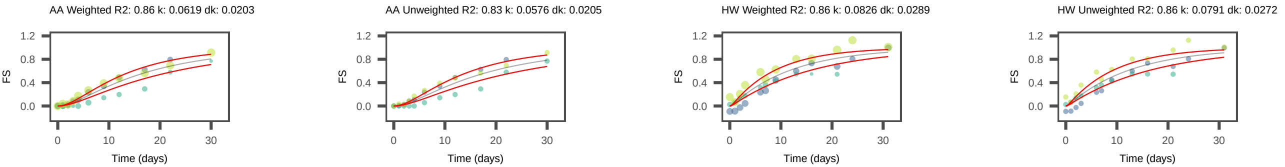

HBA

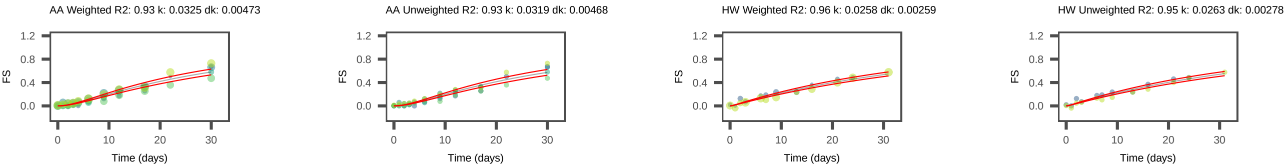

HBB1

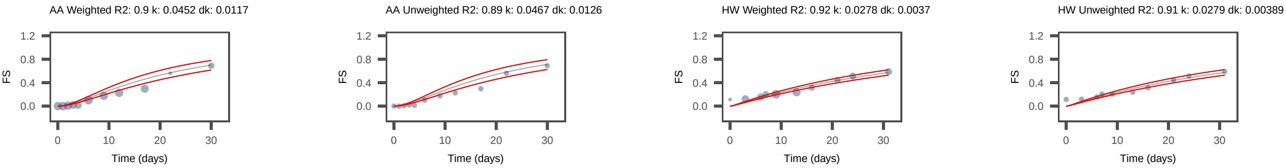

HCDH

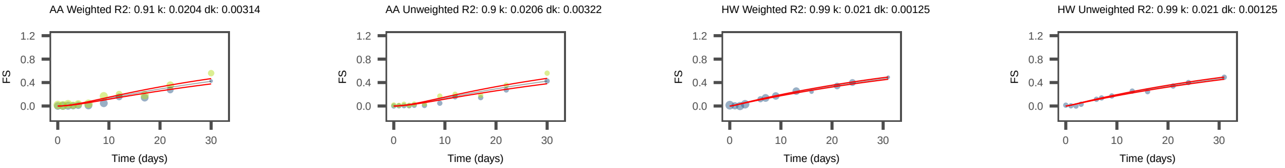

HINT1

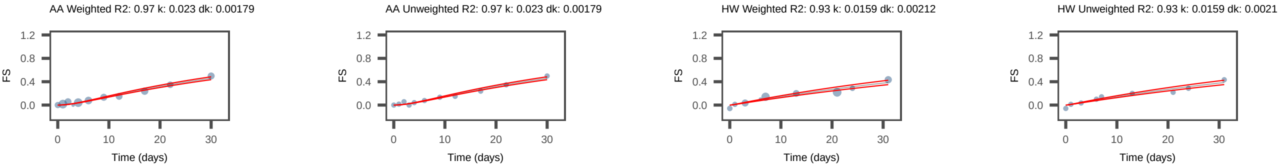

HS90B

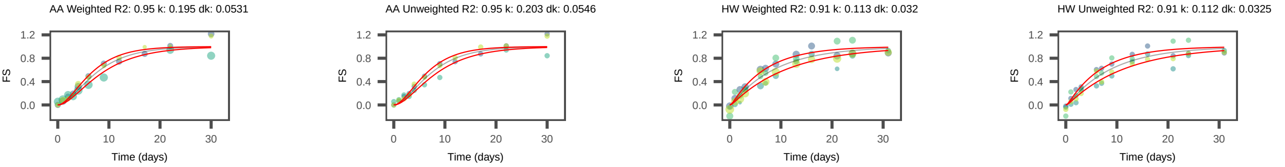

HSP7C

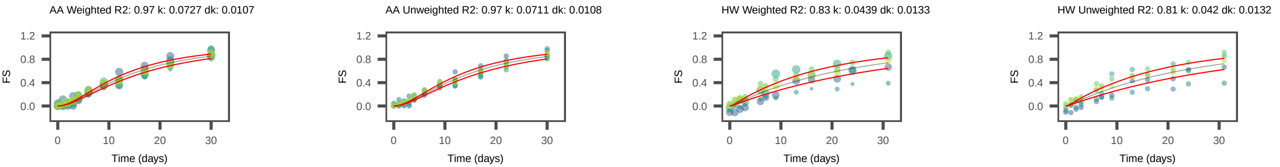

HSPB6

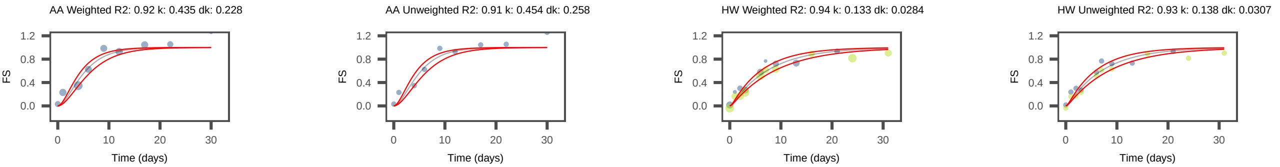

HXK2

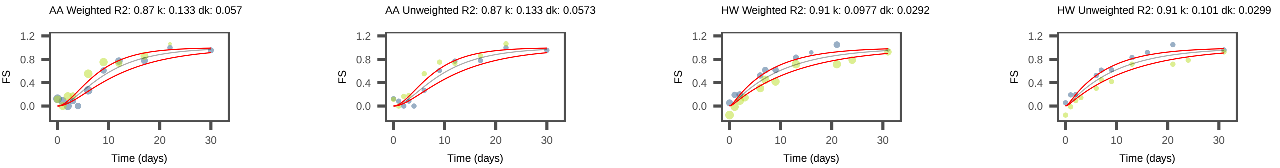

IDH3A

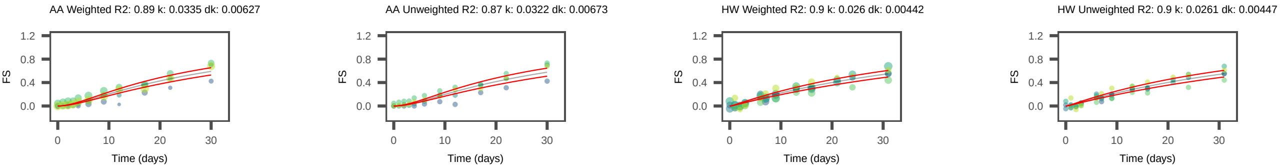

IDHG1

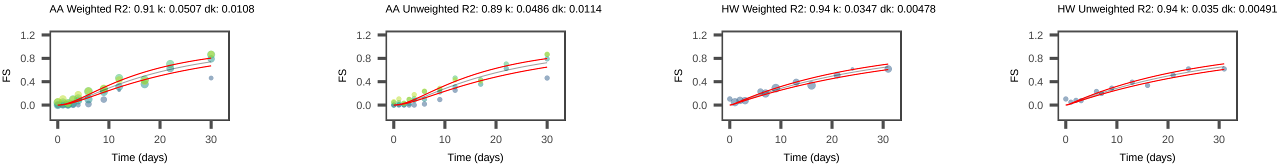

IDHP

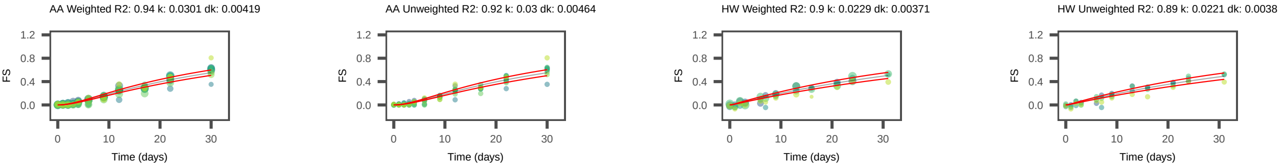

KAD1

AA Weighted R2: 0.99 k: 0.0188 dk: 0.00104

AA Unweighted R2: 0.99 k: 0.0188 dk: 0.00104

HW Weighted R2: 0.96 k: 0.0166 dk: 0.00135

HW Unweighted R2: 0.96 k: 0.0164 dk: 0.00139

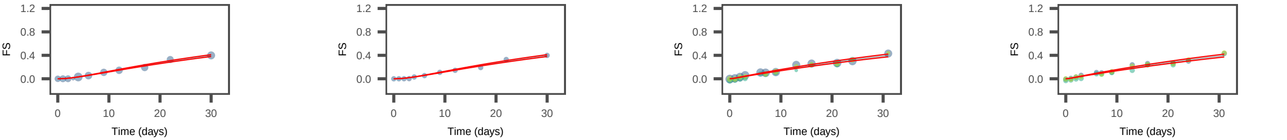

KCRM

AA Weighted R2: 0.95 k: 0.0227 dk: 0.00223

AA Unweighted R2: 0.95 k: 0.0219 dk: 0.0022

HW Weighted R2: 0.91 k: 0.0196 dk: 0.0028

HW Unweighted R2: 0.88 k: 0.02 dk: 0.00321

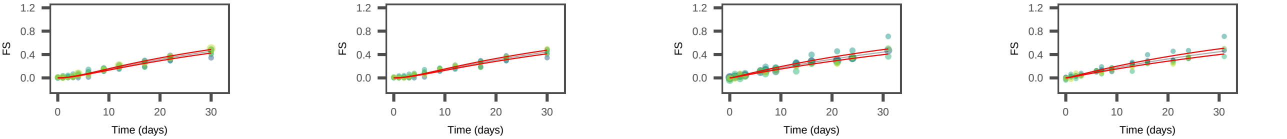

KCRS

AA Weighted R2: 0.94 k: 0.0347 dk: 0.00456

AA Unweighted R2: 0.93 k: 0.0333 dk: 0.00485

HW Weighted R2: 0.96 k: 0.0249 dk: 0.00239

HW Unweighted R2: 0.96 k: 0.0248 dk: 0.0026

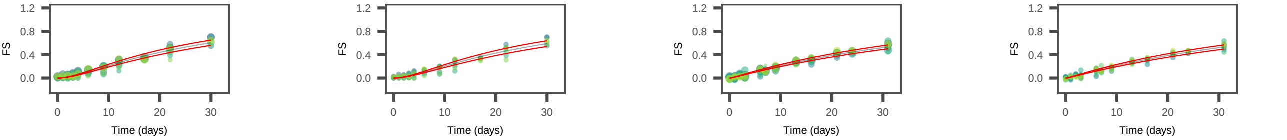

KLH41

AA Weighted R2: 0.96 k: 0.0703 dk: 0.0115

AA Unweighted R2: 0.96 k: 0.0705 dk: 0.012

HW Weighted R2: 0.91 k: 0.057 dk: 0.0138

HW Unweighted R2: 0.92 k: 0.0575 dk: 0.0137

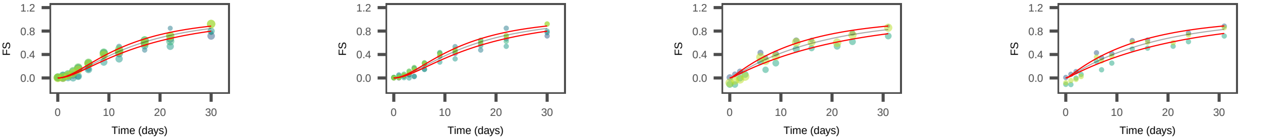

KPB1

AA Weighted R2: 0.96 k: 0.11 dk: 0.022

AA Unweighted R2: 0.97 k: 0.11 dk: 0.0214

HW Weighted R2: 0.95 k: 0.0809 dk: 0.0185

HW Unweighted R2: 0.95 k: 0.0814 dk: 0.0192

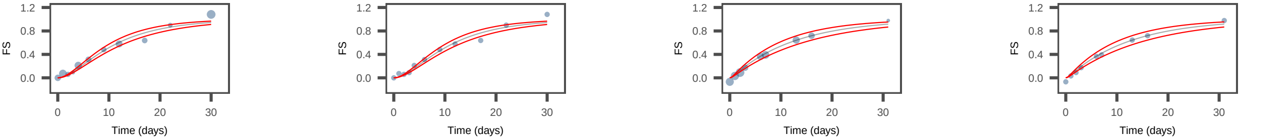

KPBB

AA Weighted R2: 0.95 k: 0.0599 dk: 0.0104

AA Unweighted R2: 0.95 k: 0.0588 dk: 0.0104

HW Weighted R2: 0.99 k: 0.0568 dk: 0.00476

HW Unweighted R2: 0.99 k: 0.0568 dk: 0.00478

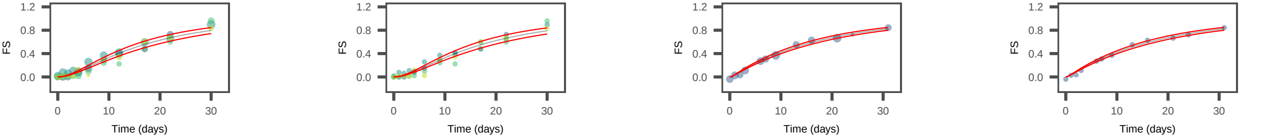

KPYM

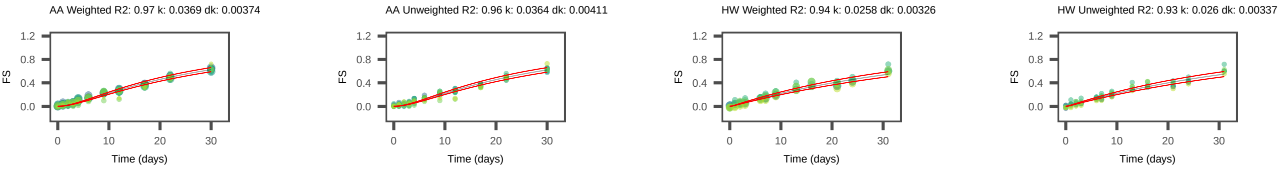

LDB3

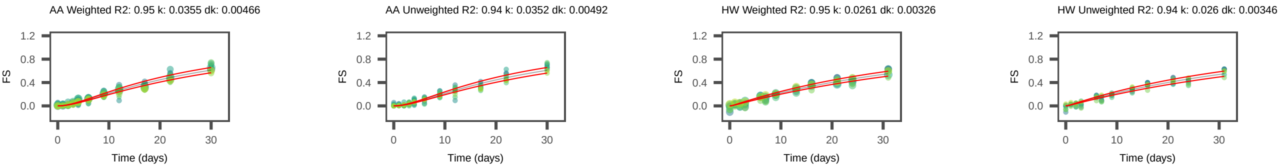

LDHA

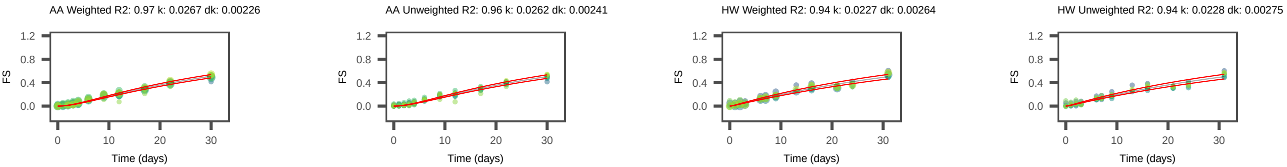

LDHB

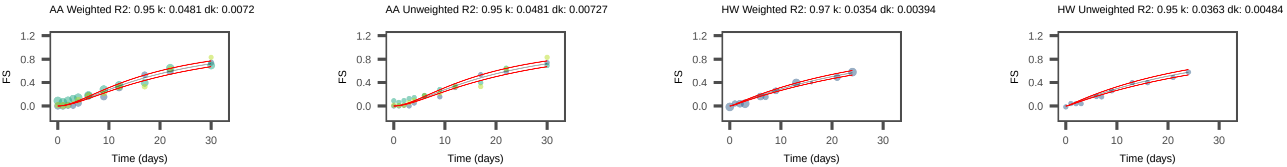

LEG1

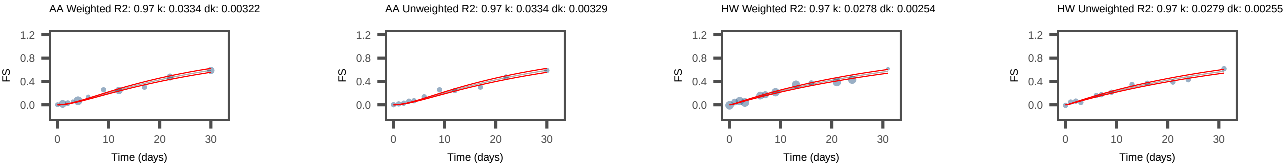

LGUL

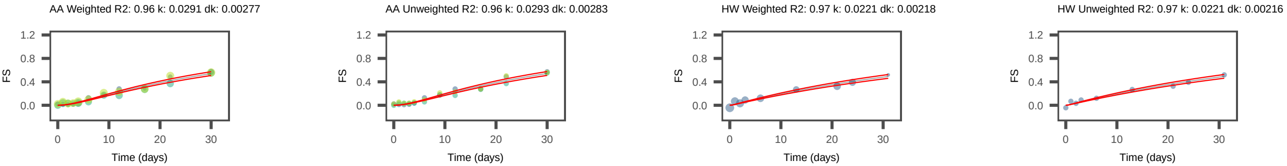

M2OM

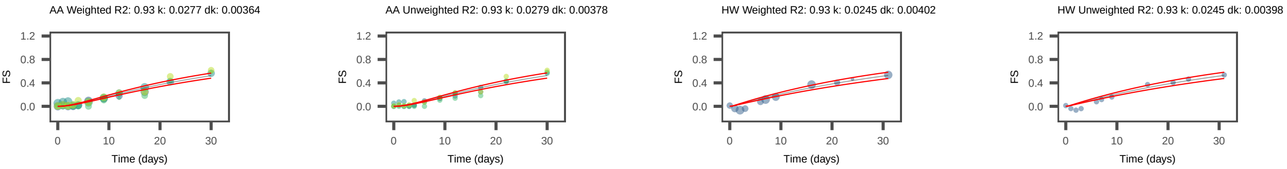

MDHC

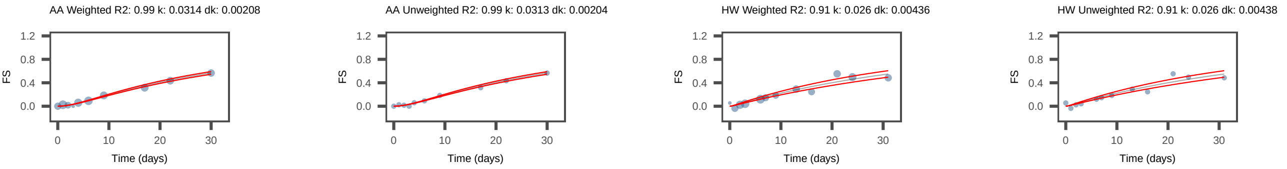

MDHM

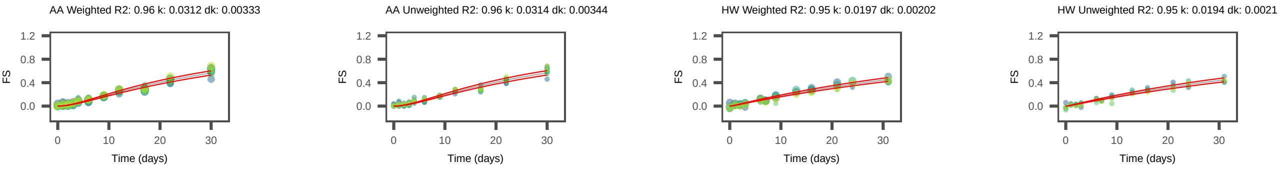

MIF

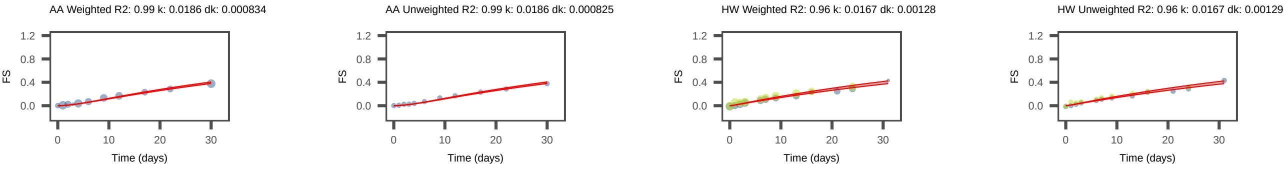

MLRS

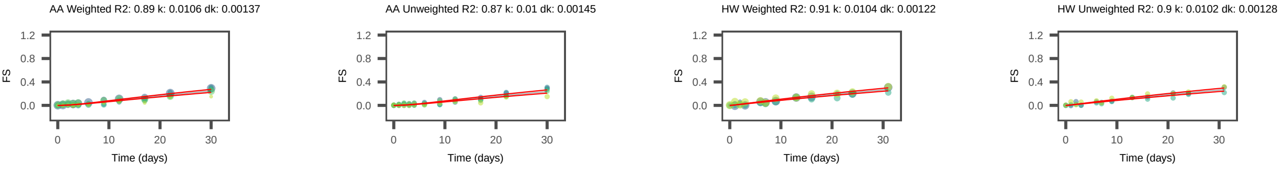

MMSA

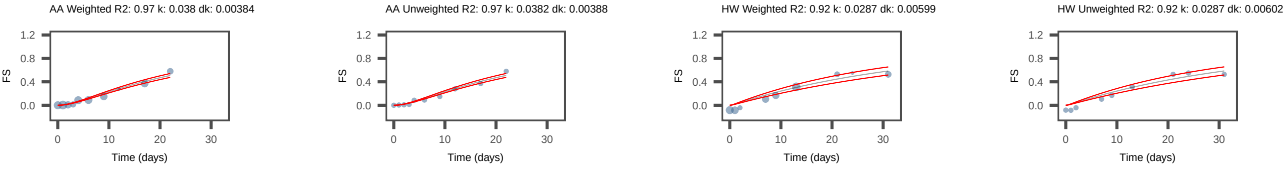

MPC2

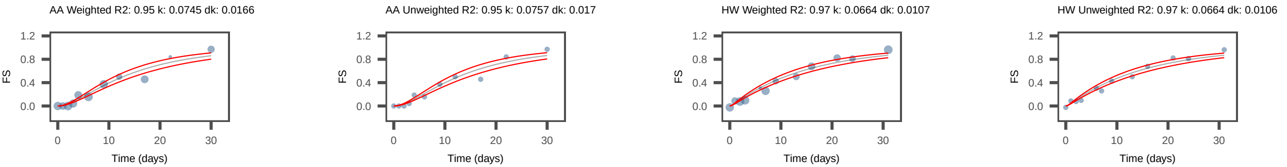

MPCP

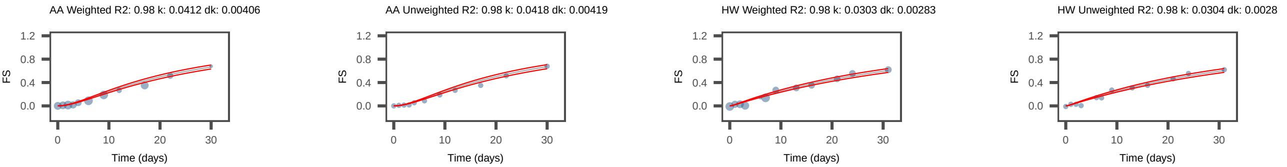

MYG

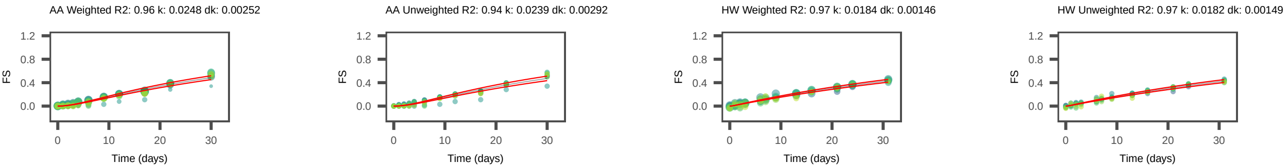

MYH1

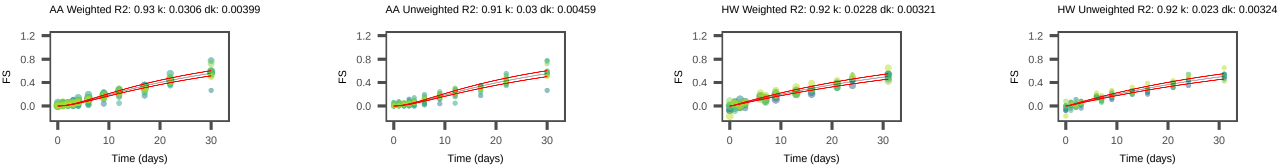

MYH4

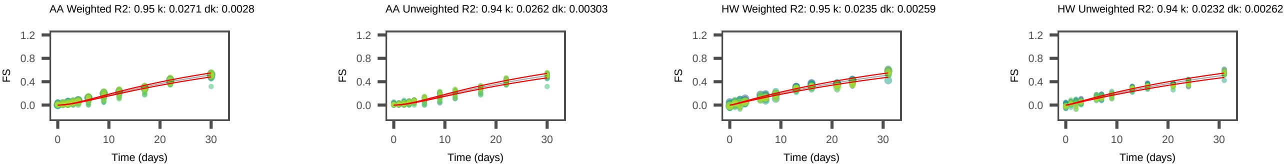

MYH7

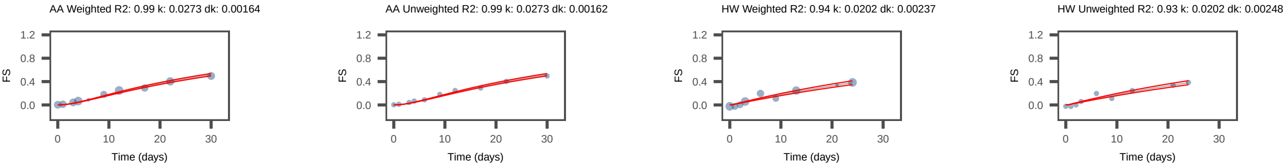

MYH8

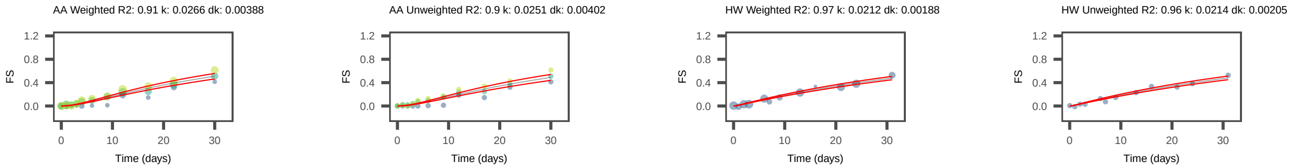

MYL1

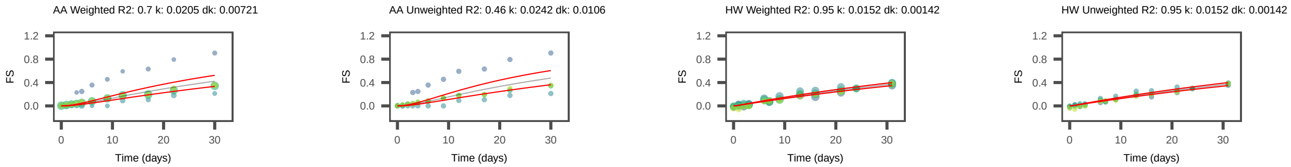

MYOM1

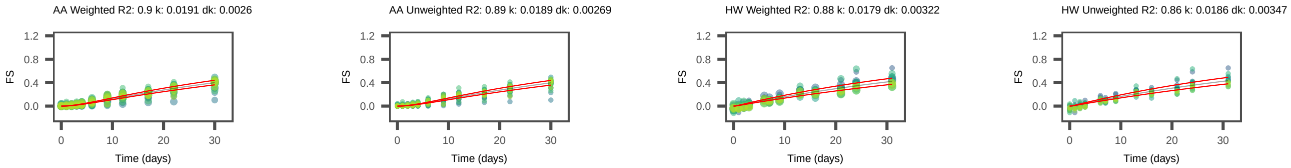

MYOTI

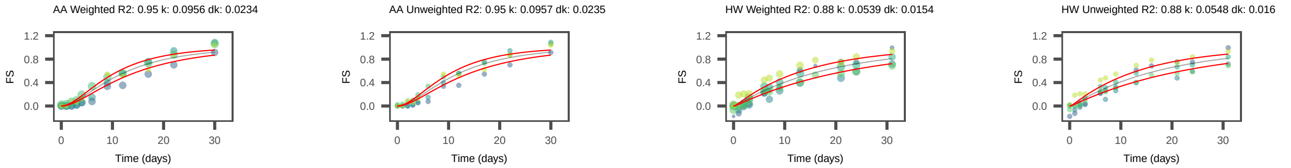

MYOZ1

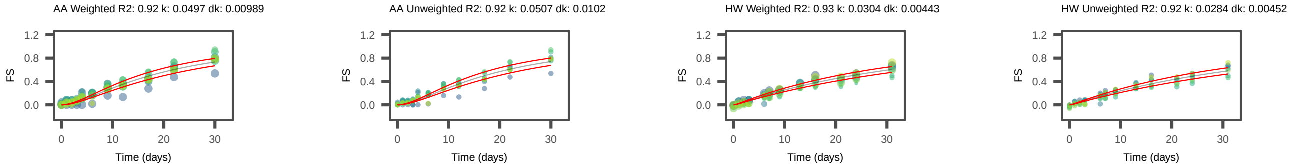

MYPC2

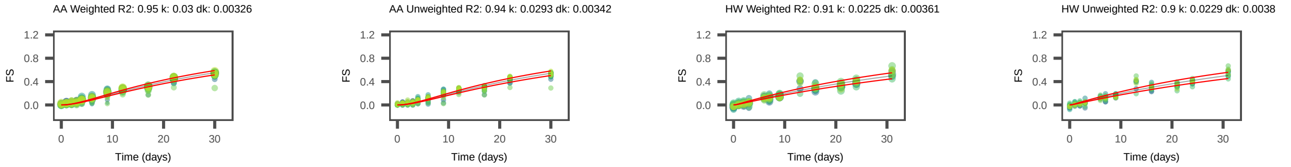

NACAM

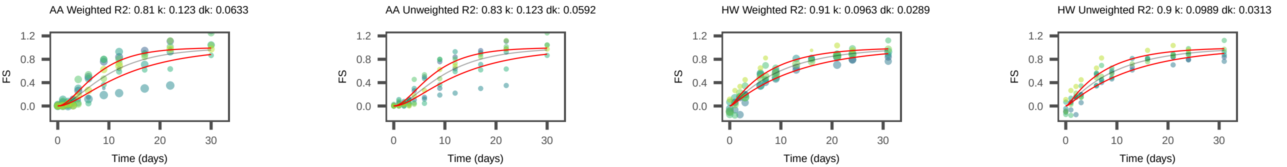

NDKB

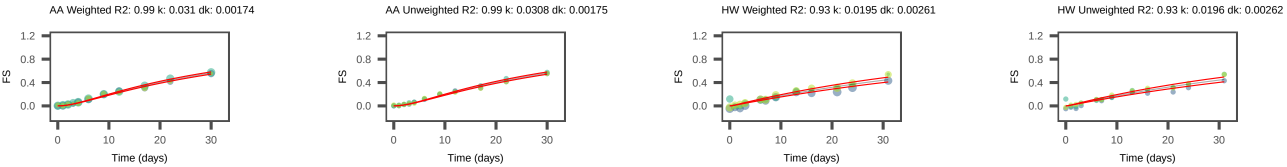

NDRG2

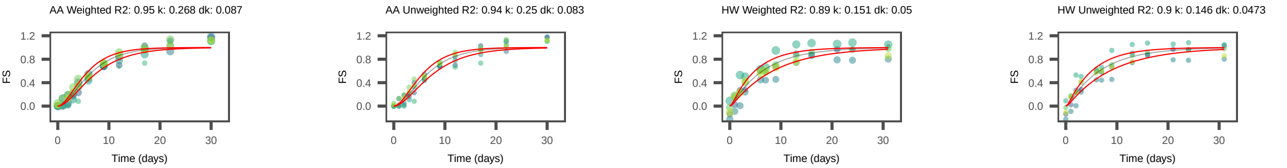

NDUA7

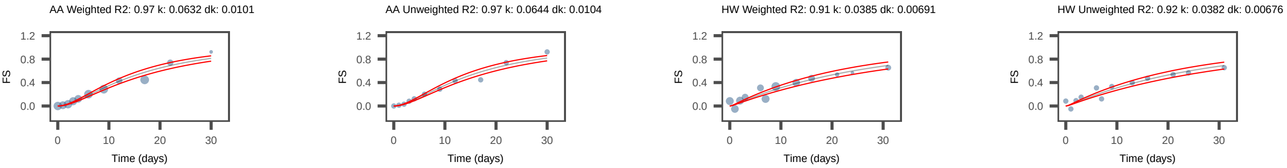

NDUA9

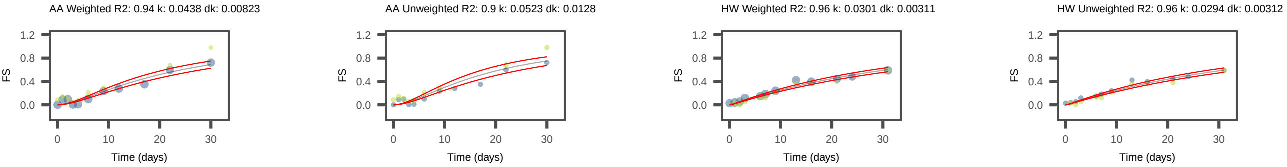

NDUAA

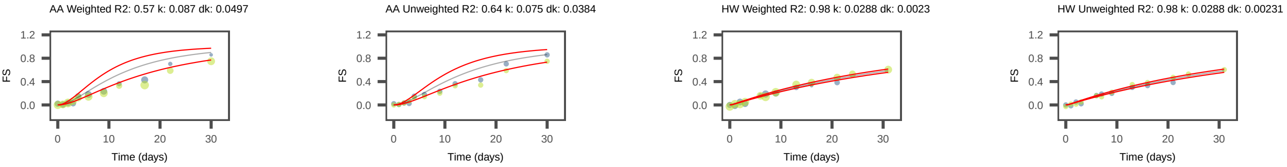

NDUAD

AA Weighted R2: 0.97 k: 0.0411 dk: 0.00462 | AA Unweighted R2: 0.97 k: 0.0414 dk: 0.00473 | HW Weighted R2: 0.92 k: 0.0238 dk: 0.00387 | HW Unweighted R2: 0.93 k: 0.0239 dk: 0.00383

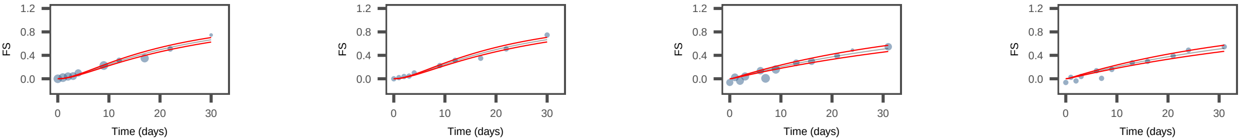

NDUB9

AA Weighted R2: 0.96 k: 0.0405 dk: 0.00551 | AA Unweighted R2: 0.96 k: 0.0409 dk: 0.00555 | HW Weighted R2: 0.92 k: 0.0174 dk: 0.00258 | HW Unweighted R2: 0.92 k: 0.0174 dk: 0.00257

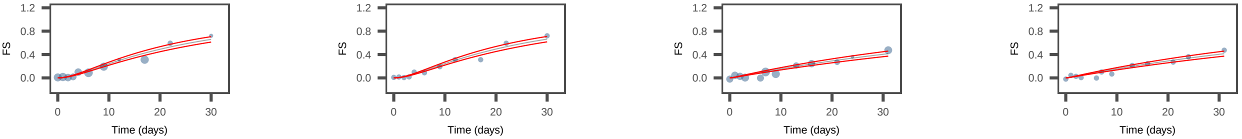

NDUBA

AA Weighted R2: 0.94 k: 0.014 dk: 0.00155 | AA Unweighted R2: 0.94 k: 0.0141 dk: 0.00159 | HW Weighted R2: 0.9 k: 0.016 dk: 0.00242 | HW Unweighted R2: 0.9 k: 0.0159 dk: 0.00244

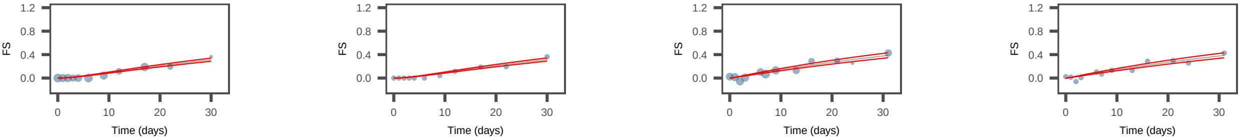

NDUS1

AA Weighted R2: 0.87 k: 0.0398 dk: 0.00936 | AA Unweighted R2: 0.86 k: 0.0394 dk: 0.00954 | HW Weighted R2: 0.96 k: 0.0328 dk: 0.00402 | HW Unweighted R2: 0.96 k: 0.033 dk: 0.0041

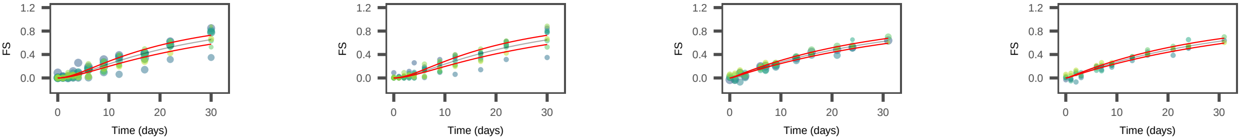

NDUS2

AA Weighted R2: 0.95 k: 0.0638 dk: 0.0116 | AA Unweighted R2: 0.95 k: 0.0644 dk: 0.0117 | HW Weighted R2: 0.96 k: 0.0333 dk: 0.00436 | HW Unweighted R2: 0.96 k: 0.0334 dk: 0.00438

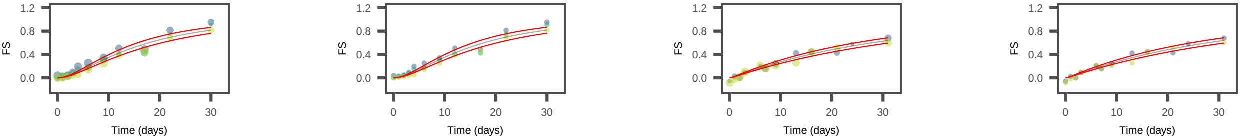

NDUS3

AA Weighted R2: 0.95 k: 0.0486 dk: 0.00804 | AA Unweighted R2: 0.95 k: 0.0495 dk: 0.00867 | HW Weighted R2: 0.9 k: 0.0275 dk: 0.0057 | HW Unweighted R2: 0.9 k: 0.0275 dk: 0.00567

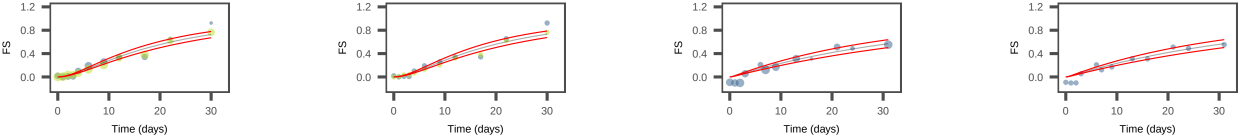

NDUS4

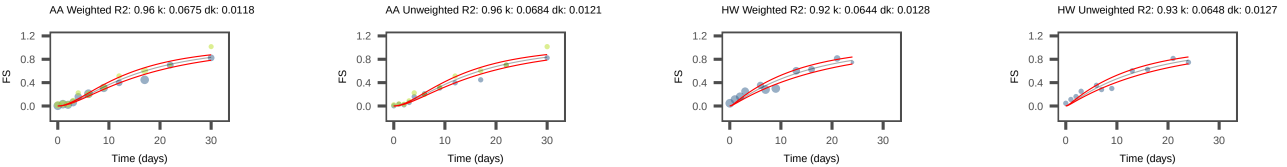

NDUS8

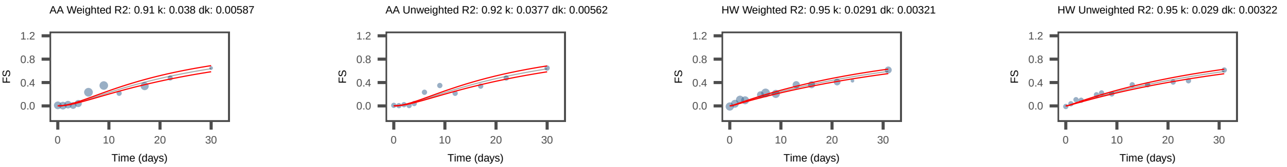

NDUV1

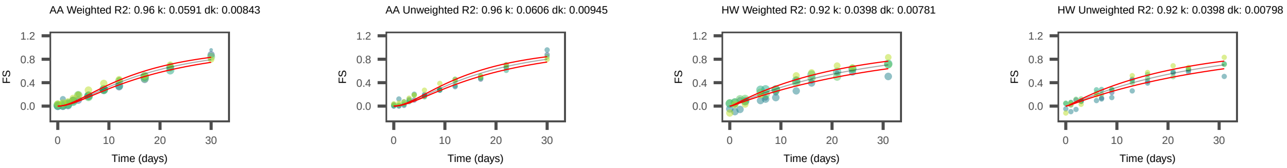

NNTM

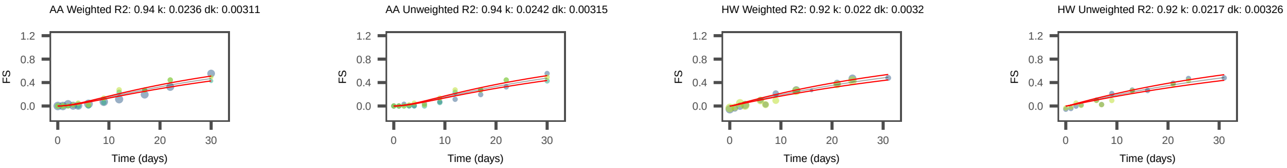

OBSCN

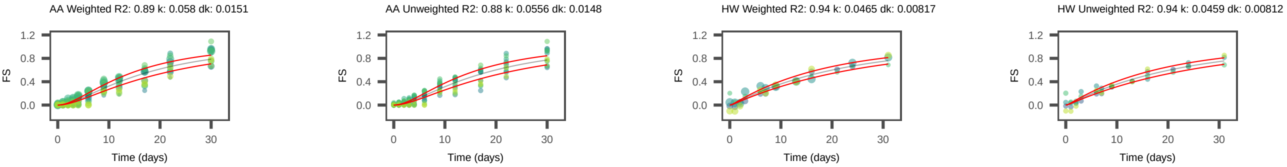

ODO1

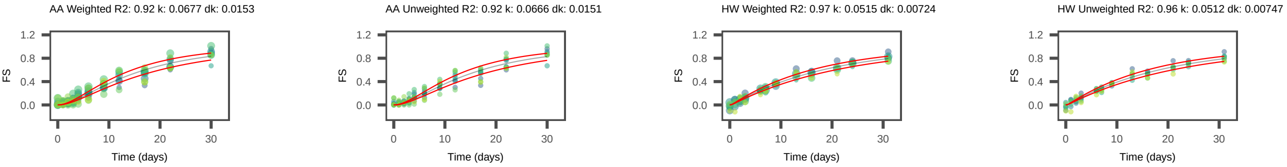

ODP2

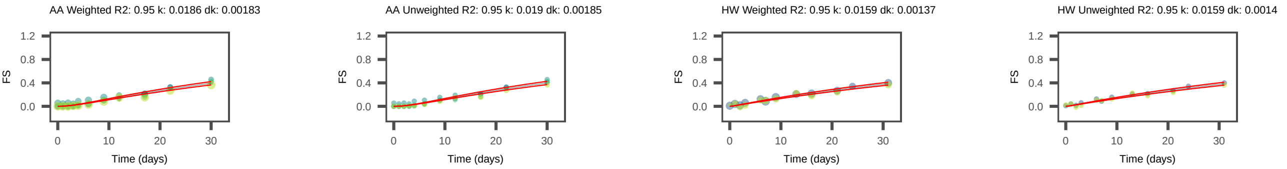

ODPB

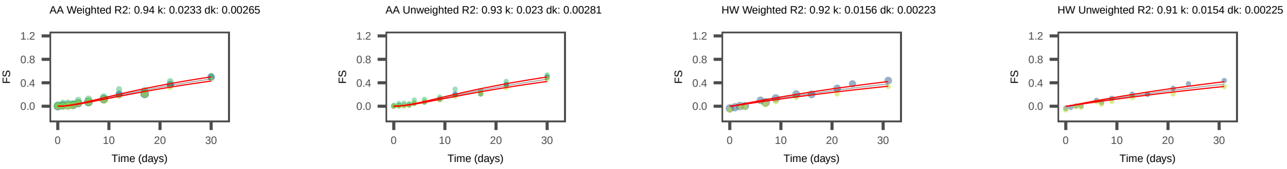

PADI2

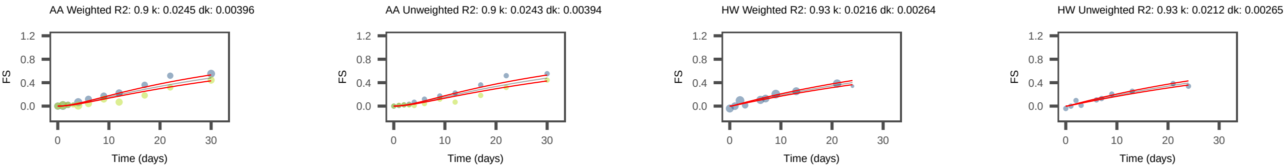

PARK7

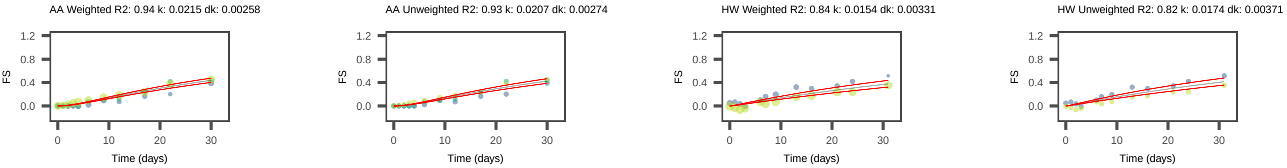

PDLI5

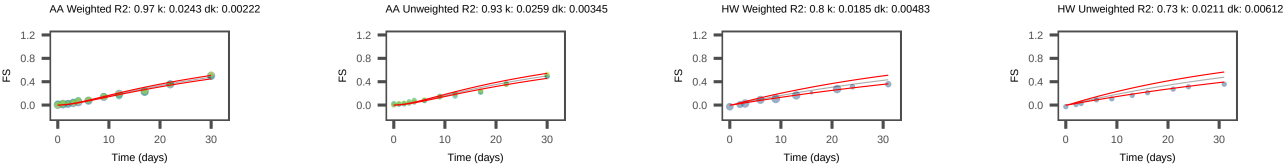

PFKAM

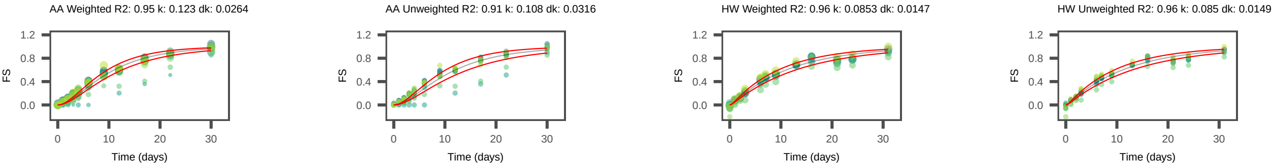

PGAM2

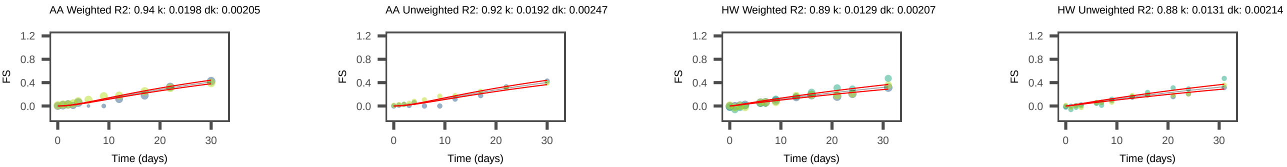

PGBM

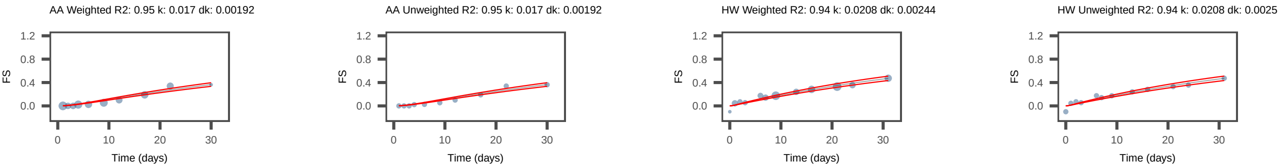

PGK1

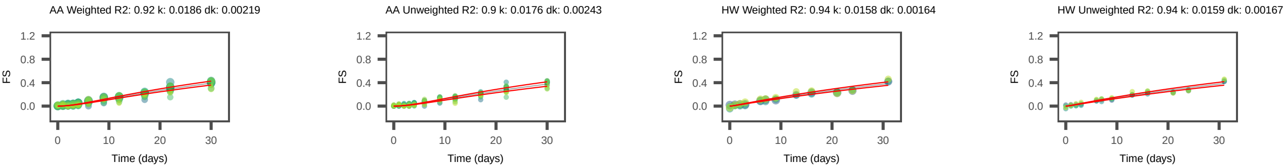

PGM1

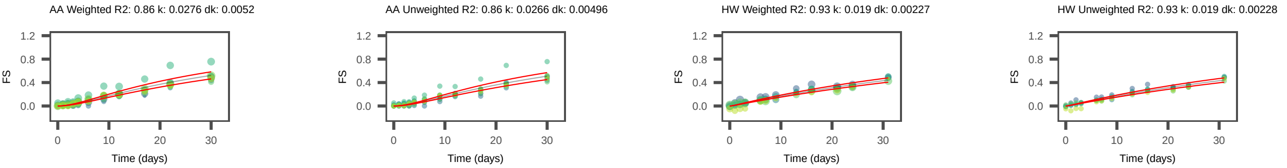

PGS2

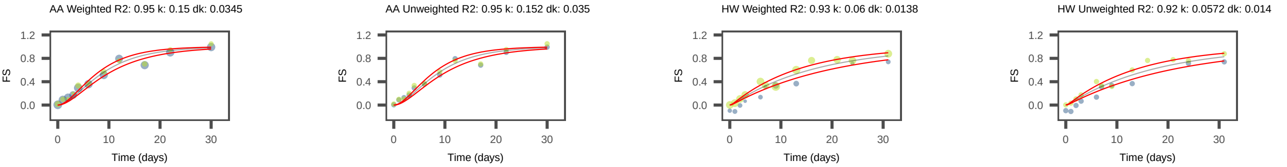

PLEC

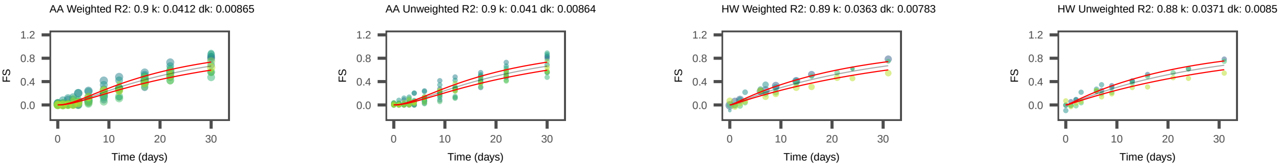

PLIN4

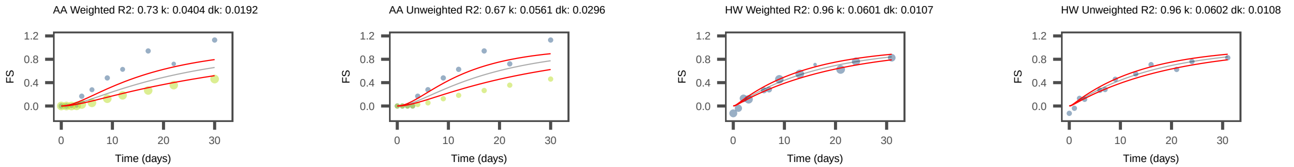

PRDX2

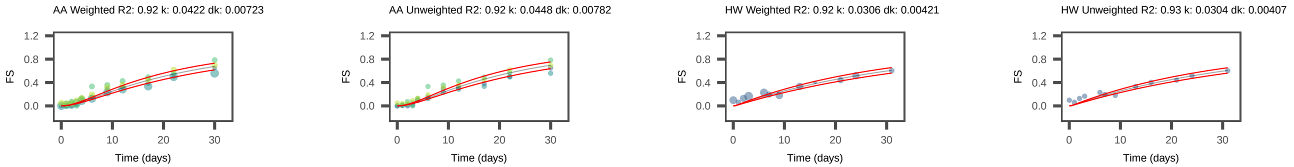

PRDX3

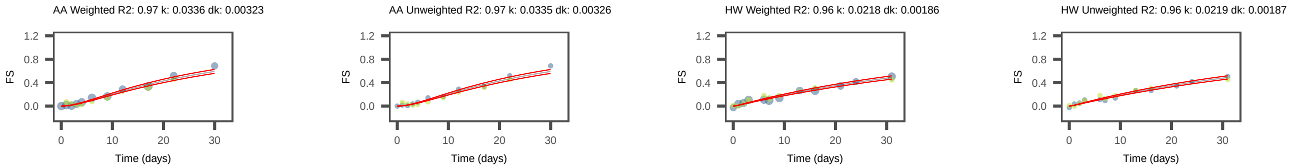

PRDX6

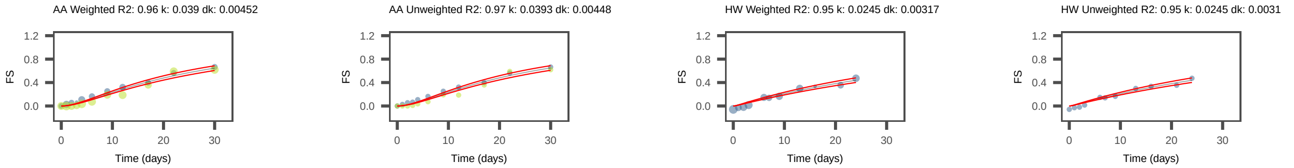

PROF1

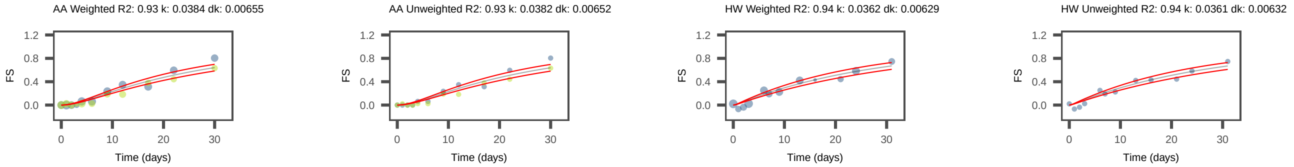

PRVA

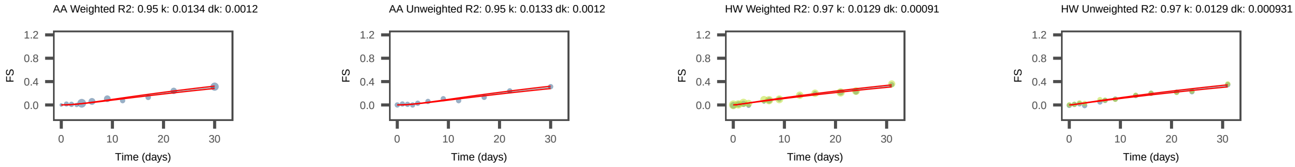

PSA5

AA Weighted R2: 0.94 k: 0.088 dk: 0.0228 | AA Unweighted R2: 0.93 k: 0.088 dk: 0.0234 | HW Weighted R2: 0.95 k: 0.0717 dk: 0.0151 | HW Unweighted R2: 0.95 k: 0.0716 dk: 0.0146

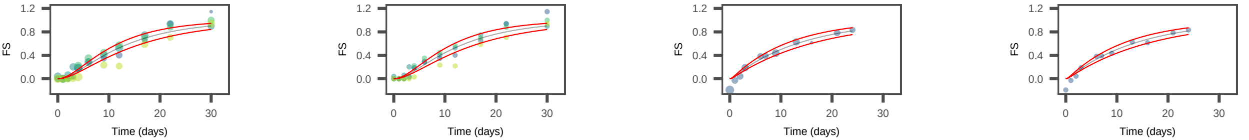

PURA1

AA Weighted R2: 0.91 k: 0.068 dk: 0.0167 | AA Unweighted R2: 0.91 k: 0.0663 dk: 0.0167 | HW Weighted R2: 0.91 k: 0.0753 dk: 0.0181 | HW Unweighted R2: 0.91 k: 0.0767 dk: 0.0191

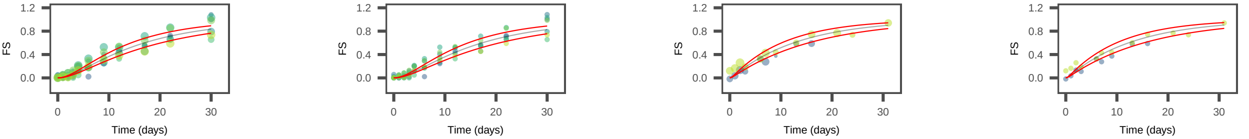

PYGM

AA Weighted R2: 0.97 k: 0.0597 dk: 0.00765 | AA Unweighted R2: 0.96 k: 0.0592 dk: 0.00804 | HW Weighted R2: 0.95 k: 0.0419 dk: 0.00666 | HW Unweighted R2: 0.94 k: 0.041 dk: 0.00678

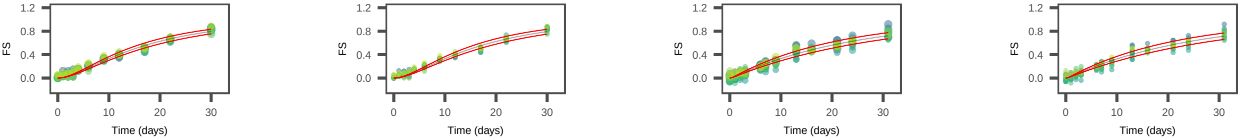

QCR1

AA Weighted R2: 0.96 k: 0.0301 dk: 0.00302 | AA Unweighted R2: 0.96 k: 0.0299 dk: 0.00307 | HW Weighted R2: 0.97 k: 0.0215 dk: 0.0018 | HW Unweighted R2: 0.97 k: 0.0217 dk: 0.00197

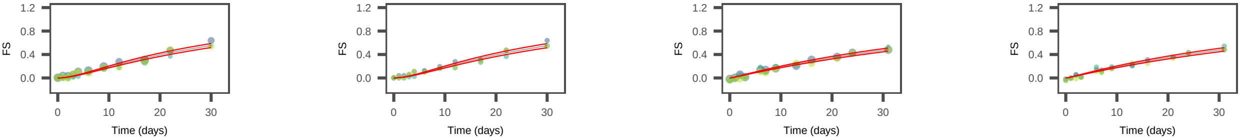

QCR2

AA Weighted R2: 0.94 k: 0.0246 dk: 0.00272 | AA Unweighted R2: 0.92 k: 0.0238 dk: 0.00301 | HW Weighted R2: 0.97 k: 0.0214 dk: 0.0016 | HW Unweighted R2: 0.98 k: 0.0215 dk: 0.00159

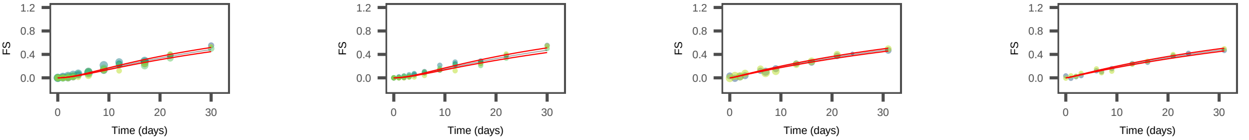

RS10

AA Weighted R2: 0.99 k: 0.0836 dk: 0.00723 | AA Unweighted R2: 0.99 k: 0.0834 dk: 0.00727 | HW Weighted R2: 0.94 k: 0.0602 dk: 0.0146 | HW Unweighted R2: 0.94 k: 0.0603 dk: 0.0148

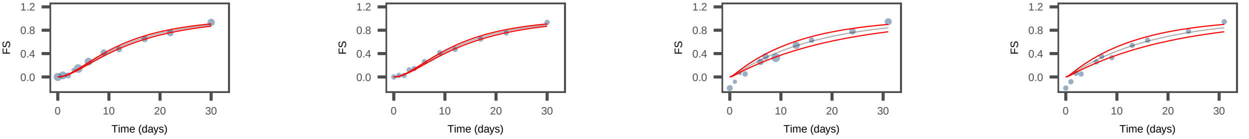

RS2

AA Weighted R2: 0.88 k: 0.0828 dk: 0.0262

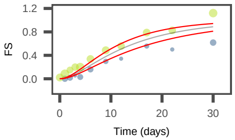

AA Unweighted R2: 0.87 k: 0.077 dk: 0.0252

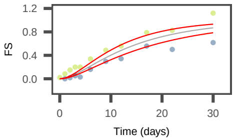

HW Weighted R2: 0.95 k: 0.0743 dk: 0.0126

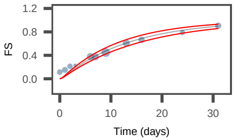

HW Unweighted R2: 0.94 k: 0.0744 dk: 0.0128

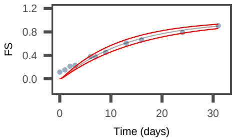

RS25

AA Weighted R2: 0.98 k: 0.0869 dk: 0.0109

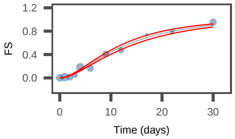

AA Unweighted R2: 0.98 k: 0.0869 dk: 0.011

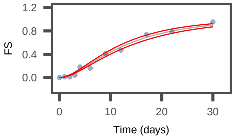

HW Weighted R2: 0.95 k: 0.0698 dk: 0.0134

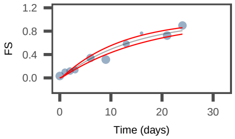

HW Unweighted R2: 0.95 k: 0.0703 dk: 0.0135

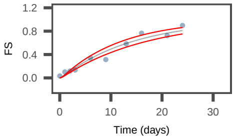

RS7

AA Weighted R2: 0.96 k: 0.0461 dk: 0.0055

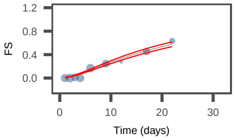

AA Unweighted R2: 0.96 k: 0.046 dk: 0.00556

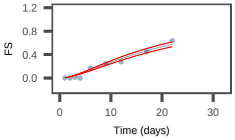

HW Weighted R2: 0.92 k: 0.0557 dk: 0.0123

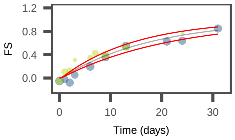

HW Unweighted R2: 0.92 k: 0.0565 dk: 0.0127

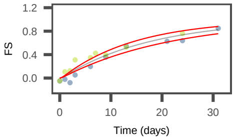

RTN2

AA Weighted R2: 0.97 k: 0.04 dk: 0.00381

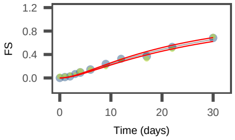

AA Unweighted R2: 0.97 k: 0.0399 dk: 0.00391

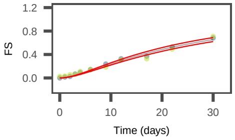

HW Weighted R2: 0.93 k: 0.0337 dk: 0.00504

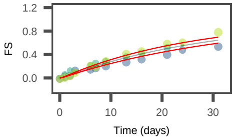

HW Unweighted R2: 0.93 k: 0.0337 dk: 0.00497

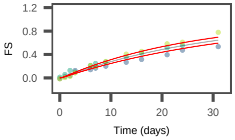

RYR1

AA Weighted R2: 0.91 k: 0.0408 dk: 0.00826

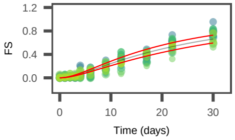

AA Unweighted R2: 0.9 k: 0.0402 dk: 0.00832

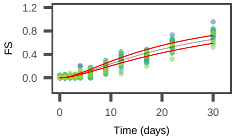

HW Weighted R2: 0.82 k: 0.0332 dk: 0.00916

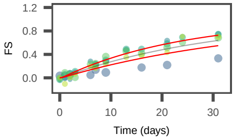

HW Unweighted R2: 0.84 k: 0.0346 dk: 0.00894

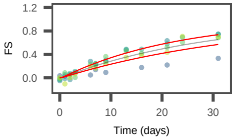

SCOT1

AA Weighted R2: 0.98 k: 0.0512 dk: 0.00584

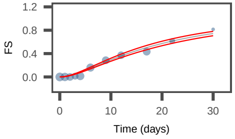

AA Unweighted R2: 0.98 k: 0.0516 dk: 0.00594

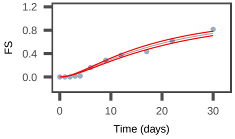

HW Weighted R2: 0.95 k: 0.0328 dk: 0.00489

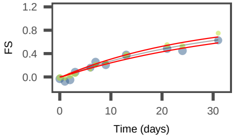

HW Unweighted R2: 0.95 k: 0.0334 dk: 0.00486

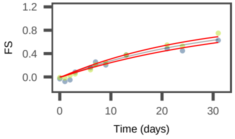

SDHA

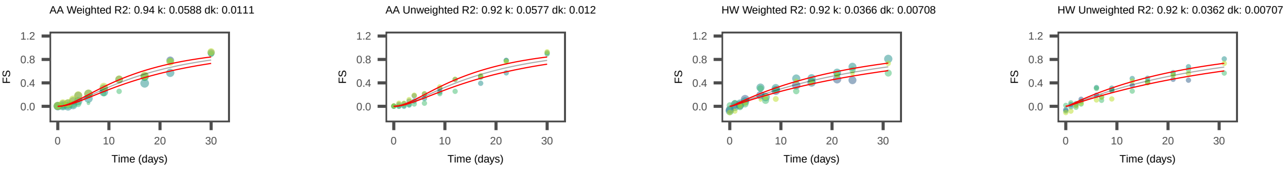

SDHB

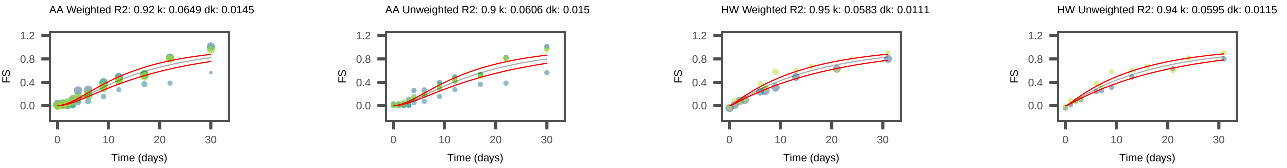

SH3BG

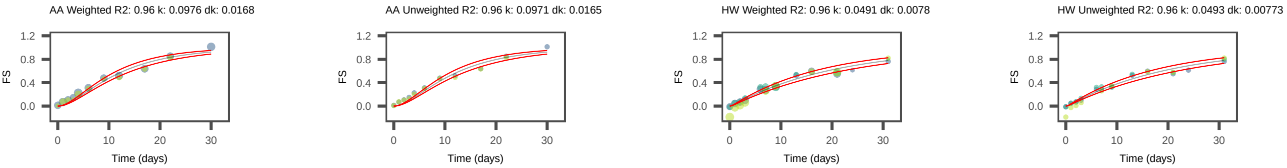

SODC

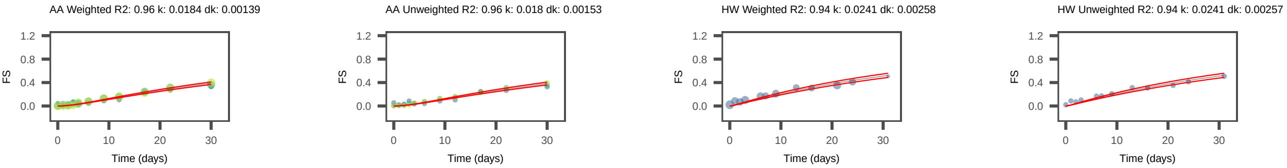

SODM

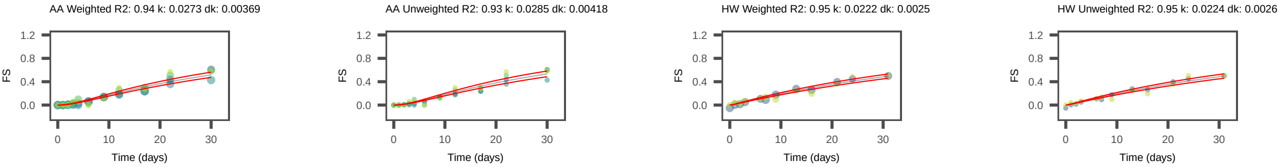

SPB6

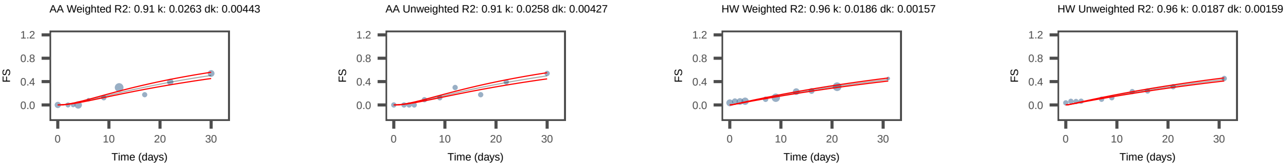

SRCA

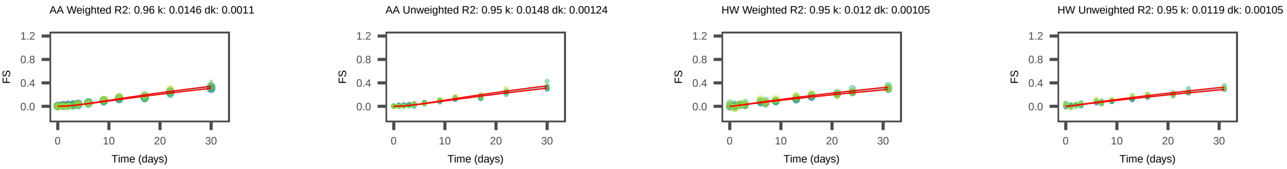

SUCB1

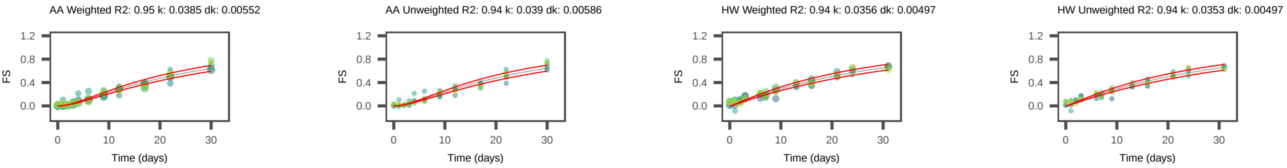

SYNP2

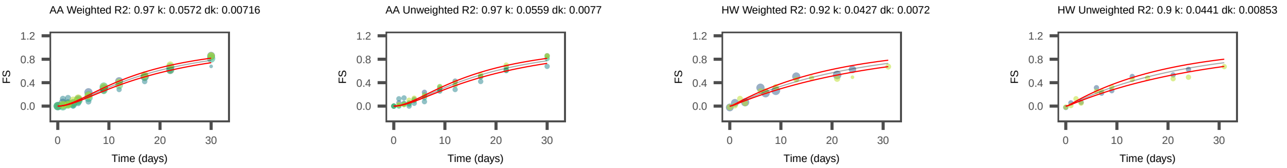

TCTP

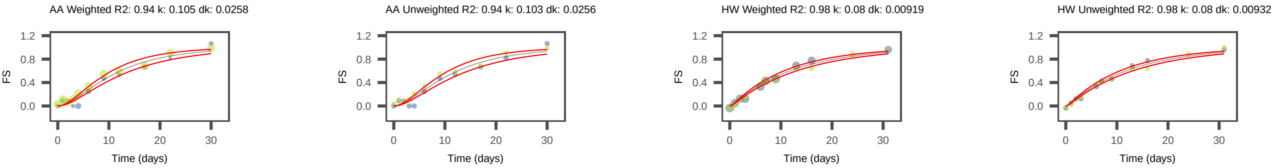

TERA

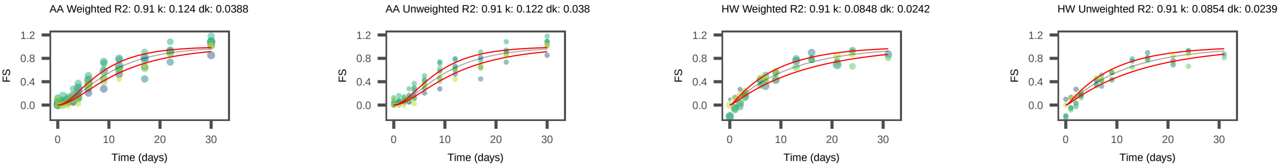

THIL

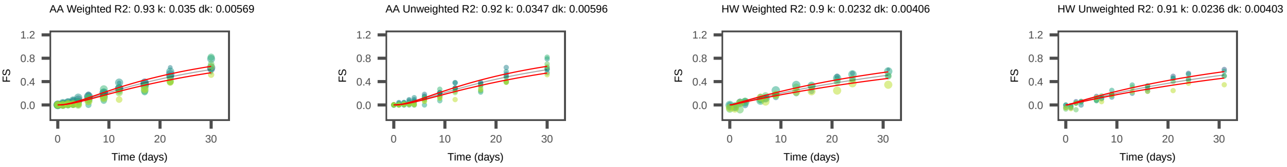

THIM

AA Weighted R2: 0.95 k: 0.0322 dk: 0.00377 | AA Unweighted R2: 0.95 k: 0.0311 dk: 0.00355 | HW Weighted R2: 0.94 k: 0.0232 dk: 0.00301 | HW Unweighted R2: 0.94 k: 0.0236 dk: 0.00313

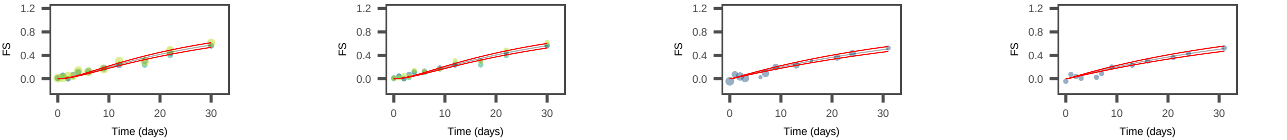

TITIN

AA Weighted R2: 0.91 k: 0.0236 dk: 0.0034 | AA Unweighted R2: 0.89 k: 0.0234 dk: 0.00361 | HW Weighted R2: 0.88 k: 0.0194 dk: 0.00321 | HW Unweighted R2: 0.87 k: 0.0196 dk: 0.00332

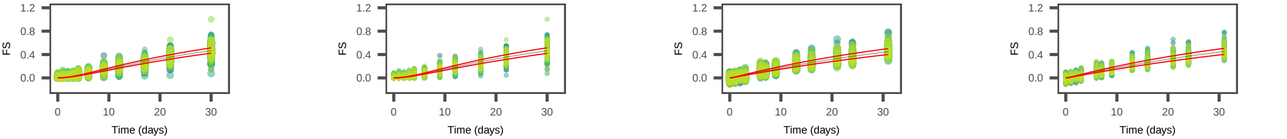

TMOD4

AA Weighted R2: 0.91 k: 0.0524 dk: 0.0113 | AA Unweighted R2: 0.91 k: 0.0525 dk: 0.0112 | HW Weighted R2: 0.96 k: 0.0387 dk: 0.00529 | HW Unweighted R2: 0.96 k: 0.0389 dk: 0.00537

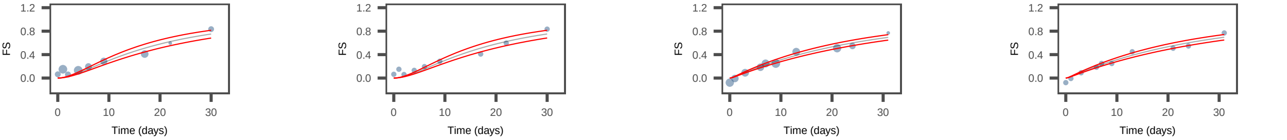

TNNC2

AA Weighted R2: 0.97 k: 0.0477 dk: 0.00588 | AA Unweighted R2: 0.96 k: 0.046 dk: 0.00589 | HW Weighted R2: 0.93 k: 0.0424 dk: 0.00769 | HW Unweighted R2: 0.93 k: 0.0416 dk: 0.0075

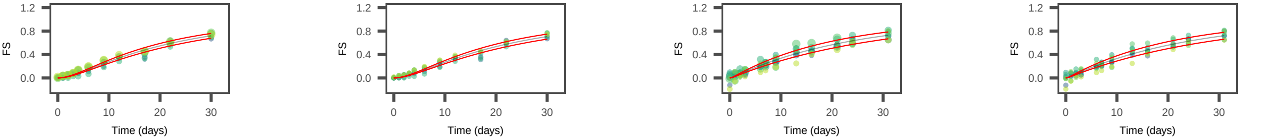

TNNI2

AA Weighted R2: 0.94 k: 0.0431 dk: 0.00689 | AA Unweighted R2: 0.92 k: 0.0412 dk: 0.00792 | HW Weighted R2: 0.94 k: 0.032 dk: 0.005 | HW Unweighted R2: 0.93 k: 0.0336 dk: 0.00543

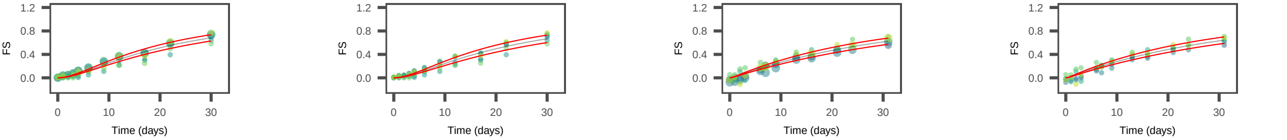

TNNT3

AA Weighted R2: 0.95 k: 0.0457 dk: 0.00644 | AA Unweighted R2: 0.94 k: 0.0455 dk: 0.00705 | HW Weighted R2: 0.95 k: 0.0383 dk: 0.00549 | HW Unweighted R2: 0.95 k: 0.0373 dk: 0.00536

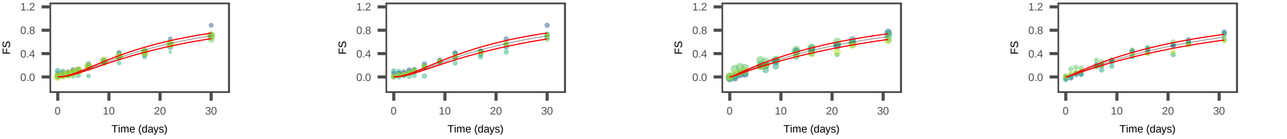

TPIS

AA Weighted R2: 0.96 k: 0.0214 dk: 0.00172 | AA Unweighted R2: 0.96 k: 0.0208 dk: 0.00184 | HW Weighted R2: 0.89 k: 0.0177 dk: 0.00271 | HW Unweighted R2: 0.88 k: 0.0177 dk: 0.00288

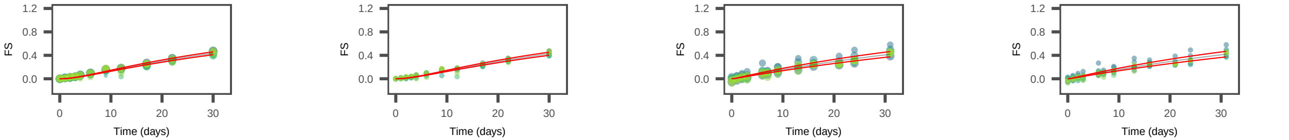

TPM1

AA Weighted R2: 0.93 k: 0.0222 dk: 0.00235 | AA Unweighted R2: 0.93 k: 0.022 dk: 0.00232 | HW Weighted R2: 0.95 k: 0.0196 dk: 0.00188 | HW Unweighted R2: 0.94 k: 0.0199 dk: 0.00216

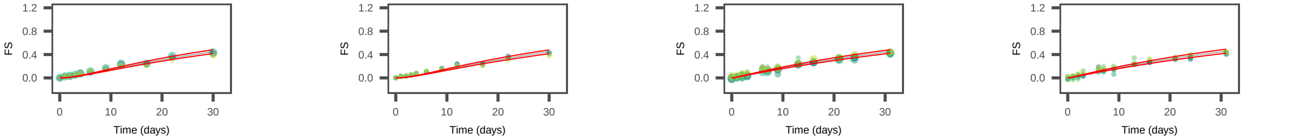

TPM2

AA Weighted R2: 0.93 k: 0.0115 dk: 0.00116 | AA Unweighted R2: 0.94 k: 0.0115 dk: 0.00106 | HW Weighted R2: 0.95 k: 0.0141 dk: 0.00128 | HW Unweighted R2: 0.95 k: 0.0142 dk: 0.00128

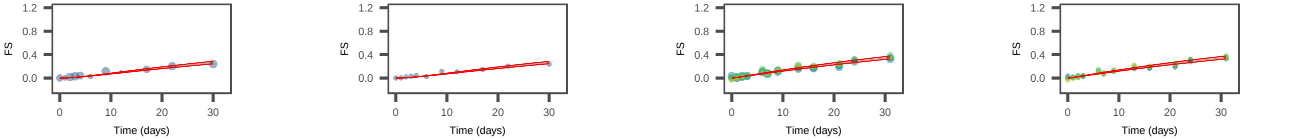

TRFE

AA Weighted R2: 0.96 k: 0.612 dk: 0.263 | AA Unweighted R2: 0.96 k: 0.598 dk: 0.251 | HW Weighted R2: 0.89 k: 0.248 dk: 0.0866 | HW Unweighted R2: 0.86 k: 0.237 dk: 0.0919

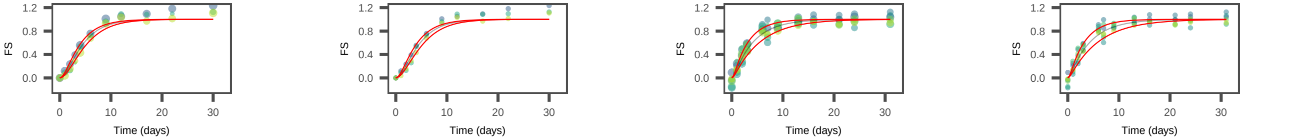

UCRI

AA Weighted R2: 0.96 k: 0.0426 dk: 0.00495 | AA Unweighted R2: 0.96 k: 0.0427 dk: 0.0051 | HW Weighted R2: 0.95 k: 0.025 dk: 0.00305 | HW Unweighted R2: 0.95 k: 0.025 dk: 0.00312

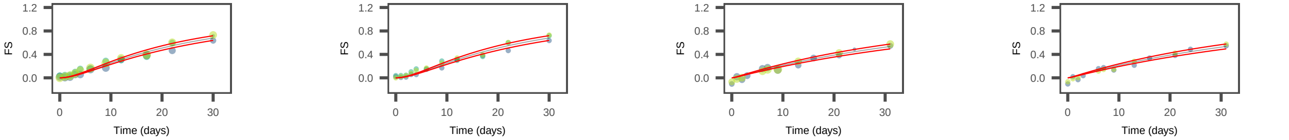

UGPA

AA Weighted R2: 0.96 k: 0.0766 dk: 0.0124 | AA Unweighted R2: 0.96 k: 0.0748 dk: 0.0125 | HW Weighted R2: 0.97 k: 0.0656 dk: 0.00884 | HW Unweighted R2: 0.97 k: 0.0662 dk: 0.00928

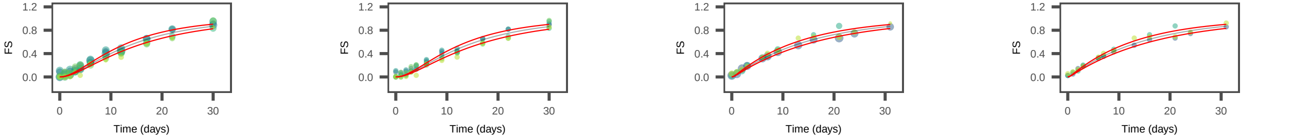

VDAC1

AA Weighted R2: 0.97 k: 0.041 dk: 0.00449

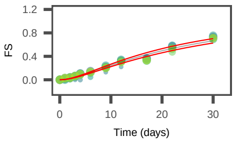

AA Unweighted R2: 0.97 k: 0.0409 dk: 0.00473

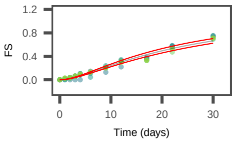

HW Weighted R2: 0.95 k: 0.0247 dk: 0.00298

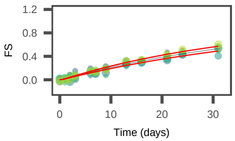

HW Unweighted R2: 0.94 k: 0.0246 dk: 0.00318

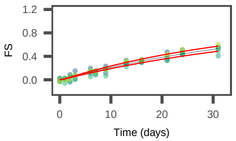

VDAC2

AA Weighted R2: 0.94 k: 0.0423 dk: 0.0066

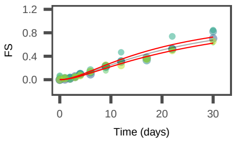

AA Unweighted R2: 0.94 k: 0.0426 dk: 0.00699

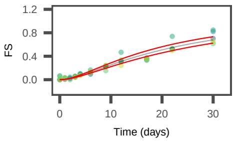

HW Weighted R2: 0.93 k: 0.0255 dk: 0.00342

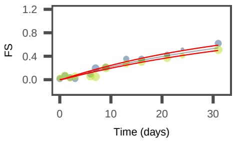

HW Unweighted R2: 0.94 k: 0.0256 dk: 0.00342

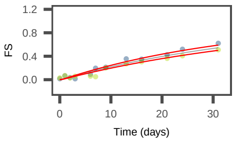

VDAC3

AA Weighted R2: 0.95 k: 0.0351 dk: 0.00468

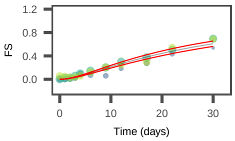

AA Unweighted R2: 0.94 k: 0.0342 dk: 0.00477

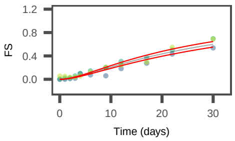

HW Weighted R2: 0.93 k: 0.0264 dk: 0.00342

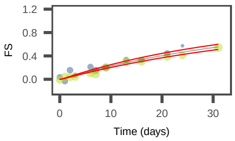

HW Unweighted R2: 0.93 k: 0.0266 dk: 0.00354

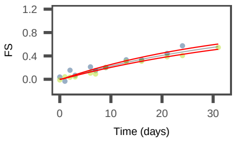

VIME

AA Weighted R2: 0.81 k: 0.066 dk: 0.0219

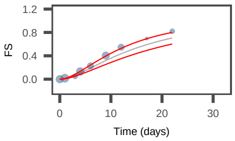

AA Unweighted R2: 0.79 k: 0.0618 dk: 0.0216

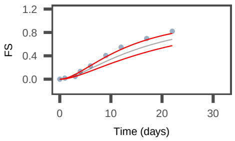

HW Weighted R2: 0.92 k: 0.0591 dk: 0.0154

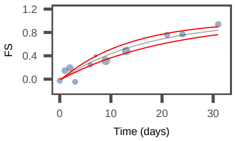

HW Unweighted R2: 0.92 k: 0.0599 dk: 0.0157

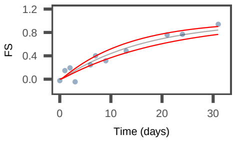

Supplement: Supplemental Data S9 [file mmc10.pdf]
