# Supplementary material for: Harmonizing Labeling and Analytical Strategies to Obtain Protein Turnover Rates in Intact Adult Animals
Source: Mol Cell Proteomics. 2022 May 28;21(7):100252. doi: 10.1016/j.mcpro.2022.100252 (PMC9249856; doi:10.1016/j.mcpro.2022.100252)
Supplement: Supplemental Data S10 [file mmc11.pdf]

Supplemental Figures

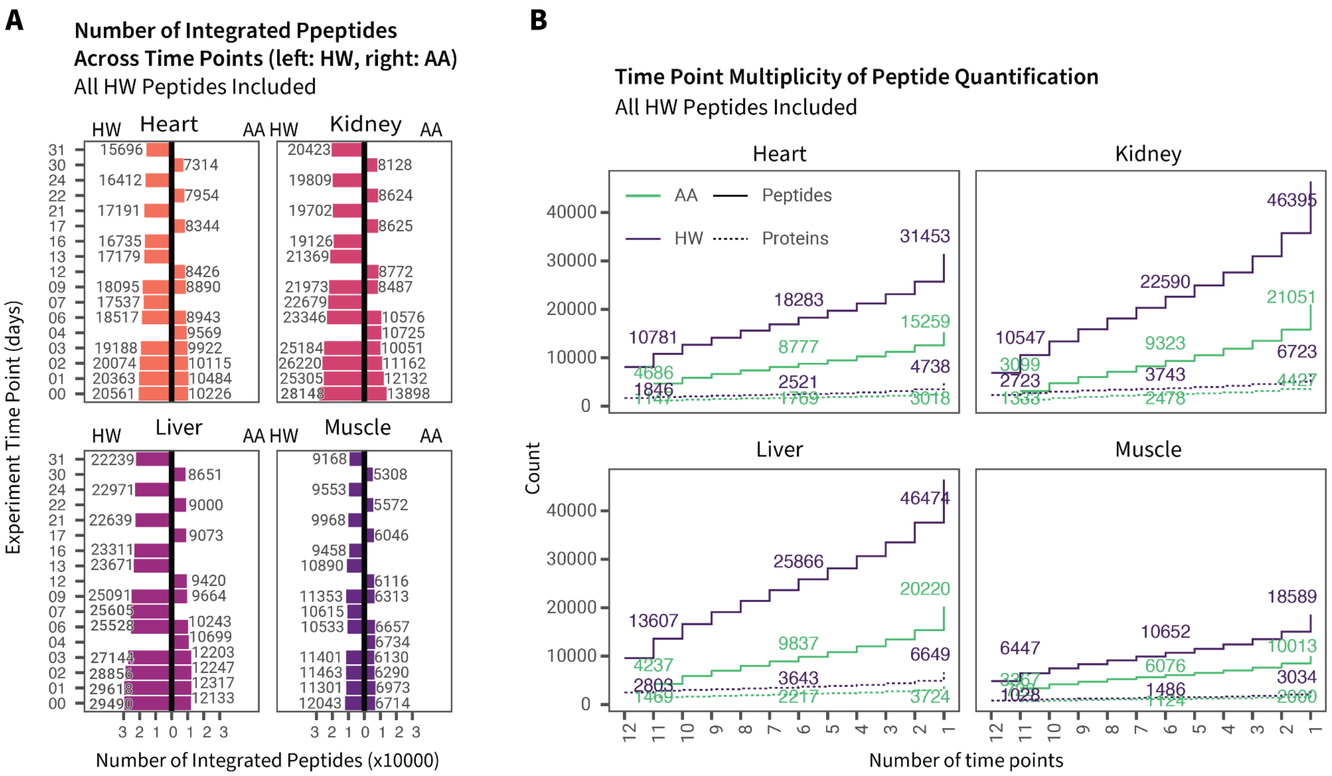

Supp. Figure S1. Peptide quantification performance inclusive of all HW labeling peptides.
A. As in Figure 3, for each tissue, each bar defines the number of peptides integrated over each experimental time point in the labeling period. Here only peptides containing one lysine are included in the AA labeling experiment, whereas all quantified peptides are included in the HW labeling experiment. **B.** For each tissue, the cumulative number of peptides (solid line) and unique proteins (dashed lines) quantified at increasing number of minimal time points in the heavy water (HW) labeling (green) and amino acid (AA) labeling (blue) data sets.

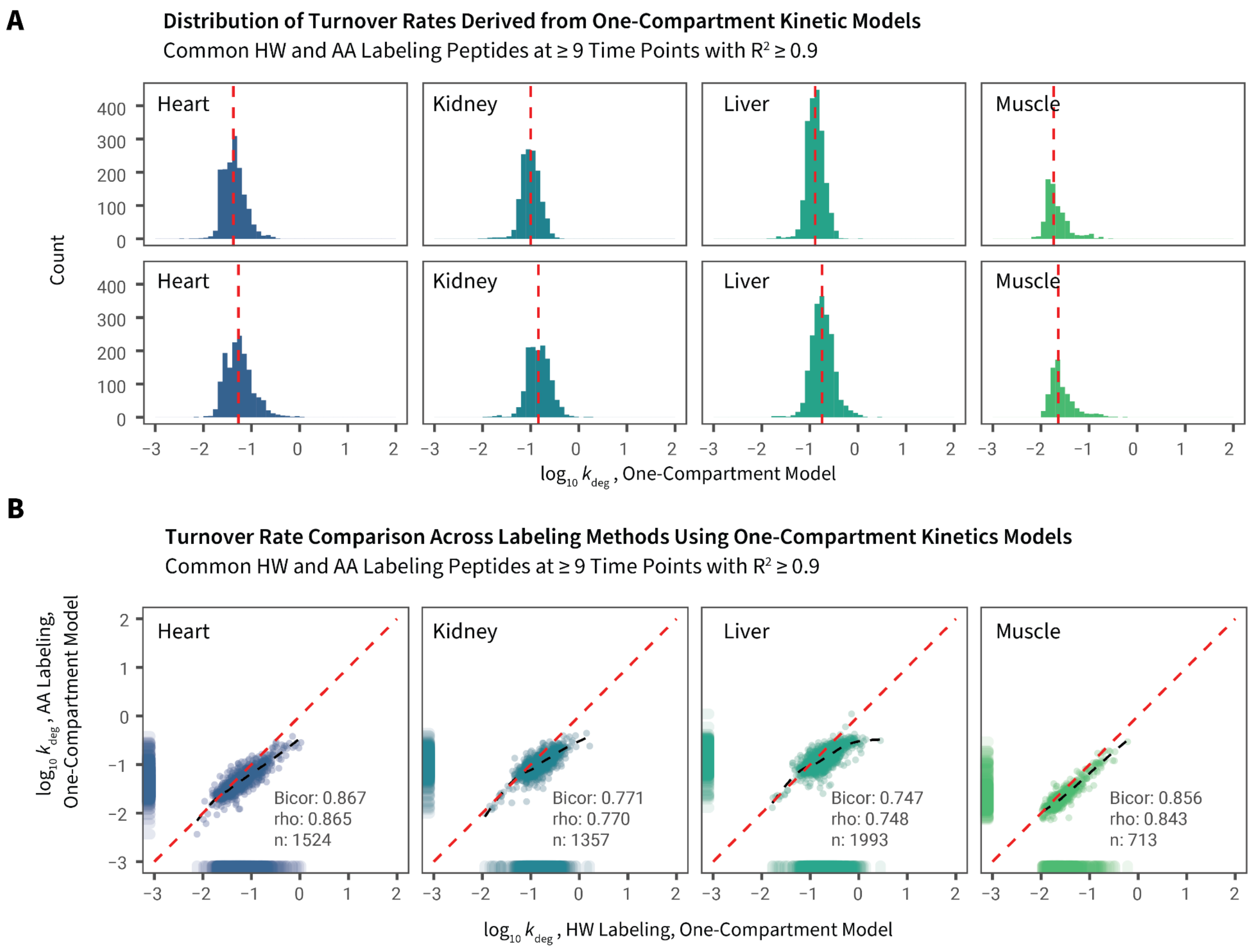

Supp. Figure S2. Comparison of HW and AA labeling data in one-compartment fitting.
A. Histogram showing distribution of k_{deg} across tissues and between common HW and AA labels using a simple exponential model (quantified time points ≥ 9, R^2 ≥ 0.9, one lysine) where peptide isotope enrichment is described by a single rate constant (k_{deg}). Red dashed lines denote medians. **B.** Scatterplots of shared proteins quantified by HW and AA in each tissue using the one-compartment model (quantified time points ≥ 9, R^2 ≥ 0.9, one lysine). Numbers denote robust correlation (biweight midcorrelation; bicor) coefficients and numbers of compared peptides (n).

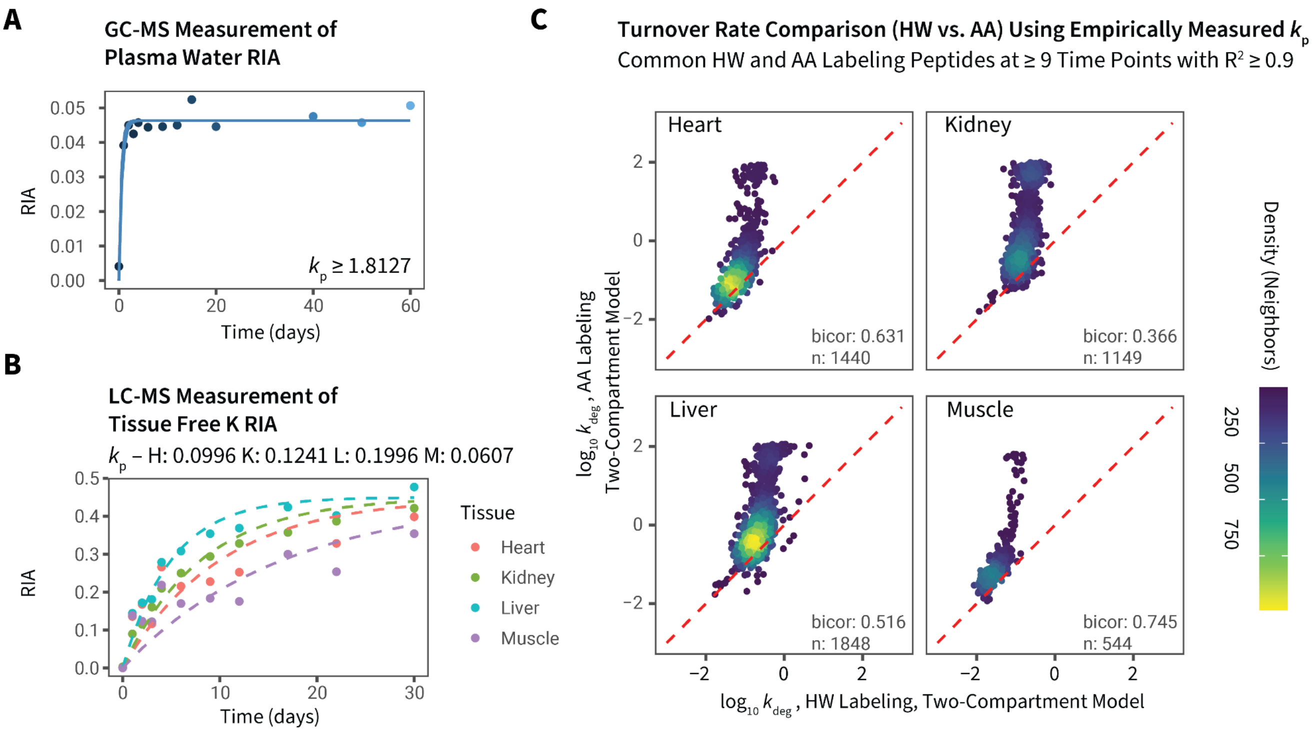

Supp. Figure S3. Empirical measures of tissue precursor RIA values at each time point.
Precursor relative isotope abundance (RIA_p) was measured using **A.** GC-MS of plasma samples in HW labeling and **B.** LC-MS of tissue free lysine in AA labeling. The precursor RIA over time data were fitted to a simple exponential model to find the best fit k_p using nonlinear least squares. **C.** Using the empirically- derived k_p values in a two-compartment model led to apparent high k_{deg} peptides in AA labeling, since the peptide RIA rise curves for fast-turnover peptides do not converge to the model when it is constrained by an underestimated k_p. X-axis: $\log_{10}$ turnover rate constants (k_{deg}) of HW labeling; y-axis: $\log_{10}$ turnover rate constants of AA labeling. Each data point represents one peptide. Peptides with one lysine integrated at ≥ 9 time points and fitted to a two-compartment model at $R^2 \geq 0.9$ are included. Red dash line: unity. Number: biweight midcorrelation (bicor) and number of individual peptides compared in HW vs. AA. In this panel, the marginal rugs refer to distributions of each individual axis regardless of whether a pairwise data point (commonly quantified peptide) is present.

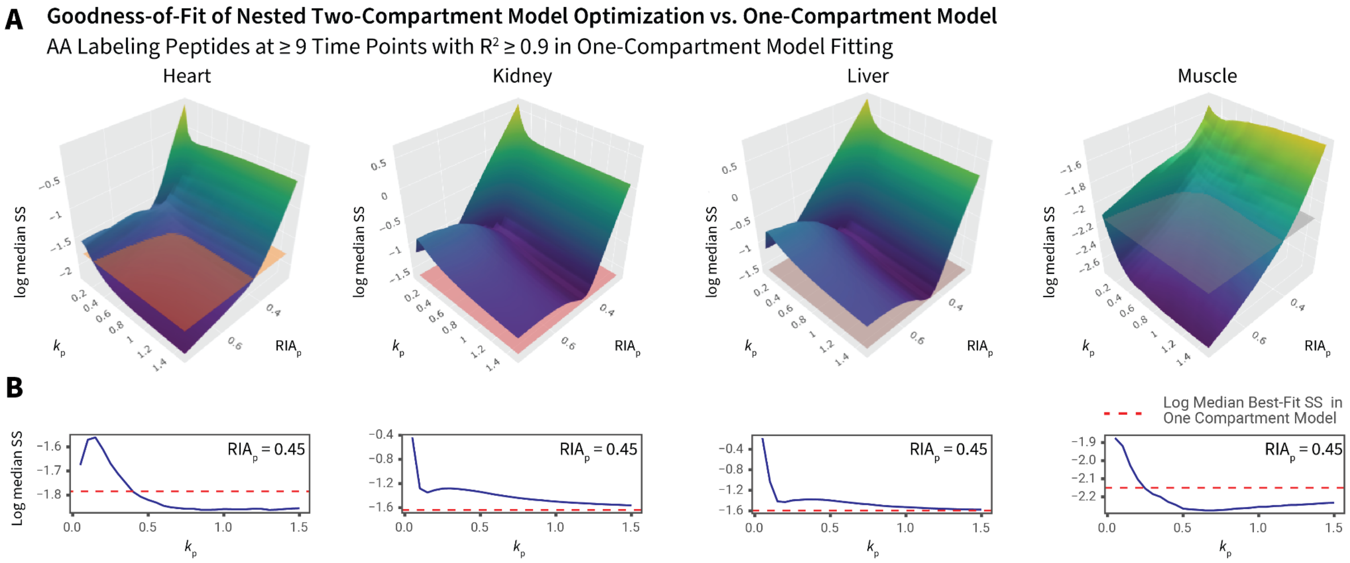

Supp. Figure S4. Determination of precursor RIA kinetics using proteome-wide nested optimization.
A. We performed multiple rounds of two-compartment model curve-fitting by iterating through different k_p and plateau precursor RIA_p values from 0.05 to 2.0 at 0.05 increments (x-axis). Peptides quantified at ≥ 9 time points and fitted with R^2 ≥ 0.9 in the one-compartment model were used. The median sums-of-squares of the residuals of fitting of each peptide time series in the two-compartment model in each tissue (z-axis) were compared to that from the one-compartment model (horizontal mesh). **B.** Corresponding two-dimensional cross-sections at various k_p values with asymptotic RIA_p fixed at 0.45. Red dash line: median sums-of-squares of peptide fitting in the simple exponential (one-compartment) model.

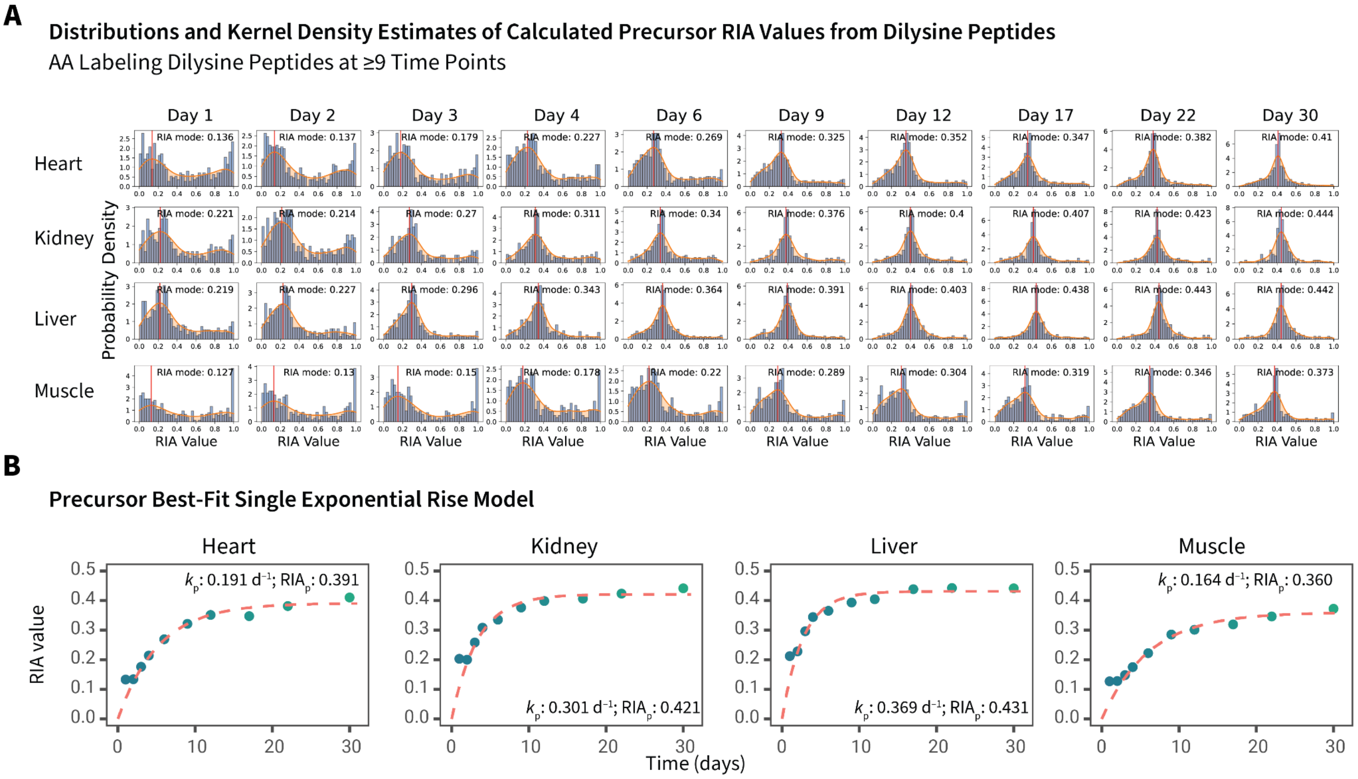

Supp. Figure S5. Determining AA precursor kinetics from dilysine peptides using Gaussian KDE.
A. Distribution of calculated RIA values derived from mass isotopomer analysis of the intensities of the m_6 and m_{12} peaks of peptides containing two lysine residues and quantified at ≥ 9 time points (bars). The best-estimate single tissue precursor RIA values for each time point for each tissue were derived from the modes of Gaussian kernel density estimations (red curve). **B.** The estimated tissue-specific precursor RIA values were fitted to a simple exponential model to derive the precursor rate constant k_p and asymptotic precursor RIA value RIA_p.

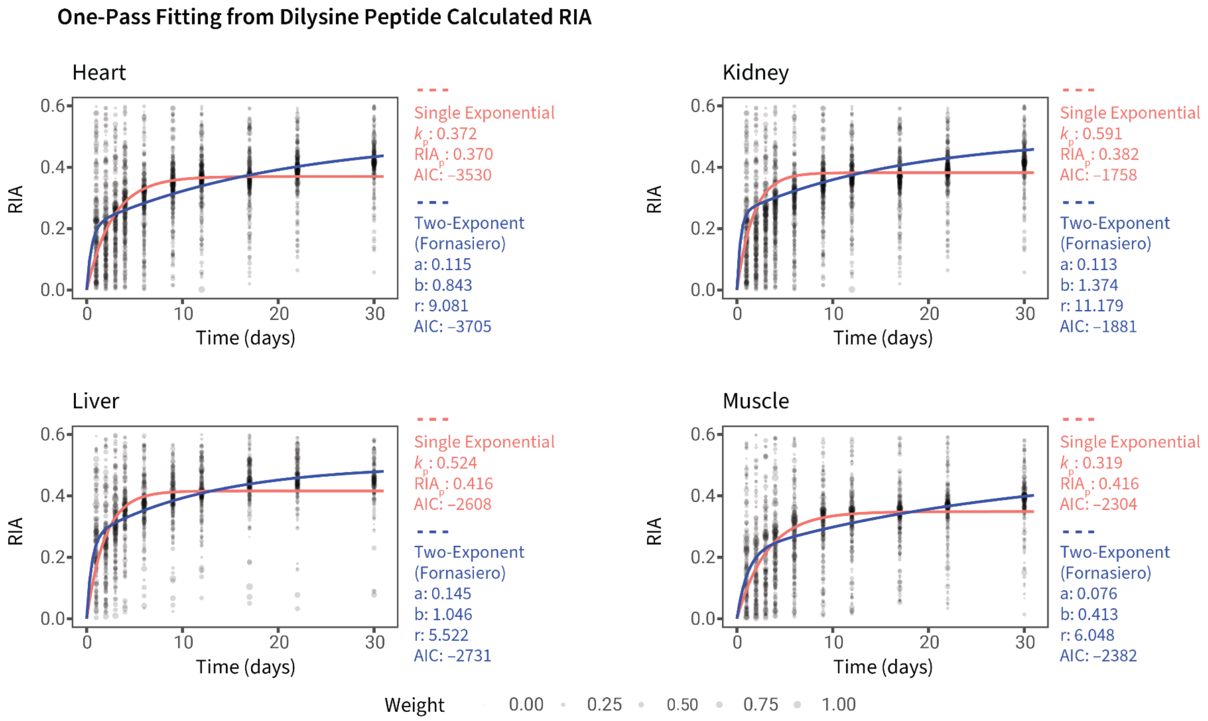

Supp. Figure S6. Determining AA precursor kinetics from dilysine peptides using weighted fitting. The distributions of calculated RIA values using the m_6 and m_{12} peaks of dilysine peptides (y-axis) at each time point (x-axis) are shown across the four tissues, calculated as in Supplemental Figure S5. Data points with RIA between 0 and 0.6 are included and fitted directly to a simple exponential (red) model to find the best-fit k_p and plateau RIA_p; or the double exponential (blue) model described in Fornasiero *et al.*[11] to find the best-fit values for the parameters *a*, *b*, and *r* in the Fornasiero model. Weighted nonlinear least squares fitting was performed using the square of normalized peptide intensity of each data point as weight.

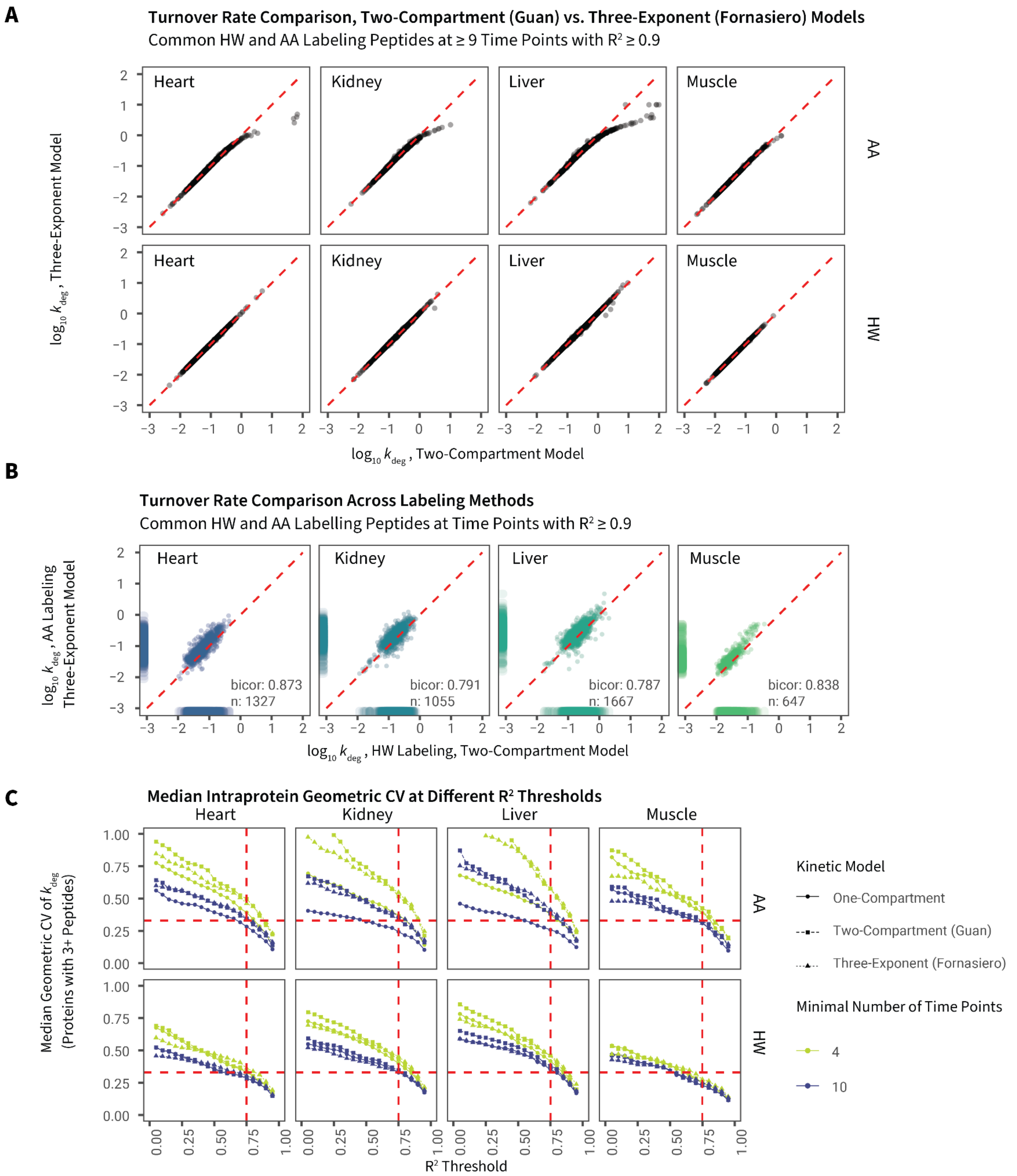

Supp. Figure S7. Comparison of the two-compartment and three-exponent models.
A. Scatterplots showing the $\log_{10}$ peptide turnover rate constants in each tissue from HW and AA labeling derived using the Guan et al. two-compartment model (x-axis) and the Fornasiero three-exponent model (y-axis). Model parameters were derived using one-pass fitting to dilysine peptide RIA values as in Supplemental Figure S6. Each data point represents one common peptide quantified at ≥ 9 time points and fitted to each model as $R^2 \geq 0.9$. Red dashed lines: unity. **B.** Scatterplots comparing the $\log_{10}$ turnover rate constants in HW labeling (x-axis) derived using the two-compartment model with those in AA labeling (y-axis) derived using the three-exponent model. Numbers represent robust biweight midcorrelation (bicor) between HW and AA. **C.** Relationships between data variance as measured by intra-protein geometric coefficients of variation (CV) for proteins quantified with ≥ 3 peptides across multiple fitting R^2 threshold (x-axis), with different time point multiplicity filters (color), and different kinetics models.

Variance increased significantly when peptide time-series with lower fitting R^2 were included, and the two compartment models showed higher variance than the simple exponential model.

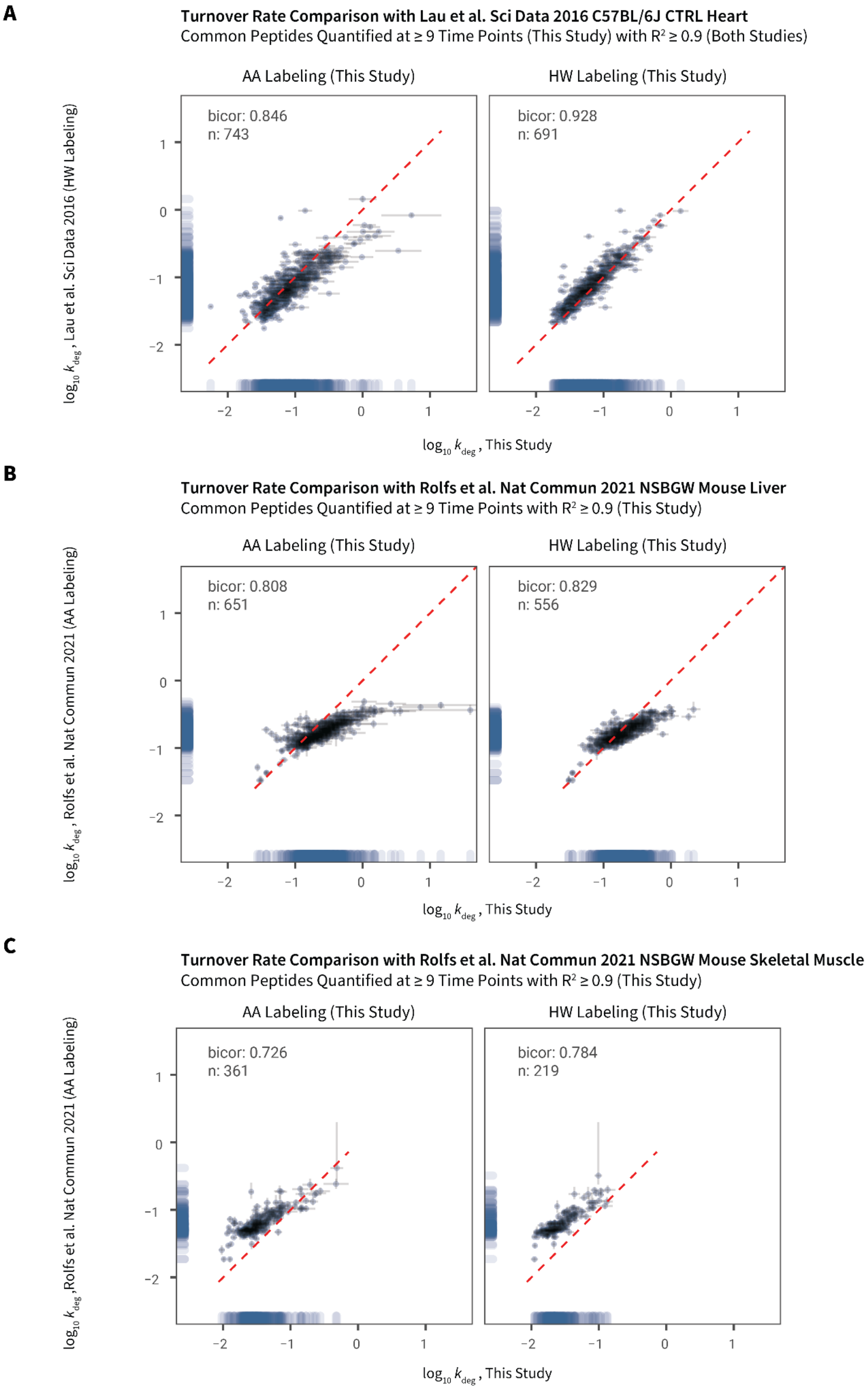

Supplemental Figure S8. Comparison of turnover rate constants with a previous study.

Scatterplots showing the log10 peptide turnover rates quantified with $R^2 \geq 0.9$ and at ≥ 9 time points in AA labeling (left) and HW labeling (right) in this study (x-axis) against the log10 peptide turnover rates in prior studies (y-axis): **A.** Peptides quantified with $R^2 \geq 0.9$ in C57BL/6J mouse heart in Lau et al. 2016 (HW labeling), vs. C57BL/6JOlaHsd mouse heart peptides in this study; **B.** reported peptides in NSBGW mouse liver in Rolfs et al. 2021, vs. C57BL/6JOlaHsd mouse liver peptides in this study; **C.** reported peptides in NSBGW skeletal (sternocleidomastoid) muscle in Rolfs et al. 2021, vs. C57BL/6JOlaHsd mouse skeletal muscle (pooled hindlimb) in this study. Bicor: biweight midcorrelation; n: number of compared peptides. Error bars: dk_{deg} of fitting. Dashed red line: unity.

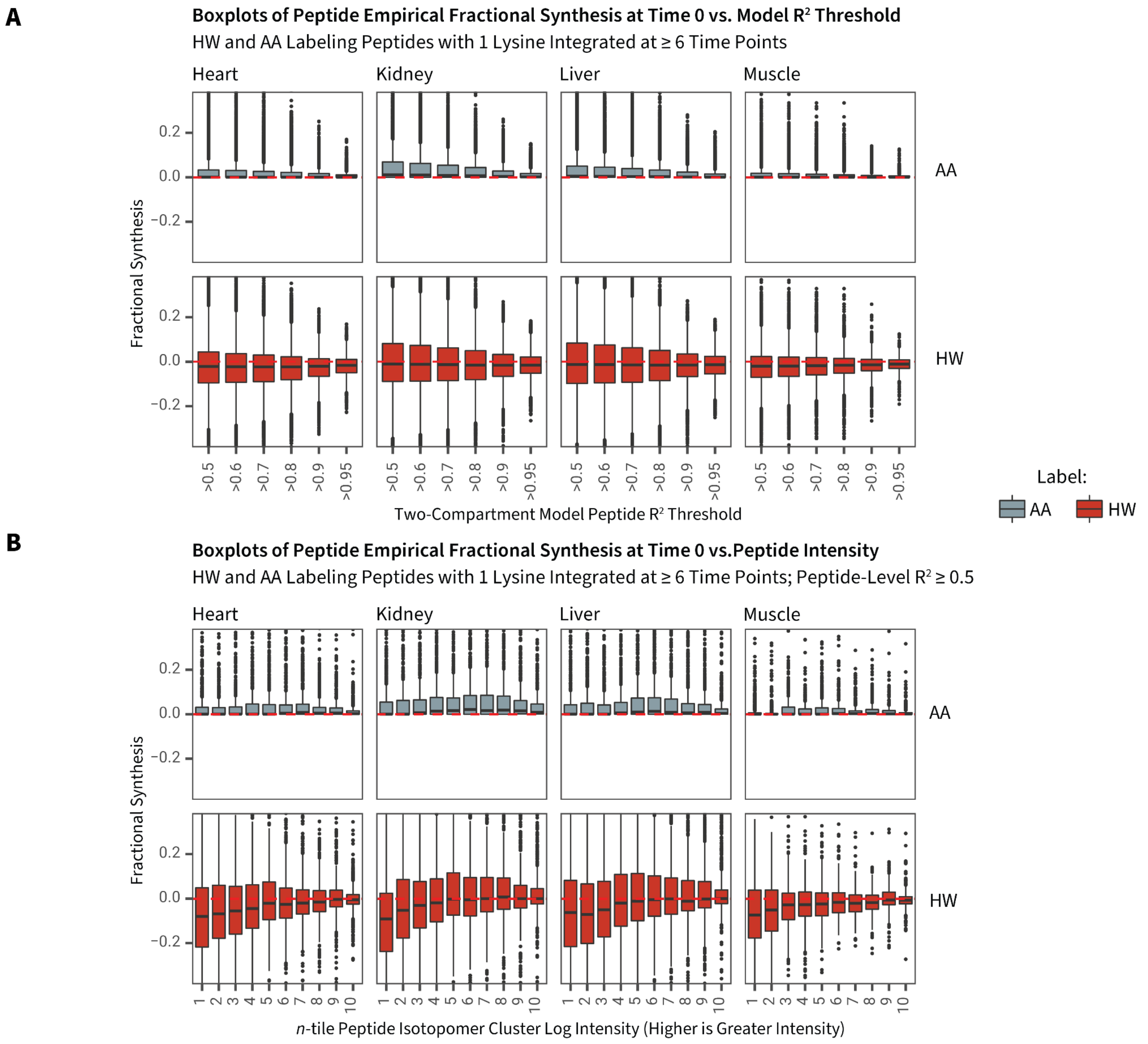

Supplemental Figure S9. Comparison of quantitative errors at the isotopomer integration level
A. Boxplots showing quantitative errors across labeling methods and organs at the individual isotopomer integration level, considering only the day 0 time point where true fractional synthesis is expected to be 0 prior to labeling commencement. HW and AA labeling peptides with 1 lysine, integrated at ≥ 6 time points, and fitted to a two-compartment model at various R^2 thresholds (x-axis) are included. **B.** As above, for HW and AA labeling peptides with 1 lysine, integrated at ≥ 6 time points, with peptide-level $R^2 \geq 0.5$. The peptide isotopomers are separated into equal deciles based on log total peptide isotopomer intensity (higher decile = higher intensity).

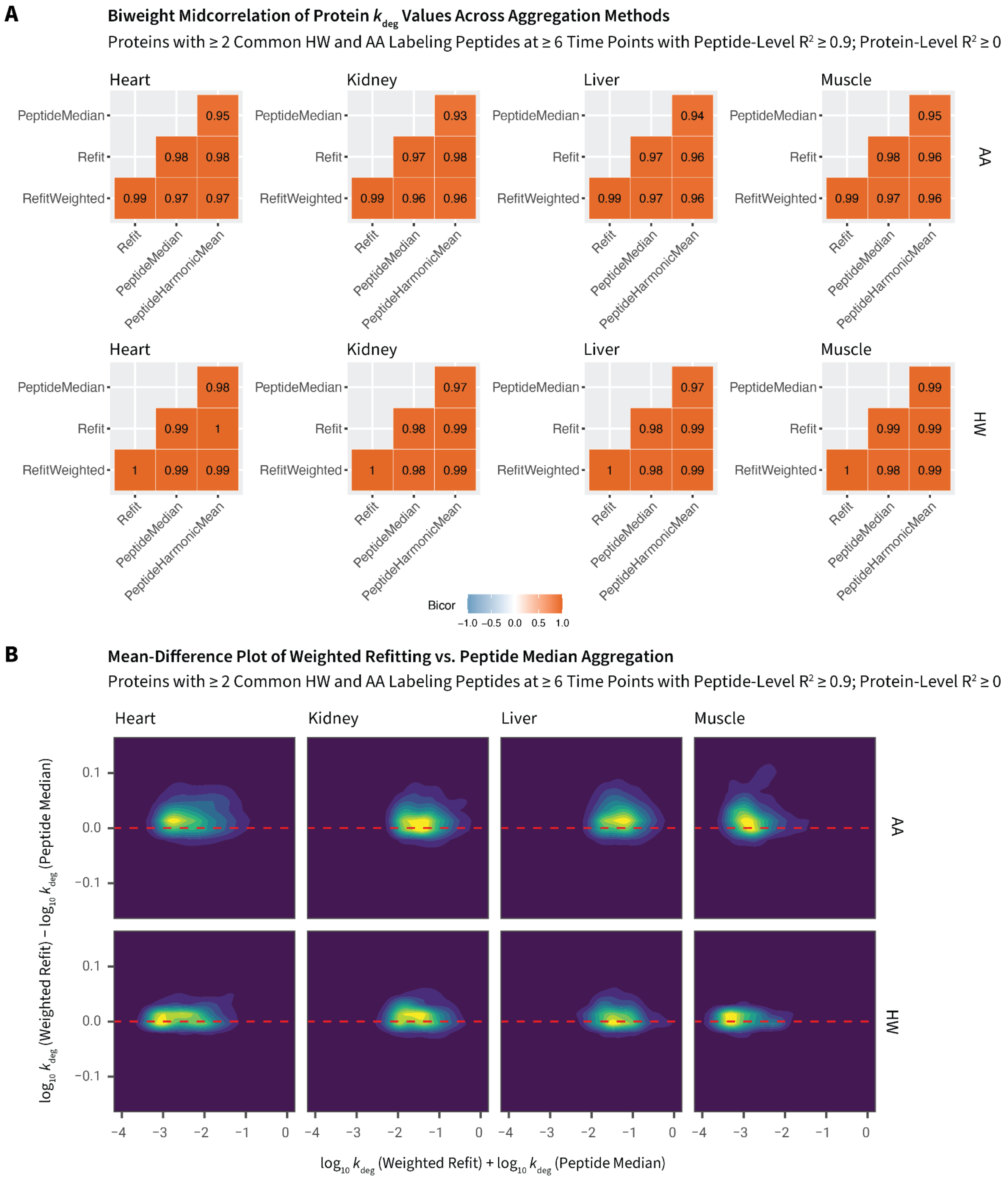

Supplemental Figure S10. Protein level data aggregation methods.
A. Correlation plots showing the biweight midcorrelation (Bicod) coefficients between four examined data aggregation methods to derive protein-level turnover rate constants. Proteins with 2 or more unique-mapping peptides in HW and AA labeling integrated at 6 or more time points with peptide-level $R^2 \geq 0.9$ and protein-level $R^2 \geq 0$ are compared. Colors represent correlation coefficients. **B.** Density contour plots of the sum (x-axis) and difference (y-axis) between two aggregation methods: weighted regitting at the peptide level and summary of protein-level turnover rates as the median of peptide-level turnover rates. Proteins with 2 or more unique-mapping peptides in HW and AA labeling integrated at 6 or more time points with peptide-level $R^2 \geq 0.9$ and protein-level $R^2 \geq 0$ are compared. Colors represent data density. Red dashed lines represent zero difference between two aggregation methods.

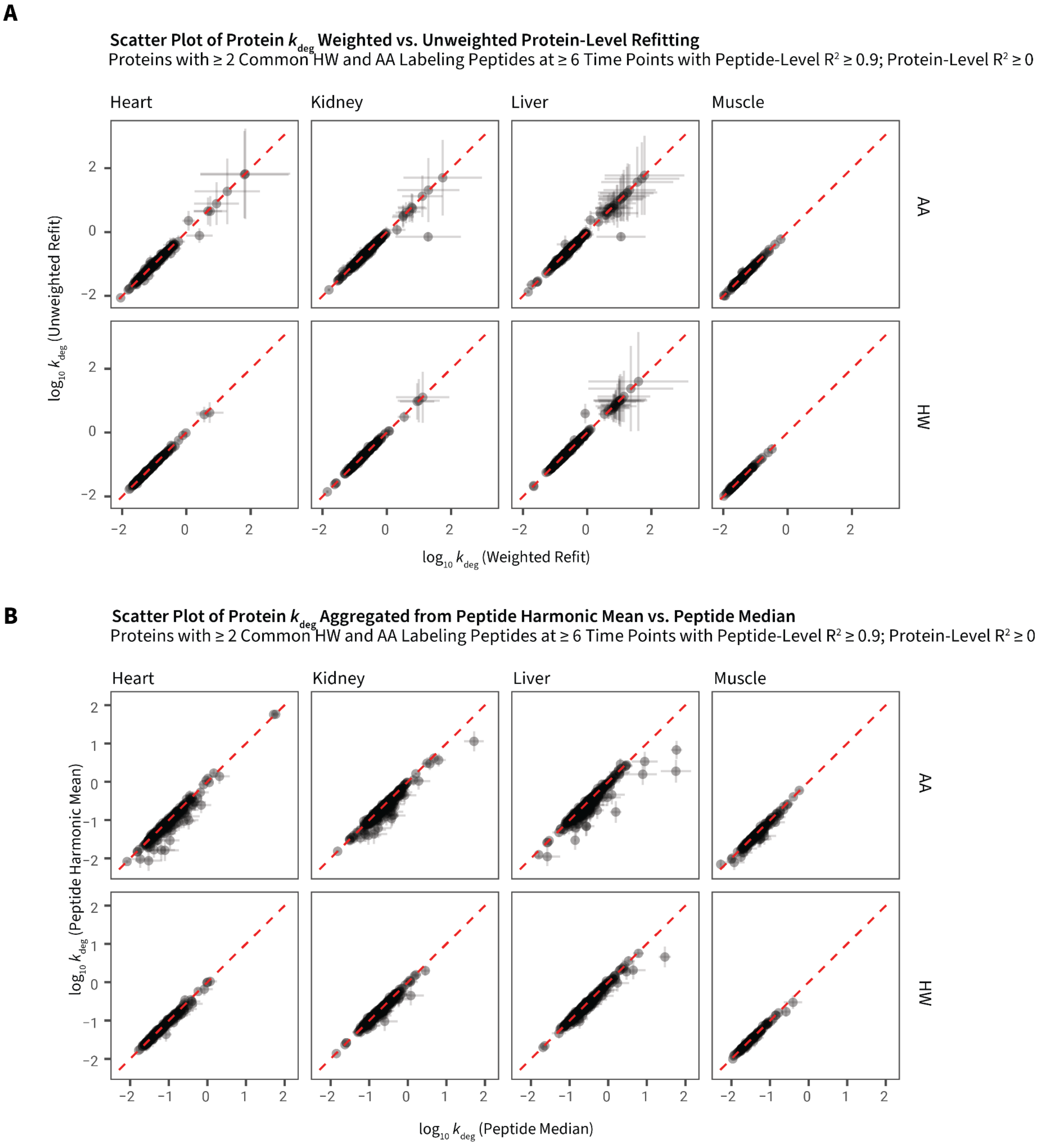

Supplemental Figure S11Bias and uncertainties in protein-level data aggregations.
A. Scatterplot showing data distribution and uncertainty of $\log_{10}$ k_{deg} between (y-axis) protein-level curve-fitting using all fractional synthesis data from qualifying peptides in the protein and (x-axis) refitting using the log normalized peptide intensity as weight. Proteins with 2 or more unique-mapping peptides in HW and AA labeling integrated at 6 or more time points with peptide-level $R^2 \geq 0.9$ and protein-level $R^2 \geq 0$ are compared. **B.** Scatterplot showing data distribution and uncertainty of $\log_{10}$ k_{deg} between (y-axis) collapsing peptide-level data as proteins using harmonic mean of peptide k_{deg} and (x-axis) collapsing peptide-level data as proteins using median of peptide k_{deg}. Error bars represent harmonic mean standard deviation (y-axis) and median absolute deviation (x axis).

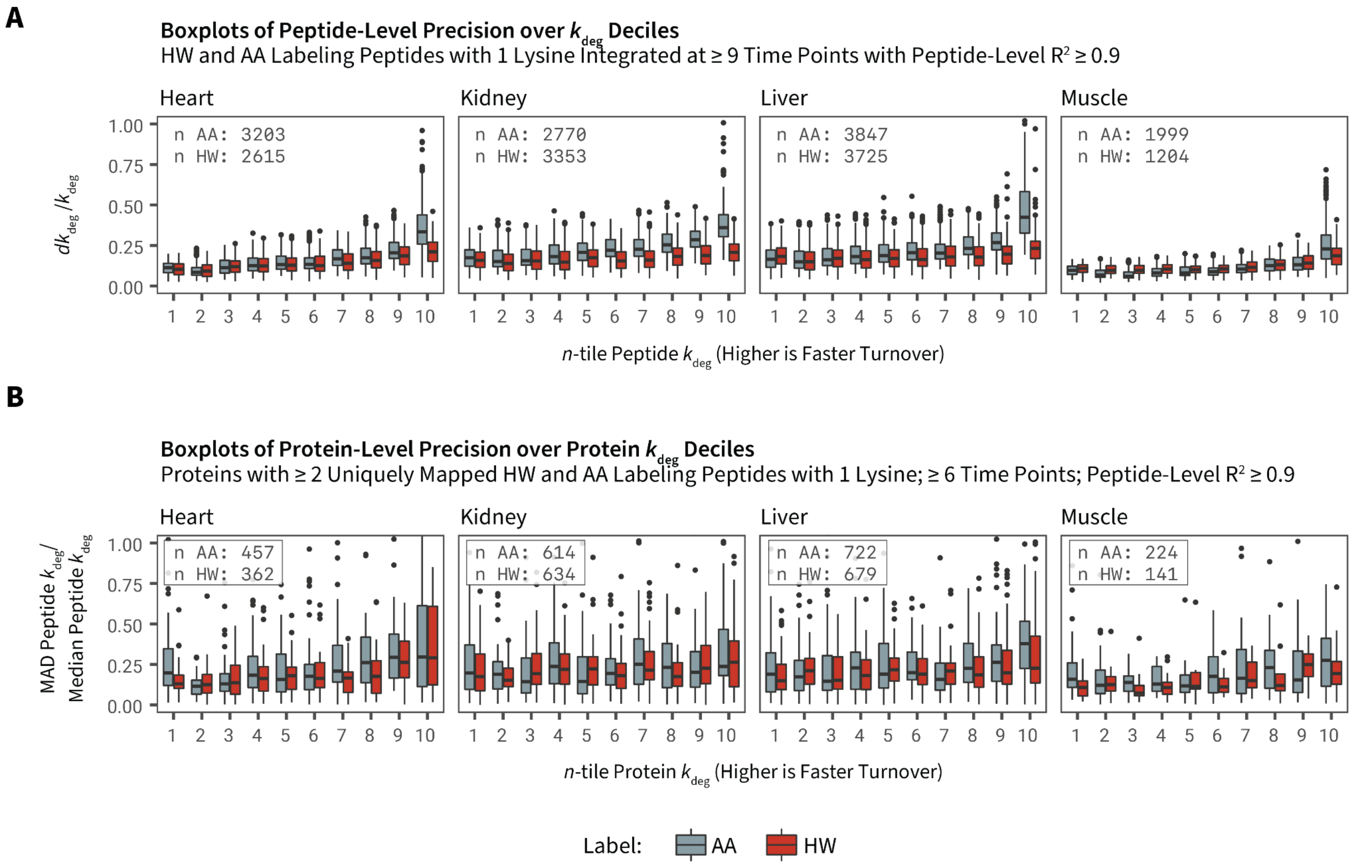

Supplemental Figure S12. Comparison of quantitative errors at peptide-fitting and protein levels.
A. Boxplots showing quantitative precision across labeling methods and organs at the peptide level, calculated as dk_{deg} divided by k_{deg} of kinetic curve-fitting in peptides with lower vs. higher k_{deg}. Peptide k_{deg} values are separated into equal deciles. HW and AA labeling peptides with 1 lysine, integrated at ≥ 9 time points, and fitted with R^2 ≥ 0.9 are included in the comparison. n: number of total data points in each organ. **B.** Boxplots showing quantitative precision across labeling methods and organs at the protein level, calculated as median absolute deviation of k_{deg} divided by median of k_{deg} of constituent peptides. Protein k_{deg} values are separated into equal deciles. Proteins with ≥2 uniquely mapped HW and AA labeling peptides with 1 lysine, integrated at ≥ 6 time points, and fitted with R^2 ≥ 0.9 are included in the comparison.

Supplemental Data

All Supplemental Data are available online on *figshare* at
https://doi.org/10.6084/m9.figshare.17096636.v4

Supplemental Data S1: Table containing turnover rate constants of peptides
Supplemental Data S2: Fitted curves for common peptides in HW and AA labeling (≥ 9 time points; $R^2 \geq 0.9$) in the heart
Supplemental Data S3: Fitted curves for common peptides in HW and AA labeling (≥ 9 time points; $R^2 \geq 0.9$) in the kidney
Supplemental Data S4: Fitted curves for common peptides in HW and AA labeling (≥ 9 time points; $R^2 \geq 0.9$) in the liver
Supplemental Data S5: Fitted curves for common peptides in HW and AA labeling (≥ 9 time points; $R^2 \geq 0.9$) in the muscle
Supplemental Data S6: Table containing turnover rate constants at the protein level from weighted combined fitting (≥ 9 time points; peptide $R^2 \geq 0.9$)
Supplemental Data S7: Protein-level fitted curves for common peptides in HW and AA labeling (≥ 9 time points; $R^2 \geq 0.9$) in the heart
Supplemental Data S8: Protein-level fitted curves for common peptides in HW and AA labeling (≥ 9 time points; $R^2 \geq 0.9$) in the kidney
Supplemental Data S9: Protein-level fitted curves for common peptides in HW and AA labeling (≥ 9 time points; $R^2 \geq 0.9$) in the liver
Supplemental Data S10: Protein-level fitted curves for common peptides in HW and AA labeling (≥ 9 time points; $R^2 \geq 0.9$) in the muscle
